# Identification of *ACOT13* and *PTGER2* as novel candidate genes of autosomal dominant polycystic kidney disease through whole exome sequencing

**DOI:** 10.1186/s40001-021-00613-8

**Published:** 2021-12-09

**Authors:** Na Du, Dan Dong, Luyao Sun, Lihe Che, Xiaohua Li, Yong Liu, Bin Wang

**Affiliations:** 1 Infectious Diseases Department, The First Hospital of Jilin University, No.1 Xinmin Street, Changchun, 130021 Jilin China; 2 Genetic Diagnosis Center, The First Hospital of Jilin University, No.1 Xinmin Street, Changchun, 130021 Jilin China; 3 Department of Obstetrics and Gynecology, The First Hospital of Jilin University, No.1 Xinmin Street, Changchun, 130021 Jilin China

**Keywords:** Whole exome sequencing, Gene mutations, Polycystic kidney disease, *ACOT13*, *PTGER2*

## Abstract

**Background:**

Autosomal dominant polycystic kidney disease (ADPKD) is the most common monogenic kidney disorder. Half of the patients would slowly progress to end-stage renal disease. However, the potential target for ADPKD treatment is still lacking.

**Methods:**

Four ADPKD patients and two healthy family members were included in this study. The peripheral blood samples were obtained and tested by the whole exome sequencing (WES). The autosomal mutations in ADPKD patients were retained as candidate sites. The Gene Ontology (GO), Kyoto Encyclopedia of Genes and Genomes (KEGG) enrichment, and protein–protein interaction network (PPI) analyses were performed by clusterProfiler R package. A dataset containing 18 ADPKD patients and three normal samples were downloaded from the Gene Expression Omnibus (GEO) database and analyzed using the limma R package.

**Results:**

A total of six mutant genes were identified based on the dominant genetic pattern and most of them had not been reported to be associated with ADPKD. Furthermore, 19 harmful genes were selected according to the harmfulness of mutation. GO and KEGG enrichment analyses showed that the processes of single-organism cellular process, response to stimulus, plasma membrane, cell periphery, and anion binding as well as cyclic adenosine monophosphate (cAMP) signaling pathway and pathways in cancer were significantly enriched. Through integrating PPI and gene expression analyses, acyl-CoA thioesterase 13 (*ACOT13*), which has not been reported to be related to ADPKD, and prostaglandin E receptor 2 (*PTGER2*) were identified as potential genes associated with ADPKD.

**Conclusions:**

Through combination of WES, gene expression, and PPI network analyses, we identified *ACOT13* and *PTGER2* as potential ADPKD-related genes.

**Supplementary Information:**

The online version contains supplementary material available at 10.1186/s40001-021-00613-8.

## Introduction

Polycystic kidney disease (PKD) is a group of monogenic disorders, and is the common cause of end-stage renal disease. Most adult patients are affected by the autosomal dominant form (ADPKD), while the autosomal recessive polycystic kidney disease (ARPKD) is a rarer form that usually presents perinatally or in early childhood [1]. Mutations in *PKD1* and *PKD2*, which encode polycystin 1 and 2 (PC1 and PC2) proteins, are the most common causes of ADPKD. PC2, a cation channel, is a member of the transient receptor potential (TRP) family of ion channels [2]. It was reported that defects in *PKD2* trigger changes in mitochondrial energy metabolism [3]. the roles of PC1 and PC1PC2 complex are poorly understood [4]. Although PKD is inherited monogenically, it is heterogeneous in phenotype, gene, and allele [1], and 7% of ADPKD families are genetically unresolved [5]. Moreover, the molecular mechanisms underlying the renal dysfunction resulted from mutations in PKD genes and the physiological functions of polycystin proteins are also still unclear [6].

The targeted resequencing by pooling long-range polymerase chain reaction (LR-PCR) amplicons has been used in the identification of mutations in PKD. Despite its high sensitivity, specificity, and accuracy, the challenge in data interpretation limits the development of potential targets in PKD therapy [7]. In recent years, the whole genome sequencing (WGS) and whole exome sequencing (WES) have been increasingly applied to the diagnostic evaluation of patients with suspected genetic disorders, including detection of rare genetic events and new mutations contributing to disease [8]. Mallawaarachchi AC et al. reported that WGS could overcome the pseudogene homology and provide an efficient strategy for ADPKD diagnosis [9]. Moreover, the results of Daniela AB et al. confirmed that WES could detect the causative mutation in 2/3 of the affected individuals with chronic kidney disease including ARPKD, which would allow the identification of potential genes associated with kidney disease [10].

Here, we performed the WES using the blood samples of four ADPKD patients and two healthy family members to analyze their genes variation. In addition, a gene expression dataset containing 18 ADPKD patients and three normal samples were obtained from the Gene Expression Omnibus (GEO) database. Through integrated analyses of gene mutation, gene expression, gene function enrichment, and protein–protein interaction (PPI), we identified two genes (*ACOT13* and *PTGER2*) which were potentially associated with the pathogenesis of ADPKD.

## Materials and methods

### Clinical information

**Fig. 1:**
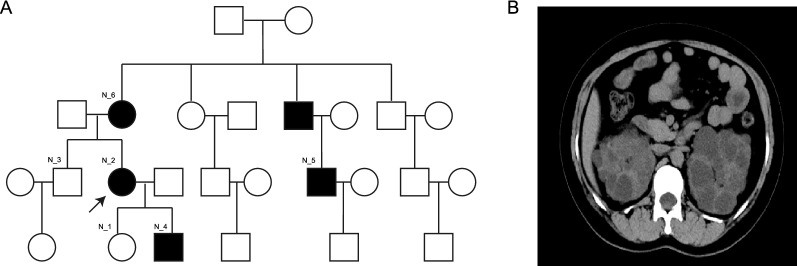
Family status of polycystic kidney patients. **A** Pedigree plot of N_2 with polycystic kidney disease. **B** Abdominal computed tomography (CT) of N_2. In this figure, the polycystic kidney disease cases included the patient's mother N_6, son N_4, and cousin N_5. The normal controls without disease consisted of the patient's younger brother N_3 and daughter N_1

This study was approved by the local ethics committee (approval number: 2019-307). An ADPKD patient treated in our hospital and five members of this family were included in this study. This patient had a history of polycystic kidney disease, polycystic liver disease, and kidney stones for 13 years (Additional file 1: Table S1). The pedigree of this family is shown in Fig. 1A. The N_2 was this proband. The N_4, N_5, and N_6 were all ADPKD patients (black), whereas N_3 and N_1 were healthy people (white). In total, there were four ADPKD patients and two healthy controls in our study. The clinical symptoms of this proband included fever, back pain, and hematuria. The computed tomography (CT) examination of N_2 revealed multiple cysts in the liver and bilateral polycystic kidney. Some lesions were complex cysts, with changes in the right perirenal exudation that had blurred outlines and increased density (Fig. 1B).

### Whole exome sequencing

The genome DNA was extracted from peripheral blood samples using a DNA extraction kit (Tiangen Biotech, Beijing, China), and the exomes were captured using the Agilent SureSelect Human All Exon V6 kit (Agilent Technologies, Santa Clara, CA) according to the instructions of the kits. Whole-exome sequencing was performed using the Illumina Novaseq6000 instruments with paired-end 150-bp sequencing reads. Raw sequencing reads were preprocessed to remove the low-quality bases and reads using fastp [11], an ultra-fast all-in-one FASTQ preprocessor, and the default parameters were adopted. The sequence reads were aligned to the human genome (Build-UCSC hg19) using the BWA (Burrow–Wheeler Aligner, http://bio-bwa.sourceforge.net/) software. Then, single nucleotide polymorphism (SNP) and Insertion/deletion (Indel) were identified with the SAMtools software (http://samtools.sourceforge.net/). Using the ANNOVAR software (http://annovar.openbioinformatics.org/en/latest/user-guide/download/), functional annotation was performed for the identified SNP and Indel to investigate their genomic locations and variation information (Additional file 2: Table S2).

### Screening of candidate SNP/Indel for ADPKD

We first removed mutations with frequencies higher than 1% in at least one of the four databases (1000g_all, esp6500si_all, gnomAD_ALL, and gnomAD_EAS). Then, mutations in exonic or splicing (10 bp upstream and downstream of the exon) positions were retained. Small fragments (< 10 bp) non-frameshifting Indel mutations in the repeat region were also removed. In addition, mutations that met one of the following conditions were retained: (a) The sites that were considered as harmful by at least half of the four softwares SIFT [12], Polyphen [13], MutationTaster [14], and CADD [15] basing on the scores; (b) mutations that were predicted to affect the splicing by dbscSNV [16]. Then, the genetic mutations that were classified into Pathogenic and Likely Pathogenic ones according to the Criteria and guidelines for grading clinical significance of single gene mutations of American College of Medical Genetics and Genomics (ACMG, https://www.acmg.net/) were selected as candidate sites. For the screening of dominant genetic pattern, on the basis of mutation site filtering, the sites showing autosomal mutations in ADPKD patients, which, however, could not be detected in normal controls were retained as candidate sites.

### Functional enrichment and PPI analyses

The Gene Ontology (GO; http://geneontology.org) (including Biological Process, Molecular Function, and Cellular Component) and Kyoto Encyclopedia of Genes and Genomes (KEGG; https://www.genome.jp/kegg/) enrichment analyses were conducted to analyze the function of candidate SNP/Indel-related genes using the clusterProfiler package in R [17]. In addition, PPI network analysis of candidate SNP/Indel-related genes was carried out using the STRING database (https://string-db.org/cgi/input.pl).

### Differential expression analysis

The mRNA expression profile data of GSE7869 [18] was downloaded from the GEO database (https://ncbi.nlm.nih.gov/geo), which was detected based on Affymetrix Human Genome U133 Plus 2.0 Array. The GSE7869 dataset contained 18 ADPKD samples and three normal samples. The mRNA expression profile data was analyzed using the limma function package in R [19]. Differentially expressed genes (DEGs) were selected using the thresholds of absolute value of differential expression (|Log2FC|) > 0.5 and *P* value < 0.05.

### Statistical analysis

Statistical analyses were performed using R software v3.5.2. *P* value < 0.05 was considered statistically significant in all statistical analyses.

## Results

### Mutational landscape

**Fig. 2:**
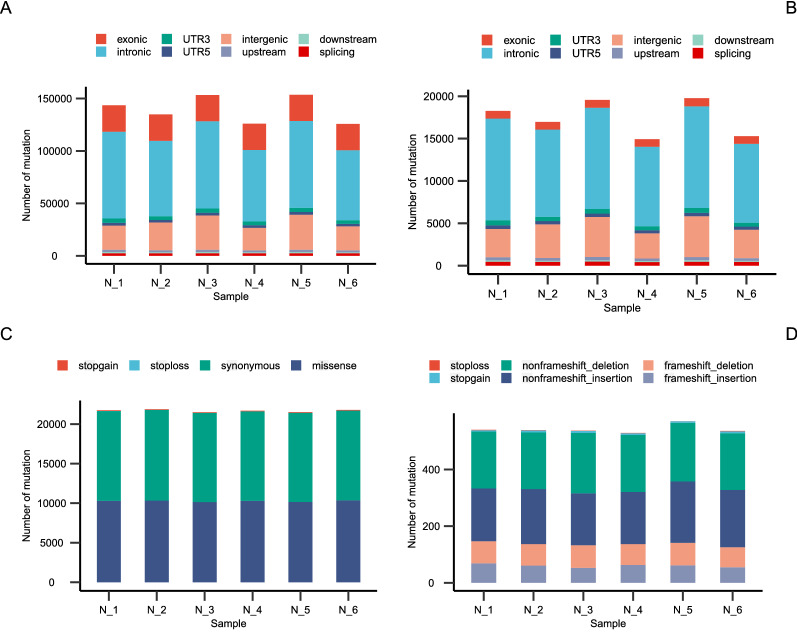
Distribution of mutations. Distribution across genomic features of all single-nucleotide variations (SNVs) (**A**) and Indels (**B**). Distribution across genomic features of SNVs (**C**) and Indels (**D**) located in coding region

The total number of SNPs in the six samples was concentrated between 100,000 and 150,000, and they were mainly located in the intron region, exon region, and intergenic region (Fig. 2A). The total number of Indels in the six samples was concentrated between 15,000 and 20,000, and these Indels mainly distributed in the intron and intergenic region (Fig. 2B). We next explored the landscape of mutations that were located in the coding region. Consequently, the SNPs of six samples in the coding region mainly included anonymous, missense, and stoploss mutations, whose cumulative number was nearly 21,000 (Fig. 2C). The Indels of six samples in the coding region mainly consisted of nonframeshift_deletion and nonframeshift_insertion (Fig. 2D).

### Potential mutations and genes for ADPKD

Six mutant genes, including *AGRN*, *ACOT13*, *ADCY4*, *HEATR5A*, *PTGER2*, and *ADAM21*, were screened using the dominant genetic pattern. They were heterozygously mutated in ADPKD samples but not mutated in normal samples*.* Most of these genes have not been reported to be related to the occurrence of ADPKD. Meanwhile, 19 genes were also selected according to the harmfulness of mutation, including *MUTYH, USH2A*, *HBS1L*, *GLI3*, *SBDS*, *SND1*, *ABCA2*, *RPS6KA4*, *FLVCR1*, *ATIC*, *SCN11A*, *ATP6V1A*, *GLRA1*, *PRMT8*, *PKD1*, *INSL3*, *SUPT5H*, *NCF4*, and *GPR143*. To study the relationship between those gene mutations and ADPKD, we first analyzed the effects of gene mutations on proteins. Consequently, all those genes had at least one mutation that could affect the coding of protein, indicating the potential roles of those mutations (Table 1).

**Table 1:** The protein changes of 25 genes

Gene name	Region	AA change	Screening method
AGRN	Exonic	NM_198576: exon10: c.G1855A: p. V619M	Dominant inheritance pattern
ACOT13	Exonic	NM_018473: exon3: c.G295T: p.D99Y	Dominant inheritance pattern
		NM_001160094: exon4: c.G226T: p. D76Y	
ADCY4	Splicing		Dominant inheritance pattern
HEATR5A	Exonic	NM_015473: exon17: c.C2449G: p.H817D	Dominant inheritance pattern
PTGER2	Exonic	NM_000956: exon1: c.G401A: p. R134H	Dominant inheritance pattern
ADAM21	Exonic	NM_003813: exon2: c.A945G: p. I315M	Dominant inheritance pattern
ABCA2	Exonic	NM_001606: exon9: c.C1045T: p.Q349X	Harmful screening
		NM_212533: exon9: c.C1135T: p. Q379X	
ATIC	Exonic	NM_004044: exon15: c.A1568T: p. E523V	Harmful screening
ATP6V1A	Exonic	NM_001690: exon4: c.415dupA: p.C138fs	Harmful screening
FLVCR1	Exonic	NM_014053: exon1: c.A551C: p. N184T	Harmful screening
GLI3	Splicing		Harmful screening
GLRA1	Exonic	NM_001292000: exon8:c.C1075T: p.R359C	Harmful screening
		NM_000171: exon9:c.C1324T: p. R442C	
		NM_001146040: exon9:c.C1348T: p. R450C	
GPR143	Exonic	NM_000273: exon1: c.134_179del: p. L45fs	Harmful screening
HBS1L	Exonic	NM_001145158: exon15: c. A1762T: p.K588X	Harmful screening
		NM_006620: exon16: c.A1888T: p. K630X	
INSL3	Exonic	NM_001265587: exon1: c.148dupC: p. R50fs	Harmful screening
		NM_005543: exon1: c.148dupC: p. R50fs	
MUTYH	Splicing		Harmful screening
NCF4	Exonic	NM_000631: exon3:c.C178T: p.R60C	Harmful screening
		NM_013416: exon3:c.C178T: p. R60C	
PKD1	Exonic	NM_000296: exon39:c.C11254T: p.R3752W	Harmful screening
		NM_001009944: exon39:c.C11257T: p. R3753W	
PRMT8	Exonic	NM_001256536: exon2: c.54_66del: p. N18fs NM_019854: exon2: c.81_93del: p. N27fs	Harmful screening
RPS6KA4	Exonic	NM_001006944: exon7: c.G755A: p.R252Q	Harmful screening
		NM_001300802: exon7: c.G755A: p.R252Q	
		NM_001318361: exon7: c.G566A: p. R189Q	
		NM_003942: exon7: c.G755A: p. R252Q	
SBDS	Splicing		Harmful screening
SCN11A	Exonic	NM_014139: exon14: c.G2386A: p.V796M NM_001349253: exon 18: c.G2386A: p. V796M	Harmful screening
SND1	Exonic	NM_014390: exon7:c.C694T: p. R232X	Harmful screening
SUPT5H	Splicing		Harmful screening
USH2A	Splicing		Harmful screening

### Significantly enriched KEGG pathways and GO terms of potential ADPKD-related genes

**Fig. 3:**
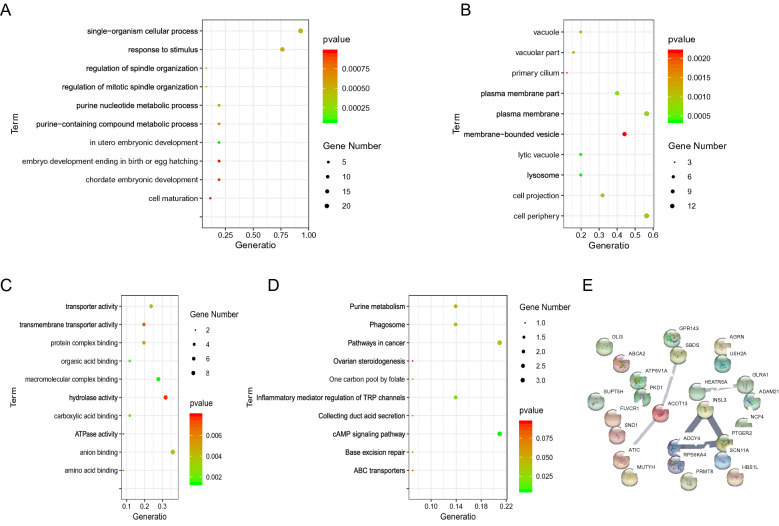
Significantly enriched functions and PPI network of the 25 potential ADPKD-related genes screened by WES analysis. Significantly enriched biological processes (**A**), cellular components (**B**), molecular functions (**C**), and KEGG pathways (**D**). Dot size shows the count of enriched genes, and dot color represents P value. (**E**) PPI network of the 25 potential ADPKD-related genes screened by WES analysis

The results of GO and KEGG enrichment analyses of the 25 potential genes associated with ADPKD are shown in Fig. 3A (Biological Process), Fig. 3B (Cellular Component), Fig. 3C (Molecular Function), and Fig. 3D (KEGG). GO analysis showed that these 25 genes were significantly enriched in the biological processes (BP) of single-organism cellular process and response to stimulus, cell components (CC) of plasma membrane and cell periphery, and molecular function (MF) of anion binding. In addition, the KEGG pathway analysis revealed that these 25 genes were mainly enriched in cyclic adenosine monophosphate (cAMP) signaling pathway and pathways in cancer.

### *ACOT13* and *PTGER2* might be candidate genes associated with ADPKD

**Fig. 4:**
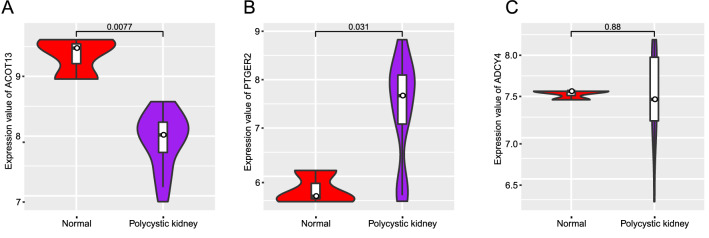
Boxplots illustrating the mRNA expression levels of *ACOT13* (**A**), *PTGER2* (**B**), and *ADCY4* (**C**) between ADPKD patients and normal controls

The PPI network analysis was performed to explore the association among the 25 potential ADPKD-related genes. As shown in Fig. 3E, most of those genes were independent in the network. *ACOT13* had direct interaction with both *SBDS* and *ATIC*; *PTGER2*, *ADCY4*, and *INSL3* were predicted to interact with each other (Fig. 3E). What’s more, *ACOT13*, *PTGER2*, and *ADCY4* were genes that only mutated in ADPKD patients. , based on the expression data downloaded from the GEO database, we compared the expression levels of *ACOT13*, *PTGER2*, and *ADCY4* between the ADPKD patients and the normal controls. Compared with the normal controls, the expression level of *ACOT13* was significantly lower in ADPKD patients (Fig. 4A), while *PTGER2* presented a higher expression level in ADPKD patients (Fig. 4B). *ADCY4* did not exhibit significant expression difference between ADPKD patients and normal samples (Fig. 4C). Combining the information, we speculated that *ACOT13* and *PTGER2* might be candidate genes associated with ADPKD.

## Discussion

ADPKD is caused by mutation in one of two genes-78% of cases are caused by mutation in *PKD1* on chromosome 16 and 15% of cases are caused by mutation in *PKD2* on chromosome 4 [20]. The cystic kidney diseases have also been termed ciliopathies. The PC1 and PC2 proteins, encoded by *PKD1* and *PKD2*, are both located on the primary cilia and act as flow sensors in the kidney [21]. In this study, the harmful mutation in *PKD1*, as well as other 18 known harmful genes, were detected in the samples of ADPKD patients. Moreover, six mutants, including *AGRN*, *ACOT13*, *ADCY4*, *HEATR5A*, *PTGER2*, and *ADAM21*, were screened using the dominant genetic pattern and most of them had not been previously recognized as ADPKD-related pathogenic genes.

The pathogenic proteins in ADPKD are mainly responsible for transmitting information from the external environment to the cells [22]. We analyzed the potential functions of the 25 identified genes by GO and KEGG enrichment analyses. The results showed that the BPs of single-organism cellular process, response to stimulus, as well as CCs of plasma membrane and cell periphery were significantly enriched. Besides, ADPKD cells can shift their mode of energy production from oxidative phosphorylation to other pathways, and these alterations in cell metabolism have emerged as a hallmark of ADPKD [6]. The alterations in modulation of energy production and utilization in ADPKD are dependent on several inner cellular signaling pathways, such as AMP-activated protein kinase (AMPK), calcium signaling at mitochondria-associated membranes, mammalian target of rapamycin complex 1 (mTORC1), cAMP, and cystic fibrosis transmembrane conductance regulator (CFTR)-mediated ion transport [23, 24]. Here, the cAMP signaling pathway was identified as the significantly enriched one in these ADPKD samples. These results support the reliability of the mutations we screened. Meanwhile, despite functional information was partly revealed by enrichment analysis, more details of the 25 identified genes deserved further exploration in ADPKD in the future.

The PPI and differential expression analyses indicated that compared with normal people, *ACOT13* and *PTGER2* were mutated and differentially expressed in ADPKD patients, and might be the potential genes associated with ADPKD. ACOT13 protein is a member of acyl-CoA thioesterases (Acots) enzymes, which catalyze the reaction of hydrolysis of fatty acyl-CoA molecules into free fatty acids plus CoASH. ACOT13 is enriched in oxidative tissues, and is associated with mitochondria [25]. Lin et al. reported that PC1 affected mitochondria morphology and function, which might play a key role in regulating mitochondrial function and cellular metabolism [26]. To our knowledge, there have not been reports on the relationship between *ACOT13* and ADPKD. The expression of *PTGER2* affects the biologic behavior of various types of malignant tumors [27–29], which should be related to the enrichment of pathways in cancer in KEGG analysis. Otherwise, the role of prostaglandin E (2) (PGE2) in the cystogenesis in genetically nonorthologous models of ADPKD has already been studied. Liu et al. found that PGE2 could activate the aberrant signaling pathways in PC-1-deficient epithelia, and mediate the proliferation and chloride secretion in ADPKD cystic renal epithelia [30]. Elberg et al. have indicated the role of *PTGER2* in mediation of PGE2 effect on inducing formation of cyst through combined biochemical, pharmacological, and functional analyses in ADPKD [31]. This further confirms the reliability of our approach on pathogenic genes screening in ADPKD, which may be applicable in other diseases. It also indirectly supports the reliability of *ACOT13* as a potential ADPKD-related gene. However, the underlying biological function of *ACOT13* in ADPKD still warrants further studies.

## Conclusions

In this study, we identified two potential ADPKD-related genes, including *ACOT13* and *PTGER2*, by analyzing the WES results of four ADPKD patients and two healthy family members combining with the gene expression data from GEO database. Our results may be helpful for further studies in the underlying pathologic causes of ADPKD. However, due to the small number of patients in our study, there remains some uncertainty about the potential role of *ACOT13* and *PTGER2*, which still warrants further studies.

## Supplementary Information

**Additional file 1: Table S1.** Clinical details of ADPKD patients.

**Additional file 2: Table S2.** Raw molecular data.

## Data Availability

All data generated and analysed during this study are included in this published article and its additional information files.
