# Supplementary material for: Identification of ACOT13 and PTGER2 as novel candidate genes of autosomal dominant polycystic kidney disease through whole exome sequencing
Source: Eur J Med Res. 2021 Dec 9;26:142. doi: 10.1186/s40001-021-00613-8 (PMC8656035; doi:10.1186/s40001-021-00613-8)
Supplement: Supplementary file 1 — Additional file 1: Table S1. Clinical details of ADPKD patients. [file 40001_2021_613_MOESM1_ESM.docx]

| Table S1 Clinical details of ADPKD patients | | | | | |
| --- | --- | --- | --- | --- | --- |
| No | Relationship | Gender | Age | Origin | Patient of Normal |
| 1 | Son | Male | 19 | China | Patient |
| 2 | Self | Female | 42 | China | Patient |
| 3 | Brother | Male | 40 | China | Normal |
| 4 | Daughter | Female | 8 | China | Normal |
| 5 | Brother | Male | 38 | China | Patient |
| 6 | Mother | Female | 72 | China | Patient |
