# Supplementary material for: Identification of ACOT13 and PTGER2 as novel candidate genes of autosomal dominant polycystic kidney disease through whole exome sequencing
Source: Eur J Med Res. 2021 Dec 9;26:142. doi: 10.1186/s40001-021-00613-8 (PMC8656035; doi:10.1186/s40001-021-00613-8)
Supplement: Supplementary file 2 — Additional file 2: Table S2. Raw molecular data. [file 40001_2021_613_MOESM2_ESM.docx]

Table S2 Raw Molecular Data

sample	chr	pos	ref	alt	af	gene	symbol	function

| exon | changeC | changeP | siftScore | siftPred |
| --- | --- | --- | --- | --- |
| exon6 | c.744delC | p.P248fs | . | . |
| exon5 | c.570delC | p.S190fs | . | . |

CTC1	1	1147102 CG	C	0.581 NM_003327	TNFRSF4	frameshift_deleti

on

CTC1	1	1245156 GC	G	0.538 NM_153339	PUSL1	frameshift_deleti

on

CTC1	1	1268995 GCTGCT GCTC

G	0.5 NM_152228	TAS1R3	nonframeshift_de exon6	c.1711_1719del p.571_573del	.	.

letion

frameshift_inserti exon3	c.290_291insT	p.P97fs	.	. on

| CTC1 | 1 | 1309587 T | TA | 0.246 NM_001127229 | AURKAIP1 |
| --- | --- | --- | --- | --- | --- |
| CTC1 | 1 | 1684347 C | CCCT | 0.833 NM_001198995 | NADK |
| CTC1 | 1 | 2433822 AG | A | 0.833 NM_014638 | PLCH2 |
| CTC1 | 1 | 3328476 CT | C | 0.667 NM_022114 | PRDM16 |
| CTC1 | 1 | 3328593 CG | C | 0.556 NM_022114 | PRDM16 |
| CTC1 | 1 | 7796591 TC | T | 0.38 NM_015215 | CAMTA1 |
| CTC1 | 1 | 8397939 AG | A | 0.436 NM_001080397 | SLC45A1 |
| CTC1 | 1 | 9662174 TG | T | 0.609 NM_001010866 | TMEM201 |
| CTC1 | 1 | 9782184 AC | A | 0.75 NM_005026 | PIK3CD |
| CTC1 | 1 | 10166507 TG | T | 0.162 NM_001105562 | UBE4B |
| CTC1 | 1 | 10477117 TC | T | 0.349 NM_002631 | PGD |
| CTC1 | 1 | 11015126 TG | T | 0.235 NM_001170754 | C1orf127 |
| CTC1 | 1 | 11307714 CG | C | 0.308 NM_004958 | MTOR |
| CTC1 | 1 | 11854005 TG | T | 0.355 NM_005957 | MTHFR |

GTGCTG

nonframeshift_in exon10	c.1240_1241ins p.G414delinsE	.	. sertion

frameshift_deleti	.	.

| exon21 | AGG  c.2934delG | G  p.E978fs |
| --- | --- | --- |
| exon9 | c.1716delT | p.A572fs |
| exon9 | c.1833delG | p.T611fs |
| exon13 | c.3255delC | p.I1085fs |
| exon6 | c.1662delG | p.Q554fs |
| exon6 | c.1005delG | p.L335fs |
| exon17 | c.2208delC | p.D736fs |
| exon7 | c.1063delG | p.A355fs |
| exon9 | c.919delC | p.P307fs |
| exon9 | c.895delC | p.Q299fs |
| exon8 | c.1192delC | p.R398fs |
| exon9 | c.1488delC | p.P496fs |

on

frameshift_deleti	.	.

on

frameshift_deleti	.	.

on

frameshift_deleti	.	.

on

frameshift_deleti	.	.

on

frameshift_deleti	.	.

on

frameshift_deleti	.	.

on

frameshift_deleti	.	.

on

frameshift_deleti	.	.

on

frameshift_deleti	.	.

on

frameshift_deleti	.	.

on

frameshift_deleti	.	.

on

CTC1	1	11866343 CTGCTG CAGGTG

A

G	0.179 NM_001256959	CLCN6	nonframeshift_de exon1	c.25_45del	p.9_15del	.	.

letion

frameshift_inserti exon26	c.6234dupT	p.A2078fs	.	. on

| CTC1 | 1 | 12364579 C | CT | 0.172 NM_015378 | VPS13D |
| --- | --- | --- | --- | --- | --- |
| CTC1 | 1 | 12820856 AT | A | 0.429 NM_152290 | C1orf158 |
| CTC1 | 1 | 14105121 TGAA | T | 0.308 NM_001007257 | PRDM2 |
| CTC1 | 1 | 15752492 AC | A | 0.938 NM_024329 | EFHD2 |
| CTC1 | 1 | 17084536 TGGAAC | T | 0.399 NM_001271733 | MST1L |

frameshift_deleti	exon4	c.558delT	p.H186fs	.	. on

nonframeshift_de exon3	c.229_231del	p.77_77del	.	. letion

frameshift_deleti	exon2	c.435delC	p.D145fs	.	. on

nonframeshift_de exon12	c.1556_1561del p.519_521del	.	. letion

CTC1	1	17085995 GCCCC	G	0.593 NM_001271733	MST1L	frameshift_deleti	.	.

| exon7 | c.898_901del | p.G300fs |
| --- | --- | --- |
| exon7 | c.894_897del | p.G298fs |
| exon6 | c.591delC | p.P197fs |
| exon13 | c.1504delC | p.R502fs |
| exon84 | c.12459delC | p.A4153fs |
| exon46 | c.6820delA | p.T2274fs |

on

CTC1	1	17085999 CCCCG	C	0.426 NM_001271733	MST1L	frameshift_deleti	.	.

on

frameshift_deleti	.	.

| CTC1 | 1 | 17350518 TG | T | 0.364 NM_003000 | SDHB |
| --- | --- | --- | --- | --- | --- |
| CTC1 | 1 | 17401395 CG | C | 1 NM_007365 | PADI2 |
| CTC1 | 1 | 19432270 TG | T | 0.286 NM_020765 | UBR4 |
| CTC1 | 1 | 19479806 GT | G | 0.273 NM_020765 | UBR4 |
| CTC1 | 1 | 19499580 G | GA | 0.571 NM_020765 | UBR4 |
| CTC1 | 1 | 19510643 CA | C | 1 NM_020765 | UBR4 |
| CTC1 | 1 | 21014225 AT | A | 0.815 NM_001122819 | KIF17 |
| CTC1 | 1 | 21585200 TG | T | 0.17 NM_001113347 | ECE1 |
| CTC1 | 1 | 21806636 TG | T | 0.202 NM_001256417 | NBPF3 |
| CTC1 | 1 | 21924504 GAC | G | 0.28 NM_001145658 | RAP1GAP |
| CTC1 | 1 | 21943956 AG | A | 0.326 NM_001145658 | RAP1GAP |
| CTC1 | 1 | 22005911 T | TA | 0.16 NM_032236 | USP48 |
| CTC1 | 1 | 22149825 CGT | C | 0.333 NM_005529 | HSPG2 |
| CTC1 | 1 | 22186686 GC | G | 0.94 NM_005529 | HSPG2 |
| CTC1 | 1 | 22838362 C | CA | 0.844 NM_014870 | ZBTB40 |
| CTC1 | 1 | 22838560 CAAG | C | 0.133 NM_014870 | ZBTB40 |
| CTC1 | 1 | 23111173 GA | G | 0.478 NM_004442 | EPHB2 |
| CTC1 | 1 | 23420433 A | AT | 0.667 NM_001142546 | LUZP1 |
| CTC1 | 1 | 23420684 C | CT | 0.385 NM_001142546 | LUZP1 |
| CTC1 | 1 | 23688917 CG | C | 1 NM_030634 | ZNF436 |
| CTC1 | 1 | 23850973 CT | C | 1 NM_004091 | E2F2 |
| CTC1 | 1 | 24122440 C | CA | 0.6 NM_001127621 | GALE |

on

frameshift_deleti	.	.

on

frameshift_deleti	.	.

on

frameshift_deleti	.	.

on

frameshift_inserti exon25	c.3298dupT	p.S1100fs	.	. on

stopgain	exon16	c.1964delT	p.L655X	.	.

frameshift_deleti	exon8	c.1593delA	p.K531fs	.	. on

frameshift_deleti	exon4	c.711delC	p.N237fs	.	. on

frameshift_deleti	exon10	c.1092delG	p.L364fs	.	. on

frameshift_deleti exon23	c.2123_2124del p.C708fs	.	. on

frameshift_deleti	exon7	c.325delC	p.L109fs	.	. on

stoploss	exon27	c.3106dupT	p.X1036delinsL .	.

frameshift_deleti exon97	c.13158_13159 p.T4386fs	.	.

on	del

frameshift_deleti	exon40	c.4997delG	p.G1666fs	.	. on

frameshift_inserti exon11	c.2197dupA	p.A732fs	.	. on

nonframeshift_de exon11	c.2395_2397del p.799_799del	.	. letion

frameshift_deleti	exon3	c.416delA	p.D139fs	.	. on

frameshift_inserti exon3	c.321dupA	p.S108fs	.	. on

frameshift_inserti exon3	c.70dupA	p.S24fs	.	. on

frameshift_deleti	exon3	c.957delC	p.S319fs	.	. on

frameshift_deleti	exon2	c.259delA	p.R87fs	.	. on

stoploss	exon11	c.1045dupT	p.X349delinsL	.	.

nonframeshift_de exon1	c.396_398del	p.132_133del	.	. letion

| CTC1 | 1 | 26349532 GCCT | G | 0.137 NM_004455 | EXTL1 |
| --- | --- | --- | --- | --- | --- |
| CTC1 | 1 | 26598557 GT | G | 0.828 NM_001281518 | CEP85 |
| CTC1 | 1 | 27240013 TG | T | 0.367 NM_021969 | NR0B2 |
| CTC1 | 1 | 27736473 G | GA | 0.25 NM_006990 | WASF2 |
| CTC1 | 1 | 27875352 AAGG | A | 0.227 NM_001029882 | AHDC1 |
| CTC1 | 1 | 27995747 CA | C | 0.417 NM_022872 | IFI6 |
| CTC1 | 1 | 28233497 TA | T | 0.184 NM_002946 | RPA2 |

frameshift_deleti	exon4	c.86delT	p.V29fs	.	. on

frameshift_deleti	exon1	c.418delC	p.Q140fs	.	. on

frameshift_inserti exon8	c.1051dupT	p.S351fs	.	. on

nonframeshift_de exon6	c.3272_3274del p.1091_1092de .	.

letion	l

frameshift_deleti	exon2	c.78delT	p.N26fs	.	. on

frameshift_deleti	exon4	c.274delT	p.Y92fs	.	. on

| CTC1 | 1 | 31349737 | TG | T | 0.286 | NM_014654 | SDC3 | frameshift_deleti  on | exon3 | c.531delC | p.A177fs | . | . |
| --- | --- | --- | --- | --- | --- | --- | --- | --- | --- | --- | --- | --- | --- |
| CTC1 | 1 | 31501678 | G | GA | 0.36 | NM_001020658 | PUM1 | frameshift_inserti  on | exon3 | c.396dupT | p.Q133fs | . | . |
| CTC1 | 1 | 32156800 | TG | T | 0.75 | NM_001856 | COL16A1 | frameshift_deleti  on | exon20 | c.1425delC | p.P475fs | . | . |
| CTC1 | 1 | 32162829 | GT | G | 0.727 | NM_001856 | COL16A1 | frameshift_deleti  on | exon7 | c.729delA | p.L243fs | . | . |
| CTC1 | 1 | 32221861 | AGTT | A | 1 | NM_001703 | BAI2 | nonframeshift_de  letion | exon4 | c.574_576del | p.192_192del | . | . |
| CTC1 | 1 | 32623872 | TA | T | 1 | NM_012316 | KPNA6 | frameshift_deleti  on | exon5 | c.337delA | p.S113fs | . | . |
| CTC1 | 1 | 33583659 | TC | T | 0.658 | NM_052998 | ADC | frameshift_deleti  on | exon11 | c.1187delC | p.S396fs | . | . |
| CTC1 | 1 | 35223750 | GT | G | 0.274 | NM_005268 | GJB5 | stoploss | exon2 | c.820delT | p.X274E | . | . |
| CTC1 | 1 | 35476567 | CT | C | 0.413 | NM_007167 | ZMYM6 | frameshift_deleti on | exon9 | c.1132delA | p.S378fs | . | . |
| CTC1 | 1 | 35484992 | C | CGGG | 0.182 | NM_007167 | ZMYM6 | nonframeshift_in  sertion | exon4 | c.389_390insCC  C | p.L130delinsLP | . | . |
| CTC1 | 1 | 36565659 | TG | T | 0.199 | NM_005202 | COL8A2 | frameshift_deleti  on | exon1 | c.184delC | p.Q62fs | . | . |
| CTC1 | 1 | 36752589 | GC | G | 0.522 | NM_005119 | THRAP3 | frameshift_deleti  on | exon4 | c.759delC | p.S253fs | . | . |
| CTC1 | 1 | 38334174 | TG | T | 0.5 | NM_005540 | INPP5B | frameshift_deleti  on | exon20 | c.2299delC | p.Q767fs | . | . |
| CTC1 | 1 | 38488383 | GA | G | 0.987 | NM_016037 | UTP11L | frameshift_deleti  on | exon7 | c.581delA | p.E194fs | . | . |
| CTC1 | 1 | 39824852 | GA | G | 0.315 | NM_012090 | MACF1 | frameshift_deleti  on | exon41 | c.5975delA | p.E1992fs | . | . |
| CTC1 | 1 | 39878319 | AG | A | 0.259 | NM_015038 | KIAA0754 | frameshift_deleti  on | exon1 | c.2383delG | p.G795fs | . | . |
| CTC1 | 1 | 40981007 | CTG | C | 0.973 | NM_022774 | EXO5 | frameshift_deleti  on | exon3 | c.792_793del | p.S264fs | . | . |
| CTC1 | 1 | 41475238 | GA | G | 0.436 | NM_001905 | CTPS1 | frameshift_deleti  on | exon17 | c.1669delA | p.K557fs | . | . |
| CTC1 | 1 | 43621972 | TC | T | 0.6 | NM_001101376 | FAM183A | frameshift_deleti  on | exon4 | c.394delC | p.H132fs | . | . |
| CTC1 | 1 | 43850131 | TG | T | 0.222 | NM_052877 | MED8 | frameshift_deleti  on | exon8 | c.895delC | p.Q299fs | . | . |
| CTC1 | 1 | 46079794 | GA | G | 0.357 | NM_001195193 | NASP | frameshift_deleti  on | exon6 | c.1342delA | p.I448fs | . | . |
| CTC1 | 1 | 47280746 | GAT | G | 0.643 | NM_000779 | CYP4B1 | frameshift_deleti  on | exon8 | c.881_882del | p.D294fs | . | . |
| CTC1 | 1 | 47400184 | GC | G | 0.769 | NM_000778 | CYP4A11 | frameshift_deleti  on | exon7 | c.837delG | p.E279fs | . | . |
| CTC1 | 1 | 48699331 | GGT | G | 0.224 | NM_001011547 | SLC5A9 | frameshift_deleti  on | exon9 | c.1039_1040del | p.V347fs | . | . |
| CTC1 | 1 | 49224822 | TG | T | 0.333 | NM_024603 | BEND5 | frameshift_deleti  on | exon3 | c.494delC | p.P165fs | . | . |
| CTC1 | 1 | 52306063 | ATCT | A | 1 | NM_001101662 | NRD1 | nonframeshift_de  letion | exon2 | c.462_464del | p.154_155del | . | . |
| CTC1 | 1 | 52823559 | TG | T | 0.31 | NM_032449 | CC2D1B | frameshift_deleti  on | exon14 | c.1490delC | p.P497fs | . | . |
| CTC1 | 1 | 53513546 | CA | C | 0.632 | NM_001007250 | SCP2 | frameshift_deleti  on | exon3 | c.144delA | p.A48fs | . | . |
| CTC1 | 1 | 54325814 | CA | C | 0.6 | NM_018982 | YIPF1 | frameshift_deleti  on | exon10 | c.843delT | p.F281fs | . | . |
| CTC1 | 1 | 54344046 | CA | C | 0.326 | NM_018982 | YIPF1 | frameshift_deleti  on | exon6 | c.305delT | p.L102fs | . | . |
| CTC1 | 1 | 54605318 | T | TGG | 1 | NM_201546 | CDCP2 | frameshift_inserti  on | exon4 | c.1224_1225ins  CC | p.M409fs | . | . |
| CTC1 | 1 | 54605318 | T | TGGG | 1 | NM_201546 | CDCP2 | nonframeshift_in  sertion | exon4 | c.1224_1225ins  CCC | p.M409delinsP  M | . | . |
| CTC1 | 1 | 54605326 | G | GGC | 0.478 | NM_201546 | CDCP2 | frameshift_inserti  on | exon4 | c.1216_1217ins  GC | p.A406fs | . | . |
| CTC1 | 1 | 57173317 | TCG | T | 0.6 | NM_006252 | PRKAA2 | frameshift_deleti  on | exon9 | c.1591_1592del | p.R531fs | . | . |
| CTC1 | 1 | 57431593 | CT | C | 0.636 | NM_000066 | C8B | frameshift_deleti  on | exon1 | c.28delA | p.R10fs | . | . |
| CTC1 | 1 | 62299427 | AG | A | 0.41 | NM_176877 | INADL | frameshift_deleti  on | exon17 | c.2083delG | p.G695fs | . | . |
| CTC1 | 1 | 62675654 | T | TGGA | 0.241 | NM_019079 | L1TD1 | nonframeshift_in  sertion | exon4 | c.1208_1209ins  GGA | p.L403delinsLE | . | . |
| CTC1 | 1 | 62675665 | G | GAGA | 0.188 | NM_019079 | L1TD1 | nonframeshift_in  sertion | exon4 | c.1219_1220ins  AGA | p.E407delinsEK | . | . |
| CTC1 | 1 | 62740601 | TG | T | 0.6 | NM_181712 | KANK4 | frameshift_deleti  on | exon3 | c.174delC | p.H58fs | . | . |
| CTC1 | 1 | 66384500 | CA | C | 0.333 | NM_001037341 | PDE4B | frameshift_deleti  on | exon3 | c.264delA | p.T88fs | . | . |
| CTC1 | 1 | 78426163 | TA | T | 0.469 | NM_003902 | FUBP1 | stopgain | exon15 | c.1361delT | p.L454X | . | . |
| CTC1 | 1 | 85279851 | GC | G | 0.259 | NM_012152 | LPAR3 | frameshift_deleti on | exon3 | c.739delG | p.A247fs | . | . |
| CTC1 | 1 | 85537632 | CA | C | 0.529 | NM_001288563 | WDR63 | frameshift_deleti  on | exon2 | c.9delA | p.P3fs | . | . |
| CTC1 | 1 | 85592223 | AC | A | 0.176 | NM_001288563 | WDR63 | frameshift_deleti  on | exon19 | c.2026delC | p.P676fs | . | . |
| CTC1 | 1 | 85724228 | C | CT | 0.238 | NM_198077 | C1orf52 | frameshift_inserti  on | exon2 | c.453dupA | p.G152fs | . | . |
| CTC1 | 1 | 86820492 | GT | G | 0.45 | NM_001184766 | ODF2L | frameshift_deleti  on | exon13 | c.1322delA | p.N441fs | . | . |
| CTC1 | 1 | 86904594 | AT | A | 0.281 | NM_006536 | CLCA2 | frameshift_deleti  on | exon7 | c.1009delT | p.F337fs | . | . |
| CTC1 | 1 | 87045896 | TCCTAC  A | T | 1 | NM_012128 | CLCA4 | nonframeshift_de  letion | exon14 | c.2629_2634del | p.877_878del | . | . |
| CTC1 | 1 | 89448455 | AC | A | 0.168 | NM_019610 | RBMXL1 | stopgain | exon2 | c.1054delG | p.V352X | . | . |
| CTC1 | 1 | 89729541 | CA | C | 0.8 | NM_001134486 | GBP5 | frameshift_deleti on | exon8 | c.1239delT | p.F413fs | . | . |
| CTC1 | 1 | 90400962 | AC | A | 0.714 | NM_001134479 | LRRC8D | frameshift_deleti  on | exon3 | c.2336delC | p.T779fs | . | . |
| CTC1 | 1 | 94041588 | GC | G | 0.714 | NM_001261410 | BCAR3 | frameshift_deleti  on | exon6 | c.1511delG | p.R504fs | . | . |
| CTC1 | 1 | 94643240 | CA | C | 0.6 | NM_004815 | ARHGAP29 | frameshift_deleti  on | exon22 | c.2832delT | p.F944fs | . | . |
| CTC1 | 1 | 98348833 | TC | T | 0.364 | NM_000110 | DPYD | frameshift_deleti  on | exon2 | c.136delG | p.D46fs | . | . |
| CTC1 | 1 | 100154941 | AT | A | 0.376 | NM_017734 | PALMD | frameshift_deleti  on | exon7 | c.1126delT | p.F376fs | . | . |
| CTC1 | 1 | 100618027 | CT | C | 0.5 | NM_001256387 | LRRC39 | frameshift_deleti  on | exon8 | c.865delA | p.S289fs | . | . |
| CTC1 | 1 | 103343610 | CG | C | 0.14 | NM_080630 | COL11A1 | frameshift_deleti  on | exon65 | c.5037delC | p.F1679fs | . | . |

| CTC1 | 1 | 109200195 | TA | T | 0.333 | NM_001102592 | HENMT1 | frameshift_deleti  on | exon3 | c.27delT | p.S9fs | . | . |
| --- | --- | --- | --- | --- | --- | --- | --- | --- | --- | --- | --- | --- | --- |
| CTC1 | 1 | 109554060 | AG | A | 0.28 | NM_001142550 | WDR47 | frameshift_deleti  on | exon5 | c.628delC | p.L210fs | . | . |
| CTC1 | 1 | 109840227 | GT | G | 0.375 | NM_001010985 | MYBPHL | frameshift_deleti  on | exon3 | c.246delA | p.K82fs | . | . |
| CTC1 | 1 | 110950322 | GA | G | 0.75 | NM_006402 | LAMTOR5 | frameshift_deleti  on | exon1 | c.166delT | p.S56fs | . | . |
| CTC1 | 1 | 110950324 | GC | G | 0.692 | NM_006402 | LAMTOR5 | frameshift_deleti  on | exon1 | c.164delG | p.R55fs | . | . |
| CTC1 | 1 | 110996673 | CG | C | 0.5 | NM_032414 | PROK1 | frameshift_deleti  on | exon2 | c.164delG | p.R55fs | . | . |
| CTC1 | 1 | 111957282 | ATC | A | 0.286 | NM_002557 | OVGP1 | frameshift_deleti  on | exon11 | c.1839_1840del | p.Q613fs | . | . |
| CTC1 | 1 | 112991643 | AT | A | 0.571 | NM_018704 | CTTNBP2NL | frameshift_deleti  on | exon4 | c.180delT | p.D60fs | . | . |
| CTC1 | 1 | 114267494 | AT | A | 0.571 | NM_006608 | PHTF1 | frameshift_deleti  on | exon6 | c.509delA | p.N170fs | . | . |
| CTC1 | 1 | 119965065 | TC | T | 0.173 | NM_000198 | HSD3B2 | frameshift_deleti  on | exon4 | c.942delC | p.F314fs | . | . |
| CTC1 | 1 | 120437253 | TG | T | 0.207 | NM_021794 | ADAM30 | frameshift_deleti  on | exon1 | c.1706delC | p.P569fs | . | . |
| CTC1 | 1 | 120437410 | TC | T | 0.655 | NM_021794 | ADAM30 | frameshift_deleti  on | exon1 | c.1549delG | p.E517fs | . | . |
| CTC1 | 1 | 120438701 | GT | G | 0.25 | NM_021794 | ADAM30 | frameshift_deleti  on | exon1 | c.258delA | p.R86fs | . | . |
| CTC1 | 1 | 120458108 | G | GA | 0.208 | NM_024408 | NOTCH2 | frameshift_inserti  on | exon34 | c.7236dupT | p.P2413fs | . | . |
| CTC1 | 1 | 120460348 | A | AT | 0.304 | NM_024408 | NOTCH2 | frameshift_inserti  on | exon33 | c.5966dupA | p.N1989fs | . | . |
| CTC1 | 1 | 144879278 | CT | C | 0.474 | NM_001198834 | PDE4DIP | frameshift_deleti  on | exon27 | c.4171delA | p.S1391fs | . | . |
| CTC1 | 1 | 144923728 | GT | G | 0.217 | NM_001002811 | PDE4DIP | frameshift_deleti  on | exon2 | c.1218delA | p.E406fs | . | . |
| CTC1 | 1 | 145562330 | TG | T | 1 | NM_001280799 | ANKRD35 | frameshift_deleti  on | exon8 | c.1749delG | p.V583fs | . | . |
| CTC1 | 1 | 145761340 | GA | G | 0.388 | NM_001201326 | PDZK1 | frameshift_deleti  on | exon5 | c.821delA | p.E274fs | . | . |
| CTC1 | 1 | 150129150 | TC | T | 0.267 | NM_016274 | PLEKHO1 | frameshift_deleti  on | exon4 | c.365delC | p.S122fs | . | . |
| CTC1 | 1 | 150199039 | CTCCTCT | C | 1 | NM_001136478 | ANP32E | nonframeshift_de  letion | exon4 | c.453_458del | p.151_153del | . | . |
| CTC1 | 1 | 151237697 | AT | A | 0.396 | NM_002810 | PSMD4 | frameshift_deleti  on | exon5 | c.426delT | p.N142fs | . | . |
| CTC1 | 1 | 151260656 | TGCCTG  CCTTGG | T | 0.806 | NM_020832 | ZNF687 | frameshift_deleti  on | exon2 | c.1890_1900del | p.L630fs | . | . |
| CTC1 | 1 | 151378050 | TC | T | 0.577 | NM_145796 | POGZ | frameshift_deleti  on | exon17 | c.3175delG | p.E1059fs | . | . |
| CTC1 | 1 | 151380943 | GC | G | 0.345 | NM_145796 | POGZ | frameshift_deleti  on | exon12 | c.1890delG | p.L630fs | . | . |
| CTC1 | 1 | 151491124 | TA | T | 0.446 | NM_020770 | CGN | frameshift_deleti  on | exon2 | c.130delA | p.K44fs | . | . |
| CTC1 | 1 | 151491159 | TGC | T | 0.136 | NM_020770 | CGN | frameshift_deleti  on | exon2 | c.165_166del | p.V55fs | . | . |
| CTC1 | 1 | 151774724 | C | CCA | 0.259 | NM_001004432 | LINGO4 | frameshift_inserti  on | exon2 | c.456_457insTG | p.G153fs | . | . |
| CTC1 | 1 | 152195728 | AT | A | 0.971 | NM_001009931 | HRNR | frameshift_deleti  on | exon2 | c.1delA | p.M1fs | . | . |
| CTC1 | 1 | 152681680 | C | CAGCT  CTGGG GGCTG | 0.75 | NM_178356 | LCE4A | nonframeshift_in sertion | exon1 | c.129_130insA  GCTCTGGGGG CTGCTGT | p.S43delinsSSS GGCC | . | . |
| CTC1 | 1 | 152681683 | C | CTCTG  GGGGC TGCTGT | 0.696 | NM_178356 | LCE4A | nonframeshift_in sertion | exon1 | c.132_133insTC  TGGGGGCTGC TGTAGT | p.S44delinsSSG GCCS | . | . |
| CTC1 | 1 | 152681686 | T | TGGGG  GCTGC TGTAG | 0.63 | NM_178356 | LCE4A | nonframeshift_in sertion | exon1 | c.135_136insG  GGGGCTGCTG TAGCTGG | p.S45delinsSG GCCSW | . | . |
| CTC1 | 1 | 152785189 | AC | A | 0.382 | NM_178349 | LCE1B | frameshift_deleti  on | exon1 | c.268delC | p.P90fs | . | . |
| CTC1 | 1 | 152975658 | AGAGCC  AGGCTG TACCAA | A | 1 | NM_001097589 | SPRR3 | nonframeshift_de letion | exon2 | c.163_186del | p.55_62del | . | . |
| CTC1 | 1 | 153536250 | AT | A | 0.257 | NM_005978 | S100A2 | stopgain | exon2 | c.100delA | p.M34X | . | . |
| CTC1 | 1 | 153661961 | CT | C | 0.569 | NM_000906 | NPR1 | frameshift_deleti on | exon18 | c.2722delT | p.F908fs | . | . |
| CTC1 | 1 | 153748613 | GGC | G | 0.375 | NM_024330 | SLC27A3 | frameshift_deleti  on | exon1 | c.782_783del | p.G261fs | . | . |
| CTC1 | 1 | 153749547 | AG | A | 0.222 | NM_024330 | SLC27A3 | frameshift_deleti  on | exon3 | c.1029delG | p.K343fs | . | . |
| CTC1 | 1 | 154218810 | TC | T | 0.415 | NM_001127320 | UBAP2L | frameshift_deleti  on | exon11 | c.974delC | p.S325fs | . | . |
| CTC1 | 1 | 154474013 | TG | T | 0.833 | NM_001010846 | SHE | frameshift_deleti  on | exon1 | c.489delC | p.S163fs | . | . |
| CTC1 | 1 | 154842199 | G | GGCTG  CTGCT | 1 | NM_001204087 | KCNN3 | nonframeshift_in  sertion | exon1 | c.241_242insA  GCAGCAGCAG | p.P81delinsQQ  QQQP | . | . |
| CTC1 | 1 | 154842199 | G | GGCTG  CTGCT | 1 | NM_001204087 | KCNN3 | nonframeshift_in  sertion | exon1 | c.241_242insA  GCAGCAGCAG | p.P81delinsQQ  QQP | . | . |
| CTC1 | 1 | 154842330 | TTGC | T | 0.182 | NM_001204087 | KCNN3 | nonframeshift_de  letion | exon1 | c.108_110del | p.36_37del | . | . |
| CTC1 | 1 | 155221332 | A | AG | 0.417 | NM_198264 | FAM189B | frameshift_inserti  on | exon4 | c.566dupC | p.P189fs | . | . |
| CTC1 | 1 | 155292373 | CT | C | 0.543 | NM_001105203 | RUSC1 | frameshift_deleti  on | exon2 | c.810delT | p.P270fs | . | . |
| CTC1 | 1 | 155887375 | GGGGGC  T | G | 0.667 | NM_014949 | KIAA0907 | nonframeshift_de  letion | exon11 | c.1349_1354del | p.450_452del | . | . |
| CTC1 | 1 | 156046686 | AGAG | A | 0.165 | NM_001093725 | MEX3A | nonframeshift_de  letion | exon2 | c.1239_1241del | p.413_414del | . | . |
| CTC1 | 1 | 156233251 | G | GA | 0.333 | NM_015327 | SMG5 | frameshift_inserti  on | exon13 | c.1965dupT | p.P656fs | . | . |
| CTC1 | 1 | 156281901 | G | GA | 0.417 | NM_001008800 | CCT3 | frameshift_inserti  on | exon9 | c.971dupT | p.F324fs | . | . |
| CTC1 | 1 | 156509668 | TC | T | 0.65 | NM_178229 | IQGAP3 | frameshift_deleti  on | exon24 | c.2853delG | p.Q951fs | . | . |
| CTC1 | 1 | 156563290 | AT | A | 1 | NM_144772 | APOA1BP | frameshift_deleti  on | exon5 | c.608delT | p.I203fs | . | . |
| CTC1 | 1 | 156565049 | A | AAC | 1 | NM_015590 | GPATCH4 | frameshift_inserti  on | exon8 | c.1083_1084ins  GT | p.F362fs | . | . |
| CTC1 | 1 | 156737961 | TG | T | 0.268 | NM_005973 | PRCC | frameshift_deleti  on | exon1 | c.399delG | p.L133fs | . | . |
| CTC1 | 1 | 156917982 | AT | A | 0.376 | NM_014784 | ARHGEF11 | frameshift_deleti  on | exon23 | c.2023delA | p.M675fs | . | . |

| CTC1 | 1 | 156926295 | CCT | C | 0.188 | NM_014784 | ARHGEF11 | frameshift_deleti  on | exon18 | c.1466_1467del | p.E489fs | . | . |
| --- | --- | --- | --- | --- | --- | --- | --- | --- | --- | --- | --- | --- | --- |
| CTC1 | 1 | 156933038 | AC | A | 0.827 | NM_014784 | ARHGEF11 | frameshift_deleti  on | exon12 | c.976delG | p.V326fs | . | . |
| CTC1 | 1 | 157556227 | AC | A | 0.289 | NM_031282 | FCRL4 | frameshift_deleti  on | exon6 | c.865delG | p.V289fs | . | . |
| CTC1 | 1 | 158913724 | G | GA | 0.346 | NM_152501 | PYHIN1 | frameshift_inserti  on | exon6 | c.1148dupA | p.E383fs | . | . |
| CTC1 | 1 | 159778044 | G | GA | 0.194 | NM_001004310 | FCRL6 | frameshift_inserti  on | exon3 | c.130dupA | p.K43fs | . | . |
| CTC1 | 1 | 160456936 | AT | A | 0.32 | NM_001184716 | SLAMF6 | frameshift_deleti  on | exon6 | c.575delA | p.N192fs | . | . |
| CTC1 | 1 | 161089086 | AT | A | 0.218 | NM_001185093 | NIT1 | frameshift_deleti  on | exon2 | c.217delT | p.F73fs | . | . |
| CTC1 | 1 | 161123850 | AC | A | 0.855 | NM_016406 | UFC1 | frameshift_deleti  on | exon1 | c.64delC | p.P22fs | . | . |
| CTC1 | 1 | 161495142 | GGA | G | 0.136 | NM_002155 | HSPA6 | frameshift_deleti  on | exon1 | c.695_696del | p.G232fs | . | . |
| CTC1 | 1 | 161496305 | TG | T | 0.633 | NM_002155 | HSPA6 | frameshift_deleti  on | exon1 | c.1858delG | p.G620fs | . | . |
| CTC1 | 1 | 162536100 | CA | C | 0.167 | NM_003115 | UAP1 | frameshift_deleti  on | exon2 | c.243delA | p.T81fs | . | . |
| CTC1 | 1 | 165182886 | AG | A | 0.239 | NM_001174069 | LMX1A | frameshift_deleti  on | exon5 | c.660delC | p.P220fs | . | . |
| CTC1 | 1 | 165380301 | TC | T | 0.18 | NM_001256571 | RXRG | frameshift_deleti  on | exon4 | c.298delG | p.E100fs | . | . |
| CTC1 | 1 | 169347750 | TA | T | 0.187 | NM_003666 | BLZF1 | frameshift_deleti  on | exon4 | c.652delA | p.K218fs | . | . |
| CTC1 | 1 | 169494076 | CA | C | 0.375 | NM_000130 | F5 | frameshift_deleti  on | exon19 | c.5786delT | p.L1929fs | . | . |
| CTC1 | 1 | 169529847 | A | AT | 0.301 | NM_000130 | F5 | frameshift_inserti  on | exon4 | c.530dupA | p.N177fs | . | . |
| CTC1 | 1 | 170993648 | T | TA | 0.19 | NM_001163629 | MROH9 | frameshift_inserti  on | exon18 | c.2022dupA | p.L674fs | . | . |
| CTC1 | 1 | 171244501 | TA | T | 0.8 | NM_001282692 | FMO1 | frameshift_deleti  on | exon3 | c.351delA | p.V117fs | . | . |
| CTC1 | 1 | 171621514 | TG | T | 0.23 | NM_000261 | MYOC | frameshift_deleti  on | exon1 | c.237delC | p.S79fs | . | . |
| CTC1 | 1 | 171753277 | AC | A | 0.19 | NM_014955 | METTL13 | frameshift_deleti  on | exon2 | c.294delC | p.H98fs | . | . |
| CTC1 | 1 | 173155874 | CT | C | 0.273 | NM_003326 | TNFSF4 | frameshift_deleti  on | exon3 | c.332delA | p.Q111fs | . | . |
| CTC1 | 1 | 175331839 | GC | G | 0.307 | NM_003285 | TNR | frameshift_deleti  on | exon14 | c.2813delG | p.G938fs | . | . |
| CTC1 | 1 | 176563694 | CA | C | 0.311 | NM_020318 | PAPPA2 | frameshift_deleti  on | exon3 | c.955delA | p.K319fs | . | . |
| CTC1 | 1 | 176738917 | CA | C | 0.189 | NM_020318 | PAPPA2 | frameshift_deleti  on | exon16 | c.4499delA | p.Q1500fs | . | . |
| CTC1 | 1 | 179338089 | GA | G | 0.273 | NM_144696 | AXDND1 | frameshift_deleti  on | exon3 | c.253delA | p.K85fs | . | . |
| CTC1 | 1 | 180000572 | TTG | T | 0.4 | NM_014810 | CEP350 | frameshift_deleti  on | exon15 | c.3669_3670del | p.F1223fs | . | . |
| CTC1 | 1 | 180159679 | GC | G | 0.574 | NM_001004128 | QSOX1 | frameshift_deleti  on | exon10 | c.1253delC | p.A418fs | . | . |
| CTC1 | 1 | 181726067 | GC | G | 0.185 | NM_001205294 | CACNA1E | frameshift_deleti  on | exon29 | c.4078delC | p.Q1360fs | . | . |
| CTC1 | 1 | 182517549 | CA | C | 0.398 | NM_001137669 | RGSL1 | frameshift_deleti  on | exon16 | c.2768delA | p.Q923fs | . | . |
| CTC1 | 1 | 183511224 | GA | G | 0.467 | NM_001174061 | SMG7 | frameshift_deleti  on | exon13 | c.1304delA | p.E435fs | . | . |
| CTC1 | 1 | 186086700 | CA | C | 0.921 | NM_031935 | HMCN1 | frameshift_deleti  on | exon77 | c.11794delA | p.K3932fs | . | . |
| CTC1 | 1 | 186273951 | GTCC | G | 0.137 | NM_001127708 | PRG4 | nonframeshift_de  letion | exon5 | c.373_375del | p.125_125del | . | . |
| CTC1 | 1 | 186645737 | AG | A | 0.3 | NM_000963 | PTGS2 | frameshift_deleti  on | exon7 | c.831delC | p.V277fs | . | . |
| CTC1 | 1 | 196300368 | TA | T | 0.273 | NM_001287820 | KCNT2 | frameshift_deleti  on | exon17 | c.1870delT | p.Y624fs | . | . |
| CTC1 | 1 | 197112046 | AT | A | 0.643 | NM_001206846 | ASPM | frameshift_deleti  on | exon3 | c.1335delA | p.K445fs | . | . |
| CTC1 | 1 | 197404231 | AT | A | 0.22 | NM_001193640 | CRB1 | frameshift_deleti  on | exon7 | c.2903delT | p.I968fs | . | . |
| CTC1 | 1 | 198700790 | AT | A | 0.875 | NM_080921 | PTPRC | frameshift_deleti  on | exon15 | c.1427delT | p.I476fs | . | . |
| CTC1 | 1 | 200376709 | GT | G | 0.733 | NM_001281293 | ZNF281 | frameshift_deleti  on | exon2 | c.2124delA | p.K708fs | . | . |
| CTC1 | 1 | 200378174 | CT | C | 0.406 | NM_001281293 | ZNF281 | frameshift_deleti  on | exon2 | c.659delA | p.K220fs | . | . |
| CTC1 | 1 | 200378731 | TGCC | T | 0.146 | NM_001281293 | ZNF281 | nonframeshift_de  letion | exon2 | c.100_102del | p.34_34del | . | . |
| CTC1 | 1 | 201332436 | AC | A | 0.637 | NM_001001432 | TNNT2 | frameshift_deleti  on | exon10 | c.542delG | p.G181fs | . | . |
| CTC1 | 1 | 203186947 | G | GCCAA  GACCA TGGCC CCGCC | 0.141 | NM_001256125 | CHIT1 | nonframeshift_in sertion | exon9 | c.1018_1019ins  GACTGGGCGG GGCCATGGTC TTGG | p.A340delinsGL GGAMVLA | . | . |
| CTC1 | 1 | 203186950 | C | CAGAC  CATGG CCCCG CCCAG | 0.429 | NM_001256125 | CHIT1 | stopgain | exon9 | c.1015_1016ins  AGGGACTGGG CGGGGCCATG GTCT | p.W339delinsX | . | . |
| CTC1 | 1 | 203186960 | C | CCCCG  CCCAG TCCCTA GACCA | 0.309 | NM_001256125 | CHIT1 | stopgain | exon9 | c.1005_1006ins  TCCATGGTCTA GGGACTGGGC GGG | p.A336delinsS MVX | . | . |
| CTC1 | 1 | 203690484 | A | AT | 0.148 | NM_001001396 | ATP2B4 | frameshift_inserti  on | exon17 | c.2759dupT | p.I920fs | . | . |
| CTC1 | 1 | 204433601 | TC | T | 0.474 | NM_002646 | PIK3C2B | frameshift_deleti  on | exon5 | c.1165delG | p.E389fs | . | . |
| CTC1 | 1 | 205273029 | AC | A | 0.246 | NM_030952 | NUAK2 | stopgain | exon7 | c.1435delG | p.V479X | . | . |
| CTC1 | 1 | 205273268 | AG | A | 0.256 | NM_030952 | NUAK2 | frameshift_deleti on | exon7 | c.1196delC | p.P399fs | . | . |
| CTC1 | 1 | 206809096 | TG | T | 1 | NM_003582 | DYRK3 | frameshift_deleti  on | exon1 | c.49delG | p.G17fs | . | . |
| CTC1 | 1 | 207791439 | CA | C | 0.357 | NM_000573 | CR1 | frameshift_deleti  on | exon34 | c.5564delA | p.Q1855fs | . | . |
| CTC1 | 1 | 207871200 | AT | A | 0.316 | NM_175710 | CR1L | frameshift_deleti  on | exon7 | c.1125delT | p.D375fs | . | . |
| CTC1 | 1 | 207930513 | TAC | T | 0.117 | NM_002389 | CD46 | frameshift_deleti  on | exon2 | c.253_254del | p.T85fs | . | . |
| CTC1 | 1 | 209605636 | TAGCAG  CAGCAG | T | 0.947 | NM_001104548 | MIR205HG | nonframeshift_de  letion | exon4 | c.252_263del | p.84_88del | . | . |

| CTC1 | 1 | 211751483 | CGTGGC  CGTGGC CGTGAC CCCCGT | C | 1 | NM_021194 | SLC30A1 | nonframeshift_de letion | exon1 | c.445_471del | p.149_157del | . | . |
| --- | --- | --- | --- | --- | --- | --- | --- | --- | --- | --- | --- | --- | --- |
| CTC1 | 1 | 213031885 | G | GT | 0.216 | NM_014053 | FLVCR1 | frameshift_inserti  on | exon1 | c.92dupT | p.V31fs | . | . |
| CTC1 | 1 | 213403864 | AC | A | 0.711 | NM_001136138 | RPS6KC1 | frameshift_deleti  on | exon8 | c.1034delC | p.T345fs | . | . |
| CTC1 | 1 | 214557048 | GCCT | G | 0.161 | NM_005401 | PTPN14 | nonframeshift_de  letion | exon13 | c.2147_2149del | p.716_717del | . | . |
| CTC1 | 1 | 214820722 | CA | C | 0.667 | NM_016343 | CENPF | frameshift_deleti  on | exon13 | c.7810delA | p.K2604fs | . | . |
| CTC1 | 1 | 215956179 | CT | C | 0.231 | NM_206933 | USH2A | frameshift_deleti  on | exon53 | c.10485delA | p.K3495fs | . | . |
| CTC1 | 1 | 216062030 | AC | A | 0.381 | NM_206933 | USH2A | frameshift_deleti  on | exon41 | c.7960delG | p.V2654fs | . | . |
| CTC1 | 1 | 216420542 | CA | C | 0.833 | NM_007123 | USH2A | frameshift_deleti  on | exon13 | c.2193delT | p.F731fs | . | . |
| CTC1 | 1 | 216595603 | CA | C | 0.444 | NM_007123 | USH2A | frameshift_deleti  on | exon2 | c.75delT | p.F25fs | . | . |
| CTC1 | 1 | 219347259 | GC | G | 0.297 | NM_138794 | LYPLAL1 | frameshift_deleti  on | exon1 | c.28delC | p.Q10fs | . | . |
| CTC1 | 1 | 222717490 | GT | G | 0.449 | NM_024746 | HHIPL2 | frameshift_deleti  on | exon2 | c.362delA | p.N121fs | . | . |
| CTC1 | 1 | 222742869 | AT | A | 0.289 | NM_139352 | TAF1A | frameshift_deleti  on | exon6 | c.534delA | p.K178fs | . | . |
| CTC1 | 1 | 223178990 | TA | T | 0.552 | NM_032890 | DISP1 | frameshift_deleti  on | exon10 | c.4252delA | p.K1418fs | . | . |
| CTC1 | 1 | 223285692 | G | GC | 0.173 | NM_003268 | TLR5 | frameshift_inserti  on | exon6 | c.681dupG | p.L228fs | . | . |
| CTC1 | 1 | 223536702 | TTGC | T | 0.914 | NM_001037175 | SUSD4 | nonframeshift_de  letion | exon2 | c.63_65del | p.21_22del | . | . |
| CTC1 | 1 | 223536728 | G | GA | 0.5 | NM_001037175 | SUSD4 | frameshift_inserti  on | exon2 | c.39dupT | p.L14fs | . | . |
| CTC1 | 1 | 223934832 | CT | C | 0.4 | NM_001146068 | CAPN2 | frameshift_deleti  on | exon5 | c.461delT | p.L154fs | . | . |
| CTC1 | 1 | 225211337 | CA | C | 0.625 | NM_001145154 | DNAH14 | frameshift_deleti  on | exon11 | c.1129delA | p.K377fs | . | . |
| CTC1 | 1 | 225334832 | AG | A | 0.489 | NM_001373 | DNAH14 | frameshift_deleti  on | exon30 | c.4771delG | p.V1591fs | . | . |
| CTC1 | 1 | 228559805 | CA | C | 0.5 | NM_001098623 | OBSCN | frameshift_deleti  on | exon94 | c.21327delA | p.P7109fs | . | . |
| CTC1 | 1 | 228879065 | AC | A | 0.223 | NM_021205 | RHOU | frameshift_deleti  on | exon3 | c.356delC | p.T119fs | . | . |
| CTC1 | 1 | 233363045 | TG | T | 0.267 | NM_014801 | PCNXL2 | stopgain | exon10 | c.2430delC | p.Y810X | . | . |
| CTC1 | 1 | 235582782 | AT | A | 0.294 | NM_001287802 | TBCE | frameshift_deleti on | exon4 | c.28delT | p.C10fs | . | . |
| CTC1 | 1 | 236721685 | GA | G | 0.213 | NM_018072 | HEATR1 | frameshift_deleti  on | exon36 | c.5055delT | p.N1685fs | . | . |
| CTC1 | 1 | 236899899 | TC | T | 0.992 | NM_001278344 | ACTN2 | frameshift_deleti  on | exon10 | c.91delC | p.P31fs | . | . |
| CTC1 | 1 | 237052633 | GT | G | 0.178 | NM_000254 | MTR | frameshift_deleti  on | exon28 | c.3005delT | p.V1002fs | . | . |
| CTC1 | 1 | 240976930 | T | TC | 0.5 | NM_001282773 | RGS7 | frameshift_inserti  on | exon11 | c.784dupG | p.E262fs | . | . |
| CTC1 | 1 | 241842804 | A | AT | 0.222 | NM_144625 | WDR64 | frameshift_inserti  on | exon5 | c.501_502insT | p.T167fs | . | . |
| CTC1 | 1 | 243289805 | AT | A | 0.227 | NM_001042404 | CEP170 | frameshift_deleti  on | exon19 | c.4406delA | p.H1469fs | . | . |
| CTC1 | 1 | 247978541 | AAGG | A | 1 | NM_001001966 | OR14A16 | nonframeshift_de  letion | exon1 | c.488_490del | p.163_164del | . | . |
| CTC1 | 1 | 248903121 | CT | C | 0.182 | NM_001085474 | LYPD8 | frameshift_deleti  on | exon1 | c.30delA | p.K10fs | . | . |
| CTC1 | 2 | 1670244 | CA | C | 0.253 | NM_012293 | PXDN | frameshift_deleti  on | exon10 | c.1032delT | p.F344fs | . | . |
| CTC1 | 2 | 11725368 | GC | G | 0.3 | NM_014668 | GREB1 | frameshift_deleti  on | exon8 | c.984delC | p.C328fs | . | . |
| CTC1 | 2 | 17962848 | TA | T | 0.591 | NM_001130009 | GEN1 | frameshift_deleti  on | exon14 | c.2370delA | p.L790fs | . | . |
| CTC1 | 2 | 18765868 | CGCT | C | 0.171 | NM_001199103 | NT5C1B-  RDH14 | nonframeshift_de  letion | exon4 | c.638_640del | p.213_214del | . | . |
| CTC1 | 2 | 20478345 | T | TA | 0.741 | NM_001282752 | PUM2 | frameshift_inserti  on | exon12 | c.1787dupT | p.L596fs | . | . |
| CTC1 | 2 | 21231474 | CA | C | 0.577 | NM_000384 | APOB | frameshift_deleti  on | exon26 | c.8265delT | p.F2755fs | . | . |
| CTC1 | 2 | 24484497 | CT | C | 0.514 | NM_019595 | ITSN2 | frameshift_deleti  on | exon20 | c.2388delA | p.K796fs | . | . |
| CTC1 | 2 | 26653737 | AG | A | 0.333 | NM_145038 | DRC1 | frameshift_deleti  on | exon6 | c.763delG | p.E255fs | . | . |
| CTC1 | 2 | 27247058 | GA | G | 0.273 | NM_012326 | MAPRE3 | frameshift_deleti  on | exon4 | c.363delA | p.G121fs | . | . |
| CTC1 | 2 | 27263276 | CT | C | 0.7 | NM_001083590 | TMEM214 | frameshift_deleti  on | exon15 | c.1721delT | p.L574fs | . | . |
| CTC1 | 2 | 27307395 | AC | A | 0.32 | NM_007046 | EMILIN1 | frameshift_deleti  on | exon5 | c.2554delC | p.P852fs | . | . |
| CTC1 | 2 | 27355146 | AG | A | 0.425 | NM_013388 | PREB | frameshift_deleti  on | exon6 | c.877delC | p.L293fs | . | . |
| CTC1 | 2 | 27424236 | TC | T | 0.37 | NM_021095 | SLC5A6 | frameshift_deleti  on | exon15 | c.1629delG | p.L543fs | . | . |
| CTC1 | 2 | 27430505 | AC | A | 1 | NM_021095 | SLC5A6 | stopgain | exon3 | c.13delG | p.V5X | . | . |
| CTC1 | 2 | 27458468 | GTC | G | 0.217 | NM_004341 | CAD | frameshift_deleti on | exon25 | c.4035_4036del | p.S1345fs | . | . |
| CTC1 | 2 | 27459371 | TG | T | 0.474 | NM_004341 | CAD | frameshift_deleti  on | exon26 | c.4295delG | p.C1432fs | . | . |
| CTC1 | 2 | 27479701 | A | AGGG | 0.571 | NM_003459 | SLC30A3 | nonframeshift_in  sertion | exon6 | c.837_838insCC  C | p.S280delinsPS | . | . |
| CTC1 | 2 | 27900710 | CT | C | 0.6 | NM_018158 | SLC4A1AP | frameshift_deleti  on | exon8 | c.1683delT | p.T561fs | . | . |
| CTC1 | 2 | 28748775 | TG | T | 0.346 | NM_001170585 | PLB1 | frameshift_deleti  on | exon6 | c.289delG | p.E97fs | . | . |
| CTC1 | 2 | 29052115 | T | TA | 0.294 | NM_001008779 | SPDYA | frameshift_inserti  on | exon4 | c.483dupA | p.L161fs | . | . |
| CTC1 | 2 | 29287926 | C | CGCT | 0.438 | NM_001029883 | C2orf71 | nonframeshift_in  sertion | exon2 | c.3675_3676ins  AGC | p.E1226delinsS  E | . | . |
| CTC1 | 2 | 29287932 | T | TGCC | 0.352 | NM_001029883 | C2orf71 | nonframeshift_in  sertion | exon2 | c.3669_3670ins  GGC | p.S1224delinsG  S | . | . |
| CTC1 | 2 | 30961284 | TG | T | 0.174 | NM_144575 | CAPN13 | frameshift_deleti  on | exon16 | c.1575delC | p.L525fs | . | . |
| CTC1 | 2 | 31411215 | AT | A | 0.714 | NM_001145122 | CAPN14 | frameshift_deleti  on | exon14 | c.1498delA | p.I500fs | . | . |
| CTC1 | 2 | 31573030 | GC | G | 0.562 | NM_000379 | XDH | frameshift_deleti  on | exon25 | c.2690delG | p.G897fs | . | . |

| CTC1 | 2 | 33783752 | GT | G | 0.446 | NM_015376 | RASGRP3 | frameshift_deleti  on | exon16 | c.1717delT | p.F573fs | . | . |
| --- | --- | --- | --- | --- | --- | --- | --- | --- | --- | --- | --- | --- | --- |
| CTC1 | 2 | 33810286 | CT | C | 0.625 | NM_015475 | FAM98A | frameshift_deleti  on | exon8 | c.1113delA | p.R371fs | . | . |
| CTC1 | 2 | 37292085 | GC | G | 0.185 | NM_019024 | HEATR5B | frameshift_deleti  on | exon10 | c.1381delG | p.A461fs | . | . |
| CTC1 | 2 | 40656284 | TA | T | 0.278 | NM_001112800 | SLC8A1 | stopgain | exon1 | c.1136delT | p.L379X | . | . |
| CTC1 | 2 | 43015722 | AC | A | 1 | NM_012205 | HAAO | frameshift_deleti on | exon2 | c.105delG | p.M35fs | . | . |
| CTC1 | 2 | 43801477 | GT | G | 0.333 | NM_001083953 | THADA | frameshift_deleti  on | exon11 | c.1726delA | p.T576fs | . | . |
| CTC1 | 2 | 46986038 | GA | G | 1 | NM_014011 | SOCS5 | frameshift_deleti  on | exon2 | c.370delA | p.K124fs | . | . |
| CTC1 | 2 | 48027213 | TC | T | 0.194 | NM_001281492 | MSH6 | frameshift_deleti  on | exon2 | c.1702delC | p.Q568fs | . | . |
| CTC1 | 2 | 48037480 | GTT | G | 0.273 | NM_001190274 | FBXO11 | frameshift_deleti  on | exon19 | c.2311_2312del | p.N771fs | . | . |
| CTC1 | 2 | 50780160 | CA | C | 0.234 | NM_004801 | NRXN1 | frameshift_deleti  on | exon9 | c.1323delT | p.V441fs | . | . |
| CTC1 | 2 | 55096480 | GA | G | 0.2 | NM_001039753 | EML6 | frameshift_deleti  on | exon14 | c.2215delA | p.K739fs | . | . |
| CTC1 | 2 | 55126912 | CA | C | 0.872 | NM_001039753 | EML6 | frameshift_deleti  on | exon21 | c.3118delA | p.K1040fs | . | . |
| CTC1 | 2 | 62096668 | CA | C | 0.333 | NM_001256721 | CCT4 | frameshift_deleti  on | exon12 | c.1421delT | p.L474fs | . | . |
| CTC1 | 2 | 63631604 | TG | T | 0.667 | NM_015910 | WDPCP | stopgain | exon10 | c.1013delC | p.S338X | . | . |
| CTC1 | 2 | 68753335 | CA | C | 0.625 | NM_173545 | APLF | frameshift_deleti on | exon6 | c.766delA | p.K256fs | . | . |
| CTC1 | 2 | 68882163 | GA | G | 0.434 | NM_138964 | PROKR1 | frameshift_deleti  on | exon2 | c.638delA | p.E213fs | . | . |
| CTC1 | 2 | 69304598 | C | CT | 0.414 | NM_018153 | ANTXR1 | frameshift_inserti  on | exon8 | c.621dupT | p.A207fs | . | . |
| CTC1 | 2 | 69709931 | C | CA | 0.171 | NM_014911 | AAK1 | frameshift_inserti  on | exon18 | c.2378_2379ins  T | p.E793fs | . | . |
| CTC1 | 2 | 69741753 | CTGT | C | 1 | NM_014911 | AAK1 | nonframeshift_de  letion | exon13 | c.1623_1625del | p.541_542del | . | . |
| CTC1 | 2 | 69741762 | TTGC | T | 0.947 | NM_014911 | AAK1 | nonframeshift_de  letion | exon13 | c.1614_1616del | p.538_539del | . | . |
| CTC1 | 2 | 70143310 | TA | T | 0.421 | NM_001202513 | MXD1 | frameshift_deleti  on | exon2 | c.132delA | p.L44fs | . | . |
| CTC1 | 2 | 70502134 | GA | G | 0.458 | NM_016297 | PCYOX1 | frameshift_deleti  on | exon4 | c.539delA | p.E180fs | . | . |
| CTC1 | 2 | 71887714 | AT | A | 0.239 | NM_001130976 | DYSF | frameshift_deleti  on | exon43 | c.4778delT | p.I1593fs | . | . |
| CTC1 | 2 | 72725625 | TG | T | 0.162 | NM_015189 | EXOC6B | frameshift_deleti  on | exon13 | c.1294delC | p.Q432fs | . | . |
| CTC1 | 2 | 73457293 | TCC | T | 0.222 | NM_032319 | PRADC1 | frameshift_deleti  on | exon2 | c.114_115del | p.G38fs | . | . |
| CTC1 | 2 | 73676430 | CT | C | 0.5 | NM_015120 | ALMS1 | frameshift_deleti  on | exon8 | c.2774delT | p.L925fs | . | . |
| CTC1 | 2 | 74042590 | G | GA | 0.576 | NM_001080474 | C2orf78 | frameshift_inserti  on | exon3 | c.1241dupA | p.E414fs | . | . |
| CTC1 | 2 | 74482995 | TCG | T | 0.116 | NM_021196 | SLC4A5 | frameshift_deleti  on | exon8 | c.930_931del | p.G310fs | . | . |
| CTC1 | 2 | 74641906 | GA | G | 1 | NM_001145054 | C2orf81 | frameshift_deleti  on | exon4 | c.1316delT | p.L439fs | . | . |
| CTC1 | 2 | 74687691 | G | GC | 0.833 | NM_012477 | WBP1 | frameshift_inserti  on | exon4 | c.693_694insC | p.V231fs | . | . |
| CTC1 | 2 | 74699295 | CT | C | 0.19 | NM_053050 | MRPL53 | frameshift_deleti  on | exon3 | c.289delA | p.R97fs | . | . |
| CTC1 | 2 | 74709288 | GC | G | 0.4 | NM_032779 | CCDC142 | frameshift_deleti  on | exon1 | c.676delG | p.A226fs | . | . |
| CTC1 | 2 | 74757159 | GT | G | 0.25 | NM_013247 | HTRA2 | frameshift_deleti  on | exon1 | c.27delT | p.G9fs | . | . |
| CTC1 | 2 | 85576677 | GA | G | 0.391 | NM_017750 | RETSAT | frameshift_deleti  on | exon5 | c.826delT | p.S276fs | . | . |
| CTC1 | 2 | 85626393 | AC | A | 0.978 | NM_001256139 | CAPG | frameshift_deleti  on | exon6 | c.531delG | p.W177fs | . | . |
| CTC1 | 2 | 85843563 | CG | C | 0.308 | NM_001256725 | USP39 | frameshift_deleti  on | exon1 | c.246delG | p.S82fs | . | . |
| CTC1 | 2 | 95847040 | TGCG | T | 1 | NM_001017396 | ZNF2 | nonframeshift_de  letion | exon4 | c.342_344del | p.114_115del | . | . |
| CTC1 | 2 | 95847789 | GT | G | 0.296 | NM_001017396 | ZNF2 | frameshift_deleti  on | exon4 | c.1091delT | p.V364fs | . | . |
| CTC1 | 2 | 95952579 | CT | C | 0.455 | NM_001165977 | PROM2 | frameshift_deleti  on | exon18 | c.2069delT | p.L690fs | . | . |
| CTC1 | 2 | 97637958 | GC | G | 0.829 | NM_001122646 | FAM178B | frameshift_deleti  on | exon3 | c.243delG | p.R81fs | . | . |
| CTC1 | 2 | 97638056 | AC | A | 0.356 | NM_001122646 | FAM178B | frameshift_deleti  on | exon3 | c.145delG | p.V49fs | . | . |
| CTC1 | 2 | 97788680 | AT | A | 1 | NM_001164315 | ANKRD36 | frameshift_deleti  on | exon4 | c.544delT | p.F182fs | . | . |
| CTC1 | 2 | 98834340 | GT | G | 0.95 | NM_144992 | VWA3B | frameshift_deleti  on | exon14 | c.1869delT | p.R623fs | . | . |
| CTC1 | 2 | 99198194 | TG | T | 0.225 | NM_001566 | INPP4A | frameshift_deleti  on | exon26 | c.2841delG | p.L947fs | . | . |
| CTC1 | 2 | 99291527 | GC | G | 0.22 | NM_012214 | MGAT4A | frameshift_deleti  on | exon4 | c.373delG | p.A125fs | . | . |
| CTC1 | 2 | 99936212 | TTTG | T | 0.187 | NM_005783 | TXNDC9 | nonframeshift_de  letion | exon5 | c.611_613del | p.204_205del | . | . |
| CTC1 | 2 | 99978231 | TG | T | 0.8 | NM_015904 | EIF5B | frameshift_deleti  on | exon4 | c.868delG | p.G290fs | . | . |
| CTC1 | 2 | 100020208 | C | CT | 0.69 | NM_001037872 | REV1 | frameshift_inserti  on | exon19 | c.3112dupA | p.R1038fs | . | . |
| CTC1 | 2 | 101898334 | AT | A | 0.4 | NM_173647 | RNF149 | frameshift_deleti  on | exon6 | c.1145delA | p.N382fs | . | . |
| CTC1 | 2 | 102480426 | C | CAGTC  AGACC TCTGA | 0.409 | NM_001242560 | MAP4K4 | frameshift_inserti on | exon16 | c.1755_1756ins  AGTCAGACCT CTGACTTAG | p.L585fs | . | . |
| CTC1 | 2 | 105858803 | CG | C | 0.679 | NM_007227 | GPR45 | frameshift_deleti  on | exon1 | c.489delG | p.A163fs | . | . |
| CTC1 | 2 | 107052693 | G | GT | 1 | NM_001144013 | RGPD3 | frameshift_inserti  on | exon12 | c.1643dupA | p.N548fs | . | . |
| CTC1 | 2 | 111398652 | GA | G | 0.35 | NM_001278616 | BUB1 | frameshift_deleti  on | exon22 | c.2853delT | p.F951fs | . | . |
| CTC1 | 2 | 113088851 | A | AG | 0.833 | NM_198581 | ZC3H6 | frameshift_inserti  on | exon12 | c.2357dupG | p.R786fs | . | . |
| CTC1 | 2 | 113786603 | CT | C | 0.378 | NM_014438 | IL36B | frameshift_deleti  on | exon4 | c.173delA | p.K58fs | . | . |
| CTC1 | 2 | 113940257 | TG | T | 0.706 | NM_012455 | PSD4 | frameshift_deleti  on | exon2 | c.225delG | p.L75fs | . | . |
| CTC1 | 2 | 114256908 | TA | T | 0.233 | NM_012184 | FOXD4L1 | frameshift_deleti  on | exon1 | c.76delA | p.K26fs | . | . |

| CTC1 | 2 | 120194465 | GC | G | 0.311 | NM_183240 | TMEM37 | frameshift_deleti  on | exon2 | c.23delC | p.A8fs | . | . |
| --- | --- | --- | --- | --- | --- | --- | --- | --- | --- | --- | --- | --- | --- |
| CTC1 | 2 | 120194651 | A | AGTGT  GC | 1 | NM_183240 | TMEM37 | nonframeshift_in  sertion | exon2 | c.208_209insGT  GTGC | p.T70delinsSVP | . | . |
| CTC1 | 2 | 120836137 | CA | C | 0.588 | NM_001184937 | EPB41L5 | frameshift_deleti  on | exon10 | c.784delA | p.K262fs | . | . |
| CTC1 | 2 | 120900680 | G | GA | 0.441 | NM_001184937 | EPB41L5 | frameshift_inserti  on | exon19 | c.1702dupA | p.L567fs | . | . |
| CTC1 | 2 | 121989460 | TG | T | 0.278 | NM_014553 | TFCP2L1 | frameshift_deleti  on | exon13 | c.1282delC | p.H428fs | . | . |
| CTC1 | 2 | 124979309 | T | TC | 0.333 | NM_130773 | CNTNAP5 | frameshift_inserti  on | exon2 | c.110_111insC | p.L37fs | . | . |
| CTC1 | 2 | 128480298 | TC | T | 0.3 | NM_018383 | WDR33 | frameshift_deleti  on | exon14 | c.1384delG | p.D462fs | . | . |
| CTC1 | 2 | 130832417 | CA | C | 0.217 | NM_001099771 | POTEF | frameshift_deleti  on | exon17 | c.2627delT | p.L876fs | . | . |
| CTC1 | 2 | 130918794 | CG | C | 0.583 | NM_017951 | SMPD4 | frameshift_deleti  on | exon11 | c.1032delC | p.P344fs | . | . |
| CTC1 | 2 | 135907984 | GT | G | 0.767 | NM_001172435 | RAB3GAP1 | frameshift_deleti  on | exon18 | c.1970delT | p.V657fs | . | . |
| CTC1 | 2 | 135957924 | CA | C | 0.958 | NM_001286568 | ZRANB3 | frameshift_deleti  on | exon21 | c.3221delT | p.L1074fs | . | . |
| CTC1 | 2 | 141242980 | CA | C | 0.667 | NM_018557 | LRP1B | frameshift_deleti  on | exon59 | c.9356delT | p.L3119fs | . | . |
| CTC1 | 2 | 149806864 | CG | C | 0.5 | NM_004522 | KIF5C | frameshift_deleti  on | exon10 | c.857delG | p.R286fs | . | . |
| CTC1 | 2 | 152317778 | CA | C | 0.536 | NM_001177665 | RIF1 | frameshift_deleti  on | exon26 | c.3203delA | p.Q1068fs | . | . |
| CTC1 | 2 | 152322200 | AC | A | 0.273 | NM_001177665 | RIF1 | frameshift_deleti  on | exon29 | c.6167delC | p.T2056fs | . | . |
| CTC1 | 2 | 152421606 | A | AT | 0.6 | NM_004543 | NEB | frameshift_inserti  on | exon89 | c.13283dupA | p.D4428fs | . | . |
| CTC1 | 2 | 153476066 | G | GCCA | 0.75 | NM_052905 | FMNL2 | nonframeshift_in  sertion | exon15 | c.1671_1672ins  CCA | p.P557delinsPP | . | . |
| CTC1 | 2 | 157185021 | AT | A | 0.448 | NM_006186 | NR4A2 | frameshift_deleti  on | exon4 | c.888delA | p.K296fs | . | . |
| CTC1 | 2 | 158626995 | GC | G | 0.583 | NM_001105 | ACVR1 | frameshift_deleti  on | exon7 | c.674delG | p.G225fs | . | . |
| CTC1 | 2 | 159028716 | TGAAGT  CCAGCT TCTCGA AGTGCT | T | 0.2 | NM_138803 | CCDC148 | nonframeshift_de letion | exon14 | c.1652_1684del | p.551_562del | . | . |
| CTC1 | 2 | 159195560 | TC | T | 0.395 | NM_001171637 | CCDC148 | frameshift_deleti  on | exon4 | c.265delG | p.E89fs | . | . |
| CTC1 | 2 | 160628506 | CA | C | 0.769 | NM_001198763 | CD302 | frameshift_deleti  on | exon5 | c.380delT | p.L127fs | . | . |
| CTC1 | 2 | 164466149 | AT | A | 0.342 | NM_018086 | FIGN | frameshift_deleti  on | exon3 | c.2192delA | p.N731fs | . | . |
| CTC1 | 2 | 166237148 | AT | A | 0.326 | NM_001040143 | SCN2A | frameshift_deleti  on | exon23 | c.4356delT | p.Y1452fs | . | . |
| CTC1 | 2 | 167133580 | TA | T | 0.375 | NM_002977 | SCN9A | frameshift_deleti  on | exon16 | c.2753delT | p.I918fs | . | . |
| CTC1 | 2 | 167262515 | CA | C | 0.46 | NM_002976 | SCN7A | frameshift_deleti  on | exon25 | c.4623delT | p.F1541fs | . | . |
| CTC1 | 2 | 167322407 | GA | G | 0.667 | NM_002976 | SCN7A | frameshift_deleti  on | exon7 | c.754delT | p.S252fs | . | . |
| CTC1 | 2 | 168106715 | GA | G | 0.75 | NM_001199144 | XIRP2 | frameshift_deleti  on | exon7 | c.8148delA | p.G2716fs | . | . |
| CTC1 | 2 | 169721394 | TC | T | 0.2 | NM_001171632 | NOSTRIN | frameshift_deleti  on | exon15 | c.1352delC | p.S451fs | . | . |
| CTC1 | 2 | 169996990 | CA | C | 0.328 | NM_004525 | LRP2 | frameshift_deleti  on | exon72 | c.13173delT | p.F4391fs | . | . |
| CTC1 | 2 | 170028559 | AT | A | 1 | NM_004525 | LRP2 | frameshift_deleti  on | exon58 | c.11228delA | p.N3743fs | . | . |
| CTC1 | 2 | 173825536 | AC | A | 0.179 | NM_001100397 | RAPGEF4 | frameshift_deleti  on | exon4 | c.155delC | p.T52fs | . | . |
| CTC1 | 2 | 174777840 | TA | T | 0.375 | NM_001017371 | SP3 | frameshift_deleti  on | exon4 | c.1782delT | p.F594fs | . | . |
| CTC1 | 2 | 177202245 | AT | A | 0.571 | NM_006554 | MTX2 | frameshift_deleti  on | exon10 | c.646delT | p.F216fs | . | . |
| CTC1 | 2 | 178415704 | GT | G | 0.6 | NM_152517 | TTC30B | frameshift_deleti  on | exon1 | c.1787delA | p.N596fs | . | . |
| CTC1 | 2 | 179399257 | TC | T | 0.412 | NM_003319 | TTN | frameshift_deleti  on | exon186 | c.74889delG | p.M24963fs | . | . |
| CTC1 | 2 | 179399405 | TGG | T | 0.333 | NM_003319 | TTN | frameshift_deleti  on | exon186 | c.74740_74741  del | p.P24914fs | . | . |
| CTC1 | 2 | 179425266 | ATATT | A | 0.37 | NM_003319 | TTN | frameshift_deleti  on | exon154 | c.58394_58397  del | p.K19465fs | . | . |
| CTC1 | 2 | 179427033 | C | CT | 0.6 | NM_003319 | TTN | frameshift_inserti  on | exon154 | c.56630dupA | p.K18877fs | . | . |
| CTC1 | 2 | 179429366 | AG | A | 0.152 | NM_003319 | TTN | frameshift_deleti  on | exon154 | c.54297delC | p.C18099fs | . | . |
| CTC1 | 2 | 179432061 | TA | T | 0.281 | NM_003319 | TTN | stopgain | exon154 | c.51602delT | p.L17201X | . | . |
| CTC1 | 2 | 179435602 | GC | G | 0.221 | NM_003319 | TTN | frameshift_deleti on | exon154 | c.48061delG | p.A16021fs | . | . |
| CTC1 | 2 | 179447809 | TG | T | 0.806 | NM_003319 | TTN | frameshift_deleti  on | exon141 | c.38525delC | p.A12842fs | . | . |
| CTC1 | 2 | 179454181 | TG | T | 0.611 | NM_003319 | TTN | frameshift_deleti  on | exon132 | c.35075delC | p.T11692fs | . | . |
| CTC1 | 2 | 179477254 | AT | A | 0.71 | NM_003319 | TTN | frameshift_deleti  on | exon94 | c.22802delA | p.N7601fs | . | . |
| CTC1 | 2 | 179486720 | CT | C | 0.489 | NM_003319 | TTN | frameshift_deleti  on | exon72 | c.17733delA | p.K5911fs | . | . |
| CTC1 | 2 | 179582280 | CA | C | 0.214 | NM_133378 | TTN | frameshift_deleti  on | exon84 | c.21588delT | p.T7196fs | . | . |
| CTC1 | 2 | 182399547 | TA | T | 0.233 | NM_000885 | ITGA4 | frameshift_deleti  on | exon27 | c.2889delA | p.L963fs | . | . |
| CTC1 | 2 | 182786739 | TG | T | 1 | NM_001287504 | SSFA2 | frameshift_deleti  on | exon14 | c.2751delG | p.M917fs | . | . |
| CTC1 | 2 | 185802211 | C | CACA | 1 | NM_194250 | ZNF804A | nonframeshift_in  sertion | exon4 | c.2088_2089ins  ACA | p.N696delinsN  T | . | . |
| CTC1 | 2 | 185802213 | C | CAAG | 0.953 | NM_194250 | ZNF804A | nonframeshift_in  sertion | exon4 | c.2090_2091ins  AAG | p.T697delinsTR | . | . |
| CTC1 | 2 | 185802215 | A | ACAC | 0.955 | NM_194250 | ZNF804A | nonframeshift_in  sertion | exon4 | c.2092_2093ins  CAC | p.I698delinsTL | . | . |
| CTC1 | 2 | 186659635 | GA | G | 0.314 | NM_173651 | FSIP2 | frameshift_deleti  on | exon16 | c.8040delA | p.G2680fs | . | . |
| CTC1 | 2 | 187559047 | G | GCAA | 0.909 | NM_177454 | FAM171B | nonframeshift_in  sertion | exon1 | c.147_148insCA  A | p.Q49delinsQQ | . | . |
| CTC1 | 2 | 190532024 | TTGC | T | 0.385 | NM_019048 | ASNSD1 | nonframeshift_de  letion | exon4 | c.1167_1169del | p.389_390del | . | . |
| CTC1 | 2 | 190532551 | GC | G | 0.545 | NM_019048 | ASNSD1 | frameshift_deleti  on | exon5 | c.1527delC | p.R509fs | . | . |

| CTC1 | 2 | 201399881 | AG | A | 0.273 | NM_001160033 | SGOL2 | frameshift_deleti  on | exon3 | c.297delG | p.K99fs | . | . |
| --- | --- | --- | --- | --- | --- | --- | --- | --- | --- | --- | --- | --- | --- |
| CTC1 | 2 | 202025343 | CA | C | 0.304 | NM_001202515 | CFLAR | frameshift_deleti  on | exon5 | c.248delA | p.H83fs | . | . |
| CTC1 | 2 | 204260396 | GA | G | 0.296 | NM_001282926 | ABI2 | frameshift_deleti  on | exon6 | c.726delA | p.G242fs | . | . |
| CTC1 | 2 | 206057960 | G | GA | 0.375 | NM_152526 | PARD3B | frameshift_inserti  on | exon14 | c.1871dupA | p.D624fs | . | . |
| CTC1 | 2 | 207025418 | GA | G | 0.412 | NM_001959 | EEF1B2 | frameshift_deleti  on | exon2 | c.188delA | p.E63fs | . | . |
| CTC1 | 2 | 207988779 | GT | G | 0.3 | NM_001270943 | KLF7 | frameshift_deleti  on | exon2 | c.352delA | p.T118fs | . | . |
| CTC1 | 2 | 208615759 | CT | C | 0.667 | NM_001142300 | CCNYL1 | frameshift_deleti  on | exon7 | c.733delT | p.F245fs | . | . |
| CTC1 | 2 | 209179976 | TG | T | 0.408 | NM_015040 | PIKFYVE | frameshift_deleti  on | exon15 | c.1887delG | p.L629fs | . | . |
| CTC1 | 2 | 210557774 | GA | G | 0.444 | NM_002374 | MAP2 | frameshift_deleti  on | exon7 | c.881delA | p.E294fs | . | . |
| CTC1 | 2 | 210699663 | AG | A | 0.174 | NM_032504 | UNC80 | frameshift_deleti  on | exon18 | c.2971delG | p.G991fs | . | . |
| CTC1 | 2 | 218713576 | GC | G | 0.321 | NM_022648 | TNS1 | frameshift_deleti  on | exon17 | c.1288delG | p.A430fs | . | . |
| CTC1 | 2 | 219483364 | GC | G | 1 | NM_032726 | PLCD4 | frameshift_deleti  on | exon4 | c.245delC | p.A82fs | . | . |
| CTC1 | 2 | 219508128 | C | CA | 1 | NM_001105537 | ZNF142 | frameshift_inserti  on | exon8 | c.3110_3111ins  T | p.R1037fs | . | . |
| CTC1 | 2 | 219677462 | CT | C | 0.253 | NM_000784 | CYP27A1 | frameshift_deleti  on | exon4 | c.835delT | p.F279fs | . | . |
| CTC1 | 2 | 219695020 | GC | G | 0.506 | NM_017431 | PRKAG3 | frameshift_deleti  on | exon4 | c.313delG | p.A105fs | . | . |
| CTC1 | 2 | 219919938 | TG | T | 0.306 | NM_002181 | IHH | frameshift_deleti  on | exon3 | c.1226delC | p.A409fs | . | . |
| CTC1 | 2 | 220026777 | GC | G | 0.304 | NM_144712 | SLC23A3 | frameshift_deleti  on | exon11 | c.1335delG | p.Q445fs | . | . |
| CTC1 | 2 | 220110798 | TG | T | 0.673 | NM_001008910 | STK16 | frameshift_deleti  on | exon2 | c.78delG | p.L26fs | . | . |
| CTC1 | 2 | 220111513 | AC | A | 0.259 | NM_001008910 | STK16 | frameshift_deleti  on | exon3 | c.222delC | p.H74fs | . | . |
| CTC1 | 2 | 220342820 | AC | A | 0.904 | NM_005876 | SPEG | frameshift_deleti  on | exon22 | c.5021delC | p.T1674fs | . | . |
| CTC1 | 2 | 220466144 | GA | G | 0.386 | NM_052902 | STK11IP | frameshift_deleti  on | exon3 | c.283delA | p.K95fs | . | . |
| CTC1 | 2 | 220473427 | AC | A | 0.343 | NM_052902 | STK11IP | frameshift_deleti  on | exon15 | c.1760delC | p.T587fs | . | . |
| CTC1 | 2 | 222347267 | TG | T | 0.27 | NM_004438 | EPHA4 | frameshift_deleti  on | exon5 | c.1122delC | p.P374fs | . | . |
| CTC1 | 2 | 222347379 | TG | T | 0.188 | NM_004438 | EPHA4 | stopgain | exon5 | c.1010delC | p.S337X | . | . |
| CTC1 | 2 | 223158979 | C | CT | 0.493 | NM_000438 | PAX3 | frameshift_inserti on | exon4 | c.492dupA | p.G165fs | . | . |
| CTC1 | 2 | 223489014 | TC | T | 0.189 | NM_005687 | FARSB | frameshift_deleti  on | exon12 | c.1146delG | p.P382fs | . | . |
| CTC1 | 2 | 223917554 | GA | G | 0.889 | NM_080671 | KCNE4 | frameshift_deleti  on | exon2 | c.160delA | p.K54fs | . | . |
| CTC1 | 2 | 224770711 | TG | T | 0.476 | NM_020830 | WDFY1 | frameshift_deleti  on | exon4 | c.286delC | p.H96fs | . | . |
| CTC1 | 2 | 227661345 | CT | C | 0.4 | NM_005544 | IRS1 | frameshift_deleti  on | exon1 | c.2109delA | p.V703fs | . | . |
| CTC1 | 2 | 228128600 | TC | T | 0.527 | NM_000091 | COL4A3 | frameshift_deleti  on | exon21 | c.1256delC | p.S419fs | . | . |
| CTC1 | 2 | 228882385 | TG | T | 0.5 | NM_001142644 | SPHKAP | frameshift_deleti  on | exon7 | c.3184delC | p.Q1062fs | . | . |
| CTC1 | 2 | 228883393 | TG | T | 0.857 | NM_001142644 | SPHKAP | frameshift_deleti  on | exon7 | c.2176delC | p.H726fs | . | . |
| CTC1 | 2 | 231775564 | GT | G | 0.422 | NM_005683 | GPR55 | frameshift_deleti  on | exon2 | c.113delA | p.N38fs | . | . |
| CTC1 | 2 | 231861032 | TCAGCA  GCCTAG CCCTGA ATCCAC | T | 0.55 | NM_139073 | SPATA3 | nonframeshift_de letion | exon1 | c.85_111del | p.29_37del | . | . |
| CTC1 | 2 | 231949792 | T | TA | 0.304 | NM_001191037 | PSMD1 | frameshift_inserti  on | exon15 | c.1783dupA | p.G594fs | . | . |
| CTC1 | 2 | 232070962 | TTC | T | 0.143 | NM_025139 | ARMC9 | frameshift_deleti  on | exon2 | c.12_13del | p.I4fs | . | . |
| CTC1 | 2 | 232156098 | AG | A | 0.455 | NM_025139 | ARMC9 | frameshift_deleti  on | exon18 | c.1660delG | p.E554fs | . | . |
| CTC1 | 2 | 232325414 | TTCA | T | 1 | NM_005381 | NCL | nonframeshift_de  letion | exon4 | c.774_776del | p.258_259del | . | . |
| CTC1 | 2 | 233697763 | TGCA | T | 0.2 | NM_001103148 | GIGYF2 | nonframeshift_de  letion | exon21 | c.2709_2711del | p.903_904del | . | . |
| CTC1 | 2 | 233709092 | AC | A | 0.375 | NM_001103148 | GIGYF2 | frameshift_deleti  on | exon24 | c.3096delC | p.H1032fs | . | . |
| CTC1 | 2 | 233785143 | GCTC | G | 0.492 | NM_001114090 | NGEF | nonframeshift_de  letion | exon3 | c.400_402del | p.134_134del | . | . |
| CTC1 | 2 | 233839383 | CT | C | 0.4 | NM_019850 | NGEF | frameshift_deleti  on | exon2 | c.217delA | p.S73fs | . | . |
| CTC1 | 2 | 234235829 | CA | C | 0.235 | NM_000541 | SAG | frameshift_deleti  on | exon7 | c.499delA | p.K167fs | . | . |
| CTC1 | 2 | 234372900 | TG | T | 1 | NM_003648 | DGKD | frameshift_deleti  on | exon26 | c.3146delG | p.C1049fs | . | . |
| CTC1 | 2 | 234418766 | CA | C | 0.467 | NM_018218 | USP40 | frameshift_deleti  on | exon20 | c.2574delT | p.F858fs | . | . |
| CTC1 | 2 | 236761414 | C | CCAGG | 0.594 | NM_001244888 | AGAP1 | frameshift_inserti  on | exon10 | c.1135_1136ins  CAGG | p.P379fs | . | . |
| CTC1 | 2 | 239177606 | GT | G | 0.196 | NM_022817 | PER2 | frameshift_deleti  on | exon7 | c.781delA | p.T261fs | . | . |
| CTC1 | 2 | 241696840 | ATCC | A | 0.438 | NM_001244008 | KIF1A | nonframeshift_de  letion | exon27 | c.2751_2753del | p.917_918del | . | . |
| CTC1 | 2 | 242438408 | CG | C | 0.609 | NM_001271978 | STK25 | frameshift_deleti  on | exon6 | c.766delC | p.R256fs | . | . |
| CTC1 | 2 | 242811951 | C | CA | 0.417 | NM_173821 | CXXC11 | frameshift_inserti  on | exon1 | c.43_44insA | p.L15fs | . | . |
| CTC1 | 2 | 242815068 | AC | A | 0.212 | NM_173821 | CXXC11 | frameshift_deleti  on | exon2 | c.1362delC | p.D454fs | . | . |
| CTC1 | 3 | 4856761 | A | AT | 0.333 | NM_002222 | ITPR1 | frameshift_inserti  on | exon55 | c.7538dupT | p.I2513fs | . | . |
| CTC1 | 3 | 8677029 | TC | T | 0.19 | NM_001256748 | SSUH2 | frameshift_deleti  on | exon3 | c.165delG | p.G55fs | . | . |
| CTC1 | 3 | 9780851 | GT | G | 0.316 | NM_001003694 | BRPF1 | frameshift_deleti  on | exon3 | c.769delT | p.Y257fs | . | . |
| CTC1 | 3 | 9781257 | CG | C | 0.25 | NM_001003694 | BRPF1 | frameshift_deleti  on | exon3 | c.1175delG | p.R392fs | . | . |
| CTC1 | 3 | 9911623 | TA | T | 0.921 | NM_001199552 | CIDEC | frameshift_deleti  on | exon5 | c.496delT | p.Y166fs | . | . |

| CTC1 | 3 | 10105585 | CA | C | 0.265 | NM_001018115 | FANCD2 | frameshift_deleti  on | exon21 | c.1938delA | p.P646fs | . | . |
| --- | --- | --- | --- | --- | --- | --- | --- | --- | --- | --- | --- | --- | --- |
| CTC1 | 3 | 10276211 | G | GA | 0.162 | NM_001570 | IRAK2 | frameshift_inserti  on | exon11 | c.1342dupA | p.E447fs | . | . |
| CTC1 | 3 | 10283837 | C | CA | 0.889 | NM_001570 | IRAK2 | frameshift_inserti  on | exon13 | c.1804dupA | p.A601fs | . | . |
| CTC1 | 3 | 10291120 | ACTC | A | 0.117 | NM_014760 | TATDN2 | nonframeshift_de  letion | exon2 | c.237_239del | p.79_80del | . | . |
| CTC1 | 3 | 10291166 | CG | C | 0.246 | NM_014760 | TATDN2 | frameshift_deleti  on | exon2 | c.283delG | p.G95fs | . | . |
| CTC1 | 3 | 10312148 | CA | C | 0.359 | NM_014760 | TATDN2 | frameshift_deleti  on | exon4 | c.1283delA | p.Q428fs | . | . |
| CTC1 | 3 | 10491199 | TA | T | 0.609 | NM_001001331 | ATP2B2 | frameshift_deleti  on | exon2 | c.28delT | p.Y10fs | . | . |
| CTC1 | 3 | 12544805 | A | AG | 0.368 | NM_001145392 | TSEN2 | frameshift_inserti  on | exon5 | c.354dupG | p.Q118fs | . | . |
| CTC1 | 3 | 15057715 | GC | G | 0.274 | NM_003298 | NR2C2 | frameshift_deleti  on | exon5 | c.388delC | p.P130fs | . | . |
| CTC1 | 3 | 15062393 | G | GT | 0.833 | NM_003298 | NR2C2 | frameshift_inserti  on | exon6 | c.568dupT | p.Q189fs | . | . |
| CTC1 | 3 | 15457309 | CT | C | 0.657 | NM_152396 | METTL6 | frameshift_deleti  on | exon4 | c.500delA | p.K167fs | . | . |
| CTC1 | 3 | 15711905 | AG | A | 0.357 | NM_001195099 | ANKRD28 | frameshift_deleti  on | exon26 | c.2571delC | p.S857fs | . | . |
| CTC1 | 3 | 16411764 | GA | G | 0.22 | NM_015150 | RFTN1 | frameshift_deleti  on | exon6 | c.848delT | p.F283fs | . | . |
| CTC1 | 3 | 18391132 | CCTG | C | 0.3 | NM_001131010 | SATB1 | nonframeshift_de  letion | exon11 | c.1819_1821del | p.607_607del | . | . |
| CTC1 | 3 | 19551856 | TG | T | 0.242 | NM_144633 | KCNH8 | frameshift_deleti  on | exon12 | c.2043delG | p.V681fs | . | . |
| CTC1 | 3 | 20187891 | AC | A | 0.198 | NM_003884 | KAT2B | frameshift_deleti  on | exon14 | c.2089delC | p.Q697fs | . | . |
| CTC1 | 3 | 30892381 | TC | T | 0.545 | NM_207359 | GADL1 | frameshift_deleti  on | exon5 | c.480delG | p.L160fs | . | . |
| CTC1 | 3 | 31725332 | GA | G | 0.308 | NM_001174060 | OSBPL10 | frameshift_deleti  on | exon7 | c.1327delT | p.S443fs | . | . |
| CTC1 | 3 | 37365988 | GA | G | 1 | NM_002078 | GOLGA4 | frameshift_deleti  on | exon14 | c.2612delA | p.E871fs | . | . |
| CTC1 | 3 | 37792024 | TG | T | 0.582 | NM_002207 | ITGA9 | frameshift_deleti  on | exon23 | c.2506delG | p.G836fs | . | . |
| CTC1 | 3 | 38061690 | TC | T | 0.241 | NM_001130964 | PLCD1 | frameshift_deleti  on | exon2 | c.250delG | p.E84fs | . | . |
| CTC1 | 3 | 38065914 | AG | A | 0.429 | NM_001130964 | PLCD1 | frameshift_deleti  on | exon1 | c.10delC | p.L4fs | . | . |
| CTC1 | 3 | 39374233 | TG | T | 0.756 | NM_005201 | CCR8 | frameshift_deleti  on | exon2 | c.412delG | p.A138fs | . | . |
| CTC1 | 3 | 40503520 | A | ACTGC  TG | 0.955 | NM_001034996 | RPL14 | nonframeshift_in  sertion | exon6 | c.445_446insCT  GCTG | p.T149delinsTA  A | . | . |
| CTC1 | 3 | 42251577 | C | CGGAG  GA | 1 | NM_001265609 | TRAK1 | nonframeshift_in  sertion | exon13 | c.1841_1842ins  GGAGGA | p.T614delinsTE  E | . | . |
| CTC1 | 3 | 42680268 | AGAG | A | 0.25 | NM_005385 | NKTR | nonframeshift_de  letion | exon13 | c.3073_3075del | p.1025_1025de  l | . | . |
| CTC1 | 3 | 44399355 | AC | A | 0.661 | NM_001029839 | TCAIM | frameshift_deleti  on | exon3 | c.153delC | p.H51fs | . | . |
| CTC1 | 3 | 44636646 | CAG | C | 0.532 | NM_173658 | ZNF660 | frameshift_deleti  on | exon3 | c.962_963del | p.Q321fs | . | . |
| CTC1 | 3 | 44700979 | C | CA | 0.259 | NM_003420 | ZNF35 | frameshift_inserti  on | exon4 | c.1125dupA | p.A375fs | . | . |
| CTC1 | 3 | 44794944 | CT | C | 0.556 | NM_020696 | KIAA1143 | frameshift_deleti  on | exon3 | c.353delA | p.K118fs | . | . |
| CTC1 | 3 | 45554630 | GC | G | 0.667 | NM_015340 | LARS2 | frameshift_deleti  on | exon16 | c.1765delC | p.P589fs | . | . |
| CTC1 | 3 | 45823637 | TG | T | 0.204 | NM_020208 | SLC6A20 | frameshift_deleti  on | exon2 | c.199delC | p.Q67fs | . | . |
| CTC1 | 3 | 45942308 | A | AT | 0.238 | NM_031200 | CCR9 | frameshift_inserti  on | exon3 | c.29dupT | p.I10fs | . | . |
| CTC1 | 3 | 46009287 | C | CA | 0.429 | NM_024513 | FYCO1 | frameshift_inserti  on | exon8 | c.1538_1539ins  T | p.R513fs | . | . |
| CTC1 | 3 | 46063399 | TA | T | 0.661 | NM_001024644 | XCR1 | frameshift_deleti  on | exon2 | c.40delT | p.Y14fs | . | . |
| CTC1 | 3 | 46713445 | TA | T | 0.431 | NM_182775 | ALS2CL | frameshift_deleti  on | exon9 | c.653delT | p.V218fs | . | . |
| CTC1 | 3 | 46713677 | GA | G | 0.429 | NM_182775 | ALS2CL | frameshift_deleti  on | exon8 | c.527delT | p.V176fs | . | . |
| CTC1 | 3 | 47956364 | GT | G | 1 | NM_001134364 | MAP4 | frameshift_deleti  on | exon8 | c.1941delA | p.K647fs | . | . |
| CTC1 | 3 | 48336692 | C | CT | 0.526 | NM_005793 | NME6 | frameshift_inserti  on | exon5 | c.290dupA | p.K97fs | . | . |
| CTC1 | 3 | 48965063 | GGAA | G | 0.213 | NM_006321 | ARIH2 | nonframeshift_de  letion | exon3 | c.73_75del | p.25_25del | . | . |
| CTC1 | 3 | 49054782 | GC | G | 0.607 | NM_001009996 | DALRD3 | frameshift_deleti  on | exon5 | c.805delG | p.A269fs | . | . |
| CTC1 | 3 | 49139080 | CT | C | 0.279 | NM_001272073 | QARS | frameshift_deleti  on | exon9 | c.728delA | p.K243fs | . | . |
| CTC1 | 3 | 49211782 | AC | A | 0.667 | NM_173546 | KLHDC8B | frameshift_deleti  on | exon3 | c.488delC | p.T163fs | . | . |
| CTC1 | 3 | 49278690 | C | CA | 0.6 | NM_001135197 | CCDC36 | frameshift_inserti  on | exon4 | c.264dupA | p.T88fs | . | . |
| CTC1 | 3 | 49693066 | GC | G | 0.18 | NM_003458 | BSN | frameshift_deleti  on | exon5 | c.6078delC | p.G2026fs | . | . |
| CTC1 | 3 | 49775844 | C | CA | 0.309 | NM_001242829 | IP6K1 | frameshift_inserti  on | exon3 | c.234dupT | p.V79fs | . | . |
| CTC1 | 3 | 49850157 | TC | T | 0.375 | NM_003335 | UBA7 | frameshift_deleti  on | exon5 | c.481delG | p.D161fs | . | . |
| CTC1 | 3 | 50155887 | TGA | T | 0.4 | NM_005778 | RBM5 | frameshift_deleti  on | exon25 | c.2447_2448del | p.*816fs | . | . |
| CTC1 | 3 | 50332219 | AG | A | 0.28 | NM_001200029 | HYAL3 | frameshift_deleti  on | exon2 | c.814delC | p.L272fs | . | . |
| CTC1 | 3 | 50368829 | AG | A | 0.556 | NM_001206957 | RASSF1 | frameshift_deleti  on | exon4 | c.358delC | p.L120fs | . | . |
| CTC1 | 3 | 50386429 | GT | G | 0.37 | NM_006545 | NPRL2 | frameshift_deleti  on | exon5 | c.460delA | p.T154fs | . | . |
| CTC1 | 3 | 51430821 | GCCA | G | 0.158 | NM_013286 | RBM15B | nonframeshift_de  letion | exon1 | c.1992_1994del | p.664_665del | . | . |
| CTC1 | 3 | 52027853 | T | TCCTTG  G | 1 | NM_000992 | RPL29 | nonframeshift_in  sertion | exon4 | c.391_392insCC  AAGG | p.D131delinsA  KD | . | . |
| CTC1 | 3 | 52360310 | TCG | T | 0.686 | NM_015512 | DNAH1 | frameshift_deleti  on | exon4 | c.562_563del | p.R188fs | . | . |
| CTC1 | 3 | 52554220 | GC | G | 0.456 | NM_015136 | STAB1 | frameshift_deleti  on | exon52 | c.5414delC | p.A1805fs | . | . |
| CTC1 | 3 | 52554869 | C | CT | 0.349 | NM_015136 | STAB1 | frameshift_inserti  on | exon55 | c.5757dupT | p.P1919fs | . | . |
| CTC1 | 3 | 53218958 | CT | C | 0.701 | NM_212539 | PRKCD | frameshift_deleti  on | exon9 | c.857delT | p.L286fs | . | . |

| CTC1 | 3 | 53264548 | AT | A | 0.439 | NM_001064 | TKT | frameshift_deleti  on | exon8 | c.1031delA | p.N344fs | . | . |
| --- | --- | --- | --- | --- | --- | --- | --- | --- | --- | --- | --- | --- | --- |
| CTC1 | 3 | 53844266 | AG | A | 0.306 | NM_001128839 | CACNA1D | frameshift_deleti  on | exon45 | c.6062delG | p.S2021fs | . | . |
| CTC1 | 3 | 54952758 | TC | T | 0.542 | NM_020678 | LRTM1 | frameshift_deleti  on | exon3 | c.765delG | p.L255fs | . | . |
| CTC1 | 3 | 56650051 | A | ACTT | 1 | NM_001012506 | CCDC66 | nonframeshift_in  sertion | exon13 | c.1711_1712ins  CTT | p.T571delinsTS | . | . |
| CTC1 | 3 | 56650054 | T | TCTC | 1 | NM_001012506 | CCDC66 | nonframeshift_in  sertion | exon13 | c.1714_1715ins  CTC | p.S572delinsSP | . | . |
| CTC1 | 3 | 57132440 | CCA | C | 0.308 | NM_017563 | IL17RD | frameshift_deleti  on | exon12 | c.1289_1290del | p.V430fs | . | . |
| CTC1 | 3 | 58728235 | CA | C | 0.422 | NM_198463 | C3orf67 | frameshift_deleti  on | exon16 | c.1643delT | p.L548fs | . | . |
| CTC1 | 3 | 62357291 | CT | C | 0.478 | NM_018008 | FEZF2 | frameshift_deleti  on | exon3 | c.903delA | p.G301fs | . | . |
| CTC1 | 3 | 64004374 | CT | C | 0.205 | NM_001271780 | PSMD6 | frameshift_deleti  on | exon5 | c.612delA | p.K204fs | . | . |
| CTC1 | 3 | 64009034 | TG | T | 0.4 | NM_001271779 | PSMD6 | frameshift_deleti  on | exon1 | c.61delC | p.Q21fs | . | . |
| CTC1 | 3 | 64599171 | AC | A | 0.218 | NM_182920 | ADAMTS9 | frameshift_deleti  on | exon22 | c.3203delG | p.C1068fs | . | . |
| CTC1 | 3 | 77645884 | GA | G | 0.769 | NM_002942 | ROBO2 | frameshift_deleti  on | exon19 | c.2838delA | p.G946fs | . | . |
| CTC1 | 3 | 87018006 | TC | T | 0.163 | NM_016206 | VGLL3 | frameshift_deleti  on | exon3 | c.670delG | p.D224fs | . | . |
| CTC1 | 3 | 87027856 | TCTC | T | 0.267 | NM_016206 | VGLL3 | nonframeshift_de  letion | exon2 | c.220_222del | p.74_74del | . | . |
| CTC1 | 3 | 87294878 | GA | G | 0.538 | NM_001244644 | CHMP2B | frameshift_deleti  on | exon2 | c.19delA | p.K7fs | . | . |
| CTC1 | 3 | 89390120 | GC | G | 0.5 | NM_005233 | EPHA3 | frameshift_deleti  on | exon4 | c.870delC | p.C290fs | . | . |
| CTC1 | 3 | 97594881 | AT | A | 0.489 | NM_153605 | CRYBG3 | frameshift_deleti  on | exon4 | c.4844delT | p.I1615fs | . | . |
| CTC1 | 3 | 98002190 | AT | A | 0.74 | NM_001005482 | OR5H2 | frameshift_deleti  on | exon1 | c.460delT | p.F154fs | . | . |
| CTC1 | 3 | 100568899 | A | AT | 0.375 | NM_015429 | ABI3BP | frameshift_inserti  on | exon15 | c.1364_1365ins  A | p.P455fs | . | . |
| CTC1 | 3 | 105572443 | CA | C | 0.625 | NM_170662 | CBLB | frameshift_deleti  on | exon3 | c.233delT | p.L78fs | . | . |
| CTC1 | 3 | 108475362 | GC | G | 0.342 | NM_032579 | RETNLB | frameshift_deleti  on | exon2 | c.200delG | p.C67fs | . | . |
| CTC1 | 3 | 113376110 | TTGC | T | 1 | NM_001009899 | KIAA2018 | nonframeshift_de  letion | exon7 | c.4416_4418del | p.1472_1473de  l | . | . |
| CTC1 | 3 | 113737652 | T | TC | 0.513 | NM_020817 | KIAA1407 | frameshift_inserti  on | exon8 | c.1035dupG | p.K346fs | . | . |
| CTC1 | 3 | 113753989 | CT | C | 0.267 | NM_020817 | KIAA1407 | frameshift_deleti  on | exon6 | c.600delA | p.K200fs | . | . |
| CTC1 | 3 | 119462878 | G | GT | 0.181 | NM_033364 | MAATS1 | frameshift_inserti  on | exon14 | c.1738dupT | p.M579fs | . | . |
| CTC1 | 3 | 119911837 | C | CT | 0.325 | NM_001168271 | GPR156 | frameshift_inserti  on | exon4 | c.422dupA | p.K141fs | . | . |
| CTC1 | 3 | 120408710 | CT | C | 0.304 | NM_173825 | RABL3 | frameshift_deleti  on | exon8 | c.670delA | p.R224fs | . | . |
| CTC1 | 3 | 121351293 | T | TCTCA  GGCTC | 0.914 | NM_005335 | HCLS1 | nonframeshift_in  sertion | exon12 | c.1125_1126ins  CCCGAGCCTG | p.N376delinsPE  PEN | . | . |
| CTC1 | 3 | 121351315 | G | GGGCT  CAGGC | 1 | NM_005335 | HCLS1 | nonframeshift_in  sertion | exon12 | c.1103_1104ins  TGAGCCTGAG | p.P368delinsPE  PEP | . | . |
| CTC1 | 3 | 121351321 | A | AGGCT  CAGGC | 0.986 | NM_005335 | HCLS1 | nonframeshift_in  sertion | exon12 | c.1097_1098ins  CGAGCCTGAG | p.P366delinsPE  PEP | . | . |
| CTC1 | 3 | 121353138 | CT | C | 0.308 | NM_005335 | HCLS1 | frameshift_deleti  on | exon10 | c.818delA | p.K273fs | . | . |
| CTC1 | 3 | 121414289 | AT | A | 0.6 | NM_001256488 | GOLGB1 | frameshift_deleti  on | exon12 | c.4840delA | p.I1614fs | . | . |
| CTC1 | 3 | 122170472 | GA | G | 0.8 | NM_002264 | KPNA1 | frameshift_deleti  on | exon8 | c.661delT | p.S221fs | . | . |
| CTC1 | 3 | 122427106 | GAT | G | 0.37 | NM_017554 | PARP14 | frameshift_deleti  on | exon9 | c.3611_3612del | p.D1204fs | . | . |
| CTC1 | 3 | 123366133 | AG | A | 0.243 | NM_053026 | MYLK | frameshift_deleti  on | exon26 | c.4349delC | p.P1450fs | . | . |
| CTC1 | 3 | 123419116 | GTTC | G | 1 | NM_053026 | MYLK | nonframeshift_de  letion | exon17 | c.2989_2991del | p.997_997del | . | . |
| CTC1 | 3 | 123634283 | AT | A | 0.889 | NM_022757 | CCDC14 | frameshift_deleti  on | exon12 | c.2081delA | p.N694fs | . | . |
| CTC1 | 3 | 124646705 | A | AAAG | 1 | NM_033049 | MUC13 | nonframeshift_in  sertion | exon2 | c.184_185insCT  T | p.F62delinsSF | . | . |
| CTC1 | 3 | 124689625 | TG | T | 0.179 | NM_020733 | HEG1 | frameshift_deleti  on | exon17 | c.4016delC | p.P1339fs | . | . |
| CTC1 | 3 | 124951768 | AT | A | 0.429 | NM_021964 | ZNF148 | frameshift_deleti  on | exon9 | c.1801delA | p.M601fs | . | . |
| CTC1 | 3 | 127774524 | GT | G | 0.351 | NM_013336 | SEC61A1 | frameshift_deleti  on | exon4 | c.151delT | p.F51fs | . | . |
| CTC1 | 3 | 128292234 | A | AT | 0.5 | NM_007354 | C3orf27 | frameshift_inserti  on | exon3 | c.338_339insA | p.P113fs | . | . |
| CTC1 | 3 | 128356720 | GT | G | 0.333 | NM_002950 | RPN1 | frameshift_deleti  on | exon3 | c.554delA | p.Y185fs | . | . |
| CTC1 | 3 | 128690196 | TG | T | 0.44 | NM_020741 | KIAA1257 | frameshift_deleti  on | exon8 | c.1101delC | p.P367fs | . | . |
| CTC1 | 3 | 129156567 | TA | T | 0.264 | NM_001276270 | MBD4 | frameshift_deleti  on | exon2 | c.330delT | p.F110fs | . | . |
| CTC1 | 3 | 132358348 | GA | G | 0.354 | NM_032169 | ACAD11 | frameshift_deleti  on | exon5 | c.689delT | p.F230fs | . | . |
| CTC1 | 3 | 133377860 | CT | C | 0.359 | NM_007027 | TOPBP1 | frameshift_deleti  on | exon3 | c.218delA | p.K73fs | . | . |
| CTC1 | 3 | 134078998 | G | GA | 0.422 | NM_001278683 | AMOTL2 | frameshift_inserti  on | exon7 | c.2006dupT | p.F669fs | . | . |
| CTC1 | 3 | 135870064 | TA | T | 0.5 | NM_001145417 | MSL2 | frameshift_deleti  on | exon2 | c.1436delT | p.V479fs | . | . |
| CTC1 | 3 | 140178552 | GC | G | 0.673 | NM_022131 | CLSTN2 | frameshift_deleti  on | exon7 | c.1164delC | p.G388fs | . | . |
| CTC1 | 3 | 142133008 | GC | G | 0.297 | NM_001282857 | XRN1 | frameshift_deleti  on | exon14 | c.1561delG | p.A521fs | . | . |
| CTC1 | 3 | 142772536 | GA | G | 0.342 | NM_001080415 | U2SURP | frameshift_deleti  on | exon26 | c.2675delA | p.E892fs | . | . |
| CTC1 | 3 | 142772632 | G | GA | 0.187 | NM_001080415 | U2SURP | frameshift_inserti  on | exon26 | c.2771dupA | p.E924fs | . | . |
| CTC1 | 3 | 148714659 | TG | T | 0.5 | NM_001184720 | GYG1 | frameshift_deleti  on | exon4 | c.450delG | p.L150fs | . | . |
| CTC1 | 3 | 149563882 | CT | C | 0.435 | NM_183381 | RNF13 | frameshift_deleti  on | exon2 | c.70delT | p.F24fs | . | . |
| CTC1 | 3 | 149684299 | AT | A | 0.667 | NM_053024 | PFN2 | frameshift_deleti  on | exon3 | c.399delA | p.K133fs | . | . |
| CTC1 | 3 | 150391746 | AT | A | 0.429 | NM_152394 | FAM194A | frameshift_deleti  on | exon11 | c.1339delA | p.I447fs | . | . |

| CTC1 | 3 | 150396294 | G | GA | 1 | NM_152394 | FAM194A | frameshift_inserti  on | exon10 | c.1158dupT | p.P387fs | . | . |
| --- | --- | --- | --- | --- | --- | --- | --- | --- | --- | --- | --- | --- | --- |
| CTC1 | 3 | 151012090 | AAG | A | 0.643 | NM_023915 | GPR87 | frameshift_deleti  on | exon3 | c.942_943del | p.Y314fs | . | . |
| CTC1 | 3 | 151012168 | TG | T | 0.741 | NM_023915 | GPR87 | frameshift_deleti  on | exon3 | c.865delC | p.Q289fs | . | . |
| CTC1 | 3 | 151055778 | ACC | A | 0.714 | NM_176876 | P2RY12 | frameshift_deleti  on | exon2 | c.854_855del | p.W285fs | . | . |
| CTC1 | 3 | 151095929 | CA | C | 0.382 | NM_053002 | MED12L | frameshift_deleti  on | exon29 | c.4342delA | p.K1448fs | . | . |
| CTC1 | 3 | 155314089 | A | AG | 0.25 | NM_001130960 | PLCH1 | frameshift_inserti  on | exon2 | c.121dupC | p.L41fs | . | . |
| CTC1 | 3 | 158310270 | GT | G | 0.679 | NM_001130156 | MLF1 | frameshift_deleti  on | exon2 | c.21delT | p.S7fs | . | . |
| CTC1 | 3 | 158980511 | A | AT | 0.5 | NM_001042706 | IQCJ | frameshift_inserti  on | exon4 | c.331dupT | p.T110fs | . | . |
| CTC1 | 3 | 164907496 | TG | T | 1 | NM_014926 | SLITRK3 | frameshift_deleti  on | exon2 | c.1122delC | p.P374fs | . | . |
| CTC1 | 3 | 168813026 | AT | A | 0.5 | NM_001163999 | MECOM | frameshift_deleti  on | exon11 | c.2268delA | p.K756fs | . | . |
| CTC1 | 3 | 170786716 | AC | A | 0.5 | NM_001161566 | TNIK | frameshift_deleti  on | exon27 | c.3343delG | p.V1115fs | . | . |
| CTC1 | 3 | 172236202 | C | CA | 0.25 | NM_001190943 | TNFSF10 | frameshift_inserti  on | exon2 | c.185dupT | p.L62fs | . | . |
| CTC1 | 3 | 179525586 | A | AG | 0.267 | NM_001256753 | PEX5L | frameshift_inserti  on | exon12 | c.1374dupC | p.L459fs | . | . |
| CTC1 | 3 | 183960729 | GA | G | 0.444 | NM_001006941 | ALG3 | frameshift_deleti  on | exon8 | c.881delT | p.L294fs | . | . |
| CTC1 | 3 | 184070857 | GC | G | 0.472 | NM_001171088 | CLCN2 | frameshift_deleti  on | exon17 | c.1974delG | p.G658fs | . | . |
| CTC1 | 3 | 184947287 | AC | A | 1 | NM_001166415 | EHHADH | frameshift_deleti  on | exon4 | c.107delG | p.G36fs | . | . |
| CTC1 | 3 | 185410487 | AT | A | 0.32 | NM_001007225 | IGF2BP2 | stopgain | exon5 | c.403delA | p.I135X | . | . |
| CTC1 | 3 | 186459397 | AC | A | 0.312 | NM_001102416 | KNG1 | frameshift_deleti on | exon10 | c.1213delC | p.P405fs | . | . |
| CTC1 | 3 | 186792098 | AC | A | 0.875 | NM_003032 | ST6GAL1 | frameshift_deleti  on | exon6 | c.957delC | p.N319fs | . | . |
| CTC1 | 3 | 194336340 | T | TC | 0.188 | NM_001011655 | TMEM44 | frameshift_inserti  on | exon7 | c.869_870insG | p.Q290fs | . | . |
| CTC1 | 3 | 194790453 | C | CG | 0.226 | NM_152531 | XXYLT1 | frameshift_inserti  on | exon4 | c.1172dupC | p.P391fs | . | . |
| CTC1 | 3 | 195518112 | T | TGTCTC  CTGCG | 1 | NM_018406 | MUC4 | nonframeshift_in  sertion | exon2 | c.338_339insTG  TTACGCAGGA | p.T113delinsTV  TQET | . | . |
| CTC1 | 3 | 195518117 | C | CCTGC  GTAAC | 0.923 | NM_018406 | MUC4 | nonframeshift_in  sertion | exon2 | c.333_334insCA  GACTGTTACG | p.E112delinsQT  VTQE | . | . |
| CTC1 | 3 | 195597005 | TG | T | 0.818 | NM_001010938 | TNK2 | frameshift_deleti  on | exon11 | c.1711delC | p.Q571fs | . | . |
| CTC1 | 3 | 196281140 | T | TA | 0.278 | NM_182627 | WDR53 | frameshift_inserti  on | exon4 | c.1018_1019ins  T | p.N340fs | . | . |
| CTC1 | 3 | 196381515 | G | GT | 0.203 | NM_198565 | NRROS | frameshift_inserti  on | exon2 | c.106dupT | p.K35fs | . | . |
| CTC1 | 3 | 196612167 | A | AT | 0.5 | NM_152699 | SENP5 | frameshift_inserti  on | exon2 | c.116dupT | p.I39fs | . | . |
| CTC1 | 3 | 197762743 | G | GA | 0.478 | NM_033029 | LMLN | frameshift_inserti  on | exon15 | c.1697dupA | p.D566fs | . | . |
| CTC1 | 4 | 898516 | C | CA | 0.775 | NM_001286833 | GAK | frameshift_inserti  on | exon2 | c.196dupT | p.C66fs | . | . |
| CTC1 | 4 | 1346863 | GC | G | 0.667 | NM_020894 | UVSSA | frameshift_deleti  on | exon5 | c.597delC | p.C199fs | . | . |
| CTC1 | 4 | 1918685 | CT | C | 1 | NM_133334 | WHSC1 | frameshift_deleti  on | exon3 | c.849delT | p.A283fs | . | . |
| CTC1 | 4 | 2074702 | TG | T | 0.811 | NM_181808 | POLN | frameshift_deleti  on | exon23 | c.2509delC | p.Q837fs | . | . |
| CTC1 | 4 | 2943972 | G | GA | 0.438 | NM_003703 | NOP14 | frameshift_inserti  on | exon14 | c.1997dupT | p.L666fs | . | . |
| CTC1 | 4 | 2955250 | CT | C | 0.644 | NM_003703 | NOP14 | frameshift_deleti  on | exon5 | c.734delA | p.K245fs | . | . |
| CTC1 | 4 | 3430294 | AG | A | 0.538 | NM_198227 | RGS12 | frameshift_deleti  on | exon14 | c.1478delG | p.R493fs | . | . |
| CTC1 | 4 | 6537000 | G | GA | 0.634 | NM_001206994 | PPP2R2C | frameshift_inserti  on | exon2 | c.46dupT | p.S16fs | . | . |
| CTC1 | 4 | 6864659 | GT | G | 0.75 | NM_001100590 | KIAA0232 | frameshift_deleti  on | exon6 | c.2551delT | p.F851fs | . | . |
| CTC1 | 4 | 8021974 | GC | G | 0.476 | NM_001130084 | ABLIM2 | frameshift_deleti  on | exon12 | c.1224delG | p.P408fs | . | . |
| CTC1 | 4 | 8079366 | GC | G | 0.591 | NM_001130083 | ABLIM2 | frameshift_deleti  on | exon6 | c.663delG | p.G221fs | . | . |
| CTC1 | 4 | 9943590 | TC | T | 0.857 | NM_020041 | SLC2A9 | frameshift_deleti  on | exon6 | c.760delG | p.D254fs | . | . |
| CTC1 | 4 | 10445892 | TG | T | 0.312 | NM_053042 | ZNF518B | frameshift_deleti  on | exon3 | c.2060delC | p.A687fs | . | . |
| CTC1 | 4 | 10446563 | GA | G | 0.237 | NM_053042 | ZNF518B | frameshift_deleti  on | exon3 | c.1389delT | p.F463fs | . | . |
| CTC1 | 4 | 10502900 | TC | T | 0.66 | NM_052964 | CLNK | frameshift_deleti  on | exon18 | c.1119delG | p.G373fs | . | . |
| CTC1 | 4 | 17528696 | CT | C | 0.509 | NM_001079827 | CLRN2 | frameshift_deleti  on | exon3 | c.691delT | p.L231fs | . | . |
| CTC1 | 4 | 25353253 | AT | A | 0.667 | NM_024936 | ZCCHC4 | frameshift_deleti  on | exon8 | c.954delT | p.Y318fs | . | . |
| CTC1 | 4 | 25678227 | GT | G | 0.476 | NM_001177998 | SLC34A2 | frameshift_deleti  on | exon13 | c.1927delT | p.C643fs | . | . |
| CTC1 | 4 | 36231034 | TA | T | 0.267 | NM_015230 | ARAP2 | frameshift_deleti  on | exon2 | c.74delT | p.L25fs | . | . |
| CTC1 | 4 | 37841486 | AT | A | 0.703 | NM_018290 | PGM2 | frameshift_deleti  on | exon5 | c.459delT | p.H153fs | . | . |
| CTC1 | 4 | 38119733 | GCC | G | 0.333 | NM_001253913 | TBC1D1 | frameshift_deleti  on | exon3 | c.174_175del | p.G58fs | . | . |
| CTC1 | 4 | 38924509 | AAC | A | 0.5 | NM_138389 | FAM114A1 | frameshift_deleti  on | exon9 | c.1065_1066del | p.E355fs | . | . |
| CTC1 | 4 | 39850541 | CT | C | 0.52 | NM_001100399 | PDS5A | frameshift_deleti  on | exon28 | c.3268delA | p.S1090fs | . | . |
| CTC1 | 4 | 40434713 | GGCGGC  TGCGGC | G | 0.2 | NM_019027 | RBM47 | nonframeshift_de  letion | exon4 | c.1278_1289del | p.426_430del | . | . |
| CTC1 | 4 | 40776831 | TC | T | 0.55 | NM_024677 | NSUN7 | frameshift_deleti  on | exon6 | c.690delC | p.V230fs | . | . |
| CTC1 | 4 | 40937098 | C | CA | 0.222 | NM_001166050 | APBB2 | frameshift_inserti  on | exon8 | c.1099dupT | p.W367fs | . | . |
| CTC1 | 4 | 41956135 | AC | A | 0.5 | NM_018126 | TMEM33 | frameshift_deleti  on | exon7 | c.664delC | p.P222fs | . | . |
| CTC1 | 4 | 42122168 | G | GT | 0.727 | NM_001159547 | BEND4 | stopgain | exon5 | c.1289dupA | p.Y430_S431de  linsX | . | . |
| CTC1 | 4 | 48114364 | TC | T | 0.214 | NM_003328 | TXK | frameshift_deleti  on | exon4 | c.339delG | p.E113fs | . | . |
| CTC1 | 4 | 48608508 | GA | G | 0.786 | NM_015030 | FRYL | frameshift_deleti  on | exon10 | c.687delT | p.F229fs | . | . |

| CTC1 | 4 | 55561871 | AG | A | 0.259 | NM_000222 | KIT | frameshift_deleti  on | exon2 | c.262delG | p.E88fs | . | . |
| --- | --- | --- | --- | --- | --- | --- | --- | --- | --- | --- | --- | --- | --- |
| CTC1 | 4 | 55984902 | AC | A | 0.625 | NM_002253 | KDR | stopgain | exon3 | c.226delG | p.V76X | . | . |
| CTC1 | 4 | 57182264 | CAGA | C | 0.244 | NM_020722 | KIAA1211 | nonframeshift_de letion | exon8 | c.2597_2599del | p.866_867del | . | . |
| CTC1 | 4 | 57215857 | A | AT | 0.895 | NM_001286670 | AASDH | frameshift_inserti  on | exon8 | c.604dupA | p.I202fs | . | . |
| CTC1 | 4 | 57215857 | A | ATT | 0.895 | NM_001286670 | AASDH | frameshift_inserti  on | exon8 | c.604_605insAA | p.I202fs | . | . |
| CTC1 | 4 | 57839322 | CT | C | 0.571 | NM_032313 | NOA1 | frameshift_deleti  on | exon3 | c.1506delA | p.K502fs | . | . |
| CTC1 | 4 | 62936592 | CA | C | 0.3 | NM_015236 | LPHN3 | frameshift_deleti  on | exon23 | c.4377delA | p.S1459fs | . | . |
| CTC1 | 4 | 68355715 | CT | C | 0.625 | NM_001812 | CENPC | frameshift_deleti  on | exon17 | c.2639delA | p.K880fs | . | . |
| CTC1 | 4 | 69513013 | C | CA | 0.331 | NM_001076 | UGT2B15 | frameshift_inserti  on | exon6 | c.1401dupT | p.V468fs | . | . |
| CTC1 | 4 | 74735284 | GC | G | 0.181 | NM_001511 | CXCL1 | frameshift_deleti  on | exon1 | c.98delC | p.A33fs | . | . |
| CTC1 | 4 | 75938174 | TC | T | 0.263 | NM_015393 | PARM1 | frameshift_deleti  on | exon2 | c.584delC | p.S195fs | . | . |
| CTC1 | 4 | 76573905 | TG | T | 0.917 | NM_203504 | G3BP2 | frameshift_deleti  on | exon8 | c.746delC | p.P249fs | . | . |
| CTC1 | 4 | 77065318 | C | CTGT | 0.643 | NM_017426 | NUP54 | nonframeshift_in  sertion | exon3 | c.278_279insAC  A | p.Q93delinsQQ | . | . |
| CTC1 | 4 | 77817352 | GT | G | 0.333 | NM_001029870 | SOWAHB | frameshift_deleti  on | exon1 | c.1650delA | p.K550fs | . | . |
| CTC1 | 4 | 79393382 | AC | A | 0.385 | NM_025074 | FRAS1 | frameshift_deleti  on | exon52 | c.7421delC | p.T2474fs | . | . |
| CTC1 | 4 | 79792082 | CCAG | C | 0.194 | NM_017593 | BMP2K | nonframeshift_de  letion | exon11 | c.1378_1380del | p.460_460del | . | . |
| CTC1 | 4 | 79792136 | ACAGCA  GCAG | A | 0.523 | NM_017593 | BMP2K | nonframeshift_de  letion | exon11 | c.1432_1440del | p.478_480del | . | . |
| CTC1 | 4 | 81187991 | TTCC | T | 0.105 | NM_004464 | FGF5 | nonframeshift_de  letion | exon1 | c.14_16del | p.5_6del | . | . |
| CTC1 | 4 | 84369936 | CT | C | 0.778 | NM_133636 | HELQ | frameshift_deleti  on | exon3 | c.1190delA | p.K397fs | . | . |
| CTC1 | 4 | 84511398 | TC | T | 1 | NM_032717 | AGPAT9 | frameshift_deleti  on | exon6 | c.692delC | p.S231fs | . | . |
| CTC1 | 4 | 85617125 | GT | G | 0.6 | NM_014991 | WDFY3 | frameshift_deleti  on | exon58 | c.8898delA | p.K2966fs | . | . |
| CTC1 | 4 | 85617217 | TA | T | 0.727 | NM_014991 | WDFY3 | frameshift_deleti  on | exon58 | c.8806delT | p.Y2936fs | . | . |
| CTC1 | 4 | 85630177 | CCT | C | 0.722 | NM_014991 | WDFY3 | frameshift_deleti  on | exon53 | c.8100_8101del | p.R2700fs | . | . |
| CTC1 | 4 | 85654695 | T | TCC | 0.667 | NM_014991 | WDFY3 | frameshift_inserti  on | exon44 | c.7060_7061ins  GG | p.E2354fs | . | . |
| CTC1 | 4 | 85699772 | GA | G | 0.31 | NM_014991 | WDFY3 | frameshift_deleti  on | exon27 | c.4401delT | p.L1467fs | . | . |
| CTC1 | 4 | 85781717 | GC | G | 0.5 | NM_014991 | WDFY3 | frameshift_deleti  on | exon4 | c.27delG | p.G9fs | . | . |
| CTC1 | 4 | 87684173 | TC | T | 0.625 | NM_080684 | PTPN13 | frameshift_deleti  on | exon21 | c.3275delC | p.S1092fs | . | . |
| CTC1 | 4 | 88533723 | GA | G | 0.529 | NM_014208 | DSPP | frameshift_deleti  on | exon4 | c.386delA | p.E129fs | . | . |
| CTC1 | 4 | 88973218 | CA | C | 0.772 | NM_000297 | PKD2 | frameshift_deleti  on | exon7 | c.1625delA | p.Q542fs | . | . |
| CTC1 | 4 | 90816589 | TA | T | 0.385 | NM_007351 | MMRN1 | frameshift_deleti  on | exon1 | c.468delA | p.L156fs | . | . |
| CTC1 | 4 | 91230254 | AT | A | 0.667 | NM_001145065 | CCSER1 | frameshift_deleti  on | exon2 | c.820delT | p.F274fs | . | . |
| CTC1 | 4 | 95377523 | AT | A | 0.5 | NM_001256429 | PDLIM5 | frameshift_deleti  on | exon3 | c.226delT | p.F76fs | . | . |
| CTC1 | 4 | 99264304 | GA | G | 1 | NM_001100426 | RAP1GDS1 | frameshift_deleti  on | exon3 | c.131delA | p.E44fs | . | . |
| CTC1 | 4 | 100445706 | CA | C | 0.381 | NM_032149 | C4orf17 | frameshift_deleti  on | exon4 | c.367delA | p.K123fs | . | . |
| CTC1 | 4 | 109841745 | GA | G | 0.364 | NM_001256074 | COL25A1 | frameshift_deleti  on | exon10 | c.711delT | p.P237fs | . | . |
| CTC1 | 4 | 110682690 | CA | C | 0.211 | NM_000204 | CFI | frameshift_deleti  on | exon4 | c.640delT | p.C214fs | . | . |
| CTC1 | 4 | 110865004 | AT | A | 0.579 | NM_001178130 | EGF | frameshift_deleti  on | exon4 | c.517delT | p.F173fs | . | . |
| CTC1 | 4 | 111539366 | TG | T | 0.469 | NM_000325 | PITX2 | frameshift_deleti  on | exon3 | c.889delC | p.Q297fs | . | . |
| CTC1 | 4 | 113181980 | GA | G | 0.462 | NM_001128426 | AP1AR | frameshift_deleti  on | exon5 | c.245delA | p.E82fs | . | . |
| CTC1 | 4 | 113510987 | GAA | G | 0.167 | NM_018392 | C4orf21 | frameshift_deleti  on | exon11 | c.3018_3019del | p.V1006fs | . | . |
| CTC1 | 4 | 114824638 | CA | C | 0.6 | NM_024590 | ARSJ | frameshift_deleti  on | exon2 | c.591delT | p.F197fs | . | . |
| CTC1 | 4 | 121958586 | TG | T | 1 | NM_024574 | NDNF | frameshift_deleti  on | exon4 | c.539delC | p.P180fs | . | . |
| CTC1 | 4 | 122250856 | CT | C | 0.471 | NM_198179 | QRFPR | frameshift_deleti  on | exon6 | c.908delA | p.K303fs | . | . |
| CTC1 | 4 | 123663586 | CCT | C | 0.6 | NM_152618 | BBS12 | frameshift_deleti  on | exon2 | c.540_541del | p.A180fs | . | . |
| CTC1 | 4 | 124011765 | GTT | G | 0.8 | NM_145207 | SPATA5 | frameshift_deleti  on | exon14 | c.2246_2247del | p.V749fs | . | . |
| CTC1 | 4 | 126370072 | A | AT | 0.636 | NM_024582 | FAT4 | frameshift_inserti  on | exon9 | c.7901_7902ins  T | p.K2634fs | . | . |
| CTC1 | 4 | 128554278 | AT | A | 1 | NM_015693 | INTU | frameshift_deleti  on | exon1 | c.90delT | p.D30fs | . | . |
| CTC1 | 4 | 128685440 | GC | G | 0.273 | NM_031291 | SLC25A31 | frameshift_deleti  on | exon3 | c.404delC | p.A135fs | . | . |
| CTC1 | 4 | 128719767 | CT | C | 0.286 | NM_014278 | HSPA4L | frameshift_deleti  on | exon4 | c.333delT | p.P111fs | . | . |
| CTC1 | 4 | 128748518 | T | TA | 0.543 | NM_014278 | HSPA4L | frameshift_inserti  on | exon17 | c.2106dupA | p.L702fs | . | . |
| CTC1 | 4 | 128807217 | CA | C | 0.333 | NM_001190799 | PLK4 | frameshift_deleti  on | exon4 | c.597delA | p.P199fs | . | . |
| CTC1 | 4 | 129783365 | GC | G | 0.25 | NM_001287437 | JADE1 | frameshift_deleti  on | exon9 | c.1453delC | p.Q485fs | . | . |
| CTC1 | 4 | 140651584 | TCTG | T | 1 | NM_018717 | MAML3 | nonframeshift_de  letion | exon4 | c.2302_2304del | p.768_768del | . | . |
| CTC1 | 4 | 140651584 | TCTGCTG | T | 1 | NM_018717 | MAML3 | nonframeshift_de  letion | exon4 | c.2299_2304del | p.767_768del | . | . |
| CTC1 | 4 | 140811063 | TTGCTGC  TGCTGC | T | 1 | NM_018717 | MAML3 | frameshift_deleti  on | exon3 | c.1513_1514del | p.Q505fs | . | . |
| CTC1 | 4 | 142643079 | GT | G | 0.333 | NM_000585 | IL15 | frameshift_deleti  on | exon5 | c.114delT | p.C38fs | . | . |
| CTC1 | 4 | 144532622 | TG | T | 0.2 | NM_001168235 | FREM3 | frameshift_deleti  on | exon6 | c.5837delC | p.P1946fs | . | . |

| CTC1 | 4 | 146813426 | GC | G | 0.889 | NM_178835 | ZNF827 | frameshift_deleti  on | exon3 | c.1234delG | p.A412fs | . | . |
| --- | --- | --- | --- | --- | --- | --- | --- | --- | --- | --- | --- | --- | --- |
| CTC1 | 4 | 147560457 | TGGCGG  CGGC | T | 1 | NM_004575 | POU4F2 | nonframeshift_de  letion | exon1 | c.166_174del | p.56_58del | . | . |
| CTC1 | 4 | 147560457 | TGGCGG  CGGCGG CGGCGG | T | 1 | NM_004575 | POU4F2 | nonframeshift_de letion | exon1 | c.166_189del | p.56_63del | . | . |
| CTC1 | 4 | 148605110 | CG | C | 0.667 | NM_138364 | PRMT9 | frameshift_deleti  on | exon1 | c.28delC | p.R10fs | . | . |
| CTC1 | 4 | 148778777 | C | CA | 0.45 | NM_024605 | ARHGAP10 | frameshift_inserti  on | exon5 | c.459dupA | p.A153fs | . | . |
| CTC1 | 4 | 151935662 | TG | T | 0.875 | NM_001199282 | LRBA | frameshift_deleti  on | exon2 | c.132delC | p.I44fs | . | . |
| CTC1 | 4 | 152054249 | TC | T | 0.223 | NM_001128924 | SH3D19 | frameshift_deleti  on | exon9 | c.1677delG | p.G559fs | . | . |
| CTC1 | 4 | 152203384 | C | CA | 0.335 | NM_183375 | PRSS48 | frameshift_inserti  on | exon3 | c.301dupA | p.S100fs | . | . |
| CTC1 | 4 | 153244292 | TTG | T | 0.188 | NM_001013415 | FBXW7 | frameshift_deleti  on | exon11 | c.1509_1510del | p.N503fs | . | . |
| CTC1 | 4 | 153332604 | TCTC | T | 0.2 | NM_033632 | FBXW7 | nonframeshift_de  letion | exon2 | c.349_351del | p.117_117del | . | . |
| CTC1 | 4 | 159592912 | CG | C | 0.818 | NM_001008393 | C4orf46 | frameshift_deleti  on | exon1 | c.41delC | p.P14fs | . | . |
| CTC1 | 4 | 162841593 | CT | C | 0.923 | NM_001128427 | FSTL5 | frameshift_deleti  on | exon4 | c.368delA | p.K123fs | . | . |
| CTC1 | 4 | 169209414 | CA | C | 0.409 | NM_017631 | DDX60 | frameshift_deleti  on | exon9 | c.1093delT | p.W365fs | . | . |
| CTC1 | 4 | 174219354 | GC | G | 0.261 | NM_017423 | GALNT7 | frameshift_deleti  on | exon6 | c.1055delC | p.A352fs | . | . |
| CTC1 | 4 | 177056382 | TC | T | 0.556 | NM_181265 | WDR17 | frameshift_deleti  on | exon8 | c.1223delC | p.S408fs | . | . |
| CTC1 | 4 | 183713978 | C | CA | 0.267 | NM_001080477 | TENM3 | frameshift_inserti  on | exon25 | c.6154dupA | p.G2051fs | . | . |
| CTC1 | 4 | 185606779 | TA | T | 0.917 | NM_152683 | PRIMPOL | frameshift_deleti  on | exon11 | c.1237delA | p.I413fs | . | . |
| CTC1 | 4 | 186361782 | AT | A | 1 | NM_001114357 | C4orf47 | frameshift_deleti  on | exon5 | c.624delT | p.Y208fs | . | . |
| CTC1 | 4 | 186544619 | CGGT | C | 0.128 | NM_001145674 | SORBS2 | nonframeshift_de  letion | exon7 | c.1661_1663del | p.554_555del | . | . |
| CTC1 | 4 | 190864381 | GA | G | 0.467 | NM_004477 | FRG1 | frameshift_deleti  on | exon2 | c.88delA | p.K30fs | . | . |
| CTC1 | 5 | 251517 | GC | G | 0.375 | NM_004168 | SDHA | frameshift_deleti  on | exon13 | c.1729delC | p.Q577fs | . | . |
| CTC1 | 5 | 5457591 | CA | C | 0.273 | NM_015325 | KIAA0947 | frameshift_deleti  on | exon12 | c.839delA | p.Q280fs | . | . |
| CTC1 | 5 | 14508490 | A | AT | 0.333 | NM_007118 | TRIO | frameshift_inserti  on | exon57 | c.9254dupT | p.I3085fs | . | . |
| CTC1 | 5 | 16479001 | CA | C | 0.773 | NM_019000 | FAM134B | frameshift_deleti  on | exon4 | c.342delT | p.F114fs | . | . |
| CTC1 | 5 | 16769185 | GT | G | 0.556 | NM_012334 | MYO10 | frameshift_deleti  on | exon10 | c.1057delA | p.T353fs | . | . |
| CTC1 | 5 | 31323034 | TG | T | 0.293 | NM_004932 | CDH6 | frameshift_deleti  on | exon12 | c.1993delG | p.G665fs | . | . |
| CTC1 | 5 | 31551486 | TG | T | 0.5 | NM_018356 | C5orf22 | frameshift_deleti  on | exon8 | c.1147delG | p.V383fs | . | . |
| CTC1 | 5 | 34688347 | GAA | G | 0.435 | NM_001145525 | RAI14 | frameshift_deleti  on | exon4 | c.40_41del | p.K14fs | . | . |
| CTC1 | 5 | 34818952 | TA | T | 0.333 | NM_001145522 | RAI14 | frameshift_deleti  on | exon12 | c.904delA | p.T302fs | . | . |
| CTC1 | 5 | 35697815 | TG | T | 0.257 | NM_024867 | SPEF2 | frameshift_deleti  on | exon15 | c.2062delG | p.A688fs | . | . |
| CTC1 | 5 | 37169268 | TAC | T | 0.5 | NM_023073 | C5orf42 | frameshift_deleti  on | exon34 | c.6856_6857del | p.V2286fs | . | . |
| CTC1 | 5 | 37703105 | TC | T | 0.19 | NM_018034 | WDR70 | frameshift_deleti  on | exon13 | c.1333delC | p.Q445fs | . | . |
| CTC1 | 5 | 37727125 | TG | T | 0.375 | NM_018034 | WDR70 | frameshift_deleti  on | exon17 | c.1856delG | p.W619fs | . | . |
| CTC1 | 5 | 38407033 | CT | C | 0.375 | NM_182798 | EGFLAM | frameshift_deleti  on | exon3 | c.231delT | p.P77fs | . | . |
| CTC1 | 5 | 43453860 | A | AT | 0.158 | NM_022483 | C5orf28 | frameshift_inserti  on | exon2 | c.211dupA | p.I71fs | . | . |
| CTC1 | 5 | 49706718 | AT | A | 0.309 | NM_198449 | EMB | frameshift_deleti  on | exon4 | c.464delA | p.N155fs | . | . |
| CTC1 | 5 | 53839132 | AC | A | 0.494 | NM_001102575 | SNX18 | frameshift_deleti  on | exon2 | c.1746delC | p.H582fs | . | . |
| CTC1 | 5 | 55155365 | TG | T | 0.388 | NM_001242636 | IL31RA | frameshift_deleti  on | exon2 | c.36delG | p.L12fs | . | . |
| CTC1 | 5 | 55164689 | TC | T | 0.211 | NM_001242636 | IL31RA | frameshift_deleti  on | exon3 | c.169delC | p.P57fs | . | . |
| CTC1 | 5 | 55212604 | AT | A | 0.5 | NM_001242636 | IL31RA | frameshift_deleti  on | exon15 | c.1895delT | p.I632fs | . | . |
| CTC1 | 5 | 56207018 | G | GA | 0.6 | NM_001171990 | SETD9 | frameshift_inserti  on | exon2 | c.122dupA | p.E41fs | . | . |
| CTC1 | 5 | 66416852 | AT | A | 0.235 | NM_015183 | MAST4 | frameshift_deleti  on | exon13 | c.1101delT | p.N367fs | . | . |
| CTC1 | 5 | 68565038 | CT | C | 0.417 | NM_001799 | CDK7 | frameshift_deleti  on | exon9 | c.633delT | p.P211fs | . | . |
| CTC1 | 5 | 71756883 | GC | G | 0.371 | NM_152625 | ZNF366 | frameshift_deleti  on | exon2 | c.440delG | p.G147fs | . | . |
| CTC1 | 5 | 74014168 | GT | G | 0.415 | NM_000521 | HEXB | frameshift_deleti  on | exon10 | c.1223delT | p.V408fs | . | . |
| CTC1 | 5 | 74403377 | C | CT | 0.63 | NM_001164443 | ANKRD31 | frameshift_inserti  on | exon20 | c.4137dupA | p.A1380fs | . | . |
| CTC1 | 5 | 74444408 | TG | T | 0.833 | NM_001164443 | ANKRD31 | frameshift_deleti  on | exon13 | c.1897delC | p.Q633fs | . | . |
| CTC1 | 5 | 77409691 | TG | T | 0.308 | NM_001271769 | AP3B1 | frameshift_deleti  on | exon19 | c.1986delC | p.D662fs | . | . |
| CTC1 | 5 | 77536740 | AT | A | 0.429 | NM_001271769 | AP3B1 | frameshift_deleti  on | exon3 | c.77delA | p.N26fs | . | . |
| CTC1 | 5 | 78077772 | TG | T | 0.269 | NM_000046 | ARSB | frameshift_deleti  on | exon7 | c.1238delC | p.A413fs | . | . |
| CTC1 | 5 | 78375275 | GA | G | 0.259 | NM_001178005 | BHMT2 | frameshift_deleti  on | exon3 | c.251delA | p.E84fs | . | . |
| CTC1 | 5 | 78585947 | TC | T | 0.207 | NM_152405 | JMY | frameshift_deleti  on | exon3 | c.1211delC | p.S404fs | . | . |
| CTC1 | 5 | 78596125 | CA | C | 0.409 | NM_152405 | JMY | frameshift_deleti  on | exon5 | c.1678delA | p.K560fs | . | . |
| CTC1 | 5 | 79031253 | CA | C | 0.417 | NM_153610 | CMYA5 | frameshift_deleti  on | exon2 | c.6666delA | p.T2222fs | . | . |
| CTC1 | 5 | 82817412 | GA | G | 0.323 | NM_001164098 | VCAN | frameshift_deleti  on | exon7 | c.3288delA | p.G1096fs | . | . |
| CTC1 | 5 | 82817467 | GAA | G | 0.219 | NM_001164098 | VCAN | frameshift_deleti  on | exon7 | c.3343_3344del | p.K1115fs | . | . |
| CTC1 | 5 | 82836696 | CA | C | 0.383 | NM_001164097 | VCAN | frameshift_deleti  on | exon7 | c.4914delA | p.A1638fs | . | . |

| CTC1 | 5 | 87498813 | TA | T | 0.667 | NM_001289007 | TMEM161B | frameshift_deleti  on | exon9 | c.879delT | p.I293fs | . | . |
| --- | --- | --- | --- | --- | --- | --- | --- | --- | --- | --- | --- | --- | --- |
| CTC1 | 5 | 93788681 | G | GA | 1 | NM_001145678 | KIAA0825 | frameshift_inserti  on | exon13 | c.2339dupT | p.F780fs | . | . |
| CTC1 | 5 | 93979109 | CA | C | 0.217 | NM_032290 | ANKRD32 | frameshift_deleti  on | exon5 | c.564delA | p.P188fs | . | . |
| CTC1 | 5 | 101834279 | AC | A | 0.215 | NM_001289002 | SLCO6A1 | frameshift_deleti  on | exon1 | c.269delG | p.G90fs | . | . |
| CTC1 | 5 | 112399725 | GC | G | 1 | NM_002387 | MCC | frameshift_deleti  on | exon12 | c.1598delG | p.C533fs | . | . |
| CTC1 | 5 | 114879064 | T | TG | 0.132 | NM_020177 | FEM1C | frameshift_inserti  on | exon2 | c.126dupC | p.T43fs | . | . |
| CTC1 | 5 | 118500912 | CT | C | 0.55 | NM_005509 | DMXL1 | frameshift_deleti  on | exon21 | c.4908delT | p.P1636fs | . | . |
| CTC1 | 5 | 121362699 | GA | G | 0.571 | NM_152546 | SRFBP1 | frameshift_deleti  on | exon8 | c.1169delA | p.E390fs | . | . |
| CTC1 | 5 | 126769148 | AT | A | 0.359 | NM_001256545 | MEGF10 | frameshift_deleti  on | exon14 | c.1788delT | p.D596fs | . | . |
| CTC1 | 5 | 127522321 | TA | T | 0.5 | NM_001256461 | SLC12A2 | nonframeshift_de  letion | exon26 | c.3590delA | p.X1197X | . | . |
| CTC1 | 5 | 131307291 | TG | T | 0.357 | NM_001205251 | ACSL6 | frameshift_deleti  on | exon13 | c.1085delC | p.P362fs | . | . |
| CTC1 | 5 | 131545010 | GT | G | 0.478 | NM_001017973 | P4HA2 | frameshift_deleti  on | exon7 | c.723delA | p.E241fs | . | . |
| CTC1 | 5 | 131630623 | TG | T | 1 | NM_003059 | SLC22A4 | frameshift_deleti  on | exon1 | c.315delG | p.L105fs | . | . |
| CTC1 | 5 | 132736516 | TA | T | 0.314 | NM_015082 | FSTL4 | frameshift_deleti  on | exon4 | c.322delT | p.Y108fs | . | . |
| CTC1 | 5 | 133914698 | TG | T | 0.651 | NM_015288 | JADE2 | frameshift_deleti  on | exon11 | c.2065delG | p.A689fs | . | . |
| CTC1 | 5 | 137275937 | GT | G | 0.25 | NM_001101800 | FAM13B | frameshift_deleti  on | exon22 | c.2640delA | p.K880fs | . | . |
| CTC1 | 5 | 137513262 | AT | A | 0.288 | NM_001164326 | BRD8 | frameshift_deleti  on | exon2 | c.113delA | p.N38fs | . | . |
| CTC1 | 5 | 137519430 | TCC | T | 0.127 | NM_005733 | KIF20A | frameshift_deleti  on | exon10 | c.1194_1195del | p.V398fs | . | . |
| CTC1 | 5 | 139819704 | TC | T | 0.312 | NM_001197030 | ANKHD1 | frameshift_deleti  on | exon4 | c.619delC | p.R207fs | . | . |
| CTC1 | 5 | 139936730 | ACC | A | 0.305 | NM_001253764 | SRA1 | frameshift_deleti  on | exon1 | c.306_306del | p.E102fs | . | . |
| CTC1 | 5 | 140182575 | AT | A | 0.833 | NM_018906 | PCDHA3 | frameshift_deleti  on | exon1 | c.1794delT | p.D598fs | . | . |
| CTC1 | 5 | 140203334 | GACAGC  C | G | 0.353 | NM_018908 | PCDHA5 | nonframeshift_de  letion | exon1 | c.1975_1980del | p.659_660del | . | . |
| CTC1 | 5 | 140237997 | TA | T | 0.28 | NM_018901 | PCDHA10 | frameshift_deleti  on | exon1 | c.2365delA | p.I789fs | . | . |
| CTC1 | 5 | 140480288 | GT | G | 0.378 | NM_018937 | PCDHB3 | frameshift_deleti  on | exon1 | c.56delT | p.V19fs | . | . |
| CTC1 | 5 | 140501653 | GT | G | 0.214 | NM_018938 | PCDHB4 | frameshift_deleti  on | exon1 | c.74delT | p.V25fs | . | . |
| CTC1 | 5 | 140579998 | TG | T | 0.333 | NM_018931 | PCDHB11 | frameshift_deleti  on | exon1 | c.652delG | p.G218fs | . | . |
| CTC1 | 5 | 140581577 | AC | A | 0.6 | NM_018931 | PCDHB11 | frameshift_deleti  on | exon1 | c.2231delC | p.T744fs | . | . |
| CTC1 | 5 | 140711281 | GC | G | 0.431 | NM_018912 | PCDHGA1 | frameshift_deleti  on | exon1 | c.1031delC | p.A344fs | . | . |
| CTC1 | 5 | 140739776 | TC | T | 0.406 | NM_018923 | PCDHGB2 | frameshift_deleti  on | exon1 | c.75delC | p.F25fs | . | . |
| CTC1 | 5 | 140755048 | CA | C | 0.465 | NM_018919 | PCDHGA6 | frameshift_deleti  on | exon1 | c.1399delA | p.R467fs | . | . |
| CTC1 | 5 | 140773236 | GA | G | 0.596 | NM_014004 | PCDHGA8 | frameshift_deleti  on | exon1 | c.857delA | p.E286fs | . | . |
| CTC1 | 5 | 140773291 | CA | C | 0.562 | NM_014004 | PCDHGA8 | frameshift_deleti  on | exon1 | c.912delA | p.A304fs | . | . |
| CTC1 | 5 | 140793272 | GC | G | 0.812 | NM_018913 | PCDHGA10 | frameshift_deleti  on | exon1 | c.531delC | p.S177fs | . | . |
| CTC1 | 5 | 140857273 | GC | G | 0.242 | NM_002588 | PCDHGC3 | frameshift_deleti  on | exon1 | c.1591delC | p.R531fs | . | . |
| CTC1 | 5 | 140871138 | CT | C | 0.481 | NM_018929 | PCDHGC5 | frameshift_deleti  on | exon1 | c.2332delT | p.F778fs | . | . |
| CTC1 | 5 | 140953563 | TGGA | T | 0.367 | NM_001079812 | DIAPH1 | nonframeshift_de  letion | exon15 | c.1824_1826del | p.608_609del | . | . |
| CTC1 | 5 | 141316919 | GC | G | 0.192 | NM_001142603 | KIAA0141 | frameshift_deleti  on | exon11 | c.1307delC | p.A436fs | . | . |
| CTC1 | 5 | 146258290 | A | AGCTG  CTGCT GCTGC | 1 | NM_181675 | PPP2R2B | nonframeshift_in sertion | exon1 | c.57_58insAGC  AGCAGCAGCA GCAGC | p.C20delinsSSS SSSC | . | . |
| CTC1 | 5 | 146777324 | C | CT | 0.333 | NM_001197294 | DPYSL3 | frameshift_inserti  on | exon12 | c.1707dupA | p.D570fs | . | . |
| CTC1 | 5 | 147793824 | AT | A | 0.174 | NM_001271723 | FBXO38 | frameshift_deleti  on | exon10 | c.1220delT | p.I407fs | . | . |
| CTC1 | 5 | 147819270 | AT | A | 0.212 | NM_001271723 | FBXO38 | frameshift_deleti  on | exon18 | c.2351delT | p.I784fs | . | . |
| CTC1 | 5 | 148407702 | GC | G | 0.969 | NM_024577 | SH3TC2 | frameshift_deleti  on | exon11 | c.1592delG | p.C531fs | . | . |
| CTC1 | 5 | 149003606 | TC | T | 0.9 | NM_001001669 | ARHGEF37 | frameshift_deleti  on | exon10 | c.1368delC | p.F456fs | . | . |
| CTC1 | 5 | 149374879 | CT | C | 1 | NM_001243253 | TIGD6 | frameshift_deleti  on | exon2 | c.1032delA | p.Q344fs | . | . |
| CTC1 | 5 | 149375741 | C | CT | 1 | NM_001243253 | TIGD6 | frameshift_inserti  on | exon2 | c.170dupA | p.K57fs | . | . |
| CTC1 | 5 | 149924988 | CA | C | 0.917 | NM_001543 | NDST1 | frameshift_deleti  on | exon11 | c.2086delA | p.K696fs | . | . |
| CTC1 | 5 | 150076428 | TG | T | 0.417 | NM_018047 | RBM22 | frameshift_deleti  on | exon5 | c.296delC | p.A99fs | . | . |
| CTC1 | 5 | 150133085 | CA | C | 0.35 | NM_001135643 | DCTN4 | frameshift_deleti  on | exon3 | c.340delT | p.C114fs | . | . |
| CTC1 | 5 | 150657044 | TA | T | 0.303 | NM_181774 | SLC36A3 | stopgain | exon10 | c.1322delT | p.L441X | . | . |
| CTC1 | 5 | 150701720 | AG | A | 0.6 | NM_181776 | SLC36A2 | frameshift_deleti on | exon9 | c.1066delC | p.L356fs | . | . |
| CTC1 | 5 | 150900870 | GC | G | 0.48 | NM_001447 | FAT2 | frameshift_deleti  on | exon18 | c.11283delG | p.P3761fs | . | . |
| CTC1 | 5 | 150947023 | AT | A | 0.9 | NM_001447 | FAT2 | frameshift_deleti  on | exon1 | c.1469delA | p.N490fs | . | . |
| CTC1 | 5 | 150947622 | C | CT | 0.56 | NM_001447 | FAT2 | frameshift_inserti  on | exon1 | c.870dupA | p.V291fs | . | . |
| CTC1 | 5 | 151049224 | CA | C | 0.286 | NM_003118 | SPARC | frameshift_deleti  on | exon6 | c.451delT | p.Y151fs | . | . |
| CTC1 | 5 | 151202401 | GA | G | 0.185 | NM_000171 | GLRA1 | frameshift_deleti  on | exon9 | c.1182delT | p.P394fs | . | . |
| CTC1 | 5 | 154275847 | CT | C | 0.358 | NM_001252156 | GEMIN5 | frameshift_deleti  on | exon24 | c.3398delA | p.K1133fs | . | . |
| CTC1 | 5 | 156479568 | C | CGTT | 1 | NM_012206 | HAVCR1 | nonframeshift_in  sertion | exon3 | c.476_477insAA  C | p.T159delinsTT | . | . |

| CTC1 | 5 | 156712431 | TC | T | 0.258 | NM_001037332 | CYFIP2 | frameshift_deleti  on | exon2 | c.61delC | p.P21fs | . | . |
| --- | --- | --- | --- | --- | --- | --- | --- | --- | --- | --- | --- | --- | --- |
| CTC1 | 5 | 157182185 | AG | A | 1 | NM_173491 | LSM11 | frameshift_deleti  on | exon4 | c.997delG | p.V333fs | . | . |
| CTC1 | 5 | 159463777 | AT | A | 1 | NM_001282500 | TTC1 | frameshift_deleti  on | exon4 | c.472delT | p.F158fs | . | . |
| CTC1 | 5 | 159992829 | TG | T | 0.484 | NM_025153 | ATP10B | frameshift_deleti  on | exon26 | c.4016delC | p.P1339fs | . | . |
| CTC1 | 5 | 167674657 | GC | G | 1 | NM_001122679 | TENM2 | frameshift_deleti  on | exon27 | c.6687delC | p.G2229fs | . | . |
| CTC1 | 5 | 167674932 | AC | A | 0.6 | NM_001122679 | TENM2 | frameshift_deleti  on | exon27 | c.6962delC | p.T2321fs | . | . |
| CTC1 | 5 | 169533133 | AC | A | 0.543 | NM_012188 | FOXI1 | frameshift_deleti  on | exon1 | c.173delC | p.T58fs | . | . |
| CTC1 | 5 | 169805943 | TG | T | 0.207 | NM_004137 | KCNMB1 | frameshift_deleti  on | exon4 | c.340delC | p.Q114fs | . | . |
| CTC1 | 5 | 170235713 | AT | A | 1 | NM_014211 | GABRP | frameshift_deleti  on | exon8 | c.790delT | p.F264fs | . | . |
| CTC1 | 5 | 175811094 | C | CGT | 1 | NM_001256539 | NOP16 | frameshift_inserti  on | exon5 | c.586_587insAC | p.R196fs | . | . |
| CTC1 | 5 | 175959052 | GC | G | 0.9 | NM_014901 | RNF44 | frameshift_deleti  on | exon3 | c.249delG | p.L83fs | . | . |
| CTC1 | 5 | 176684025 | GT | G | 0.5 | NM_022455 | NSD1 | frameshift_deleti  on | exon13 | c.4840delT | p.F1614fs | . | . |
| CTC1 | 5 | 176936571 | CA | C | 0.415 | NM_024872 | DOK3 | frameshift_deleti  on | exon2 | c.138delT | p.L46fs | . | . |
| CTC1 | 5 | 177546761 | GC | G | 0.333 | NM_015111 | N4BP3 | frameshift_deleti  on | exon2 | c.178delC | p.H60fs | . | . |
| CTC1 | 5 | 178309899 | CA | C | 0.5 | NM_058230 | ZNF354B | frameshift_deleti  on | exon5 | c.447delA | p.T149fs | . | . |
| CTC1 | 5 | 178459579 | GA | G | 0.349 | NM_001136116 | ZNF879 | frameshift_deleti  on | exon5 | c.631delA | p.K211fs | . | . |
| CTC1 | 5 | 180432828 | G | GA | 0.452 | NM_197975 | BTNL3 | frameshift_inserti  on | exon8 | c.1358dupA | p.E453fs | . | . |
| CTC1 | 5 | 180552124 | AC | A | 0.2 | NM_001258283 | OR2V1 | frameshift_deleti  on | exon1 | c.180delG | p.M60fs | . | . |
| CTC1 | 5 | 180687250 | TC | T | 0.262 | NM_032765 | TRIM52 | frameshift_deleti  on | exon1 | c.564delG | p.K188fs | . | . |
| CTC1 | 6 | 572629 | GT | G | 0.348 | NM_018303 | EXOC2 | frameshift_deleti  on | exon13 | c.1333delA | p.T445fs | . | . |
| CTC1 | 6 | 4117675 | ATC | A | 0.145 | NM_001166010 | ECI2 | frameshift_deleti  on | exon9 | c.804_805del | p.E268fs | . | . |
| CTC1 | 6 | 6174979 | AT | A | 0.5 | NM_000129 | F13A1 | frameshift_deleti  on | exon12 | c.1580delA | p.N527fs | . | . |
| CTC1 | 6 | 7585038 | AC | A | 0.25 | NM_001008844 | DSP | frameshift_deleti  on | exon24 | c.5747delC | p.T1916fs | . | . |
| CTC1 | 6 | 10557286 | TA | T | 0.574 | NM_001491 | GCNT2 | frameshift_deleti  on | exon1 | c.631delA | p.K211fs | . | . |
| CTC1 | 6 | 10586678 | TG | T | 0.304 | NM_145655 | GCNT2 | frameshift_deleti  on | exon1 | c.457delG | p.A153fs | . | . |
| CTC1 | 6 | 12122378 | A | AC | 0.4 | NM_002114 | HIVEP1 | frameshift_inserti  on | exon4 | c.2350_2351ins  C | p.K784fs | . | . |
| CTC1 | 6 | 15496555 | C | CT | 0.375 | NM_001267040 | JARID2 | frameshift_inserti  on | exon7 | c.583_584insT | p.P195fs | . | . |
| CTC1 | 6 | 16145265 | TG | T | 0.948 | NM_013262 | MYLIP | frameshift_deleti  on | exon6 | c.966delG | p.V322fs | . | . |
| CTC1 | 6 | 17629486 | TA | T | 0.75 | NM_001278210 | NUP153 | frameshift_deleti  on | exon17 | c.2817delT | p.F939fs | . | . |
| CTC1 | 6 | 24547429 | A | AT | 0.588 | NM_001252328 | KIAA0319 | frameshift_inserti  on | exon17 | c.1415dupA | p.N472fs | . | . |
| CTC1 | 6 | 24810010 | AC | A | 0.571 | NM_001286445 | FAM65B | stopgain | exon21 | c.2977delG | p.V993X | . | . |
| CTC1 | 6 | 24832597 | GT | G | 0.367 | NM_001286445 | FAM65B | frameshift_deleti on | exon16 | c.2230delA | p.T744fs | . | . |
| CTC1 | 6 | 25581166 | CA | C | 0.333 | NM_001173977 | LRRC16A | frameshift_deleti  on | exon30 | c.2758delA | p.K920fs | . | . |
| CTC1 | 6 | 26021921 | TA | T | 0.5 | NM_003538 | HIST1H4A | frameshift_deleti  on | exon1 | c.16delA | p.K6fs | . | . |
| CTC1 | 6 | 26225751 | AG | A | 0.191 | NM_003532 | HIST1H3E | frameshift_deleti  on | exon1 | c.370delG | p.D124fs | . | . |
| CTC1 | 6 | 27835029 | CT | C | 0.182 | NM_005322 | HIST1H1B | frameshift_deleti  on | exon1 | c.278delA | p.K93fs | . | . |
| CTC1 | 6 | 27861552 | CG | C | 0.737 | NM_003527 | HIST1H2BO | frameshift_deleti  on | exon1 | c.313delG | p.G105fs | . | . |
| CTC1 | 6 | 27879088 | C | CT | 0.173 | NM_033057 | OR2B2 | frameshift_inserti  on | exon1 | c.1009dupA | p.S337fs | . | . |
| CTC1 | 6 | 28194915 | CA | C | 0.4 | NM_001199479 | ZSCAN9 | frameshift_deleti  on | exon2 | c.54delA | p.A18fs | . | . |
| CTC1 | 6 | 28227152 | GCC | G | 0.585 | NM_001007531 | NKAPL | frameshift_deleti  on | exon1 | c.4_5del | p.P2fs | . | . |
| CTC1 | 6 | 28402449 | GA | G | 0.923 | NM_001012455 | ZSCAN23 | frameshift_deleti  on | exon4 | c.962delT | p.F321fs | . | . |
| CTC1 | 6 | 28963439 | TG | T | 0.391 | NM_001010877 | ZNF311 | frameshift_deleti  on | exon7 | c.1339delC | p.Q447fs | . | . |
| CTC1 | 6 | 29627338 | ACT | A | 0.194 | NM_001008228 | MOG | frameshift_deleti  on | exon2 | c.332_333del | p.T111fs | . | . |
| CTC1 | 6 | 30297479 | TG | T | 0.538 | NM_001199119 | TRIM39-  RPP21 | frameshift_deleti  on | exon1 | c.386delG | p.C129fs | . | . |
| CTC1 | 6 | 30459074 | AG | A | 1 | NM_005516 | HLA-E | frameshift_deleti  on | exon4 | c.772delG | p.G258fs | . | . |
| CTC1 | 6 | 30514047 | CA | C | 0.443 | NM_005275 | GNL1 | frameshift_deleti  on | exon12 | c.1625delT | p.L542fs | . | . |
| CTC1 | 6 | 30515136 | TG | T | 0.224 | NM_005275 | GNL1 | frameshift_deleti  on | exon9 | c.1270delC | p.Q424fs | . | . |
| CTC1 | 6 | 30558477 | G | GA | 0.361 | NM_001090 | ABCF1 | frameshift_inserti  on | exon24 | c.2424dupA | p.X808delinsX | . | . |
| CTC1 | 6 | 30899497 | TC | T | 0.592 | NM_205854 | SFTA2 | frameshift_deleti  on | exon2 | c.136delG | p.E46fs | . | . |
| CTC1 | 6 | 30917700 | GC | G | 0.195 | NM_080870 | DPCR1 | frameshift_deleti  on | exon2 | c.1460delC | p.A487fs | . | . |
| CTC1 | 6 | 31127435 | GC | G | 0.811 | NM_001077511 | TCF19 | frameshift_deleti  on | exon2 | c.190delC | p.P64fs | . | . |
| CTC1 | 6 | 31617106 | GC | G | 0.476 | NM_001098534 | BAG6 | frameshift_deleti  on | exon4 | c.292delG | p.A98fs | . | . |
| CTC1 | 6 | 31637681 | GC | G | 0.182 | NM_001282385 | CSNK2B | frameshift_deleti  on | exon7 | c.618delC | p.S206fs | . | . |
| CTC1 | 6 | 31709055 | TC | T | 0.288 | NM_002441 | MSH5 | frameshift_deleti  on | exon3 | c.264delC | p.L88fs | . | . |
| CTC1 | 6 | 31733705 | CG | C | 1 | NM_025258 | VWA7 | frameshift_deleti  on | exon16 | c.2453delC | p.P818fs | . | . |
| CTC1 | 6 | 31857308 | CTCT | C | 0.135 | NM_001289413 | EHMT2 | nonframeshift_de  letion | exon7 | c.1104_1106del | p.368_369del | . | . |
| CTC1 | 6 | 31868895 | A | AG | 0.213 | NM_001178063 | C2 | frameshift_inserti  on | exon1 | c.53dupG | p.R18fs | . | . |
| CTC1 | 6 | 31903707 | GC | G | 1 | NM_001282457 | C2 | frameshift_deleti  on | exon3 | c.120delC | p.S40fs | . | . |

| CTC1 | 6 | 31922429 | CT | C | 0.242 | NM_002904 | NELFE | frameshift_deleti  on | exon7 | c.644delA | p.E215fs | . | . |
| --- | --- | --- | --- | --- | --- | --- | --- | --- | --- | --- | --- | --- | --- |
| CTC1 | 6 | 32012891 | TC | T | 0.445 | NM_032470 | TNXB | frameshift_deleti  on | exon1 | c.105delG | p.Q35fs | . | . |
| CTC1 | 6 | 32150445 | AC | A | 0.395 | NM_001136 | AGER | frameshift_deleti  on | exon7 | c.721delG | p.V241fs | . | . |
| CTC1 | 6 | 32261705 | T | TTTC | 0.333 | NM_006781 | C6orf10 | nonframeshift_in  sertion | exon23 | c.744_745insG  AA | p.N249delinsE  N | . | . |
| CTC1 | 6 | 32336588 | A | AT | 0.192 | NM_001286474 | C6orf10 | frameshift_inserti  on | exon3 | c.103dupA | p.M35fs | . | . |
| CTC1 | 6 | 32411673 | AG | A | 0.375 | NM_019111 | HLA-DRA | frameshift_deleti  on | exon4 | c.752delG | p.R251fs | . | . |
| CTC1 | 6 | 32917458 | TG | T | 0.792 | NM_006120 | HLA-DMA | frameshift_deleti  on | exon3 | c.581delC | p.P194fs | . | . |
| CTC1 | 6 | 33690685 | GA | G | 0.267 | NM_054111 | IP6K3 | frameshift_deleti  on | exon6 | c.1044delT | p.A348fs | . | . |
| CTC1 | 6 | 35392192 | CA | C | 1 | NM_001171820 | PPARD | frameshift_deleti  on | exon5 | c.421delA | p.K141fs | . | . |
| CTC1 | 6 | 36020489 | AC | A | 0.846 | NM_001315 | MAPK14 | frameshift_deleti  on | exon2 | c.131delC | p.T44fs | . | . |
| CTC1 | 6 | 36689438 | TC | T | 0.617 | NM_001257357 | RAB44 | frameshift_deleti  on | exon9 | c.1528delC | p.P510fs | . | . |
| CTC1 | 6 | 36689500 | AG | A | 0.612 | NM_001257357 | RAB44 | frameshift_deleti  on | exon9 | c.1590delG | p.K530fs | . | . |
| CTC1 | 6 | 36710247 | T | TC | 0.2 | NM_020939 | CPNE5 | frameshift_inserti  on | exon21 | c.1579dupG | p.D527fs | . | . |
| CTC1 | 6 | 36931065 | CA | C | 0.353 | NM_153370 | PI16 | frameshift_deleti  on | exon5 | c.948delA | p.P316fs | . | . |
| CTC1 | 6 | 36931252 | CA | C | 0.24 | NM_153370 | PI16 | frameshift_deleti  on | exon5 | c.1135delA | p.K379fs | . | . |
| CTC1 | 6 | 38819420 | TG | T | 0.61 | NM_001206927 | DNAH8 | frameshift_deleti  on | exon39 | c.5437delG | p.G1813fs | . | . |
| CTC1 | 6 | 38841083 | TA | T | 0.412 | NM_001206927 | DNAH8 | frameshift_deleti  on | exon52 | c.7552delA | p.T2518fs | . | . |
| CTC1 | 6 | 41029307 | CT | C | 0.164 | NM_006789 | APOBEC2 | frameshift_deleti  on | exon2 | c.373delT | p.S125fs | . | . |
| CTC1 | 6 | 41754570 | A | ACTC | 1 | NM_013397 | PRICKLE4 | nonframeshift_in  sertion | exon8 | c.858_859insCT  C | p.T286delinsTL | . | . |
| CTC1 | 6 | 41754573 | C | CCTT | 1 | NM_013397 | PRICKLE4 | nonframeshift_in  sertion | exon8 | c.861_862insCT  T | p.L287delinsLL | . | . |
| CTC1 | 6 | 41754574 | C | CTTT | 0.99 | NM_013397 | PRICKLE4 | nonframeshift_in  sertion | exon8 | c.862_863insTT  T | p.L288delinsLF | . | . |
| CTC1 | 6 | 42713673 | GC | G | 0.154 | NM_003192 | TBCC | frameshift_deleti  on | exon1 | c.138delG | p.K46fs | . | . |
| CTC1 | 6 | 42796804 | GC | G | 0.686 | NM_015349 | GLTSCR1L | frameshift_deleti  on | exon5 | c.734delC | p.A245fs | . | . |
| CTC1 | 6 | 42897357 | TTGC | T | 0.31 | NM_006586 | CNPY3 | nonframeshift_de  letion | exon1 | c.50_52del | p.17_18del | . | . |
| CTC1 | 6 | 42905841 | T | TGGG | 0.167 | NM_006586 | CNPY3 | nonframeshift_in  sertion | exon5 | c.509_510insG  GG | p.V170delinsV  G | . | . |
| CTC1 | 6 | 42977148 | T | TC | 0.8 | NM_180977 | PPP2R5D | frameshift_inserti  on | exon10 | c.1023dupC | p.F341fs | . | . |
| CTC1 | 6 | 43040661 | G | GT | 1 | NM_001289035 | KLC4 | frameshift_inserti  on | exon12 | c.1357_1358ins  T | p.E453fs | . | . |
| CTC1 | 6 | 43098002 | C | CA | 0.398 | NM_001270398 | PTK7 | frameshift_inserti  on | exon4 | c.529_530insA | p.L177fs | . | . |
| CTC1 | 6 | 49660525 | CT | C | 0.245 | NM_001142407 | CRISP2 | frameshift_deleti  on | exon9 | c.692delA | p.K231fs | . | . |
| CTC1 | 6 | 55638982 | GT | G | 0.333 | NM_021073 | BMP5 | frameshift_deleti  on | exon4 | c.891delA | p.K297fs | . | . |
| CTC1 | 6 | 56566736 | GA | G | 1 | NM_183380 | DST | frameshift_deleti  on | exon4 | c.270delT | p.F90fs | . | . |
| CTC1 | 6 | 56999554 | TC | T | 1 | NM_001031623 | ZNF451 | frameshift_deleti  on | exon7 | c.589delC | p.P197fs | . | . |
| CTC1 | 6 | 57012217 | CA | C | 1 | NM_001031623 | ZNF451 | frameshift_deleti  on | exon10 | c.1335delA | p.P445fs | . | . |
| CTC1 | 6 | 57015657 | CA | C | 0.25 | NM_001031623 | ZNF451 | frameshift_deleti  on | exon11 | c.2750delA | p.Q917fs | . | . |
| CTC1 | 6 | 57185269 | GA | G | 0.857 | NM_001282487 | PRIM2 | frameshift_deleti  on | exon3 | c.170delA | p.E57fs | . | . |
| CTC1 | 6 | 65098680 | TG | T | 0.917 | NM_001142800 | EYS | frameshift_deleti  on | exon29 | c.5980delC | p.Q1994fs | . | . |
| CTC1 | 6 | 69944930 | G | GA | 0.174 | NM_001704 | BAI3 | frameshift_inserti  on | exon19 | c.2615dupA | p.E872fs | . | . |
| CTC1 | 6 | 71238032 | CT | C | 0.37 | NM_001162529 | FAM135A | frameshift_deleti  on | exon14 | c.3653delT | p.L1218fs | . | . |
| CTC1 | 6 | 73110347 | T | TC | 0.833 | NM_001168411 | RIMS1 | frameshift_inserti  on | exon6 | c.592dupC | p.T197fs | . | . |
| CTC1 | 6 | 74125243 | CT | C | 0.429 | NM_018665 | DDX43 | frameshift_deleti  on | exon15 | c.1770delT | p.T590fs | . | . |
| CTC1 | 6 | 74228547 | C | CT | 0.169 | NM_001402 | EEF1A1 | frameshift_inserti  on | exon5 | c.645dupA | p.V216fs | . | . |
| CTC1 | 6 | 75807002 | AC | A | 0.205 | NM_080645 | COL12A1 | frameshift_deleti  on | exon44 | c.5051delG | p.G1684fs | . | . |
| CTC1 | 6 | 76023936 | CTTG | C | 0.3 | NM_015687 | FILIP1 | nonframeshift_de  letion | exon5 | c.1609_1611del | p.537_537del | . | . |
| CTC1 | 6 | 76407207 | TTTTA | T | 1 | NM_001100409 | SENP6 | frameshift_deleti  on | exon17 | c.2242_2245del | p.F748fs | . | . |
| CTC1 | 6 | 79577303 | CAA | C | 0.273 | NM_001010844 | IRAK1BP1 | frameshift_deleti  on | exon1 | c.11_12del | p.Q4fs | . | . |
| CTC1 | 6 | 80741214 | A | AT | 0.357 | NM_001166691 | TTK | frameshift_inserti  on | exon14 | c.1550dupT | p.I517fs | . | . |
| CTC1 | 6 | 83075888 | G | GT | 1 | NM_001166392 | TPBG | frameshift_inserti  on | exon2 | c.1210_1211ins  T | p.E404fs | . | . |
| CTC1 | 6 | 84799174 | C | CA | 0.206 | NM_138409 | MRAP2 | frameshift_inserti  on | exon4 | c.593dupA | p.Q198fs | . | . |
| CTC1 | 6 | 86346855 | CA | C | 0.364 | NM_001159673 | SYNCRIP | frameshift_deleti  on | exon5 | c.201delT | p.F67fs | . | . |
| CTC1 | 6 | 87969702 | CA | C | 0.6 | NM_015021 | ZNF292 | frameshift_deleti  on | exon8 | c.6356delA | p.Q2119fs | . | . |
| CTC1 | 6 | 90383171 | C | CA | 0.373 | NM_014611 | MDN1 | stopgain | exon80 | c.13257dupT | p.E4420_V4421  delinsX | . | . |
| CTC1 | 6 | 90513119 | AT | A | 0.839 | NM_014611 | MDN1 | frameshift_deleti  on | exon2 | c.256delA | p.I86fs | . | . |
| CTC1 | 6 | 97679360 | CT | C | 0.4 | NM_198468 | MMS22L | frameshift_deleti  on | exon13 | c.1470delA | p.K490fs | . | . |
| CTC1 | 6 | 106553144 | AC | A | 0.733 | NM_182907 | PRDM1 | frameshift_deleti  on | exon3 | c.708delC | p.H236fs | . | . |
| CTC1 | 6 | 107390489 | CG | C | 0.833 | NM_001080450 | BEND3 | frameshift_deleti  on | exon5 | c.1905delC | p.F635fs | . | . |
| CTC1 | 6 | 109215705 | GC | G | 0.5 | NM_001286609 | ARMC2 | frameshift_deleti  on | exon5 | c.213delC | p.C71fs | . | . |
| CTC1 | 6 | 109294698 | TG | T | 0.206 | NM_001286609 | ARMC2 | frameshift_deleti  on | exon17 | c.2091delG | p.L697fs | . | . |

| CTC1 | 6 | 110062719 | GT | G | 0.743 | NM_014845 | FIG4 | frameshift_deleti  on | exon8 | c.849delT | p.R283fs | . | . |
| --- | --- | --- | --- | --- | --- | --- | --- | --- | --- | --- | --- | --- | --- |
| CTC1 | 6 | 111693957 | A | AT | 1 | NM_002912 | REV3L | frameshift_inserti  on | exon14 | c.5600dupA | p.N1867fs | . | . |
| CTC1 | 6 | 116600465 | T | TCAC | 1 | NM_003309 | TSPYL1 | nonframeshift_in  sertion | exon1 | c.528_529insGT  G | p.K177delinsVK | . | . |
| CTC1 | 6 | 116600467 | A | ACCC | 0.915 | NM_003309 | TSPYL1 | nonframeshift_in  sertion | exon1 | c.526_527insG  GG | p.V176delinsG  V | . | . |
| CTC1 | 6 | 116600469 | C | CACT | 0.916 | NM_003309 | TSPYL1 | nonframeshift_in  sertion | exon1 | c.524_525insA  GT | p.V175delinsV  V | . | . |
| CTC1 | 6 | 116600471 | C | CCAT | 0.979 | NM_003309 | TSPYL1 | nonframeshift_in  sertion | exon1 | c.522_523insAT  G | p.V175delinsM  V | . | . |
| CTC1 | 6 | 116758308 | GC | G | 0.3 | NM_001080976 | DSE | frameshift_deleti  on | exon6 | c.2678delC | p.A893fs | . | . |
| CTC1 | 6 | 118886728 | AATTT | A | 0.333 | NM_001042475 | CEP85L | frameshift_deleti  on | exon3 | c.980_983del | p.Q327fs | . | . |
| CTC1 | 6 | 119136359 | TA | T | 0.173 | NM_017696 | MCM9 | stopgain | exon12 | c.3059delT | p.L1020X | . | . |
| CTC1 | 6 | 131184810 | TCA | T | 0.188 | NM_001135554 | EPB41L2 | frameshift_deleti on | exon13 | c.1880_1881del | p.V627fs | . | . |
| CTC1 | 6 | 131191072 | ACTG | A | 0.429 | NM_001199388 | EPB41L2 | nonframeshift_de  letion | exon13 | c.2025_2027del | p.675_676del | . | . |
| CTC1 | 6 | 132061444 | CG | C | 0.273 | NM_005021 | ENPP3 | frameshift_deleti  on | exon24 | c.2382delG | p.P794fs | . | . |
| CTC1 | 6 | 133072355 | CT | C | 1 | NM_004665 | VNN2 | frameshift_deleti  on | exon5 | c.1128delA | p.K376fs | . | . |
| CTC1 | 6 | 134492178 | C | CT | 0.728 | NM_001143677 | SGK1 | frameshift_inserti  on | exon10 | c.1104_1105ins  A | p.G369fs | . | . |
| CTC1 | 6 | 135539079 | AT | A | 0.163 | NM_001161657 | MYB | frameshift_deleti  on | exon14 | c.1630delT | p.Y544fs | . | . |
| CTC1 | 6 | 135784433 | AT | A | 0.225 | NM_001134830 | AHI1 | frameshift_deleti  on | exon6 | c.760delA | p.I254fs | . | . |
| CTC1 | 6 | 137146399 | CT | C | 0.476 | NM_000288 | PEX7 | frameshift_deleti  on | exon2 | c.179delT | p.L60fs | . | . |
| CTC1 | 6 | 137323464 | TTG | T | 0.186 | NM_001278723 | IL20RA | frameshift_deleti  on | exon6 | c.558_559del | p.D186fs | . | . |
| CTC1 | 6 | 138200113 | GA | G | 0.258 | NM_001270507 | TNFAIP3 | frameshift_deleti  on | exon7 | c.1532delA | p.D511fs | . | . |
| CTC1 | 6 | 138656008 | G | GC | 0.385 | NM_020340 | KIAA1244 | frameshift_inserti  on | exon33 | c.6026dupC | p.A2009fs | . | . |
| CTC1 | 6 | 143828476 | CA | C | 0.6 | NM_032020 | FUCA2 | frameshift_deleti  on | exon2 | c.309delT | p.F103fs | . | . |
| CTC1 | 6 | 144086503 | GC | G | 0.361 | NM_001100165 | PHACTR2 | frameshift_deleti  on | exon5 | c.561delC | p.S187fs | . | . |
| CTC1 | 6 | 144258802 | TG | T | 0.231 | NM_001013623 | ZC2HC1B | frameshift_deleti  on | exon7 | c.616delG | p.A206fs | . | . |
| CTC1 | 6 | 144262780 | AG | A | 0.692 | NM_001289038 | PLAGL1 | frameshift_deleti  on | exon4 | c.1016delC | p.P339fs | . | . |
| CTC1 | 6 | 146121253 | GT | G | 0.609 | NM_032145 | FBXO30 | frameshift_deleti  on | exon3 | c.2226delA | p.K742fs | . | . |
| CTC1 | 6 | 147109546 | TA | T | 0.545 | NM_024694 | ADGB | frameshift_deleti  on | exon33 | c.4338delA | p.V1446fs | . | . |
| CTC1 | 6 | 147136203 | C | CA | 0.323 | NM_024694 | ADGB | frameshift_inserti  on | exon36 | c.4855dupA | p.D1618fs | . | . |
| CTC1 | 6 | 150291220 | C | CT | 0.833 | NM_025218 | ULBP1 | frameshift_inserti  on | exon4 | c.695dupT | p.L232fs | . | . |
| CTC1 | 6 | 151673438 | TA | T | 0.899 | NM_144497 | AKAP12 | frameshift_deleti  on | exon2 | c.3619delA | p.K1207fs | . | . |
| CTC1 | 6 | 151687551 | CCGGGA  GGAG | C | 0.16 | NM_020861 | ZBTB2 | nonframeshift_de  letion | exon3 | c.641_649del | p.214_217del | . | . |
| CTC1 | 6 | 151789830 | TA | T | 0.814 | NM_001286562 | C6orf211 | frameshift_deleti  on | exon4 | c.555delA | p.V185fs | . | . |
| CTC1 | 6 | 153316453 | AT | A | 0.385 | NM_001114184 | MTRF1L | frameshift_deleti  on | exon3 | c.340delA | p.I114fs | . | . |
| CTC1 | 6 | 155153422 | TG | T | 0.583 | NM_014892 | SCAF8 | frameshift_deleti  on | exon20 | c.2710delG | p.G904fs | . | . |
| CTC1 | 6 | 155154326 | CA | C | 0.902 | NM_014892 | SCAF8 | frameshift_deleti  on | exon20 | c.3614delA | p.Q1205fs | . | . |
| CTC1 | 6 | 155451273 | GC | G | 0.246 | NM_012454 | TIAM2 | frameshift_deleti  on | exon3 | c.917delC | p.A306fs | . | . |
| CTC1 | 6 | 159457858 | CT | C | 0.857 | NM_001278733 | TAGAP | frameshift_deleti  on | exon6 | c.1007delA | p.K336fs | . | . |
| CTC1 | 6 | 159660779 | GCCACC  ACCCGC CGCACG | G | 0.778 | NM_032532 | FNDC1 | nonframeshift_de letion | exon14 | c.4412_4429del | p.1471_1477de l | . | . |
| CTC1 | 6 | 160671634 | GA | G | 0.306 | NM_003058 | SLC22A2 | frameshift_deleti  on | exon3 | c.618delT | p.F206fs | . | . |
| CTC1 | 6 | 161026213 | AC | A | 0.7 | NM_005577 | LPA | frameshift_deleti  on | exon19 | c.2809delG | p.V937fs | . | . |
| CTC1 | 6 | 161519350 | CCTG | C | 1 | NM_005922 | MAP3K4 | nonframeshift_de  letion | exon17 | c.3566_3568del | p.1189_1190de  l | . | . |
| CTC1 | 6 | 166914390 | GC | G | 0.357 | NM_021135 | RPS6KA2 | frameshift_deleti  on | exon7 | c.588delG | p.G196fs | . | . |
| CTC1 | 6 | 167585729 | GC | G | 0.352 | NM_001145121 | TCP10L2 | frameshift_deleti  on | exon2 | c.98delC | p.A33fs | . | . |
| CTC1 | 6 | 168443250 | GA | G | 0.333 | NM_030615 | KIF25 | frameshift_deleti  on | exon8 | c.840delA | p.G280fs | . | . |
| CTC1 | 6 | 169634907 | C | CA | 0.409 | NM_003247 | THBS2 | stopgain | exon11 | c.1572dupT | p.E525_P526de  linsX | . | . |
| CTC1 | 6 | 169649038 | TC | T | 0.282 | NM_003247 | THBS2 | frameshift_deleti  on | exon4 | c.82delG | p.D28fs | . | . |
| CTC1 | 6 | 170697466 | T | TC | 0.552 | NM_001286381 | FAM120B | frameshift_inserti  on | exon5 | c.372dupC | p.F124fs | . | . |
| CTC1 | 6 | 170871037 | GCAA | G | 0.621 | NM_001172085 | TBP | nonframeshift_de  letion | exon2 | c.154_156del | p.52_52del | . | . |
| CTC1 | 6 | 170871046 | ACAGCA  GCAG | A | 1 | NM_001172085 | TBP | nonframeshift_de  letion | exon2 | c.163_171del | p.55_57del | . | . |
| CTC1 | 6 | 170871046 | ACAGCA  GCAGCA | A | 1 | NM_001172085 | TBP | nonframeshift_de  letion | exon2 | c.163_174del | p.55_58del | . | . |
| CTC1 | 7 | 716901 | G | GT | 0.339 | NM_001164758 | PRKAR1B | frameshift_inserti  on | exon4 | c.404dupA | p.N135fs | . | . |
| CTC1 | 7 | 2552881 | A | AGATG | 0.8 | NM_001166355 | LFNG | frameshift_inserti  on | exon2 | c.138_139insG  ATG | p.T46fs | . | . |
| CTC1 | 7 | 2552902 | G | GATGA | 0.714 | NM_001166355 | LFNG | frameshift_inserti  on | exon2 | c.159_160insAT  GA | p.W53fs | . | . |
| CTC1 | 7 | 2701364 | CA | C | 0.857 | NM_025250 | TTYH3 | frameshift_deleti  on | exon14 | c.1564delA | p.S522fs | . | . |
| CTC1 | 7 | 4780545 | TC | T | 0.279 | NM_001037165 | FOXK1 | frameshift_deleti  on | exon2 | c.638delC | p.S213fs | . | . |
| CTC1 | 7 | 5334545 | AG | A | 0.455 | NM_001040661 | SLC29A4 | frameshift_deleti  on | exon6 | c.600delG | p.Q200fs | . | . |
| CTC1 | 7 | 6080829 | AT | A | 0.338 | NM_001134335 | EIF2AK1 | frameshift_deleti  on | exon9 | c.809delA | p.N270fs | . | . |

| CTC1 | 7 | 6547888 | G | GA | 0.333 | NM_001145118 | GRID2IP | frameshift_inserti  on | exon13 | c.2271_2272ins  T | p.P758fs | . | . |
| --- | --- | --- | --- | --- | --- | --- | --- | --- | --- | --- | --- | --- | --- |
| CTC1 | 7 | 11022256 | GA | G | 0.317 | NM_014660 | PHF14 | frameshift_deleti  on | exon3 | c.371delA | p.E124fs | . | . |
| CTC1 | 7 | 15725797 | ATGG | A | 1 | NM_005924 | MEOX2 | nonframeshift_de  letion | exon1 | c.228_230del | p.76_77del | . | . |
| CTC1 | 7 | 16705119 | GA | G | 0.271 | NM_001159767 | BZW2 | frameshift_deleti  on | exon2 | c.52delA | p.K18fs | . | . |
| CTC1 | 7 | 17349565 | AG | A | 0.182 | NM_001621 | AHR | frameshift_deleti  on | exon2 | c.72delG | p.K24fs | . | . |
| CTC1 | 7 | 17379452 | A | AC | 0.333 | NM_001621 | AHR | frameshift_inserti  on | exon10 | c.2004dupC | p.D668fs | . | . |
| CTC1 | 7 | 20683132 | GT | G | 0.188 | NM_001163941 | ABCB5 | frameshift_deleti  on | exon7 | c.556delT | p.F186fs | . | . |
| CTC1 | 7 | 20725363 | TA | T | 0.222 | NM_178559 | ABCB5 | frameshift_deleti  on | exon7 | c.580delA | p.T194fs | . | . |
| CTC1 | 7 | 26224961 | CT | C | 0.852 | NM_004289 | NFE2L3 | frameshift_deleti  on | exon4 | c.1644delT | p.P548fs | . | . |
| CTC1 | 7 | 26386068 | TC | T | 0.293 | NM_001199835 | SNX10 | frameshift_deleti  on | exon2 | c.7delC | p.P3fs | . | . |
| CTC1 | 7 | 27135316 | A | ATGG | 0.214 | NM_005522 | HOXA1 | nonframeshift_in  sertion | exon1 | c.215_216insCC  A | p.H72delinsHH | . | . |
| CTC1 | 7 | 34889211 | CGG | C | 0.267 | NM_207172 | NPSR1 | frameshift_deleti  on | exon9 | c.1061_1062del | p.R354fs | . | . |
| CTC1 | 7 | 37989893 | GT | G | 0.382 | NM_001242948 | EPDR1 | frameshift_deleti  on | exon3 | c.388delT | p.F130fs | . | . |
| CTC1 | 7 | 39379287 | CCAG | C | 0.179 | NM_001166018 | POU6F2 | nonframeshift_de  letion | exon6 | c.559_561del | p.187_187del | . | . |
| CTC1 | 7 | 39379485 | AC | A | 0.619 | NM_001166018 | POU6F2 | frameshift_deleti  on | exon6 | c.757delC | p.P253fs | . | . |
| CTC1 | 7 | 39472826 | C | CT | 0.25 | NM_001166018 | POU6F2 | frameshift_inserti  on | exon8 | c.1178dupT | p.L393fs | . | . |
| CTC1 | 7 | 44150637 | GC | G | 0.5 | NM_001129 | AEBP1 | frameshift_deleti  on | exon13 | c.1612delC | p.L538fs | . | . |
| CTC1 | 7 | 44185280 | GC | G | 0.875 | NM_000162 | GCK | frameshift_deleti  on | exon9 | c.1068delG | p.G356fs | . | . |
| CTC1 | 7 | 44573423 | TC | T | 0.759 | NM_001101648 | NPC1L1 | frameshift_deleti  on | exon7 | c.2195delG | p.R732fs | . | . |
| CTC1 | 7 | 44839430 | AC | A | 0.712 | NM_021130 | PPIA | frameshift_deleti  on | exon4 | c.320delC | p.T107fs | . | . |
| CTC1 | 7 | 45931568 | GT | G | 0.414 | NM_000596 | IGFBP1 | frameshift_deleti  on | exon3 | c.558delT | p.S186fs | . | . |
| CTC1 | 7 | 47835617 | CT | C | 0.244 | NM_138295 | PKD1L1 | frameshift_deleti  on | exon55 | c.8324delA | p.K2775fs | . | . |
| CTC1 | 7 | 50534920 | G | GA | 0.343 | NM_001242888 | DDC | frameshift_inserti  on | exon11 | c.999dupT | p.R334fs | . | . |
| CTC1 | 7 | 50534973 | TG | T | 0.455 | NM_001242888 | DDC | frameshift_deleti  on | exon11 | c.946delC | p.Q316fs | . | . |
| CTC1 | 7 | 51092938 | TG | T | 0.75 | NM_015198 | COBL | frameshift_deleti  on | exon12 | c.3635delC | p.P1212fs | . | . |
| CTC1 | 7 | 51098567 | GTCT | G | 0.4 | NM_015198 | COBL | nonframeshift_de  letion | exon9 | c.1443_1445del | p.481_482del | . | . |
| CTC1 | 7 | 56087313 | TG | T | 0.552 | NM_004577 | PSPH | frameshift_deleti  on | exon5 | c.254delC | p.P85fs | . | . |
| CTC1 | 7 | 64378025 | TCA | T | 0.438 | NM_021148 | ZNF273 | frameshift_deleti  on | exon2 | c.170_171del | p.S57fs | . | . |
| CTC1 | 7 | 65419353 | T | TTTA | 0.626 | NM_001284342 | VKORC1L1 | nonframeshift_in  sertion | exon2 | c.487_488insTT  A | p.F163delinsFI | . | . |
| CTC1 | 7 | 66103264 | A | AG | 0.361 | NM_001167961 | KCTD7 | frameshift_inserti  on | exon3 | c.340dupG | p.S113fs | . | . |
| CTC1 | 7 | 66104010 | CT | C | 0.44 | NM_001167961 | KCTD7 | frameshift_deleti  on | exon4 | c.662delT | p.L221fs | . | . |
| CTC1 | 7 | 73256387 | CT | C | 0.271 | NM_152559 | WBSCR27 | frameshift_deleti  on | exon2 | c.83delA | p.K28fs | . | . |
| CTC1 | 7 | 73638086 | AT | A | 0.357 | NM_014146 | LAT2 | frameshift_deleti  on | exon10 | c.422delT | p.I141fs | . | . |
| CTC1 | 7 | 73933871 | A | AC | 0.333 | NM_001199207 | GTF2IRD1 | frameshift_inserti  on | exon6 | c.834_835insC | p.P278fs | . | . |
| CTC1 | 7 | 73969748 | TC | T | 0.333 | NM_016328 | GTF2IRD1 | frameshift_deleti  on | exon19 | c.1993delC | p.P665fs | . | . |
| CTC1 | 7 | 73969760 | GC | G | 0.533 | NM_016328 | GTF2IRD1 | frameshift_deleti  on | exon19 | c.2005delC | p.P669fs | . | . |
| CTC1 | 7 | 76240785 | CA | C | 0.887 | NM_012230 | POMZP3 | frameshift_deleti  on | exon6 | c.560delT | p.L187fs | . | . |
| CTC1 | 7 | 82474706 | CT | C | 0.857 | NM_014510 | PCLO | frameshift_deleti  on | exon13 | c.13926delA | p.S4642fs | . | . |
| CTC1 | 7 | 82581488 | T | TTCA | 1 | NM_014510 | PCLO | nonframeshift_in  sertion | exon5 | c.8780_8781ins  TGA | p.E2927delinsD  E | . | . |
| CTC1 | 7 | 82785143 | AT | A | 0.496 | NM_014510 | PCLO | frameshift_deleti  on | exon2 | c.813delA | p.K271fs | . | . |
| CTC1 | 7 | 87160612 | TC | T | 0.263 | NM_000927 | ABCB1 | frameshift_deleti  on | exon22 | c.2682delG | p.G894fs | . | . |
| CTC1 | 7 | 87229433 | CT | C | 0.478 | NM_000927 | ABCB1 | frameshift_deleti  on | exon3 | c.67delA | p.S23fs | . | . |
| CTC1 | 7 | 90042407 | AT | A | 0.36 | NM_001185073 | CLDN12 | frameshift_deleti  on | exon3 | c.418delT | p.F140fs | . | . |
| CTC1 | 7 | 90895896 | CA | C | 0.5 | NM_003505 | FZD1 | frameshift_deleti  on | exon1 | c.1702delA | p.S568fs | . | . |
| CTC1 | 7 | 92140311 | CT | C | 1 | NM_000466 | PEX1 | frameshift_deleti  on | exon8 | c.1533delA | p.K511fs | . | . |
| CTC1 | 7 | 92731025 | AT | A | 0.462 | NM_001193307 | SAMD9 | frameshift_deleti  on | exon2 | c.4385delA | p.N1462fs | . | . |
| CTC1 | 7 | 92733007 | CA | C | 0.308 | NM_001193307 | SAMD9 | frameshift_deleti  on | exon2 | c.2403delT | p.F801fs | . | . |
| CTC1 | 7 | 94059674 | TTG | T | 0.344 | NM_000089 | COL1A2 | frameshift_deleti  on | exon52 | c.4071_4072del | p.F1357fs | . | . |
| CTC1 | 7 | 94059675 | TG | T | 0.167 | NM_000089 | COL1A2 | frameshift_deleti  on | exon52 | c.4072delG | p.V1358fs | . | . |
| CTC1 | 7 | 94293545 | CG | C | 0.194 | NM_001040152 | PEG10 | frameshift_deleti  on | exon2 | c.678delG | p.S226fs | . | . |
| CTC1 | 7 | 95157515 | TG | T | 0.151 | NM_016116 | ASB4 | frameshift_deleti  on | exon3 | c.879delG | p.L293fs | . | . |
| CTC1 | 7 | 95442569 | TA | T | 0.5 | NM_001135556 | DYNC1I1 | frameshift_deleti  on | exon4 | c.235delA | p.M79fs | . | . |
| CTC1 | 7 | 95813647 | GT | G | 0.5 | NM_001160210 | SLC25A13 | frameshift_deleti  on | exon11 | c.1121delA | p.N374fs | . | . |
| CTC1 | 7 | 96747051 | GTTT | G | 0.816 | NM_020186 | ACN9 | nonframeshift_de  letion | exon1 | c.17_19del | p.6_7del | . | . |
| CTC1 | 7 | 98563467 | CA | C | 0.487 | NM_003496 | TRRAP | frameshift_deleti  on | exon47 | c.7051delA | p.K2351fs | . | . |
| CTC1 | 7 | 99129833 | TC | T | 0.312 | NM_014569 | ZKSCAN5 | frameshift_deleti  on | exon7 | c.2482delC | p.P828fs | . | . |
| CTC1 | 7 | 99662511 | GTAGT | G | 1 | NM_017715 | ZNF3 | frameshift_deleti  on | exon6 | c.292_295del | p.T98fs | . | . |

| CTC1 | 7 | 99711790 | GC | G | 0.258 | NM_001190415 | TAF6 | frameshift_deleti  on | exon2 | c.153delG | p.L51fs | . | . |
| --- | --- | --- | --- | --- | --- | --- | --- | --- | --- | --- | --- | --- | --- |
| CTC1 | 7 | 99802340 | GC | G | 0.931 | NM_001282718 | STAG3 | frameshift_deleti  on | exon25 | c.2720delC | p.A907fs | . | . |
| CTC1 | 7 | 100029158 | CG | C | 0.769 | NM_019606 | MEPCE | frameshift_deleti  on | exon1 | c.1518delG | p.P506fs | . | . |
| CTC1 | 7 | 100276029 | CA | C | 0.857 | NM_005273 | GNB2 | frameshift_deleti  on | exon9 | c.709delA | p.N237fs | . | . |
| CTC1 | 7 | 100679374 | CG | C | 0.214 | NM_001040105 | MUC17 | frameshift_deleti  on | exon3 | c.4678delG | p.G1560fs | . | . |
| CTC1 | 7 | 100731820 | GC | G | 0.895 | NM_030961 | TRIM56 | frameshift_deleti  on | exon3 | c.1228delC | p.P410fs | . | . |
| CTC1 | 7 | 102574999 | TGAA | T | 0.421 | NM_001031692 | LRRC17 | nonframeshift_de  letion | exon2 | c.640_642del | p.214_214del | . | . |
| CTC1 | 7 | 102669162 | AT | A | 1 | NM_001111038 | FBXL13 | frameshift_deleti  on | exon4 | c.101delA | p.N34fs | . | . |
| CTC1 | 7 | 105254801 | AGAG | A | 0.193 | NM_138495 | ATXN7L1 | nonframeshift_de  letion | exon8 | c.1605_1607del | p.535_536del | . | . |
| CTC1 | 7 | 105733404 | A | AT | 0.231 | NM_182715 | SYPL1 | frameshift_inserti  on | exon4 | c.581dupA | p.N194fs | . | . |
| CTC1 | 7 | 107618579 | TG | T | 0.278 | NM_002291 | LAMB1 | frameshift_deleti  on | exon9 | c.912delC | p.T304fs | . | . |
| CTC1 | 7 | 107834711 | A | AT | 0.203 | NM_001037132 | NRCAM | frameshift_inserti  on | exon13 | c.1624dupA | p.I542fs | . | . |
| CTC1 | 7 | 108113040 | AGG | A | 0.533 | NM_001256008 | PNPLA8 | frameshift_deleti  on | exon10 | c.2152_2153del | p.P718fs | . | . |
| CTC1 | 7 | 121651176 | CT | C | 0.234 | NM_001206838 | PTPRZ1 | frameshift_deleti  on | exon12 | c.2077delT | p.F693fs | . | . |
| CTC1 | 7 | 121651563 | AT | A | 0.429 | NM_002851 | PTPRZ1 | frameshift_deleti  on | exon12 | c.2464delT | p.F822fs | . | . |
| CTC1 | 7 | 121943788 | TG | T | 0.375 | NM_001024613 | FEZF1 | frameshift_deleti  on | exon1 | c.703delC | p.Q235fs | . | . |
| CTC1 | 7 | 121965536 | C | CA | 0.5 | NM_001009571 | CADPS2 | frameshift_inserti  on | exon27 | c.3588dupT | p.V1197fs | . | . |
| CTC1 | 7 | 126746733 | C | CG | 0.5 | NM_000845 | GRM8 | frameshift_inserti  on | exon2 | c.543_544insC | p.E182fs | . | . |
| CTC1 | 7 | 127894791 | AG | A | 0.538 | NM_000230 | LEP | frameshift_deleti  on | exon3 | c.480delG | p.Q160fs | . | . |
| CTC1 | 7 | 128586568 | TG | T | 0.55 | NM_001098627 | IRF5 | frameshift_deleti  on | exon4 | c.400delG | p.E134fs | . | . |
| CTC1 | 7 | 128587351 | CACTCT  GCAGCC GCCCAC TCTGCG | C | 1 | NM_001098627 | IRF5 | nonframeshift_de letion | exon6 | c.502_531del | p.168_177del | . | . |
| CTC1 | 7 | 130357651 | C | CTT | 0.28 | NM_052933 | TSGA13 | frameshift_inserti  on | exon6 | c.452_453insAA | p.K151fs | . | . |
| CTC1 | 7 | 133692543 | TG | T | 0.75 | NM_021807 | EXOC4 | frameshift_deleti  on | exon17 | c.2643delG | p.L881fs | . | . |
| CTC1 | 7 | 137612913 | CTGG | C | 1 | NM_001253775 | CREB3L2 | nonframeshift_de  letion | exon2 | c.299_301del | p.100_101del | . | . |
| CTC1 | 7 | 138603678 | AG | A | 0.455 | NM_001164665 | KIAA1549 | frameshift_deleti  on | exon2 | c.693delC | p.T231fs | . | . |
| CTC1 | 7 | 138732498 | TA | T | 0.833 | NM_020119 | ZC3HAV1 | frameshift_deleti  on | exon13 | c.2550delT | p.F850fs | . | . |
| CTC1 | 7 | 139311408 | CG | C | 0.48 | NM_001113239 | HIPK2 | frameshift_deleti  on | exon6 | c.1557delC | p.I519fs | . | . |
| CTC1 | 7 | 139611096 | C | CT | 0.733 | NM_001061 | TBXAS1 | frameshift_inserti  on | exon4 | c.313dupT | p.N104fs | . | . |
| CTC1 | 7 | 140273655 | TA | T | 0.842 | NM_015689 | DENND2A | frameshift_deleti  on | exon4 | c.1398delT | p.F466fs | . | . |
| CTC1 | 7 | 141422994 | CA | C | 0.455 | NM_001105558 | WEE2 | frameshift_deleti  on | exon6 | c.942delA | p.P314fs | . | . |
| CTC1 | 7 | 142460835 | GA | G | 0.242 | NM_002769 | PRSS1 | frameshift_deleti  on | exon5 | c.709delA | p.K237fs | . | . |
| CTC1 | 7 | 142991794 | CCA | C | 0.261 | NM_001224 | CASP2 | frameshift_deleti  on | exon6 | c.583_584del | p.H195fs | . | . |
| CTC1 | 7 | 143096871 | CCG | C | 0.25 | NM_005232 | EPHA1 | frameshift_deleti  on | exon4 | c.706_707del | p.R236fs | . | . |
| CTC1 | 7 | 143955885 | GAGGCT  GTGAAA | G | 0.3 | NM_001005328 | OR2A7 | nonframeshift_de  letion | exon1 | c.822_836del | p.274_279del | . | . |
| CTC1 | 7 | 149506211 | G | GC | 1 | NM_198455 | SSPO | frameshift_inserti  on | exon63 | c.9204dupC | p.S3068fs | . | . |
| CTC1 | 7 | 150706330 | GA | G | 0.286 | NM_000603 | NOS3 | frameshift_deleti  on | exon19 | c.2300delA | p.E767fs | . | . |
| CTC1 | 7 | 150912780 | C | CA | 0.197 | NM_005692 | ABCF2 | frameshift_inserti  on | exon13 | c.1439dupT | p.L480fs | . | . |
| CTC1 | 7 | 151052855 | GA | G | 0.3 | NM_001243351 | NUB1 | frameshift_deleti  on | exon6 | c.490delA | p.K164fs | . | . |
| CTC1 | 7 | 151668145 | TA | T | 0.545 | NM_145292 | GALNTL5 | frameshift_deleti  on | exon3 | c.364delA | p.K122fs | . | . |
| CTC1 | 7 | 154172077 | AG | A | 0.208 | NM_001039350 | DPP6 | frameshift_deleti  on | exon3 | c.221delG | p.S74fs | . | . |
| CTC1 | 7 | 154752604 | TA | T | 0.484 | NM_007349 | PAXIP1 | stopgain | exon12 | c.2432delT | p.L811X | . | . |
| CTC1 | 7 | 157364175 | TG | T | 0.2 | NM_130842 | PTPRN2 | frameshift_deleti on | exon19 | c.2742delC | p.N914fs | . | . |
| CTC1 | 7 | 158672394 | AG | A | 0.289 | NM_018051 | WDR60 | frameshift_deleti  on | exon5 | c.594delG | p.K198fs | . | . |
| CTC1 | 8 | 2820137 | A | AT | 0.412 | NM_033225 | CSMD1 | frameshift_inserti  on | exon61 | c.9478dupA | p.I3160fs | . | . |
| CTC1 | 8 | 8185275 | T | TA | 0.214 | NM_001080826 | SGK223 | frameshift_inserti  on | exon4 | c.3016dupT | p.Y1006fs | . | . |
| CTC1 | 8 | 8234868 | C | CGCCG  CT | 1 | NM_001080826 | SGK223 | nonframeshift_in  sertion | exon2 | c.1050_1051ins  AGCGGC | p.A351delinsS  GA | . | . |
| CTC1 | 8 | 10468057 | AG | A | 0.175 | NM_178857 | RP1L1 | frameshift_deleti  on | exon4 | c.3550delC | p.L1184fs | . | . |
| CTC1 | 8 | 11666218 | GTCCCA  C | G | 1 | NM_001287750 | FDFT1 | nonframeshift_de  letion | exon1 | c.193_198del | p.65_66del | . | . |
| CTC1 | 8 | 17092231 | A | AT | 0.34 | NM_013354 | CNOT7 | frameshift_inserti  on | exon5 | c.611dupA | p.N204fs | . | . |
| CTC1 | 8 | 17815107 | T | TGAG | 0.25 | NM_006197 | PCM1 | nonframeshift_in  sertion | exon13 | c.1863_1864ins  GAG | p.D621delinsD  E | . | . |
| CTC1 | 8 | 17847416 | CA | C | 0.306 | NM_006197 | PCM1 | frameshift_deleti  on | exon27 | c.4460delA | p.Q1487fs | . | . |
| CTC1 | 8 | 21956544 | CA | C | 0.197 | NM_022749 | FAM160B2 | frameshift_deleti  on | exon8 | c.1052delA | p.H351fs | . | . |
| CTC1 | 8 | 21985339 | AG | A | 0.444 | NM_005144 | HR | frameshift_deleti  on | exon3 | c.615delC | p.G205fs | . | . |
| CTC1 | 8 | 22051639 | CGCA | C | 0.724 | NM_001199 | BMP1 | nonframeshift_de  letion | exon10 | c.1250_1252del | p.417_418del | . | . |
| CTC1 | 8 | 22459362 | AG | A | 0.591 | NM_001013842 | C8orf58 | frameshift_deleti  on | exon3 | c.655delG | p.G219fs | . | . |
| CTC1 | 8 | 22995423 | TA | T | 0.532 | NM_003840 | TNFRSF10D | frameshift_deleti  on | exon9 | c.1120delT | p.Y374fs | . | . |

| CTC1 | 8 | 23049371 | A | AT | 0.534 | NM_003844 | TNFRSF10A | frameshift_inserti  on | exon10 | c.1242dupA | p.W415fs | . | . |
| --- | --- | --- | --- | --- | --- | --- | --- | --- | --- | --- | --- | --- | --- |
| CTC1 | 8 | 23305349 | TG | T | 0.484 | NM_001128930 | ENTPD4 | frameshift_deleti  on | exon4 | c.255delC | p.P85fs | . | . |
| CTC1 | 8 | 25337575 | A | AG | 0.167 | NM_152562 | CDCA2 | frameshift_inserti  on | exon8 | c.967_968insG | p.K323fs | . | . |
| CTC1 | 8 | 27255177 | ATGG | A | 0.154 | NM_173175 | PTK2B | nonframeshift_de  letion | exon2 | c.77_79del | p.26_27del | . | . |
| CTC1 | 8 | 27898621 | CT | C | 0.36 | NM_001010906 | NUGGC | frameshift_deleti  on | exon13 | c.1557delA | p.E519fs | . | . |
| CTC1 | 8 | 28209225 | GGCA | G | 0.203 | NM_018660 | ZNF395 | nonframeshift_de  letion | exon7 | c.1017_1019del | p.339_340del | . | . |
| CTC1 | 8 | 29194910 | CG | C | 0.22 | NM_001394 | DUSP4 | frameshift_deleti  on | exon4 | c.817delC | p.R273fs | . | . |
| CTC1 | 8 | 30699821 | TG | T | 0.364 | NM_031271 | TEX15 | frameshift_deleti  on | exon1 | c.6712delC | p.H2238fs | . | . |
| CTC1 | 8 | 30700602 | TG | T | 0.385 | NM_031271 | TEX15 | frameshift_deleti  on | exon1 | c.5931delC | p.N1977fs | . | . |
| CTC1 | 8 | 30982448 | CA | C | 0.308 | NM_000553 | WRN | frameshift_deleti  on | exon23 | c.2758delA | p.K920fs | . | . |
| CTC1 | 8 | 35624516 | TG | T | 0.377 | NM_080872 | UNC5D | frameshift_deleti  on | exon15 | c.2411delG | p.C804fs | . | . |
| CTC1 | 8 | 37964645 | CA | C | 0.8 | NM_001105214 | ASH2L | frameshift_deleti  on | exon3 | c.81delA | p.T27fs | . | . |
| CTC1 | 8 | 41399570 | AC | A | 0.175 | NM_032336 | GINS4 | frameshift_deleti  on | exon8 | c.637delC | p.P213fs | . | . |
| CTC1 | 8 | 42045454 | CG | C | 0.548 | NM_033011 | PLAT | frameshift_deleti  on | exon4 | c.195delC | p.P65fs | . | . |
| CTC1 | 8 | 42286357 | GC | G | 0.333 | NM_001257180 | SLC20A2 | frameshift_deleti  on | exon10 | c.1712delG | p.G571fs | . | . |
| CTC1 | 8 | 42939996 | AG | A | 0.417 | NM_002027 | FNTA | frameshift_deleti  on | exon8 | c.990delG | p.K330fs | . | . |
| CTC1 | 8 | 53555319 | TA | T | 0.263 | NM_001083617 | RB1CC1 | frameshift_deleti  on | exon17 | c.4040delT | p.I1347fs | . | . |
| CTC1 | 8 | 54142003 | GA | G | 0.296 | NM_000912 | OPRK1 | frameshift_deleti  on | exon4 | c.996delT | p.F332fs | . | . |
| CTC1 | 8 | 54163481 | GT | G | 0.183 | NM_000912 | OPRK1 | frameshift_deleti  on | exon2 | c.116delA | p.N39fs | . | . |
| CTC1 | 8 | 55540115 | GA | G | 0.875 | NM_006269 | RP1 | frameshift_deleti  on | exon4 | c.3674delA | p.E1225fs | . | . |
| CTC1 | 8 | 57353999 | GC | G | 0.392 | NM_006211 | PENK | frameshift_deleti  on | exon2 | c.635delG | p.G212fs | . | . |
| CTC1 | 8 | 59739445 | GT | G | 0.538 | NM_014729 | TOX | frameshift_deleti  on | exon6 | c.940delA | p.T314fs | . | . |
| CTC1 | 8 | 66635817 | AG | A | 0.238 | NM_001242318 | PDE7A | frameshift_deleti  on | exon12 | c.1201delC | p.L401fs | . | . |
| CTC1 | 8 | 67831346 | TG | T | 0.327 | NM_173518 | MCMDC2 | frameshift_deleti  on | exon15 | c.1989delG | p.L663fs | . | . |
| CTC1 | 8 | 70744651 | CG | C | 0.31 | NM_001146008 | SLCO5A1 | frameshift_deleti  on | exon1 | c.257delC | p.P86fs | . | . |
| CTC1 | 8 | 71556323 | AT | A | 0.625 | NM_016027 | LACTB2 | frameshift_deleti  on | exon4 | c.568delA | p.I190fs | . | . |
| CTC1 | 8 | 71593443 | TG | T | 0.458 | NM_001011720 | XKR9 | frameshift_deleti  on | exon3 | c.151delG | p.G51fs | . | . |
| CTC1 | 8 | 73480317 | AC | A | 0.5 | NM_004770 | KCNB2 | frameshift_deleti  on | exon2 | c.349delC | p.L117fs | . | . |
| CTC1 | 8 | 77776502 | C | CT | 0.29 | NM_024721 | ZFHX4 | frameshift_inserti  on | exon11 | c.10553dupT | p.L3518fs | . | . |
| CTC1 | 8 | 81411778 | CA | C | 0.194 | NM_001105539 | ZBTB10 | frameshift_deleti  on | exon2 | c.1023delA | p.P341fs | . | . |
| CTC1 | 8 | 86027421 | G | GA | 0.273 | NM_033402 | LRRCC1 | frameshift_inserti  on | exon5 | c.632dupA | p.E211fs | . | . |
| CTC1 | 8 | 86126827 | C | CAACA  TT | 1 | NM_001099673 | C8orf59 | nonframeshift_in  sertion | exon4 | c.264_265insAA  TGTT | p.D89delinsNV  D | . | . |
| CTC1 | 8 | 94805468 | CT | C | 0.5 | NM_153704 | TMEM67 | frameshift_deleti  on | exon16 | c.1619delT | p.L540fs | . | . |
| CTC1 | 8 | 98725970 | GC | G | 0.182 | NM_178812 | MTDH | frameshift_deleti  on | exon9 | c.1354delC | p.Q452fs | . | . |
| CTC1 | 8 | 99560295 | TG | T | 0.825 | NM_001256313 | STK3 | frameshift_deleti  on | exon7 | c.709delC | p.Q237fs | . | . |
| CTC1 | 8 | 99962892 | A | AT | 0.188 | NM_001142462 | OSR2 | frameshift_inserti  on | exon3 | c.666dupT | p.H222fs | . | . |
| CTC1 | 8 | 100654103 | GC | G | 0.179 | NM_017890 | VPS13B | frameshift_deleti  on | exon34 | c.5361delC | p.R1787fs | . | . |
| CTC1 | 8 | 100654683 | TG | T | 0.289 | NM_017890 | VPS13B | frameshift_deleti  on | exon34 | c.5941delG | p.D1981fs | . | . |
| CTC1 | 8 | 100796593 | T | TA | 0.385 | NM_017890 | VPS13B | frameshift_inserti  on | exon43 | c.7906dupA | p.C2635fs | . | . |
| CTC1 | 8 | 100975166 | TG | T | 0.204 | NM_001286693 | RGS22 | frameshift_deleti  on | exon23 | c.3112delC | p.Q1038fs | . | . |
| CTC1 | 8 | 100990153 | AT | A | 1 | NM_001286693 | RGS22 | frameshift_deleti  on | exon21 | c.2967delA | p.K989fs | . | . |
| CTC1 | 8 | 103307306 | CA | C | 0.303 | NM_001282873 | UBR5 | frameshift_deleti  on | exon32 | c.4091delT | p.L1364fs | . | . |
| CTC1 | 8 | 106814346 | A | AG | 0.571 | NM_012082 | ZFPM2 | frameshift_inserti  on | exon8 | c.2036_2037ins  G | p.D679fs | . | . |
| CTC1 | 8 | 109094849 | G | GA | 0.384 | NM_001282863 | RSPO2 | frameshift_inserti  on | exon2 | c.17dupT | p.F6fs | . | . |
| CTC1 | 8 | 109248421 | AT | A | 0.207 | NM_001568 | EIF3E | frameshift_deleti  on | exon4 | c.334delA | p.M112fs | . | . |
| CTC1 | 8 | 110492365 | GC | G | 0.875 | NM_177531 | PKHD1L1 | frameshift_deleti  on | exon55 | c.9325delC | p.Q3109fs | . | . |
| CTC1 | 8 | 117866539 | ACTC | A | 0.138 | NM_006265 | RAD21 | nonframeshift_de  letion | exon9 | c.1103_1105del | p.368_369del | . | . |
| CTC1 | 8 | 119938826 | GT | G | 0.532 | NM_002546 | TNFRSF11B | frameshift_deleti  on | exon4 | c.723delA | p.K241fs | . | . |
| CTC1 | 8 | 121210055 | GC | G | 0.333 | NM_021110 | COL14A1 | frameshift_deleti  on | exon7 | c.599delC | p.A200fs | . | . |
| CTC1 | 8 | 125072482 | AC | A | 0.353 | NM_001039112 | FER1L6 | frameshift_deleti  on | exon23 | c.2937delC | p.Y979fs | . | . |
| CTC1 | 8 | 125339627 | CA | C | 0.239 | NM_194291 | TMEM65 | frameshift_deleti  on | exon3 | c.384delT | p.F128fs | . | . |
| CTC1 | 8 | 133858154 | C | CT | 0.242 | NM_001277196 | PHF20L1 | frameshift_inserti  on | exon20 | c.2963dupT | p.L988fs | . | . |
| CTC1 | 8 | 142185339 | CT | C | 0.19 | NM_014957 | DENND3 | frameshift_deleti  on | exon14 | c.2077delT | p.Y693fs | . | . |
| CTC1 | 8 | 142228702 | CT | C | 0.5 | NM_001080431 | SLC45A4 | frameshift_deleti  on | exon4 | c.883delA | p.S295fs | . | . |
| CTC1 | 8 | 142231755 | GCC | G | 0.366 | NM_001080431 | SLC45A4 | frameshift_deleti  on | exon2 | c.196_197del | p.G66fs | . | . |
| CTC1 | 8 | 144650712 | GGCTGC  TGACCT CAGCAG | G | 0.75 | NM_001100878 | MROH6 | frameshift_deleti on | exon10 | c.1632_1644del | p.R544fs | . | . |
| CTC1 | 8 | 144996097 | TCC | T | 1 | NM_201378 | PLEC | frameshift_deleti  on | exon32 | c.7848_7849del | p.R2616fs | . | . |

| CTC1 | 8 | 145052080 | CG | C | 1 | NM_032789 | PARP10 | frameshift_deleti  on | exon10 | c.2725delC | p.R909fs | . | . |
| --- | --- | --- | --- | --- | --- | --- | --- | --- | --- | --- | --- | --- | --- |
| CTC1 | 8 | 145057430 | GC | G | 0.338 | NM_032789 | PARP10 | frameshift_deleti  on | exon8 | c.2326delG | p.A776fs | . | . |
| CTC1 | 8 | 145139050 | T | TC | 0.585 | NM_003801 | GPAA1 | frameshift_inserti  on | exon6 | c.631dupC | p.S210fs | . | . |
| CTC1 | 8 | 145617534 | TGGGGG  TGCAAG | T | 0.675 | NM_174922 | ADCK5 | frameshift_deleti  on | exon12 | c.1257_1267del | p.L419fs | . | . |
| CTC1 | 9 | 2039776 | ACAG | A | 0.348 | NM_001289396 | SMARCA2 | nonframeshift_de  letion | exon4 | c.667_669del | p.223_223del | . | . |
| CTC1 | 9 | 4572301 | GA | G | 0.471 | NM_004170 | SLC1A1 | frameshift_deleti  on | exon7 | c.681delA | p.G227fs | . | . |
| CTC1 | 9 | 4572312 | GA | G | 0.632 | NM_004170 | SLC1A1 | frameshift_deleti  on | exon7 | c.692delA | p.E231fs | . | . |
| CTC1 | 9 | 5456122 | AT | A | 0.438 | NM_001267706 | CD274 | frameshift_deleti  on | exon2 | c.10delT | p.F4fs | . | . |
| CTC1 | 9 | 7169966 | CA | C | 0.429 | NM_001146694 | KDM4C | frameshift_deleti  on | exon21 | c.3071delA | p.Q1024fs | . | . |
| CTC1 | 9 | 12775861 | T | TGGCG  GCGGC | 1 | NM_203403 | LURAP1L | nonframeshift_in  sertion | exon1 | c.147_148insG  GCGGCGGC | p.G49delinsGG  GG | . | . |
| CTC1 | 9 | 12775861 | T | TGGCG  GC | 1 | NM_203403 | LURAP1L | nonframeshift_in  sertion | exon1 | c.147_148insG  GCGGC | p.G49delinsGG  G | . | . |
| CTC1 | 9 | 14619643 | TC | T | 0.542 | NM_178566 | ZDHHC21 | frameshift_deleti  on | exon9 | c.658delG | p.E220fs | . | . |
| CTC1 | 9 | 14747031 | GA | G | 0.186 | NM_001177704 | FREM1 | frameshift_deleti  on | exon11 | c.1635delT | p.F545fs | . | . |
| CTC1 | 9 | 14747707 | TA | T | 1 | NM_001177704 | FREM1 | frameshift_deleti  on | exon9 | c.1423delT | p.Y475fs | . | . |
| CTC1 | 9 | 14770785 | GT | G | 0.277 | NM_001177704 | FREM1 | frameshift_deleti  on | exon3 | c.484delA | p.T162fs | . | . |
| CTC1 | 9 | 16436374 | C | CGG | 0.4 | NM_017637 | BNC2 | frameshift_inserti  on | exon6 | c.1817_1818ins  CC | p.P606fs | . | . |
| CTC1 | 9 | 18777751 | AGC | A | 0.167 | NM_001040272 | ADAMTSL1 | frameshift_deleti  on | exon19 | c.3525_3526del | p.Q1175fs | . | . |
| CTC1 | 9 | 19324412 | CA | C | 1 | NM_017925 | DENND4C | frameshift_deleti  on | exon13 | c.1861delA | p.K621fs | . | . |
| CTC1 | 9 | 19346530 | CT | C | 0.7 | NM_017925 | DENND4C | frameshift_deleti  on | exon22 | c.3617delT | p.L1206fs | . | . |
| CTC1 | 9 | 20414263 | GA | G | 0.556 | NM_001286691 | MLLT3 | frameshift_deleti  on | exon5 | c.571delT | p.S191fs | . | . |
| CTC1 | 9 | 21227899 | TG | T | 0.571 | NM_021268 | IFNA17 | frameshift_deleti  on | exon1 | c.273delC | p.F91fs | . | . |
| CTC1 | 9 | 22008767 | C | CT | 0.571 | NM_078487 | CDKN2B | frameshift_inserti  on | exon1 | c.185dupA | p.Q62fs | . | . |
| CTC1 | 9 | 26917098 | TA | T | 0.273 | NM_001031689 | PLAA | frameshift_deleti  on | exon10 | c.1482delT | p.F494fs | . | . |
| CTC1 | 9 | 27197438 | C | CA | 0.537 | NM_000459 | TEK | frameshift_inserti  on | exon12 | c.1751dupA | p.Q584fs | . | . |
| CTC1 | 9 | 27949125 | AG | A | 0.941 | NM_001258282 | LINGO2 | frameshift_deleti  on | exon7 | c.1544delC | p.P515fs | . | . |
| CTC1 | 9 | 32543433 | TA | T | 0.75 | NM_001195622 | TOPORS | frameshift_deleti  on | exon2 | c.894delT | p.F298fs | . | . |
| CTC1 | 9 | 32784598 | AC | A | 0.767 | NM_212558 | TMEM215 | frameshift_deleti  on | exon2 | c.418delC | p.P140fs | . | . |
| CTC1 | 9 | 32987559 | C | CT | 0.537 | NM_001195250 | APTX | frameshift_inserti  on | exon3 | c.345dupA | p.D116fs | . | . |
| CTC1 | 9 | 33115990 | TG | T | 0.257 | NM_001497 | B4GALT1 | frameshift_deleti  on | exon4 | c.957delC | p.N319fs | . | . |
| CTC1 | 9 | 33255883 | CT | C | 0.474 | NM_001172415 | BAG1 | frameshift_deleti  on | exon6 | c.582delA | p.K194fs | . | . |
| CTC1 | 9 | 33369957 | AT | A | 0.414 | NM_002504 | NFX1 | frameshift_deleti  on | exon24 | c.3345delT | p.Y1115fs | . | . |
| CTC1 | 9 | 33467732 | GC | G | 0.386 | NM_022917 | NOL6 | frameshift_deleti  on | exon12 | c.1558delG | p.A520fs | . | . |
| CTC1 | 9 | 34257138 | G | GT | 0.214 | NM_194313 | KIF24 | frameshift_inserti  on | exon11 | c.2466dupA | p.L823fs | . | . |
| CTC1 | 9 | 34339160 | CA | C | 0.406 | NM_001244390 | NUDT2 | frameshift_deleti  on | exon2 | c.124delA | p.K42fs | . | . |
| CTC1 | 9 | 34385671 | A | AG | 0.375 | NM_032596 | C9orf24 | frameshift_inserti  on | exon2 | c.243dupC | p.Y82fs | . | . |
| CTC1 | 9 | 34996419 | CT | C | 0.294 | NM_001135005 | DNAJB5 | frameshift_deleti  on | exon3 | c.586delT | p.F196fs | . | . |
| CTC1 | 9 | 35095544 | AC | A | 0.309 | NM_032634 | PIGO | frameshift_deleti  on | exon2 | c.18delG | p.V6fs | . | . |
| CTC1 | 9 | 35752712 | AC | A | 0.821 | NM_001080496 | RGP1 | frameshift_deleti  on | exon9 | c.1018delC | p.P340fs | . | . |
| CTC1 | 9 | 35811485 | ACCAGG  ACCAGA | A | 1 | NM_001039592 | SPAG8 | nonframeshift_de  letion | exon2 | c.546_557del | p.182_186del | . | . |
| CTC1 | 9 | 37763650 | GT | G | 0.696 | NM_001286951 | TRMT10B | frameshift_deleti  on | exon3 | c.36delT | p.R12fs | . | . |
| CTC1 | 9 | 71437573 | CA | C | 0.6 | NM_001278253 | PIP5K1B | frameshift_deleti  on | exon3 | c.44delA | p.Q15fs | . | . |
| CTC1 | 9 | 71998565 | A | AT | 0.581 | NM_001127608 | FAM189A2 | frameshift_inserti  on | exon7 | c.515dupT | p.I172fs | . | . |
| CTC1 | 9 | 79318375 | GGTGAC  AGCCTG | G | 1 | NM_015225 | PRUNE2 | nonframeshift_de  letion | exon9 | c.8139_8153del | p.2713_2718de  l | . | . |
| CTC1 | 9 | 79324135 | A | ATGAC  TGTTGC | 0.609 | NM_015225 | PRUNE2 | nonframeshift_in  sertion | exon8 | c.3054_3055ins  CTGCAACAGT | p.S1019delinsL  QQSS | . | . |
| CTC1 | 9 | 82333828 | AG | A | 0.312 | NM_001282753 | TLE4 | frameshift_deleti  on | exon13 | c.1326delG | p.K442fs | . | . |
| CTC1 | 9 | 84610094 | GA | G | 0.756 | NM_001001670 | SPATA31D1 | frameshift_deleti  on | exon4 | c.4710delA | p.G1570fs | . | . |
| CTC1 | 9 | 84610097 | AAT | A | 0.162 | NM_001001670 | SPATA31D1 | frameshift_deleti  on | exon4 | c.4713_4714del | p.K1571fs | . | . |
| CTC1 | 9 | 85863107 | GC | G | 0.186 | NM_001244962 | FRMD3 | frameshift_deleti  on | exon2 | c.490delG | p.A164fs | . | . |
| CTC1 | 9 | 86485506 | CG | C | 0.75 | NM_001271927 | KIF27 | frameshift_deleti  on | exon11 | c.2486delC | p.P829fs | . | . |
| CTC1 | 9 | 86498833 | C | CT | 0.264 | NM_001271927 | KIF27 | frameshift_inserti  on | exon10 | c.2339dupA | p.K780fs | . | . |
| CTC1 | 9 | 88937851 | ATTT | A | 0.136 | NM_001185074 | ZCCHC6 | nonframeshift_de  letion | exon9 | c.2442_2444del | p.814_815del | . | . |
| CTC1 | 9 | 88961383 | TG | T | 0.25 | NM_001185059 | ZCCHC6 | frameshift_deleti  on | exon3 | c.532delC | p.Q178fs | . | . |
| CTC1 | 9 | 90322156 | CT | C | 0.926 | NM_001288729 | DAPK1 | frameshift_deleti  on | exon26 | c.4171delT | p.F1391fs | . | . |
| CTC1 | 9 | 91262501 | GC | G | 0.241 | NM_001100111 | RP13-  60M5.2 | frameshift_deleti  on | exon2 | c.141delG | p.L47fs | . | . |
| CTC1 | 9 | 91994052 | TG | T | 0.372 | NM_006378 | SEMA4D | frameshift_deleti  on | exon18 | c.2155delC | p.Q719fs | . | . |
| CTC1 | 9 | 93637011 | AC | A | 0.5 | NM_001135052 | SYK | frameshift_deleti  on | exon8 | c.993delC | p.D331fs | . | . |
| CTC1 | 9 | 94519795 | GAC | G | 0.375 | NM_004560 | ROR2 | frameshift_deleti  on | exon3 | c.220_221del | p.V74fs | . | . |

| CTC1 | 9 | 95237024 | CTCA | C | 0.889 | NM_001193335 | ASPN | nonframeshift_de  letion | exon2 | c.153_155del | p.51_52del | . | . |
| --- | --- | --- | --- | --- | --- | --- | --- | --- | --- | --- | --- | --- | --- |
| CTC1 | 9 | 96439004 | C | CCCTG  CCTCC | 0.933 | NM_005392 | PHF2 | nonframeshift_in  sertion | exon21 | c.2961_2962ins  CCTGCCTCCA | p.T987delinsTP  ASTT | . | . |
| CTC1 | 9 | 99580599 | TC | T | 0.323 | NM_001001662 | ZNF782 | frameshift_deleti  on | exon6 | c.1705delG | p.E569fs | . | . |
| CTC1 | 9 | 99581089 | T | TC | 0.412 | NM_001001662 | ZNF782 | frameshift_inserti  on | exon6 | c.1215dupG | p.K406fs | . | . |
| CTC1 | 9 | 99581668 | GCC | G | 0.222 | NM_001001662 | ZNF782 | frameshift_deleti  on | exon6 | c.635_636del | p.G212fs | . | . |
| CTC1 | 9 | 100070396 | GC | G | 0.242 | NM_020893 | CCDC180 | frameshift_deleti  on | exon2 | c.184delC | p.Q62fs | . | . |
| CTC1 | 9 | 100092968 | A | AGAGG  AG | 1 | NM_020893 | CCDC180 | nonframeshift_in  sertion | exon18 | c.2325_2326ins  GAGGAG | p.E775delinsEE  E | . | . |
| CTC1 | 9 | 100092971 | G | GGAGG  AT | 0.875 | NM_020893 | CCDC180 | nonframeshift_in  sertion | exon18 | c.2328_2329ins  GAGGAT | p.E776delinsEE  D | . | . |
| CTC1 | 9 | 100919926 | TG | T | 0.571 | NM_003389 | CORO2A | frameshift_deleti  on | exon2 | c.16delC | p.Q6fs | . | . |
| CTC1 | 9 | 101785639 | GC | G | 0.395 | NM_001855 | COL15A1 | frameshift_deleti  on | exon14 | c.1763delC | p.A588fs | . | . |
| CTC1 | 9 | 107298999 | AC | A | 0.333 | NM_001001961 | OR13C3 | frameshift_deleti  on | exon1 | c.95delG | p.G32fs | . | . |
| CTC1 | 9 | 109691674 | CA | C | 1 | NM_021224 | ZNF462 | frameshift_deleti  on | exon3 | c.5482delA | p.N1828fs | . | . |
| CTC1 | 9 | 112211100 | CA | C | 0.573 | NM_001145369 | PTPN3 | frameshift_deleti  on | exon1 | c.10delT | p.C4fs | . | . |
| CTC1 | 9 | 112900341 | G | GGAAG  CT | 1 | NM_001004065 | AKAP2 | nonframeshift_in  sertion | exon2 | c.2091_2092ins  GAAGCT | p.E697delinsEE  A | . | . |
| CTC1 | 9 | 114304057 | C | CA | 0.423 | NM_133464 | ZNF483 | frameshift_inserti  on | exon6 | c.843dupA | p.S281fs | . | . |
| CTC1 | 9 | 114500667 | AT | A | 0.323 | NM_001080551 | C9orf84 | stopgain | exon8 | c.1000delA | p.M334X | . | . |
| CTC1 | 9 | 115221843 | TAA | T | 0.283 | NM_001195822 | HSDL2 | frameshift_deleti on | exon8 | c.912_913del | p.V304fs | . | . |
| CTC1 | 9 | 115964835 | CT | C | 0.259 | NM_015258 | FKBP15 | frameshift_deleti  on | exon6 | c.473delA | p.K158fs | . | . |
| CTC1 | 9 | 116022560 | TG | T | 0.315 | NM_001859 | SLC31A1 | frameshift_deleti  on | exon5 | c.381delG | p.M127fs | . | . |
| CTC1 | 9 | 116053077 | TC | T | 0.333 | NM_001244926 | PRPF4 | frameshift_deleti  on | exon12 | c.1244delC | p.S415fs | . | . |
| CTC1 | 9 | 116136060 | TC | T | 0.429 | NM_031219 | HDHD3 | frameshift_deleti  on | exon2 | c.574delG | p.D192fs | . | . |
| CTC1 | 9 | 116152940 | GT | G | 0.228 | NM_000031 | ALAD | frameshift_deleti  on | exon6 | c.413delA | p.N138fs | . | . |
| CTC1 | 9 | 117804607 | TG | T | 0.277 | NM_002160 | TNC | frameshift_deleti  on | exon18 | c.5138delC | p.P1713fs | . | . |
| CTC1 | 9 | 123586782 | TA | T | 0.357 | NM_001270427 | PSMD5 | frameshift_deleti  on | exon6 | c.866delT | p.L289fs | . | . |
| CTC1 | 9 | 123870152 | CA | C | 0.3 | NM_007018 | CNTRL | frameshift_deleti  on | exon6 | c.882delA | p.T294fs | . | . |
| CTC1 | 9 | 125148875 | AC | A | 1 | NM_001271164 | PTGS1 | frameshift_deleti  on | exon8 | c.1017delC | p.H339fs | . | . |
| CTC1 | 9 | 130495753 | TG | T | 0.795 | NM_001252018 | TOR2A | frameshift_deleti  on | exon2 | c.17delC | p.P6fs | . | . |
| CTC1 | 9 | 130533494 | GC | G | 0.633 | NM_005489 | SH2D3C | frameshift_deleti  on | exon1 | c.14delG | p.G5fs | . | . |
| CTC1 | 9 | 131133691 | TC | T | 0.636 | NM_001135947 | URM1 | frameshift_deleti  on | exon1 | c.33delC | p.F11fs | . | . |
| CTC1 | 9 | 131346144 | GT | G | 0.316 | NM_001130438 | SPTAN1 | frameshift_deleti  on | exon16 | c.2090delT | p.V697fs | . | . |
| CTC1 | 9 | 131397500 | G | GA | 0.405 | NM_052844 | WDR34 | frameshift_inserti  on | exon6 | c.851dupT | p.F284fs | . | . |
| CTC1 | 9 | 131599981 | TG | T | 0.354 | NM_001122672 | CCBL1 | frameshift_deleti  on | exon5 | c.399delC | p.P133fs | . | . |
| CTC1 | 9 | 131718707 | GT | G | 0.375 | NM_015354 | NUP188 | frameshift_deleti  on | exon4 | c.241delT | p.F81fs | . | . |
| CTC1 | 9 | 133577579 | AC | A | 0.163 | NM_001282708 | EXOSC2 | frameshift_deleti  on | exon6 | c.477delC | p.H159fs | . | . |
| CTC1 | 9 | 133759489 | CAAG | C | 0.143 | NM_005157 | ABL1 | nonframeshift_de  letion | exon11 | c.1813_1815del | p.605_605del | . | . |
| CTC1 | 9 | 134021543 | TG | T | 1 | NM_005085 | NUP214 | frameshift_deleti  on | exon13 | c.1798delG | p.V600fs | . | . |
| CTC1 | 9 | 134073510 | TG | T | 0.958 | NM_005085 | NUP214 | frameshift_deleti  on | exon29 | c.4630delG | p.A1544fs | . | . |
| CTC1 | 9 | 134319620 | C | CT | 0.444 | NM_013318 | PRRC2B | frameshift_inserti  on | exon5 | c.518_519insT | p.P173fs | . | . |
| CTC1 | 9 | 134385441 | TC | T | 0.208 | NM_007171 | POMT1 | frameshift_deleti  on | exon8 | c.758delC | p.S253fs | . | . |
| CTC1 | 9 | 134955094 | CT | C | 0.387 | NM_001253881 | MED27 | frameshift_deleti  on | exon1 | c.137delA | p.E46fs | . | . |
| CTC1 | 9 | 135285753 | C | CT | 0.312 | NM_001282957 | C9orf171 | frameshift_inserti  on | exon1 | c.95_96insT | p.P32fs | . | . |
| CTC1 | 9 | 135537711 | C | CT | 0.225 | NM_022779 | DDX31 | frameshift_inserti  on | exon3 | c.659dupA | p.K220fs | . | . |
| CTC1 | 9 | 136568048 | TG | T | 0.274 | NM_001134707 | SARDH | frameshift_deleti  on | exon13 | c.1657delC | p.H553fs | . | . |
| CTC1 | 9 | 136918482 | GC | G | 0.447 | NM_007371 | BRD3 | frameshift_deleti  on | exon2 | c.117delG | p.L39fs | . | . |
| CTC1 | 9 | 138662897 | AG | A | 1 | NM_001272003 | KCNT1 | frameshift_deleti  on | exon17 | c.1830delG | p.E610fs | . | . |
| CTC1 | 9 | 139258784 | GC | G | 1 | NM_052813 | CARD9 | frameshift_deleti  on | exon13 | c.1580delG | p.G527fs | . | . |
| CTC1 | 9 | 139277994 | CGCTGC  TGCT | C | 1 | NM_003086 | SNAPC4 | nonframeshift_de  letion | exon15 | c.1618_1626del | p.540_542del | . | . |
| CTC1 | 9 | 139290167 | GT | G | 1 | NM_003086 | SNAPC4 | frameshift_deleti  on | exon3 | c.232delA | p.T78fs | . | . |
| CTC1 | 9 | 139366476 | CG | C | 0.254 | NM_001276418 | SEC16A | frameshift_deleti  on | exon3 | c.3654delC | p.P1218fs | . | . |
| CTC1 | 9 | 139400201 | A | AT | 0.182 | NM_017617 | NOTCH1 | frameshift_inserti  on | exon25 | c.4146dupA | p.C1383fs | . | . |
| CTC1 | 9 | 140953124 | CG | C | 0.18 | NM_000718 | CACNA1B | frameshift_deleti  on | exon29 | c.4413delG | p.P1471fs | . | . |
| CTC1 | 9 | 141107536 | GGCAGC  AGCAGC | G | 1 | NM_001145249 | FAM157B | nonframeshift_de  letion | exon2 | c.219_233del | p.73_78del | . | . |
| CTC1 | 9 | 141107666 | GA | G | 1 | NM_001145249 | FAM157B | frameshift_deleti  on | exon2 | c.349delA | p.K117fs | . | . |
| CTC1 | 10 | 3190392 | CG | C | 0.492 | NM_001242307 | PITRM1 | frameshift_deleti  on | exon17 | c.1962delC | p.P654fs | . | . |
| CTC1 | 10 | 5789966 | CCTGCC  TCAG | C | 1 | NM_017782 | FAM208B | nonframeshift_de  letion | exon15 | c.4583_4591del | p.1528_1531de  l | . | . |
| CTC1 | 10 | 6061457 | GA | G | 0.417 | NM_000417 | IL2RA | frameshift_deleti  on | exon6 | c.660delT | p.F220fs | . | . |

| CTC1 | 10 | 8007049 | AAAAAG  GAACTA AAGACT | A | 0.111 | NM_031923 | TAF3 | frameshift_deleti on | exon3 | c.1577_1599del | p.K526fs | . | . |
| --- | --- | --- | --- | --- | --- | --- | --- | --- | --- | --- | --- | --- | --- |
| CTC1 | 10 | 12191617 | TC | T | 0.524 | NM_001142628 | SEC61A2 | frameshift_deleti  on | exon4 | c.179delC | p.S60fs | . | . |
| CTC1 | 10 | 13361172 | AT | A | 0.471 | NM_001195602 | SEPHS1 | frameshift_deleti  on | exon8 | c.947delA | p.N316fs | . | . |
| CTC1 | 10 | 15255569 | A | AT | 0.385 | NM_001010924 | FAM171A1 | frameshift_inserti  on | exon8 | c.2017_2018ins  A | p.L673fs | . | . |
| CTC1 | 10 | 18690961 | G | GA | 0.456 | NM_000724 | CACNB2 | frameshift_inserti  on | exon2 | c.158dupA | p.E53fs | . | . |
| CTC1 | 10 | 18807854 | C | CA | 0.429 | NM_000724 | CACNB2 | frameshift_inserti  on | exon8 | c.737dupA | p.Q246fs | . | . |
| CTC1 | 10 | 23622075 | GA | G | 0.481 | NM_153714 | C10orf67 | frameshift_deleti  on | exon2 | c.245delT | p.F82fs | . | . |
| CTC1 | 10 | 26463386 | CA | C | 0.562 | NM_017433 | MYO3A | frameshift_deleti  on | exon30 | c.4194delA | p.T1398fs | . | . |
| CTC1 | 10 | 27406614 | GT | G | 0.381 | NM_001253866 | YME1L1 | frameshift_deleti  on | exon14 | c.1510delA | p.T504fs | . | . |
| CTC1 | 10 | 27687534 | C | CT | 0.897 | NM_001034842 | PTCHD3 | frameshift_inserti  on | exon4 | c.1992dupA | p.V665fs | . | . |
| CTC1 | 10 | 27692214 | GCT | G | 0.4 | NM_001034842 | PTCHD3 | frameshift_deleti  on | exon3 | c.1282_1283del | p.S428fs | . | . |
| CTC1 | 10 | 30316500 | ACTG | A | 0.121 | NM_020848 | KIAA1462 | nonframeshift_de  letion | exon3 | c.2574_2576del | p.858_859del | . | . |
| CTC1 | 10 | 30918523 | GA | G | 0.276 | NM_183058 | LYZL2 | frameshift_deleti  on | exon1 | c.111delT | p.F37fs | . | . |
| CTC1 | 10 | 32132412 | TG | T | 0.571 | NM_001270699 | ARHGAP12 | frameshift_deleti  on | exon4 | c.1131delC | p.T377fs | . | . |
| CTC1 | 10 | 32197334 | CA | C | 0.25 | NM_001270699 | ARHGAP12 | frameshift_deleti  on | exon1 | c.449delT | p.L150fs | . | . |
| CTC1 | 10 | 34671720 | TA | T | 0.615 | NM_001184790 | PARD3 | frameshift_deleti  on | exon8 | c.1014delT | p.F338fs | . | . |
| CTC1 | 10 | 38241124 | G | GA | 0.5 | NM_145011 | ZNF25 | frameshift_inserti  on | exon6 | c.1301_1302ins  T | p.T434fs | . | . |
| CTC1 | 10 | 38407078 | G | GA | 0.308 | NM_001178101 | ZNF37A | frameshift_inserti  on | exon7 | c.1000dupA | p.G333fs | . | . |
| CTC1 | 10 | 43882571 | G | GC | 0.421 | NM_001098208 | HNRNPF | frameshift_inserti  on | exon3 | c.761dupG | p.G254fs | . | . |
| CTC1 | 10 | 46121920 | C | CA | 0.889 | NM_001128324 | ZFAND4 | frameshift_inserti  on | exon7 | c.1350dupT | p.G451fs | . | . |
| CTC1 | 10 | 46969390 | GC | G | 0.5 | NM_031912 | SYT15 | frameshift_deleti  on | exon2 | c.70delG | p.A24fs | . | . |
| CTC1 | 10 | 46969400 | ACAG | A | 0.158 | NM_031912 | SYT15 | nonframeshift_de  letion | exon2 | c.58_60del | p.20_20del | . | . |
| CTC1 | 10 | 49446055 | AC | A | 0.31 | NM_001018071 | FRMPD2 | frameshift_deleti  on | exon8 | c.899delG | p.G300fs | . | . |
| CTC1 | 10 | 50018590 | GT | G | 0.992 | NM_020945 | WDFY4 | frameshift_deleti  on | exon29 | c.4855delT | p.F1619fs | . | . |
| CTC1 | 10 | 50172062 | TG | T | 0.542 | NM_020945 | WDFY4 | frameshift_deleti  on | exon54 | c.8400delG | p.L2800fs | . | . |
| CTC1 | 10 | 50178206 | TG | T | 0.638 | NM_020945 | WDFY4 | frameshift_deleti  on | exon57 | c.8801delG | p.W2934fs | . | . |
| CTC1 | 10 | 50178274 | GGT | G | 0.6 | NM_020945 | WDFY4 | frameshift_deleti  on | exon57 | c.8869_8870del | p.V2957fs | . | . |
| CTC1 | 10 | 50340100 | CG | C | 0.326 | NM_001164484 | FAM170B | frameshift_deleti  on | exon2 | c.409delC | p.R137fs | . | . |
| CTC1 | 10 | 50725140 | TA | T | 0.6 | NM_170753 | PGBD3 | frameshift_deleti  on | exon2 | c.20delT | p.L7fs | . | . |
| CTC1 | 10 | 53458650 | CCCAGG  G | C | 0.333 | NM_015235 | CSTF2T | nonframeshift_de  letion | exon1 | c.654_659del | p.218_220del | . | . |
| CTC1 | 10 | 60461854 | GC | G | 0.4 | NM_001080512 | BICC1 | frameshift_deleti  on | exon3 | c.259delC | p.Q87fs | . | . |
| CTC1 | 10 | 62631304 | GC | G | 0.429 | NM_014836 | RHOBTB1 | frameshift_deleti  on | exon11 | c.2026delG | p.A676fs | . | . |
| CTC1 | 10 | 63852488 | CG | C | 0.21 | NM_001244638 | ARID5B | frameshift_deleti  on | exon7 | c.2538delG | p.S846fs | . | . |
| CTC1 | 10 | 64573470 | A | AGGCG  GC | 0.808 | NM_000399 | EGR2 | nonframeshift_in  sertion | exon2 | c.927_928insG  CCGCC | p.Y310delinsAA  Y | . | . |
| CTC1 | 10 | 64573470 | A | AGGC | 0.808 | NM_000399 | EGR2 | nonframeshift_in  sertion | exon2 | c.927_928insG  CC | p.Y310delinsAY | . | . |
| CTC1 | 10 | 64573486 | G | GGCT | 0.625 | NM_000399 | EGR2 | nonframeshift_in  sertion | exon2 | c.911_912insA  GC | p.A304delinsA  A | . | . |
| CTC1 | 10 | 64967950 | ACTAAA  C | A | 1 | NM_001282948 | JMJD1C | nonframeshift_de  letion | exon9 | c.2927_2932del | p.976_978del | . | . |
| CTC1 | 10 | 68688092 | CT | C | 0.234 | NM_178011 | LRRTM3 | frameshift_deleti  on | exon2 | c.1419delT | p.T473fs | . | . |
| CTC1 | 10 | 70097713 | GA | G | 0.503 | NM_012207 | HNRNPH3 | frameshift_deleti  on | exon3 | c.212delA | p.E71fs | . | . |
| CTC1 | 10 | 70405053 | CA | C | 0.426 | NM_030625 | TET1 | frameshift_deleti  on | exon4 | c.2568delA | p.P856fs | . | . |
| CTC1 | 10 | 70405476 | CA | C | 0.357 | NM_030625 | TET1 | frameshift_deleti  on | exon4 | c.2991delA | p.S997fs | . | . |
| CTC1 | 10 | 70406137 | CA | C | 0.412 | NM_030625 | TET1 | frameshift_deleti  on | exon4 | c.3652delA | p.K1218fs | . | . |
| CTC1 | 10 | 70723100 | TA | T | 0.545 | NM_001256910 | DDX21 | frameshift_deleti  on | exon4 | c.458delA | p.Y153fs | . | . |
| CTC1 | 10 | 70730059 | AC | A | 0.207 | NM_001256910 | DDX21 | frameshift_deleti  on | exon8 | c.1136delC | p.T379fs | . | . |
| CTC1 | 10 | 71005928 | GA | G | 0.209 | NM_025130 | HKDC1 | frameshift_deleti  on | exon8 | c.970delA | p.K324fs | . | . |
| CTC1 | 10 | 72498740 | AC | A | 0.809 | NM_080722 | ADAMTS14 | frameshift_deleti  on | exon11 | c.1743delC | p.N581fs | . | . |
| CTC1 | 10 | 73050752 | GT | G | 0.19 | NM_001244889 | UNC5B | frameshift_deleti  on | exon8 | c.1148delT | p.V383fs | . | . |
| CTC1 | 10 | 74594138 | TG | T | 0.714 | NM_001270679 | MCU | frameshift_deleti  on | exon2 | c.173delG | p.W58fs | . | . |
| CTC1 | 10 | 74684379 | GA | G | 0.279 | NM_152635 | OIT3 | frameshift_deleti  on | exon7 | c.1345delA | p.K449fs | . | . |
| CTC1 | 10 | 75413290 | TC | T | 0.429 | NM_001114133 | SYNPO2L | frameshift_deleti  on | exon3 | c.378delG | p.G126fs | . | . |
| CTC1 | 10 | 75528784 | CT | C | 0.2 | NM_198597 | SEC24C | frameshift_deleti  on | exon17 | c.2299delT | p.F767fs | . | . |
| CTC1 | 10 | 76781905 | GGAA | G | 0.882 | NM_001256468 | KAT6B | nonframeshift_de  letion | exon16 | c.2740_2742del | p.914_914del | . | . |
| CTC1 | 10 | 76781905 | GGAAGA  A | G | 0.882 | NM_001256468 | KAT6B | nonframeshift_de  letion | exon16 | c.2740_2745del | p.914_915del | . | . |
| CTC1 | 10 | 79396253 | CG | C | 0.262 | NM_001271522 | KCNMA1 | frameshift_deleti  on | exon2 | c.398delC | p.P133fs | . | . |
| CTC1 | 10 | 81057388 | CA | C | 0.548 | NM_020338 | ZMIZ1 | frameshift_deleti  on | exon14 | c.1433delA | p.Q478fs | . | . |
| CTC1 | 10 | 88415929 | TG | T | 0.4 | NM_001030015 | OPN4 | frameshift_deleti  on | exon2 | c.163delG | p.A55fs | . | . |

| CTC1 | 10 | 88911877 | CA | C | 0.824 | NM_019054 | FAM35A | frameshift_deleti  on | exon3 | c.767delA | p.Q256fs | . | . |
| --- | --- | --- | --- | --- | --- | --- | --- | --- | --- | --- | --- | --- | --- |
| CTC1 | 10 | 88988406 | GT | G | 0.462 | NM_001099338 | NUTM2A | frameshift_deleti  on | exon2 | c.770delT | p.V257fs | . | . |
| CTC1 | 10 | 91066440 | GC | G | 0.417 | NM_001547 | IFIT2 | frameshift_deleti  on | exon2 | c.728delC | p.A243fs | . | . |
| CTC1 | 10 | 91479451 | CGAG | C | 0.119 | NM_001284259 | KIF20B | nonframeshift_de  letion | exon13 | c.1711_1713del | p.571_571del | . | . |
| CTC1 | 10 | 95791037 | GA | G | 0.5 | NM_001288989 | PLCE1 | frameshift_deleti  on | exon2 | c.235delA | p.K79fs | . | . |
| CTC1 | 10 | 96025579 | CA | C | 0.542 | NM_001165979 | PLCE1 | frameshift_deleti  on | exon15 | c.3222delA | p.S1074fs | . | . |
| CTC1 | 10 | 96827314 | TG | T | 0.294 | NM_000770 | CYP2C8 | frameshift_deleti  on | exon2 | c.302delC | p.P101fs | . | . |
| CTC1 | 10 | 98743632 | AG | A | 0.227 | NM_015652 | C10orf12 | frameshift_deleti  on | exon1 | c.2486delG | p.R829fs | . | . |
| CTC1 | 10 | 98744176 | TC | T | 0.441 | NM_015652 | C10orf12 | frameshift_deleti  on | exon1 | c.3030delC | p.L1010fs | . | . |
| CTC1 | 10 | 99141540 | CA | C | 0.442 | NM_001284337 | RRP12 | frameshift_deleti  on | exon8 | c.951delT | p.F317fs | . | . |
| CTC1 | 10 | 99211901 | CTG | C | 0.938 | NM_001287804 | ZDHHC16 | frameshift_deleti  on | exon3 | c.299_300del | p.L100fs | . | . |
| CTC1 | 10 | 101165589 | A | AGG | 0.286 | NM_002079 | GOT1 | frameshift_inserti  on | exon5 | c.565_566insCC | p.L189fs | . | . |
| CTC1 | 10 | 101601749 | GT | G | 0.178 | NM_000392 | ABCC2 | frameshift_deleti  on | exon26 | c.3641delT | p.V1214fs | . | . |
| CTC1 | 10 | 101657940 | TG | T | 0.789 | NM_015221 | DNMBP | frameshift_deleti  on | exon9 | c.2822delC | p.P941fs | . | . |
| CTC1 | 10 | 101953745 | G | GT | 0.625 | NM_001278 | CHUK | frameshift_inserti  on | exon18 | c.1969_1970ins  A | p.A657fs | . | . |
| CTC1 | 10 | 101953746 | C | CA | 0.8 | NM_001278 | CHUK | frameshift_inserti  on | exon18 | c.1968dupT | p.A657fs | . | . |
| CTC1 | 10 | 104159911 | AG | A | 0.333 | NM_001261403 | NFKB2 | frameshift_deleti  on | exon14 | c.1545delG | p.Q515fs | . | . |
| CTC1 | 10 | 104181787 | GA | G | 0.467 | NM_024326 | FBXL15 | frameshift_deleti  on | exon3 | c.452delA | p.D151fs | . | . |
| CTC1 | 10 | 104417011 | TG | T | 0.229 | NM_030912 | TRIM8 | frameshift_deleti  on | exon6 | c.1557delG | p.L519fs | . | . |
| CTC1 | 10 | 104417040 | C | CT | 0.333 | NM_030912 | TRIM8 | frameshift_inserti  on | exon6 | c.1585_1586ins  T | p.Q529fs | . | . |
| CTC1 | 10 | 104597032 | CT | C | 0.312 | NM_000102 | CYP17A1 | frameshift_deleti  on | exon1 | c.86delA | p.K29fs | . | . |
| CTC1 | 10 | 105165795 | TG | T | 0.41 | NM_014976 | PDCD11 | frameshift_deleti  on | exon6 | c.619delG | p.G207fs | . | . |
| CTC1 | 10 | 105891896 | TG | T | 0.308 | NM_025145 | WDR96 | frameshift_deleti  on | exon36 | c.4654delC | p.Q1552fs | . | . |
| CTC1 | 10 | 106927052 | TG | T | 0.39 | NM_014978 | SORCS3 | frameshift_deleti  on | exon13 | c.1847delG | p.W616fs | . | . |
| CTC1 | 10 | 108536400 | TG | T | 0.235 | NM_001013031 | SORCS1 | stopgain | exon4 | c.776delC | p.S259X | . | . |
| CTC1 | 10 | 112540907 | CG | C | 0.364 | NM_001134363 | RBM20 | frameshift_deleti on | exon2 | c.541delG | p.G181fs | . | . |
| CTC1 | 10 | 112724218 | T | TG | 0.5 | NM_001269039 | SHOC2 | frameshift_inserti  on | exon1 | c.103dupG | p.F34fs | . | . |
| CTC1 | 10 | 113940338 | TA | T | 0.8 | NM_001244949 | GPAM | frameshift_deleti  on | exon4 | c.117delT | p.F39fs | . | . |
| CTC1 | 10 | 116085784 | C | CCCG | 1 | NM_001287824 | AFAP1L2 | nonframeshift_in  sertion | exon5 | c.330_331insC  GG | p.G111delinsR  G | . | . |
| CTC1 | 10 | 116085785 | C | CCGT | 1 | NM_001287824 | AFAP1L2 | nonframeshift_in  sertion | exon5 | c.329_330insAC  G | p.L110delinsLR | . | . |
| CTC1 | 10 | 116931101 | C | CTT | 1 | NM_001276282 | ATRNL1 | frameshift_inserti  on | exon8 | c.1399_1400ins  TT | p.L467fs | . | . |
| CTC1 | 10 | 119798845 | AT | A | 0.389 | NM_014904 | RAB11FIP2 | frameshift_deleti  on | exon3 | c.902delA | p.N301fs | . | . |
| CTC1 | 10 | 124596919 | CA | C | 0.314 | NM_022034 | CUZD1 | frameshift_deleti  on | exon4 | c.599delT | p.F200fs | . | . |
| CTC1 | 10 | 126523350 | GA | G | 0.273 | NM_032182 | FAM175B | frameshift_deleti  on | exon9 | c.1059delA | p.G353fs | . | . |
| CTC1 | 10 | 126716178 | AC | A | 0.259 | NM_022802 | CTBP2 | frameshift_deleti  on | exon1 | c.150delG | p.W50fs | . | . |
| CTC1 | 10 | 127530469 | TA | T | 0.714 | NM_018180 | DHX32 | stopgain | exon7 | c.1385delT | p.L462X | . | . |
| CTC1 | 10 | 127548493 | CA | C | 1 | NM_018180 | DHX32 | frameshift_deleti on | exon3 | c.527delT | p.L176fs | . | . |
| CTC1 | 10 | 129905111 | CTG | C | 0.22 | NM_001145966 | MKI67 | frameshift_deleti  on | exon12 | c.3911_3912del | p.T1304fs | . | . |
| CTC1 | 10 | 134693063 | TG | T | 0.195 | NM_001200049 | TTC40 | frameshift_deleti  on | exon29 | c.3871delC | p.Q1291fs | . | . |
| CTC1 | 10 | 135368892 | TG | T | 0.6 | NM_001143763 | SYCE1 | frameshift_deleti  on | exon12 | c.880delC | p.Q294fs | . | . |
| CTC1 | 11 | 198535 | TC | T | 0.667 | NM_001286136 | ODF3 | frameshift_deleti  on | exon5 | c.485delC | p.S162fs | . | . |
| CTC1 | 11 | 686987 | TC | T | 0.303 | NM_021008 | DEAF1 | frameshift_deleti  on | exon5 | c.674delG | p.G225fs | . | . |
| CTC1 | 11 | 1028917 | TC | T | 0.3 | NM_005961 | MUC6 | frameshift_deleti  on | exon12 | c.1424delG | p.G475fs | . | . |
| CTC1 | 11 | 1491432 | C | CA | 0.214 | NM_001172223 | MOB2 | frameshift_inserti  on | exon5 | c.776_777insT | p.P259fs | . | . |
| CTC1 | 11 | 1619376 | AC | A | 0.222 | NM_001004325 | KRTAP5-2 | frameshift_deleti  on | exon1 | c.104delG | p.R35fs | . | . |
| CTC1 | 11 | 1857394 | TG | T | 0.385 | NM_138567 | SYT8 | frameshift_deleti  on | exon5 | c.439delG | p.G147fs | . | . |
| CTC1 | 11 | 2592603 | A | AG | 0.444 | NM_000218 | KCNQ1 | frameshift_inserti  on | exon4 | c.654dupG | p.K218fs | . | . |
| CTC1 | 11 | 4967865 | GC | G | 0.686 | NM_001005329 | OR51A4 | frameshift_deleti  on | exon1 | c.465delG | p.M155fs | . | . |
| CTC1 | 11 | 4976751 | GA | G | 0.269 | NM_001004748 | OR51A2 | frameshift_deleti  on | exon1 | c.192delT | p.F64fs | . | . |
| CTC1 | 11 | 5010907 | GA | G | 0.714 | NM_021801 | MMP26 | frameshift_deleti  on | exon2 | c.130delA | p.K44fs | . | . |
| CTC1 | 11 | 5020655 | TG | T | 0.364 | NM_001004755 | OR51L1 | frameshift_deleti  on | exon1 | c.444delG | p.L148fs | . | . |
| CTC1 | 11 | 5566051 | C | CA | 0.545 | NM_001005289 | OR52H1 | frameshift_inserti  on | exon1 | c.702dupT | p.G235fs | . | . |
| CTC1 | 11 | 5730866 | AC | A | 0.429 | NM_001199573 | TRIM22 | frameshift_deleti  on | exon8 | c.1474delC | p.P492fs | . | . |
| CTC1 | 11 | 5989474 | AC | A | 0.333 | NM_001146033 | OR56A5 | frameshift_deleti  on | exon1 | c.250delG | p.V84fs | . | . |
| CTC1 | 11 | 6661761 | TG | T | 0.657 | NM_003737 | DCHS1 | frameshift_deleti  on | exon2 | c.1083delC | p.S361fs | . | . |
| CTC1 | 11 | 6662745 | CCAG | C | 0.368 | NM_003737 | DCHS1 | nonframeshift_de  letion | exon2 | c.97_99del | p.33_33del | . | . |
| CTC1 | 11 | 6891116 | GC | G | 0.35 | NM_001004460 | OR10A2 | frameshift_deleti  on | exon1 | c.132delC | p.S44fs | . | . |
| CTC1 | 11 | 6942365 | A | AT | 0.286 | NM_001004684 | OR2D3 | frameshift_inserti  on | exon1 | c.134dupT | p.I45fs | . | . |

| CTC1 | 11 | 7063944 | TG | T | 0.316 | NM_176822 | NLRP14 | frameshift_deleti  on | exon4 | c.688delG | p.A230fs | . | . |
| --- | --- | --- | --- | --- | --- | --- | --- | --- | --- | --- | --- | --- | --- |
| CTC1 | 11 | 7531403 | AG | A | 0.373 | NM_198474 | OLFML1 | frameshift_deleti  on | exon3 | c.1194delG | p.K398fs | . | . |
| CTC1 | 11 | 7661047 | TCTC | T | 0.5 | NM_001256569 | PPFIBP2 | nonframeshift_de  letion | exon11 | c.893_895del | p.298_299del | . | . |
| CTC1 | 11 | 7722930 | TG | T | 1 | NM_198185 | OVCH2 | frameshift_deleti  on | exon6 | c.651delC | p.P217fs | . | . |
| CTC1 | 11 | 7725304 | AC | A | 0.489 | NM_198185 | OVCH2 | frameshift_deleti  on | exon3 | c.230delG | p.C77fs | . | . |
| CTC1 | 11 | 7846730 | AC | A | 0.6 | NM_153445 | OR5P3 | frameshift_deleti  on | exon1 | c.789delG | p.K263fs | . | . |
| CTC1 | 11 | 8673349 | TG | T | 0.183 | NM_014818 | TRIM66 | frameshift_deleti  on | exon2 | c.6delC | p.A2fs | . | . |
| CTC1 | 11 | 9537831 | CG | C | 0.68 | NM_001282657 | ZNF143 | frameshift_deleti  on | exon13 | c.1516delG | p.A506fs | . | . |
| CTC1 | 11 | 10875248 | TA | T | 0.346 | NM_001143667 | ZBED5 | frameshift_deleti  on | exon3 | c.1244delT | p.L415fs | . | . |
| CTC1 | 11 | 11988581 | AT | A | 0.461 | NM_001018057 | DKK3 | frameshift_deleti  on | exon5 | c.592delA | p.M198fs | . | . |
| CTC1 | 11 | 12278502 | CT | C | 1 | NM_001282667 | MICAL2 | frameshift_deleti  on | exon16 | c.2386delT | p.F796fs | . | . |
| CTC1 | 11 | 12785947 | GA | G | 0.455 | NM_021961 | TEAD1 | frameshift_deleti  on | exon3 | c.169delA | p.K57fs | . | . |
| CTC1 | 11 | 13514016 | T | TC | 0.875 | NM_000315 | PTH | frameshift_inserti  on | exon3 | c.283dupG | p.E95fs | . | . |
| CTC1 | 11 | 15260486 | TG | T | 0.822 | NM_001278316 | INSC | frameshift_deleti  on | exon9 | c.1134delG | p.M378fs | . | . |
| CTC1 | 11 | 17351782 | CA | C | 0.455 | NM_005013 | NUCB2 | frameshift_deleti  on | exon12 | c.1112delA | p.Q371fs | . | . |
| CTC1 | 11 | 17352477 | TACA | T | 1 | NM_005013 | NUCB2 | nonframeshift_de  letion | exon13 | c.1203_1205del | p.401_402del | . | . |
| CTC1 | 11 | 17574676 | CT | C | 0.226 | NM_001277269 | OTOG | frameshift_deleti  on | exon4 | c.340delT | p.C114fs | . | . |
| CTC1 | 11 | 18054862 | TG | T | 0.939 | NM_004179 | TPH1 | frameshift_deleti  on | exon3 | c.360delC | p.N120fs | . | . |
| CTC1 | 11 | 18362941 | CA | C | 0.476 | NM_005316 | GTF2H1 | frameshift_deleti  on | exon6 | c.742delA | p.K248fs | . | . |
| CTC1 | 11 | 18728741 | TG | T | 0.5 | NM_173588 | IGSF22 | frameshift_deleti  on | exon21 | c.3299delC | p.T1100fs | . | . |
| CTC1 | 11 | 19901573 | AC | A | 0.393 | NM_001111018 | NAV2 | frameshift_deleti  on | exon5 | c.479delC | p.T160fs | . | . |
| CTC1 | 11 | 31816207 | GT | G | 0.778 | NM_001258465 | PAX6 | frameshift_deleti  on | exon7 | c.652delA | p.T218fs | . | . |
| CTC1 | 11 | 32956857 | CT | C | 0.25 | NM_001076786 | QSER1 | frameshift_deleti  on | exon4 | c.3667delT | p.L1223fs | . | . |
| CTC1 | 11 | 33565170 | GA | G | 0.955 | NM_012194 | KIAA1549L | frameshift_deleti  on | exon1 | c.1171delA | p.K391fs | . | . |
| CTC1 | 11 | 34118816 | TCC | T | 0.667 | NM_005898 | CAPRIN1 | frameshift_deleti  on | exon17 | c.1975_1976del | p.P659fs | . | . |
| CTC1 | 11 | 34988274 | AC | A | 0.349 | NM_001135024 | PDHX | frameshift_deleti  on | exon6 | c.685delC | p.P229fs | . | . |
| CTC1 | 11 | 35240899 | CA | C | 0.354 | NM_001202556 | CD44 | frameshift_deleti  on | exon6 | c.705delA | p.T235fs | . | . |
| CTC1 | 11 | 44079914 | G | GA | 0.525 | NM_001031854 | ACCSL | frameshift_inserti  on | exon12 | c.1376dupA | p.E459fs | . | . |
| CTC1 | 11 | 45245857 | C | CA | 0.211 | NM_001256695 | PRDM11 | frameshift_inserti  on | exon7 | c.833dupA | p.Q278fs | . | . |
| CTC1 | 11 | 45671825 | GC | G | 0.188 | NM_003654 | CHST1 | frameshift_deleti  on | exon4 | c.648delG | p.L216fs | . | . |
| CTC1 | 11 | 45923599 | AC | A | 0.69 | NM_005456 | MAPK8IP1 | frameshift_deleti  on | exon4 | c.592delC | p.P198fs | . | . |
| CTC1 | 11 | 46724716 | CAGTGG  TGACAG | C | 1 | NM_001184751 | ZNF408 | nonframeshift_de  letion | exon4 | c.552_563del | p.184_188del | . | . |
| CTC1 | 11 | 46724785 | CT | C | 0.259 | NM_001184751 | ZNF408 | frameshift_deleti  on | exon4 | c.621delT | p.P207fs | . | . |
| CTC1 | 11 | 47254421 | GT | G | 0.281 | NM_000107 | DDB2 | frameshift_deleti  on | exon4 | c.514delT | p.F172fs | . | . |
| CTC1 | 11 | 47609353 | GC | G | 0.571 | NM_001164379 | FAM180B | frameshift_deleti  on | exon2 | c.137delC | p.A46fs | . | . |
| CTC1 | 11 | 47776132 | GTC | G | 0.162 | NM_015308 | FNBP4 | frameshift_deleti  on | exon3 | c.396_397del | p.E132fs | . | . |
| CTC1 | 11 | 55135744 | C | CT | 0.408 | NM_001005275 | OR4A15 | frameshift_inserti  on | exon1 | c.386dupT | p.L129fs | . | . |
| CTC1 | 11 | 55587915 | CA | C | 0.385 | NM_001001952 | OR5D18 | frameshift_deleti  on | exon1 | c.811delA | p.T271fs | . | . |
| CTC1 | 11 | 55890156 | GT | G | 0.462 | NM_001005201 | OR8H3 | frameshift_deleti  on | exon1 | c.309delT | p.C103fs | . | . |
| CTC1 | 11 | 57077537 | AG | A | 0.474 | NM_033396 | TNKS1BP1 | frameshift_deleti  on | exon6 | c.2647delC | p.L883fs | . | . |
| CTC1 | 11 | 57511933 | A | AT | 0.565 | NM_001145101 | BTBD18 | frameshift_inserti  on | exon3 | c.1811dupA | p.D604fs | . | . |
| CTC1 | 11 | 57947416 | CCTT | C | 0.162 | NM_001005212 | OR9Q1 | nonframeshift_de  letion | exon3 | c.501_503del | p.167_168del | . | . |
| CTC1 | 11 | 57958767 | GA | G | 1 | NM_001005283 | OR9Q2 | frameshift_deleti  on | exon1 | c.806delA | p.E269fs | . | . |
| CTC1 | 11 | 57995878 | AG | A | 0.323 | NM_001004471 | OR10Q1 | frameshift_deleti  on | exon1 | c.469delC | p.L157fs | . | . |
| CTC1 | 11 | 59224987 | TA | T | 0.322 | NM_001004708 | OR4D6 | frameshift_deleti  on | exon1 | c.555delA | p.V185fs | . | . |
| CTC1 | 11 | 61026449 | T | TG | 0.45 | NM_152718 | VWCE | frameshift_inserti  on | exon20 | c.2565dupC | p.T856fs | . | . |
| CTC1 | 11 | 61131931 | TG | T | 0.65 | NM_016464 | TMEM138 | frameshift_deleti  on | exon2 | c.70delG | p.V24fs | . | . |
| CTC1 | 11 | 61205580 | CCA | C | 0.167 | NM_017841 | SDHAF2 | frameshift_deleti  on | exon3 | c.366_367del | p.A122fs | . | . |
| CTC1 | 11 | 61254638 | TA | T | 0.259 | NM_001170753 | PPP1R32 | frameshift_deleti  on | exon10 | c.910delA | p.N304fs | . | . |
| CTC1 | 11 | 61544899 | AC | A | 0.481 | NM_001127392 | MYRF | frameshift_deleti  on | exon12 | c.1755delC | p.H585fs | . | . |
| CTC1 | 11 | 61917657 | G | GC | 0.389 | NM_020238 | INCENP | frameshift_inserti  on | exon17 | c.2509dupC | p.K836fs | . | . |
| CTC1 | 11 | 62286815 | AT | A | 0.857 | NM_001620 | AHNAK | frameshift_deleti  on | exon5 | c.15073delA | p.M5025fs | . | . |
| CTC1 | 11 | 62291683 | C | CT | 0.273 | NM_001620 | AHNAK | frameshift_inserti  on | exon5 | c.10205dupA | p.K3402fs | . | . |
| CTC1 | 11 | 62297989 | CT | C | 0.317 | NM_001620 | AHNAK | frameshift_deleti  on | exon5 | c.3899delA | p.E1300fs | . | . |
| CTC1 | 11 | 62363226 | TG | T | 0.696 | NM_004739 | MTA2 | frameshift_deleti  on | exon13 | c.1251delC | p.T417fs | . | . |
| CTC1 | 11 | 62489351 | CT | C | 0.536 | NM_001079559 | HNRNPUL2 | frameshift_deleti  on | exon8 | c.1422delA | p.K474fs | . | . |
| CTC1 | 11 | 62490350 | TC | T | 0.186 | NM_001079559 | HNRNPUL2 | frameshift_deleti  on | exon5 | c.916delG | p.E306fs | . | . |
| CTC1 | 11 | 62767229 | TG | T | 0.317 | NM_001184736 | SLC22A8 | frameshift_deleti  on | exon3 | c.153delC | p.P51fs | . | . |

| CTC1 | 11 | 63525750 | GA | G | 0.667 | NM_001265591 | RTN3 | frameshift_deleti  on | exon4 | c.533delA | p.E178fs | . | . |
| --- | --- | --- | --- | --- | --- | --- | --- | --- | --- | --- | --- | --- | --- |
| CTC1 | 11 | 63719987 | C | CT | 0.237 | NM_024771 | NAA40 | frameshift_inserti  on | exon5 | c.361dupT | p.A120fs | . | . |
| CTC1 | 11 | 63997028 | CT | C | 0.347 | NM_001128613 | NUDT22 | frameshift_deleti  on | exon4 | c.658delT | p.F220fs | . | . |
| CTC1 | 11 | 64054304 | TC | T | 1 | NM_001170880 | GPR137 | frameshift_deleti  on | exon1 | c.309delC | p.V103fs | . | . |
| CTC1 | 11 | 64534484 | T | TTG | 0.7 | NM_001178030 | SF1 | frameshift_inserti  on | exon12 | c.1844_1845ins  CA | p.P615fs | . | . |
| CTC1 | 11 | 64677338 | GC | G | 0.4 | NM_015104 | ATG2A | frameshift_deleti  on | exon14 | c.1921delG | p.A641fs | . | . |
| CTC1 | 11 | 65386530 | TG | T | 1 | NM_032223 | PCNXL3 | frameshift_deleti  on | exon6 | c.1698delG | p.V566fs | . | . |
| CTC1 | 11 | 65486193 | G | GC | 0.368 | NM_001206833 | KAT5 | frameshift_inserti  on | exon10 | c.1242dupC | p.S414fs | . | . |
| CTC1 | 11 | 66392573 | C | CT | 0.194 | NM_006328 | RBM14 | frameshift_inserti  on | exon2 | c.1227dupT | p.S409fs | . | . |
| CTC1 | 11 | 66611450 | G | GA | 0.425 | NM_005133 | RCE1 | frameshift_inserti  on | exon3 | c.313dupA | p.L104fs | . | . |
| CTC1 | 11 | 67077577 | TC | T | 0.385 | NM_017857 | SSH3 | frameshift_deleti  on | exon13 | c.1451delC | p.S484fs | . | . |
| CTC1 | 11 | 67265103 | CA | C | 0.23 | NM_001130848 | PITPNM1 | frameshift_deleti  on | exon13 | c.1829delT | p.L610fs | . | . |
| CTC1 | 11 | 67267269 | TGG | T | 0.314 | NM_001130848 | PITPNM1 | frameshift_deleti  on | exon8 | c.1094_1095del | p.P365fs | . | . |
| CTC1 | 11 | 67810247 | CG | C | 1 | NM_006019 | TCIRG1 | frameshift_deleti  on | exon4 | c.335delG | p.R112fs | . | . |
| CTC1 | 11 | 68326079 | GA | G | 0.579 | NM_001164160 | PPP6R3 | frameshift_deleti  on | exon8 | c.778delA | p.K260fs | . | . |
| CTC1 | 11 | 68675800 | GA | G | 0.867 | NM_002180 | IGHMBP2 | frameshift_deleti  on | exon3 | c.445delA | p.K149fs | . | . |
| CTC1 | 11 | 68855361 | CG | C | 0.562 | NM_139075 | TPCN2 | frameshift_deleti  on | exon25 | c.2200delG | p.G734fs | . | . |
| CTC1 | 11 | 70332640 | AT | A | 0.336 | NM_133266 | SHANK2 | frameshift_deleti  on | exon10 | c.1993delA | p.M665fs | . | . |
| CTC1 | 11 | 71249125 | A | AGGCT  GTGGC TCCGG | 0.136 | NM_021046 | KRTAP5-8 | nonframeshift_in sertion | exon1 | c.24_25insGGC  TGTGGCTCCG GCTGTGGG | p.G8delinsGGC GSGCG | . | . |
| CTC1 | 11 | 72308587 | TG | T | 0.24 | NM_001143839 | PDE2A | frameshift_deleti  on | exon4 | c.378delC | p.A126fs | . | . |
| CTC1 | 11 | 73021068 | TG | T | 0.395 | NM_014786 | ARHGEF17 | frameshift_deleti  on | exon1 | c.1386delG | p.L462fs | . | . |
| CTC1 | 11 | 73076940 | GC | G | 0.313 | NM_014786 | ARHGEF17 | frameshift_deleti  on | exon20 | c.5944delC | p.P1982fs | . | . |
| CTC1 | 11 | 73789440 | G | GA | 0.993 | NM_001286577 | C2CD3 | frameshift_inserti  on | exon23 | c.4322dupT | p.F1441fs | . | . |
| CTC1 | 11 | 74618307 | AC | A | 0.209 | NM_001270380 | XRRA1 | stopgain | exon9 | c.643delG | p.V215X | . | . |
| CTC1 | 11 | 75590945 | AT | A | 0.242 | NM_003369 | UVRAG | frameshift_deleti on | exon4 | c.294delT | p.D98fs | . | . |
| CTC1 | 11 | 76886489 | CA | C | 0.833 | NM_000260 | MYO7A | frameshift_deleti  on | exon18 | c.2167delA | p.K723fs | . | . |
| CTC1 | 11 | 76954788 | T | TA | 0.388 | NM_182833 | GDPD4 | frameshift_inserti  on | exon12 | c.1191dupT | p.I398fs | . | . |
| CTC1 | 11 | 83252840 | TA | T | 0.283 | NM_001142702 | DLG2 | frameshift_deleti  on | exon3 | c.132delT | p.F44fs | . | . |
| CTC1 | 11 | 85406281 | GT | G | 0.733 | NM_032379 | SYTL2 | frameshift_deleti  on | exon9 | c.1087delA | p.T363fs | . | . |
| CTC1 | 11 | 87020616 | CA | C | 0.185 | NM_001168724 | TMEM135 | frameshift_deleti  on | exon9 | c.773delA | p.H258fs | . | . |
| CTC1 | 11 | 92087167 | CT | C | 0.241 | NM_001008781 | FAT3 | frameshift_deleti  on | exon1 | c.1890delT | p.S630fs | . | . |
| CTC1 | 11 | 93122200 | AT | A | 0.571 | NM_181645 | CCDC67 | frameshift_deleti  on | exon9 | c.951delT | p.Y317fs | . | . |
| CTC1 | 11 | 94113926 | GC | G | 0.389 | NM_016540 | GPR83 | frameshift_deleti  on | exon4 | c.660delG | p.V220fs | . | . |
| CTC1 | 11 | 94153332 | GATC | G | 0.188 | NM_005590 | MRE11A | nonframeshift_de  letion | exon19 | c.1999_2001del | p.667_667del | . | . |
| CTC1 | 11 | 94316637 | TC | T | 0.353 | NM_152431 | PIWIL4 | frameshift_deleti  on | exon5 | c.538delC | p.Q180fs | . | . |
| CTC1 | 11 | 95712310 | GT | G | 0.333 | NM_032427 | MAML2 | frameshift_deleti  on | exon5 | c.3272delA | p.N1091fs | . | . |
| CTC1 | 11 | 101375195 | TA | T | 0.889 | NM_004621 | TRPC6 | frameshift_deleti  on | exon2 | c.504delT | p.A168fs | . | . |
| CTC1 | 11 | 102708054 | CT | C | 0.333 | NM_002422 | MMP3 | frameshift_deleti  on | exon9 | c.1307delA | p.K436fs | . | . |
| CTC1 | 11 | 103306695 | TG | T | 0.404 | NM_001377 | DYNC2H1 | frameshift_deleti  on | exon85 | c.12392delG | p.W4131fs | . | . |
| CTC1 | 11 | 105923593 | GA | G | 0.467 | NM_152433 | KBTBD3 | frameshift_deleti  on | exon3 | c.1822delT | p.S608fs | . | . |
| CTC1 | 11 | 105967570 | CT | C | 0.231 | NM_015423 | AASDHPPT | frameshift_deleti  on | exon6 | c.867delT | p.P289fs | . | . |
| CTC1 | 11 | 107260857 | AT | A | 0.278 | NM_152434 | CWF19L2 | frameshift_deleti  on | exon12 | c.1814delA | p.N605fs | . | . |
| CTC1 | 11 | 108032119 | GT | G | 0.835 | NM_002519 | NPAT | frameshift_deleti  on | exon17 | c.3693delA | p.K1231fs | . | . |
| CTC1 | 11 | 108062907 | AT | A | 0.333 | NM_002519 | NPAT | frameshift_deleti  on | exon4 | c.227delA | p.N76fs | . | . |
| CTC1 | 11 | 108154998 | AT | A | 0.462 | NM_000051 | ATM | frameshift_deleti  on | exon26 | c.3792delT | p.H1264fs | . | . |
| CTC1 | 11 | 108380910 | TG | T | 0.267 | NM_015065 | EXPH5 | frameshift_deleti  on | exon6 | c.5323delC | p.Q1775fs | . | . |
| CTC1 | 11 | 108562623 | GT | G | 0.541 | NM_004398 | DDX10 | frameshift_deleti  on | exon8 | c.997delT | p.F333fs | . | . |
| CTC1 | 11 | 111746149 | TA | T | 0.892 | NM_138378 | FDXACB1 | frameshift_deleti  on | exon5 | c.1371delT | p.F457fs | . | . |
| CTC1 | 11 | 113078609 | CG | C | 0.23 | NM_000615 | NCAM1 | frameshift_deleti  on | exon8 | c.802delG | p.D268fs | . | . |
| CTC1 | 11 | 116729010 | CTGT | C | 0.159 | NM_001281749 | SIK3 | nonframeshift_de  letion | exon20 | c.2844_2846del | p.948_949del | . | . |
| CTC1 | 11 | 117332233 | CG | C | 0.306 | NM_020693 | DSCAML1 | frameshift_deleti  on | exon18 | c.3524delC | p.P1175fs | . | . |
| CTC1 | 11 | 117342742 | TC | T | 0.321 | NM_020693 | DSCAML1 | frameshift_deleti  on | exon15 | c.2974delG | p.D992fs | . | . |
| CTC1 | 11 | 117789312 | CGGGCT  GGAGAT | C | 1 | NM_001077263 | TMPRSS13 | nonframeshift_de  letion | exon2 | c.248_262del | p.83_88del | . | . |
| CTC1 | 11 | 117870276 | GC | G | 0.755 | NM_001558 | IL10RA | frameshift_deleti  on | exon7 | c.1658delC | p.A553fs | . | . |
| CTC1 | 11 | 118257231 | GA | G | 0.385 | NM_001204077 | UBE4A | frameshift_deleti  on | exon16 | c.2492delA | p.E831fs | . | . |
| CTC1 | 11 | 118345024 | AG | A | 0.444 | NM_001197104 | KMT2A | frameshift_deleti  on | exon3 | c.3151delG | p.A1051fs | . | . |
| CTC1 | 11 | 118373915 | TA | T | 0.208 | NM_001197104 | KMT2A | frameshift_deleti  on | exon27 | c.7309delA | p.K2437fs | . | . |

| CTC1 | 11 | 118376393 | CT | C | 0.364 | NM_001197104 | KMT2A | frameshift_deleti  on | exon27 | c.9787delT | p.S3263fs | . | . |
| --- | --- | --- | --- | --- | --- | --- | --- | --- | --- | --- | --- | --- | --- |
| CTC1 | 11 | 118378255 | CA | C | 0.246 | NM_001197104 | KMT2A | frameshift_deleti  on | exon28 | c.10767delA | p.A3589fs | . | . |
| CTC1 | 11 | 118502129 | GT | G | 0.355 | NM_001144758 | PHLDB1 | frameshift_deleti  on | exon7 | c.2034delT | p.S678fs | . | . |
| CTC1 | 11 | 118886567 | AC | A | 0.333 | NM_001028 | RPS25 | frameshift_deleti  on | exon4 | c.365delG | p.G122fs | . | . |
| CTC1 | 11 | 118897364 | GC | G | 0.323 | NM_001164280 | SLC37A4 | frameshift_deleti  on | exon6 | c.818delG | p.G273fs | . | . |
| CTC1 | 11 | 118920537 | AT | A | 0.759 | NM_001130991 | HYOU1 | frameshift_deleti  on | exon15 | c.1677delA | p.V559fs | . | . |
| CTC1 | 11 | 118920539 | AC | A | 0.444 | NM_001130991 | HYOU1 | frameshift_deleti  on | exon15 | c.1675delG | p.V559fs | . | . |
| CTC1 | 11 | 118926266 | GT | G | 0.286 | NM_001130991 | HYOU1 | frameshift_deleti  on | exon4 | c.202delA | p.T68fs | . | . |
| CTC1 | 11 | 119053858 | CG | C | 0.667 | NM_001282143 | NLRX1 | frameshift_deleti  on | exon10 | c.2639delG | p.R880fs | . | . |
| CTC1 | 11 | 119182015 | GT | G | 0.887 | NM_006500 | MCAM | frameshift_deleti  on | exon12 | c.1523delA | p.N508fs | . | . |
| CTC1 | 11 | 119206983 | AG | A | 0.183 | NM_032015 | RNF26 | frameshift_deleti  on | exon1 | c.1152delG | p.Q384fs | . | . |
| CTC1 | 11 | 120347447 | GA | G | 0.429 | NM_001198665 | ARHGEF12 | frameshift_deleti  on | exon33 | c.3299delA | p.E1100fs | . | . |
| CTC1 | 11 | 122677173 | AG | A | 0.25 | NM_032873 | UBASH3B | frameshift_deleti  on | exon12 | c.1652delG | p.S551fs | . | . |
| CTC1 | 11 | 123988420 | G | GT | 0.423 | NM_014622 | VWA5A | frameshift_inserti  on | exon3 | c.85dupT | p.E28fs | . | . |
| CTC1 | 11 | 124500644 | G | GA | 0.625 | NM_032811 | TBRG1 | frameshift_inserti  on | exon7 | c.841dupA | p.G280fs | . | . |
| CTC1 | 11 | 124524663 | A | AT | 0.36 | NM_170601 | SIAE | frameshift_inserti  on | exon4 | c.431_432insA | p.S144fs | . | . |
| CTC1 | 11 | 124628354 | T | TC | 1 | NM_138961 | ESAM | frameshift_inserti  on | exon2 | c.139dupG | p.E47fs | . | . |
| CTC1 | 11 | 125281698 | AC | A | 0.186 | NM_022062 | PKNOX2 | frameshift_deleti  on | exon10 | c.874delC | p.R292fs | . | . |
| CTC1 | 11 | 125765959 | C | CT | 0.208 | NM_031307 | PUS3 | stopgain | exon2 | c.220_221insA | p.W74_G75deli  nsX | . | . |
| CTC1 | 11 | 126145251 | TG | T | 0.175 | NM_017547 | FOXRED1 | frameshift_deleti  on | exon6 | c.662delG | p.W221fs | . | . |
| CTC1 | 11 | 128839216 | TC | T | 0.231 | NM_014715 | ARHGAP32 | frameshift_deleti  on | exon13 | c.4802delG | p.R1601fs | . | . |
| CTC1 | 11 | 129812382 | TG | T | 0.417 | NM_199438 | PRDM10 | frameshift_deleti  on | exon3 | c.646delC | p.Q216fs | . | . |
| CTC1 | 11 | 130131376 | A | AT | 0.6 | NM_014155 | ZBTB44 | frameshift_inserti  on | exon2 | c.392dupA | p.N131fs | . | . |
| CTC1 | 11 | 130332463 | CT | C | 0.5 | NM_139055 | ADAMTS15 | frameshift_deleti  on | exon4 | c.1331delT | p.L444fs | . | . |
| CTC1 | 11 | 130343487 | T | TC | 0.571 | NM_139055 | ADAMTS15 | frameshift_inserti  on | exon8 | c.2625dupC | p.V875fs | . | . |
| CTC1 | 11 | 130780029 | G | GT | 0.639 | NM_014758 | SNX19 | frameshift_inserti  on | exon4 | c.1917dupA | p.L640fs | . | . |
| CTC1 | 12 | 974355 | T | TC | 0.957 | NM_213655 | WNK1 | frameshift_inserti  on | exon9 | c.2220dupC | p.L740fs | . | . |
| CTC1 | 12 | 2062323 | T | TTGC | 0.452 | NM_152640 | DCP1B | nonframeshift_in  sertion | exon7 | c.782_783insG  CA | p.Q261delinsQ  Q | . | . |
| CTC1 | 12 | 2062332 | C | CTGT | 0.312 | NM_152640 | DCP1B | nonframeshift_in  sertion | exon7 | c.773_774insAC  A | p.Q258delinsQ  Q | . | . |
| CTC1 | 12 | 2695044 | GC | G | 0.289 | NM_000719 | CACNA1C | frameshift_deleti  on | exon18 | c.2505delC | p.S835fs | . | . |
| CTC1 | 12 | 2786974 | G | GA | 0.5 | NM_001129837 | CACNA1C | frameshift_inserti  on | exon40 | c.5057dupA | p.E1686fs | . | . |
| CTC1 | 12 | 2794919 | AC | A | 0.453 | NM_001129837 | CACNA1C | frameshift_deleti  on | exon43 | c.5616delC | p.D1872fs | . | . |
| CTC1 | 12 | 2973853 | GC | G | 1 | NM_001243088 | FOXM1 | frameshift_deleti  on | exon6 | c.1040delG | p.G347fs | . | . |
| CTC1 | 12 | 3046829 | GA | G | 0.75 | NM_001160408 | TULP3 | frameshift_deleti  on | exon9 | c.958delA | p.K320fs | . | . |
| CTC1 | 12 | 3048547 | TTG | T | 0.199 | NM_001160408 | TULP3 | frameshift_deleti  on | exon11 | c.1267_1268del | p.C423fs | . | . |
| CTC1 | 12 | 4721879 | GA | G | 0.513 | NM_001282285 | DYRK4 | frameshift_deleti  on | exon4 | c.459delA | p.R153fs | . | . |
| CTC1 | 12 | 5603835 | G | GT | 0.214 | NM_002527 | NTF3 | frameshift_inserti  on | exon1 | c.456dupT | p.C152fs | . | . |
| CTC1 | 12 | 6425477 | CT | C | 0.533 | NM_001144857 | PLEKHG6 | frameshift_deleti  on | exon6 | c.568delT | p.F190fs | . | . |
| CTC1 | 12 | 6619804 | G | GT | 0.333 | NM_014865 | NCAPD2 | frameshift_inserti  on | exon5 | c.272_273insT | p.R91fs | . | . |
| CTC1 | 12 | 6981731 | AG | A | 0.247 | NM_001146316 | SPSB2 | frameshift_deleti  on | exon2 | c.334delC | p.L112fs | . | . |
| CTC1 | 12 | 7174411 | G | GA | 0.267 | NM_001734 | C1S | frameshift_inserti  on | exon9 | c.1057dupA | p.L352fs | . | . |
| CTC1 | 12 | 7249505 | TC | T | 0.377 | NM_016546 | C1RL | frameshift_deleti  on | exon6 | c.945delG | p.G315fs | . | . |
| CTC1 | 12 | 7519865 | TGA | T | 0.185 | NM_174941 | CD163L1 | frameshift_deleti  on | exon18 | c.4244_4245del | p.L1415fs | . | . |
| CTC1 | 12 | 9098995 | G | GAAC | 0.583 | NM_001207024 | M6PR | nonframeshift_in  sertion | exon2 | c.5_6insGTT | p.F2delinsLF | . | . |
| CTC1 | 12 | 9225323 | GC | G | 0.225 | NM_000014 | A2M | frameshift_deleti  on | exon30 | c.3900delG | p.L1300fs | . | . |
| CTC1 | 12 | 9994445 | GTGT | G | 1 | NM_016523 | KLRF1 | nonframeshift_de  letion | exon4 | c.373_375del | p.125_125del | . | . |
| CTC1 | 12 | 10568391 | AT | A | 0.385 | NM_002261 | KLRC3 | stopgain | exon6 | c.589delA | p.I197X | . | . |
| CTC1 | 12 | 11174672 | TG | T | 0.333 | NM_176888 | TAS2R19 | frameshift_deleti on | exon1 | c.498delC | p.I166fs | . | . |
| CTC1 | 12 | 12419662 | GC | G | 0.32 | NM_002336 | LRP6 | frameshift_deleti  on | exon1 | c.7delG | p.A3fs | . | . |
| CTC1 | 12 | 12630664 | CTGCAC  GCTG | C | 0.375 | NM_030640 | DUSP16 | nonframeshift_de  letion | exon7 | c.1092_1100del | p.364_367del | . | . |
| CTC1 | 12 | 12630665 | TGCACG  CTGG | T | 0.462 | NM_030640 | DUSP16 | nonframeshift_de  letion | exon7 | c.1091_1099del | p.364_367del | . | . |
| CTC1 | 12 | 13140128 | CT | C | 0.25 | NM_015987 | HEBP1 | frameshift_deleti  on | exon3 | c.355delA | p.S119fs | . | . |
| CTC1 | 12 | 14019070 | TG | T | 0.361 | NM_000834 | GRIN2B | frameshift_deleti  on | exon2 | c.72delC | p.S24fs | . | . |
| CTC1 | 12 | 14613481 | AT | A | 0.4 | NM_001286514 | ATF7IP | frameshift_deleti  on | exon9 | c.2209delT | p.L737fs | . | . |
| CTC1 | 12 | 14775119 | CT | C | 0.222 | NM_004963 | GUCY2C | frameshift_deleti  on | exon22 | c.2420delA | p.K807fs | . | . |
| CTC1 | 12 | 15777244 | CT | C | 0.25 | NM_004447 | EPS8 | frameshift_deleti  on | exon19 | c.2141delA | p.Q714fs | . | . |
| CTC1 | 12 | 16757804 | CT | C | 0.75 | NM_001243611 | LMO3 | frameshift_deleti  on | exon1 | c.19delA | p.S7fs | . | . |

| CTC1 | 12 | 18435398 | GCCC | G | 0.731 | NM_001288772 | PIK3C2G | nonframeshift_de  letion | exon2 | c.384_386del | p.128_129del | . | . |
| --- | --- | --- | --- | --- | --- | --- | --- | --- | --- | --- | --- | --- | --- |
| CTC1 | 12 | 21015413 | AG | A | 0.55 | NM_019844 | SLCO1B3 | frameshift_deleti  on | exon7 | c.550delG | p.G184fs | . | . |
| CTC1 | 12 | 21590682 | TC | T | 0.786 | NM_024854 | PYROXD1 | frameshift_deleti  on | exon1 | c.19delC | p.P7fs | . | . |
| CTC1 | 12 | 21997466 | A | AG | 0.471 | NM_005691 | ABCC9 | frameshift_inserti  on | exon26 | c.3265dupC | p.L1089fs | . | . |
| CTC1 | 12 | 27077300 | AT | A | 0.667 | NM_018164 | ASUN | frameshift_deleti  on | exon7 | c.791delA | p.N264fs | . | . |
| CTC1 | 12 | 27081670 | TA | T | 0.345 | NM_018164 | ASUN | frameshift_deleti  on | exon4 | c.468delT | p.N156fs | . | . |
| CTC1 | 12 | 27148215 | CA | C | 0.157 | NM_016551 | TM7SF3 | frameshift_deleti  on | exon5 | c.644delT | p.L215fs | . | . |
| CTC1 | 12 | 27472331 | GA | G | 0.554 | NM_015000 | STK38L | frameshift_deleti  on | exon12 | c.1151delA | p.E384fs | . | . |
| CTC1 | 12 | 31256271 | AC | A | 0.6 | NM_004399 | DDX11 | frameshift_deleti  on | exon24 | c.2329delC | p.P777fs | . | . |
| CTC1 | 12 | 40740641 | AT | A | 1 | NM_198578 | LRRK2 | frameshift_deleti  on | exon42 | c.6197delT | p.I2066fs | . | . |
| CTC1 | 12 | 42854412 | AT | A | 0.273 | NM_001144881 | PRICKLE1 | frameshift_deleti  on | exon8 | c.1694delA | p.N565fs | . | . |
| CTC1 | 12 | 42858713 | AT | A | 0.326 | NM_001144881 | PRICKLE1 | frameshift_deleti  on | exon7 | c.1122delA | p.K374fs | . | . |
| CTC1 | 12 | 46320091 | G | GT | 0.364 | NM_004719 | SCAF11 | frameshift_inserti  on | exon11 | c.3392_3393ins  A | p.F1131fs | . | . |
| CTC1 | 12 | 47172515 | CA | C | 0.346 | NM_001143824 | SLC38A4 | frameshift_deleti  on | exon10 | c.761delT | p.L254fs | . | . |
| CTC1 | 12 | 47471231 | CA | C | 0.409 | NM_181847 | AMIGO2 | frameshift_deleti  on | exon2 | c.1554delT | p.F518fs | . | . |
| CTC1 | 12 | 48192387 | G | GT | 0.779 | NM_001098416 | HDAC7 | frameshift_inserti  on | exon4 | c.306dupA | p.Q103fs | . | . |
| CTC1 | 12 | 49308298 | CT | C | 0.839 | NM_033124 | CCDC65 | frameshift_deleti  on | exon3 | c.413delT | p.L138fs | . | . |
| CTC1 | 12 | 49719554 | GC | G | 0.818 | NM_005480 | TROAP | frameshift_deleti  on | exon5 | c.521delC | p.A174fs | . | . |
| CTC1 | 12 | 49742874 | AC | A | 1 | NM_024902 | DNAJC22 | frameshift_deleti  on | exon2 | c.220delC | p.P74fs | . | . |
| CTC1 | 12 | 50036068 | AC | A | 0.289 | NM_001031698 | PRPF40B | frameshift_deleti  on | exon20 | c.1936delC | p.R646fs | . | . |
| CTC1 | 12 | 50045734 | GA | G | 0.25 | NM_198900 | FMNL3 | frameshift_deleti  on | exon13 | c.1431delT | p.P477fs | . | . |
| CTC1 | 12 | 50187585 | A | AT | 0.348 | NM_001037806 | NCKAP5L | frameshift_inserti  on | exon9 | c.3189_3190ins  A | p.W1064fs | . | . |
| CTC1 | 12 | 50500153 | TG | T | 0.456 | NM_001257199 | GPD1 | frameshift_deleti  on | exon4 | c.375delG | p.M125fs | . | . |
| CTC1 | 12 | 51857448 | AC | A | 0.333 | NM_001039960 | SLC4A8 | frameshift_deleti  on | exon11 | c.1300delC | p.Q434fs | . | . |
| CTC1 | 12 | 52756035 | CTG | C | 0.6 | NM_002283 | KRT85 | frameshift_deleti  on | exon7 | c.1296_1297del | p.H432fs | . | . |
| CTC1 | 12 | 52756098 | GA | G | 0.333 | NM_002283 | KRT85 | frameshift_deleti  on | exon7 | c.1234delT | p.S412fs | . | . |
| CTC1 | 12 | 52822188 | TG | T | 0.742 | NM_004693 | KRT75 | frameshift_deleti  on | exon7 | c.1233delC | p.A411fs | . | . |
| CTC1 | 12 | 52822236 | C | CA | 0.552 | NM_004693 | KRT75 | frameshift_inserti  on | exon7 | c.1185dupT | p.A396fs | . | . |
| CTC1 | 12 | 52941716 | GT | G | 0.292 | NM_033448 | KRT71 | frameshift_deleti  on | exon6 | c.1028delA | p.N343fs | . | . |
| CTC1 | 12 | 53189413 | ACCAAA  GCCACC AGCCCC | A | 1 | NM_057088 | KRT3 | nonframeshift_de letion | exon1 | c.396_413del | p.132_138del | . | . |
| CTC1 | 12 | 53207615 | AC | A | 0.438 | NM_002272 | KRT4 | frameshift_deleti  on | exon1 | c.227delG | p.G76fs | . | . |
| CTC1 | 12 | 53578734 | CAAG | C | 0.191 | NM_001004304 | ZNF740 | nonframeshift_de  letion | exon3 | c.70_72del | p.24_24del | . | . |
| CTC1 | 12 | 54686452 | GT | G | 0.831 | NM_001136023 | NFE2 | frameshift_deleti  on | exon3 | c.827delA | p.N276fs | . | . |
| CTC1 | 12 | 55759191 | AT | A | 1 | NM_001005497 | OR6C75 | frameshift_deleti  on | exon1 | c.298delT | p.F100fs | . | . |
| CTC1 | 12 | 56082676 | CG | C | 0.333 | NM_001144996 | ITGA7 | frameshift_deleti  on | exon22 | c.2921delC | p.A974fs | . | . |
| CTC1 | 12 | 56500466 | GA | G | 0.8 | NM_006191 | PA2G4 | frameshift_deleti  on | exon2 | c.184delA | p.K62fs | . | . |
| CTC1 | 12 | 56638705 | CG | C | 0.418 | NM_173595 | ANKRD52 | frameshift_deleti  on | exon23 | c.2560delC | p.R854fs | . | . |
| CTC1 | 12 | 56642617 | AG | A | 0.417 | NM_173595 | ANKRD52 | frameshift_deleti  on | exon16 | c.1629delC | p.P543fs | . | . |
| CTC1 | 12 | 56712914 | TTC | T | 0.254 | NM_001127460 | PAN2 | frameshift_deleti  on | exon24 | c.3332_3333del | p.R1111fs | . | . |
| CTC1 | 12 | 56822753 | AC | A | 0.476 | NM_003920 | TIMELESS | frameshift_deleti  on | exon11 | c.1217delG | p.R406fs | . | . |
| CTC1 | 12 | 56822785 | GC | G | 0.304 | NM_003920 | TIMELESS | frameshift_deleti  on | exon11 | c.1185delG | p.R395fs | . | . |
| CTC1 | 12 | 56995396 | TG | T | 0.55 | NM_013449 | BAZ2A | frameshift_deleti  on | exon20 | c.4010delC | p.T1337fs | . | . |
| CTC1 | 12 | 57114187 | ACT | A | 0.273 | NM_001113203 | NACA | frameshift_deleti  on | exon3 | c.1125_1126del | p.P375fs | . | . |
| CTC1 | 12 | 57905638 | GT | G | 0.8 | NM_004990 | MARS | frameshift_deleti  on | exon12 | c.1527delT | p.G509fs | . | . |
| CTC1 | 12 | 57919555 | AC | A | 0.667 | NM_052897 | MBD6 | frameshift_deleti  on | exon6 | c.805delC | p.P269fs | . | . |
| CTC1 | 12 | 57921001 | AC | A | 1 | NM_052897 | MBD6 | frameshift_deleti  on | exon7 | c.2074delC | p.P692fs | . | . |
| CTC1 | 12 | 58142985 | AC | A | 0.185 | NM_000075 | CDK4 | frameshift_deleti  on | exon7 | c.798delG | p.E266fs | . | . |
| CTC1 | 12 | 59266381 | AT | A | 0.205 | NM_001136051 | LRIG3 | frameshift_deleti  on | exon19 | c.3152delA | p.N1051fs | . | . |
| CTC1 | 12 | 63544507 | TC | T | 1 | NM_000706 | AVPR1A | frameshift_deleti  on | exon1 | c.109delG | p.E37fs | . | . |
| CTC1 | 12 | 64712546 | A | AGTT | 0.46 | NM_001170633 | C12orf56 | nonframeshift_in  sertion | exon4 | c.702_703insAA  C | p.S235delinsNS | . | . |
| CTC1 | 12 | 64890975 | CG | C | 0.25 | NM_013254 | TBK1 | frameshift_deleti  on | exon18 | c.1896delG | p.S632fs | . | . |
| CTC1 | 12 | 66786479 | C | CA | 0.235 | NM_001178074 | GRIP1 | frameshift_inserti  on | exon17 | c.2090dupT | p.L697fs | . | . |
| CTC1 | 12 | 70195416 | GA | G | 0.438 | NM_001024647 | RAB3IP | frameshift_deleti  on | exon6 | c.428delA | p.E143fs | . | . |
| CTC1 | 12 | 74932090 | CA | C | 0.271 | NM_001136262 | ATXN7L3B | frameshift_deleti  on | exon1 | c.199delA | p.K67fs | . | . |
| CTC1 | 12 | 75692653 | AT | A | 0.345 | NM_001286548 | CAPS2 | frameshift_deleti  on | exon11 | c.308delA | p.N103fs | . | . |
| CTC1 | 12 | 78513266 | CA | C | 0.462 | NM_001024383 | NAV3 | frameshift_deleti  on | exon15 | c.3291delA | p.P1097fs | . | . |
| CTC1 | 12 | 82747015 | AT | A | 0.8 | NM_014167 | CCDC59 | frameshift_deleti  on | exon4 | c.640delA | p.I214fs | . | . |

| CTC1 | 12 | 85638627 | GC | G | 0.418 | NM_001079910 | LRRIQ1 | frameshift_deleti  on | exon27 | c.5078delC | p.A1693fs | . | . |
| --- | --- | --- | --- | --- | --- | --- | --- | --- | --- | --- | --- | --- | --- |
| CTC1 | 12 | 89918230 | G | GA | 0.5 | NM_003774 | GALNT4 | frameshift_inserti  on | exon1 | c.96dupT | p.H33fs | . | . |
| CTC1 | 12 | 89919723 | GGA | G | 0.316 | NM_001199782 | POC1B-  GALNT4 | frameshift_deleti  on | exon1 | c.208_209del | p.S70fs | . | . |
| CTC1 | 12 | 89919734 | GA | G | 0.211 | NM_001199782 | POC1B-  GALNT4 | frameshift_deleti  on | exon1 | c.198delT | p.H66fs | . | . |
| CTC1 | 12 | 91348253 | TC | T | 1 | NM_152638 | CCER1 | frameshift_deleti  on | exon1 | c.266delG | p.G89fs | . | . |
| CTC1 | 12 | 91502554 | A | AT | 0.595 | NM_002345 | LUM | frameshift_inserti  on | exon2 | c.202dupA | p.I68fs | . | . |
| CTC1 | 12 | 91546921 | AT | A | 0.714 | NM_133505 | DCN | frameshift_deleti  on | exon2 | c.256delA | p.I86fs | . | . |
| CTC1 | 12 | 93149639 | CA | C | 0.5 | NM_001004330 | PLEKHG7 | frameshift_deleti  on | exon7 | c.530delA | p.Q177fs | . | . |
| CTC1 | 12 | 94575317 | TG | T | 0.4 | NM_005761 | PLXNC1 | stopgain | exon3 | c.1300delG | p.V434X | . | . |
| CTC1 | 12 | 94580150 | TG | T | 1 | NM_005761 | PLXNC1 | frameshift_deleti on | exon4 | c.1341delG | p.V447fs | . | . |
| CTC1 | 12 | 95604614 | TC | T | 0.237 | NM_018351 | FGD6 | frameshift_deleti  on | exon2 | c.445delG | p.E149fs | . | . |
| CTC1 | 12 | 100613798 | AT | A | 1 | NM_022496 | ACTR6 | frameshift_deleti  on | exon10 | c.936delT | p.H312fs | . | . |
| CTC1 | 12 | 101368641 | GC | G | 0.246 | NM_001286616 | ANO4 | frameshift_deleti  on | exon6 | c.577delC | p.P193fs | . | . |
| CTC1 | 12 | 101750797 | TG | T | 0.444 | NM_014503 | UTP20 | frameshift_deleti  on | exon43 | c.5629delG | p.G1877fs | . | . |
| CTC1 | 12 | 102036236 | A | AT | 0.5 | NM_001254722 | MYBPC1 | frameshift_inserti  on | exon7 | c.553dupT | p.V184fs | . | . |
| CTC1 | 12 | 104341187 | TGAA | T | 0.947 | NM_003299 | HSP90B1 | nonframeshift_de  letion | exon17 | c.2362_2364del | p.788_788del | . | . |
| CTC1 | 12 | 109055871 | CT | C | 0.684 | NM_001105237 | CORO1C | frameshift_deleti  on | exon4 | c.540delA | p.E180fs | . | . |
| CTC1 | 12 | 110011231 | GCAGGC  CAAGAC GGCTCC | G | 0.2 | NM_052845 | MMAB | nonframeshift_de letion | exon1 | c.37_54del | p.13_18del | . | . |
| CTC1 | 12 | 110429494 | C | CT | 1 | NM_001135213 | GIT2 | frameshift_inserti  on | exon2 | c.123dupA | p.G42fs | . | . |
| CTC1 | 12 | 112622603 | CA | C | 0.216 | NM_001109662 | HECTD4 | frameshift_deleti  on | exon61 | c.9764delT | p.L3255fs | . | . |
| CTC1 | 12 | 116446917 | C | CA | 0.138 | NM_015335 | MED13L | frameshift_inserti  on | exon10 | c.1300dupT | p.C434fs | . | . |
| CTC1 | 12 | 117665244 | CG | C | 0.595 | NM_001204213 | NOS1 | frameshift_deleti  on | exon22 | c.2599delC | p.R867fs | . | . |
| CTC1 | 12 | 118199247 | C | CG | 0.441 | NM_173598 | KSR2 | frameshift_inserti  on | exon4 | c.467dupC | p.P156fs | . | . |
| CTC1 | 12 | 118456902 | CT | C | 1 | NM_001206801 | RFC5 | frameshift_deleti  on | exon2 | c.92delT | p.L31fs | . | . |
| CTC1 | 12 | 118506327 | TTCCTCC | T | 1 | NM_019086 | VSIG10 | nonframeshift_de  letion | exon8 | c.1416_1421del | p.472_474del | . | . |
| CTC1 | 12 | 118509224 | GC | G | 0.467 | NM_019086 | VSIG10 | frameshift_deleti  on | exon6 | c.1269delG | p.L423fs | . | . |
| CTC1 | 12 | 118693252 | CA | C | 0.8 | NM_016281 | TAOK3 | frameshift_deleti  on | exon3 | c.120delT | p.F40fs | . | . |
| CTC1 | 12 | 120196427 | TG | T | 0.561 | NM_007174 | CIT | frameshift_deleti  on | exon20 | c.2372delC | p.A791fs | . | . |
| CTC1 | 12 | 120221778 | CA | C | 0.535 | NM_001206999 | CIT | frameshift_deleti  on | exon12 | c.1478delT | p.L493fs | . | . |
| CTC1 | 12 | 120587806 | TC | T | 0.4 | NM_006836 | GCN1L1 | frameshift_deleti  on | exon35 | c.4350delG | p.G1450fs | . | . |
| CTC1 | 12 | 120634668 | CA | C | 0.25 | NM_001002 | RPLP0 | frameshift_deleti  on | exon8 | c.861delT | p.A287fs | . | . |
| CTC1 | 12 | 120984266 | C | CA | 0.423 | NM_014868 | RNF10 | frameshift_inserti  on | exon2 | c.217dupA | p.P72fs | . | . |
| CTC1 | 12 | 121678327 | C | CTTTT | 0.933 | NM_001270486 | CAMKK2 | frameshift_inserti  on | exon16 | c.1614_1615ins  AAAA | p.G539fs | . | . |
| CTC1 | 12 | 121678327 | C | CTTT | 0.933 | NM_001270486 | CAMKK2 | nonframeshift_in  sertion | exon16 | c.1614_1615ins  AAA | p.G539delinsK  G | . | . |
| CTC1 | 12 | 121678626 | CG | C | 0.581 | NM_153500 | CAMKK2 | frameshift_deleti  on | exon15 | c.1470delC | p.P490fs | . | . |
| CTC1 | 12 | 121768468 | AG | A | 0.273 | NM_001137559 | ANAPC5 | frameshift_deleti  on | exon9 | c.742delC | p.L248fs | . | . |
| CTC1 | 12 | 122396250 | A | AT | 0.222 | NM_001178003 | WDR66 | frameshift_inserti  on | exon12 | c.1804dupT | p.L601fs | . | . |
| CTC1 | 12 | 123834889 | C | CA | 0.348 | NM_001167856 | SBNO1 | frameshift_inserti  on | exon1 | c.99dupT | p.A34fs | . | . |
| CTC1 | 12 | 124297923 | AT | A | 0.25 | NM_207437 | DNAH10 | frameshift_deleti  on | exon19 | c.3004delT | p.L1002fs | . | . |
| CTC1 | 12 | 124416514 | C | CA | 0.3 | NM_207437 | DNAH10 | frameshift_inserti  on | exon75 | c.12802dupA | p.S4267fs | . | . |
| CTC1 | 12 | 124824721 | C | CGCCG  CTGCT | 1 | NM_001077261 | NCOR2 | nonframeshift_in  sertion | exon38 | c.5487_5488ins  AGCAGCGGC | p.G1830delinsS  SGG | . | . |
| CTC1 | 12 | 124824908 | TC | T | 0.444 | NM_001077261 | NCOR2 | frameshift_deleti  on | exon37 | c.5389delG | p.D1797fs | . | . |
| CTC1 | 12 | 124887058 | G | GGCTG  CT | 1 | NM_001077261 | NCOR2 | nonframeshift_in  sertion | exon16 | c.1528_1529ins  AGCAGC | p.P510delinsQ  QP | . | . |
| CTC1 | 12 | 124887058 | G | GGCT | 1 | NM_001077261 | NCOR2 | nonframeshift_in  sertion | exon16 | c.1528_1529ins  AGC | p.P510delinsQ  P | . | . |
| CTC1 | 12 | 124915227 | CG | C | 0.213 | NM_001077261 | NCOR2 | frameshift_deleti  on | exon11 | c.988delC | p.R330fs | . | . |
| CTC1 | 12 | 125465269 | ACTC | A | 0.148 | NM_032656 | DHX37 | nonframeshift_de  letion | exon4 | c.502_504del | p.168_168del | . | . |
| CTC1 | 12 | 129190201 | TG | T | 1 | NM_001136103 | TMEM132C | frameshift_deleti  on | exon9 | c.2689delG | p.A897fs | . | . |
| CTC1 | 12 | 129559163 | GT | G | 0.225 | NM_133448 | TMEM132D | frameshift_deleti  on | exon9 | c.2556delA | p.G852fs | . | . |
| CTC1 | 12 | 130184659 | TC | T | 0.361 | NM_133448 | TMEM132D | frameshift_deleti  on | exon2 | c.663delG | p.G221fs | . | . |
| CTC1 | 12 | 132198766 | TGAG | T | 0.3 | NM_001261411 | SFSWAP | nonframeshift_de  letion | exon2 | c.370_372del | p.124_124del | . | . |
| CTC1 | 12 | 132426442 | G | GA | 0.4 | NM_001002019 | PUS1 | frameshift_inserti  on | exon5 | c.1067dupA | p.E356fs | . | . |
| CTC1 | 12 | 132547087 | G | GCAA | 0.354 | NM_015409 | EP400 | nonframeshift_in  sertion | exon47 | c.8175_8176ins  CAA | p.Q2725delins  QQ | . | . |
| CTC1 | 12 | 132547093 | A | ACAG | 0.742 | NM_015409 | EP400 | nonframeshift_in  sertion | exon47 | c.8181_8182ins  CAG | p.Q2727delins  QQ | . | . |
| CTC1 | 12 | 132547111 | G | GCAA | 0.413 | NM_015409 | EP400 | nonframeshift_in  sertion | exon47 | c.8199_8200ins  CAA | p.Q2733delins  QQ | . | . |
| CTC1 | 12 | 132547127 | C | CAGG | 0.449 | NM_015409 | EP400 | nonframeshift_in  sertion | exon47 | c.8215_8216ins  AGG | p.Q2739delins  QE | . | . |

| CTC1 | 12 | 132837539 | CG | C | 0.333 | NM_001122636 | GALNT9 | frameshift_deleti  on | exon4 | c.755delC | p.T252fs | . | . |
| --- | --- | --- | --- | --- | --- | --- | --- | --- | --- | --- | --- | --- | --- |
| CTC1 | 12 | 133281226 | C | CT | 0.157 | NM_018663 | PXMP2 | frameshift_inserti  on | exon5 | c.542dupT | p.L181fs | . | . |
| CTC1 | 12 | 133306346 | AT | A | 0.455 | NM_015114 | ANKLE2 | frameshift_deleti  on | exon11 | c.2401delA | p.M801fs | . | . |
| CTC1 | 12 | 133306740 | CA | C | 0.263 | NM_015114 | ANKLE2 | frameshift_deleti  on | exon11 | c.2007delT | p.F669fs | . | . |
| CTC1 | 12 | 133698069 | GC | G | 0.429 | NM_001277291 | ZNF891 | frameshift_deleti  on | exon2 | c.435delG | p.M145fs | . | . |
| CTC1 | 12 | 133780616 | TG | T | 0.407 | NM_001165881 | ZNF268 | frameshift_deleti  on | exon6 | c.2345delG | p.C782fs | . | . |
| CTC1 | 13 | 20067011 | AG | A | 0.312 | NM_130785 | TPTE2 | frameshift_deleti  on | exon4 | c.97delC | p.L33fs | . | . |
| CTC1 | 13 | 21295915 | GC | G | 0.278 | NM_138284 | IL17D | frameshift_deleti  on | exon3 | c.432delC | p.R144fs | . | . |
| CTC1 | 13 | 21383247 | CT | C | 0.243 | NM_022459 | XPO4 | frameshift_deleti  on | exon11 | c.1467delA | p.A489fs | . | . |
| CTC1 | 13 | 23906110 | GT | G | 0.765 | NM_001278055 | SACS | frameshift_deleti  on | exon8 | c.11463delA | p.K3821fs | . | . |
| CTC1 | 13 | 25479835 | A | AG | 0.5 | NM_018451 | CENPJ | frameshift_inserti  on | exon7 | c.2340dupC | p.S781fs | . | . |
| CTC1 | 13 | 25479901 | AC | A | 0.197 | NM_018451 | CENPJ | frameshift_deleti  on | exon7 | c.2274delG | p.L758fs | . | . |
| CTC1 | 13 | 25841981 | G | GA | 0.342 | NM_004685 | MTMR6 | frameshift_inserti  on | exon3 | c.239dupT | p.F80fs | . | . |
| CTC1 | 13 | 30062082 | GA | G | 0.232 | NM_015233 | MTUS2 | frameshift_deleti  on | exon4 | c.383delA | p.E128fs | . | . |
| CTC1 | 13 | 32972434 | CA | C | 0.5 | NM_000059 | BRCA2 | frameshift_deleti  on | exon27 | c.9785delA | p.Q3262fs | . | . |
| CTC1 | 13 | 34410270 | TG | T | 0.5 | NM_002915 | RFC3 | frameshift_deleti  on | exon9 | c.910delG | p.D304fs | . | . |
| CTC1 | 13 | 37453594 | C | CT | 0.188 | NM_001127217 | SMAD9 | frameshift_inserti  on | exon2 | c.232_233insA | p.R78fs | . | . |
| CTC1 | 13 | 37679278 | TTGG | T | 0.217 | NM_145203 | CSNK1A1L | nonframeshift_de  letion | exon1 | c.113_115del | p.38_39del | . | . |
| CTC1 | 13 | 42352122 | TG | T | 0.429 | NM_001009814 | VWA8 | frameshift_deleti  on | exon20 | c.2347delC | p.Q783fs | . | . |
| CTC1 | 13 | 42877324 | CA | C | 0.833 | NM_016248 | AKAP11 | frameshift_deleti  on | exon8 | c.4443delA | p.P1481fs | . | . |
| CTC1 | 13 | 46170718 | CCCCAG  ATACTCT TCCTCCT | C | 0.327 | NM_182542 | FAM194B | frameshift_deleti on | exon3 | c.404_422del | p.E135fs | . | . |
| CTC1 | 13 | 46170719 | CCCAGA  TACTCTT CCTCCT | C | 0.826 | NM_182542 | FAM194B | nonframeshift_de letion | exon3 | c.404_421del | p.135_141del | . | . |
| CTC1 | 13 | 46276987 | TC | T | 0.75 | NM_152719 | SPERT | frameshift_deleti  on | exon2 | c.154delC | p.Q52fs | . | . |
| CTC1 | 13 | 46701778 | AT | A | 0.492 | NM_002298 | LCP1 | frameshift_deleti  on | exon16 | c.1831delA | p.M611fs | . | . |
| CTC1 | 13 | 50586127 | TG | T | 0.462 | NM_213590 | TRIM13 | frameshift_deleti  on | exon2 | c.52delG | p.D18fs | . | . |
| CTC1 | 13 | 52440043 | C | CA | 0.145 | NM_031290 | CCDC70 | frameshift_inserti  on | exon2 | c.529_530insA | p.L177fs | . | . |
| CTC1 | 13 | 52536041 | GC | G | 0.312 | NM_000053 | ATP7B | frameshift_deleti  on | exon6 | c.1877delG | p.G626fs | . | . |
| CTC1 | 13 | 58206964 | TG | T | 1 | NM_001040429 | PCDH17 | frameshift_deleti  on | exon1 | c.285delG | p.L95fs | . | . |
| CTC1 | 13 | 76395495 | CT | C | 0.385 | NM_015842 | LMO7 | frameshift_deleti  on | exon11 | c.1692delT | p.T564fs | . | . |
| CTC1 | 13 | 79929447 | AT | A | 0.767 | NM_001286631 | RBM26 | frameshift_deleti  on | exon12 | c.1776delA | p.I592fs | . | . |
| CTC1 | 13 | 79929449 | AT | A | 0.77 | NM_001286631 | RBM26 | frameshift_deleti  on | exon12 | c.1774delA | p.I592fs | . | . |
| CTC1 | 13 | 95822792 | CA | C | 0.278 | NM_001105515 | ABCC4 | stopgain | exon14 | c.1817delT | p.L606X | . | . |
| CTC1 | 13 | 99127118 | CAG | C | 0.167 | NM_001286649 | STK24 | frameshift_deleti on | exon4 | c.495_496del | p.F165fs | . | . |
| CTC1 | 13 | 103338708 | GT | G | 0.421 | NM_001010977 | METTL21C | frameshift_deleti  on | exon4 | c.467delA | p.N156fs | . | . |
| CTC1 | 13 | 103395122 | GC | G | 0.157 | NM_001146197 | CCDC168 | frameshift_deleti  on | exon4 | c.7924delG | p.A2642fs | . | . |
| CTC1 | 13 | 103395949 | CA | C | 0.205 | NM_001146197 | CCDC168 | frameshift_deleti  on | exon4 | c.7097delT | p.M2366fs | . | . |
| CTC1 | 13 | 103397355 | GT | G | 0.4 | NM_001146197 | CCDC168 | frameshift_deleti  on | exon4 | c.5691delA | p.K1897fs | . | . |
| CTC1 | 13 | 108862167 | GA | G | 0.591 | NM_001098268 | LIG4 | frameshift_deleti  on | exon2 | c.1449delT | p.F483fs | . | . |
| CTC1 | 13 | 111274613 | GA | G | 0.571 | NM_001242881 | CARKD | frameshift_deleti  on | exon2 | c.152delA | p.E51fs | . | . |
| CTC1 | 13 | 115090131 | CG | C | 0.454 | NM_001164144 | CHAMP1 | frameshift_deleti  on | exon3 | c.815delG | p.R272fs | . | . |
| CTC1 | 13 | 115090299 | TCAG | T | 0.286 | NM_001164144 | CHAMP1 | nonframeshift_de  letion | exon3 | c.983_985del | p.328_329del | . | . |
| CTC1 | 14 | 20482972 | T | TA | 0.278 | NM_001004712 | OR4K14 | frameshift_inserti  on | exon1 | c.380dupT | p.I127fs | . | . |
| CTC1 | 14 | 20502910 | CT | C | 1 | NM_001004714 | OR4K13 | frameshift_deleti  on | exon1 | c.7delA | p.R3fs | . | . |
| CTC1 | 14 | 21238637 | TC | T | 0.359 | NM_022360 | EDDM3B | frameshift_deleti  on | exon2 | c.329delC | p.S110fs | . | . |
| CTC1 | 14 | 21859208 | GC | G | 0.646 | NM_001170629 | CHD8 | frameshift_deleti  on | exon36 | c.7079delG | p.R2360fs | . | . |
| CTC1 | 14 | 22038070 | A | AG | 0.778 | NM_001005465 | OR10G3 | frameshift_inserti  on | exon1 | c.805dupC | p.L269fs | . | . |
| CTC1 | 14 | 23315101 | CG | C | 0.471 | NM_004995 | MMP14 | frameshift_deleti  on | exon10 | c.1603delG | p.G535fs | . | . |
| CTC1 | 14 | 23456502 | TG | T | 0.39 | NM_001130708 | C14orf93 | frameshift_deleti  on | exon7 | c.1538delC | p.P513fs | . | . |
| CTC1 | 14 | 23468198 | GGA | G | 1 | NM_001130708 | C14orf93 | frameshift_deleti  on | exon2 | c.33_34del | p.P11fs | . | . |
| CTC1 | 14 | 23468200 | AG | A | 1 | NM_001130708 | C14orf93 | frameshift_deleti  on | exon2 | c.32delC | p.P11fs | . | . |
| CTC1 | 14 | 23548783 | A | AGAAC  GT | 1 | NM_001164815 | ACIN1 | nonframeshift_in  sertion | exon5 | c.1814_1815ins  ACGTTC | p.S605delinsSR  S | . | . |
| CTC1 | 14 | 23549019 | T | TC | 0.214 | NM_001164815 | ACIN1 | frameshift_inserti  on | exon5 | c.1578_1579ins  G | p.S527fs | . | . |
| CTC1 | 14 | 23855270 | TC | T | 0.444 | NM_002471 | MYH6 | frameshift_deleti  on | exon34 | c.5029delG | p.E1677fs | . | . |
| CTC1 | 14 | 23944767 | GT | G | 0.368 | NM_001042635 | NGDN | frameshift_deleti  on | exon5 | c.284delT | p.V95fs | . | . |
| CTC1 | 14 | 24003040 | CT | C | 0.533 | NM_033400 | ZFHX2 | frameshift_deleti  on | exon2 | c.1494delA | p.G498fs | . | . |
| CTC1 | 14 | 24646406 | G | GGAA | 1 | NM_001048205 | REC8 | nonframeshift_in  sertion | exon8 | c.681_682insG  AA | p.E227delinsEE | . | . |

| CTC1 | 14 | 24769865 | GA | G | 0.5 | NM_001286367 | NOP9 | frameshift_deleti  on | exon2 | c.500delA | p.E167fs | . | . |
| --- | --- | --- | --- | --- | --- | --- | --- | --- | --- | --- | --- | --- | --- |
| CTC1 | 14 | 24799154 | CA | C | 0.875 | NM_001198568 | ADCY4 | frameshift_deleti  on | exon8 | c.1128delT | p.C376fs | . | . |
| CTC1 | 14 | 24803756 | CCAG | C | 0.119 | NM_001198568 | ADCY4 | nonframeshift_de  letion | exon1 | c.100_102del | p.34_34del | . | . |
| CTC1 | 14 | 33291417 | ATT | A | 0.267 | NM_004274 | AKAP6 | frameshift_deleti  on | exon13 | c.4399_4400del | p.F1467fs | . | . |
| CTC1 | 14 | 44976127 | AT | A | 0.377 | NM_032135 | FSCB | frameshift_deleti  on | exon1 | c.63delA | p.K21fs | . | . |
| CTC1 | 14 | 45414924 | CAG | C | 0.298 | NM_017658 | KLHL28 | frameshift_deleti  on | exon2 | c.206_207del | p.S69fs | . | . |
| CTC1 | 14 | 45414925 | AG | A | 0.237 | NM_017658 | KLHL28 | frameshift_deleti  on | exon2 | c.206delC | p.S69fs | . | . |
| CTC1 | 14 | 51716243 | GT | G | 0.75 | NM_030755 | TMX1 | frameshift_deleti  on | exon6 | c.548delT | p.V183fs | . | . |
| CTC1 | 14 | 55169002 | GT | G | 0.231 | NM_001161576 | SAMD4A | frameshift_deleti  on | exon2 | c.420delT | p.R140fs | . | . |
| CTC1 | 14 | 55433268 | AT | A | 0.5 | NM_001008396 | WDHD1 | frameshift_deleti  on | exon17 | c.1865delA | p.N622fs | . | . |
| CTC1 | 14 | 55852783 | CT | C | 0.314 | NM_014924 | ATG14 | frameshift_deleti  on | exon5 | c.440delA | p.K147fs | . | . |
| CTC1 | 14 | 56078899 | CA | C | 0.362 | NM_001079521 | KTN1 | frameshift_deleti  on | exon2 | c.134delA | p.Q45fs | . | . |
| CTC1 | 14 | 57710975 | GTC | G | 0.186 | NM_006544 | EXOC5 | frameshift_deleti  on | exon4 | c.371_372del | p.R124fs | . | . |
| CTC1 | 14 | 58831659 | TG | T | 0.263 | NM_002892 | ARID4A | frameshift_deleti  on | exon20 | c.2853delG | p.L951fs | . | . |
| CTC1 | 14 | 58941401 | CA | C | 0.353 | NM_001244193 | KIAA0586 | frameshift_deleti  on | exon16 | c.2292delA | p.T764fs | . | . |
| CTC1 | 14 | 59108407 | CT | C | 0.857 | NM_001079520 | DACT1 | frameshift_deleti  on | exon3 | c.577delT | p.F193fs | . | . |
| CTC1 | 14 | 59112382 | G | GA | 0.846 | NM_001079520 | DACT1 | frameshift_inserti  on | exon4 | c.931dupA | p.Q310fs | . | . |
| CTC1 | 14 | 61190562 | GGCC | G | 0.127 | NM_017420 | SIX4 | nonframeshift_de  letion | exon1 | c.228_230del | p.76_77del | . | . |
| CTC1 | 14 | 64461916 | AT | A | 0.583 | NM_015180 | SYNE2 | frameshift_deleti  on | exon23 | c.2937delT | p.H979fs | . | . |
| CTC1 | 14 | 64464997 | AG | A | 0.209 | NM_015180 | SYNE2 | frameshift_deleti  on | exon26 | c.3305delG | p.S1102fs | . | . |
| CTC1 | 14 | 64473918 | AC | A | 0.281 | NM_015180 | SYNE2 | frameshift_deleti  on | exon31 | c.4556delC | p.T1519fs | . | . |
| CTC1 | 14 | 64604595 | GA | G | 0.5 | NM_015180 | SYNE2 | frameshift_deleti  on | exon79 | c.14738delA | p.E4913fs | . | . |
| CTC1 | 14 | 65482382 | C | CT | 0.3 | NM_002028 | FNTB | frameshift_inserti  on | exon4 | c.323dupT | p.L108fs | . | . |
| CTC1 | 14 | 70490078 | G | GA | 0.19 | NM_001034852 | SMOC1 | frameshift_inserti  on | exon11 | c.1205_1206ins  A | p.R402fs | . | . |
| CTC1 | 14 | 70517783 | CA | C | 0.261 | NM_182936 | SLC8A3 | frameshift_deleti  on | exon3 | c.143delT | p.L48fs | . | . |
| CTC1 | 14 | 73574601 | AT | A | 0.25 | NM_021239 | RBM25 | frameshift_deleti  on | exon13 | c.1536delT | p.Y512fs | . | . |
| CTC1 | 14 | 73743719 | GC | G | 0.5 | NM_001005745 | NUMB | frameshift_deleti  on | exon11 | c.1345delG | p.A449fs | . | . |
| CTC1 | 14 | 74968205 | CT | C | 0.353 | NM_000428 | LTBP2 | frameshift_deleti  on | exon35 | c.5258delA | p.E1753fs | . | . |
| CTC1 | 14 | 75373749 | TG | T | 0.488 | NM_031464 | RPS6KL1 | frameshift_deleti  on | exon11 | c.1617delC | p.F539fs | . | . |
| CTC1 | 14 | 75416166 | TC | T | 0.386 | NM_001207012 | PGF | frameshift_deleti  on | exon3 | c.208delG | p.E70fs | . | . |
| CTC1 | 14 | 76238161 | CA | C | 0.357 | NM_015072 | TTLL5 | frameshift_deleti  on | exon21 | c.2101delA | p.S701fs | . | . |
| CTC1 | 14 | 76437922 | A | AT | 0.431 | NM_003239 | TGFB3 | frameshift_inserti  on | exon2 | c.491dupA | p.N164fs | . | . |
| CTC1 | 14 | 77270220 | GT | G | 0.375 | NM_015305 | ANGEL1 | frameshift_deleti  on | exon6 | c.1415delA | p.Y472fs | . | . |
| CTC1 | 14 | 79175635 | TA | T | 0.207 | NM_004796 | NRXN3 | frameshift_deleti  on | exon4 | c.179delA | p.Y60fs | . | . |
| CTC1 | 14 | 80158553 | GA | G | 0.533 | NM_001105250 | NRXN3 | frameshift_deleti  on | exon4 | c.640delA | p.K214fs | . | . |
| CTC1 | 14 | 88938601 | GC | G | 0.312 | NM_007039 | PTPN21 | frameshift_deleti  on | exon15 | c.2857delG | p.A953fs | . | . |
| CTC1 | 14 | 91444771 | CT | C | 0.958 | NM_004755 | RPS6KA5 | frameshift_deleti  on | exon3 | c.272delA | p.K91fs | . | . |
| CTC1 | 14 | 91779674 | TG | T | 0.227 | NM_001080414 | CCDC88C | frameshift_deleti  on | exon15 | c.2485delC | p.Q829fs | . | . |
| CTC1 | 14 | 93125640 | G | GCCA | 0.154 | NM_024832 | RIN3 | nonframeshift_in  sertion | exon7 | c.2161_2162ins  CCA | p.A721delinsAT | . | . |
| CTC1 | 14 | 93397758 | AGAG | A | 0.109 | NM_001275 | CHGA | nonframeshift_de  letion | exon6 | c.520_522del | p.174_174del | . | . |
| CTC1 | 14 | 93727958 | TA | T | 1 | NM_001289133 | BTBD7 | frameshift_deleti  on | exon3 | c.383delT | p.I128fs | . | . |
| CTC1 | 14 | 94069746 | A | AC | 0.35 | NM_020818 | UNC79 | frameshift_inserti  on | exon26 | c.3206dupC | p.T1069fs | . | . |
| CTC1 | 14 | 94528665 | GC | G | 0.368 | NM_020414 | DDX24 | frameshift_deleti  on | exon3 | c.1020delG | p.G340fs | . | . |
| CTC1 | 14 | 95562809 | AT | A | 0.237 | NM_001195573 | DICER1 | frameshift_deleti  on | exon22 | c.4447delA | p.M1483fs | . | . |
| CTC1 | 14 | 99929840 | AT | A | 0.233 | NM_032233 | SETD3 | frameshift_deleti  on | exon3 | c.178delA | p.I60fs | . | . |
| CTC1 | 14 | 101004720 | CG | C | 0.25 | NM_001159531 | BEGAIN | frameshift_deleti  on | exon6 | c.1367delC | p.P456fs | . | . |
| CTC1 | 14 | 101347556 | AG | A | 0.474 | NM_001134888 | RTL1 | frameshift_deleti  on | exon1 | c.3569delC | p.P1190fs | . | . |
| CTC1 | 14 | 102356620 | G | GA | 0.455 | NM_002719 | PPP2R5C | frameshift_inserti  on | exon6 | c.677dupA | p.E226fs | . | . |
| CTC1 | 14 | 103406215 | CT | C | 0.885 | NM_006035 | CDC42BPB | frameshift_deleti  on | exon33 | c.4660delA | p.R1554fs | . | . |
| CTC1 | 14 | 103406569 | GC | G | 0.37 | NM_006035 | CDC42BPB | frameshift_deleti  on | exon31 | c.4500delG | p.L1500fs | . | . |
| CTC1 | 14 | 103410425 | TG | T | 0.56 | NM_006035 | CDC42BPB | frameshift_deleti  on | exon30 | c.4210delC | p.Q1404fs | . | . |
| CTC1 | 14 | 105055118 | TGACGG  GCAG | T | 1 | NM_001008404 | C14orf180 | frameshift_deleti  on | exon5 | c.482_819del | p.*161fs | . | . |
| CTC1 | 14 | 105177455 | AC | A | 1 | NM_001031714 | INF2 | frameshift_deleti  on | exon15 | c.2351delC | p.T784fs | . | . |
| CTC1 | 14 | 105410818 | AT | A | 1 | NM_138420 | AHNAK2 | frameshift_deleti  on | exon7 | c.10969delA | p.M3657fs | . | . |
| CTC1 | 14 | 105412703 | C | CA | 0.3 | NM_138420 | AHNAK2 | stopgain | exon7 | c.9084dupT | p.E3029_P3030  delinsX | . | . |
| CTC1 | 14 | 105412809 | TG | T | 0.333 | NM_138420 | AHNAK2 | frameshift_deleti  on | exon7 | c.8978delC | p.P2993fs | . | . |
| CTC1 | 14 | 105957456 | GT | G | 0.409 | NM_001134877 | C14orf80 | frameshift_deleti  on | exon2 | c.18delT | p.G6fs | . | . |

| CTC1 | 15 | 23579214 | AGGATG  GGTAGG GAGGTG | A | 0.571 | NM_001271664 | LOC440243 | nonframeshift_de letion | exon1 | c.57_74del | p.19_25del | . | . |
| --- | --- | --- | --- | --- | --- | --- | --- | --- | --- | --- | --- | --- | --- |
| CTC1 | 15 | 29346833 | GC | G | 0.5 | NM_001130414 | APBA2 | frameshift_deleti  on | exon3 | c.747delC | p.S249fs | . | . |
| CTC1 | 15 | 30377033 | GA | G | 0.663 | NM_001282472 | GOLGA8J | frameshift_deleti  on | exon2 | c.109delA | p.K37fs | . | . |
| CTC1 | 15 | 31197859 | TG | T | 1 | NM_001146094 | FAN1 | frameshift_deleti  on | exon2 | c.994delG | p.A332fs | . | . |
| CTC1 | 15 | 31253221 | ACCT | A | 0.218 | NM_017762 | MTMR10 | nonframeshift_de  letion | exon7 | c.618_620del | p.206_207del | . | . |
| CTC1 | 15 | 32450616 | TC | T | 0.232 | NM_000746 | CHRNA7 | frameshift_deleti  on | exon7 | c.603delC | p.I201fs | . | . |
| CTC1 | 15 | 32926145 | GA | G | 0.358 | NM_001286479 | ARHGAP11A | frameshift_deleti  on | exon10 | c.681delA | p.G227fs | . | . |
| CTC1 | 15 | 33381122 | CTT | C | 0.622 | NM_001277313 | FMN1 | frameshift_deleti  on | exon5 | c.1919_1920del | p.K640fs | . | . |
| CTC1 | 15 | 33926894 | TG | T | 0.191 | NM_001036 | RYR3 | frameshift_deleti  on | exon25 | c.3136delG | p.G1046fs | . | . |
| CTC1 | 15 | 34112962 | TCA | T | 0.8 | NM_001243996 | RYR3 | frameshift_deleti  on | exon77 | c.10750_10751  del | p.Q3584fs | . | . |
| CTC1 | 15 | 34396404 | GA | G | 0.526 | NM_152595 | PGBD4 | frameshift_deleti  on | exon1 | c.1673delA | p.E558fs | . | . |
| CTC1 | 15 | 34648751 | GA | G | 0.286 | NM_001284293 | NUTM1 | frameshift_deleti  on | exon7 | c.2513delA | p.E838fs | . | . |
| CTC1 | 15 | 38591742 | CA | C | 0.35 | NM_152594 | SPRED1 | frameshift_deleti  on | exon2 | c.202delA | p.K68fs | . | . |
| CTC1 | 15 | 39874451 | A | AG | 0.545 | NM_003246 | THBS1 | frameshift_inserti  on | exon3 | c.126dupG | p.K42fs | . | . |
| CTC1 | 15 | 40861630 | A | AT | 0.333 | NM_001286407 | RPUSD2 | frameshift_inserti  on | exon1 | c.94_95insT | p.K32fs | . | . |
| CTC1 | 15 | 40866029 | TA | T | 0.487 | NM_001286407 | RPUSD2 | frameshift_deleti  on | exon3 | c.1025delA | p.Y342fs | . | . |
| CTC1 | 15 | 40915811 | AT | A | 0.733 | NM_144508 | CASC5 | frameshift_deleti  on | exon10 | c.3350delT | p.I1117fs | . | . |
| CTC1 | 15 | 41812760 | A | AG | 0.909 | NM_015540 | RPAP1 | frameshift_inserti  on | exon22 | c.3623dupC | p.P1208fs | . | . |
| CTC1 | 15 | 42058347 | G | GA | 0.167 | NM_001080541 | MGA | frameshift_inserti  on | exon23 | c.7441dupA | p.R2480fs | . | . |
| CTC1 | 15 | 42731575 | CA | C | 0.357 | NM_001284306 | ZNF106 | stopgain | exon7 | c.1685delT | p.L562X | . | . |
| CTC1 | 15 | 42749212 | TTCTTCC | T | 0.132 | NM_022473 | ZNF106 | nonframeshift_de letion | exon1 | c.186_191del | p.62_64del | . | . |
| CTC1 | 15 | 42968112 | GA | G | 0.571 | NM_020759 | STARD9 | frameshift_deleti  on | exon20 | c.1814delA | p.D605fs | . | . |
| CTC1 | 15 | 42985407 | TG | T | 0.31 | NM_020759 | STARD9 | frameshift_deleti  on | exon23 | c.11632delG | p.G3878fs | . | . |
| CTC1 | 15 | 43027815 | AG | A | 0.5 | NM_138477 | CDAN1 | frameshift_deleti  on | exon4 | c.835delC | p.L279fs | . | . |
| CTC1 | 15 | 43069278 | GT | G | 1 | NM_173500 | TTBK2 | frameshift_deleti  on | exon12 | c.1359delA | p.K453fs | . | . |
| CTC1 | 15 | 43571832 | AG | A | 0.197 | NM_052955 | TGM7 | frameshift_deleti  on | exon10 | c.1668delC | p.D556fs | . | . |
| CTC1 | 15 | 43668404 | AC | A | 0.74 | NM_001286414 | TUBGCP4 | frameshift_deleti  on | exon2 | c.188delC | p.T63fs | . | . |
| CTC1 | 15 | 43708511 | CA | C | 0.274 | NM_001141979 | TP53BP1 | frameshift_deleti  on | exon22 | c.4784delT | p.L1595fs | . | . |
| CTC1 | 15 | 43828431 | AC | A | 0.264 | NM_001190214 | PPIP5K1 | frameshift_deleti  on | exon29 | c.3319delG | p.V1107fs | . | . |
| CTC1 | 15 | 44058135 | GC | G | 0.261 | NM_005313 | PDIA3 | frameshift_deleti  on | exon7 | c.771delC | p.G257fs | . | . |
| CTC1 | 15 | 45389881 | G | GT | 0.192 | NM_014080 | DUOX2 | frameshift_inserti  on | exon28 | c.3623dupA | p.H1208fs | . | . |
| CTC1 | 15 | 48063885 | TA | T | 0.5 | NM_020858 | SEMA6D | frameshift_deleti  on | exon17 | c.2940delA | p.L980fs | . | . |
| CTC1 | 15 | 48595055 | CA | C | 0.887 | NM_000338 | SLC12A1 | frameshift_deleti  on | exon27 | c.3274delA | p.K1092fs | . | . |
| CTC1 | 15 | 49149953 | GC | G | 0.293 | NM_203349 | SHC4 | frameshift_deleti  on | exon7 | c.1005delG | p.G335fs | . | . |
| CTC1 | 15 | 50535423 | AT | A | 0.464 | NM_002112 | HDC | frameshift_deleti  on | exon11 | c.1158delA | p.K386fs | . | . |
| CTC1 | 15 | 50884240 | G | GC | 0.4 | NM_017672 | TRPM7 | frameshift_inserti  on | exon26 | c.4191_4192ins  G | p.P1398fs | . | . |
| CTC1 | 15 | 51770507 | GC | G | 0.429 | NM_001174117 | DMXL2 | frameshift_deleti  on | exon24 | c.4885delG | p.A1629fs | . | . |
| CTC1 | 15 | 51828576 | CT | C | 0.27 | NM_001174116 | DMXL2 | frameshift_deleti  on | exon12 | c.2100delA | p.K700fs | . | . |
| CTC1 | 15 | 52356583 | GA | G | 0.75 | NM_002748 | MAPK6 | frameshift_deleti  on | exon6 | c.1553delA | p.E518fs | . | . |
| CTC1 | 15 | 52418175 | CA | C | 0.6 | NM_006578 | GNB5 | frameshift_deleti  on | exon9 | c.852delT | p.F284fs | . | . |
| CTC1 | 15 | 52537575 | CT | C | 0.529 | NM_018728 | MYO5C | frameshift_deleti  on | exon18 | c.2153delA | p.K718fs | . | . |
| CTC1 | 15 | 54306395 | TA | T | 0.19 | NM_001080534 | UNC13C | frameshift_deleti  on | exon1 | c.1296delA | p.I432fs | . | . |
| CTC1 | 15 | 54592568 | TG | T | 0.273 | NM_001080534 | UNC13C | frameshift_deleti  on | exon11 | c.4266delG | p.M1422fs | . | . |
| CTC1 | 15 | 55790024 | G | GA | 0.625 | NM_001033559 | DYX1C1 | frameshift_inserti  on | exon3 | c.156dupT | p.L53fs | . | . |
| CTC1 | 15 | 57543582 | AC | A | 0.718 | NM_207040 | TCF12 | frameshift_deleti  on | exon7 | c.640delC | p.P214fs | . | . |
| CTC1 | 15 | 59179702 | CT | C | 1 | NM_001013843 | SLTM | frameshift_deleti  on | exon18 | c.2358delA | p.K786fs | . | . |
| CTC1 | 15 | 60768330 | GA | G | 0.367 | NM_001276385 | NARG2 | frameshift_deleti  on | exon3 | c.77delT | p.F26fs | . | . |
| CTC1 | 15 | 62209628 | TC | T | 0.278 | NM_017684 | VPS13C | frameshift_deleti  on | exon58 | c.7837delG | p.E2613fs | . | . |
| CTC1 | 15 | 62999420 | CT | C | 0.714 | NM_015059 | TLN2 | frameshift_deleti  on | exon17 | c.2141delT | p.L714fs | . | . |
| CTC1 | 15 | 63008617 | GC | G | 0.75 | NM_015059 | TLN2 | frameshift_deleti  on | exon20 | c.2723delC | p.A908fs | . | . |
| CTC1 | 15 | 63068976 | A | AT | 0.75 | NM_015059 | TLN2 | frameshift_inserti  on | exon40 | c.5382dupT | p.H1794fs | . | . |
| CTC1 | 15 | 63964721 | CG | C | 0.438 | NM_003922 | HERC1 | frameshift_deleti  on | exon39 | c.8018delC | p.P2673fs | . | . |
| CTC1 | 15 | 65236853 | GA | G | 0.299 | NM_182703 | ANKDD1A | frameshift_deleti  on | exon12 | c.1071delA | p.G357fs | . | . |
| CTC1 | 15 | 65392002 | AG | A | 0.408 | NM_001163692 | UBAP1L | frameshift_deleti  on | exon3 | c.749delC | p.P250fs | . | . |
| CTC1 | 15 | 65968785 | TC | T | 0.45 | NM_005848 | DENND4A | frameshift_deleti  on | exon23 | c.4234delG | p.E1412fs | . | . |
| CTC1 | 15 | 69076841 | G | GC | 0.6 | NM_006305 | ANP32A | frameshift_inserti  on | exon4 | c.420dupG | p.Q141fs | . | . |
| CTC1 | 15 | 69548641 | GC | G | 0.364 | NM_015554 | GLCE | frameshift_deleti  on | exon3 | c.497delC | p.A166fs | . | . |

| CTC1 | 15 | 70358456 | TGGG | T | 0.385 | NM_001282982 | TLE3 | nonframeshift_de  letion | exon5 | c.303_305del | p.101_102del | . | . |
| --- | --- | --- | --- | --- | --- | --- | --- | --- | --- | --- | --- | --- | --- |
| CTC1 | 15 | 71276480 | GCAA | G | 1 | NM_001199017 | LRRC49 | nonframeshift_de  letion | exon11 | c.1069_1071del | p.357_357del | . | . |
| CTC1 | 15 | 73002055 | AT | A | 0.8 | NM_033028 | BBS4 | frameshift_deleti  on | exon3 | c.92delT | p.I31fs | . | . |
| CTC1 | 15 | 74708153 | GA | G | 0.5 | NM_001146029 | SEMA7A | frameshift_deleti  on | exon7 | c.932delT | p.F311fs | . | . |
| CTC1 | 15 | 74751190 | GC | G | 1 | NM_001286740 | UBL7 | frameshift_deleti  on | exon2 | c.18delG | p.W6fs | . | . |
| CTC1 | 15 | 74836289 | TCAGCA  G | T | 0.183 | NM_006465 | ARID3B | nonframeshift_de  letion | exon2 | c.13_18del | p.5_6del | . | . |
| CTC1 | 15 | 75320733 | CG | C | 0.667 | NM_021823 | PPCDC | frameshift_deleti  on | exon2 | c.75delG | p.T25fs | . | . |
| CTC1 | 15 | 75977701 | AG | A | 0.276 | NM_001897 | CSPG4 | frameshift_deleti  on | exon4 | c.4130delC | p.P1377fs | . | . |
| CTC1 | 15 | 77471993 | GTGC | G | 0.265 | NM_024776 | PEAK1 | nonframeshift_de  letion | exon5 | c.2273_2275del | p.758_759del | . | . |
| CTC1 | 15 | 78290543 | CA | C | 0.259 | NM_144572 | TBC1D2B | frameshift_deleti  on | exon13 | c.2850delT | p.P950fs | . | . |
| CTC1 | 15 | 79324604 | CG | C | 0.548 | NM_001145648 | RASGRF1 | frameshift_deleti  on | exon7 | c.1012delC | p.R338fs | . | . |
| CTC1 | 15 | 83499629 | ACCT | A | 0.286 | NM_001080435 | WHAMM | nonframeshift_de  letion | exon9 | c.1921_1923del | p.641_641del | . | . |
| CTC1 | 15 | 83499641 | TCCA | T | 0.312 | NM_001080435 | WHAMM | nonframeshift_de  letion | exon9 | c.1933_1935del | p.645_645del | . | . |
| CTC1 | 15 | 84908568 | AG | A | 0.778 | NM_001267536 | GOLGA6L4 | frameshift_deleti  on | exon6 | c.463delG | p.G155fs | . | . |
| CTC1 | 15 | 87097702 | GA | G | 0.5 | NM_152336 | AGBL1 | frameshift_deleti  on | exon20 | c.2791delA | p.M931fs | . | . |
| CTC1 | 15 | 91449228 | AG | A | 0.5 | NM_006122 | MAN2A2 | frameshift_deleti  on | exon4 | c.690delG | p.K230fs | . | . |
| CTC1 | 15 | 91455864 | C | CT | 0.188 | NM_006122 | MAN2A2 | frameshift_inserti  on | exon15 | c.2440dupT | p.A813fs | . | . |
| CTC1 | 15 | 91827393 | AC | A | 0.268 | NM_001167580 | SV2B | frameshift_deleti  on | exon10 | c.1198delC | p.P400fs | . | . |
| CTC1 | 15 | 100185837 | AAT | A | 0.933 | NM_001130926 | MEF2A | frameshift_deleti  on | exon3 | c.127_128del | p.I43fs | . | . |
| CTC1 | 15 | 100252709 | CCAGCA  G | C | 1 | NM_001130928 | MEF2A | nonframeshift_de  letion | exon8 | c.1024_1029del | p.342_343del | . | . |
| CTC1 | 15 | 100252709 | CCAG | C | 1 | NM_001130928 | MEF2A | nonframeshift_de  letion | exon8 | c.1024_1026del | p.342_342del | . | . |
| CTC1 | 15 | 102215917 | AC | A | 1 | NM_152334 | TARSL2 | frameshift_deleti  on | exon13 | c.1673delG | p.C558fs | . | . |
| CTC1 | 16 | 109308 | GA | G | 0.306 | NM_022450 | RHBDF1 | frameshift_deleti  on | exon16 | c.1932delT | p.F644fs | . | . |
| CTC1 | 16 | 418581 | G | GC | 0.257 | NM_006428 | MRPL28 | frameshift_inserti  on | exon4 | c.495dupG | p.L166fs | . | . |
| CTC1 | 16 | 845184 | CTGTG | C | 0.174 | NM_022092 | CHTF18 | frameshift_deleti  on | exon16 | c.2004_2007del | p.A668fs | . | . |
| CTC1 | 16 | 943050 | TC | T | 0.714 | NM_022773 | LMF1 | frameshift_deleti  on | exon5 | c.685delG | p.D229fs | . | . |
| CTC1 | 16 | 1568339 | TG | T | 0.343 | NM_014714 | IFT140 | frameshift_deleti  on | exon30 | c.4059delC | p.P1353fs | . | . |
| CTC1 | 16 | 1712585 | C | CT | 0.169 | NM_020825 | CRAMP1L | frameshift_inserti  on | exon11 | c.2509dupT | p.T836fs | . | . |
| CTC1 | 16 | 1889268 | AG | A | 0.273 | NM_001142398 | FAHD1 | frameshift_deleti  on | exon2 | c.643delG | p.V215fs | . | . |
| CTC1 | 16 | 2282560 | AC | A | 1 | NM_001288776 | E4F1 | frameshift_deleti  on | exon5 | c.714delC | p.H238fs | . | . |
| CTC1 | 16 | 2819118 | TTCC | T | 0.368 | NM_016333 | SRRM2 | nonframeshift_de  letion | exon12 | c.7855_7857del | p.2619_2619de  l | . | . |
| CTC1 | 16 | 3025747 | TG | T | 0.288 | NM_001258450 | PKMYT1 | frameshift_deleti  on | exon4 | c.237delC | p.P79fs | . | . |
| CTC1 | 16 | 3100073 | CG | C | 0.566 | NM_022468 | MMP25 | frameshift_deleti  on | exon3 | c.297delG | p.A99fs | . | . |
| CTC1 | 16 | 3786070 | CT | C | 0.942 | NM_001079846 | CREBBP | frameshift_deleti  on | exon27 | c.4580delA | p.K1527fs | . | . |
| CTC1 | 16 | 4016378 | T | TG | 0.3 | NM_001116 | ADCY9 | frameshift_inserti  on | exon11 | c.3459dupC | p.N1154fs | . | . |
| CTC1 | 16 | 4557994 | CG | C | 0.776 | NM_001286271 | HMOX2 | frameshift_deleti  on | exon3 | c.399delG | p.S133fs | . | . |
| CTC1 | 16 | 5058601 | CAG | C | 0.239 | NM_014692 | SEC14L5 | frameshift_deleti  on | exon14 | c.1753_1754del | p.S585fs | . | . |
| CTC1 | 16 | 10274031 | TG | T | 0.978 | NM_001134407 | GRIN2A | frameshift_deleti  on | exon2 | c.237delC | p.P79fs | . | . |
| CTC1 | 16 | 10995380 | AGG | A | 0.526 | NM_000246 | CIITA | frameshift_deleti  on | exon6 | c.447_448del | p.E149fs | . | . |
| CTC1 | 16 | 11065080 | CTA | C | 0.471 | NM_001243403 | CLEC16A | frameshift_deleti  on | exon5 | c.592_593del | p.Y198fs | . | . |
| CTC1 | 16 | 12172707 | CA | C | 0.429 | NM_032167 | SNX29 | frameshift_deleti  on | exon11 | c.1338delA | p.P446fs | . | . |
| CTC1 | 16 | 15122863 | GT | G | 0.7 | NM_001285450 | PDXDC1 | frameshift_deleti  on | exon14 | c.1265delT | p.V422fs | . | . |
| CTC1 | 16 | 15138209 | GT | G | 0.462 | NM_001270767 | NTAN1 | frameshift_deleti  on | exon3 | c.99delA | p.K33fs | . | . |
| CTC1 | 16 | 15715743 | T | TG | 0.51 | NM_001184998 | KIAA0430 | frameshift_inserti  on | exon12 | c.2485dupC | p.H829fs | . | . |
| CTC1 | 16 | 19049279 | GT | G | 0.391 | NM_001160364 | TMC7 | frameshift_deleti  on | exon8 | c.760delT | p.F254fs | . | . |
| CTC1 | 16 | 19056748 | A | AT | 0.3 | NM_001160364 | TMC7 | frameshift_inserti  on | exon11 | c.1212dupT | p.D404fs | . | . |
| CTC1 | 16 | 19725725 | CT | C | 0.881 | NM_001012991 | KNOP1 | frameshift_deleti  on | exon2 | c.632delA | p.K211fs | . | . |
| CTC1 | 16 | 20330975 | AC | A | 0.295 | NM_001007242 | GP2 | frameshift_deleti  on | exon5 | c.532delG | p.V178fs | . | . |
| CTC1 | 16 | 21261096 | A | AT | 0.455 | NM_145865 | ANKS4B | frameshift_inserti  on | exon2 | c.210dupT | p.H70fs | . | . |
| CTC1 | 16 | 23203821 | TG | T | 0.941 | NM_001039 | SCNN1G | frameshift_deleti  on | exon4 | c.768delG | p.L256fs | . | . |
| CTC1 | 16 | 24358058 | C | CT | 0.643 | NM_006539 | CACNG3 | frameshift_inserti  on | exon2 | c.216dupT | p.A72fs | . | . |
| CTC1 | 16 | 24573184 | AG | A | 0.763 | NM_006910 | RBBP6 | frameshift_deleti  on | exon10 | c.992delG | p.R331fs | . | . |
| CTC1 | 16 | 24788422 | AGCAGC  CACAGC | A | 0.111 | NM_014494 | TNRC6A | nonframeshift_de  letion | exon5 | c.333_344del | p.111_115del | . | . |
| CTC1 | 16 | 24802242 | CT | C | 0.237 | NM_014494 | TNRC6A | frameshift_deleti  on | exon6 | c.2280delT | p.T760fs | . | . |
| CTC1 | 16 | 27781273 | AT | A | 0.467 | NM_015202 | KIAA0556 | frameshift_deleti  on | exon21 | c.4068delT | p.D1356fs | . | . |
| CTC1 | 16 | 28844486 | CAG | C | 0.117 | NM_007245 | ATXN2L | frameshift_deleti  on | exon14 | c.1767_1768del | p.S589fs | . | . |
| CTC1 | 16 | 28854304 | AT | A | 0.558 | NM_003321 | TUFM | frameshift_deleti  on | exon10 | c.1359delA | p.K453fs | . | . |

| CTC1 | 16 | 28880592 | T | TA | 0.192 | NM_015503 | SH2B1 | frameshift_inserti  on | exon4 | c.1198dupA | p.L399fs | . | . |
| --- | --- | --- | --- | --- | --- | --- | --- | --- | --- | --- | --- | --- | --- |
| CTC1 | 16 | 28947851 | TC | T | 0.511 | NM_001178098 | CD19 | frameshift_deleti  on | exon7 | c.1015delC | p.P339fs | . | . |
| CTC1 | 16 | 29810484 | TC | T | 0.191 | NM_001256270 | KIF22 | frameshift_deleti  on | exon5 | c.535delC | p.H179fs | . | . |
| CTC1 | 16 | 30018164 | GC | G | 0.145 | NM_001282068 | DOC2A | frameshift_deleti  on | exon8 | c.819delG | p.L273fs | . | . |
| CTC1 | 16 | 30037125 | CT | C | 0.556 | NM_031478 | FAM57B | frameshift_deleti  on | exon4 | c.461delA | p.K154fs | . | . |
| CTC1 | 16 | 30078772 | G | GA | 0.571 | NM_001127617 | ALDOA | frameshift_inserti  on | exon3 | c.115dupA | p.G38fs | . | . |
| CTC1 | 16 | 30128101 | TCG | T | 0.188 | NM_001109891 | MAPK3 | frameshift_deleti  on | exon7 | c.894_895del | p.A298fs | . | . |
| CTC1 | 16 | 30495217 | TG | T | 0.358 | NM_001114380 | ITGAL | frameshift_deleti  on | exon6 | c.544delG | p.G182fs | . | . |
| CTC1 | 16 | 30537318 | TC | T | 0.667 | NM_024671 | ZNF768 | frameshift_deleti  on | exon2 | c.142delG | p.D48fs | . | . |
| CTC1 | 16 | 30740429 | GC | G | 0.587 | NM_006662 | SRCAP | frameshift_deleti  on | exon26 | c.5802delC | p.S1934fs | . | . |
| CTC1 | 16 | 30997839 | CA | C | 0.351 | NM_001142777 | HSD3B7 | frameshift_deleti  on | exon4 | c.419delA | p.H140fs | . | . |
| CTC1 | 16 | 31120700 | CT | C | 1 | NM_001122957 | BCKDK | frameshift_deleti  on | exon2 | c.157delT | p.F53fs | . | . |
| CTC1 | 16 | 31926853 | GA | G | 0.361 | NM_003414 | ZNF267 | frameshift_deleti  on | exon4 | c.1284delA | p.R428fs | . | . |
| CTC1 | 16 | 47462768 | AT | A | 1 | NM_030790 | ITFG1 | frameshift_deleti  on | exon6 | c.601delA | p.M201fs | . | . |
| CTC1 | 16 | 48226504 | AC | A | 0.324 | NM_033151 | ABCC11 | frameshift_deleti  on | exon19 | c.2632delG | p.V878fs | . | . |
| CTC1 | 16 | 51173145 | AC | A | 0.322 | NM_001127892 | SALL1 | frameshift_deleti  on | exon2 | c.2696delG | p.G899fs | . | . |
| CTC1 | 16 | 52473503 | CTGT | C | 0.632 | NM_001080430 | TOX3 | nonframeshift_de  letion | exon7 | c.1362_1364del | p.454_455del | . | . |
| CTC1 | 16 | 52473512 | TTGC | T | 0.848 | NM_001080430 | TOX3 | nonframeshift_de  letion | exon7 | c.1353_1355del | p.451_452del | . | . |
| CTC1 | 16 | 57080538 | TC | T | 0.324 | NM_032206 | NLRC5 | frameshift_deleti  on | exon21 | c.3324delC | p.I1108fs | . | . |
| CTC1 | 16 | 57416109 | G | GA | 0.36 | NM_002996 | CX3CL1 | frameshift_inserti  on | exon3 | c.360dupA | p.G120fs | . | . |
| CTC1 | 16 | 57503647 | AC | A | 0.268 | NM_032940 | POLR2C | frameshift_deleti  on | exon6 | c.414delC | p.D138fs | . | . |
| CTC1 | 16 | 57507644 | AG | A | 0.185 | NM_018110 | DOK4 | stopgain | exon8 | c.742delC | p.L248X | . | . |
| CTC1 | 16 | 57714475 | GT | G | 0.231 | NM_170776 | GPR97 | frameshift_deleti on | exon8 | c.828delT | p.C276fs | . | . |
| CTC1 | 16 | 57804461 | AG | A | 0.643 | NM_001130099 | KIFC3 | stopgain | exon6 | c.499delC | p.L167X | . | . |
| CTC1 | 16 | 57805569 | GT | G | 0.194 | NM_001130100 | KIFC3 | frameshift_deleti on | exon5 | c.410delA | p.D137fs | . | . |
| CTC1 | 16 | 57847634 | A | ATGCA  GGTG | 0.846 | NM_001278081 | LOC388282 | frameshift_inserti  on | exon2 | c.137_138insTG  CAGGTG | p.Q46fs | . | . |
| CTC1 | 16 | 58314425 | TG | T | 0.314 | NM_001080492 | PRSS54 | frameshift_deleti  on | exon7 | c.890delC | p.T297fs | . | . |
| CTC1 | 16 | 58614616 | CA | C | 0.427 | NM_001265612 | CNOT1 | frameshift_deleti  on | exon12 | c.1263delT | p.F421fs | . | . |
| CTC1 | 16 | 58621749 | TC | T | 0.184 | NM_001265612 | CNOT1 | frameshift_deleti  on | exon4 | c.243delG | p.L81fs | . | . |
| CTC1 | 16 | 66642276 | C | CT | 0.162 | NM_181553 | CMTM3 | frameshift_inserti  on | exon2 | c.213dupT | p.P71fs | . | . |
| CTC1 | 16 | 66946614 | TTC | T | 0.268 | NM_001204746 | CDH16 | frameshift_deleti  on | exon9 | c.942_943del | p.Q314fs | . | . |
| CTC1 | 16 | 67202989 | C | CA | 0.466 | NM_001040667 | HSF4 | frameshift_inserti  on | exon13 | c.1240dupA | p.D413fs | . | . |
| CTC1 | 16 | 67221410 | TG | T | 0.556 | NM_178516 | EXOC3L1 | frameshift_deleti  on | exon5 | c.757delC | p.Q253fs | . | . |
| CTC1 | 16 | 67231939 | AC | A | 0.512 | NM_001950 | E2F4 | frameshift_deleti  on | exon10 | c.1156delC | p.P386fs | . | . |
| CTC1 | 16 | 67690387 | TG | T | 0.396 | NM_001013838 | RLTPR | frameshift_deleti  on | exon35 | c.3876delG | p.L1292fs | . | . |
| CTC1 | 16 | 68308863 | G | GA | 0.93 | NM_003983 | SLC7A6 | frameshift_inserti  on | exon3 | c.235dupA | p.G78fs | . | . |
| CTC1 | 16 | 68344680 | CG | C | 0.5 | NM_032178 | SLC7A6OS | frameshift_deleti  on | exon1 | c.149delC | p.A50fs | . | . |
| CTC1 | 16 | 69353327 | AC | A | 0.317 | NM_013245 | VPS4A | frameshift_deleti  on | exon6 | c.502delC | p.P168fs | . | . |
| CTC1 | 16 | 69988262 | TG | T | 0.286 | NM_001136214 | CLEC18A | frameshift_deleti  on | exon4 | c.243delG | p.L81fs | . | . |
| CTC1 | 16 | 70172895 | C | CT | 0.353 | NM_017990 | PDPR | frameshift_inserti  on | exon11 | c.1285dupT | p.T428fs | . | . |
| CTC1 | 16 | 70506448 | AG | A | 0.375 | NM_145059 | FUK | frameshift_deleti  on | exon14 | c.1347delG | p.Q449fs | . | . |
| CTC1 | 16 | 70710831 | T | TG | 0.429 | NM_138383 | MTSS1L | frameshift_inserti  on | exon10 | c.782dupC | p.P261fs | . | . |
| CTC1 | 16 | 70894725 | CA | C | 0.528 | NM_001270974 | HYDIN | frameshift_deleti  on | exon70 | c.11856delT | p.F3952fs | . | . |
| CTC1 | 16 | 71319169 | GT | G | 0.5 | NM_001099642 | CMTR2 | frameshift_deleti  on | exon3 | c.654delA | p.G218fs | . | . |
| CTC1 | 16 | 71392703 | GC | G | 0.667 | NM_001740 | CALB2 | frameshift_deleti  on | exon1 | c.9delC | p.G3fs | . | . |
| CTC1 | 16 | 71689309 | TA | T | 0.8 | NM_001289003 | PHLPP2 | frameshift_deleti  on | exon16 | c.2217delT | p.D739fs | . | . |
| CTC1 | 16 | 72832482 | AC | A | 0.239 | NM_001164766 | ZFHX3 | frameshift_deleti  on | exon8 | c.1356delG | p.G452fs | . | . |
| CTC1 | 16 | 75240031 | TC | T | 1 | NM_001025200 | CTRB2 | frameshift_deleti  on | exon2 | c.112delG | p.E38fs | . | . |
| CTC1 | 16 | 75563914 | CA | C | 0.435 | NM_024533 | CHST5 | frameshift_deleti  on | exon3 | c.368delT | p.L123fs | . | . |
| CTC1 | 16 | 77401502 | CT | C | 0.667 | NM_199355 | ADAMTS18 | frameshift_deleti  on | exon4 | c.613delA | p.R205fs | . | . |
| CTC1 | 16 | 77910298 | GA | G | 0.308 | NM_020927 | VAT1L | frameshift_deleti  on | exon5 | c.755delA | p.D252fs | . | . |
| CTC1 | 16 | 81078409 | G | GT | 0.516 | NM_015251 | ATMIN | frameshift_inserti  on | exon4 | c.2307dupT | p.S769fs | . | . |
| CTC1 | 16 | 81242148 | GTT | G | 1 | NM_001076780 | PKD1L2 | frameshift_deleti  on | exon4 | c.706_707del | p.N236fs | . | . |
| CTC1 | 16 | 84126814 | GA | G | 0.468 | NM_003791 | MBTPS1 | frameshift_deleti  on | exon6 | c.824delT | p.F275fs | . | . |
| CTC1 | 16 | 84164849 | AG | A | 0.375 | NM_001146051 | HSDL1 | frameshift_deleti  on | exon3 | c.77delC | p.A26fs | . | . |
| CTC1 | 16 | 84188182 | GT | G | 0.947 | NM_178452 | DNAAF1 | frameshift_deleti  on | exon4 | c.354delT | p.G118fs | . | . |
| CTC1 | 16 | 84256432 | C | CG | 1 | NM_172347 | KCNG4 | frameshift_inserti  on | exon3 | c.950dupC | p.P317fs | . | . |
| CTC1 | 16 | 85100758 | AC | A | 0.515 | NM_001286565 | KIAA0513 | frameshift_deleti  on | exon2 | c.82delC | p.P28fs | . | . |

| CTC1 | 16 | 87448063 | GC | G | 0.234 | NM_015144 | ZCCHC14 | frameshift_deleti  on | exon10 | c.1148delG | p.S383fs | . | . |
| --- | --- | --- | --- | --- | --- | --- | --- | --- | --- | --- | --- | --- | --- |
| CTC1 | 16 | 88066729 | AC | A | 0.704 | NM_001173540 | BANP | frameshift_deleti  on | exon8 | c.980delC | p.T327fs | . | . |
| CTC1 | 16 | 88500133 | GA | G | 0.5 | NM_001127464 | ZNF469 | frameshift_deleti  on | exon2 | c.6172delA | p.R2058fs | . | . |
| CTC1 | 16 | 88780634 | AGGTGT  G | A | 1 | NM_001012759 | CTU2 | frameshift_deleti  on | exon10 | c.1097_1097del | p.R366fs | . | . |
| CTC1 | 16 | 88783449 | CA | C | 0.25 | NM_001142864 | PIEZO1 | frameshift_deleti  on | exon45 | c.6641delT | p.L2214fs | . | . |
| CTC1 | 16 | 88798835 | AC | A | 0.589 | NM_001142864 | PIEZO1 | frameshift_deleti  on | exon21 | c.2898delG | p.V966fs | . | . |
| CTC1 | 17 | 263285 | C | CT | 0.158 | NM_001013672 | C17orf97 | frameshift_inserti  on | exon2 | c.651_652insT | p.P217fs | . | . |
| CTC1 | 17 | 986779 | TG | T | 0.333 | NM_001092 | ABR | frameshift_deleti  on | exon4 | c.508delC | p.Q170fs | . | . |
| CTC1 | 17 | 1554996 | GC | G | 1 | NM_006445 | PRPF8 | frameshift_deleti  on | exon40 | c.6455delG | p.G2152fs | . | . |
| CTC1 | 17 | 1582972 | GC | G | 0.358 | NM_006445 | PRPF8 | frameshift_deleti  on | exon9 | c.1219delG | p.A407fs | . | . |
| CTC1 | 17 | 1670275 | GCCC | G | 0.583 | NM_002615 | SERPINF1 | nonframeshift_de  letion | exon2 | c.72_74del | p.24_25del | . | . |
| CTC1 | 17 | 1747938 | GC | G | 0.778 | NM_002945 | RPA1 | frameshift_deleti  on | exon4 | c.231delC | p.C77fs | . | . |
| CTC1 | 17 | 2203780 | AT | A | 0.556 | NM_017575 | SMG6 | frameshift_deleti  on | exon2 | c.266delA | p.N89fs | . | . |
| CTC1 | 17 | 2606645 | GC | G | 0.296 | NM_015229 | CLUH | frameshift_deleti  on | exon3 | c.327delG | p.G109fs | . | . |
| CTC1 | 17 | 2908704 | AC | A | 0.284 | NM_001100398 | RAP1GAP2 | frameshift_deleti  on | exon14 | c.1198delC | p.P400fs | . | . |
| CTC1 | 17 | 3477014 | GT | G | 1 | NM_080706 | TRPV1 | frameshift_deleti  on | exon12 | c.2015delA | p.Y672fs | . | . |
| CTC1 | 17 | 3495591 | CT | C | 0.333 | NM_080706 | TRPV1 | frameshift_deleti  on | exon1 | c.53delA | p.K18fs | . | . |
| CTC1 | 17 | 4586236 | CG | C | 0.424 | NM_014389 | PELP1 | frameshift_deleti  on | exon4 | c.581delC | p.P194fs | . | . |
| CTC1 | 17 | 4645710 | AT | A | 0.588 | NM_001136046 | ZMYND15 | frameshift_deleti  on | exon5 | c.1069delT | p.F357fs | . | . |
| CTC1 | 17 | 4936451 | AC | A | 0.279 | NM_001104577 | SLC52A1 | frameshift_deleti  on | exon5 | c.1147delG | p.V383fs | . | . |
| CTC1 | 17 | 5039116 | GC | G | 0.308 | NM_004505 | USP6 | stopgain | exon9 | c.558delC | p.C186X | . | . |
| CTC1 | 17 | 5442772 | C | CT | 0.28 | NM_001033053 | NLRP1 | frameshift_inserti on | exon7 | c.2832_2833ins A | p.G945fs | . | . |
| CTC1 | 17 | 6013050 | GC | G | 1 | NM_015253 | WSCD1 | frameshift_deleti  on | exon6 | c.974delC | p.A325fs | . | . |
| CTC1 | 17 | 6387013 | TC | T | 0.698 | NM_001165966 | PITPNM3 | frameshift_deleti  on | exon5 | c.302delG | p.G101fs | . | . |
| CTC1 | 17 | 6524277 | CT | C | 0.667 | NM_014804 | KIAA0753 | frameshift_deleti  on | exon7 | c.1145delA | p.K382fs | . | . |
| CTC1 | 17 | 6544519 | G | GA | 0.259 | NM_032731 | TXNDC17 | frameshift_inserti  on | exon1 | c.118dupA | p.G39fs | . | . |
| CTC1 | 17 | 6555546 | C | CG | 1 | NM_001105520 | C17orf100 | frameshift_inserti  on | exon1 | c.314dupG | p.R105fs | . | . |
| CTC1 | 17 | 6930082 | GA | G | 0.312 | NM_181844 | BCL6B | frameshift_deleti  on | exon7 | c.1114delA | p.K372fs | . | . |
| CTC1 | 17 | 7156293 | GT | G | 0.373 | NM_203414 | ELP5 | frameshift_deleti  on | exon3 | c.213delT | p.G71fs | . | . |
| CTC1 | 17 | 7339206 | TG | T | 0.854 | NM_178518 | TMEM102 | frameshift_deleti  on | exon2 | c.17delG | p.W6fs | . | . |
| CTC1 | 17 | 7640417 | C | CT | 0.5 | NM_020877 | DNAH2 | frameshift_inserti  on | exon7 | c.1012dupT | p.T337fs | . | . |
| CTC1 | 17 | 7750177 | TACCAC  CACCAC CACCAC CACCAC | T | 1 | NM_001080424 | KDM6B | nonframeshift_de letion | exon9 | c.753_779del | p.251_260del | . | . |
| CTC1 | 17 | 7750177 | T | TACCA  CCACC | 1 | NM_001080424 | KDM6B | nonframeshift_in  sertion | exon9 | c.752_753insAC  CACCACC | p.L251delinsLP  PP | . | . |
| CTC1 | 17 | 7804199 | AT | A | 0.426 | NM_001005271 | CHD3 | frameshift_deleti  on | exon19 | c.3186delT | p.N1062fs | . | . |
| CTC1 | 17 | 7918801 | CCG | C | 0.821 | NM_000180 | GUCY2D | frameshift_deleti  on | exon15 | c.2926_2927del | p.R976fs | . | . |
| CTC1 | 17 | 8092648 | GC | G | 0.667 | NM_017622 | C17orf59 | frameshift_deleti  on | exon1 | c.810delG | p.E270fs | . | . |
| CTC1 | 17 | 8448768 | CA | C | 1 | NM_005964 | MYH10 | frameshift_deleti  on | exon12 | c.1398delT | p.F466fs | . | . |
| CTC1 | 17 | 8449933 | TA | T | 0.833 | NM_005964 | MYH10 | frameshift_deleti  on | exon11 | c.1176delT | p.F392fs | . | . |
| CTC1 | 17 | 10350527 | GA | G | 0.667 | NM_017533 | MYH4 | frameshift_deleti  on | exon35 | c.4971delT | p.T1657fs | . | . |
| CTC1 | 17 | 10419333 | CCT | C | 0.308 | NM_005963 | MYH1 | frameshift_deleti  on | exon5 | c.413_414del | p.E138fs | . | . |
| CTC1 | 17 | 10448707 | TG | T | 1 | NM_001100112 | MYH2 | frameshift_deleti  on | exon5 | c.460delC | p.H154fs | . | . |
| CTC1 | 17 | 11593500 | A | AG | 0.6 | NM_001372 | DNAH9 | frameshift_inserti  on | exon20 | c.4361_4362ins  G | p.H1454fs | . | . |
| CTC1 | 17 | 12887952 | AC | A | 0.241 | NM_014859 | ARHGAP44 | frameshift_deleti  on | exon20 | c.2045delC | p.T682fs | . | . |
| CTC1 | 17 | 16326086 | GC | G | 1 | NM_016113 | TRPV2 | frameshift_deleti  on | exon4 | c.509delC | p.A170fs | . | . |
| CTC1 | 17 | 16335489 | GTGC | G | 0.25 | NM_016113 | TRPV2 | nonframeshift_de  letion | exon12 | c.1865_1867del | p.622_623del | . | . |
| CTC1 | 17 | 17039561 | CCAG | C | 1 | NM_015134 | MPRIP | nonframeshift_de  letion | exon6 | c.534_536del | p.178_179del | . | . |
| CTC1 | 17 | 17039561 | CCAGCA  G | C | 1 | NM_015134 | MPRIP | nonframeshift_de  letion | exon6 | c.534_539del | p.178_180del | . | . |
| CTC1 | 17 | 17039573 | GCAGC | G | 0.211 | NM_015134 | MPRIP | frameshift_deleti  on | exon6 | c.546_549del | p.S182fs | . | . |
| CTC1 | 17 | 17696469 | TG | T | 0.333 | NM_030665 | RAI1 | frameshift_deleti  on | exon3 | c.208delG | p.A70fs | . | . |
| CTC1 | 17 | 17723625 | T | TA | 0.562 | NM_004176 | SREBF1 | frameshift_inserti  on | exon2 | c.301_302insT | p.Q101fs | . | . |
| CTC1 | 17 | 18043870 | GC | G | 0.244 | NM_016239 | MYO15A | frameshift_deleti  on | exon19 | c.5252delC | p.A1751fs | . | . |
| CTC1 | 17 | 18047063 | A | AC | 0.17 | NM_016239 | MYO15A | frameshift_inserti  on | exon26 | c.6000dupC | p.H2000fs | . | . |
| CTC1 | 17 | 18653082 | CT | C | 0.375 | NM_001267585 | FBXW10 | frameshift_deleti  on | exon3 | c.719delT | p.L240fs | . | . |
| CTC1 | 17 | 18874685 | C | CGGT | 0.19 | NM_001039999 | FAM83G | nonframeshift_in  sertion | exon6 | c.2458_2459ins  ACC | p.R820delinsH  R | . | . |
| CTC1 | 17 | 26692224 | A | AG | 0.992 | NM_001080837 | SEBOX | frameshift_inserti  on | exon1 | c.27dupC | p.W10fs | . | . |
| CTC1 | 17 | 26851597 | GC | G | 0.579 | NM_003593 | FOXN1 | frameshift_deleti  on | exon2 | c.201delC | p.S67fs | . | . |

| CTC1 | 17 | 26862048 | AC | A | 0.92 | NM_003593 | FOXN1 | frameshift_deleti  on | exon7 | c.1460delC | p.T487fs | . | . |
| --- | --- | --- | --- | --- | --- | --- | --- | --- | --- | --- | --- | --- | --- |
| CTC1 | 17 | 26918752 | C | CA | 0.286 | NM_006461 | SPAG5 | frameshift_inserti  on | exon4 | c.1400_1401ins  T | p.Q467fs | . | . |
| CTC1 | 17 | 26960099 | A | AC | 0.179 | NM_014680 | KIAA0100 | frameshift_inserti  on | exon20 | c.3665_3666ins  G | p.H1222fs | . | . |
| CTC1 | 17 | 27028587 | AC | A | 0.37 | NM_003170 | SUPT6H | frameshift_deleti  on | exon37 | c.5126delC | p.T1709fs | . | . |
| CTC1 | 17 | 27030608 | AT | A | 0.676 | NM_152465 | PROCA1 | frameshift_deleti  on | exon4 | c.978delA | p.K326fs | . | . |
| CTC1 | 17 | 27308578 | TG | T | 0.281 | NM_001098635 | SEZ6 | frameshift_deleti  on | exon2 | c.534delC | p.S178fs | . | . |
| CTC1 | 17 | 27613995 | TG | T | 0.6 | NM_020772 | NUFIP2 | frameshift_deleti  on | exon2 | c.1016delC | p.P339fs | . | . |
| CTC1 | 17 | 28776688 | GC | G | 0.24 | NM_001199775 | CPD | frameshift_deleti  on | exon13 | c.2251delC | p.P751fs | . | . |
| CTC1 | 17 | 29162727 | AT | A | 1 | NM_024857 | ATAD5 | frameshift_deleti  on | exon2 | c.1629delT | p.D543fs | . | . |
| CTC1 | 17 | 29276354 | TA | T | 0.377 | NM_018404 | ADAP2 | frameshift_deleti  on | exon7 | c.708delA | p.L236fs | . | . |
| CTC1 | 17 | 32961845 | TGAG | T | 0.431 | NM_207313 | TMEM132E | nonframeshift_de  letion | exon8 | c.1447_1449del | p.483_483del | . | . |
| CTC1 | 17 | 33454195 | AG | A | 0.364 | NM_017559 | FNDC8 | frameshift_deleti  on | exon2 | c.345delG | p.E115fs | . | . |
| CTC1 | 17 | 33769078 | G | GA | 0.516 | NM_144682 | SLFN13 | frameshift_inserti  on | exon5 | c.1425dupT | p.L476fs | . | . |
| CTC1 | 17 | 33805120 | AT | A | 0.441 | NM_001195790 | SLFN12L | frameshift_deleti  on | exon3 | c.1177delA | p.I393fs | . | . |
| CTC1 | 17 | 34079833 | T | TC | 0.737 | NM_139285 | GAS2L2 | frameshift_inserti  on | exon1 | c.36dupG | p.T13fs | . | . |
| CTC1 | 17 | 35783619 | TG | T | 0.4 | NM_001166105 | TADA2A | frameshift_deleti  on | exon3 | c.37delG | p.D13fs | . | . |
| CTC1 | 17 | 39324229 | T | TGCAG  CAGGT | 0.214 | NM_033187 | KRTAP4-3 | nonframeshift_in  sertion | exon1 | c.195_196insCT  GACCACCTGC | p.R66delinsLTT  CCR | . | . |
| CTC1 | 17 | 39411670 | T | TACCT  GCTGC | 0.375 | NM_030975 | KRTAP9-9 | nonframeshift_in  sertion | exon1 | c.33_34insACC  TGCTGCAGGA | p.P11delinsPTC  CRT | . | . |
| CTC1 | 17 | 39538269 | AGCT | A | 0.375 | NM_021013 | KRT34 | nonframeshift_de  letion | exon1 | c.353_355del | p.118_119del | . | . |
| CTC1 | 17 | 39742943 | GC | G | 0.32 | NM_000526 | KRT14 | frameshift_deleti  on | exon1 | c.143delG | p.G48fs | . | . |
| CTC1 | 17 | 40343165 | GCC | G | 0.25 | NM_001142622 | GHDC | frameshift_deleti  on | exon6 | c.834_835del | p.G278fs | . | . |
| CTC1 | 17 | 40345559 | GGCA | G | 0.147 | NM_001142622 | GHDC | nonframeshift_de  letion | exon3 | c.38_40del | p.13_14del | . | . |
| CTC1 | 17 | 40453406 | AG | A | 0.252 | NM_001288719 | STAT5A | frameshift_deleti  on | exon8 | c.1014delG | p.Q338fs | . | . |
| CTC1 | 17 | 40468877 | G | GA | 0.4 | NM_003150 | STAT3 | frameshift_inserti  on | exon23 | c.2183_2184ins  T | p.R728fs | . | . |
| CTC1 | 17 | 41056014 | GC | G | 0.662 | NM_000151 | G6PC | frameshift_deleti  on | exon2 | c.298delC | p.P100fs | . | . |
| CTC1 | 17 | 41063181 | TG | T | 0.538 | NM_000151 | G6PC | frameshift_deleti  on | exon5 | c.813delG | p.L271fs | . | . |
| CTC1 | 17 | 41174210 | AGGC | A | 0.625 | NM_006373 | VAT1 | nonframeshift_de  letion | exon1 | c.127_129del | p.43_43del | . | . |
| CTC1 | 17 | 42226329 | C | CA | 0.226 | NM_001171251 | C17orf53 | frameshift_inserti  on | exon3 | c.1159dupA | p.P386fs | . | . |
| CTC1 | 17 | 42475059 | GA | G | 0.286 | NM_001002909 | GPATCH8 | frameshift_deleti  on | exon8 | c.4385delT | p.L1462fs | . | . |
| CTC1 | 17 | 43317913 | TG | T | 0.5 | NM_005892 | FMNL1 | frameshift_deleti  on | exon12 | c.1152delG | p.V384fs | . | . |
| CTC1 | 17 | 46876997 | TC | T | 1 | NM_001130918 | TTLL6 | frameshift_deleti  on | exon6 | c.736delG | p.E246fs | . | . |
| CTC1 | 17 | 48266769 | TCG | T | 0.5 | NM_000088 | COL1A1 | frameshift_deleti  on | exon39 | c.2796_2797del | p.G932fs | . | . |
| CTC1 | 17 | 48266872 | CA | C | 0.465 | NM_000088 | COL1A1 | frameshift_deleti  on | exon39 | c.2694delT | p.P898fs | . | . |
| CTC1 | 17 | 48696267 | TG | T | 0.469 | NM_198376 | CACNA1G | frameshift_deleti  on | exon31 | c.5578delG | p.G1860fs | . | . |
| CTC1 | 17 | 48917781 | TG | T | 0.213 | NM_175575 | WFIKKN2 | frameshift_deleti  on | exon2 | c.1133delG | p.C378fs | . | . |
| CTC1 | 17 | 49302393 | AT | A | 0.5 | NM_017643 | MBTD1 | frameshift_deleti  on | exon3 | c.129delA | p.T43fs | . | . |
| CTC1 | 17 | 49371283 | CA | C | 0.282 | NM_016001 | UTP18 | frameshift_deleti  on | exon12 | c.1524delA | p.T508fs | . | . |
| CTC1 | 17 | 56056604 | TTGC | T | 0.5 | NM_007146 | VEZF1 | nonframeshift_de  letion | exon5 | c.1044_1046del | p.348_349del | . | . |
| CTC1 | 17 | 56060542 | AG | A | 0.714 | NM_007146 | VEZF1 | frameshift_deleti  on | exon2 | c.245delC | p.A82fs | . | . |
| CTC1 | 17 | 56233367 | AT | A | 0.316 | NM_012374 | OR4D1 | frameshift_deleti  on | exon1 | c.854delT | p.M285fs | . | . |
| CTC1 | 17 | 56598926 | AT | A | 0.273 | NM_001198713 | 4-Sep | frameshift_deleti  on | exon8 | c.972delA | p.K324fs | . | . |
| CTC1 | 17 | 57288193 | TG | T | 0.439 | NM_018149 | SMG8 | frameshift_deleti  on | exon1 | c.782delG | p.C261fs | . | . |
| CTC1 | 17 | 57963472 | GT | G | 0.462 | NM_001193609 | TUBD1 | frameshift_deleti  on | exon3 | c.291delA | p.K97fs | . | . |
| CTC1 | 17 | 58740797 | AC | A | 0.278 | NM_003620 | PPM1D | frameshift_deleti  on | exon6 | c.1703delC | p.T568fs | . | . |
| CTC1 | 17 | 59858267 | AT | A | 0.889 | NM_032043 | BRIP1 | frameshift_deleti  on | exon12 | c.1727delA | p.N576fs | . | . |
| CTC1 | 17 | 61623174 | CT | C | 0.3 | NM_001278919 | KCNH6 | frameshift_deleti  on | exon13 | c.2789delT | p.L930fs | . | . |
| CTC1 | 17 | 61656729 | TG | T | 0.5 | NM_005828 | DCAF7 | frameshift_deleti  on | exon3 | c.330delG | p.L110fs | . | . |
| CTC1 | 17 | 61869869 | GT | G | 0.773 | NM_203499 | DDX42 | frameshift_deleti  on | exon3 | c.320delT | p.V107fs | . | . |
| CTC1 | 17 | 62049120 | GT | G | 0.356 | NM_000334 | SCN4A | frameshift_deleti  on | exon4 | c.572delA | p.D191fs | . | . |
| CTC1 | 17 | 62126490 | CA | C | 0.489 | NM_001433 | ERN1 | frameshift_deleti  on | exon18 | c.2325delT | p.F775fs | . | . |
| CTC1 | 17 | 62130323 | CT | C | 0.211 | NM_001433 | ERN1 | frameshift_deleti  on | exon17 | c.2069delA | p.K690fs | . | . |
| CTC1 | 17 | 62489012 | CT | C | 0.378 | NM_007215 | POLG2 | frameshift_deleti  on | exon2 | c.688delA | p.S230fs | . | . |
| CTC1 | 17 | 62855741 | GC | G | 0.522 | NM_199340 | LRRC37A3 | frameshift_deleti  on | exon11 | c.4522delG | p.A1508fs | . | . |
| CTC1 | 17 | 63200376 | TG | T | 0.222 | NM_001081955 | RGS9 | frameshift_deleti  on | exon15 | c.1152delG | p.V384fs | . | . |
| CTC1 | 17 | 63530173 | TG | T | 0.357 | NM_004655 | AXIN2 | frameshift_deleti  on | exon10 | c.2261delC | p.A754fs | . | . |
| CTC1 | 17 | 66253102 | CA | C | 0.5 | NM_001033574 | AMZ2 | frameshift_deleti  on | exon6 | c.902delA | p.Q301fs | . | . |
| CTC1 | 17 | 67084345 | AT | A | 0.29 | NM_080284 | ABCA6 | frameshift_deleti  on | exon28 | c.3660delA | p.K1220fs | . | . |

| CTC1 | 17 | 72245508 | CA | C | 1 | NM_032646 | TTYH2 | frameshift_deleti  on | exon8 | c.901delA | p.S301fs | . | . |
| --- | --- | --- | --- | --- | --- | --- | --- | --- | --- | --- | --- | --- | --- |
| CTC1 | 17 | 72843611 | T | TG | 0.288 | NM_000835 | GRIN2C | frameshift_inserti  on | exon9 | c.1836dupC | p.N613fs | . | . |
| CTC1 | 17 | 72860460 | CG | C | 1 | NM_001258015 | FDXR | frameshift_deleti  on | exon8 | c.691delC | p.R231fs | . | . |
| CTC1 | 17 | 73106609 | GC | G | 0.636 | NM_024585 | ARMC7 | frameshift_deleti  on | exon2 | c.144delC | p.S48fs | . | . |
| CTC1 | 17 | 73808633 | GA | G | 0.31 | NM_001080419 | UNK | frameshift_deleti  on | exon4 | c.590delA | p.E197fs | . | . |
| CTC1 | 17 | 74287804 | TA | T | 0.5 | NM_032134 | QRICH2 | frameshift_deleti  on | exon4 | c.2505delT | p.F835fs | . | . |
| CTC1 | 17 | 74475185 | TG | T | 0.393 | NM_001005498 | RHBDF2 | frameshift_deleti  on | exon4 | c.446delC | p.P149fs | . | . |
| CTC1 | 17 | 76528677 | AC | A | 0.273 | NM_173628 | DNAH17 | frameshift_deleti  on | exon20 | c.3000delG | p.E1000fs | . | . |
| CTC1 | 17 | 76570987 | CA | C | 0.333 | NM_173628 | DNAH17 | frameshift_deleti  on | exon2 | c.152delT | p.V51fs | . | . |
| CTC1 | 17 | 76798548 | TTTTTTC | T | 1 | NM_025090 | USP36 | nonframeshift_de  letion | exon17 | c.2874_2879del | p.958_960del | . | . |
| CTC1 | 17 | 77769090 | GCT | G | 0.613 | NM_020649 | CBX8 | frameshift_deleti  on | exon5 | c.512_513del | p.E171fs | . | . |
| CTC1 | 17 | 77769113 | CT | C | 0.786 | NM_020649 | CBX8 | frameshift_deleti  on | exon5 | c.490delA | p.R164fs | . | . |
| CTC1 | 17 | 78023709 | TG | T | 0.362 | NM_001243342 | CCDC40 | frameshift_deleti  on | exon6 | c.861delG | p.L287fs | . | . |
| CTC1 | 17 | 78064140 | G | GAAC | 1 | NM_001243342 | CCDC40 | nonframeshift_in  sertion | exon18 | c.3035_3036ins  AAC | p.R1012delinsR  T | . | . |
| CTC1 | 17 | 78064145 | A | ACAC | 1 | NM_001243342 | CCDC40 | nonframeshift_in  sertion | exon18 | c.3040_3041ins  CAC | p.R1014delinsT  R | . | . |
| CTC1 | 17 | 78086726 | GC | G | 0.178 | NM_000152 | GAA | frameshift_deleti  on | exon14 | c.1941delC | p.C647fs | . | . |
| CTC1 | 17 | 78112942 | GT | G | 0.192 | NM_014740 | EIF4A3 | frameshift_deleti  on | exon7 | c.605delA | p.Y202fs | . | . |
| CTC1 | 17 | 78157774 | GA | G | 0.242 | NM_001257970 | CARD14 | frameshift_deleti  on | exon4 | c.413delA | p.E138fs | . | . |
| CTC1 | 17 | 78178123 | AC | A | 0.571 | NM_024110 | CARD14 | frameshift_deleti  on | exon17 | c.2382delC | p.D794fs | . | . |
| CTC1 | 17 | 78350304 | GC | G | 0.611 | NM_001256071 | RNF213 | frameshift_deleti  on | exon52 | c.13390delC | p.P4464fs | . | . |
| CTC1 | 17 | 79225326 | CT | C | 0.25 | NM_001037984 | SLC38A10 | frameshift_deleti  on | exon14 | c.2031delA | p.R677fs | . | . |
| CTC1 | 17 | 79792728 | G | GA | 0.91 | NM_001007533 | PPP1R27 | frameshift_inserti  on | exon1 | c.101dupT | p.F34fs | . | . |
| CTC1 | 17 | 80085596 | AC | A | 0.608 | NM_198082 | CCDC57 | frameshift_deleti  on | exon16 | c.2534delG | p.G845fs | . | . |
| CTC1 | 17 | 80350306 | CG | C | 0.525 | NM_024648 | OGFOD3 | frameshift_deleti  on | exon9 | c.927delC | p.P309fs | . | . |
| CTC1 | 17 | 80364327 | AG | A | 0.364 | NM_024648 | OGFOD3 | frameshift_deleti  on | exon5 | c.454delC | p.L152fs | . | . |
| CTC1 | 18 | 2590068 | CA | C | 0.356 | NM_006101 | NDC80 | frameshift_deleti  on | exon10 | c.923delA | p.Q308fs | . | . |
| CTC1 | 18 | 2697093 | T | TC | 0.667 | NM_015295 | SMCHD1 | frameshift_inserti  on | exon9 | c.1104_1105ins  C | p.V368fs | . | . |
| CTC1 | 18 | 7023281 | AC | A | 0.875 | NM_005559 | LAMA1 | frameshift_deleti  on | exon19 | c.2582delG | p.G861fs | . | . |
| CTC1 | 18 | 9887313 | CA | C | 0.229 | NM_001098529 | TXNDC2 | frameshift_deleti  on | exon2 | c.838delA | p.K280fs | . | . |
| CTC1 | 18 | 19433143 | CT | C | 0.281 | NM_020774 | MIB1 | frameshift_deleti  on | exon18 | c.2630delT | p.L877fs | . | . |
| CTC1 | 18 | 21141476 | C | CA | 0.889 | NM_000271 | NPC1 | frameshift_inserti  on | exon5 | c.478dupT | p.C160fs | . | . |
| CTC1 | 18 | 21229135 | GC | G | 0.188 | NM_173505 | ANKRD29 | frameshift_deleti  on | exon2 | c.43delG | p.A15fs | . | . |
| CTC1 | 18 | 21437856 | GT | G | 0.667 | NM_001127717 | LAMA3 | frameshift_deleti  on | exon33 | c.4186delT | p.F1396fs | . | . |
| CTC1 | 18 | 22057175 | CA | C | 0.229 | NM_001143828 | HRH4 | frameshift_deleti  on | exon2 | c.559delA | p.K187fs | . | . |
| CTC1 | 18 | 28666641 | CA | C | 0.422 | NM_004949 | DSC2 | frameshift_deleti  on | exon7 | c.839delT | p.L280fs | . | . |
| CTC1 | 18 | 28710546 | C | CA | 0.191 | NM_024421 | DSC1 | frameshift_inserti  on | exon16 | c.2615_2616ins  T | p.E872fs | . | . |
| CTC1 | 18 | 28934450 | GA | G | 0.302 | NM_001942 | DSG1 | frameshift_deleti  on | exon15 | c.2292delA | p.G764fs | . | . |
| CTC1 | 18 | 29044335 | TC | T | 0.821 | NM_001944 | DSG3 | stopgain | exon9 | c.1262delC | p.S421X | . | . |
| CTC1 | 18 | 29867901 | CT | C | 0.234 | NM_001242409 | GAREM | frameshift_deleti on | exon4 | c.658delA | p.S220fs | . | . |
| CTC1 | 18 | 31319073 | AC | A | 0.727 | NM_030632 | ASXL3 | frameshift_deleti  on | exon11 | c.1706delC | p.T569fs | . | . |
| CTC1 | 18 | 32455331 | AG | A | 1 | NM_001198943 | DTNA | frameshift_deleti  on | exon8 | c.862delG | p.G288fs | . | . |
| CTC1 | 18 | 32822623 | GC | G | 0.784 | NM_001135178 | ZNF397 | frameshift_deleti  on | exon2 | c.190delC | p.P64fs | . | . |
| CTC1 | 18 | 32834215 | AG | A | 0.388 | NM_001288711 | ZSCAN30 | frameshift_deleti  on | exon2 | c.122delC | p.A41fs | . | . |
| CTC1 | 18 | 32949402 | TG | T | 0.273 | NM_145756 | ZNF396 | frameshift_deleti  on | exon4 | c.784delC | p.Q262fs | . | . |
| CTC1 | 18 | 33779972 | TC | T | 0.231 | NM_017947 | MOCOS | frameshift_deleti  on | exon4 | c.627delC | p.V209fs | . | . |
| CTC1 | 18 | 42456670 | C | CTCTT | 0.941 | NM_001130110 | SETBP1 | frameshift_inserti  on | exon4 | c.681_682insTC  TT | p.L227fs | . | . |
| CTC1 | 18 | 43446961 | TA | T | 0.4 | NM_020964 | EPG5 | frameshift_deleti  on | exon38 | c.6422delT | p.L2141fs | . | . |
| CTC1 | 18 | 43479412 | CT | C | 0.5 | NM_020964 | EPG5 | frameshift_deleti  on | exon27 | c.4765delA | p.S1589fs | . | . |
| CTC1 | 18 | 43833701 | T | TCTG | 0.184 | NM_001008239 | C18orf25 | nonframeshift_in  sertion | exon3 | c.754_755insCT  G | p.S252delinsSA | . | . |
| CTC1 | 18 | 43833704 | G | GCTA | 0.184 | NM_001008239 | C18orf25 | nonframeshift_in  sertion | exon3 | c.757_758insCT  A | p.G253delinsA  S | . | . |
| CTC1 | 18 | 45567445 | G | GA | 0.32 | NM_001039360 | ZBTB7C | frameshift_inserti  on | exon2 | c.33dupT | p.P12fs | . | . |
| CTC1 | 18 | 55283125 | GA | G | 0.8 | NM_004539 | NARS | frameshift_deleti  on | exon3 | c.175delT | p.S59fs | . | . |
| CTC1 | 18 | 56246439 | CT | C | 0.292 | NM_052947 | ALPK2 | frameshift_deleti  on | exon4 | c.1568delA | p.K523fs | . | . |
| CTC1 | 18 | 59195270 | GT | G | 0.432 | NM_031891 | CDH20 | frameshift_deleti  on | exon6 | c.1089delT | p.R363fs | . | . |
| CTC1 | 18 | 60646559 | GCAA | G | 0.283 | NM_194449 | PHLPP1 | nonframeshift_de  letion | exon17 | c.5050_5052del | p.1684_1684de  l | . | . |
| CTC1 | 18 | 60646562 | ACAG | A | 0.46 | NM_194449 | PHLPP1 | nonframeshift_de  letion | exon17 | c.5053_5055del | p.1685_1685de  l | . | . |
| CTC1 | 18 | 61151722 | GA | G | 0.619 | NM_002639 | SERPINB5 | frameshift_deleti  on | exon2 | c.62delA | p.E21fs | . | . |

| CTC1 | 18 | 66721318 | GAGAA | G | 0.188 | NM_024781 | CCDC102B | frameshift_deleti  on | exon8 | c.1487_1490del | p.E496fs | . | . |
| --- | --- | --- | --- | --- | --- | --- | --- | --- | --- | --- | --- | --- | --- |
| CTC1 | 18 | 67614289 | CA | C | 0.571 | NM_006566 | CD226 | frameshift_deleti  on | exon3 | c.62delT | p.V21fs | . | . |
| CTC1 | 18 | 67857864 | CA | C | 0.6 | NM_173630 | RTTN | frameshift_deleti  on | exon9 | c.1098delT | p.T366fs | . | . |
| CTC1 | 18 | 67863850 | T | TCTC | 1 | NM_173630 | RTTN | nonframeshift_in  sertion | exon7 | c.727_728insG  AG | p.D243delinsG  D | . | . |
| CTC1 | 18 | 67863854 | C | CTCA | 0.962 | NM_173630 | RTTN | stopgain | exon7 | c.723_724insTG  A | p.G242delinsX | . | . |
| CTC1 | 18 | 72223591 | G | GTGC | 0.917 | NM_032649 | CNDP1 | nonframeshift_in  sertion | exon2 | c.43_44insTGC | p.V15delinsVL | . | . |
| CTC1 | 18 | 72223597 | C | CTG | 0.688 | NM_032649 | CNDP1 | frameshift_inserti  on | exon2 | c.49_50insTG | p.L17fs | . | . |
| CTC1 | 18 | 72997837 | A | AG | 0.267 | NM_005786 | TSHZ1 | frameshift_inserti  on | exon2 | c.340_341insG | p.T114fs | . | . |
| CTC1 | 19 | 1535193 | GCCC | G | 1 | NM_001243079 | PLK5 | nonframeshift_de  letion | exon14 | c.956_958del | p.319_320del | . | . |
| CTC1 | 19 | 3976697 | TG | T | 0.607 | NM_001961 | EEF2 | frameshift_deleti  on | exon15 | c.2431delC | p.Q811fs | . | . |
| CTC1 | 19 | 4159716 | CCT | C | 0.923 | NM_001271995 | CREB3L3 | frameshift_deleti  on | exon4 | c.511_512del | p.L171fs | . | . |
| CTC1 | 19 | 4499632 | AAGCTG  GCCGGG | A | 1 | NM_001001520 | HDGFRP2 | frameshift_deleti  on | exon14 | c.1721_1722del | p.K574fs | . | . |
| CTC1 | 19 | 5138002 | T | TC | 0.833 | NM_015015 | KDM4B | frameshift_inserti  on | exon18 | c.2472dupC | p.V824fs | . | . |
| CTC1 | 19 | 5218507 | G | GA | 0.181 | NM_130853 | PTPRS | frameshift_inserti  on | exon17 | c.2678dupT | p.L893fs | . | . |
| CTC1 | 19 | 5604698 | AT | A | 0.346 | NM_014649 | SAFB2 | frameshift_deleti  on | exon11 | c.1454delA | p.N485fs | . | . |
| CTC1 | 19 | 5641945 | TC | T | 0.314 | NM_001201338 | SAFB | frameshift_deleti  on | exon4 | c.535delC | p.Q179fs | . | . |
| CTC1 | 19 | 5653181 | GT | G | 0.462 | NM_001201340 | SAFB | frameshift_deleti  on | exon9 | c.1143delT | p.G381fs | . | . |
| CTC1 | 19 | 5783634 | TG | T | 0.917 | NM_001134316 | PRR22 | frameshift_deleti  on | exon3 | c.623delC | p.P208fs | . | . |
| CTC1 | 19 | 5892955 | GT | G | 0.25 | NM_001193375 | NDUFA11 | frameshift_deleti  on | exon4 | c.659delA | p.N220fs | . | . |
| CTC1 | 19 | 6772853 | TC | T | 0.165 | NM_001258206 | VAV1 | frameshift_deleti  on | exon1 | c.36delC | p.I12fs | . | . |
| CTC1 | 19 | 7126651 | C | CA | 0.868 | NM_001079817 | INSR | frameshift_inserti  on | exon15 | c.2920_2921ins  T | p.G974fs | . | . |
| CTC1 | 19 | 7607672 | AG | A | 0.336 | NM_001166114 | PNPLA6 | frameshift_deleti  on | exon12 | c.1389delG | p.Q463fs | . | . |
| CTC1 | 19 | 7684944 | C | CG | 0.448 | NM_020196 | XAB2 | frameshift_inserti  on | exon17 | c.2283_2284ins  C | p.G762fs | . | . |
| CTC1 | 19 | 7992542 | CG | C | 0.833 | NM_006351 | TIMM44 | frameshift_deleti  on | exon12 | c.1238delC | p.P413fs | . | . |
| CTC1 | 19 | 8431138 | G | GT | 0.467 | NM_001039667 | ANGPTL4 | frameshift_inserti  on | exon3 | c.482_483insT | p.R161fs | . | . |
| CTC1 | 19 | 8669974 | GC | G | 0.947 | NM_030957 | ADAMTS10 | frameshift_deleti  on | exon4 | c.357delG | p.R119fs | . | . |
| CTC1 | 19 | 9010658 | G | GA | 0.545 | NM_024690 | MUC16 | frameshift_inserti  on | exon38 | c.39002_39003i  nsT | p.P13001fs | . | . |
| CTC1 | 19 | 9011377 | A | AT | 0.205 | NM_024690 | MUC16 | frameshift_inserti  on | exon36 | c.38855dupA | p.H12952fs | . | . |
| CTC1 | 19 | 9014589 | T | TG | 0.333 | NM_024690 | MUC16 | frameshift_inserti  on | exon31 | c.38385dupC | p.N12796fs | . | . |
| CTC1 | 19 | 9049395 | TC | T | 0.45 | NM_024690 | MUC16 | frameshift_deleti  on | exon5 | c.32235delG | p.G10745fs | . | . |
| CTC1 | 19 | 9061115 | G | GT | 0.571 | NM_024690 | MUC16 | frameshift_inserti  on | exon3 | c.26330_26331i  nsA | p.L8777fs | . | . |
| CTC1 | 19 | 9407408 | CT | C | 0.2 | NM_198535 | ZNF699 | frameshift_deleti  on | exon5 | c.671delA | p.Q224fs | . | . |
| CTC1 | 19 | 9524970 | GT | G | 0.625 | NM_001271314 | ZNF266 | frameshift_deleti  on | exon11 | c.630delA | p.K210fs | . | . |
| CTC1 | 19 | 9721745 | TG | T | 0.178 | NM_152289 | ZNF561 | stopgain | exon6 | c.591delC | p.Y197X | . | . |
| CTC1 | 19 | 10221006 | CCG | C | 0.333 | NM_001040664 | PPAN- P2RY11 | frameshift_deleti on | exon9 | c.830_831del | p.P277fs | . | . |
| CTC1 | 19 | 10265344 | CGG | C | 0.2 | NM_001379 | DNMT1 | frameshift_deleti  on | exon20 | c.1700_1701del | p.A567fs | . | . |
| CTC1 | 19 | 10265345 | G | GA | 0.211 | NM_001379 | DNMT1 | frameshift_inserti  on | exon20 | c.1700_1701ins  T | p.A567fs | . | . |
| CTC1 | 19 | 10431459 | CT | C | 0.164 | NM_133452 | RAVER1 | frameshift_deleti  on | exon9 | c.1692delA | p.P564fs | . | . |
| CTC1 | 19 | 10741955 | GC | G | 0.316 | NM_001145056 | SLC44A2 | frameshift_deleti  on | exon6 | c.330delC | p.C110fs | . | . |
| CTC1 | 19 | 11097624 | GC | G | 0.542 | NM_001128845 | SMARCA4 | frameshift_deleti  on | exon4 | c.805delC | p.P269fs | . | . |
| CTC1 | 19 | 11098362 | GC | G | 0.556 | NM_001128845 | SMARCA4 | frameshift_deleti  on | exon5 | c.881delC | p.A294fs | . | . |
| CTC1 | 19 | 11319767 | GCC | G | 0.5 | NM_020812 | DOCK6 | frameshift_deleti  on | exon38 | c.4762_4763del | p.G1588fs | . | . |
| CTC1 | 19 | 12127392 | GT | G | 0.274 | NM_001080411 | ZNF433 | frameshift_deleti  on | exon4 | c.289delA | p.T97fs | . | . |
| CTC1 | 19 | 12429565 | TG | T | 0.333 | NM_145276 | ZNF563 | frameshift_deleti  on | exon4 | c.1273delC | p.Q425fs | . | . |
| CTC1 | 19 | 12874233 | GC | G | 0.191 | NM_001100176 | HOOK2 | frameshift_deleti  on | exon22 | c.2016delG | p.L672fs | . | . |
| CTC1 | 19 | 13011416 | TA | T | 0.857 | NM_001105578 | SYCE2 | frameshift_deleti  on | exon4 | c.352delT | p.Y118fs | . | . |
| CTC1 | 19 | 13059017 | AC | A | 0.405 | NM_001270362 | RAD23A | frameshift_deleti  on | exon3 | c.262delC | p.P88fs | . | . |
| CTC1 | 19 | 13059153 | TC | T | 0.722 | NM_001270362 | RAD23A | frameshift_deleti  on | exon3 | c.398delC | p.S133fs | . | . |
| CTC1 | 19 | 13192502 | CG | C | 0.25 | NM_001271043 | NFIX | frameshift_deleti  on | exon8 | c.1112delG | p.R371fs | . | . |
| CTC1 | 19 | 13988558 | C | CGAG | 0.478 | NM_001098622 | NANOS3 | nonframeshift_in  sertion | exon1 | c.496_497insG  AG | p.R166delinsR  G | . | . |
| CTC1 | 19 | 14162967 | GC | G | 0.424 | NM_004843 | IL27RA | frameshift_deleti  on | exon14 | c.1777delC | p.P593fs | . | . |
| CTC1 | 19 | 16640580 | TTGC | T | 0.6 | NM_006387 | CHERP | nonframeshift_de  letion | exon8 | c.1005_1007del | p.335_336del | . | . |
| CTC1 | 19 | 17306135 | AC | A | 0.276 | NM_001130065 | MYO9B | frameshift_deleti  on | exon22 | c.3900delC | p.Y1300fs | . | . |
| CTC1 | 19 | 17476531 | CG | C | 0.6 | NM_031310 | PLVAP | frameshift_deleti  on | exon3 | c.742delC | p.R248fs | . | . |
| CTC1 | 19 | 17643077 | GC | G | 0.8 | NM_001098524 | FAM129C | frameshift_deleti  on | exon4 | c.286delC | p.P96fs | . | . |
| CTC1 | 19 | 19115105 | GC | G | 0.4 | NM_001017392 | SUGP2 | frameshift_deleti  on | exon7 | c.2800delG | p.A934fs | . | . |
| CTC1 | 19 | 19136034 | GT | G | 0.279 | NM_001017392 | SUGP2 | frameshift_deleti  on | exon3 | c.1122delA | p.K374fs | . | . |

| CTC1 | 19 | 19381069 | CGCAGG  TATGGC TCTCCCT | C | 0.673 | NM_001001524 | TM6SF2 | frameshift_deleti on | exon4 | c.298_313del | p.G100fs | . | . |
| --- | --- | --- | --- | --- | --- | --- | --- | --- | --- | --- | --- | --- | --- |
| CTC1 | 19 | 19576246 | TG | T | 0.471 | NM_017660 | GATAD2A | frameshift_deleti  on | exon2 | c.93delG | p.M31fs | . | . |
| CTC1 | 19 | 19906452 | CA | C | 0.286 | NM_001145404 | ZNF506 | frameshift_deleti  on | exon3 | c.147delT | p.F49fs | . | . |
| CTC1 | 19 | 20229536 | TA | T | 0.417 | NM_007138 | ZNF90 | frameshift_deleti  on | exon4 | c.1174delA | p.S392fs | . | . |
| CTC1 | 19 | 20728760 | TC | T | 0.149 | NM_001159293 | ZNF737 | frameshift_deleti  on | exon4 | c.248delG | p.R83fs | . | . |
| CTC1 | 19 | 20807177 | G | GA | 0.5 | NM_001076675 | ZNF626 | frameshift_inserti  on | exon4 | c.1505dupT | p.I502fs | . | . |
| CTC1 | 19 | 21239802 | GA | G | 0.333 | NM_001172671 | ZNF430 | frameshift_deleti  on | exon5 | c.686delA | p.E229fs | . | . |
| CTC1 | 19 | 21299774 | T | TATA | 0.258 | NM_182515 | ZNF714 | nonframeshift_in  sertion | exon5 | c.304_305insAT  A | p.Y102delinsYN | . | . |
| CTC1 | 19 | 21299852 | AT | A | 0.387 | NM_182515 | ZNF714 | frameshift_deleti  on | exon5 | c.383delT | p.I128fs | . | . |
| CTC1 | 19 | 21948579 | TC | T | 0.286 | NM_173531 | ZNF100 | frameshift_deleti  on | exon2 | c.12delG | p.P4fs | . | . |
| CTC1 | 19 | 22498749 | GT | G | 0.571 | NM_001242680 | ZNF729 | frameshift_deleti  on | exon4 | c.2531delT | p.V844fs | . | . |
| CTC1 | 19 | 22846879 | TG | T | 0.545 | NM_020855 | ZNF492 | frameshift_deleti  on | exon4 | c.409delG | p.G137fs | . | . |
| CTC1 | 19 | 23544871 | TA | T | 0.317 | NM_003430 | ZNF91 | frameshift_deleti  on | exon4 | c.909delT | p.F303fs | . | . |
| CTC1 | 19 | 31768251 | TG | T | 0.318 | NM_020856 | TSHZ3 | frameshift_deleti  on | exon2 | c.2447delC | p.A816fs | . | . |
| CTC1 | 19 | 31769637 | C | CT | 0.437 | NM_020856 | TSHZ3 | frameshift_inserti  on | exon2 | c.1061dupA | p.K354fs | . | . |
| CTC1 | 19 | 33414384 | TG | T | 0.185 | NM_032816 | CEP89 | frameshift_deleti  on | exon12 | c.1228delC | p.Q410fs | . | . |
| CTC1 | 19 | 34710339 | TC | T | 0.235 | NM_001114093 | LSM14A | frameshift_deleti  on | exon7 | c.826delC | p.R276fs | . | . |
| CTC1 | 19 | 34810856 | GT | G | 0.593 | NM_014686 | KIAA0355 | frameshift_deleti  on | exon3 | c.541delT | p.F181fs | . | . |
| CTC1 | 19 | 35450079 | TG | T | 0.471 | NM_175872 | ZNF792 | frameshift_deleti  on | exon4 | c.679delC | p.Q227fs | . | . |
| CTC1 | 19 | 35996841 | CT | C | 0.435 | NM_001126059 | DMKN | frameshift_deleti  on | exon4 | c.224delA | p.K75fs | . | . |
| CTC1 | 19 | 36002361 | ACTGCT  GCCACC ACTGCT GCCGCC ACTGCT GCCGCC | A | 1 | NM_001126057 | DMKN | nonframeshift_de letion | exon5 | c.819_869del | p.273_290del | . | . |
| CTC1 | 19 | 36214830 | TA | T | 0.75 | NM_014727 | KMT2B | frameshift_deleti  on | exon8 | c.3257delA | p.Y1086fs | . | . |
| CTC1 | 19 | 36348336 | TG | T | 0.388 | NM_032123 | KIRREL2 | frameshift_deleti  on | exon2 | c.152delG | p.W51fs | . | . |
| CTC1 | 19 | 37117077 | ACC | A | 0.235 | NM_001256838 | ZNF382 | frameshift_deleti  on | exon5 | c.276_277del | p.Y92fs | . | . |
| CTC1 | 19 | 37383153 | AC | A | 0.521 | NM_001037232 | ZNF829 | frameshift_deleti  on | exon6 | c.539delG | p.G180fs | . | . |
| CTC1 | 19 | 37488204 | TA | T | 0.375 | NM_001204839 | ZNF568 | frameshift_deleti  on | exon9 | c.1420delA | p.K474fs | . | . |
| CTC1 | 19 | 37619121 | CAG | C | 0.308 | NM_144689 | ZNF420 | frameshift_deleti  on | exon5 | c.1229_1230del | p.Q410fs | . | . |
| CTC1 | 19 | 37642884 | GC | G | 0.222 | NM_001288800 | ZNF585A | frameshift_deleti  on | exon5 | c.1916delG | p.C639fs | . | . |
| CTC1 | 19 | 38028150 | TCA | T | 0.167 | NM_001013659 | ZNF793 | frameshift_deleti  on | exon8 | c.591_592del | p.F197fs | . | . |
| CTC1 | 19 | 38056793 | CTT | C | 0.8 | NM_016536 | ZNF571 | frameshift_deleti  on | exon4 | c.535_536del | p.K179fs | . | . |
| CTC1 | 19 | 38102982 | TA | T | 1 | NM_001172226 | ZNF540 | frameshift_deleti  on | exon4 | c.706delA | p.K236fs | . | . |
| CTC1 | 19 | 38380004 | TG | T | 0.297 | NM_031951 | WDR87 | frameshift_deleti  on | exon6 | c.4189delC | p.Q1397fs | . | . |
| CTC1 | 19 | 38385371 | AC | A | 0.667 | NM_031951 | WDR87 | frameshift_deleti  on | exon4 | c.854delG | p.G285fs | . | . |
| CTC1 | 19 | 38742013 | G | GA | 0.25 | NM_001243947 | PPP1R14A | frameshift_inserti  on | exon3 | c.307dupT | p.S103fs | . | . |
| CTC1 | 19 | 38795550 | G | GAAGA  AGGAG | 1 | NM_033520 | C19orf33 | nonframeshift_in  sertion | exon4 | c.267_268insAA  GAAGGAGAAG | p.K89delinsKKK  EKG | . | . |
| CTC1 | 19 | 38876643 | GC | G | 0.5 | NM_152657 | GGN | frameshift_deleti  on | exon3 | c.1258delG | p.A420fs | . | . |
| CTC1 | 19 | 38882863 | CCCT | C | 0.173 | NM_001042522 | SPRED3 | nonframeshift_de  letion | exon3 | c.359_361del | p.120_121del | . | . |
| CTC1 | 19 | 38966000 | AC | A | 0.376 | NM_000540 | RYR1 | frameshift_deleti  on | exon29 | c.4204delC | p.P1402fs | . | . |
| CTC1 | 19 | 38985174 | AT | A | 0.323 | NM_000540 | RYR1 | frameshift_deleti  on | exon39 | c.6458delT | p.M2153fs | . | . |
| CTC1 | 19 | 39401287 | GCGCCT  CTCTCCT | G | 0.12 | NM_001243212 | CCER2 | frameshift_deleti  on | exon4 | c.614_626del | p.G205fs | . | . |
| CTC1 | 19 | 39663567 | CG | C | 0.667 | NM_001014832 | PAK4 | frameshift_deleti  on | exon3 | c.215delG | p.R72fs | . | . |
| CTC1 | 19 | 39798963 | GC | G | 0.295 | NM_020862 | LRFN1 | frameshift_deleti  on | exon2 | c.1625delG | p.G542fs | . | . |
| CTC1 | 19 | 39912861 | CCT | C | 0.359 | NM_022835 | PLEKHG2 | frameshift_deleti  on | exon17 | c.1611_1612del | p.P537fs | . | . |
| CTC1 | 19 | 40873722 | TCG | T | 0.5 | NM_001031696 | PLD3 | frameshift_deleti  on | exon6 | c.366_367del | p.I122fs | . | . |
| CTC1 | 19 | 40880522 | TCA | T | 1 | NM_001031696 | PLD3 | frameshift_deleti  on | exon10 | c.1015_1016del | p.H339fs | . | . |
| CTC1 | 19 | 41072126 | CCCAGG  AGCCGC TCCTGC AGAGCC | C | 0.647 | NM_020971 | SPTBN4 | nonframeshift_de letion | exon30 | c.6198_6230del | p.2066_2077de l | . | . |
| CTC1 | 19 | 41072131 | GAGCCG  CTCCTG CAGAGC CGGGAG | G | 0.667 | NM_020971 | SPTBN4 | frameshift_deleti on | exon30 | c.6203_6234del | p.E2068fs | . | . |
| CTC1 | 19 | 41188843 | G | GC | 0.364 | NM_004756 | NUMBL | frameshift_inserti  on | exon4 | c.279_280insG | p.R94fs | . | . |
| CTC1 | 19 | 41596376 | TG | T | 0.676 | NM_000766 | CYP2A13 | frameshift_deleti  on | exon4 | c.562delG | p.G188fs | . | . |
| CTC1 | 19 | 42383668 | AG | A | 0.286 | NM_001783 | CD79A | frameshift_deleti  on | exon3 | c.444delG | p.E148fs | . | . |
| CTC1 | 19 | 42474455 | AG | A | 0.275 | NM_001256213 | ATP1A3 | frameshift_deleti  on | exon18 | c.2456delC | p.P819fs | . | . |
| CTC1 | 19 | 42830480 | G | GA | 0.444 | NM_001271938 | MEGF8 | frameshift_inserti  on | exon1 | c.86dupA | p.D29fs | . | . |

| CTC1 | 19 | 42910507 | TC | T | 1 | NM_005357 | LIPE | frameshift_deleti  on | exon7 | c.2170delG | p.D724fs | . | . |
| --- | --- | --- | --- | --- | --- | --- | --- | --- | --- | --- | --- | --- | --- |
| CTC1 | 19 | 42946921 | TAG | T | 0.131 | NM_198477 | CXCL17 | frameshift_deleti  on | exon1 | c.62_63del | p.S21fs | . | . |
| CTC1 | 19 | 43268140 | AG | A | 0.992 | NM_001130167 | PSG8 | frameshift_deleti  on | exon2 | c.357delC | p.S119fs | . | . |
| CTC1 | 19 | 44276182 | GC | G | 0.371 | NM_002250 | KCNN4 | frameshift_deleti  on | exon4 | c.788delG | p.G263fs | . | . |
| CTC1 | 19 | 44515225 | TC | T | 0.429 | NM_006300 | ZNF230 | frameshift_deleti  on | exon5 | c.1035delC | p.F345fs | . | . |
| CTC1 | 19 | 45494531 | G | GA | 0.5 | NM_001282175 | CLPTM1 | frameshift_inserti  on | exon12 | c.1413_1414ins  A | p.P471fs | . | . |
| CTC1 | 19 | 45575047 | AG | A | 0.857 | NM_145288 | ZNF296 | frameshift_deleti  on | exon3 | c.1239delC | p.P413fs | . | . |
| CTC1 | 19 | 45912489 | CAAG | C | 0.125 | NM_012099 | CD3EAP | nonframeshift_de  letion | exon3 | c.1264_1266del | p.422_422del | . | . |
| CTC1 | 19 | 46190845 | AC | A | 0.4 | NM_004597 | SNRPD2 | frameshift_deleti  on | exon3 | c.322delG | p.V108fs | . | . |
| CTC1 | 19 | 46196165 | GC | G | 0.697 | NM_001163377 | QPCTL | frameshift_deleti  on | exon1 | c.205delC | p.R69fs | . | . |
| CTC1 | 19 | 46280671 | AG | A | 0.97 | NM_001288765 | DMPK | frameshift_deleti  on | exon6 | c.792delC | p.P264fs | . | . |
| CTC1 | 19 | 46307726 | GT | G | 0.356 | NM_030785 | RSPH6A | frameshift_deleti  on | exon3 | c.1436delA | p.N479fs | . | . |
| CTC1 | 19 | 46347328 | CA | C | 0.154 | NM_004819 | SYMPK | frameshift_deleti  on | exon8 | c.806delT | p.M269fs | . | . |
| CTC1 | 19 | 46509860 | TG | T | 0.24 | NM_001267723 | CCDC61 | frameshift_deleti  on | exon4 | c.276delG | p.L92fs | . | . |
| CTC1 | 19 | 47282128 | TG | T | 0.222 | NM_001145145 | SLC1A5 | frameshift_deleti  on | exon4 | c.255delC | p.G85fs | . | . |
| CTC1 | 19 | 47878866 | CA | C | 0.146 | NM_014681 | DHX34 | frameshift_deleti  on | exon10 | c.2209delA | p.K737fs | . | . |
| CTC1 | 19 | 47879314 | TC | T | 0.889 | NM_014681 | DHX34 | frameshift_deleti  on | exon11 | c.2442delC | p.V814fs | . | . |
| CTC1 | 19 | 47918017 | TCCTC | T | 0.833 | NM_001009813 | MEIS3 | frameshift_deleti  on | exon6 | c.499_502del | p.E167fs | . | . |
| CTC1 | 19 | 48201932 | AC | A | 0.462 | NM_015711 | GLTSCR1 | frameshift_deleti  on | exon12 | c.3291delC | p.H1097fs | . | . |
| CTC1 | 19 | 48789671 | GC | G | 0.173 | NM_153608 | ZNF114 | frameshift_deleti  on | exon5 | c.791delC | p.A264fs | . | . |
| CTC1 | 19 | 48967610 | AC | A | 0.208 | NM_013348 | KCNJ14 | frameshift_deleti  on | exon3 | c.888delC | p.D296fs | . | . |
| CTC1 | 19 | 49120607 | CGA | C | 0.333 | NM_001270490 | RPL18 | frameshift_deleti  on | exon2 | c.75_76del | p.S25fs | . | . |
| CTC1 | 19 | 49129299 | GC | G | 0.667 | NM_001204160 | SPHK2 | frameshift_deleti  on | exon2 | c.84delC | p.R28fs | . | . |
| CTC1 | 19 | 49657709 | GACA | G | 0.398 | NM_002152 | HRC | nonframeshift_de  letion | exon1 | c.783_785del | p.261_262del | . | . |
| CTC1 | 19 | 49657710 | ACAT | A | 0.957 | NM_002152 | HRC | nonframeshift_de  letion | exon1 | c.782_784del | p.261_262del | . | . |
| CTC1 | 19 | 49657710 | ACATCA  T | A | 0.957 | NM_002152 | HRC | nonframeshift_de  letion | exon1 | c.779_784del | p.260_262del | . | . |
| CTC1 | 19 | 49657889 | T | TTCC | 0.762 | NM_002152 | HRC | nonframeshift_in  sertion | exon1 | c.605_606insG  GA | p.E202delinsEE | . | . |
| CTC1 | 19 | 49657915 | C | CCTT | 0.714 | NM_002152 | HRC | nonframeshift_in  sertion | exon1 | c.579_580insAA  G | p.E194delinsKE | . | . |
| CTC1 | 19 | 49658033 | GC | G | 0.25 | NM_002152 | HRC | frameshift_deleti  on | exon1 | c.461delG | p.S154fs | . | . |
| CTC1 | 19 | 49658036 | G | GT | 0.342 | NM_002152 | HRC | frameshift_inserti  on | exon1 | c.458_459insA | p.P153fs | . | . |
| CTC1 | 19 | 50434169 | TG | T | 0.25 | NM_001193646 | ATF5 | frameshift_deleti  on | exon2 | c.63delG | p.L21fs | . | . |
| CTC1 | 19 | 50510971 | CCTGGT  CTTCTGA GGGCTA | C | 0.457 | NM_001025778 | VRK3 | nonframeshift_de letion | exon4 | c.231_251del | p.77_84del | . | . |
| CTC1 | 19 | 50548776 | GA | G | 0.36 | NM_001006656 | ZNF473 | frameshift_deleti  on | exon5 | c.1077delA | p.R359fs | . | . |
| CTC1 | 19 | 50549561 | TG | T | 0.364 | NM_001006656 | ZNF473 | frameshift_deleti  on | exon5 | c.1862delG | p.W621fs | . | . |
| CTC1 | 19 | 50758526 | GC | G | 0.75 | NM_001145809 | MYH14 | frameshift_deleti  on | exon17 | c.1996delC | p.P666fs | . | . |
| CTC1 | 19 | 50868835 | GGCA | G | 0.216 | NM_004851 | NAPSA | nonframeshift_de  letion | exon1 | c.41_43del | p.14_15del | . | . |
| CTC1 | 19 | 50881822 | A | ACAG | 0.269 | NM_001256647 | NR1H2 | nonframeshift_in  sertion | exon5 | c.225_226insCA  G | p.K75delinsKQ | . | . |
| CTC1 | 19 | 51170267 | CG | C | 0.455 | NM_016148 | SHANK1 | frameshift_deleti  on | exon22 | c.4949delC | p.P1650fs | . | . |
| CTC1 | 19 | 51835892 | T | TG | 1 | NM_001163922 | VSIG10L | frameshift_inserti  on | exon10 | c.2576dupC | p.A859fs | . | . |
| CTC1 | 19 | 51892239 | TA | T | 0.283 | NM_001193623 | C19orf84 | stoploss | exon2 | c.559delT | p.X187R | . | . |
| CTC1 | 19 | 52660534 | G | GT | 0.326 | NM_001102657 | ZNF836 | frameshift_inserti on | exon5 | c.401dupA | p.N134fs | . | . |
| CTC1 | 19 | 52793544 | AT | A | 0.283 | NM_001010851 | ZNF766 | frameshift_deleti  on | exon4 | c.501delT | p.D167fs | . | . |
| CTC1 | 19 | 52794033 | AT | A | 0.769 | NM_001010851 | ZNF766 | frameshift_deleti  on | exon4 | c.990delT | p.N330fs | . | . |
| CTC1 | 19 | 53014589 | G | GA | 0.222 | NM_001099694 | ZNF578 | frameshift_inserti  on | exon6 | c.956dupA | p.E319fs | . | . |
| CTC1 | 19 | 53343992 | CA | C | 0.214 | NM_001008801 | ZNF468 | frameshift_deleti  on | exon4 | c.1554delT | p.S518fs | . | . |
| CTC1 | 19 | 53454475 | C | CACT | 1 | NM_001202456 | ZNF816 | nonframeshift_in  sertion | exon4 | c.552_553insA  GT | p.G185delinsS  G | . | . |
| CTC1 | 19 | 53645289 | AT | A | 0.167 | NM_001172674 | ZNF347 | frameshift_deleti  on | exon5 | c.794delA | p.N265fs | . | . |
| CTC1 | 19 | 53741258 | CTA | C | 0.857 | NM_182609 | ZNF677 | frameshift_deleti  on | exon5 | c.720_721del | p.Y240fs | . | . |
| CTC1 | 19 | 53911751 | CA | C | 0.192 | NM_001040185 | ZNF765 | frameshift_deleti  on | exon4 | c.944delA | p.Q315fs | . | . |
| CTC1 | 19 | 54649750 | AT | A | 0.462 | NM_014516 | CNOT3 | frameshift_deleti  on | exon9 | c.809delT | p.I270fs | . | . |
| CTC1 | 19 | 54667535 | CA | C | 0.683 | NM_001145303 | TMC4 | frameshift_deleti  on | exon8 | c.1215delT | p.F405fs | . | . |
| CTC1 | 19 | 55087292 | CCT | C | 0.301 | NM_001130917 | LILRA2 | frameshift_deleti  on | exon6 | c.972_973del | p.P324fs | . | . |
| CTC1 | 19 | 55087407 | TC | T | 0.333 | NM_001130917 | LILRA2 | frameshift_deleti  on | exon6 | c.1087delC | p.P363fs | . | . |
| CTC1 | 19 | 55525850 | C | CT | 0.207 | NM_001083899 | GP6 | frameshift_inserti  on | exon8 | c.1462dupA | p.R488fs | . | . |
| CTC1 | 19 | 55790886 | A | AGCCG  CCGCC | 0.692 | NM_012267 | HSPBP1 | nonframeshift_in  sertion | exon2 | c.90_91insGGC  GGCGGC | p.S31delinsGG  GS | . | . |
| CTC1 | 19 | 55832354 | G | GA | 0.291 | NM_001085488 | TMEM150B | frameshift_inserti  on | exon3 | c.50dupT | p.I17fs | . | . |

| CTC1 | 19 | 55869879 | GGCTCC  TTCATCT CGCCCC | G | 1 | NM_001145402 | FAM71E2 | nonframeshift_de letion | exon9 | c.2333_2356del | p.778_786del | . | . |
| --- | --- | --- | --- | --- | --- | --- | --- | --- | --- | --- | --- | --- | --- |
| CTC1 | 19 | 56052337 | A | AC | 0.949 | NM_001199824 | SBK3 | frameshift_inserti  on | exon4 | c.954dupG | p.F319fs | . | . |
| CTC1 | 19 | 56466577 | GA | G | 0.333 | NM_176811 | NLRP8 | frameshift_deleti  on | exon3 | c.1154delA | p.E385fs | . | . |
| CTC1 | 19 | 57089381 | GA | G | 0.439 | NM_001001668 | ZNF470 | frameshift_deleti  on | exon6 | c.1585delA | p.K529fs | . | . |
| CTC1 | 19 | 57325643 | G | GC | 0.312 | NM_001146186 | PEG3 | frameshift_inserti  on | exon7 | c.4166dupG | p.G1389fs | . | . |
| CTC1 | 19 | 57802788 | CA | C | 0.734 | NM_006635 | ZNF460 | frameshift_deleti  on | exon3 | c.880delA | p.N294fs | . | . |
| CTC1 | 19 | 57802980 | TCA | T | 0.126 | NM_006635 | ZNF460 | frameshift_deleti  on | exon3 | c.1072_1073del | p.H358fs | . | . |
| CTC1 | 19 | 57988666 | A | AGCC | 1 | NM_001024596 | ZNF772 | nonframeshift_in  sertion | exon1 | c.11_12insGGC | p.A4delinsAA | . | . |
| CTC1 | 19 | 58265554 | AT | A | 0.17 | NM_173632 | ZNF776 | frameshift_deleti  on | exon3 | c.1057delT | p.C353fs | . | . |
| CTC1 | 19 | 58352656 | CT | C | 0.526 | NM_001204818 | ZNF587B | frameshift_deleti  on | exon3 | c.615delT | p.S205fs | . | . |
| CTC1 | 19 | 58385869 | C | CATA | 1 | NM_001144989 | ZNF814 | nonframeshift_in  sertion | exon3 | c.888_889insTA  T | p.E297delinsYE | . | . |
| CTC1 | 19 | 58385870 | A | ATAT | 0.913 | NM_001144989 | ZNF814 | nonframeshift_in  sertion | exon3 | c.887_888insAT  A | p.H296delinsQ  Y | . | . |
| CTC1 | 19 | 58806433 | CG | C | 0.633 | NM_021089 | ZNF8 | frameshift_deleti  on | exon4 | c.1260delG | p.A420fs | . | . |
| CTC1 | 19 | 58944913 | CCA | C | 0.106 | NM_003433 | ZNF132 | frameshift_deleti  on | exon3 | c.1896_1897del | p.C632fs | . | . |
| CTC1 | 19 | 58989189 | AG | A | 1 | NM_017908 | ZNF446 | frameshift_deleti  on | exon3 | c.529delG | p.V177fs | . | . |
| CTC1 | 19 | 59009890 | TC | T | 0.222 | NM_012254 | SLC27A5 | stopgain | exon10 | c.2064delG | p.W688X | . | . |
| CTC1 | 19 | 59023278 | GAGC | G | 0.167 | NM_012254 | SLC27A5 | nonframeshift_de letion | exon1 | c.42_44del | p.14_15del | . | . |
| CTC1 | 20 | 126310 | ACC | A | 0.324 | NM_030931 | DEFB126 | frameshift_deleti  on | exon2 | c.314_315del | p.T105fs | . | . |
| CTC1 | 20 | 1286235 | AC | A | 0.433 | NM_014723 | SNPH | frameshift_deleti  on | exon6 | c.1023delC | p.D341fs | . | . |
| CTC1 | 20 | 1896051 | CCGA | C | 1 | NM_001040023 | SIRPA | nonframeshift_de  letion | exon2 | c.387_389del | p.129_130del | . | . |
| CTC1 | 20 | 2636360 | GC | G | 0.895 | NM_006392 | NOP56 | frameshift_deleti  on | exon7 | c.878delC | p.A293fs | . | . |
| CTC1 | 20 | 2736430 | AC | A | 0.444 | NM_001110514 | EBF4 | frameshift_deleti  on | exon16 | c.1687delC | p.P563fs | . | . |
| CTC1 | 20 | 2774881 | CG | C | 0.621 | NM_001184699 | CPXM1 | frameshift_deleti  on | exon14 | c.1937delC | p.P646fs | . | . |
| CTC1 | 20 | 3129667 | C | CA | 0.905 | NM_021826 | FASTKD5 | frameshift_inserti  on | exon2 | c.49dupT | p.C17fs | . | . |
| CTC1 | 20 | 3181046 | AT | A | 0.529 | NM_023935 | DDRGK1 | frameshift_deleti  on | exon3 | c.349delA | p.I117fs | . | . |
| CTC1 | 20 | 3649632 | ATCTGG  ACT | A | 1 | NM_153202 | ADAM33 | frameshift_deleti  on | exon21 | c.2334_2341del | p.Q778fs | . | . |
| CTC1 | 20 | 3781491 | CT | C | 0.5 | NM_001287516 | CDC25B | frameshift_deleti  on | exon6 | c.370delT | p.S124fs | . | . |
| CTC1 | 20 | 5098270 | TC | T | 0.556 | NM_182649 | PCNA | frameshift_deleti  on | exon4 | c.427delG | p.E143fs | . | . |
| CTC1 | 20 | 6022294 | CT | C | 0.333 | NM_152611 | LRRN4 | frameshift_deleti  on | exon5 | c.1596delA | p.E532fs | . | . |
| CTC1 | 20 | 9317780 | TTG | T | 0.44 | NM_000933 | PLCB4 | frameshift_deleti  on | exon2 | c.93_94del | p.F31fs | . | . |
| CTC1 | 20 | 9546783 | CG | C | 0.556 | NM_177990 | PAK7 | frameshift_deleti  on | exon5 | c.1238delC | p.P413fs | . | . |
| CTC1 | 20 | 14307910 | GT | G | 1 | NM_013281 | FLRT3 | frameshift_deleti  on | exon2 | c.242delA | p.N81fs | . | . |
| CTC1 | 20 | 17923755 | CT | C | 0.9 | NM_001282454 | SNX5 | frameshift_deleti  on | exon12 | c.847delA | p.R283fs | . | . |
| CTC1 | 20 | 17934751 | GC | G | 0.162 | NM_014426 | SNX5 | frameshift_deleti  on | exon4 | c.277delG | p.A93fs | . | . |
| CTC1 | 20 | 20024215 | TC | T | 0.36 | NM_001278628 | CRNKL1 | frameshift_deleti  on | exon7 | c.892delG | p.E298fs | . | . |
| CTC1 | 20 | 20484002 | GT | G | 0.444 | NM_020343 | RALGAPA2 | frameshift_deleti  on | exon35 | c.5200delA | p.T1734fs | . | . |
| CTC1 | 20 | 20571847 | T | TG | 0.267 | NM_020343 | RALGAPA2 | frameshift_inserti  on | exon17 | c.2314_2315ins  C | p.E772fs | . | . |
| CTC1 | 20 | 23349456 | TA | T | 0.314 | NM_022482 | GZF1 | frameshift_deleti  on | exon3 | c.1518delA | p.L506fs | . | . |
| CTC1 | 20 | 23586258 | TG | T | 0.324 | NM_001008693 | CST9 | frameshift_deleti  on | exon1 | c.243delC | p.S81fs | . | . |
| CTC1 | 20 | 23728494 | T | TC | 0.2 | NM_001898 | CST1 | frameshift_inserti  on | exon3 | c.384dupG | p.N129fs | . | . |
| CTC1 | 20 | 25457049 | GCTCCC  A | G | 1 | NM_025176 | NINL | nonframeshift_de  letion | exon17 | c.2872_2877del | p.958_959del | . | . |
| CTC1 | 20 | 30737525 | TG | T | 1 | NM_014742 | TM9SF4 | frameshift_deleti  on | exon10 | c.1044delG | p.L348fs | . | . |
| CTC1 | 20 | 31017746 | CCAG | C | 0.162 | NM_015338 | ASXL1 | nonframeshift_de  letion | exon7 | c.609_611del | p.203_204del | . | . |
| CTC1 | 20 | 33330967 | TTGC | T | 0.172 | NM_014071 | NCOA6 | nonframeshift_de  letion | exon11 | c.3090_3092del | p.1030_1031de  l | . | . |
| CTC1 | 20 | 33586985 | GGA | G | 0.444 | NM_020884 | MYH7B | frameshift_deleti  on | exon36 | c.4444_4445del | p.E1482fs | . | . |
| CTC1 | 20 | 33839805 | CA | C | 0.34 | NM_006690 | MMP24 | frameshift_deleti  on | exon3 | c.494delA | p.Q165fs | . | . |
| CTC1 | 20 | 34053883 | GT | G | 0.188 | NM_007186 | CEP250 | frameshift_deleti  on | exon7 | c.347delT | p.V116fs | . | . |
| CTC1 | 20 | 34129855 | GC | G | 0.258 | NM_015966 | ERGIC3 | frameshift_deleti  on | exon1 | c.10delC | p.L4fs | . | . |
| CTC1 | 20 | 34219624 | T | TG | 0.268 | NM_001198863 | CPNE1 | frameshift_inserti  on | exon7 | c.612dupC | p.S205fs | . | . |
| CTC1 | 20 | 34309667 | CA | C | 0.233 | NM_001242599 | RBM39 | frameshift_deleti  on | exon8 | c.753delT | p.F251fs | . | . |
| CTC1 | 20 | 35060934 | GC | G | 0.516 | NM_014902 | DLGAP4 | frameshift_deleti  on | exon2 | c.815delC | p.A272fs | . | . |
| CTC1 | 20 | 36937434 | G | GA | 0.362 | NM_001725 | BPI | frameshift_inserti  on | exon3 | c.361dupA | p.G120fs | . | . |
| CTC1 | 20 | 37555322 | C | CGCGG  CG | 0.933 | NM_030919 | FAM83D | nonframeshift_in  sertion | exon1 | c.327_328insG  CGGCG | p.G109delinsG  AA | . | . |
| CTC1 | 20 | 37555322 | C | CGCG | 0.933 | NM_030919 | FAM83D | nonframeshift_in  sertion | exon1 | c.327_328insG  CG | p.G109delinsG  A | . | . |
| CTC1 | 20 | 39725878 | GC | G | 1 | NM_003286 | TOP1 | frameshift_deleti  on | exon10 | c.750delC | p.S250fs | . | . |
| CTC1 | 20 | 40709520 | T | TA | 0.571 | NM_007050 | PTPRT | stoploss | exon31 | c.4324dupT | p.X1442delinsL | . | . |

| CTC1 | 20 | 42089624 | CA | C | 0.833 | NM_006275 | SRSF6 | frameshift_deleti  on | exon6 | c.957delA | p.P319fs | . | . |
| --- | --- | --- | --- | --- | --- | --- | --- | --- | --- | --- | --- | --- | --- |
| CTC1 | 20 | 43851516 | GA | G | 0.381 | NM_003008 | SEMG2 | frameshift_deleti  on | exon2 | c.1244delA | p.E415fs | . | . |
| CTC1 | 20 | 44045264 | TG | T | 0.96 | NM_001184728 | PIGT | frameshift_deleti  on | exon2 | c.296delG | p.W99fs | . | . |
| CTC1 | 20 | 44470489 | CT | C | 0.296 | NM_005469 | ACOT8 | frameshift_deleti  on | exon6 | c.947delA | p.E316fs | . | . |
| CTC1 | 20 | 44519041 | CG | C | 1 | NM_001278535 | NEURL2 | frameshift_deleti  on | exon1 | c.589delC | p.R197fs | . | . |
| CTC1 | 20 | 44520237 | CCTG | C | 0.26 | NM_000308 | CTSA | nonframeshift_de  letion | exon2 | c.85_87del | p.29_29del | . | . |
| CTC1 | 20 | 44979149 | TG | T | 0.333 | NM_001281457 | SLC35C2 | frameshift_deleti  on | exon10 | c.639delC | p.P213fs | . | . |
| CTC1 | 20 | 45131399 | GT | G | 1 | NM_018102 | ZNF334 | frameshift_deleti  on | exon5 | c.578delA | p.N193fs | . | . |
| CTC1 | 20 | 45212284 | GC | G | 0.688 | NM_001193339 | SLC13A3 | frameshift_deleti  on | exon8 | c.995delG | p.G332fs | . | . |
| CTC1 | 20 | 45865227 | GA | G | 0.571 | NM_001281771 | ZMYND8 | frameshift_deleti  on | exon15 | c.2242delT | p.S748fs | . | . |
| CTC1 | 20 | 47246068 | GC | G | 0.21 | NM_020820 | PREX1 | frameshift_deleti  on | exon37 | c.4684delG | p.A1562fs | . | . |
| CTC1 | 20 | 47273614 | CG | C | 0.455 | NM_020820 | PREX1 | frameshift_deleti  on | exon18 | c.2086delC | p.R696fs | . | . |
| CTC1 | 20 | 47711446 | CA | C | 0.389 | NM_001256135 | CSE1L | frameshift_deleti  on | exon23 | c.2605delA | p.K869fs | . | . |
| CTC1 | 20 | 47855517 | GA | G | 1 | NM_017895 | DDX27 | frameshift_deleti  on | exon15 | c.1813delA | p.K605fs | . | . |
| CTC1 | 20 | 48127616 | GC | G | 1 | NM_000961 | PTGIS | frameshift_deleti  on | exon9 | c.1306delG | p.A436fs | . | . |
| CTC1 | 20 | 48127683 | CG | C | 0.385 | NM_000961 | PTGIS | frameshift_deleti  on | exon9 | c.1239delC | p.D413fs | . | . |
| CTC1 | 20 | 48568673 | CA | C | 0.696 | NM_018683 | RNF114 | frameshift_deleti  on | exon6 | c.683delA | p.Q228fs | . | . |
| CTC1 | 20 | 49211153 | TCAG | T | 0.5 | NM_080829 | FAM65C | nonframeshift_de  letion | exon17 | c.2134_2136del | p.712_712del | . | . |
| CTC1 | 20 | 50092086 | TC | T | 0.326 | NM_001136021 | NFATC2 | frameshift_deleti  on | exon4 | c.1383delG | p.G461fs | . | . |
| CTC1 | 20 | 50701760 | AT | A | 0.667 | NM_199427 | ZFP64 | frameshift_deleti  on | exon9 | c.1273delA | p.I425fs | . | . |
| CTC1 | 20 | 52198546 | TTG | T | 0.184 | NM_006526 | ZNF217 | frameshift_deleti  on | exon1 | c.818_819del | p.P273fs | . | . |
| CTC1 | 20 | 52198547 | TG | T | 0.433 | NM_006526 | ZNF217 | frameshift_deleti  on | exon1 | c.818delC | p.P273fs | . | . |
| CTC1 | 20 | 56136614 | TGAG | T | 0.25 | NM_002591 | PCK1 | nonframeshift_de  letion | exon2 | c.148_150del | p.50_50del | . | . |
| CTC1 | 20 | 57769585 | CA | C | 0.6 | NM_178457 | ZNF831 | frameshift_deleti  on | exon1 | c.3512delA | p.Q1171fs | . | . |
| CTC1 | 20 | 58467279 | AT | A | 0.357 | NM_014258 | SYCP2 | frameshift_deleti  on | exon23 | c.2129delA | p.N710fs | . | . |
| CTC1 | 20 | 60882442 | TG | T | 0.468 | NM_001281437 | ADRM1 | frameshift_deleti  on | exon5 | c.441delG | p.L147fs | . | . |
| CTC1 | 20 | 62190693 | A | AG | 0.538 | NM_033405 | HELZ2 | frameshift_inserti  on | exon14 | c.6148dupC | p.L2050fs | . | . |
| CTC1 | 20 | 62595009 | CG | C | 0.31 | NM_020713 | ZNF512B | frameshift_deleti  on | exon10 | c.1659delC | p.A553fs | . | . |
| CTC1 | 20 | 62839748 | GC | G | 0.313 | NM_004535 | MYT1 | frameshift_deleti  on | exon7 | c.1200delC | p.C400fs | . | . |
| CTC1 | 21 | 15535768 | CA | C | 0.8 | NM_198996 | LIPI | frameshift_deleti  on | exon7 | c.1040delT | p.L347fs | . | . |
| CTC1 | 21 | 15746546 | GT | G | 0.357 | NM_006948 | HSPA13 | frameshift_deleti  on | exon5 | c.807delA | p.K269fs | . | . |
| CTC1 | 21 | 17205866 | AG | A | 0.542 | NM_001283041 | USP25 | frameshift_deleti  on | exon17 | c.2194delG | p.A732fs | . | . |
| CTC1 | 21 | 19701585 | CT | C | 0.303 | NM_002772 | TMPRSS15 | frameshift_deleti  on | exon15 | c.1680delA | p.K560fs | . | . |
| CTC1 | 21 | 27372468 | GC | G | 1 | NM_001136130 | APP | frameshift_deleti  on | exon6 | c.726delG | p.G242fs | . | . |
| CTC1 | 21 | 28210345 | CT | C | 0.429 | NM_006988 | ADAMTS1 | frameshift_deleti  on | exon9 | c.2456delA | p.E819fs | . | . |
| CTC1 | 21 | 31913981 | AG | A | 1 | NM_181612 | KRTAP19-6 | frameshift_deleti  on | exon1 | c.171delC | p.F57fs | . | . |
| CTC1 | 21 | 34707842 | TA | T | 0.356 | NM_000629 | IFNAR1 | frameshift_deleti  on | exon2 | c.90delA | p.L30fs | . | . |
| CTC1 | 21 | 34948684 | G | GA | 1 | NM_138927 | SON | frameshift_inserti  on | exon12 | c.7236dupA | p.G2412fs | . | . |
| CTC1 | 21 | 34948696 | GA | G | 1 | NM_138927 | SON | frameshift_deleti  on | exon12 | c.7248delA | p.R2416fs | . | . |
| CTC1 | 21 | 35468452 | A | AT | 0.184 | NM_006933 | SLC5A3 | frameshift_inserti  on | exon2 | c.956dupT | p.I319fs | . | . |
| CTC1 | 21 | 37620850 | AC | A | 0.333 | NM_005128 | DOPEY2 | frameshift_deleti  on | exon21 | c.4983delC | p.Y1661fs | . | . |
| CTC1 | 21 | 38115852 | CA | C | 0.5 | NM_005069 | SIM2 | frameshift_deleti  on | exon9 | c.1164delA | p.P388fs | . | . |
| CTC1 | 21 | 43413625 | GT | G | 1 | NM_020727 | ZBTB21 | frameshift_deleti  on | exon2 | c.579delA | p.K193fs | . | . |
| CTC1 | 21 | 46078114 | CG | C | 0.488 | NM_198697 | KRTAP12-3 | frameshift_deleti  on | exon1 | c.219delG | p.S73fs | . | . |
| CTC1 | 21 | 46951915 | CCAG | C | 0.15 | NM_001205207 | SLC19A1 | nonframeshift_de  letion | exon2 | c.214_216del | p.72_72del | . | . |
| CTC1 | 21 | 47546037 | GA | G | 0.813 | NM_001849 | COL6A2 | frameshift_deleti  on | exon26 | c.2309delA | p.E770fs | . | . |
| CTC1 | 22 | 17688170 | CA | C | 0.553 | NM_001282225 | CECR1 | frameshift_deleti  on | exon3 | c.332delT | p.L111fs | . | . |
| CTC1 | 22 | 18378138 | TC | T | 0.26 | NM_001122731 | MICAL3 | frameshift_deleti  on | exon9 | c.1360delG | p.E454fs | . | . |
| CTC1 | 22 | 19164195 | TG | T | 0.356 | NM_001256534 | SLC25A1 | frameshift_deleti  on | exon6 | c.663delC | p.P221fs | . | . |
| CTC1 | 22 | 19189003 | A | AC | 1 | NM_001835 | CLTCL1 | frameshift_inserti  on | exon23 | c.3601dupG | p.V1201fs | . | . |
| CTC1 | 22 | 20920813 | ACAG | A | 0.236 | NM_001003891 | MED15 | nonframeshift_de  letion | exon7 | c.751_753del | p.251_251del | . | . |
| CTC1 | 22 | 23498201 | TA | T | 0.935 | NM_004914 | RAB36 | frameshift_deleti  on | exon6 | c.575delA | p.Y192fs | . | . |
| CTC1 | 22 | 23964282 | GCAT | G | 1 | NM_016449 | C22orf43 | nonframeshift_de  letion | exon4 | c.377_379del | p.126_127del | . | . |
| CTC1 | 22 | 24622605 | CT | C | 0.455 | NM_001099782 | GGT5 | frameshift_deleti  on | exon6 | c.935delA | p.K312fs | . | . |
| CTC1 | 22 | 26423411 | AT | A | 0.303 | NM_032608 | MYO18B | frameshift_deleti  on | exon43 | c.7472delT | p.I2491fs | . | . |
| CTC1 | 22 | 26709777 | CCA | C | 0.667 | NM_001184773 | SEZ6L | frameshift_deleti  on | exon9 | c.1925_1926del | p.P642fs | . | . |
| CTC1 | 22 | 26709778 | CA | C | 0.9 | NM_001184773 | SEZ6L | frameshift_deleti  on | exon9 | c.1926delA | p.P642fs | . | . |

| CTC1 | 22 | 27026319 | G | GT | 0.355 | NM_001886 | CRYBA4 | frameshift_inserti  on | exon6 | c.460dupT | p.Q153fs | . | . |
| --- | --- | --- | --- | --- | --- | --- | --- | --- | --- | --- | --- | --- | --- |
| CTC1 | 22 | 29091132 | GC | G | 0.455 | NM_145862 | CHEK2 | frameshift_deleti  on | exon11 | c.1270delG | p.A424fs | . | . |
| CTC1 | 22 | 29695775 | C | CA | 0.167 | NM_001163286 | EWSR1 | frameshift_inserti  on | exon15 | c.1697_1698ins  A | p.P566fs | . | . |
| CTC1 | 22 | 30734788 | CG | C | 0.303 | NM_001005409 | SF3A1 | frameshift_deleti  on | exon11 | c.1537delC | p.R513fs | . | . |
| CTC1 | 22 | 30975826 | GT | G | 0.231 | NM_001243225 | PES1 | frameshift_deleti  on | exon12 | c.1250delA | p.H417fs | . | . |
| CTC1 | 22 | 31522376 | C | CA | 0.449 | NM_001002837 | INPP5J | frameshift_inserti  on | exon3 | c.183dupA | p.T61fs | . | . |
| CTC1 | 22 | 31529190 | G | GA | 0.17 | NM_001284287 | INPP5J | frameshift_inserti  on | exon9 | c.995_996insA | p.R332fs | . | . |
| CTC1 | 22 | 31671291 | GC | G | 0.608 | NM_001031801 | LIMK2 | frameshift_deleti  on | exon14 | c.1707delC | p.S569fs | . | . |
| CTC1 | 22 | 32111250 | C | CT | 1 | NM_173566 | PRR14L | frameshift_inserti  on | exon4 | c.2574_2575ins  A | p.D859fs | . | . |
| CTC1 | 22 | 32215089 | TG | T | 0.288 | NM_001136029 | DEPDC5 | frameshift_deleti  on | exon21 | c.1749delG | p.M583fs | . | . |
| CTC1 | 22 | 32293532 | CT | C | 0.737 | NM_001242897 | DEPDC5 | frameshift_deleti  on | exon37 | c.3969delT | p.P1323fs | . | . |
| CTC1 | 22 | 36122937 | AG | A | 0.316 | NM_030642 | APOL5 | frameshift_deleti  on | exon3 | c.823delG | p.G275fs | . | . |
| CTC1 | 22 | 38120175 | GCCT | G | 0.255 | NM_001039141 | TRIOBP | nonframeshift_de  letion | exon7 | c.1613_1615del | p.538_539del | . | . |
| CTC1 | 22 | 38823129 | GA | G | 0.971 | NM_004981 | KCNJ4 | frameshift_deleti  on | exon2 | c.1008delT | p.F336fs | . | . |
| CTC1 | 22 | 39530416 | A | AG | 0.647 | NM_175709 | CBX7 | frameshift_inserti  on | exon5 | c.587dupC | p.P196fs | . | . |
| CTC1 | 22 | 40055454 | AT | A | 1 | NM_001003406 | CACNA1I | frameshift_deleti  on | exon12 | c.2243delT | p.I748fs | . | . |
| CTC1 | 22 | 40361999 | A | AT | 0.25 | NM_004810 | GRAP2 | frameshift_inserti  on | exon5 | c.296_297insT | p.E99fs | . | . |
| CTC1 | 22 | 41175086 | GT | G | 0.417 | NM_001282727 | SLC25A17 | frameshift_deleti  on | exon3 | c.158delA | p.N53fs | . | . |
| CTC1 | 22 | 41551048 | AC | A | 0.518 | NM_001429 | EP300 | frameshift_deleti  on | exon17 | c.3193delC | p.L1065fs | . | . |
| CTC1 | 22 | 41921254 | C | CA | 0.319 | NM_001098 | ACO2 | frameshift_inserti  on | exon14 | c.1664dupA | p.H555fs | . | . |
| CTC1 | 22 | 42128546 | GT | G | 0.31 | NM_152513 | MEI1 | frameshift_deleti  on | exon11 | c.1271delT | p.V424fs | . | . |
| CTC1 | 22 | 42301464 | GGCCAG  CGAGAG CGGGCC | G | 0.914 | NM_004599 | SREBF2 | frameshift_deleti on | exon19 | c.3227_3249del | p.G1076fs | . | . |
| CTC1 | 22 | 42457065 | AT | A | 0.778 | NM_000262 | NAGA | frameshift_deleti  on | exon8 | c.963delA | p.K321fs | . | . |
| CTC1 | 22 | 42478031 | AT | A | 0.433 | NM_033318 | SMDT1 | frameshift_deleti  on | exon2 | c.290delT | p.I97fs | . | . |
| CTC1 | 22 | 42605951 | CT | C | 0.314 | NM_005650 | TCF20 | frameshift_deleti  on | exon1 | c.5360delA | p.K1787fs | . | . |
| CTC1 | 22 | 43614455 | TCG | T | 0.125 | NM_173050 | SCUBE1 | frameshift_deleti  on | exon15 | c.1695_1696del | p.C565fs | . | . |
| CTC1 | 22 | 44168845 | C | CT | 0.941 | NM_022785 | EFCAB6 | frameshift_inserti  on | exon4 | c.277dupA | p.S93fs | . | . |
| CTC1 | 22 | 45798220 | CT | C | 1 | NM_148674 | SMC1B | frameshift_deleti  on | exon5 | c.846delA | p.K282fs | . | . |
| CTC1 | 22 | 46327098 | G | GC | 0.239 | NM_058238 | WNT7B | frameshift_inserti  on | exon3 | c.449dupG | p.G150fs | . | . |
| CTC1 | 22 | 47193432 | TCTCAG  CAG | T | 0.24 | NM_001284305 | TBC1D22A | frameshift_deleti  on | exon4 | c.412_419del | p.L138fs | . | . |
| CTC1 | 22 | 50217020 | TCA | T | 0.239 | NM_014577 | BRD1 | frameshift_deleti  on | exon1 | c.944_945del | p.V315fs | . | . |
| CTC1 | 22 | 50280309 | CG | C | 0.667 | NM_014838 | ZBED4 | frameshift_deleti  on | exon2 | c.3000delG | p.P1000fs | . | . |
| CTC1 | 22 | 50658131 | CA | C | 0.408 | NM_020461 | TUBGCP6 | frameshift_deleti  on | exon18 | c.4256delT | p.L1419fs | . | . |
| CTC1 | 22 | 51142341 | TACG | T | 0.698 | NM_033517 | SHANK3 | nonframeshift_de  letion | exon13 | c.1625_1627del | p.542_543del | . | . |
| CTC1 | X | 1505522 | CA | C | 0.333 | NM_001636 | SLC25A6 | frameshift_deleti  on | exon4 | c.869delT | p.L290fs | . | . |
| CTC1 | X | 2825313 | CA | C | 0.424 | NM_001669 | ARSD | stoploss | exon10 | c.1780delT | p.X594E | . | . |
| CTC1 | X | 2998964 | CA | C | 0.443 | NM_001201538 | ARSF | frameshift_deleti on | exon5 | c.317delA | p.Q106fs | . | . |
| CTC1 | X | 9900662 | CA | C | 0.204 | NM_001649 | SHROOM2 | frameshift_deleti  on | exon6 | c.3340delA | p.S1114fs | . | . |
| CTC1 | X | 10104720 | C | CA | 0.571 | NM_015691 | WWC3 | frameshift_inserti  on | exon20 | c.2811_2812ins  A | p.P937fs | . | . |
| CTC1 | X | 11316378 | AC | A | 0.229 | NM_182681 | AMELX | frameshift_deleti  on | exon3 | c.78delC | p.Y26fs | . | . |
| CTC1 | X | 15826364 | GC | G | 0.667 | NM_005089 | ZRSR2 | frameshift_deleti  on | exon6 | c.409delC | p.Q137fs | . | . |
| CTC1 | X | 16857958 | T | TA | 0.439 | NM_001168683 | TXLNG | frameshift_inserti  on | exon7 | c.772dupA | p.I257fs | . | . |
| CTC1 | X | 16876814 | GT | G | 0.3 | NM_001198719 | RBBP7 | frameshift_deleti  on | exon4 | c.597delA | p.K199fs | . | . |
| CTC1 | X | 17745554 | GT | G | 0.85 | NM_198270 | NHS | frameshift_deleti  on | exon6 | c.3266delT | p.V1089fs | . | . |
| CTC1 | X | 18264711 | TG | T | 0.727 | NM_006089 | SCML2 | frameshift_deleti  on | exon13 | c.1807delC | p.Q603fs | . | . |
| CTC1 | X | 18323099 | TC | T | 0.333 | NM_006089 | SCML2 | frameshift_deleti  on | exon7 | c.722delG | p.G241fs | . | . |
| CTC1 | X | 18668686 | C | CT | 0.294 | NM_003159 | CDKL5 | frameshift_inserti  on | exon20 | c.2955dupT | p.A985fs | . | . |
| CTC1 | X | 19416400 | TCG | T | 0.9 | NM_001001671 | MAP3K15 | frameshift_deleti  on | exon15 | c.2008_2009del | p.R670fs | . | . |
| CTC1 | X | 19425384 | TC | T | 0.232 | NM_001001671 | MAP3K15 | frameshift_deleti  on | exon13 | c.1765delG | p.E589fs | . | . |
| CTC1 | X | 20195175 | CA | C | 0.75 | NM_004586 | RPS6KA3 | stopgain | exon11 | c.872delT | p.L291X | . | . |
| CTC1 | X | 20211610 | TG | T | 1 | NM_004586 | RPS6KA3 | frameshift_deleti on | exon7 | c.587delC | p.P196fs | . | . |
| CTC1 | X | 24512986 | A | AT | 0.222 | NM_001142386 | PDK3 | frameshift_inserti  on | exon2 | c.235dupT | p.G78fs | . | . |
| CTC1 | X | 29973278 | AC | A | 0.583 | NM_014271 | IL1RAPL1 | frameshift_deleti  on | exon11 | c.1433delC | p.T478fs | . | . |
| CTC1 | X | 29973335 | CG | C | 0.909 | NM_014271 | IL1RAPL1 | frameshift_deleti  on | exon11 | c.1490delG | p.R497fs | . | . |
| CTC1 | X | 34148137 | G | GA | 0.157 | NM_203408 | FAM47A | frameshift_inserti  on | exon1 | c.2258dupT | p.I753fs | . | . |
| CTC1 | X | 36103479 | GT | G | 0.462 | NM_173695 | CHDC2 | frameshift_deleti  on | exon5 | c.466delT | p.F156fs | . | . |
| CTC1 | X | 36397516 | T | TG | 1 | NM_001098843 | CXorf30 | frameshift_inserti  on | exon18 | c.1575_1576ins  G | p.F525fs | . | . |

| CTC1 | X | 38079975 | CGCA | C | 0.135 | NM_001170750 | SRPX | nonframeshift_de  letion | exon1 | c.68_70del | p.23_24del | . | . |
| --- | --- | --- | --- | --- | --- | --- | --- | --- | --- | --- | --- | --- | --- |
| CTC1 | X | 38146164 | TA | T | 1 | NM_001034853 | RPGR | frameshift_deleti  on | exon15 | c.2087delT | p.L696fs | . | . |
| CTC1 | X | 46712918 | CA | C | 0.5 | NM_006915 | RP2 | frameshift_deleti  on | exon2 | c.111delA | p.P37fs | . | . |
| CTC1 | X | 46845159 | CA | C | 0.889 | NM_001077445 | JADE3 | frameshift_deleti  on | exon3 | c.93delA | p.S31fs | . | . |
| CTC1 | X | 47030560 | GGGA | G | 0.238 | NM_001204467 | RBM10 | nonframeshift_de  letion | exon4 | c.336_338del | p.112_113del | . | . |
| CTC1 | X | 47272602 | AG | A | 0.441 | NM_003446 | ZNF157 | frameshift_deleti  on | exon4 | c.1131delG | p.Q377fs | . | . |
| CTC1 | X | 48661165 | GC | G | 0.533 | NM_006044 | HDAC6 | frameshift_deleti  on | exon2 | c.67delC | p.P23fs | . | . |
| CTC1 | X | 48887805 | CCCT | C | 0.533 | NM_001282142 | TFE3 | nonframeshift_de  letion | exon10 | c.1274_1276del | p.425_426del | . | . |
| CTC1 | X | 48895540 | TG | T | 0.85 | NM_001282142 | TFE3 | frameshift_deleti  on | exon5 | c.564delC | p.P188fs | . | . |
| CTC1 | X | 49855081 | AC | A | 0.182 | NM_000084 | CLCN5 | frameshift_deleti  on | exon10 | c.1844delC | p.T615fs | . | . |
| CTC1 | X | 49856807 | CA | C | 0.473 | NM_000084 | CLCN5 | frameshift_deleti  on | exon12 | c.2173delA | p.K725fs | . | . |
| CTC1 | X | 50376436 | AT | A | 0.34 | NM_020717 | SHROOM4 | frameshift_deleti  on | exon4 | c.2636delA | p.D879fs | . | . |
| CTC1 | X | 50377103 | CT | C | 0.479 | NM_020717 | SHROOM4 | frameshift_deleti  on | exon4 | c.1969delA | p.S657fs | . | . |
| CTC1 | X | 53106644 | GA | G | 1 | NM_018969 | GPR173 | frameshift_deleti  on | exon2 | c.842delA | p.E281fs | . | . |
| CTC1 | X | 53279832 | CG | C | 0.478 | NM_001111125 | IQSEC2 | frameshift_deleti  on | exon5 | c.1925delC | p.P642fs | . | . |
| CTC1 | X | 53409489 | CA | C | 0.233 | NM_006306 | SMC1A | frameshift_deleti  on | exon21 | c.3222delT | p.F1074fs | . | . |
| CTC1 | X | 53578325 | AG | A | 0.517 | NM_031407 | HUWE1 | frameshift_deleti  on | exon64 | c.8997delC | p.P2999fs | . | . |
| CTC1 | X | 54838676 | AC | A | 0.667 | NM_014599 | MAGED2 | frameshift_deleti  on | exon7 | c.1078delC | p.L360fs | . | . |
| CTC1 | X | 55033806 | C | CG | 0.611 | NM_001271748 | APEX2 | frameshift_inserti  on | exon5 | c.983dupG | p.R328fs | . | . |
| CTC1 | X | 57146292 | CT | C | 0.656 | NM_001006681 | SPIN2B | frameshift_deleti  on | exon2 | c.770delA | p.K257fs | . | . |
| CTC1 | X | 57620132 | AT | A | 1 | NM_007157 | ZXDB | frameshift_deleti  on | exon1 | c.1652delT | p.I551fs | . | . |
| CTC1 | X | 69263505 | T | TG | 0.32 | NM_001002254 | AWAT2 | frameshift_inserti  on | exon4 | c.294dupC | p.S99fs | . | . |
| CTC1 | X | 70320696 | CGG | C | 0.273 | NM_005938 | FOXO4 | frameshift_deleti  on | exon2 | c.617_618del | p.R206fs | . | . |
| CTC1 | X | 70627470 | C | CG | 0.308 | NM_001286074 | TAF1 | frameshift_inserti  on | exon27 | c.4215dupG | p.T1405fs | . | . |
| CTC1 | X | 71426475 | AT | A | 0.297 | NM_017669 | ERCC6L | frameshift_deleti  on | exon2 | c.2141delA | p.N714fs | . | . |
| CTC1 | X | 72433207 | GA | G | 0.562 | NM_021963 | NAP1L2 | frameshift_deleti  on | exon1 | c.1121delT | p.F374fs | . | . |
| CTC1 | X | 74517343 | GT | G | 0.833 | NM_145052 | UPRT | frameshift_deleti  on | exon4 | c.518delT | p.V173fs | . | . |
| CTC1 | X | 76711870 | TA | T | 0.404 | NM_003868 | FGF16 | frameshift_deleti  on | exon2 | c.209delA | p.Y70fs | . | . |
| CTC1 | X | 76845309 | TA | T | 0.389 | NM_138270 | ATRX | frameshift_deleti  on | exon26 | c.6097delT | p.Y2033fs | . | . |
| CTC1 | X | 78426605 | TCA | T | 0.857 | NM_032553 | GPR174 | frameshift_deleti  on | exon1 | c.102_103del | p.L34fs | . | . |
| CTC1 | X | 79971677 | C | CT | 0.667 | NM_153252 | BRWD3 | frameshift_inserti  on | exon20 | c.2303dupA | p.K768fs | . | . |
| CTC1 | X | 84362346 | CA | C | 0.357 | NM_001012980 | SATL1 | frameshift_deleti  on | exon1 | c.1628delT | p.L543fs | . | . |
| CTC1 | X | 84519402 | AT | A | 0.375 | NM_021998 | ZNF711 | frameshift_deleti  on | exon5 | c.745delT | p.F249fs | . | . |
| CTC1 | X | 92964605 | GA | G | 0.957 | NM_001171111 | FAM133A | frameshift_deleti  on | exon3 | c.188delA | p.E63fs | . | . |
| CTC1 | X | 99922321 | TTC | T | 0.176 | NM_014467 | SRPX2 | frameshift_deleti  on | exon9 | c.1013_1014del | p.F338fs | . | . |
| CTC1 | X | 100548021 | CG | C | 0.643 | NM_024885 | TAF7L | frameshift_deleti  on | exon1 | c.12delC | p.P4fs | . | . |
| CTC1 | X | 100747413 | TG | T | 0.3 | NM_001256155 | ARMCX4 | frameshift_deleti  on | exon2 | c.3838delG | p.G1280fs | . | . |
| CTC1 | X | 100808018 | GA | G | 0.286 | NM_016608 | ARMCX1 | frameshift_deleti  on | exon4 | c.106delA | p.K36fs | . | . |
| CTC1 | X | 101910948 | A | AT | 0.75 | NM_001099411 | GPRASP1 | frameshift_inserti  on | exon3 | c.2108dupT | p.I703fs | . | . |
| CTC1 | X | 101911860 | TG | T | 0.278 | NM_001099411 | GPRASP1 | frameshift_deleti  on | exon3 | c.3020delG | p.W1007fs | . | . |
| CTC1 | X | 105970297 | CG | C | 0.333 | NM_194463 | RNF128 | frameshift_deleti  on | exon1 | c.155delG | p.R52fs | . | . |
| CTC1 | X | 106808267 | CG | C | 0.5 | NM_032428 | FRMPD3 | frameshift_deleti  on | exon13 | c.1367delG | p.R456fs | . | . |
| CTC1 | X | 107408188 | CA | C | 0.583 | NM_001287759 | COL4A6 | frameshift_deleti  on | exon38 | c.3816delT | p.P1272fs | . | . |
| CTC1 | X | 110987953 | TACC | T | 0.4 | NM_001099922 | ALG13 | nonframeshift_de  letion | exon24 | c.2754_2756del | p.918_919del | . | . |
| CTC1 | X | 112066049 | AT | A | 0.49 | NM_001113490 | AMOT | frameshift_deleti  on | exon1 | c.305delA | p.N102fs | . | . |
| CTC1 | X | 114357141 | TC | T | 0.5 | NM_001243963 | LRCH2 | frameshift_deleti  on | exon19 | c.2075delG | p.R692fs | . | . |
| CTC1 | X | 114419072 | AT | A | 0.682 | NM_001243963 | LRCH2 | frameshift_deleti  on | exon3 | c.522delA | p.K174fs | . | . |
| CTC1 | X | 117960317 | TG | T | 0.37 | NM_173798 | ZCCHC12 | frameshift_deleti  on | exon4 | c.1111delG | p.E371fs | . | . |
| CTC1 | X | 119387833 | C | CTGA | 0.885 | NM_006777 | ZBTB33 | nonframeshift_in  sertion | exon2 | c.563_564insTG  A | p.S188delinsSD | . | . |
| CTC1 | X | 119760761 | CT | C | 0.8 | NM_001011551 | C1GALT1C1 | frameshift_deleti  on | exon2 | c.260delA | p.K87fs | . | . |
| CTC1 | X | 122336600 | T | TG | 1 | NM_001256743 | GRIA3 | frameshift_inserti  on | exon3 | c.382dupG | p.G127fs | . | . |
| CTC1 | X | 122830579 | TG | T | 1 | NM_001081550 | THOC2 | frameshift_deleti  on | exon6 | c.458delC | p.T153fs | . | . |
| CTC1 | X | 123185197 | AT | A | 0.19 | NM_006603 | STAG2 | frameshift_deleti  on | exon12 | c.1150delT | p.Y384fs | . | . |
| CTC1 | X | 123615698 | TC | T | 0.361 | NM_014253 | TENM1 | frameshift_deleti  on | exon21 | c.3811delG | p.D1271fs | . | . |
| CTC1 | X | 128709883 | GA | G | 0.487 | NM_000276 | OCRL | frameshift_deleti  on | exon17 | c.1724delA | p.E575fs | . | . |
| CTC1 | X | 134033384 | TA | T | 1 | NM_019556 | MOSPD1 | frameshift_deleti  on | exon2 | c.79delT | p.Y27fs | . | . |
| CTC1 | X | 135430309 | TC | T | 0.273 | NM_153834 | GPR112 | frameshift_deleti  on | exon6 | c.4445delC | p.S1482fs | . | . |

| CTC1 | X | 135432131 | CT | C | 0.625 | NM_153834 | GPR112 | frameshift_deleti  on | exon6 | c.6267delT | p.P2089fs | . | . |
| --- | --- | --- | --- | --- | --- | --- | --- | --- | --- | --- | --- | --- | --- |
| CTC1 | X | 135453651 | GT | G | 0.306 | NM_153834 | GPR112 | frameshift_deleti  on | exon14 | c.7562delT | p.V2521fs | . | . |
| CTC1 | X | 135474444 | AGAT | A | 1 | NM_153834 | GPR112 | nonframeshift_de  letion | exon17 | c.7966_7968del | p.2656_2656de  l | . | . |
| CTC1 | X | 135574194 | A | AT | 0.75 | NM_001727 | BRS3 | frameshift_inserti  on | exon3 | c.861dupT | p.N287fs | . | . |
| CTC1 | X | 135630889 | TG | T | 0.277 | NM_016267 | VGLL1 | frameshift_deleti  on | exon3 | c.357delG | p.L119fs | . | . |
| CTC1 | X | 135961585 | A | ATT | 0.459 | NM_001164803 | RBMX | frameshift_inserti  on | exon2 | c.1_2insAA | p.M1fs | . | . |
| CTC1 | X | 135961585 | ATG | A | 0.459 | NM_002139 | RBMX | frameshift_deleti  on | wholegen  e,RBMX | NM_001164803 | wholegene | . | . |
| CTC1 | X | 148069065 | C | CA | 0.214 | NM_001170628 | AFF2 | frameshift_inserti  on | exon17 | c.2716dupA | p.D905fs | . | . |
| CTC1 | X | 149639324 | ACAG | A | 0.375 | NM_001177466 | MAMLD1 | nonframeshift_de  letion | exon2 | c.1405_1407del | p.469_469del | . | . |
| CTC1 | X | 150349557 | CCACCA  CTGGCC | C | 1 | NM_004224 | GPR50 | nonframeshift_de  letion | exon2 | c.1503_1514del | p.501_505del | . | . |
| CTC1 | X | 152087569 | CGAG | C | 0.159 | NM_001178113 | ZNF185 | nonframeshift_de  letion | exon2 | c.70_72del | p.24_24del | . | . |
| CTC1 | X | 152137988 | TAG | T | 0.219 | NM_001178115 | ZNF185 | frameshift_deleti  on | exon7 | c.846_847del | p.L282fs | . | . |
| CTC1 | X | 152773851 | TC | T | 0.771 | NM_001711 | BGN | frameshift_deleti  on | exon8 | c.1056delC | p.F352fs | . | . |
| CTC1 | X | 153592420 | TC | T | 0.989 | NM_001110556 | FLNA | frameshift_deleti  on | exon15 | c.2249delG | p.G750fs | . | . |
| CTC1 | X | 154005088 | CAAG | C | 0.333 | NM_001142463 | DKC1 | nonframeshift_de  letion | exon15 | c.1477_1479del | p.493_493del | . | . |
| CTC1 | X | 154158106 | GT | G | 0.206 | NM_000132 | F8 | frameshift_deleti  on | exon14 | c.3958delA | p.T1320fs | . | . |
| CTC1 | X | 155239798 | GAGC | G | 0.4 | NM_002186 | IL9R | nonframeshift_de  letion | exon9 | c.1291_1293del | p.431_431del | . | . |
| CTC1 | Y | 2655346 | AT | A | 0.226 | NM_003140 | SRY | frameshift_deleti  on | exon1 | c.298delA | p.M100fs | . | . |
| CTC2 | 1 | 1650797 | AT | A | 0.611 | NM_024011 | CDK11A | frameshift_deleti  on | exon4 | c.324delA | p.K108fs | . | . |
| CTC2 | 1 | 2252895 | TCC | T | 0.231 | NM_024848 | MORN1 | frameshift_deleti  on | exon14 | c.1419_1420del | p.L473fs | . | . |
| CTC2 | 1 | 2252954 | CG | C | 0.524 | NM_024848 | MORN1 | frameshift_deleti  on | exon14 | c.1361delC | p.P454fs | . | . |
| CTC2 | 1 | 2440361 | CT | C | 1 | NM_018216 | PANK4 | frameshift_deleti  on | exon19 | c.2246delA | p.K749fs | . | . |
| CTC2 | 1 | 3424399 | GCA | G | 0.211 | NM_001409 | MEGF6 | frameshift_deleti  on | exon14 | c.1747_1748del | p.C583fs | . | . |
| CTC2 | 1 | 3645919 | TG | T | 0.286 | NM_001126240 | TP73 | frameshift_deleti  on | exon8 | c.957delG | p.L319fs | . | . |
| CTC2 | 1 | 3645985 | GGCA | G | 0.588 | NM_001126240 | TP73 | nonframeshift_de  letion | exon8 | c.1023_1025del | p.341_342del | . | . |
| CTC2 | 1 | 6512130 | CAGG | C | 0.3 | NM_031475 | ESPN | nonframeshift_de  letion | exon10 | c.2300_2302del | p.767_768del | . | . |
| CTC2 | 1 | 6529182 | TTCC | T | 0.333 | NM_001042664 | PLEKHG5 | nonframeshift_de  letion | exon19 | c.2166_2168del | p.722_723del | . | . |
| CTC2 | 1 | 6680014 | TG | T | 0.912 | NM_153812 | PHF13 | frameshift_deleti  on | exon3 | c.294delG | p.L98fs | . | . |
| CTC2 | 1 | 6680068 | TGAA | T | 0.157 | NM_153812 | PHF13 | nonframeshift_de  letion | exon3 | c.348_350del | p.116_117del | . | . |
| CTC2 | 1 | 7796418 | TG | T | 0.569 | NM_015215 | CAMTA1 | frameshift_deleti  on | exon13 | c.3082delG | p.G1028fs | . | . |
| CTC2 | 1 | 7798213 | GA | G | 0.571 | NM_015215 | CAMTA1 | frameshift_deleti  on | exon16 | c.3854delA | p.E1285fs | . | . |
| CTC2 | 1 | 7798325 | AT | A | 1 | NM_015215 | CAMTA1 | frameshift_deleti  on | exon16 | c.3966delT | p.N1322fs | . | . |
| CTC2 | 1 | 8390377 | TC | T | 0.994 | NM_001080397 | SLC45A1 | frameshift_deleti  on | exon4 | c.825delC | p.V275fs | . | . |
| CTC2 | 1 | 9009345 | TG | T | 0.487 | NM_001215 | CA6 | frameshift_deleti  on | exon2 | c.104delG | p.W35fs | . | . |
| CTC2 | 1 | 9165578 | CG | C | 0.434 | NM_024980 | GPR157 | frameshift_deleti  on | exon3 | c.758delC | p.P253fs | . | . |
| CTC2 | 1 | 10473278 | C | CA | 0.5 | NM_002631 | PGD | frameshift_inserti  on | exon8 | c.814_815insA | p.L272fs | . | . |
| CTC2 | 1 | 10703339 | AG | A | 1 | NM_001079843 | CASZ1 | frameshift_deleti  on | exon19 | c.3897delC | p.H1299fs | . | . |
| CTC2 | 1 | 11008623 | CG | C | 0.804 | NM_001170754 | C1orf127 | frameshift_deleti  on | exon12 | c.1568delC | p.P523fs | . | . |
| CTC2 | 1 | 11291088 | TA | T | 0.341 | NM_004958 | MTOR | stopgain | exon18 | c.2672delT | p.L891X | . | . |
| CTC2 | 1 | 11579531 | TC | T | 0.323 | NM_020780 | PTCHD2 | frameshift_deleti on | exon8 | c.2010delC | p.I670fs | . | . |
| CTC2 | 1 | 12321138 | CA | C | 0.714 | NM_015378 | VPS13D | frameshift_deleti  on | exon12 | c.1347delA | p.P449fs | . | . |
| CTC2 | 1 | 12463960 | CA | C | 0.375 | NM_018156 | VPS13D | frameshift_deleti  on | exon62 | c.11890delA | p.K3964fs | . | . |
| CTC2 | 1 | 12885310 | CA | C | 1 | NM_001146344 | PRAMEF11 | frameshift_deleti  on | exon4 | c.800delT | p.L267fs | . | . |
| CTC2 | 1 | 12939575 | GT | G | 0.351 | NM_001009611 | PRAMEF4 | frameshift_deleti  on | exon4 | c.1226delA | p.N409fs | . | . |
| CTC2 | 1 | 14106394 | A | ACTC | 0.637 | NM_001007257 | PRDM2 | nonframeshift_in  sertion | exon3 | c.1501_1502ins  CTC | p.T501delinsTP | . | . |
| CTC2 | 1 | 14106619 | AG | A | 0.589 | NM_001007257 | PRDM2 | frameshift_deleti  on | exon3 | c.1727delG | p.S576fs | . | . |
| CTC2 | 1 | 15668354 | C | CA | 0.667 | NM_052929 | FHAD1 | frameshift_inserti  on | exon15 | c.2030dupA | p.Q677fs | . | . |
| CTC2 | 1 | 16257033 | CT | C | 0.444 | NM_015001 | SPEN | frameshift_deleti  on | exon11 | c.4299delT | p.S1433fs | . | . |
| CTC2 | 1 | 16259739 | CA | C | 0.345 | NM_015001 | SPEN | frameshift_deleti  on | exon11 | c.7005delA | p.T2335fs | . | . |
| CTC2 | 1 | 16260574 | CA | C | 0.548 | NM_015001 | SPEN | frameshift_deleti  on | exon11 | c.7840delA | p.I2614fs | . | . |
| CTC2 | 1 | 16719764 | AG | A | 0.23 | NM_001114600 | SZRD1 | frameshift_deleti  on | exon3 | c.144delG | p.Q48fs | . | . |
| CTC2 | 1 | 16725972 | GC | G | 0.304 | NM_198546 | SPATA21 | frameshift_deleti  on | exon12 | c.1218delG | p.L406fs | . | . |
| CTC2 | 1 | 16774451 | CG | C | 0.228 | NM_001145277 | NECAP2 | frameshift_deleti  on | exon3 | c.281delG | p.R94fs | . | . |
| CTC2 | 1 | 17085514 | GCCCCA  GGGTTA GGGCCC TGGCGG GGCCGG | G | 0.509 | NM_001271733 | MST1L | frameshift_deleti on | exon10 | c.1161_1176del | p.Q387fs | . | . |

| CTC2 | 1 | 17715314 | C | CT | 1 | NM_207421 | PADI6 | frameshift_inserti  on | exon8 | c.901_902insT | p.R301fs | . | . |
| --- | --- | --- | --- | --- | --- | --- | --- | --- | --- | --- | --- | --- | --- |
| CTC2 | 1 | 18152725 | GC | G | 0.941 | NM_030812 | ACTL8 | frameshift_deleti  on | exon3 | c.813delC | p.S271fs | . | . |
| CTC2 | 1 | 19453097 | GC | G | 0.367 | NM_020765 | UBR4 | frameshift_deleti  on | exon64 | c.9280delG | p.A3094fs | . | . |
| CTC2 | 1 | 19524456 | TA | T | 0.337 | NM_020765 | UBR4 | frameshift_deleti  on | exon6 | c.735delT | p.A245fs | . | . |
| CTC2 | 1 | 19567592 | AC | A | 1 | NM_001271429 | EMC1 | frameshift_deleti  on | exon5 | c.487delG | p.V163fs | . | . |
| CTC2 | 1 | 20082248 | CG | C | 0.6 | NM_181719 | TMCO4 | frameshift_deleti  on | exon7 | c.393delC | p.D131fs | . | . |
| CTC2 | 1 | 21221902 | GT | G | 0.714 | NM_003760 | EIF4G3 | frameshift_deleti  on | exon12 | c.1923delA | p.K641fs | . | . |
| CTC2 | 1 | 22084244 | AC | A | 0.205 | NM_001032730 | USP48 | frameshift_deleti  on | exon2 | c.166delG | p.V56fs | . | . |
| CTC2 | 1 | 22139002 | GC | G | 1 | NM_001013693 | LDLRAD2 | frameshift_deleti  on | exon1 | c.59delC | p.A20fs | . | . |
| CTC2 | 1 | 23419869 | C | CA | 0.185 | NM_001142546 | LUZP1 | stopgain | exon3 | c.885dupT | p.E296_K297de  linsX | . | . |
| CTC2 | 1 | 24078340 | GC | G | 0.75 | NM_003198 | TCEB3 | frameshift_deleti  on | exon4 | c.1324delC | p.P442fs | . | . |
| CTC2 | 1 | 24397608 | GGC | G | 0.435 | NM_152372 | MYOM3 | frameshift_deleti  on | exon25 | c.3147_3148del | p.S1049fs | . | . |
| CTC2 | 1 | 26150180 | GT | G | 0.353 | NM_001099625 | MTFR1L | frameshift_deleti  on | exon3 | c.71delT | p.V24fs | . | . |
| CTC2 | 1 | 26356976 | GC | G | 0.444 | NM_004455 | EXTL1 | frameshift_deleti  on | exon4 | c.992delC | p.A331fs | . | . |
| CTC2 | 1 | 26517791 | TG | T | 1 | NM_198137 | CATSPER4 | frameshift_deleti  on | exon2 | c.228delG | p.M76fs | . | . |
| CTC2 | 1 | 26595119 | GC | G | 0.796 | NM_001281517 | CEP85 | frameshift_deleti  on | exon7 | c.1335delC | p.S445fs | . | . |
| CTC2 | 1 | 26629328 | A | AG | 0.408 | NM_001077262 | UBXN11 | frameshift_inserti  on | exon2 | c.46dupC | p.L16fs | . | . |
| CTC2 | 1 | 26688268 | AG | A | 0.4 | NM_001114759 | ZNF683 | frameshift_deleti  on | exon6 | c.1388delC | p.S463fs | . | . |
| CTC2 | 1 | 27100114 | GC | G | 0.588 | NM_006015 | ARID1A | frameshift_deleti  on | exon16 | c.3911delC | p.A1304fs | . | . |
| CTC2 | 1 | 27268295 | TC | T | 0.667 | NM_006600 | NUDC | frameshift_deleti  on | exon4 | c.416delC | p.S139fs | . | . |
| CTC2 | 1 | 27428567 | CT | C | 0.561 | NM_003047 | SLC9A1 | frameshift_deleti  on | exon9 | c.1874delA | p.K625fs | . | . |
| CTC2 | 1 | 27679873 | CT | C | 0.818 | NM_001193308 | SYTL1 | frameshift_deleti  on | exon14 | c.1444delT | p.F482fs | . | . |
| CTC2 | 1 | 29314081 | AG | A | 0.238 | NM_001166005 | EPB41 | frameshift_deleti  on | exon2 | c.133delG | p.G45fs | . | . |
| CTC2 | 1 | 32127261 | CG | C | 0.241 | NM_001856 | COL16A1 | frameshift_deleti  on | exon59 | c.3725delC | p.P1242fs | . | . |
| CTC2 | 1 | 32257860 | GC | G | 0.983 | NM_001281988 | SPOCD1 | frameshift_deleti  on | exon14 | c.1396delG | p.A466fs | . | . |
| CTC2 | 1 | 32692097 | AT | A | 1 | NM_003757 | EIF3I | frameshift_deleti  on | exon6 | c.495delT | p.H165fs | . | . |
| CTC2 | 1 | 33276593 | TC | T | 0.569 | NM_003680 | YARS | frameshift_deleti  on | exon2 | c.122delG | p.G41fs | . | . |
| CTC2 | 1 | 33291919 | TG | T | 0.875 | NM_001256121 | S100PBP | frameshift_deleti  on | exon3 | c.220delG | p.G74fs | . | . |
| CTC2 | 1 | 35251063 | AG | A | 0.277 | NM_001005752 | GJB3 | frameshift_deleti  on | exon2 | c.701delG | p.S234fs | . | . |
| CTC2 | 1 | 35824813 | G | GA | 0.407 | NM_005095 | ZMYM4 | frameshift_inserti  on | exon3 | c.374dupA | p.E125fs | . | . |
| CTC2 | 1 | 36055613 | GC | G | 1 | NM_178548 | TFAP2E | frameshift_deleti  on | exon5 | c.869delC | p.A290fs | . | . |
| CTC2 | 1 | 36769459 | C | CT | 0.308 | NM_005119 | THRAP3 | frameshift_inserti  on | exon12 | c.2710dupT | p.A903fs | . | . |
| CTC2 | 1 | 36935370 | TG | T | 0.667 | NM_000760 | CSF3R | frameshift_deleti  on | exon11 | c.1356delC | p.P452fs | . | . |
| CTC2 | 1 | 39340689 | TC | T | 0.24 | NM_030772 | GJA9 | frameshift_deleti  on | exon2 | c.1081delG | p.E361fs | . | . |
| CTC2 | 1 | 39854323 | AT | A | 0.328 | NM_012090 | MACF1 | frameshift_deleti  on | exon52 | c.9624delT | p.D3208fs | . | . |
| CTC2 | 1 | 40035284 | GT | G | 0.909 | NM_001135653 | PABPC4 | frameshift_deleti  on | exon5 | c.720delA | p.K240fs | . | . |
| CTC2 | 1 | 41990501 | CA | C | 0.294 | NM_001127714 | HIVEP3 | frameshift_deleti  on | exon5 | c.5287delT | p.C1763fs | . | . |
| CTC2 | 1 | 42047031 | GA | G | 0.277 | NM_001127714 | HIVEP3 | frameshift_deleti  on | exon3 | c.3437delT | p.F1146fs | . | . |
| CTC2 | 1 | 43240921 | C | CA | 1 | NM_024097 | C1orf50 | frameshift_inserti  on | exon5 | c.457dupA | p.Y152fs | . | . |
| CTC2 | 1 | 43885538 | TG | T | 0.503 | NM_015284 | SZT2 | frameshift_deleti  on | exon8 | c.1014delG | p.L338fs | . | . |
| CTC2 | 1 | 43887793 | GC | G | 0.75 | NM_015284 | SZT2 | frameshift_deleti  on | exon12 | c.1667delC | p.A556fs | . | . |
| CTC2 | 1 | 45243431 | G | GC | 0.371 | NM_001012 | RPS8 | frameshift_inserti  on | exon4 | c.358dupC | p.L119fs | . | . |
| CTC2 | 1 | 45792979 | CT | C | 0.449 | NM_032756 | HPDL | frameshift_deleti  on | exon1 | c.160delT | p.F54fs | . | . |
| CTC2 | 1 | 45793281 | GC | G | 0.8 | NM_032756 | HPDL | frameshift_deleti  on | exon1 | c.462delC | p.G154fs | . | . |
| CTC2 | 1 | 47124359 | CT | C | 0.893 | NM_001042546 | ATPAF1 | frameshift_deleti  on | exon3 | c.494delA | p.K165fs | . | . |
| CTC2 | 1 | 47173652 | GT | G | 0.857 | NM_014774 | EFCAB14 | frameshift_deleti  on | exon3 | c.405delA | p.K135fs | . | . |
| CTC2 | 1 | 48999846 | TA | T | 0.72 | NM_032785 | AGBL4 | stoploss | exon14 | c.1510delT | p.X504K | . | . |
| CTC2 | 1 | 49052731 | GA | G | 0.273 | NM_032785 | AGBL4 | frameshift_deleti on | exon11 | c.1211delT | p.F404fs | . | . |
| CTC2 | 1 | 49511345 | A | AG | 0.273 | NM_032785 | AGBL4 | frameshift_inserti  on | exon5 | c.504_505insC | p.Y169fs | . | . |
| CTC2 | 1 | 52248266 | CA | C | 0.513 | NM_148905 | OSBPL9 | frameshift_deleti  on | exon12 | c.931delA | p.K311fs | . | . |
| CTC2 | 1 | 52827212 | TTCC | T | 0.188 | NM_032449 | CC2D1B | nonframeshift_de  letion | exon4 | c.288_290del | p.96_97del | . | . |
| CTC2 | 1 | 53480648 | C | CA | 0.286 | NM_001193599 | SCP2 | frameshift_inserti  on | exon11 | c.1097dupA | p.H366fs | . | . |
| CTC2 | 1 | 53732230 | TG | T | 0.561 | NM_033300 | LRP8 | frameshift_deleti  on | exon7 | c.831delC | p.P277fs | . | . |
| CTC2 | 1 | 55566555 | AT | A | 0.248 | NM_015306 | USP24 | frameshift_deleti  on | exon44 | c.5227delA | p.M1743fs | . | . |
| CTC2 | 1 | 57224389 | CT | C | 0.467 | NM_001004303 | C1orf168 | frameshift_deleti  on | exon6 | c.1097delA | p.K366fs | . | . |
| CTC2 | 1 | 57406562 | C | CT | 0.231 | NM_000066 | C8B | frameshift_inserti  on | exon9 | c.1357_1358ins  A | p.G453fs | . | . |
| CTC2 | 1 | 57476834 | CT | C | 0.828 | NM_021080 | DAB1 | frameshift_deleti  on | exon15 | c.1555delA | p.S519fs | . | . |
| CTC2 | 1 | 62237119 | GA | G | 0.385 | NM_176877 | INADL | frameshift_deleti  on | exon6 | c.542delA | p.E181fs | . | . |

| CTC2 | 1 | 62293145 | CT | C | 0.48 | NM_176877 | INADL | frameshift_deleti  on | exon16 | c.1871delT | p.L624fs | . | . |
| --- | --- | --- | --- | --- | --- | --- | --- | --- | --- | --- | --- | --- | --- |
| CTC2 | 1 | 62676121 | TC | T | 0.552 | NM_019079 | L1TD1 | stopgain | exon4 | c.1676delC | p.S559X | . | . |
| CTC2 | 1 | 62738926 | TG | T | 0.647 | NM_181712 | KANK4 | frameshift_deleti on | exon3 | c.1849delC | p.Q617fs | . | . |
| CTC2 | 1 | 62979226 | CT | C | 0.286 | NM_001272000 | DOCK7 | frameshift_deleti  on | exon32 | c.4077delA | p.K1359fs | . | . |
| CTC2 | 1 | 64643375 | CT | C | 0.513 | NM_005012 | ROR1 | frameshift_deleti  on | exon9 | c.1652delT | p.L551fs | . | . |
| CTC2 | 1 | 65874462 | TA | T | 0.214 | NM_001256864 | DNAJC6 | frameshift_deleti  on | exon17 | c.2631delA | p.L877fs | . | . |
| CTC2 | 1 | 66058374 | CA | C | 0.658 | NM_001198687 | LEPR | frameshift_deleti  on | exon5 | c.530delA | p.Q177fs | . | . |
| CTC2 | 1 | 67450366 | TG | T | 0.5 | NM_001077701 | MIER1 | frameshift_deleti  on | exon13 | c.1323delG | p.M441fs | . | . |
| CTC2 | 1 | 67833666 | TG | T | 1 | NM_001258215 | IL12RB2 | frameshift_deleti  on | exon10 | c.1418delG | p.W473fs | . | . |
| CTC2 | 1 | 77627324 | GT | G | 0.179 | NM_005482 | PIGK | frameshift_deleti  on | exon7 | c.656delA | p.N219fs | . | . |
| CTC2 | 1 | 77984309 | C | CA | 0.613 | NM_012093 | AK5 | frameshift_inserti  on | exon11 | c.1131dupA | p.T377fs | . | . |
| CTC2 | 1 | 78024344 | T | TATA | 0.714 | NM_012093 | AK5 | nonframeshift_in  sertion | exon14 | c.1600_1601ins  ATA | p.S534delinsYT | . | . |
| CTC2 | 1 | 78024345 | C | CTAT | 1 | NM_012093 | AK5 | nonframeshift_in  sertion | exon14 | c.1601_1602ins  TAT | p.S534delinsSI | . | . |
| CTC2 | 1 | 78024348 | T | TTAG | 0.954 | NM_012093 | AK5 | nonframeshift_in  sertion | exon14 | c.1604_1605ins  TAG | p.I535delinsIS | . | . |
| CTC2 | 1 | 79116115 | CT | C | 0.5 | NM_006417 | IFI44 | frameshift_deleti  on | exon2 | c.236delT | p.L79fs | . | . |
| CTC2 | 1 | 85624724 | GT | G | 0.816 | NM_032184 | SYDE2 | frameshift_deleti  on | exon7 | c.3293delA | p.N1098fs | . | . |
| CTC2 | 1 | 87045832 | CA | C | 0.3 | NM_012128 | CLCA4 | frameshift_deleti  on | exon14 | c.2565delA | p.S855fs | . | . |
| CTC2 | 1 | 87045895 | ATCCTA  C | A | 1 | NM_012128 | CLCA4 | nonframeshift_de  letion | exon14 | c.2628_2633del | p.876_878del | . | . |
| CTC2 | 1 | 87045896 | TCCTAC  A | T | 1 | NM_012128 | CLCA4 | nonframeshift_de  letion | exon14 | c.2629_2634del | p.877_878del | . | . |
| CTC2 | 1 | 87538738 | CA | C | 0.286 | NM_001134492 | HS2ST1 | frameshift_deleti  on | exon2 | c.247delA | p.K83fs | . | . |
| CTC2 | 1 | 89270129 | TA | T | 0.474 | NM_006256 | PKN2 | frameshift_deleti  on | exon9 | c.1338delA | p.V446fs | . | . |
| CTC2 | 1 | 89613295 | CT | C | 0.583 | NM_207398 | GBP7 | frameshift_deleti  on | exon8 | c.1319delA | p.K440fs | . | . |
| CTC2 | 1 | 92193220 | AT | A | 0.447 | NM_001195683 | TGFBR3 | frameshift_deleti  on | exon7 | c.880delA | p.I294fs | . | . |
| CTC2 | 1 | 94941223 | AC | A | 0.657 | NM_001122674 | ABCD3 | frameshift_deleti  on | exon7 | c.558delC | p.D186fs | . | . |
| CTC2 | 1 | 100436099 | TCTCG | T | 0.165 | NM_001271685 | SLC35A3 | frameshift_deleti  on | exon1 | c.8_11del | p.S3fs | . | . |
| CTC2 | 1 | 100459144 | AT | A | 0.573 | NM_001271684 | SLC35A3 | frameshift_deleti  on | exon2 | c.35delT | p.I12fs | . | . |
| CTC2 | 1 | 104122120 | TA | T | 0.246 | NM_020978 | AMY2B | nonframeshift_de  letion | exon12 | c.1535delA | p.X512X | . | . |
| CTC2 | 1 | 109191607 | TA | T | 0.389 | NM_001102592 | HENMT1 | frameshift_deleti  on | exon8 | c.762delT | p.F254fs | . | . |
| CTC2 | 1 | 109391417 | CT | C | 0.429 | NM_152763 | AKNAD1 | frameshift_deleti  on | exon5 | c.1201delA | p.R401fs | . | . |
| CTC2 | 1 | 109477401 | TG | T | 0.714 | NM_001278203 | CLCC1 | frameshift_deleti  on | exon6 | c.991delC | p.Q331fs | . | . |
| CTC2 | 1 | 109824342 | AG | A | 0.328 | NM_001005290 | PSRC1 | frameshift_deleti  on | exon4 | c.417delC | p.P139fs | . | . |
| CTC2 | 1 | 109824457 | CT | C | 0.225 | NM_001005290 | PSRC1 | frameshift_deleti  on | exon4 | c.302delA | p.E101fs | . | . |
| CTC2 | 1 | 109878897 | TG | T | 0.458 | NM_001205228 | SORT1 | frameshift_deleti  on | exon11 | c.924delC | p.N308fs | . | . |
| CTC2 | 1 | 110257856 | CT | C | 0.468 | NM_000851 | GSTM5 | frameshift_deleti  on | exon7 | c.562delT | p.F188fs | . | . |
| CTC2 | 1 | 110593622 | TTG | T | 0.424 | NM_001270768 | STRIP1 | frameshift_deleti  on | exon19 | c.1717_1718del | p.C573fs | . | . |
| CTC2 | 1 | 111957501 | CTCACA  GACTGA TGACTC | C | 1 | NM_002557 | OVGP1 | nonframeshift_de letion | exon11 | c.1598_1621del | p.533_541del | . | . |
| CTC2 | 1 | 113239101 | CT | C | 0.853 | NM_001130079 | MOV10 | frameshift_deleti  on | exon13 | c.1927delT | p.F643fs | . | . |
| CTC2 | 1 | 114280867 | GT | G | 0.25 | NM_006608 | PHTF1 | frameshift_deleti  on | exon4 | c.195delA | p.K65fs | . | . |
| CTC2 | 1 | 114401101 | AG | A | 0.857 | NM_001193431 | PTPN22 | frameshift_deleti  on | exon4 | c.367delC | p.L123fs | . | . |
| CTC2 | 1 | 117527491 | AG | A | 0.195 | NM_020440 | PTGFRN | frameshift_deleti  on | exon8 | c.2358delG | p.Q786fs | . | . |
| CTC2 | 1 | 118495203 | TTG | T | 0.162 | NM_006784 | WDR3 | frameshift_deleti  on | exon19 | c.2070_2071del | p.V690fs | . | . |
| CTC2 | 1 | 119428047 | G | GA | 0.637 | NM_152380 | TBX15 | frameshift_inserti  on | exon8 | c.798dupT | p.P267fs | . | . |
| CTC2 | 1 | 119929353 | AT | A | 0.424 | NM_016527 | HAO2 | frameshift_deleti  on | exon5 | c.671delT | p.I224fs | . | . |
| CTC2 | 1 | 120457975 | GT | G | 0.4 | NM_024408 | NOTCH2 | frameshift_deleti  on | exon34 | c.7369delA | p.T2457fs | . | . |
| CTC2 | 1 | 144915623 | AG | A | 0.8 | NM_001002811 | PDE4DIP | frameshift_deleti  on | exon10 | c.2290delC | p.L764fs | . | . |
| CTC2 | 1 | 145561101 | TC | T | 0.667 | NM_001280799 | ANKRD35 | frameshift_deleti  on | exon8 | c.520delC | p.P174fs | . | . |
| CTC2 | 1 | 146465883 | AC | A | 0.968 | NM_001278141 | NBPF12 | frameshift_deleti  on | exon37 | c.4137delC | p.N1379fs | . | . |
| CTC2 | 1 | 146466055 | CT | C | 0.692 | NM_001278141 | NBPF12 | frameshift_deleti  on | exon37 | c.4309delT | p.F1437fs | . | . |
| CTC2 | 1 | 147096286 | GT | G | 0.3 | NM_004326 | BCL9 | frameshift_deleti  on | exon10 | c.3808delT | p.F1270fs | . | . |
| CTC2 | 1 | 149858158 | AG | A | 0.771 | NM_003528 | HIST2H2BE | frameshift_deleti  on | exon1 | c.32delC | p.P11fs | . | . |
| CTC2 | 1 | 150199039 | CTCCTCT | C | 1 | NM_001136478 | ANP32E | nonframeshift_de  letion | exon4 | c.453_458del | p.151_153del | . | . |
| CTC2 | 1 | 150234602 | GA | G | 0.27 | NM_012113 | CA14 | frameshift_deleti  on | exon4 | c.303delA | p.R101fs | . | . |
| CTC2 | 1 | 150443508 | TG | T | 0.875 | NM_015203 | RPRD2 | frameshift_deleti  on | exon11 | c.2085delG | p.L695fs | . | . |
| CTC2 | 1 | 150526115 | CA | C | 0.263 | NM_001288607 | ADAMTSL4 | frameshift_deleti  on | exon6 | c.649delA | p.T217fs | . | . |
| CTC2 | 1 | 150550894 | GA | G | 0.889 | NM_021960 | MCL1 | frameshift_deleti  on | exon2 | c.761delT | p.F254fs | . | . |
| CTC2 | 1 | 150679090 | GTT | G | 0.707 | NM_001199829 | HORMAD1 | frameshift_deleti  on | exon9 | c.720_721del | p.K240fs | . | . |

| CTC2 | 1 | 150804817 | TG | T | 0.733 | NM_001197325 | ARNT | frameshift_deleti  on | exon8 | c.816delC | p.N272fs | . | . |
| --- | --- | --- | --- | --- | --- | --- | --- | --- | --- | --- | --- | --- | --- |
| CTC2 | 1 | 150923459 | AC | A | 0.475 | NM_001145415 | SETDB1 | frameshift_deleti  on | exon13 | c.2107delC | p.P703fs | . | . |
| CTC2 | 1 | 150974774 | CG | C | 0.283 | NM_001163259 | FAM63A | frameshift_deleti  on | exon2 | c.34delC | p.R12fs | . | . |
| CTC2 | 1 | 151020649 | A | AG | 0.8 | NM_017860 | C1orf56 | frameshift_inserti  on | exon1 | c.327dupG | p.E109fs | . | . |
| CTC2 | 1 | 151106907 | TC | T | 1 | NM_001178062 | SEMA6C | frameshift_deleti  on | exon17 | c.1608delG | p.G536fs | . | . |
| CTC2 | 1 | 151259261 | AT | A | 0.711 | NM_020832 | ZNF687 | frameshift_deleti  on | exon2 | c.495delT | p.H165fs | . | . |
| CTC2 | 1 | 151260659 | C | CT | 0.42 | NM_020832 | ZNF687 | frameshift_inserti  on | exon2 | c.1893dupT | p.P631fs | . | . |
| CTC2 | 1 | 151261935 | GC | G | 0.265 | NM_020832 | ZNF687 | frameshift_deleti  on | exon5 | c.2554delC | p.P852fs | . | . |
| CTC2 | 1 | 151314788 | CT | C | 0.35 | NM_000449 | RFX5 | frameshift_deleti  on | exon11 | c.1724delA | p.K575fs | . | . |
| CTC2 | 1 | 151315339 | GC | G | 1 | NM_000449 | RFX5 | frameshift_deleti  on | exon11 | c.1173delG | p.L391fs | . | . |
| CTC2 | 1 | 152060487 | A | AC | 0.37 | NM_001008536 | TCHHL1 | frameshift_inserti  on | exon2 | c.132_133insG | p.F45fs | . | . |
| CTC2 | 1 | 152081234 | AT | A | 0.635 | NM_007113 | TCHH | frameshift_deleti  on | exon3 | c.4458delA | p.K1486fs | . | . |
| CTC2 | 1 | 152681680 | C | CAGCT  CTGGG GGCTG | 0.41 | NM_178356 | LCE4A | nonframeshift_in sertion | exon1 | c.129_130insA  GCTCTGGGGG CTGCTGT | p.S43delinsSSS GGCC | . | . |
| CTC2 | 1 | 152681686 | T | TGGGG  GCTGC TGTAG | 0.377 | NM_178356 | LCE4A | nonframeshift_in sertion | exon1 | c.135_136insG  GGGGCTGCTG TAGCTCC | p.S45delinsSG GCCSS | . | . |
| CTC2 | 1 | 152749015 | TG | T | 0.394 | NM_178354 | LCE1F | frameshift_deleti  on | exon1 | c.169delG | p.G57fs | . | . |
| CTC2 | 1 | 152770451 | G | GA | 0.295 | NM_178352 | LCE1D | frameshift_inserti  on | exon2 | c.181_182insA | p.G61fs | . | . |
| CTC2 | 1 | 152777900 | TG | T | 0.929 | NM_178351 | LCE1C | frameshift_deleti  on | exon2 | c.54delC | p.P18fs | . | . |
| CTC2 | 1 | 153318653 | TC | T | 0.382 | NM_020393 | PGLYRP4 | stopgain | exon3 | c.63delG | p.W21X | . | . |
| CTC2 | 1 | 153785879 | CT | C | 0.2 | NM_020699 | GATAD2B | frameshift_deleti on | exon8 | c.1265delA | p.Q422fs | . | . |
| CTC2 | 1 | 153905229 | AC | A | 0.333 | NM_014856 | DENND4B | frameshift_deleti  on | exon23 | c.3647delG | p.G1216fs | . | . |
| CTC2 | 1 | 153912691 | CG | C | 0.263 | NM_014856 | DENND4B | frameshift_deleti  on | exon11 | c.1543delC | p.R515fs | . | . |
| CTC2 | 1 | 154142920 | TC | T | 0.35 | NM_001278188 | TPM3 | frameshift_deleti  on | exon6 | c.421delG | p.E141fs | . | . |
| CTC2 | 1 | 154145427 | CT | C | 0.192 | NM_001278188 | TPM3 | frameshift_deleti  on | exon3 | c.213delA | p.E71fs | . | . |
| CTC2 | 1 | 154295759 | GC | G | 1 | NM_080429 | AQP10 | frameshift_deleti  on | exon4 | c.414delC | p.G138fs | . | . |
| CTC2 | 1 | 154931368 | TC | T | 0.667 | NM_138300 | PYGO2 | frameshift_deleti  on | exon3 | c.1107delG | p.L369fs | . | . |
| CTC2 | 1 | 155160035 | GA | G | 1 | NM_001044390 | MUC1 | frameshift_deleti  on | exon5 | c.282delT | p.F94fs | . | . |
| CTC2 | 1 | 155265071 | AG | A | 0.273 | NM_000298 | PKLR | frameshift_deleti  on | exon5 | c.529delC | p.L177fs | . | . |
| CTC2 | 1 | 155290698 | TC | T | 0.341 | NM_001039517 | RUSC1-AS1 | frameshift_deleti  on | exon2 | c.581delG | p.G194fs | . | . |
| CTC2 | 1 | 155340302 | GC | G | 0.742 | NM_018489 | ASH1L | frameshift_deleti  on | exon12 | c.6678delG | p.M2226fs | . | . |
| CTC2 | 1 | 155887375 | GGGGGC  T | G | 0.176 | NM_014949 | KIAA0907 | nonframeshift_de  letion | exon11 | c.1349_1354del | p.450_452del | . | . |
| CTC2 | 1 | 156047168 | TG | T | 0.421 | NM_001093725 | MEX3A | frameshift_deleti  on | exon2 | c.759delC | p.T253fs | . | . |
| CTC2 | 1 | 156212357 | CG | C | 0.4 | NM_199173 | BGLAP | frameshift_deleti  on | exon2 | c.79delG | p.G27fs | . | . |
| CTC2 | 1 | 156263923 | CCG | C | 0.571 | NM_001256605 | C1orf85 | frameshift_deleti  on | exon3 | c.424_425del | p.R142fs | . | . |
| CTC2 | 1 | 156263924 | CG | C | 0.769 | NM_001256605 | C1orf85 | frameshift_deleti  on | exon3 | c.424delC | p.R142fs | . | . |
| CTC2 | 1 | 156438739 | A | AT | 0.259 | NM_001271629 | MEF2D | frameshift_inserti  on | exon9 | c.1058dupA | p.N353fs | . | . |
| CTC2 | 1 | 156565049 | A | AAC | 1 | NM_015590 | GPATCH4 | frameshift_inserti  on | exon8 | c.1083_1084ins  GT | p.F362fs | . | . |
| CTC2 | 1 | 156565406 | TG | T | 0.842 | NM_015590 | GPATCH4 | frameshift_deleti  on | exon8 | c.726delC | p.I242fs | . | . |
| CTC2 | 1 | 156627542 | T | TC | 0.387 | NM_021948 | BCAN | frameshift_inserti  on | exon11 | c.2286dupC | p.V762fs | . | . |
| CTC2 | 1 | 156697301 | AG | A | 0.393 | NM_030980 | ISG20L2 | frameshift_deleti  on | exon1 | c.143delC | p.P48fs | . | . |
| CTC2 | 1 | 156823957 | CG | C | 0.214 | NM_014215 | INSRR | frameshift_deleti  on | exon2 | c.223delC | p.R75fs | . | . |
| CTC2 | 1 | 157659611 | T | TA | 0.72 | NM_052939 | FCRL3 | frameshift_inserti  on | exon10 | c.1786dupT | p.Y596fs | . | . |
| CTC2 | 1 | 157737056 | GCC | G | 0.46 | NM_030764 | FCRL2 | frameshift_deleti  on | exon6 | c.1125_1126del | p.G375fs | . | . |
| CTC2 | 1 | 157765943 | AT | A | 0.235 | NM_001159398 | FCRL1 | frameshift_deleti  on | exon11 | c.1232delA | p.Y411fs | . | . |
| CTC2 | 1 | 158262074 | GT | G | 0.176 | NM_001765 | CD1C | frameshift_deleti  on | exon3 | c.530delT | p.V177fs | . | . |
| CTC2 | 1 | 158687276 | GA | G | 0.547 | NM_001005327 | OR6K3 | frameshift_deleti  on | exon1 | c.629delT | p.F210fs | . | . |
| CTC2 | 1 | 158913676 | GA | G | 0.727 | NM_152501 | PYHIN1 | frameshift_deleti  on | exon6 | c.1100delA | p.E367fs | . | . |
| CTC2 | 1 | 160851009 | GC | G | 0.364 | NM_017625 | ITLN1 | frameshift_deleti  on | exon5 | c.498delG | p.L166fs | . | . |
| CTC2 | 1 | 161088951 | C | CT | 0.455 | NM_001185093 | NIT1 | frameshift_inserti  on | exon2 | c.82dupT | p.S27fs | . | . |
| CTC2 | 1 | 161093631 | GT | G | 0.241 | NM_001039711 | DEDD | frameshift_deleti  on | exon3 | c.430delA | p.T144fs | . | . |
| CTC2 | 1 | 161160945 | AG | A | 0.857 | NM_005099 | ADAMTS4 | frameshift_deleti  on | exon9 | c.2496delC | p.P832fs | . | . |
| CTC2 | 1 | 161494778 | CG | C | 0.236 | NM_002155 | HSPA6 | frameshift_deleti  on | exon1 | c.331delG | p.G111fs | . | . |
| CTC2 | 1 | 161681171 | GC | G | 0.145 | NM_032738 | FCRLA | frameshift_deleti  on | exon3 | c.458delC | p.A153fs | . | . |
| CTC2 | 1 | 162769587 | TG | T | 0.3 | NM_016371 | HSD17B7 | stopgain | exon5 | c.503delG | p.W168X | . | . |
| CTC2 | 1 | 162824632 | C | CA | 0.625 | NM_178550 | C1orf110 | frameshift_inserti on | exon4 | c.831dupT | p.G278fs | . | . |
| CTC2 | 1 | 163039267 | TA | T | 0.347 | NM_001102445 | RGS4 | frameshift_deleti  on | exon2 | c.285delA | p.L95fs | . | . |
| CTC2 | 1 | 163318805 | CA | C | 0.9 | NM_031423 | NUF2 | frameshift_deleti  on | exon13 | c.1196delA | p.Q399fs | . | . |
| CTC2 | 1 | 165218675 | TGA | T | 0.552 | NM_001174069 | LMX1A | frameshift_deleti  on | exon4 | c.464_465del | p.L155fs | . | . |

| CTC2 | 1 | 169345877 | AG | A | 1 | NM_003666 | BLZF1 | frameshift_deleti  on | exon3 | c.129delG | p.K43fs | . | . |
| --- | --- | --- | --- | --- | --- | --- | --- | --- | --- | --- | --- | --- | --- |
| CTC2 | 1 | 169676576 | AG | A | 0.233 | NM_000655 | SELL | frameshift_deleti  on | exon4 | c.525delC | p.P175fs | . | . |
| CTC2 | 1 | 171249950 | GAT | G | 0.5 | NM_001282692 | FMO1 | frameshift_deleti  on | exon5 | c.666_667del | p.G222fs | . | . |
| CTC2 | 1 | 171481259 | CA | C | 0.286 | NM_015172 | PRRC2C | frameshift_deleti  on | exon2 | c.33delA | p.A11fs | . | . |
| CTC2 | 1 | 172062008 | G | GA | 0.23 | NM_001136127 | DNM3 | frameshift_inserti  on | exon13 | c.1539dupA | p.G513fs | . | . |
| CTC2 | 1 | 172356280 | A | AT | 0.733 | NM_001136127 | DNM3 | frameshift_inserti  on | exon18 | c.2055dupT | p.D685fs | . | . |
| CTC2 | 1 | 173880968 | T | TA | 0.526 | NM_000488 | SERPINC1 | frameshift_inserti  on | exon3 | c.592dupT | p.Y198fs | . | . |
| CTC2 | 1 | 173939747 | CT | C | 0.191 | NM_172071 | RC3H1 | frameshift_deleti  on | exon8 | c.1229delA | p.Q410fs | . | . |
| CTC2 | 1 | 176740297 | GC | G | 0.312 | NM_020318 | PAPPA2 | frameshift_deleti  on | exon17 | c.4697delC | p.A1566fs | . | . |
| CTC2 | 1 | 178423658 | CT | C | 0.298 | NM_004841 | RASAL2 | frameshift_deleti  on | exon10 | c.1643delT | p.L548fs | . | . |
| CTC2 | 1 | 178423722 | TG | T | 0.22 | NM_004841 | RASAL2 | frameshift_deleti  on | exon10 | c.1707delG | p.L569fs | . | . |
| CTC2 | 1 | 179437637 | GA | G | 1 | NM_144696 | AXDND1 | frameshift_deleti  on | exon17 | c.1859delA | p.E620fs | . | . |
| CTC2 | 1 | 179833933 | GT | G | 1 | NM_022347 | TOR1AIP2 | frameshift_deleti  on | exon3 | c.378delA | p.K126fs | . | . |
| CTC2 | 1 | 180163430 | GC | G | 0.213 | NM_001004128 | QSOX1 | frameshift_deleti  on | exon11 | c.1372delC | p.Q458fs | . | . |
| CTC2 | 1 | 180165721 | CT | C | 0.185 | NM_001004128 | QSOX1 | frameshift_deleti  on | exon12 | c.1794delT | p.A598fs | . | . |
| CTC2 | 1 | 180382528 | AT | A | 0.407 | NM_032360 | ACBD6 | frameshift_deleti  on | exon5 | c.545delA | p.N182fs | . | . |
| CTC2 | 1 | 180756954 | GA | G | 0.385 | NM_001135669 | XPR1 | frameshift_deleti  on | exon3 | c.188delA | p.E63fs | . | . |
| CTC2 | 1 | 183101632 | GA | G | 0.293 | NM_002293 | LAMC1 | frameshift_deleti  on | exon21 | c.3665delA | p.E1222fs | . | . |
| CTC2 | 1 | 183498130 | AG | A | 0.8 | NM_001174061 | SMG7 | frameshift_deleti  on | exon6 | c.535delG | p.A179fs | . | . |
| CTC2 | 1 | 185069010 | AG | A | 0.333 | NM_007212 | RNF2 | frameshift_deleti  on | exon6 | c.826delG | p.G276fs | . | . |
| CTC2 | 1 | 186023046 | GC | G | 0.305 | NM_031935 | HMCN1 | frameshift_deleti  on | exon44 | c.6791delC | p.A2264fs | . | . |
| CTC2 | 1 | 186039796 | AG | A | 0.232 | NM_031935 | HMCN1 | frameshift_deleti  on | exon52 | c.8047delG | p.E2683fs | . | . |
| CTC2 | 1 | 186277344 | CA | C | 0.164 | NM_001127710 | PRG4 | frameshift_deleti  on | exon4 | c.2092delA | p.K698fs | . | . |
| CTC2 | 1 | 186957575 | TC | T | 0.269 | NM_024420 | PLA2G4A | frameshift_deleti  on | exon18 | c.2186delC | p.S729fs | . | . |
| CTC2 | 1 | 196695703 | C | CA | 0.667 | NM_000186 | CFH | frameshift_inserti  on | exon13 | c.1978dupA | p.C659fs | . | . |
| CTC2 | 1 | 197030151 | GT | G | 0.571 | NM_001994 | F13B | frameshift_deleti  on | exon4 | c.505delA | p.T169fs | . | . |
| CTC2 | 1 | 200529975 | CT | C | 0.72 | NM_014875 | KIF14 | frameshift_deleti  on | exon26 | c.4104delA | p.K1368fs | . | . |
| CTC2 | 1 | 200870066 | AC | A | 0.328 | NM_001142569 | C1orf106 | frameshift_deleti  on | exon5 | c.300delC | p.D100fs | . | . |
| CTC2 | 1 | 201046241 | AGC | A | 0.099 | NM_000069 | CACNA1S | frameshift_deleti  on | exon12 | c.1632_1633del | p.S544fs | . | . |
| CTC2 | 1 | 201179027 | TGGA | T | 0.545 | NM_001164586 | IGFN1 | nonframeshift_de  letion | exon12 | c.5007_5009del | p.1669_1670de  l | . | . |
| CTC2 | 1 | 201687657 | G | GA | 0.217 | NM_020443 | NAV1 | frameshift_inserti  on | exon3 | c.1000_1001ins  A | p.G334fs | . | . |
| CTC2 | 1 | 201750212 | GC | G | 0.286 | NM_001167738 | NAV1 | frameshift_deleti  on | exon3 | c.266delC | p.A89fs | . | . |
| CTC2 | 1 | 201777562 | GC | G | 0.952 | NM_001167738 | NAV1 | frameshift_deleti  on | exon16 | c.2681delC | p.A894fs | . | . |
| CTC2 | 1 | 201843997 | AGAG | A | 0.714 | NM_018085 | IPO9 | nonframeshift_de  letion | exon22 | c.2872_2874del | p.958_958del | . | . |
| CTC2 | 1 | 202288092 | GC | G | 0.474 | NM_001017404 | LGR6 | frameshift_deleti  on | exon16 | c.2245delC | p.P749fs | . | . |
| CTC2 | 1 | 202300995 | CT | C | 1 | NM_014176 | UBE2T | frameshift_deleti  on | exon7 | c.561delA | p.V187fs | . | . |
| CTC2 | 1 | 202573646 | G | GTTC | 0.5 | NM_001136504 | SYT2 | nonframeshift_in  sertion | exon3 | c.281_282insG  AA | p.N94delinsKN | . | . |
| CTC2 | 1 | 202573647 | T | TTCG | 0.331 | NM_001136504 | SYT2 | nonframeshift_in  sertion | exon3 | c.280_281insC  GA | p.N94delinsTN | . | . |
| CTC2 | 1 | 202702883 | TG | T | 0.727 | NM_006618 | KDM5B | frameshift_deleti  on | exon23 | c.3554delC | p.P1185fs | . | . |
| CTC2 | 1 | 203054753 | TG | T | 0.636 | NM_002479 | MYOG | frameshift_deleti  on | exon1 | c.336delC | p.P112fs | . | . |
| CTC2 | 1 | 203186959 | G | GCCCC  GCCCA GTCCC TAGAC | 0.578 | NM_001256125 | CHIT1 | stopgain | exon9 | c.1006_1007ins  TCATGGTCTAG GGACTGGGCG GGG | p.A336delinsV MVX | . | . |
| CTC2 | 1 | 203766873 | CG | C | 0.857 | NM_001174108 | ZBED6 | frameshift_deleti  on | exon1 | c.224delG | p.R75fs | . | . |
| CTC2 | 1 | 203768494 | AT | A | 0.247 | NM_001174108 | ZBED6 | frameshift_deleti  on | exon1 | c.1845delT | p.H615fs | . | . |
| CTC2 | 1 | 203787734 | AT | A | 0.9 | NM_014827 | ZC3H11A | frameshift_deleti  on | exon6 | c.92delT | p.I31fs | . | . |
| CTC2 | 1 | 203818945 | TC | T | 0.312 | NM_014827 | ZC3H11A | frameshift_deleti  on | exon17 | c.1731delC | p.V577fs | . | . |
| CTC2 | 1 | 204085763 | AAGC | A | 0.125 | NM_005686 | SOX13 | nonframeshift_de  letion | exon5 | c.548_550del | p.183_184del | . | . |
| CTC2 | 1 | 204125874 | T | TG | 0.375 | NM_000537 | REN | frameshift_inserti  on | exon7 | c.748dupC | p.Q250fs | . | . |
| CTC2 | 1 | 204199705 | TGG | T | 0.207 | NM_014935 | PLEKHA6 | frameshift_deleti  on | exon18 | c.2417_2418del | p.P806fs | . | . |
| CTC2 | 1 | 204412665 | TG | T | 0.692 | NM_002646 | PIK3C2B | frameshift_deleti  on | exon20 | c.2927delC | p.A976fs | . | . |
| CTC2 | 1 | 204438637 | TG | T | 0.5 | NM_002646 | PIK3C2B | frameshift_deleti  on | exon3 | c.293delC | p.P98fs | . | . |
| CTC2 | 1 | 205052948 | GT | G | 1 | NM_203376 | TMEM81 | frameshift_deleti  on | exon1 | c.500delA | p.N167fs | . | . |
| CTC2 | 1 | 205052948 | GTT | G | 1 | NM_203376 | TMEM81 | frameshift_deleti  on | exon1 | c.499_500del | p.N167fs | . | . |
| CTC2 | 1 | 205628506 | TG | T | 0.556 | NM_033102 | SLC45A3 | frameshift_deleti  on | exon5 | c.1517delC | p.P506fs | . | . |
| CTC2 | 1 | 207039911 | AT | A | 0.4 | NM_018724 | IL20 | frameshift_deleti  on | exon3 | c.309delT | p.H103fs | . | . |
| CTC2 | 1 | 207106385 | AC | A | 0.56 | NM_002644 | PIGR | frameshift_deleti  on | exon7 | c.1831delG | p.V611fs | . | . |
| CTC2 | 1 | 207222794 | A | AT | 0.315 | NM_018566 | YOD1 | frameshift_inserti  on | exon2 | c.617dupA | p.N206fs | . | . |

| CTC2 | 1 | 207803964 | C | CA | 0.25 | NM_000573 | CR1 | frameshift_inserti  on | exon38 | c.6106dupA | p.S2035fs | . | . |
| --- | --- | --- | --- | --- | --- | --- | --- | --- | --- | --- | --- | --- | --- |
| CTC2 | 1 | 208225764 | TG | T | 0.5 | NM_025179 | PLXNA2 | stopgain | exon15 | c.2900delC | p.S967X | . | . |
| CTC2 | 1 | 209605636 | TAGCAG CAGCAG | T | 0.909 | NM_001104548 | MIR205HG | nonframeshift_de letion | exon4 | c.252_263del | p.84_88del | . | . |
| CTC2 | 1 | 209791909 | TC | T | 0.438 | NM_001017402 | LAMB3 | frameshift_deleti  on | exon18 | c.2796delG | p.M932fs | . | . |
| CTC2 | 1 | 209805956 | GCC | G | 0.551 | NM_001017402 | LAMB3 | frameshift_deleti  on | exon7 | c.792_793del | p.G264fs | . | . |
| CTC2 | 1 | 212799325 | G | GT | 0.176 | NM_153606 | FAM71A | frameshift_inserti  on | exon1 | c.1106_1107ins  T | p.S369fs | . | . |
| CTC2 | 1 | 213178491 | C | CT | 0.727 | NM_144567 | ANGEL2 | frameshift_inserti  on | exon5 | c.1017dupA | p.D340fs | . | . |
| CTC2 | 1 | 214825157 | AG | A | 0.468 | NM_016343 | CENPF | frameshift_deleti  on | exon15 | c.8089delG | p.A2697fs | . | . |
| CTC2 | 1 | 214826306 | AT | A | 0.28 | NM_016343 | CENPF | frameshift_deleti  on | exon16 | c.8297delT | p.I2766fs | . | . |
| CTC2 | 1 | 215821042 | A | AG | 0.357 | NM_206933 | USH2A | frameshift_inserti  on | exon67 | c.14612dupC | p.P4871fs | . | . |
| CTC2 | 1 | 215987242 | CA | C | 0.5 | NM_206933 | USH2A | frameshift_deleti  on | exon49 | c.9574delT | p.C3192fs | . | . |
| CTC2 | 1 | 216595428 | CA | C | 0.545 | NM_007123 | USH2A | frameshift_deleti  on | exon2 | c.250delT | p.C84fs | . | . |
| CTC2 | 1 | 220300148 | GT | G | 0.217 | NM_018060 | IARS2 | frameshift_deleti  on | exon14 | c.1801delT | p.F601fs | . | . |
| CTC2 | 1 | 220356109 | CT | C | 0.667 | NM_012414 | RAB3GAP2 | frameshift_deleti  on | exon20 | c.2162delA | p.K721fs | . | . |
| CTC2 | 1 | 222713607 | AG | A | 0.567 | NM_024746 | HHIPL2 | frameshift_deleti  on | exon4 | c.1194delC | p.P398fs | . | . |
| CTC2 | 1 | 222801627 | GC | G | 0.235 | NM_198551 | MIA3 | frameshift_deleti  on | exon4 | c.1066delC | p.Q356fs | . | . |
| CTC2 | 1 | 222835404 | TG | T | 0.849 | NM_198551 | MIA3 | frameshift_deleti  on | exon25 | c.5121delG | p.V1707fs | . | . |
| CTC2 | 1 | 222837458 | CT | C | 0.273 | NM_198551 | MIA3 | frameshift_deleti  on | exon27 | c.5382delT | p.P1794fs | . | . |
| CTC2 | 1 | 222892372 | AG | A | 0.704 | NM_001288579 | BROX | frameshift_deleti  on | exon3 | c.208delG | p.G70fs | . | . |
| CTC2 | 1 | 223156433 | CT | C | 0.238 | NM_032890 | DISP1 | frameshift_deleti  on | exon5 | c.522delT | p.P174fs | . | . |
| CTC2 | 1 | 223402681 | CG | C | 0.206 | NM_017982 | SUSD4 | frameshift_deleti  on | exon6 | c.773delC | p.P258fs | . | . |
| CTC2 | 1 | 223983829 | CT | C | 0.987 | NM_001031685 | TP53BP2 | frameshift_deleti  on | exon13 | c.2411delA | p.K804fs | . | . |
| CTC2 | 1 | 224586257 | GA | G | 0.585 | NM_001115113 | WDR26 | frameshift_deleti  on | exon11 | c.1555delT | p.S519fs | . | . |
| CTC2 | 1 | 225230537 | AT | A | 0.722 | NM_001373 | DNAH14 | frameshift_deleti  on | exon13 | c.1490delT | p.I497fs | . | . |
| CTC2 | 1 | 225334823 | CT | C | 0.913 | NM_001373 | DNAH14 | frameshift_deleti  on | exon30 | c.4762delT | p.F1588fs | . | . |
| CTC2 | 1 | 226027655 | AC | A | 0.314 | NM_000120 | EPHX1 | frameshift_deleti  on | exon6 | c.849delC | p.Y283fs | . | . |
| CTC2 | 1 | 226032933 | C | CA | 0.222 | NM_000120 | EPHX1 | frameshift_inserti  on | exon9 | c.1254dupA | p.P418fs | . | . |
| CTC2 | 1 | 226340153 | AT | A | 0.211 | NM_022735 | ACBD3 | frameshift_deleti  on | exon7 | c.1257delA | p.E419fs | . | . |
| CTC2 | 1 | 226555246 | CT | C | 0.357 | NM_001618 | PARP1 | frameshift_deleti  on | exon17 | c.2340delA | p.G780fs | . | . |
| CTC2 | 1 | 227165172 | CG | C | 0.719 | NM_020247 | ADCK3 | frameshift_deleti  on | exon5 | c.679delG | p.G227fs | . | . |
| CTC2 | 1 | 227257511 | CA | C | 0.789 | NM_014826 | CDC42BPA | stopgain | exon20 | c.2627delT | p.L876X | . | . |
| CTC2 | 1 | 228033808 | AT | A | 0.494 | NM_183062 | PRSS38 | frameshift_deleti on | exon5 | c.881delT | p.I294fs | . | . |
| CTC2 | 1 | 228464875 | G | GA | 0.308 | NM_001098623 | OBSCN | frameshift_inserti  on | exon24 | c.6615_6616ins  A | p.L2205fs | . | . |
| CTC2 | 1 | 228504632 | AG | A | 0.4 | NM_001098623 | OBSCN | frameshift_deleti  on | exon51 | c.13509delG | p.E4503fs | . | . |
| CTC2 | 1 | 228596127 | GA | G | 0.274 | NM_001024940 | TRIM17 | frameshift_deleti  on | exon7 | c.1208delT | p.F403fs | . | . |
| CTC2 | 1 | 229772763 | TC | T | 0.652 | NM_014777 | URB2 | frameshift_deleti  on | exon4 | c.2404delC | p.P802fs | . | . |
| CTC2 | 1 | 229773363 | AGG | A | 0.2 | NM_014777 | URB2 | frameshift_deleti  on | exon4 | c.3004_3005del | p.G1002fs | . | . |
| CTC2 | 1 | 231155871 | AC | A | 0.221 | NM_198552 | FAM89A | frameshift_deleti  on | exon2 | c.292delG | p.V98fs | . | . |
| CTC2 | 1 | 231700459 | GT | G | 0.304 | NM_005999 | TSNAX | frameshift_deleti  on | exon6 | c.682delT | p.F228fs | . | . |
| CTC2 | 1 | 232600892 | CT | C | 0.762 | NM_020808 | SIPA1L2 | frameshift_deleti  on | exon7 | c.2513delA | p.K838fs | . | . |
| CTC2 | 1 | 235715452 | T | TC | 1 | NM_004485 | GNG4 | frameshift_inserti  on | exon3 | c.184dupG | p.E62fs | . | . |
| CTC2 | 1 | 235866183 | TG | T | 0.22 | NM_000081 | LYST | frameshift_deleti  on | exon45 | c.10237delC | p.Q3413fs | . | . |
| CTC2 | 1 | 235909661 | TA | T | 0.696 | NM_000081 | LYST | stopgain | exon29 | c.7946delT | p.L2649X | . | . |
| CTC2 | 1 | 235922635 | GT | G | 0.5 | NM_000081 | LYST | frameshift_deleti on | exon23 | c.6517delA | p.T2173fs | . | . |
| CTC2 | 1 | 236368436 | G | GA | 0.288 | NM_003272 | GPR137B | frameshift_inserti  on | exon6 | c.978dupA | p.G326fs | . | . |
| CTC2 | 1 | 236368442 | TC | T | 0.393 | NM_003272 | GPR137B | frameshift_deleti  on | exon6 | c.984delC | p.V328fs | . | . |
| CTC2 | 1 | 236572515 | GATC | G | 0.138 | NM_080738 | EDARADD | nonframeshift_de  letion | exon2 | c.32_34del | p.11_12del | . | . |
| CTC2 | 1 | 236729993 | CA | C | 0.667 | NM_018072 | HEATR1 | frameshift_deleti  on | exon30 | c.4260delT | p.F1420fs | . | . |
| CTC2 | 1 | 236987447 | GC | G | 0.677 | NM_000254 | MTR | frameshift_deleti  on | exon9 | c.794delC | p.A265fs | . | . |
| CTC2 | 1 | 237025593 | GA | G | 0.362 | NM_000254 | MTR | frameshift_deleti  on | exon21 | c.2255delA | p.E752fs | . | . |
| CTC2 | 1 | 237870543 | AG | A | 1 | NM_001035 | RYR2 | frameshift_deleti  on | exon68 | c.9876delG | p.E3292fs | . | . |
| CTC2 | 1 | 237924327 | AG | A | 0.2 | NM_001035 | RYR2 | frameshift_deleti  on | exon84 | c.11476delG | p.G3826fs | . | . |
| CTC2 | 1 | 243328905 | T | TG | 0.2 | NM_001042404 | CEP170 | frameshift_inserti  on | exon12 | c.2062dupC | p.Q688fs | . | . |
| CTC2 | 1 | 244580965 | CA | C | 0.8 | NM_001126 | ADSS | frameshift_deleti  on | exon10 | c.1034delT | p.L345fs | . | . |
| CTC2 | 1 | 245530502 | GA | G | 0.87 | NM_018012 | KIF26B | frameshift_deleti  on | exon3 | c.833delA | p.E278fs | . | . |
| CTC2 | 1 | 246498708 | TG | T | 0.174 | NM_001167740 | SMYD3 | frameshift_deleti  on | exon3 | c.296delC | p.P99fs | . | . |
| CTC2 | 1 | 247978192 | TG | T | 0.378 | NM_001001966 | OR14A16 | frameshift_deleti  on | exon1 | c.839delC | p.P280fs | . | . |
| CTC2 | 1 | 248112694 | G | GA | 0.235 | NM_001001963 | OR2L8 | frameshift_inserti  on | exon1 | c.536dupA | p.D179fs | . | . |

| CTC2 | 1 | 248309248 | TC | T | 0.455 | NM_001004690 | OR2M5 | frameshift_deleti  on | exon1 | c.800delC | p.S267fs | . | . |
| --- | --- | --- | --- | --- | --- | --- | --- | --- | --- | --- | --- | --- | --- |
| CTC2 | 1 | 248366545 | TG | T | 0.25 | NM_001004689 | OR2M3 | frameshift_deleti  on | exon1 | c.177delG | p.M59fs | . | . |
| CTC2 | 1 | 248402480 | AG | A | 0.213 | NM_017504 | OR2M4 | frameshift_deleti  on | exon1 | c.251delG | p.S84fs | . | . |
| CTC2 | 1 | 248525638 | CA | C | 1 | NM_001004696 | OR2T4 | frameshift_deleti  on | exon1 | c.757delA | p.I253fs | . | . |
| CTC2 | 1 | 248756524 | AG | A | 0.2 | NM_001004693 | OR2T10 | frameshift_deleti  on | exon1 | c.545delC | p.P182fs | . | . |
| CTC2 | 1 | 248801944 | TCAGCA  CG | T | 0.178 | NM_001001827 | OR2T35 | frameshift_deleti  on | exon1 | c.609_615del | p.C203fs | . | . |
| CTC2 | 1 | 249151538 | GT | G | 0.411 | NM_001136036 | ZNF692 | frameshift_deleti  on | exon4 | c.384delA | p.G128fs | . | . |
| CTC2 | 2 | 3673658 | TC | T | 1 | NM_199235 | COLEC11 | frameshift_deleti  on | exon4 | c.170delC | p.S57fs | . | . |
| CTC2 | 2 | 3749151 | GGAA | G | 1 | NM_018436 | ALLC | nonframeshift_de  letion | exon11 | c.901_903del | p.301_301del | . | . |
| CTC2 | 2 | 9098741 | AG | A | 0.5 | NM_138799 | MBOAT2 | frameshift_deleti  on | exon2 | c.105delC | p.A35fs | . | . |
| CTC2 | 2 | 10559900 | GC | G | 1 | NM_001258357 | HPCAL1 | frameshift_deleti  on | exon3 | c.18delC | p.S6fs | . | . |
| CTC2 | 2 | 11945242 | ACCCAT | A | 0.12 | NM_145693 | LPIN1 | frameshift_deleti  on | exon16 | c.2102_2106del | p.T701fs | . | . |
| CTC2 | 2 | 15467886 | GA | G | 0.486 | NM_015909 | NBAS | frameshift_deleti  on | exon38 | c.4569delT | p.F1523fs | . | . |
| CTC2 | 2 | 17922952 | GT | G | 1 | NM_024624 | SMC6 | frameshift_deleti  on | exon3 | c.164delA | p.N55fs | . | . |
| CTC2 | 2 | 17927203 | CT | C | 0.364 | NM_024624 | SMC6 | frameshift_deleti  on | exon2 | c.10delA | p.R4fs | . | . |
| CTC2 | 2 | 17962651 | AG | A | 0.649 | NM_001130009 | GEN1 | frameshift_deleti  on | exon14 | c.2173delG | p.G725fs | . | . |
| CTC2 | 2 | 18765868 | CGCT | C | 0.481 | NM_001199103 | NT5C1B-  RDH14 | nonframeshift_de  letion | exon4 | c.638_640del | p.213_214del | . | . |
| CTC2 | 2 | 19553209 | CA | C | 0.608 | NM_145260 | OSR1 | frameshift_deleti  on | exon2 | c.357delT | p.F119fs | . | . |
| CTC2 | 2 | 21225111 | C | CT | 0.318 | NM_000384 | APOB | frameshift_inserti  on | exon29 | c.13182_13183i  nsA | p.G4395fs | . | . |
| CTC2 | 2 | 24240171 | TA | T | 0.886 | NM_001080473 | MFSD2B | frameshift_deleti  on | exon5 | c.488delA | p.Y163fs | . | . |
| CTC2 | 2 | 24276779 | A | AT | 0.227 | NM_004116 | FKBP1B | frameshift_inserti  on | exon2 | c.46dupT | p.T15fs | . | . |
| CTC2 | 2 | 24342486 | TG | T | 0.3 | NM_199346 | PFN4 | frameshift_deleti  on | exon4 | c.321delC | p.G107fs | . | . |
| CTC2 | 2 | 26693553 | CCTT | C | 0.143 | NM_194322 | OTOF | nonframeshift_de  letion | exon14 | c.1858_1860del | p.620_620del | . | . |
| CTC2 | 2 | 27001339 | TG | T | 0.205 | NM_017877 | SLC35F6 | frameshift_deleti  on | exon6 | c.1077delG | p.L359fs | . | . |
| CTC2 | 2 | 27306255 | CT | C | 1 | NM_007046 | EMILIN1 | frameshift_deleti  on | exon4 | c.1817delT | p.L606fs | . | . |
| CTC2 | 2 | 27590940 | GACC | G | 0.34 | NM_172195 | EIF2B4 | nonframeshift_de  letion | exon6 | c.714_716del | p.238_239del | . | . |
| CTC2 | 2 | 28002400 | GA | G | 0.162 | NM_145330 | MRPL33 | frameshift_deleti  on | exon3 | c.143delA | p.D48fs | . | . |
| CTC2 | 2 | 28821537 | AC | A | 0.229 | NM_001170585 | PLB1 | frameshift_deleti  on | exon34 | c.2352delC | p.N784fs | . | . |
| CTC2 | 2 | 28821625 | TG | T | 0.774 | NM_001170585 | PLB1 | frameshift_deleti  on | exon34 | c.2440delG | p.E814fs | . | . |
| CTC2 | 2 | 29287926 | C | CGCT | 0.875 | NM_001029883 | C2orf71 | nonframeshift_in  sertion | exon2 | c.3675_3676ins  AGC | p.E1226delinsS  E | . | . |
| CTC2 | 2 | 29296688 | CT | C | 0.491 | NM_001029883 | C2orf71 | frameshift_deleti  on | exon1 | c.439delA | p.R147fs | . | . |
| CTC2 | 2 | 29356658 | AT | A | 0.444 | NM_001287527 | CLIP4 | frameshift_deleti  on | exon5 | c.506delT | p.I169fs | . | . |
| CTC2 | 2 | 29416423 | C | CT | 0.5 | NM_004304 | ALK | stopgain | exon29 | c.4529_4530ins  A | p.W1510_F151  1delinsX | . | . |
| CTC2 | 2 | 31165133 | T | TG | 0.264 | NM_024572 | GALNT14 | frameshift_inserti  on | exon9 | c.864dupC | p.K289fs | . | . |
| CTC2 | 2 | 31588950 | A | AC | 0.319 | NM_000379 | XDH | frameshift_inserti  on | exon22 | c.2347dupG | p.V783fs | . | . |
| CTC2 | 2 | 32658751 | TA | T | 0.267 | NM_016252 | BIRC6 | frameshift_deleti  on | exon13 | c.3289delA | p.K1097fs | . | . |
| CTC2 | 2 | 32819024 | GTC | G | 0.25 | NM_016252 | BIRC6 | frameshift_deleti  on | exon67 | c.13399_13400  del | p.S4467fs | . | . |
| CTC2 | 2 | 32819062 | CAG | C | 0.25 | NM_016252 | BIRC6 | frameshift_deleti  on | exon67 | c.13437_13438  del | p.P4479fs | . | . |
| CTC2 | 2 | 33411997 | AC | A | 0.171 | NM_000627 | LTBP1 | frameshift_deleti  on | exon2 | c.299delC | p.T100fs | . | . |
| CTC2 | 2 | 33500925 | TG | T | 0.448 | NM_000627 | LTBP1 | frameshift_deleti  on | exon14 | c.1950delG | p.V650fs | . | . |
| CTC2 | 2 | 37509744 | TG | T | 0.273 | NM_005813 | PRKD3 | frameshift_deleti  on | exon6 | c.928delC | p.H310fs | . | . |
| CTC2 | 2 | 37520327 | T | TA | 0.227 | NM_005813 | PRKD3 | frameshift_inserti  on | exon2 | c.375dupT | p.T126fs | . | . |
| CTC2 | 2 | 37586889 | AT | A | 1 | NM_012413 | QPCT | frameshift_deleti  on | exon3 | c.435delT | p.Y145fs | . | . |
| CTC2 | 2 | 38179336 | TAAA | T | 0.178 | NM_144713 | RMDN2 | nonframeshift_de  letion | exon2 | c.979_981del | p.327_327del | . | . |
| CTC2 | 2 | 38298228 | GT | G | 0.61 | NM_000104 | CYP1B1 | frameshift_deleti  on | exon3 | c.1268delA | p.N423fs | . | . |
| CTC2 | 2 | 39250240 | T | TC | 0.25 | NM_005633 | SOS1 | frameshift_inserti  on | exon10 | c.1328_1329ins  G | p.E443fs | . | . |
| CTC2 | 2 | 39971579 | G | GA | 0.283 | NM_025264 | THUMPD2 | frameshift_inserti  on | exon9 | c.1117dupT | p.S373fs | . | . |
| CTC2 | 2 | 43459871 | CA | C | 0.3 | NM_001083953 | THADA | frameshift_deleti  on | exon37 | c.5446delT | p.C1816fs | . | . |
| CTC2 | 2 | 43924445 | GC | G | 0.788 | NM_172069 | PLEKHH2 | frameshift_deleti  on | exon7 | c.639delC | p.S213fs | . | . |
| CTC2 | 2 | 43927371 | GA | G | 0.327 | NM_172069 | PLEKHH2 | frameshift_deleti  on | exon8 | c.1275delA | p.G425fs | . | . |
| CTC2 | 2 | 43927691 | AAG | A | 0.135 | NM_172069 | PLEKHH2 | frameshift_deleti  on | exon8 | c.1595_1596del | p.K532fs | . | . |
| CTC2 | 2 | 43927692 | AG | A | 0.4 | NM_172069 | PLEKHH2 | frameshift_deleti  on | exon8 | c.1596delG | p.K532fs | . | . |
| CTC2 | 2 | 44047061 | A | AT | 0.409 | NM_022436 | ABCG5 | frameshift_inserti  on | exon11 | c.1641dupA | p.F548fs | . | . |
| CTC2 | 2 | 45704166 | A | AC | 1 | NM_018079 | SRBD1 | frameshift_inserti  on | exon16 | c.2014_2015ins  G | p.I672fs | . | . |
| CTC2 | 2 | 47604157 | C | CT | 1 | NM_002354 | EPCAM | frameshift_inserti  on | exon5 | c.497dupT | p.L166fs | . | . |
| CTC2 | 2 | 48018131 | TG | T | 1 | NM_000179 | MSH6 | frameshift_deleti  on | exon2 | c.327delG | p.L109fs | . | . |
| CTC2 | 2 | 50723130 | TTGA | T | 0.131 | NM_004801 | NRXN1 | nonframeshift_de  letion | exon15 | c.2980_2982del | p.994_994del | . | . |

| CTC2 | 2 | 50758466 | TG | T | 0.476 | NM_004801 | NRXN1 | frameshift_deleti  on | exon11 | c.2245delC | p.Q749fs | . | . |
| --- | --- | --- | --- | --- | --- | --- | --- | --- | --- | --- | --- | --- | --- |
| CTC2 | 2 | 54858668 | CA | C | 0.455 | NM_178313 | SPTBN1 | frameshift_deleti  on | exon15 | c.3446delA | p.Q1149fs | . | . |
| CTC2 | 2 | 60679784 | TG | T | 0.433 | NM_018014 | BCL11A | frameshift_deleti  on | exon5 | c.2247delC | p.P749fs | . | . |
| CTC2 | 2 | 60688968 | AG | A | 1 | NM_018014 | BCL11A | frameshift_deleti  on | exon4 | c.1078delC | p.L360fs | . | . |
| CTC2 | 2 | 60689445 | CT | C | 0.835 | NM_018014 | BCL11A | frameshift_deleti  on | exon4 | c.601delA | p.S201fs | . | . |
| CTC2 | 2 | 61145683 | AC | A | 0.889 | NM_002908 | REL | frameshift_deleti  on | exon7 | c.796delC | p.P266fs | . | . |
| CTC2 | 2 | 61413782 | TG | T | 0.302 | NM_152392 | AHSA2 | frameshift_deleti  on | exon6 | c.276delG | p.V92fs | . | . |
| CTC2 | 2 | 61712929 | CA | C | 0.413 | NM_003400 | XPO1 | frameshift_deleti  on | exon20 | c.2481delT | p.F827fs | . | . |
| CTC2 | 2 | 61722674 | AT | A | 0.857 | NM_003400 | XPO1 | frameshift_deleti  on | exon11 | c.962delA | p.N321fs | . | . |
| CTC2 | 2 | 62099356 | AC | A | 0.273 | NM_001256721 | CCT4 | frameshift_deleti  on | exon11 | c.1261delG | p.V421fs | . | . |
| CTC2 | 2 | 62450267 | AG | A | 0.378 | NM_006577 | B3GNT2 | frameshift_deleti  on | exon2 | c.913delG | p.G305fs | . | . |
| CTC2 | 2 | 65296910 | AC | A | 1 | NM_015147 | CEP68 | frameshift_deleti  on | exon2 | c.333delC | p.D111fs | . | . |
| CTC2 | 2 | 65301418 | AT | A | 0.313 | NM_015147 | CEP68 | frameshift_deleti  on | exon4 | c.1888delT | p.F630fs | . | . |
| CTC2 | 2 | 66667059 | CGGGG | C | 0.194 | NM_002398 | MEIS1 | frameshift_deleti  on | exon3 | c.325_328del | p.G109fs | . | . |
| CTC2 | 2 | 67630780 | GA | G | 0.4 | NM_019002 | ETAA1 | frameshift_deleti  on | exon5 | c.967delA | p.K323fs | . | . |
| CTC2 | 2 | 67631372 | AG | A | 0.429 | NM_019002 | ETAA1 | frameshift_deleti  on | exon5 | c.1559delG | p.R520fs | . | . |
| CTC2 | 2 | 69201773 | T | TA | 0.161 | NM_019617 | GKN1 | frameshift_inserti  on | exon1 | c.6_7insA | p.L2fs | . | . |
| CTC2 | 2 | 69741753 | CTGT | C | 0.994 | NM_014911 | AAK1 | nonframeshift_de  letion | exon13 | c.1623_1625del | p.541_542del | . | . |
| CTC2 | 2 | 69741759 | TTGTTGC | T | 0.675 | NM_014911 | AAK1 | nonframeshift_de  letion | exon13 | c.1614_1619del | p.538_540del | . | . |
| CTC2 | 2 | 69741762 | TTGC | T | 0.891 | NM_014911 | AAK1 | nonframeshift_de  letion | exon13 | c.1614_1616del | p.538_539del | . | . |
| CTC2 | 2 | 70504509 | TA | T | 0.727 | NM_016297 | PCYOX1 | frameshift_deleti  on | exon6 | c.1504delA | p.K502fs | . | . |
| CTC2 | 2 | 72362514 | TC | T | 0.919 | NM_001277742 | CYP26B1 | frameshift_deleti  on | exon2 | c.238delG | p.E80fs | . | . |
| CTC2 | 2 | 73452047 | CT | C | 0.25 | NM_006062 | SMYD5 | frameshift_deleti  on | exon11 | c.995delT | p.L332fs | . | . |
| CTC2 | 2 | 73675227 | T | TCTC | 1 | NM_015120 | ALMS1 | nonframeshift_in  sertion | exon8 | c.1570_1571ins  CTC | p.S524delinsSP | . | . |
| CTC2 | 2 | 73957839 | C | CA | 0.625 | NM_016058 | TPRKB | frameshift_inserti  on | exon4 | c.288dupT | p.G97fs | . | . |
| CTC2 | 2 | 74042223 | TA | T | 0.235 | NM_001080474 | C2orf78 | frameshift_deleti  on | exon3 | c.874delA | p.T292fs | . | . |
| CTC2 | 2 | 74042265 | GC | G | 0.724 | NM_001080474 | C2orf78 | frameshift_deleti  on | exon3 | c.916delC | p.P306fs | . | . |
| CTC2 | 2 | 74166144 | CA | C | 0.75 | NM_080916 | DGUOK | frameshift_deleti  on | exon2 | c.251delA | p.Q84fs | . | . |
| CTC2 | 2 | 74746339 | CG | C | 0.267 | NM_133637 | DQX1 | frameshift_deleti  on | exon11 | c.1824delC | p.D608fs | . | . |
| CTC2 | 2 | 74782720 | GC | G | 1 | NM_001381 | DOK1 | frameshift_deleti  on | exon3 | c.380delC | p.A127fs | . | . |
| CTC2 | 2 | 74884993 | AT | A | 0.5 | NM_004263 | SEMA4F | frameshift_deleti  on | exon4 | c.372delT | p.N124fs | . | . |
| CTC2 | 2 | 75923343 | CT | C | 0.812 | NM_001201334 | GCFC2 | frameshift_deleti  on | exon5 | c.308delA | p.K103fs | . | . |
| CTC2 | 2 | 85280486 | TA | T | 0.607 | NM_020122 | KCMF1 | frameshift_deleti  on | exon7 | c.1101delA | p.L367fs | . | . |
| CTC2 | 2 | 85554258 | CT | C | 0.714 | NM_001206841 | TGOLN2 | frameshift_deleti  on | exon2 | c.596delA | p.E199fs | . | . |
| CTC2 | 2 | 85598251 | CT | C | 0.4 | NM_032213 | ELMOD3 | frameshift_deleti  on | exon7 | c.404delT | p.L135fs | . | . |
| CTC2 | 2 | 85662574 | CTG | C | 0.193 | NM_198482 | SH2D6 | frameshift_deleti  on | exon2 | c.208_209del | p.C70fs | . | . |
| CTC2 | 2 | 86255723 | C | CT | 0.312 | NM_015425 | POLR1A | frameshift_inserti  on | exon32 | c.4890_4891ins  A | p.V1631fs | . | . |
| CTC2 | 2 | 86327243 | TC | T | 0.536 | NM_015425 | POLR1A | frameshift_deleti  on | exon2 | c.129delG | p.G43fs | . | . |
| CTC2 | 2 | 86361973 | AT | A | 0.333 | NM_017952 | PTCD3 | frameshift_deleti  on | exon21 | c.1642delT | p.F548fs | . | . |
| CTC2 | 2 | 86437716 | CT | C | 0.75 | NM_016622 | MRPL35 | frameshift_deleti  on | exon4 | c.493delT | p.F165fs | . | . |
| CTC2 | 2 | 86697498 | CA | C | 0.4 | NM_001146688 | KDM3A | frameshift_deleti  on | exon11 | c.1692delA | p.S564fs | . | . |
| CTC2 | 2 | 88874907 | GC | G | 0.505 | NM_004836 | EIF2AK3 | frameshift_deleti  on | exon13 | c.2093delG | p.R698fs | . | . |
| CTC2 | 2 | 95818928 | TC | T | 0.235 | NM_032788 | ZNF514 | frameshift_deleti  on | exon3 | c.70delG | p.D24fs | . | . |
| CTC2 | 2 | 95944853 | GC | G | 0.659 | NM_001165977 | PROM2 | frameshift_deleti  on | exon10 | c.1236delC | p.R412fs | . | . |
| CTC2 | 2 | 96967286 | CA | C | 0.455 | NM_014014 | SNRNP200 | frameshift_deleti  on | exon4 | c.549delT | p.G183fs | . | . |
| CTC2 | 2 | 97327999 | GA | G | 0.378 | NM_001113382 | FER1L5 | frameshift_deleti  on | exon12 | c.1016delA | p.E339fs | . | . |
| CTC2 | 2 | 97335931 | TC | T | 0.184 | NM_001113382 | FER1L5 | frameshift_deleti  on | exon17 | c.1424delC | p.S475fs | . | . |
| CTC2 | 2 | 97361603 | AC | A | 0.583 | NM_001113382 | FER1L5 | frameshift_deleti  on | exon35 | c.4101delC | p.D1367fs | . | . |
| CTC2 | 2 | 97533576 | GC | G | 0.395 | NM_017789 | SEMA4C | frameshift_deleti  on | exon2 | c.47delG | p.G16fs | . | . |
| CTC2 | 2 | 97637770 | CCT | C | 0.958 | NM_001122646 | FAM178B | frameshift_deleti  on | exon3 | c.430_431del | p.R144fs | . | . |
| CTC2 | 2 | 98834401 | GT | G | 0.739 | NM_144992 | VWA3B | frameshift_deleti  on | exon14 | c.1930delT | p.F644fs | . | . |
| CTC2 | 2 | 99778880 | CT | C | 0.44 | NM_145199 | LIPT1 | frameshift_deleti  on | exon2 | c.461delT | p.L154fs | . | . |
| CTC2 | 2 | 100009439 | GA | G | 0.227 | NM_015904 | EIF5B | frameshift_deleti  on | exon18 | c.2759delA | p.E920fs | . | . |
| CTC2 | 2 | 100623798 | AC | A | 0.396 | NM_001025108 | AFF3 | frameshift_deleti  on | exon5 | c.373delG | p.V125fs | . | . |
| CTC2 | 2 | 101624364 | AT | A | 0.514 | NM_001102426 | TBC1D8 | frameshift_deleti  on | exon20 | c.3341delA | p.N1114fs | . | . |
| CTC2 | 2 | 102475508 | TCAGCA  GCAGCT | T | 0.54 | NM_001242559 | MAP4K4 | nonframeshift_de  letion | exon14 | c.1447_1461del | p.483_487del | . | . |
| CTC2 | 2 | 102475511 | GC | G | 0.177 | NM_001242559 | MAP4K4 | frameshift_deleti  on | exon14 | c.1450delC | p.Q484fs | . | . |

| CTC2 | 2 | 102638678 | A | AT | 0.333 | NM_001261419 | IL1R2 | frameshift_inserti  on | exon6 | c.719dupT | p.I240fs | . | . |
| --- | --- | --- | --- | --- | --- | --- | --- | --- | --- | --- | --- | --- | --- |
| CTC2 | 2 | 105897104 | AC | A | 0.412 | NM_001142621 | TGFBRAP1 | frameshift_deleti  on | exon6 | c.1197delG | p.R399fs | . | . |
| CTC2 | 2 | 108489257 | TA | T | 0.4 | NM_182588 | RGPD4 | frameshift_deleti  on | exon20 | c.4798delA | p.K1600fs | . | . |
| CTC2 | 2 | 109102318 | TA | T | 0.6 | NM_181453 | GCC2 | frameshift_deleti  on | exon15 | c.3747delA | p.L1249fs | . | . |
| CTC2 | 2 | 109463370 | C | CA | 0.234 | NM_144978 | CCDC138 | frameshift_inserti  on | exon12 | c.1501dupA | p.L500fs | . | . |
| CTC2 | 2 | 109539895 | TC | T | 0.237 | NM_022336 | EDAR | frameshift_deleti  on | exon5 | c.370delG | p.E124fs | . | . |
| CTC2 | 2 | 110303805 | TG | T | 0.302 | NM_178584 | 10-Sep | frameshift_deleti  on | exon9 | c.1101delC | p.A367fs | . | . |
| CTC2 | 2 | 111427090 | AT | A | 0.55 | NM_001278616 | BUB1 | frameshift_deleti  on | exon5 | c.446delA | p.N149fs | . | . |
| CTC2 | 2 | 112614415 | AC | A | 0.962 | NM_022662 | ANAPC1 | frameshift_deleti  on | exon12 | c.1406delG | p.G469fs | . | . |
| CTC2 | 2 | 112944986 | TG | T | 1 | NM_001128165 | FBLN7 | frameshift_deleti  on | exon7 | c.1086delG | p.L362fs | . | . |
| CTC2 | 2 | 112945059 | C | CT | 1 | NM_001128165 | FBLN7 | frameshift_inserti  on | exon7 | c.1159dupT | p.I386fs | . | . |
| CTC2 | 2 | 113089227 | TA | T | 1 | NM_198581 | ZC3H6 | frameshift_deleti  on | exon12 | c.2733delA | p.L911fs | . | . |
| CTC2 | 2 | 113514128 | GT | G | 0.436 | NM_152515 | CKAP2L | frameshift_deleti  on | exon4 | c.819delA | p.K273fs | . | . |
| CTC2 | 2 | 113763452 | GA | G | 0.424 | NM_014440 | IL36A | frameshift_deleti  on | exon1 | c.5delA | p.E2fs | . | . |
| CTC2 | 2 | 120194651 | A | AGTGT  GC | 1 | NM_183240 | TMEM37 | nonframeshift_in  sertion | exon2 | c.208_209insGT  GTGC | p.T70delinsSVP | . | . |
| CTC2 | 2 | 120231082 | TG | T | 0.683 | NM_002980 | SCTR | frameshift_deleti  on | exon4 | c.351delC | p.P117fs | . | . |
| CTC2 | 2 | 125367394 | CT | C | 0.491 | NM_130773 | CNTNAP5 | frameshift_deleti  on | exon12 | c.1771delT | p.C591fs | . | . |
| CTC2 | 2 | 125367405 | AC | A | 0.509 | NM_130773 | CNTNAP5 | stopgain | exon12 | c.1782delC | p.Y594X | . | . |
| CTC2 | 2 | 128047345 | CG | C | 0.452 | NM_000122 | ERCC3 | frameshift_deleti on | exon5 | c.576delC | p.P192fs | . | . |
| CTC2 | 2 | 128324342 | TC | T | 0.186 | NM_001080527 | MYO7B | frameshift_deleti  on | exon5 | c.411delC | p.I137fs | . | . |
| CTC2 | 2 | 128477869 | G | GT | 0.5 | NM_018383 | WDR33 | frameshift_inserti  on | exon16 | c.1729_1730ins  A | p.S577fs | . | . |
| CTC2 | 2 | 128930218 | CA | C | 0.69 | NM_020120 | UGGT1 | frameshift_deleti  on | exon29 | c.3177delA | p.A1059fs | . | . |
| CTC2 | 2 | 131520338 | C | CA | 0.753 | NM_001105193 | AMER3 | frameshift_inserti  on | exon2 | c.694dupA | p.C231fs | . | . |
| CTC2 | 2 | 132238237 | TC | T | 0.3 | NM_080386 | TUBA3D | frameshift_deleti  on | exon4 | c.972delC | p.V324fs | . | . |
| CTC2 | 2 | 133531390 | T | TA | 0.375 | NM_207481 | NCKAP5 | frameshift_inserti  on | exon14 | c.1169dupT | p.I390fs | . | . |
| CTC2 | 2 | 133540985 | AC | A | 0.5 | NM_207363 | NCKAP5 | frameshift_deleti  on | exon14 | c.3398delG | p.G1133fs | . | . |
| CTC2 | 2 | 136107718 | TC | T | 0.6 | NM_001286568 | ZRANB3 | frameshift_deleti  on | exon5 | c.426delG | p.L142fs | . | . |
| CTC2 | 2 | 136389511 | ACC | A | 0.409 | NM_001282800 | R3HDM1 | frameshift_deleti  on | exon6 | c.471_472del | p.D157fs | . | . |
| CTC2 | 2 | 136594183 | TC | T | 0.236 | NM_002299 | LCT | frameshift_deleti  on | exon1 | c.556delG | p.E186fs | . | . |
| CTC2 | 2 | 136627854 | TA | T | 0.792 | NM_005915 | MCM6 | frameshift_deleti  on | exon3 | c.331delT | p.Y111fs | . | . |
| CTC2 | 2 | 139428884 | C | CA | 0.571 | NM_007226 | NXPH2 | frameshift_inserti  on | exon2 | c.402dupT | p.V135fs | . | . |
| CTC2 | 2 | 140992369 | G | GT | 0.158 | NM_018557 | LRP1B | frameshift_inserti  on | exon90 | c.13644dupA | p.P4549fs | . | . |
| CTC2 | 2 | 141201968 | A | AT | 0.381 | NM_018557 | LRP1B | frameshift_inserti  on | exon65 | c.10224dupA | p.C3409fs | . | . |
| CTC2 | 2 | 141474343 | AT | A | 0.255 | NM_018557 | LRP1B | frameshift_deleti  on | exon36 | c.5800delA | p.I1934fs | . | . |
| CTC2 | 2 | 145147156 | AT | A | 0.526 | NM_001171653 | ZEB2 | frameshift_deleti  on | exon9 | c.3434delA | p.N1145fs | . | . |
| CTC2 | 2 | 145156369 | GT | G | 0.833 | NM_001171653 | ZEB2 | frameshift_deleti  on | exon7 | c.2312delA | p.N771fs | . | . |
| CTC2 | 2 | 149226527 | CCT | C | 0.75 | NM_018328 | MBD5 | frameshift_deleti  on | exon9 | c.1016_1017del | p.P339fs | . | . |
| CTC2 | 2 | 149247776 | G | GA | 0.167 | NM_018328 | MBD5 | frameshift_inserti  on | exon12 | c.3877dupA | p.G1292fs | . | . |
| CTC2 | 2 | 149868118 | ATC | A | 0.962 | NM_004522 | KIF5C | frameshift_deleti  on | exon25 | c.2803_2804del | p.S935fs | . | . |
| CTC2 | 2 | 152285340 | CT | C | 0.5 | NM_001177665 | RIF1 | frameshift_deleti  on | exon8 | c.830delT | p.L277fs | . | . |
| CTC2 | 2 | 152447939 | TG | T | 0.474 | NM_001164507 | NEB | frameshift_deleti  on | exon96 | c.14856delC | p.V4952fs | . | . |
| CTC2 | 2 | 153476066 | G | GCCA | 0.833 | NM_052905 | FMNL2 | nonframeshift_in  sertion | exon15 | c.1671_1672ins  CCA | p.P557delinsPP | . | . |
| CTC2 | 2 | 153515484 | CT | C | 1 | NM_017892 | PRPF40A | frameshift_deleti  on | exon24 | c.2545delA | p.S849fs | . | . |
| CTC2 | 2 | 157186581 | CA | C | 0.467 | NM_006186 | NR4A2 | frameshift_deleti  on | exon3 | c.117delT | p.F39fs | . | . |
| CTC2 | 2 | 157439418 | TG | T | 0.892 | NM_000408 | GPD2 | frameshift_deleti  on | exon17 | c.2173delG | p.G725fs | . | . |
| CTC2 | 2 | 159535149 | CA | C | 0.78 | NM_001005476 | PKP4 | frameshift_deleti  on | exon20 | c.3185delA | p.Q1062fs | . | . |
| CTC2 | 2 | 160084335 | GA | G | 0.36 | NM_001145909 | TANC1 | frameshift_deleti  on | exon25 | c.3886delA | p.K1296fs | . | . |
| CTC2 | 2 | 165381517 | CA | C | 1 | NM_004490 | GRB14 | frameshift_deleti  on | exon5 | c.674delT | p.L225fs | . | . |
| CTC2 | 2 | 165551345 | A | AT | 0.66 | NM_001278461 | COBLL1 | frameshift_inserti  on | exon11 | c.2556dupA | p.F853fs | . | . |
| CTC2 | 2 | 165557044 | CT | C | 0.524 | NM_001278461 | COBLL1 | frameshift_deleti  on | exon9 | c.1447delA | p.S483fs | . | . |
| CTC2 | 2 | 166179723 | CT | C | 0.364 | NM_001040143 | SCN2A | frameshift_deleti  on | exon11 | c.1730delT | p.L577fs | . | . |
| CTC2 | 2 | 166848251 | TTTGG | T | 0.182 | NM_001165963 | SCN1A | frameshift_deleti  on | exon26 | c.5530_5533del | p.P1844fs | . | . |
| CTC2 | 2 | 166908262 | CA | C | 0.308 | NM_001165963 | SCN1A | frameshift_deleti  on | exon6 | c.930delT | p.F310fs | . | . |
| CTC2 | 2 | 167266730 | T | TA | 0.349 | NM_002976 | SCN7A | frameshift_inserti  on | exon23 | c.3710dupT | p.L1237fs | . | . |
| CTC2 | 2 | 169764478 | AT | A | 0.376 | NM_021176 | G6PC2 | frameshift_deleti  on | exon5 | c.958delT | p.F320fs | . | . |
| CTC2 | 2 | 170070317 | A | AT | 0.244 | NM_004525 | LRP2 | frameshift_inserti  on | exon36 | c.5889_5890ins  A | p.W1964fs | . | . |
| CTC2 | 2 | 170077068 | CA | C | 0.357 | NM_004525 | LRP2 | stopgain | exon34 | c.5543delT | p.L1848X | . | . |
| CTC2 | 2 | 170131698 | CT | C | 0.587 | NM_004525 | LRP2 | frameshift_deleti on | exon14 | c.1822delA | p.S608fs | . | . |

| CTC2 | 2 | 170591532 | TA | T | 0.4 | NM_144711 | KLHL23 | frameshift_deleti  on | exon2 | c.9delA | p.L3fs | . | . |
| --- | --- | --- | --- | --- | --- | --- | --- | --- | --- | --- | --- | --- | --- |
| CTC2 | 2 | 171702067 | TC | T | 1 | NM_000817 | GAD1 | frameshift_deleti  on | exon8 | c.804delC | p.F268fs | . | . |
| CTC2 | 2 | 171704238 | CT | C | 0.68 | NM_000817 | GAD1 | frameshift_deleti  on | exon11 | c.1056delT | p.A352fs | . | . |
| CTC2 | 2 | 172935782 | AT | A | 0.913 | NM_199227 | METAP1D | frameshift_deleti  on | exon6 | c.689delT | p.I230fs | . | . |
| CTC2 | 2 | 173955831 | AG | A | 0.389 | NM_016653 | ZAK | frameshift_deleti  on | exon2 | c.73delG | p.G25fs | . | . |
| CTC2 | 2 | 174820757 | TG | T | 1 | NM_001017371 | SP3 | stopgain | exon2 | c.278delC | p.S93X | . | . |
| CTC2 | 2 | 178416194 | ATT | A | 0.455 | NM_152517 | TTC30B | frameshift_deleti on | exon1 | c.1296_1297del | p.K432fs | . | . |
| CTC2 | 2 | 178482132 | ATT | A | 0.613 | NM_152275 | TTC30A | frameshift_deleti  on | exon1 | c.1296_1297del | p.K432fs | . | . |
| CTC2 | 2 | 178494173 | G | GGGA | 1 | NM_001077196 | PDE11A | nonframeshift_in  sertion | exon17 | c.1431_1432ins  TCC | p.P478delinsSP | . | . |
| CTC2 | 2 | 179251857 | TG | T | 0.286 | NM_001201481 | OSBPL6 | frameshift_deleti  on | exon19 | c.2055delG | p.M685fs | . | . |
| CTC2 | 2 | 179393566 | AT | A | 0.194 | NM_003319 | TTN | frameshift_deleti  on | exon188 | c.79716delA | p.K26572fs | . | . |
| CTC2 | 2 | 179394730 | AT | A | 0.607 | NM_003319 | TTN | frameshift_deleti  on | exon187 | c.79292delA | p.N26431fs | . | . |
| CTC2 | 2 | 179394831 | CT | C | 0.459 | NM_003319 | TTN | frameshift_deleti  on | exon187 | c.79191delA | p.Q26397fs | . | . |
| CTC2 | 2 | 179396458 | TA | T | 0.394 | NM_003319 | TTN | frameshift_deleti  on | exon186 | c.77688delT | p.F25896fs | . | . |
| CTC2 | 2 | 179412814 | GT | G | 0.354 | NM_003319 | TTN | frameshift_deleti  on | exon167 | c.66343delA | p.T22115fs | . | . |
| CTC2 | 2 | 179434313 | G | GT | 0.253 | NM_003319 | TTN | frameshift_inserti  on | exon154 | c.49350dupA | p.L16451fs | . | . |
| CTC2 | 2 | 179438172 | AG | A | 0.75 | NM_003319 | TTN | frameshift_deleti  on | exon154 | c.45491delC | p.P15164fs | . | . |
| CTC2 | 2 | 179449418 | CTT | C | 0.261 | NM_003319 | TTN | frameshift_deleti  on | exon138 | c.37753_37754  del | p.K12585fs | . | . |
| CTC2 | 2 | 179454090 | TA | T | 0.205 | NM_003319 | TTN | frameshift_deleti  on | exon132 | c.35166delT | p.I11722fs | . | . |
| CTC2 | 2 | 179454363 | C | CA | 0.906 | NM_003319 | TTN | frameshift_inserti  on | exon132 | c.34893dupT | p.G11632fs | . | . |
| CTC2 | 2 | 179468897 | GT | G | 0.444 | NM_003319 | TTN | frameshift_deleti  on | exon110 | c.27321delA | p.K9107fs | . | . |
| CTC2 | 2 | 179516423 | C | CT | 0.714 | NM_133378 | TTN | frameshift_inserti  on | exon160 | c.32130_32131i  nsA | p.A10711fs | . | . |
| CTC2 | 2 | 179550015 | AT | A | 0.65 | NM_133378 | TTN | frameshift_deleti  on | exon126 | c.28702delA | p.I9568fs | . | . |
| CTC2 | 2 | 179554091 | A | AT | 0.216 | NM_133378 | TTN | frameshift_inserti  on | exon121 | c.28204dupA | p.I9402fs | . | . |
| CTC2 | 2 | 179556743 | CT | C | 0.75 | NM_133378 | TTN | frameshift_deleti  on | exon118 | c.28029delA | p.K9343fs | . | . |
| CTC2 | 2 | 179571350 | CT | C | 0.677 | NM_133378 | TTN | frameshift_deleti  on | exon99 | c.25518delA | p.K8506fs | . | . |
| CTC2 | 2 | 179593776 | TG | T | 0.186 | NM_133378 | TTN | frameshift_deleti  on | exon62 | c.15256delC | p.Q5086fs | . | . |
| CTC2 | 2 | 179613123 | AT | A | 0.714 | NM_133379 | TTN | frameshift_deleti  on | exon46 | c.14003delA | p.N4668fs | . | . |
| CTC2 | 2 | 179614925 | CT | C | 0.429 | NM_133379 | TTN | frameshift_deleti  on | exon46 | c.12201delA | p.K4067fs | . | . |
| CTC2 | 2 | 180810141 | AT | A | 0.54 | NM_020943 | CWC22 | frameshift_deleti  on | exon20 | c.2441delA | p.N814fs | . | . |
| CTC2 | 2 | 185802211 | C | CACA | 1 | NM_194250 | ZNF804A | nonframeshift_in  sertion | exon4 | c.2088_2089ins  ACA | p.N696delinsN  T | . | . |
| CTC2 | 2 | 186654176 | GA | G | 0.444 | NM_173651 | FSIP2 | frameshift_deleti  on | exon16 | c.2581delA | p.K861fs | . | . |
| CTC2 | 2 | 186666165 | CA | C | 0.625 | NM_173651 | FSIP2 | frameshift_deleti  on | exon17 | c.12400delA | p.K4134fs | . | . |
| CTC2 | 2 | 186671860 | GA | G | 0.375 | NM_173651 | FSIP2 | frameshift_deleti  on | exon17 | c.18095delA | p.E6032fs | . | . |
| CTC2 | 2 | 187618665 | TG | T | 1 | NM_177454 | FAM171B | frameshift_deleti  on | exon6 | c.902delG | p.W301fs | . | . |
| CTC2 | 2 | 188217036 | AT | A | 1 | NM_001271751 | CALCRL | frameshift_deleti  on | exon12 | c.932delA | p.N311fs | . | . |
| CTC2 | 2 | 190437653 | TG | T | 0.958 | NM_014585 | SLC40A1 | stopgain | exon4 | c.305delC | p.S102X | . | . |
| CTC2 | 2 | 190439978 | CA | C | 0.364 | NM_014585 | SLC40A1 | stopgain | exon3 | c.179delT | p.L60X | . | . |
| CTC2 | 2 | 190531541 | TG | T | 0.444 | NM_019048 | ASNSD1 | frameshift_deleti on | exon4 | c.684delG | p.V228fs | . | . |
| CTC2 | 2 | 197171952 | TG | T | 0.38 | NM_020760 | HECW2 | frameshift_deleti  on | exon12 | c.2590delC | p.Q864fs | . | . |
| CTC2 | 2 | 197187329 | CA | C | 0.947 | NM_020760 | HECW2 | frameshift_deleti  on | exon7 | c.756delT | p.F252fs | . | . |
| CTC2 | 2 | 197777687 | TA | T | 0.314 | NM_024989 | PGAP1 | frameshift_deleti  on | exon4 | c.567delT | p.F189fs | . | . |
| CTC2 | 2 | 198436980 | CT | C | 0.516 | NM_144629 | RFTN2 | frameshift_deleti  on | exon9 | c.1257delA | p.K419fs | . | . |
| CTC2 | 2 | 198508896 | CT | C | 0.938 | NM_144629 | RFTN2 | frameshift_deleti  on | exon3 | c.423delA | p.K141fs | . | . |
| CTC2 | 2 | 201342593 | GA | G | 0.5 | NM_001100424 | SPATS2L | frameshift_deleti  on | exon12 | c.1310delA | p.E437fs | . | . |
| CTC2 | 2 | 201355001 | TC | T | 0.375 | NM_152387 | KCTD18 | frameshift_deleti  on | exon7 | c.1102delG | p.D368fs | . | . |
| CTC2 | 2 | 201397808 | CA | C | 0.846 | NM_001160033 | SGOL2 | frameshift_deleti  on | exon2 | c.111delA | p.S37fs | . | . |
| CTC2 | 2 | 201464438 | CA | C | 0.591 | NM_001159 | AOX1 | frameshift_deleti  on | exon5 | c.406delA | p.T136fs | . | . |
| CTC2 | 2 | 202323552 | GA | G | 0.41 | NM_001206864 | STRADB | frameshift_deleti  on | exon3 | c.59delA | p.E20fs | . | . |
| CTC2 | 2 | 202545680 | GA | G | 0.314 | NM_033066 | MPP4 | frameshift_deleti  on | exon10 | c.809delT | p.F270fs | . | . |
| CTC2 | 2 | 202572634 | CA | C | 0.34 | NM_020919 | ALS2 | frameshift_deleti  on | exon28 | c.4360delT | p.C1454fs | . | . |
| CTC2 | 2 | 203420125 | AG | A | 0.885 | NM_001204 | BMPR2 | frameshift_deleti  on | exon12 | c.1738delG | p.G580fs | . | . |
| CTC2 | 2 | 203420128 | GGA | G | 0.375 | NM_001204 | BMPR2 | frameshift_deleti  on | exon12 | c.1741_1742del | p.E581fs | . | . |
| CTC2 | 2 | 206480261 | AG | A | 0.263 | NM_057177 | PARD3B | frameshift_deleti  on | exon22 | c.3136delG | p.G1046fs | . | . |
| CTC2 | 2 | 206628586 | GT | G | 0.364 | NM_003872 | NRP2 | frameshift_deleti  on | exon13 | c.2234delT | p.V745fs | . | . |
| CTC2 | 2 | 206927622 | AC | A | 0.571 | NM_017759 | INO80D | frameshift_deleti  on | exon3 | c.118delG | p.V40fs | . | . |
| CTC2 | 2 | 206927636 | GA | G | 0.523 | NM_017759 | INO80D | frameshift_deleti  on | exon3 | c.104delT | p.F35fs | . | . |
| CTC2 | 2 | 207988814 | TG | T | 0.892 | NM_001270943 | KLF7 | frameshift_deleti  on | exon2 | c.317delC | p.P106fs | . | . |

| CTC2 | 2 | 210858122 | G | GA | 0.359 | NM_182587 | UNC80 | frameshift_inserti  on | exon61 | c.9211dupA | p.R3070fs | . | . |
| --- | --- | --- | --- | --- | --- | --- | --- | --- | --- | --- | --- | --- | --- |
| CTC2 | 2 | 216200783 | GA | G | 0.6 | NM_004044 | ATIC | frameshift_deleti  on | exon11 | c.1035delA | p.G345fs | . | . |
| CTC2 | 2 | 217332689 | G | GA | 0.375 | NM_001127207 | SMARCAL1 | frameshift_inserti  on | exon14 | c.2165dupA | p.E722fs | . | . |
| CTC2 | 2 | 218712886 | GGCT | G | 0.133 | NM_022648 | TNS1 | nonframeshift_de  letion | exon17 | c.1976_1978del | p.659_660del | . | . |
| CTC2 | 2 | 218999694 | TG | T | 0.416 | NM_001557 | CXCR2 | frameshift_deleti  on | exon3 | c.171delG | p.L57fs | . | . |
| CTC2 | 2 | 219508272 | G | GT | 0.278 | NM_001105537 | ZNF142 | frameshift_inserti  on | exon8 | c.2966dupA | p.N989fs | . | . |
| CTC2 | 2 | 220078020 | AG | A | 0.48 | NM_005689 | ABCB6 | frameshift_deleti  on | exon12 | c.1747delC | p.L583fs | . | . |
| CTC2 | 2 | 223489176 | TG | T | 0.294 | NM_005687 | FARSB | frameshift_deleti  on | exon12 | c.984delC | p.A328fs | . | . |
| CTC2 | 2 | 223559246 | TG | T | 0.182 | NM_058165 | MOGAT1 | frameshift_deleti  on | exon4 | c.645delG | p.L215fs | . | . |
| CTC2 | 2 | 224849551 | TG | T | 0.303 | NM_001136528 | SERPINE2 | frameshift_deleti  on | exon5 | c.801delC | p.A267fs | . | . |
| CTC2 | 2 | 224863047 | A | AT | 0.28 | NM_001136528 | SERPINE2 | frameshift_inserti  on | exon3 | c.271dupA | p.I91fs | . | . |
| CTC2 | 2 | 225360635 | TA | T | 0.753 | NM_001257197 | CUL3 | frameshift_deleti  on | exon12 | c.1557delT | p.S519fs | . | . |
| CTC2 | 2 | 225634992 | TG | T | 0.566 | NM_014689 | DOCK10 | frameshift_deleti  on | exon55 | c.6379delC | p.Q2127fs | . | . |
| CTC2 | 2 | 227662917 | TC | T | 0.909 | NM_005544 | IRS1 | frameshift_deleti  on | exon1 | c.537delG | p.L179fs | . | . |
| CTC2 | 2 | 227946863 | G | GC | 0.35 | NM_000092 | COL4A4 | frameshift_inserti  on | exon23 | c.1663dupG | p.A555fs | . | . |
| CTC2 | 2 | 228159763 | GA | G | 0.361 | NM_000091 | COL4A3 | frameshift_deleti  on | exon40 | c.3503delA | p.E1168fs | . | . |
| CTC2 | 2 | 228883556 | AC | A | 0.682 | NM_001142644 | SPHKAP | frameshift_deleti  on | exon7 | c.2013delG | p.L671fs | . | . |
| CTC2 | 2 | 228883767 | TG | T | 0.756 | NM_001142644 | SPHKAP | frameshift_deleti  on | exon7 | c.1802delC | p.P601fs | . | . |
| CTC2 | 2 | 230675871 | TC | T | 0.182 | NM_001284216 | TRIP12 | frameshift_deleti  on | exon11 | c.1000delG | p.D334fs | . | . |
| CTC2 | 2 | 231265734 | GA | G | 0.444 | NM_138402 | SP140L | frameshift_deleti  on | exon16 | c.1379delA | p.E460fs | . | . |
| CTC2 | 2 | 231308970 | AT | A | 0.303 | NM_001206703 | SP100 | frameshift_deleti  on | exon3 | c.274delT | p.F92fs | . | . |
| CTC2 | 2 | 231861005 | CCA | C | 0.223 | NM_139073 | SPATA3 | frameshift_deleti  on | exon1 | c.58_59del | p.Q20fs | . | . |
| CTC2 | 2 | 233712226 | CACA | C | 1 | NM_001103148 | GIGYF2 | nonframeshift_de  letion | exon26 | c.3612_3614del | p.1204_1205de  l | . | . |
| CTC2 | 2 | 234186242 | TG | T | 0.606 | NM_198890 | ATG16L1 | frameshift_deleti  on | exon6 | c.495delG | p.V165fs | . | . |
| CTC2 | 2 | 234368483 | AG | A | 0.463 | NM_003648 | DGKD | frameshift_deleti  on | exon22 | c.2644delG | p.G882fs | . | . |
| CTC2 | 2 | 234429739 | T | TTTC | 0.25 | NM_018218 | USP40 | nonframeshift_in  sertion | exon16 | c.2255_2256ins  GAA | p.K752delinsKK | . | . |
| CTC2 | 2 | 234669250 | TG | T | 0.26 | NM_000463 | UGT1A1 | frameshift_deleti  on | exon1 | c.318delG | p.L106fs | . | . |
| CTC2 | 2 | 234752872 | AG | A | 0.692 | NM_001282963 | HJURP | frameshift_deleti  on | exon4 | c.297delC | p.A99fs | . | . |
| CTC2 | 2 | 235951246 | AC | A | 0.471 | NM_014521 | SH3BP4 | frameshift_deleti  on | exon4 | c.1834delC | p.P612fs | . | . |
| CTC2 | 2 | 235961213 | TGAA | T | 0.364 | NM_014521 | SH3BP4 | nonframeshift_de  letion | exon5 | c.2487_2489del | p.829_830del | . | . |
| CTC2 | 2 | 236761414 | C | CCAGG | 0.33 | NM_001244888 | AGAP1 | frameshift_inserti  on | exon10 | c.1135_1136ins  CAGG | p.P379fs | . | . |
| CTC2 | 2 | 238233441 | TC | T | 0.194 | NM_057166 | COL6A3 | frameshift_deleti  on | exon41 | c.7688delG | p.G2563fs | . | . |
| CTC2 | 2 | 238253154 | TC | T | 0.138 | NM_057166 | COL6A3 | frameshift_deleti  on | exon33 | c.5685delG | p.V1895fs | . | . |
| CTC2 | 2 | 238664779 | TC | T | 0.35 | NM_001137551 | LRRFIP1 | frameshift_deleti  on | exon6 | c.529delC | p.Q177fs | . | . |
| CTC2 | 2 | 239171681 | GC | G | 0.173 | NM_022817 | PER2 | frameshift_deleti  on | exon10 | c.1064delG | p.G355fs | . | . |
| CTC2 | 2 | 241465151 | GC | G | 1 | NM_001282780 | ANKMY1 | frameshift_deleti  on | exon5 | c.595delG | p.A199fs | . | . |
| CTC2 | 2 | 241658469 | GC | G | 0.553 | NM_004321 | KIF1A | frameshift_deleti  on | exon44 | c.4864delG | p.A1622fs | . | . |
| CTC2 | 2 | 242066155 | GC | G | 1 | NM_001252119 | PASK | frameshift_deleti  on | exon10 | c.2174delG | p.G725fs | . | . |
| CTC2 | 2 | 242380937 | GC | G | 0.537 | NM_001282983 | FARP2 | frameshift_deleti  on | exon13 | c.1378delC | p.P460fs | . | . |
| CTC2 | 2 | 242572557 | AG | A | 0.5 | NM_015963 | THAP4 | frameshift_deleti  on | exon2 | c.1014delC | p.A338fs | . | . |
| CTC2 | 3 | 2924851 | CA | C | 0.333 | NM_001206955 | CNTN4 | frameshift_deleti  on | exon8 | c.676delA | p.K226fs | . | . |
| CTC2 | 3 | 3182290 | G | GA | 0.3 | NM_182916 | TRNT1 | frameshift_inserti  on | exon4 | c.440dupA | p.D147fs | . | . |
| CTC2 | 3 | 3887857 | T | TG | 1 | NM_020873 | LRRN1 | frameshift_inserti  on | exon2 | c.1533dupG | p.V511fs | . | . |
| CTC2 | 3 | 5022018 | GA | G | 0.6 | NM_003670 | BHLHE40 | frameshift_deleti  on | exon3 | c.184delA | p.K62fs | . | . |
| CTC2 | 3 | 9489684 | CA | C | 0.452 | NM_001080517 | SETD5 | frameshift_deleti  on | exon15 | c.2098delA | p.K700fs | . | . |
| CTC2 | 3 | 9934691 | GGC | G | 0.4 | NM_032492 | JAGN1 | frameshift_deleti  on | exon2 | c.183_184del | p.R61fs | . | . |
| CTC2 | 3 | 10119801 | C | CT | 1 | NM_001018115 | FANCD2 | frameshift_inserti  on | exon30 | c.2897dupT | p.L966fs | . | . |
| CTC2 | 3 | 12978167 | GC | G | 0.621 | NM_001134382 | IQSEC1 | frameshift_deleti  on | exon3 | c.348delG | p.G116fs | . | . |
| CTC2 | 3 | 13660478 | AC | A | 0.348 | NM_001004019 | FBLN2 | frameshift_deleti  on | exon7 | c.2015delC | p.T672fs | . | . |
| CTC2 | 3 | 14508142 | GC | G | 0.195 | NM_001134367 | SLC6A6 | frameshift_deleti  on | exon7 | c.852delC | p.R284fs | . | . |
| CTC2 | 3 | 14513771 | AT | A | 0.357 | NM_001134367 | SLC6A6 | frameshift_deleti  on | exon10 | c.1156delT | p.F386fs | . | . |
| CTC2 | 3 | 14861345 | AG | A | 0.375 | NM_152536 | FGD5 | frameshift_deleti  on | exon1 | c.768delG | p.K256fs | . | . |
| CTC2 | 3 | 15467866 | TC | T | 0.167 | NM_152396 | METTL6 | frameshift_deleti  on | exon2 | c.152delG | p.R51fs | . | . |
| CTC2 | 3 | 15686741 | CT | C | 0.367 | NM_000060 | BTD | frameshift_deleti  on | exon4 | c.1379delT | p.L460fs | . | . |
| CTC2 | 3 | 15711999 | CA | C | 0.4 | NM_001195099 | ANKRD28 | frameshift_deleti  on | exon26 | c.2477delT | p.L826fs | . | . |
| CTC2 | 3 | 15731622 | TA | T | 0.321 | NM_001195099 | ANKRD28 | frameshift_deleti  on | exon16 | c.1314delT | p.F438fs | . | . |
| CTC2 | 3 | 17053508 | TA | T | 0.533 | NM_015184 | PLCL2 | frameshift_deleti  on | exon2 | c.2293delA | p.K765fs | . | . |

| CTC2 | 3 | 20193828 | GA | G | 0.679 | NM_003884 | KAT2B | frameshift_deleti  on | exon18 | c.2311delA | p.K771fs | . | . |
| --- | --- | --- | --- | --- | --- | --- | --- | --- | --- | --- | --- | --- | --- |
| CTC2 | 3 | 25671887 | T | TC | 1 | NM_001068 | TOP2B | frameshift_inserti  on | exon12 | c.1463dupG | p.G488fs | . | . |
| CTC2 | 3 | 28365592 | TG | T | 0.833 | NM_022461 | AZI2 | frameshift_deleti  on | exon8 | c.1119delC | p.P373fs | . | . |
| CTC2 | 3 | 28365779 | TG | T | 1 | NM_022461 | AZI2 | stopgain | exon8 | c.932delC | p.S311X | . | . |
| CTC2 | 3 | 28454863 | GT | G | 0.391 | NM_001040432 | ZCWPW2 | frameshift_deleti on | exon2 | c.305delT | p.V102fs | . | . |
| CTC2 | 3 | 30713129 | GA | G | 0.74 | NM_003242 | TGFBR2 | frameshift_deleti  on | exon4 | c.455delA | p.E152fs | . | . |
| CTC2 | 3 | 33195355 | TC | T | 0.478 | NM_015551 | SUSD5 | frameshift_deleti  on | exon5 | c.768delG | p.G256fs | . | . |
| CTC2 | 3 | 33738399 | TA | T | 0.667 | NM_015097 | CLASP2 | stopgain | exon2 | c.221delT | p.L74X | . | . |
| CTC2 | 3 | 35763149 | GA | G | 0.393 | NM_001267617 | ARPP21 | frameshift_deleti on | exon12 | c.887delA | p.E296fs | . | . |
| CTC2 | 3 | 36872831 | C | CA | 0.5 | NM_014831 | TRANK1 | frameshift_inserti  on | exon21 | c.8110_8111ins  T | p.R2704fs | . | . |
| CTC2 | 3 | 37369176 | CG | C | 0.389 | NM_002078 | GOLGA4 | frameshift_deleti  on | exon14 | c.5800delG | p.G1934fs | . | . |
| CTC2 | 3 | 39140280 | AG | A | 0.242 | NM_001278790 | GORASP1 | frameshift_deleti  on | exon5 | c.555delC | p.T185fs | . | . |
| CTC2 | 3 | 39151605 | TG | T | 0.259 | NM_001105513 | TTC21A | frameshift_deleti  on | exon3 | c.244delG | p.A82fs | . | . |
| CTC2 | 3 | 39230294 | AG | A | 0.581 | NM_001198621 | XIRP1 | frameshift_deleti  on | exon2 | c.642delC | p.P214fs | . | . |
| CTC2 | 3 | 40503520 | A | ACTGC  TG | 1 | NM_001034996 | RPL14 | nonframeshift_in  sertion | exon6 | c.445_446insCT  GCTG | p.T149delinsTA  A | . | . |
| CTC2 | 3 | 42835688 | GT | G | 0.333 | NM_001099668 | HIGD1A | frameshift_deleti  on | exon2 | c.99delA | p.K33fs | . | . |
| CTC2 | 3 | 44284344 | GCCA | G | 0.3 | NM_001145030 | TOPAZ1 | nonframeshift_de  letion | exon2 | c.347_349del | p.116_117del | . | . |
| CTC2 | 3 | 44839346 | CA | C | 0.815 | NM_020242 | KIF15 | frameshift_deleti  on | exon10 | c.997delA | p.K333fs | . | . |
| CTC2 | 3 | 46751073 | TAAG | T | 0.75 | NM_147196 | TMIE | nonframeshift_de  letion | exon4 | c.367_369del | p.123_123del | . | . |
| CTC2 | 3 | 47165838 | CT | C | 0.424 | NM_014159 | SETD2 | frameshift_deleti  on | exon3 | c.287delA | p.K96fs | . | . |
| CTC2 | 3 | 47543281 | TG | T | 0.903 | NM_001031703 | ELP6 | frameshift_deleti  on | exon5 | c.383delC | p.P128fs | . | . |
| CTC2 | 3 | 47887879 | TC | T | 0.457 | NM_138615 | DHX30 | frameshift_deleti  on | exon11 | c.1318delC | p.R440fs | . | . |
| CTC2 | 3 | 47898959 | GA | G | 0.3 | NM_001134364 | MAP4 | frameshift_deleti  on | exon16 | c.3109delT | p.S1037fs | . | . |
| CTC2 | 3 | 48265011 | C | CA | 0.413 | NM_004345 | CAMP | frameshift_inserti  on | exon1 | c.20dupA | p.Q7fs | . | . |
| CTC2 | 3 | 48453861 | TC | T | 1 | NM_001130082 | PLXNB1 | frameshift_deleti  on | exon26 | c.5022delG | p.L1674fs | . | . |
| CTC2 | 3 | 48613970 | TC | T | 0.278 | NM_000094 | COL7A1 | frameshift_deleti  on | exon68 | c.5720delG | p.G1907fs | . | . |
| CTC2 | 3 | 48667886 | GA | G | 0.488 | NM_001281733 | SLC26A6 | frameshift_deleti  on | exon9 | c.989delT | p.F330fs | . | . |
| CTC2 | 3 | 48667929 | GAA | G | 0.538 | NM_001281733 | SLC26A6 | frameshift_deleti  on | exon9 | c.945_946del | p.S315fs | . | . |
| CTC2 | 3 | 48669824 | AG | A | 0.377 | NM_001040454 | SLC26A6 | frameshift_deleti  on | exon4 | c.375delC | p.T125fs | . | . |
| CTC2 | 3 | 48682493 | GCC | G | 0.135 | NM_001407 | CELSR3 | frameshift_deleti  on | exon25 | c.7945_7946del | p.G2649fs | . | . |
| CTC2 | 3 | 48683075 | TC | T | 1 | NM_001407 | CELSR3 | frameshift_deleti  on | exon24 | c.7597delG | p.E2533fs | . | . |
| CTC2 | 3 | 48810523 | TA | T | 0.321 | NM_004157 | PRKAR2A | stopgain | exon6 | c.560delT | p.L187X | . | . |
| CTC2 | 3 | 49201543 | GAC | G | 0.135 | NM_022903 | CCDC71 | frameshift_deleti on | exon2 | c.97_98del | p.V33fs | . | . |
| CTC2 | 3 | 49278717 | T | TG | 0.833 | NM_001135197 | CCDC36 | frameshift_inserti  on | exon4 | c.290_291insG | p.I97fs | . | . |
| CTC2 | 3 | 49314067 | GCAA | G | 0.636 | NM_198562 | C3orf62 | nonframeshift_de  letion | exon1 | c.236_238del | p.79_80del | . | . |
| CTC2 | 3 | 49337988 | CA | C | 0.692 | NM_199443 | USP4 | frameshift_deleti  on | exon10 | c.1282delT | p.C428fs | . | . |
| CTC2 | 3 | 49680315 | TG | T | 0.518 | NM_003458 | BSN | frameshift_deleti  on | exon3 | c.1249delG | p.G417fs | . | . |
| CTC2 | 3 | 49690953 | TC | T | 0.376 | NM_003458 | BSN | frameshift_deleti  on | exon5 | c.3965delC | p.S1322fs | . | . |
| CTC2 | 3 | 50155887 | TGA | T | 0.345 | NM_005778 | RBM5 | frameshift_deleti  on | exon25 | c.2447_2448del | p.*816fs | . | . |
| CTC2 | 3 | 51624505 | AGAG | A | 0.147 | NM_015106 | RAD54L2 | nonframeshift_de  letion | exon2 | c.70_72del | p.24_24del | . | . |
| CTC2 | 3 | 51696999 | AC | A | 0.583 | NM_015106 | RAD54L2 | frameshift_deleti  on | exon22 | c.3968delC | p.T1323fs | . | . |
| CTC2 | 3 | 52326946 | A | AG | 0.204 | NM_145262 | GLYCTK | frameshift_inserti  on | exon5 | c.1377dupG | p.E459fs | . | . |
| CTC2 | 3 | 52384536 | AAGGTC  ATGGAT GACTAC | A | 0.318 | NM_015512 | DNAH1 | nonframeshift_de letion | exon16 | c.2660_2677del | p.887_893del | . | . |
| CTC2 | 3 | 52392652 | TA | T | 0.257 | NM_015512 | DNAH1 | frameshift_deleti  on | exon25 | c.4166delA | p.Y1389fs | . | . |
| CTC2 | 3 | 52414148 | TA | T | 0.279 | NM_015512 | DNAH1 | frameshift_deleti  on | exon48 | c.7606delA | p.K2536fs | . | . |
| CTC2 | 3 | 52432925 | AG | A | 0.263 | NM_015512 | DNAH1 | frameshift_deleti  on | exon76 | c.12150delG | p.K4050fs | . | . |
| CTC2 | 3 | 52643494 | CT | C | 0.258 | NM_018313 | PBRM1 | frameshift_deleti  on | exon17 | c.2401delA | p.S801fs | . | . |
| CTC2 | 3 | 52729510 | CA | C | 0.87 | NM_018446 | GLT8D1 | frameshift_deleti  on | exon8 | c.738delT | p.F246fs | . | . |
| CTC2 | 3 | 53531422 | GC | G | 0.667 | NM_000720 | CACNA1D | frameshift_deleti  on | exon2 | c.312delC | p.R104fs | . | . |
| CTC2 | 3 | 53778787 | TGAA | T | 0.958 | NM_001128839 | CACNA1D | nonframeshift_de  letion | exon23 | c.2940_2942del | p.980_981del | . | . |
| CTC2 | 3 | 56591278 | T | TGGGG  TAAGC | 0.732 | NM_001141947 | CCDC66 | stopgain | exon1 | c.8_9insGGGGT  AAGCA | p.L3_G4delinsL  GX | . | . |
| CTC2 | 3 | 56593606 | CA | C | 0.338 | NM_001141947 | CCDC66 | frameshift_deleti  on | exon3 | c.87delA | p.S29fs | . | . |
| CTC2 | 3 | 57405237 | GT | G | 0.312 | NM_178504 | DNAH12 | frameshift_deleti  on | exon37 | c.5513delA | p.N1838fs | . | . |
| CTC2 | 3 | 57542812 | AC | A | 0.281 | NM_177966 | PDE12 | frameshift_deleti  on | exon1 | c.707delC | p.T236fs | . | . |
| CTC2 | 3 | 57613953 | CG | C | 1 | NM_152678 | DENND6A | frameshift_deleti  on | exon20 | c.1814delC | p.T605fs | . | . |
| CTC2 | 3 | 57616505 | GT | G | 1 | NM_152678 | DENND6A | frameshift_deleti  on | exon17 | c.1453delA | p.T485fs | . | . |

| CTC2 | 3 | 58191250 | TA | T | 0.226 | NM_004944 | DNASE1L3 | frameshift_deleti  on | exon3 | c.267delT | p.I89fs | . | . |
| --- | --- | --- | --- | --- | --- | --- | --- | --- | --- | --- | --- | --- | --- |
| CTC2 | 3 | 58552413 | GT | G | 0.386 | NM_001076778 | FAM107A | frameshift_deleti  on | exon4 | c.336delA | p.K112fs | . | . |
| CTC2 | 3 | 63981999 | GAAAGA | G | 0.226 | NM_001128149 | ATXN7 | frameshift_deleti  on | exon8 | c.2067_2071del | p.G689fs | . | . |
| CTC2 | 3 | 65425560 | T | TCTG | 1 | NM_001033057 | MAGI1 | nonframeshift_in  sertion | exon9 | c.1263_1264ins  CAG | p.T422delinsQT | . | . |
| CTC2 | 3 | 69169101 | CT | C | 0.191 | NM_198271 | LMOD3 | frameshift_deleti  on | exon2 | c.404delA | p.K135fs | . | . |
| CTC2 | 3 | 69434980 | TG | T | 0.3 | NM_015123 | FRMD4B | frameshift_deleti  on | exon1 | c.160delC | p.Q54fs | . | . |
| CTC2 | 3 | 72428420 | T | TA | 0.344 | NM_012234 | RYBP | frameshift_inserti  on | exon2 | c.286_287insT | p.N96fs | . | . |
| CTC2 | 3 | 77623661 | AC | A | 0.353 | NM_002942 | ROBO2 | frameshift_deleti  on | exon14 | c.1984delC | p.P662fs | . | . |
| CTC2 | 3 | 78987997 | CA | C | 0.184 | NM_001145845 | ROBO1 | frameshift_deleti  on | exon2 | c.135delT | p.P45fs | . | . |
| CTC2 | 3 | 89528560 | GT | G | 0.548 | NM_005233 | EPHA3 | frameshift_deleti  on | exon17 | c.2861delT | p.V954fs | . | . |
| CTC2 | 3 | 97194251 | TG | T | 0.455 | NM_001278300 | EPHA6 | frameshift_deleti  on | exon5 | c.127delG | p.G43fs | . | . |
| CTC2 | 3 | 97673298 | TTG | T | 0.333 | NM_001042533 | MINA | frameshift_deleti  on | exon5 | c.721_722del | p.Q241fs | . | . |
| CTC2 | 3 | 100074038 | GA | G | 0.205 | NM_020202 | NIT2 | frameshift_deleti  on | exon10 | c.758delA | p.E253fs | . | . |
| CTC2 | 3 | 100092444 | CA | C | 0.348 | NM_014820 | TOMM70A | frameshift_deleti  on | exon8 | c.1272delT | p.F424fs | . | . |
| CTC2 | 3 | 105264138 | AC | A | 0.462 | NM_001243280 | ALCAM | frameshift_deleti  on | exon9 | c.1064delC | p.T355fs | . | . |
| CTC2 | 3 | 107097188 | AC | A | 0.5 | NM_032600 | CCDC54 | frameshift_deleti  on | exon1 | c.755delC | p.T252fs | . | . |
| CTC2 | 3 | 108103565 | AC | A | 0.583 | NM_014981 | MYH15 | frameshift_deleti  on | exon40 | c.5659delG | p.V1887fs | . | . |
| CTC2 | 3 | 108276156 | TA | T | 0.538 | NM_020890 | KIAA1524 | frameshift_deleti  on | exon17 | c.2118delT | p.D706fs | . | . |
| CTC2 | 3 | 108672608 | A | AC | 0.75 | NM_005459 | GUCA1C | frameshift_inserti  on | exon1 | c.1_2insG | p.M1fs | . | . |
| CTC2 | 3 | 109052839 | C | CT | 0.188 | NM_018189 | DPPA4 | stopgain | exon2 | c.55_56insA | p.W19_T20deli  nsX | . | . |
| CTC2 | 3 | 110863767 | CT | C | 0.6 | NM_001243288 | PVRL3 | frameshift_deleti  on | exon6 | c.1023delT | p.S341fs | . | . |
| CTC2 | 3 | 111697942 | TG | T | 0.349 | NM_001272069 | ABHD10 | frameshift_deleti  on | exon1 | c.35delG | p.W12fs | . | . |
| CTC2 | 3 | 111845872 | AT | A | 0.818 | NM_001190260 | GCSAM | frameshift_deleti  on | exon3 | c.106delA | p.I36fs | . | . |
| CTC2 | 3 | 112300038 | AT | A | 0.7 | NM_017945 | SLC35A5 | frameshift_deleti  on | exon6 | c.1075delT | p.F359fs | . | . |
| CTC2 | 3 | 113172591 | CA | C | 0.556 | NM_144718 | SPICE1 | frameshift_deleti  on | exon14 | c.1863delT | p.F621fs | . | . |
| CTC2 | 3 | 113761624 | TCA | T | 0.125 | NM_020817 | KIAA1407 | frameshift_deleti  on | exon4 | c.339_340del | p.G113fs | . | . |
| CTC2 | 3 | 114070374 | GT | G | 0.327 | NM_001164342 | ZBTB20 | frameshift_deleti  on | exon4 | c.550delA | p.T184fs | . | . |
| CTC2 | 3 | 115805261 | T | TA | 0.625 | NM_002338 | LSAMP | stopgain | exon2 | c.297_298insT | p.K100_V101de  linsX | . | . |
| CTC2 | 3 | 119134727 | TG | T | 0.478 | NM_020754 | ARHGAP31 | frameshift_deleti  on | exon12 | c.3952delG | p.G1318fs | . | . |
| CTC2 | 3 | 120499942 | TG | T | 0.205 | NM_005513 | GTF2E1 | frameshift_deleti  on | exon5 | c.946delG | p.D316fs | . | . |
| CTC2 | 3 | 121208253 | AT | A | 0.308 | NM_199420 | POLQ | frameshift_deleti  on | exon16 | c.3524delA | p.N1175fs | . | . |
| CTC2 | 3 | 121208945 | TA | T | 0.263 | NM_199420 | POLQ | frameshift_deleti  on | exon16 | c.2832delT | p.F944fs | . | . |
| CTC2 | 3 | 121238885 | TG | T | 0.703 | NM_199420 | POLQ | frameshift_deleti  on | exon9 | c.1300delC | p.Q434fs | . | . |
| CTC2 | 3 | 121351293 | T | TCTCA  GGCTC | 0.803 | NM_005335 | HCLS1 | nonframeshift_in  sertion | exon12 | c.1125_1126ins  CCCGAGCCTG | p.N376delinsPE  PEN | . | . |
| CTC2 | 3 | 121351315 | G | GGGCT  CAGGC | 0.985 | NM_005335 | HCLS1 | nonframeshift_in  sertion | exon12 | c.1103_1104ins  TGAGCCTGAG | p.P368delinsPE  PEP | . | . |
| CTC2 | 3 | 121351322 | G | GGCTC  AGGCT | 0.917 | NM_005335 | HCLS1 | nonframeshift_in  sertion | exon12 | c.1096_1097ins  GAGAGCCTGA | p.P366delinsRE  PEP | . | . |
| CTC2 | 3 | 121351328 | G | GGCTC  AGGCT | 1 | NM_005335 | HCLS1 | nonframeshift_in  sertion | exon12 | c.1090_1091ins  TTGAGCCTGA | p.P364delinsLE  PEP | . | . |
| CTC2 | 3 | 121500676 | CA | C | 1 | NM_001023571 | IQCB1 | frameshift_deleti  on | exon10 | c.924delT | p.F308fs | . | . |
| CTC2 | 3 | 121650548 | AC | A | 0.6 | NM_001145998 | SLC15A2 | frameshift_deleti  on | exon18 | c.1635delC | p.D545fs | . | . |
| CTC2 | 3 | 121994727 | TG | T | 0.429 | NM_000388 | CASR | frameshift_deleti  on | exon5 | c.1447delG | p.G483fs | . | . |
| CTC2 | 3 | 122002637 | TG | T | 0.472 | NM_000388 | CASR | frameshift_deleti  on | exon7 | c.1837delG | p.G613fs | . | . |
| CTC2 | 3 | 122133940 | CA | C | 0.698 | NM_019069 | WDR5B | frameshift_deleti  on | exon1 | c.435delT | p.F145fs | . | . |
| CTC2 | 3 | 122411352 | AT | A | 0.857 | NM_017554 | PARP14 | frameshift_deleti  on | exon4 | c.561delT | p.D187fs | . | . |
| CTC2 | 3 | 122419195 | TA | T | 0.926 | NM_017554 | PARP14 | frameshift_deleti  on | exon6 | c.1795delA | p.K599fs | . | . |
| CTC2 | 3 | 123333029 | CT | C | 0.375 | NM_053031 | MYLK | frameshift_deleti  on | exon3 | c.384delA | p.G128fs | . | . |
| CTC2 | 3 | 123419116 | GTTC | G | 1 | NM_053026 | MYLK | nonframeshift_de  letion | exon17 | c.2989_2991del | p.997_997del | . | . |
| CTC2 | 3 | 123667836 | CG | C | 0.376 | NM_022757 | CCDC14 | frameshift_deleti  on | exon5 | c.516delC | p.H172fs | . | . |
| CTC2 | 3 | 123680026 | GC | G | 0.471 | NM_022757 | CCDC14 | frameshift_deleti  on | exon1 | c.138delG | p.R46fs | . | . |
| CTC2 | 3 | 124646705 | A | AAAG | 1 | NM_033049 | MUC13 | nonframeshift_in  sertion | exon2 | c.184_185insCT  T | p.F62delinsSF | . | . |
| CTC2 | 3 | 125216948 | TA | T | 0.278 | NM_003794 | SNX4 | stopgain | exon3 | c.353delT | p.L118X | . | . |
| CTC2 | 3 | 126160747 | AT | A | 0.474 | NM_025112 | ZXDC | stopgain | exon8 | c.2254delA | p.M752X | . | . |
| CTC2 | 3 | 126216902 | GC | G | 1 | NM_144639 | UROC1 | frameshift_deleti on | exon14 | c.1429delG | p.A477fs | . | . |
| CTC2 | 3 | 126268814 | CG | C | 0.261 | NM_152533 | C3orf22 | frameshift_deleti  on | exon4 | c.322delC | p.R108fs | . | . |
| CTC2 | 3 | 126736464 | CT | C | 0.52 | NM_032242 | PLXNA1 | frameshift_deleti  on | exon17 | c.3474delT | p.T1158fs | . | . |
| CTC2 | 3 | 128060348 | TG | T | 0.15 | NM_021937 | EEFSEC | frameshift_deleti  on | exon5 | c.1060delG | p.A354fs | . | . |
| CTC2 | 3 | 128292318 | ATC | A | 0.266 | NM_007354 | C3orf27 | frameshift_deleti  on | exon3 | c.253_254del | p.D85fs | . | . |
| CTC2 | 3 | 128696986 | TG | T | 0.647 | NM_020741 | KIAA1257 | frameshift_deleti  on | exon5 | c.709delC | p.Q237fs | . | . |

| CTC2 | 3 | 129168754 | AT | A | 0.392 | NM_001280541 | IFT122 | frameshift_deleti  on | exon2 | c.83delT | p.I28fs | . | . |
| --- | --- | --- | --- | --- | --- | --- | --- | --- | --- | --- | --- | --- | --- |
| CTC2 | 3 | 129185793 | CAG | C | 0.375 | NM_052989 | IFT122 | frameshift_deleti  on | exon8 | c.625_626del | p.R209fs | . | . |
| CTC2 | 3 | 129238571 | TC | T | 0.916 | NM_052990 | IFT122 | frameshift_deleti  on | exon26 | c.3303delC | p.F1101fs | . | . |
| CTC2 | 3 | 129389620 | GA | G | 0.333 | NM_001017395 | TMCC1 | frameshift_deleti  on | exon4 | c.1063delT | p.S355fs | . | . |
| CTC2 | 3 | 129546679 | TGCA | T | 0.343 | NM_001017395 | TMCC1 | nonframeshift_de  letion | exon3 | c.540_542del | p.180_181del | . | . |
| CTC2 | 3 | 129695976 | CT | C | 0.276 | NM_007117 | TRH | frameshift_deleti  on | exon3 | c.647delT | p.L216fs | . | . |
| CTC2 | 3 | 130424589 | AT | A | 0.957 | NM_014602 | PIK3R4 | frameshift_deleti  on | exon12 | c.2747delA | p.N916fs | . | . |
| CTC2 | 3 | 130873913 | G | GA | 0.257 | NM_001146003 | NEK11 | frameshift_inserti  on | exon9 | c.911dupA | p.E304fs | . | . |
| CTC2 | 3 | 131181666 | G | GA | 0.241 | NM_007208 | MRPL3 | frameshift_inserti  on | exon10 | c.947dupT | p.F316fs | . | . |
| CTC2 | 3 | 132407709 | TA | T | 0.227 | NM_153240 | NPHP3 | stopgain | exon21 | c.2909delT | p.L970X | . | . |
| CTC2 | 3 | 133098738 | TTC | T | 0.375 | NM_001136469 | TMEM108 | frameshift_deleti on | exon4 | c.184_185del | p.S62fs | . | . |
| CTC2 | 3 | 133356724 | ACAT | A | 0.553 | NM_007027 | TOPBP1 | nonframeshift_de  letion | exon14 | c.2513_2515del | p.838_839del | . | . |
| CTC2 | 3 | 133672553 | GC | G | 0.429 | NM_005630 | SLCO2A1 | frameshift_deleti  on | exon5 | c.677delG | p.G226fs | . | . |
| CTC2 | 3 | 134078263 | TG | T | 0.269 | NM_001278683 | AMOTL2 | frameshift_deleti  on | exon8 | c.2139delC | p.G713fs | . | . |
| CTC2 | 3 | 134277050 | GA | G | 0.392 | NM_025180 | CEP63 | frameshift_deleti  on | exon14 | c.1535delA | p.E512fs | . | . |
| CTC2 | 3 | 135720501 | ACC | A | 0.134 | NM_002718 | PPP2R3A | frameshift_deleti  on | exon2 | c.162_163del | p.D54fs | . | . |
| CTC2 | 3 | 136649428 | AT | A | 0.8 | NM_001190796 | NCK1 | frameshift_deleti  on | exon1 | c.27delT | p.D9fs | . | . |
| CTC2 | 3 | 138188928 | GT | G | 0.579 | NM_031913 | ESYT3 | frameshift_deleti  on | exon16 | c.1531delT | p.W511fs | . | . |
| CTC2 | 3 | 139237359 | TG | T | 1 | NM_002899 | RBP1 | frameshift_deleti  on | exon3 | c.443delC | p.T148fs | . | . |
| CTC2 | 3 | 141231020 | TA | T | 0.408 | NM_006506 | RASA2 | frameshift_deleti  on | exon2 | c.150delA | p.L50fs | . | . |
| CTC2 | 3 | 142389904 | A | AT | 0.519 | NM_001145319 | PLS1 | frameshift_inserti  on | exon4 | c.305dupT | p.I102fs | . | . |
| CTC2 | 3 | 142524889 | CAA | C | 1 | NM_003304 | TRPC1 | frameshift_deleti  on | exon12 | c.2093_2094del | p.Q698fs | . | . |
| CTC2 | 3 | 142542400 | TC | T | 0.667 | NM_013363 | PCOLCE2 | frameshift_deleti  on | exon7 | c.922delG | p.E308fs | . | . |
| CTC2 | 3 | 143550916 | CT | C | 0.448 | NM_173653 | SLC9A9 | frameshift_deleti  on | exon2 | c.322delA | p.R108fs | . | . |
| CTC2 | 3 | 145917783 | GT | G | 0.471 | NM_001128305 | PLSCR4 | frameshift_deleti  on | exon6 | c.440delA | p.N147fs | . | . |
| CTC2 | 3 | 147123247 | TG | T | 0.969 | NM_001168379 | ZIC4 | frameshift_deleti  on | exon1 | c.11delC | p.P4fs | . | . |
| CTC2 | 3 | 148562534 | AT | A | 0.261 | NM_001871 | CPB1 | frameshift_deleti  on | exon8 | c.759delT | p.N253fs | . | . |
| CTC2 | 3 | 148894087 | T | TC | 0.333 | NM_000096 | CP | frameshift_inserti  on | exon18 | c.3130dupG | p.D1044fs | . | . |
| CTC2 | 3 | 148894124 | TTC | T | 0.78 | NM_000096 | CP | frameshift_deleti  on | exon18 | c.3092_3093del | p.R1031fs | . | . |
| CTC2 | 3 | 148903070 | TGG | T | 0.526 | NM_000096 | CP | frameshift_deleti  on | exon12 | c.2239_2240del | p.P747fs | . | . |
| CTC2 | 3 | 148905971 | A | AT | 0.379 | NM_000096 | CP | frameshift_inserti  on | exon10 | c.1731dupA | p.F578fs | . | . |
| CTC2 | 3 | 149677866 | AT | A | 0.286 | NM_183381 | RNF13 | frameshift_deleti  on | exon9 | c.725delT | p.I242fs | . | . |
| CTC2 | 3 | 150403761 | CAT | C | 0.375 | NM_152394 | FAM194A | frameshift_deleti  on | exon5 | c.656_657del | p.Y219fs | . | . |
| CTC2 | 3 | 150421548 | TTCC | T | 0.127 | NM_152394 | FAM194A | nonframeshift_de  letion | exon1 | c.135_137del | p.45_46del | . | . |
| CTC2 | 3 | 150877785 | G | GCC | 0.96 | NM_053002 | MED12L | frameshift_inserti  on | exon7 | c.1004_1005ins  CC | p.G335fs | . | . |
| CTC2 | 3 | 150877786 | C | CCA | 0.659 | NM_053002 | MED12L | frameshift_inserti  on | exon7 | c.1005_1006ins  CA | p.G335fs | . | . |
| CTC2 | 3 | 150877790 | C | CCT | 0.61 | NM_053002 | MED12L | frameshift_inserti  on | exon7 | c.1009_1010ins  CT | p.P337fs | . | . |
| CTC2 | 3 | 150877792 | C | CCT | 0.73 | NM_053002 | MED12L | frameshift_inserti  on | exon7 | c.1011_1012ins  CT | p.P337fs | . | . |
| CTC2 | 3 | 151045929 | CA | C | 0.321 | NM_176894 | P2RY13 | frameshift_deleti  on | exon2 | c.914delT | p.L305fs | . | . |
| CTC2 | 3 | 151148113 | CCAG | C | 0.152 | NM_053002 | MED12L | nonframeshift_de  letion | exon42 | c.6331_6333del | p.2111_2111de  l | . | . |
| CTC2 | 3 | 151174927 | TA | T | 0.373 | NM_178822 | IGSF10 | frameshift_deleti  on | exon2 | c.210delT | p.V70fs | . | . |
| CTC2 | 3 | 151545885 | AT | A | 0.545 | NM_001086 | AADAC | frameshift_deleti  on | exon5 | c.1126delT | p.F376fs | . | . |
| CTC2 | 3 | 151598364 | CA | C | 0.625 | NM_033050 | SUCNR1 | frameshift_deleti  on | exon3 | c.34delA | p.K12fs | . | . |
| CTC2 | 3 | 155199758 | CCA | C | 0.14 | NM_014996 | PLCH1 | frameshift_deleti  on | exon22 | c.3965_3966del | p.V1322fs | . | . |
| CTC2 | 3 | 155200452 | AG | A | 0.211 | NM_014996 | PLCH1 | frameshift_deleti  on | exon22 | c.3272delC | p.S1091fs | . | . |
| CTC2 | 3 | 156867285 | GC | G | 0.429 | NM_020307 | CCNL1 | frameshift_deleti  on | exon9 | c.1121delG | p.S374fs | . | . |
| CTC2 | 3 | 156867286 | CT | C | 1 | NM_020307 | CCNL1 | frameshift_deleti  on | exon9 | c.1120delA | p.S374fs | . | . |
| CTC2 | 3 | 161221295 | CA | C | 0.185 | NM_001080440 | OTOL1 | frameshift_deleti  on | exon4 | c.1000delA | p.K334fs | . | . |
| CTC2 | 3 | 168833952 | AG | A | 0.185 | NM_001105078 | MECOM | frameshift_deleti  on | exon7 | c.1143delC | p.P381fs | . | . |
| CTC2 | 3 | 169999037 | TC | T | 1 | NM_002740 | PRKCI | frameshift_deleti  on | exon10 | c.967delC | p.Q323fs | . | . |
| CTC2 | 3 | 170013707 | GA | G | 1 | NM_002740 | PRKCI | frameshift_deleti  on | exon15 | c.1427delA | p.E476fs | . | . |
| CTC2 | 3 | 170723848 | ATG | A | 0.262 | NM_001278658 | SLC2A2 | frameshift_deleti  on | exon5 | c.300_301del | p.H100fs | . | . |
| CTC2 | 3 | 170727794 | G | GA | 0.273 | NM_001278658 | SLC2A2 | frameshift_inserti  on | exon3 | c.91dupT | p.S31fs | . | . |
| CTC2 | 3 | 172229441 | CA | C | 0.287 | NM_003810 | TNFSF10 | stopgain | exon3 | c.278delT | p.L93X | . | . |
| CTC2 | 3 | 176769364 | TGGCTG CAGCTG | T | 0.161 | NM_024665 | TBL1XR1 | nonframeshift_de letion | exon5 | c.343_354del | p.115_118del | . | . |
| CTC2 | 3 | 178941880 | GT | G | 0.351 | NM_006218 | PIK3CA | frameshift_deleti  on | exon15 | c.2200delT | p.F734fs | . | . |
| CTC2 | 3 | 180322680 | GA | G | 0.75 | NM_001042601 | TTC14 | frameshift_deleti  on | exon6 | c.743delA | p.E248fs | . | . |

| CTC2 | 3 | 182871699 | TG | T | 0.778 | NM_014398 | LAMP3 | frameshift_deleti  on | exon2 | c.529delC | p.H177fs | . | . |
| --- | --- | --- | --- | --- | --- | --- | --- | --- | --- | --- | --- | --- | --- |
| CTC2 | 3 | 183887845 | AC | A | 0.529 | NM_004423 | DVL3 | frameshift_deleti  on | exon14 | c.1551delC | p.D517fs | . | . |
| CTC2 | 3 | 183907532 | GC | G | 0.22 | NM_018358 | ABCF3 | frameshift_deleti  on | exon13 | c.1302delC | p.R434fs | . | . |
| CTC2 | 3 | 184073304 | TC | T | 0.222 | NM_001171088 | CLCN2 | frameshift_deleti  on | exon11 | c.1051delG | p.E351fs | . | . |
| CTC2 | 3 | 186522373 | AT | A | 0.429 | NM_002916 | RFC4 | frameshift_deleti  on | exon2 | c.129delA | p.K43fs | . | . |
| CTC2 | 3 | 190026086 | TG | T | 0.229 | NM_021101 | CLDN1 | frameshift_deleti  on | exon4 | c.615delC | p.S205fs | . | . |
| CTC2 | 3 | 190106172 | GT | G | 0.727 | NM_006580 | CLDN16 | frameshift_deleti  on | exon1 | c.265delT | p.F89fs | . | . |
| CTC2 | 3 | 193082055 | GT | G | 0.5 | NM_198505 | ATP13A5 | frameshift_deleti  on | exon2 | c.77delA | p.Y26fs | . | . |
| CTC2 | 3 | 195298194 | TG | T | 0.237 | NM_001647 | APOD | frameshift_deleti  on | exon4 | c.287delC | p.P96fs | . | . |
| CTC2 | 3 | 195512684 | CA | C | 0.702 | NM_018406 | MUC4 | frameshift_deleti  on | exon2 | c.5766delT | p.P1922fs | . | . |
| CTC2 | 3 | 195516859 | CT | C | 0.333 | NM_018406 | MUC4 | frameshift_deleti  on | exon2 | c.1591delA | p.R531fs | . | . |
| CTC2 | 3 | 195518112 | T | TGTCTC  CTGCG | 1 | NM_018406 | MUC4 | nonframeshift_in  sertion | exon2 | c.338_339insTG  TTACGCAGGA | p.T113delinsTV  TQET | . | . |
| CTC2 | 3 | 195518359 | TC | T | 0.317 | NM_018406 | MUC4 | frameshift_deleti  on | exon2 | c.91delG | p.E31fs | . | . |
| CTC2 | 3 | 196454978 | AC | A | 0.625 | NM_001166304 | PIGX | frameshift_deleti  on | exon4 | c.504delC | p.N168fs | . | . |
| CTC2 | 3 | 196613137 | CT | C | 0.468 | NM_152699 | SENP5 | frameshift_deleti  on | exon2 | c.1086delT | p.S362fs | . | . |
| CTC2 | 3 | 197409452 | GGT | G | 0.583 | NM_014687 | KIAA0226 | frameshift_deleti  on | exon14 | c.2013_2014del | p.S671fs | . | . |
| CTC2 | 4 | 367052 | AT | A | 0.9 | NM_003441 | ZNF141 | frameshift_deleti  on | exon4 | c.827delT | p.I276fs | . | . |
| CTC2 | 4 | 437111 | AT | A | 0.312 | NM_133474 | ZNF721 | frameshift_deleti  on | exon3 | c.1144delA | p.I382fs | . | . |
| CTC2 | 4 | 679672 | A | AT | 0.4 | NM_032219 | MFSD7 | frameshift_inserti  on | exon4 | c.536dupA | p.N179fs | . | . |
| CTC2 | 4 | 1087327 | G | GCTGC  CCAGG CTGGA | 1 | NM_001193318 | RNF212 | nonframeshift_in sertion | exon4 | c.721_722insG  GCTGGCTCCA GCCTGGGCAG | p.S241delinsW LAPAWAA | . | . |
| CTC2 | 4 | 1379716 | GT | G | 0.333 | NM_020894 | UVSSA | frameshift_deleti  on | exon14 | c.2098delT | p.F700fs | . | . |
| CTC2 | 4 | 2641578 | AC | A | 0.571 | NM_001256666 | FAM193A | frameshift_deleti  on | exon4 | c.283delC | p.Q95fs | . | . |
| CTC2 | 4 | 2661685 | TG | T | 1 | NM_001256666 | FAM193A | frameshift_deleti  on | exon8 | c.777delG | p.V259fs | . | . |
| CTC2 | 4 | 2829034 | CG | C | 0.815 | NM_001122681 | SH3BP2 | frameshift_deleti  on | exon6 | c.507delG | p.T169fs | . | . |
| CTC2 | 4 | 2877768 | AC | A | 0.312 | NM_001119 | ADD1 | frameshift_deleti  on | exon2 | c.127delC | p.P43fs | . | . |
| CTC2 | 4 | 3021394 | CA | C | 0.571 | NM_001004056 | GRK4 | frameshift_deleti  on | exon8 | c.673delA | p.K225fs | . | . |
| CTC2 | 4 | 3107090 | GC | G | 0.312 | NM_002111 | HTT | frameshift_deleti  on | exon5 | c.536delC | p.A179fs | . | . |
| CTC2 | 4 | 3176717 | A | AT | 0.526 | NM_002111 | HTT | frameshift_inserti  on | exon33 | c.4290_4291ins  T | p.I1430fs | . | . |
| CTC2 | 4 | 3319731 | AT | A | 0.459 | NM_002926 | RGS12 | frameshift_deleti  on | exon2 | c.1835delT | p.I612fs | . | . |
| CTC2 | 4 | 3319747 | G | GA | 0.327 | NM_002926 | RGS12 | frameshift_inserti  on | exon2 | c.1851dupA | p.R617fs | . | . |
| CTC2 | 4 | 4422621 | AT | A | 0.714 | NM_016930 | STX18 | frameshift_deleti  on | exon10 | c.881delA | p.N294fs | . | . |
| CTC2 | 4 | 5733340 | CT | C | 0.538 | NM_153717 | EVC | frameshift_deleti  on | exon4 | c.574delT | p.F192fs | . | . |
| CTC2 | 4 | 6863790 | CA | C | 0.75 | NM_001100590 | KIAA0232 | frameshift_deleti  on | exon6 | c.1682delA | p.Q561fs | . | . |
| CTC2 | 4 | 8089991 | TC | T | 0.455 | NM_001130083 | ABLIM2 | frameshift_deleti  on | exon4 | c.358delG | p.D120fs | . | . |
| CTC2 | 4 | 8218717 | TGA | T | 0.52 | NM_018986 | SH3TC1 | frameshift_deleti  on | exon7 | c.663_664del | p.V221fs | . | . |
| CTC2 | 4 | 11401092 | GCA | G | 0.467 | NM_005114 | HS3ST1 | frameshift_deleti  on | exon2 | c.536_537del | p.V179fs | . | . |
| CTC2 | 4 | 15556720 | TC | T | 1 | NM_001080522 | CC2D2A | frameshift_deleti  on | exon21 | c.2513delC | p.S838fs | . | . |
| CTC2 | 4 | 15689732 | AC | A | 0.667 | NM_001145191 | FAM200B | frameshift_deleti  on | exon2 | c.1133delC | p.T378fs | . | . |
| CTC2 | 4 | 15964507 | C | CT | 0.983 | NM_031950 | FGFBP2 | frameshift_inserti  on | exon1 | c.245dupA | p.Q82fs | . | . |
| CTC2 | 4 | 15981077 | AT | A | 0.318 | NM_001145847 | PROM1 | frameshift_deleti  on | exon25 | c.2495delA | p.N832fs | . | . |
| CTC2 | 4 | 17814046 | AG | A | 0.333 | NM_022346 | NCAPG | frameshift_deleti  on | exon2 | c.315delG | p.K105fs | . | . |
| CTC2 | 4 | 17885792 | GT | G | 0.556 | NM_001166139 | LCORL | frameshift_deleti  on | exon7 | c.1359delA | p.K453fs | . | . |
| CTC2 | 4 | 17885977 | GT | G | 0.64 | NM_001166139 | LCORL | frameshift_deleti  on | exon7 | c.1174delA | p.T392fs | . | . |
| CTC2 | 4 | 22437081 | G | GA | 0.941 | NM_145290 | GPR125 | frameshift_inserti  on | exon10 | c.1295dupT | p.L432fs | . | . |
| CTC2 | 4 | 25783918 | A | AT | 0.245 | NM_015187 | SEL1L3 | frameshift_inserti  on | exon15 | c.2402dupA | p.N801fs | . | . |
| CTC2 | 4 | 25819799 | TG | T | 0.5 | NM_015187 | SEL1L3 | frameshift_deleti  on | exon9 | c.1524delC | p.P508fs | . | . |
| CTC2 | 4 | 37846018 | GC | G | 0.909 | NM_018290 | PGM2 | frameshift_deleti  on | exon7 | c.754delC | p.H252fs | . | . |
| CTC2 | 4 | 37962189 | GT | G | 1 | NM_006607 | PTTG2 | frameshift_deleti  on | exon1 | c.135delT | p.R45fs | . | . |
| CTC2 | 4 | 38799297 | CT | C | 0.412 | NM_003263 | TLR1 | frameshift_deleti  on | exon4 | c.1155delA | p.K385fs | . | . |
| CTC2 | 4 | 39343973 | G | GA | 1 | NM_001204747 | RFC1 | frameshift_inserti  on | exon4 | c.322dupT | p.S108fs | . | . |
| CTC2 | 4 | 39843646 | TC | T | 0.194 | NM_001100399 | PDS5A | frameshift_deleti  on | exon31 | c.3578delG | p.G1193fs | . | . |
| CTC2 | 4 | 39929676 | TG | T | 0.4 | NM_001100399 | PDS5A | frameshift_deleti  on | exon3 | c.246delC | p.P82fs | . | . |
| CTC2 | 4 | 40121854 | TA | T | 0.508 | NM_018177 | N4BP2 | frameshift_deleti  on | exon9 | c.2124delA | p.V708fs | . | . |
| CTC2 | 4 | 40122489 | G | GA | 0.814 | NM_018177 | N4BP2 | frameshift_inserti  on | exon9 | c.2759dupA | p.E920fs | . | . |
| CTC2 | 4 | 41648507 | G | GGA | 0.36 | NM_001112720 | LIMCH1 | frameshift_inserti  on | exon6 | c.764_765insG  A | p.R255fs | . | . |
| CTC2 | 4 | 42145752 | GA | G | 0.64 | NM_001159547 | BEND4 | frameshift_deleti  on | exon3 | c.746delT | p.F249fs | . | . |
| CTC2 | 4 | 47514618 | AG | A | 0.296 | NM_020453 | ATP10D | frameshift_deleti  on | exon2 | c.62delG | p.R21fs | . | . |

| CTC2 | 4 | 47680007 | AC | A | 0.333 | NM_001278585 | CORIN | frameshift_deleti  on | exon7 | c.884delG | p.C295fs | . | . |
| --- | --- | --- | --- | --- | --- | --- | --- | --- | --- | --- | --- | --- | --- |
| CTC2 | 4 | 48116377 | GAACAG  C | G | 0.551 | NM_003328 | TXK | nonframeshift_de  letion | exon2 | c.53_58del | p.18_20del | . | . |
| CTC2 | 4 | 52946052 | A | AT | 0.211 | NM_145263 | SPATA18 | frameshift_inserti  on | exon9 | c.1323dupT | p.Y441fs | . | . |
| CTC2 | 4 | 53610774 | AC | A | 0.367 | NM_001242690 | ERVMER34-  1 | frameshift_deleti  on | exon3 | c.913delG | p.V305fs | . | . |
| CTC2 | 4 | 53611268 | T | TA | 0.944 | NM_001242690 | ERVMER34-  1 | frameshift_inserti  on | exon3 | c.419dupT | p.I140fs | . | . |
| CTC2 | 4 | 55984902 | AC | A | 1 | NM_002253 | KDR | stopgain | exon3 | c.226delG | p.V76X | . | . |
| CTC2 | 4 | 57272707 | AC | A | 0.175 | NM_002703 | PPAT | frameshift_deleti on | exon3 | c.355delG | p.V119fs | . | . |
| CTC2 | 4 | 57301536 | TC | T | 0.349 | NM_002703 | PPAT | frameshift_deleti  on | exon1 | c.107delG | p.G36fs | . | . |
| CTC2 | 4 | 57356742 | CA | C | 0.308 | NM_001267722 | SRP72 | frameshift_deleti  on | exon13 | c.1251delA | p.P417fs | . | . |
| CTC2 | 4 | 68358626 | A | AT | 0.833 | NM_001812 | CENPC | frameshift_inserti  on | exon15 | c.2379dupA | p.S794fs | . | . |
| CTC2 | 4 | 68610313 | TC | T | 0.577 | NM_000406 | GNRHR | frameshift_deleti  on | exon2 | c.714delG | p.L238fs | . | . |
| CTC2 | 4 | 69512868 | C | CA | 0.318 | NM_001076 | UGT2B15 | frameshift_inserti  on | exon6 | c.1546dupT | p.C516fs | . | . |
| CTC2 | 4 | 69798388 | CT | C | 0.31 | NM_024743 | UGT2A3 | frameshift_deleti  on | exon3 | c.953delA | p.K318fs | . | . |
| CTC2 | 4 | 69962425 | CT | C | 0.339 | NM_001074 | UGT2B7 | frameshift_deleti  on | exon1 | c.188delT | p.L63fs | . | . |
| CTC2 | 4 | 70160467 | GT | G | 0.826 | NM_053039 | UGT2B28 | frameshift_deleti  on | exon6 | c.1531delT | p.F511fs | . | . |
| CTC2 | 4 | 70460201 | T | TC | 0.259 | NM_001105677 | UGT2A2 | frameshift_inserti  on | exon5 | c.1324dupG | p.E442fs | . | . |
| CTC2 | 4 | 70898884 | GA | G | 0.367 | NM_000200 | HTN3 | frameshift_deleti  on | exon5 | c.104delA | p.E35fs | . | . |
| CTC2 | 4 | 71630238 | CA | C | 0.69 | NM_001037442 | RUFY3 | frameshift_deleti  on | exon4 | c.518delA | p.Q173fs | . | . |
| CTC2 | 4 | 72994376 | AG | A | 0.5 | NM_004885 | NPFFR2 | frameshift_deleti  on | exon2 | c.375delG | p.K125fs | . | . |
| CTC2 | 4 | 73990935 | TC | T | 0.981 | NM_198889 | ANKRD17 | frameshift_deleti  on | exon16 | c.2575delG | p.E859fs | . | . |
| CTC2 | 4 | 74309134 | AT | A | 0.229 | NM_001134 | AFP | frameshift_deleti  on | exon6 | c.687delT | p.N229fs | . | . |
| CTC2 | 4 | 76902609 | G | GA | 0.364 | NM_001288983 | SDAD1 | frameshift_inserti  on | exon3 | c.209_210insT | p.Y70fs | . | . |
| CTC2 | 4 | 77065318 | C | CTGT | 1 | NM_017426 | NUP54 | nonframeshift_in  sertion | exon3 | c.278_279insAC  A | p.Q93delinsQQ | . | . |
| CTC2 | 4 | 77082879 | AG | A | 0.306 | NM_001204255 | SCARB2 | frameshift_deleti  on | exon9 | c.994delC | p.L332fs | . | . |
| CTC2 | 4 | 77116873 | GC | G | 0.462 | NM_001204255 | SCARB2 | frameshift_deleti  on | exon2 | c.261delG | p.G87fs | . | . |
| CTC2 | 4 | 77357321 | GT | G | 0.367 | NM_020859 | SHROOM3 | frameshift_deleti  on | exon1 | c.117delT | p.G39fs | . | . |
| CTC2 | 4 | 79293913 | AC | A | 0.462 | NM_001166133 | FRAS1 | frameshift_deleti  on | exon24 | c.2912delC | p.T971fs | . | . |
| CTC2 | 4 | 79371343 | AC | A | 0.905 | NM_025074 | FRAS1 | frameshift_deleti  on | exon45 | c.6314delC | p.T2105fs | . | . |
| CTC2 | 4 | 79430080 | GC | G | 0.636 | NM_025074 | FRAS1 | frameshift_deleti  on | exon63 | c.9701delC | p.A3234fs | . | . |
| CTC2 | 4 | 80328131 | CA | C | 0.39 | NM_033214 | GK2 | frameshift_deleti  on | exon1 | c.1223delT | p.L408fs | . | . |
| CTC2 | 4 | 81283989 | AG | A | 0.304 | NM_001206997 | C4orf22 | frameshift_deleti  on | exon2 | c.194delG | p.R65fs | . | . |
| CTC2 | 4 | 82061688 | TCA | T | 0.273 | NM_001282481 | PRKG2 | frameshift_deleti  on | exon7 | c.281_282del | p.V94fs | . | . |
| CTC2 | 4 | 82073202 | CT | C | 0.75 | NM_001282485 | PRKG2 | frameshift_deleti  on | exon8 | c.996delA | p.K332fs | . | . |
| CTC2 | 4 | 82369333 | TG | T | 0.715 | NM_152545 | RASGEF1B | frameshift_deleti  on | exon5 | c.543delC | p.S181fs | . | . |
| CTC2 | 4 | 83772604 | GA | G | 0.3 | NM_016211 | SEC31A | frameshift_deleti  on | exon17 | c.2190delT | p.F730fs | . | . |
| CTC2 | 4 | 83772724 | CT | C | 0.375 | NM_016211 | SEC31A | frameshift_deleti  on | exon17 | c.2070delA | p.K690fs | . | . |
| CTC2 | 4 | 84362544 | C | CT | 0.8 | NM_133636 | HELQ | frameshift_inserti  on | exon7 | c.1575dupA | p.E526fs | . | . |
| CTC2 | 4 | 84390207 | TA | T | 0.3 | NM_139076 | FAM175A | frameshift_deleti  on | exon6 | c.573delT | p.F191fs | . | . |
| CTC2 | 4 | 86916263 | AT | A | 0.5 | NM_031305 | ARHGAP24 | frameshift_deleti  on | exon6 | c.1178delT | p.I393fs | . | . |
| CTC2 | 4 | 87556434 | CT | C | 0.385 | NM_006264 | PTPN13 | frameshift_deleti  on | exon2 | c.26delT | p.L9fs | . | . |
| CTC2 | 4 | 87679477 | GA | G | 0.5 | NM_080684 | PTPN13 | frameshift_deleti  on | exon18 | c.2716delA | p.K906fs | . | . |
| CTC2 | 4 | 88035970 | C | CA | 0.8 | NM_005935 | AFF1 | frameshift_inserti  on | exon11 | c.1965dupA | p.A655fs | . | . |
| CTC2 | 4 | 88537072 | TAGCAG  TGAC | T | 1 | NM_014208 | DSPP | nonframeshift_de  letion | exon5 | c.3259_3267del | p.1087_1089de  l | . | . |
| CTC2 | 4 | 88537297 | CAGCAG  CGAT | C | 0.75 | NM_014208 | DSPP | nonframeshift_de  letion | exon5 | c.3484_3492del | p.1162_1164de  l | . | . |
| CTC2 | 4 | 89199490 | CT | C | 1 | NM_152542 | PPM1K | frameshift_deleti  on | exon2 | c.245delA | p.K82fs | . | . |
| CTC2 | 4 | 89329744 | CA | C | 0.581 | NM_001165136 | HERC6 | frameshift_deleti  on | exon11 | c.1344delA | p.T448fs | . | . |
| CTC2 | 4 | 89381317 | CA | C | 1 | NM_016323 | HERC5 | frameshift_deleti  on | exon3 | c.462delA | p.S154fs | . | . |
| CTC2 | 4 | 89618451 | TC | T | 0.652 | NM_153757 | NAP1L5 | frameshift_deleti  on | exon1 | c.454delG | p.E152fs | . | . |
| CTC2 | 4 | 89618847 | TC | T | 0.222 | NM_153757 | NAP1L5 | frameshift_deleti  on | exon1 | c.58delG | p.E20fs | . | . |
| CTC2 | 4 | 94750909 | GC | G | 0.395 | NM_005172 | ATOH1 | frameshift_deleti  on | exon1 | c.833delC | p.A278fs | . | . |
| CTC2 | 4 | 100529971 | G | GA | 0.375 | NM_000253 | MTTP | frameshift_inserti  on | exon13 | c.1607dupA | p.E536fs | . | . |
| CTC2 | 4 | 101342518 | CA | C | 0.375 | NM_001159694 | EMCN | frameshift_deleti  on | exon7 | c.545delT | p.V182fs | . | . |
| CTC2 | 4 | 103952923 | GA | G | 0.611 | NM_178833 | SLC9B2 | frameshift_deleti  on | exon10 | c.1188delT | p.F396fs | . | . |
| CTC2 | 4 | 104030028 | CG | C | 0.324 | NM_001286734 | CENPE | frameshift_deleti  on | exon46 | c.7579delC | p.R2527fs | . | . |
| CTC2 | 4 | 106588619 | A | AT | 0.143 | NM_001242729 | ARHGEF38 | frameshift_inserti  on | exon13 | c.1908dupT | p.Y636fs | . | . |
| CTC2 | 4 | 106848503 | AC | A | 0.262 | NM_001033047 | NPNT | frameshift_deleti  on | exon3 | c.184delC | p.P62fs | . | . |
| CTC2 | 4 | 107237742 | TC | T | 1 | NM_001142416 | AIMP1 | frameshift_deleti  on | exon1 | c.39delC | p.L13fs | . | . |
| CTC2 | 4 | 111398059 | CA | C | 0.3 | NM_001977 | ENPEP | frameshift_deleti  on | exon1 | c.490delA | p.K164fs | . | . |

| CTC2 | 4 | 113352687 | CA | C | 0.213 | NM_001253884 | ALPK1 | frameshift_deleti  on | exon10 | c.1751delA | p.Q584fs | . | . |
| --- | --- | --- | --- | --- | --- | --- | --- | --- | --- | --- | --- | --- | --- |
| CTC2 | 4 | 114274616 | TC | T | 0.27 | NM_001148 | ANK2 | frameshift_deleti  on | exon38 | c.4843delC | p.P1615fs | . | . |
| CTC2 | 4 | 114276427 | TG | T | 1 | NM_001148 | ANK2 | frameshift_deleti  on | exon38 | c.6654delG | p.L2218fs | . | . |
| CTC2 | 4 | 119612696 | G | GA | 0.5 | NM_020961 | METTL14 | frameshift_inserti  on | exon4 | c.254dupA | p.E85fs | . | . |
| CTC2 | 4 | 119686065 | TG | T | 0.2 | NM_014822 | SEC24D | frameshift_deleti  on | exon10 | c.1187delC | p.P396fs | . | . |
| CTC2 | 4 | 119948188 | GA | G | 1 | NM_001128933 | SYNPO2 | frameshift_deleti  on | exon3 | c.665delA | p.E222fs | . | . |
| CTC2 | 4 | 119952609 | GC | G | 0.943 | NM_001128933 | SYNPO2 | frameshift_deleti  on | exon4 | c.2680delC | p.Q894fs | . | . |
| CTC2 | 4 | 121957508 | AT | A | 0.429 | NM_024574 | NDNF | frameshift_deleti  on | exon4 | c.1617delA | p.K539fs | . | . |
| CTC2 | 4 | 122782705 | GT | G | 1 | NM_018190 | BBS7 | frameshift_deleti  on | exon4 | c.294delA | p.K98fs | . | . |
| CTC2 | 4 | 123122273 | TC | T | 1 | NM_015312 | KIAA1109 | frameshift_deleti  on | exon13 | c.1491delC | p.F497fs | . | . |
| CTC2 | 4 | 125590560 | GA | G | 0.382 | NM_001167882 | ANKRD50 | frameshift_deleti  on | exon3 | c.3334delT | p.S1112fs | . | . |
| CTC2 | 4 | 126369820 | A | AT | 0.895 | NM_024582 | FAT4 | frameshift_inserti  on | exon9 | c.7650dupT | p.D2550fs | . | . |
| CTC2 | 4 | 126372420 | GT | G | 0.553 | NM_024582 | FAT4 | frameshift_deleti  on | exon9 | c.10250delT | p.V3417fs | . | . |
| CTC2 | 4 | 126412055 | CAA | C | 0.16 | NM_024582 | FAT4 | frameshift_deleti  on | exon17 | c.14079_14080  del | p.P4693fs | . | . |
| CTC2 | 4 | 128748509 | CA | C | 0.262 | NM_014278 | HSPA4L | frameshift_deleti  on | exon17 | c.2097delA | p.P699fs | . | . |
| CTC2 | 4 | 128807697 | CA | C | 0.526 | NM_001190799 | PLK4 | frameshift_deleti  on | exon4 | c.1077delA | p.A359fs | . | . |
| CTC2 | 4 | 134071317 | G | GC | 0.182 | NM_020815 | PCDH10 | frameshift_inserti  on | exon1 | c.23dupC | p.A8fs | . | . |
| CTC2 | 4 | 138442190 | TG | T | 1 | NM_019035 | PCDH18 | frameshift_deleti  on | exon4 | c.3400delC | p.Q1134fs | . | . |
| CTC2 | 4 | 139144433 | CG | C | 1 | NM_014331 | SLC7A11 | frameshift_deleti  on | exon4 | c.565delC | p.R189fs | . | . |
| CTC2 | 4 | 139163201 | GAC | G | 0.141 | NM_014331 | SLC7A11 | frameshift_deleti  on | exon1 | c.21_22del | p.V7fs | . | . |
| CTC2 | 4 | 140394127 | TA | T | 0.471 | NM_031296 | RAB33B | frameshift_deleti  on | exon2 | c.538delA | p.K180fs | . | . |
| CTC2 | 4 | 140810993 | TA | T | 0.706 | NM_018717 | MAML3 | frameshift_deleti  on | exon3 | c.1584delT | p.F528fs | . | . |
| CTC2 | 4 | 140811063 | TTGCTGC  TGCTGC | T | 1 | NM_018717 | MAML3 | frameshift_deleti  on | exon3 | c.1513_1514del | p.Q505fs | . | . |
| CTC2 | 4 | 146770678 | T | TA | 0.295 | NM_178835 | ZNF827 | stopgain | exon6 | c.2016dupT | p.K673_E674de  linsX | . | . |
| CTC2 | 4 | 148768338 | GA | G | 1 | NM_024605 | ARHGAP10 | frameshift_deleti  on | exon4 | c.350delA | p.E117fs | . | . |
| CTC2 | 4 | 148968107 | GT | G | 0.8 | NM_024605 | ARHGAP10 | frameshift_deleti  on | exon20 | c.1933delT | p.S645fs | . | . |
| CTC2 | 4 | 152629151 | GC | G | 0.372 | NM_004564 | PET112 | frameshift_deleti  on | exon6 | c.865delG | p.A289fs | . | . |
| CTC2 | 4 | 153690932 | CT | C | 0.312 | NM_145720 | TIGD4 | frameshift_deleti  on | exon2 | c.1224delA | p.G408fs | . | . |
| CTC2 | 4 | 153691418 | AT | A | 0.818 | NM_145720 | TIGD4 | frameshift_deleti  on | exon2 | c.738delA | p.K246fs | . | . |
| CTC2 | 4 | 153691502 | CA | C | 0.75 | NM_145720 | TIGD4 | frameshift_deleti  on | exon2 | c.654delT | p.V218fs | . | . |
| CTC2 | 4 | 154624637 | GT | G | 0.571 | NM_003264 | TLR2 | frameshift_deleti  on | exon3 | c.579delT | p.S193fs | . | . |
| CTC2 | 4 | 154624690 | AT | A | 0.28 | NM_003264 | TLR2 | frameshift_deleti  on | exon3 | c.632delT | p.I211fs | . | . |
| CTC2 | 4 | 155243558 | AG | A | 0.2 | NM_017639 | DCHS2 | frameshift_deleti  on | exon13 | c.2735delC | p.P912fs | . | . |
| CTC2 | 4 | 155256132 | GA | G | 0.5 | NM_001142552 | DCHS2 | frameshift_deleti  on | exon4 | c.2600delT | p.F867fs | . | . |
| CTC2 | 4 | 155532977 | TA | T | 0.417 | NM_000509 | FGG | stopgain | exon4 | c.380delT | p.L127X | . | . |
| CTC2 | 4 | 159568213 | GT | G | 1 | NM_001253729 | RXFP1 | frameshift_deleti on | exon14 | c.1473delT | p.G491fs | . | . |
| CTC2 | 4 | 164050476 | TG | T | 0.319 | NM_138386 | NAF1 | frameshift_deleti  on | exon8 | c.1057delC | p.Q353fs | . | . |
| CTC2 | 4 | 165118260 | CT | C | 0.308 | NM_012403 | ANP32C | frameshift_deleti  on | exon1 | c.603delA | p.G201fs | . | . |
| CTC2 | 4 | 166215600 | A | AT | 0.222 | NM_001161522 | KLHL2 | frameshift_inserti  on | exon5 | c.370_371insT | p.T124fs | . | . |
| CTC2 | 4 | 166408623 | GT | G | 0.278 | NM_001873 | CPE | frameshift_deleti  on | exon6 | c.1008delT | p.C336fs | . | . |
| CTC2 | 4 | 166910541 | TG | T | 0.412 | NM_001204760 | TLL1 | frameshift_deleti  on | exon2 | c.179delG | p.W60fs | . | . |
| CTC2 | 4 | 169305801 | CCAG | C | 0.277 | NM_001012967 | DDX60L | nonframeshift_de  letion | exon30 | c.4075_4077del | p.1359_1359de  l | . | . |
| CTC2 | 4 | 170028321 | GC | G | 0.367 | NM_020870 | SH3RF1 | frameshift_deleti  on | exon11 | c.2174delG | p.G725fs | . | . |
| CTC2 | 4 | 170985932 | TC | T | 0.32 | NM_016228 | AADAT | stopgain | exon11 | c.1071delG | p.W357X | . | . |
| CTC2 | 4 | 174219315 | GAA | G | 0.525 | NM_017423 | GALNT7 | frameshift_deleti on | exon6 | c.1016_1017del | p.E339fs | . | . |
| CTC2 | 4 | 184368300 | TG | T | 1 | NM_017632 | CDKN2AIP | frameshift_deleti  on | exon3 | c.1464delG | p.L488fs | . | . |
| CTC2 | 4 | 186244836 | A | AT | 0.545 | NM_031953 | SNX25 | frameshift_inserti  on | exon9 | c.1140dupT | p.Y380fs | . | . |
| CTC2 | 4 | 187005229 | GT | G | 0.929 | NM_003265 | TLR3 | frameshift_deleti  on | exon4 | c.2390delT | p.V797fs | . | . |
| CTC2 | 4 | 187075694 | AG | A | 0.359 | NM_001006655 | FAM149A | frameshift_deleti  on | exon5 | c.286delG | p.G96fs | . | . |
| CTC2 | 4 | 187149353 | A | AT | 0.526 | NM_000892 | KLKB1 | frameshift_inserti  on | exon2 | c.5dupT | p.I2fs | . | . |
| CTC2 | 4 | 187157954 | TA | T | 0.812 | NM_000892 | KLKB1 | frameshift_deleti  on | exon5 | c.349delA | p.K117fs | . | . |
| CTC2 | 4 | 187524427 | C | CT | 0.276 | NM_005245 | FAT1 | frameshift_inserti  on | exon19 | c.11252dupA | p.K3751fs | . | . |
| CTC2 | 4 | 187557872 | TC | T | 0.862 | NM_005245 | FAT1 | frameshift_deleti  on | exon5 | c.3838delG | p.D1280fs | . | . |
| CTC2 | 5 | 163056 | GC | G | 0.211 | NM_052909 | PLEKHG4B | frameshift_deleti  on | exon11 | c.1802delC | p.A601fs | . | . |
| CTC2 | 5 | 886757 | TA | T | 0.912 | NM_001009877 | BRD9 | frameshift_deleti  on | exon7 | c.623delT | p.V208fs | . | . |
| CTC2 | 5 | 1477550 | AC | A | 0.279 | NM_024830 | LPCAT1 | frameshift_deleti  on | exon9 | c.867delG | p.L289fs | . | . |
| CTC2 | 5 | 5303420 | TGGC | T | 0.169 | NM_139056 | ADAMTS16 | nonframeshift_de  letion | exon19 | c.2830_2832del | p.944_944del | . | . |
| CTC2 | 5 | 5461722 | AT | A | 0.359 | NM_015325 | KIAA0947 | frameshift_deleti  on | exon13 | c.2276delT | p.I759fs | . | . |

| CTC2 | 5 | 7892869 | AT | A | 0.359 | NM_002454 | MTRR | frameshift_deleti  on | exon11 | c.1401delT | p.H467fs | . | . |
| --- | --- | --- | --- | --- | --- | --- | --- | --- | --- | --- | --- | --- | --- |
| CTC2 | 5 | 10391767 | AGAG | A | 0.135 | NM_001270661 | 6-Mar | nonframeshift_de  letion | exon4 | c.376_378del | p.126_126del | . | . |
| CTC2 | 5 | 11411753 | CT | C | 0.448 | NM_001288715 | CTNND2 | frameshift_deleti  on | exon4 | c.60delA | p.K20fs | . | . |
| CTC2 | 5 | 13830807 | GT | G | 0.75 | NM_001369 | DNAH5 | frameshift_deleti  on | exon36 | c.5959delA | p.T1987fs | . | . |
| CTC2 | 5 | 13841159 | G | GA | 0.245 | NM_001369 | DNAH5 | frameshift_inserti  on | exon34 | c.5564dupT | p.I1855fs | . | . |
| CTC2 | 5 | 13841906 | CAA | C | 0.186 | NM_001369 | DNAH5 | frameshift_deleti  on | exon33 | c.5377_5378del | p.L1793fs | . | . |
| CTC2 | 5 | 13885331 | CT | C | 0.542 | NM_001369 | DNAH5 | frameshift_deleti  on | exon19 | c.2749delA | p.R917fs | . | . |
| CTC2 | 5 | 14387713 | T | TG | 0.429 | NM_007118 | TRIO | frameshift_inserti  on | exon22 | c.3738dupG | p.L1246fs | . | . |
| CTC2 | 5 | 14387739 | TC | T | 0.441 | NM_007118 | TRIO | stopgain | exon22 | c.3764delC | p.S1255X | . | . |
| CTC2 | 5 | 14399055 | CAA | C | 0.657 | NM_007118 | TRIO | frameshift_deleti on | exon30 | c.4491_4492del | p.P1497fs | . | . |
| CTC2 | 5 | 14741974 | TG | T | 0.19 | NM_054027 | ANKH | frameshift_deleti  on | exon8 | c.972delC | p.I324fs | . | . |
| CTC2 | 5 | 14749413 | TC | T | 1 | NM_054027 | ANKH | frameshift_deleti  on | exon6 | c.689delG | p.G230fs | . | . |
| CTC2 | 5 | 16694592 | AC | A | 0.902 | NM_012334 | MYO10 | frameshift_deleti  on | exon27 | c.3687delG | p.L1229fs | . | . |
| CTC2 | 5 | 23526526 | T | TCC | 0.3 | NM_020227 | PRDM9 | frameshift_inserti  on | exon11 | c.1329_1330ins  CC | p.D443fs | . | . |
| CTC2 | 5 | 24498524 | T | TA | 0.545 | NM_006727 | CDH10 | stopgain | exon9 | c.1497_1498ins  T | p.N500_A501d  elinsX | . | . |
| CTC2 | 5 | 24509852 | T | TA | 0.538 | NM_006727 | CDH10 | frameshift_inserti  on | exon7 | c.1078dupT | p.Y360fs | . | . |
| CTC2 | 5 | 31534420 | TC | T | 1 | NM_018356 | C5orf22 | frameshift_deleti  on | exon2 | c.124delC | p.P42fs | . | . |
| CTC2 | 5 | 32089592 | GC | G | 0.471 | NM_178140 | PDZD2 | frameshift_deleti  on | exon19 | c.6039delC | p.C2013fs | . | . |
| CTC2 | 5 | 33457438 | CT | C | 0.345 | NM_152295 | TARS | frameshift_deleti  on | exon9 | c.915delT | p.P305fs | . | . |
| CTC2 | 5 | 33576816 | AT | A | 0.348 | NM_030955 | ADAMTS12 | frameshift_deleti  on | exon19 | c.3314delA | p.N1105fs | . | . |
| CTC2 | 5 | 35876577 | CA | C | 0.667 | NM_002185 | IL7R | frameshift_deleti  on | exon8 | c.1370delA | p.Q457fs | . | . |
| CTC2 | 5 | 35955827 | AT | A | 0.619 | NM_152404 | UGT3A1 | frameshift_deleti  on | exon6 | c.1214delA | p.N405fs | . | . |
| CTC2 | 5 | 36037906 | CT | C | 0.389 | NM_001168316 | UGT3A2 | frameshift_deleti  on | exon5 | c.1185delA | p.E395fs | . | . |
| CTC2 | 5 | 36038003 | GT | G | 0.286 | NM_001168316 | UGT3A2 | frameshift_deleti  on | exon5 | c.1088delA | p.N363fs | . | . |
| CTC2 | 5 | 37302885 | AT | A | 1 | NM_001278312 | NUP155 | frameshift_deleti  on | exon28 | c.3250delA | p.M1084fs | . | . |
| CTC2 | 5 | 38370526 | GC | G | 0.389 | NM_001205301 | EGFLAM | frameshift_deleti  on | exon6 | c.675delC | p.S225fs | . | . |
| CTC2 | 5 | 38482106 | GC | G | 0.625 | NM_001127671 | LIFR | frameshift_deleti  on | exon20 | c.2884delG | p.A962fs | . | . |
| CTC2 | 5 | 38506688 | TA | T | 0.5 | NM_001127671 | LIFR | stopgain | exon8 | c.1037delT | p.L346X | . | . |
| CTC2 | 5 | 38952490 | TG | T | 0.6 | NM_001285439 | RICTOR | frameshift_deleti on | exon30 | c.2934delC | p.T978fs | . | . |
| CTC2 | 5 | 40681703 | AC | A | 0.778 | NM_000958 | PTGER4 | frameshift_deleti  on | exon2 | c.609delC | p.N203fs | . | . |
| CTC2 | 5 | 41862799 | TG | T | 0.821 | NM_000436 | OXCT1 | frameshift_deleti  on | exon2 | c.131delC | p.T44fs | . | . |
| CTC2 | 5 | 41929930 | TGG | T | 0.8 | NM_012176 | FBXO4 | frameshift_deleti  on | exon3 | c.558_559del | p.L186fs | . | . |
| CTC2 | 5 | 43649256 | AG | A | 0.453 | NM_012343 | NNT | frameshift_deleti  on | exon11 | c.1453delG | p.G485fs | . | . |
| CTC2 | 5 | 44388570 | TG | T | 0.545 | NM_004465 | FGF10 | frameshift_deleti  on | exon1 | c.214delC | p.H72fs | . | . |
| CTC2 | 5 | 50057708 | CG | C | 0.831 | NM_001178056 | PARP8 | frameshift_deleti  on | exon6 | c.400delG | p.E134fs | . | . |
| CTC2 | 5 | 55407067 | GA | G | 0.491 | NM_024669 | ANKRD55 | frameshift_deleti  on | exon10 | c.1507delT | p.S503fs | . | . |
| CTC2 | 5 | 59064143 | TG | T | 0.4 | NM_001197218 | PDE4D | frameshift_deleti  on | exon1 | c.192delC | p.P64fs | . | . |
| CTC2 | 5 | 60834681 | AC | A | 0.269 | NM_020928 | ZSWIM6 | frameshift_deleti  on | exon11 | c.2423delC | p.T808fs | . | . |
| CTC2 | 5 | 60839919 | C | CT | 0.556 | NM_020928 | ZSWIM6 | frameshift_inserti  on | exon14 | c.3424dupT | p.L1141fs | . | . |
| CTC2 | 5 | 61714860 | AC | A | 0.6 | NM_001134779 | IPO11 | frameshift_deleti  on | exon1 | c.21delC | p.H7fs | . | . |
| CTC2 | 5 | 64881929 | AC | A | 0.603 | NM_001278929 | PPWD1 | frameshift_deleti  on | exon9 | c.1251delC | p.D417fs | . | . |
| CTC2 | 5 | 65054426 | CG | C | 0.316 | NM_020726 | NLN | frameshift_deleti  on | exon2 | c.75delG | p.T25fs | . | . |
| CTC2 | 5 | 65349723 | GA | G | 0.264 | NM_001006600 | ERBB2IP | frameshift_deleti  on | exon21 | c.2578delA | p.K860fs | . | . |
| CTC2 | 5 | 68669814 | GA | G | 0.6 | NM_133339 | RAD17 | frameshift_deleti  on | exon2 | c.201delA | p.G67fs | . | . |
| CTC2 | 5 | 71616290 | TG | T | 0.75 | NM_001284403 | PTCD2 | frameshift_deleti  on | exon1 | c.82delG | p.G28fs | . | . |
| CTC2 | 5 | 74021846 | TACTCA  A | T | 0.86 | NM_170691 | GFM2 | nonframeshift_de  letion | exon17 | c.1685_1690del | p.562_564del | . | . |
| CTC2 | 5 | 74491715 | TTCA | T | 0.545 | NM_001164443 | ANKRD31 | nonframeshift_de  letion | exon7 | c.755_757del | p.252_253del | . | . |
| CTC2 | 5 | 77409617 | GT | G | 0.385 | NM_001271769 | AP3B1 | frameshift_deleti  on | exon19 | c.2060delA | p.N687fs | . | . |
| CTC2 | 5 | 77523233 | TA | T | 0.545 | NM_001271769 | AP3B1 | frameshift_deleti  on | exon5 | c.351delT | p.V117fs | . | . |
| CTC2 | 5 | 79035080 | GA | G | 0.5 | NM_153610 | CMYA5 | frameshift_deleti  on | exon2 | c.10493delA | p.E3498fs | . | . |
| CTC2 | 5 | 79734710 | GT | G | 1 | NM_001284237 | ZFYVE16 | frameshift_deleti  on | exon3 | c.2207delT | p.V736fs | . | . |
| CTC2 | 5 | 86672241 | GA | G | 0.636 | NM_002890 | RASA1 | frameshift_deleti  on | exon16 | c.2044delA | p.K682fs | . | . |
| CTC2 | 5 | 89923276 | CACA | C | 0.545 | NM_032119 | GPR98 | nonframeshift_de  letion | exon7 | c.922_924del | p.308_308del | . | . |
| CTC2 | 5 | 89923547 | GT | G | 0.833 | NM_032119 | GPR98 | frameshift_deleti  on | exon7 | c.1193delT | p.V398fs | . | . |
| CTC2 | 5 | 90083926 | CT | C | 0.588 | NM_032119 | GPR98 | frameshift_deleti  on | exon68 | c.13693delT | p.L4565fs | . | . |
| CTC2 | 5 | 90672555 | AT | A | 1 | NM_020801 | ARRDC3 | frameshift_deleti  on | exon3 | c.392delA | p.H131fs | . | . |
| CTC2 | 5 | 96339142 | GA | G | 0.621 | NM_005575 | LNPEP | frameshift_deleti  on | exon9 | c.1678delA | p.K560fs | . | . |

| CTC2 | 5 | 98215282 | GGA | G | 0.296 | NM_001270 | CHD1 | frameshift_deleti  on | exon22 | c.3209_3210del | p.L1070fs | . | . |
| --- | --- | --- | --- | --- | --- | --- | --- | --- | --- | --- | --- | --- | --- |
| CTC2 | 5 | 102345545 | TA | T | 0.5 | NM_138821 | PAM | frameshift_deleti  on | exon19 | c.1986delA | p.I662fs | . | . |
| CTC2 | 5 | 102611663 | AC | A | 0.429 | NM_033211 | C5orf30 | frameshift_deleti  on | exon3 | c.44delC | p.T15fs | . | . |
| CTC2 | 5 | 109963560 | G | GT | 0.279 | NM_001039763 | TMEM232 | frameshift_inserti  on | exon6 | c.522dupA | p.R175fs | . | . |
| CTC2 | 5 | 111530273 | CT | C | 0.479 | NM_022140 | EPB41L4A | frameshift_deleti  on | exon17 | c.1493delA | p.K498fs | . | . |
| CTC2 | 5 | 112179463 | CT | C | 0.9 | NM_001127511 | APC | frameshift_deleti  on | exon14 | c.8119delT | p.F2707fs | . | . |
| CTC2 | 5 | 112874859 | TA | T | 1 | NM_022828 | YTHDC2 | frameshift_deleti  on | exon8 | c.1192delA | p.K398fs | . | . |
| CTC2 | 5 | 118469273 | AC | A | 0.221 | NM_005509 | DMXL1 | frameshift_deleti  on | exon12 | c.1655delC | p.T552fs | . | . |
| CTC2 | 5 | 120022200 | TG | T | 0.263 | NM_016644 | PRR16 | frameshift_deleti  on | exon3 | c.643delG | p.V215fs | . | . |
| CTC2 | 5 | 120022355 | CA | C | 0.714 | NM_016644 | PRR16 | frameshift_deleti  on | exon3 | c.798delA | p.P266fs | . | . |
| CTC2 | 5 | 122506734 | GC | G | 0.5 | NM_001136239 | PRDM6 | frameshift_deleti  on | exon6 | c.1429delC | p.Q477fs | . | . |
| CTC2 | 5 | 125952950 | TC | T | 0.714 | NM_032177 | PHAX | frameshift_deleti  on | exon4 | c.841delC | p.R281fs | . | . |
| CTC2 | 5 | 128302136 | GA | G | 0.351 | NM_001017372 | SLC27A6 | frameshift_deleti  on | exon1 | c.307delA | p.K103fs | . | . |
| CTC2 | 5 | 129520926 | A | AG | 1 | NM_175856 | CHSY3 | frameshift_inserti  on | exon3 | c.2092dupG | p.R697fs | . | . |
| CTC2 | 5 | 131326657 | TG | T | 0.337 | NM_001009185 | ACSL6 | frameshift_deleti  on | exon3 | c.273delC | p.D91fs | . | . |
| CTC2 | 5 | 131995416 | TAC | T | 1 | NM_002188 | IL13 | frameshift_deleti  on | exon3 | c.230_231del | p.Y77fs | . | . |
| CTC2 | 5 | 132097532 | CT | C | 0.6 | NM_001098811 | 8-Sep | frameshift_deleti  on | exon6 | c.752delA | p.K251fs | . | . |
| CTC2 | 5 | 132234072 | AG | A | 0.288 | NM_014423 | AFF4 | frameshift_deleti  on | exon10 | c.1238delC | p.P413fs | . | . |
| CTC2 | 5 | 133534882 | CA | C | 0.185 | NM_002715 | PPP2CA | frameshift_deleti  on | exon6 | c.751delT | p.C251fs | . | . |
| CTC2 | 5 | 133914189 | CT | C | 0.207 | NM_015288 | JADE2 | frameshift_deleti  on | exon11 | c.1556delT | p.L519fs | . | . |
| CTC2 | 5 | 134153253 | CA | C | 0.562 | NM_014829 | DDX46 | frameshift_deleti  on | exon20 | c.2679delA | p.A893fs | . | . |
| CTC2 | 5 | 134366970 | ACG | A | 0.459 | NM_002653 | PITX1 | frameshift_deleti  on | exon2 | c.396_397del | p.R132fs | . | . |
| CTC2 | 5 | 138209803 | G | GA | 0.221 | NM_015564 | LRRTM2 | frameshift_inserti  on | exon2 | c.446_447insT | p.H149fs | . | . |
| CTC2 | 5 | 138658519 | AG | A | 0.343 | NM_001194956 | MATR3 | frameshift_deleti  on | exon11 | c.1148delG | p.S383fs | . | . |
| CTC2 | 5 | 138739663 | CT | C | 0.579 | NM_194296 | SPATA24 | frameshift_deleti  on | exon1 | c.86delA | p.Q29fs | . | . |
| CTC2 | 5 | 140032592 | TGA | T | 0.156 | NM_006083 | IK | frameshift_deleti  on | exon5 | c.268_269del | p.E90fs | . | . |
| CTC2 | 5 | 140039713 | GA | G | 1 | NM_006083 | IK | frameshift_deleti  on | exon15 | c.1289delA | p.E430fs | . | . |
| CTC2 | 5 | 140235917 | GC | G | 0.739 | NM_018901 | PCDHA10 | frameshift_deleti  on | exon1 | c.285delC | p.C95fs | . | . |
| CTC2 | 5 | 140248974 | TGC | T | 0.8 | NM_018902 | PCDHA11 | frameshift_deleti  on | exon1 | c.287_288del | p.C96fs | . | . |
| CTC2 | 5 | 140432302 | CA | C | 0.429 | NM_013340 | PCDHB1 | frameshift_deleti  on | exon1 | c.1248delA | p.S416fs | . | . |
| CTC2 | 5 | 140580828 | AG | A | 0.235 | NM_018931 | PCDHB11 | frameshift_deleti  on | exon1 | c.1482delG | p.Q494fs | . | . |
| CTC2 | 5 | 140711008 | G | GA | 0.824 | NM_018912 | PCDHGA1 | frameshift_inserti  on | exon1 | c.758dupA | p.E253fs | . | . |
| CTC2 | 5 | 140739828 | CA | C | 0.889 | NM_018923 | PCDHGB2 | frameshift_deleti  on | exon1 | c.127delA | p.K43fs | . | . |
| CTC2 | 5 | 140773406 | C | CA | 0.468 | NM_014004 | PCDHGA8 | frameshift_inserti  on | exon1 | c.1027dupA | p.D342fs | . | . |
| CTC2 | 5 | 140783404 | TG | T | 0.667 | NM_018921 | PCDHGA9 | frameshift_deleti  on | exon1 | c.886delG | p.E296fs | . | . |
| CTC2 | 5 | 140788413 | TG | T | 1 | NM_018926 | PCDHGB6 | frameshift_deleti  on | exon1 | c.645delG | p.L215fs | . | . |
| CTC2 | 5 | 140866272 | AC | A | 0.85 | NM_018928 | PCDHGC4 | frameshift_deleti  on | exon1 | c.1533delC | p.N511fs | . | . |
| CTC2 | 5 | 140866327 | GA | G | 0.167 | NM_018928 | PCDHGC4 | frameshift_deleti  on | exon1 | c.1588delA | p.T530fs | . | . |
| CTC2 | 5 | 140869578 | TG | T | 1 | NM_018929 | PCDHGC5 | frameshift_deleti  on | exon1 | c.772delG | p.G258fs | . | . |
| CTC2 | 5 | 140870536 | CA | C | 0.8 | NM_018929 | PCDHGC5 | frameshift_deleti  on | exon1 | c.1730delA | p.Q577fs | . | . |
| CTC2 | 5 | 140874377 | GC | G | 0.455 | NM_002588 | PCDHGC3 | frameshift_deleti  on | exon2 | c.2435delC | p.A812fs | . | . |
| CTC2 | 5 | 140953563 | TGGA | T | 0.239 | NM_001079812 | DIAPH1 | nonframeshift_de  letion | exon15 | c.1824_1826del | p.608_609del | . | . |
| CTC2 | 5 | 141009619 | TC | T | 0.8 | NM_003883 | HDAC3 | frameshift_deleti  on | exon4 | c.354delG | p.L118fs | . | . |
| CTC2 | 5 | 141025373 | CA | C | 0.227 | NM_033449 | FCHSD1 | frameshift_deleti  on | exon13 | c.1275delT | p.S425fs | . | . |
| CTC2 | 5 | 141033553 | GC | G | 0.4 | NM_022481 | ARAP3 | frameshift_deleti  on | exon33 | c.4598delG | p.C1533fs | . | . |
| CTC2 | 5 | 145512465 | CA | C | 1 | NM_020117 | LARS | frameshift_deleti  on | exon23 | c.2391delT | p.F797fs | . | . |
| CTC2 | 5 | 145616968 | CA | C | 0.5 | NM_018989 | RBM27 | frameshift_deleti  on | exon8 | c.1253delA | p.Q418fs | . | . |
| CTC2 | 5 | 147796565 | TC | T | 0.69 | NM_001271723 | FBXO38 | frameshift_deleti  on | exon12 | c.1417delC | p.R473fs | . | . |
| CTC2 | 5 | 149374879 | CT | C | 1 | NM_001243253 | TIGD6 | frameshift_deleti  on | exon2 | c.1032delA | p.Q344fs | . | . |
| CTC2 | 5 | 149503909 | TG | T | 1 | NM_002609 | PDGFRB | frameshift_deleti  on | exon14 | c.1926delC | p.S642fs | . | . |
| CTC2 | 5 | 149515433 | GC | G | 0.409 | NM_002609 | PDGFRB | frameshift_deleti  on | exon3 | c.48delG | p.L16fs | . | . |
| CTC2 | 5 | 149755752 | G | GA | 0.5 | NM_000356 | TCOF1 | frameshift_inserti  on | exon12 | c.1771dupA | p.R590fs | . | . |
| CTC2 | 5 | 150489357 | GA | G | 0.667 | NM_001193544 | ANXA6 | frameshift_deleti  on | exon21 | c.1544delT | p.F515fs | . | . |
| CTC2 | 5 | 150639455 | TC | T | 0.545 | NM_000405 | GM2A | frameshift_deleti  on | exon2 | c.222delC | p.V74fs | . | . |
| CTC2 | 5 | 150678237 | GC | G | 0.5 | NM_001145017 | SLC36A3 | frameshift_deleti  on | exon2 | c.135delG | p.M45fs | . | . |
| CTC2 | 5 | 150696643 | AG | A | 0.429 | NM_181776 | SLC36A2 | frameshift_deleti  on | exon10 | c.1186delC | p.L396fs | . | . |
| CTC2 | 5 | 150901206 | TC | T | 0.337 | NM_001447 | FAT2 | frameshift_deleti  on | exon18 | c.10947delG | p.R3649fs | . | . |
| CTC2 | 5 | 151784506 | CA | C | 0.791 | NM_020167 | NMUR2 | frameshift_deleti  on | exon1 | c.168delT | p.F56fs | . | . |

| CTC2 | 5 | 154092508 | CA | C | 1 | NM_015315 | LARP1 | frameshift_deleti  on | exon1 | c.24delA | p.S8fs | . | . |
| --- | --- | --- | --- | --- | --- | --- | --- | --- | --- | --- | --- | --- | --- |
| CTC2 | 5 | 154210424 | AACC | A | 0.714 | NM_032385 | FAXDC2 | nonframeshift_de  letion | exon5 | c.300_302del | p.100_101del | . | . |
| CTC2 | 5 | 156533759 | TC | T | 0.767 | NM_032782 | HAVCR2 | frameshift_deleti  on | exon2 | c.272delG | p.G91fs | . | . |
| CTC2 | 5 | 156757810 | TC | T | 0.214 | NM_001037332 | CYFIP2 | frameshift_deleti  on | exon20 | c.2218delC | p.P740fs | . | . |
| CTC2 | 5 | 166802383 | TA | T | 0.774 | NM_001122679 | TENM2 | frameshift_deleti  on | exon2 | c.408delA | p.I136fs | . | . |
| CTC2 | 5 | 169535058 | G | GT | 0.412 | NM_012188 | FOXI1 | frameshift_inserti  on | exon2 | c.580_581insT | p.G194fs | . | . |
| CTC2 | 5 | 171621296 | CAAG | C | 0.214 | NM_001171183 | EFCAB9 | nonframeshift_de  letion | exon1 | c.121_123del | p.41_41del | . | . |
| CTC2 | 5 | 171766355 | CT | C | 1 | NM_001017995 | SH3PXD2B | frameshift_deleti  on | exon13 | c.1753delA | p.S585fs | . | . |
| CTC2 | 5 | 175919206 | GT | G | 0.385 | NM_014613 | FAF2 | frameshift_deleti  on | exon5 | c.357delT | p.R119fs | . | . |
| CTC2 | 5 | 176636702 | CA | C | 1 | NM_022455 | NSD1 | frameshift_deleti  on | exon5 | c.1303delA | p.K435fs | . | . |
| CTC2 | 5 | 176930171 | AGAG | A | 0.571 | NM_001144876 | DOK3 | nonframeshift_de  letion | exon5 | c.559_561del | p.187_187del | . | . |
| CTC2 | 5 | 176930176 | AGGG | A | 0.451 | NM_001144876 | DOK3 | nonframeshift_de  letion | exon5 | c.554_556del | p.185_186del | . | . |
| CTC2 | 5 | 179663387 | TCGA | T | 0.923 | NM_002752 | MAPK9 | nonframeshift_de  letion | exon12 | c.1269_1271del | p.423_424del | . | . |
| CTC2 | 5 | 180277726 | CA | C | 0.387 | NM_001172638 | ZFP62 | frameshift_deleti  on | exon2 | c.768delT | p.G256fs | . | . |
| CTC2 | 5 | 180278318 | GT | G | 0.632 | NM_001172638 | ZFP62 | frameshift_deleti  on | exon2 | c.176delA | p.N59fs | . | . |
| CTC2 | 6 | 3273615 | CG | C | 0.423 | NM_001286455 | SLC22A23 | frameshift_deleti  on | exon10 | c.891delC | p.S297fs | . | . |
| CTC2 | 6 | 4052962 | TA | T | 1 | NM_003913 | PRPF4B | frameshift_deleti  on | exon11 | c.2322delA | p.L774fs | . | . |
| CTC2 | 6 | 10529470 | CT | C | 0.577 | NM_145649 | GCNT2 | frameshift_deleti  on | exon3 | c.327delT | p.T109fs | . | . |
| CTC2 | 6 | 10556862 | AGTC | A | 0.3 | NM_001491 | GCNT2 | nonframeshift_de  letion | exon1 | c.207_209del | p.69_70del | . | . |
| CTC2 | 6 | 10556863 | GTCT | G | 0.392 | NM_001491 | GCNT2 | nonframeshift_de  letion | exon1 | c.208_210del | p.70_70del | . | . |
| CTC2 | 6 | 10586771 | C | CA | 0.276 | NM_145655 | GCNT2 | frameshift_inserti  on | exon1 | c.549_550insA | p.P183fs | . | . |
| CTC2 | 6 | 10702685 | AAG | A | 0.833 | NM_017906 | PAK1IP1 | frameshift_deleti  on | exon3 | c.332_333del | p.K111fs | . | . |
| CTC2 | 6 | 10773282 | AT | A | 0.387 | NM_001242957 | MAK | frameshift_deleti  on | exon13 | c.1656delA | p.K552fs | . | . |
| CTC2 | 6 | 10927472 | GAGC | G | 0.5 | NM_001040274 | SYCP2L | nonframeshift_de  letion | exon17 | c.1313_1315del | p.438_439del | . | . |
| CTC2 | 6 | 13053701 | AT | A | 0.281 | NM_001242648 | PHACTR1 | frameshift_deleti  on | exon5 | c.356delT | p.I119fs | . | . |
| CTC2 | 6 | 17799602 | G | GA | 0.203 | NM_001105566 | KIF13A | frameshift_inserti  on | exon22 | c.2684dupT | p.F895fs | . | . |
| CTC2 | 6 | 25606342 | GA | G | 0.293 | NM_001173977 | LRRC16A | frameshift_deleti  on | exon35 | c.3671delA | p.E1224fs | . | . |
| CTC2 | 6 | 25969553 | GA | G | 0.232 | NM_006355 | TRIM38 | frameshift_deleti  on | exon4 | c.413delA | p.E138fs | . | . |
| CTC2 | 6 | 26199816 | T | TC | 0.309 | NM_003522 | HIST1H2BF | frameshift_inserti  on | exon1 | c.31dupC | p.A10fs | . | . |
| CTC2 | 6 | 27278539 | TG | T | 0.889 | NM_033482 | POM121L2 | frameshift_deleti  on | exon1 | c.1410delC | p.P470fs | . | . |
| CTC2 | 6 | 27799137 | C | CT | 0.5 | NM_003541 | HIST1H4K | frameshift_inserti  on | exon1 | c.168_169insA | p.G57fs | . | . |
| CTC2 | 6 | 27858334 | G | GA | 1 | NM_003535 | HIST1H3J | frameshift_inserti  on | exon1 | c.236dupT | p.F79fs | . | . |
| CTC2 | 6 | 27879285 | TC | T | 0.556 | NM_033057 | OR2B2 | frameshift_deleti  on | exon1 | c.812delG | p.G271fs | . | . |
| CTC2 | 6 | 28121140 | AC | A | 0.18 | NM_001278122 | ZKSCAN8 | stopgain | exon6 | c.522delC | p.Y174X | . | . |
| CTC2 | 6 | 28359206 | TG | T | 0.226 | NM_001163391 | ZSCAN12 | frameshift_deleti on | exon4 | c.860delC | p.S287fs | . | . |
| CTC2 | 6 | 29141669 | TC | T | 0.483 | NM_030905 | OR2J2 | frameshift_deleti  on | exon1 | c.258delC | p.L86fs | . | . |
| CTC2 | 6 | 29142309 | AG | A | 0.53 | NM_030905 | OR2J2 | frameshift_deleti  on | exon1 | c.898delG | p.G300fs | . | . |
| CTC2 | 6 | 29394902 | TG | T | 0.294 | NM_013937 | OR11A1 | frameshift_deleti  on | exon1 | c.516delC | p.P172fs | . | . |
| CTC2 | 6 | 29395227 | GA | G | 0.844 | NM_013937 | OR11A1 | frameshift_deleti  on | exon1 | c.191delT | p.F64fs | . | . |
| CTC2 | 6 | 29455333 | AC | A | 0.182 | NM_052967 | MAS1L | frameshift_deleti  on | exon1 | c.346delG | p.V116fs | . | . |
| CTC2 | 6 | 29556307 | CA | C | 0.268 | NM_007160 | OR2H2 | frameshift_deleti  on | exon1 | c.587delA | p.Q196fs | . | . |
| CTC2 | 6 | 29556342 | G | GC | 0.75 | NM_007160 | OR2H2 | frameshift_inserti  on | exon1 | c.622dupC | p.V207fs | . | . |
| CTC2 | 6 | 29640752 | GA | G | 0.375 | NM_001109809 | ZFP57 | frameshift_deleti  on | exon4 | c.1135delT | p.S379fs | . | . |
| CTC2 | 6 | 30128416 | GC | G | 0.207 | NM_006778 | TRIM10 | frameshift_deleti  on | exon1 | c.219delG | p.W73fs | . | . |
| CTC2 | 6 | 30154079 | TTCC | T | 0.17 | NM_001242783 | TRIM26 | nonframeshift_de  letion | exon9 | c.1191_1193del | p.397_398del | . | . |
| CTC2 | 6 | 30558477 | G | GA | 1 | NM_001090 | ABCF1 | frameshift_inserti  on | exon24 | c.2424dupA | p.X808delinsX | . | . |
| CTC2 | 6 | 30573758 | TG | T | 0.273 | NM_002714 | PPP1R10 | frameshift_deleti  on | exon10 | c.794delC | p.P265fs | . | . |
| CTC2 | 6 | 30679788 | TTC | T | 0.4 | NM_014641 | MDC1 | frameshift_deleti  on | exon5 | c.1929_1930del | p.E643fs | . | . |
| CTC2 | 6 | 30876847 | GT | G | 0.357 | NM_001517 | GTF2H4 | frameshift_deleti  on | exon2 | c.35delT | p.V12fs | . | . |
| CTC2 | 6 | 31127381 | GC | G | 1 | NM_001077511 | TCF19 | frameshift_deleti  on | exon2 | c.136delC | p.P46fs | . | . |
| CTC2 | 6 | 31525881 | CCAG | C | 0.15 | NM_001144961 | NFKBIL1 | nonframeshift_de  letion | exon4 | c.595_597del | p.199_199del | . | . |
| CTC2 | 6 | 31594575 | GA | G | 0.308 | NM_004638 | PRRC2A | frameshift_deleti  on | exon10 | c.1010delA | p.E337fs | . | . |
| CTC2 | 6 | 31675706 | TG | T | 0.15 | NM_001003693 | LY6G6F | frameshift_deleti  on | exon3 | c.442delG | p.V148fs | . | . |
| CTC2 | 6 | 31695462 | GC | G | 0.556 | NM_013974 | DDAH2 | frameshift_deleti  on | exon6 | c.598delG | p.A200fs | . | . |
| CTC2 | 6 | 31726894 | TC | T | 0.323 | NM_002441 | MSH5 | frameshift_deleti  on | exon16 | c.1348delC | p.P450fs | . | . |
| CTC2 | 6 | 31732089 | AT | A | 0.962 | NM_001039651 | SAPCD1 | frameshift_deleti  on | exon4 | c.399delT | p.D133fs | . | . |
| CTC2 | 6 | 31911220 | GCC | G | 0.158 | NM_001178063 | C2 | frameshift_deleti  on | exon8 | c.842_843del | p.A281fs | . | . |

| CTC2 | 6 | 31937671 | TC | T | 0.5 | NM_005510 | DXO | frameshift_deleti  on | exon7 | c.1173delG | p.K391fs | . | . |
| --- | --- | --- | --- | --- | --- | --- | --- | --- | --- | --- | --- | --- | --- |
| CTC2 | 6 | 32006229 | GC | G | 0.556 | NM_000500 | CYP21A2 | frameshift_deleti  on | exon1 | c.31delC | p.P11fs | . | . |
| CTC2 | 6 | 32020721 | CG | C | 0.684 | NM_019105 | TNXB | frameshift_deleti  on | exon26 | c.8834delC | p.P2945fs | . | . |
| CTC2 | 6 | 32149385 | GC | G | 0.316 | NM_001206954 | AGER | frameshift_deleti  on | exon8 | c.846delG | p.L282fs | . | . |
| CTC2 | 6 | 32190350 | CT | C | 0.333 | NM_004557 | NOTCH4 | frameshift_deleti  on | exon3 | c.388delA | p.R130fs | . | . |
| CTC2 | 6 | 32372899 | CCA | C | 0.222 | NM_019602 | BTNL2 | frameshift_deleti  on | exon2 | c.242_243del | p.V81fs | . | . |
| CTC2 | 6 | 32948181 | GA | G | 0.489 | NM_001199456 | BRD2 | frameshift_deleti  on | exon11 | c.2069delA | p.E690fs | . | . |
| CTC2 | 6 | 33232582 | CT | C | 0.862 | NM_001289176 | VPS52 | frameshift_deleti  on | exon11 | c.809delA | p.K270fs | . | . |
| CTC2 | 6 | 33286920 | A | AG | 1 | NM_001254717 | DAXX | frameshift_inserti  on | exon6 | c.1791dupC | p.L598fs | . | . |
| CTC2 | 6 | 33653537 | TG | T | 0.367 | NM_002224 | ITPR3 | frameshift_deleti  on | exon41 | c.5601delG | p.V1867fs | . | . |
| CTC2 | 6 | 35392149 | AG | A | 0.259 | NM_001171820 | PPARD | frameshift_deleti  on | exon5 | c.378delG | p.K126fs | . | . |
| CTC2 | 6 | 35747207 | C | CA | 1 | NM_207409 | CLPSL2 | frameshift_inserti  on | exon3 | c.283_284insA | p.R95fs | . | . |
| CTC2 | 6 | 36181683 | AC | A | 0.972 | NM_015695 | BRPF3 | frameshift_deleti  on | exon8 | c.2510delC | p.T837fs | . | . |
| CTC2 | 6 | 36447376 | CA | C | 0.31 | NM_001286580 | KCTD20 | frameshift_deleti  on | exon4 | c.112delA | p.K38fs | . | . |
| CTC2 | 6 | 36485500 | GC | G | 0.364 | NM_007271 | STK38 | frameshift_deleti  on | exon6 | c.507delG | p.L169fs | . | . |
| CTC2 | 6 | 36683364 | ATGC | A | 0.769 | NM_001257357 | RAB44 | nonframeshift_de  letion | exon4 | c.429_431del | p.143_144del | . | . |
| CTC2 | 6 | 36710087 | TG | T | 0.304 | NM_020939 | CPNE5 | frameshift_deleti  on | exon21 | c.1739delC | p.P580fs | . | . |
| CTC2 | 6 | 36724043 | CT | C | 0.347 | NM_020939 | CPNE5 | frameshift_deleti  on | exon13 | c.887delA | p.K296fs | . | . |
| CTC2 | 6 | 38723854 | A | AG | 0.304 | NM_001206927 | DNAH8 | frameshift_inserti  on | exon10 | c.1515dupG | p.K505fs | . | . |
| CTC2 | 6 | 38862540 | AT | A | 0.314 | NM_001206927 | DNAH8 | frameshift_deleti  on | exon59 | c.8648delT | p.I2883fs | . | . |
| CTC2 | 6 | 38883019 | AT | A | 0.538 | NM_001206927 | DNAH8 | frameshift_deleti  on | exon68 | c.10007delT | p.I3336fs | . | . |
| CTC2 | 6 | 40400211 | AG | A | 0.3 | NM_020737 | LRFN2 | frameshift_deleti  on | exon2 | c.641delC | p.P214fs | . | . |
| CTC2 | 6 | 41553184 | AAG | A | 0.667 | NM_001012426 | FOXP4 | frameshift_deleti  on | exon5 | c.440_441del | p.K147fs | . | . |
| CTC2 | 6 | 41553185 | AG | A | 0.994 | NM_001012426 | FOXP4 | frameshift_deleti  on | exon5 | c.441delG | p.K147fs | . | . |
| CTC2 | 6 | 41621168 | TCTG | T | 0.182 | NM_005586 | MDFI | nonframeshift_de  letion | exon5 | c.597_599del | p.199_200del | . | . |
| CTC2 | 6 | 41738600 | TG | T | 0.345 | NM_006653 | FRS3 | frameshift_deleti  on | exon7 | c.1235delC | p.P412fs | . | . |
| CTC2 | 6 | 42075175 | C | CT | 0.25 | NM_001164446 | C6orf132 | frameshift_inserti  on | exon4 | c.474dupA | p.G159fs | . | . |
| CTC2 | 6 | 42224775 | GC | G | 0.5 | NM_033502 | TRERF1 | frameshift_deleti  on | exon11 | c.2401delG | p.A801fs | . | . |
| CTC2 | 6 | 42619791 | CA | C | 0.4 | NM_015255 | UBR2 | frameshift_deleti  on | exon24 | c.2597delA | p.Q866fs | . | . |
| CTC2 | 6 | 42936681 | GC | G | 0.5 | NM_000287 | PEX6 | frameshift_deleti  on | exon6 | c.1409delG | p.G470fs | . | . |
| CTC2 | 6 | 43019436 | CA | C | 0.944 | NM_001168370 | CUL7 | frameshift_deleti  on | exon3 | c.897delT | p.F299fs | . | . |
| CTC2 | 6 | 43251216 | TC | T | 0.909 | NM_032538 | TTBK1 | frameshift_deleti  on | exon14 | c.2739delC | p.V913fs | . | . |
| CTC2 | 6 | 43307391 | G | GC | 0.308 | NM_014345 | ZNF318 | frameshift_inserti  on | exon10 | c.4344_4345ins  G | p.P1449fs | . | . |
| CTC2 | 6 | 43418647 | TC | T | 0.525 | NM_001286655 | DLK2 | frameshift_deleti  on | exon6 | c.670delG | p.D224fs | . | . |
| CTC2 | 6 | 43746259 | CA | C | 0.886 | NM_001025366 | VEGFA | frameshift_deleti  on | exon4 | c.919delA | p.K307fs | . | . |
| CTC2 | 6 | 44224124 | CG | C | 0.815 | NM_001286509 | SLC35B2 | frameshift_deleti  on | exon3 | c.299delC | p.P100fs | . | . |
| CTC2 | 6 | 44227796 | TG | T | 0.401 | NM_004556 | NFKBIE | frameshift_deleti  on | exon5 | c.1420delC | p.Q474fs | . | . |
| CTC2 | 6 | 44268961 | TG | T | 0.978 | NM_020745 | AARS2 | frameshift_deleti  on | exon21 | c.2724delC | p.P908fs | . | . |
| CTC2 | 6 | 44371674 | GT | G | 0.348 | NM_001253 | CDC5L | frameshift_deleti  on | exon6 | c.669delT | p.G223fs | . | . |
| CTC2 | 6 | 49754461 | AT | A | 0.596 | NM_138733 | PGK2 | stopgain | exon1 | c.439delA | p.I147X | . | . |
| CTC2 | 6 | 50786631 | TG | T | 0.31 | NM_003221 | TFAP2B | frameshift_deleti on | exon1 | c.28delG | p.A10fs | . | . |
| CTC2 | 6 | 53372278 | CT | C | 0.243 | NM_001197115 | GCLC | frameshift_deleti  on | exon8 | c.969delA | p.E323fs | . | . |
| CTC2 | 6 | 53784417 | AT | A | 0.308 | NM_018214 | LRRC1 | frameshift_deleti  on | exon12 | c.1229delT | p.I410fs | . | . |
| CTC2 | 6 | 55620376 | CA | C | 0.714 | NM_021073 | BMP5 | stopgain | exon7 | c.1319delT | p.L440X | . | . |
| CTC2 | 6 | 56481008 | CT | C | 0.84 | NM_001723 | DST | frameshift_deleti on | exon24 | c.7256delA | p.K2419fs | . | . |
| CTC2 | 6 | 56492892 | CA | C | 0.235 | NM_001723 | DST | frameshift_deleti  on | exon19 | c.2931delT | p.D977fs | . | . |
| CTC2 | 6 | 62688026 | TC | T | 0.692 | NM_152688 | KHDRBS2 | frameshift_deleti  on | exon4 | c.427delG | p.E143fs | . | . |
| CTC2 | 6 | 65146148 | TA | T | 0.468 | NM_001142800 | EYS | frameshift_deleti  on | exon28 | c.5845delT | p.Y1949fs | . | . |
| CTC2 | 6 | 65707480 | GC | G | 0.484 | NM_001142800 | EYS | frameshift_deleti  on | exon14 | c.2253delG | p.L751fs | . | . |
| CTC2 | 6 | 70386155 | CA | C | 0.39 | NM_018368 | LMBRD1 | stopgain | exon16 | c.1517delT | p.L506X | . | . |
| CTC2 | 6 | 71567864 | CA | C | 0.294 | NM_001281439 | SMAP1 | frameshift_deleti on | exon9 | c.1121delA | p.Q374fs | . | . |
| CTC2 | 6 | 72011443 | CA | C | 0.596 | NM_024576 | OGFRL1 | frameshift_deleti  on | exon7 | c.1048delA | p.K350fs | . | . |
| CTC2 | 6 | 73713656 | AT | A | 0.282 | NM_001160130 | KCNQ5 | frameshift_deleti  on | exon2 | c.425delT | p.I142fs | . | . |
| CTC2 | 6 | 73843182 | GC | G | 0.333 | NM_001160130 | KCNQ5 | frameshift_deleti  on | exon9 | c.1260delC | p.S420fs | . | . |
| CTC2 | 6 | 74079390 | C | CG | 0.231 | NM_001080507 | OOEP | frameshift_inserti  on | exon1 | c.125dupC | p.P42fs | . | . |
| CTC2 | 6 | 74229690 | CT | C | 0.515 | NM_001402 | EEF1A1 | frameshift_deleti  on | exon2 | c.59delA | p.K20fs | . | . |
| CTC2 | 6 | 76595800 | AC | A | 1 | NM_004999 | MYO6 | frameshift_deleti  on | exon24 | c.2497delC | p.H833fs | . | . |
| CTC2 | 6 | 79752586 | CA | C | 0.345 | NM_017934 | PHIP | frameshift_deleti  on | exon7 | c.573delT | p.F191fs | . | . |

| CTC2 | 6 | 80715677 | T | TA | 0.636 | NM_001166691 | TTK | frameshift_inserti  on | exon2 | c.118dupA | p.N39fs | . | . |
| --- | --- | --- | --- | --- | --- | --- | --- | --- | --- | --- | --- | --- | --- |
| CTC2 | 6 | 84233724 | GT | G | 0.571 | NM_153362 | PRSS35 | stopgain | exon2 | c.565delT | p.L189X | . | . |
| CTC2 | 6 | 84234129 | GT | G | 0.278 | NM_153362 | PRSS35 | frameshift_deleti on | exon2 | c.970delT | p.F324fs | . | . |
| CTC2 | 6 | 84304192 | CA | C | 0.217 | NM_001256718 | SNAP91 | frameshift_deleti  on | exon15 | c.975delT | p.F325fs | . | . |
| CTC2 | 6 | 84884542 | CT | C | 1 | NM_001286206 | KIAA1009 | frameshift_deleti  on | exon15 | c.1700delA | p.K567fs | . | . |
| CTC2 | 6 | 87968206 | TA | T | 0.5 | NM_015021 | ZNF292 | frameshift_deleti  on | exon8 | c.4860delA | p.L1620fs | . | . |
| CTC2 | 6 | 87994471 | TG | T | 0.556 | NM_198568 | GJB7 | stopgain | exon3 | c.159delC | p.C53X | . | . |
| CTC2 | 6 | 88187189 | CT | C | 0.953 | NM_001168398 | SLC35A1 | frameshift_deleti on | exon2 | c.127delT | p.F43fs | . | . |
| CTC2 | 6 | 88326058 | TA | T | 1 | NM_001197259 | ORC3 | frameshift_deleti  on | exon8 | c.472delA | p.K158fs | . | . |
| CTC2 | 6 | 90402853 | CT | C | 1 | NM_014611 | MDN1 | frameshift_deleti  on | exon63 | c.9895delA | p.R3299fs | . | . |
| CTC2 | 6 | 90455227 | AT | A | 0.917 | NM_014611 | MDN1 | frameshift_deleti  on | exon28 | c.4022delA | p.N1341fs | . | . |
| CTC2 | 6 | 90573965 | AC | A | 0.216 | NM_001137667 | CASP8AP2 | frameshift_deleti  on | exon7 | c.2538delC | p.D846fs | . | . |
| CTC2 | 6 | 90577003 | GA | G | 1 | NM_001137667 | CASP8AP2 | frameshift_deleti  on | exon8 | c.3995delA | p.E1332fs | . | . |
| CTC2 | 6 | 90642319 | TC | T | 0.433 | NM_001170794 | BACH2 | frameshift_deleti  on | exon7 | c.2333delG | p.G778fs | . | . |
| CTC2 | 6 | 97053935 | CT | C | 0.462 | NM_001170807 | FHL5 | frameshift_deleti  on | exon4 | c.493delT | p.F165fs | . | . |
| CTC2 | 6 | 99817666 | A | AT | 0.727 | NM_017421 | COQ3 | frameshift_inserti  on | exon7 | c.919dupA | p.M307fs | . | . |
| CTC2 | 6 | 99848797 | CT | C | 0.359 | NM_015491 | PNISR | frameshift_deleti  on | exon11 | c.2036delA | p.K679fs | . | . |
| CTC2 | 6 | 99893878 | TA | T | 0.417 | NM_001080481 | USP45 | stopgain | exon14 | c.1769delT | p.L590X | . | . |
| CTC2 | 6 | 100841614 | GA | G | 0.681 | NM_005068 | SIM1 | frameshift_deleti on | exon10 | c.1318delT | p.S440fs | . | . |
| CTC2 | 6 | 109764211 | AG | A | 0.238 | NM_003080 | SMPD2 | frameshift_deleti  on | exon8 | c.657delG | p.K219fs | . | . |
| CTC2 | 6 | 109787523 | T | TG | 0.2 | NM_014797 | ZBTB24 | frameshift_inserti  on | exon7 | c.1624_1625ins  C | p.Y542fs | . | . |
| CTC2 | 6 | 110428292 | CT | C | 0.392 | NM_001024936 | WASF1 | frameshift_deleti  on | exon5 | c.527delA | p.K176fs | . | . |
| CTC2 | 6 | 110531959 | GA | G | 0.353 | NM_015891 | CDC40 | frameshift_deleti  on | exon6 | c.681delA | p.G227fs | . | . |
| CTC2 | 6 | 110935779 | TCTG | T | 0.456 | NM_015076 | CDK19 | nonframeshift_de  letion | exon13 | c.1465_1467del | p.489_489del | . | . |
| CTC2 | 6 | 110953294 | CA | C | 0.7 | NM_015076 | CDK19 | frameshift_deleti  on | exon6 | c.584delT | p.V195fs | . | . |
| CTC2 | 6 | 111693957 | AT | A | 1 | NM_002912 | REV3L | frameshift_deleti  on | exon14 | c.5600delA | p.N1867fs | . | . |
| CTC2 | 6 | 111912969 | AG | A | 0.27 | NM_001164281 | TRAF3IP2 | frameshift_deleti  on | exon2 | c.320delC | p.P107fs | . | . |
| CTC2 | 6 | 112407770 | A | AT | 0.571 | NM_016262 | TUBE1 | frameshift_inserti  on | exon3 | c.140dupA | p.N47fs | . | . |
| CTC2 | 6 | 112499328 | AT | A | 0.354 | NM_001105206 | LAMA4 | frameshift_deleti  on | exon10 | c.1183delA | p.I395fs | . | . |
| CTC2 | 6 | 116574408 | AC | A | 0.216 | NM_021648 | TSPYL4 | frameshift_deleti  on | exon1 | c.763delG | p.V255fs | . | . |
| CTC2 | 6 | 116757352 | TG | T | 0.5 | NM_001080976 | DSE | frameshift_deleti  on | exon6 | c.1722delG | p.L574fs | . | . |
| CTC2 | 6 | 116837117 | AC | A | 1 | NM_153711 | FAM26E | frameshift_deleti  on | exon2 | c.896delC | p.T299fs | . | . |
| CTC2 | 6 | 119238772 | CT | C | 0.296 | NM_017696 | MCM9 | frameshift_deleti  on | exon4 | c.857delA | p.K286fs | . | . |
| CTC2 | 6 | 119252681 | CA | C | 0.4 | NM_017696 | MCM9 | frameshift_deleti  on | exon1 | c.207delT | p.F69fs | . | . |
| CTC2 | 6 | 119341162 | TG | T | 0.571 | NM_001100411 | FAM184A | frameshift_deleti  on | exon4 | c.952delC | p.Q318fs | . | . |
| CTC2 | 6 | 121768170 | TG | T | 0.429 | NM_000165 | GJA1 | frameshift_deleti  on | exon2 | c.178delG | p.G60fs | . | . |
| CTC2 | 6 | 123699033 | CT | C | 0.432 | NM_001251987 | TRDN | frameshift_deleti  on | exon17 | c.1199delA | p.K400fs | . | . |
| CTC2 | 6 | 129637084 | G | GA | 0.35 | NM_000426 | LAMA2 | frameshift_inserti  on | exon26 | c.3914dupA | p.E1305fs | . | . |
| CTC2 | 6 | 129774200 | GT | G | 0.7 | NM_000426 | LAMA2 | frameshift_deleti  on | exon46 | c.6498delT | p.S2166fs | . | . |
| CTC2 | 6 | 130404840 | C | CT | 1 | NM_001007102 | L3MBTL3 | frameshift_inserti  on | exon14 | c.1323dupT | p.A441fs | . | . |
| CTC2 | 6 | 133767844 | GA | G | 0.789 | NM_004100 | EYA4 | frameshift_deleti  on | exon4 | c.161delA | p.E54fs | . | . |
| CTC2 | 6 | 134303970 | AT | A | 0.851 | NM_001253676 | TBPL1 | frameshift_deleti  on | exon4 | c.235delT | p.F79fs | . | . |
| CTC2 | 6 | 134349812 | TG | T | 0.283 | NM_145176 | SLC2A12 | frameshift_deleti  on | exon2 | c.1150delC | p.Q384fs | . | . |
| CTC2 | 6 | 135749862 | TA | T | 0.357 | NM_001134830 | AHI1 | frameshift_deleti  on | exon17 | c.2527delT | p.Y843fs | . | . |
| CTC2 | 6 | 136582575 | CT | C | 0.455 | NM_001077440 | BCLAF1 | frameshift_deleti  on | exon11 | c.2431delA | p.R811fs | . | . |
| CTC2 | 6 | 137479557 | GA | G | 0.25 | NM_052962 | IL22RA2 | frameshift_deleti  on | exon3 | c.123delT | p.F41fs | . | . |
| CTC2 | 6 | 138619809 | G | GA | 0.433 | NM_020340 | KIAA1244 | frameshift_inserti  on | exon22 | c.3716dupA | p.E1239fs | . | . |
| CTC2 | 6 | 138655867 | TC | T | 0.293 | NM_020340 | KIAA1244 | frameshift_deleti  on | exon33 | c.5885delC | p.S1962fs | . | . |
| CTC2 | 6 | 138727138 | C | CA | 0.562 | NM_014320 | HEBP2 | frameshift_inserti  on | exon3 | c.270dupA | p.T90fs | . | . |
| CTC2 | 6 | 142741064 | TG | T | 0.647 | NM_001032394 | GPR126 | frameshift_deleti  on | exon21 | c.3059delG | p.C1020fs | . | . |
| CTC2 | 6 | 143093365 | AC | A | 0.36 | NM_006734 | HIVEP2 | frameshift_deleti  on | exon5 | c.2510delG | p.C837fs | . | . |
| CTC2 | 6 | 144508266 | A | AG | 0.263 | NM_003764 | STX11 | frameshift_inserti  on | exon2 | c.502_503insG | p.M168fs | . | . |
| CTC2 | 6 | 151673968 | CA | C | 0.386 | NM_144497 | AKAP12 | frameshift_deleti  on | exon2 | c.4149delA | p.A1383fs | . | . |
| CTC2 | 6 | 151936619 | CA | C | 0.25 | NM_025059 | CCDC170 | frameshift_deleti  on | exon10 | c.1753delA | p.K585fs | . | . |
| CTC2 | 6 | 153296408 | GA | G | 0.358 | NM_001142522 | FBXO5 | frameshift_deleti  on | exon2 | c.313delT | p.S105fs | . | . |
| CTC2 | 6 | 155597070 | CT | C | 0.25 | NM_001001346 | CLDN20 | frameshift_deleti  on | exon2 | c.218delT | p.L73fs | . | . |
| CTC2 | 6 | 155752052 | TA | T | 0.239 | NM_015718 | NOX3 | stopgain | exon8 | c.815delT | p.L272X | . | . |
| CTC2 | 6 | 160211645 | GGTT | G | 0.623 | NM_014161 | MRPL18 | nonframeshift_de letion | exon1 | c.27_29del | p.9_10del | . | . |

| CTC2 | 6 | 165848750 | GT | G | 0.393 | NM_001130690 | PDE10A | frameshift_deleti  on | exon6 | c.511delA | p.T171fs | . | . |
| --- | --- | --- | --- | --- | --- | --- | --- | --- | --- | --- | --- | --- | --- |
| CTC2 | 6 | 167550080 | TA | T | 0.7 | NM_004367 | CCR6 | frameshift_deleti  on | exon3 | c.363delA | p.L121fs | . | . |
| CTC2 | 6 | 168315857 | TG | T | 0.368 | NM_001207008 | MLLT4 | frameshift_deleti  on | exon17 | c.2241delG | p.L747fs | . | . |
| CTC2 | 6 | 168708520 | TG | T | 1 | NM_001286350 | DACT2 | frameshift_deleti  on | exon3 | c.1406delC | p.A469fs | . | . |
| CTC2 | 6 | 168708700 | CG | C | 0.652 | NM_001286350 | DACT2 | frameshift_deleti  on | exon3 | c.1226delC | p.P409fs | . | . |
| CTC2 | 6 | 169625332 | TC | T | 1 | NM_003247 | THBS2 | frameshift_deleti  on | exon18 | c.2680delG | p.D894fs | . | . |
| CTC2 | 6 | 169857702 | GT | G | 0.228 | NM_001202550 | WDR27 | frameshift_deleti  on | exon22 | c.2169delA | p.K723fs | . | . |
| CTC2 | 6 | 170871013 | A | ACAG | 0.959 | NM_001172085 | TBP | nonframeshift_in  sertion | exon2 | c.129_130insCA  G | p.Q43delinsQQ | . | . |
| CTC2 | 6 | 170871034 | G | GCAA | 0.836 | NM_001172085 | TBP | nonframeshift_in  sertion | exon2 | c.150_151insCA  A | p.Q50delinsQQ | . | . |
| CTC2 | 6 | 170871037 | GCAA | G | 0.883 | NM_001172085 | TBP | nonframeshift_de  letion | exon2 | c.154_156del | p.52_52del | . | . |
| CTC2 | 6 | 170871046 | ACAG | A | 1 | NM_001172085 | TBP | nonframeshift_de  letion | exon2 | c.163_165del | p.55_55del | . | . |
| CTC2 | 6 | 170871046 | ACAGCA  GCAGCA | A | 1 | NM_001172085 | TBP | nonframeshift_de  letion | exon2 | c.163_174del | p.55_58del | . | . |
| CTC2 | 7 | 720280 | CG | C | 0.672 | NM_001164758 | PRKAR1B | frameshift_deleti  on | exon3 | c.260delC | p.P87fs | . | . |
| CTC2 | 7 | 1527437 | GCT | G | 1 | NM_001080453 | INTS1 | frameshift_deleti  on | exon19 | c.2473_2474del | p.S825fs | . | . |
| CTC2 | 7 | 2566502 | GC | G | 0.231 | NM_001040167 | LFNG | frameshift_deleti  on | exon7 | c.1021delC | p.R341fs | . | . |
| CTC2 | 7 | 4008946 | CG | C | 0.636 | NM_152744 | SDK1 | frameshift_deleti  on | exon11 | c.1605delG | p.S535fs | . | . |
| CTC2 | 7 | 4167066 | AT | A | 0.357 | NM_152744 | SDK1 | frameshift_deleti  on | exon26 | c.3878delT | p.I1293fs | . | . |
| CTC2 | 7 | 5103661 | GA | G | 0.438 | NM_021163 | RBAK | frameshift_deleti  on | exon5 | c.575delA | p.E192fs | . | . |
| CTC2 | 7 | 5391572 | GC | G | 0.24 | NM_001080495 | TNRC18 | frameshift_deleti  on | exon17 | c.5347delG | p.A1783fs | . | . |
| CTC2 | 7 | 6026885 | TC | T | 0.359 | NM_000535 | PMS2 | frameshift_deleti  on | exon11 | c.1510delG | p.E504fs | . | . |
| CTC2 | 7 | 6189654 | C | CTCGT  CA | 0.366 | NM_032172 | USP42 | nonframeshift_in  sertion | exon13 | c.1827_1828ins  TCGTCA | p.A609delinsAS  S | . | . |
| CTC2 | 7 | 7550730 | CT | C | 0.6 | NM_001037763 | COL28A1 | frameshift_deleti  on | exon9 | c.918delA | p.K306fs | . | . |
| CTC2 | 7 | 7613109 | CAA | C | 0.167 | NM_019005 | MIOS | frameshift_deleti  on | exon4 | c.1004_1005del | p.Q335fs | . | . |
| CTC2 | 7 | 11415499 | CT | C | 0.571 | NM_015204 | THSD7A | frameshift_deleti  on | exon27 | c.4895delA | p.K1632fs | . | . |
| CTC2 | 7 | 15725797 | ATGG | A | 1 | NM_005924 | MEOX2 | nonframeshift_de  letion | exon1 | c.228_230del | p.76_77del | . | . |
| CTC2 | 7 | 18067389 | AT | A | 0.261 | NM_175886 | PRPS1L1 | frameshift_deleti  on | exon1 | c.16delA | p.I6fs | . | . |
| CTC2 | 7 | 21640371 | AG | A | 0.25 | NM_001277115 | DNAH11 | frameshift_deleti  on | exon16 | c.3079delG | p.V1027fs | . | . |
| CTC2 | 7 | 21781578 | TC | T | 0.561 | NM_001277115 | DNAH11 | frameshift_deleti  on | exon49 | c.7949delC | p.S2650fs | . | . |
| CTC2 | 7 | 21827054 | CAA | C | 0.51 | NM_001277115 | DNAH11 | frameshift_deleti  on | exon60 | c.9778_9779del | p.K3260fs | . | . |
| CTC2 | 7 | 23239992 | AT | A | 0.667 | NM_007342 | NUPL2 | frameshift_deleti  on | exon7 | c.901delT | p.F301fs | . | . |
| CTC2 | 7 | 23775187 | GA | G | 0.5 | NM_001260504 | STK31 | frameshift_deleti  on | exon7 | c.446delA | p.E149fs | . | . |
| CTC2 | 7 | 24758763 | TG | T | 0.267 | NM_001127453 | DFNA5 | frameshift_deleti  on | exon4 | c.478delC | p.Q160fs | . | . |
| CTC2 | 7 | 25207951 | TG | T | 0.25 | NM_138811 | C7orf31 | frameshift_deleti  on | exon3 | c.267delC | p.G89fs | . | . |
| CTC2 | 7 | 25267939 | AT | A | 0.571 | NM_022150 | NPVF | frameshift_deleti  on | exon1 | c.119delA | p.N40fs | . | . |
| CTC2 | 7 | 28858734 | TCC | T | 0.17 | NM_001011666 | CREB5 | frameshift_deleti  on | exon7 | c.949_950del | p.P317fs | . | . |
| CTC2 | 7 | 29105694 | G | GA | 1 | NM_019029 | CPVL | frameshift_inserti  on | exon10 | c.907dupT | p.S303fs | . | . |
| CTC2 | 7 | 31124969 | TG | T | 0.22 | NM_001199637 | ADCYAP1R1 | frameshift_deleti  on | exon7 | c.519delG | p.V173fs | . | . |
| CTC2 | 7 | 31126572 | GT | G | 1 | NM_001199637 | ADCYAP1R1 | frameshift_deleti  on | exon9 | c.777delT | p.C259fs | . | . |
| CTC2 | 7 | 31877528 | GA | G | 0.234 | NM_001191056 | PDE1C | frameshift_deleti  on | exon10 | c.1037delT | p.F346fs | . | . |
| CTC2 | 7 | 33055422 | AG | A | 0.515 | NM_001002010 | NT5C3A | frameshift_deleti  on | exon8 | c.768delC | p.A256fs | . | . |
| CTC2 | 7 | 33066489 | GT | G | 0.211 | NM_001002010 | NT5C3A | frameshift_deleti  on | exon2 | c.191delA | p.N64fs | . | . |
| CTC2 | 7 | 33185924 | TG | T | 0.556 | NM_001033604 | BBS9 | frameshift_deleti  on | exon2 | c.61delG | p.D21fs | . | . |
| CTC2 | 7 | 34118772 | AC | A | 0.606 | NM_133468 | BMPER | frameshift_deleti  on | exon13 | c.1383delC | p.Y461fs | . | . |
| CTC2 | 7 | 34867055 | CT | C | 1 | NM_207172 | NPSR1 | frameshift_deleti  on | exon5 | c.522delT | p.S174fs | . | . |
| CTC2 | 7 | 36445969 | CA | C | 0.37 | NM_001284301 | ANLN | frameshift_deleti  on | exon4 | c.668delA | p.Q223fs | . | . |
| CTC2 | 7 | 36660402 | GT | G | 0.781 | NM_001177507 | AOAH | frameshift_deleti  on | exon8 | c.590delA | p.N197fs | . | . |
| CTC2 | 7 | 43546810 | AG | A | 0.236 | NM_001287059 | HECW1 | frameshift_deleti  on | exon20 | c.3605delG | p.R1202fs | . | . |
| CTC2 | 7 | 44114002 | GA | G | 0.493 | NM_001284330 | POLM | frameshift_deleti  on | exon6 | c.825delT | p.L275fs | . | . |
| CTC2 | 7 | 44555413 | A | AC | 0.474 | NM_001101648 | NPC1L1 | frameshift_inserti  on | exon18 | c.3784_3785ins  G | p.L1262fs | . | . |
| CTC2 | 7 | 44573423 | TC | T | 1 | NM_001101648 | NPC1L1 | frameshift_deleti  on | exon7 | c.2195delG | p.R732fs | . | . |
| CTC2 | 7 | 44621372 | A | AG | 0.308 | NM_182547 | TMED4 | frameshift_inserti  on | exon2 | c.210dupC | p.S71fs | . | . |
| CTC2 | 7 | 45122669 | GC | G | 0.762 | NM_001146334 | NACAD | frameshift_deleti  on | exon2 | c.3109delG | p.A1037fs | . | . |
| CTC2 | 7 | 45145057 | TC | T | 0.741 | NM_001261834 | TBRG4 | frameshift_deleti  on | exon3 | c.750delG | p.M250fs | . | . |
| CTC2 | 7 | 50512975 | CA | C | 0.311 | NM_001287495 | FIGNL1 | frameshift_deleti  on | exon2 | c.2010delT | p.F670fs | . | . |
| CTC2 | 7 | 51098567 | GTCT | G | 0.189 | NM_015198 | COBL | nonframeshift_de  letion | exon9 | c.1443_1445del | p.481_482del | . | . |
| CTC2 | 7 | 51150793 | TC | T | 0.435 | NM_001287436 | COBL | frameshift_deleti  on | exon8 | c.1228delG | p.E410fs | . | . |
| CTC2 | 7 | 64167521 | CT | C | 0.488 | NM_001282360 | ZNF107 | frameshift_deleti  on | exon3 | c.951delT | p.A317fs | . | . |

| CTC2 | 7 | 64439593 | CA | C | 0.268 | NM_015852 | ZNF117 | frameshift_deleti  on | exon4 | c.355delT | p.C119fs | . | . |
| --- | --- | --- | --- | --- | --- | --- | --- | --- | --- | --- | --- | --- | --- |
| CTC2 | 7 | 65419353 | T | TTTA | 0.667 | NM_001284342 | VKORC1L1 | nonframeshift_in  sertion | exon2 | c.487_488insTT  A | p.F163delinsFI | . | . |
| CTC2 | 7 | 65419353 | TTTA | T | 0.667 | NM_001284342 | VKORC1L1 | nonframeshift_de  letion | exon2 | c.488_490del | p.163_164del | . | . |
| CTC2 | 7 | 65419360 | T | TTAC | 0.33 | NM_001284342 | VKORC1L1 | nonframeshift_in  sertion | exon2 | c.494_495insTA  C | p.I165delinsIT | . | . |
| CTC2 | 7 | 70255576 | GCCA | G | 0.24 | NM_001127231 | AUTS2 | nonframeshift_de  letion | exon18 | c.3303_3305del | p.1101_1102de  l | . | . |
| CTC2 | 7 | 72856736 | AT | A | 0.301 | NM_032408 | BAZ1B | frameshift_deleti  on | exon19 | c.4241delA | p.N1414fs | . | . |
| CTC2 | 7 | 72985117 | TG | T | 0.896 | NM_012453 | TBL2 | frameshift_deleti  on | exon7 | c.1063delC | p.H355fs | . | . |
| CTC2 | 7 | 73011211 | CG | C | 0.237 | NM_032951 | MLXIPL | frameshift_deleti  on | exon10 | c.1654delC | p.R552fs | . | . |
| CTC2 | 7 | 73511427 | G | GT | 0.291 | NM_001204426 | LIMK1 | frameshift_inserti  on | exon3 | c.208dupT | p.K69fs | . | . |
| CTC2 | 7 | 73535316 | CA | C | 0.279 | NM_001204426 | LIMK1 | frameshift_deleti  on | exon14 | c.1617delA | p.P539fs | . | . |
| CTC2 | 7 | 73664104 | CA | C | 0.314 | NM_002914 | RFC2 | frameshift_deleti  on | exon3 | c.189delT | p.F63fs | . | . |
| CTC2 | 7 | 75191382 | AT | A | 0.421 | NM_001243198 | HIP1 | frameshift_deleti  on | exon12 | c.1112delA | p.D371fs | . | . |
| CTC2 | 7 | 75610381 | AAC | A | 0.174 | NM_000941 | POR | frameshift_deleti  on | exon6 | c.533_534del | p.N178fs | . | . |
| CTC2 | 7 | 80439975 | CT | C | 0.75 | NM_006379 | SEMA3C | frameshift_deleti  on | exon6 | c.489delA | p.K163fs | . | . |
| CTC2 | 7 | 81598206 | CA | C | 0.778 | NM_000722 | CACNA2D1 | frameshift_deleti  on | exon29 | c.2391delT | p.P797fs | . | . |
| CTC2 | 7 | 82583630 | TG | T | 1 | NM_014510 | PCLO | frameshift_deleti  on | exon5 | c.6638delC | p.P2213fs | . | . |
| CTC2 | 7 | 82763828 | TC | T | 1 | NM_014510 | PCLO | frameshift_deleti  on | exon3 | c.3037delG | p.E1013fs | . | . |
| CTC2 | 7 | 86571382 | AGTG | A | 0.326 | NM_152748 | KIAA1324L | nonframeshift_de  letion | exon4 | c.189_191del | p.63_64del | . | . |
| CTC2 | 7 | 86848742 | CT | C | 1 | NM_024315 | TMEM243 | frameshift_deleti  on | exon1 | c.77delA | p.K26fs | . | . |
| CTC2 | 7 | 87179239 | AT | A | 0.26 | NM_000927 | ABCB1 | frameshift_deleti  on | exon14 | c.1481delA | p.N494fs | . | . |
| CTC2 | 7 | 87180053 | CT | C | 0.316 | NM_000927 | ABCB1 | frameshift_deleti  on | exon11 | c.1100delA | p.K367fs | . | . |
| CTC2 | 7 | 87792481 | TG | T | 0.75 | NM_004194 | ADAM22 | frameshift_deleti  on | exon23 | c.2063delG | p.C688fs | . | . |
| CTC2 | 7 | 91974320 | TA | T | 0.171 | NM_019004 | ANKIB1 | frameshift_deleti  on | exon7 | c.1026delA | p.V342fs | . | . |
| CTC2 | 7 | 92355021 | CA | C | 0.952 | NM_001145306 | CDK6 | frameshift_deleti  on | exon4 | c.455delT | p.L152fs | . | . |
| CTC2 | 7 | 92730800 | AT | A | 0.382 | NM_001193307 | SAMD9 | frameshift_deleti  on | exon2 | c.4610delA | p.N1537fs | . | . |
| CTC2 | 7 | 92733216 | AT | A | 1 | NM_001193307 | SAMD9 | frameshift_deleti  on | exon2 | c.2194delA | p.I732fs | . | . |
| CTC2 | 7 | 92762819 | T | TA | 0.684 | NM_152703 | SAMD9L | frameshift_inserti  on | exon5 | c.2465dupT | p.L822fs | . | . |
| CTC2 | 7 | 94029581 | GC | G | 1 | NM_000089 | COL1A2 | frameshift_deleti  on | exon5 | c.207delC | p.G69fs | . | . |
| CTC2 | 7 | 94232672 | A | AT | 0.29 | NM_001099400 | SGCE | frameshift_inserti  on | exon6 | c.754dupA | p.M252fs | . | . |
| CTC2 | 7 | 94539559 | CA | C | 0.571 | NM_001166161 | PPP1R9A | frameshift_deleti  on | exon1 | c.135delA | p.T45fs | . | . |
| CTC2 | 7 | 94917868 | TC | T | 0.442 | NM_001166163 | PPP1R9A | frameshift_deleti  on | exon13 | c.2899delC | p.P967fs | . | . |
| CTC2 | 7 | 96747051 | GTTT | G | 0.857 | NM_020186 | ACN9 | nonframeshift_de  letion | exon1 | c.17_19del | p.6_7del | . | . |
| CTC2 | 7 | 98507840 | T | TC | 0.323 | NM_001244580 | TRRAP | frameshift_inserti  on | exon15 | c.1512_1513ins  C | p.P504fs | . | . |
| CTC2 | 7 | 98519470 | TG | T | 0.478 | NM_001244580 | TRRAP | frameshift_deleti  on | exon21 | c.2718delG | p.L906fs | . | . |
| CTC2 | 7 | 99170886 | TG | T | 0.583 | NM_001009960 | ZNF655 | frameshift_deleti  on | exon3 | c.1156delG | p.G386fs | . | . |
| CTC2 | 7 | 99319947 | A | AT | 0.214 | NM_000765 | CYP3A7 | frameshift_inserti  on | exon3 | c.189dupA | p.C64fs | . | . |
| CTC2 | 7 | 99662111 | CAAT | C | 0.556 | NM_145914 | ZSCAN21 | nonframeshift_de  letion | exon4 | c.1294_1296del | p.432_432del | . | . |
| CTC2 | 7 | 99757606 | TG | T | 0.826 | NM_024637 | GAL3ST4 | frameshift_deleti  on | exon4 | c.1405delC | p.Q469fs | . | . |
| CTC2 | 7 | 99917278 | CT | C | 0.6 | NM_001004351 | SPDYE3 | frameshift_deleti  on | exon9 | c.1438delT | p.L480fs | . | . |
| CTC2 | 7 | 100304565 | TA | T | 1 | NM_005837 | POP7 | frameshift_deleti  on | exon2 | c.113delA | p.Y38fs | . | . |
| CTC2 | 7 | 100680793 | CA | C | 1 | NM_001040105 | MUC17 | frameshift_deleti  on | exon3 | c.6097delA | p.S2033fs | . | . |
| CTC2 | 7 | 100681235 | AC | A | 0.25 | NM_001040105 | MUC17 | frameshift_deleti  on | exon3 | c.6539delC | p.T2180fs | . | . |
| CTC2 | 7 | 100816611 | CG | C | 0.397 | NM_198571 | NAT16 | frameshift_deleti  on | exon3 | c.502delC | p.R168fs | . | . |
| CTC2 | 7 | 100839609 | CA | C | 0.7 | NM_178176 | MOGAT3 | frameshift_deleti  on | exon6 | c.729delT | p.F243fs | . | . |
| CTC2 | 7 | 100859567 | TC | T | 0.64 | NM_001084 | PLOD3 | frameshift_deleti  on | exon4 | c.378delG | p.L126fs | . | . |
| CTC2 | 7 | 102953526 | A | AG | 0.775 | NM_001129887 | DNAJC2 | frameshift_inserti  on | exon14 | c.1499dupC | p.P500fs | . | . |
| CTC2 | 7 | 103020963 | T | TG | 0.405 | NM_001167962 | SLC26A5 | frameshift_inserti  on | exon14 | c.1451_1452ins  C | p.E484fs | . | . |
| CTC2 | 7 | 103050927 | TA | T | 0.208 | NM_001167962 | SLC26A5 | frameshift_deleti  on | exon7 | c.639delT | p.F213fs | . | . |
| CTC2 | 7 | 103143499 | CT | C | 0.591 | NM_005045 | RELN | frameshift_deleti  on | exon52 | c.8452delA | p.R2818fs | . | . |
| CTC2 | 7 | 105254801 | AGAG | A | 0.207 | NM_138495 | ATXN7L1 | nonframeshift_de  letion | exon8 | c.1605_1607del | p.535_536del | . | . |
| CTC2 | 7 | 105902116 | TC | T | 0.226 | NM_005746 | NAMPT | frameshift_deleti  on | exon8 | c.1006delG | p.E336fs | . | . |
| CTC2 | 7 | 107423429 | GT | G | 0.308 | NM_000111 | SLC26A3 | frameshift_deleti  on | exon10 | c.1228delA | p.T410fs | . | . |
| CTC2 | 7 | 107866768 | AG | A | 0.625 | NM_001037132 | NRCAM | frameshift_deleti  on | exon6 | c.604delC | p.L202fs | . | . |
| CTC2 | 7 | 113518063 | AT | A | 0.507 | NM_002711 | PPP1R3A | frameshift_deleti  on | exon4 | c.3083delA | p.N1028fs | . | . |
| CTC2 | 7 | 113519043 | ATTC | A | 0.154 | NM_002711 | PPP1R3A | nonframeshift_de  letion | exon4 | c.2101_2103del | p.701_701del | . | . |
| CTC2 | 7 | 116960639 | CA | C | 1 | NM_003391 | WNT2 | frameshift_deleti  on | exon2 | c.291delT | p.F97fs | . | . |
| CTC2 | 7 | 117149115 | GA | G | 0.474 | NM_000492 | CFTR | frameshift_deleti  on | exon3 | c.193delA | p.K65fs | . | . |

| CTC2 | 7 | 117432604 | CA | C | 0.356 | NM_033427 | CTTNBP2 | frameshift_deleti  on | exon4 | c.645delT | p.A215fs | . | . |
| --- | --- | --- | --- | --- | --- | --- | --- | --- | --- | --- | --- | --- | --- |
| CTC2 | 7 | 120446609 | AT | A | 0.173 | NM_012338 | TSPAN12 | frameshift_deleti  on | exon7 | c.605delA | p.Y202fs | . | . |
| CTC2 | 7 | 121652901 | G | GA | 0.304 | NM_002851 | PTPRZ1 | frameshift_inserti  on | exon12 | c.3802dupA | p.E1267fs | . | . |
| CTC2 | 7 | 123092875 | AC | A | 0.7 | NM_001282855 | IQUB | frameshift_deleti  on | exon13 | c.2297delG | p.G766fs | . | . |
| CTC2 | 7 | 123600016 | GT | G | 0.298 | NM_001174044 | SPAM1 | frameshift_deleti  on | exon5 | c.1524delT | p.S508fs | . | . |
| CTC2 | 7 | 127235862 | CA | C | 0.71 | NM_020369 | FSCN3 | frameshift_deleti  on | exon2 | c.647delA | p.H216fs | . | . |
| CTC2 | 7 | 127544845 | TATCC | T | 0.838 | NM_014390 | SND1 | frameshift_deleti  on | exon14 | c.1501_1504del | p.I501fs | . | . |
| CTC2 | 7 | 127670048 | GGGT | G | 0.333 | NM_022143 | LRRC4 | nonframeshift_de  letion | exon2 | c.643_645del | p.215_215del | . | . |
| CTC2 | 7 | 127670049 | GGT | G | 0.952 | NM_022143 | LRRC4 | frameshift_deleti  on | exon2 | c.643_644del | p.T215fs | . | . |
| CTC2 | 7 | 127670051 | TGA | T | 0.951 | NM_022143 | LRRC4 | frameshift_deleti  on | exon2 | c.641_642del | p.L214fs | . | . |
| CTC2 | 7 | 127979368 | TG | T | 0.974 | NM_018077 | RBM28 | stopgain | exon3 | c.284delC | p.S95X | . | . |
| CTC2 | 7 | 128315821 | CA | C | 0.5 | NM_001012454 | FAM71F2 | frameshift_deleti on | exon2 | c.274delA | p.N92fs | . | . |
| CTC2 | 7 | 128482346 | GC | G | 0.375 | NM_001127487 | FLNC | frameshift_deleti  on | exon14 | c.2184delC | p.C728fs | . | . |
| CTC2 | 7 | 128492749 | G | GC | 0.336 | NM_001127487 | FLNC | frameshift_inserti  on | exon35 | c.5849dupC | p.A1950fs | . | . |
| CTC2 | 7 | 128494512 | CG | C | 0.929 | NM_001127487 | FLNC | frameshift_deleti  on | exon40 | c.6675delG | p.S2225fs | . | . |
| CTC2 | 7 | 128587351 | CACTCT  GCAGCC GCCCAC TCTGCG | C | 1 | NM_001098627 | IRF5 | nonframeshift_de letion | exon6 | c.502_531del | p.168_177del | . | . |
| CTC2 | 7 | 129756357 | GT | G | 0.571 | NM_014997 | KLHDC10 | frameshift_deleti  on | exon3 | c.327delT | p.G109fs | . | . |
| CTC2 | 7 | 130025754 | CA | C | 1 | NM_001868 | CPA1 | frameshift_deleti  on | exon9 | c.1063delA | p.K355fs | . | . |
| CTC2 | 7 | 134879733 | TG | T | 1 | NM_014149 | WDR91 | frameshift_deleti  on | exon9 | c.1347delC | p.S449fs | . | . |
| CTC2 | 7 | 136699978 | C | CT | 0.853 | NM_001006629 | CHRM2 | frameshift_inserti  on | exon2 | c.367dupT | p.Y122fs | . | . |
| CTC2 | 7 | 137612913 | CTGG | C | 1 | NM_001253775 | CREB3L2 | nonframeshift_de  letion | exon2 | c.299_301del | p.100_101del | . | . |
| CTC2 | 7 | 137792215 | GA | G | 0.333 | NM_001190906 | AKR1D1 | frameshift_deleti  on | exon6 | c.622delA | p.K208fs | . | . |
| CTC2 | 7 | 138719402 | CA | C | 0.4 | NM_080660 | ZC3HAV1L | frameshift_deleti  on | exon2 | c.387delT | p.H129fs | . | . |
| CTC2 | 7 | 139611102 | TA | T | 0.279 | NM_001061 | TBXAS1 | frameshift_deleti  on | exon4 | c.319delA | p.N107fs | . | . |
| CTC2 | 7 | 140453149 | CA | C | 0.25 | NM_004333 | BRAF | frameshift_deleti  on | exon15 | c.1785delT | p.F595fs | . | . |
| CTC2 | 7 | 141352612 | AC | A | 0.329 | NM_018238 | AGK | frameshift_deleti  on | exon16 | c.1158delC | p.D386fs | . | . |
| CTC2 | 7 | 142563326 | G | GC | 0.304 | NM_001280795 | EPHB6 | frameshift_inserti  on | exon4 | c.168dupC | p.C56fs | . | . |
| CTC2 | 7 | 142626646 | GC | G | 0.835 | NM_019841 | TRPV5 | frameshift_deleti  on | exon4 | c.363delG | p.L121fs | . | . |
| CTC2 | 7 | 142983890 | G | GT | 0.373 | NM_001282876 | TMEM139 | frameshift_inserti  on | exon3 | c.620dupT | p.V207fs | . | . |
| CTC2 | 7 | 143792399 | A | AT | 0.379 | NM_001004135 | OR2A12 | frameshift_inserti  on | exon1 | c.200dupT | p.I67fs | . | . |
| CTC2 | 7 | 148523622 | GC | G | 0.474 | NM_152998 | EZH2 | frameshift_deleti  on | exon7 | c.713delG | p.S238fs | . | . |
| CTC2 | 7 | 148523694 | TG | T | 0.381 | NM_152998 | EZH2 | frameshift_deleti  on | exon7 | c.641delC | p.P214fs | . | . |
| CTC2 | 7 | 150417345 | GA | G | 1 | NM_001199577 | GIMAP1-  GIMAP5 | frameshift_deleti  on | exon3 | c.254delA | p.D85fs | . | . |
| CTC2 | 7 | 150656661 | TG | T | 0.343 | NM_000238 | KCNH2 | frameshift_deleti  on | exon3 | c.470delC | p.P157fs | . | . |
| CTC2 | 7 | 150741077 | GTC | G | 0.538 | NM_001282293 | ABCB8 | frameshift_deleti  on | exon14 | c.1522_1523del | p.S508fs | . | . |
| CTC2 | 7 | 150748964 | GC | G | 0.512 | NM_004769 | ASIC3 | frameshift_deleti  on | exon7 | c.1283delC | p.A428fs | . | . |
| CTC2 | 7 | 150840917 | AT | A | 0.5 | NM_001281300 | AGAP3 | frameshift_deleti  on | exon16 | c.1631delT | p.I544fs | . | . |
| CTC2 | 7 | 150915880 | AT | A | 0.857 | NM_005692 | ABCF2 | stopgain | exon9 | c.1096delA | p.M366X | . | . |
| CTC2 | 7 | 151073864 | T | TC | 0.833 | NM_001243351 | NUB1 | frameshift_inserti on | exon14 | c.1709dupC | p.S570fs | . | . |
| CTC2 | 7 | 151133341 | TC | T | 0.667 | NM_144727 | CRYGN | frameshift_deleti  on | exon3 | c.340delG | p.E114fs | . | . |
| CTC2 | 7 | 151835937 | CA | C | 0.435 | NM_170606 | KMT2C | frameshift_deleti  on | exon58 | c.14586delT | p.F4862fs | . | . |
| CTC2 | 7 | 151945311 | C | CA | 0.294 | NM_170606 | KMT2C | frameshift_inserti  on | exon14 | c.2207dupT | p.L736fs | . | . |
| CTC2 | 7 | 155604538 | TC | T | 0.438 | NM_000193 | SHH | frameshift_deleti  on | exon1 | c.278delG | p.G93fs | . | . |
| CTC2 | 7 | 156761856 | AC | A | 0.571 | NM_138400 | NOM1 | frameshift_deleti  on | exon10 | c.2375delC | p.T792fs | . | . |
| CTC2 | 7 | 157449134 | C | CG | 0.188 | NM_130842 | PTPRN2 | frameshift_inserti  on | exon13 | c.2059_2060ins  C | p.S687fs | . | . |
| CTC2 | 8 | 3047596 | CG | C | 0.722 | NM_033225 | CSMD1 | frameshift_deleti  on | exon34 | c.5235delC | p.P1745fs | . | . |
| CTC2 | 8 | 3200858 | TG | T | 1 | NM_033225 | CSMD1 | frameshift_deleti  on | exon23 | c.3588delC | p.T1196fs | . | . |
| CTC2 | 8 | 7308377 | TG | T | 0.563 | NM_058207 | SPAG11B | frameshift_deleti  on | exon2 | c.100delC | p.Q34fs | . | . |
| CTC2 | 8 | 8234868 | C | CGCCG  CT | 0.941 | NM_001080826 | SGK223 | nonframeshift_in  sertion | exon2 | c.1050_1051ins  AGCGGC | p.A351delinsS  GA | . | . |
| CTC2 | 8 | 8887542 | TAAC | T | 1 | NM_153332 | ERI1 | frameshift_deleti  on | exon7 | c.1049_1310del | p.*350fs | . | . |
| CTC2 | 8 | 9413644 | GC | G | 1 | NM_003747 | TNKS | frameshift_deleti  on | exon1 | c.196delC | p.P66fs | . | . |
| CTC2 | 8 | 10583250 | AT | A | 0.875 | NM_031439 | SOX7 | frameshift_deleti  on | exon2 | c.1164delA | p.S388fs | . | . |
| CTC2 | 8 | 12943923 | AG | A | 1 | NM_001164271 | DLC1 | frameshift_deleti  on | exon13 | c.2808delC | p.T936fs | . | . |
| CTC2 | 8 | 16850690 | CT | C | 0.6 | NM_019851 | FGF20 | frameshift_deleti  on | exon3 | c.526delA | p.R176fs | . | . |
| CTC2 | 8 | 16963077 | GT | G | 0.5 | NM_181723 | MICU3 | frameshift_deleti  on | exon11 | c.1242delT | p.S414fs | . | . |
| CTC2 | 8 | 17402079 | G | GA | 0.5 | NM_001008539 | SLC7A2 | frameshift_inserti  on | exon3 | c.497dupA | p.D166fs | . | . |

| CTC2 | 8 | 21958369 | AC | A | 0.576 | NM_022749 | FAM160B2 | frameshift_deleti  on | exon12 | c.1503delC | p.D501fs | . | . |
| --- | --- | --- | --- | --- | --- | --- | --- | --- | --- | --- | --- | --- | --- |
| CTC2 | 8 | 22872268 | CT | C | 0.743 | NM_001160037 | RHOBTB2 | frameshift_deleti  on | exon8 | c.1859delT | p.L620fs | . | . |
| CTC2 | 8 | 23056865 | CG | C | 0.737 | NM_003844 | TNFRSF10A | frameshift_deleti  on | exon8 | c.927delC | p.F309fs | . | . |
| CTC2 | 8 | 23112899 | TG | T | 0.404 | NM_152272 | CHMP7 | frameshift_deleti  on | exon4 | c.612delG | p.L204fs | . | . |
| CTC2 | 8 | 23114032 | TG | T | 1 | NM_152272 | CHMP7 | frameshift_deleti  on | exon5 | c.718delG | p.G240fs | . | . |
| CTC2 | 8 | 23225714 | C | CG | 0.939 | NM_002318 | LOXL2 | frameshift_inserti  on | exon2 | c.150dupC | p.A51fs | . | . |
| CTC2 | 8 | 27310653 | G | GA | 0.268 | NM_173175 | PTK2B | frameshift_inserti  on | exon27 | c.2446dupA | p.Q815fs | . | . |
| CTC2 | 8 | 27312052 | TG | T | 0.618 | NM_173175 | PTK2B | frameshift_deleti  on | exon29 | c.2613delG | p.V871fs | . | . |
| CTC2 | 8 | 28206660 | CG | C | 0.556 | NM_018660 | ZNF395 | frameshift_deleti  on | exon9 | c.1411delC | p.R471fs | . | . |
| CTC2 | 8 | 28209273 | TG | T | 0.857 | NM_018660 | ZNF395 | frameshift_deleti  on | exon7 | c.971delC | p.T324fs | . | . |
| CTC2 | 8 | 28956669 | AT | A | 0.263 | NM_015254 | KIF13B | frameshift_deleti  on | exon36 | c.4304delA | p.N1435fs | . | . |
| CTC2 | 8 | 30695297 | TC | T | 0.82 | NM_031271 | TEX15 | frameshift_deleti  on | exon3 | c.7353delG | p.K2451fs | . | . |
| CTC2 | 8 | 30700208 | AT | A | 0.65 | NM_031271 | TEX15 | frameshift_deleti  on | exon1 | c.6325delA | p.M2109fs | . | . |
| CTC2 | 8 | 30922430 | GT | G | 0.435 | NM_000553 | WRN | frameshift_deleti  on | exon5 | c.356delT | p.V119fs | . | . |
| CTC2 | 8 | 30969274 | G | GA | 0.773 | NM_000553 | WRN | frameshift_inserti  on | exon19 | c.2233dupA | p.G744fs | . | . |
| CTC2 | 8 | 32505334 | CG | C | 0.67 | NM_013959 | NRG1 | frameshift_deleti  on | exon1 | c.99delG | p.P33fs | . | . |
| CTC2 | 8 | 33356764 | TC | T | 0.296 | NM_025115 | TTI2 | frameshift_deleti  on | exon7 | c.1453delG | p.E485fs | . | . |
| CTC2 | 8 | 33449726 | C | CT | 1 | NM_024025 | DUSP26 | frameshift_inserti  on | exon4 | c.440dupA | p.K147fs | . | . |
| CTC2 | 8 | 37704564 | TC | T | 0.448 | NM_018310 | BRF2 | frameshift_deleti  on | exon3 | c.343delG | p.E115fs | . | . |
| CTC2 | 8 | 38646336 | AG | A | 0.6 | NM_006283 | TACC1 | frameshift_deleti  on | exon2 | c.277delG | p.E93fs | . | . |
| CTC2 | 8 | 38677469 | TC | T | 0.318 | NM_001146216 | TACC1 | frameshift_deleti  on | exon3 | c.123delC | p.V41fs | . | . |
| CTC2 | 8 | 38677767 | AG | A | 0.429 | NM_001146216 | TACC1 | frameshift_deleti  on | exon3 | c.421delG | p.A141fs | . | . |
| CTC2 | 8 | 38848855 | TA | T | 0.75 | NM_001024380 | TM2D2 | frameshift_deleti  on | exon4 | c.511delT | p.Y171fs | . | . |
| CTC2 | 8 | 38940531 | TC | T | 0.495 | NM_003816 | ADAM9 | frameshift_deleti  on | exon18 | c.2008delC | p.P670fs | . | . |
| CTC2 | 8 | 39068712 | CA | C | 0.357 | NM_145004 | ADAM32 | frameshift_deleti  on | exon12 | c.1103delA | p.Q368fs | . | . |
| CTC2 | 8 | 39604009 | TC | T | 0.73 | NM_001278113 | ADAM2 | frameshift_deleti  on | exon18 | c.2098delG | p.E700fs | . | . |
| CTC2 | 8 | 41530042 | TTCC | T | 0.733 | NM_000037 | ANK1 | nonframeshift_de  letion | exon38 | c.4923_4925del | p.1641_1642de  l | . | . |
| CTC2 | 8 | 41552120 | AG | A | 0.308 | NM_000037 | ANK1 | frameshift_deleti  on | exon28 | c.3316delC | p.L1106fs | . | . |
| CTC2 | 8 | 43147688 | AG | A | 0.665 | NM_001002920 | POTEA | frameshift_deleti  on | exon1 | c.62delG | p.R21fs | . | . |
| CTC2 | 8 | 53030929 | AT | A | 0.517 | NM_014682 | ST18 | frameshift_deleti  on | exon24 | c.2827delA | p.I943fs | . | . |
| CTC2 | 8 | 56866527 | T | TG | 0.364 | NM_001111097 | LYN | frameshift_inserti  on | exon8 | c.712dupG | p.F237fs | . | . |
| CTC2 | 8 | 56911008 | AT | A | 0.394 | NM_001111097 | LYN | frameshift_deleti  on | exon11 | c.1092delT | p.D364fs | . | . |
| CTC2 | 8 | 59059265 | TCG | T | 0.148 | NM_147189 | FAM110B | frameshift_deleti  on | exon5 | c.477_478del | p.F159fs | . | . |
| CTC2 | 8 | 68107638 | AC | A | 0.391 | NM_024790 | CSPP1 | frameshift_deleti  on | exon29 | c.3477delC | p.D1159fs | . | . |
| CTC2 | 8 | 68107801 | GC | G | 0.323 | NM_024790 | CSPP1 | frameshift_deleti  on | exon29 | c.3640delC | p.Q1214fs | . | . |
| CTC2 | 8 | 68972920 | TG | T | 0.178 | NM_024870 | PREX2 | frameshift_deleti  on | exon11 | c.1246delG | p.V416fs | . | . |
| CTC2 | 8 | 77620212 | AC | A | 0.654 | NM_024721 | ZFHX4 | frameshift_deleti  on | exon3 | c.3023delC | p.T1008fs | . | . |
| CTC2 | 8 | 77766604 | CAG | C | 0.318 | NM_024721 | ZFHX4 | frameshift_deleti  on | exon10 | c.7448_7449del | p.Q2483fs | . | . |
| CTC2 | 8 | 80992634 | CA | C | 0.303 | NM_001025252 | TPD52 | frameshift_deleti  on | exon1 | c.54delT | p.F18fs | . | . |
| CTC2 | 8 | 81430752 | CA | C | 0.238 | NM_001105539 | ZBTB10 | frameshift_deleti  on | exon4 | c.2076delA | p.S692fs | . | . |
| CTC2 | 8 | 82714722 | TA | T | 0.8 | NM_152837 | SNX16 | stopgain | exon6 | c.743delT | p.L248X | . | . |
| CTC2 | 8 | 86126827 | C | CAACA TT | 1 | NM_001099673 | C8orf59 | nonframeshift_in sertion | exon4 | c.264_265insAA TGTT | p.D89delinsNV D | . | . |
| CTC2 | 8 | 86385926 | CA | C | 0.278 | NM_000067 | CA2 | frameshift_deleti  on | exon3 | c.238delA | p.K80fs | . | . |
| CTC2 | 8 | 87424023 | CA | C | 0.345 | NM_007013 | WWP1 | frameshift_deleti  on | exon9 | c.982delA | p.K328fs | . | . |
| CTC2 | 8 | 90782077 | AG | A | 0.204 | NM_003821 | RIPK2 | frameshift_deleti  on | exon4 | c.562delG | p.G188fs | . | . |
| CTC2 | 8 | 91075371 | AT | A | 0.403 | NM_004929 | CALB1 | frameshift_deleti  on | exon9 | c.556delA | p.M186fs | . | . |
| CTC2 | 8 | 94935541 | T | TA | 1 | NM_001161781 | PDP1 | frameshift_inserti  on | exon2 | c.1254_1255ins  A | p.D418fs | . | . |
| CTC2 | 8 | 95444390 | C | CG | 0.29 | NM_001256141 | FSBP | frameshift_inserti  on | exon2 | c.868dupC | p.R290fs | . | . |
| CTC2 | 8 | 95844378 | CA | C | 0.8 | NM_017864 | INTS8 | frameshift_deleti  on | exon6 | c.730delA | p.K244fs | . | . |
| CTC2 | 8 | 97345716 | CA | C | 0.217 | NM_014754 | PTDSS1 | frameshift_deleti  on | exon13 | c.1345delA | p.N449fs | . | . |
| CTC2 | 8 | 99045393 | CA | C | 0.39 | NM_002380 | MATN2 | frameshift_deleti  on | exon17 | c.2706delA | p.T902fs | . | . |
| CTC2 | 8 | 99045800 | GAAGA | G | 0.217 | NM_002380 | MATN2 | frameshift_deleti  on | exon18 | c.2729_2732del | p.E910fs | . | . |
| CTC2 | 8 | 99264809 | CT | C | 0.375 | NM_024759 | NIPAL2 | frameshift_deleti  on | exon3 | c.257delA | p.K86fs | . | . |
| CTC2 | 8 | 99266303 | CAA | C | 0.412 | NM_024759 | NIPAL2 | frameshift_deleti  on | exon2 | c.146_147del | p.F49fs | . | . |
| CTC2 | 8 | 99560295 | TG | T | 0.937 | NM_001256313 | STK3 | frameshift_deleti  on | exon7 | c.709delC | p.Q237fs | . | . |
| CTC2 | 8 | 99560297 | GGC | G | 0.455 | NM_001256313 | STK3 | frameshift_deleti  on | exon7 | c.706_707del | p.A236fs | . | . |
| CTC2 | 8 | 100712113 | CA | C | 0.185 | NM_017890 | VPS13B | frameshift_deleti  on | exon36 | c.6483delA | p.S2161fs | . | . |
| CTC2 | 8 | 101075832 | CT | C | 0.273 | NM_001286693 | RGS22 | frameshift_deleti  on | exon6 | c.620delA | p.K207fs | . | . |

| CTC2 | 8 | 101153762 | ATC | A | 1 | NM_001029860 | FBXO43 | frameshift_deleti  on | exon2 | c.718_719del | p.D240fs | . | . |
| --- | --- | --- | --- | --- | --- | --- | --- | --- | --- | --- | --- | --- | --- |
| CTC2 | 8 | 101153763 | TC | T | 1 | NM_001029860 | FBXO43 | frameshift_deleti  on | exon2 | c.718delG | p.D240fs | . | . |
| CTC2 | 8 | 103305918 | TA | T | 0.308 | NM_001282873 | UBR5 | frameshift_deleti  on | exon34 | c.4503delT | p.S1501fs | . | . |
| CTC2 | 8 | 103311769 | TC | T | 0.55 | NM_001282873 | UBR5 | frameshift_deleti  on | exon24 | c.3112delG | p.D1038fs | . | . |
| CTC2 | 8 | 103338887 | CA | C | 0.316 | NM_001282873 | UBR5 | frameshift_deleti  on | exon13 | c.1485delT | p.G495fs | . | . |
| CTC2 | 8 | 104432522 | CT | C | 0.72 | NM_015420 | DCAF13 | frameshift_deleti  on | exon2 | c.558delT | p.P186fs | . | . |
| CTC2 | 8 | 105263839 | TG | T | 0.294 | NM_001282882 | RIMS2 | frameshift_deleti  on | exon6 | c.650delG | p.W217fs | . | . |
| CTC2 | 8 | 106813580 | C | CA | 0.4 | NM_012082 | ZFPM2 | frameshift_inserti  on | exon8 | c.1271dupA | p.Q424fs | . | . |
| CTC2 | 8 | 109254092 | AT | A | 0.259 | NM_001568 | EIF3E | frameshift_deleti  on | exon2 | c.140delA | p.D47fs | . | . |
| CTC2 | 8 | 110441656 | G | GA | 0.152 | NM_177531 | PKHD1L1 | frameshift_inserti  on | exon26 | c.3088_3089ins  A | p.G1030fs | . | . |
| CTC2 | 8 | 110447532 | GTT | G | 0.175 | NM_177531 | PKHD1L1 | frameshift_deleti  on | exon29 | c.3455_3456del | p.V1152fs | . | . |
| CTC2 | 8 | 110476744 | AT | A | 0.259 | NM_177531 | PKHD1L1 | frameshift_deleti  on | exon49 | c.7684delT | p.F2562fs | . | . |
| CTC2 | 8 | 113563010 | AT | A | 0.6 | NM_052900 | CSMD3 | frameshift_deleti  on | exon26 | c.4141delA | p.I1381fs | . | . |
| CTC2 | 8 | 120079574 | AT | A | 0.4 | NM_006438 | COLEC10 | frameshift_deleti  on | exon1 | c.55delT | p.F19fs | . | . |
| CTC2 | 8 | 121239513 | AC | A | 0.316 | NM_021110 | COL14A1 | frameshift_deleti  on | exon17 | c.2060delC | p.T687fs | . | . |
| CTC2 | 8 | 124146399 | G | GA | 0.25 | NM_145647 | TBC1D31 | frameshift_inserti  on | exon17 | c.2453dupA | p.E818fs | . | . |
| CTC2 | 8 | 124154648 | GA | G | 0.867 | NM_145647 | TBC1D31 | frameshift_deleti  on | exon19 | c.2788delA | p.K930fs | . | . |
| CTC2 | 8 | 124266973 | TTTG | T | 0.6 | NM_001017926 | ZHX1 | nonframeshift_de  letion | exon3 | c.1211_1213del | p.404_405del | . | . |
| CTC2 | 8 | 124267955 | GA | G | 0.32 | NM_001017926 | ZHX1 | frameshift_deleti  on | exon3 | c.231delT | p.F77fs | . | . |
| CTC2 | 8 | 124350057 | CA | C | 0.917 | NM_014109 | ATAD2 | frameshift_deleti  on | exon21 | c.2858delT | p.L953fs | . | . |
| CTC2 | 8 | 124358509 | A | AT | 0.389 | NM_014109 | ATAD2 | frameshift_inserti  on | exon18 | c.2348dupA | p.N783fs | . | . |
| CTC2 | 8 | 124453556 | GA | G | 0.9 | NM_001283024 | WDYHV1 | frameshift_deleti  on | exon6 | c.340delA | p.N114fs | . | . |
| CTC2 | 8 | 124992928 | AG | A | 0.504 | NM_001039112 | FER1L6 | frameshift_deleti  on | exon11 | c.1288delG | p.D430fs | . | . |
| CTC2 | 8 | 130861594 | TG | T | 0.179 | NM_016623 | FAM49B | frameshift_deleti  on | exon10 | c.734delC | p.T245fs | . | . |
| CTC2 | 8 | 131070217 | CT | C | 0.221 | NM_018482 | ASAP1 | frameshift_deleti  on | exon29 | c.3297delA | p.E1099fs | . | . |
| CTC2 | 8 | 131164994 | G | GA | 0.5 | NM_018482 | ASAP1 | frameshift_inserti  on | exon13 | c.1067dupT | p.I356fs | . | . |
| CTC2 | 8 | 132991601 | CGG | C | 0.254 | NM_015137 | EFR3A | frameshift_deleti  on | exon14 | c.1509_1510del | p.P503fs | . | . |
| CTC2 | 8 | 141407759 | G | GA | 0.333 | NM_001160372 | TRAPPC9 | frameshift_inserti  on | exon7 | c.1093dupT | p.S365fs | . | . |
| CTC2 | 8 | 141712732 | CA | C | 0.348 | NM_001199649 | PTK2 | frameshift_deleti  on | exon25 | c.2303delT | p.L768fs | . | . |
| CTC2 | 8 | 143365832 | AT | A | 0.271 | NM_145003 | TSNARE1 | frameshift_deleti  on | exon11 | c.1304delA | p.D435fs | . | . |
| CTC2 | 8 | 143746717 | TAG | T | 0.545 | NM_001077527 | JRK | frameshift_deleti  on | exon2 | c.759_760del | p.A253fs | . | . |
| CTC2 | 8 | 143857051 | GC | G | 1 | NM_023946 | LYNX1 | frameshift_deleti  on | exon3 | c.113delG | p.R38fs | . | . |
| CTC2 | 8 | 144332337 | CCA | C | 0.808 | NM_001271156 | ZFP41 | frameshift_deleti  on | exon2 | c.325_326del | p.H109fs | . | . |
| CTC2 | 8 | 144413472 | TC | T | 0.273 | NM_052963 | TOP1MT | stopgain | exon2 | c.159delG | p.W53X | . | . |
| CTC2 | 8 | 144900113 | AC | A | 0.273 | NM_001271097 | PUF60 | frameshift_deleti on | exon6 | c.597delG | p.V199fs | . | . |
| CTC2 | 8 | 144906519 | TGCCGC  CGCC | T | 0.643 | NM_001271096 | PUF60 | nonframeshift_de  letion | exon2 | c.63_71del | p.21_24del | . | . |
| CTC2 | 8 | 145095581 | TG | T | 0.197 | NM_001134374 | SPATC1 | frameshift_deleti  on | exon3 | c.880delG | p.A294fs | . | . |
| CTC2 | 8 | 145140304 | TG | T | 0.287 | NM_003801 | GPAA1 | frameshift_deleti  on | exon10 | c.1374delG | p.V458fs | . | . |
| CTC2 | 8 | 145223237 | AC | A | 0.415 | NM_001099281 | MROH1 | frameshift_deleti  on | exon3 | c.63delC | p.D21fs | . | . |
| CTC2 | 8 | 145245805 | CT | C | 1 | NM_001099281 | MROH1 | frameshift_deleti  on | exon7 | c.682delT | p.F228fs | . | . |
| CTC2 | 8 | 145622756 | GA | G | 0.954 | NM_013291 | CPSF1 | frameshift_deleti  on | exon22 | c.2330delT | p.F777fs | . | . |
| CTC2 | 8 | 145659067 | CG | C | 0.875 | NM_013432 | TONSL | frameshift_deleti  on | exon22 | c.3462delC | p.H1154fs | . | . |
| CTC2 | 8 | 145661718 | C | CG | 0.778 | NM_013432 | TONSL | frameshift_inserti  on | exon17 | c.2097_2098ins  C | p.E700fs | . | . |
| CTC2 | 8 | 145736285 | AG | A | 0.194 | NM_138431 | MFSD3 | frameshift_deleti  on | exon4 | c.1065delG | p.Q355fs | . | . |
| CTC2 | 8 | 145773767 | C | CG | 0.5 | NM_025251 | ARHGAP39 | frameshift_inserti  on | exon6 | c.702_703insC | p.D235fs | . | . |
| CTC2 | 9 | 1057185 | CA | C | 0.235 | NM_181872 | DMRT2 | frameshift_deleti  on | exon4 | c.1599delA | p.T533fs | . | . |
| CTC2 | 9 | 2191377 | AT | A | 0.8 | NM_001289398 | SMARCA2 | frameshift_deleti  on | exon6 | c.681delT | p.D227fs | . | . |
| CTC2 | 9 | 4585414 | TG | T | 1 | NM_004170 | SLC1A1 | frameshift_deleti  on | exon12 | c.1432delG | p.E478fs | . | . |
| CTC2 | 9 | 5300385 | CA | C | 0.343 | NM_134441 | RLN2 | frameshift_deleti  on | exon2 | c.270delT | p.F90fs | . | . |
| CTC2 | 9 | 5339620 | GC | G | 0.604 | NM_006911 | RLN1 | frameshift_deleti  on | exon1 | c.126delG | p.A42fs | . | . |
| CTC2 | 9 | 5339708 | AC | A | 0.704 | NM_006911 | RLN1 | frameshift_deleti  on | exon1 | c.38delG | p.C13fs | . | . |
| CTC2 | 9 | 5787162 | GT | G | 0.75 | NM_024896 | ERMP1 | frameshift_deleti  on | exon15 | c.2696delA | p.Y899fs | . | . |
| CTC2 | 9 | 5920035 | GT | G | 1 | NM_001017969 | KIAA2026 | frameshift_deleti  on | exon8 | c.5960delA | p.N1987fs | . | . |
| CTC2 | 9 | 8504260 | CT | C | 0.393 | NM_001171025 | PTPRD | frameshift_deleti  on | exon10 | c.1792delA | p.M598fs | . | . |
| CTC2 | 9 | 12775861 | T | TGGCG  GCGGC | 1 | NM_203403 | LURAP1L | nonframeshift_in  sertion | exon1 | c.147_148insG  GCGGCGGC | p.G49delinsGG  GG | . | . |
| CTC2 | 9 | 14120458 | GA | G | 0.3 | NM_001282787 | NFIB | frameshift_deleti  on | exon7 | c.469delT | p.S157fs | . | . |
| CTC2 | 9 | 14398604 | GT | G | 0.226 | NM_001190738 | NFIB | frameshift_deleti  on | exon1 | c.26delA | p.D9fs | . | . |
| CTC2 | 9 | 14823286 | T | TG | 0.467 | NM_144966 | FREM1 | frameshift_inserti  on | exon14 | c.2208dupC | p.M737fs | . | . |

| CTC2 | 9 | 14857594 | A | AT | 0.333 | NM_144966 | FREM1 | frameshift_inserti  on | exon6 | c.784dupA | p.I262fs | . | . |
| --- | --- | --- | --- | --- | --- | --- | --- | --- | --- | --- | --- | --- | --- |
| CTC2 | 9 | 19058149 | AT | A | 0.293 | NM_001270890 | HAUS6 | frameshift_deleti  on | exon16 | c.2510delA | p.N837fs | . | . |
| CTC2 | 9 | 19520905 | CA | C | 0.455 | NM_001193288 | SLC24A2 | frameshift_deleti  on | exon8 | c.1671delT | p.F557fs | . | . |
| CTC2 | 9 | 20414343 | ACTG | A | 0.138 | NM_001286691 | MLLT3 | nonframeshift_de  letion | exon5 | c.489_491del | p.163_164del | . | . |
| CTC2 | 9 | 20885126 | AT | A | 0.6 | NM_017794 | FOCAD | stopgain | exon23 | c.2523delT | p.Y841X | . | . |
| CTC2 | 9 | 33068870 | GC | G | 0.565 | NM_018225 | SMU1 | frameshift_deleti on | exon4 | c.452delG | p.G151fs | . | . |
| CTC2 | 9 | 33442465 | AG | A | 0.289 | NM_004925 | AQP3 | frameshift_deleti  on | exon5 | c.543delC | p.P181fs | . | . |
| CTC2 | 9 | 34990740 | TG | T | 0.431 | NM_001135005 | DNAJB5 | frameshift_deleti  on | exon1 | c.114delG | p.L38fs | . | . |
| CTC2 | 9 | 34996837 | TA | T | 1 | NM_001135005 | DNAJB5 | frameshift_deleti  on | exon3 | c.1004delA | p.Y335fs | . | . |
| CTC2 | 9 | 35101448 | CG | C | 0.514 | NM_001287033 | STOML2 | frameshift_deleti  on | exon5 | c.415delC | p.R139fs | . | . |
| CTC2 | 9 | 35107754 | TCC | T | 1 | NM_025182 | FAM214B | frameshift_deleti  on | exon3 | c.516_517del | p.G172fs | . | . |
| CTC2 | 9 | 35258995 | TA | T | 0.87 | NM_006377 | UNC13B | frameshift_deleti  on | exon7 | c.475delA | p.S159fs | . | . |
| CTC2 | 9 | 35548440 | GC | G | 1 | NM_001135999 | RUSC2 | frameshift_deleti  on | exon2 | c.1923delC | p.G641fs | . | . |
| CTC2 | 9 | 35650506 | GC | G | 1 | NM_014450 | SIT1 | frameshift_deleti  on | exon2 | c.228delG | p.G76fs | . | . |
| CTC2 | 9 | 35673970 | G | GC | 0.675 | NM_001216 | CA9 | frameshift_inserti  on | exon1 | c.15dupC | p.C5fs | . | . |
| CTC2 | 9 | 35802738 | GA | G | 0.571 | NM_003995 | NPR2 | frameshift_deleti  on | exon12 | c.1826delA | p.E609fs | . | . |
| CTC2 | 9 | 35811485 | ACCAGG  ACCAGA | A | 1 | NM_001039592 | SPAG8 | nonframeshift_de  letion | exon2 | c.546_557del | p.182_186del | . | . |
| CTC2 | 9 | 35906347 | CCTG | C | 0.364 | NM_001039792 | HRCT1 | nonframeshift_de  letion | exon1 | c.64_66del | p.22_22del | . | . |
| CTC2 | 9 | 35957732 | C | CA | 0.163 | NM_019897 | OR2S2 | stopgain | exon1 | c.363dupT | p.D122_R123d  elinsX | . | . |
| CTC2 | 9 | 37729761 | C | CG | 0.762 | NM_014907 | FRMPD1 | frameshift_inserti  on | exon8 | c.650dupG | p.R217fs | . | . |
| CTC2 | 9 | 37745557 | TC | T | 1 | NM_014907 | FRMPD1 | frameshift_deleti  on | exon16 | c.3529delC | p.P1177fs | . | . |
| CTC2 | 9 | 40705615 | CA | C | 0.388 | NM_001083124 | SPATA31A3 | frameshift_deleti  on | exon4 | c.3273delA | p.P1091fs | . | . |
| CTC2 | 9 | 69420403 | G | GA | 0.273 | NM_001098805 | ANKRD20A4 | frameshift_inserti  on | exon13 | c.1294dupA | p.K431fs | . | . |
| CTC2 | 9 | 71628080 | TTC | T | 0.444 | NM_002732 | PRKACG | frameshift_deleti  on | exon1 | c.927_928del | p.K309fs | . | . |
| CTC2 | 9 | 73461480 | GT | G | 1 | NM_001007470 | TRPM3 | frameshift_deleti  on | exon4 | c.30delA | p.K10fs | . | . |
| CTC2 | 9 | 74838075 | CT | C | 0.151 | NM_001242505 | GDA | frameshift_deleti  on | exon7 | c.647delT | p.L216fs | . | . |
| CTC2 | 9 | 77277492 | CA | C | 0.9 | NM_006914 | RORB | frameshift_deleti  on | exon6 | c.863delA | p.Q288fs | . | . |
| CTC2 | 9 | 77377698 | TC | T | 0.2 | NM_001177310 | TRPM6 | frameshift_deleti  on | exon26 | c.3873delG | p.Q1291fs | . | . |
| CTC2 | 9 | 77390952 | AC | A | 0.353 | NM_001177310 | TRPM6 | frameshift_deleti  on | exon24 | c.3234delG | p.L1078fs | . | . |
| CTC2 | 9 | 79323052 | AT | A | 0.593 | NM_015225 | PRUNE2 | frameshift_deleti  on | exon8 | c.4137delA | p.K1379fs | . | . |
| CTC2 | 9 | 79441614 | TG | T | 0.846 | NM_015225 | PRUNE2 | stopgain | exon5 | c.542delC | p.S181X | . | . |
| CTC2 | 9 | 80880409 | GA | G | 0.364 | NM_001098802 | CEP78 | frameshift_deleti on | exon14 | c.1751delA | p.E584fs | . | . |
| CTC2 | 9 | 84208071 | CG | C | 0.688 | NM_005077 | TLE1 | frameshift_deleti  on | exon15 | c.1449delC | p.H483fs | . | . |
| CTC2 | 9 | 86284193 | AG | A | 1 | NM_013438 | UBQLN1 | frameshift_deleti  on | exon7 | c.1154delC | p.T385fs | . | . |
| CTC2 | 9 | 87356826 | TG | T | 0.147 | NM_001007097 | NTRK2 | frameshift_deleti  on | exon10 | c.1180delG | p.D394fs | . | . |
| CTC2 | 9 | 88916326 | TC | T | 0.714 | NM_001185074 | ZCCHC6 | frameshift_deleti  on | exon19 | c.3576delG | p.G1192fs | . | . |
| CTC2 | 9 | 88943338 | A | AC | 0.333 | NM_001185074 | ZCCHC6 | frameshift_inserti  on | exon7 | c.1155_1156ins  G | p.W386fs | . | . |
| CTC2 | 9 | 88952385 | GA | G | 0.321 | NM_001185059 | ZCCHC6 | frameshift_deleti  on | exon10 | c.1403delT | p.F468fs | . | . |
| CTC2 | 9 | 90264961 | CT | C | 0.2 | NM_001288729 | DAPK1 | frameshift_deleti  on | exon16 | c.1555delT | p.S519fs | . | . |
| CTC2 | 9 | 90534170 | C | CGTCA  TCTTGT CTCCC | 0.648 | NM_001145124 | SPATA31C1 | nonframeshift_in sertion | exon2 | c.190_191insGT  CATCTTGTCTC CCAGG | p.R64delinsRHL VSQG | . | . |
| CTC2 | 9 | 90534174 | A | ATCTTG  TCTCCC AGCGT | 0.667 | NM_001145124 | SPATA31C1 | nonframeshift_in sertion | exon2 | c.194_195insTC  TTGTCTCCCAG CGTCT | p.H65delinsHL VSQRL | . | . |
| CTC2 | 9 | 90534195 | T | TTGTCT  CCCAG CGTCA | 0.7 | NM_001145124 | SPATA31C1 | nonframeshift_in sertion | exon2 | c.215_216insTG  TCTCCCAGCGT CATCC | p.L72delinsLVS QRHP | . | . |
| CTC2 | 9 | 91262533 | CT | C | 0.335 | NM_001100111 | RP13-  60M5.2 | frameshift_deleti  on | exon2 | c.109delA | p.S37fs | . | . |
| CTC2 | 9 | 92008538 | AT | A | 0.308 | NM_001142287 | SEMA4D | frameshift_deleti  on | exon9 | c.446delA | p.N149fs | . | . |
| CTC2 | 9 | 93627359 | CT | C | 0.317 | NM_001135052 | SYK | frameshift_deleti  on | exon6 | c.827delT | p.L276fs | . | . |
| CTC2 | 9 | 94172844 | G | GA | 0.146 | NM_005384 | NFIL3 | frameshift_inserti  on | exon2 | c.172dupT | p.S58fs | . | . |
| CTC2 | 9 | 94830249 | CA | C | 0.397 | NM_001281303 | SPTLC1 | frameshift_deleti  on | exon6 | c.558delT | p.F186fs | . | . |
| CTC2 | 9 | 95003185 | GC | G | 0.248 | NM_002161 | IARS | frameshift_deleti  on | exon30 | c.3235delG | p.A1079fs | . | . |
| CTC2 | 9 | 95237024 | CTCA | C | 0.909 | NM_001193335 | ASPN | nonframeshift_de  letion | exon2 | c.153_155del | p.51_52del | . | . |
| CTC2 | 9 | 95237024 | CTCATC  A | C | 0.909 | NM_001193335 | ASPN | nonframeshift_de  letion | exon2 | c.150_155del | p.50_52del | . | . |
| CTC2 | 9 | 95482687 | CG | C | 0.632 | NM_001003800 | BICD2 | frameshift_deleti  on | exon4 | c.956delC | p.T319fs | . | . |
| CTC2 | 9 | 95609712 | AT | A | 0.727 | NM_001007101 | ZNF484 | frameshift_deleti  on | exon4 | c.1248delA | p.K416fs | . | . |
| CTC2 | 9 | 95640110 | TC | T | 0.455 | NM_001261458 | ZNF484 | frameshift_deleti  on | exon1 | c.12delG | p.L4fs | . | . |
| CTC2 | 9 | 97823078 | GA | G | 0.316 | NM_032823 | C9orf3 | frameshift_deleti  on | exon12 | c.1922delA | p.D641fs | . | . |
| CTC2 | 9 | 99522471 | AT | A | 0.261 | NM_014930 | ZNF510 | frameshift_deleti  on | exon6 | c.640delA | p.I214fs | . | . |

| CTC2 | 9 | 100092968 | A | AGAGG  AG | 0.941 | NM_020893 | CCDC180 | nonframeshift_in  sertion | exon18 | c.2325_2326ins  GAGGAG | p.E775delinsEE  E | . | . |
| --- | --- | --- | --- | --- | --- | --- | --- | --- | --- | --- | --- | --- | --- |
| CTC2 | 9 | 100092968 | A | AGAG | 0.941 | NM_020893 | CCDC180 | nonframeshift_in  sertion | exon18 | c.2325_2326ins  GAG | p.E775delinsEE | . | . |
| CTC2 | 9 | 100092981 | G | GAGGA  GA | 0.852 | NM_020893 | CCDC180 | nonframeshift_in  sertion | exon18 | c.2338_2339ins  AGGAGA | p.E780delinsEE  K | . | . |
| CTC2 | 9 | 100222520 | CT | C | 0.328 | NM_014290 | TDRD7 | frameshift_deleti  on | exon7 | c.917delT | p.L306fs | . | . |
| CTC2 | 9 | 100693040 | CT | C | 0.464 | NM_197978 | HEMGN | frameshift_deleti  on | exon3 | c.636delA | p.Q212fs | . | . |
| CTC2 | 9 | 100777656 | AGG | A | 0.259 | NM_006401 | ANP32B | frameshift_deleti  on | exon7 | c.700_701del | p.G234fs | . | . |
| CTC2 | 9 | 100893325 | GGC | G | 0.729 | NM_003389 | CORO2A | frameshift_deleti  on | exon7 | c.780_781del | p.V260fs | . | . |
| CTC2 | 9 | 101900367 | T | TA | 0.236 | NM_001130916 | TGFBR1 | frameshift_inserti  on | exon3 | c.571dupA | p.N190fs | . | . |
| CTC2 | 9 | 102590615 | TCAC | T | 0.444 | NM_006981 | NR4A3 | nonframeshift_de  letion | exon3 | c.292_294del | p.98_98del | . | . |
| CTC2 | 9 | 102888757 | CT | C | 0.308 | NM_014425 | INVS | frameshift_deleti  on | exon3 | c.200delT | p.L67fs | . | . |
| CTC2 | 9 | 104124731 | TG | T | 0.83 | NM_001127610 | BAAT | frameshift_deleti  on | exon4 | c.1235delC | p.P412fs | . | . |
| CTC2 | 9 | 104385667 | CAG | C | 0.136 | NM_133445 | GRIN3A | frameshift_deleti  on | exon5 | c.2545_2546del | p.L849fs | . | . |
| CTC2 | 9 | 105767954 | CA | C | 0.8 | NM_001340 | CYLC2 | frameshift_deleti  on | exon5 | c.1042delA | p.K348fs | . | . |
| CTC2 | 9 | 107361026 | CA | C | 1 | NM_001004482 | OR13C5 | stopgain | exon1 | c.668delT | p.L223X | . | . |
| CTC2 | 9 | 107554239 | CA | C | 0.842 | NM_005502 | ABCA1 | frameshift_deleti on | exon43 | c.5797delT | p.C1933fs | . | . |
| CTC2 | 9 | 108397372 | GT | G | 0.34 | NM_006731 | FKTN | frameshift_deleti  on | exon10 | c.1214delT | p.V405fs | . | . |
| CTC2 | 9 | 108536189 | CT | C | 0.353 | NM_018112 | TMEM38B | frameshift_deleti  on | exon6 | c.705delT | p.P235fs | . | . |
| CTC2 | 9 | 109701338 | AC | A | 0.286 | NM_021224 | ZNF462 | frameshift_deleti  on | exon7 | c.6378delC | p.H2126fs | . | . |
| CTC2 | 9 | 111618004 | CG | C | 1 | NM_006686 | ACTL7B | frameshift_deleti  on | exon1 | c.206delC | p.P69fs | . | . |
| CTC2 | 9 | 111706292 | CA | C | 1 | NM_001286974 | CTNNAL1 | frameshift_deleti  on | exon16 | c.1890delT | p.F630fs | . | . |
| CTC2 | 9 | 111710494 | GT | G | 0.714 | NM_001286974 | CTNNAL1 | frameshift_deleti  on | exon15 | c.1855delA | p.T619fs | . | . |
| CTC2 | 9 | 112166858 | TC | T | 0.339 | NM_001145372 | PTPN3 | frameshift_deleti  on | exon8 | c.826delG | p.E276fs | . | . |
| CTC2 | 9 | 112899948 | AT | A | 0.647 | NM_001004065 | AKAP2 | frameshift_deleti  on | exon2 | c.1699delT | p.L567fs | . | . |
| CTC2 | 9 | 113169067 | TC | T | 1 | NM_153366 | SVEP1 | frameshift_deleti  on | exon38 | c.8812delG | p.D2938fs | . | . |
| CTC2 | 9 | 113196634 | GT | G | 0.429 | NM_153366 | SVEP1 | frameshift_deleti  on | exon30 | c.5040delA | p.Q1680fs | . | . |
| CTC2 | 9 | 115216427 | CT | C | 0.196 | NM_001195822 | HSDL2 | frameshift_deleti  on | exon7 | c.782delT | p.L261fs | . | . |
| CTC2 | 9 | 115964903 | GC | G | 0.32 | NM_015258 | FKBP15 | frameshift_deleti  on | exon6 | c.405delG | p.R135fs | . | . |
| CTC2 | 9 | 116187645 | GGCA | G | 0.391 | NM_001278629 | C9orf43 | nonframeshift_de  letion | exon10 | c.888_890del | p.296_297del | . | . |
| CTC2 | 9 | 116245283 | CTG | C | 0.267 | NM_001282923 | RGS3 | frameshift_deleti  on | exon3 | c.82_83del | p.C28fs | . | . |
| CTC2 | 9 | 116855735 | GCC | G | 0.308 | NM_138424 | KIF12 | frameshift_deleti  on | exon12 | c.1027_1028del | p.G343fs | . | . |
| CTC2 | 9 | 116945338 | G | GC | 0.214 | NM_032888 | COL27A1 | frameshift_inserti  on | exon5 | c.1999_2000ins  C | p.G667fs | . | . |
| CTC2 | 9 | 117143565 | TC | T | 1 | NM_030767 | AKNA | frameshift_deleti  on | exon2 | c.48delG | p.G16fs | . | . |
| CTC2 | 9 | 117838794 | TC | T | 0.206 | NM_002160 | TNC | frameshift_deleti  on | exon8 | c.2734delG | p.E912fs | . | . |
| CTC2 | 9 | 117852909 | TC | T | 0.366 | NM_002160 | TNC | frameshift_deleti  on | exon2 | c.388delG | p.E130fs | . | . |
| CTC2 | 9 | 118949874 | TC | T | 0.156 | NM_002581 | PAPPA | frameshift_deleti  on | exon2 | c.858delC | p.L286fs | . | . |
| CTC2 | 9 | 119737503 | CT | C | 0.213 | NM_014010 | ASTN2 | frameshift_deleti  on | exon9 | c.1719delA | p.E573fs | . | . |
| CTC2 | 9 | 123249709 | TG | T | 0.562 | NM_001011649 | CDK5RAP2 | frameshift_deleti  on | exon14 | c.1488delC | p.F496fs | . | . |
| CTC2 | 9 | 125391075 | CAG | C | 0.137 | NM_001004450 | OR1B1 | frameshift_deleti  on | exon1 | c.738_739del | p.T246fs | . | . |
| CTC2 | 9 | 125645532 | GT | G | 0.389 | NM_001100588 | RC3H2 | frameshift_deleti  on | exon5 | c.709delA | p.T237fs | . | . |
| CTC2 | 9 | 125645593 | TC | T | 0.353 | NM_001100588 | RC3H2 | frameshift_deleti  on | exon5 | c.648delG | p.R216fs | . | . |
| CTC2 | 9 | 125673622 | CT | C | 0.452 | NM_006626 | ZBTB6 | frameshift_deleti  on | exon2 | c.729delA | p.K243fs | . | . |
| CTC2 | 9 | 128001027 | AT | A | 0.625 | NM_005347 | HSPA5 | frameshift_deleti  on | exon6 | c.1075delA | p.I359fs | . | . |
| CTC2 | 9 | 130100424 | CA | C | 0.268 | NM_032293 | GARNL3 | frameshift_deleti  on | exon12 | c.1013delA | p.Q338fs | . | . |
| CTC2 | 9 | 130438181 | CA | C | 1 | NM_001032221 | STXBP1 | frameshift_deleti  on | exon14 | c.1210delA | p.K404fs | . | . |
| CTC2 | 9 | 130868636 | TG | T | 0.75 | NM_001006641 | SLC25A25 | frameshift_deleti  on | exon8 | c.1014delG | p.L338fs | . | . |
| CTC2 | 9 | 130925763 | TG | T | 0.333 | NM_024112 | C9orf16 | frameshift_deleti  on | exon2 | c.122delG | p.C41fs | . | . |
| CTC2 | 9 | 130928622 | T | TG | 0.391 | NM_001257976 | CIZ1 | frameshift_inserti  on | exon16 | c.2247dupC | p.N750fs | . | . |
| CTC2 | 9 | 130953108 | AGCT | A | 0.149 | NM_001131015 | CIZ1 | nonframeshift_de  letion | exon2 | c.26_28del | p.9_10del | . | . |
| CTC2 | 9 | 131020795 | GCCT | G | 0.25 | NM_004486 | GOLGA2 | nonframeshift_de  letion | exon21 | c.2144_2146del | p.715_716del | . | . |
| CTC2 | 9 | 131371431 | GA | G | 0.235 | NM_001195532 | SPTAN1 | frameshift_deleti  on | exon35 | c.4567delA | p.K1523fs | . | . |
| CTC2 | 9 | 131388735 | AGAG | A | 0.118 | NM_001195532 | SPTAN1 | nonframeshift_de  letion | exon47 | c.6271_6273del | p.2091_2091de  l | . | . |
| CTC2 | 9 | 131483484 | TTCC | T | 0.182 | NM_032799 | ZDHHC12 | nonframeshift_de  letion | exon5 | c.777_779del | p.259_260del | . | . |
| CTC2 | 9 | 131504877 | AC | A | 0.451 | NM_006336 | ZER1 | frameshift_deleti  on | exon9 | c.1506delG | p.L502fs | . | . |
| CTC2 | 9 | 131583075 | ACC | A | 0.581 | NM_004435 | ENDOG | frameshift_deleti  on | exon2 | c.560_561del | p.T187fs | . | . |
| CTC2 | 9 | 132637614 | GA | G | 0.3 | NM_001008563 | USP20 | frameshift_deleti  on | exon20 | c.2075delA | p.E692fs | . | . |
| CTC2 | 9 | 132890977 | CG | C | 0.393 | NM_001287346 | GPR107 | frameshift_deleti  on | exon17 | c.933delG | p.P311fs | . | . |
| CTC2 | 9 | 133760412 | GA | G | 0.226 | NM_005157 | ABL1 | frameshift_deleti  on | exon11 | c.2736delA | p.G912fs | . | . |

| CTC2 | 9 | 134019960 | CT | C | 0.5 | NM_005085 | NUP214 | frameshift_deleti  on | exon12 | c.1589delT | p.L530fs | . | . |
| --- | --- | --- | --- | --- | --- | --- | --- | --- | --- | --- | --- | --- | --- |
| CTC2 | 9 | 135277372 | CT | C | 1 | NM_007344 | TTF1 | frameshift_deleti  on | exon2 | c.836delA | p.K279fs | . | . |
| CTC2 | 9 | 138590227 | AC | A | 0.8 | NM_001012415 | SOHLH1 | frameshift_deleti  on | exon3 | c.292delG | p.V98fs | . | . |
| CTC2 | 9 | 139341772 | TC | T | 0.827 | NM_001276418 | SEC16A | frameshift_deleti  on | exon26 | c.6603delG | p.G2201fs | . | . |
| CTC2 | 9 | 139371462 | AG | A | 0.527 | NM_001276418 | SEC16A | frameshift_deleti  on | exon2 | c.605delC | p.P202fs | . | . |
| CTC2 | 9 | 139571415 | T | TC | 0.24 | NM_001012727 | AGPAT2 | frameshift_inserti  on | exon3 | c.489dupG | p.N164fs | . | . |
| CTC2 | 9 | 139616703 | C | CG | 0.7 | NM_152421 | FAM69B | frameshift_inserti  on | exon4 | c.434dupG | p.R145fs | . | . |
| CTC2 | 9 | 139972668 | GGCCGG  TGAGCG CCACGG GACCCG | G | 0.143 | NM_207309 | UAP1L1 | nonframeshift_de letion | exon2 | c.457_486del | p.153_162del | . | . |
| CTC2 | 9 | 140040335 | TG | T | 1 | NM_000832 | GRIN1 | frameshift_deleti  on | exon3 | c.552delG | p.L184fs | . | . |
| CTC2 | 9 | 140355119 | GA | G | 1 | NM_152286 | PNPLA7 | frameshift_deleti  on | exon33 | c.3836delT | p.F1279fs | . | . |
| CTC2 | 10 | 410516 | G | GA | 0.179 | NM_014974 | DIP2C | frameshift_inserti  on | exon20 | c.2274dupT | p.P759fs | . | . |
| CTC2 | 10 | 1313175 | CG | C | 1 | NM_018702 | ADARB2 | frameshift_deleti  on | exon4 | c.1166delC | p.A389fs | . | . |
| CTC2 | 10 | 5416053 | C | CT | 1 | NM_053049 | UCN3 | frameshift_inserti  on | exon2 | c.371dupT | p.L124fs | . | . |
| CTC2 | 10 | 5435777 | AAC | A | 0.146 | NM_001171864 | TUBAL3 | frameshift_deleti  on | exon4 | c.922_923del | p.V308fs | . | . |
| CTC2 | 10 | 5435778 | AC | A | 0.178 | NM_001171864 | TUBAL3 | frameshift_deleti  on | exon4 | c.922delG | p.V308fs | . | . |
| CTC2 | 10 | 5789879 | G | GT | 0.305 | NM_017782 | FAM208B | frameshift_inserti  on | exon15 | c.4496dupT | p.V1499fs | . | . |
| CTC2 | 10 | 5789966 | CCTGCC  TCAG | C | 1 | NM_017782 | FAM208B | nonframeshift_de  letion | exon15 | c.4583_4591del | p.1528_1531de  l | . | . |
| CTC2 | 10 | 7318901 | AT | A | 0.472 | NM_001018039 | SFMBT2 | frameshift_deleti  on | exon7 | c.822delA | p.K274fs | . | . |
| CTC2 | 10 | 8115955 | AC | A | 0.917 | NM_001002295 | GATA3 | frameshift_deleti  on | exon6 | c.1305delC | p.H435fs | . | . |
| CTC2 | 10 | 11047395 | GC | G | 0.375 | NM_001025077 | CELF2 | frameshift_deleti  on | exon1 | c.46delC | p.L16fs | . | . |
| CTC2 | 10 | 11523847 | CA | C | 0.75 | NM_001080491 | USP6NL | frameshift_deleti  on | exon13 | c.1050delT | p.F350fs | . | . |
| CTC2 | 10 | 13240750 | TG | T | 0.48 | NM_018518 | MCM10 | frameshift_deleti  on | exon16 | c.2182delG | p.E728fs | . | . |
| CTC2 | 10 | 13246263 | GC | G | 0.163 | NM_018518 | MCM10 | frameshift_deleti  on | exon18 | c.2398delC | p.Q800fs | . | . |
| CTC2 | 10 | 13378253 | AT | A | 0.314 | NM_001195602 | SEPHS1 | stopgain | exon3 | c.193delA | p.M65X | . | . |
| CTC2 | 10 | 13538833 | GC | G | 0.622 | NM_001100912 | BEND7 | frameshift_deleti on | exon3 | c.386delG | p.C129fs | . | . |
| CTC2 | 10 | 15648349 | AG | A | 1 | NM_003638 | ITGA8 | frameshift_deleti  on | exon18 | c.1836delC | p.T612fs | . | . |
| CTC2 | 10 | 20106104 | CG | C | 0.464 | NM_001282736 | PLXDC2 | frameshift_deleti  on | exon1 | c.97delG | p.G33fs | . | . |
| CTC2 | 10 | 20453402 | TG | T | 0.4 | NM_001282736 | PLXDC2 | frameshift_deleti  on | exon6 | c.643delG | p.V215fs | . | . |
| CTC2 | 10 | 21805480 | TTCC | T | 0.143 | NM_207371 | SKIDA1 | nonframeshift_de  letion | exon4 | c.1269_1271del | p.423_424del | . | . |
| CTC2 | 10 | 21805697 | CGGTGG  TGGTGG TGGTGG TGGTGA | C | 0.833 | NM_207371 | SKIDA1 | nonframeshift_de letion | exon4 | c.1028_1054del | p.343_352del | . | . |
| CTC2 | 10 | 21805702 | GTGGTG  GTGGTG GTGGTG | G | 1 | NM_207371 | SKIDA1 | nonframeshift_de letion | exon4 | c.1032_1049del | p.344_350del | . | . |
| CTC2 | 10 | 24835119 | CCTG | C | 0.9 | NM_001282768 | KIAA1217 | nonframeshift_de  letion | exon18 | c.3917_3919del | p.1306_1307de  l | . | . |
| CTC2 | 10 | 24880598 | GA | G | 1 | NM_020824 | ARHGAP21 | frameshift_deleti  on | exon23 | c.4016delT | p.F1339fs | . | . |
| CTC2 | 10 | 27454076 | T | TA | 0.5 | NM_001172303 | MASTL | frameshift_inserti  on | exon5 | c.637_638insA | p.S213fs | . | . |
| CTC2 | 10 | 27687534 | C | CT | 0.565 | NM_001034842 | PTCHD3 | frameshift_inserti  on | exon4 | c.1992dupA | p.V665fs | . | . |
| CTC2 | 10 | 28023489 | G | GT | 0.353 | NM_001242702 | MKX | frameshift_inserti  on | exon5 | c.733dupA | p.T245fs | . | . |
| CTC2 | 10 | 29821689 | T | TC | 0.221 | NM_021738 | SVIL | frameshift_inserti  on | exon8 | c.1606dupG | p.E536fs | . | . |
| CTC2 | 10 | 29822089 | TG | T | 0.354 | NM_021738 | SVIL | frameshift_deleti  on | exon8 | c.1206delC | p.P402fs | . | . |
| CTC2 | 10 | 34759013 | CT | C | 0.357 | NM_001184785 | PARD3 | frameshift_deleti  on | exon4 | c.581delA | p.K194fs | . | . |
| CTC2 | 10 | 34806007 | T | TG | 0.556 | NM_001184785 | PARD3 | frameshift_inserti  on | exon3 | c.302dupC | p.P101fs | . | . |
| CTC2 | 10 | 35855028 | AT | A | 0.587 | NM_001282854 | CCNY | frameshift_deleti  on | exon7 | c.426delT | p.Y142fs | . | . |
| CTC2 | 10 | 46122247 | GTA | G | 0.385 | NM_001128324 | ZFAND4 | frameshift_deleti  on | exon7 | c.1022_1023del | p.I341fs | . | . |
| CTC2 | 10 | 46264968 | TTC | T | 0.9 | NM_001169107 | FAM21C | frameshift_deleti  on | exon19 | c.1870_1871del | p.S624fs | . | . |
| CTC2 | 10 | 49943954 | TTCA | T | 0.446 | NM_020945 | WDFY4 | nonframeshift_de  letion | exon11 | c.1718_1720del | p.573_574del | . | . |
| CTC2 | 10 | 49943956 | CA | C | 0.244 | NM_020945 | WDFY4 | frameshift_deleti  on | exon11 | c.1720delA | p.I574fs | . | . |
| CTC2 | 10 | 49951551 | AC | A | 0.556 | NM_020945 | WDFY4 | frameshift_deleti  on | exon12 | c.2418delC | p.D806fs | . | . |
| CTC2 | 10 | 49986864 | G | GT | 0.246 | NM_020945 | WDFY4 | frameshift_inserti  on | exon18 | c.3385dupT | p.E1128fs | . | . |
| CTC2 | 10 | 50030463 | GC | G | 0.484 | NM_020945 | WDFY4 | frameshift_deleti  on | exon35 | c.5864delC | p.A1955fs | . | . |
| CTC2 | 10 | 51769551 | GA | G | 0.545 | NM_001077665 | AGAP6 | frameshift_deleti  on | exon8 | c.1667delA | p.E556fs | . | . |
| CTC2 | 10 | 54040664 | CT | C | 0.292 | NM_001098512 | PRKG1 | frameshift_deleti  on | exon13 | c.1475delT | p.L492fs | . | . |
| CTC2 | 10 | 61412644 | CA | C | 1 | NM_194298 | SLC16A9 | frameshift_deleti  on | exon6 | c.1415delT | p.L472fs | . | . |
| CTC2 | 10 | 61414127 | C | CT | 0.222 | NM_194298 | SLC16A9 | frameshift_inserti  on | exon5 | c.656dupA | p.K219fs | . | . |
| CTC2 | 10 | 61834575 | CT | C | 0.524 | NM_020987 | ANK3 | frameshift_deleti  on | exon37 | c.6063delA | p.K2021fs | . | . |
| CTC2 | 10 | 61842429 | CT | C | 0.266 | NM_001149 | ANK3 | frameshift_deleti  on | exon12 | c.1668delA | p.K556fs | . | . |

| CTC2 | 10 | 61905735 | T | TA | 0.8 | NM_001204404 | ANK3 | frameshift_inserti  on | exon24 | c.2607dupT | p.T870fs | . | . |
| --- | --- | --- | --- | --- | --- | --- | --- | --- | --- | --- | --- | --- | --- |
| CTC2 | 10 | 62332252 | GT | G | 0.759 | NM_001204404 | ANK3 | frameshift_deleti  on | exon1 | c.33delA | p.K11fs | . | . |
| CTC2 | 10 | 64936211 | TC | T | 0.32 | NM_001282948 | JMJD1C | frameshift_deleti  on | exon23 | c.6700delG | p.E2234fs | . | . |
| CTC2 | 10 | 64967950 | ACTAAA  C | A | 0.411 | NM_001282948 | JMJD1C | nonframeshift_de  letion | exon9 | c.2927_2932del | p.976_978del | . | . |
| CTC2 | 10 | 69672694 | TG | T | 0.232 | NM_001142498 | SIRT1 | frameshift_deleti  on | exon7 | c.937delG | p.G313fs | . | . |
| CTC2 | 10 | 69676251 | TG | T | 0.164 | NM_001142498 | SIRT1 | frameshift_deleti  on | exon8 | c.1261delG | p.G421fs | . | . |
| CTC2 | 10 | 69695974 | CA | C | 0.833 | NM_001278186 | HERC4 | frameshift_deleti  on | exon20 | c.1824delT | p.F608fs | . | . |
| CTC2 | 10 | 70098302 | AT | A | 0.389 | NM_012207 | HNRNPH3 | frameshift_deleti  on | exon4 | c.295delT | p.F99fs | . | . |
| CTC2 | 10 | 70516174 | CCG | C | 0.933 | NM_001282959 | CCAR1 | frameshift_deleti  on | exon13 | c.1726_1727del | p.R576fs | . | . |
| CTC2 | 10 | 70856980 | CT | C | 0.545 | NM_002727 | SRGN | frameshift_deleti  on | exon2 | c.221delT | p.L74fs | . | . |
| CTC2 | 10 | 71016815 | AG | A | 0.286 | NM_025130 | HKDC1 | frameshift_deleti  on | exon13 | c.1861delG | p.G621fs | . | . |
| CTC2 | 10 | 71166906 | GC | G | 0.225 | NM_001057 | TACR2 | frameshift_deleti  on | exon4 | c.871delG | p.A291fs | . | . |
| CTC2 | 10 | 73053263 | G | GT | 0.327 | NM_001244889 | UNC5B | frameshift_inserti  on | exon11 | c.1842dupT | p.C614fs | . | . |
| CTC2 | 10 | 75258907 | TCA | T | 0.824 | NM_152586 | USP54 | frameshift_deleti  on | exon22 | c.4533_4534del | p.C1511fs | . | . |
| CTC2 | 10 | 75506755 | TC | T | 0.464 | NM_198597 | SEC24C | frameshift_deleti  on | exon2 | c.166delC | p.P56fs | . | . |
| CTC2 | 10 | 75542944 | GA | G | 0.5 | NM_203298 | CHCHD1 | nonframeshift_de  letion | exon3 | c.357delA | p.X119X | . | . |
| CTC2 | 10 | 75860835 | GC | G | 0.195 | NM_003373 | VCL | frameshift_deleti  on | exon14 | c.2003delC | p.A668fs | . | . |
| CTC2 | 10 | 75898136 | AT | A | 0.649 | NM_012095 | AP3M1 | stopgain | exon2 | c.1delA | p.M1X | . | . |
| CTC2 | 10 | 76781902 | AGAG | A | 0.426 | NM_001256468 | KAT6B | nonframeshift_de letion | exon16 | c.2737_2739del | p.913_913del | . | . |
| CTC2 | 10 | 76781905 | GGAA | G | 0.724 | NM_001256468 | KAT6B | nonframeshift_de  letion | exon16 | c.2740_2742del | p.914_914del | . | . |
| CTC2 | 10 | 76972038 | AT | A | 0.711 | NM_001184823 | VDAC2 | frameshift_deleti  on | exon3 | c.83delT | p.I28fs | . | . |
| CTC2 | 10 | 78704621 | GC | G | 0.31 | NM_001271518 | KCNMA1 | frameshift_deleti  on | exon22 | c.2487delG | p.S829fs | . | . |
| CTC2 | 10 | 81904076 | GC | G | 0.615 | NM_001012973 | PLAC9 | frameshift_deleti  on | exon3 | c.261delC | p.S87fs | . | . |
| CTC2 | 10 | 85968611 | GA | G | 0.292 | NM_001171971 | CDHR1 | frameshift_deleti  on | exon12 | c.1295delA | p.E432fs | . | . |
| CTC2 | 10 | 86018325 | AT | A | 0.226 | NM_001012722 | RGR | frameshift_deleti  on | exon6 | c.693delT | p.N231fs | . | . |
| CTC2 | 10 | 88220961 | CA | C | 0.5 | NM_015045 | WAPAL | frameshift_deleti  on | exon10 | c.2453delT | p.L818fs | . | . |
| CTC2 | 10 | 89272897 | CT | C | 0.571 | NM_001178118 | MINPP1 | frameshift_deleti  on | exon3 | c.250delT | p.F84fs | . | . |
| CTC2 | 10 | 91144165 | A | AT | 0.169 | NM_001010987 | IFIT1B | frameshift_inserti  on | exon2 | c.1095_1096ins  T | p.K365fs | . | . |
| CTC2 | 10 | 91198667 | AC | A | 0.235 | NM_213606 | SLC16A12 | frameshift_deleti  on | exon6 | c.721delG | p.V241fs | . | . |
| CTC2 | 10 | 93752002 | TA | T | 1 | NM_003972 | BTAF1 | frameshift_deleti  on | exon21 | c.2982delA | p.V994fs | . | . |
| CTC2 | 10 | 94235690 | AT | A | 0.6 | NM_001165946 | IDE | frameshift_deleti  on | exon6 | c.280delA | p.M94fs | . | . |
| CTC2 | 10 | 94833823 | G | GC | 0.667 | NM_000783 | CYP26A1 | frameshift_inserti  on | exon1 | c.133dupC | p.L44fs | . | . |
| CTC2 | 10 | 95287825 | TG | T | 0.3 | NM_001127182 | CEP55 | frameshift_deleti  on | exon9 | c.1311delG | p.L437fs | . | . |
| CTC2 | 10 | 95347130 | AT | A | 0.255 | NM_001195755 | FFAR4 | frameshift_deleti  on | exon3 | c.851delT | p.I284fs | . | . |
| CTC2 | 10 | 95556984 | A | AT | 0.19 | NM_005097 | LGI1 | frameshift_inserti  on | exon8 | c.1099dupT | p.G366fs | . | . |
| CTC2 | 10 | 97081762 | TG | T | 0.3 | NM_006434 | SORBS1 | frameshift_deleti  on | exon19 | c.1831delC | p.Q611fs | . | . |
| CTC2 | 10 | 97114673 | TC | T | 0.333 | NM_001034957 | SORBS1 | frameshift_deleti  on | exon14 | c.1297delG | p.E433fs | . | . |
| CTC2 | 10 | 97373537 | AT | A | 0.327 | NM_001017423 | ALDH18A1 | frameshift_deleti  on | exon15 | c.1878delA | p.P626fs | . | . |
| CTC2 | 10 | 98743884 | TC | T | 0.251 | NM_015652 | C10orf12 | frameshift_deleti  on | exon1 | c.2738delC | p.S913fs | . | . |
| CTC2 | 10 | 98807537 | TG | T | 0.455 | NM_003061 | SLIT1 | frameshift_deleti  on | exon16 | c.1543delC | p.H515fs | . | . |
| CTC2 | 10 | 99126263 | CT | C | 0.871 | NM_001284337 | RRP12 | frameshift_deleti  on | exon25 | c.3030delA | p.K1010fs | . | . |
| CTC2 | 10 | 99126521 | GCTC | G | 0.351 | NM_001284337 | RRP12 | nonframeshift_de  letion | exon24 | c.2890_2892del | p.964_964del | . | . |
| CTC2 | 10 | 99148316 | C | CT | 0.269 | NM_001284337 | RRP12 | frameshift_inserti  on | exon4 | c.518dupA | p.K173fs | . | . |
| CTC2 | 10 | 100195419 | AT | A | 0.452 | NM_000195 | HPS1 | frameshift_deleti  on | exon4 | c.227delA | p.N76fs | . | . |
| CTC2 | 10 | 101439115 | AT | A | 0.366 | NM_020354 | ENTPD7 | frameshift_deleti  on | exon4 | c.290delT | p.I97fs | . | . |
| CTC2 | 10 | 102005629 | C | CT | 0.6 | NM_018294 | CWF19L1 | frameshift_inserti  on | exon9 | c.890dupA | p.K297fs | . | . |
| CTC2 | 10 | 102258462 | CT | C | 0.27 | NM_015490 | SEC31B | frameshift_deleti  on | exon14 | c.1683delA | p.K561fs | . | . |
| CTC2 | 10 | 102698442 | CTT | C | 0.4 | NM_001136123 | FAM178A | frameshift_deleti  on | exon11 | c.2604_2605del | p.S868fs | . | . |
| CTC2 | 10 | 102744439 | G | GT | 0.895 | NM_001203244 | SEMA4G | frameshift_inserti  on | exon14 | c.1899dupT | p.C633fs | . | . |
| CTC2 | 10 | 102749046 | TG | T | 0.667 | NM_001163812 | C10orf2 | frameshift_deleti  on | exon1 | c.1080delG | p.L360fs | . | . |
| CTC2 | 10 | 102777933 | C | CT | 0.326 | NM_001195263 | PDZD7 | frameshift_inserti  on | exon9 | c.1444_1445ins  A | p.R482fs | . | . |
| CTC2 | 10 | 102798448 | TG | T | 0.975 | NM_030971 | SFXN3 | frameshift_deleti  on | exon10 | c.833delG | p.C278fs | . | . |
| CTC2 | 10 | 102896663 | GC | G | 1 | NM_005521 | TLX1 | frameshift_deleti  on | exon3 | c.987delC | p.C329fs | . | . |
| CTC2 | 10 | 103567646 | CA | C | 1 | NM_001142434 | MGEA5 | frameshift_deleti  on | exon5 | c.492delT | p.F164fs | . | . |
| CTC2 | 10 | 104123526 | G | GA | 0.684 | NM_001199378 | GBF1 | frameshift_inserti  on | exon17 | c.2078dupA | p.E693fs | . | . |
| CTC2 | 10 | 104129005 | CT | C | 1 | NM_001199378 | GBF1 | frameshift_deleti  on | exon24 | c.3012delT | p.P1004fs | . | . |
| CTC2 | 10 | 104164361 | GC | G | 0.357 | NM_002779 | PSD | frameshift_deleti  on | exon15 | c.2678delG | p.S893fs | . | . |

| CTC2 | 10 | 105238785 | TC | T | 0.587 | NM_001129742 | CALHM3 | frameshift_deleti  on | exon1 | c.4delG | p.D2fs | . | . |
| --- | --- | --- | --- | --- | --- | --- | --- | --- | --- | --- | --- | --- | --- |
| CTC2 | 10 | 108459005 | GCC | G | 0.132 | NM_001013031 | SORCS1 | frameshift_deleti  on | exon9 | c.1378_1379del | p.G460fs | . | . |
| CTC2 | 10 | 108489835 | GCA | G | 0.623 | NM_001013031 | SORCS1 | frameshift_deleti  on | exon6 | c.995_996del | p.V332fs | . | . |
| CTC2 | 10 | 111630602 | TG | T | 0.8 | NM_001167604 | XPNPEP1 | frameshift_deleti  on | exon17 | c.1510delC | p.H504fs | . | . |
| CTC2 | 10 | 112635817 | TA | T | 0.333 | NM_001199492 | PDCD4 | frameshift_deleti  on | exon2 | c.33delA | p.V11fs | . | . |
| CTC2 | 10 | 115609254 | AT | A | 0.578 | NM_014881 | DCLRE1A | frameshift_deleti  on | exon2 | c.1609delA | p.I537fs | . | . |
| CTC2 | 10 | 115639361 | AT | A | 0.243 | NM_198514 | NHLRC2 | frameshift_deleti  on | exon4 | c.817delT | p.F273fs | . | . |
| CTC2 | 10 | 115894725 | CT | C | 0.212 | NM_018017 | C10orf118 | frameshift_deleti  on | exon10 | c.1601delA | p.Q534fs | . | . |
| CTC2 | 10 | 116251613 | TG | T | 0.576 | NM_001003407 | ABLIM1 | frameshift_deleti  on | exon7 | c.738delC | p.P246fs | . | . |
| CTC2 | 10 | 118630761 | G | GC | 0.538 | NM_001242699 | ENO4 | frameshift_inserti  on | exon9 | c.1174dupC | p.L391fs | . | . |
| CTC2 | 10 | 121189899 | CA | C | 0.469 | NM_005308 | GRK5 | frameshift_deleti  on | exon7 | c.547delA | p.K183fs | . | . |
| CTC2 | 10 | 122664849 | GC | G | 0.667 | NM_018117 | WDR11 | frameshift_deleti  on | exon26 | c.3213delC | p.C1071fs | . | . |
| CTC2 | 10 | 123247592 | CA | C | 0.643 | NM_001144914 | FGFR2 | frameshift_deleti  on | exon11 | c.1562delT | p.L521fs | . | . |
| CTC2 | 10 | 123846917 | TTC | T | 0.17 | NM_206862 | TACC2 | frameshift_deleti  on | exon4 | c.4903_4904del | p.S1635fs | . | . |
| CTC2 | 10 | 123954640 | GA | G | 0.212 | NM_006997 | TACC2 | frameshift_deleti  on | exon2 | c.155delA | p.E52fs | . | . |
| CTC2 | 10 | 124457529 | A | AT | 0.694 | NM_001010912 | C10orf120 | frameshift_inserti  on | exon3 | c.727dupA | p.M243fs | . | . |
| CTC2 | 10 | 124753858 | GT | G | 0.322 | NM_001271840 | IKZF5 | frameshift_deleti  on | exon5 | c.697delA | p.T233fs | . | . |
| CTC2 | 10 | 124800159 | AC | A | 0.5 | NM_001609 | ACADSB | frameshift_deleti  on | exon4 | c.482delC | p.T161fs | . | . |
| CTC2 | 10 | 125539690 | A | AT | 0.178 | NM_198148 | CPXM2 | frameshift_inserti  on | exon7 | c.970dupA | p.M324fs | . | . |
| CTC2 | 10 | 126523147 | C | CA | 0.25 | NM_032182 | FAM175B | frameshift_inserti  on | exon9 | c.855_856insA | p.S285fs | . | . |
| CTC2 | 10 | 126678237 | TG | T | 0.5 | NM_022802 | CTBP2 | frameshift_deleti  on | exon9 | c.2807delC | p.P936fs | . | . |
| CTC2 | 10 | 127782681 | CA | C | 0.276 | NM_001288973 | ADAM12 | frameshift_deleti  on | exon11 | c.1017delT | p.L339fs | . | . |
| CTC2 | 10 | 128836023 | CCTG | C | 0.257 | NM_001380 | DOCK1 | nonframeshift_de  letion | exon19 | c.1891_1893del | p.631_631del | . | . |
| CTC2 | 10 | 129877957 | CT | C | 0.735 | NM_130435 | PTPRE | frameshift_deleti  on | exon17 | c.1853delT | p.L618fs | . | . |
| CTC2 | 10 | 129902930 | GT | G | 0.462 | NM_001145966 | MKI67 | frameshift_deleti  on | exon12 | c.6093delA | p.K2031fs | . | . |
| CTC2 | 10 | 129904132 | GT | G | 0.286 | NM_001145966 | MKI67 | frameshift_deleti  on | exon12 | c.4891delA | p.T1631fs | . | . |
| CTC2 | 10 | 129905111 | CTG | C | 0.266 | NM_001145966 | MKI67 | frameshift_deleti  on | exon12 | c.3911_3912del | p.T1304fs | . | . |
| CTC2 | 10 | 134004301 | CT | C | 1 | NM_006426 | DPYSL4 | frameshift_deleti  on | exon2 | c.91delT | p.F31fs | . | . |
| CTC2 | 10 | 135053549 | GTCT | G | 0.926 | NM_014468 | VENTX | nonframeshift_de  letion | exon3 | c.517_519del | p.173_173del | . | . |
| CTC2 | 10 | 135193981 | CT | C | 0.304 | NM_152911 | PAOX | frameshift_deleti  on | exon2 | c.661delT | p.F221fs | . | . |
| CTC2 | 11 | 1263535 | G | GA | 0.392 | NM_002458 | MUC5B | frameshift_inserti  on | exon31 | c.5426dupA | p.E1809fs | . | . |
| CTC2 | 11 | 1619172 | ACCCCC  ACAGGA GCCACA GCCCCC | A | 0.975 | NM_001004325 | KRTAP5-2 | nonframeshift_de letion | exon1 | c.279_308del | p.93_103del | . | . |
| CTC2 | 11 | 2320812 | G | GA | 0.427 | NM_001142946 | C11orf21 | frameshift_inserti  on | exon4 | c.410_411insT | p.C137fs | . | . |
| CTC2 | 11 | 3125542 | CA | C | 1 | NM_001144063 | OSBPL5 | frameshift_deleti  on | exon9 | c.920delT | p.L307fs | . | . |
| CTC2 | 11 | 4592706 | T | TAC | 0.885 | NM_144663 | C11orf40 | frameshift_inserti  on | exon4 | c.600_601insGT | p.M201fs | . | . |
| CTC2 | 11 | 4703914 | TG | T | 0.28 | NM_030774 | OR51E2 | frameshift_deleti  on | exon2 | c.27delC | p.A9fs | . | . |
| CTC2 | 11 | 5068506 | GT | G | 1 | NM_001001916 | OR52J3 | frameshift_deleti  on | exon1 | c.752delT | p.V251fs | . | . |
| CTC2 | 11 | 5153584 | CA | C | 0.623 | NM_001005160 | OR52A5 | frameshift_deleti  on | exon1 | c.288delT | p.F96fs | . | . |
| CTC2 | 11 | 5364070 | TG | T | 0.7 | NM_001005567 | OR51B5 | frameshift_deleti  on | exon5 | c.684delC | p.S228fs | . | . |
| CTC2 | 11 | 5529233 | CG | C | 0.429 | NM_017481 | UBQLN3 | frameshift_deleti  on | exon2 | c.1555delC | p.R519fs | . | . |
| CTC2 | 11 | 5529917 | CTGG | C | 0.206 | NM_017481 | UBQLN3 | nonframeshift_de  letion | exon2 | c.869_871del | p.290_291del | . | . |
| CTC2 | 11 | 5530202 | TG | T | 0.737 | NM_017481 | UBQLN3 | frameshift_deleti  on | exon2 | c.586delC | p.H196fs | . | . |
| CTC2 | 11 | 5799066 | TGAA | T | 0.105 | NM_001001922 | OR52N5 | nonframeshift_de  letion | exon1 | c.796_798del | p.266_266del | . | . |
| CTC2 | 11 | 6221033 | G | GA | 0.25 | NM_001005178 | OR52W1 | frameshift_inserti  on | exon1 | c.580_581insA | p.V194fs | . | . |
| CTC2 | 11 | 6520136 | GA | G | 1 | NM_173589 | DNHD1 | frameshift_deleti  on | exon2 | c.692delA | p.E231fs | . | . |
| CTC2 | 11 | 6570073 | CA | C | 0.2 | NM_144666 | DNHD1 | frameshift_deleti  on | exon24 | c.7298delA | p.Q2433fs | . | . |
| CTC2 | 11 | 6638543 | TGG | T | 0.783 | NM_000391 | TPP1 | frameshift_deleti  on | exon5 | c.495_496del | p.P165fs | . | . |
| CTC2 | 11 | 6653530 | CT | C | 1 | NM_003737 | DCHS1 | frameshift_deleti  on | exon6 | c.3212delA | p.K1071fs | . | . |
| CTC2 | 11 | 6897911 | AT | A | 0.278 | NM_207186 | OR10A4 | frameshift_deleti  on | exon1 | c.34delT | p.F12fs | . | . |
| CTC2 | 11 | 7070911 | TA | T | 0.979 | NM_176822 | NLRP14 | frameshift_deleti  on | exon6 | c.2134delA | p.I712fs | . | . |
| CTC2 | 11 | 7661047 | TCTC | T | 1 | NM_001256569 | PPFIBP2 | nonframeshift_de  letion | exon11 | c.893_895del | p.298_299del | . | . |
| CTC2 | 11 | 8248625 | AG | A | 0.183 | NM_001270428 | LMO1 | frameshift_deleti  on | exon3 | c.258delC | p.N86fs | . | . |
| CTC2 | 11 | 8642789 | CCAG | C | 0.286 | NM_014818 | TRIM66 | nonframeshift_de  letion | exon15 | c.2804_2806del | p.935_936del | . | . |
| CTC2 | 11 | 8974792 | GA | G | 0.429 | NM_001286094 | TMEM9B | frameshift_deleti  on | exon3 | c.176delT | p.L59fs | . | . |
| CTC2 | 11 | 8985843 | TC | T | 0.292 | NM_020644 | TMEM9B | frameshift_deleti  on | exon1 | c.17delG | p.G6fs | . | . |
| CTC2 | 11 | 9806778 | TG | T | 1 | NM_030962 | SBF2 | frameshift_deleti  on | exon37 | c.5124delC | p.D1708fs | . | . |

| CTC2 | 11 | 9864200 | AG | A | 0.333 | NM_030962 | SBF2 | frameshift_deleti  on | exon25 | c.3227delC | p.P1076fs | . | . |
| --- | --- | --- | --- | --- | --- | --- | --- | --- | --- | --- | --- | --- | --- |
| CTC2 | 11 | 10789895 | GT | G | 0.304 | NM_014633 | CTR9 | frameshift_deleti  on | exon16 | c.1967delT | p.V656fs | . | . |
| CTC2 | 11 | 10874907 | GT | G | 0.303 | NM_001143667 | ZBED5 | frameshift_deleti  on | exon3 | c.1585delA | p.T529fs | . | . |
| CTC2 | 11 | 11314614 | TG | T | 0.833 | NM_198516 | GALNT18 | frameshift_deleti  on | exon10 | c.1638delC | p.A546fs | . | . |
| CTC2 | 11 | 11988568 | G | GC | 0.857 | NM_001018057 | DKK3 | frameshift_inserti  on | exon5 | c.605dupG | p.G202fs | . | . |
| CTC2 | 11 | 12316039 | AG | A | 0.524 | NM_032867 | MICALCL | frameshift_deleti  on | exon3 | c.1062delG | p.K354fs | . | . |
| CTC2 | 11 | 12316344 | G | GCTCC  TC | 1 | NM_032867 | MICALCL | nonframeshift_in  sertion | exon3 | c.1366_1367ins  CTCCTC | p.A456delinsA  PP | . | . |
| CTC2 | 11 | 12379863 | ATC | A | 0.156 | NM_032867 | MICALCL | frameshift_deleti  on | exon9 | c.1926_1927del | p.D642fs | . | . |
| CTC2 | 11 | 13435149 | G | GA | 0.25 | NM_032320 | BTBD10 | frameshift_inserti  on | exon6 | c.735dupT | p.P246fs | . | . |
| CTC2 | 11 | 14902150 | CA | C | 0.645 | NM_024514 | CYP2R1 | frameshift_deleti  on | exon3 | c.531delT | p.F177fs | . | . |
| CTC2 | 11 | 17352476 | TTACA | T | 0.917 | NM_005013 | NUCB2 | frameshift_deleti  on | exon13 | c.1202_1205del | p.L401fs | . | . |
| CTC2 | 11 | 17352477 | TACA | T | 1 | NM_005013 | NUCB2 | nonframeshift_de  letion | exon13 | c.1203_1205del | p.401_402del | . | . |
| CTC2 | 11 | 17424214 | GC | G | 0.365 | NM_000352 | ABCC8 | frameshift_deleti  on | exon29 | c.3643delG | p.A1215fs | . | . |
| CTC2 | 11 | 17580690 | AC | A | 0.24 | NM_001277269 | OTOG | frameshift_deleti  on | exon10 | c.1232delC | p.T411fs | . | . |
| CTC2 | 11 | 17653757 | GC | G | 0.667 | NM_001277269 | OTOG | frameshift_deleti  on | exon41 | c.7093delC | p.R2365fs | . | . |
| CTC2 | 11 | 18010190 | CT | C | 0.6 | NM_012139 | SERGEF | frameshift_deleti  on | exon8 | c.797delA | p.K266fs | . | . |
| CTC2 | 11 | 18127530 | A | ACCT | 1 | NM_138421 | SAAL1 | nonframeshift_in  sertion | exon1 | c.58_59insAGG | p.V20delinsEV | . | . |
| CTC2 | 11 | 18127530 | A | ACCTC  CT | 1 | NM_138421 | SAAL1 | nonframeshift_in  sertion | exon1 | c.58_59insAGG  AGG | p.V20delinsEEV | . | . |
| CTC2 | 11 | 19955331 | TG | T | 0.6 | NM_001111018 | NAV2 | frameshift_deleti  on | exon7 | c.1350delG | p.L450fs | . | . |
| CTC2 | 11 | 19955466 | C | CT | 0.5 | NM_001111018 | NAV2 | frameshift_inserti  on | exon7 | c.1485dupT | p.P495fs | . | . |
| CTC2 | 11 | 20388924 | TA | T | 0.667 | NM_001098523 | HTATIP2 | nonframeshift_de  letion | exon2 | c.401delA | p.X134X | . | . |
| CTC2 | 11 | 20699599 | AT | A | 0.949 | NM_001288714 | NELL1 | frameshift_deleti  on | exon2 | c.178delT | p.F60fs | . | . |
| CTC2 | 11 | 26587126 | CTG | C | 0.287 | NM_145650 | MUC15 | frameshift_deleti  on | exon2 | c.278_279del | p.A93fs | . | . |
| CTC2 | 11 | 26719984 | CA | C | 0.407 | NM_178498 | SLC5A12 | frameshift_deleti  on | exon7 | c.919delT | p.W307fs | . | . |
| CTC2 | 11 | 27114815 | GAA | G | 1 | NM_003986 | BBOX1 | frameshift_deleti  on | exon5 | c.436_437del | p.K146fs | . | . |
| CTC2 | 11 | 27679650 | CT | C | 0.367 | NM_170735 | BDNF | frameshift_deleti  on | exon1 | c.461delA | p.K154fs | . | . |
| CTC2 | 11 | 28351963 | AT | A | 0.875 | NM_001113528 | METTL15 | frameshift_deleti  on | exon7 | c.800delT | p.I267fs | . | . |
| CTC2 | 11 | 33054507 | G | GA | 0.625 | NM_001077242 | DEPDC7 | frameshift_inserti  on | exon8 | c.1337dupA | p.D446fs | . | . |
| CTC2 | 11 | 33094050 | AC | A | 0.79 | NM_001145541 | TCP11L1 | frameshift_deleti  on | exon10 | c.1359delC | p.Y453fs | . | . |
| CTC2 | 11 | 33720047 | C | CT | 0.333 | NM_001166692 | C11orf91 | frameshift_inserti  on | exon2 | c.509dupA | p.K170fs | . | . |
| CTC2 | 11 | 35218402 | C | CACA | 0.32 | NM_000610 | CD44 | nonframeshift_in  sertion | exon6 | c.777_778insAC  A | p.H259delinsH  T | . | . |
| CTC2 | 11 | 35218404 | C | CAAG | 0.28 | NM_000610 | CD44 | nonframeshift_in  sertion | exon6 | c.779_780insAA  G | p.T260delinsTR | . | . |
| CTC2 | 11 | 40136150 | TC | T | 0.455 | NM_020929 | LRRC4C | frameshift_deleti  on | exon5 | c.1692delG | p.R564fs | . | . |
| CTC2 | 11 | 40137730 | AC | A | 0.571 | NM_020929 | LRRC4C | frameshift_deleti  on | exon5 | c.112delG | p.V38fs | . | . |
| CTC2 | 11 | 43351531 | CG | C | 0.545 | NM_001243747 | API5 | frameshift_deleti  on | exon7 | c.626delG | p.R209fs | . | . |
| CTC2 | 11 | 43413477 | AC | A | 0.544 | NM_018259 | TTC17 | frameshift_deleti  on | exon5 | c.639delC | p.H213fs | . | . |
| CTC2 | 11 | 44089412 | GA | G | 0.333 | NM_001127219 | ACCS | frameshift_deleti  on | exon2 | c.236delA | p.E79fs | . | . |
| CTC2 | 11 | 45247687 | TC | T | 0.615 | NM_001256695 | PRDM11 | frameshift_deleti  on | exon8 | c.1512delC | p.I504fs | . | . |
| CTC2 | 11 | 45247698 | AAGC | A | 0.462 | NM_001256695 | PRDM11 | nonframeshift_de  letion | exon8 | c.1523_1525del | p.508_509del | . | . |
| CTC2 | 11 | 46396001 | CG | C | 1 | NM_001199268 | DGKZ | frameshift_deleti  on | exon15 | c.1390delG | p.G464fs | . | . |
| CTC2 | 11 | 46398073 | GC | G | 0.593 | NM_001199268 | DGKZ | frameshift_deleti  on | exon23 | c.2093delC | p.A698fs | . | . |
| CTC2 | 11 | 46456492 | TG | T | 0.337 | NM_001267783 | AMBRA1 | frameshift_deleti  on | exon13 | c.2370delC | p.P790fs | . | . |
| CTC2 | 11 | 46724716 | CAGTGG  TGACAG | C | 1 | NM_001184751 | ZNF408 | nonframeshift_de  letion | exon4 | c.552_563del | p.184_188del | . | . |
| CTC2 | 11 | 46772949 | TG | T | 0.875 | NM_014756 | CKAP5 | frameshift_deleti  on | exon38 | c.5088delC | p.P1696fs | . | . |
| CTC2 | 11 | 47504337 | C | CT | 0.231 | NM_001025596 | CELF1 | frameshift_inserti  on | exon6 | c.596dupA | p.Q199fs | . | . |
| CTC2 | 11 | 47712063 | GTA | G | 1 | NM_024783 | AGBL2 | frameshift_deleti  on | exon10 | c.1194_1195del | p.Y398fs | . | . |
| CTC2 | 11 | 48149449 | CG | C | 0.457 | NM_001098503 | PTPRJ | frameshift_deleti  on | exon7 | c.1212delG | p.A404fs | . | . |
| CTC2 | 11 | 48285980 | ACCT | A | 0.978 | NM_001004726 | OR4X1 | nonframeshift_de  letion | exon1 | c.569_571del | p.190_191del | . | . |
| CTC2 | 11 | 48285981 | CCTT | C | 1 | NM_001004726 | OR4X1 | nonframeshift_de  letion | exon1 | c.570_572del | p.190_191del | . | . |
| CTC2 | 11 | 48286317 | GA | G | 0.505 | NM_001004726 | OR4X1 | frameshift_deleti  on | exon1 | c.906delA | p.G302fs | . | . |
| CTC2 | 11 | 55681973 | GC | G | 0.278 | NM_001001960 | OR5W2 | frameshift_deleti  on | exon1 | c.85delG | p.A29fs | . | . |
| CTC2 | 11 | 56757033 | CT | C | 0.283 | NM_001005323 | OR5AK2 | frameshift_deleti  on | exon1 | c.646delT | p.F216fs | . | . |
| CTC2 | 11 | 56757122 | TG | T | 0.226 | NM_001005323 | OR5AK2 | frameshift_deleti  on | exon1 | c.735delG | p.L245fs | . | . |
| CTC2 | 11 | 56949679 | AG | A | 0.316 | NM_001005210 | LRRC55 | frameshift_deleti  on | exon1 | c.313delG | p.D105fs | . | . |
| CTC2 | 11 | 57088152 | TTTGGG  C | T | 0.286 | NM_033396 | TNKS1BP1 | nonframeshift_de  letion | exon3 | c.123_128del | p.41_43del | . | . |
| CTC2 | 11 | 57088169 | GT | G | 0.857 | NM_033396 | TNKS1BP1 | frameshift_deleti  on | exon3 | c.111delA | p.K37fs | . | . |

| CTC2 | 11 | 57506670 | GA | G | 0.7 | NM_001144012 | TMX2 | frameshift_deleti  on | exon6 | c.569delA | p.E190fs | . | . |
| --- | --- | --- | --- | --- | --- | --- | --- | --- | --- | --- | --- | --- | --- |
| CTC2 | 11 | 57506728 | CTG | C | 0.211 | NM_001144012 | TMX2 | frameshift_deleti  on | exon6 | c.627_628del | p.S209fs | . | . |
| CTC2 | 11 | 57512799 | TG | T | 0.5 | NM_001145101 | BTBD18 | frameshift_deleti  on | exon3 | c.945delC | p.G315fs | . | . |
| CTC2 | 11 | 57564114 | T | TCC | 0.6 | NM_001085469 | CTNND1 | frameshift_inserti  on | exon3 | c.303_304insCC | p.L101fs | . | . |
| CTC2 | 11 | 57958863 | ACT | A | 1 | NM_001005283 | OR9Q2 | frameshift_deleti  on | exon1 | c.902_903del | p.T301fs | . | . |
| CTC2 | 11 | 57995628 | GC | G | 0.8 | NM_001004471 | OR10Q1 | frameshift_deleti  on | exon1 | c.719delG | p.R240fs | . | . |
| CTC2 | 11 | 57995878 | AG | A | 0.8 | NM_001004471 | OR10Q1 | frameshift_deleti  on | exon1 | c.469delC | p.L157fs | . | . |
| CTC2 | 11 | 58125677 | GT | G | 0.312 | NM_001005489 | OR5B17 | frameshift_deleti  on | exon1 | c.865delA | p.T289fs | . | . |
| CTC2 | 11 | 58491889 | CT | C | 0.194 | NM_005838 | GLYAT | frameshift_deleti  on | exon2 | c.80delA | p.K27fs | . | . |
| CTC2 | 11 | 58893009 | TC | T | 0.234 | NM_001142704 | FAM111B | frameshift_deleti  on | exon2 | c.1350delC | p.F450fs | . | . |
| CTC2 | 11 | 59419938 | TG | T | 0.258 | NM_152716 | PATL1 | frameshift_deleti  on | exon11 | c.1424delC | p.P475fs | . | . |
| CTC2 | 11 | 59950642 | CT | C | 0.273 | NM_001247999 | MS4A6A | frameshift_deleti  on | exon2 | c.36delA | p.K12fs | . | . |
| CTC2 | 11 | 60183013 | AT | A | 0.215 | NM_001079692 | MS4A14 | frameshift_deleti  on | exon4 | c.522delT | p.D174fs | . | . |
| CTC2 | 11 | 60689545 | GC | G | 0.263 | NM_024092 | TMEM109 | frameshift_deleti  on | exon4 | c.641delC | p.A214fs | . | . |
| CTC2 | 11 | 61120545 | GT | G | 0.292 | NM_001161452 | CYB561A3 | frameshift_deleti  on | exon3 | c.459delA | p.K153fs | . | . |
| CTC2 | 11 | 61254489 | CAG | C | 0.159 | NM_001170753 | PPP1R32 | frameshift_deleti  on | exon9 | c.845_846del | p.Q282fs | . | . |
| CTC2 | 11 | 61673956 | TG | T | 0.4 | NM_013401 | RAB3IL1 | frameshift_deleti  on | exon5 | c.638delC | p.P213fs | . | . |
| CTC2 | 11 | 62340164 | AGC | A | 0.5 | NM_001404 | EEF1G | frameshift_deleti  on | exon2 | c.61_62del | p.A21fs | . | . |
| CTC2 | 11 | 62343420 | GC | G | 0.714 | NM_022830 | TUT1 | frameshift_deleti  on | exon9 | c.1884delG | p.G628fs | . | . |
| CTC2 | 11 | 62484621 | CT | C | 1 | NM_001079559 | HNRNPUL2 | frameshift_deleti  on | exon11 | c.1820delA | p.E607fs | . | . |
| CTC2 | 11 | 63586018 | AG | A | 0.407 | NM_138471 | C11orf84 | frameshift_deleti  on | exon4 | c.628delG | p.G210fs | . | . |
| CTC2 | 11 | 63974882 | TGG | T | 0.531 | NM_031471 | FERMT3 | frameshift_deleti  on | exon2 | c.47_48del | p.W16fs | . | . |
| CTC2 | 11 | 64051746 | GA | G | 0.641 | NM_004322 | BAD | frameshift_deleti  on | exon1 | c.94delT | p.S32fs | . | . |
| CTC2 | 11 | 64508453 | CG | C | 0.773 | NM_001098670 | RASGRP2 | frameshift_deleti  on | exon5 | c.337delC | p.R113fs | . | . |
| CTC2 | 11 | 64595197 | CA | C | 1 | NM_017525 | CDC42BPG | frameshift_deleti  on | exon31 | c.4025delT | p.V1342fs | . | . |
| CTC2 | 11 | 64880873 | TG | T | 1 | NM_001277233 | TM7SF2 | frameshift_deleti  on | exon4 | c.487delG | p.G163fs | . | . |
| CTC2 | 11 | 64896081 | TG | T | 0.368 | NM_032431 | SYVN1 | frameshift_deleti  on | exon15 | c.1697delC | p.P566fs | . | . |
| CTC2 | 11 | 64896185 | GCCT | G | 0.37 | NM_032431 | SYVN1 | frameshift_deleti  on | exon15 | c.1593_1593del | p.G531fs | . | . |
| CTC2 | 11 | 64939447 | A | AC | 0.328 | NM_001008778 | SPDYC | frameshift_inserti  on | exon3 | c.234dupC | p.D78fs | . | . |
| CTC2 | 11 | 64940333 | CTG | C | 0.439 | NM_001008778 | SPDYC | frameshift_deleti  on | exon6 | c.696_697del | p.P232fs | . | . |
| CTC2 | 11 | 65397121 | C | CT | 0.348 | NM_032223 | PCNXL3 | frameshift_inserti  on | exon26 | c.4132dupT | p.A1377fs | . | . |
| CTC2 | 11 | 65416785 | GCC | G | 0.645 | NM_006747 | SIPA1 | frameshift_deleti  on | exon10 | c.2360_2361del | p.A787fs | . | . |
| CTC2 | 11 | 65819899 | TGCC | T | 0.5 | NM_006842 | SF3B2 | nonframeshift_de  letion | exon1 | c.45_47del | p.15_16del | . | . |
| CTC2 | 11 | 66032657 | GC | G | 0.426 | NM_001134774 | KLC2 | frameshift_deleti  on | exon10 | c.1055delC | p.A352fs | . | . |
| CTC2 | 11 | 66136563 | GAGA | G | 0.95 | NM_001532 | SLC29A2 | nonframeshift_de  letion | exon4 | c.380_382del | p.127_128del | . | . |
| CTC2 | 11 | 66189965 | AC | A | 0.55 | NM_178864 | NPAS4 | frameshift_deleti  on | exon3 | c.372delC | p.D124fs | . | . |
| CTC2 | 11 | 66190608 | TG | T | 0.353 | NM_178864 | NPAS4 | frameshift_deleti  on | exon5 | c.714delG | p.L238fs | . | . |
| CTC2 | 11 | 66359102 | GC | G | 0.467 | NM_018219 | CCDC87 | frameshift_deleti  on | exon1 | c.1384delG | p.A462fs | . | . |
| CTC2 | 11 | 66472649 | TG | T | 1 | NM_006946 | SPTBN2 | frameshift_deleti  on | exon14 | c.2097delC | p.L699fs | . | . |
| CTC2 | 11 | 67067528 | AC | A | 1 | NM_207354 | ANKRD13D | frameshift_deleti  on | exon10 | c.1008delC | p.D336fs | . | . |
| CTC2 | 11 | 67163486 | AT | A | 0.354 | NM_001243224 | RAD9A | frameshift_deleti  on | exon4 | c.436delT | p.F146fs | . | . |
| CTC2 | 11 | 67166423 | GC | G | 0.55 | NM_206873 | PPP1CA | frameshift_deleti  on | exon4 | c.602delG | p.C201fs | . | . |
| CTC2 | 11 | 67173096 | TG | T | 0.958 | NM_001256508 | TBC1D10C | stopgain | exon5 | c.392delG | p.W131X | . | . |
| CTC2 | 11 | 67191498 | TCA | T | 0.308 | NM_020811 | CARNS1 | frameshift_deleti on | exon9 | c.1911_1912del | p.F637fs | . | . |
| CTC2 | 11 | 67200255 | A | AT | 1 | NM_003952 | RPS6KB2 | frameshift_inserti  on | exon7 | c.563_564insT | p.Q188fs | . | . |
| CTC2 | 11 | 67262358 | TG | T | 1 | NM_001130848 | PITPNM1 | frameshift_deleti  on | exon18 | c.2697delC | p.P899fs | . | . |
| CTC2 | 11 | 67412574 | GA | G | 0.739 | NM_080658 | ACY3 | frameshift_deleti  on | exon6 | c.575delT | p.F192fs | . | . |
| CTC2 | 11 | 67838198 | TTG | T | 0.3 | NM_212469 | CHKA | frameshift_deleti  on | exon4 | c.701_702del | p.T234fs | . | . |
| CTC2 | 11 | 67939053 | GT | G | 0.34 | NM_016028 | SUV420H1 | frameshift_deleti  on | exon7 | c.776delA | p.N259fs | . | . |
| CTC2 | 11 | 68527088 | TC | T | 0.265 | NM_001031847 | CPT1A | frameshift_deleti  on | exon18 | c.2183delG | p.G728fs | . | . |
| CTC2 | 11 | 68704021 | AC | A | 0.824 | NM_002180 | IGHMBP2 | frameshift_deleti  on | exon13 | c.2074delC | p.Q692fs | . | . |
| CTC2 | 11 | 70052293 | GGCT | G | 0.135 | NM_003824 | FADD | nonframeshift_de  letion | exon2 | c.342_344del | p.114_115del | . | . |
| CTC2 | 11 | 70319400 | TG | T | 0.588 | NM_133266 | SHANK2 | frameshift_deleti  on | exon11 | c.3359delC | p.P1120fs | . | . |
| CTC2 | 11 | 71293505 | AGCAGC  AGGGCT TGCAGC AGCTGG | A | 0.348 | NM_001005405 | KRTAP5-11 | nonframeshift_de letion | exon1 | c.352_378del | p.118_126del | . | . |
| CTC2 | 11 | 71726703 | TG | T | 0.857 | NM_006185 | NUMA1 | frameshift_deleti  on | exon15 | c.1845delC | p.A615fs | . | . |
| CTC2 | 11 | 72406496 | GC | G | 1 | NM_001135190 | ARAP1 | frameshift_deleti  on | exon23 | c.2593delG | p.A865fs | . | . |

| CTC2 | 11 | 73021625 | AT | A | 1 | NM_014786 | ARHGEF17 | frameshift_deleti  on | exon1 | c.1943delT | p.I648fs | . | . |
| --- | --- | --- | --- | --- | --- | --- | --- | --- | --- | --- | --- | --- | --- |
| CTC2 | 11 | 73074393 | TC | T | 1 | NM_014786 | ARHGEF17 | frameshift_deleti  on | exon15 | c.5140delC | p.R1714fs | . | . |
| CTC2 | 11 | 73670655 | GA | G | 1 | NM_153614 | DNAJB13 | frameshift_deleti  on | exon3 | c.290delA | p.E97fs | . | . |
| CTC2 | 11 | 73950231 | TAG | T | 0.4 | NM_001271593 | PPME1 | frameshift_deleti  on | exon9 | c.765_766del | p.I255fs | . | . |
| CTC2 | 11 | 75133691 | TG | T | 0.5 | NM_001039548 | KLHL35 | frameshift_deleti  on | exon6 | c.1684delC | p.Q562fs | . | . |
| CTC2 | 11 | 76207278 | CA | C | 0.333 | NM_020193 | C11orf30 | frameshift_deleti  on | exon9 | c.1129delA | p.K377fs | . | . |
| CTC2 | 11 | 76224464 | TA | T | 0.913 | NM_020193 | C11orf30 | frameshift_deleti  on | exon10 | c.1399delA | p.K467fs | . | . |
| CTC2 | 11 | 76256969 | CAT | C | 0.32 | NM_020193 | C11orf30 | frameshift_deleti  on | exon20 | c.3403_3404del | p.I1135fs | . | . |
| CTC2 | 11 | 76507072 | CT | C | 0.229 | NM_001258210 | TSKU | frameshift_deleti  on | exon2 | c.413delT | p.L138fs | . | . |
| CTC2 | 11 | 77051796 | TCC | T | 0.213 | NM_001128620 | PAK1 | frameshift_deleti  on | exon11 | c.1009_1010del | p.G337fs | . | . |
| CTC2 | 11 | 77348714 | GC | G | 0.5 | NM_001293 | CLNS1A | frameshift_deleti  on | exon1 | c.45delG | p.G15fs | . | . |
| CTC2 | 11 | 77387977 | CA | C | 0.583 | NM_016578 | RSF1 | frameshift_deleti  on | exon13 | c.3200delT | p.L1067fs | . | . |
| CTC2 | 11 | 77924811 | C | CA | 0.284 | NM_020798 | USP35 | frameshift_inserti  on | exon11 | c.3010dupA | p.C1003fs | . | . |
| CTC2 | 11 | 82560202 | CT | C | 0.926 | NM_005040 | PRCP | frameshift_deleti  on | exon6 | c.809delA | p.Q270fs | . | . |
| CTC2 | 11 | 82877218 | GA | G | 0.789 | NM_015885 | PCF11 | frameshift_deleti  on | exon5 | c.1280delA | p.E427fs | . | . |
| CTC2 | 11 | 85436169 | A | ATG | 1 | NM_206927 | SYTL2 | frameshift_inserti  on | exon1 | c.1330_1331ins  CA | p.L444fs | . | . |
| CTC2 | 11 | 85593684 | GA | G | 0.294 | NM_001286159 | CCDC83 | frameshift_deleti  on | exon4 | c.310delA | p.K104fs | . | . |
| CTC2 | 11 | 88924410 | CT | C | 0.203 | NM_000372 | TYR | frameshift_deleti  on | exon2 | c.861delT | p.S287fs | . | . |
| CTC2 | 11 | 89447360 | GT | G | 0.309 | NM_001146162 | TRIM77 | frameshift_deleti  on | exon3 | c.509delT | p.V170fs | . | . |
| CTC2 | 11 | 93458982 | GA | G | 0.342 | NM_033395 | KIAA1731 | frameshift_deleti  on | exon23 | c.6485delA | p.E2162fs | . | . |
| CTC2 | 11 | 94278105 | A | AGGG | 0.3 | NM_002033 | FUT4 | nonframeshift_in  sertion | exon1 | c.806_807insG  GG | p.D269delinsE  G | . | . |
| CTC2 | 11 | 94758931 | TC | T | 0.282 | NM_001161630 | KDM4E | frameshift_deleti  on | exon1 | c.211delC | p.P71fs | . | . |
| CTC2 | 11 | 95516375 | TA | T | 0.556 | NM_144664 | FAM76B | frameshift_deleti  on | exon5 | c.416delT | p.L139fs | . | . |
| CTC2 | 11 | 95825374 | TTGCTGC  TGC | T | 1 | NM_032427 | MAML2 | nonframeshift_de  letion | exon2 | c.1812_1820del | p.604_607del | . | . |
| CTC2 | 11 | 100844046 | TG | T | 0.228 | NM_152432 | ARHGAP42 | frameshift_deleti  on | exon18 | c.1692delG | p.L564fs | . | . |
| CTC2 | 11 | 101375201 | G | GA | 0.727 | NM_004621 | TRPC6 | frameshift_inserti  on | exon2 | c.498dupT | p.L167fs | . | . |
| CTC2 | 11 | 102564789 | GT | G | 0.806 | NM_022122 | MMP27 | frameshift_deleti  on | exon8 | c.1040delA | p.N347fs | . | . |
| CTC2 | 11 | 102576368 | AT | A | 0.36 | NM_022122 | MMP27 | frameshift_deleti  on | exon1 | c.77delA | p.N26fs | . | . |
| CTC2 | 11 | 102651237 | G | GA | 0.529 | NM_002425 | MMP10 | frameshift_inserti  on | exon1 | c.85dupT | p.S29fs | . | . |
| CTC2 | 11 | 102668790 | ACAG | A | 0.263 | NM_002421 | MMP1 | nonframeshift_de  letion | exon1 | c.31_33del | p.11_11del | . | . |
| CTC2 | 11 | 102825196 | CA | C | 0.267 | NM_002427 | MMP13 | frameshift_deleti  on | exon3 | c.501delT | p.F167fs | . | . |
| CTC2 | 11 | 103191833 | AT | A | 0.75 | NM_001377 | DYNC2H1 | frameshift_deleti  on | exon81 | c.11802delT | p.Y3934fs | . | . |
| CTC2 | 11 | 103349968 | TA | T | 0.714 | NM_001377 | DYNC2H1 | frameshift_deleti  on | exon89 | c.12912delA | p.L4304fs | . | . |
| CTC2 | 11 | 105881450 | TG | T | 0.205 | NM_032424 | MSANTD4 | frameshift_deleti  on | exon2 | c.194delC | p.T65fs | . | . |
| CTC2 | 11 | 108056029 | TG | T | 1 | NM_002519 | NPAT | frameshift_deleti  on | exon10 | c.835delC | p.Q279fs | . | . |
| CTC2 | 11 | 108068103 | TA | T | 0.455 | NM_002519 | NPAT | frameshift_deleti  on | exon2 | c.81delT | p.F27fs | . | . |
| CTC2 | 11 | 108196152 | AT | A | 0.268 | NM_000051 | ATM | frameshift_deleti  on | exon46 | c.6689delT | p.I2230fs | . | . |
| CTC2 | 11 | 110035265 | TG | T | 1 | NM_033390 | ZC3H12C | frameshift_deleti  on | exon6 | c.1456delG | p.A486fs | . | . |
| CTC2 | 11 | 110462816 | GA | G | 0.227 | NM_001258415 | ARHGAP20 | frameshift_deleti  on | exon10 | c.958delT | p.S320fs | . | . |
| CTC2 | 11 | 111594477 | TG | T | 0.429 | NM_015191 | SIK2 | frameshift_deleti  on | exon15 | c.2406delG | p.L802fs | . | . |
| CTC2 | 11 | 113270343 | CG | C | 0.324 | NM_178510 | ANKK1 | frameshift_deleti  on | exon8 | c.1653delG | p.A551fs | . | . |
| CTC2 | 11 | 114320644 | G | GA | 0.553 | NM_015523 | REXO2 | frameshift_inserti  on | exon7 | c.662dupA | p.E221fs | . | . |
| CTC2 | 11 | 114577151 | CT | C | 0.8 | NM_182495 | NXPE2 | frameshift_deleti  on | exon6 | c.1180delT | p.F394fs | . | . |
| CTC2 | 11 | 116797940 | AT | A | 0.8 | NM_001281749 | SIK3 | stopgain | exon4 | c.610delA | p.I204X | . | . |
| CTC2 | 11 | 117870124 | TA | T | 0.849 | NM_001558 | IL10RA | frameshift_deleti on | exon7 | c.1506delA | p.L502fs | . | . |
| CTC2 | 11 | 118065067 | TC | T | 0.676 | NM_001286570 | AMICA1 | frameshift_deleti  on | exon9 | c.1040delG | p.G347fs | . | . |
| CTC2 | 11 | 118252148 | GC | G | 0.472 | NM_001204077 | UBE4A | frameshift_deleti  on | exon12 | c.1920delC | p.R640fs | . | . |
| CTC2 | 11 | 118772573 | TCATGG  GCACCT | T | 0.125 | NM_182557 | BCL9L | nonframeshift_de  letion | exon6 | c.1867_1878del | p.623_626del | . | . |
| CTC2 | 11 | 118971821 | AC | A | 0.32 | NM_001382 | DPAGT1 | frameshift_deleti  on | exon2 | c.188delG | p.G63fs | . | . |
| CTC2 | 11 | 121444996 | TG | T | 0.235 | NM_003105 | SORL1 | frameshift_deleti  on | exon24 | c.3385delG | p.G1129fs | . | . |
| CTC2 | 11 | 121986425 | CT | C | 1 | NM_001001786 | BLID | frameshift_deleti  on | exon1 | c.205delA | p.R69fs | . | . |
| CTC2 | 11 | 123448280 | TC | T | 0.833 | NM_001286563 | GRAMD1B | frameshift_deleti  on | exon2 | c.230delC | p.S77fs | . | . |
| CTC2 | 11 | 123900699 | GC | G | 0.364 | NM_001004464 | OR10G8 | frameshift_deleti  on | exon1 | c.371delC | p.A124fs | . | . |
| CTC2 | 11 | 123988549 | GA | G | 0.526 | NM_014622 | VWA5A | frameshift_deleti  on | exon3 | c.214delA | p.K72fs | . | . |
| CTC2 | 11 | 123994944 | C | CT | 0.849 | NM_014622 | VWA5A | frameshift_inserti  on | exon10 | c.1166dupT | p.L389fs | . | . |
| CTC2 | 11 | 124294530 | TG | T | 0.341 | NM_001005196 | OR8B4 | frameshift_deleti  on | exon1 | c.237delC | p.P79fs | . | . |
| CTC2 | 11 | 124519626 | TA | T | 0.429 | NM_170601 | SIAE | frameshift_deleti  on | exon5 | c.610delT | p.Y204fs | . | . |
| CTC2 | 11 | 124740617 | TG | T | 0.312 | NM_022370 | ROBO3 | frameshift_deleti  on | exon6 | c.1027delG | p.V343fs | . | . |

| CTC2 | 11 | 125452300 | A | AC | 1 | NM_001007277 | EI24 | frameshift_inserti  on | exon9 | c.733dupC | p.R244fs | . | . |
| --- | --- | --- | --- | --- | --- | --- | --- | --- | --- | --- | --- | --- | --- |
| CTC2 | 11 | 125864229 | CG | C | 0.446 | NM_001243597 | CDON | frameshift_deleti  on | exon14 | c.2599delC | p.R867fs | . | . |
| CTC2 | 11 | 126080895 | TG | T | 0.197 | NM_001144827 | RPUSD4 | frameshift_deleti  on | exon2 | c.244delC | p.Q82fs | . | . |
| CTC2 | 11 | 128628157 | G | GA | 0.455 | NM_002017 | FLI1 | frameshift_inserti  on | exon2 | c.167dupA | p.E56fs | . | . |
| CTC2 | 11 | 128844150 | TG | T | 0.598 | NM_014715 | ARHGAP32 | frameshift_deleti  on | exon11 | c.1852delC | p.Q618fs | . | . |
| CTC2 | 11 | 129780428 | TCTG | T | 0.138 | NM_199439 | PRDM10 | nonframeshift_de  letion | exon14 | c.2704_2706del | p.902_902del | . | . |
| CTC2 | 11 | 133816137 | TCG | T | 0.762 | NM_001277285 | IGSF9B | frameshift_deleti  on | exon2 | c.79_80del | p.R27fs | . | . |
| CTC2 | 12 | 521004 | TC | T | 1 | NM_001130148 | CCDC77 | frameshift_deleti  on | exon2 | c.35delC | p.S12fs | . | . |
| CTC2 | 12 | 977664 | TC | T | 0.308 | NM_001184985 | WNK1 | frameshift_deleti  on | exon9 | c.2773delC | p.P925fs | . | . |
| CTC2 | 12 | 1005304 | AT | A | 0.583 | NM_014823 | WNK1 | frameshift_deleti  on | exon22 | c.4908delT | p.H1636fs | . | . |
| CTC2 | 12 | 1292542 | GC | G | 0.619 | NM_178039 | ERC1 | frameshift_deleti  on | exon10 | c.2029delC | p.Q677fs | . | . |
| CTC2 | 12 | 2062040 | AC | A | 0.231 | NM_152640 | DCP1B | frameshift_deleti  on | exon7 | c.1065delG | p.L355fs | . | . |
| CTC2 | 12 | 2062323 | T | TTGC | 0.571 | NM_152640 | DCP1B | nonframeshift_in  sertion | exon7 | c.782_783insG  CA | p.Q261delinsQ  Q | . | . |
| CTC2 | 12 | 3392263 | GT | G | 0.217 | NM_001168320 | TSPAN9 | frameshift_deleti  on | exon8 | c.702delT | p.G234fs | . | . |
| CTC2 | 12 | 4409143 | A | AC | 0.583 | NM_001759 | CCND2 | frameshift_inserti  on | exon5 | c.839dupC | p.T280fs | . | . |
| CTC2 | 12 | 5021032 | CG | C | 0.877 | NM_000217 | KCNA1 | frameshift_deleti  on | exon2 | c.489delG | p.S163fs | . | . |
| CTC2 | 12 | 5603982 | TCA | T | 0.167 | NM_002527 | NTF3 | frameshift_deleti  on | exon1 | c.603_604del | p.V201fs | . | . |
| CTC2 | 12 | 5860096 | GC | G | 0.508 | NM_001278596 | ANO2 | frameshift_deleti  on | exon11 | c.1098delG | p.W366fs | . | . |
| CTC2 | 12 | 6125328 | CT | C | 0.5 | NM_000552 | VWF | frameshift_deleti  on | exon31 | c.5381delA | p.K1794fs | . | . |
| CTC2 | 12 | 6666679 | TG | T | 0.329 | NM_001033714 | NOP2 | frameshift_deleti  on | exon16 | c.1906delC | p.Q636fs | . | . |
| CTC2 | 12 | 6777069 | TTGC | T | 0.727 | NM_001039920 | ZNF384 | nonframeshift_de  letion | exon9 | c.1194_1196del | p.398_399del | . | . |
| CTC2 | 12 | 6969378 | TG | T | 0.889 | NM_001098536 | USP5 | frameshift_deleti  on | exon10 | c.1195delG | p.G399fs | . | . |
| CTC2 | 12 | 6980442 | GCA | G | 0.6 | NM_001146316 | SPSB2 | frameshift_deleti  on | exon3 | c.704_705del | p.V235fs | . | . |
| CTC2 | 12 | 7045891 | ACAG | A | 0.474 | NM_001007026 | ATN1 | nonframeshift_de  letion | exon5 | c.1462_1464del | p.488_488del | . | . |
| CTC2 | 12 | 7048092 | TC | T | 1 | NM_001007026 | ATN1 | frameshift_deleti  on | exon7 | c.2967delC | p.F989fs | . | . |
| CTC2 | 12 | 7252590 | TG | T | 0.178 | NM_016546 | C1RL | frameshift_deleti  on | exon4 | c.522delC | p.A174fs | . | . |
| CTC2 | 12 | 7261752 | TC | T | 0.87 | NM_016546 | C1RL | frameshift_deleti  on | exon1 | c.24delG | p.G8fs | . | . |
| CTC2 | 12 | 7556383 | TTC | T | 0.286 | NM_174941 | CD163L1 | frameshift_deleti  on | exon6 | c.1154_1155del | p.R385fs | . | . |
| CTC2 | 12 | 8083922 | TC | T | 0.877 | NM_006931 | SLC2A3 | frameshift_deleti  on | exon4 | c.428delG | p.G143fs | . | . |
| CTC2 | 12 | 8200557 | ACAG | A | 0.286 | NM_018416 | FOXJ2 | nonframeshift_de  letion | exon7 | c.898_900del | p.300_300del | . | . |
| CTC2 | 12 | 8202048 | TC | T | 0.456 | NM_018416 | FOXJ2 | frameshift_deleti  on | exon9 | c.1419delC | p.F473fs | . | . |
| CTC2 | 12 | 8245572 | AC | A | 0.222 | NM_015509 | NECAP1 | frameshift_deleti  on | exon6 | c.598delC | p.P200fs | . | . |
| CTC2 | 12 | 8374781 | C | CACG | 0.527 | NM_018088 | FAM90A1 | nonframeshift_in  sertion | exon7 | c.1031_1032ins  CGT | p.T344delinsTV | . | . |
| CTC2 | 12 | 8692526 | AAG | A | 0.391 | NM_014358 | CLEC4E | frameshift_deleti  on | exon2 | c.53_54del | p.S18fs | . | . |
| CTC2 | 12 | 8904570 | GC | G | 0.302 | NM_020734 | RIMKLB | frameshift_deleti  on | exon5 | c.425delC | p.A142fs | . | . |
| CTC2 | 12 | 9847367 | TG | T | 1 | NM_001197319 | CLEC2D | frameshift_deleti  on | exon3 | c.259delG | p.G87fs | . | . |
| CTC2 | 12 | 9908960 | A | AG | 0.267 | NM_001781 | CD69 | frameshift_inserti  on | exon2 | c.119dupC | p.P40fs | . | . |
| CTC2 | 12 | 9994445 | GTGT | G | 1 | NM_016523 | KLRF1 | nonframeshift_de  letion | exon4 | c.373_375del | p.125_125del | . | . |
| CTC2 | 12 | 10206932 | AT | A | 0.488 | NM_207345 | CLEC9A | frameshift_deleti  on | exon5 | c.155delT | p.I52fs | . | . |
| CTC2 | 12 | 10868312 | TC | T | 0.485 | NM_001145426 | YBX3 | frameshift_deleti  on | exon4 | c.430delG | p.D144fs | . | . |
| CTC2 | 12 | 11183175 | T | TCC | 0.222 | NM_176885 | TAS2R31 | frameshift_inserti  on | exon1 | c.759_760insG  G | p.S254fs | . | . |
| CTC2 | 12 | 11339542 | A | AT | 0.3 | NM_181429 | TAS2R42 | frameshift_inserti  on | exon1 | c.1dupA | p.M1fs | . | . |
| CTC2 | 12 | 13140137 | CT | C | 0.333 | NM_015987 | HEBP1 | frameshift_deleti  on | exon3 | c.346delA | p.S116fs | . | . |
| CTC2 | 12 | 13717312 | AG | A | 0.208 | NM_000834 | GRIN2B | frameshift_deleti  on | exon13 | c.2859delC | p.P953fs | . | . |
| CTC2 | 12 | 15800106 | TC | T | 0.25 | NM_004447 | EPS8 | frameshift_deleti  on | exon15 | c.1522delG | p.E508fs | . | . |
| CTC2 | 12 | 16704166 | TG | T | 0.6 | NM_001001395 | LMO3 | frameshift_deleti  on | exon4 | c.427delC | p.Q143fs | . | . |
| CTC2 | 12 | 18435398 | GCCC | G | 0.677 | NM_001288772 | PIK3C2G | nonframeshift_de  letion | exon2 | c.384_386del | p.128_129del | . | . |
| CTC2 | 12 | 18715748 | TC | T | 0.636 | NM_004570 | PIK3C2G | frameshift_deleti  on | exon26 | c.3580delC | p.Q1194fs | . | . |
| CTC2 | 12 | 22354797 | T | TG | 0.707 | NM_003034 | ST8SIA1 | frameshift_inserti  on | exon5 | c.759dupC | p.N254fs | . | . |
| CTC2 | 12 | 26571999 | AT | A | 0.273 | NM_002223 | ITPR2 | frameshift_deleti  on | exon50 | c.7092delA | p.E2364fs | . | . |
| CTC2 | 12 | 27181313 | AC | A | 0.4 | NM_004264 | MED21 | frameshift_deleti  on | exon4 | c.355delC | p.Q119fs | . | . |
| CTC2 | 12 | 29469931 | TG | T | 0.513 | NM_001271784 | FAR2 | frameshift_deleti  on | exon8 | c.823delG | p.G275fs | . | . |
| CTC2 | 12 | 30862874 | A | AT | 0.821 | NM_023925 | CAPRIN2 | frameshift_inserti  on | exon17 | c.3195dupA | p.Y1066fs | . | . |
| CTC2 | 12 | 31242375 | TG | T | 0.25 | NM_001257144 | DDX11 | frameshift_deleti  on | exon8 | c.832delG | p.V278fs | . | . |
| CTC2 | 12 | 32458938 | TG | T | 1 | NM_001003398 | BICD1 | frameshift_deleti  on | exon4 | c.888delG | p.L296fs | . | . |
| CTC2 | 12 | 32481210 | AC | A | 1 | NM_001003398 | BICD1 | frameshift_deleti  on | exon5 | c.1822delC | p.P608fs | . | . |
| CTC2 | 12 | 39735365 | TTCC | T | 0.13 | NM_001173463 | KIF21A | nonframeshift_de  letion | exon13 | c.1821_1823del | p.607_608del | . | . |

| CTC2 | 12 | 39745775 | AT | A | 0.833 | NM_001173463 | KIF21A | frameshift_deleti  on | exon11 | c.1476delA | p.K492fs | . | . |
| --- | --- | --- | --- | --- | --- | --- | --- | --- | --- | --- | --- | --- | --- |
| CTC2 | 12 | 40076534 | TG | T | 0.833 | NM_001031748 | C12orf40 | frameshift_deleti  on | exon8 | c.809delG | p.W270fs | . | . |
| CTC2 | 12 | 40697798 | TC | T | 0.28 | NM_198578 | LRRK2 | frameshift_deleti  on | exon27 | c.3640delC | p.P1214fs | . | . |
| CTC2 | 12 | 42854116 | TC | T | 0.242 | NM_001144881 | PRICKLE1 | frameshift_deleti  on | exon8 | c.1990delG | p.E664fs | . | . |
| CTC2 | 12 | 44140032 | AT | A | 0.8 | NM_001271826 | PUS7L | frameshift_deleti  on | exon3 | c.140delA | p.N47fs | . | . |
| CTC2 | 12 | 47629801 | CA | C | 0.3 | NM_001281429 | PCED1B | frameshift_deleti  on | exon3 | c.956delA | p.Q319fs | . | . |
| CTC2 | 12 | 48491809 | GA | G | 0.35 | NM_001267594 | SENP1 | frameshift_deleti  on | exon3 | c.102delT | p.F34fs | . | . |
| CTC2 | 12 | 48543193 | GA | G | 0.701 | NM_024095 | ASB8 | frameshift_deleti  on | exon4 | c.822delT | p.L274fs | . | . |
| CTC2 | 12 | 49072859 | CA | C | 0.769 | NM_017822 | KANSL2 | frameshift_deleti  on | exon4 | c.504delT | p.S168fs | . | . |
| CTC2 | 12 | 49087433 | T | TATG | 0.123 | NM_001240 | CCNT1 | nonframeshift_in  sertion | exon9 | c.1563_1564ins  CAT | p.N522delinsH  N | . | . |
| CTC2 | 12 | 49443844 | GC | G | 0.2 | NM_003482 | KMT2D | frameshift_deleti  on | exon11 | c.3526delG | p.A1176fs | . | . |
| CTC2 | 12 | 49888718 | AC | A | 0.247 | NM_023071 | SPATS2 | frameshift_deleti  on | exon7 | c.460delC | p.L154fs | . | . |
| CTC2 | 12 | 50050608 | TG | T | 0.988 | NM_198900 | FMNL3 | frameshift_deleti  on | exon7 | c.635delC | p.P212fs | . | . |
| CTC2 | 12 | 50190207 | TG | T | 0.282 | NM_001037806 | NCKAP5L | frameshift_deleti  on | exon8 | c.1435delC | p.Q479fs | . | . |
| CTC2 | 12 | 50369369 | TG | T | 0.667 | NM_001652 | AQP6 | frameshift_deleti  on | exon4 | c.765delG | p.V255fs | . | . |
| CTC2 | 12 | 50480115 | CAA | C | 0.122 | NM_003076 | SMARCD1 | frameshift_deleti  on | exon2 | c.350_351del | p.Q117fs | . | . |
| CTC2 | 12 | 50745054 | GCC | G | 0.222 | NM_001145475 | FAM186A | frameshift_deleti  on | exon4 | c.5559_5560del | p.W1853fs | . | . |
| CTC2 | 12 | 51281177 | CA | C | 0.346 | NM_182559 | TMPRSS12 | frameshift_deleti  on | exon5 | c.929delA | p.Q310fs | . | . |
| CTC2 | 12 | 51446028 | AG | A | 0.397 | NM_001243689 | LETMD1 | frameshift_deleti  on | exon3 | c.429delG | p.E143fs | . | . |
| CTC2 | 12 | 51450021 | TG | T | 0.333 | NM_001243689 | LETMD1 | frameshift_deleti  on | exon6 | c.795delG | p.L265fs | . | . |
| CTC2 | 12 | 51457651 | AC | A | 0.435 | NM_030809 | CSRNP2 | frameshift_deleti  on | exon5 | c.1509delG | p.W503fs | . | . |
| CTC2 | 12 | 51457732 | TC | T | 0.378 | NM_030809 | CSRNP2 | frameshift_deleti  on | exon5 | c.1428delG | p.G476fs | . | . |
| CTC2 | 12 | 51754532 | TC | T | 0.324 | NM_007210 | GALNT6 | frameshift_deleti  on | exon7 | c.1139delG | p.G380fs | . | . |
| CTC2 | 12 | 52946498 | CG | C | 0.803 | NM_033448 | KRT71 | frameshift_deleti  on | exon1 | c.363delC | p.P121fs | . | . |
| CTC2 | 12 | 53045603 | G | GACGC  CGCTG CCACC | 0.5 | NM_000423 | KRT2 | nonframeshift_in sertion | exon1 | c.323_324insTG  GAGGTGGCAG CGGCGT | p.F108delinsFG GGSGV | . | . |
| CTC2 | 12 | 53045615 | A | ACCTC  CAAAG CCGCT | 1 | NM_000423 | KRT2 | nonframeshift_in sertion | exon1 | c.311_312insC  GGCAGCGGCT TTGGAGG | p.G104delinsG GSGFGG | . | . |
| CTC2 | 12 | 53045618 | T | TCCAA  AGCCG CGGCC | 0.706 | NM_000423 | KRT2 | nonframeshift_in sertion | exon1 | c.308_309insG  GGCGGCCGCG GCTTTGG | p.G103delinsG GGRGFG | . | . |
| CTC2 | 12 | 53189413 | ACCAAA  GCCACC AGCCCC | A | 1 | NM_057088 | KRT3 | nonframeshift_de letion | exon1 | c.396_413del | p.132_138del | . | . |
| CTC2 | 12 | 53207583 | C | CCACC  AAAGC CACCA GTGCC GAAAC CAGCT | 1 | NM_002272 | KRT4 | nonframeshift_in sertion | exon1 | c.259_260insCC GGCGGCTTCG GAGCTGGTTTC GGCACTGGTG GCTTTGGTG | p.G87delinsAG GFGAGFGTGGF GG | . | . |
| CTC2 | 12 | 53225288 | AC | A | 0.444 | NM_175834 | KRT79 | frameshift_deleti  on | exon2 | c.599delG | p.G200fs | . | . |
| CTC2 | 12 | 53449013 | AC | A | 0.288 | NM_015319 | TENC1 | frameshift_deleti  on | exon8 | c.597delC | p.N199fs | . | . |
| CTC2 | 12 | 53468442 | AT | A | 1 | NM_032840 | SPRYD3 | frameshift_deleti  on | exon5 | c.497delA | p.N166fs | . | . |
| CTC2 | 12 | 53687192 | TC | T | 0.308 | NM_012291 | ESPL1 | frameshift_deleti  on | exon31 | c.6298delC | p.P2100fs | . | . |
| CTC2 | 12 | 53776715 | AACT | A | 0.255 | NM_001251825 | SP1 | nonframeshift_de  letion | exon3 | c.841_843del | p.281_281del | . | . |
| CTC2 | 12 | 53777074 | CCAT | C | 0.6 | NM_001251825 | SP1 | nonframeshift_de  letion | exon3 | c.1200_1202del | p.400_401del | . | . |
| CTC2 | 12 | 53800461 | G | GA | 0.161 | NM_001251825 | SP1 | frameshift_inserti  on | exon4 | c.1625dupA | p.D542fs | . | . |
| CTC2 | 12 | 53876739 | TC | T | 0.643 | NM_001193511 | MAP3K12 | frameshift_deleti  on | exon11 | c.1847delG | p.G616fs | . | . |
| CTC2 | 12 | 53901214 | TAAC | T | 0.114 | NM_003717 | NPFF | nonframeshift_de  letion | exon1 | c.42_44del | p.14_15del | . | . |
| CTC2 | 12 | 53918417 | CT | C | 0.462 | NM_001130060 | ATF7 | frameshift_deleti  on | exon10 | c.992delA | p.K331fs | . | . |
| CTC2 | 12 | 54799108 | TC | T | 0.5 | NM_002205 | ITGA5 | frameshift_deleti  on | exon12 | c.1066delG | p.D356fs | . | . |
| CTC2 | 12 | 55615273 | GTC | G | 0.271 | NM_001005280 | OR10A7 | frameshift_deleti  on | exon1 | c.466_467del | p.S156fs | . | . |
| CTC2 | 12 | 55688164 | G | GA | 0.286 | NM_001005493 | OR6C6 | frameshift_inserti  on | exon1 | c.852dupT | p.P285fs | . | . |
| CTC2 | 12 | 55725859 | GC | G | 0.933 | NM_054104 | OR6C3 | frameshift_deleti  on | exon1 | c.376delC | p.P126fs | . | . |
| CTC2 | 12 | 55886569 | CA | C | 0.25 | NM_001005519 | OR6C68 | frameshift_deleti  on | exon1 | c.409delA | p.K137fs | . | . |
| CTC2 | 12 | 56086752 | TC | T | 0.416 | NM_001144996 | ITGA7 | frameshift_deleti  on | exon21 | c.2731delG | p.D911fs | . | . |
| CTC2 | 12 | 56495488 | TG | T | 0.833 | NM_001982 | ERBB3 | frameshift_deleti  on | exon28 | c.3679delG | p.V1227fs | . | . |
| CTC2 | 12 | 56522217 | TG | T | 0.633 | NM_001184796 | ESYT1 | frameshift_deleti  on | exon1 | c.115delG | p.G39fs | . | . |
| CTC2 | 12 | 56631078 | T | TA | 0.3 | NM_001135195 | SLC39A5 | frameshift_inserti  on | exon10 | c.1433_1434ins  A | p.F478fs | . | . |
| CTC2 | 12 | 56745206 | GA | G | 0.467 | NM_005419 | STAT2 | frameshift_deleti  on | exon9 | c.810delT | p.F270fs | . | . |
| CTC2 | 12 | 56814354 | TG | T | 0.194 | NM_003920 | TIMELESS | frameshift_deleti  on | exon26 | c.3226delC | p.Q1076fs | . | . |
| CTC2 | 12 | 57437064 | GT | G | 0.5 | NM_005379 | MYO1A | frameshift_deleti  on | exon11 | c.970delA | p.T324fs | . | . |
| CTC2 | 12 | 57453681 | TG | T | 0.52 | NM_015257 | TMEM194A | frameshift_deleti  on | exon8 | c.1096delC | p.Q366fs | . | . |

| CTC2 | 12 | 57625295 | TG | T | 0.767 | NM_001166356 | SHMT2 | frameshift_deleti  on | exon3 | c.264delG | p.L88fs | . | . |
| --- | --- | --- | --- | --- | --- | --- | --- | --- | --- | --- | --- | --- | --- |
| CTC2 | 12 | 57637596 | TA | T | 0.5 | NM_001286257 | STAC3 | stoploss | exon9 | c.535delT | p.X179R | . | . |
| CTC2 | 12 | 57674204 | TTGC | T | 0.538 | NM_014925 | R3HDM2 | nonframeshift_de letion | exon12 | c.1236_1238del | p.412_413del | . | . |
| CTC2 | 12 | 57864954 | C | CT | 0.308 | NM_001160045 | GLI1 | frameshift_inserti  on | exon10 | c.2047_2048ins  T | p.Q683fs | . | . |
| CTC2 | 12 | 64712546 | A | AGTT | 0.186 | NM_001170633 | C12orf56 | nonframeshift_in  sertion | exon4 | c.702_703insAA  C | p.S235delinsNS | . | . |
| CTC2 | 12 | 69222667 | G | GA | 1 | NM_001145339 | MDM2 | frameshift_inserti  on | exon6 | c.476dupA | p.E159fs | . | . |
| CTC2 | 12 | 69744009 | CA | C | 0.824 | NM_000239 | LYZ | frameshift_deleti  on | exon2 | c.259delA | p.K87fs | . | . |
| CTC2 | 12 | 69991510 | CA | C | 0.258 | NM_001198842 | CCT2 | frameshift_deleti  on | exon12 | c.1055delA | p.Q352fs | . | . |
| CTC2 | 12 | 70091473 | AT | A | 0.5 | NM_001282614 | BEST3 | frameshift_deleti  on | exon2 | c.105delA | p.E35fs | . | . |
| CTC2 | 12 | 70965752 | GT | G | 0.287 | NM_001206971 | PTPRB | frameshift_deleti  on | exon9 | c.2033delA | p.N678fs | . | . |
| CTC2 | 12 | 70990022 | TC | T | 1 | NM_001206971 | PTPRB | frameshift_deleti  on | exon3 | c.410delG | p.G137fs | . | . |
| CTC2 | 12 | 71950444 | TG | T | 0.243 | NM_001277227 | LGR5 | frameshift_deleti  on | exon5 | c.475delG | p.D159fs | . | . |
| CTC2 | 12 | 73012717 | CT | C | 0.375 | NM_013381 | TRHDE | frameshift_deleti  on | exon13 | c.2234delT | p.L745fs | . | . |
| CTC2 | 12 | 76424916 | TTGCTGC | T | 1 | NM_007350 | PHLDA1 | nonframeshift_de  letion | exon1 | c.600_605del | p.200_202del | . | . |
| CTC2 | 12 | 76424916 | TTGC | T | 1 | NM_007350 | PHLDA1 | nonframeshift_de  letion | exon1 | c.603_605del | p.201_202del | . | . |
| CTC2 | 12 | 76424937 | TTGC | T | 1 | NM_007350 | PHLDA1 | nonframeshift_de  letion | exon1 | c.582_584del | p.194_195del | . | . |
| CTC2 | 12 | 78401090 | C | CT | 0.167 | NM_001024383 | NAV3 | frameshift_inserti  on | exon8 | c.1773dupT | p.A591fs | . | . |
| CTC2 | 12 | 78452820 | AC | A | 0.634 | NM_001024383 | NAV3 | frameshift_deleti  on | exon12 | c.2562delC | p.N854fs | . | . |
| CTC2 | 12 | 78513574 | GA | G | 0.295 | NM_001024383 | NAV3 | frameshift_deleti  on | exon15 | c.3599delA | p.E1200fs | . | . |
| CTC2 | 12 | 80888809 | AT | A | 0.212 | NM_001145026 | PTPRQ | frameshift_deleti  on | exon8 | c.1123delT | p.S375fs | . | . |
| CTC2 | 12 | 81101690 | GC | G | 0.485 | NM_002469 | MYF6 | frameshift_deleti  on | exon1 | c.193delC | p.P65fs | . | . |
| CTC2 | 12 | 86199113 | AT | A | 1 | NM_005447 | RASSF9 | frameshift_deleti  on | exon2 | c.674delA | p.N225fs | . | . |
| CTC2 | 12 | 88390227 | CA | C | 0.8 | NM_152589 | C12orf50 | frameshift_deleti  on | exon6 | c.404delT | p.L135fs | . | . |
| CTC2 | 12 | 88505589 | TG | T | 1 | NM_025114 | CEP290 | frameshift_deleti  on | exon21 | c.2098delC | p.H700fs | . | . |
| CTC2 | 12 | 88912618 | CAT | C | 0.396 | NM_000899 | KITLG | frameshift_deleti  on | exon4 | c.217_218del | p.M73fs | . | . |
| CTC2 | 12 | 91501953 | GT | G | 0.912 | NM_002345 | LUM | frameshift_deleti  on | exon2 | c.803delA | p.N268fs | . | . |
| CTC2 | 12 | 94620974 | AG | A | 0.231 | NM_005761 | PLXNC1 | frameshift_deleti  on | exon9 | c.1944delG | p.K648fs | . | . |
| CTC2 | 12 | 97339521 | GA | G | 0.75 | NM_001135177 | NEDD1 | frameshift_deleti  on | exon12 | c.1439delA | p.E480fs | . | . |
| CTC2 | 12 | 100691881 | AC | A | 0.253 | NM_017988 | SCYL2 | frameshift_deleti  on | exon4 | c.409delC | p.P137fs | . | . |
| CTC2 | 12 | 101739399 | TC | T | 0.39 | NM_014503 | UTP20 | frameshift_deleti  on | exon37 | c.4674delC | p.F1558fs | . | . |
| CTC2 | 12 | 101748793 | TC | T | 0.346 | NM_014503 | UTP20 | frameshift_deleti  on | exon41 | c.5292delC | p.F1764fs | . | . |
| CTC2 | 12 | 101790235 | AT | A | 0.476 | NM_001177 | ARL1 | frameshift_deleti  on | exon5 | c.456delA | p.K152fs | . | . |
| CTC2 | 12 | 102127373 | CCT | C | 0.111 | NM_001177948 | SYCP3 | frameshift_deleti  on | exon6 | c.431_432del | p.E144fs | . | . |
| CTC2 | 12 | 108917237 | C | CT | 0.6 | NM_014706 | SART3 | frameshift_inserti  on | exon19 | c.2888dupA | p.K963fs | . | . |
| CTC2 | 12 | 108942923 | TC | T | 0.269 | NM_014706 | SART3 | frameshift_deleti  on | exon2 | c.379delG | p.E127fs | . | . |
| CTC2 | 12 | 109331097 | AT | A | 0.296 | NM_018711 | SVOP | stopgain | exon9 | c.754delA | p.M252X | . | . |
| CTC2 | 12 | 109614057 | CT | C | 0.388 | NM_001093 | ACACB | frameshift_deleti on | exon8 | c.1427delT | p.L476fs | . | . |
| CTC2 | 12 | 110034262 | GC | G | 0.409 | NM_000431 | MVK | frameshift_deleti  on | exon11 | c.1072delC | p.Q358fs | . | . |
| CTC2 | 12 | 110778504 | C | CA | 0.25 | NM_001681 | ATP2A2 | frameshift_inserti  on | exon14 | c.1802_1803ins  A | p.P601fs | . | . |
| CTC2 | 12 | 110989601 | AG | A | 0.325 | NM_139283 | PPTC7 | frameshift_deleti  on | exon2 | c.395delC | p.P132fs | . | . |
| CTC2 | 12 | 111885228 | GA | G | 0.316 | NM_005475 | SH2B3 | frameshift_deleti  on | exon6 | c.1117delA | p.K373fs | . | . |
| CTC2 | 12 | 111886000 | AC | A | 0.189 | NM_005475 | SH2B3 | frameshift_deleti  on | exon8 | c.1623delC | p.H541fs | . | . |
| CTC2 | 12 | 112478303 | AT | A | 0.857 | NM_024953 | NAA25 | frameshift_deleti  on | exon21 | c.2519delA | p.N840fs | . | . |
| CTC2 | 12 | 112752331 | CG | C | 0.889 | NM_001109662 | HECTD4 | frameshift_deleti  on | exon3 | c.282delC | p.F94fs | . | . |
| CTC2 | 12 | 113707592 | GC | G | 0.22 | NM_017901 | TPCN1 | frameshift_deleti  on | exon7 | c.691delC | p.P231fs | . | . |
| CTC2 | 12 | 116460375 | G | GA | 0.818 | NM_015335 | MED13L | frameshift_inserti  on | exon5 | c.510dupT | p.L171fs | . | . |
| CTC2 | 12 | 117962889 | AG | A | 0.433 | NM_173598 | KSR2 | frameshift_deleti  on | exon14 | c.1899delC | p.P633fs | . | . |
| CTC2 | 12 | 118016929 | G | GT | 0.526 | NM_173598 | KSR2 | frameshift_inserti  on | exon7 | c.1232dupA | p.N411fs | . | . |
| CTC2 | 12 | 118199328 | TC | T | 0.741 | NM_173598 | KSR2 | frameshift_deleti  on | exon4 | c.386delG | p.G129fs | . | . |
| CTC2 | 12 | 119909799 | CTT | C | 0.382 | NM_178499 | CCDC60 | frameshift_deleti  on | exon3 | c.172_173del | p.F58fs | . | . |
| CTC2 | 12 | 121746335 | TG | T | 0.312 | NM_001137559 | ANAPC5 | frameshift_deleti  on | exon17 | c.1879delC | p.Q627fs | . | . |
| CTC2 | 12 | 122284976 | CT | C | 1 | NM_002150 | HPD | frameshift_deleti  on | exon10 | c.740delA | p.K247fs | . | . |
| CTC2 | 12 | 122729205 | CCT | C | 0.468 | NM_022916 | VPS33A | frameshift_deleti  on | exon7 | c.878_879del | p.E293fs | . | . |
| CTC2 | 12 | 123042029 | CG | C | 1 | NM_014708 | KNTC1 | frameshift_deleti  on | exon17 | c.1372delG | p.A458fs | . | . |
| CTC2 | 12 | 123252076 | CA | C | 0.667 | NM_003677 | DENR | frameshift_deleti  on | exon6 | c.338delA | p.Q113fs | . | . |
| CTC2 | 12 | 123276590 | GA | G | 0.34 | NM_201435 | CCDC62 | frameshift_deleti  on | exon6 | c.695delA | p.E232fs | . | . |
| CTC2 | 12 | 123342913 | GC | G | 0.517 | NM_003959 | HIP1R | frameshift_deleti  on | exon20 | c.1982delC | p.A661fs | . | . |

| CTC2 | 12 | 123651247 | TG | T | 0.481 | NM_022782 | MPHOSPH9 | frameshift_deleti  on | exon17 | c.2545delC | p.Q849fs | . | . |
| --- | --- | --- | --- | --- | --- | --- | --- | --- | --- | --- | --- | --- | --- |
| CTC2 | 12 | 124103223 | TGCA | T | 0.384 | NM_020936 | DDX55 | nonframeshift_de  letion | exon12 | c.1173_1175del | p.391_392del | . | . |
| CTC2 | 12 | 124303583 | TC | T | 0.429 | NM_207437 | DNAH10 | frameshift_deleti  on | exon21 | c.3514delC | p.L1172fs | . | . |
| CTC2 | 12 | 124413073 | TG | T | 0.521 | NM_207437 | DNAH10 | frameshift_deleti  on | exon69 | c.11892delG | p.L3964fs | . | . |
| CTC2 | 12 | 124840098 | GC | G | 0.484 | NM_001077261 | NCOR2 | frameshift_deleti  on | exon25 | c.3230delG | p.G1077fs | . | . |
| CTC2 | 12 | 124887057 | G | GGGCT  GC | 0.651 | NM_001077261 | NCOR2 | nonframeshift_in  sertion | exon16 | c.1529_1530ins  GCAGCC | p.P510delinsP  QP | . | . |
| CTC2 | 12 | 124887058 | G | GGCTG  CT | 0.933 | NM_001077261 | NCOR2 | nonframeshift_in  sertion | exon16 | c.1528_1529ins  AGCAGC | p.P510delinsQ  QP | . | . |
| CTC2 | 12 | 132510294 | CCT | C | 0.444 | NM_015409 | EP400 | frameshift_deleti  on | exon25 | c.4960_4961del | p.L1654fs | . | . |
| CTC2 | 12 | 132512055 | TC | T | 1 | NM_015409 | EP400 | frameshift_deleti  on | exon26 | c.5089delC | p.P1697fs | . | . |
| CTC2 | 12 | 132527853 | TC | T | 0.188 | NM_015409 | EP400 | frameshift_deleti  on | exon33 | c.6213delC | p.L2071fs | . | . |
| CTC2 | 12 | 132530021 | C | CA | 1 | NM_015409 | EP400 | frameshift_inserti  on | exon38 | c.6942_6943ins  A | p.P2314fs | . | . |
| CTC2 | 12 | 132547068 | G | GGCA | 0.333 | NM_015409 | EP400 | nonframeshift_in  sertion | exon47 | c.8156_8157ins  GCA | p.R2719delinsR  Q | . | . |
| CTC2 | 12 | 132547093 | A | ACAG | 0.8 | NM_015409 | EP400 | nonframeshift_in  sertion | exon47 | c.8181_8182ins  CAG | p.Q2727delins  QQ | . | . |
| CTC2 | 12 | 132862951 | TGGCCG  CCAAGC CACCCT GGCCCA GGCCTC CTGGCC | T | 0.278 | NM_001122636 | GALNT9 | frameshift_deleti on | exon2 | c.252_303del | p.P84fs | . | . |
| CTC2 | 12 | 133197898 | CG | C | 1 | NM_012226 | P2RX2 | frameshift_deleti  on | exon7 | c.748delG | p.G250fs | . | . |
| CTC2 | 12 | 133698462 | GT | G | 0.192 | NM_001277291 | ZNF891 | frameshift_deleti  on | exon2 | c.42delA | p.K14fs | . | . |
| CTC2 | 12 | 133768106 | AT | A | 0.389 | NM_001165887 | ZNF268 | frameshift_deleti  on | exon3 | c.66delT | p.D22fs | . | . |
| CTC2 | 12 | 133770067 | GA | G | 0.273 | NM_001165887 | ZNF268 | frameshift_deleti  on | exon5 | c.311delA | p.E104fs | . | . |
| CTC2 | 13 | 20010428 | CA | C | 0.416 | NM_001271850 | TPTE2 | frameshift_deleti  on | exon2 | c.66delT | p.F22fs | . | . |
| CTC2 | 13 | 20244415 | TG | T | 0.281 | NM_017520 | MPHOSPH8 | frameshift_deleti  on | exon12 | c.2370delG | p.L790fs | . | . |
| CTC2 | 13 | 20763108 | GC | G | 0.333 | NM_004004 | GJB2 | frameshift_deleti  on | exon2 | c.612delG | p.L204fs | . | . |
| CTC2 | 13 | 20763333 | C | CT | 0.273 | NM_004004 | GJB2 | frameshift_inserti  on | exon2 | c.387dupA | p.G130fs | . | . |
| CTC2 | 13 | 23909937 | TC | T | 0.6 | NM_001278055 | SACS | frameshift_deleti  on | exon8 | c.7636delG | p.D2546fs | . | . |
| CTC2 | 13 | 23914381 | CTT | C | 0.792 | NM_001278055 | SACS | frameshift_deleti  on | exon8 | c.3191_3192del | p.K1064fs | . | . |
| CTC2 | 13 | 25034185 | GC | G | 0.417 | NM_006437 | PARP4 | frameshift_deleti  on | exon18 | c.2222delG | p.G741fs | . | . |
| CTC2 | 13 | 25483944 | GT | G | 0.217 | NM_018451 | CENPJ | frameshift_deleti  on | exon4 | c.848delA | p.N283fs | . | . |
| CTC2 | 13 | 26133139 | G | GA | 0.4 | NM_016529 | ATP8A2 | frameshift_inserti  on | exon14 | c.1293dupA | p.G431fs | . | . |
| CTC2 | 13 | 26975608 | CCAG | C | 0.222 | NM_001260 | CDK8 | nonframeshift_de  letion | exon12 | c.1117_1119del | p.373_373del | . | . |
| CTC2 | 13 | 27250721 | GA | G | 0.512 | NM_006646 | WASF3 | frameshift_deleti  on | exon7 | c.577delA | p.K193fs | . | . |
| CTC2 | 13 | 28901650 | TC | T | 0.435 | NM_002019 | FLT1 | frameshift_deleti  on | exon20 | c.2744delG | p.G915fs | . | . |
| CTC2 | 13 | 31036683 | AT | A | 0.843 | NM_002128 | HMGB1 | frameshift_deleti  on | exon4 | c.462delA | p.K154fs | . | . |
| CTC2 | 13 | 31287978 | G | GGTAT | 0.75 | NM_001204406 | ALOX5AP | frameshift_inserti  on | exon1 | c.116_117insGT  AT | p.W39fs | . | . |
| CTC2 | 13 | 31803364 | CT | C | 1 | NM_194318 | B3GALTL | frameshift_deleti  on | exon4 | c.204delT | p.S68fs | . | . |
| CTC2 | 13 | 32776140 | AC | A | 0.385 | NM_023037 | FRY | frameshift_deleti  on | exon30 | c.3810delC | p.N1270fs | . | . |
| CTC2 | 13 | 32805393 | AT | A | 0.312 | NM_023037 | FRY | frameshift_deleti  on | exon41 | c.5484delT | p.N1828fs | . | . |
| CTC2 | 13 | 32906876 | CA | C | 0.261 | NM_000059 | BRCA2 | frameshift_deleti  on | exon10 | c.1262delA | p.Q421fs | . | . |
| CTC2 | 13 | 32932058 | AT | A | 0.379 | NM_000059 | BRCA2 | frameshift_deleti  on | exon16 | c.7798delT | p.F2600fs | . | . |
| CTC2 | 13 | 32977140 | TG | T | 0.364 | NM_001286459 | N4BP2L1 | frameshift_deleti  on | exon5 | c.670delC | p.H224fs | . | . |
| CTC2 | 13 | 33232381 | A | AT | 0.75 | NM_015032 | PDS5B | frameshift_inserti  on | exon4 | c.319dupT | p.I106fs | . | . |
| CTC2 | 13 | 37269471 | TC | T | 0.5 | NM_203451 | SERTM1 | frameshift_deleti  on | exon2 | c.257delC | p.S86fs | . | . |
| CTC2 | 13 | 37446867 | AC | A | 0.435 | NM_001127217 | SMAD9 | frameshift_deleti  on | exon3 | c.597delG | p.Q199fs | . | . |
| CTC2 | 13 | 37447007 | TG | T | 0.394 | NM_001127217 | SMAD9 | frameshift_deleti  on | exon3 | c.457delC | p.Q153fs | . | . |
| CTC2 | 13 | 37559854 | TTC | T | 0.562 | NM_001142364 | ALG5 | frameshift_deleti  on | exon5 | c.379_380del | p.E127fs | . | . |
| CTC2 | 13 | 38153372 | C | CA | 0.52 | NM_001135934 | POSTN | frameshift_inserti  on | exon13 | c.1784dupT | p.L595fs | . | . |
| CTC2 | 13 | 38153374 | G | GC | 0.586 | NM_001135934 | POSTN | frameshift_inserti  on | exon13 | c.1782_1783ins  G | p.L595fs | . | . |
| CTC2 | 13 | 39262863 | CA | C | 0.287 | NM_207361 | FREM2 | frameshift_deleti  on | exon1 | c.1383delA | p.A461fs | . | . |
| CTC2 | 13 | 41705591 | TG | T | 0.241 | NM_152903 | KBTBD6 | frameshift_deleti  on | exon1 | c.1056delC | p.P352fs | . | . |
| CTC2 | 13 | 41949650 | CCTT | C | 0.24 | NM_024561 | NAA16 | nonframeshift_de  letion | exon20 | c.2508_2510del | p.836_837del | . | . |
| CTC2 | 13 | 42245155 | G | GT | 0.333 | NM_015058 | VWA8 | frameshift_inserti  on | exon37 | c.4537dupA | p.T1513fs | . | . |
| CTC2 | 13 | 43361018 | TG | T | 0.273 | NM_182508 | FAM216B | frameshift_deleti  on | exon3 | c.220delG | p.G74fs | . | . |
| CTC2 | 13 | 45580336 | GA | G | 1 | NM_018559 | GPALPP1 | frameshift_deleti  on | exon3 | c.222delA | p.R74fs | . | . |
| CTC2 | 13 | 46170719 | CCCAGA  TACTCTT CCTCCT | C | 0.714 | NM_182542 | FAM194B | nonframeshift_de letion | exon3 | c.404_421del | p.135_141del | . | . |
| CTC2 | 13 | 46802814 | GA | G | 0.625 | NM_001282460 | LRRC63 | frameshift_deleti  on | exon4 | c.779delA | p.E260fs | . | . |
| CTC2 | 13 | 48655786 | AC | A | 0.353 | NM_001270629 | MED4 | frameshift_deleti  on | exon5 | c.364delG | p.V122fs | . | . |

| CTC2 | 13 | 48947575 | AT | A | 0.477 | NM_000321 | RB1 | frameshift_deleti  on | exon12 | c.1163delT | p.I388fs | . | . |
| --- | --- | --- | --- | --- | --- | --- | --- | --- | --- | --- | --- | --- | --- |
| CTC2 | 13 | 50034296 | A | AT | 0.75 | NM_001160308 | SETDB2 | frameshift_inserti  on | exon2 | c.71dupT | p.I24fs | . | . |
| CTC2 | 13 | 50280291 | TG | T | 0.459 | NM_002267 | KPNA3 | frameshift_deleti  on | exon14 | c.1151delC | p.T384fs | . | . |
| CTC2 | 13 | 50502133 | A | AT | 0.25 | NM_001127482 | SPRYD7 | frameshift_inserti  on | exon2 | c.194dupA | p.N65fs | . | . |
| CTC2 | 13 | 51503622 | AT | A | 0.212 | NM_001142279 | RNASEH2B | frameshift_deleti  on | exon3 | c.149delT | p.I50fs | . | . |
| CTC2 | 13 | 52990217 | CT | C | 0.333 | NM_001282168 | VPS36 | frameshift_deleti  on | exon13 | c.974delA | p.K325fs | . | . |
| CTC2 | 13 | 53239843 | T | TA | 0.852 | NM_006704 | SUGT1 | frameshift_inserti  on | exon9 | c.495dupA | p.V165fs | . | . |
| CTC2 | 13 | 53422441 | G | GT | 1 | NM_002590 | PCDH8 | frameshift_inserti  on | exon1 | c.130dupA | p.T44fs | . | . |
| CTC2 | 13 | 61987634 | TG | T | 0.476 | NM_022843 | PCDH20 | frameshift_deleti  on | exon2 | c.597delC | p.I199fs | . | . |
| CTC2 | 13 | 75936412 | TG | T | 0.857 | NM_001286658 | TBC1D4 | frameshift_deleti  on | exon2 | c.829delC | p.H277fs | . | . |
| CTC2 | 13 | 75936587 | AG | A | 0.309 | NM_001286658 | TBC1D4 | frameshift_deleti  on | exon2 | c.654delC | p.P218fs | . | . |
| CTC2 | 13 | 76395548 | AG | A | 0.204 | NM_015842 | LMO7 | frameshift_deleti  on | exon11 | c.1745delG | p.S582fs | . | . |
| CTC2 | 13 | 76395734 | AG | A | 0.5 | NM_015842 | LMO7 | frameshift_deleti  on | exon11 | c.1931delG | p.R644fs | . | . |
| CTC2 | 13 | 76429454 | GC | G | 0.339 | NM_005358 | LMO7 | frameshift_deleti  on | exon28 | c.3875delC | p.A1292fs | . | . |
| CTC2 | 13 | 77570074 | TA | T | 0.4 | NM_006493 | CLN5 | frameshift_deleti  on | exon3 | c.525delA | p.L175fs | . | . |
| CTC2 | 13 | 77635324 | AC | A | 0.181 | NM_015057 | MYCBP2 | frameshift_deleti  on | exon76 | c.13015delG | p.V4339fs | . | . |
| CTC2 | 13 | 77715040 | C | CT | 0.471 | NM_015057 | MYCBP2 | frameshift_inserti  on | exon50 | c.7341dupA | p.G2448fs | . | . |
| CTC2 | 13 | 77760018 | A | AT | 0.88 | NM_015057 | MYCBP2 | frameshift_inserti  on | exon31 | c.4431_4432ins  A | p.Y1478fs | . | . |
| CTC2 | 13 | 77825334 | T | TG | 0.231 | NM_015057 | MYCBP2 | frameshift_inserti  on | exon15 | c.2332_2333ins  C | p.Y778fs | . | . |
| CTC2 | 13 | 88328110 | AG | A | 0.214 | NM_015567 | SLITRK5 | frameshift_deleti  on | exon2 | c.468delG | p.E156fs | . | . |
| CTC2 | 13 | 95715056 | TA | T | 0.6 | NM_005845 | ABCC4 | frameshift_deleti  on | exon26 | c.3267delT | p.F1089fs | . | . |
| CTC2 | 13 | 96282287 | CA | C | 1 | NM_014934 | DZIP1 | frameshift_deleti  on | exon7 | c.765delT | p.S255fs | . | . |
| CTC2 | 13 | 98009741 | TC | T | 0.471 | NM_144778 | MBNL2 | frameshift_deleti  on | exon6 | c.811delC | p.P271fs | . | . |
| CTC2 | 13 | 100921019 | TG | T | 0.333 | NM_001127692 | PCCA | frameshift_deleti  on | exon10 | c.819delG | p.V273fs | . | . |
| CTC2 | 13 | 103390832 | CT | C | 0.556 | NM_001146197 | CCDC168 | frameshift_deleti  on | exon4 | c.12214delA | p.R4072fs | . | . |
| CTC2 | 13 | 103400977 | TA | T | 0.314 | NM_001146197 | CCDC168 | stopgain | exon4 | c.2069delT | p.L690X | . | . |
| CTC2 | 13 | 103401551 | GT | G | 0.538 | NM_001146197 | CCDC168 | frameshift_deleti on | exon4 | c.1495delA | p.T499fs | . | . |
| CTC2 | 13 | 103527950 | A | AT | 0.435 | NM_000123 | ERCC5 | frameshift_inserti  on | exon15 | c.3259dupT | p.G1086fs | . | . |
| CTC2 | 13 | 106118674 | CT | C | 0.281 | NM_172370 | DAOA | frameshift_deleti  on | exon1 | c.38delT | p.L13fs | . | . |
| CTC2 | 13 | 106119437 | GT | G | 0.438 | NM_172370 | DAOA | frameshift_deleti  on | exon2 | c.81delT | p.G27fs | . | . |
| CTC2 | 13 | 110814757 | CG | C | 0.297 | NM_001845 | COL4A1 | frameshift_deleti  on | exon48 | c.4281delC | p.P1427fs | . | . |
| CTC2 | 13 | 110819508 | GA | G | 0.203 | NM_001845 | COL4A1 | frameshift_deleti  on | exon44 | c.3945delT | p.F1315fs | . | . |
| CTC2 | 13 | 110827000 | TA | T | 1 | NM_001845 | COL4A1 | frameshift_deleti  on | exon38 | c.3294delT | p.L1098fs | . | . |
| CTC2 | 13 | 111896231 | AT | A | 1 | NM_001113512 | ARHGEF7 | frameshift_deleti  on | exon6 | c.686delT | p.I229fs | . | . |
| CTC2 | 13 | 113086811 | CT | C | 0.164 | NM_145248 | SPACA7 | frameshift_deleti  on | exon6 | c.500delT | p.L167fs | . | . |
| CTC2 | 13 | 113208379 | CG | C | 1 | NM_001286277 | TUBGCP3 | frameshift_deleti  on | exon8 | c.927delC | p.L309fs | . | . |
| CTC2 | 14 | 20404486 | AT | A | 0.375 | NM_001004063 | OR4K1 | frameshift_deleti  on | exon1 | c.662delT | p.I221fs | . | . |
| CTC2 | 14 | 20612657 | AT | A | 0.35 | NM_001004724 | OR4N5 | frameshift_deleti  on | exon1 | c.764delT | p.I255fs | . | . |
| CTC2 | 14 | 20711023 | CT | C | 0.298 | NM_001004479 | OR11H4 | frameshift_deleti  on | exon1 | c.74delT | p.L25fs | . | . |
| CTC2 | 14 | 20841474 | AGG | A | 0.327 | NM_007110 | TEP1 | frameshift_deleti  on | exon47 | c.6767_6768del | p.A2256fs | . | . |
| CTC2 | 14 | 20872023 | CT | C | 0.203 | NM_007110 | TEP1 | frameshift_deleti  on | exon6 | c.1052delA | p.K351fs | . | . |
| CTC2 | 14 | 23391694 | CA | C | 0.25 | NM_001282956 | PRMT5 | frameshift_deleti  on | exon11 | c.1140delT | p.F380fs | . | . |
| CTC2 | 14 | 23548777 | T | TGAAC  GA | 0.932 | NM_001164815 | ACIN1 | nonframeshift_in  sertion | exon5 | c.1820_1821ins  TCGTTC | p.S607delinsSR  S | . | . |
| CTC2 | 14 | 23548783 | A | AGAAC  GT | 0.994 | NM_001164815 | ACIN1 | nonframeshift_in  sertion | exon5 | c.1814_1815ins  ACGTTC | p.S605delinsSR  S | . | . |
| CTC2 | 14 | 23548784 | G | GAACG  TA | 0.926 | NM_001164815 | ACIN1 | nonframeshift_in  sertion | exon5 | c.1813_1814ins  TACGTT | p.S605delinsLR  S | . | . |
| CTC2 | 14 | 23548787 | C | CGTGA  AT | 0.947 | NM_001164815 | ACIN1 | nonframeshift_in  sertion | exon5 | c.1810_1811ins  ATTCAC | p.R604delinsHS  R | . | . |
| CTC2 | 14 | 23548793 | C | CGTGA  AA | 0.96 | NM_001164815 | ACIN1 | nonframeshift_in  sertion | exon5 | c.1804_1805ins  TTTCAC | p.R602delinsLS  R | . | . |
| CTC2 | 14 | 23548794 | G | GTGAA  CA | 0.953 | NM_001164815 | ACIN1 | nonframeshift_in  sertion | exon5 | c.1803_1804ins  TGTTCA | p.R602delinsCS  R | . | . |
| CTC2 | 14 | 23548797 | A | AACGT  GAC | 0.913 | NM_001164815 | ACIN1 | frameshift_inserti  on | exon5 | c.1800_1801ins  GTCACGT | p.S601fs | . | . |
| CTC2 | 14 | 23600697 | GC | G | 0.174 | NM_001267037 | SLC7A8 | frameshift_deleti  on | exon5 | c.413delG | p.C138fs | . | . |
| CTC2 | 14 | 23991502 | TC | T | 0.638 | NM_033400 | ZFHX2 | frameshift_deleti  on | exon10 | c.7387delG | p.E2463fs | . | . |
| CTC2 | 14 | 23994056 | CA | C | 0.232 | NM_033400 | ZFHX2 | frameshift_deleti  on | exon9 | c.5094delT | p.L1698fs | . | . |
| CTC2 | 14 | 24534335 | ACCCCC  T | A | 0.786 | NM_138360 | LRRC16B | nonframeshift_de  letion | exon33 | c.3250_3255del | p.1084_1085de  l | . | . |
| CTC2 | 14 | 24610341 | AC | A | 0.329 | NM_016049 | EMC9 | frameshift_deleti  on | exon2 | c.172delG | p.V58fs | . | . |
| CTC2 | 14 | 24615750 | CG | C | 1 | NM_002818 | PSME2 | frameshift_deleti  on | exon1 | c.40delC | p.R14fs | . | . |
| CTC2 | 14 | 24646406 | G | GGAA | 0.995 | NM_001048205 | REC8 | nonframeshift_in  sertion | exon8 | c.681_682insG  AA | p.E227delinsEE | . | . |
| CTC2 | 14 | 24646409 | A | AGAC | 0.891 | NM_001048205 | REC8 | nonframeshift_in  sertion | exon8 | c.684_685insG  AC | p.E228delinsED | . | . |

| CTC2 | 14 | 30133016 | AT | A | 1 | NM_002742 | PRKD1 | frameshift_deleti  on | exon4 | c.584delA | p.N195fs | . | . |
| --- | --- | --- | --- | --- | --- | --- | --- | --- | --- | --- | --- | --- | --- |
| CTC2 | 14 | 31371744 | GT | G | 0.556 | NM_014574 | STRN3 | frameshift_deleti  on | exon14 | c.1882delA | p.T628fs | . | . |
| CTC2 | 14 | 31922483 | AT | A | 0.231 | NM_080664 | DTD2 | frameshift_deleti  on | exon2 | c.178delA | p.M60fs | . | . |
| CTC2 | 14 | 33292449 | GTC | G | 0.603 | NM_004274 | AKAP6 | frameshift_deleti  on | exon13 | c.5431_5432del | p.S1811fs | . | . |
| CTC2 | 14 | 39777765 | TC | T | 0.179 | NM_001247988 | CTAGE5 | frameshift_deleti  on | exon13 | c.1081delC | p.Q361fs | . | . |
| CTC2 | 14 | 44974033 | GT | G | 0.667 | NM_032135 | FSCB | frameshift_deleti  on | exon1 | c.2157delA | p.P719fs | . | . |
| CTC2 | 14 | 45475393 | TG | T | 0.722 | NM_015091 | FAM179B | stopgain | exon5 | c.2828delG | p.W943X | . | . |
| CTC2 | 14 | 45696908 | AT | A | 0.303 | NM_018353 | MIS18BP1 | frameshift_deleti on | exon9 | c.1703delA | p.N568fs | . | . |
| CTC2 | 14 | 51058324 | AT | A | 1 | NM_015915 | ATL1 | frameshift_deleti  on | exon4 | c.490delT | p.F164fs | . | . |
| CTC2 | 14 | 51223917 | TAG | T | 1 | NM_020921 | NIN | frameshift_deleti  on | exon18 | c.3829_3830del | p.L1277fs | . | . |
| CTC2 | 14 | 51233573 | A | AT | 0.605 | NM_016350 | NIN | frameshift_inserti  on | exon13 | c.1469dupA | p.N490fs | . | . |
| CTC2 | 14 | 53360081 | CT | C | 0.889 | NM_001134999 | FERMT2 | frameshift_deleti  on | exon4 | c.455delA | p.K152fs | . | . |
| CTC2 | 14 | 54897100 | TC | T | 0.883 | NM_005776 | CNIH1 | frameshift_deleti  on | exon4 | c.285delG | p.M95fs | . | . |
| CTC2 | 14 | 55453902 | GC | G | 0.444 | NM_001008396 | WDHD1 | frameshift_deleti  on | exon13 | c.1360delG | p.A454fs | . | . |
| CTC2 | 14 | 56756986 | G | GA | 0.331 | NM_021255 | PELI2 | frameshift_inserti  on | exon5 | c.509dupA | p.E170fs | . | . |
| CTC2 | 14 | 59113335 | TG | T | 0.238 | NM_001079520 | DACT1 | frameshift_deleti  on | exon4 | c.1884delG | p.V628fs | . | . |
| CTC2 | 14 | 59797400 | CA | C | 0.207 | NM_014992 | DAAM1 | frameshift_deleti  on | exon12 | c.1555delA | p.S519fs | . | . |
| CTC2 | 14 | 59834303 | CG | C | 0.162 | NM_001270520 | DAAM1 | frameshift_deleti  on | exon24 | c.2984delG | p.R995fs | . | . |
| CTC2 | 14 | 61190487 | CGCG | C | 0.779 | NM_017420 | SIX4 | nonframeshift_de  letion | exon1 | c.303_305del | p.101_102del | . | . |
| CTC2 | 14 | 62249093 | GAAGA | G | 0.6 | NM_003082 | SNAPC1 | frameshift_deleti  on | exon8 | c.955_958del | p.K319fs | . | . |
| CTC2 | 14 | 63175098 | TGA | T | 0.148 | NM_139318 | KCNH5 | frameshift_deleti  on | exon11 | c.2093_2094del | p.L698fs | . | . |
| CTC2 | 14 | 64687322 | AG | A | 0.36 | NM_182910 | SYNE2 | frameshift_deleti  on | exon4 | c.553delG | p.G185fs | . | . |
| CTC2 | 14 | 68129223 | CA | C | 1 | NM_006370 | VTI1B | frameshift_deleti  on | exon2 | c.144delT | p.F48fs | . | . |
| CTC2 | 14 | 68191886 | TAC | T | 0.429 | NM_152443 | RDH12 | frameshift_deleti  on | exon5 | c.259_260del | p.T87fs | . | . |
| CTC2 | 14 | 68191922 | CCTATCC  GACACC | C | 0.2 | NM_152443 | RDH12 | frameshift_deleti  on | exon5 | c.295_310del | p.L99fs | . | . |
| CTC2 | 14 | 70991406 | CA | C | 0.5 | NM_003814 | ADAM20 | frameshift_deleti  on | exon2 | c.218delT | p.L73fs | . | . |
| CTC2 | 14 | 72818880 | CA | C | 0.309 | NM_001204423 | RGS6 | frameshift_deleti  on | exon2 | c.58delA | p.K20fs | . | . |
| CTC2 | 14 | 73685872 | AT | A | 0.636 | NM_000021 | PSEN1 | frameshift_deleti  on | exon12 | c.1280delT | p.I427fs | . | . |
| CTC2 | 14 | 74823930 | GC | G | 0.923 | NM_018228 | VRTN | frameshift_deleti  on | exon2 | c.445delC | p.P149fs | . | . |
| CTC2 | 14 | 76437548 | C | CT | 0.301 | NM_003239 | TGFB3 | frameshift_inserti  on | exon3 | c.566dupA | p.K189fs | . | . |
| CTC2 | 14 | 77333746 | GA | G | 0.769 | NM_194287 | C14orf166B | frameshift_deleti  on | exon13 | c.1408delA | p.K470fs | . | . |
| CTC2 | 14 | 77872382 | TG | T | 0.342 | NM_001113475 | NOXRED1 | frameshift_deleti  on | exon5 | c.778delC | p.Q260fs | . | . |
| CTC2 | 14 | 79423609 | CA | C | 0.33 | NM_004796 | NRXN3 | frameshift_deleti  on | exon8 | c.1182delA | p.A394fs | . | . |
| CTC2 | 14 | 81736959 | TA | T | 0.5 | NM_033104 | STON2 | frameshift_deleti  on | exon6 | c.2667delT | p.T889fs | . | . |
| CTC2 | 14 | 81736960 | AG | A | 0.5 | NM_033104 | STON2 | frameshift_deleti  on | exon6 | c.2666delC | p.T889fs | . | . |
| CTC2 | 14 | 81862456 | TC | T | 0.267 | NM_033104 | STON2 | frameshift_deleti  on | exon3 | c.154delG | p.E52fs | . | . |
| CTC2 | 14 | 89305892 | GA | G | 0.66 | NM_001288781 | TTC8 | frameshift_deleti  on | exon3 | c.212delA | p.E71fs | . | . |
| CTC2 | 14 | 91340133 | AG | A | 1 | NM_004755 | RPS6KA5 | frameshift_deleti  on | exon16 | c.2002delC | p.L668fs | . | . |
| CTC2 | 14 | 91805746 | TG | T | 1 | NM_001080414 | CCDC88C | frameshift_deleti  on | exon8 | c.684delC | p.P228fs | . | . |
| CTC2 | 14 | 92470658 | AT | A | 0.344 | NM_004239 | TRIP11 | frameshift_deleti  on | exon11 | c.3661delA | p.M1221fs | . | . |
| CTC2 | 14 | 92915439 | CT | C | 0.326 | NM_153646 | SLC24A4 | frameshift_deleti  on | exon10 | c.760delT | p.F254fs | . | . |
| CTC2 | 14 | 93151477 | C | CT | 0.257 | NM_024832 | RIN3 | frameshift_inserti  on | exon9 | c.2614dupT | p.A871fs | . | . |
| CTC2 | 14 | 93396101 | GT | G | 0.514 | NM_001275 | CHGA | frameshift_deleti  on | exon5 | c.297delT | p.G99fs | . | . |
| CTC2 | 14 | 93708631 | CT | C | 0.889 | NM_001289133 | BTBD7 | frameshift_deleti  on | exon9 | c.2333delA | p.K778fs | . | . |
| CTC2 | 14 | 94526771 | AT | A | 0.538 | NM_020414 | DDX24 | frameshift_deleti  on | exon5 | c.1585delA | p.M529fs | . | . |
| CTC2 | 14 | 94582131 | G | GGCCA  TGGCA | 0.918 | NM_001288959 | IFI27 | nonframeshift_in  sertion | exon3 | c.1_2insGCCAT  GGCA | p.G1delinsGHG  S | . | . |
| CTC2 | 14 | 95085645 | TA | T | 0.267 | NM_001085 | SERPINA3 | frameshift_deleti  on | exon3 | c.758delA | p.Y253fs | . | . |
| CTC2 | 14 | 95557625 | CG | C | 0.441 | NM_001271282 | DICER1 | frameshift_deleti  on | exon25 | c.5441delC | p.S1814fs | . | . |
| CTC2 | 14 | 95599788 | CT | C | 0.5 | NM_001195573 | DICER1 | frameshift_deleti  on | exon1 | c.7delA | p.S3fs | . | . |
| CTC2 | 14 | 96157102 | GC | G | 0.366 | NM_004918 | TCL1B | frameshift_deleti  on | exon2 | c.193delC | p.Q65fs | . | . |
| CTC2 | 14 | 96797840 | G | GA | 0.208 | NM_018036 | ATG2B | frameshift_inserti  on | exon11 | c.1602dupT | p.P535fs | . | . |
| CTC2 | 14 | 99866455 | GT | G | 0.431 | NM_032233 | SETD3 | frameshift_deleti  on | exon12 | c.1318delA | p.T440fs | . | . |
| CTC2 | 14 | 100381022 | CA | C | 1 | NM_004434 | EML1 | frameshift_deleti  on | exon15 | c.1741delA | p.K581fs | . | . |
| CTC2 | 14 | 102252499 | CA | C | 1 | NM_001161726 | PPP2R5C | frameshift_deleti  on | exon3 | c.239delA | p.Q80fs | . | . |
| CTC2 | 14 | 102505523 | TC | T | 1 | NM_001376 | DYNC1H1 | frameshift_deleti  on | exon60 | c.11393delC | p.S3798fs | . | . |
| CTC2 | 14 | 102717326 | TG | T | 0.364 | NM_001272011 | MOK | frameshift_deleti  on | exon6 | c.322delC | p.Q108fs | . | . |
| CTC2 | 14 | 102843071 | TC | T | 0.894 | NM_001172631 | TECPR2 | stopgain | exon2 | c.14delC | p.S5X | . | . |
| CTC2 | 14 | 103434929 | G | GA | 0.727 | NM_006035 | CDC42BPB | frameshift_inserti on | exon15 | c.2119dupT | p.S707fs | . | . |

| CTC2 | 14 | 104040472 | TA | T | 1 | NM_032374 | APOPT1 | frameshift_deleti  on | exon3 | c.390delA | p.L130fs | . | . |
| --- | --- | --- | --- | --- | --- | --- | --- | --- | --- | --- | --- | --- | --- |
| CTC2 | 14 | 104492052 | AT | A | 0.583 | NM_153046 | TDRD9 | frameshift_deleti  on | exon26 | c.2871delT | p.D957fs | . | . |
| CTC2 | 14 | 105180792 | AG | A | 0.364 | NM_001031714 | INF2 | frameshift_deleti  on | exon21 | c.3294delG | p.E1098fs | . | . |
| CTC2 | 14 | 105181037 | GC | G | 0.438 | NM_001031714 | INF2 | frameshift_deleti  on | exon21 | c.3539delC | p.A1180fs | . | . |
| CTC2 | 14 | 105409879 | AT | A | 0.735 | NM_138420 | AHNAK2 | frameshift_deleti  on | exon7 | c.11908delA | p.M3970fs | . | . |
| CTC2 | 14 | 105415789 | AC | A | 1 | NM_138420 | AHNAK2 | frameshift_deleti  on | exon7 | c.5998delG | p.V2000fs | . | . |
| CTC2 | 14 | 105416371 | TC | T | 1 | NM_138420 | AHNAK2 | frameshift_deleti  on | exon7 | c.5416delG | p.E1806fs | . | . |
| CTC2 | 14 | 105859569 | GC | G | 1 | NM_001243127 | PACS2 | frameshift_deleti  on | exon23 | c.2224delC | p.P742fs | . | . |
| CTC2 | 15 | 23049294 | GAGC | G | 0.289 | NM_001142275 | NIPA1 | nonframeshift_de  letion | exon5 | c.297_299del | p.99_100del | . | . |
| CTC2 | 15 | 28211899 | GA | G | 0.3 | NM_000275 | OCA2 | frameshift_deleti  on | exon15 | c.1572delT | p.F524fs | . | . |
| CTC2 | 15 | 28424331 | CA | C | 0.419 | NM_004667 | HERC2 | stopgain | exon58 | c.8975delT | p.L2992X | . | . |
| CTC2 | 15 | 28437122 | C | CT | 0.19 | NM_004667 | HERC2 | frameshift_inserti on | exon53 | c.8435_8436ins A | p.S2812fs | . | . |
| CTC2 | 15 | 28465661 | GC | G | 0.588 | NM_004667 | HERC2 | frameshift_deleti  on | exon37 | c.5781delG | p.K1927fs | . | . |
| CTC2 | 15 | 28508205 | TG | T | 0.97 | NM_004667 | HERC2 | frameshift_deleti  on | exon15 | c.1980delC | p.V660fs | . | . |
| CTC2 | 15 | 28517430 | CA | C | 0.857 | NM_004667 | HERC2 | frameshift_deleti  on | exon9 | c.1013delT | p.L338fs | . | . |
| CTC2 | 15 | 29397738 | TC | T | 0.889 | NM_001130414 | APBA2 | stopgain | exon9 | c.1646delC | p.S549X | . | . |
| CTC2 | 15 | 30000851 | TG | T | 1 | NM_175610 | TJP1 | frameshift_deleti on | exon24 | c.4521delC | p.D1507fs | . | . |
| CTC2 | 15 | 30010769 | CA | C | 0.389 | NM_175610 | TJP1 | frameshift_deleti  on | exon20 | c.3336delT | p.F1112fs | . | . |
| CTC2 | 15 | 30700168 | C | CTTG | 0.232 | NM_001282484 | GOLGA8R | nonframeshift_in  sertion | exon11 | c.813_814insCA  A | p.D272delinsQ  D | . | . |
| CTC2 | 15 | 30926625 | AG | A | 0.226 | NM_001039841 | ARHGAP11B | frameshift_deleti  on | exon4 | c.551delG | p.R184fs | . | . |
| CTC2 | 15 | 33445279 | TGG | T | 0.143 | NM_001277313 | FMN1 | frameshift_deleti  on | exon4 | c.1835_1836del | p.T612fs | . | . |
| CTC2 | 15 | 33926833 | TG | T | 0.169 | NM_001036 | RYR3 | frameshift_deleti  on | exon25 | c.3075delG | p.L1025fs | . | . |
| CTC2 | 15 | 34376676 | CT | C | 0.283 | NM_020154 | EMC7 | frameshift_deleti  on | exon5 | c.587delA | p.Q196fs | . | . |
| CTC2 | 15 | 34628788 | TGG | T | 1 | NM_001042497 | SLC12A6 | frameshift_deleti  on | exon1 | c.92_93del | p.T31fs | . | . |
| CTC2 | 15 | 37184461 | TC | T | 0.586 | NM_170675 | MEIS2 | frameshift_deleti  on | exon12 | c.1346delG | p.G449fs | . | . |
| CTC2 | 15 | 38794544 | GA | G | 0.519 | NM_001128602 | RASGRP1 | frameshift_deleti  on | exon11 | c.1401delT | p.F467fs | . | . |
| CTC2 | 15 | 39881239 | TC | T | 1 | NM_003246 | THBS1 | frameshift_deleti  on | exon11 | c.1726delC | p.P576fs | . | . |
| CTC2 | 15 | 41071772 | AG | A | 0.658 | NM_018163 | DNAJC17 | frameshift_deleti  on | exon3 | c.177delC | p.A59fs | . | . |
| CTC2 | 15 | 41364137 | AC | A | 1 | NM_017553 | INO80 | frameshift_deleti  on | exon12 | c.1514delG | p.G505fs | . | . |
| CTC2 | 15 | 41813032 | AG | A | 0.755 | NM_015540 | RPAP1 | frameshift_deleti  on | exon22 | c.3351delC | p.P1117fs | . | . |
| CTC2 | 15 | 42115873 | TC | T | 0.6 | NM_014994 | MAPKBP1 | frameshift_deleti  on | exon29 | c.3828delC | p.V1276fs | . | . |
| CTC2 | 15 | 42154798 | TGAGGC  CGTGGG CTGCCT CGGGGC | T | 0.308 | NM_016642 | SPTBN5 | nonframeshift_de letion | exon43 | c.7291_7323del | p.2431_2441de l | . | . |
| CTC2 | 15 | 42442920 | C | CA | 0.474 | NM_213600 | PLA2G4F | frameshift_inserti  on | exon8 | c.656dupT | p.L219fs | . | . |
| CTC2 | 15 | 42483324 | TA | T | 0.429 | NM_015289 | VPS39 | stopgain | exon4 | c.245delT | p.L82X | . | . |
| CTC2 | 15 | 42492143 | C | CA | 0.4 | NM_015289 | VPS39 | frameshift_inserti on | exon2 | c.89dupT | p.V30fs | . | . |
| CTC2 | 15 | 42749171 | CT | C | 0.462 | NM_022473 | ZNF106 | frameshift_deleti  on | exon1 | c.232delA | p.R78fs | . | . |
| CTC2 | 15 | 42978446 | TG | T | 0.992 | NM_020759 | STARD9 | frameshift_deleti  on | exon23 | c.4671delG | p.L1557fs | . | . |
| CTC2 | 15 | 43256186 | AT | A | 1 | NM_174916 | UBR1 | frameshift_deleti  on | exon42 | c.4646delA | p.N1549fs | . | . |
| CTC2 | 15 | 43545804 | AG | A | 0.308 | NM_004245 | TGM5 | stopgain | exon4 | c.337delC | p.L113X | . | . |
| CTC2 | 15 | 43577093 | TTG | T | 0.137 | NM_052955 | TGM7 | frameshift_deleti on | exon7 | c.921_922del | p.H307fs | . | . |
| CTC2 | 15 | 43662007 | AG | A | 0.798 | NM_152455 | ZSCAN29 | frameshift_deleti  on | exon1 | c.104delC | p.A35fs | . | . |
| CTC2 | 15 | 43713286 | GCCTT | G | 0.859 | NM_001141979 | TP53BP1 | frameshift_deleti  on | exon20 | c.4183_4186del | p.K1395fs | . | . |
| CTC2 | 15 | 43895520 | C | CA | 0.286 | NM_153700 | STRC | stopgain | exon23 | c.4464dupT | p.E1489_D1490  delinsX | . | . |
| CTC2 | 15 | 43990331 | GC | G | 0.727 | NM_001015001 | CKMT1A | frameshift_deleti  on | exon8 | c.997delC | p.P333fs | . | . |
| CTC2 | 15 | 44134710 | GC | G | 0.197 | NM_001167941 | WDR76 | frameshift_deleti  on | exon6 | c.639delC | p.S213fs | . | . |
| CTC2 | 15 | 44967799 | TCC | T | 0.723 | NM_001145112 | PATL2 | frameshift_deleti  on | exon3 | c.105_106del | p.E35fs | . | . |
| CTC2 | 15 | 45444549 | CG | C | 0.444 | NM_175940 | DUOX1 | frameshift_deleti  on | exon25 | c.3260delG | p.R1087fs | . | . |
| CTC2 | 15 | 45491243 | GCTC | G | 0.161 | NM_138356 | SHF | nonframeshift_de  letion | exon2 | c.27_29del | p.9_10del | . | . |
| CTC2 | 15 | 45661533 | CA | C | 0.5 | NM_001482 | GATM | frameshift_deleti  on | exon3 | c.474delT | p.F158fs | . | . |
| CTC2 | 15 | 49034180 | CA | C | 0.4 | NM_014985 | CEP152 | frameshift_deleti  on | exon24 | c.3784delT | p.C1262fs | . | . |
| CTC2 | 15 | 49118203 | C | CT | 0.344 | NM_203349 | SHC4 | frameshift_inserti  on | exon12 | c.1857dupA | p.D620fs | . | . |
| CTC2 | 15 | 50866615 | A | AT | 0.186 | NM_017672 | TRPM7 | frameshift_inserti  on | exon36 | c.5163dupA | p.C1722fs | . | . |
| CTC2 | 15 | 51291347 | GA | G | 0.494 | NM_001252127 | AP4E1 | frameshift_deleti  on | exon19 | c.2759delA | p.E920fs | . | . |
| CTC2 | 15 | 51772993 | TG | T | 0.253 | NM_001174117 | DMXL2 | frameshift_deleti  on | exon22 | c.4401delC | p.S1467fs | . | . |
| CTC2 | 15 | 51988112 | GA | G | 0.441 | NM_001165257 | SCG3 | frameshift_deleti  on | exon7 | c.214delA | p.K72fs | . | . |
| CTC2 | 15 | 52258028 | T | TA | 0.282 | NM_001286430 | LEO1 | frameshift_inserti  on | exon2 | c.731_732insT | p.Q244fs | . | . |

| CTC2 | 15 | 52901648 | CT | C | 0.778 | NM_001286495 | FAM214A | frameshift_deleti  on | exon5 | c.1483delA | p.R495fs | . | . |
| --- | --- | --- | --- | --- | --- | --- | --- | --- | --- | --- | --- | --- | --- |
| CTC2 | 15 | 52902150 | TA | T | 0.222 | NM_001286495 | FAM214A | frameshift_deleti  on | exon5 | c.981delT | p.F327fs | . | . |
| CTC2 | 15 | 53049989 | CT | C | 0.394 | NM_004498 | ONECUT1 | frameshift_deleti  on | exon2 | c.1160delA | p.K387fs | . | . |
| CTC2 | 15 | 55710624 | G | GT | 0.423 | NM_001198784 | C15orf65 | frameshift_inserti  on | exon2 | c.223dupT | p.Q74fs | . | . |
| CTC2 | 15 | 57734627 | TC | T | 0.516 | NM_032866 | CGNL1 | frameshift_deleti  on | exon4 | c.1755delC | p.I585fs | . | . |
| CTC2 | 15 | 59465980 | CG | C | 0.551 | NM_004998 | MYO1E | frameshift_deleti  on | exon21 | c.2298delC | p.F766fs | . | . |
| CTC2 | 15 | 59910604 | TG | T | 0.8 | NM_004751 | GCNT3 | frameshift_deleti  on | exon3 | c.168delG | p.L56fs | . | . |
| CTC2 | 15 | 60734717 | CT | C | 0.372 | NM_001018089 | NARG2 | frameshift_deleti  on | exon12 | c.1911delA | p.K637fs | . | . |
| CTC2 | 15 | 60793253 | AT | A | 0.267 | NM_134262 | RORA | frameshift_deleti  on | exon8 | c.1033delA | p.I345fs | . | . |
| CTC2 | 15 | 62209628 | TC | T | 0.308 | NM_017684 | VPS13C | frameshift_deleti  on | exon58 | c.7837delG | p.E2613fs | . | . |
| CTC2 | 15 | 63356309 | GC | G | 0.769 | NM_000366 | TPM1 | frameshift_deleti  on | exon9 | c.820delC | p.L274fs | . | . |
| CTC2 | 15 | 63955325 | GC | G | 1 | NM_003922 | HERC1 | frameshift_deleti  on | exon44 | c.8758delG | p.A2920fs | . | . |
| CTC2 | 15 | 64430310 | TTC | T | 0.186 | NM_001242933 | SNX1 | frameshift_deleti  on | exon15 | c.1589_1590del | p.F530fs | . | . |
| CTC2 | 15 | 64792139 | GAGT | G | 0.429 | NM_015042 | ZNF609 | nonframeshift_de  letion | exon1 | c.522_524del | p.174_175del | . | . |
| CTC2 | 15 | 65864661 | AT | A | 0.791 | NM_016395 | PTPLAD1 | frameshift_deleti  on | exon10 | c.970delT | p.F324fs | . | . |
| CTC2 | 15 | 66209272 | GC | G | 1 | NM_032445 | MEGF11 | frameshift_deleti  on | exon17 | c.2108delG | p.G703fs | . | . |
| CTC2 | 15 | 66625414 | TA | T | 0.165 | NM_001143688 | DIS3L | frameshift_deleti  on | exon17 | c.2930delA | p.Y977fs | . | . |
| CTC2 | 15 | 71124464 | AC | A | 0.619 | NM_001286679 | LARP6 | frameshift_deleti  on | exon3 | c.850delG | p.V284fs | . | . |
| CTC2 | 15 | 71276480 | GCAA | G | 1 | NM_001199017 | LRRC49 | nonframeshift_de  letion | exon11 | c.1069_1071del | p.357_357del | . | . |
| CTC2 | 15 | 72109940 | CG | C | 0.261 | NM_014249 | NR2E3 | frameshift_deleti  on | exon9 | c.1149delG | p.A383fs | . | . |
| CTC2 | 15 | 72192209 | AC | A | 0.25 | NM_006901 | MYO9A | frameshift_deleti  on | exon24 | c.3288delG | p.E1096fs | . | . |
| CTC2 | 15 | 72338371 | AT | A | 0.3 | NM_006901 | MYO9A | frameshift_deleti  on | exon2 | c.533delA | p.Y178fs | . | . |
| CTC2 | 15 | 72462224 | TC | T | 0.375 | NM_001012642 | GRAMD2 | frameshift_deleti  on | exon2 | c.97delG | p.E33fs | . | . |
| CTC2 | 15 | 72641578 | AG | A | 0.635 | NM_000520 | HEXA | frameshift_deleti  on | exon8 | c.827delC | p.P276fs | . | . |
| CTC2 | 15 | 72645482 | CG | C | 0.242 | NM_000520 | HEXA | frameshift_deleti  on | exon5 | c.496delC | p.R166fs | . | . |
| CTC2 | 15 | 72647949 | AT | A | 0.548 | NM_000520 | HEXA | frameshift_deleti  on | exon3 | c.362delA | p.N121fs | . | . |
| CTC2 | 15 | 73996650 | GC | G | 1 | NM_025240 | CD276 | frameshift_deleti  on | exon4 | c.553delC | p.P185fs | . | . |
| CTC2 | 15 | 74483829 | GC | G | 0.274 | NM_001142617 | STRA6 | frameshift_deleti  on | exon9 | c.723delG | p.G241fs | . | . |
| CTC2 | 15 | 74536400 | TAAG | T | 0.346 | NM_025055 | CCDC33 | nonframeshift_de  letion | exon2 | c.97_99del | p.33_33del | . | . |
| CTC2 | 15 | 77310850 | GC | G | 0.353 | NM_003978 | PSTPIP1 | frameshift_deleti  on | exon3 | c.191delC | p.A64fs | . | . |
| CTC2 | 15 | 77425681 | GA | G | 0.912 | NM_024776 | PEAK1 | frameshift_deleti  on | exon7 | c.3742delT | p.S1248fs | . | . |
| CTC2 | 15 | 78893622 | AT | A | 0.494 | NM_000743 | CHRNA3 | frameshift_deleti  on | exon5 | c.1361delA | p.N454fs | . | . |
| CTC2 | 15 | 79606227 | GT | G | 1 | NM_007364 | TMED3 | frameshift_deleti  on | exon2 | c.298delT | p.F100fs | . | . |
| CTC2 | 15 | 81166234 | GGA | G | 0.5 | NM_018689 | CEMIP | frameshift_deleti  on | exon2 | c.15_16del | p.G5fs | . | . |
| CTC2 | 15 | 81166235 | GA | G | 0.952 | NM_018689 | CEMIP | frameshift_deleti  on | exon2 | c.16delA | p.R6fs | . | . |
| CTC2 | 15 | 83428241 | GA | G | 0.577 | NM_001281806 | FSD2 | frameshift_deleti  on | exon12 | c.1973delT | p.F658fs | . | . |
| CTC2 | 15 | 83488134 | AT | A | 0.875 | NM_001080435 | WHAMM | frameshift_deleti  on | exon5 | c.1165delT | p.F389fs | . | . |
| CTC2 | 15 | 83499537 | CA | C | 0.314 | NM_001080435 | WHAMM | frameshift_deleti  on | exon9 | c.1829delA | p.Q610fs | . | . |
| CTC2 | 15 | 83499641 | TCCA | T | 0.273 | NM_001080435 | WHAMM | nonframeshift_de  letion | exon9 | c.1933_1935del | p.645_645del | . | . |
| CTC2 | 15 | 83523511 | AG | A | 0.353 | NM_004839 | HOMER2 | frameshift_deleti  on | exon6 | c.535delC | p.L179fs | . | . |
| CTC2 | 15 | 85189577 | CA | C | 0.167 | NM_032856 | WDR73 | frameshift_deleti  on | exon6 | c.354delT | p.D118fs | . | . |
| CTC2 | 15 | 85399807 | AT | A | 0.235 | NM_020778 | ALPK3 | frameshift_deleti  on | exon6 | c.2445delT | p.D815fs | . | . |
| CTC2 | 15 | 85400699 | A | AT | 0.6 | NM_020778 | ALPK3 | frameshift_inserti  on | exon6 | c.3336_3337ins  T | p.P1112fs | . | . |
| CTC2 | 15 | 85666416 | GA | G | 1 | NM_173454 | PDE8A | frameshift_deleti  on | exon18 | c.1940delA | p.E647fs | . | . |
| CTC2 | 15 | 86810293 | C | CT | 0.4 | NM_152336 | AGBL1 | frameshift_inserti  on | exon12 | c.1686_1687ins  T | p.A562fs | . | . |
| CTC2 | 15 | 89866103 | AG | A | 0.76 | NM_001126131 | POLG | frameshift_deleti  on | exon14 | c.2295delC | p.P765fs | . | . |
| CTC2 | 15 | 90414743 | AT | A | 1 | NM_005829 | AP3S2 | frameshift_deleti  on | exon4 | c.308delA | p.N103fs | . | . |
| CTC2 | 15 | 91292880 | GT | G | 0.406 | NM_000057 | BLM | frameshift_deleti  on | exon3 | c.383delT | p.V128fs | . | . |
| CTC2 | 15 | 91292995 | C | CA | 0.269 | NM_000057 | BLM | frameshift_inserti  on | exon3 | c.498dupA | p.S166fs | . | . |
| CTC2 | 15 | 99511804 | A | AC | 0.556 | NM_001102612 | PGPEP1L | frameshift_inserti  on | exon5 | c.493dupG | p.V165fs | . | . |
| CTC2 | 15 | 99701997 | AG | A | 0.342 | NM_001288616 | TTC23 | frameshift_deleti  on | exon9 | c.940delC | p.L314fs | . | . |
| CTC2 | 15 | 100252709 | CCAGCA  G | C | 1 | NM_001130928 | MEF2A | nonframeshift_de  letion | exon8 | c.1024_1029del | p.342_343del | . | . |
| CTC2 | 15 | 100252709 | CCAGCA  GCAG | C | 1 | NM_001130928 | MEF2A | nonframeshift_de  letion | exon8 | c.1024_1032del | p.342_344del | . | . |
| CTC2 | 15 | 100252738 | AGCAGC  C | A | 0.875 | NM_001130928 | MEF2A | nonframeshift_de  letion | exon8 | c.1053_1058del | p.351_353del | . | . |
| CTC2 | 15 | 100673426 | GC | G | 0.831 | NM_139057 | ADAMTS17 | frameshift_deleti  on | exon11 | c.1567delG | p.A523fs | . | . |
| CTC2 | 15 | 101588859 | TC | T | 0.571 | NM_024652 | LRRK1 | frameshift_deleti  on | exon22 | c.3297delC | p.L1099fs | . | . |
| CTC2 | 16 | 341240 | CG | C | 0.953 | NM_003502 | AXIN1 | frameshift_deleti  on | exon9 | c.2243delC | p.P748fs | . | . |

| CTC2 | 16 | 450140 | C | CAG | 0.976 | NM_001286435 | NME4 | frameshift_inserti  on | exon5 | c.472_473insA  G | p.R158fs | . | . |
| --- | --- | --- | --- | --- | --- | --- | --- | --- | --- | --- | --- | --- | --- |
| CTC2 | 16 | 618280 | CT | C | 0.545 | NM_176677 | NHLRC4 | frameshift_deleti  on | exon2 | c.234delT | p.A78fs | . | . |
| CTC2 | 16 | 716518 | AGCGTC  TGGGCC TCCGAC | A | 0.5 | NM_145294 | WDR90 | frameshift_deleti on | exon38 | c.4805_4826del | p.S1602fs | . | . |
| CTC2 | 16 | 1818814 | A | AC | 0.556 | NM_001040439 | MAPK8IP3 | frameshift_inserti  on | exon31 | c.3983dupC | p.T1328fs | . | . |
| CTC2 | 16 | 1836559 | TC | T | 0.629 | NM_012225 | NUBP2 | frameshift_deleti  on | exon2 | c.39delC | p.V13fs | . | . |
| CTC2 | 16 | 2024602 | AC | A | 1 | NM_006453 | TBL3 | frameshift_deleti  on | exon5 | c.302delC | p.T101fs | . | . |
| CTC2 | 16 | 2025422 | TG | T | 0.583 | NM_006453 | TBL3 | frameshift_deleti  on | exon9 | c.789delG | p.L263fs | . | . |
| CTC2 | 16 | 2126149 | AT | A | 0.306 | NM_000548 | TSC2 | frameshift_deleti  on | exon24 | c.2721delT | p.D907fs | . | . |
| CTC2 | 16 | 2260585 | CCAG | C | 0.417 | NM_182563 | BRICD5 | nonframeshift_de  letion | exon2 | c.115_117del | p.39_39del | . | . |
| CTC2 | 16 | 2481190 | AG | A | 1 | NM_001761 | CCNF | frameshift_deleti  on | exon2 | c.77delG | p.R26fs | . | . |
| CTC2 | 16 | 2812838 | AAT | A | 0.176 | NM_016333 | SRRM2 | frameshift_deleti  on | exon11 | c.2310_2311del | p.K770fs | . | . |
| CTC2 | 16 | 3633335 | GC | G | 0.299 | NM_032444 | SLX4 | frameshift_deleti  on | exon14 | c.4915delG | p.A1639fs | . | . |
| CTC2 | 16 | 4386957 | AGCC | A | 0.75 | NM_032575 | GLIS2 | nonframeshift_de  letion | exon6 | c.1008_1010del | p.336_337del | . | . |
| CTC2 | 16 | 4723606 | C | CCG | 1 | NM_001142289 | MGRN1 | frameshift_inserti  on | exon10 | c.903_904insC  G | p.S301fs | . | . |
| CTC2 | 16 | 4910770 | GA | G | 1 | NM_001079514 | UBN1 | frameshift_deleti  on | exon7 | c.778delA | p.K260fs | . | . |
| CTC2 | 16 | 4924904 | G | GC | 0.778 | NM_001079514 | UBN1 | frameshift_inserti  on | exon15 | c.2493_2494ins  C | p.K831fs | . | . |
| CTC2 | 16 | 4933899 | A | AT | 0.947 | NM_002705 | PPL | frameshift_inserti  on | exon22 | c.4756dupA | p.I1586fs | . | . |
| CTC2 | 16 | 4934228 | CAGG | C | 0.174 | NM_002705 | PPL | nonframeshift_de  letion | exon22 | c.4425_4427del | p.1475_1476de  l | . | . |
| CTC2 | 16 | 4935462 | TGG | T | 0.207 | NM_002705 | PPL | frameshift_deleti  on | exon22 | c.3192_3193del | p.P1064fs | . | . |
| CTC2 | 16 | 5050870 | CG | C | 1 | NM_014692 | SEC14L5 | frameshift_deleti  on | exon10 | c.1075delG | p.E359fs | . | . |
| CTC2 | 16 | 5140179 | TA | T | 0.429 | NM_001289029 | FAM86A | stopgain | exon4 | c.464delT | p.L155X | . | . |
| CTC2 | 16 | 9009317 | G | GA | 0.4 | NM_001286457 | USP7 | frameshift_inserti on | exon9 | c.923dupT | p.F308fs | . | . |
| CTC2 | 16 | 11362841 | CA | C | 0.6 | NM_005425 | TNP2 | frameshift_deleti  on | exon1 | c.278delT | p.M93fs | . | . |
| CTC2 | 16 | 11785761 | GC | G | 0.505 | NM_015914 | TXNDC11 | frameshift_deleti  on | exon8 | c.1284delG | p.V428fs | . | . |
| CTC2 | 16 | 12450071 | GC | G | 0.196 | NM_032167 | SNX29 | frameshift_deleti  on | exon16 | c.1835delC | p.A612fs | . | . |
| CTC2 | 16 | 14340562 | AG | A | 0.767 | NM_014048 | MKL2 | frameshift_deleti  on | exon12 | c.1446delG | p.K482fs | . | . |
| CTC2 | 16 | 14951391 | CT | C | 0.231 | NM_014287 | NOMO1 | frameshift_deleti  on | exon11 | c.1100delT | p.L367fs | . | . |
| CTC2 | 16 | 18555019 | C | CA | 0.222 | NM_001004060 | NOMO2 | frameshift_inserti  on | exon7 | c.654dupT | p.V219fs | . | . |
| CTC2 | 16 | 19063031 | AC | A | 0.636 | NM_001160364 | TMC7 | frameshift_deleti  on | exon13 | c.1435delC | p.P479fs | . | . |
| CTC2 | 16 | 19477492 | AGC | A | 1 | NM_024780 | TMC5 | frameshift_deleti  on | exon5 | c.837_838del | p.Q279fs | . | . |
| CTC2 | 16 | 19580799 | GTCC | G | 0.4 | NM_020314 | C16orf62 | nonframeshift_de  letion | exon3 | c.439_441del | p.147_147del | . | . |
| CTC2 | 16 | 20331030 | TG | T | 0.651 | NM_001007242 | GP2 | frameshift_deleti  on | exon5 | c.477delC | p.I159fs | . | . |
| CTC2 | 16 | 21245119 | AC | A | 0.452 | NM_145865 | ANKS4B | frameshift_deleti  on | exon1 | c.62delC | p.T21fs | . | . |
| CTC2 | 16 | 23541209 | G | GA | 0.436 | NM_001083614 | EARS2 | frameshift_inserti  on | exon6 | c.1076dupT | p.L359fs | . | . |
| CTC2 | 16 | 23596743 | AG | A | 0.826 | NM_005003 | NDUFAB1 | frameshift_deleti  on | exon3 | c.292delC | p.L98fs | . | . |
| CTC2 | 16 | 23713575 | TG | T | 0.909 | NM_033266 | ERN2 | frameshift_deleti  on | exon11 | c.1244delC | p.P415fs | . | . |
| CTC2 | 16 | 24372998 | CCA | C | 0.383 | NM_006539 | CACNG3 | frameshift_deleti  on | exon4 | c.763_764del | p.H255fs | . | . |
| CTC2 | 16 | 24788402 | GCAGCA  GCAGCA | G | 0.433 | NM_014494 | TNRC6A | nonframeshift_de  letion | exon5 | c.313_327del | p.105_109del | . | . |
| CTC2 | 16 | 24788422 | AGCAGC  CACAGC | A | 0.932 | NM_014494 | TNRC6A | nonframeshift_de  letion | exon5 | c.333_344del | p.111_115del | . | . |
| CTC2 | 16 | 24788428 | CACAGC  CGCAGC | C | 0.268 | NM_014494 | TNRC6A | nonframeshift_de  letion | exon5 | c.339_353del | p.113_118del | . | . |
| CTC2 | 16 | 27246556 | CA | C | 0.471 | NM_145080 | NSMCE1 | frameshift_deleti  on | exon3 | c.200delT | p.L67fs | . | . |
| CTC2 | 16 | 27353535 | GC | G | 1 | NM_001257406 | IL4R | frameshift_deleti  on | exon3 | c.165delC | p.S55fs | . | . |
| CTC2 | 16 | 27448933 | AT | A | 1 | NM_021798 | IL21R | frameshift_deleti  on | exon4 | c.278delT | p.I93fs | . | . |
| CTC2 | 16 | 27481583 | TTG | T | 0.569 | NM_001286242 | GTF3C1 | frameshift_deleti  on | exon31 | c.4658_4659del | p.T1553fs | . | . |
| CTC2 | 16 | 28935915 | TG | T | 0.297 | NM_024816 | RABEP2 | frameshift_deleti  on | exon2 | c.82delC | p.Q28fs | . | . |
| CTC2 | 16 | 28950251 | AG | A | 0.708 | NM_001178098 | CD19 | frameshift_deleti  on | exon14 | c.1642delG | p.G548fs | . | . |
| CTC2 | 16 | 30035346 | A | AT | 0.298 | NM_001109659 | C16orf92 | frameshift_inserti  on | exon3 | c.251dupT | p.I84fs | . | . |
| CTC2 | 16 | 30095087 | TG | T | 0.554 | NM_002720 | PPP4C | frameshift_deleti  on | exon7 | c.590delG | p.W197fs | . | . |
| CTC2 | 16 | 30106710 | GC | G | 0.262 | NM_031477 | YPEL3 | frameshift_deleti  on | exon1 | c.93delG | p.G31fs | . | . |
| CTC2 | 16 | 30393480 | A | AT | 0.25 | NM_052838 | 1-Sep | frameshift_inserti  on | exon4 | c.255dupA | p.S86fs | . | . |
| CTC2 | 16 | 30545681 | GC | G | 0.524 | NM_023931 | ZNF747 | frameshift_deleti  on | exon1 | c.319delG | p.A107fs | . | . |
| CTC2 | 16 | 30616641 | G | GT | 0.242 | NM_138447 | ZNF689 | frameshift_inserti  on | exon3 | c.446_447insA | p.P149fs | . | . |
| CTC2 | 16 | 30667368 | CTT | C | 0.308 | NM_024031 | PRR14 | frameshift_deleti  on | exon12 | c.1495_1496del | p.F499fs | . | . |
| CTC2 | 16 | 30723247 | TG | T | 0.714 | NM_006662 | SRCAP | frameshift_deleti  on | exon12 | c.1585delG | p.E529fs | . | . |
| CTC2 | 16 | 30723601 | C | CA | 0.208 | NM_006662 | SRCAP | frameshift_inserti  on | exon13 | c.1834_1835ins  A | p.L612fs | . | . |
| CTC2 | 16 | 30735570 | CT | C | 0.223 | NM_006662 | SRCAP | frameshift_deleti  on | exon25 | c.4826delT | p.L1609fs | . | . |

| CTC2 | 16 | 30750353 | A | AT | 0.196 | NM_006662 | SRCAP | frameshift_inserti  on | exon34 | c.8993dupT | p.I2998fs | . | . |
| --- | --- | --- | --- | --- | --- | --- | --- | --- | --- | --- | --- | --- | --- |
| CTC2 | 16 | 30771766 | GC | G | 0.261 | NM_001014979 | C16orf93 | frameshift_deleti  on | exon4 | c.307delG | p.A103fs | . | . |
| CTC2 | 16 | 30780190 | TG | T | 0.569 | NM_001207034 | RNF40 | frameshift_deleti  on | exon13 | c.1830delG | p.L610fs | . | . |
| CTC2 | 16 | 30794903 | AG | A | 0.417 | NM_001080417 | ZNF629 | frameshift_deleti  on | exon3 | c.745delC | p.L249fs | . | . |
| CTC2 | 16 | 30795211 | CG | C | 0.409 | NM_001080417 | ZNF629 | frameshift_deleti  on | exon3 | c.437delC | p.A146fs | . | . |
| CTC2 | 16 | 30904065 | TC | T | 0.409 | NM_001286526 | BCL7C | frameshift_deleti  on | exon4 | c.283delG | p.E95fs | . | . |
| CTC2 | 16 | 31131764 | GC | G | 0.279 | NM_032188 | KAT8 | frameshift_deleti  on | exon3 | c.392delC | p.A131fs | . | . |
| CTC2 | 16 | 31143780 | GC | G | 0.8 | NM_002773 | PRSS8 | frameshift_deleti  on | exon5 | c.674delG | p.G225fs | . | . |
| CTC2 | 16 | 31152023 | TG | T | 1 | NM_001258290 | PRSS36 | frameshift_deleti  on | exon13 | c.1941delC | p.A647fs | . | . |
| CTC2 | 16 | 31196276 | TC | T | 0.925 | NM_001170634 | FUS | frameshift_deleti  on | exon6 | c.538delC | p.Q180fs | . | . |
| CTC2 | 16 | 31502193 | GC | G | 0.75 | NM_022744 | C16orf58 | frameshift_deleti  on | exon13 | c.1369delG | p.A457fs | . | . |
| CTC2 | 16 | 31504298 | CT | C | 0.885 | NM_022744 | C16orf58 | frameshift_deleti  on | exon10 | c.1087delA | p.N363fs | . | . |
| CTC2 | 16 | 46771990 | G | GC | 0.316 | NM_182493 | MYLK3 | frameshift_inserti  on | exon3 | c.633dupG | p.L212fs | . | . |
| CTC2 | 16 | 47694652 | TG | T | 0.205 | NM_000293 | PHKB | frameshift_deleti  on | exon22 | c.2119delG | p.E707fs | . | . |
| CTC2 | 16 | 48121892 | TG | T | 0.197 | NM_033226 | ABCC12 | frameshift_deleti  on | exon25 | c.3579delC | p.L1193fs | . | . |
| CTC2 | 16 | 48145347 | CT | C | 0.333 | NM_033226 | ABCC12 | frameshift_deleti  on | exon16 | c.2280delA | p.K760fs | . | . |
| CTC2 | 16 | 48250173 | AC | A | 0.152 | NM_033151 | ABCC11 | stopgain | exon6 | c.802delG | p.V268X | . | . |
| CTC2 | 16 | 48292529 | AT | A | 0.612 | NM_031490 | LONP2 | frameshift_deleti on | exon4 | c.602delT | p.I201fs | . | . |
| CTC2 | 16 | 48595764 | CA | C | 0.313 | NM_153029 | N4BP1 | frameshift_deleti  on | exon2 | c.789delT | p.F263fs | . | . |
| CTC2 | 16 | 50368677 | CCT | C | 1 | NM_001173984 | BRD7 | frameshift_deleti  on | exon7 | c.830_831del | p.E277fs | . | . |
| CTC2 | 16 | 50368677 | CCTCT | C | 1 | NM_001173984 | BRD7 | frameshift_deleti  on | exon7 | c.828_831del | p.R276fs | . | . |
| CTC2 | 16 | 50642212 | TG | T | 1 | NM_033119 | NKD1 | frameshift_deleti  on | exon4 | c.201delG | p.V67fs | . | . |
| CTC2 | 16 | 52473512 | TTGC | T | 0.182 | NM_001080430 | TOX3 | nonframeshift_de  letion | exon7 | c.1353_1355del | p.451_452del | . | . |
| CTC2 | 16 | 52484229 | GA | G | 0.833 | NM_001080430 | TOX3 | frameshift_deleti  on | exon4 | c.637delT | p.S213fs | . | . |
| CTC2 | 16 | 53190041 | CT | C | 0.588 | NM_025134 | CHD9 | frameshift_deleti  on | exon2 | c.41delT | p.L14fs | . | . |
| CTC2 | 16 | 53190178 | TC | T | 0.174 | NM_025134 | CHD9 | frameshift_deleti  on | exon2 | c.178delC | p.P60fs | . | . |
| CTC2 | 16 | 53358078 | C | CA | 0.265 | NM_025134 | CHD9 | frameshift_inserti  on | exon39 | c.7918dupA | p.G2639fs | . | . |
| CTC2 | 16 | 53730085 | TG | T | 0.3 | NM_001127897 | RPGRIP1L | frameshift_deleti  on | exon3 | c.207delC | p.R69fs | . | . |
| CTC2 | 16 | 56692581 | TCGCAG  GTGTCTC CTGCAC CTGCGC | T | 0.438 | NM_005949 | MT1F | frameshift_deleti on | exon2 | c.29_54del | p.G10fs | . | . |
| CTC2 | 16 | 56995967 | GC | G | 0.178 | NM_000078 | CETP | frameshift_deleti  on | exon1 | c.77delC | p.A26fs | . | . |
| CTC2 | 16 | 57060706 | CTG | C | 0.2 | NM_032206 | NLRC5 | frameshift_deleti  on | exon5 | c.1852_1853del | p.C618fs | . | . |
| CTC2 | 16 | 60392815 | TG | T | 1 | NM_001282301 | LOC729159 | frameshift_deleti  on | exon1 | c.791delC | p.P264fs | . | . |
| CTC2 | 16 | 61859071 | GT | G | 0.542 | NM_001796 | CDH8 | frameshift_deleti  on | exon5 | c.679delA | p.T227fs | . | . |
| CTC2 | 16 | 66592236 | AT | A | 0.583 | NM_001040138 | CKLF | frameshift_deleti  on | exon2 | c.223delT | p.F75fs | . | . |
| CTC2 | 16 | 66943191 | CA | C | 0.17 | NM_001204746 | CDH16 | frameshift_deleti  on | exon16 | c.2079delT | p.L693fs | . | . |
| CTC2 | 16 | 67214278 | CG | C | 0.5 | NM_001040715 | KIAA0895L | frameshift_deleti  on | exon2 | c.235delC | p.R79fs | . | . |
| CTC2 | 16 | 67214367 | CA | C | 0.275 | NM_001040715 | KIAA0895L | frameshift_deleti  on | exon2 | c.146delT | p.L49fs | . | . |
| CTC2 | 16 | 67229793 | ACAG | A | 0.365 | NM_001950 | E2F4 | nonframeshift_de  letion | exon7 | c.918_920del | p.306_307del | . | . |
| CTC2 | 16 | 67300016 | TGAG | T | 0.217 | NM_004594 | SLC9A5 | nonframeshift_de  letion | exon15 | c.2107_2109del | p.703_703del | . | . |
| CTC2 | 16 | 67683465 | TG | T | 1 | NM_001013838 | RLTPR | frameshift_deleti  on | exon20 | c.1863delG | p.M621fs | . | . |
| CTC2 | 16 | 67691556 | GC | G | 0.95 | NM_001082486 | ACD | frameshift_deleti  on | exon12 | c.1587delG | p.L529fs | . | . |
| CTC2 | 16 | 67692831 | CG | C | 0.571 | NM_001082486 | ACD | frameshift_deleti  on | exon7 | c.902delC | p.T301fs | . | . |
| CTC2 | 16 | 67863854 | CT | C | 0.175 | NM_025082 | CENPT | frameshift_deleti  on | exon12 | c.999delA | p.A333fs | . | . |
| CTC2 | 16 | 68397707 | GC | G | 0.343 | NM_018667 | SMPD3 | frameshift_deleti  on | exon6 | c.1617delG | p.G539fs | . | . |
| CTC2 | 16 | 68597151 | AT | A | 0.227 | NM_133458 | ZFP90 | frameshift_deleti  on | exon4 | c.462delT | p.N154fs | . | . |
| CTC2 | 16 | 69074237 | CA | C | 0.294 | NM_024562 | TANGO6 | frameshift_deleti  on | exon17 | c.3022delA | p.K1008fs | . | . |
| CTC2 | 16 | 69143405 | T | TCC | 0.364 | NM_001199280 | HAS3 | frameshift_inserti  on | exon2 | c.107_108insCC | p.I36fs | . | . |
| CTC2 | 16 | 69967879 | GT | G | 0.792 | NM_199424 | WWP2 | frameshift_deleti  on | exon7 | c.373delT | p.F125fs | . | . |
| CTC2 | 16 | 69972981 | AC | A | 0.895 | NM_199424 | WWP2 | frameshift_deleti  on | exon12 | c.1079delC | p.T360fs | . | . |
| CTC2 | 16 | 70572300 | CT | C | 0.643 | NM_012426 | SF3B3 | frameshift_deleti  on | exon7 | c.901delT | p.F301fs | . | . |
| CTC2 | 16 | 70975639 | CA | C | 0.782 | NM_001270974 | HYDIN | frameshift_deleti  on | exon43 | c.6752delT | p.L2251fs | . | . |
| CTC2 | 16 | 71512211 | A | AG | 0.216 | NM_006961 | ZNF19 | frameshift_inserti  on | exon5 | c.193dupC | p.L65fs | . | . |
| CTC2 | 16 | 71571524 | TG | T | 0.25 | NM_001166395 | CHST4 | frameshift_deleti  on | exon2 | c.945delG | p.M315fs | . | . |
| CTC2 | 16 | 71885217 | AT | A | 0.5 | NM_001137675 | ATXN1L | frameshift_deleti  on | exon3 | c.1575delT | p.H525fs | . | . |
| CTC2 | 16 | 71885456 | CG | C | 0.409 | NM_001137675 | ATXN1L | frameshift_deleti  on | exon3 | c.1814delG | p.R605fs | . | . |
| CTC2 | 16 | 71956511 | AATGCC  C | A | 0.971 | NM_001270979 | IST1 | nonframeshift_de  letion | exon3 | c.244_249del | p.82_83del | . | . |

| CTC2 | 16 | 72828434 | CA | C | 1 | NM_001164766 | ZFHX3 | frameshift_deleti  on | exon8 | c.5404delT | p.C1802fs | . | . |
| --- | --- | --- | --- | --- | --- | --- | --- | --- | --- | --- | --- | --- | --- |
| CTC2 | 16 | 72831357 | CTTG | C | 0.15 | NM_001164766 | ZFHX3 | nonframeshift_de  letion | exon8 | c.2479_2481del | p.827_827del | . | . |
| CTC2 | 16 | 72991960 | CG | C | 1 | NM_006885 | ZFHX3 | frameshift_deleti  on | exon2 | c.2084delC | p.P695fs | . | . |
| CTC2 | 16 | 74335009 | GA | G | 0.403 | NM_002811 | PSMD7 | frameshift_deleti  on | exon3 | c.227delA | p.E76fs | . | . |
| CTC2 | 16 | 74923709 | CA | C | 0.429 | NM_030581 | WDR59 | frameshift_deleti  on | exon21 | c.2086delT | p.W696fs | . | . |
| CTC2 | 16 | 75276871 | AC | A | 0.264 | NM_001170715 | BCAR1 | frameshift_deleti  on | exon2 | c.183delG | p.W61fs | . | . |
| CTC2 | 16 | 75276954 | GC | G | 0.212 | NM_001170715 | BCAR1 | frameshift_deleti  on | exon2 | c.100delG | p.A34fs | . | . |
| CTC2 | 16 | 81077073 | TCA | T | 0.222 | NM_015251 | ATMIN | frameshift_deleti  on | exon4 | c.971_972del | p.S324fs | . | . |
| CTC2 | 16 | 81213351 | CA | C | 0.307 | NM_001278423 | PKD1L2 | frameshift_deleti  on | exon2 | c.103delT | p.W35fs | . | . |
| CTC2 | 16 | 81242148 | GTT | G | 1 | NM_001076780 | PKD1L2 | frameshift_deleti  on | exon4 | c.706_707del | p.N236fs | . | . |
| CTC2 | 16 | 81253795 | C | CT | 0.938 | NM_001076780 | PKD1L2 | frameshift_inserti  on | exon1 | c.180dupA | p.G61fs | . | . |
| CTC2 | 16 | 81385193 | AG | A | 0.571 | NM_022041 | GAN | frameshift_deleti  on | exon2 | c.174delG | p.K58fs | . | . |
| CTC2 | 16 | 81396123 | CGG | C | 0.415 | NM_022041 | GAN | frameshift_deleti  on | exon6 | c.994_995del | p.G332fs | . | . |
| CTC2 | 16 | 81733347 | GA | G | 0.979 | NM_030629 | CMIP | frameshift_deleti  on | exon15 | c.1415delA | p.E472fs | . | . |
| CTC2 | 16 | 82033702 | TC | T | 0.194 | NM_145168 | SDR42E1 | frameshift_deleti  on | exon3 | c.195delG | p.E65fs | . | . |
| CTC2 | 16 | 83842993 | AG | A | 0.364 | NM_001537 | HSBP1 | frameshift_deleti  on | exon3 | c.197delG | p.S66fs | . | . |
| CTC2 | 16 | 84012074 | AT | A | 0.303 | NM_019065 | NECAB2 | frameshift_deleti  on | exon3 | c.253delT | p.F85fs | . | . |
| CTC2 | 16 | 84066995 | GAGC | G | 0.185 | NM_001080442 | SLC38A8 | nonframeshift_de  letion | exon3 | c.465_467del | p.155_156del | . | . |
| CTC2 | 16 | 85813388 | CG | C | 0.5 | NM_006067 | EMC8 | frameshift_deleti  on | exon5 | c.558delC | p.F186fs | . | . |
| CTC2 | 16 | 85834865 | TG | T | 1 | NM_001861 | COX4I1 | frameshift_deleti  on | exon2 | c.55delG | p.V19fs | . | . |
| CTC2 | 16 | 87637859 | CG | C | 0.231 | NM_001271604 | JPH3 | frameshift_deleti  on | exon2 | c.397delG | p.G133fs | . | . |
| CTC2 | 16 | 87637917 | G | GCTA | 0.838 | NM_001271604 | JPH3 | nonframeshift_in  sertion | exon2 | c.454_455insCT  A | p.A152delinsAT | . | . |
| CTC2 | 16 | 87637920 | G | GCT | 0.5 | NM_001271604 | JPH3 | frameshift_inserti  on | exon2 | c.457_458insCT | p.A153fs | . | . |
| CTC2 | 16 | 87741961 | TC | T | 1 | NM_001184854 | KLHDC4 | frameshift_deleti  on | exon9 | c.1387delG | p.D463fs | . | . |
| CTC2 | 16 | 88502524 | TGA | T | 1 | NM_001127464 | ZNF469 | frameshift_deleti  on | exon2 | c.8563_8564del | p.E2855fs | . | . |
| CTC2 | 16 | 88504778 | GC | G | 1 | NM_001127464 | ZNF469 | frameshift_deleti  on | exon2 | c.10817delC | p.A3606fs | . | . |
| CTC2 | 16 | 88643931 | GT | G | 0.275 | NM_144604 | ZC3H18 | frameshift_deleti  on | exon2 | c.401delT | p.V134fs | . | . |
| CTC2 | 16 | 88776664 | AG | A | 1 | NM_001012759 | CTU2 | frameshift_deleti  on | exon4 | c.257delG | p.S86fs | . | . |
| CTC2 | 16 | 88805046 | G | GA | 0.875 | NM_001142864 | PIEZO1 | frameshift_inserti  on | exon6 | c.563dupT | p.F188fs | . | . |
| CTC2 | 16 | 88930705 | G | GC | 0.625 | NM_001080487 | PABPN1L | frameshift_inserti  on | exon6 | c.736dupG | p.A246fs | . | . |
| CTC2 | 16 | 90097818 | AG | A | 0.17 | NM_001286209 | GAS8 | frameshift_deleti  on | exon3 | c.128delG | p.R43fs | . | . |
| CTC2 | 17 | 649201 | CG | C | 0.688 | NM_015721 | GEMIN4 | frameshift_deleti  on | exon2 | c.2081delC | p.P694fs | . | . |
| CTC2 | 17 | 1840760 | T | TA | 0.556 | NM_178568 | RTN4RL1 | frameshift_inserti  on | exon2 | c.355_356insT | p.E119fs | . | . |
| CTC2 | 17 | 2203419 | CTA | C | 0.267 | NM_017575 | SMG6 | frameshift_deleti  on | exon2 | c.626_627del | p.V209fs | . | . |
| CTC2 | 17 | 2995642 | A | AT | 0.4 | NM_002548 | OR1D2 | frameshift_inserti  on | exon1 | c.648_649insA | p.S217fs | . | . |
| CTC2 | 17 | 3967801 | GC | G | 0.556 | NM_015113 | ZZEF1 | frameshift_deleti  on | exon29 | c.4571delG | p.R1524fs | . | . |
| CTC2 | 17 | 5009742 | T | TA | 0.541 | NM_014519 | ZNF232 | frameshift_inserti  on | exon5 | c.711dupT | p.T238fs | . | . |
| CTC2 | 17 | 5383441 | AAT | A | 0.3 | NM_016041 | DERL2 | frameshift_deleti  on | exon6 | c.545_546del | p.Y182fs | . | . |
| CTC2 | 17 | 5463327 | CCTTTCT  CTGATTT CTCTCTC | C | 0.68 | NM_001033053 | NLRP1 | frameshift_deleti on | exon4 | c.666_688del | p.R222fs | . | . |
| CTC2 | 17 | 6555546 | C | CG | 1 | NM_001105520 | C17orf100 | frameshift_inserti  on | exon1 | c.314dupG | p.R105fs | . | . |
| CTC2 | 17 | 7251526 | GC | G | 0.833 | NM_014716 | ACAP1 | frameshift_deleti  on | exon16 | c.1495delC | p.P499fs | . | . |
| CTC2 | 17 | 7324848 | A | AC | 0.833 | NM_199339 | SPEM1 | frameshift_inserti  on | exon3 | c.855dupC | p.Y285fs | . | . |
| CTC2 | 17 | 7351909 | TG | T | 0.34 | NM_000747 | CHRNB1 | frameshift_deleti  on | exon7 | c.623delG | p.W208fs | . | . |
| CTC2 | 17 | 7369236 | GC | G | 0.32 | NM_001128833 | ZBTB4 | frameshift_deleti  on | exon3 | c.884delG | p.G295fs | . | . |
| CTC2 | 17 | 7480899 | GAC | G | 0.183 | NM_001204510 | EIF4A1 | frameshift_deleti  on | exon8 | c.782_783del | p.D261fs | . | . |
| CTC2 | 17 | 7490013 | T | TG | 0.2 | NM_004870 | MPDU1 | frameshift_inserti  on | exon4 | c.306_307insG | p.S102fs | . | . |
| CTC2 | 17 | 7509034 | GA | G | 0.317 | NM_004860 | FXR2 | frameshift_deleti  on | exon3 | c.156delT | p.I52fs | . | . |
| CTC2 | 17 | 7636592 | AT | A | 1 | NM_020877 | DNAH2 | frameshift_deleti  on | exon4 | c.588delT | p.H196fs | . | . |
| CTC2 | 17 | 7680944 | TGG | T | 0.43 | NM_020877 | DNAH2 | frameshift_deleti  on | exon32 | c.5240_5241del | p.W1747fs | . | . |
| CTC2 | 17 | 7689515 | TC | T | 1 | NM_020877 | DNAH2 | frameshift_deleti  on | exon39 | c.6204delC | p.F2068fs | . | . |
| CTC2 | 17 | 7700768 | GC | G | 0.676 | NM_020877 | DNAH2 | frameshift_deleti  on | exon51 | c.8001delC | p.G2667fs | . | . |
| CTC2 | 17 | 7736028 | TG | T | 0.375 | NM_020877 | DNAH2 | frameshift_deleti  on | exon82 | c.12859delG | p.G4287fs | . | . |
| CTC2 | 17 | 7792363 | TG | T | 0.318 | NM_001005273 | CHD3 | frameshift_deleti  on | exon1 | c.46delG | p.D16fs | . | . |
| CTC2 | 17 | 7980339 | C | CA | 0.238 | NM_001139 | ALOX12B | frameshift_inserti  on | exon9 | c.1243_1244ins  T | p.R415fs | . | . |
| CTC2 | 17 | 8053442 | TG | T | 1 | NM_002616 | PER1 | frameshift_deleti  on | exon4 | c.375delC | p.S125fs | . | . |
| CTC2 | 17 | 8137860 | CT | C | 0.278 | NM_025099 | CTC1 | frameshift_deleti  on | exon10 | c.1730delA | p.E577fs | . | . |
| CTC2 | 17 | 8192145 | CT | C | 1 | NM_001177801 | RANGRF | frameshift_deleti  on | exon1 | c.40delT | p.F14fs | . | . |

| CTC2 | 17 | 8216551 | TC | T | 0.175 | NM_025014 | ARHGEF15 | frameshift_deleti  on | exon3 | c.914delC | p.S305fs | . | . |
| --- | --- | --- | --- | --- | --- | --- | --- | --- | --- | --- | --- | --- | --- |
| CTC2 | 17 | 8457287 | GC | G | 1 | NM_005964 | MYH10 | frameshift_deleti  on | exon7 | c.775delG | p.A259fs | . | . |
| CTC2 | 17 | 10408151 | AT | A | 0.924 | NM_005963 | MYH1 | frameshift_deleti  on | exon22 | c.2666delA | p.N889fs | . | . |
| CTC2 | 17 | 10451207 | CAA | C | 0.273 | NM_001100112 | MYH2 | frameshift_deleti  on | exon3 | c.29_30del | p.F10fs | . | . |
| CTC2 | 17 | 10551956 | TC | T | 0.226 | NM_002470 | MYH3 | frameshift_deleti  on | exon8 | c.652delG | p.E218fs | . | . |
| CTC2 | 17 | 11672569 | TG | T | 0.232 | NM_001372 | DNAH9 | frameshift_deleti  on | exon38 | c.7476delG | p.L2492fs | . | . |
| CTC2 | 17 | 12883414 | GA | G | 0.164 | NM_014859 | ARHGAP44 | frameshift_deleti  on | exon19 | c.1804delA | p.K602fs | . | . |
| CTC2 | 17 | 15343525 | C | CTTA | 1 | NM_001204477 | CDRT4 | stopgain | exon3 | c.27_28insTAA | p.V10delinsX | . | . |
| CTC2 | 17 | 15518996 | TG | T | 0.245 | NM_001282540 | CDRT1 | frameshift_deleti on | exon2 | c.632delC | p.P211fs | . | . |
| CTC2 | 17 | 15909788 | AC | A | 0.421 | NM_001271420 | TTC19 | frameshift_deleti  on | exon7 | c.262delC | p.Q88fs | . | . |
| CTC2 | 17 | 16005094 | TTC | T | 0.222 | NM_001190438 | NCOR1 | frameshift_deleti  on | exon17 | c.1879_1880del | p.E627fs | . | . |
| CTC2 | 17 | 17039561 | CCAG | C | 1 | NM_015134 | MPRIP | nonframeshift_de  letion | exon6 | c.534_536del | p.178_179del | . | . |
| CTC2 | 17 | 18047860 | AG | A | 0.208 | NM_016239 | MYO15A | frameshift_deleti  on | exon28 | c.6228delG | p.Q2076fs | . | . |
| CTC2 | 17 | 18141792 | TG | T | 0.238 | NM_004140 | LLGL1 | frameshift_deleti  on | exon16 | c.2076delG | p.L692fs | . | . |
| CTC2 | 17 | 18671964 | TG | T | 0.772 | NM_001267585 | FBXW10 | frameshift_deleti  on | exon10 | c.1823delG | p.C608fs | . | . |
| CTC2 | 17 | 19451390 | C | CT | 0.804 | NM_018242 | SLC47A1 | frameshift_inserti  on | exon4 | c.400dupT | p.L133fs | . | . |
| CTC2 | 17 | 19866162 | CA | C | 0.69 | NM_007202 | AKAP10 | frameshift_deleti  on | exon3 | c.309delT | p.F103fs | . | . |
| CTC2 | 17 | 21101732 | TC | T | 1 | NM_003876 | TMEM11 | frameshift_deleti  on | exon2 | c.483delG | p.R161fs | . | . |
| CTC2 | 17 | 26692224 | A | AG | 1 | NM_001080837 | SEBOX | frameshift_inserti  on | exon1 | c.27dupC | p.W10fs | . | . |
| CTC2 | 17 | 26696950 | TG | T | 0.585 | NM_000638 | VTN | frameshift_deleti  on | exon2 | c.181delC | p.Q61fs | . | . |
| CTC2 | 17 | 26861899 | AC | A | 0.667 | NM_003593 | FOXN1 | frameshift_deleti  on | exon7 | c.1311delC | p.N437fs | . | . |
| CTC2 | 17 | 26883854 | AC | A | 0.302 | NM_033198 | PIGS | frameshift_deleti  on | exon9 | c.1070delG | p.G357fs | . | . |
| CTC2 | 17 | 26890919 | AT | A | 0.295 | NM_033198 | PIGS | stopgain | exon4 | c.292delA | p.M98X | . | . |
| CTC2 | 17 | 27014488 | AG | A | 0.778 | NM_003170 | SUPT6H | frameshift_deleti on | exon23 | c.3006delG | p.K1002fs | . | . |
| CTC2 | 17 | 27049867 | TG | T | 1 | NM_000984 | RPL23A | stopgain | exon3 | c.337delG | p.V113X | . | . |
| CTC2 | 17 | 27065254 | TG | T | 0.389 | NM_178170 | NEK8 | frameshift_deleti on | exon8 | c.1214delG | p.C405fs | . | . |
| CTC2 | 17 | 27066171 | GC | G | 0.889 | NM_178170 | NEK8 | frameshift_deleti  on | exon10 | c.1370delC | p.A457fs | . | . |
| CTC2 | 17 | 27182256 | TG | T | 1 | NM_005702 | ERAL1 | frameshift_deleti  on | exon1 | c.205delG | p.A69fs | . | . |
| CTC2 | 17 | 27445174 | TA | T | 0.417 | NM_078471 | MYO18A | frameshift_deleti  on | exon10 | c.1926delT | p.F642fs | . | . |
| CTC2 | 17 | 27943979 | TG | T | 0.353 | NM_032854 | CORO6 | frameshift_deleti  on | exon6 | c.834delC | p.S278fs | . | . |
| CTC2 | 17 | 28386585 | TC | T | 0.594 | NM_198529 | EFCAB5 | frameshift_deleti  on | exon14 | c.2604delC | p.V868fs | . | . |
| CTC2 | 17 | 28712201 | ACG | A | 0.222 | NM_001199775 | CPD | frameshift_deleti  on | exon2 | c.201_202del | p.D67fs | . | . |
| CTC2 | 17 | 28749877 | CA | C | 0.607 | NM_001199775 | CPD | frameshift_deleti  on | exon5 | c.753delA | p.P251fs | . | . |
| CTC2 | 17 | 28808187 | GA | G | 1 | NM_001007025 | GOSR1 | frameshift_deleti  on | exon2 | c.59delA | p.E20fs | . | . |
| CTC2 | 17 | 29162787 | GA | G | 0.667 | NM_024857 | ATAD5 | frameshift_deleti  on | exon2 | c.1689delA | p.R563fs | . | . |
| CTC2 | 17 | 29560167 | TG | T | 0.375 | NM_000267 | NF1 | frameshift_deleti  on | exon27 | c.3645delG | p.M1215fs | . | . |
| CTC2 | 17 | 31341027 | GC | G | 0.356 | NM_001094 | ASIC2 | frameshift_deleti  on | exon10 | c.1494delG | p.V498fs | . | . |
| CTC2 | 17 | 33316508 | GC | G | 0.402 | NM_002311 | LIG3 | frameshift_deleti  on | exon4 | c.716delC | p.A239fs | . | . |
| CTC2 | 17 | 33430316 | CG | C | 0.268 | NM_133629 | RAD51D | frameshift_deleti  on | exon5 | c.358delC | p.R120fs | . | . |
| CTC2 | 17 | 33460248 | CA | C | 0.257 | NM_001014445 | NLE1 | frameshift_deleti  on | exon11 | c.510delT | p.V170fs | . | . |
| CTC2 | 17 | 33460491 | AC | A | 0.812 | NM_001014445 | NLE1 | frameshift_deleti  on | exon10 | c.364delG | p.V122fs | . | . |
| CTC2 | 17 | 33504051 | GC | G | 0.278 | NM_001033576 | UNC45B | frameshift_deleti  on | exon16 | c.2042delC | p.A681fs | . | . |
| CTC2 | 17 | 34252551 | CA | C | 0.984 | NM_001163122 | RDM1 | frameshift_deleti  on | exon2 | c.297delT | p.F99fs | . | . |
| CTC2 | 17 | 34853517 | CT | C | 0.4 | NM_001281432 | ZNHIT3 | frameshift_deleti  on | exon5 | c.355delT | p.S119fs | . | . |
| CTC2 | 17 | 34854294 | TC | T | 0.217 | NM_025109 | MYO19 | frameshift_deleti  on | exon22 | c.1972delG | p.E658fs | . | . |
| CTC2 | 17 | 35564609 | GC | G | 0.275 | NM_198837 | ACACA | frameshift_deleti  on | exon29 | c.3527delG | p.G1176fs | . | . |
| CTC2 | 17 | 35880695 | AT | A | 0.8 | NM_001163547 | SYNRG | frameshift_deleti  on | exon18 | c.3104delA | p.N1035fs | . | . |
| CTC2 | 17 | 35937648 | A | AT | 0.238 | NM_007247 | SYNRG | frameshift_inserti  on | exon7 | c.652dupA | p.I218fs | . | . |
| CTC2 | 17 | 36485932 | TG | T | 0.714 | NM_001004334 | GPR179 | frameshift_deleti  on | exon11 | c.3519delC | p.S1173fs | . | . |
| CTC2 | 17 | 36622493 | AC | A | 0.952 | NM_001199417 | ARHGAP23 | frameshift_deleti  on | exon7 | c.570delC | p.Y190fs | . | . |
| CTC2 | 17 | 36874191 | GA | G | 0.308 | NM_005937 | MLLT6 | frameshift_deleti  on | exon12 | c.1928delA | p.E643fs | . | . |
| CTC2 | 17 | 36997605 | TTC | T | 0.296 | NM_001080465 | C17orf98 | frameshift_deleti  on | exon1 | c.36_37del | p.E12fs | . | . |
| CTC2 | 17 | 37676323 | CA | C | 0.528 | NM_015083 | CDK12 | frameshift_deleti  on | exon11 | c.3079delA | p.K1027fs | . | . |
| CTC2 | 17 | 37880262 | AC | A | 0.538 | NM_001289937 | ERBB2 | frameshift_deleti  on | exon19 | c.2307delC | p.D769fs | . | . |
| CTC2 | 17 | 37922262 | GTC | G | 0.179 | NM_001284516 | IKZF3 | frameshift_deleti  on | exon3 | c.568_569del | p.D190fs | . | . |
| CTC2 | 17 | 38151278 | C | CA | 0.579 | NM_002809 | PSMD3 | frameshift_inserti  on | exon7 | c.1053_1054ins  A | p.P351fs | . | . |
| CTC2 | 17 | 38452964 | TG | T | 0.333 | NM_001254 | CDC6 | frameshift_deleti  on | exon9 | c.1195delG | p.E399fs | . | . |
| CTC2 | 17 | 38978765 | ATCC | A | 0.179 | NM_000421 | KRT10 | nonframeshift_de  letion | exon1 | c.70_72del | p.24_24del | . | . |

| CTC2 | 17 | 39305775 | T | TGGCA  GCAGC | 0.87 | NM_033188 | KRTAP4-5 | nonframeshift_in  sertion | exon1 | c.244_245insG  CCCCAGCTGC | p.Q82delinsRP  SCCQ | . | . |
| --- | --- | --- | --- | --- | --- | --- | --- | --- | --- | --- | --- | --- | --- |
| CTC2 | 17 | 39324229 | T | TGCAG  CAGGT | 0.5 | NM_033187 | KRTAP4-3 | nonframeshift_in  sertion | exon1 | c.195_196insCT  GACCACCTGC | p.R66delinsLTT  CCR | . | . |
| CTC2 | 17 | 39411870 | CA | C | 0.333 | NM_030975 | KRTAP9-9 | frameshift_deleti  on | exon1 | c.234delA | p.T78fs | . | . |
| CTC2 | 17 | 39464759 | AC | A | 0.382 | NM_001146182 | KRTAP16-1 | frameshift_deleti  on | exon1 | c.746delG | p.C249fs | . | . |
| CTC2 | 17 | 39723636 | AC | A | 0.509 | NM_000226 | KRT9 | frameshift_deleti  on | exon7 | c.1760delG | p.G587fs | . | . |
| CTC2 | 17 | 40179053 | TG | T | 0.246 | NM_001242704 | ZNF385C | frameshift_deleti  on | exon8 | c.1136delC | p.P379fs | . | . |
| CTC2 | 17 | 40179638 | TG | T | 0.193 | NM_001242704 | ZNF385C | frameshift_deleti  on | exon7 | c.1035delC | p.L345fs | . | . |
| CTC2 | 17 | 40183752 | C | CT | 0.152 | NM_001242704 | ZNF385C | frameshift_inserti  on | exon4 | c.323dupA | p.K108fs | . | . |
| CTC2 | 17 | 40476747 | GT | G | 0.3 | NM_003150 | STAT3 | frameshift_deleti  on | exon17 | c.1581delA | p.T527fs | . | . |
| CTC2 | 17 | 40818395 | TG | T | 0.357 | NM_016437 | TUBG2 | frameshift_deleti  on | exon10 | c.1052delG | p.W351fs | . | . |
| CTC2 | 17 | 40823434 | TA | T | 0.974 | NM_024927 | PLEKHH3 | stopgain | exon8 | c.1218delT | p.C406X | . | . |
| CTC2 | 17 | 40849711 | TG | T | 0.2 | NM_003632 | CNTNAP1 | frameshift_deleti on | exon22 | c.3709delG | p.E1237fs | . | . |
| CTC2 | 17 | 41004373 | CCT | C | 0.25 | NM_001277731 | AOC3 | frameshift_deleti  on | exon1 | c.1014_1015del | p.S338fs | . | . |
| CTC2 | 17 | 41131414 | TAG | T | 1 | NM_001142654 | PTGES3L | frameshift_deleti  on | exon2 | c.183_184del | p.F61fs | . | . |
| CTC2 | 17 | 41243738 | GC | G | 0.262 | NM_007297 | BRCA1 | frameshift_deleti  on | exon9 | c.3668delG | p.C1223fs | . | . |
| CTC2 | 17 | 41246031 | CT | C | 0.333 | NM_007297 | BRCA1 | frameshift_deleti  on | exon9 | c.1375delA | p.R459fs | . | . |
| CTC2 | 17 | 41352571 | TG | T | 0.922 | NM_005899 | NBR1 | frameshift_deleti  on | exon17 | c.2415delG | p.L805fs | . | . |
| CTC2 | 17 | 41477323 | TG | T | 0.199 | NM_001661 | ARL4D | frameshift_deleti  on | exon2 | c.224delG | p.W75fs | . | . |
| CTC2 | 17 | 41570823 | GA | G | 0.163 | NM_004941 | DHX8 | frameshift_deleti  on | exon7 | c.875delA | p.E292fs | . | . |
| CTC2 | 17 | 41598215 | C | CT | 0.194 | NM_004941 | DHX8 | frameshift_inserti  on | exon20 | c.3035dupT | p.L1012fs | . | . |
| CTC2 | 17 | 42170844 | CCTG | C | 0.305 | NM_001015053 | HDAC5 | nonframeshift_de  letion | exon5 | c.364_366del | p.122_122del | . | . |
| CTC2 | 17 | 42171031 | AGCT | A | 0.149 | NM_001015053 | HDAC5 | nonframeshift_de  letion | exon4 | c.266_268del | p.89_90del | . | . |
| CTC2 | 17 | 42478488 | GA | G | 0.333 | NM_001002909 | GPATCH8 | frameshift_deleti  on | exon8 | c.956delT | p.F319fs | . | . |
| CTC2 | 17 | 42744494 | GT | G | 0.256 | NM_001145080 | C17orf104 | frameshift_deleti  on | exon5 | c.1216delT | p.F406fs | . | . |
| CTC2 | 17 | 43515302 | G | GC | 1 | NM_014798 | PLEKHM1 | frameshift_inserti  on | exon12 | c.3092dupG | p.S1031fs | . | . |
| CTC2 | 17 | 43516844 | TG | T | 0.619 | NM_014798 | PLEKHM1 | frameshift_deleti  on | exon11 | c.3057delC | p.V1019fs | . | . |
| CTC2 | 17 | 43516961 | TC | T | 0.444 | NM_014798 | PLEKHM1 | frameshift_deleti  on | exon11 | c.2940delG | p.L980fs | . | . |
| CTC2 | 17 | 44060964 | TC | T | 0.986 | NM_001123066 | MAPT | frameshift_deleti  on | exon6 | c.795delC | p.I265fs | . | . |
| CTC2 | 17 | 44076769 | GT | G | 0.5 | NM_001007532 | STH | frameshift_deleti  on | exon1 | c.125delT | p.V42fs | . | . |
| CTC2 | 17 | 45695761 | AG | A | 0.957 | NM_006310 | NPEPPS | frameshift_deleti  on | exon20 | c.2342delG | p.R781fs | . | . |
| CTC2 | 17 | 46135772 | TG | T | 0.186 | NM_003204 | NFE2L1 | frameshift_deleti  on | exon6 | c.1089delG | p.L363fs | . | . |
| CTC2 | 17 | 46136093 | AG | A | 0.207 | NM_003204 | NFE2L1 | frameshift_deleti  on | exon6 | c.1410delG | p.E470fs | . | . |
| CTC2 | 17 | 46801116 | CA | C | 0.737 | NM_001282276 | PRAC2 | frameshift_deleti  on | exon1 | c.51delA | p.S17fs | . | . |
| CTC2 | 17 | 48275356 | TG | T | 0.237 | NM_000088 | COL1A1 | frameshift_deleti  on | exon8 | c.595delC | p.Q199fs | . | . |
| CTC2 | 17 | 48435869 | TG | T | 0.348 | NM_022167 | XYLT2 | frameshift_deleti  on | exon10 | c.2244delG | p.L748fs | . | . |
| CTC2 | 17 | 48646639 | CT | C | 0.497 | NM_001256324 | CACNA1G | frameshift_deleti  on | exon3 | c.469delT | p.F157fs | . | . |
| CTC2 | 17 | 48684279 | GT | G | 0.397 | NM_001256332 | CACNA1G | frameshift_deleti  on | exon23 | c.4373delT | p.V1458fs | . | . |
| CTC2 | 17 | 48685391 | GC | G | 0.4 | NM_198376 | CACNA1G | frameshift_deleti  on | exon24 | c.4648delC | p.Q1550fs | . | . |
| CTC2 | 17 | 48776958 | AG | A | 0.5 | NM_052855 | ANKRD40 | frameshift_deleti  on | exon3 | c.579delC | p.P193fs | . | . |
| CTC2 | 17 | 48940989 | GT | G | 0.404 | NM_005749 | TOB1 | frameshift_deleti  on | exon2 | c.389delA | p.N130fs | . | . |
| CTC2 | 17 | 49302412 | GT | G | 0.419 | NM_017643 | MBTD1 | frameshift_deleti  on | exon3 | c.110delA | p.N37fs | . | . |
| CTC2 | 17 | 54940098 | AGAT | A | 0.119 | NM_003647 | DGKE | nonframeshift_de  letion | exon12 | c.1651_1653del | p.551_551del | . | . |
| CTC2 | 17 | 56277007 | C | CA | 0.543 | NM_000502 | EPX | frameshift_inserti  on | exon9 | c.1390dupA | p.A463fs | . | . |
| CTC2 | 17 | 56345202 | GA | G | 0.426 | NM_001160102 | LPO | frameshift_deleti  on | exon11 | c.1738delA | p.K580fs | . | . |
| CTC2 | 17 | 56382929 | TG | T | 0.847 | NM_024418 | BZRAP1 | frameshift_deleti  on | exon27 | c.5152delC | p.Q1718fs | . | . |
| CTC2 | 17 | 56584107 | CA | C | 0.304 | NM_004687 | MTMR4 | frameshift_deleti  on | exon10 | c.987delT | p.C329fs | . | . |
| CTC2 | 17 | 57134313 | GC | G | 0.5 | NM_001005207 | TRIM37 | frameshift_deleti  on | exon13 | c.1121delG | p.G374fs | . | . |
| CTC2 | 17 | 57643120 | TC | T | 0.25 | NM_001166301 | DHX40 | frameshift_deleti  on | exon1 | c.89delC | p.S30fs | . | . |
| CTC2 | 17 | 58372071 | TG | T | 0.317 | NM_032582 | USP32 | frameshift_deleti  on | exon4 | c.383delC | p.P128fs | . | . |
| CTC2 | 17 | 58946006 | AT | A | 0.2 | NM_001099432 | BCAS3 | frameshift_deleti  on | exon8 | c.547delT | p.F183fs | . | . |
| CTC2 | 17 | 59155847 | CT | C | 0.412 | NM_017679 | BCAS3 | frameshift_deleti  on | exon21 | c.2285delT | p.L762fs | . | . |
| CTC2 | 17 | 59560607 | C | CA | 0.545 | NM_018488 | TBX4 | frameshift_inserti  on | exon8 | c.1369dupA | p.P456fs | . | . |
| CTC2 | 17 | 59945020 | AT | A | 0.326 | NM_020748 | INTS2 | frameshift_deleti  on | exon25 | c.3512delA | p.N1171fs | . | . |
| CTC2 | 17 | 60472525 | GA | G | 0.308 | NM_173503 | EFCAB3 | frameshift_deleti  on | exon6 | c.465delA | p.R155fs | . | . |
| CTC2 | 17 | 60650664 | GC | G | 0.431 | NM_001284363 | TLK2 | frameshift_deleti  on | exon10 | c.962delC | p.A321fs | . | . |
| CTC2 | 17 | 61570844 | AC | A | 0.6 | NM_001178057 | ACE | frameshift_deleti  on | exon9 | c.1239delC | p.H413fs | . | . |
| CTC2 | 17 | 61681958 | AC | A | 1 | NM_016360 | TACO1 | frameshift_deleti  on | exon2 | c.346delC | p.H116fs | . | . |

| CTC2 | 17 | 61911584 | GT | G | 0.621 | NM_001098426 | SMARCD2 | frameshift_deleti  on | exon8 | c.1025delA | p.N342fs | . | . |
| --- | --- | --- | --- | --- | --- | --- | --- | --- | --- | --- | --- | --- | --- |
| CTC2 | 17 | 61949606 | GT | G | 0.42 | NM_022645 | CSH2 | frameshift_deleti  on | exon3 | c.248delA | p.N83fs | . | . |
| CTC2 | 17 | 61949661 | CG | C | 0.275 | NM_022645 | CSH2 | frameshift_deleti  on | exon3 | c.193delC | p.R65fs | . | . |
| CTC2 | 17 | 62521994 | GA | G | 0.524 | NM_138363 | CEP95 | frameshift_deleti  on | exon9 | c.1017delA | p.G339fs | . | . |
| CTC2 | 17 | 62530757 | GA | G | 0.254 | NM_138363 | CEP95 | frameshift_deleti  on | exon17 | c.1973delA | p.E658fs | . | . |
| CTC2 | 17 | 62533186 | C | CA | 0.24 | NM_138363 | CEP95 | frameshift_inserti  on | exon19 | c.2246dupA | p.Q749fs | . | . |
| CTC2 | 17 | 65338373 | TA | T | 0.417 | NM_174871 | PSMD12 | frameshift_deleti  on | exon9 | c.1029delT | p.I343fs | . | . |
| CTC2 | 17 | 65907377 | CA | C | 0.192 | NM_182641 | BPTF | frameshift_deleti  on | exon11 | c.3378delA | p.P1126fs | . | . |
| CTC2 | 17 | 65955758 | T | TCCTCC  AGCC | 0.938 | NM_182641 | BPTF | nonframeshift_in  sertion | exon24 | c.8028_8029ins  CCTCCAGCC | p.A2676delinsA  PPA | . | . |
| CTC2 | 17 | 65955758 | T | TCCTCC  AGC | 0.938 | NM_182641 | BPTF | frameshift_inserti  on | exon24 | c.8028_8029ins  CCTCCAGC | p.A2676fs | . | . |
| CTC2 | 17 | 65955761 | T | TCCAG  CCCCC | 0.375 | NM_182641 | BPTF | nonframeshift_in  sertion | exon24 | c.8031_8032ins  CCAGCCCCC | p.P2677delinsP  PAP | . | . |
| CTC2 | 17 | 65955778 | CT | C | 0.333 | NM_182641 | BPTF | frameshift_deleti  on | exon24 | c.8049delT | p.P2683fs | . | . |
| CTC2 | 17 | 66253102 | CA | C | 0.742 | NM_001033574 | AMZ2 | frameshift_deleti  on | exon6 | c.902delA | p.Q301fs | . | . |
| CTC2 | 17 | 66899455 | CT | C | 0.455 | NM_007168 | ABCA8 | frameshift_deleti  on | exon18 | c.2463delA | p.K821fs | . | . |
| CTC2 | 17 | 67004215 | GT | G | 0.4 | NM_080283 | ABCA9 | frameshift_deleti  on | exon24 | c.3308delA | p.N1103fs | . | . |
| CTC2 | 17 | 67501943 | TA | T | 0.476 | NM_002758 | MAP2K6 | frameshift_deleti  on | exon2 | c.40delA | p.K14fs | . | . |
| CTC2 | 17 | 68171646 | AT | A | 0.445 | NM_000891 | KCNJ2 | frameshift_deleti  on | exon2 | c.467delT | p.I156fs | . | . |
| CTC2 | 17 | 70119005 | ACG | A | 0.965 | NM_000346 | SOX9 | frameshift_deleti  on | exon2 | c.578_579del | p.T193fs | . | . |
| CTC2 | 17 | 70645311 | G | GA | 0.357 | NM_001159770 | SLC39A11 | frameshift_inserti  on | exon8 | c.788dupT | p.F263fs | . | . |
| CTC2 | 17 | 71248793 | TC | T | 0.299 | NM_001129885 | CPSF4L | frameshift_deleti  on | exon5 | c.472delG | p.E158fs | . | . |
| CTC2 | 17 | 71397167 | AC | A | 0.306 | NM_001144952 | SDK2 | frameshift_deleti  on | exon21 | c.2962delG | p.V988fs | . | . |
| CTC2 | 17 | 71431717 | C | CG | 0.688 | NM_001144952 | SDK2 | frameshift_inserti  on | exon9 | c.1066dupC | p.R356fs | . | . |
| CTC2 | 17 | 72943181 | AC | A | 0.571 | NM_001272005 | OTOP3 | frameshift_deleti  on | exon6 | c.1178delC | p.T393fs | . | . |
| CTC2 | 17 | 73237022 | T | TA | 0.25 | NM_001172704 | GGA3 | frameshift_inserti  on | exon10 | c.696_697insT | p.I233fs | . | . |
| CTC2 | 17 | 73317772 | TCTG | T | 1 | NM_203506 | GRB2 | nonframeshift_de  letion | exon4 | c.310_312del | p.104_104del | . | . |
| CTC2 | 17 | 73518175 | GC | G | 0.385 | NM_207346 | TSEN54 | frameshift_deleti  on | exon8 | c.1014delC | p.C338fs | . | . |
| CTC2 | 17 | 73662539 | TA | T | 0.333 | NM_001003715 | RECQL5 | frameshift_deleti  on | exon2 | c.98delT | p.L33fs | . | . |
| CTC2 | 17 | 73758946 | AC | A | 1 | NM_000154 | GALK1 | frameshift_deleti  on | exon5 | c.631delG | p.V211fs | . | . |
| CTC2 | 17 | 73774935 | TG | T | 0.233 | NM_005324 | H3F3B | frameshift_deleti  on | exon3 | c.237delC | p.F79fs | . | . |
| CTC2 | 17 | 74097884 | CA | C | 0.6 | NM_001282313 | EXOC7 | frameshift_deleti  on | exon2 | c.63delT | p.P21fs | . | . |
| CTC2 | 17 | 74288392 | CACCAG  GTTGCA CCAAAC CACGCT | C | 0.7 | NM_032134 | QRICH2 | nonframeshift_de letion | exon4 | c.1888_1917del | p.630_639del | . | . |
| CTC2 | 17 | 74288564 | CTGA | C | 0.974 | NM_032134 | QRICH2 | nonframeshift_de  letion | exon4 | c.1743_1745del | p.581_582del | . | . |
| CTC2 | 17 | 74288656 | TCTGATC  TGCACC AGATTG TACCAA | T | 0.889 | NM_032134 | QRICH2 | nonframeshift_de letion | exon4 | c.1624_1653del | p.542_551del | . | . |
| CTC2 | 17 | 74570619 | GC | G | 0.419 | NM_006456 | ST6GALNAC  2 | frameshift_deleti  on | exon3 | c.188delG | p.G63fs | . | . |
| CTC2 | 17 | 74928755 | C | CT | 0.343 | NM_198955 | MGAT5B | frameshift_inserti  on | exon10 | c.1354dupT | p.G451fs | . | . |
| CTC2 | 17 | 76045607 | TG | T | 0.14 | NM_001142640 | TNRC6C | frameshift_deleti  on | exon4 | c.465delG | p.L155fs | . | . |
| CTC2 | 17 | 76047420 | TACC | T | 0.123 | NM_001142640 | TNRC6C | nonframeshift_de  letion | exon4 | c.2278_2280del | p.760_760del | . | . |
| CTC2 | 17 | 76440760 | CCA | C | 0.286 | NM_173628 | DNAH17 | frameshift_deleti  on | exon71 | c.11452_11453  del | p.W3818fs | . | . |
| CTC2 | 17 | 76455901 | CT | C | 0.821 | NM_173628 | DNAH17 | frameshift_deleti  on | exon60 | c.9617delA | p.K3206fs | . | . |
| CTC2 | 17 | 76694917 | CG | C | 0.273 | NM_004762 | CYTH1 | frameshift_deleti  on | exon8 | c.683delC | p.P228fs | . | . |
| CTC2 | 17 | 76888142 | AT | A | 0.375 | NM_001243540 | LOC1006535  15 | frameshift_deleti  on | exon3 | c.443delA | p.N148fs | . | . |
| CTC2 | 17 | 77093419 | GTAT | G | 0.138 | NM_001082575 | RBFOX3 | nonframeshift_de  letion | exon11 | c.707_709del | p.236_237del | . | . |
| CTC2 | 17 | 77758130 | AG | A | 0.263 | NM_005189 | CBX2 | frameshift_deleti  on | exon5 | c.889delG | p.G297fs | . | . |
| CTC2 | 17 | 78326784 | CT | C | 0.25 | NM_001256071 | RNF213 | frameshift_deleti  on | exon33 | c.10349delT | p.L3450fs | . | . |
| CTC2 | 17 | 78389617 | TC | T | 0.56 | NM_001164637 | ENDOV | frameshift_deleti  on | exon2 | c.225delC | p.L75fs | . | . |
| CTC2 | 17 | 78444743 | TC | T | 0.316 | NM_002522 | NPTX1 | frameshift_deleti  on | exon5 | c.1168delG | p.E390fs | . | . |
| CTC2 | 17 | 79211190 | C | CA | 0.316 | NM_144679 | ENTHD2 | frameshift_inserti  on | exon2 | c.117dupT | p.A40fs | . | . |
| CTC2 | 17 | 79660698 | GGACGA | G | 0.397 | NM_004712 | HGS | frameshift_deleti  on | exon10 | c.757_761del | p.D253fs | . | . |
| CTC2 | 17 | 79661880 | TG | T | 0.148 | NM_004712 | HGS | frameshift_deleti  on | exon12 | c.973delG | p.E325fs | . | . |
| CTC2 | 17 | 79977255 | CT | C | 0.583 | NM_001271007 | STRA13 | frameshift_deleti  on | exon3 | c.90delA | p.E30fs | . | . |
| CTC2 | 17 | 80141650 | CT | C | 0.215 | NM_198082 | CCDC57 | frameshift_deleti  on | exon8 | c.1210delA | p.R404fs | . | . |
| CTC2 | 17 | 80788958 | AC | A | 0.206 | NM_024702 | ZNF750 | frameshift_deleti  on | exon2 | c.1372delG | p.V458fs | . | . |
| CTC2 | 17 | 80861324 | CA | C | 0.636 | NM_005993 | TBCD | frameshift_deleti  on | exon19 | c.1775delA | p.Q592fs | . | . |
| CTC2 | 18 | 3129354 | GT | G | 0.826 | NM_003803 | MYOM1 | frameshift_deleti  on | exon18 | c.2669delA | p.N890fs | . | . |
| CTC2 | 18 | 3174177 | CG | C | 0.257 | NM_003803 | MYOM1 | frameshift_deleti  on | exon7 | c.1051delC | p.R351fs | . | . |

| CTC2 | 18 | 7014026 | AC | A | 0.273 | NM_005559 | LAMA1 | frameshift_deleti  on | exon23 | c.3150delG | p.G1050fs | . | . |
| --- | --- | --- | --- | --- | --- | --- | --- | --- | --- | --- | --- | --- | --- |
| CTC2 | 18 | 9256399 | TA | T | 0.875 | NM_001083625 | ANKRD12 | frameshift_deleti  on | exon8 | c.3066delA | p.L1022fs | . | . |
| CTC2 | 18 | 11881014 | CA | C | 0.583 | NM_001261444 | GNAL | frameshift_deleti  on | exon8 | c.406delA | p.K136fs | . | . |
| CTC2 | 18 | 11885731 | CG | C | 1 | NM_001242904 | MPPE1 | frameshift_deleti  on | exon7 | c.762delC | p.P254fs | . | . |
| CTC2 | 18 | 12125957 | TC | T | 1 | NM_001277333 | ANKRD62 | frameshift_deleti  on | exon13 | c.2138delC | p.S713fs | . | . |
| CTC2 | 18 | 12351393 | AT | A | 0.429 | NM_006796 | AFG3L2 | frameshift_deleti  on | exon11 | c.1337delA | p.N446fs | . | . |
| CTC2 | 18 | 13884962 | GC | G | 0.412 | NM_000529 | MC2R | frameshift_deleti  on | exon2 | c.555delG | p.M185fs | . | . |
| CTC2 | 18 | 13885154 | AC | A | 0.533 | NM_000529 | MC2R | frameshift_deleti  on | exon2 | c.363delG | p.L121fs | . | . |
| CTC2 | 18 | 19079786 | TG | T | 0.393 | NM_001142966 | GREB1L | frameshift_deleti  on | exon22 | c.3489delG | p.L1163fs | . | . |
| CTC2 | 18 | 19100758 | A | ATCT | 1 | NM_001142966 | GREB1L | nonframeshift_in  sertion | exon32 | c.5582_5583ins  TCT | p.D1861delins  DL | . | . |
| CTC2 | 18 | 19359545 | A | AT | 0.228 | NM_020774 | MIB1 | frameshift_inserti  on | exon6 | c.807_808insT | p.G269fs | . | . |
| CTC2 | 18 | 20577596 | CA | C | 0.303 | NM_002894 | RBBP8 | frameshift_deleti  on | exon14 | c.2043delA | p.S681fs | . | . |
| CTC2 | 18 | 21044488 | C | CT | 0.182 | NM_003831 | RIOK3 | frameshift_inserti  on | exon5 | c.439_440insT | p.P147fs | . | . |
| CTC2 | 18 | 21112198 | C | CT | 0.522 | NM_000271 | NPC1 | frameshift_inserti  on | exon25 | c.3804dupA | p.G1269fs | . | . |
| CTC2 | 18 | 21478791 | AT | A | 0.24 | NM_000227 | LAMA3 | frameshift_deleti  on | exon8 | c.877delT | p.F293fs | . | . |
| CTC2 | 18 | 29044107 | CT | C | 0.65 | NM_001944 | DSG3 | frameshift_deleti  on | exon9 | c.1034delT | p.L345fs | . | . |
| CTC2 | 18 | 32825510 | GA | G | 0.565 | NM_001135178 | ZNF397 | frameshift_deleti  on | exon4 | c.842delA | p.E281fs | . | . |
| CTC2 | 18 | 32825950 | TA | T | 0.571 | NM_001135178 | ZNF397 | frameshift_deleti  on | exon4 | c.1282delA | p.N428fs | . | . |
| CTC2 | 18 | 39618786 | TA | T | 0.352 | NM_002647 | PIK3C3 | frameshift_deleti  on | exon18 | c.2011delA | p.K671fs | . | . |
| CTC2 | 18 | 42456670 | C | CTCTT | 1 | NM_001130110 | SETBP1 | frameshift_inserti  on | exon4 | c.681_682insTC  TT | p.L227fs | . | . |
| CTC2 | 18 | 42532080 | C | CG | 0.25 | NM_015559 | SETBP1 | frameshift_inserti  on | exon4 | c.2775_2776ins  G | p.D925fs | . | . |
| CTC2 | 18 | 43329860 | GA | G | 0.242 | NM_001146037 | SLC14A1 | frameshift_deleti  on | exon9 | c.1283delA | p.E428fs | . | . |
| CTC2 | 18 | 43833701 | T | TCTG | 0.933 | NM_001008239 | C18orf25 | nonframeshift_in  sertion | exon3 | c.754_755insCT  G | p.S252delinsSA | . | . |
| CTC2 | 18 | 43833702 | C | CTGA | 0.904 | NM_001008239 | C18orf25 | nonframeshift_in  sertion | exon3 | c.755_756insTG  A | p.S252delinsSD | . | . |
| CTC2 | 18 | 46284771 | GA | G | 1 | NM_014772 | CTIF | frameshift_deleti  on | exon8 | c.1067delA | p.E356fs | . | . |
| CTC2 | 18 | 47390650 | CCGTGG  CTGGAG TTATTCT | C | 0.125 | NM_001080467 | MYO5B | frameshift_deleti on | exon28 | c.3685_3703del | p.Q1229fs | . | . |
| CTC2 | 18 | 47405425 | T | TGAG | 1 | NM_001080467 | MYO5B | nonframeshift_in  sertion | exon24 | c.3165_3166ins  CTC | p.M1056delins  LM | . | . |
| CTC2 | 18 | 48584772 | CA | C | 0.309 | NM_005359 | SMAD4 | frameshift_deleti  on | exon7 | c.851delA | p.Q284fs | . | . |
| CTC2 | 18 | 50866123 | GC | G | 0.357 | NM_005215 | DCC | frameshift_deleti  on | exon15 | c.2206delC | p.P736fs | . | . |
| CTC2 | 18 | 50866218 | CT | C | 0.333 | NM_005215 | DCC | frameshift_deleti  on | exon15 | c.2301delT | p.P767fs | . | . |
| CTC2 | 18 | 55362501 | CA | C | 0.875 | NM_005603 | ATP8B1 | frameshift_deleti  on | exon10 | c.841delT | p.W281fs | . | . |
| CTC2 | 18 | 56203281 | GT | G | 1 | NM_052947 | ALPK2 | frameshift_deleti  on | exon5 | c.4137delA | p.K1379fs | . | . |
| CTC2 | 18 | 56247323 | TG | T | 0.667 | NM_052947 | ALPK2 | frameshift_deleti  on | exon4 | c.684delC | p.D228fs | . | . |
| CTC2 | 18 | 56586629 | TG | T | 0.262 | NM_018181 | ZNF532 | frameshift_deleti  on | exon4 | c.1111delG | p.G371fs | . | . |
| CTC2 | 18 | 57122083 | CT | C | 0.265 | NM_133459 | CCBE1 | frameshift_deleti  on | exon6 | c.653delA | p.K218fs | . | . |
| CTC2 | 18 | 61151722 | GA | G | 0.818 | NM_002639 | SERPINB5 | frameshift_deleti  on | exon2 | c.62delA | p.E21fs | . | . |
| CTC2 | 18 | 61562569 | GT | G | 0.189 | NM_002575 | SERPINB2 | frameshift_deleti  on | exon3 | c.241delT | p.F81fs | . | . |
| CTC2 | 18 | 61570321 | TC | T | 0.4 | NM_002575 | SERPINB2 | frameshift_deleti  on | exon8 | c.1031delC | p.S344fs | . | . |
| CTC2 | 18 | 61600349 | CA | C | 0.917 | NM_005024 | SERPINB10 | frameshift_deleti  on | exon6 | c.702delA | p.P234fs | . | . |
| CTC2 | 18 | 67742618 | CA | C | 0.361 | NM_173630 | RTTN | frameshift_deleti  on | exon33 | c.4533delT | p.F1511fs | . | . |
| CTC2 | 18 | 67863850 | T | TCTC | 1 | NM_173630 | RTTN | nonframeshift_in  sertion | exon7 | c.727_728insG  AG | p.D243delinsG  D | . | . |
| CTC2 | 18 | 71987766 | TC | T | 0.6 | NM_001174123 | C18orf63 | frameshift_deleti  on | exon3 | c.154delC | p.Q52fs | . | . |
| CTC2 | 18 | 72178120 | AT | A | 0.538 | NM_001168499 | CNDP2 | frameshift_deleti  on | exon3 | c.278delT | p.I93fs | . | . |
| CTC2 | 18 | 72223591 | G | GTGC | 0.929 | NM_032649 | CNDP1 | nonframeshift_in  sertion | exon2 | c.43_44insTGC | p.V15delinsVL | . | . |
| CTC2 | 18 | 72345011 | GA | G | 0.906 | NM_001146189 | ZNF407 | frameshift_deleti  on | exon1 | c.2037delA | p.G679fs | . | . |
| CTC2 | 18 | 72346987 | G | GA | 0.515 | NM_001146189 | ZNF407 | frameshift_inserti  on | exon1 | c.4013dupA | p.E1338fs | . | . |
| CTC2 | 18 | 74082488 | TG | T | 1 | NM_014643 | ZNF516 | frameshift_deleti  on | exon7 | c.3426delC | p.P1142fs | . | . |
| CTC2 | 18 | 74092160 | C | CT | 0.364 | NM_014643 | ZNF516 | frameshift_inserti  on | exon4 | c.1909dupA | p.R637fs | . | . |
| CTC2 | 18 | 77475187 | TGGA | T | 0.289 | NM_001202504 | CTDP1 | nonframeshift_de  letion | exon8 | c.1371_1373del | p.457_458del | . | . |
| CTC2 | 19 | 2189760 | AG | A | 0.309 | NM_032482 | DOT1L | frameshift_deleti  on | exon4 | c.231delG | p.K77fs | . | . |
| CTC2 | 19 | 2211785 | TAC | T | 0.583 | NM_032482 | DOT1L | frameshift_deleti  on | exon16 | c.1502_1503del | p.Y501fs | . | . |
| CTC2 | 19 | 2877281 | AG | A | 0.171 | NM_024967 | ZNF556 | frameshift_deleti  on | exon4 | c.326delG | p.R109fs | . | . |
| CTC2 | 19 | 3961141 | CG | C | 1 | NM_001348 | DAPK3 | frameshift_deleti  on | exon6 | c.647delC | p.P216fs | . | . |
| CTC2 | 19 | 4446114 | CA | C | 0.667 | NM_001171091 | UBXN6 | frameshift_deleti  on | exon10 | c.972delT | p.F324fs | . | . |
| CTC2 | 19 | 4659952 | TG | T | 0.667 | NM_019107 | C19orf10 | frameshift_deleti  on | exon5 | c.432delC | p.T144fs | . | . |
| CTC2 | 19 | 4816773 | C | CA | 1 | NM_182919 | TICAM1 | frameshift_inserti  on | exon2 | c.1616dupT | p.M539fs | . | . |

| CTC2 | 19 | 5208307 | GC | G | 0.36 | NM_130853 | PTPRS | frameshift_deleti  on | exon27 | c.4241delG | p.G1414fs | . | . |
| --- | --- | --- | --- | --- | --- | --- | --- | --- | --- | --- | --- | --- | --- |
| CTC2 | 19 | 5240320 | GC | G | 0.731 | NM_130853 | PTPRS | frameshift_deleti  on | exon9 | c.1554delG | p.L518fs | . | . |
| CTC2 | 19 | 6040051 | GTGGA | G | 0.154 | NM_000635 | RFX2 | frameshift_deleti  on | exon5 | c.458_461del | p.I153fs | . | . |
| CTC2 | 19 | 6707926 | CA | C | 0.148 | NM_000064 | C3 | frameshift_deleti  on | exon15 | c.1859delT | p.V620fs | . | . |
| CTC2 | 19 | 6732086 | CA | C | 1 | NM_001080452 | GPR108 | frameshift_deleti  on | exon13 | c.1205delT | p.L402fs | . | . |
| CTC2 | 19 | 6741245 | GA | G | 0.567 | NM_001288962 | TRIP10 | frameshift_deleti  on | exon3 | c.151delA | p.K51fs | . | . |
| CTC2 | 19 | 6741274 | AG | A | 0.548 | NM_001288962 | TRIP10 | frameshift_deleti  on | exon3 | c.180delG | p.K60fs | . | . |
| CTC2 | 19 | 7267382 | CA | C | 1 | NM_000208 | INSR | frameshift_deleti  on | exon2 | c.625delT | p.C209fs | . | . |
| CTC2 | 19 | 7754202 | CA | C | 0.469 | NM_001207019 | FCER2 | frameshift_deleti  on | exon10 | c.839delT | p.V280fs | . | . |
| CTC2 | 19 | 8174578 | CG | C | 0.474 | NM_032447 | FBN3 | frameshift_deleti  on | exon34 | c.4392delC | p.P1464fs | . | . |
| CTC2 | 19 | 8435980 | AG | A | 0.522 | NM_001039667 | ANGPTL4 | frameshift_deleti  on | exon4 | c.589delG | p.V197fs | . | . |
| CTC2 | 19 | 8436221 | AG | A | 0.333 | NM_001039667 | ANGPTL4 | frameshift_deleti  on | exon5 | c.741delG | p.E247fs | . | . |
| CTC2 | 19 | 8503350 | GC | G | 0.68 | NM_001005416 | 2-Mar | frameshift_deleti  on | exon4 | c.452delC | p.A151fs | . | . |
| CTC2 | 19 | 9002656 | GC | G | 0.352 | NM_024690 | MUC16 | frameshift_deleti  on | exon51 | c.40159delG | p.A13387fs | . | . |
| CTC2 | 19 | 9021175 | GC | G | 0.365 | NM_024690 | MUC16 | frameshift_deleti  on | exon19 | c.37147delG | p.A12383fs | . | . |
| CTC2 | 19 | 9076323 | GAA | G | 0.333 | NM_024690 | MUC16 | frameshift_deleti  on | exon3 | c.11121_11122  del | p.V3707fs | . | . |
| CTC2 | 19 | 9085857 | TG | T | 0.375 | NM_024690 | MUC16 | frameshift_deleti  on | exon1 | c.5957delC | p.P1986fs | . | . |
| CTC2 | 19 | 9090443 | TG | T | 0.709 | NM_024690 | MUC16 | frameshift_deleti  on | exon1 | c.1371delC | p.P457fs | . | . |
| CTC2 | 19 | 9491643 | CT | C | 1 | NM_001172651 | ZNF177 | frameshift_deleti  on | exon6 | c.637delT | p.F213fs | . | . |
| CTC2 | 19 | 9639430 | AC | A | 1 | NM_024106 | ZNF426 | frameshift_deleti  on | exon8 | c.1290delG | p.G430fs | . | . |
| CTC2 | 19 | 9806772 | TG | T | 0.567 | NM_001199814 | ZNF812 | frameshift_deleti  on | exon3 | c.22delC | p.H8fs | . | . |
| CTC2 | 19 | 10108801 | CT | C | 0.254 | NM_015719 | COL5A3 | frameshift_deleti  on | exon9 | c.1134delA | p.K378fs | . | . |
| CTC2 | 19 | 10251843 | CT | C | 0.247 | NM_001379 | DNMT1 | frameshift_deleti  on | exon30 | c.3283delA | p.S1095fs | . | . |
| CTC2 | 19 | 10597458 | TC | T | 1 | NM_012289 | KEAP1 | frameshift_deleti  on | exon6 | c.1744delG | p.E582fs | . | . |
| CTC2 | 19 | 11305171 | TG | T | 0.868 | NM_015493 | KANK2 | frameshift_deleti  on | exon1 | c.16delC | p.H6fs | . | . |
| CTC2 | 19 | 11489113 | TG | T | 0.391 | NM_000121 | EPOR | frameshift_deleti  on | exon8 | c.1073delC | p.P358fs | . | . |
| CTC2 | 19 | 11558340 | AGAG | A | 0.364 | NM_001289102 | PRKCSH | nonframeshift_de  letion | exon10 | c.937_939del | p.313_313del | . | . |
| CTC2 | 19 | 11569068 | TC | T | 0.241 | NM_001420 | ELAVL3 | frameshift_deleti  on | exon5 | c.520delG | p.D174fs | . | . |
| CTC2 | 19 | 11664579 | GC | G | 1 | NM_032377 | ELOF1 | frameshift_deleti  on | exon4 | c.233delG | p.C78fs | . | . |
| CTC2 | 19 | 11942399 | TC | T | 0.326 | NM_152357 | ZNF440 | frameshift_deleti  on | exon4 | c.409delC | p.Q137fs | . | . |
| CTC2 | 19 | 12036083 | G | GA | 0.625 | NM_001271848 | ZNF700 | frameshift_inserti  on | exon1 | c.59dupA | p.E20fs | . | . |
| CTC2 | 19 | 12155708 | GT | G | 0.676 | NM_001080404 | ZNF878 | frameshift_deleti  on | exon4 | c.507delA | p.K169fs | . | . |
| CTC2 | 19 | 12503422 | AT | A | 0.667 | NM_001080821 | ZNF799 | frameshift_deleti  on | exon3 | c.185delA | p.N62fs | . | . |
| CTC2 | 19 | 12822119 | AGTC | A | 1 | NM_001136196 | TNPO2 | nonframeshift_de  letion | exon11 | c.1105_1107del | p.369_369del | . | . |
| CTC2 | 19 | 12858289 | C | CA | 0.333 | NM_004317 | ASNA1 | frameshift_inserti  on | exon6 | c.799dupA | p.A266fs | . | . |
| CTC2 | 19 | 13446645 | TC | T | 1 | NM_001127221 | CACNA1A | frameshift_deleti  on | exon7 | c.1056delG | p.L352fs | . | . |
| CTC2 | 19 | 13928165 | C | CT | 0.234 | NM_023072 | ZSWIM4 | frameshift_inserti  on | exon7 | c.1316_1317ins  T | p.P439fs | . | . |
| CTC2 | 19 | 14194117 | CT | C | 0.444 | NM_001277378 | C19orf67 | frameshift_deleti  on | exon4 | c.698delA | p.K233fs | . | . |
| CTC2 | 19 | 14580780 | AC | A | 0.353 | NM_002741 | PKN1 | frameshift_deleti  on | exon18 | c.2274delC | p.D758fs | . | . |
| CTC2 | 19 | 14626855 | GT | G | 0.296 | NM_006145 | DNAJB1 | frameshift_deleti  on | exon3 | c.919delA | p.T307fs | . | . |
| CTC2 | 19 | 15052983 | T | TATC | 1 | NM_012377 | OR7C2 | nonframeshift_in  sertion | exon1 | c.683_684insAT  C | p.V228delinsVS | . | . |
| CTC2 | 19 | 15350584 | TCAC | T | 1 | NM_058243 | BRD4 | nonframeshift_de  letion | exon16 | c.3328_3330del | p.1110_1110de  l | . | . |
| CTC2 | 19 | 15473002 | C | CT | 0.219 | NM_005858 | AKAP8 | frameshift_inserti  on | exon10 | c.1220dupA | p.K407fs | . | . |
| CTC2 | 19 | 15547682 | CG | C | 0.294 | NM_021241 | WIZ | frameshift_deleti  on | exon3 | c.463delC | p.R155fs | . | . |
| CTC2 | 19 | 15574935 | GT | G | 0.23 | NM_022904 | RASAL3 | frameshift_deleti  on | exon2 | c.234delA | p.S78fs | . | . |
| CTC2 | 19 | 16664587 | TC | T | 0.318 | NM_024881 | SLC35E1 | frameshift_deleti  on | exon6 | c.1135delG | p.D379fs | . | . |
| CTC2 | 19 | 16980530 | AG | A | 0.275 | NM_015260 | SIN3B | frameshift_deleti  on | exon14 | c.2163delG | p.Q721fs | . | . |
| CTC2 | 19 | 17115098 | CA | C | 0.4 | NM_015692 | CPAMD8 | frameshift_deleti  on | exon8 | c.798delT | p.F266fs | . | . |
| CTC2 | 19 | 17305658 | TC | T | 0.529 | NM_001130065 | MYO9B | frameshift_deleti  on | exon22 | c.3423delC | p.V1141fs | . | . |
| CTC2 | 19 | 17379850 | G | GC | 0.379 | NM_001033549 | BABAM1 | frameshift_inserti  on | exon2 | c.236dupC | p.A79fs | . | . |
| CTC2 | 19 | 17892623 | GC | G | 0.292 | NM_001161359 | FCHO1 | frameshift_deleti  on | exon21 | c.1782delC | p.S594fs | . | . |
| CTC2 | 19 | 18375479 | GC | G | 0.697 | NM_001145304 | KIAA1683 | frameshift_deleti  on | exon3 | c.2870delG | p.G957fs | . | . |
| CTC2 | 19 | 18376583 | T | TC | 0.4 | NM_001145304 | KIAA1683 | frameshift_inserti  on | exon3 | c.1766dupG | p.G589fs | . | . |
| CTC2 | 19 | 18376952 | GT | G | 0.941 | NM_001145304 | KIAA1683 | frameshift_deleti  on | exon3 | c.1397delA | p.N466fs | . | . |
| CTC2 | 19 | 18547653 | CG | C | 0.138 | NM_001253389 | ISYNA1 | frameshift_deleti  on | exon2 | c.65delC | p.A22fs | . | . |
| CTC2 | 19 | 19038831 | A | AT | 0.396 | NM_019070 | DDX49 | frameshift_inserti  on | exon12 | c.1250dupT | p.I417fs | . | . |
| CTC2 | 19 | 19349116 | AT | A | 0.778 | NM_004386 | NCAN | frameshift_deleti  on | exon11 | c.3306delT | p.Y1102fs | . | . |
| CTC2 | 19 | 19653201 | TG | T | 1 | NM_153221 | CILP2 | frameshift_deleti  on | exon5 | c.611delG | p.C204fs | . | . |

| CTC2 | 19 | 19655661 | CG | C | 1 | NM_153221 | CILP2 | frameshift_deleti  on | exon8 | c.2308delG | p.G770fs | . | . |
| --- | --- | --- | --- | --- | --- | --- | --- | --- | --- | --- | --- | --- | --- |
| CTC2 | 19 | 19770431 | TC | T | 1 | NM_020410 | ATP13A1 | frameshift_deleti  on | exon3 | c.655delG | p.E219fs | . | . |
| CTC2 | 19 | 19770513 | CG | C | 1 | NM_020410 | ATP13A1 | frameshift_deleti  on | exon3 | c.573delC | p.P191fs | . | . |
| CTC2 | 19 | 20002404 | TA | T | 0.231 | NM_021047 | ZNF253 | frameshift_deleti  on | exon4 | c.349delA | p.K117fs | . | . |
| CTC2 | 19 | 20307870 | TG | T | 1 | NM_052852 | ZNF486 | frameshift_deleti  on | exon4 | c.352delG | p.G118fs | . | . |
| CTC2 | 19 | 20807177 | G | GA | 0.45 | NM_001076675 | ZNF626 | frameshift_inserti  on | exon4 | c.1505dupT | p.I502fs | . | . |
| CTC2 | 19 | 21299936 | CT | C | 0.237 | NM_182515 | ZNF714 | frameshift_deleti  on | exon5 | c.467delT | p.L156fs | . | . |
| CTC2 | 19 | 22469352 | GA | G | 0.92 | NM_001242680 | ZNF729 | frameshift_deleti  on | exon1 | c.26delA | p.E9fs | . | . |
| CTC2 | 19 | 22942051 | CT | C | 0.268 | NM_001080409 | ZNF99 | frameshift_deleti  on | exon4 | c.659delA | p.K220fs | . | . |
| CTC2 | 19 | 23159628 | TC | T | 0.167 | NM_001267716 | ZNF728 | frameshift_deleti  on | exon4 | c.510delG | p.K170fs | . | . |
| CTC2 | 19 | 23837419 | AT | A | 0.37 | NM_138330 | ZNF675 | frameshift_deleti  on | exon4 | c.315delA | p.K105fs | . | . |
| CTC2 | 19 | 23926622 | AG | A | 0.75 | NM_138286 | ZNF681 | frameshift_deleti  on | exon4 | c.1729delC | p.L577fs | . | . |
| CTC2 | 19 | 23927741 | CCA | C | 0.533 | NM_138286 | ZNF681 | frameshift_deleti  on | exon4 | c.609_610del | p.C203fs | . | . |
| CTC2 | 19 | 30193736 | GT | G | 0.42 | NM_001282929 | C19orf12 | frameshift_deleti  on | exon2 | c.116delA | p.D39fs | . | . |
| CTC2 | 19 | 30312716 | A | AT | 0.212 | NM_001238 | CCNE1 | frameshift_inserti  on | exon8 | c.698dupT | p.I233fs | . | . |
| CTC2 | 19 | 32844413 | TC | T | 0.161 | NM_014910 | ZNF507 | frameshift_deleti  on | exon2 | c.678delC | p.V226fs | . | . |
| CTC2 | 19 | 35757303 | AT | A | 0.297 | NM_001260490 | LSR | frameshift_deleti  on | exon3 | c.641delT | p.I214fs | . | . |
| CTC2 | 19 | 36237740 | AC | A | 0.304 | NM_001281532 | PSENEN | frameshift_deleti  on | exon4 | c.299delC | p.T100fs | . | . |
| CTC2 | 19 | 36583665 | GGCA | G | 0.333 | NM_001083961 | WDR62 | nonframeshift_de  letion | exon19 | c.2286_2288del | p.762_763del | . | . |
| CTC2 | 19 | 37006018 | GT | G | 0.562 | NM_001166037 | ZNF260 | frameshift_deleti  on | exon3 | c.122delA | p.N41fs | . | . |
| CTC2 | 19 | 37383234 | CTCT | C | 0.606 | NM_001037232 | ZNF829 | nonframeshift_de  letion | exon6 | c.456_458del | p.152_153del | . | . |
| CTC2 | 19 | 37677480 | T | TG | 0.211 | NM_152279 | ZNF585B | frameshift_inserti  on | exon5 | c.958dupC | p.H320fs | . | . |
| CTC2 | 19 | 38027827 | GA | G | 0.667 | NM_001013659 | ZNF793 | frameshift_deleti  on | exon8 | c.268delA | p.K90fs | . | . |
| CTC2 | 19 | 38709286 | TC | T | 0.909 | NM_001135155 | DPF1 | frameshift_deleti  on | exon5 | c.523delG | p.E175fs | . | . |
| CTC2 | 19 | 38828020 | TC | T | 0.225 | NM_021185 | CATSPERG | frameshift_deleti  on | exon2 | c.147delC | p.V49fs | . | . |
| CTC2 | 19 | 38842984 | CGA | C | 0.458 | NM_021185 | CATSPERG | frameshift_deleti  on | exon8 | c.910_911del | p.E304fs | . | . |
| CTC2 | 19 | 38993348 | TG | T | 0.314 | NM_000540 | RYR1 | frameshift_deleti  on | exon48 | c.7817delG | p.C2606fs | . | . |
| CTC2 | 19 | 39087732 | TC | T | 0.405 | NM_001042600 | MAP4K1 | frameshift_deleti  on | exon25 | c.1882delG | p.E628fs | . | . |
| CTC2 | 19 | 39208571 | TCAA | T | 0.632 | NM_004924 | ACTN4 | nonframeshift_de  letion | exon11 | c.1149_1151del | p.383_384del | . | . |
| CTC2 | 19 | 39292451 | TG | T | 1 | NM_006149 | LGALS4 | frameshift_deleti  on | exon10 | c.924delC | p.D308fs | . | . |
| CTC2 | 19 | 39367354 | TC | T | 0.233 | NM_001195833 | RINL | frameshift_deleti  on | exon2 | c.28delG | p.E10fs | . | . |
| CTC2 | 19 | 39576000 | GC | G | 0.48 | NM_001004318 | PAPL | frameshift_deleti  on | exon2 | c.92delC | p.A31fs | . | . |
| CTC2 | 19 | 39804953 | C | CCAG | 0.127 | NM_020862 | LRFN1 | nonframeshift_in  sertion | exon1 | c.1023_1024ins  CTG | p.G342delinsLG | . | . |
| CTC2 | 19 | 40276728 | CTT | C | 0.636 | NM_001143832 | LEUTX | frameshift_deleti  on | exon3 | c.461_462del | p.L154fs | . | . |
| CTC2 | 19 | 40331276 | ACCG | A | 0.248 | NM_001436 | FBL | nonframeshift_de  letion | exon2 | c.159_161del | p.53_54del | . | . |
| CTC2 | 19 | 40368338 | AC | A | 0.455 | NM_003890 | FCGBP | frameshift_deleti  on | exon28 | c.13009delG | p.V4337fs | . | . |
| CTC2 | 19 | 40834434 | TG | T | 0.385 | NM_001256440 | C19orf47 | frameshift_deleti  on | exon6 | c.435delC | p.N145fs | . | . |
| CTC2 | 19 | 41096173 | AC | A | 0.611 | NM_138392 | SHKBP1 | frameshift_deleti  on | exon16 | c.1614delC | p.D538fs | . | . |
| CTC2 | 19 | 41726597 | CG | C | 1 | NM_001699 | AXL | frameshift_deleti  on | exon2 | c.143delG | p.R48fs | . | . |
| CTC2 | 19 | 41850753 | GA | G | 0.6 | NM_000660 | TGFB1 | frameshift_deleti  on | exon3 | c.532delT | p.S178fs | . | . |
| CTC2 | 19 | 42410139 | GC | G | 0.34 | NM_198977 | ARHGEF1 | frameshift_deleti  on | exon25 | c.2355delC | p.G785fs | . | . |
| CTC2 | 19 | 42799235 | GC | G | 0.571 | NM_015125 | CIC | frameshift_deleti  on | exon20 | c.4720delC | p.P1574fs | . | . |
| CTC2 | 19 | 42837857 | GC | G | 0.316 | NM_001271938 | MEGF8 | frameshift_deleti  on | exon2 | c.289delC | p.P97fs | . | . |
| CTC2 | 19 | 42860561 | TG | T | 0.824 | NM_001410 | MEGF8 | frameshift_deleti  on | exon25 | c.4378delG | p.G1460fs | . | . |
| CTC2 | 19 | 42863093 | C | CT | 0.417 | NM_001410 | MEGF8 | frameshift_inserti  on | exon29 | c.5130dupT | p.P1710fs | . | . |
| CTC2 | 19 | 42865132 | CT | C | 0.486 | NM_001410 | MEGF8 | frameshift_deleti  on | exon31 | c.5494delT | p.C1832fs | . | . |
| CTC2 | 19 | 43025594 | TG | T | 0.389 | NM_001024912 | CEACAM1 | frameshift_deleti  on | exon4 | c.782delC | p.A261fs | . | . |
| CTC2 | 19 | 43233990 | A | AT | 0.619 | NM_021016 | PSG3 | frameshift_inserti  on | exon4 | c.927dupA | p.C310fs | . | . |
| CTC2 | 19 | 43698584 | AT | A | 0.684 | NM_001276495 | PSG4 | stopgain | exon4 | c.871delA | p.I291X | . | . |
| CTC2 | 19 | 43968596 | AGGGCC TGCGCT | A | 0.107 | NM_014400 | LYPD3 | nonframeshift_de letion | exon2 | c.80_91del | p.27_31del | . | . |
| CTC2 | 19 | 44001334 | CTG | C | 0.366 | NM_198850 | PHLDB3 | frameshift_deleti  on | exon6 | c.759_760del | p.D253fs | . | . |
| CTC2 | 19 | 44223309 | AG | A | 1 | NM_019612 | IRGC | frameshift_deleti  on | exon2 | c.600delG | p.E200fs | . | . |
| CTC2 | 19 | 44605324 | AG | A | 0.397 | NM_013398 | ZNF224 | frameshift_deleti  on | exon5 | c.182delG | p.R61fs | . | . |
| CTC2 | 19 | 44840813 | CA | C | 0.286 | NM_013380 | ZNF112 | stopgain | exon3 | c.182delT | p.L61X | . | . |
| CTC2 | 19 | 44981954 | AT | A | 0.667 | NM_001278508 | ZNF180 | frameshift_deleti on | exon4 | c.668delA | p.N223fs | . | . |
| CTC2 | 19 | 45028255 | GC | G | 0.6 | NM_001102597 | CEACAM20 | frameshift_deleti  on | exon3 | c.235delG | p.A79fs | . | . |
| CTC2 | 19 | 45406363 | TG | T | 0.372 | NM_001128917 | TOMM40 | frameshift_deleti  on | exon9 | c.1024delG | p.G342fs | . | . |

| CTC2 | 19 | 46178017 | GC | G | 0.429 | NM_000164 | GIPR | frameshift_deleti  on | exon7 | c.567delC | p.S189fs | . | . |
| --- | --- | --- | --- | --- | --- | --- | --- | --- | --- | --- | --- | --- | --- |
| CTC2 | 19 | 46181373 | GC | G | 0.478 | NM_000164 | GIPR | frameshift_deleti  on | exon12 | c.1042delC | p.P348fs | . | . |
| CTC2 | 19 | 46303818 | A | AT | 0.409 | NM_030785 | RSPH6A | frameshift_inserti  on | exon5 | c.1801dupA | p.I601fs | . | . |
| CTC2 | 19 | 47423035 | AG | A | 0.34 | NM_004491 | ARHGAP35 | frameshift_deleti  on | exon1 | c.1104delG | p.K368fs | . | . |
| CTC2 | 19 | 47979881 | TG | T | 1 | NM_007059 | KPTN | frameshift_deleti  on | exon11 | c.1089delC | p.S363fs | . | . |
| CTC2 | 19 | 47980177 | A | AC | 0.333 | NM_007059 | KPTN | frameshift_inserti  on | exon10 | c.881dupG | p.G294fs | . | . |
| CTC2 | 19 | 48565284 | AG | A | 0.247 | NM_001159322 | PLA2G4C | frameshift_deleti  on | exon14 | c.1257delC | p.S419fs | . | . |
| CTC2 | 19 | 48734106 | AG | A | 0.4 | NM_001184900 | CARD8 | frameshift_deleti  on | exon6 | c.699delC | p.P233fs | . | . |
| CTC2 | 19 | 48977251 | AC | A | 0.308 | NM_004228 | CYTH2 | frameshift_deleti  on | exon6 | c.525delC | p.N175fs | . | . |
| CTC2 | 19 | 49248471 | CT | C | 0.55 | NM_182575 | IZUMO1 | frameshift_deleti  on | exon3 | c.309delA | p.K103fs | . | . |
| CTC2 | 19 | 49398306 | TA | T | 0.488 | NM_003323 | TULP2 | frameshift_deleti  on | exon6 | c.462delT | p.F154fs | . | . |
| CTC2 | 19 | 49651405 | GC | G | 0.312 | NM_003660 | PPFIA3 | frameshift_deleti  on | exon24 | c.2902delC | p.P968fs | . | . |
| CTC2 | 19 | 49657710 | ACAT | A | 0.683 | NM_002152 | HRC | nonframeshift_de  letion | exon1 | c.782_784del | p.261_262del | . | . |
| CTC2 | 19 | 49657751 | ATCG | A | 0.344 | NM_002152 | HRC | nonframeshift_de  letion | exon1 | c.741_743del | p.247_248del | . | . |
| CTC2 | 19 | 49657889 | T | TTCC | 0.932 | NM_002152 | HRC | nonframeshift_in  sertion | exon1 | c.605_606insG  GA | p.E202delinsEE | . | . |
| CTC2 | 19 | 49657892 | C | CTCT | 0.568 | NM_002152 | HRC | nonframeshift_in  sertion | exon1 | c.602_603insA  GA | p.E201delinsEE | . | . |
| CTC2 | 19 | 49657893 | T | TCCA | 0.545 | NM_002152 | HRC | nonframeshift_in  sertion | exon1 | c.601_602insTG  G | p.E201delinsVE | . | . |
| CTC2 | 19 | 49657900 | C | CCTG | 0.77 | NM_002152 | HRC | nonframeshift_in  sertion | exon1 | c.594_595insCA  G | p.E199delinsQE | . | . |
| CTC2 | 19 | 50017516 | AC | A | 0.875 | NM_004107 | FCGRT | frameshift_deleti  on | exon3 | c.375delC | p.N125fs | . | . |
| CTC2 | 19 | 50119216 | AG | A | 0.833 | NM_020719 | PRR12 | frameshift_deleti  on | exon9 | c.5238delG | p.K1746fs | . | . |
| CTC2 | 19 | 50193697 | C | CG | 0.875 | NM_001101340 | ADM5 | frameshift_inserti  on | exon2 | c.409_410insG | p.L137fs | . | . |
| CTC2 | 19 | 50364596 | GC | G | 1 | NM_007254 | PNKP | frameshift_deleti  on | exon17 | c.1474delG | p.A492fs | . | . |
| CTC2 | 19 | 50368513 | AG | A | 1 | NM_007254 | PNKP | frameshift_deleti  on | exon4 | c.368delC | p.P123fs | . | . |
| CTC2 | 19 | 50510971 | CCTGGT  CTTCTGA GGGCTA | C | 1 | NM_001025778 | VRK3 | nonframeshift_de letion | exon4 | c.231_251del | p.77_84del | . | . |
| CTC2 | 19 | 50510989 | ACAGCT  GGTCTTC TGAGGG | A | 1 | NM_001025778 | VRK3 | frameshift_deleti on | exon4 | c.212_233del | p.G71fs | . | . |
| CTC2 | 19 | 50881820 | A | AAAC | 1 | NM_001256647 | NR1H2 | nonframeshift_in  sertion | exon5 | c.223_224insAA  C | p.K75delinsKQ | . | . |
| CTC2 | 19 | 50881822 | A | ACAG | 0.905 | NM_001256647 | NR1H2 | nonframeshift_in  sertion | exon5 | c.225_226insCA  G | p.K75delinsKQ | . | . |
| CTC2 | 19 | 50881823 | C | CAGT | 0.583 | NM_001256647 | NR1H2 | stopgain | exon5 | c.226_227insA  GT | p.Q76delinsQX | . | . |
| CTC2 | 19 | 50906369 | TG | T | 0.8 | NM_001256849 | POLD1 | frameshift_deleti  on | exon9 | c.1031delG | p.W344fs | . | . |
| CTC2 | 19 | 50926117 | AG | A | 0.571 | NM_001243999 | SPIB | frameshift_deleti  on | exon4 | c.163delG | p.A55fs | . | . |
| CTC2 | 19 | 50983896 | GC | G | 0.318 | NM_175063 | EMC10 | frameshift_deleti  on | exon5 | c.462delC | p.G154fs | . | . |
| CTC2 | 19 | 51378097 | AC | A | 0.438 | NM_001002231 | KLK2 | frameshift_deleti  on | exon2 | c.168delC | p.H56fs | . | . |
| CTC2 | 19 | 51503418 | GTA | G | 0.134 | NM_007196 | KLK8 | frameshift_deleti  on | exon4 | c.325_326del | p.Y109fs | . | . |
| CTC2 | 19 | 51584924 | AT | A | 0.186 | NM_022046 | KLK14 | frameshift_deleti  on | exon4 | c.124delA | p.I42fs | . | . |
| CTC2 | 19 | 51845252 | AC | A | 0.989 | NM_001163922 | VSIG10L | stopgain | exon2 | c.49delG | p.V17X | . | . |
| CTC2 | 19 | 51919194 | TG | T | 0.886 | NM_001171156 | SIGLEC10 | frameshift_deleti on | exon5 | c.807delC | p.N269fs | . | . |
| CTC2 | 19 | 51919873 | A | AT | 1 | NM_001256795 | LOC1001290  83 | frameshift_inserti  on | exon2 | c.205_206insT | p.T69fs | . | . |
| CTC2 | 19 | 51961616 | GGCA | G | 0.134 | NM_014442 | SIGLEC8 | nonframeshift_de  letion | exon1 | c.23_25del | p.8_9del | . | . |
| CTC2 | 19 | 52004791 | G | GC | 0.49 | NM_053003 | SIGLEC12 | frameshift_inserti  on | exon1 | c.196dupG | p.A66fs | . | . |
| CTC2 | 19 | 52496939 | CT | C | 0.364 | NM_198480 | ZNF615 | frameshift_deleti  on | exon6 | c.1389delA | p.K463fs | . | . |
| CTC2 | 19 | 52705286 | AG | A | 0.5 | NM_014225 | PPP2R1A | frameshift_deleti  on | exon2 | c.169delG | p.D57fs | . | . |
| CTC2 | 19 | 52803669 | CTG | C | 1 | NM_144684 | ZNF480 | frameshift_deleti  on | exon2 | c.5_6del | p.L2fs | . | . |
| CTC2 | 19 | 52857032 | TG | T | 0.276 | NM_001161425 | ZNF610 | frameshift_deleti  on | exon4 | c.162delG | p.L54fs | . | . |
| CTC2 | 19 | 52887348 | C | CCT | 0.429 | NM_001145434 | ZNF880 | frameshift_inserti  on | exon4 | c.515_516insCT | p.P172fs | . | . |
| CTC2 | 19 | 53304102 | AC | A | 0.474 | NM_006969 | ZNF28 | frameshift_deleti  on | exon4 | c.995delG | p.C332fs | . | . |
| CTC2 | 19 | 53552520 | CT | C | 0.483 | NM_001191055 | ERVV-2 | frameshift_deleti  on | exon2 | c.17delT | p.L6fs | . | . |
| CTC2 | 19 | 53761716 | TG | T | 0.5 | NM_173856 | VN1R2 | frameshift_deleti  on | exon1 | c.89delG | p.C30fs | . | . |
| CTC2 | 19 | 53762539 | AC | A | 0.679 | NM_173856 | VN1R2 | frameshift_deleti  on | exon1 | c.912delC | p.Y304fs | . | . |
| CTC2 | 19 | 54140228 | AG | A | 0.163 | NM_001012728 | DPRX | frameshift_deleti  on | exon3 | c.563delG | p.S188fs | . | . |
| CTC2 | 19 | 54308572 | CT | C | 1 | NM_001277126 | NLRP12 | frameshift_deleti  on | exon5 | c.2378delA | p.E793fs | . | . |
| CTC2 | 19 | 54867613 | TG | T | 0.673 | NM_001289023 | LAIR1 | frameshift_deleti  on | exon8 | c.612delC | p.D204fs | . | . |
| CTC2 | 19 | 55175796 | GA | G | 0.269 | NM_001278429 | LILRB4 | frameshift_deleti  on | exon3 | c.603delA | p.G201fs | . | . |
| CTC2 | 19 | 55285083 | AG | A | 0.717 | NM_014218 | KIR2DL1 | frameshift_deleti  on | exon3 | c.370delG | p.G124fs | . | . |
| CTC2 | 19 | 55449590 | G | GA | 0.582 | NM_001127255 | NLRP7 | frameshift_inserti  on | exon5 | c.1950dupT | p.P651fs | . | . |
| CTC2 | 19 | 55593216 | GTGC | G | 0.526 | NM_017729 | EPS8L1 | nonframeshift_de  letion | exon4 | c.470_472del | p.157_158del | . | . |

| CTC2 | 19 | 55703106 | AC | A | 0.481 | NM_001161440 | PTPRH | frameshift_deleti  on | exon9 | c.1726delG | p.V576fs | . | . |
| --- | --- | --- | --- | --- | --- | --- | --- | --- | --- | --- | --- | --- | --- |
| CTC2 | 19 | 56052337 | A | AC | 0.984 | NM_001199824 | SBK3 | frameshift_inserti  on | exon4 | c.954dupG | p.F319fs | . | . |
| CTC2 | 19 | 56319288 | AG | A | 0.444 | NM_145007 | NLRP11 | frameshift_deleti  on | exon6 | c.1933delC | p.L645fs | . | . |
| CTC2 | 19 | 56320313 | CT | C | 0.204 | NM_145007 | NLRP11 | frameshift_deleti  on | exon5 | c.1662delA | p.E554fs | . | . |
| CTC2 | 19 | 56459546 | C | CT | 0.625 | NM_176811 | NLRP8 | frameshift_inserti  on | exon1 | c.279dupT | p.P93fs | . | . |
| CTC2 | 19 | 56467148 | TC | T | 0.762 | NM_176811 | NLRP8 | frameshift_deleti  on | exon3 | c.1725delC | p.F575fs | . | . |
| CTC2 | 19 | 57286285 | AT | A | 0.317 | NM_015363 | ZIM2 | frameshift_deleti  on | exon11 | c.1354delA | p.I452fs | . | . |
| CTC2 | 19 | 57640495 | CA | C | 0.526 | NM_020903 | USP29 | frameshift_deleti  on | exon4 | c.453delA | p.S151fs | . | . |
| CTC2 | 19 | 57642118 | AG | A | 0.312 | NM_020903 | USP29 | frameshift_deleti  on | exon4 | c.2076delG | p.Q692fs | . | . |
| CTC2 | 19 | 57867538 | AT | A | 0.256 | NM_020657 | ZNF304 | frameshift_deleti  on | exon3 | c.302delT | p.I101fs | . | . |
| CTC2 | 19 | 57868077 | GT | G | 0.236 | NM_020657 | ZNF304 | frameshift_deleti  on | exon3 | c.841delT | p.C281fs | . | . |
| CTC2 | 19 | 58018469 | TG | T | 0.949 | NM_198542 | ZNF773 | frameshift_deleti  on | exon4 | c.1007delG | p.C336fs | . | . |
| CTC2 | 19 | 58059083 | AC | A | 0.224 | NM_001277090 | ZNF550 | frameshift_deleti  on | exon4 | c.528delG | p.W176fs | . | . |
| CTC2 | 19 | 58197989 | AT | A | 0.304 | NM_001270938 | ZNF551 | frameshift_deleti  on | exon3 | c.263delT | p.I88fs | . | . |
| CTC2 | 19 | 58290825 | TG | T | 1 | NM_017652 | ZNF586 | frameshift_deleti  on | exon3 | c.871delG | p.E291fs | . | . |
| CTC2 | 19 | 58352656 | CT | C | 1 | NM_001204818 | ZNF587B | frameshift_deleti  on | exon3 | c.615delT | p.S205fs | . | . |
| CTC2 | 19 | 58437852 | TA | T | 0.256 | NM_133460 | ZNF418 | frameshift_deleti  on | exon4 | c.1696delT | p.Y566fs | . | . |
| CTC2 | 19 | 58500009 | CA | C | 0.432 | NM_025027 | ZNF606 | frameshift_deleti  on | exon5 | c.257delT | p.L86fs | . | . |
| CTC2 | 19 | 58500035 | GC | G | 0.363 | NM_025027 | ZNF606 | frameshift_deleti  on | exon5 | c.231delG | p.G77fs | . | . |
| CTC2 | 19 | 58578712 | AG | A | 0.467 | NM_001164530 | ZNF135 | frameshift_deleti  on | exon4 | c.897delG | p.Q299fs | . | . |
| CTC2 | 20 | 377137 | TG | T | 0.333 | NM_021158 | TRIB3 | frameshift_deleti  on | exon4 | c.881delG | p.C294fs | . | . |
| CTC2 | 20 | 2312737 | GA | G | 0.376 | NM_003245 | TGM3 | frameshift_deleti  on | exon10 | c.1424delA | p.E475fs | . | . |
| CTC2 | 20 | 2397946 | AG | A | 0.469 | NM_001254734 | TGM6 | frameshift_deleti  on | exon10 | c.1406delG | p.R469fs | . | . |
| CTC2 | 20 | 2596820 | TG | T | 0.209 | NM_080751 | TMC2 | frameshift_deleti  on | exon15 | c.1911delG | p.L637fs | . | . |
| CTC2 | 20 | 2739561 | CT | C | 0.248 | NM_001110514 | EBF4 | frameshift_deleti  on | exon17 | c.1734delT | p.S578fs | . | . |
| CTC2 | 20 | 3028426 | GT | G | 0.667 | NM_030811 | MRPS26 | frameshift_deleti  on | exon4 | c.530delT | p.V177fs | . | . |
| CTC2 | 20 | 3245129 | GA | G | 1 | NM_001009984 | C20orf194 | frameshift_deleti  on | exon31 | c.2827delT | p.S943fs | . | . |
| CTC2 | 20 | 3564725 | GC | G | 1 | NM_001207047 | ATRN | frameshift_deleti  on | exon17 | c.2598delC | p.C866fs | . | . |
| CTC2 | 20 | 3584788 | CA | C | 0.417 | NM_001207047 | ATRN | frameshift_deleti  on | exon24 | c.3333delA | p.S1111fs | . | . |
| CTC2 | 20 | 3765749 | TCC | T | 1 | NM_001810 | CENPB | frameshift_deleti  on | exon1 | c.1380_1381del | p.E460fs | . | . |
| CTC2 | 20 | 3888833 | C | CA | 1 | NM_024960 | PANK2 | frameshift_inserti  on | exon2 | c.17dupA | p.Q6fs | . | . |
| CTC2 | 20 | 5163080 | CA | C | 1 | NM_003818 | CDS2 | frameshift_deleti  on | exon6 | c.578delA | p.Q193fs | . | . |
| CTC2 | 20 | 5554568 | GC | G | 1 | NM_019593 | GPCPD1 | frameshift_deleti  on | exon11 | c.1048delG | p.A350fs | . | . |
| CTC2 | 20 | 5903282 | TGAG | T | 0.214 | NM_001819 | CHGB | nonframeshift_de  letion | exon4 | c.493_495del | p.165_165del | . | . |
| CTC2 | 20 | 6093203 | TTTC | T | 0.222 | NM_017671 | FERMT1 | nonframeshift_de  letion | exon4 | c.450_452del | p.150_151del | . | . |
| CTC2 | 20 | 6096488 | TA | T | 0.375 | NM_017671 | FERMT1 | frameshift_deleti  on | exon3 | c.354delT | p.F118fs | . | . |
| CTC2 | 20 | 6096619 | CA | C | 0.333 | NM_017671 | FERMT1 | frameshift_deleti  on | exon3 | c.223delT | p.W75fs | . | . |
| CTC2 | 20 | 10386256 | CA | C | 0.333 | NM_018848 | MKKS | frameshift_deleti  on | exon6 | c.1351delT | p.C451fs | . | . |
| CTC2 | 20 | 13052905 | AT | A | 0.346 | NM_018327 | SPTLC3 | frameshift_deleti  on | exon3 | c.306delT | p.D102fs | . | . |
| CTC2 | 20 | 13251163 | GT | G | 0.419 | NM_080826 | ISM1 | frameshift_deleti  on | exon2 | c.152delT | p.V51fs | . | . |
| CTC2 | 20 | 13856753 | TA | T | 0.328 | NM_001271539 | SEL1L2 | stopgain | exon12 | c.1034delT | p.L345X | . | . |
| CTC2 | 20 | 16030474 | GC | G | 0.253 | NM_001033087 | MACROD2 | frameshift_deleti on | exon14 | c.596delC | p.A199fs | . | . |
| CTC2 | 20 | 25287527 | CA | C | 0.375 | NM_001042472 | ABHD12 | frameshift_deleti  on | exon10 | c.891delT | p.F297fs | . | . |
| CTC2 | 20 | 25657229 | CCT | C | 0.889 | NM_015655 | ZNF337 | frameshift_deleti  on | exon5 | c.693_694del | p.T231fs | . | . |
| CTC2 | 20 | 30137908 | CG | C | 0.622 | NM_030789 | HM13 | frameshift_deleti  on | exon7 | c.709delG | p.E237fs | . | . |
| CTC2 | 20 | 30789951 | AG | A | 0.542 | NM_002657 | PLAGL2 | frameshift_deleti  on | exon2 | c.30delC | p.P10fs | . | . |
| CTC2 | 20 | 31040070 | AG | A | 0.733 | NM_080616 | C20orf112 | frameshift_deleti  on | exon7 | c.1058delC | p.P353fs | . | . |
| CTC2 | 20 | 31367942 | TC | T | 0.462 | NM_175850 | DNMT3B | frameshift_deleti  on | exon1 | c.22delC | p.P8fs | . | . |
| CTC2 | 20 | 31984736 | CT | C | 1 | NM_001278167 | CDK5RAP1 | frameshift_deleti  on | exon2 | c.134delA | p.E45fs | . | . |
| CTC2 | 20 | 32264635 | TG | T | 0.417 | NM_005225 | E2F1 | frameshift_deleti  on | exon7 | c.1216delC | p.H406fs | . | . |
| CTC2 | 20 | 32267695 | C | CT | 0.409 | NM_005225 | E2F1 | frameshift_inserti  on | exon3 | c.437_438insA | p.S146fs | . | . |
| CTC2 | 20 | 32295525 | CG | C | 0.353 | NM_007238 | PXMP4 | frameshift_deleti  on | exon4 | c.625delC | p.R209fs | . | . |
| CTC2 | 20 | 33875230 | GGC | G | 0.333 | NM_178468 | FAM83C | frameshift_deleti  on | exon4 | c.1350_1351del | p.R450fs | . | . |
| CTC2 | 20 | 33875232 | C | CG | 0.333 | NM_178468 | FAM83C | frameshift_inserti  on | exon4 | c.1349_1350ins  C | p.R450fs | . | . |
| CTC2 | 20 | 33875330 | C | CT | 0.391 | NM_178468 | FAM83C | frameshift_inserti  on | exon4 | c.1251dupA | p.G418fs | . | . |
| CTC2 | 20 | 34090453 | TG | T | 0.179 | NM_007186 | CEP250 | frameshift_deleti  on | exon30 | c.4257delG | p.L1419fs | . | . |
| CTC2 | 20 | 34218920 | AG | A | 0.394 | NM_001198863 | CPNE1 | frameshift_deleti  on | exon11 | c.897delC | p.P299fs | . | . |
| CTC2 | 20 | 35422286 | TG | T | 0.5 | NM_080627 | SOGA1 | frameshift_deleti  on | exon14 | c.4198delC | p.H1400fs | . | . |

| CTC2 | 20 | 36572535 | AG | A | 0.622 | NM_080607 | VSTM2L | frameshift_deleti  on | exon4 | c.496delG | p.G166fs | . | . |
| --- | --- | --- | --- | --- | --- | --- | --- | --- | --- | --- | --- | --- | --- |
| CTC2 | 20 | 37534711 | C | CT | 0.323 | NM_015568 | PPP1R16B | frameshift_inserti  on | exon7 | c.797dupT | p.L266fs | . | . |
| CTC2 | 20 | 40033402 | GTCT | G | 0.236 | NM_032221 | CHD6 | nonframeshift_de  letion | exon37 | c.7976_7978del | p.2659_2660de  l | . | . |
| CTC2 | 20 | 40033414 | TTCC | T | 0.167 | NM_032221 | CHD6 | nonframeshift_de  letion | exon37 | c.7964_7966del | p.2655_2656de  l | . | . |
| CTC2 | 20 | 40117139 | TC | T | 0.25 | NM_032221 | CHD6 | frameshift_deleti  on | exon13 | c.1785delG | p.V595fs | . | . |
| CTC2 | 20 | 42169425 | TG | T | 0.798 | NM_015478 | L3MBTL1 | frameshift_deleti  on | exon18 | c.2095delG | p.E699fs | . | . |
| CTC2 | 20 | 44437756 | GC | G | 0.222 | NM_052951 | DNTTIP1 | frameshift_deleti  on | exon11 | c.728delC | p.A243fs | . | . |
| CTC2 | 20 | 44469609 | TG | T | 0.25 | NM_033421 | SNX21 | frameshift_deleti  on | exon4 | c.780delG | p.L260fs | . | . |
| CTC2 | 20 | 44470581 | G | GC | 0.364 | NM_005469 | ACOT8 | frameshift_inserti  on | exon6 | c.855dupG | p.L286fs | . | . |
| CTC2 | 20 | 44511373 | CT | C | 0.218 | NM_080603 | ZSWIM1 | frameshift_deleti  on | exon2 | c.143delT | p.L48fs | . | . |
| CTC2 | 20 | 44522661 | CA | C | 0.241 | NM_001167594 | CTSA | frameshift_deleti  on | exon7 | c.731delA | p.Q244fs | . | . |
| CTC2 | 20 | 44567861 | GGT | G | 0.133 | NM_022104 | PCIF1 | frameshift_deleti  on | exon4 | c.133_134del | p.V45fs | . | . |
| CTC2 | 20 | 45353802 | AGCT | A | 0.471 | NM_030777 | SLC2A10 | nonframeshift_de  letion | exon2 | c.128_130del | p.43_44del | . | . |
| CTC2 | 20 | 46279836 | ACAG | A | 0.526 | NM_001174087 | NCOA3 | nonframeshift_de  letion | exon20 | c.3760_3762del | p.1254_1254de  l | . | . |
| CTC2 | 20 | 47342829 | TA | T | 0.312 | NM_020820 | PREX1 | frameshift_deleti  on | exon5 | c.618delT | p.L206fs | . | . |
| CTC2 | 20 | 47700578 | CT | C | 0.323 | NM_001256135 | CSE1L | frameshift_deleti  on | exon14 | c.1331delT | p.L444fs | . | . |
| CTC2 | 20 | 47841733 | GA | G | 0.545 | NM_017895 | DDX27 | frameshift_deleti  on | exon6 | c.691delA | p.K231fs | . | . |
| CTC2 | 20 | 47989844 | AC | A | 0.375 | NM_004975 | KCNB1 | frameshift_deleti  on | exon2 | c.2252delG | p.G751fs | . | . |
| CTC2 | 20 | 48439613 | TG | T | 0.284 | NM_001260491 | SLC9A8 | frameshift_deleti  on | exon3 | c.225delG | p.L75fs | . | . |
| CTC2 | 20 | 49209608 | CAG | C | 0.184 | NM_080829 | FAM65C | frameshift_deleti  on | exon18 | c.2324_2325del | p.S775fs | . | . |
| CTC2 | 20 | 49218656 | GC | G | 0.25 | NM_080829 | FAM65C | frameshift_deleti  on | exon13 | c.1599delG | p.Q533fs | . | . |
| CTC2 | 20 | 49510920 | AG | A | 0.367 | NM_001282532 | ADNP | frameshift_deleti  on | exon4 | c.330delC | p.P110fs | . | . |
| CTC2 | 20 | 50290765 | GA | G | 0.816 | NM_006045 | ATP9A | frameshift_deleti  on | exon11 | c.963delT | p.L321fs | . | . |
| CTC2 | 20 | 51871543 | AT | A | 0.37 | NM_001193421 | TSHZ2 | frameshift_deleti  on | exon2 | c.1538delT | p.I513fs | . | . |
| CTC2 | 20 | 52192922 | TG | T | 0.867 | NM_006526 | ZNF217 | frameshift_deleti  on | exon3 | c.2380delC | p.Q794fs | . | . |
| CTC2 | 20 | 52193128 | TA | T | 0.254 | NM_006526 | ZNF217 | stopgain | exon3 | c.2174delT | p.L725X | . | . |
| CTC2 | 20 | 56234744 | TC | T | 1 | NM_001255976 | PMEPA1 | frameshift_deleti on | exon2 | c.34delG | p.E12fs | . | . |
| CTC2 | 20 | 57564014 | G | GT | 0.235 | NM_198976 | NELFCD | frameshift_inserti  on | exon5 | c.496_497insT | p.D166fs | . | . |
| CTC2 | 20 | 57572701 | TC | T | 0.896 | NM_001336 | CTSZ | frameshift_deleti  on | exon4 | c.594delG | p.G198fs | . | . |
| CTC2 | 20 | 57829587 | TA | T | 0.188 | NM_178457 | ZNF831 | frameshift_deleti  on | exon5 | c.4824delA | p.L1608fs | . | . |
| CTC2 | 20 | 58468220 | TG | T | 0.571 | NM_014258 | SYCP2 | frameshift_deleti  on | exon21 | c.1769delC | p.A590fs | . | . |
| CTC2 | 20 | 60770912 | GC | G | 0.667 | NM_015666 | MTG2 | frameshift_deleti  on | exon3 | c.260delC | p.A87fs | . | . |
| CTC2 | 20 | 60898714 | TC | T | 1 | NM_005560 | LAMA5 | frameshift_deleti  on | exon45 | c.5861delG | p.G1954fs | . | . |
| CTC2 | 20 | 61918962 | CA | C | 0.933 | NM_001281484 | ARFGAP1 | frameshift_deleti  on | exon12 | c.620delA | p.Q207fs | . | . |
| CTC2 | 20 | 62839353 | AGAGGA  G | A | 0.2 | NM_004535 | MYT1 | nonframeshift_de  letion | exon7 | c.805_810del | p.269_270del | . | . |
| CTC2 | 20 | 62839358 | AG | A | 1 | NM_004535 | MYT1 | frameshift_deleti  on | exon7 | c.810delG | p.E270fs | . | . |
| CTC2 | 20 | 62851225 | AG | A | 0.556 | NM_004535 | MYT1 | frameshift_deleti  on | exon13 | c.2132delG | p.S711fs | . | . |
| CTC2 | 21 | 10942924 | CCTT | C | 0.134 | NM_199260 | TPTE | nonframeshift_de  letion | exon10 | c.546_548del | p.182_183del | . | . |
| CTC2 | 21 | 16340377 | TC | T | 0.5 | NM_003489 | NRIP1 | frameshift_deleti  on | exon4 | c.136delG | p.E46fs | . | . |
| CTC2 | 21 | 30331835 | TA | T | 0.353 | NM_015565 | LTN1 | frameshift_deleti  on | exon13 | c.2675delT | p.L892fs | . | . |
| CTC2 | 21 | 31744438 | TG | T | 0.333 | NM_181621 | KRTAP13-2 | frameshift_deleti  on | exon1 | c.93delC | p.P31fs | . | . |
| CTC2 | 21 | 31768591 | CCCA | C | 0.217 | NM_181599 | KRTAP13-1 | nonframeshift_de  letion | exon1 | c.188_190del | p.63_64del | . | . |
| CTC2 | 21 | 31913981 | AG | A | 1 | NM_181612 | KRTAP19-6 | frameshift_deleti  on | exon1 | c.171delC | p.F57fs | . | . |
| CTC2 | 21 | 32015337 | AT | A | 0.327 | NM_001128077 | KRTAP20-3 | frameshift_deleti  on | exon1 | c.131delT | p.I44fs | . | . |
| CTC2 | 21 | 32582400 | TG | T | 0.25 | NM_003253 | TIAM1 | frameshift_deleti  on | exon12 | c.2348delC | p.P783fs | . | . |
| CTC2 | 21 | 34003928 | A | AAGTA  TT | 1 | NM_001160306 | SYNJ1 | nonframeshift_in  sertion | exon28 | c.3957_3958ins  AATACT | p.L1320delinsN  TL | . | . |
| CTC2 | 21 | 34132284 | CT | C | 0.894 | NM_013329 | PAXBP1 | frameshift_deleti  on | exon6 | c.996delA | p.A332fs | . | . |
| CTC2 | 21 | 34903857 | C | CT | 0.667 | NM_000819 | GART | frameshift_inserti  on | exon6 | c.534dupA | p.A179fs | . | . |
| CTC2 | 21 | 34948684 | G | GA | 1 | NM_138927 | SON | frameshift_inserti  on | exon12 | c.7236dupA | p.G2412fs | . | . |
| CTC2 | 21 | 34948685 | A | AG | 1 | NM_138927 | SON | frameshift_inserti  on | exon12 | c.7237dupG | p.G2412fs | . | . |
| CTC2 | 21 | 34948696 | GA | G | 1 | NM_138927 | SON | frameshift_deleti  on | exon12 | c.7248delA | p.R2416fs | . | . |
| CTC2 | 21 | 35514877 | TC | T | 0.895 | NM_032476 | MRPS6 | frameshift_deleti  on | exon3 | c.356delC | p.S119fs | . | . |
| CTC2 | 21 | 37429481 | TC | T | 1 | NM_001007259 | SETD4 | frameshift_deleti  on | exon3 | c.94delG | p.E32fs | . | . |
| CTC2 | 21 | 37603169 | TG | T | 1 | NM_005128 | DOPEY2 | frameshift_deleti  on | exon14 | c.2088delG | p.L696fs | . | . |
| CTC2 | 21 | 40578127 | AT | A | 0.5 | NM_018963 | BRWD1 | frameshift_deleti  on | exon37 | c.4270delA | p.I1424fs | . | . |
| CTC2 | 21 | 40883671 | G | GAGA | 1 | NM_001001713 | SH3BGR | nonframeshift_in  sertion | exon6 | c.356_357insA  GA | p.G119delinsG  E | . | . |
| CTC2 | 21 | 43945899 | AT | A | 0.421 | NM_018964 | SLC37A1 | frameshift_deleti  on | exon4 | c.71delT | p.I24fs | . | . |

| CTC2 | 21 | 44324012 | CA | C | 0.791 | NM_021075 | NDUFV3 | frameshift_deleti  on | exon3 | c.891delA | p.S297fs | . | . |
| --- | --- | --- | --- | --- | --- | --- | --- | --- | --- | --- | --- | --- | --- |
| CTC2 | 21 | 44492254 | TG | T | 0.714 | NM_000071 | CBS | frameshift_deleti  on | exon3 | c.49delC | p.H17fs | . | . |
| CTC2 | 21 | 45472319 | CA | C | 0.462 | NM_003274 | TRAPPC10 | frameshift_deleti  on | exon4 | c.445delA | p.K149fs | . | . |
| CTC2 | 21 | 45774617 | TG | T | 0.221 | NM_003307 | TRPM2 | frameshift_deleti  on | exon2 | c.253delG | p.G85fs | . | . |
| CTC2 | 21 | 45970759 | ACTTGC  AGCAGA | A | 1 | NM_198693 | KRTAP10-2 | nonframeshift_de  letion | exon1 | c.568_582del | p.190_194del | . | . |
| CTC2 | 21 | 46047230 | AC | A | 0.667 | NM_198690 | KRTAP10-9 | frameshift_deleti  on | exon1 | c.143delC | p.T48fs | . | . |
| CTC2 | 21 | 47412714 | GA | G | 0.5 | NM_001848 | COL6A1 | frameshift_deleti  on | exon19 | c.1326delA | p.R442fs | . | . |
| CTC2 | 21 | 47711279 | AT | A | 0.447 | NM_001006114 | YBEY | frameshift_deleti  on | exon3 | c.243delT | p.D81fs | . | . |
| CTC2 | 21 | 47754404 | ACAGT | A | 0.144 | NM_006031 | PCNT | frameshift_deleti  on | exon3 | c.362_365del | p.T121fs | . | . |
| CTC2 | 21 | 47952069 | TG | T | 0.5 | NM_001146114 | DIP2A | stopgain | exon8 | c.1033delG | p.V345X | . | . |
| CTC2 | 21 | 47987467 | CG | C | 0.323 | NM_001146116 | DIP2A | frameshift_deleti on | exon38 | c.4637delG | p.R1546fs | . | . |
| CTC2 | 22 | 17469039 | G | GA | 0.317 | NM_001037814 | GAB4 | frameshift_inserti  on | exon3 | c.496dupT | p.S166fs | . | . |
| CTC2 | 22 | 18362165 | A | AT | 0.308 | NM_001136004 | MICAL3 | frameshift_inserti  on | exon17 | c.2343dupA | p.Y782fs | . | . |
| CTC2 | 22 | 18562757 | ACCC | A | 0.373 | NM_001127649 | PEX26 | nonframeshift_de  letion | exon2 | c.349_351del | p.117_117del | . | . |
| CTC2 | 22 | 19168280 | CT | C | 1 | NM_001835 | CLTCL1 | frameshift_deleti  on | exon30 | c.4695delA | p.Q1565fs | . | . |
| CTC2 | 22 | 19189003 | A | AC | 1 | NM_001835 | CLTCL1 | frameshift_inserti  on | exon23 | c.3601dupG | p.V1201fs | . | . |
| CTC2 | 22 | 20230403 | TG | T | 0.926 | NM_023004 | RTN4R | frameshift_deleti  on | exon2 | c.252delC | p.T84fs | . | . |
| CTC2 | 22 | 20920813 | ACAG | A | 0.389 | NM_001003891 | MED15 | nonframeshift_de  letion | exon7 | c.751_753del | p.251_251del | . | . |
| CTC2 | 22 | 21107255 | AG | A | 0.216 | NM_058004 | PI4KA | frameshift_deleti  on | exon25 | c.2922delC | p.N974fs | . | . |
| CTC2 | 22 | 21172809 | GGA | G | 0.265 | NM_058004 | PI4KA | frameshift_deleti  on | exon7 | c.816_817del | p.P272fs | . | . |
| CTC2 | 22 | 21172811 | AG | A | 0.441 | NM_058004 | PI4KA | frameshift_deleti  on | exon7 | c.815delC | p.P272fs | . | . |
| CTC2 | 22 | 21384466 | TC | T | 1 | NM_004173 | SLC7A4 | frameshift_deleti  on | exon3 | c.1156delG | p.D386fs | . | . |
| CTC2 | 22 | 22868515 | CG | C | 0.208 | NM_080740 | ZNF280A | frameshift_deleti  on | exon2 | c.1439delC | p.P480fs | . | . |
| CTC2 | 22 | 24087255 | GC | G | 0.243 | NM_021916 | ZNF70 | frameshift_deleti  on | exon2 | c.72delG | p.G24fs | . | . |
| CTC2 | 22 | 24219982 | CCTCCCT  GCCTCT | C | 0.714 | NM_001024938 | SLC2A11 | frameshift_deleti  on | exon6 | c.612_624del | p.A204fs | . | . |
| CTC2 | 22 | 24582005 | AC | A | 0.438 | NM_019601 | SUSD2 | frameshift_deleti  on | exon9 | c.1362delC | p.H454fs | . | . |
| CTC2 | 22 | 25246352 | TGA | T | 0.236 | NM_001039948 | SGSM1 | frameshift_deleti  on | exon5 | c.409_410del | p.E137fs | . | . |
| CTC2 | 22 | 25275449 | GT | G | 0.513 | NM_001098497 | SGSM1 | frameshift_deleti  on | exon14 | c.1452delT | p.C484fs | . | . |
| CTC2 | 22 | 25294044 | C | CA | 0.32 | NM_001098498 | SGSM1 | frameshift_inserti  on | exon18 | c.1945_1946ins  A | p.P649fs | . | . |
| CTC2 | 22 | 27008070 | CG | C | 0.211 | NM_001887 | CRYBB1 | frameshift_deleti  on | exon3 | c.264delC | p.F88fs | . | . |
| CTC2 | 22 | 28504227 | AT | A | 0.64 | NM_001145418 | TTC28 | frameshift_deleti  on | exon7 | c.1605delA | p.K535fs | . | . |
| CTC2 | 22 | 29092918 | AC | A | 0.268 | NM_007194 | CHEK2 | frameshift_deleti  on | exon10 | c.1065delG | p.L355fs | . | . |
| CTC2 | 22 | 29694812 | CGG | C | 0.167 | NM_001163286 | EWSR1 | frameshift_deleti  on | exon13 | c.1340_1341del | p.R447fs | . | . |
| CTC2 | 22 | 29837818 | A | AT | 0.296 | NM_021026 | RFPL1 | frameshift_inserti  on | exon2 | c.661_662insT | p.R221fs | . | . |
| CTC2 | 22 | 29885203 | AG | A | 0.5 | NM_021076 | NEFH | frameshift_deleti  on | exon4 | c.1575delG | p.K525fs | . | . |
| CTC2 | 22 | 30070826 | GC | G | 0.286 | NM_181830 | NF2 | frameshift_deleti  on | exon11 | c.1094delC | p.A365fs | . | . |
| CTC2 | 22 | 30416032 | AG | A | 0.375 | NM_021090 | MTMR3 | frameshift_deleti  on | exon17 | c.2385delG | p.Q795fs | . | . |
| CTC2 | 22 | 30659990 | TC | T | 1 | NM_020530 | OSM | frameshift_deleti  on | exon3 | c.640delG | p.E214fs | . | . |
| CTC2 | 22 | 31289132 | TGC | T | 0.406 | NM_001282742 | OSBP2 | frameshift_deleti  on | exon7 | c.526_527del | p.A176fs | . | . |
| CTC2 | 22 | 31976309 | CA | C | 0.4 | NM_001258326 | SFI1 | frameshift_deleti  on | exon10 | c.962delA | p.Q321fs | . | . |
| CTC2 | 22 | 32109640 | CA | C | 0.457 | NM_173566 | PRR14L | frameshift_deleti  on | exon4 | c.4184delT | p.L1395fs | . | . |
| CTC2 | 22 | 32110786 | CT | C | 0.5 | NM_173566 | PRR14L | frameshift_deleti  on | exon4 | c.3038delA | p.K1013fs | . | . |
| CTC2 | 22 | 32587030 | GC | G | 0.464 | NM_006605 | RFPL2 | frameshift_deleti  on | exon2 | c.682delG | p.A228fs | . | . |
| CTC2 | 22 | 33255186 | TG | T | 0.678 | NM_000362 | TIMP3 | frameshift_deleti  on | exon5 | c.459delG | p.L153fs | . | . |
| CTC2 | 22 | 36627395 | TCAG | T | 0.625 | NM_030882 | APOL2 | nonframeshift_de  letion | exon4 | c.125_127del | p.42_43del | . | . |
| CTC2 | 22 | 37524383 | TG | T | 0.817 | NM_000878 | IL2RB | frameshift_deleti  on | exon10 | c.1408delC | p.Q470fs | . | . |
| CTC2 | 22 | 37962664 | C | CT | 0.227 | NM_152243 | CDC42EP1 | frameshift_inserti  on | exon2 | c.309dupT | p.P103fs | . | . |
| CTC2 | 22 | 38025519 | TG | T | 0.321 | NM_001001560 | GGA1 | stopgain | exon10 | c.884delG | p.W295X | . | . |
| CTC2 | 22 | 38040669 | AC | A | 0.158 | NM_018957 | SH3BP1 | frameshift_deleti on | exon8 | c.645delC | p.H215fs | . | . |
| CTC2 | 22 | 38318009 | TG | T | 0.286 | NM_033386 | MICALL1 | frameshift_deleti  on | exon6 | c.601delG | p.G201fs | . | . |
| CTC2 | 22 | 38883914 | C | CA | 0.263 | NM_001098504 | DDX17 | frameshift_inserti  on | exon12 | c.1653dupT | p.V552fs | . | . |
| CTC2 | 22 | 39136316 | C | CT | 0.467 | NM_001199579 | SUN2 | frameshift_inserti  on | exon12 | c.1374dupA | p.E459fs | . | . |
| CTC2 | 22 | 39175590 | A | AT | 0.162 | NM_005740 | DNAL4 | frameshift_inserti  on | exon4 | c.181dupA | p.M61fs | . | . |
| CTC2 | 22 | 39482443 | AT | A | 0.185 | NM_021822 | APOBEC3G | frameshift_deleti  on | exon6 | c.896delT | p.I299fs | . | . |
| CTC2 | 22 | 39777822 | C | CCAA | 1 | NM_004711 | SYNGR1 | nonframeshift_in  sertion | exon4 | c.605_606insCA  A | p.P202delinsP  N | . | . |
| CTC2 | 22 | 39925521 | G | GT | 0.733 | NM_194326 | RPS19BP1 | frameshift_inserti  on | exon4 | c.386_387insA | p.F129fs | . | . |
| CTC2 | 22 | 40757339 | GC | G | 0.813 | NM_000026 | ADSL | frameshift_deleti  on | exon8 | c.856delC | p.Q286fs | . | . |

| CTC2 | 22 | 40807616 | CG | C | 0.603 | NM_001282660 | MKL1 | frameshift_deleti  on | exon12 | c.2573delC | p.P858fs | . | . |
| --- | --- | --- | --- | --- | --- | --- | --- | --- | --- | --- | --- | --- | --- |
| CTC2 | 22 | 41077529 | CT | C | 0.622 | NM_005297 | MCHR1 | frameshift_deleti  on | exon2 | c.867delT | p.P289fs | . | . |
| CTC2 | 22 | 41783416 | TG | T | 0.336 | NM_001145398 | TEF | frameshift_deleti  on | exon2 | c.130delG | p.V44fs | . | . |
| CTC2 | 22 | 42046776 | TA | T | 0.452 | NM_001288978 | XRCC6 | frameshift_deleti  on | exon7 | c.861delA | p.L287fs | . | . |
| CTC2 | 22 | 42086789 | TC | T | 0.684 | NM_001142964 | C22orf46 | frameshift_deleti  on | exon1 | c.159delC | p.V53fs | . | . |
| CTC2 | 22 | 42607728 | GT | G | 0.277 | NM_005650 | TCF20 | frameshift_deleti  on | exon1 | c.3583delA | p.T1195fs | . | . |
| CTC2 | 22 | 43195151 | GAC | G | 0.365 | NM_001142293 | ARFGAP3 | frameshift_deleti  on | exon14 | c.1293_1294del | p.L431fs | . | . |
| CTC2 | 22 | 44011799 | TG | T | 0.267 | NM_198856 | EFCAB6 | frameshift_deleti  on | exon19 | c.2012delC | p.P671fs | . | . |
| CTC2 | 22 | 44287072 | TG | T | 0.48 | NM_138814 | PNPLA5 | frameshift_deleti  on | exon2 | c.295delC | p.Q99fs | . | . |
| CTC2 | 22 | 46722369 | TG | T | 1 | NM_016426 | GTSE1 | frameshift_deleti  on | exon9 | c.1543delG | p.G515fs | . | . |
| CTC2 | 22 | 50632049 | GC | G | 0.381 | NM_025204 | TRABD | frameshift_deleti  on | exon3 | c.79delC | p.P27fs | . | . |
| CTC2 | 22 | 50751433 | GA | G | 0.273 | NM_001001794 | DENND6B | frameshift_deleti  on | exon17 | c.1451delT | p.L484fs | . | . |
| CTC2 | 22 | 50853071 | CTGG | C | 0.294 | NM_001242898 | PPP6R2 | nonframeshift_de  letion | exon5 | c.506_508del | p.169_170del | . | . |
| CTC2 | 22 | 50886798 | GC | G | 1 | NM_002972 | SBF1 | frameshift_deleti  on | exon38 | c.5226delG | p.G1742fs | . | . |
| CTC2 | 22 | 50897812 | TGGGAC  TGGCCT | T | 0.583 | NM_002972 | SBF1 | nonframeshift_de  letion | exon28 | c.3689_3697del | p.1230_1233de  l | . | . |
| CTC2 | 22 | 50954895 | CT | C | 1 | NM_001185011 | NCAPH2 | frameshift_deleti  on | exon2 | c.129delT | p.S43fs | . | . |
| CTC2 | 22 | 50966134 | GC | G | 0.889 | NM_001113756 | TYMP | frameshift_deleti  on | exon4 | c.528delG | p.L176fs | . | . |
| CTC2 | 22 | 50967612 | TG | T | 0.917 | NM_001113756 | TYMP | frameshift_deleti  on | exon2 | c.369delC | p.D123fs | . | . |
| CTC2 | 22 | 51065699 | TG | T | 0.143 | NM_000487 | ARSA | frameshift_deleti  on | exon2 | c.359delC | p.T120fs | . | . |
| CTC2 | X | 5821614 | GC | G | 1 | NM_001282146 | NLGN4X | frameshift_deleti  on | exon5 | c.1104delG | p.M368fs | . | . |
| CTC2 | X | 8536337 | TC | T | 0.414 | NM_000216 | KAL1 | frameshift_deleti  on | exon8 | c.1142delG | p.G381fs | . | . |
| CTC2 | X | 8766476 | TA | T | 1 | NM_001171186 | FAM9A | frameshift_deleti  on | exon4 | c.264delT | p.F88fs | . | . |
| CTC2 | X | 9935890 | CA | C | 0.333 | NM_001195081 | LOC1002888  14 | frameshift_deleti  on | exon1 | c.494delA | p.Q165fs | . | . |
| CTC2 | X | 11215106 | CT | C | 0.811 | NM_001287242 | ARHGAP6 | frameshift_deleti  on | exon3 | c.218delA | p.K73fs | . | . |
| CTC2 | X | 12904984 | G | GA | 1 | NM_016562 | TLR7 | frameshift_inserti  on | exon3 | c.1358dupA | p.E453fs | . | . |
| CTC2 | X | 12938381 | CA | C | 0.958 | NM_138636 | TLR8 | frameshift_deleti  on | exon2 | c.1223delA | p.Q408fs | . | . |
| CTC2 | X | 15349997 | CG | C | 0.184 | NM_002641 | PIGA | frameshift_deleti  on | exon2 | c.55delC | p.R19fs | . | . |
| CTC2 | X | 15609951 | TA | T | 0.645 | NM_021804 | ACE2 | stopgain | exon5 | c.467delT | p.L156X | . | . |
| CTC2 | X | 16836988 | GA | G | 0.8 | NM_018360 | TXLNG | frameshift_deleti on | exon2 | c.395delA | p.E132fs | . | . |
| CTC2 | X | 18276360 | GAC | G | 0.216 | NM_006089 | SCML2 | frameshift_deleti  on | exon10 | c.1075_1076del | p.V359fs | . | . |
| CTC2 | X | 19039242 | CT | C | 0.3 | NM_001184835 | GPR64 | frameshift_deleti  on | exon11 | c.508delA | p.R170fs | . | . |
| CTC2 | X | 21887643 | CT | C | 0.454 | NM_015884 | MBTPS2 | frameshift_deleti  on | exon7 | c.818delT | p.L273fs | . | . |
| CTC2 | X | 24228926 | CA | C | 0.385 | NM_001178086 | ZFX | frameshift_deleti  on | exon6 | c.1165delA | p.K389fs | . | . |
| CTC2 | X | 24381134 | A | AG | 0.955 | NM_001136234 | SUPT20HL1 | frameshift_inserti  on | exon1 | c.258dupG | p.Q86fs | . | . |
| CTC2 | X | 24382429 | CCTGCT  CCTGCT CTAGCT | C | 1 | NM_001136234 | SUPT20HL1 | nonframeshift_de letion | exon1 | c.1553_1576del | p.518_526del | . | . |
| CTC2 | X | 27765399 | AGAGGA  GGAGGA | A | 1 | NM_001136533 | DCAF8L2 | nonframeshift_de  letion | exon1 | c.388_399del | p.130_133del | . | . |
| CTC2 | X | 27765399 | AGAGGA  GGAGGA | A | 1 | NM_001136533 | DCAF8L2 | nonframeshift_de  letion | exon1 | c.388_402del | p.130_134del | . | . |
| CTC2 | X | 27765593 | AC | A | 0.669 | NM_001136533 | DCAF8L2 | frameshift_deleti  on | exon1 | c.582delC | p.H194fs | . | . |
| CTC2 | X | 34149747 | TG | T | 0.365 | NM_203408 | FAM47A | frameshift_deleti  on | exon1 | c.648delC | p.P216fs | . | . |
| CTC2 | X | 38146323 | TG | T | 0.625 | NM_001034853 | RPGR | stopgain | exon15 | c.1928delC | p.S643X | . | . |
| CTC2 | X | 38540539 | CA | C | 0.5 | NM_004615 | TSPAN7 | frameshift_deleti on | exon6 | c.680delA | p.Q227fs | . | . |
| CTC2 | X | 40541942 | TA | T | 0.571 | NM_004229 | MED14 | frameshift_deleti  on | exon18 | c.2277delT | p.G759fs | . | . |
| CTC2 | X | 46360078 | CT | C | 0.625 | NM_001039891 | ZNF674 | frameshift_deleti  on | exon6 | c.945delA | p.K315fs | . | . |
| CTC2 | X | 47271928 | TA | T | 0.415 | NM_003446 | ZNF157 | frameshift_deleti  on | exon4 | c.457delA | p.K153fs | . | . |
| CTC2 | X | 48320666 | AC | A | 0.812 | NM_033518 | SLC38A5 | frameshift_deleti  on | exon10 | c.585delG | p.G195fs | . | . |
| CTC2 | X | 48542736 | AG | A | 0.31 | NM_000377 | WAS | frameshift_deleti  on | exon2 | c.198delG | p.K66fs | . | . |
| CTC2 | X | 48672879 | A | AG | 0.684 | NM_006044 | HDAC6 | frameshift_inserti  on | exon11 | c.840dupG | p.Q280fs | . | . |
| CTC2 | X | 48759667 | CAG | C | 0.114 | NM_005710 | PQBP1 | frameshift_deleti  on | exon4 | c.451_452del | p.R151fs | . | . |
| CTC2 | X | 48772648 | GT | G | 1 | NM_006875 | PIM2 | frameshift_deleti  on | exon4 | c.243delA | p.P81fs | . | . |
| CTC2 | X | 48819959 | TG | T | 0.595 | NM_004979 | KCND1 | frameshift_deleti  on | exon6 | c.1826delC | p.P609fs | . | . |
| CTC2 | X | 48924755 | TC | T | 0.75 | NM_001163321 | CCDC120 | frameshift_deleti  on | exon10 | c.1106delC | p.S369fs | . | . |
| CTC2 | X | 50341412 | C | CA | 0.806 | NM_020717 | SHROOM4 | frameshift_inserti  on | exon8 | c.4065_4066ins  T | p.V1356fs | . | . |
| CTC2 | X | 53114880 | AT | A | 0.345 | NM_022117 | TSPYL2 | frameshift_deleti  on | exon6 | c.1307delT | p.I436fs | . | . |
| CTC2 | X | 53253971 | AT | A | 0.848 | NM_001146702 | KDM5C | frameshift_deleti  on | exon1 | c.100delA | p.I34fs | . | . |
| CTC2 | X | 53409195 | TC | T | 0.655 | NM_006306 | SMC1A | frameshift_deleti  on | exon22 | c.3394delG | p.E1132fs | . | . |
| CTC2 | X | 53432041 | TG | T | 0.829 | NM_006306 | SMC1A | frameshift_deleti  on | exon13 | c.2098delC | p.Q700fs | . | . |
| CTC2 | X | 53457922 | AC | A | 0.512 | NM_001031745 | RIBC1 | frameshift_deleti  on | exon8 | c.1127delC | p.T376fs | . | . |

| CTC2 | X | 53570900 | TG | T | 0.174 | NM_031407 | HUWE1 | frameshift_deleti  on | exon73 | c.11280delC | p.S3760fs | . | . |
| --- | --- | --- | --- | --- | --- | --- | --- | --- | --- | --- | --- | --- | --- |
| CTC2 | X | 53586316 | TG | T | 0.172 | NM_031407 | HUWE1 | frameshift_deleti  on | exon57 | c.7913delC | p.A2638fs | . | . |
| CTC2 | X | 53586470 | GC | G | 0.235 | NM_031407 | HUWE1 | frameshift_deleti  on | exon57 | c.7759delG | p.A2587fs | . | . |
| CTC2 | X | 54186002 | G | GA | 0.488 | NM_017848 | FAM120C | frameshift_inserti  on | exon2 | c.746dupT | p.F249fs | . | . |
| CTC2 | X | 54263832 | AT | A | 0.943 | NM_001002838 | WNK3 | frameshift_deleti  on | exon20 | c.4025delA | p.N1342fs | . | . |
| CTC2 | X | 55172590 | TA | T | 0.343 | NM_001166700 | FAM104B | frameshift_deleti  on | exon3 | c.277delT | p.Y93fs | . | . |
| CTC2 | X | 57473383 | TG | T | 0.325 | NM_174912 | FAAH2 | frameshift_deleti  on | exon9 | c.1140delG | p.L380fs | . | . |
| CTC2 | X | 63410770 | GT | G | 1 | NM_152424 | AMER1 | frameshift_deleti  on | exon2 | c.2396delA | p.N799fs | . | . |
| CTC2 | X | 69498450 | CA | C | 0.394 | NM_004312 | ARR3 | frameshift_deleti  on | exon12 | c.865delA | p.K289fs | . | . |
| CTC2 | X | 69639603 | TA | T | 0.266 | NM_012310 | KIF4A | frameshift_deleti  on | exon30 | c.3466delA | p.N1156fs | . | . |
| CTC2 | X | 70330368 | T | TG | 0.383 | NM_000206 | IL2RG | frameshift_inserti  on | exon3 | c.439_440insC | p.K147fs | . | . |
| CTC2 | X | 70347296 | AC | A | 0.596 | NM_005120 | MED12 | frameshift_deleti  on | exon21 | c.2961delC | p.N987fs | . | . |
| CTC2 | X | 70361097 | ACAG | A | 0.375 | NM_005120 | MED12 | nonframeshift_de  letion | exon43 | c.6286_6288del | p.2096_2096de  l | . | . |
| CTC2 | X | 70461078 | AT | A | 0.316 | NM_001171162 | ZMYM3 | frameshift_deleti  on | exon24 | c.3882delA | p.K1294fs | . | . |
| CTC2 | X | 70609464 | TG | T | 0.364 | NM_001286074 | TAF1 | frameshift_deleti  on | exon18 | c.2791delG | p.A931fs | . | . |
| CTC2 | X | 70609505 | TTGA | T | 0.571 | NM_001286074 | TAF1 | nonframeshift_de  letion | exon18 | c.2832_2834del | p.944_945del | . | . |
| CTC2 | X | 71360531 | TG | T | 0.365 | NM_001013627 | NHSL2 | frameshift_deleti  on | exon6 | c.3134delG | p.C1045fs | . | . |
| CTC2 | X | 71425151 | TG | T | 0.739 | NM_017669 | ERCC6L | frameshift_deleti  on | exon2 | c.3465delC | p.N1155fs | . | . |
| CTC2 | X | 71787797 | TC | T | 1 | NM_001166419 | HDAC8 | frameshift_deleti  on | exon4 | c.378delG | p.L126fs | . | . |
| CTC2 | X | 72433358 | GC | G | 0.194 | NM_021963 | NAP1L2 | frameshift_deleti  on | exon1 | c.970delG | p.A324fs | . | . |
| CTC2 | X | 72433663 | GTCC | G | 0.149 | NM_021963 | NAP1L2 | nonframeshift_de  letion | exon1 | c.663_665del | p.221_222del | . | . |
| CTC2 | X | 74494156 | AC | A | 0.49 | NM_145052 | UPRT | frameshift_deleti  on | exon1 | c.68delC | p.T23fs | . | . |
| CTC2 | X | 75648642 | AC | A | 0.745 | NM_020932 | MAGEE1 | frameshift_deleti  on | exon1 | c.320delC | p.T107fs | . | . |
| CTC2 | X | 76909606 | ATCT | A | 0.156 | NM_138270 | ATRX | nonframeshift_de  letion | exon13 | c.4182_4184del | p.1394_1395de  l | . | . |
| CTC2 | X | 76920156 | AT | A | 0.261 | NM_138270 | ATRX | frameshift_deleti  on | exon10 | c.3806delA | p.N1269fs | . | . |
| CTC2 | X | 76938366 | CT | C | 0.967 | NM_138270 | ATRX | frameshift_deleti  on | exon8 | c.2267delA | p.K756fs | . | . |
| CTC2 | X | 76939297 | CT | C | 1 | NM_138270 | ATRX | frameshift_deleti  on | exon8 | c.1336delA | p.R446fs | . | . |
| CTC2 | X | 79980466 | TC | T | 0.286 | NM_153252 | BRWD3 | frameshift_deleti  on | exon15 | c.1486delG | p.D496fs | . | . |
| CTC2 | X | 83576915 | AT | A | 1 | NM_001177478 | HDX | frameshift_deleti  on | exon10 | c.1880delA | p.N627fs | . | . |
| CTC2 | X | 85403920 | TC | T | 1 | NM_001139514 | DACH2 | frameshift_deleti  on | exon1 | c.297delC | p.L99fs | . | . |
| CTC2 | X | 100125715 | AT | A | 0.339 | NM_001271815 | NOX1 | frameshift_deleti  on | exon2 | c.133delA | p.I45fs | . | . |
| CTC2 | X | 100746636 | TG | T | 0.373 | NM_001256155 | ARMCX4 | frameshift_deleti  on | exon2 | c.3061delG | p.G1021fs | . | . |
| CTC2 | X | 100808018 | GA | G | 0.556 | NM_016608 | ARMCX1 | frameshift_deleti  on | exon4 | c.106delA | p.K36fs | . | . |
| CTC2 | X | 102334792 | GT | G | 0.306 | NM_022052 | NXF3 | frameshift_deleti  on | exon13 | c.1058delA | p.Y353fs | . | . |
| CTC2 | X | 102842137 | GT | G | 0.687 | NM_001006935 | TCEAL4 | frameshift_deleti  on | exon3 | c.535delT | p.F179fs | . | . |
| CTC2 | X | 103267783 | C | CA | 0.438 | NM_001002916 | H2BFWT | frameshift_inserti  on | exon1 | c.449dupT | p.M150fs | . | . |
| CTC2 | X | 104984666 | GT | G | 0.354 | NM_017416 | IL1RAPL2 | frameshift_deleti  on | exon8 | c.1031delT | p.V344fs | . | . |
| CTC2 | X | 105190420 | A | AT | 0.5 | NM_198465 | NRK | frameshift_inserti  on | exon26 | c.4318dupT | p.E1439fs | . | . |
| CTC2 | X | 105278274 | AT | A | 0.541 | NM_000354 | SERPINA7 | frameshift_deleti  on | exon4 | c.995delA | p.N332fs | . | . |
| CTC2 | X | 106396684 | CT | C | 0.417 | NM_017681 | NUP62CL | frameshift_deleti  on | exon6 | c.394delA | p.R132fs | . | . |
| CTC2 | X | 106841056 | TGAG | T | 0.867 | NM_032428 | FRMPD3 | nonframeshift_de  letion | exon15 | c.2047_2049del | p.683_683del | . | . |
| CTC2 | X | 106841056 | TGAGGA  G | T | 0.867 | NM_032428 | FRMPD3 | nonframeshift_de  letion | exon15 | c.2047_2052del | p.683_684del | . | . |
| CTC2 | X | 107169331 | C | CT | 0.636 | NM_012216 | MID2 | frameshift_inserti  on | exon9 | c.1606dupT | p.P535fs | . | . |
| CTC2 | X | 107334741 | AC | A | 1 | NM_002814 | PSMD10 | frameshift_deleti  on | exon1 | c.9delG | p.G3fs | . | . |
| CTC2 | X | 107834834 | TC | T | 1 | NM_000495 | COL4A5 | frameshift_deleti  on | exon21 | c.1384delC | p.P462fs | . | . |
| CTC2 | X | 107869478 | GT | G | 0.227 | NM_000495 | COL4A5 | frameshift_deleti  on | exon36 | c.3146delT | p.V1049fs | . | . |
| CTC2 | X | 111095578 | A | AT | 0.143 | NM_012471 | TRPC5 | frameshift_inserti  on | exon5 | c.1324dupA | p.M442fs | . | . |
| CTC2 | X | 118971766 | GT | G | 0.625 | NM_023010 | UPF3B | frameshift_deleti  on | exon9 | c.1216delA | p.T406fs | . | . |
| CTC2 | X | 119500432 | CAGA | C | 0.186 | NM_001142447 | ATP1B4 | nonframeshift_de  letion | exon2 | c.117_119del | p.39_40del | . | . |
| CTC2 | X | 124456783 | AG | A | 0.25 | NM_001195272 | LOC1001295  20 | frameshift_deleti  on | exon1 | c.2816delG | p.R939fs | . | . |
| CTC2 | X | 124456805 | A | AC | 0.312 | NM_001195272 | LOC1001295  20 | frameshift_inserti  on | exon1 | c.2837_2838ins  C | p.Q946fs | . | . |
| CTC2 | X | 127186119 | CT | C | 0.714 | NM_138289 | ACTRT1 | frameshift_deleti  on | exon1 | c.66delA | p.K22fs | . | . |
| CTC2 | X | 128581193 | TG | T | 0.545 | NM_001282875 | SMARCA1 | frameshift_deleti  on | exon24 | c.3122delC | p.A1041fs | . | . |
| CTC2 | X | 128650420 | CA | C | 0.529 | NM_001282874 | SMARCA1 | frameshift_deleti  on | exon3 | c.315delT | p.F105fs | . | . |
| CTC2 | X | 130419105 | TA | T | 0.595 | NM_001170963 | IGSF1 | frameshift_deleti  on | exon5 | c.714delT | p.G238fs | . | . |
| CTC2 | X | 131212252 | G | GT | 0.279 | NM_194277 | FRMD7 | frameshift_inserti  on | exon12 | c.1792dupA | p.T598fs | . | . |
| CTC2 | X | 131762525 | TG | T | 1 | NM_147175 | HS6ST2 | frameshift_deleti  on | exon4 | c.1543delC | p.Q515fs | . | . |

| CTC2 | X | 132439922 | TG | T | 0.367 | NM_001448 | GPC4 | frameshift_deleti  on | exon6 | c.1032delC | p.P344fs | . | . |
| --- | --- | --- | --- | --- | --- | --- | --- | --- | --- | --- | --- | --- | --- |
| CTC2 | X | 134991899 | AC | A | 0.316 | NM_018666 | SAGE1 | frameshift_deleti  on | exon14 | c.1685delC | p.T562fs | . | . |
| CTC2 | X | 135430731 | CA | C | 0.877 | NM_153834 | GPR112 | frameshift_deleti  on | exon6 | c.4867delA | p.K1623fs | . | . |
| CTC2 | X | 135618386 | GA | G | 0.357 | NM_016267 | VGLL1 | frameshift_deleti  on | exon2 | c.208delA | p.K70fs | . | . |
| CTC2 | X | 135631071 | CG | C | 0.226 | NM_016267 | VGLL1 | frameshift_deleti  on | exon3 | c.539delG | p.R180fs | . | . |
| CTC2 | X | 135829704 | TA | T | 0.486 | NM_004840 | ARHGEF6 | frameshift_deleti  on | exon3 | c.296delT | p.V99fs | . | . |
| CTC2 | X | 135961585 | A | ATT | 0.5 | NM_001164803 | RBMX | frameshift_inserti  on | exon2 | c.1_2insAA | p.M1fs | . | . |
| CTC2 | X | 149680724 | CG | C | 0.586 | NM_001177465 | MAMLD1 | frameshift_deleti  on | exon5 | c.2379delG | p.S793fs | . | . |
| CTC2 | X | 149783169 | TA | T | 0.3 | NM_000252 | MTM1 | frameshift_deleti  on | exon5 | c.340delA | p.K114fs | . | . |
| CTC2 | X | 149867750 | GA | G | 0.929 | NM_003828 | MTMR1 | frameshift_deleti  on | exon2 | c.230delA | p.E77fs | . | . |
| CTC2 | X | 149896179 | TC | T | 0.391 | NM_003828 | MTMR1 | frameshift_deleti  on | exon5 | c.448delC | p.P150fs | . | . |
| CTC2 | X | 149924312 | GA | G | 0.348 | NM_003828 | MTMR1 | frameshift_deleti  on | exon14 | c.1809delA | p.R603fs | . | . |
| CTC2 | X | 151908811 | CT | C | 0.793 | NM_001102576 | CSAG1 | frameshift_deleti  on | exon3 | c.51delT | p.A17fs | . | . |
| CTC2 | X | 152037567 | CA | C | 0.387 | NM_015922 | NSDHL | frameshift_deleti  on | exon8 | c.1030delA | p.K344fs | . | . |
| CTC2 | X | 152960077 | CT | C | 0.357 | NM_001142805 | SLC6A8 | frameshift_deleti  on | exon11 | c.1556delT | p.L519fs | . | . |
| CTC2 | X | 153171558 | TG | T | 0.676 | NM_000054 | AVPR2 | frameshift_deleti  on | exon2 | c.599delG | p.W200fs | . | . |
| CTC2 | X | 153537738 | AG | A | 0.419 | NM_001145934 | TKTL1 | frameshift_deleti  on | exon2 | c.127delG | p.G43fs | . | . |
| CTC2 | X | 153631701 | AC | A | 0.164 | NM_001009934 | DNASE1L1 | frameshift_deleti  on | exon6 | c.433delG | p.V145fs | . | . |
| CTC2 | X | 153940903 | CA | C | 0.297 | NM_001081573 | GAB3 | frameshift_deleti  on | exon4 | c.669delT | p.F223fs | . | . |
| CTC2 | X | 154159318 | CCTG | C | 0.25 | NM_000132 | F8 | nonframeshift_de  letion | exon14 | c.2744_2746del | p.915_916del | . | . |
| CTC2 | X | 154293777 | CA | C | 0.667 | NM_001018025 | MTCP1 | frameshift_deleti  on | exon4 | c.311delT | p.L104fs | . | . |
| CTC2 | X | 154294223 | GT | G | 0.609 | NM_001018025 | MTCP1 | frameshift_deleti  on | exon2 | c.62delA | p.Y21fs | . | . |
| CTC2 | Y | 15478189 | TC | T | 1 | NM_001258265 | UTY | frameshift_deleti  on | exon6 | c.574delG | p.E192fs | . | . |
| CTC3 | 1 | 985956 | GGGC | G | 1 | NM_198576 | AGRN | nonframeshift_de  letion | exon29 | c.5127_5129del | p.1709_1710de  l | . | . |
| CTC3 | 1 | 3551607 | A | AT | 0.556 | NM_017818 | WRAP73 | frameshift_inserti  on | exon8 | c.769dupA | p.M257fs | . | . |
| CTC3 | 1 | 3645919 | TG | T | 0.158 | NM_001126240 | TP73 | frameshift_deleti  on | exon8 | c.957delG | p.L319fs | . | . |
| CTC3 | 1 | 3645985 | GGCA | G | 0.685 | NM_001126240 | TP73 | nonframeshift_de  letion | exon8 | c.1023_1025del | p.341_342del | . | . |
| CTC3 | 1 | 3754057 | GA | G | 0.429 | NM_014704 | CEP104 | frameshift_deleti  on | exon9 | c.917delT | p.L306fs | . | . |
| CTC3 | 1 | 7724027 | CG | C | 1 | NM_015215 | CAMTA1 | frameshift_deleti  on | exon9 | c.1421delG | p.R474fs | . | . |
| CTC3 | 1 | 7731015 | C | CA | 0.2 | NM_015215 | CAMTA1 | frameshift_inserti  on | exon10 | c.2698dupA | p.S899fs | . | . |
| CTC3 | 1 | 8421515 | C | CA | 0.8 | NM_001042682 | RERE | frameshift_inserti  on | exon8 | c.389_390insT | p.E130fs | . | . |
| CTC3 | 1 | 9101828 | G | GA | 0.833 | NM_001135585 | SLC2A5 | frameshift_inserti  on | exon5 | c.586dupT | p.S196fs | . | . |
| CTC3 | 1 | 10473278 | C | CA | 0.317 | NM_002631 | PGD | frameshift_inserti  on | exon8 | c.814_815insA | p.L272fs | . | . |
| CTC3 | 1 | 11174380 | AT | A | 0.381 | NM_004958 | MTOR | frameshift_deleti  on | exon53 | c.7294delA | p.M2432fs | . | . |
| CTC3 | 1 | 12090118 | G | GT | 0.75 | NM_021933 | MIIP | frameshift_inserti  on | exon8 | c.879_880insT | p.P293fs | . | . |
| CTC3 | 1 | 12318009 | AT | A | 0.415 | NM_015378 | VPS13D | frameshift_deleti  on | exon10 | c.960delT | p.Y320fs | . | . |
| CTC3 | 1 | 17326952 | CA | C | 0.706 | NM_001141973 | ATP13A2 | frameshift_deleti  on | exon9 | c.767delT | p.L256fs | . | . |
| CTC3 | 1 | 19183996 | GA | G | 0.191 | NM_152232 | TAS1R2 | frameshift_deleti  on | exon2 | c.311delT | p.I104fs | . | . |
| CTC3 | 1 | 19215938 | TC | T | 1 | NM_003748 | ALDH4A1 | frameshift_deleti  on | exon3 | c.166delG | p.D56fs | . | . |
| CTC3 | 1 | 19453097 | GC | G | 0.745 | NM_020765 | UBR4 | frameshift_deleti  on | exon64 | c.9280delG | p.A3094fs | . | . |
| CTC3 | 1 | 19480406 | AG | A | 0.281 | NM_020765 | UBR4 | frameshift_deleti  on | exon45 | c.6485delC | p.P2162fs | . | . |
| CTC3 | 1 | 20474872 | CA | C | 0.182 | NM_022819 | PLA2G2F | frameshift_deleti  on | exon5 | c.615delA | p.P205fs | . | . |
| CTC3 | 1 | 20501629 | TG | T | 0.178 | NM_001105572 | PLA2G2C | frameshift_deleti  on | exon1 | c.58delC | p.H20fs | . | . |
| CTC3 | 1 | 22202437 | AC | A | 0.6 | NM_005529 | HSPG2 | frameshift_deleti  on | exon24 | c.3101delG | p.G1034fs | . | . |
| CTC3 | 1 | 22816768 | CA | C | 0.944 | NM_014870 | ZBTB40 | frameshift_deleti  on | exon2 | c.328delA | p.K110fs | . | . |
| CTC3 | 1 | 23758301 | TGG | T | 1 | NM_001143778 | ASAP3 | frameshift_deleti  on | exon22 | c.2405_2406del | p.P802fs | . | . |
| CTC3 | 1 | 25718510 | CG | C | 0.517 | NM_020485 | RHCE | frameshift_deleti  on | exon4 | c.608delC | p.T203fs | . | . |
| CTC3 | 1 | 26150180 | GT | G | 0.403 | NM_001099625 | MTFR1L | frameshift_deleti  on | exon3 | c.71delT | p.V24fs | . | . |
| CTC3 | 1 | 26156162 | TG | T | 1 | NM_001099625 | MTFR1L | frameshift_deleti  on | exon6 | c.615delG | p.L205fs | . | . |
| CTC3 | 1 | 26356976 | GC | G | 0.974 | NM_004455 | EXTL1 | frameshift_deleti  on | exon4 | c.992delC | p.A331fs | . | . |
| CTC3 | 1 | 26366312 | GCC | G | 0.391 | NM_032513 | SLC30A2 | frameshift_deleti  on | exon6 | c.780_781del | p.W260fs | . | . |
| CTC3 | 1 | 26603035 | CA | C | 0.723 | NM_001281518 | CEP85 | frameshift_deleti  on | exon6 | c.209delA | p.Q70fs | . | . |
| CTC3 | 1 | 26608853 | GCCGGG  A | G | 0.833 | NM_001077262 | UBXN11 | nonframeshift_de  letion | exon11 | c.1134_1139del | p.378_380del | . | . |
| CTC3 | 1 | 27426930 | TG | T | 0.65 | NM_003047 | SLC9A1 | frameshift_deleti  on | exon12 | c.2315delC | p.P772fs | . | . |
| CTC3 | 1 | 31819635 | AC | A | 1 | NM_001282567 | ZCCHC17 | frameshift_deleti  on | exon5 | c.395delC | p.T132fs | . | . |
| CTC3 | 1 | 31905889 | A | ACAG | 1 | NM_001199037 | SERINC2 | nonframeshift_in  sertion | exon9 | c.1101_1102ins  CAG | p.T367delinsTQ | . | . |
| CTC3 | 1 | 32936346 | AT | A | 1 | NM_001145720 | ZBTB8B | frameshift_deleti  on | exon2 | c.122delT | p.I41fs | . | . |

| CTC3 | 1 | 33583673 | G | GA | 0.8 | NM_052998 | ADC | frameshift_inserti  on | exon11 | c.1201dupA | p.G400fs | . | . |
| --- | --- | --- | --- | --- | --- | --- | --- | --- | --- | --- | --- | --- | --- |
| CTC3 | 1 | 33958832 | TTC | T | 0.169 | NM_145238 | ZSCAN20 | frameshift_deleti  on | exon7 | c.1491_1492del | p.I497fs | . | . |
| CTC3 | 1 | 35222976 | GT | G | 0.2 | NM_005268 | GJB5 | frameshift_deleti  on | exon2 | c.46delT | p.Y16fs | . | . |
| CTC3 | 1 | 36055613 | GC | G | 1 | NM_178548 | TFAP2E | frameshift_deleti  on | exon5 | c.869delC | p.A290fs | . | . |
| CTC3 | 1 | 38048519 | C | CA | 0.429 | NM_013285 | GNL2 | frameshift_inserti  on | exon7 | c.654dupT | p.V219fs | . | . |
| CTC3 | 1 | 39853909 | T | TG | 0.444 | NM_012090 | MACF1 | frameshift_inserti  on | exon52 | c.9209_9210ins  G | p.F3070fs | . | . |
| CTC3 | 1 | 43675652 | CTT | C | 0.8 | NM_001167965 | WDR65 | frameshift_deleti  on | exon11 | c.1995_1996del | p.P665fs | . | . |
| CTC3 | 1 | 43803571 | CA | C | 0.562 | NM_005373 | MPL | frameshift_deleti  on | exon1 | c.53delA | p.Q18fs | . | . |
| CTC3 | 1 | 43903271 | TA | T | 0.455 | NM_015284 | SZT2 | frameshift_deleti  on | exon44 | c.6126delA | p.L2042fs | . | . |
| CTC3 | 1 | 45525912 | AG | A | 0.278 | NM_020883 | ZSWIM5 | frameshift_deleti  on | exon3 | c.959delC | p.P320fs | . | . |
| CTC3 | 1 | 45793281 | GC | G | 0.816 | NM_032756 | HPDL | frameshift_deleti  on | exon1 | c.462delC | p.G154fs | . | . |
| CTC3 | 1 | 46159042 | GC | G | 0.8 | NM_016486 | TMEM69 | frameshift_deleti  on | exon3 | c.210delC | p.S70fs | . | . |
| CTC3 | 1 | 47279167 | AG | A | 0.176 | NM_000779 | CYP4B1 | frameshift_deleti  on | exon5 | c.510delG | p.E170fs | . | . |
| CTC3 | 1 | 47610223 | TG | T | 1 | NM_001010969 | CYP4A22 | frameshift_deleti  on | exon8 | c.900delG | p.M300fs | . | . |
| CTC3 | 1 | 51756192 | CT | C | 0.769 | NM_001080494 | TTC39A | frameshift_deleti  on | exon15 | c.1374delA | p.K458fs | . | . |
| CTC3 | 1 | 52248266 | CAA | C | 0.857 | NM_148905 | OSBPL9 | frameshift_deleti  on | exon12 | c.931_932del | p.K311fs | . | . |
| CTC3 | 1 | 53250581 | GA | G | 0.824 | NM_024646 | ZYG11B | frameshift_deleti  on | exon5 | c.1126delA | p.N376fs | . | . |
| CTC3 | 1 | 53558380 | GC | G | 0.42 | NM_001287597 | SLC1A7 | frameshift_deleti  on | exon6 | c.660delG | p.K220fs | . | . |
| CTC3 | 1 | 53675888 | CA | C | 0.406 | NM_000098 | CPT2 | frameshift_deleti  on | exon4 | c.543delA | p.A181fs | . | . |
| CTC3 | 1 | 53712692 | A | AG | 0.3 | NM_017522 | LRP8 | stoploss | exon16 | c.2100dupC | p.X701delinsL | . | . |
| CTC3 | 1 | 55474102 | AG | A | 0.347 | NM_057176 | BSND | frameshift_deleti on | exon4 | c.765delG | p.E255fs | . | . |
| CTC3 | 1 | 55566555 | AT | A | 0.968 | NM_015306 | USP24 | frameshift_deleti  on | exon44 | c.5227delA | p.M1743fs | . | . |
| CTC3 | 1 | 60359454 | TA | T | 0.293 | NM_000775 | CYP2J2 | frameshift_deleti  on | exon9 | c.1377delT | p.F459fs | . | . |
| CTC3 | 1 | 62550296 | TG | T | 0.7 | NM_176877 | INADL | frameshift_deleti  on | exon33 | c.4354delG | p.V1452fs | . | . |
| CTC3 | 1 | 62734089 | C | CA | 0.933 | NM_181712 | KANK4 | frameshift_inserti  on | exon5 | c.2100_2101ins  T | p.G701fs | . | . |
| CTC3 | 1 | 64643777 | AT | A | 0.645 | NM_005012 | ROR1 | frameshift_deleti  on | exon9 | c.2054delT | p.I685fs | . | . |
| CTC3 | 1 | 65858397 | G | GT | 0.25 | NM_001256864 | DNAJC6 | frameshift_inserti  on | exon12 | c.1753dupT | p.L584fs | . | . |
| CTC3 | 1 | 67236143 | GA | G | 0.75 | NM_152665 | TCTEX1D1 | frameshift_deleti  on | exon3 | c.194delA | p.E65fs | . | . |
| CTC3 | 1 | 67591585 | C | CT | 0.25 | NM_001276351 | C1orf141 | frameshift_inserti  on | exon4 | c.82dupA | p.R28fs | . | . |
| CTC3 | 1 | 67833666 | TG | T | 0.2 | NM_001258215 | IL12RB2 | frameshift_deleti  on | exon10 | c.1418delG | p.W473fs | . | . |
| CTC3 | 1 | 70501843 | GA | G | 0.308 | NM_020794 | LRRC7 | frameshift_deleti  on | exon17 | c.1922delA | p.E641fs | . | . |
| CTC3 | 1 | 74819807 | GA | G | 0.72 | NM_015978 | TNNI3K | frameshift_deleti  on | exon11 | c.1172delA | p.E391fs | . | . |
| CTC3 | 1 | 75037856 | CCCCT | C | 0.789 | NM_001002912 | C1orf173 | frameshift_deleti  on | exon14 | c.3534_3537del | p.G1178fs | . | . |
| CTC3 | 1 | 75037857 | CCCT | C | 1 | NM_001002912 | C1orf173 | nonframeshift_de  letion | exon14 | c.3534_3536del | p.1178_1179de  l | . | . |
| CTC3 | 1 | 77516033 | GC | G | 0.778 | NM_030965 | ST6GALNAC  5 | frameshift_deleti  on | exon4 | c.763delC | p.P255fs | . | . |
| CTC3 | 1 | 85403517 | CGAA | C | 0.8 | NM_153259 | MCOLN2 | nonframeshift_de  letion | exon11 | c.1253_1255del | p.418_419del | . | . |
| CTC3 | 1 | 86818549 | AT | A | 0.471 | NM_001184766 | ODF2L | stopgain | exon15 | c.1639delA | p.M547X | . | . |
| CTC3 | 1 | 86959985 | AG | A | 0.962 | NM_001285 | CLCA1 | frameshift_deleti on | exon11 | c.1797delG | p.K599fs | . | . |
| CTC3 | 1 | 92546172 | TG | T | 0.513 | NM_183242 | BTBD8 | frameshift_deleti  on | exon1 | c.45delG | p.L15fs | . | . |
| CTC3 | 1 | 93307372 | CA | C | 0.941 | NM_000969 | RPL5 | frameshift_deleti  on | exon8 | c.845delA | p.Q282fs | . | . |
| CTC3 | 1 | 93621965 | GA | G | 0.833 | NM_001167830 | TMED5 | frameshift_deleti  on | exon3 | c.362delT | p.F121fs | . | . |
| CTC3 | 1 | 94652140 | TC | T | 0.204 | NM_004815 | ARHGAP29 | frameshift_deleti  on | exon16 | c.1694delG | p.R565fs | . | . |
| CTC3 | 1 | 95639393 | AT | A | 0.625 | NM_001199679 | TMEM56 | frameshift_deleti  on | exon6 | c.422delT | p.I141fs | . | . |
| CTC3 | 1 | 102271671 | A | AG | 0.466 | NM_001288821 | OLFM3 | frameshift_inserti  on | exon5 | c.719dupC | p.A240fs | . | . |
| CTC3 | 1 | 103400052 | CA | C | 0.318 | NM_080630 | COL11A1 | frameshift_deleti  on | exon44 | c.3204delT | p.D1068fs | . | . |
| CTC3 | 1 | 109533921 | AG | A | 0.438 | NM_001142550 | WDR47 | frameshift_deleti  on | exon9 | c.1745delC | p.P582fs | . | . |
| CTC3 | 1 | 109801271 | CGG | C | 1 | NM_001408 | CELSR2 | frameshift_deleti  on | exon2 | c.3529_3530del | p.G1177fs | . | . |
| CTC3 | 1 | 110128868 | AC | A | 0.461 | NM_006496 | GNAI3 | frameshift_deleti  on | exon6 | c.622delC | p.R208fs | . | . |
| CTC3 | 1 | 110169045 | GT | G | 0.353 | NM_203404 | AMPD2 | frameshift_deleti  on | exon3 | c.333delT | p.C111fs | . | . |
| CTC3 | 1 | 110559306 | A | AT | 0.193 | NM_001242673 | AHCYL1 | frameshift_inserti  on | exon9 | c.777dupT | p.D259fs | . | . |
| CTC3 | 1 | 110587464 | AG | A | 0.306 | NM_001270768 | STRIP1 | frameshift_deleti  on | exon11 | c.1005delG | p.E335fs | . | . |
| CTC3 | 1 | 111957501 | CTCACA  GACTGA TGACTC | C | 1 | NM_002557 | OVGP1 | nonframeshift_de letion | exon11 | c.1598_1621del | p.533_541del | . | . |
| CTC3 | 1 | 113266683 | CG | C | 0.33 | NM_001004440 | FAM19A3 | frameshift_deleti  on | exon4 | c.383delG | p.R128fs | . | . |
| CTC3 | 1 | 114453982 | C | CA | 0.356 | NM_022836 | DCLRE1B | frameshift_inserti  on | exon4 | c.769dupA | p.P256fs | . | . |
| CTC3 | 1 | 114940473 | CT | C | 0.271 | NM_033020 | TRIM33 | frameshift_deleti  on | exon19 | c.3129delA | p.S1043fs | . | . |
| CTC3 | 1 | 117527385 | CG | C | 0.475 | NM_020440 | PTGFRN | frameshift_deleti  on | exon8 | c.2252delG | p.R751fs | . | . |
| CTC3 | 1 | 117559944 | GA | G | 0.25 | NM_001256106 | CD101 | frameshift_deleti  on | exon5 | c.1462delA | p.K488fs | . | . |

| CTC3 | 1 | 119964595 | CA | C | 1 | NM_000198 | HSD3B2 | frameshift_deleti  on | exon4 | c.472delA | p.K158fs | . | . |
| --- | --- | --- | --- | --- | --- | --- | --- | --- | --- | --- | --- | --- | --- |
| CTC3 | 1 | 120345711 | TC | T | 0.569 | NM_001159353 | REG4 | frameshift_deleti  on | exon3 | c.144delG | p.L48fs | . | . |
| CTC3 | 1 | 120928595 | G | GC | 0.221 | NM_001244910 | FCGR1B | frameshift_inserti  on | exon5 | c.582dupG | p.L195fs | . | . |
| CTC3 | 1 | 144886321 | CT | C | 0.556 | NM_001198834 | PDE4DIP | frameshift_deleti  on | exon23 | c.2912delA | p.K971fs | . | . |
| CTC3 | 1 | 147084783 | A | AG | 0.881 | NM_004326 | BCL9 | frameshift_inserti  on | exon5 | c.156dupG | p.Q52fs | . | . |
| CTC3 | 1 | 150443345 | GC | G | 0.476 | NM_015203 | RPRD2 | frameshift_deleti  on | exon11 | c.1922delC | p.A641fs | . | . |
| CTC3 | 1 | 150940181 | AC | A | 0.188 | NM_022075 | CERS2 | frameshift_deleti  on | exon6 | c.477delG | p.W159fs | . | . |
| CTC3 | 1 | 150940336 | TA | T | 0.233 | NM_022075 | CERS2 | frameshift_deleti  on | exon5 | c.427delT | p.Y143fs | . | . |
| CTC3 | 1 | 151259097 | TG | T | 0.333 | NM_020832 | ZNF687 | frameshift_deleti  on | exon2 | c.331delG | p.G111fs | . | . |
| CTC3 | 1 | 151274738 | TG | T | 0.4 | NM_001198775 | PI4KB | frameshift_deleti  on | exon5 | c.560delC | p.T187fs | . | . |
| CTC3 | 1 | 151774724 | CA | C | 0.667 | NM_001004432 | LINGO4 | frameshift_deleti  on | exon2 | c.456delT | p.F152fs | . | . |
| CTC3 | 1 | 152276299 | TG | T | 0.297 | NM_002016 | FLG | frameshift_deleti  on | exon3 | c.11062delC | p.H3688fs | . | . |
| CTC3 | 1 | 152732960 | G | GA | 0.286 | NM_001025231 | KPRP | frameshift_inserti  on | exon2 | c.896_897insA | p.R299fs | . | . |
| CTC3 | 1 | 152800040 | CA | C | 0.227 | NM_178348 | LCE1A | frameshift_deleti  on | exon1 | c.93delA | p.P31fs | . | . |
| CTC3 | 1 | 153615784 | C | CG | 0.333 | NM_001206612 | CHTOP | frameshift_inserti  on | exon5 | c.488_489insG | p.P163fs | . | . |
| CTC3 | 1 | 153730215 | TG | T | 0.525 | NM_023015 | INTS3 | frameshift_deleti  on | exon10 | c.1126delG | p.G376fs | . | . |
| CTC3 | 1 | 153925781 | CT | C | 0.4 | NM_181715 | CRTC2 | frameshift_deleti  on | exon6 | c.567delA | p.P189fs | . | . |
| CTC3 | 1 | 154145427 | CT | C | 0.821 | NM_001278188 | TPM3 | frameshift_deleti  on | exon3 | c.213delA | p.E71fs | . | . |
| CTC3 | 1 | 154794601 | CA | C | 0.568 | NM_001204087 | KCNN3 | frameshift_deleti  on | exon2 | c.992delT | p.L331fs | . | . |
| CTC3 | 1 | 154918177 | TC | T | 0.296 | NM_020524 | PBXIP1 | frameshift_deleti  on | exon10 | c.1972delG | p.D658fs | . | . |
| CTC3 | 1 | 155025125 | TGG | T | 0.212 | NM_001261464 | ADAM15 | frameshift_deleti  on | exon2 | c.89_90del | p.W30fs | . | . |
| CTC3 | 1 | 155311768 | CA | C | 1 | NM_018489 | ASH1L | frameshift_deleti  on | exon25 | c.8418delT | p.F2806fs | . | . |
| CTC3 | 1 | 156047285 | CA | C | 0.234 | NM_001093725 | MEX3A | frameshift_deleti  on | exon2 | c.642delT | p.F214fs | . | . |
| CTC3 | 1 | 156128583 | AC | A | 0.439 | NM_001193302 | SEMA4A | frameshift_deleti  on | exon4 | c.141delC | p.D47fs | . | . |
| CTC3 | 1 | 156777063 | TG | T | 0.538 | NM_001161443 | SH2D2A | frameshift_deleti  on | exon7 | c.992delC | p.P331fs | . | . |
| CTC3 | 1 | 157103884 | CA | C | 0.604 | NM_005240 | ETV3 | frameshift_deleti  on | exon4 | c.419delT | p.L140fs | . | . |
| CTC3 | 1 | 157803223 | GCC | G | 0.235 | NM_005894 | CD5L | frameshift_deleti  on | exon5 | c.796_797del | p.G266fs | . | . |
| CTC3 | 1 | 158813813 | GCC | G | 0.368 | NM_002432 | MNDA | frameshift_deleti  on | exon4 | c.472_473del | p.P158fs | . | . |
| CTC3 | 1 | 158914715 | AC | A | 0.629 | NM_152501 | PYHIN1 | frameshift_deleti  on | exon7 | c.1243delC | p.P415fs | . | . |
| CTC3 | 1 | 159776399 | GA | G | 0.357 | NM_001004310 | FCRL6 | frameshift_deleti  on | exon2 | c.46delA | p.K16fs | . | . |
| CTC3 | 1 | 159846430 | C | CT | 0.429 | NM_012337 | CCDC19 | frameshift_inserti  on | exon10 | c.1267dupA | p.S423fs | . | . |
| CTC3 | 1 | 159898319 | TG | T | 0.63 | NM_001135050 | IGSF9 | frameshift_deleti  on | exon19 | c.2858delC | p.P953fs | . | . |
| CTC3 | 1 | 160141456 | AC | A | 0.6 | NM_144699 | ATP1A4 | frameshift_deleti  on | exon12 | c.1764delC | p.N588fs | . | . |
| CTC3 | 1 | 160183216 | TA | T | 0.353 | NM_003768 | PEA15 | frameshift_deleti  on | exon4 | c.334delA | p.I112fs | . | . |
| CTC3 | 1 | 160970051 | GA | G | 0.316 | NM_016946 | F11R | frameshift_deleti  on | exon5 | c.475delT | p.S159fs | . | . |
| CTC3 | 1 | 161044056 | GCAC | G | 0.25 | NM_030916 | PVRL4 | nonframeshift_de  letion | exon6 | c.1105_1107del | p.369_369del | . | . |
| CTC3 | 1 | 161089968 | G | GA | 0.333 | NM_001185093 | NIT1 | frameshift_inserti  on | exon5 | c.619dupA | p.E206fs | . | . |
| CTC3 | 1 | 161183509 | GT | G | 0.333 | NM_001166159 | NDUFS2 | frameshift_deleti  on | exon12 | c.1284delT | p.G428fs | . | . |
| CTC3 | 1 | 161202733 | G | GA | 0.134 | NM_001077470 | NR1I3 | frameshift_inserti  on | exon4 | c.324dupT | p.P109fs | . | . |
| CTC3 | 1 | 161976228 | TTG | T | 0.25 | NM_015441 | OLFML2B | frameshift_deleti  on | exon4 | c.580_581del | p.Q194fs | . | . |
| CTC3 | 1 | 163138064 | GT | G | 0.325 | NM_001254749 | RGS5 | frameshift_deleti  on | exon2 | c.138delA | p.K46fs | . | . |
| CTC3 | 1 | 164761845 | C | CT | 0.237 | NM_001204961 | PBX1 | frameshift_inserti  on | exon3 | c.380_381insT | p.A127fs | . | . |
| CTC3 | 1 | 167097204 | GA | G | 0.875 | NM_001080426 | DUSP27 | frameshift_deleti  on | exon5 | c.2837delA | p.E946fs | . | . |
| CTC3 | 1 | 167874249 | TC | T | 1 | NM_018417 | ADCY10 | frameshift_deleti  on | exon2 | c.129delG | p.L43fs | . | . |
| CTC3 | 1 | 170508434 | CA | C | 1 | NM_001146039 | GORAB | frameshift_deleti  on | exon2 | c.221delA | p.Q74fs | . | . |
| CTC3 | 1 | 171083418 | GC | G | 0.18 | NM_001002294 | FMO3 | frameshift_deleti  on | exon7 | c.1100delC | p.A367fs | . | . |
| CTC3 | 1 | 173472410 | CT | C | 0.917 | NM_178527 | SLC9C2 | frameshift_deleti  on | exon27 | c.3365delA | p.K1122fs | . | . |
| CTC3 | 1 | 173883957 | G | GA | 0.5 | NM_000488 | SERPINC1 | frameshift_inserti  on | exon2 | c.141dupT | p.P48fs | . | . |
| CTC3 | 1 | 179033582 | C | CT | 0.261 | NM_014864 | FAM20B | frameshift_inserti  on | exon6 | c.889_890insT | p.Q297fs | . | . |
| CTC3 | 1 | 182369331 | AC | A | 0.878 | NM_172000 | TEDDM1 | frameshift_deleti  on | exon1 | c.289delG | p.V97fs | . | . |
| CTC3 | 1 | 183909770 | CA | C | 0.303 | NM_015101 | COLGALT2 | frameshift_deleti  on | exon11 | c.1548delT | p.F516fs | . | . |
| CTC3 | 1 | 184853820 | TCAG | T | 0.172 | NM_052966 | FAM129A | nonframeshift_de  letion | exon5 | c.545_547del | p.182_183del | . | . |
| CTC3 | 1 | 184863330 | GC | G | 1 | NM_052966 | FAM129A | frameshift_deleti  on | exon3 | c.196delG | p.A66fs | . | . |
| CTC3 | 1 | 185069010 | AG | A | 0.179 | NM_007212 | RNF2 | frameshift_deleti  on | exon6 | c.826delG | p.G276fs | . | . |
| CTC3 | 1 | 197071522 | TC | T | 0.8 | NM_018136 | ASPM | frameshift_deleti  on | exon18 | c.6858delG | p.K2286fs | . | . |
| CTC3 | 1 | 197111783 | AT | A | 0.556 | NM_001206846 | ASPM | frameshift_deleti  on | exon3 | c.1598delA | p.N533fs | . | . |
| CTC3 | 1 | 200730149 | G | GC | 0.286 | NM_203459 | CAMSAP2 | frameshift_inserti  on | exon2 | c.323dupC | p.A108fs | . | . |
| CTC3 | 1 | 201181559 | A | AT | 0.4 | NM_001164586 | IGFN1 | frameshift_inserti  on | exon12 | c.7539dupT | p.D2513fs | . | . |

| CTC3 | 1 | 201184769 | TC | T | 0.684 | NM_001164586 | IGFN1 | frameshift_deleti  on | exon15 | c.9099delC | p.L3033fs | . | . |
| --- | --- | --- | --- | --- | --- | --- | --- | --- | --- | --- | --- | --- | --- |
| CTC3 | 1 | 201190710 | TC | T | 0.236 | NM_001164586 | IGFN1 | frameshift_deleti  on | exon19 | c.10038delC | p.L3346fs | . | . |
| CTC3 | 1 | 203054753 | TG | T | 0.492 | NM_002479 | MYOG | frameshift_deleti  on | exon1 | c.336delC | p.P112fs | . | . |
| CTC3 | 1 | 203741222 | AT | A | 0.368 | NM_001136190 | LAX1 | frameshift_deleti  on | exon4 | c.290delT | p.I97fs | . | . |
| CTC3 | 1 | 204095109 | TG | T | 0.273 | NM_005686 | SOX13 | stopgain | exon14 | c.1717delG | p.V573X | . | . |
| CTC3 | 1 | 204419026 | AC | A | 0.282 | NM_002646 | PIK3C2B | frameshift_deleti on | exon14 | c.2185delG | p.V729fs | . | . |
| CTC3 | 1 | 204438277 | AC | A | 1 | NM_002646 | PIK3C2B | frameshift_deleti  on | exon3 | c.653delG | p.G218fs | . | . |
| CTC3 | 1 | 204588990 | CG | C | 0.258 | NM_201630 | LRRN2 | frameshift_deleti  on | exon2 | c.130delC | p.R44fs | . | . |
| CTC3 | 1 | 205779451 | AG | A | 0.8 | NM_173854 | SLC41A1 | frameshift_deleti  on | exon2 | c.118delC | p.L40fs | . | . |
| CTC3 | 1 | 206325338 | G | GA | 0.333 | NM_001910 | CTSE | frameshift_inserti  on | exon5 | c.564dupA | p.G188fs | . | . |
| CTC3 | 1 | 206821253 | TA | T | 0.531 | NM_003582 | DYRK3 | frameshift_deleti  on | exon3 | c.711delA | p.L237fs | . | . |
| CTC3 | 1 | 207200913 | TC | T | 0.667 | NM_023938 | C1orf116 | frameshift_deleti  on | exon2 | c.30delG | p.G10fs | . | . |
| CTC3 | 1 | 209849207 | G | GA | 0.8 | NM_015714 | G0S2 | frameshift_inserti  on | exon2 | c.179dupA | p.E60fs | . | . |
| CTC3 | 1 | 209964021 | C | CT | 0.275 | NM_001206696 | IRF6 | frameshift_inserti  on | exon5 | c.593dupA | p.K198fs | . | . |
| CTC3 | 1 | 210761427 | GC | G | 0.727 | NM_001170564 | HHAT | stopgain | exon8 | c.819delC | p.C273X | . | . |
| CTC3 | 1 | 213415381 | TA | T | 1 | NM_001287220 | RPS6KC1 | frameshift_deleti on | exon9 | c.1168delA | p.K390fs | . | . |
| CTC3 | 1 | 214826306 | AT | A | 0.301 | NM_016343 | CENPF | frameshift_deleti  on | exon16 | c.8297delT | p.I2766fs | . | . |
| CTC3 | 1 | 215844397 | AT | A | 0.278 | NM_206933 | USH2A | frameshift_deleti  on | exon64 | c.14049delA | p.K4683fs | . | . |
| CTC3 | 1 | 220824050 | GA | G | 0.571 | NM_001286128 | MARK1 | frameshift_deleti  on | exon13 | c.1494delA | p.G498fs | . | . |
| CTC3 | 1 | 222802169 | CA | C | 0.786 | NM_198551 | MIA3 | frameshift_deleti  on | exon4 | c.1608delA | p.S536fs | . | . |
| CTC3 | 1 | 225268393 | G | GA | 0.219 | NM_001373 | DNAH14 | frameshift_inserti  on | exon19 | c.3080dupA | p.E1027fs | . | . |
| CTC3 | 1 | 225394771 | TA | T | 0.667 | NM_001373 | DNAH14 | frameshift_deleti  on | exon40 | c.6223delA | p.K2075fs | . | . |
| CTC3 | 1 | 227946724 | TG | T | 0.267 | NM_053052 | SNAP47 | frameshift_deleti  on | exon3 | c.662delG | p.W221fs | . | . |
| CTC3 | 1 | 228294497 | CT | C | 0.382 | NM_181441 | MRPL55 | frameshift_deleti  on | exon4 | c.350delA | p.E117fs | . | . |
| CTC3 | 1 | 228560067 | TG | T | 0.938 | NM_001098623 | OBSCN | frameshift_deleti  on | exon94 | c.21589delG | p.G7197fs | . | . |
| CTC3 | 1 | 228602513 | CG | C | 0.5 | NM_001024940 | TRIM17 | frameshift_deleti  on | exon2 | c.260delC | p.A87fs | . | . |
| CTC3 | 1 | 231473433 | C | CG | 0.778 | NM_175876 | EXOC8 | frameshift_inserti  on | exon1 | c.58_59insC | p.G20fs | . | . |
| CTC3 | 1 | 240370120 | GA | G | 0.163 | NM_020066 | FMN2 | frameshift_deleti  on | exon5 | c.2009delA | p.E670fs | . | . |
| CTC3 | 1 | 241842850 | G | GA | 0.353 | NM_144625 | WDR64 | frameshift_inserti  on | exon5 | c.548dupA | p.E183fs | . | . |
| CTC3 | 1 | 243328915 | GT | G | 0.265 | NM_001042404 | CEP170 | frameshift_deleti  on | exon12 | c.2052delA | p.K684fs | . | . |
| CTC3 | 1 | 244580965 | CA | C | 1 | NM_001126 | ADSS | frameshift_deleti  on | exon10 | c.1034delT | p.L345fs | . | . |
| CTC3 | 1 | 245018777 | GT | G | 0.571 | NM_004501 | HNRNPU | frameshift_deleti  on | exon12 | c.2243delA | p.N748fs | . | . |
| CTC3 | 1 | 247014412 | AT | A | 0.876 | NM_015446 | AHCTF1 | frameshift_deleti  on | exon33 | c.4922delA | p.N1641fs | . | . |
| CTC3 | 1 | 247014415 | TTC | T | 0.158 | NM_015446 | AHCTF1 | frameshift_deleti  on | exon33 | c.4918_4919del | p.E1640fs | . | . |
| CTC3 | 1 | 247202791 | ACTC | A | 0.5 | NM_001204220 | ZNF670 | nonframeshift_de  letion | exon2 | c.49_51del | p.17_17del | . | . |
| CTC3 | 1 | 247263957 | TA | T | 1 | NM_001142572 | ZNF669 | frameshift_deleti  on | exon4 | c.855delT | p.F285fs | . | . |
| CTC3 | 1 | 247607417 | AC | A | 0.407 | NM_183395 | NLRP3 | frameshift_deleti  on | exon5 | c.2472delC | p.H824fs | . | . |
| CTC3 | 1 | 247737671 | TG | T | 0.529 | NM_001281838 | GCSAML | frameshift_deleti  on | exon5 | c.300delG | p.V100fs | . | . |
| CTC3 | 1 | 248112753 | GT | G | 0.7 | NM_001001963 | OR2L8 | frameshift_deleti  on | exon1 | c.595delT | p.F199fs | . | . |
| CTC3 | 1 | 248525328 | C | CTA | 0.727 | NM_001004696 | OR2T4 | frameshift_inserti  on | exon1 | c.446_447insTA | p.T149fs | . | . |
| CTC3 | 1 | 248685874 | TG | T | 0.271 | NM_001013355 | OR2G6 | frameshift_deleti  on | exon1 | c.928delG | p.A310fs | . | . |
| CTC3 | 2 | 1667395 | GGT | G | 1 | NM_012293 | PXDN | frameshift_deleti  on | exon12 | c.1547_1548del | p.H516fs | . | . |
| CTC3 | 2 | 7036033 | AAT | A | 0.667 | NM_080657 | RSAD2 | frameshift_deleti  on | exon6 | c.1047_1048del | p.K349fs | . | . |
| CTC3 | 2 | 7036034 | AT | A | 0.957 | NM_080657 | RSAD2 | frameshift_deleti  on | exon6 | c.1048delT | p.Y350fs | . | . |
| CTC3 | 2 | 8930177 | GT | G | 0.365 | NM_020738 | KIDINS220 | frameshift_deleti  on | exon14 | c.1453delA | p.T485fs | . | . |
| CTC3 | 2 | 10051679 | CA | C | 0.654 | NM_005680 | TAF1B | frameshift_deleti  on | exon11 | c.1174delA | p.K392fs | . | . |
| CTC3 | 2 | 11853882 | A | AT | 0.455 | NM_001261428 | LPIN1 | frameshift_inserti  on | exon2 | c.83dupT | p.I28fs | . | . |
| CTC3 | 2 | 21231740 | TC | T | 0.312 | NM_000384 | APOB | frameshift_deleti  on | exon26 | c.7999delG | p.E2667fs | . | . |
| CTC3 | 2 | 21232132 | CA | C | 0.333 | NM_000384 | APOB | frameshift_deleti  on | exon26 | c.7607delT | p.L2536fs | . | . |
| CTC3 | 2 | 21232246 | C | CA | 0.263 | NM_000384 | APOB | frameshift_inserti  on | exon26 | c.7493_7494ins  T | p.E2498fs | . | . |
| CTC3 | 2 | 24107669 | AT | A | 1 | NM_001242338 | ATAD2B | frameshift_deleti  on | exon6 | c.749delA | p.N250fs | . | . |
| CTC3 | 2 | 25470992 | TG | T | 1 | NM_153759 | DNMT3A | frameshift_deleti  on | exon3 | c.201delC | p.P67fs | . | . |
| CTC3 | 2 | 26653669 | GA | G | 0.5 | NM_145038 | DRC1 | frameshift_deleti  on | exon6 | c.695delA | p.E232fs | . | . |
| CTC3 | 2 | 26696126 | TC | T | 1 | NM_194322 | OTOF | frameshift_deleti  on | exon11 | c.1536delG | p.L512fs | . | . |
| CTC3 | 2 | 27306780 | G | GT | 0.464 | NM_007046 | EMILIN1 | frameshift_inserti  on | exon4 | c.2341_2342ins  T | p.G781fs | . | . |
| CTC3 | 2 | 27445407 | AG | A | 0.367 | NM_004341 | CAD | frameshift_deleti  on | exon5 | c.517delG | p.G173fs | . | . |
| CTC3 | 2 | 27481644 | AC | A | 0.464 | NM_003459 | SLC30A3 | frameshift_deleti  on | exon2 | c.253delG | p.V85fs | . | . |
| CTC3 | 2 | 27481717 | GA | G | 0.211 | NM_003459 | SLC30A3 | frameshift_deleti  on | exon2 | c.180delT | p.L60fs | . | . |

| CTC3 | 2 | 27799580 | AT | A | 1 | NM_032266 | C2orf16 | frameshift_deleti  on | exon1 | c.142delT | p.F48fs | . | . |
| --- | --- | --- | --- | --- | --- | --- | --- | --- | --- | --- | --- | --- | --- |
| CTC3 | 2 | 32822944 | AG | A | 0.5 | NM_016252 | BIRC6 | frameshift_deleti  on | exon69 | c.13740delG | p.Q4580fs | . | . |
| CTC3 | 2 | 37036014 | CTGG | C | 0.497 | NM_001177970 | VIT | nonframeshift_de  letion | exon13 | c.1682_1684del | p.561_562del | . | . |
| CTC3 | 2 | 38545778 | C | CA | 0.778 | NM_001135673 | ATL2 | frameshift_inserti  on | exon4 | c.501dupT | p.A168fs | . | . |
| CTC3 | 2 | 45645565 | AT | A | 0.591 | NM_018079 | SRBD1 | frameshift_deleti  on | exon18 | c.2271delA | p.K757fs | . | . |
| CTC3 | 2 | 46707847 | GA | G | 0.5 | NM_001145051 | TMEM247 | frameshift_deleti  on | exon2 | c.422delA | p.E141fs | . | . |
| CTC3 | 2 | 47604157 | C | CT | 0.822 | NM_002354 | EPCAM | frameshift_inserti  on | exon5 | c.497dupT | p.L166fs | . | . |
| CTC3 | 2 | 54040118 | CGG | C | 1 | NM_001127398 | ERLEC1 | frameshift_deleti  on | exon10 | c.973_974del | p.G325fs | . | . |
| CTC3 | 2 | 60679784 | TG | T | 0.923 | NM_018014 | BCL11A | frameshift_deleti  on | exon5 | c.2247delC | p.P749fs | . | . |
| CTC3 | 2 | 65243758 | CT | C | 0.706 | NM_003038 | SLC1A4 | frameshift_deleti  on | exon5 | c.986delT | p.L329fs | . | . |
| CTC3 | 2 | 66665034 | GC | G | 1 | NM_002398 | MEIS1 | frameshift_deleti  on | exon2 | c.179delC | p.A60fs | . | . |
| CTC3 | 2 | 69741753 | CTGT | C | 1 | NM_014911 | AAK1 | nonframeshift_de  letion | exon13 | c.1623_1625del | p.541_542del | . | . |
| CTC3 | 2 | 71058200 | TA | T | 0.208 | NM_015717 | CD207 | frameshift_deleti  on | exon6 | c.967delT | p.Y323fs | . | . |
| CTC3 | 2 | 71304748 | TG | T | 0.868 | NM_017567 | NAGK | frameshift_deleti  on | exon9 | c.965delG | p.W322fs | . | . |
| CTC3 | 2 | 71650301 | TA | T | 0.796 | NM_001014972 | ZNF638 | frameshift_deleti  on | exon22 | c.3658delA | p.K1220fs | . | . |
| CTC3 | 2 | 73303274 | C | CT | 0.385 | NM_015470 | RAB11FIP5 | frameshift_inserti  on | exon4 | c.1604_1605ins  A | p.V535fs | . | . |
| CTC3 | 2 | 73315336 | TTGG | T | 0.25 | NM_015470 | RAB11FIP5 | nonframeshift_de  letion | exon3 | c.1407_1409del | p.469_470del | . | . |
| CTC3 | 2 | 74042265 | GC | G | 0.868 | NM_001080474 | C2orf78 | frameshift_deleti  on | exon3 | c.916delC | p.P306fs | . | . |
| CTC3 | 2 | 74315096 | AC | A | 0.231 | NM_001287491 | TET3 | frameshift_deleti  on | exon6 | c.2820delC | p.Y940fs | . | . |
| CTC3 | 2 | 74710232 | AT | A | 0.225 | NM_022492 | TTC31 | frameshift_deleti  on | exon1 | c.11delT | p.I4fs | . | . |
| CTC3 | 2 | 86327243 | TC | T | 0.691 | NM_015425 | POLR1A | frameshift_deleti  on | exon2 | c.129delG | p.G43fs | . | . |
| CTC3 | 2 | 86406728 | AT | A | 0.648 | NM_001100169 | IMMT | frameshift_deleti  on | exon3 | c.136delA | p.I46fs | . | . |
| CTC3 | 2 | 86832169 | CAT | C | 0.2 | NM_005667 | RNF103 | frameshift_deleti  on | exon4 | c.853_854del | p.M285fs | . | . |
| CTC3 | 2 | 88828715 | CA | C | 0.4 | NM_152670 | TEX37 | frameshift_deleti  on | exon4 | c.267delA | p.S89fs | . | . |
| CTC3 | 2 | 95541452 | C | CA | 0.4 | NM_001286559 | TEKT4 | frameshift_inserti  on | exon5 | c.511dupA | p.P170fs | . | . |
| CTC3 | 2 | 97329191 | CA | C | 0.429 | NM_001113382 | FER1L5 | frameshift_deleti  on | exon13 | c.1037delA | p.Q346fs | . | . |
| CTC3 | 2 | 97351085 | TG | T | 0.211 | NM_001113382 | FER1L5 | frameshift_deleti  on | exon20 | c.1800delG | p.L600fs | . | . |
| CTC3 | 2 | 97361603 | AC | A | 1 | NM_001113382 | FER1L5 | frameshift_deleti  on | exon35 | c.4101delC | p.D1367fs | . | . |
| CTC3 | 2 | 97427261 | GC | G | 1 | NM_020184 | CNNM4 | frameshift_deleti  on | exon1 | c.526delC | p.P176fs | . | . |
| CTC3 | 2 | 99291628 | TA | T | 0.264 | NM_012214 | MGAT4A | frameshift_deleti  on | exon4 | c.272delT | p.L91fs | . | . |
| CTC3 | 2 | 100022949 | CA | C | 0.326 | NM_001037872 | REV1 | frameshift_deleti  on | exon16 | c.2448delT | p.V816fs | . | . |
| CTC3 | 2 | 100218010 | CGCT | C | 0.231 | NM_001025108 | AFF3 | nonframeshift_de  letion | exon13 | c.1330_1332del | p.444_444del | . | . |
| CTC3 | 2 | 102782617 | CA | C | 0.875 | NM_000877 | IL1R1 | frameshift_deleti  on | exon6 | c.532delA | p.K178fs | . | . |
| CTC3 | 2 | 103380836 | AC | A | 0.462 | NM_144632 | TMEM182 | frameshift_deleti  on | exon3 | c.282delC | p.Y94fs | . | . |
| CTC3 | 2 | 107040803 | GT | G | 0.545 | NM_001144013 | RGPD3 | frameshift_deleti  on | exon20 | c.3619delA | p.T1207fs | . | . |
| CTC3 | 2 | 109367807 | GA | G | 0.99 | NM_006267 | RANBP2 | frameshift_deleti  on | exon10 | c.1362delA | p.R454fs | . | . |
| CTC3 | 2 | 109381418 | GT | G | 1 | NM_006267 | RANBP2 | frameshift_deleti  on | exon20 | c.4424delT | p.V1475fs | . | . |
| CTC3 | 2 | 109492593 | G | GA | 0.192 | NM_144978 | CCDC138 | frameshift_inserti  on | exon15 | c.1883dupA | p.E628fs | . | . |
| CTC3 | 2 | 110107194 | GC | G | 0.937 | NM_001099289 | SH3RF3 | frameshift_deleti  on | exon9 | c.2283delC | p.G761fs | . | . |
| CTC3 | 2 | 111551658 | GA | G | 0.357 | NM_001142807 | ACOXL | frameshift_deleti  on | exon5 | c.253delA | p.K85fs | . | . |
| CTC3 | 2 | 112944986 | TG | T | 0.853 | NM_001128165 | FBLN7 | frameshift_deleti  on | exon7 | c.1086delG | p.L362fs | . | . |
| CTC3 | 2 | 112945059 | C | CT | 0.667 | NM_001128165 | FBLN7 | frameshift_inserti  on | exon7 | c.1159dupT | p.I386fs | . | . |
| CTC3 | 2 | 113300001 | TTCCGG  CGTGTA CCGAGA | T | 1 | NM_001282772 | POLR1B | frameshift_deleti on | exon2 | c.45_69del | p.L15fs | . | . |
| CTC3 | 2 | 118735577 | AT | A | 0.419 | NM_019044 | CCDC93 | frameshift_deleti  on | exon8 | c.649delA | p.M217fs | . | . |
| CTC3 | 2 | 119752028 | AC | A | 0.353 | NM_006770 | MARCO | frameshift_deleti  on | exon17 | c.1496delC | p.T499fs | . | . |
| CTC3 | 2 | 121708877 | CCG | C | 0.25 | NM_005270 | GLI2 | frameshift_deleti  on | exon3 | c.314_315del | p.P105fs | . | . |
| CTC3 | 2 | 128046943 | CTCT | C | 0.109 | NM_000122 | ERCC3 | nonframeshift_de  letion | exon6 | c.789_791del | p.263_264del | . | . |
| CTC3 | 2 | 128253633 | TA | T | 1 | NM_017969 | IWS1 | frameshift_deleti  on | exon7 | c.1656delT | p.F552fs | . | . |
| CTC3 | 2 | 128477084 | GC | G | 0.538 | NM_018383 | WDR33 | frameshift_deleti  on | exon16 | c.2514delG | p.G838fs | . | . |
| CTC3 | 2 | 131890527 | C | CT | 0.232 | NM_001100623 | PLEKHB2 | frameshift_inserti  on | exon6 | c.387dupT | p.P129fs | . | . |
| CTC3 | 2 | 133531390 | T | TA | 0.377 | NM_207481 | NCKAP5 | frameshift_inserti  on | exon14 | c.1169dupT | p.I390fs | . | . |
| CTC3 | 2 | 135107418 | AT | A | 0.586 | NM_002410 | MGAT5 | frameshift_deleti  on | exon9 | c.1156delT | p.F386fs | . | . |
| CTC3 | 2 | 136570193 | GA | G | 1 | NM_002299 | LCT | frameshift_deleti  on | exon7 | c.2040delT | p.F680fs | . | . |
| CTC3 | 2 | 141751565 | GC | G | 0.385 | NM_018557 | LRP1B | frameshift_deleti  on | exon16 | c.2642delG | p.C881fs | . | . |
| CTC3 | 2 | 149247728 | TA | T | 0.324 | NM_018328 | MBD5 | frameshift_deleti  on | exon12 | c.3829delA | p.K1277fs | . | . |
| CTC3 | 2 | 153476066 | G | GCCA | 0.5 | NM_052905 | FMNL2 | nonframeshift_in  sertion | exon15 | c.1671_1672ins  CCA | p.P557delinsPP | . | . |

| CTC3 | 2 | 153533155 | AGCT | A | 0.14 | NM_017892 | PRPF40A | nonframeshift_de  letion | exon10 | c.792_794del | p.264_265del | . | . |
| --- | --- | --- | --- | --- | --- | --- | --- | --- | --- | --- | --- | --- | --- |
| CTC3 | 2 | 155555845 | GCC | G | 1 | NM_001260508 | KCNJ3 | frameshift_deleti  on | exon1 | c.559_560del | p.P187fs | . | . |
| CTC3 | 2 | 160628498 | A | AT | 0.269 | NM_001198763 | CD302 | frameshift_inserti  on | exon5 | c.388dupA | p.I130fs | . | . |
| CTC3 | 2 | 164467115 | AT | A | 1 | NM_018086 | FIGN | frameshift_deleti  on | exon3 | c.1226delA | p.N409fs | . | . |
| CTC3 | 2 | 166535995 | AC | A | 0.32 | NM_024969 | CSRNP3 | frameshift_deleti  on | exon5 | c.1491delC | p.N497fs | . | . |
| CTC3 | 2 | 167162316 | GAC | G | 0.16 | NM_002977 | SCN9A | frameshift_deleti  on | exon5 | c.580_581del | p.V194fs | . | . |
| CTC3 | 2 | 167162317 | AC | A | 0.537 | NM_002977 | SCN9A | frameshift_deleti  on | exon5 | c.580delG | p.V194fs | . | . |
| CTC3 | 2 | 168101608 | GT | G | 0.375 | NM_001199144 | XIRP2 | frameshift_deleti  on | exon7 | c.3041delT | p.V1014fs | . | . |
| CTC3 | 2 | 168103225 | GA | G | 0.182 | NM_001199144 | XIRP2 | frameshift_deleti  on | exon7 | c.4658delA | p.E1553fs | . | . |
| CTC3 | 2 | 169038510 | TG | T | 0.642 | NM_013233 | STK39 | frameshift_deleti  on | exon2 | c.298delC | p.Q100fs | . | . |
| CTC3 | 2 | 170097843 | CA | C | 0.214 | NM_004525 | LRP2 | frameshift_deleti  on | exon25 | c.3699delT | p.D1233fs | . | . |
| CTC3 | 2 | 171225809 | GA | G | 0.5 | NM_001083615 | MYO3B | frameshift_deleti  on | exon9 | c.894delA | p.G298fs | . | . |
| CTC3 | 2 | 171716226 | CA | C | 0.452 | NM_000817 | GAD1 | frameshift_deleti  on | exon17 | c.1620delA | p.P540fs | . | . |
| CTC3 | 2 | 175436540 | G | GT | 0.362 | NM_001077269 | WIPF1 | frameshift_inserti  on | exon5 | c.992_993insA | p.T331fs | . | . |
| CTC3 | 2 | 178416556 | CA | C | 0.645 | NM_152517 | TTC30B | frameshift_deleti  on | exon1 | c.935delT | p.L312fs | . | . |
| CTC3 | 2 | 178482475 | GA | G | 0.625 | NM_152275 | TTC30A | frameshift_deleti  on | exon1 | c.954delT | p.F318fs | . | . |
| CTC3 | 2 | 179251857 | TG | T | 0.649 | NM_001201481 | OSBPL6 | frameshift_deleti  on | exon19 | c.2055delG | p.M685fs | . | . |
| CTC3 | 2 | 179399173 | C | CT | 0.286 | NM_003319 | TTN | frameshift_inserti  on | exon186 | c.74973dupA | p.E24992fs | . | . |
| CTC3 | 2 | 179438850 | AT | A | 0.289 | NM_003319 | TTN | frameshift_deleti  on | exon154 | c.44813delA | p.N14938fs | . | . |
| CTC3 | 2 | 179463308 | TG | T | 1 | NM_003319 | TTN | frameshift_deleti  on | exon120 | c.29840delC | p.P9947fs | . | . |
| CTC3 | 2 | 179516423 | C | CT | 1 | NM_133378 | TTN | frameshift_inserti  on | exon160 | c.32130_32131i  nsA | p.A10711fs | . | . |
| CTC3 | 2 | 179517402 | CT | C | 1 | NM_001267550 | TTN | frameshift_deleti  on | exon201 | c.39020delA | p.K13007fs | . | . |
| CTC3 | 2 | 179656816 | G | GT | 0.939 | NM_001256850 | TTN | frameshift_inserti  on | exon10 | c.1644dupA | p.Q549fs | . | . |
| CTC3 | 2 | 182339723 | TG | T | 0.55 | NM_000885 | ITGA4 | frameshift_deleti  on | exon3 | c.357delG | p.L119fs | . | . |
| CTC3 | 2 | 182412566 | TG | T | 0.5 | NM_001030312 | CERKL | frameshift_deleti  on | exon6 | c.802delC | p.Q268fs | . | . |
| CTC3 | 2 | 185802211 | C | CACA | 1 | NM_194250 | ZNF804A | nonframeshift_in  sertion | exon4 | c.2088_2089ins  ACA | p.N696delinsN  T | . | . |
| CTC3 | 2 | 185803347 | C | CG | 1 | NM_194250 | ZNF804A | frameshift_inserti  on | exon4 | c.3224_3225ins  G | p.P1075fs | . | . |
| CTC3 | 2 | 186666165 | CA | C | 1 | NM_173651 | FSIP2 | frameshift_deleti  on | exon17 | c.12400delA | p.K4134fs | . | . |
| CTC3 | 2 | 187370509 | AATG | A | 0.143 | NM_018471 | ZC3H15 | nonframeshift_de  letion | exon8 | c.908_910del | p.303_304del | . | . |
| CTC3 | 2 | 187521009 | C | CA | 1 | NM_001145000 | ITGAV | frameshift_inserti  on | exon15 | c.1493dupA | p.Q498fs | . | . |
| CTC3 | 2 | 187694588 | GT | G | 1 | NM_182521 | ZSWIM2 | frameshift_deleti  on | exon8 | c.960delA | p.K320fs | . | . |
| CTC3 | 2 | 188348851 | CA | C | 0.875 | NM_001032281 | TFPI | frameshift_deleti  on | exon6 | c.627delT | p.F209fs | . | . |
| CTC3 | 2 | 189931459 | TC | T | 0.333 | NM_000393 | COL5A2 | frameshift_deleti  on | exon22 | c.1450delG | p.E484fs | . | . |
| CTC3 | 2 | 190439978 | CA | C | 0.237 | NM_014585 | SLC40A1 | stopgain | exon3 | c.179delT | p.L60X | . | . |
| CTC3 | 2 | 191390028 | AT | A | 0.5 | NM_001142645 | TMEM194B | frameshift_deleti on | exon2 | c.173delA | p.N58fs | . | . |
| CTC3 | 2 | 192228594 | TA | T | 0.588 | NM_001130158 | MYO1B | frameshift_deleti  on | exon10 | c.907delA | p.K303fs | . | . |
| CTC3 | 2 | 197943514 | AT | A | 0.889 | NM_001195144 | ANKRD44 | frameshift_deleti  on | exon16 | c.1562delA | p.N521fs | . | . |
| CTC3 | 2 | 198281614 | CT | C | 0.286 | NM_012433 | SF3B1 | frameshift_deleti  on | exon6 | c.516delA | p.L172fs | . | . |
| CTC3 | 2 | 198288542 | TC | T | 0.444 | NM_001005526 | SF3B1 | frameshift_deleti  on | exon2 | c.184delG | p.E62fs | . | . |
| CTC3 | 2 | 200213558 | TG | T | 0.364 | NM_001172509 | SATB2 | frameshift_deleti  on | exon7 | c.1038delC | p.P346fs | . | . |
| CTC3 | 2 | 203054955 | GA | G | 1 | NM_001277372 | LOC1006528  24 | frameshift_deleti  on | exon19 | c.2656delA | p.K886fs | . | . |
| CTC3 | 2 | 204009765 | AT | A | 0.387 | NM_001114132 | NBEAL1 | frameshift_deleti  on | exon32 | c.5100delT | p.H1700fs | . | . |
| CTC3 | 2 | 207024735 | GCCT | G | 0.125 | NM_001959 | EEF1B2 | nonframeshift_de  letion | exon1 | c.33_35del | p.11_12del | . | . |
| CTC3 | 2 | 207620107 | CT | C | 0.357 | NM_001039845 | MDH1B | frameshift_deleti  on | exon5 | c.535delA | p.S179fs | . | . |
| CTC3 | 2 | 209216117 | CA | C | 0.469 | NM_015040 | PIKFYVE | frameshift_deleti  on | exon38 | c.5654delA | p.Q1885fs | . | . |
| CTC3 | 2 | 219254627 | TC | T | 1 | NM_000578 | SLC11A1 | frameshift_deleti  on | exon9 | c.831delC | p.I277fs | . | . |
| CTC3 | 2 | 219290524 | CA | C | 0.333 | NM_007127 | VIL1 | frameshift_deleti  on | exon4 | c.338delA | p.Q113fs | . | . |
| CTC3 | 2 | 220078020 | AG | A | 0.92 | NM_005689 | ABCB6 | frameshift_deleti  on | exon12 | c.1747delC | p.L583fs | . | . |
| CTC3 | 2 | 220087050 | CG | C | 0.254 | NM_024085 | ATG9A | frameshift_deleti  on | exon11 | c.1970delC | p.P657fs | . | . |
| CTC3 | 2 | 220366753 | CACT | C | 1 | NM_013335 | GMPPA | nonframeshift_de  letion | exon5 | c.424_426del | p.142_142del | . | . |
| CTC3 | 2 | 220371033 | GC | G | 0.322 | NM_013335 | GMPPA | frameshift_deleti  on | exon12 | c.1052delC | p.A351fs | . | . |
| CTC3 | 2 | 220413941 | GA | G | 0.636 | NM_001005209 | TMEM198 | frameshift_deleti  on | exon4 | c.811delA | p.K271fs | . | . |
| CTC3 | 2 | 220414071 | TC | T | 1 | NM_001005209 | TMEM198 | frameshift_deleti  on | exon4 | c.941delC | p.S314fs | . | . |
| CTC3 | 2 | 223065952 | GA | G | 1 | NM_181460 | PAX3 | frameshift_deleti  on | exon9 | c.1211delT | p.F404fs | . | . |
| CTC3 | 2 | 227875182 | CT | C | 0.229 | NM_000092 | COL4A4 | frameshift_deleti  on | exon46 | c.4368delA | p.K1456fs | . | . |
| CTC3 | 2 | 227906929 | A | AT | 0.774 | NM_000092 | COL4A4 | frameshift_inserti  on | exon37 | c.3439dupA | p.M1147fs | . | . |
| CTC3 | 2 | 227946863 | G | GC | 0.868 | NM_000092 | COL4A4 | frameshift_inserti  on | exon23 | c.1663dupG | p.A555fs | . | . |

| CTC3 | 2 | 231120187 | G | GA | 0.4 | NM_001278453 | SP140 | frameshift_inserti  on | exon9 | c.839dupA | p.E280fs | . | . |
| --- | --- | --- | --- | --- | --- | --- | --- | --- | --- | --- | --- | --- | --- |
| CTC3 | 2 | 231155238 | GT | G | 0.571 | NM_001278453 | SP140 | frameshift_deleti  on | exon16 | c.1443delT | p.G481fs | . | . |
| CTC3 | 2 | 231174667 | GC | G | 0.299 | NM_001278453 | SP140 | frameshift_deleti  on | exon20 | c.1746delC | p.C582fs | . | . |
| CTC3 | 2 | 231339123 | GAA | G | 0.5 | NM_001080391 | SP100 | frameshift_deleti  on | exon17 | c.1562_1563del | p.E521fs | . | . |
| CTC3 | 2 | 232325414 | TTCA | T | 1 | NM_005381 | NCL | nonframeshift_de  letion | exon4 | c.774_776del | p.258_259del | . | . |
| CTC3 | 2 | 233712226 | CACA | C | 1 | NM_001103148 | GIGYF2 | nonframeshift_de  letion | exon26 | c.3612_3614del | p.1204_1205de  l | . | . |
| CTC3 | 2 | 233735077 | TG | T | 1 | NM_206895 | C2orf82 | frameshift_deleti  on | exon1 | c.27delG | p.M9fs | . | . |
| CTC3 | 2 | 234389859 | TC | T | 0.661 | NM_018218 | USP40 | frameshift_deleti  on | exon30 | c.3621delG | p.G1207fs | . | . |
| CTC3 | 2 | 234652341 | CT | C | 0.688 | NM_001001394 | DNAJB3 | frameshift_deleti  on | exon1 | c.221delA | p.E74fs | . | . |
| CTC3 | 2 | 234681009 | AG | A | 0.769 | NM_000463 | UGT1A1 | frameshift_deleti  on | exon5 | c.1407delG | p.K469fs | . | . |
| CTC3 | 2 | 242815106 | GT | G | 1 | NM_173821 | CXXC11 | frameshift_deleti  on | exon2 | c.1400delT | p.V467fs | . | . |
| CTC3 | 2 | 242815108 | TA | T | 1 | NM_173821 | CXXC11 | frameshift_deleti  on | exon2 | c.1402delA | p.S468fs | . | . |
| CTC3 | 3 | 3195688 | TG | T | 0.75 | NM_001173482 | CRBN | frameshift_deleti  on | exon8 | c.903delC | p.G301fs | . | . |
| CTC3 | 3 | 10301862 | AT | A | 0.251 | NM_014760 | TATDN2 | frameshift_deleti  on | exon3 | c.457delT | p.F153fs | . | . |
| CTC3 | 3 | 11858740 | AG | A | 0.308 | NM_001284401 | TAMM41 | frameshift_deleti  on | exon5 | c.633delC | p.H211fs | . | . |
| CTC3 | 3 | 12626410 | GC | G | 0.231 | NM_002880 | RAF1 | frameshift_deleti  on | exon16 | c.1738delG | p.A580fs | . | . |
| CTC3 | 3 | 18391132 | CCTG | C | 0.189 | NM_001131010 | SATB1 | nonframeshift_de  letion | exon11 | c.1819_1821del | p.607_607del | . | . |
| CTC3 | 3 | 24006630 | C | CT | 0.537 | NM_001145425 | NR1D2 | frameshift_inserti  on | exon6 | c.1085dupT | p.L362fs | . | . |
| CTC3 | 3 | 32493953 | AC | A | 0.34 | NM_181472 | CMTM7 | frameshift_deleti  on | exon3 | c.404delC | p.T135fs | . | . |
| CTC3 | 3 | 33418820 | GC | G | 0.632 | NM_001171713 | FBXL2 | frameshift_deleti  on | exon10 | c.641delC | p.A214fs | . | . |
| CTC3 | 3 | 37090022 | TG | T | 0.333 | NM_001167619 | MLH1 | frameshift_deleti  on | exon16 | c.1189delG | p.G397fs | . | . |
| CTC3 | 3 | 37369176 | CG | C | 0.437 | NM_002078 | GOLGA4 | frameshift_deleti  on | exon14 | c.5800delG | p.G1934fs | . | . |
| CTC3 | 3 | 37818920 | A | AT | 0.333 | NM_002207 | ITGA9 | frameshift_inserti  on | exon24 | c.2579_2580ins  T | p.N860fs | . | . |
| CTC3 | 3 | 38232289 | TA | T | 0.36 | NM_005109 | OXSR1 | frameshift_deleti  on | exon3 | c.252delA | p.V84fs | . | . |
| CTC3 | 3 | 38592703 | GA | G | 0.269 | NM_001099405 | SCN5A | frameshift_deleti  on | exon27 | c.5105delT | p.I1702fs | . | . |
| CTC3 | 3 | 38627184 | CA | C | 0.361 | NM_000335 | SCN5A | frameshift_deleti  on | exon16 | c.2784delT | p.L928fs | . | . |
| CTC3 | 3 | 38938413 | A | AT | 0.5 | NM_014139 | SCN11A | frameshift_inserti  on | exon14 | c.2325dupA | p.C776fs | . | . |
| CTC3 | 3 | 38938602 | AG | A | 0.269 | NM_014139 | SCN11A | frameshift_deleti  on | exon14 | c.2136delC | p.I712fs | . | . |
| CTC3 | 3 | 39226563 | AG | A | 0.848 | NM_194293 | XIRP1 | frameshift_deleti  on | exon2 | c.4373delC | p.P1458fs | . | . |
| CTC3 | 3 | 39374716 | T | TA | 0.286 | NM_005201 | CCR8 | frameshift_inserti  on | exon2 | c.895dupA | p.V298fs | . | . |
| CTC3 | 3 | 40231925 | GC | G | 0.602 | NM_001284426 | MYRIP | frameshift_deleti  on | exon8 | c.1076delC | p.A359fs | . | . |
| CTC3 | 3 | 40528460 | TA | T | 0.37 | NM_001145083 | ZNF619 | frameshift_deleti  on | exon4 | c.328delA | p.K110fs | . | . |
| CTC3 | 3 | 42234589 | TA | T | 0.274 | NM_001265609 | TRAK1 | frameshift_deleti  on | exon7 | c.571delA | p.S191fs | . | . |
| CTC3 | 3 | 42680888 | GT | G | 0.286 | NM_005385 | NKTR | frameshift_deleti  on | exon13 | c.3693delT | p.G1231fs | . | . |
| CTC3 | 3 | 43759278 | C | CG | 0.425 | NM_016006 | ABHD5 | frameshift_inserti  on | exon6 | c.890dupG | p.R297fs | . | . |
| CTC3 | 3 | 44611727 | AG | A | 0.917 | NM_001288592 | ZKSCAN7 | frameshift_deleti  on | exon5 | c.673delG | p.V225fs | . | . |
| CTC3 | 3 | 44611934 | TG | T | 0.357 | NM_001288592 | ZKSCAN7 | frameshift_deleti  on | exon5 | c.880delG | p.G294fs | . | . |
| CTC3 | 3 | 44700769 | CT | C | 0.611 | NM_003420 | ZNF35 | frameshift_deleti  on | exon4 | c.915delT | p.T305fs | . | . |
| CTC3 | 3 | 44700777 | TG | T | 0.367 | NM_003420 | ZNF35 | frameshift_deleti  on | exon4 | c.923delG | p.C308fs | . | . |
| CTC3 | 3 | 44700846 | GA | G | 0.324 | NM_003420 | ZNF35 | frameshift_deleti  on | exon4 | c.992delA | p.E331fs | . | . |
| CTC3 | 3 | 44948568 | GA | G | 0.226 | NM_003241 | TGM4 | frameshift_deleti  on | exon10 | c.1204delA | p.N402fs | . | . |
| CTC3 | 3 | 45077232 | GC | G | 0.167 | NM_003278 | CLEC3B | frameshift_deleti  on | exon3 | c.426delC | p.G142fs | . | . |
| CTC3 | 3 | 46245501 | CA | C | 0.727 | NM_001295 | CCR1 | frameshift_deleti  on | exon2 | c.303delT | p.F101fs | . | . |
| CTC3 | 3 | 46712530 | C | CT | 0.216 | NM_182775 | ALS2CL | frameshift_inserti  on | exon11 | c.846dupA | p.E283fs | . | . |
| CTC3 | 3 | 46755838 | GC | G | 0.633 | NM_013270 | PRSS50 | frameshift_deleti  on | exon4 | c.623delG | p.G208fs | . | . |
| CTC3 | 3 | 47040211 | CA | C | 0.4 | NM_015175 | NBEAL2 | frameshift_deleti  on | exon23 | c.3227delA | p.Q1076fs | . | . |
| CTC3 | 3 | 47449069 | TA | T | 0.344 | NM_015466 | PTPN23 | frameshift_deleti  on | exon12 | c.999delA | p.V333fs | . | . |
| CTC3 | 3 | 48638193 | TG | T | 0.5 | NM_003365 | UQCRC1 | frameshift_deleti  on | exon9 | c.1046delC | p.A349fs | . | . |
| CTC3 | 3 | 49713937 | TC | T | 1 | NM_001640 | APEH | frameshift_deleti  on | exon7 | c.734delC | p.S245fs | . | . |
| CTC3 | 3 | 49947729 | TCA | T | 0.241 | NM_001142501 | MON1A | frameshift_deleti  on | exon3 | c.1005_1006del | p.R335fs | . | . |
| CTC3 | 3 | 50289142 | C | CA | 0.891 | NM_001282619 | GNAI2 | frameshift_inserti  on | exon2 | c.38dupA | p.Q13fs | . | . |
| CTC3 | 3 | 51198112 | A | AT | 0.625 | NM_004947 | DOCK3 | frameshift_inserti  on | exon12 | c.1017dupT | p.D339fs | . | . |
| CTC3 | 3 | 51696956 | CT | C | 0.311 | NM_015106 | RAD54L2 | frameshift_deleti  on | exon22 | c.3925delT | p.F1309fs | . | . |
| CTC3 | 3 | 52391687 | A | AT | 1 | NM_015512 | DNAH1 | frameshift_inserti  on | exon23 | c.3917dupT | p.I1306fs | . | . |
| CTC3 | 3 | 52392652 | TA | T | 0.408 | NM_015512 | DNAH1 | frameshift_deleti  on | exon25 | c.4166delA | p.Y1389fs | . | . |
| CTC3 | 3 | 55768828 | CT | C | 0.333 | NM_015576 | ERC2 | frameshift_deleti  on | exon14 | c.2676delA | p.K892fs | . | . |
| CTC3 | 3 | 57311822 | TC | T | 0.429 | NM_130387 | ASB14 | frameshift_deleti  on | exon2 | c.702delG | p.G234fs | . | . |
| CTC3 | 3 | 57432264 | CT | C | 1 | NM_178504 | DNAH12 | frameshift_deleti  on | exon26 | c.3869delA | p.K1290fs | . | . |

| CTC3 | 3 | 64507847 | CTA | C | 0.2 | NM_182920 | ADAMTS9 | frameshift_deleti  on | exon39 | c.5806_5807del | p.*1936fs | . | . |
| --- | --- | --- | --- | --- | --- | --- | --- | --- | --- | --- | --- | --- | --- |
| CTC3 | 3 | 64587662 | AT | A | 0.286 | NM_182920 | ADAMTS9 | frameshift_deleti  on | exon26 | c.3974delA | p.H1325fs | . | . |
| CTC3 | 3 | 66431212 | GT | G | 0.312 | NM_015541 | LRIG1 | frameshift_deleti  on | exon18 | c.2843delA | p.H948fs | . | . |
| CTC3 | 3 | 69988270 | CA | C | 0.253 | NM_000248 | MITF | frameshift_deleti  on | exon3 | c.284delA | p.Q95fs | . | . |
| CTC3 | 3 | 71021190 | CG | C | 1 | NM_001244810 | FOXP1 | frameshift_deleti  on | exon18 | c.1671delC | p.P557fs | . | . |
| CTC3 | 3 | 71021292 | TA | T | 1 | NM_001244810 | FOXP1 | frameshift_deleti  on | exon18 | c.1569delT | p.F523fs | . | . |
| CTC3 | 3 | 97466344 | TG | T | 0.256 | NM_001080448 | EPHA6 | frameshift_deleti  on | exon17 | c.3207delG | p.M1069fs | . | . |
| CTC3 | 3 | 98235928 | G | GC | 0.6 | NM_001040200 | CLDND1 | frameshift_inserti  on | exon3 | c.223_224insG | p.T75fs | . | . |
| CTC3 | 3 | 100947642 | C | CT | 0.234 | NM_016247 | IMPG2 | frameshift_inserti  on | exon18 | c.3711dupA | p.V1238fs | . | . |
| CTC3 | 3 | 101504468 | CT | C | 0.333 | NM_001134456 | NXPE3 | frameshift_deleti  on | exon4 | c.29delT | p.L10fs | . | . |
| CTC3 | 3 | 107097083 | AT | A | 0.421 | NM_032600 | CCDC54 | frameshift_deleti  on | exon1 | c.650delT | p.M217fs | . | . |
| CTC3 | 3 | 107491939 | CA | C | 0.409 | NM_001142568 | BBX | frameshift_deleti  on | exon11 | c.1372delA | p.K458fs | . | . |
| CTC3 | 3 | 111667907 | GC | G | 0.333 | NM_145753 | PHLDB2 | frameshift_deleti  on | exon9 | c.2488delC | p.P830fs | . | . |
| CTC3 | 3 | 111831866 | G | GA | 0.889 | NM_024616 | C3orf52 | frameshift_inserti  on | exon5 | c.524dupA | p.E175fs | . | . |
| CTC3 | 3 | 112349025 | AT | A | 0.8 | NM_199511 | CCDC80 | frameshift_deleti  on | exon3 | c.1969delA | p.I657fs | . | . |
| CTC3 | 3 | 113098206 | AG | A | 0.438 | NM_001164496 | WDR52 | frameshift_deleti  on | exon17 | c.2244delC | p.P748fs | . | . |
| CTC3 | 3 | 113098246 | TTCC | T | 0.5 | NM_001164496 | WDR52 | nonframeshift_de  letion | exon17 | c.2202_2204del | p.734_735del | . | . |
| CTC3 | 3 | 113376353 | TG | T | 0.333 | NM_001009899 | KIAA2018 | frameshift_deleti  on | exon7 | c.4175delC | p.P1392fs | . | . |
| CTC3 | 3 | 113737652 | T | TC | 1 | NM_020817 | KIAA1407 | frameshift_inserti  on | exon8 | c.1035dupG | p.K346fs | . | . |
| CTC3 | 3 | 113761613 | TG | T | 0.786 | NM_020817 | KIAA1407 | frameshift_deleti  on | exon4 | c.351delC | p.N117fs | . | . |
| CTC3 | 3 | 113761614 | GT | G | 0.769 | NM_020817 | KIAA1407 | frameshift_deleti  on | exon4 | c.350delA | p.N117fs | . | . |
| CTC3 | 3 | 118621681 | CT | C | 0.786 | NM_001015887 | IGSF11 | frameshift_deleti  on | exon7 | c.981delA | p.K327fs | . | . |
| CTC3 | 3 | 119132845 | TG | T | 0.22 | NM_020754 | ARHGAP31 | frameshift_deleti  on | exon12 | c.2070delG | p.L690fs | . | . |
| CTC3 | 3 | 119150909 | TA | T | 0.455 | NM_018266 | TMEM39A | frameshift_deleti  on | exon9 | c.1385delT | p.L462fs | . | . |
| CTC3 | 3 | 119347693 | CT | C | 0.478 | NM_001206961 | PLA1A | frameshift_deleti  on | exon9 | c.749delT | p.L250fs | . | . |
| CTC3 | 3 | 121351293 | T | TCTCA  GGCTC | 0.769 | NM_005335 | HCLS1 | nonframeshift_in  sertion | exon12 | c.1125_1126ins  CCCGAGCCTG | p.N376delinsPE  PEN | . | . |
| CTC3 | 3 | 121351315 | G | GGGCT  CAGGC | 0.958 | NM_005335 | HCLS1 | nonframeshift_in  sertion | exon12 | c.1103_1104ins  TGAGCCTGAG | p.P368delinsPE  PEP | . | . |
| CTC3 | 3 | 121351324 | C | CTCAG  GCTCA | 0.966 | NM_005335 | HCLS1 | nonframeshift_in  sertion | exon12 | c.1094_1095ins  TCCTGAGCCT | p.E365delinsDP  EPE | . | . |
| CTC3 | 3 | 121353230 | CA | C | 0.895 | NM_005335 | HCLS1 | frameshift_deleti  on | exon10 | c.726delT | p.F242fs | . | . |
| CTC3 | 3 | 123046534 | GC | G | 0.378 | NM_001199642 | ADCY5 | frameshift_deleti  on | exon7 | c.827delG | p.G276fs | . | . |
| CTC3 | 3 | 123419115 | AGTT | A | 1 | NM_053026 | MYLK | nonframeshift_de  letion | exon17 | c.2990_2992del | p.997_998del | . | . |
| CTC3 | 3 | 123419116 | GTTC | G | 1 | NM_053026 | MYLK | nonframeshift_de  letion | exon17 | c.2989_2991del | p.997_997del | . | . |
| CTC3 | 3 | 123419118 | TCTTC | T | 0.4 | NM_053026 | MYLK | frameshift_deleti  on | exon17 | c.2986_2989del | p.E996fs | . | . |
| CTC3 | 3 | 123983422 | TC | T | 0.595 | NM_001024660 | KALRN | frameshift_deleti  on | exon4 | c.336delC | p.L112fs | . | . |
| CTC3 | 3 | 124210221 | GC | G | 0.25 | NM_001024660 | KALRN | frameshift_deleti  on | exon31 | c.4634delC | p.A1545fs | . | . |
| CTC3 | 3 | 124646705 | A | AAAG | 1 | NM_033049 | MUC13 | nonframeshift_in  sertion | exon2 | c.184_185insCT  T | p.F62delinsSF | . | . |
| CTC3 | 3 | 126180558 | AT | A | 0.386 | NM_001040653 | ZXDC | frameshift_deleti  on | exon6 | c.1946delA | p.N649fs | . | . |
| CTC3 | 3 | 127842558 | C | CA | 0.692 | NM_003707 | RUVBL1 | stopgain | exon1 | c.9dupT | p.E4_E5delinsX | . | . |
| CTC3 | 3 | 128344747 | AT | A | 0.5 | NM_002950 | RPN1 | frameshift_deleti on | exon7 | c.1244delA | p.N415fs | . | . |
| CTC3 | 3 | 128526433 | TC | T | 0.311 | NM_004637 | RAB7A | frameshift_deleti  on | exon5 | c.448delC | p.P150fs | . | . |
| CTC3 | 3 | 129183541 | CA | C | 0.636 | NM_052990 | IFT122 | frameshift_deleti  on | exon5 | c.325delA | p.K109fs | . | . |
| CTC3 | 3 | 131624265 | TA | T | 1 | NM_001289112 | CPNE4 | frameshift_deleti  on | exon2 | c.76delT | p.Y26fs | . | . |
| CTC3 | 3 | 131756399 | ACCT | A | 0.667 | NM_001289112 | CPNE4 | frameshift_deleti  on | exon1 | c.52_53del | p.R18fs | . | . |
| CTC3 | 3 | 133336004 | CT | C | 0.733 | NM_007027 | TOPBP1 | frameshift_deleti  on | exon22 | c.3758delA | p.K1253fs | . | . |
| CTC3 | 3 | 133672553 | GC | G | 0.991 | NM_005630 | SLCO2A1 | frameshift_deleti  on | exon5 | c.677delG | p.G226fs | . | . |
| CTC3 | 3 | 134362184 | TG | T | 0.667 | NM_178554 | KY | frameshift_deleti  on | exon3 | c.235delC | p.Q79fs | . | . |
| CTC3 | 3 | 137781769 | A | AT | 0.28 | NM_173543 | DZIP1L | frameshift_inserti  on | exon16 | c.2192_2193ins  A | p.P731fs | . | . |
| CTC3 | 3 | 140407097 | CACA | C | 0.667 | NM_152616 | TRIM42 | nonframeshift_de  letion | exon3 | c.1574_1576del | p.525_526del | . | . |
| CTC3 | 3 | 141327449 | GT | G | 0.4 | NM_006506 | RASA2 | frameshift_deleti  on | exon21 | c.2136delT | p.S712fs | . | . |
| CTC3 | 3 | 141724318 | AG | A | 0.875 | NM_001178139 | TFDP2 | frameshift_deleti  on | exon4 | c.150delC | p.T50fs | . | . |
| CTC3 | 3 | 148894087 | T | TC | 0.659 | NM_000096 | CP | frameshift_inserti  on | exon18 | c.3130dupG | p.D1044fs | . | . |
| CTC3 | 3 | 149469196 | CT | C | 0.667 | NM_016094 | COMMD2 | frameshift_deleti  on | exon3 | c.221delA | p.K74fs | . | . |
| CTC3 | 3 | 150645821 | G | GA | 1 | NM_174878 | CLRN1 | frameshift_inserti  on | exon3 | c.600dupT | p.L201fs | . | . |
| CTC3 | 3 | 151046621 | AG | A | 0.229 | NM_176894 | P2RY13 | frameshift_deleti  on | exon2 | c.222delC | p.S74fs | . | . |
| CTC3 | 3 | 151046622 | GC | G | 0.429 | NM_176894 | P2RY13 | frameshift_deleti  on | exon2 | c.221delG | p.S74fs | . | . |
| CTC3 | 3 | 160119733 | GT | G | 1 | NM_005496 | SMC4 | frameshift_deleti  on | exon2 | c.171delT | p.S57fs | . | . |
| CTC3 | 3 | 160804227 | AC | A | 0.571 | NM_033168 | B3GALNT1 | frameshift_deleti  on | exon4 | c.315delG | p.K105fs | . | . |

| CTC3 | 3 | 164700112 | TC | T | 1 | NM_001041 | SI | frameshift_deleti  on | exon47 | c.5333delG | p.G1778fs | . | . |
| --- | --- | --- | --- | --- | --- | --- | --- | --- | --- | --- | --- | --- | --- |
| CTC3 | 3 | 164730809 | TG | T | 0.137 | NM_001041 | SI | frameshift_deleti  on | exon34 | c.4020delC | p.N1340fs | . | . |
| CTC3 | 3 | 164908483 | CA | C | 0.474 | NM_014926 | SLITRK3 | frameshift_deleti  on | exon2 | c.135delT | p.F45fs | . | . |
| CTC3 | 3 | 167218079 | TAA | T | 0.211 | NM_178824 | WDR49 | frameshift_deleti  on | exon14 | c.1835_1836del | p.F612fs | . | . |
| CTC3 | 3 | 169644807 | GGA | G | 0.483 | NM_182610 | SAMD7 | frameshift_deleti  on | exon6 | c.758_759del | p.G253fs | . | . |
| CTC3 | 3 | 171431772 | GA | G | 0.243 | NM_001130081 | PLD1 | frameshift_deleti  on | exon9 | c.821delT | p.F274fs | . | . |
| CTC3 | 3 | 173322651 | C | CA | 0.178 | NM_014932 | NLGN1 | frameshift_inserti  on | exon3 | c.264dupA | p.P88fs | . | . |
| CTC3 | 3 | 180327613 | GA | G | 0.4 | NM_001288582 | TTC14 | frameshift_deleti  on | exon12 | c.1597delA | p.K533fs | . | . |
| CTC3 | 3 | 182591685 | AC | A | 1 | NM_014616 | ATP11B | frameshift_deleti  on | exon19 | c.2135delC | p.T712fs | . | . |
| CTC3 | 3 | 182788893 | TA | T | 0.263 | NM_020166 | MCCC1 | frameshift_deleti  on | exon7 | c.654delT | p.V218fs | . | . |
| CTC3 | 3 | 182941147 | TG | T | 1 | NM_015078 | MCF2L2 | frameshift_deleti  on | exon20 | c.2287delC | p.Q763fs | . | . |
| CTC3 | 3 | 185232224 | T | TC | 0.186 | NM_139248 | LIPH | frameshift_inserti  on | exon8 | c.1067dupG | p.G356fs | . | . |
| CTC3 | 3 | 186953911 | GCC | G | 0.138 | NM_139125 | MASP1 | frameshift_deleti  on | exon11 | c.1746_1747del | p.P582fs | . | . |
| CTC3 | 3 | 187447082 | TG | T | 0.632 | NM_001134738 | BCL6 | frameshift_deleti  on | exon4 | c.1110delC | p.P370fs | . | . |
| CTC3 | 3 | 189586423 | AAG | A | 0.921 | NM_001114980 | TP63 | frameshift_deleti  on | exon6 | c.766_767del | p.R256fs | . | . |
| CTC3 | 3 | 195498637 | GA | G | 0.333 | NM_138297 | MUC4 | frameshift_deleti  on | exon3 | c.266delT | p.F89fs | . | . |
| CTC3 | 3 | 195518112 | T | TGTCTC  CTGCG | 1 | NM_018406 | MUC4 | nonframeshift_in  sertion | exon2 | c.338_339insTG  TTACGCAGGA | p.T113delinsTV  TQET | . | . |
| CTC3 | 3 | 196863474 | AT | A | 0.569 | NM_001204387 | DLG1 | stopgain | exon6 | c.709delA | p.I237X | . | . |
| CTC3 | 4 | 1374749 | CG | C | 0.373 | NM_020894 | UVSSA | frameshift_deleti on | exon12 | c.1835delG | p.R612fs | . | . |
| CTC3 | 4 | 1377575 | TG | T | 1 | NM_020894 | UVSSA | frameshift_deleti  on | exon13 | c.1884delG | p.M628fs | . | . |
| CTC3 | 4 | 5564787 | CT | C | 0.286 | NM_001166136 | EVC2 | frameshift_deleti  on | exon22 | c.3474delA | p.K1158fs | . | . |
| CTC3 | 4 | 5693000 | A | AT | 0.566 | NM_001166136 | EVC2 | frameshift_inserti  on | exon4 | c.270dupA | p.C91fs | . | . |
| CTC3 | 4 | 6302650 | CCT | C | 1 | NM_001145853 | WFS1 | frameshift_deleti  on | exon8 | c.1129_1130del | p.L377fs | . | . |
| CTC3 | 4 | 6302651 | CT | C | 1 | NM_001145853 | WFS1 | frameshift_deleti  on | exon8 | c.1130delT | p.L377fs | . | . |
| CTC3 | 4 | 7780571 | AT | A | 0.833 | NM_198595 | AFAP1 | frameshift_deleti  on | exon13 | c.1562delA | p.N521fs | . | . |
| CTC3 | 4 | 11401092 | GCA | G | 0.29 | NM_005114 | HS3ST1 | frameshift_deleti  on | exon2 | c.536_537del | p.V179fs | . | . |
| CTC3 | 4 | 13602506 | AC | A | 0.99 | NM_148894 | BOD1L1 | frameshift_deleti  on | exon10 | c.6017delG | p.G2006fs | . | . |
| CTC3 | 4 | 15504493 | A | AT | 0.5 | NM_001080522 | CC2D2A | frameshift_inserti  on | exon7 | c.385_386insT | p.R129fs | . | . |
| CTC3 | 4 | 17885761 | A | AT | 0.312 | NM_001166139 | LCORL | frameshift_inserti  on | exon7 | c.1390dupA | p.I464fs | . | . |
| CTC3 | 4 | 22403117 | GA | G | 1 | NM_145290 | GPR125 | frameshift_deleti  on | exon16 | c.2417delT | p.F806fs | . | . |
| CTC3 | 4 | 23815600 | AT | A | 0.27 | NM_013261 | PPARGC1A | frameshift_deleti  on | exon8 | c.1505delA | p.N502fs | . | . |
| CTC3 | 4 | 39230259 | CG | C | 0.4 | NM_025132 | WDR19 | frameshift_deleti  on | exon17 | c.1932delG | p.T644fs | . | . |
| CTC3 | 4 | 39310571 | A | AT | 0.609 | NM_001204747 | RFC1 | frameshift_inserti  on | exon13 | c.1569dupA | p.S524fs | . | . |
| CTC3 | 4 | 39460015 | A | AT | 0.357 | NM_001024921 | RPL9 | frameshift_inserti  on | exon1 | c.44dupA | p.N15fs | . | . |
| CTC3 | 4 | 39558079 | CT | C | 0.667 | NM_174921 | SMIM14 | frameshift_deleti  on | exon4 | c.235delA | p.S79fs | . | . |
| CTC3 | 4 | 42077774 | GA | G | 1 | NM_006345 | SLC30A9 | frameshift_deleti  on | exon16 | c.1520delA | p.E507fs | . | . |
| CTC3 | 4 | 47514618 | AG | A | 1 | NM_020453 | ATP10D | frameshift_deleti  on | exon2 | c.62delG | p.R21fs | . | . |
| CTC3 | 4 | 56236114 | GT | G | 0.389 | NM_024592 | SRD5A3 | frameshift_deleti  on | exon5 | c.814delT | p.F272fs | . | . |
| CTC3 | 4 | 57182073 | GC | G | 0.364 | NM_020722 | KIAA1211 | frameshift_deleti  on | exon8 | c.2406delC | p.S802fs | . | . |
| CTC3 | 4 | 68358626 | A | AT | 0.961 | NM_001812 | CENPC | frameshift_inserti  on | exon15 | c.2379dupA | p.S794fs | . | . |
| CTC3 | 4 | 69343312 | GT | G | 0.2 | NM_014058 | TMPRSS11E | frameshift_deleti  on | exon8 | c.934delT | p.F312fs | . | . |
| CTC3 | 4 | 70155399 | AT | A | 0.233 | NM_053039 | UGT2B28 | frameshift_deleti  on | exon4 | c.1020delT | p.D340fs | . | . |
| CTC3 | 4 | 71509266 | CA | C | 0.455 | NM_031889 | ENAM | frameshift_deleti  on | exon9 | c.2124delA | p.S708fs | . | . |
| CTC3 | 4 | 71509552 | G | GA | 0.52 | NM_031889 | ENAM | frameshift_inserti  on | exon9 | c.2410dupA | p.Q803fs | . | . |
| CTC3 | 4 | 71510101 | CA | C | 0.818 | NM_031889 | ENAM | frameshift_deleti  on | exon9 | c.2959delA | p.K987fs | . | . |
| CTC3 | 4 | 72994376 | AG | A | 1 | NM_004885 | NPFFR2 | frameshift_deleti  on | exon2 | c.375delG | p.K125fs | . | . |
| CTC3 | 4 | 74442142 | TG | T | 0.667 | NM_001270391 | RASSF6 | frameshift_deleti  on | exon9 | c.820delC | p.Q274fs | . | . |
| CTC3 | 4 | 76879052 | TC | T | 0.217 | NM_001288983 | SDAD1 | frameshift_deleti  on | exon17 | c.1414delG | p.D472fs | . | . |
| CTC3 | 4 | 79328897 | AC | A | 0.846 | NM_001166133 | FRAS1 | frameshift_deleti  on | exon31 | c.4211delC | p.T1404fs | . | . |
| CTC3 | 4 | 79503336 | G | GA | 0.592 | NM_005139 | ANXA3 | frameshift_inserti  on | exon5 | c.205dupA | p.L68fs | . | . |
| CTC3 | 4 | 79792082 | CCAG | C | 0.273 | NM_017593 | BMP2K | nonframeshift_de  letion | exon11 | c.1378_1380del | p.460_460del | . | . |
| CTC3 | 4 | 79792136 | ACAGCA  GCAG | A | 0.933 | NM_017593 | BMP2K | nonframeshift_de  letion | exon11 | c.1432_1440del | p.478_480del | . | . |
| CTC3 | 4 | 79792154 | GCAGCA  GCAGCA | G | 0.5 | NM_017593 | BMP2K | nonframeshift_de  letion | exon11 | c.1450_1461del | p.484_487del | . | . |
| CTC3 | 4 | 83891537 | AT | A | 1 | NM_001115007 | LIN54 | frameshift_deleti  on | exon4 | c.230delA | p.N77fs | . | . |
| CTC3 | 4 | 85617271 | TTA | T | 0.833 | NM_014991 | WDFY3 | frameshift_deleti  on | exon58 | c.8751_8752del | p.Y2917fs | . | . |
| CTC3 | 4 | 86916501 | CT | C | 0.5 | NM_031305 | ARHGAP24 | frameshift_deleti  on | exon6 | c.1416delT | p.P472fs | . | . |
| CTC3 | 4 | 86916615 | AC | A | 1 | NM_031305 | ARHGAP24 | frameshift_deleti  on | exon6 | c.1530delC | p.H510fs | . | . |
| CTC3 | 4 | 87556434 | CT | C | 0.971 | NM_006264 | PTPN13 | frameshift_deleti  on | exon2 | c.26delT | p.L9fs | . | . |

| CTC3 | 4 | 88401659 | TA | T | 0.375 | NM_004684 | SPARCL1 | frameshift_deleti  on | exon9 | c.1681delT | p.Y561fs | . | . |
| --- | --- | --- | --- | --- | --- | --- | --- | --- | --- | --- | --- | --- | --- |
| CTC3 | 4 | 88534274 | TC | T | 0.281 | NM_014208 | DSPP | frameshift_deleti  on | exon4 | c.937delC | p.P313fs | . | . |
| CTC3 | 4 | 88534278 | CA | C | 0.263 | NM_014208 | DSPP | frameshift_deleti  on | exon4 | c.941delA | p.H314fs | . | . |
| CTC3 | 4 | 89625742 | AAAG | A | 0.333 | NM_001271602 | HERC3 | nonframeshift_de  letion | exon24 | c.2572_2574del | p.858_858del | . | . |
| CTC3 | 4 | 95170811 | GAATC | G | 0.167 | NM_001128429 | SMARCAD1 | frameshift_deleti  on | exon7 | c.713_716del | p.E238fs | . | . |
| CTC3 | 4 | 95220696 | TC | T | 0.565 | NM_014485 | HPGDS | frameshift_deleti  on | exon6 | c.534delG | p.R178fs | . | . |
| CTC3 | 4 | 100131650 | AC | A | 0.5 | NM_000672 | ADH6 | frameshift_deleti  on | exon4 | c.271delG | p.V91fs | . | . |
| CTC3 | 4 | 100235073 | GA | G | 1 | NM_000668 | ADH1B | frameshift_deleti  on | exon6 | c.732delT | p.P244fs | . | . |
| CTC3 | 4 | 106638821 | CT | C | 0.85 | NM_001031720 | GSTCD | frameshift_deleti  on | exon2 | c.52delT | p.F18fs | . | . |
| CTC3 | 4 | 106863684 | A | AC | 0.5 | NM_001033047 | NPNT | frameshift_inserti  on | exon8 | c.985dupC | p.P328fs | . | . |
| CTC3 | 4 | 109576728 | TA | T | 0.314 | NM_001267817 | OSTC | frameshift_deleti  on | exon2 | c.149delA | p.Y50fs | . | . |
| CTC3 | 4 | 113352408 | GT | G | 0.385 | NM_001253884 | ALPK1 | frameshift_deleti  on | exon10 | c.1472delT | p.V491fs | . | . |
| CTC3 | 4 | 113483583 | GT | G | 0.639 | NM_018392 | C4orf21 | frameshift_deleti  on | exon18 | c.4640delA | p.N1547fs | . | . |
| CTC3 | 4 | 113539476 | CT | C | 0.75 | NM_018392 | C4orf21 | frameshift_deleti  on | exon6 | c.1721delA | p.K574fs | . | . |
| CTC3 | 4 | 114824638 | CA | C | 0.559 | NM_024590 | ARSJ | frameshift_deleti  on | exon2 | c.591delT | p.F197fs | . | . |
| CTC3 | 4 | 123145823 | AG | A | 0.227 | NM_015312 | KIAA1109 | frameshift_deleti  on | exon21 | c.2785delG | p.A929fs | . | . |
| CTC3 | 4 | 123151086 | AT | A | 0.333 | NM_015312 | KIAA1109 | frameshift_deleti  on | exon24 | c.3044delT | p.M1015fs | . | . |
| CTC3 | 4 | 123151198 | AT | A | 0.292 | NM_015312 | KIAA1109 | frameshift_deleti  on | exon24 | c.3156delT | p.N1052fs | . | . |
| CTC3 | 4 | 123159284 | C | CA | 0.312 | NM_015312 | KIAA1109 | frameshift_inserti  on | exon26 | c.3613dupA | p.N1204fs | . | . |
| CTC3 | 4 | 126372305 | T | TG | 0.235 | NM_024582 | FAT4 | frameshift_inserti  on | exon9 | c.10135dupG | p.F3378fs | . | . |
| CTC3 | 4 | 126412055 | CAA | C | 0.26 | NM_024582 | FAT4 | frameshift_deleti  on | exon17 | c.14079_14080  del | p.P4693fs | . | . |
| CTC3 | 4 | 128807062 | AC | A | 1 | NM_001190799 | PLK4 | frameshift_deleti  on | exon4 | c.442delC | p.P148fs | . | . |
| CTC3 | 4 | 134073568 | TCTG | T | 0.286 | NM_020815 | PCDH10 | nonframeshift_de  letion | exon1 | c.2274_2276del | p.758_759del | . | . |
| CTC3 | 4 | 140811063 | TTGCTGC  TGCTGC | T | 1 | NM_018717 | MAML3 | frameshift_deleti  on | exon3 | c.1513_1514del | p.Q505fs | . | . |
| CTC3 | 4 | 140811063 | TTGCTGC  TGCTGCT | T | 1 | NM_018717 | MAML3 | nonframeshift_de  letion | exon2 | c.1512_1514del | p.504_505del | . | . |
| CTC3 | 4 | 144451678 | TG | T | 0.9 | NM_003601 | SMARCA5 | frameshift_deleti  on | exon9 | c.1158delG | p.M386fs | . | . |
| CTC3 | 4 | 153690932 | CT | C | 0.192 | NM_145720 | TIGD4 | frameshift_deleti  on | exon2 | c.1224delA | p.G408fs | . | . |
| CTC3 | 4 | 153691418 | AT | A | 0.931 | NM_145720 | TIGD4 | frameshift_deleti  on | exon2 | c.738delA | p.K246fs | . | . |
| CTC3 | 4 | 153691502 | CA | C | 0.714 | NM_145720 | TIGD4 | frameshift_deleti  on | exon2 | c.654delT | p.V218fs | . | . |
| CTC3 | 4 | 153691613 | TA | T | 0.333 | NM_145720 | TIGD4 | frameshift_deleti  on | exon2 | c.543delT | p.N181fs | . | . |
| CTC3 | 4 | 154506699 | AT | A | 1 | NM_001131007 | KIAA0922 | frameshift_deleti  on | exon13 | c.1248delT | p.H416fs | . | . |
| CTC3 | 4 | 155156434 | TC | T | 0.433 | NM_017639 | DCHS2 | frameshift_deleti  on | exon25 | c.8004delG | p.L2668fs | . | . |
| CTC3 | 4 | 155244401 | TTTTG | T | 1 | NM_001142552 | DCHS2 | frameshift_deleti  on | exon8 | c.4095_4098del | p.N1365fs | . | . |
| CTC3 | 4 | 155507796 | T | TAA | 0.333 | NM_000508 | FGA | frameshift_inserti  on | exon5 | c.784_785insTT | p.E262fs | . | . |
| CTC3 | 4 | 159568213 | GT | G | 1 | NM_001253729 | RXFP1 | frameshift_deleti  on | exon14 | c.1473delT | p.G491fs | . | . |
| CTC3 | 4 | 169060706 | A | AG | 0.185 | NM_007193 | ANXA10 | frameshift_inserti  on | exon3 | c.171dupG | p.E57fs | . | . |
| CTC3 | 4 | 170028321 | GC | G | 1 | NM_020870 | SH3RF1 | frameshift_deleti  on | exon11 | c.2174delG | p.G725fs | . | . |
| CTC3 | 4 | 170477237 | GA | G | 1 | NM_001199399 | NEK1 | frameshift_deleti  on | exon16 | c.1200delT | p.F400fs | . | . |
| CTC3 | 4 | 170985932 | TC | T | 0.402 | NM_016228 | AADAT | stopgain | exon11 | c.1071delG | p.W357X | . | . |
| CTC3 | 4 | 175897263 | AT | A | 0.274 | NM_001278127 | ADAM29 | frameshift_deleti on | exon2 | c.588delT | p.H196fs | . | . |
| CTC3 | 4 | 177650786 | CT | C | 1 | NM_005429 | VEGFC | frameshift_deleti  on | exon2 | c.261delA | p.K87fs | . | . |
| CTC3 | 4 | 186544528 | GT | G | 0.25 | NM_001145674 | SORBS2 | frameshift_deleti  on | exon7 | c.1754delA | p.N585fs | . | . |
| CTC3 | 4 | 187455619 | AC | A | 0.556 | NM_005958 | MTNR1A | frameshift_deleti  on | exon2 | c.276delG | p.G92fs | . | . |
| CTC3 | 4 | 189063579 | CA | C | 0.571 | NM_178556 | TRIML1 | frameshift_deleti  on | exon3 | c.679delA | p.K227fs | . | . |
| CTC3 | 5 | 10461351 | GC | G | 0.2 | NM_031916 | ROPN1L | frameshift_deleti  on | exon4 | c.474delC | p.G158fs | . | . |
| CTC3 | 5 | 13919416 | CA | C | 0.786 | NM_001369 | DNAH5 | frameshift_deleti  on | exon7 | c.843delT | p.V281fs | . | . |
| CTC3 | 5 | 33461063 | AT | A | 1 | NM_152295 | TARS | frameshift_deleti  on | exon12 | c.1308delT | p.D436fs | . | . |
| CTC3 | 5 | 33637697 | GA | G | 0.7 | NM_030955 | ADAMTS12 | frameshift_deleti  on | exon12 | c.1872delT | p.F624fs | . | . |
| CTC3 | 5 | 35013145 | C | CA | 0.61 | NM_031900 | AGXT2 | frameshift_inserti  on | exon11 | c.1101dupT | p.A368fs | . | . |
| CTC3 | 5 | 35084606 | G | GA | 0.75 | NM_001204315 | PRLR | frameshift_inserti  on | exon4 | c.338dupT | p.F113fs | . | . |
| CTC3 | 5 | 35649518 | CA | C | 1 | NM_024867 | SPEF2 | frameshift_deleti  on | exon6 | c.783delA | p.A261fs | . | . |
| CTC3 | 5 | 35876577 | CA | C | 0.957 | NM_002185 | IL7R | frameshift_deleti  on | exon8 | c.1370delA | p.Q457fs | . | . |
| CTC3 | 5 | 36680622 | CT | C | 0.462 | NM_001289940 | SLC1A3 | frameshift_deleti  on | exon6 | c.885delT | p.A295fs | . | . |
| CTC3 | 5 | 37341327 | TA | T | 1 | NM_001278312 | NUP155 | frameshift_deleti  on | exon11 | c.1110delT | p.F370fs | . | . |
| CTC3 | 5 | 38370526 | GC | G | 1 | NM_001205301 | EGFLAM | frameshift_deleti  on | exon6 | c.675delC | p.S225fs | . | . |
| CTC3 | 5 | 39341756 | CT | C | 0.659 | NM_001737 | C9 | frameshift_deleti  on | exon3 | c.229delA | p.R77fs | . | . |
| CTC3 | 5 | 40681703 | AC | A | 0.641 | NM_000958 | PTGER4 | frameshift_deleti  on | exon2 | c.609delC | p.N203fs | . | . |
| CTC3 | 5 | 41159293 | TC | T | 0.353 | NM_000065 | C6 | frameshift_deleti  on | exon12 | c.1746delG | p.K582fs | . | . |

| CTC3 | 5 | 41382494 | CA | C | 0.524 | NM_001005473 | PLCXD3 | frameshift_deleti  on | exon2 | c.245delT | p.M82fs | . | . |
| --- | --- | --- | --- | --- | --- | --- | --- | --- | --- | --- | --- | --- | --- |
| CTC3 | 5 | 41929930 | TGG | T | 0.622 | NM_012176 | FBXO4 | frameshift_deleti  on | exon3 | c.558_559del | p.L186fs | . | . |
| CTC3 | 5 | 43535725 | CA | C | 0.375 | NM_006451 | PAIP1 | frameshift_deleti  on | exon7 | c.989delT | p.L330fs | . | . |
| CTC3 | 5 | 57790543 | A | AT | 0.3 | NM_152687 | GAPT | frameshift_inserti  on | exon3 | c.181dupT | p.T60fs | . | . |
| CTC3 | 5 | 58147144 | CT | C | 0.722 | NM_138453 | RAB3C | frameshift_deleti  on | exon5 | c.651delT | p.T217fs | . | . |
| CTC3 | 5 | 61876364 | CT | C | 0.6 | NM_181506 | LRRC70 | frameshift_deleti  on | exon2 | c.1100delT | p.L367fs | . | . |
| CTC3 | 5 | 65118633 | AT | A | 0.158 | NM_020726 | NLN | frameshift_deleti  on | exon13 | c.2006delT | p.I669fs | . | . |
| CTC3 | 5 | 71016364 | GA | G | 0.221 | NM_004291 | CARTPT | frameshift_deleti  on | exon3 | c.274delA | p.K92fs | . | . |
| CTC3 | 5 | 74021846 | TACTCA  A | T | 0.807 | NM_170691 | GFM2 | nonframeshift_de  letion | exon17 | c.1685_1690del | p.562_564del | . | . |
| CTC3 | 5 | 74491715 | TTCA | T | 0.842 | NM_001164443 | ANKRD31 | nonframeshift_de  letion | exon7 | c.755_757del | p.252_253del | . | . |
| CTC3 | 5 | 74807267 | AGCC | A | 0.167 | NM_001130105 | COL4A3BP | nonframeshift_de  letion | exon2 | c.147_149del | p.49_50del | . | . |
| CTC3 | 5 | 74848354 | GA | G | 0.741 | NM_016218 | POLK | frameshift_deleti  on | exon3 | c.194delA | p.E65fs | . | . |
| CTC3 | 5 | 74907604 | CG | C | 1 | NM_001276713 | ANKDD1B | frameshift_deleti  on | exon1 | c.146delG | p.R49fs | . | . |
| CTC3 | 5 | 75871553 | GAA | G | 0.571 | NM_001285460 | IQGAP2 | frameshift_deleti  on | exon4 | c.268_269del | p.K90fs | . | . |
| CTC3 | 5 | 75964569 | AT | A | 0.333 | NM_001285461 | IQGAP2 | frameshift_deleti  on | exon11 | c.1232delT | p.M411fs | . | . |
| CTC3 | 5 | 78379594 | AG | A | 0.593 | NM_001178005 | BHMT2 | frameshift_deleti  on | exon6 | c.734delG | p.R245fs | . | . |
| CTC3 | 5 | 78936746 | TG | T | 0.808 | NM_001114394 | PAPD4 | frameshift_deleti  on | exon5 | c.539delG | p.C180fs | . | . |
| CTC3 | 5 | 79030965 | TA | T | 1 | NM_153610 | CMYA5 | frameshift_deleti  on | exon2 | c.6378delA | p.V2126fs | . | . |
| CTC3 | 5 | 79031683 | GC | G | 1 | NM_153610 | CMYA5 | frameshift_deleti  on | exon2 | c.7096delC | p.P2366fs | . | . |
| CTC3 | 5 | 79809434 | A | AT | 1 | NM_205548 | FAM151B | frameshift_inserti  on | exon3 | c.164dupT | p.M55fs | . | . |
| CTC3 | 5 | 80409719 | TA | T | 0.667 | NM_006909 | RASGRF2 | frameshift_deleti  on | exon15 | c.2451delA | p.L817fs | . | . |
| CTC3 | 5 | 86628406 | G | GT | 0.333 | NM_002890 | RASA1 | frameshift_inserti  on | exon3 | c.776dupT | p.V259fs | . | . |
| CTC3 | 5 | 95236730 | TA | T | 0.489 | NM_012081 | ELL2 | frameshift_deleti  on | exon6 | c.795delT | p.F265fs | . | . |
| CTC3 | 5 | 102285636 | AT | A | 0.27 | NM_000919 | PAM | frameshift_deleti  on | exon10 | c.756delT | p.N252fs | . | . |
| CTC3 | 5 | 102432298 | CT | C | 0.429 | NM_017676 | GIN1 | frameshift_deleti  on | exon7 | c.1240delA | p.R414fs | . | . |
| CTC3 | 5 | 108233469 | CA | C | 0.439 | NM_005246 | FER | frameshift_deleti  on | exon10 | c.1157delA | p.Q386fs | . | . |
| CTC3 | 5 | 109963560 | G | GT | 0.333 | NM_001039763 | TMEM232 | frameshift_inserti  on | exon6 | c.522dupA | p.R175fs | . | . |
| CTC3 | 5 | 110461453 | AT | A | 0.636 | NM_139281 | WDR36 | frameshift_deleti  on | exon22 | c.2667delT | p.D889fs | . | . |
| CTC3 | 5 | 110730425 | A | AT | 0.667 | NM_001744 | CAMK4 | frameshift_inserti  on | exon5 | c.405dupT | p.Y135fs | . | . |
| CTC3 | 5 | 112176739 | GA | G | 0.396 | NM_001127511 | APC | frameshift_deleti  on | exon14 | c.5395delA | p.K1799fs | . | . |
| CTC3 | 5 | 112179441 | G | GT | 0.8 | NM_001127511 | APC | frameshift_inserti  on | exon14 | c.8097dupT | p.G2699fs | . | . |
| CTC3 | 5 | 118450171 | AC | A | 0.842 | NM_005509 | DMXL1 | frameshift_deleti  on | exon6 | c.500delC | p.T167fs | . | . |
| CTC3 | 5 | 122152988 | AG | A | 0.273 | NM_001278199 | SNX2 | frameshift_deleti  on | exon10 | c.576delG | p.K192fs | . | . |
| CTC3 | 5 | 131926962 | TAA | T | 0.5 | NM_005732 | RAD50 | frameshift_deleti  on | exon10 | c.1500_1501del | p.L500fs | . | . |
| CTC3 | 5 | 132010189 | TG | T | 0.6 | NM_000589 | IL4 | frameshift_deleti  on | exon2 | c.175delG | p.A59fs | . | . |
| CTC3 | 5 | 134074351 | C | CG | 0.524 | NM_001745 | CAMLG | frameshift_inserti  on | exon1 | c.42dupG | p.P14fs | . | . |
| CTC3 | 5 | 137803109 | AC | A | 1 | NM_001964 | EGR1 | frameshift_deleti  on | exon2 | c.972delC | p.Y324fs | . | . |
| CTC3 | 5 | 137803231 | CA | C | 1 | NM_001964 | EGR1 | frameshift_deleti  on | exon2 | c.1094delA | p.Q365fs | . | . |
| CTC3 | 5 | 139905671 | T | TC | 0.615 | NM_017747 | ANKHD1 | frameshift_inserti  on | exon26 | c.4583_4584ins  C | p.F1528fs | . | . |
| CTC3 | 5 | 140750248 | GT | G | 0.238 | NM_018924 | PCDHGB3 | frameshift_deleti  on | exon1 | c.288delT | p.C96fs | . | . |
| CTC3 | 5 | 140755047 | CCA | C | 1 | NM_018919 | PCDHGA6 | frameshift_deleti  on | exon1 | c.1398_1399del | p.P466fs | . | . |
| CTC3 | 5 | 140755048 | CA | C | 1 | NM_018919 | PCDHGA6 | frameshift_deleti  on | exon1 | c.1399delA | p.R467fs | . | . |
| CTC3 | 5 | 140763288 | TG | T | 0.733 | NM_018920 | PCDHGA7 | frameshift_deleti  on | exon1 | c.823delG | p.G275fs | . | . |
| CTC3 | 5 | 140764779 | C | CG | 0.941 | NM_018920 | PCDHGA7 | frameshift_inserti  on | exon1 | c.2313_2314ins  G | p.P771fs | . | . |
| CTC3 | 5 | 140778451 | GA | G | 0.333 | NM_018925 | PCDHGB5 | frameshift_deleti  on | exon1 | c.758delA | p.E253fs | . | . |
| CTC3 | 5 | 140783538 | TA | T | 0.812 | NM_018921 | PCDHGA9 | frameshift_deleti  on | exon1 | c.1020delA | p.V340fs | . | . |
| CTC3 | 5 | 140799327 | A | AC | 1 | NM_018927 | PCDHGB7 | frameshift_inserti  on | exon1 | c.1902dupC | p.D634fs | . | . |
| CTC3 | 5 | 140953563 | TGGA | T | 0.246 | NM_001079812 | DIAPH1 | nonframeshift_de  letion | exon15 | c.1824_1826del | p.608_609del | . | . |
| CTC3 | 5 | 141329078 | CT | C | 0.375 | NM_016580 | PCDH12 | frameshift_deleti  on | exon3 | c.3048delA | p.E1016fs | . | . |
| CTC3 | 5 | 141354477 | C | CT | 0.5 | NM_183399 | RNF14 | frameshift_inserti  on | exon3 | c.263_264insT | p.P88fs | . | . |
| CTC3 | 5 | 142586870 | TC | T | 1 | NM_001135608 | ARHGAP26 | frameshift_deleti  on | exon21 | c.2097delC | p.V699fs | . | . |
| CTC3 | 5 | 145252318 | TG | T | 1 | NM_001080516 | GRXCR2 | frameshift_deleti  on | exon1 | c.213delC | p.P71fs | . | . |
| CTC3 | 5 | 145647268 | A | AC | 0.429 | NM_018989 | RBM27 | frameshift_inserti  on | exon15 | c.2389dupC | p.T796fs | . | . |
| CTC3 | 5 | 145894834 | CG | C | 1 | NM_194251 | GPR151 | frameshift_deleti  on | exon1 | c.842delC | p.P281fs | . | . |
| CTC3 | 5 | 149212553 | CA | C | 0.286 | NM_001172698 | PPARGC1B | frameshift_deleti  on | exon4 | c.801delA | p.P267fs | . | . |
| CTC3 | 5 | 149501588 | G | GA | 0.237 | NM_002609 | PDGFRB | frameshift_inserti  on | exon16 | c.2198_2199ins  T | p.T733fs | . | . |
| CTC3 | 5 | 150859022 | TG | T | 0.78 | NM_078483 | SLC36A1 | frameshift_deleti  on | exon10 | c.1132delG | p.V378fs | . | . |
| CTC3 | 5 | 150945514 | AGC | A | 0.27 | NM_001447 | FAT2 | frameshift_deleti  on | exon1 | c.2977_2978del | p.A993fs | . | . |

| CTC3 | 5 | 153432510 | T | TA | 0.367 | NM_001242336 | MFAP3 | frameshift_inserti  on | exon3 | c.327dupA | p.L109fs | . | . |
| --- | --- | --- | --- | --- | --- | --- | --- | --- | --- | --- | --- | --- | --- |
| CTC3 | 5 | 156390208 | AT | A | 0.265 | NM_001146726 | TIMD4 | frameshift_deleti  on | exon1 | c.1delA | p.M1fs | . | . |
| CTC3 | 5 | 156788531 | CA | C | 0.297 | NM_001037332 | CYFIP2 | frameshift_deleti  on | exon26 | c.2965delA | p.K989fs | . | . |
| CTC3 | 5 | 169535058 | G | GT | 0.202 | NM_012188 | FOXI1 | frameshift_inserti  on | exon2 | c.580_581insT | p.G194fs | . | . |
| CTC3 | 5 | 170235660 | AT | A | 0.833 | NM_014211 | GABRP | frameshift_deleti  on | exon8 | c.737delT | p.I246fs | . | . |
| CTC3 | 5 | 172661815 | G | GT | 0.667 | NM_001166175 | NKX2-5 | frameshift_inserti  on | exon1 | c.271_272insA | p.A91fs | . | . |
| CTC3 | 5 | 175995782 | TG | T | 0.433 | NM_001171976 | CDHR2 | frameshift_deleti  on | exon4 | c.229delG | p.G77fs | . | . |
| CTC3 | 5 | 176825161 | ATGCTG | A | 0.286 | NM_003052 | SLC34A1 | frameshift_deleti  on | exon13 | c.1795_1799del | p.C599fs | . | . |
| CTC3 | 5 | 180278318 | GT | G | 0.618 | NM_001172638 | ZFP62 | frameshift_deleti  on | exon2 | c.176delA | p.N59fs | . | . |
| CTC3 | 6 | 4128000 | TA | T | 0.659 | NM_001166010 | ECI2 | stopgain | exon5 | c.476delT | p.L159X | . | . |
| CTC3 | 6 | 5771642 | GGT | G | 0.478 | NM_006567 | FARS2 | frameshift_deleti on | exon7 | c.1337_1338del | p.G446fs | . | . |
| CTC3 | 6 | 7579612 | G | GA | 0.238 | NM_001008844 | DSP | frameshift_inserti  on | exon23 | c.3190dupA | p.Q1063fs | . | . |
| CTC3 | 6 | 7581574 | CTT | C | 0.167 | NM_004415 | DSP | frameshift_deleti  on | exon23 | c.5152_5153del | p.L1718fs | . | . |
| CTC3 | 6 | 10875067 | G | GA | 0.44 | NM_004752 | GCM2 | frameshift_inserti  on | exon5 | c.681dupT | p.P228fs | . | . |
| CTC3 | 6 | 17828553 | CA | C | 1 | NM_001105566 | KIF13A | frameshift_deleti  on | exon14 | c.1449delT | p.F483fs | . | . |
| CTC3 | 6 | 24467000 | GA | G | 0.319 | NM_001503 | GPLD1 | frameshift_deleti  on | exon10 | c.728delT | p.F243fs | . | . |
| CTC3 | 6 | 26217397 | GCT | G | 0.5 | NM_021052 | HIST1H2AE | frameshift_deleti  on | exon1 | c.196_197del | p.L66fs | . | . |
| CTC3 | 6 | 26406268 | AG | A | 1 | NM_001145008 | BTN3A1 | frameshift_deleti  on | exon3 | c.218delG | p.R73fs | . | . |
| CTC3 | 6 | 26468642 | TG | T | 0.171 | NM_001197233 | BTN2A1 | stopgain | exon7 | c.1267delG | p.V423X | . | . |
| CTC3 | 6 | 27115149 | CG | C | 0.278 | NM_080596 | HIST1H2AH | frameshift_deleti on | exon1 | c.243delG | p.P81fs | . | . |
| CTC3 | 6 | 27115170 | TC | T | 0.333 | NM_080596 | HIST1H2AH | frameshift_deleti  on | exon1 | c.264delC | p.I88fs | . | . |
| CTC3 | 6 | 27421017 | AT | A | 0.26 | NM_007149 | ZNF184 | frameshift_deleti  on | exon6 | c.320delA | p.N107fs | . | . |
| CTC3 | 6 | 29080230 | TG | T | 0.147 | NM_001005216 | OR2J3 | frameshift_deleti  on | exon1 | c.564delG | p.L188fs | . | . |
| CTC3 | 6 | 29455155 | GAC | G | 0.375 | NM_052967 | MAS1L | frameshift_deleti  on | exon1 | c.523_524del | p.V175fs | . | . |
| CTC3 | 6 | 30545852 | GC | G | 0.277 | NM_001025091 | ABCF1 | frameshift_deleti  on | exon4 | c.217delC | p.Q73fs | . | . |
| CTC3 | 6 | 30627256 | GC | G | 0.8 | NM_001164239 | DHX16 | frameshift_deleti  on | exon12 | c.1819delG | p.A607fs | . | . |
| CTC3 | 6 | 30652581 | TG | T | 1 | NM_133471 | PPP1R18 | frameshift_deleti  on | exon1 | c.1214delC | p.P405fs | . | . |
| CTC3 | 6 | 30996865 | TA | T | 0.163 | NM_001198815 | MUC22 | frameshift_deleti  on | exon3 | c.3658delA | p.T1220fs | . | . |
| CTC3 | 6 | 31118793 | TC | T | 0.3 | NM_001105563 | CCHCR1 | frameshift_deleti  on | exon5 | c.799delG | p.E267fs | . | . |
| CTC3 | 6 | 31138370 | CG | C | 0.213 | NM_002701 | POU5F1 | frameshift_deleti  on | exon1 | c.27delC | p.F9fs | . | . |
| CTC3 | 6 | 31604043 | GT | G | 0.194 | NM_004638 | PRRC2A | stopgain | exon26 | c.5683delT | p.L1895X | . | . |
| CTC3 | 6 | 32020462 | CG | C | 0.5 | NM_019105 | TNXB | frameshift_deleti on | exon26 | c.9093delC | p.S3031fs | . | . |
| CTC3 | 6 | 32948181 | GA | G | 0.292 | NM_001199456 | BRD2 | frameshift_deleti  on | exon11 | c.2069delA | p.E690fs | . | . |
| CTC3 | 6 | 33169595 | CT | C | 0.423 | NM_006979 | SLC39A7 | frameshift_deleti  on | exon2 | c.486delT | p.S162fs | . | . |
| CTC3 | 6 | 33173662 | G | GA | 0.333 | NM_014234 | HSD17B8 | frameshift_inserti  on | exon6 | c.625dupA | p.Q208fs | . | . |
| CTC3 | 6 | 33287897 | TTCC | T | 0.144 | NM_001254717 | DAXX | nonframeshift_de  letion | exon4 | c.1128_1130del | p.376_377del | . | . |
| CTC3 | 6 | 33415655 | AC | A | 1 | NM_006772 | SYNGAP1 | frameshift_deleti  on | exon18 | c.3831delC | p.H1277fs | . | . |
| CTC3 | 6 | 38950203 | A | AT | 0.571 | NM_001206927 | DNAH8 | frameshift_inserti  on | exon86 | c.12917dupT | p.I4306fs | . | . |
| CTC3 | 6 | 39158930 | T | TC | 0.333 | NM_003740 | KCNK5 | frameshift_inserti  on | exon5 | c.1235_1236ins  G | p.P412fs | . | . |
| CTC3 | 6 | 39513437 | CA | C | 0.659 | NM_001289020 | KIF6 | frameshift_deleti  on | exon11 | c.1208delT | p.L403fs | . | . |
| CTC3 | 6 | 39847039 | CT | C | 0.231 | NM_001201427 | DAAM2 | frameshift_deleti  on | exon14 | c.1632delT | p.P544fs | . | . |
| CTC3 | 6 | 41126340 | CT | C | 0.278 | NM_001271821 | TREM2 | stoploss | exon4 | c.660delA | p.X220W | . | . |
| CTC3 | 6 | 41658829 | TTGC | T | 0.137 | NM_001167827 | TFEB | nonframeshift_de letion | exon2 | c.162_164del | p.54_55del | . | . |
| CTC3 | 6 | 42532073 | GA | G | 0.19 | NM_001184801 | UBR2 | frameshift_deleti  on | exon1 | c.17delA | p.E6fs | . | . |
| CTC3 | 6 | 42821395 | AGT | A | 0.5 | NM_015349 | GLTSCR1L | frameshift_deleti  on | exon7 | c.1966_1967del | p.V656fs | . | . |
| CTC3 | 6 | 42975788 | AC | A | 1 | NM_180977 | PPP2R5D | frameshift_deleti  on | exon5 | c.525delC | p.N175fs | . | . |
| CTC3 | 6 | 43307367 | CG | C | 0.684 | NM_014345 | ZNF318 | frameshift_deleti  on | exon10 | c.4368delC | p.P1456fs | . | . |
| CTC3 | 6 | 44200139 | CG | C | 0.714 | NM_001078174 | SLC29A1 | frameshift_deleti  on | exon11 | c.1034delG | p.R345fs | . | . |
| CTC3 | 6 | 44250114 | GT | G | 0.444 | NM_182539 | TCTE1 | frameshift_deleti  on | exon4 | c.1028delA | p.N343fs | . | . |
| CTC3 | 6 | 46107699 | GA | G | 0.261 | NM_014936 | ENPP4 | frameshift_deleti  on | exon2 | c.380delA | p.E127fs | . | . |
| CTC3 | 6 | 46598747 | TG | T | 0.7 | NM_001278739 | CYP39A1 | frameshift_deleti  on | exon5 | c.276delC | p.P92fs | . | . |
| CTC3 | 6 | 49403268 | AGC | A | 1 | NM_000255 | MUT | frameshift_deleti  on | exon12 | c.2023_2024del | p.A675fs | . | . |
| CTC3 | 6 | 50682988 | AC | A | 0.36 | NM_172238 | TFAP2D | frameshift_deleti  on | exon2 | c.200delC | p.T67fs | . | . |
| CTC3 | 6 | 50811026 | ACAC | A | 1 | NM_003221 | TFAP2B | nonframeshift_de  letion | exon7 | c.1305_1307del | p.435_436del | . | . |
| CTC3 | 6 | 50811033 | CACT | C | 1 | NM_003221 | TFAP2B | nonframeshift_de  letion | exon7 | c.1312_1314del | p.438_438del | . | . |
| CTC3 | 6 | 51774124 | G | GA | 0.37 | NM_138694 | PKHD1 | frameshift_inserti  on | exon40 | c.6638dupT | p.F2213fs | . | . |
| CTC3 | 6 | 51890756 | TGG | T | 0.444 | NM_138694 | PKHD1 | frameshift_deleti  on | exon32 | c.3850_3851del | p.P1284fs | . | . |
| CTC3 | 6 | 51890757 | GGT | G | 0.723 | NM_138694 | PKHD1 | frameshift_deleti  on | exon32 | c.3849_3850del | p.S1283fs | . | . |

| CTC3 | 6 | 51910807 | TA | T | 0.472 | NM_138694 | PKHD1 | frameshift_deleti  on | exon24 | c.2586delT | p.F862fs | . | . |
| --- | --- | --- | --- | --- | --- | --- | --- | --- | --- | --- | --- | --- | --- |
| CTC3 | 6 | 53516401 | TA | T | 0.778 | NM_001003760 | KLHL31 | frameshift_deleti  on | exon3 | c.1899delT | p.S633fs | . | . |
| CTC3 | 6 | 56347663 | CT | C | 1 | NM_015548 | DST | frameshift_deleti  on | exon70 | c.13350delA | p.K4450fs | . | . |
| CTC3 | 6 | 56362723 | AT | A | 1 | NM_015548 | DST | frameshift_deleti  on | exon62 | c.12155delA | p.N4052fs | . | . |
| CTC3 | 6 | 64431052 | AT | A | 0.421 | NM_001142800 | EYS | frameshift_deleti  on | exon43 | c.8874delA | p.K2958fs | . | . |
| CTC3 | 6 | 64431644 | G | GT | 0.333 | NM_001142800 | EYS | stopgain | exon43 | c.8282dupA | p.Y2761_N2762  delinsX | . | . |
| CTC3 | 6 | 64487976 | GC | G | 0.955 | NM_001142800 | EYS | frameshift_deleti  on | exon40 | c.7820delG | p.G2607fs | . | . |
| CTC3 | 6 | 70646736 | TA | T | 1 | NM_001858 | COL19A1 | frameshift_deleti  on | exon8 | c.808delA | p.K270fs | . | . |
| CTC3 | 6 | 71235785 | AG | A | 0.5 | NM_001162529 | FAM135A | frameshift_deleti  on | exon13 | c.2999delG | p.S1000fs | . | . |
| CTC3 | 6 | 71567864 | CA | C | 0.598 | NM_001281439 | SMAP1 | frameshift_deleti  on | exon9 | c.1121delA | p.Q374fs | . | . |
| CTC3 | 6 | 72011241 | AT | A | 0.75 | NM_024576 | OGFRL1 | frameshift_deleti  on | exon7 | c.846delT | p.Y282fs | . | . |
| CTC3 | 6 | 76388323 | CT | C | 0.427 | NM_001100409 | SENP6 | frameshift_deleti  on | exon14 | c.1853delT | p.L618fs | . | . |
| CTC3 | 6 | 76566921 | CT | C | 0.579 | NM_004999 | MYO6 | frameshift_deleti  on | exon13 | c.1332delT | p.P444fs | . | . |
| CTC3 | 6 | 76582967 | C | CA | 0.556 | NM_004999 | MYO6 | frameshift_inserti  on | exon20 | c.2028dupA | p.T676fs | . | . |
| CTC3 | 6 | 83847886 | GT | G | 0.256 | NM_001199942 | DOPEY1 | frameshift_deleti  on | exon21 | c.4099delT | p.Y1367fs | . | . |
| CTC3 | 6 | 84311308 | T | TC | 0.333 | NM_001256718 | SNAP91 | frameshift_inserti  on | exon13 | c.794dupG | p.G265fs | . | . |
| CTC3 | 6 | 84884542 | CT | C | 1 | NM_001286206 | KIAA1009 | frameshift_deleti  on | exon15 | c.1700delA | p.K567fs | . | . |
| CTC3 | 6 | 87968887 | TA | T | 0.625 | NM_015021 | ZNF292 | frameshift_deleti  on | exon8 | c.5541delA | p.L1847fs | . | . |
| CTC3 | 6 | 89891718 | CAAG | C | 0.226 | NM_001256703 | GABRR1 | nonframeshift_de  letion | exon7 | c.801_803del | p.267_268del | . | . |
| CTC3 | 6 | 90556359 | TTC | T | 0.3 | NM_001137667 | CASP8AP2 | frameshift_deleti  on | exon2 | c.52_53del | p.S18fs | . | . |
| CTC3 | 6 | 90642492 | CG | C | 0.535 | NM_001170794 | BACH2 | frameshift_deleti  on | exon7 | c.2160delC | p.P720fs | . | . |
| CTC3 | 6 | 96034346 | AT | A | 0.395 | NM_024641 | MANEA | frameshift_deleti  on | exon2 | c.32delT | p.I11fs | . | . |
| CTC3 | 6 | 100368817 | TA | T | 0.429 | NM_001040179 | MCHR2 | stoploss | exon6 | c.1021delT | p.X341R | . | . |
| CTC3 | 6 | 108372370 | A | AT | 0.82 | NM_014028 | OSTM1 | frameshift_inserti on | exon4 | c.647dupA | p.N216fs | . | . |
| CTC3 | 6 | 109586149 | CG | C | 1 | NM_001277339 | LOC1009966  34 | frameshift_deleti  on | exon8 | c.1343delG | p.R448fs | . | . |
| CTC3 | 6 | 109931712 | TG | T | 0.348 | NM_001145128 | AK9 | frameshift_deleti  on | exon17 | c.1697delC | p.P566fs | . | . |
| CTC3 | 6 | 111694895 | GT | G | 1 | NM_002912 | REV3L | frameshift_deleti  on | exon14 | c.4662delA | p.K1554fs | . | . |
| CTC3 | 6 | 112029210 | AC | A | 0.304 | NM_153047 | FYN | frameshift_deleti  on | exon3 | c.357delG | p.W119fs | . | . |
| CTC3 | 6 | 116442710 | TC | T | 0.25 | NM_000493 | COL10A1 | frameshift_deleti  on | exon3 | c.568delG | p.E190fs | . | . |
| CTC3 | 6 | 116559357 | A | AT | 0.889 | NM_152729 | NT5DC1 | frameshift_inserti  on | exon10 | c.930dupT | p.Y310fs | . | . |
| CTC3 | 6 | 116600465 | T | TCAC | 1 | NM_003309 | TSPYL1 | nonframeshift_in  sertion | exon1 | c.528_529insGT  G | p.K177delinsVK | . | . |
| CTC3 | 6 | 116966933 | GT | G | 0.5 | NM_145062 | ZUFSP | frameshift_deleti  on | exon9 | c.1632delA | p.K544fs | . | . |
| CTC3 | 6 | 117641163 | AT | A | 0.5 | NM_002944 | ROS1 | frameshift_deleti  on | exon36 | c.5807delA | p.N1936fs | . | . |
| CTC3 | 6 | 117642527 | CT | C | 1 | NM_002944 | ROS1 | frameshift_deleti  on | exon35 | c.5671delA | p.S1891fs | . | . |
| CTC3 | 6 | 118791742 | CT | C | 0.571 | NM_001042475 | CEP85L | frameshift_deleti  on | exon11 | c.1979delA | p.K660fs | . | . |
| CTC3 | 6 | 119238772 | CT | C | 0.756 | NM_017696 | MCM9 | frameshift_deleti  on | exon4 | c.857delA | p.K286fs | . | . |
| CTC3 | 6 | 119324134 | TG | T | 1 | NM_001100411 | FAM184A | frameshift_deleti  on | exon9 | c.1657delC | p.Q553fs | . | . |
| CTC3 | 6 | 123332243 | C | CT | 0.333 | NM_001010852 | CLVS2 | frameshift_inserti  on | exon3 | c.504dupT | p.T168fs | . | . |
| CTC3 | 6 | 123377152 | TC | T | 0.262 | NM_001010852 | CLVS2 | frameshift_deleti  on | exon5 | c.878delC | p.S293fs | . | . |
| CTC3 | 6 | 123637604 | GC | G | 0.727 | NM_006073 | TRDN | frameshift_deleti  on | exon24 | c.1507delG | p.A503fs | . | . |
| CTC3 | 6 | 129774200 | GT | G | 0.983 | NM_000426 | LAMA2 | frameshift_deleti  on | exon46 | c.6498delT | p.S2166fs | . | . |
| CTC3 | 6 | 130454599 | GCC | G | 0.2 | NM_001007102 | L3MBTL3 | frameshift_deleti  on | exon20 | c.1895_1896del | p.A632fs | . | . |
| CTC3 | 6 | 130762131 | GA | G | 0.714 | NM_001258276 | TMEM200A | frameshift_deleti  on | exon2 | c.565delA | p.M189fs | . | . |
| CTC3 | 6 | 136470253 | AT | A | 0.357 | NM_018945 | PDE7B | frameshift_deleti  on | exon5 | c.348delT | p.D116fs | . | . |
| CTC3 | 6 | 136599248 | AGT | A | 0.381 | NM_001077440 | BCLAF1 | frameshift_deleti  on | exon4 | c.763_764del | p.T255fs | . | . |
| CTC3 | 6 | 136904874 | GC | G | 1 | NM_005923 | MAP3K5 | frameshift_deleti  on | exon24 | c.3229delG | p.A1077fs | . | . |
| CTC3 | 6 | 137323117 | GC | G | 0.468 | NM_001278723 | IL20RA | frameshift_deleti  on | exon6 | c.906delG | p.G302fs | . | . |
| CTC3 | 6 | 144081695 | TA | T | 0.69 | NM_001100164 | PHACTR2 | frameshift_deleti  on | exon5 | c.613delA | p.K205fs | . | . |
| CTC3 | 6 | 144508266 | A | AG | 0.673 | NM_003764 | STX11 | frameshift_inserti  on | exon2 | c.502_503insG | p.M168fs | . | . |
| CTC3 | 6 | 146678758 | C | CA | 0.444 | NM_001278064 | GRM1 | frameshift_inserti  on | exon5 | c.1531dupA | p.Y510fs | . | . |
| CTC3 | 6 | 147136326 | G | GA | 0.9 | NM_024694 | ADGB | frameshift_inserti  on | exon36 | c.4978dupA | p.Q1659fs | . | . |
| CTC3 | 6 | 151671462 | AC | A | 1 | NM_144497 | AKAP12 | frameshift_deleti  on | exon2 | c.1643delC | p.T548fs | . | . |
| CTC3 | 6 | 153292341 | AG | A | 0.387 | NM_001142522 | FBXO5 | frameshift_deleti  on | exon5 | c.1162delC | p.L388fs | . | . |
| CTC3 | 6 | 153293584 | GTT | G | 0.5 | NM_001142522 | FBXO5 | frameshift_deleti  on | exon4 | c.775_776del | p.N259fs | . | . |
| CTC3 | 6 | 155619624 | TC | T | 0.444 | NM_016020 | TFB1M | frameshift_deleti  on | exon3 | c.380delG | p.R127fs | . | . |
| CTC3 | 6 | 157222596 | G | GA | 0.733 | NM_017519 | ARID1B | frameshift_inserti  on | exon3 | c.1824_1825ins  A | p.P608fs | . | . |
| CTC3 | 6 | 157528641 | CA | C | 0.453 | NM_017519 | ARID1B | frameshift_deleti  on | exon19 | c.6328delA | p.K2110fs | . | . |
| CTC3 | 6 | 159190405 | CT | C | 0.636 | NM_003379 | EZR | frameshift_deleti  on | exon11 | c.1296delA | p.E432fs | . | . |

| CTC3 | 6 | 159687226 | GTT | G | 0.214 | NM_032532 | FNDC1 | frameshift_deleti  on | exon21 | c.5396_5397del | p.V1799fs | . | . |
| --- | --- | --- | --- | --- | --- | --- | --- | --- | --- | --- | --- | --- | --- |
| CTC3 | 6 | 160560897 | CTGGTA  AGT | C | 1 | NM_003057 | SLC22A1 | frameshift_deleti  on | exon7 | c.1275_1276del | p.P425fs | . | . |
| CTC3 | 6 | 167343211 | GT | G | 0.86 | NM_003730 | RNASET2 | frameshift_deleti  on | exon9 | c.635delA | p.N212fs | . | . |
| CTC3 | 6 | 167550080 | TA | T | 1 | NM_004367 | CCR6 | frameshift_deleti  on | exon3 | c.363delA | p.L121fs | . | . |
| CTC3 | 6 | 170162580 | GT | G | 0.6 | NM_001278532 | ERMARD | frameshift_deleti  on | exon8 | c.536delT | p.V179fs | . | . |
| CTC3 | 6 | 170871013 | A | ACAG | 0.875 | NM_001172085 | TBP | nonframeshift_in  sertion | exon2 | c.129_130insCA  G | p.Q43delinsQQ | . | . |
| CTC3 | 7 | 299936 | CG | C | 0.833 | NM_020223 | FAM20C | frameshift_deleti  on | exon10 | c.1746delG | p.S582fs | . | . |
| CTC3 | 7 | 1595020 | TG | T | 0.929 | NM_001097620 | TMEM184A | frameshift_deleti  on | exon2 | c.100delC | p.Q34fs | . | . |
| CTC3 | 7 | 2969676 | TG | T | 0.87 | NM_032415 | CARD11 | frameshift_deleti  on | exon12 | c.1602delC | p.A534fs | . | . |
| CTC3 | 7 | 5103631 | CA | C | 0.769 | NM_021163 | RBAK | frameshift_deleti  on | exon5 | c.545delA | p.Q182fs | . | . |
| CTC3 | 7 | 5645020 | GC | G | 0.216 | NM_003088 | FSCN1 | frameshift_deleti  on | exon5 | c.1398delC | p.G466fs | . | . |
| CTC3 | 7 | 6731569 | TA | T | 0.769 | NM_016265 | ZNF12 | frameshift_deleti  on | exon5 | c.1003delT | p.Y335fs | . | . |
| CTC3 | 7 | 19184774 | TG | T | 1 | NM_152898 | FERD3L | frameshift_deleti  on | exon1 | c.211delC | p.Q71fs | . | . |
| CTC3 | 7 | 21603991 | GT | G | 0.7 | NM_001277115 | DNAH11 | frameshift_deleti  on | exon6 | c.1171delT | p.F391fs | . | . |
| CTC3 | 7 | 21627798 | GT | G | 0.743 | NM_001277115 | DNAH11 | frameshift_deleti  on | exon10 | c.1828delT | p.Y610fs | . | . |
| CTC3 | 7 | 21945148 | TC | T | 1 | NM_001127371 | CDCA7L | frameshift_deleti  on | exon6 | c.884delG | p.R295fs | . | . |
| CTC3 | 7 | 23776644 | GA | G | 0.359 | NM_001260504 | STK31 | frameshift_deleti  on | exon8 | c.896delA | p.E299fs | . | . |
| CTC3 | 7 | 24846474 | C | CA | 1 | NM_145322 | OSBPL3 | frameshift_inserti  on | exon18 | c.2163dupT | p.A722fs | . | . |
| CTC3 | 7 | 35050091 | GC | G | 0.714 | NM_015283 | DPY19L1 | frameshift_deleti  on | exon6 | c.533delG | p.G178fs | . | . |
| CTC3 | 7 | 37934150 | GA | G | 0.5 | NM_016616 | NME8 | frameshift_deleti  on | exon16 | c.1483delA | p.K495fs | . | . |
| CTC3 | 7 | 38505815 | GT | G | 0.778 | NM_001635 | AMPH | frameshift_deleti  on | exon8 | c.623delA | p.N208fs | . | . |
| CTC3 | 7 | 39379287 | CCAG | C | 0.173 | NM_001166018 | POU6F2 | nonframeshift_de  letion | exon6 | c.559_561del | p.187_187del | . | . |
| CTC3 | 7 | 43581586 | GT | G | 1 | NM_001287059 | HECW1 | frameshift_deleti  on | exon24 | c.4136delT | p.V1379fs | . | . |
| CTC3 | 7 | 43684854 | GC | G | 0.8 | NM_018224 | COA1 | frameshift_deleti  on | exon4 | c.259delG | p.A87fs | . | . |
| CTC3 | 7 | 47333389 | CA | C | 0.324 | NM_022748 | TNS3 | stopgain | exon25 | c.3713delT | p.L1238X | . | . |
| CTC3 | 7 | 47886619 | GT | G | 0.506 | NM_138295 | PKD1L1 | frameshift_deleti on | exon32 | c.5010delA | p.K1670fs | . | . |
| CTC3 | 7 | 50595918 | AG | A | 0.5 | NM_001242888 | DDC | frameshift_deleti  on | exon4 | c.396delC | p.P132fs | . | . |
| CTC3 | 7 | 51098567 | GTCT | G | 0.955 | NM_015198 | COBL | nonframeshift_de  letion | exon9 | c.1443_1445del | p.481_482del | . | . |
| CTC3 | 7 | 56141891 | GA | G | 0.5 | NM_001146333 | SUMF2 | frameshift_deleti  on | exon3 | c.101delA | p.E34fs | . | . |
| CTC3 | 7 | 63808667 | CA | C | 0.417 | NM_001170905 | ZNF736 | frameshift_deleti  on | exon5 | c.427delA | p.K143fs | . | . |
| CTC3 | 7 | 64168428 | TG | T | 0.613 | NM_001282360 | ZNF107 | frameshift_deleti  on | exon3 | c.1858delG | p.E620fs | . | . |
| CTC3 | 7 | 64168439 | GA | G | 0.357 | NM_001282360 | ZNF107 | frameshift_deleti  on | exon3 | c.1869delA | p.G623fs | . | . |
| CTC3 | 7 | 64451918 | G | GT | 1 | NM_001007253 | ERV3-1 | frameshift_inserti  on | exon2 | c.1486dupA | p.T496fs | . | . |
| CTC3 | 7 | 65419353 | TTTA | T | 0.6 | NM_001284342 | VKORC1L1 | nonframeshift_de  letion | exon2 | c.488_490del | p.163_164del | . | . |
| CTC3 | 7 | 66410101 | TC | T | 0.458 | NM_017994 | TMEM248 | frameshift_deleti  on | exon3 | c.299delC | p.S100fs | . | . |
| CTC3 | 7 | 72754753 | AC | A | 0.972 | NM_001281304 | FKBP6 | frameshift_deleti  on | exon5 | c.613delC | p.P205fs | . | . |
| CTC3 | 7 | 75186049 | G | GA | 0.288 | NM_001243198 | HIP1 | frameshift_inserti  on | exon17 | c.1647dupT | p.Q550fs | . | . |
| CTC3 | 7 | 75614493 | GC | G | 1 | NM_000941 | POR | frameshift_deleti  on | exon12 | c.1367delC | p.A456fs | . | . |
| CTC3 | 7 | 77756614 | TG | T | 0.218 | NM_012301 | MAGI2 | frameshift_deleti  on | exon19 | c.3322delC | p.Q1108fs | . | . |
| CTC3 | 7 | 81593615 | CT | C | 0.273 | NM_000722 | CACNA2D1 | frameshift_deleti  on | exon33 | c.2634delA | p.S878fs | . | . |
| CTC3 | 7 | 83643640 | ATGAGG | A | 0.273 | NM_006080 | SEMA3A | frameshift_deleti  on | exon7 | c.690_694del | p.H230fs | . | . |
| CTC3 | 7 | 86594325 | CA | C | 0.389 | NM_001142749 | KIAA1324L | frameshift_deleti  on | exon2 | c.204delT | p.F68fs | . | . |
| CTC3 | 7 | 87056165 | TG | T | 0.811 | NM_000443 | ABCB4 | frameshift_deleti  on | exon16 | c.1964delC | p.P655fs | . | . |
| CTC3 | 7 | 87138794 | GC | G | 0.658 | NM_000927 | ABCB1 | frameshift_deleti  on | exon27 | c.3285delG | p.L1095fs | . | . |
| CTC3 | 7 | 87214889 | CA | C | 0.75 | NM_000927 | ABCB1 | frameshift_deleti  on | exon5 | c.224delT | p.M75fs | . | . |
| CTC3 | 7 | 88965297 | AC | A | 1 | NM_181646 | ZNF804B | frameshift_deleti  on | exon4 | c.3002delC | p.T1001fs | . | . |
| CTC3 | 7 | 88965624 | GT | G | 0.194 | NM_181646 | ZNF804B | frameshift_deleti  on | exon4 | c.3329delT | p.V1110fs | . | . |
| CTC3 | 7 | 91830100 | GT | G | 0.318 | NM_001013406 | KRIT1 | frameshift_deleti  on | exon17 | c.2016delA | p.K672fs | . | . |
| CTC3 | 7 | 92017174 | TA | T | 1 | NM_019004 | ANKIB1 | frameshift_deleti  on | exon13 | c.1816delA | p.K606fs | . | . |
| CTC3 | 7 | 92764915 | T | TCG | 0.263 | NM_152703 | SAMD9L | frameshift_inserti  on | exon5 | c.369_370insC  G | p.R124fs | . | . |
| CTC3 | 7 | 92848564 | AT | A | 0.231 | NM_001288810 | HEPACAM2 | frameshift_deleti  on | exon1 | c.243delA | p.E81fs | . | . |
| CTC3 | 7 | 93628484 | AT | A | 1 | NM_005868 | BET1 | frameshift_deleti  on | exon2 | c.141delA | p.K47fs | . | . |
| CTC3 | 7 | 94292688 | AG | A | 0.857 | NM_001172438 | PEG10 | frameshift_deleti  on | exon2 | c.49delG | p.G17fs | . | . |
| CTC3 | 7 | 95799425 | CA | C | 1 | NM_001160210 | SLC25A13 | frameshift_deleti  on | exon13 | c.1245delT | p.F415fs | . | . |
| CTC3 | 7 | 95813649 | T | TA | 0.56 | NM_001160210 | SLC25A13 | stopgain | exon11 | c.1119_1120ins  T | p.N374_S375de  linsX | . | . |
| CTC3 | 7 | 96747051 | GTTT | G | 0.941 | NM_020186 | ACN9 | nonframeshift_de  letion | exon1 | c.17_19del | p.6_7del | . | . |
| CTC3 | 7 | 99092184 | C | CA | 0.684 | NM_032164 | ZNF394 | frameshift_inserti  on | exon3 | c.653_654insT | p.Q218fs | . | . |

| CTC3 | 7 | 99092185 | TG | T | 0.826 | NM_032164 | ZNF394 | frameshift_deleti  on | exon3 | c.652delC | p.Q218fs | . | . |
| --- | --- | --- | --- | --- | --- | --- | --- | --- | --- | --- | --- | --- | --- |
| CTC3 | 7 | 99631578 | AC | A | 0.875 | NM_001287055 | ZKSCAN1 | frameshift_deleti  on | exon4 | c.812delC | p.T271fs | . | . |
| CTC3 | 7 | 99700528 | AG | A | 0.607 | NM_004722 | AP4M1 | frameshift_deleti  on | exon4 | c.297delG | p.E99fs | . | . |
| CTC3 | 7 | 100304565 | TA | T | 0.966 | NM_005837 | POP7 | frameshift_deleti  on | exon2 | c.113delA | p.Y38fs | . | . |
| CTC3 | 7 | 100482039 | CAGG | C | 0.156 | NM_001128852 | SRRT | nonframeshift_de  letion | exon7 | c.809_811del | p.270_271del | . | . |
| CTC3 | 7 | 100732112 | T | TC | 1 | NM_030961 | TRIM56 | frameshift_inserti  on | exon3 | c.1520dupC | p.S507fs | . | . |
| CTC3 | 7 | 102109392 | CG | C | 0.6 | NM_152892 | LRWD1 | frameshift_deleti  on | exon9 | c.1112delG | p.R371fs | . | . |
| CTC3 | 7 | 103143499 | CT | C | 0.984 | NM_005045 | RELN | frameshift_deleti  on | exon52 | c.8452delA | p.R2818fs | . | . |
| CTC3 | 7 | 104702682 | GC | G | 1 | NM_018682 | KMT2E | frameshift_deleti  on | exon3 | c.144delC | p.S48fs | . | . |
| CTC3 | 7 | 104752404 | CA | C | 0.394 | NM_018682 | KMT2E | frameshift_deleti  on | exon26 | c.4202delA | p.Q1401fs | . | . |
| CTC3 | 7 | 105254397 | AT | A | 0.462 | NM_138495 | ATXN7L1 | frameshift_deleti  on | exon8 | c.2011delA | p.M671fs | . | . |
| CTC3 | 7 | 105278963 | CT | C | 0.466 | NM_138495 | ATXN7L1 | frameshift_deleti  on | exon5 | c.666delA | p.K222fs | . | . |
| CTC3 | 7 | 107671285 | TC | T | 1 | NM_007356 | LAMB4 | frameshift_deleti  on | exon32 | c.4957delG | p.E1653fs | . | . |
| CTC3 | 7 | 111368690 | GT | G | 0.562 | NM_014705 | DOCK4 | frameshift_deleti  on | exon52 | c.5540delA | p.Y1847fs | . | . |
| CTC3 | 7 | 117022162 | TA | T | 1 | NM_130768 | ASZ1 | frameshift_deleti  on | exon8 | c.848delT | p.L283fs | . | . |
| CTC3 | 7 | 119915592 | G | GT | 1 | NM_012281 | KCND2 | frameshift_inserti  on | exon1 | c.907dupT | p.K302fs | . | . |
| CTC3 | 7 | 121651563 | AT | A | 1 | NM_002851 | PTPRZ1 | frameshift_deleti  on | exon12 | c.2464delT | p.F822fs | . | . |
| CTC3 | 7 | 124386780 | GA | G | 0.275 | NM_005302 | GPR37 | frameshift_deleti  on | exon2 | c.1640delT | p.V547fs | . | . |
| CTC3 | 7 | 128587351 | CACTCT  GCAGCC GCCCAC TCTGCG | C | 1 | NM_001098627 | IRF5 | nonframeshift_de letion | exon6 | c.502_531del | p.168_177del | . | . |
| CTC3 | 7 | 128852015 | C | CA | 0.368 | NM_005631 | SMO | frameshift_inserti  on | exon12 | c.2087_2088ins  A | p.P696fs | . | . |
| CTC3 | 7 | 129986391 | TG | T | 0.303 | NM_001127442 | CPA5 | frameshift_deleti  on | exon2 | c.66delG | p.L22fs | . | . |
| CTC3 | 7 | 134890791 | TC | T | 0.573 | NM_014149 | WDR91 | frameshift_deleti  on | exon5 | c.613delG | p.E205fs | . | . |
| CTC3 | 7 | 135312763 | AG | A | 0.8 | NM_015135 | NUP205 | frameshift_deleti  on | exon34 | c.4837delG | p.V1613fs | . | . |
| CTC3 | 7 | 137597748 | GA | G | 1 | NM_001253775 | CREB3L2 | frameshift_deleti  on | exon4 | c.571delT | p.S191fs | . | . |
| CTC3 | 7 | 137612913 | CTGG | C | 1 | NM_001253775 | CREB3L2 | nonframeshift_de  letion | exon2 | c.299_301del | p.100_101del | . | . |
| CTC3 | 7 | 138732538 | GT | G | 0.714 | NM_020119 | ZC3HAV1 | frameshift_deleti  on | exon13 | c.2510delA | p.N837fs | . | . |
| CTC3 | 7 | 138946186 | TG | T | 0.68 | NM_173569 | UBN2 | frameshift_deleti  on | exon6 | c.1095delG | p.L365fs | . | . |
| CTC3 | 7 | 139416514 | AC | A | 1 | NM_001113239 | HIPK2 | frameshift_deleti  on | exon2 | c.319delG | p.V107fs | . | . |
| CTC3 | 7 | 140301356 | TTG | T | 0.423 | NM_015689 | DENND2A | frameshift_deleti  on | exon1 | c.840_841del | p.D280fs | . | . |
| CTC3 | 7 | 141373932 | CT | C | 1 | NM_001080392 | KIAA1147 | frameshift_deleti  on | exon4 | c.615delA | p.R205fs | . | . |
| CTC3 | 7 | 142563326 | G | GC | 0.529 | NM_001280795 | EPHB6 | frameshift_inserti  on | exon4 | c.168dupC | p.C56fs | . | . |
| CTC3 | 7 | 143039491 | TC | T | 1 | NM_000083 | CLCN1 | frameshift_deleti  on | exon16 | c.1824delC | p.I608fs | . | . |
| CTC3 | 7 | 143748184 | TG | T | 1 | NM_012365 | OR2A5 | frameshift_deleti  on | exon1 | c.691delG | p.G231fs | . | . |
| CTC3 | 7 | 150696055 | AC | A | 1 | NM_001160109 | NOS3 | frameshift_deleti  on | exon7 | c.839delC | p.T280fs | . | . |
| CTC3 | 7 | 150698667 | CG | C | 0.444 | NM_001160109 | NOS3 | frameshift_deleti  on | exon11 | c.1465delG | p.G489fs | . | . |
| CTC3 | 7 | 155534784 | CA | C | 1 | NM_053043 | RBM33 | frameshift_deleti  on | exon13 | c.2322delA | p.A774fs | . | . |
| CTC3 | 7 | 158486002 | AT | A | 0.5 | NM_001281933 | NCAPG2 | stopgain | exon3 | c.265delA | p.M89X | . | . |
| CTC3 | 7 | 158695125 | CT | C | 0.5 | NM_018051 | WDR60 | frameshift_deleti on | exon10 | c.1197delT | p.P399fs | . | . |
| CTC3 | 8 | 2054077 | A | AAC | 1 | NM_003970 | MYOM2 | stopgain | exon22 | c.2780_2781ins  AC | p.Y927_L928del  insX | . | . |
| CTC3 | 8 | 2054079 | C | CTA | 1 | NM_003970 | MYOM2 | frameshift_inserti  on | exon22 | c.2782_2783ins  TA | p.L928fs | . | . |
| CTC3 | 8 | 3200858 | TG | T | 0.917 | NM_033225 | CSMD1 | frameshift_deleti  on | exon23 | c.3588delC | p.T1196fs | . | . |
| CTC3 | 8 | 7215675 | GT | G | 0.361 | NM_001164457 | ZNF705G | frameshift_deleti  on | exon5 | c.725delA | p.N242fs | . | . |
| CTC3 | 8 | 8234868 | C | CGCCG  CT | 1 | NM_001080826 | SGK223 | nonframeshift_in  sertion | exon2 | c.1050_1051ins  AGCGGC | p.A351delinsS  GA | . | . |
| CTC3 | 8 | 8887542 | TAAC | T | 1 | NM_153332 | ERI1 | frameshift_deleti  on | exon7 | c.1049_1310del | p.*350fs | . | . |
| CTC3 | 8 | 9413644 | GC | G | 0.943 | NM_003747 | TNKS | frameshift_deleti  on | exon1 | c.196delC | p.P66fs | . | . |
| CTC3 | 8 | 9577981 | AC | A | 0.579 | NM_003747 | TNKS | frameshift_deleti  on | exon12 | c.1848delC | p.D616fs | . | . |
| CTC3 | 8 | 10467272 | CCTCTG  CGGGGC | C | 1 | NM_178857 | RP1L1 | frameshift_deleti  on | exon4 | c.4319_4335del | p.E1440fs | . | . |
| CTC3 | 8 | 11282019 | CGTA | C | 1 | NM_053279 | FAM167A | nonframeshift_de  letion | exon3 | c.505_507del | p.169_169del | . | . |
| CTC3 | 8 | 11990997 | CT | C | 0.873 | NM_001256869 | USP17L7 | frameshift_deleti  on | exon1 | c.521delA | p.K174fs | . | . |
| CTC3 | 8 | 11995287 | CA | C | 0.976 | NM_201402 | USP17L2 | frameshift_deleti  on | exon1 | c.982delT | p.W328fs | . | . |
| CTC3 | 8 | 17402079 | G | GA | 1 | NM_001008539 | SLC7A2 | frameshift_inserti  on | exon3 | c.497dupA | p.D166fs | . | . |
| CTC3 | 8 | 17872098 | CA | C | 0.5 | NM_006197 | PCM1 | frameshift_deleti  on | exon36 | c.5591delA | p.Q1864fs | . | . |
| CTC3 | 8 | 18729816 | C | CA | 0.235 | NM_015310 | PSD3 | frameshift_inserti  on | exon3 | c.557_558insT | p.T186fs | . | . |
| CTC3 | 8 | 20110325 | AG | A | 0.286 | NM_021020 | LZTS1 | frameshift_deleti  on | exon2 | c.1116delC | p.S372fs | . | . |
| CTC3 | 8 | 22102973 | GA | G | 0.348 | NM_001722 | POLR3D | frameshift_deleti  on | exon2 | c.12delA | p.G4fs | . | . |
| CTC3 | 8 | 22464726 | A | AT | 0.276 | NM_021174 | CCAR2 | frameshift_inserti  on | exon6 | c.375_376insT | p.P125fs | . | . |

| CTC3 | 8 | 23003260 | TA | T | 0.344 | NM_003840 | TNFRSF10D | stopgain | exon5 | c.656delT | p.L219X | . | . |
| --- | --- | --- | --- | --- | --- | --- | --- | --- | --- | --- | --- | --- | --- |
| CTC3 | 8 | 23306309 | AC | A | 0.969 | NM_001128930 | ENTPD4 | frameshift_deleti on | exon3 | c.151delG | p.V51fs | . | . |
| CTC3 | 8 | 24249827 | T | TA | 0.944 | NM_014479 | ADAMDEC1 | frameshift_inserti  on | exon2 | c.142dupA | p.P47fs | . | . |
| CTC3 | 8 | 27312052 | TG | T | 0.928 | NM_173175 | PTK2B | frameshift_deleti  on | exon29 | c.2613delG | p.V871fs | . | . |
| CTC3 | 8 | 33449726 | C | CT | 0.974 | NM_024025 | DUSP26 | frameshift_inserti  on | exon4 | c.440dupA | p.K147fs | . | . |
| CTC3 | 8 | 37795213 | ACTGG | A | 0.2 | NM_152413 | GOT1L1 | frameshift_deleti  on | exon3 | c.348_351del | p.V116fs | . | . |
| CTC3 | 8 | 38091988 | TG | T | 1 | NM_001164232 | DDHD2 | frameshift_deleti  on | exon3 | c.298delG | p.A100fs | . | . |
| CTC3 | 8 | 38117607 | C | CA | 0.333 | NM_001164232 | DDHD2 | frameshift_inserti  on | exon17 | c.2105dupA | p.Q702fs | . | . |
| CTC3 | 8 | 41543723 | TG | T | 0.4 | NM_000037 | ANK1 | frameshift_deleti  on | exon36 | c.4336delC | p.Q1446fs | . | . |
| CTC3 | 8 | 41834626 | AG | A | 0.333 | NM_006766 | KAT6A | frameshift_deleti  on | exon7 | c.1262delC | p.P421fs | . | . |
| CTC3 | 8 | 42036480 | T | TG | 0.5 | NM_033011 | PLAT | frameshift_inserti  on | exon12 | c.1326dupC | p.N443fs | . | . |
| CTC3 | 8 | 42231780 | CA | C | 0.36 | NM_014420 | DKK4 | frameshift_deleti  on | exon4 | c.512delT | p.L171fs | . | . |
| CTC3 | 8 | 42294675 | ACGC | A | 0.48 | NM_001257180 | SLC20A2 | nonframeshift_de  letion | exon8 | c.1352_1354del | p.451_452del | . | . |
| CTC3 | 8 | 55533549 | GT | G | 0.467 | NM_006269 | RP1 | frameshift_deleti  on | exon2 | c.24delT | p.G8fs | . | . |
| CTC3 | 8 | 59404943 | TG | T | 0.796 | NM_000780 | CYP7A1 | frameshift_deleti  on | exon5 | c.1183delC | p.H395fs | . | . |
| CTC3 | 8 | 59555627 | G | GA | 0.375 | NM_001144772 | NSMAF | frameshift_inserti  on | exon2 | c.157dupT | p.S53fs | . | . |
| CTC3 | 8 | 61768722 | TG | T | 1 | NM_017780 | CHD7 | frameshift_deleti  on | exon33 | c.7126delG | p.G2376fs | . | . |
| CTC3 | 8 | 61778197 | CG | C | 0.941 | NM_017780 | CHD7 | frameshift_deleti  on | exon38 | c.8700delG | p.P2900fs | . | . |
| CTC3 | 8 | 67356849 | TA | T | 0.785 | NM_144650 | ADHFE1 | frameshift_deleti  on | exon5 | c.220delA | p.K74fs | . | . |
| CTC3 | 8 | 68956818 | T | TG | 0.208 | NM_024870 | PREX2 | frameshift_inserti  on | exon8 | c.937dupG | p.D312fs | . | . |
| CTC3 | 8 | 71646433 | TG | T | 0.455 | NM_001011720 | XKR9 | frameshift_deleti  on | exon5 | c.897delG | p.L299fs | . | . |
| CTC3 | 8 | 73480052 | TC | T | 0.661 | NM_004770 | KCNB2 | frameshift_deleti  on | exon2 | c.84delC | p.I28fs | . | . |
| CTC3 | 8 | 73480452 | GC | G | 0.231 | NM_004770 | KCNB2 | frameshift_deleti  on | exon2 | c.484delC | p.R162fs | . | . |
| CTC3 | 8 | 73849598 | GC | G | 0.895 | NM_004770 | KCNB2 | frameshift_deleti  on | exon3 | c.2009delC | p.A670fs | . | . |
| CTC3 | 8 | 76476330 | GA | G | 0.348 | NM_004133 | HNF4G | nonframeshift_de  letion | exon10 | c.1338delA | p.X446X | . | . |
| CTC3 | 8 | 77768138 | CT | C | 1 | NM_024721 | ZFHX4 | frameshift_deleti  on | exon10 | c.8982delT | p.P2994fs | . | . |
| CTC3 | 8 | 82196156 | GA | G | 0.875 | NM_001444 | FABP5 | frameshift_deleti  on | exon3 | c.302delA | p.D101fs | . | . |
| CTC3 | 8 | 82627096 | GT | G | 0.706 | NM_001170796 | ZFAND1 | frameshift_deleti  on | exon5 | c.300delA | p.K100fs | . | . |
| CTC3 | 8 | 86126827 | C | CAACA  TT | 1 | NM_001099673 | C8orf59 | nonframeshift_in  sertion | exon4 | c.264_265insAA  TGTT | p.D89delinsNV  D | . | . |
| CTC3 | 8 | 91049080 | C | CT | 0.345 | NM_001359 | DECR1 | frameshift_inserti  on | exon6 | c.578dupT | p.L193fs | . | . |
| CTC3 | 8 | 93107308 | A | AG | 0.899 | NM_001198679 | RUNX1T1 | frameshift_inserti  on | exon2 | c.212dupC | p.P71fs | . | . |
| CTC3 | 8 | 94784865 | GC | G | 0.8 | NM_153704 | TMEM67 | frameshift_deleti  on | exon7 | c.701delC | p.A234fs | . | . |
| CTC3 | 8 | 95952408 | C | CTCT | 0.625 | NM_001135733 | TP53INP1 | nonframeshift_in  sertion | exon3 | c.152_153insA  GA | p.E51delinsEE | . | . |
| CTC3 | 8 | 99560295 | TG | T | 0.967 | NM_001256313 | STK3 | frameshift_deleti  on | exon7 | c.709delC | p.Q237fs | . | . |
| CTC3 | 8 | 101076228 | CT | C | 0.767 | NM_001286693 | RGS22 | frameshift_deleti  on | exon6 | c.224delA | p.K75fs | . | . |
| CTC3 | 8 | 101232585 | G | GA | 1 | NM_003114 | SPAG1 | frameshift_inserti  on | exon13 | c.1615dupA | p.G538fs | . | . |
| CTC3 | 8 | 101232588 | A | AG | 1 | NM_003114 | SPAG1 | frameshift_inserti  on | exon13 | c.1618dupG | p.K539fs | . | . |
| CTC3 | 8 | 104053182 | A | AT | 0.4 | NM_001695 | ATP6V1C1 | frameshift_inserti  on | exon2 | c.119dupT | p.I40fs | . | . |
| CTC3 | 8 | 110400747 | C | CA | 0.231 | NM_177531 | PKHD1L1 | frameshift_inserti  on | exon7 | c.591_592insA | p.P197fs | . | . |
| CTC3 | 8 | 110422171 | G | GA | 0.933 | NM_177531 | PKHD1L1 | frameshift_inserti  on | exon19 | c.2050dupA | p.V683fs | . | . |
| CTC3 | 8 | 110457731 | AT | A | 0.267 | NM_177531 | PKHD1L1 | frameshift_deleti  on | exon38 | c.5634delT | p.N1878fs | . | . |
| CTC3 | 8 | 113662400 | TG | T | 0.4 | NM_052900 | CSMD3 | frameshift_deleti  on | exon18 | c.2870delC | p.P957fs | . | . |
| CTC3 | 8 | 121237396 | AT | A | 1 | NM_021110 | COL14A1 | frameshift_deleti  on | exon15 | c.1808delT | p.I603fs | . | . |
| CTC3 | 8 | 121530160 | TC | T | 0.8 | NM_022045 | MTBP | frameshift_deleti  on | exon19 | c.2317delC | p.H773fs | . | . |
| CTC3 | 8 | 125989259 | G | GA | 0.72 | NM_152412 | ZNF572 | frameshift_inserti  on | exon3 | c.750dupA | p.G250fs | . | . |
| CTC3 | 8 | 125989323 | TTA | T | 0.565 | NM_152412 | ZNF572 | frameshift_deleti  on | exon3 | c.814_815del | p.Y272fs | . | . |
| CTC3 | 8 | 133101784 | T | TA | 0.762 | NM_001145095 | HHLA1 | stopgain | exon7 | c.510dupT | p.K171_S172de  linsX | . | . |
| CTC3 | 8 | 143694647 | AG | A | 0.304 | NM_015193 | ARC | frameshift_deleti  on | exon1 | c.985delC | p.L329fs | . | . |
| CTC3 | 8 | 144296975 | C | CA | 0.412 | NM_178172 | GPIHBP1 | frameshift_inserti  on | exon3 | c.270dupA | p.T90fs | . | . |
| CTC3 | 8 | 145737697 | A | AG | 0.875 | NM_004260 | RECQL4 | frameshift_inserti  on | exon19 | c.3065_3066ins  C | p.R1022fs | . | . |
| CTC3 | 8 | 145770714 | AG | A | 0.333 | NM_025251 | ARHGAP39 | frameshift_deleti  on | exon7 | c.2439delC | p.A813fs | . | . |
| CTC3 | 8 | 145999616 | CTTT | C | 0.199 | NM_001286770 | ZNF34 | nonframeshift_de  letion | exon5 | c.595_597del | p.199_199del | . | . |
| CTC3 | 8 | 146157068 | TG | T | 0.774 | NM_006958 | ZNF16 | frameshift_deleti  on | exon3 | c.1104delC | p.H368fs | . | . |
| CTC3 | 9 | 732474 | AGAG | A | 0.149 | NM_153186 | KANK1 | nonframeshift_de  letion | exon5 | c.2629_2631del | p.877_877del | . | . |
| CTC3 | 9 | 2191377 | AT | A | 0.429 | NM_001289398 | SMARCA2 | frameshift_deleti  on | exon6 | c.681delT | p.D227fs | . | . |
| CTC3 | 9 | 3228878 | TA | T | 0.667 | NM_001282116 | RFX3 | stopgain | exon16 | c.1979delT | p.L660X | . | . |
| CTC3 | 9 | 3257004 | AT | A | 1 | NM_002919 | RFX3 | frameshift_deleti on | exon13 | c.1800delA | p.K600fs | . | . |

| CTC3 | 9 | 5797926 | GT | G | 1 | NM_024896 | ERMP1 | frameshift_deleti  on | exon13 | c.2276delA | p.N759fs | . | . |
| --- | --- | --- | --- | --- | --- | --- | --- | --- | --- | --- | --- | --- | --- |
| CTC3 | 9 | 6986592 | CA | C | 0.182 | NM_001146694 | KDM4C | frameshift_deleti  on | exon11 | c.1604delA | p.H535fs | . | . |
| CTC3 | 9 | 12702386 | C | CA | 0.419 | NM_000550 | TYRP1 | frameshift_inserti  on | exon5 | c.1030dupA | p.D343fs | . | . |
| CTC3 | 9 | 12775861 | T | TGGCG  GCGGC | 1 | NM_203403 | LURAP1L | nonframeshift_in  sertion | exon1 | c.147_148insG  GCGGCGGC | p.G49delinsGG  GG | . | . |
| CTC3 | 9 | 12775861 | T | TGGCG  GC | 1 | NM_203403 | LURAP1L | nonframeshift_in  sertion | exon1 | c.147_148insG  GCGGC | p.G49delinsGG  G | . | . |
| CTC3 | 9 | 12775873 | C | CGGCG  GCGGT | 1 | NM_203403 | LURAP1L | nonframeshift_in  sertion | exon1 | c.159_160insG  GCGGCGGT | p.G53delinsGG  GG | . | . |
| CTC3 | 9 | 18928083 | GT | G | 0.8 | NM_001287049 | FAM154A | frameshift_deleti  on | exon4 | c.1196delA | p.N399fs | . | . |
| CTC3 | 9 | 20823089 | GT | G | 0.35 | NM_017794 | FOCAD | frameshift_deleti  on | exon17 | c.1896delT | p.G632fs | . | . |
| CTC3 | 9 | 26907857 | CA | C | 0.4 | NM_001031689 | PLAA | frameshift_deleti  on | exon13 | c.1796delT | p.L599fs | . | . |
| CTC3 | 9 | 27950205 | CA | C | 0.326 | NM_001258282 | LINGO2 | frameshift_deleti  on | exon7 | c.464delT | p.L155fs | . | . |
| CTC3 | 9 | 33547979 | AT | A | 0.298 | NM_001244752 | ANKRD18B | frameshift_deleti  on | exon9 | c.1005delT | p.N335fs | . | . |
| CTC3 | 9 | 33548141 | C | CA | 0.471 | NM_001244752 | ANKRD18B | frameshift_inserti  on | exon9 | c.1167dupA | p.T389fs | . | . |
| CTC3 | 9 | 33943556 | GA | G | 0.667 | NM_001282529 | UBAP2 | frameshift_deleti  on | exon11 | c.775delT | p.S259fs | . | . |
| CTC3 | 9 | 34257242 | CT | C | 0.667 | NM_194313 | KIF24 | frameshift_deleti  on | exon11 | c.2362delA | p.R788fs | . | . |
| CTC3 | 9 | 34647216 | CA | C | 0.667 | NM_000155 | GALT | frameshift_deleti  on | exon2 | c.214delA | p.N72fs | . | . |
| CTC3 | 9 | 35650626 | A | AG | 0.857 | NM_014450 | SIT1 | frameshift_inserti  on | exon2 | c.108dupC | p.S37fs | . | . |
| CTC3 | 9 | 35802738 | GA | G | 0.561 | NM_003995 | NPR2 | frameshift_deleti  on | exon12 | c.1826delA | p.E609fs | . | . |
| CTC3 | 9 | 35811485 | ACCAGG  ACCAGA | A | 1 | NM_001039592 | SPAG8 | nonframeshift_de  letion | exon2 | c.546_557del | p.182_186del | . | . |
| CTC3 | 9 | 36344831 | AG | A | 0.235 | NM_194329 | RNF38 | frameshift_deleti  on | exon9 | c.1232delC | p.T411fs | . | . |
| CTC3 | 9 | 37769960 | GC | G | 0.184 | NM_001286952 | TRMT10B | frameshift_deleti  on | exon4 | c.363delC | p.C121fs | . | . |
| CTC3 | 9 | 71395455 | CA | C | 0.696 | NM_138333 | FAM122A | frameshift_deleti  on | exon1 | c.376delA | p.N126fs | . | . |
| CTC3 | 9 | 74838067 | GT | G | 1 | NM_001242505 | GDA | frameshift_deleti  on | exon7 | c.639delT | p.R213fs | . | . |
| CTC3 | 9 | 75784978 | AG | A | 0.194 | NM_000700 | ANXA1 | frameshift_deleti  on | exon13 | c.997delG | p.G333fs | . | . |
| CTC3 | 9 | 77390952 | AC | A | 0.481 | NM_001177310 | TRPM6 | frameshift_deleti  on | exon24 | c.3234delG | p.L1078fs | . | . |
| CTC3 | 9 | 79318375 | GGTGAC  AGCCTG | G | 1 | NM_015225 | PRUNE2 | nonframeshift_de  letion | exon9 | c.8139_8153del | p.2713_2718de  l | . | . |
| CTC3 | 9 | 84607032 | TC | T | 0.972 | NM_001001670 | SPATA31D1 | frameshift_deleti  on | exon4 | c.1648delC | p.P550fs | . | . |
| CTC3 | 9 | 87342711 | T | TA | 0.875 | NM_001007097 | NTRK2 | frameshift_inserti  on | exon9 | c.997dupA | p.T332fs | . | . |
| CTC3 | 9 | 90255219 | TG | T | 0.346 | NM_001288729 | DAPK1 | frameshift_deleti  on | exon8 | c.637delG | p.G213fs | . | . |
| CTC3 | 9 | 90538349 | G | GCCC | 0.138 | NM_001145124 | SPATA31C1 | nonframeshift_in  sertion | exon4 | c.3527_3528ins  CCC | p.R1176delinsR  P | . | . |
| CTC3 | 9 | 96291772 | GC | G | 1 | NM_001286722 | FAM120A | frameshift_deleti  on | exon9 | c.1642delC | p.P548fs | . | . |
| CTC3 | 9 | 96428118 | GA | G | 1 | NM_005392 | PHF2 | frameshift_deleti  on | exon15 | c.2089delA | p.K697fs | . | . |
| CTC3 | 9 | 100092962 | G | GGAGG  AC | 0.708 | NM_020893 | CCDC180 | nonframeshift_in  sertion | exon18 | c.2319_2320ins  GAGGAC | p.K773delinsKE  D | . | . |
| CTC3 | 9 | 100092968 | A | AGAGG  AG | 1 | NM_020893 | CCDC180 | nonframeshift_in  sertion | exon18 | c.2325_2326ins  GAGGAG | p.E775delinsEE  E | . | . |
| CTC3 | 9 | 100092968 | A | AGAG | 1 | NM_020893 | CCDC180 | nonframeshift_in  sertion | exon18 | c.2325_2326ins  GAG | p.E775delinsEE | . | . |
| CTC3 | 9 | 100092972 | G | GAGGA  GT | 0.898 | NM_020893 | CCDC180 | stopgain | exon18 | c.2329_2330ins  AGGAGT | p.E777delinsEE  X | . | . |
| CTC3 | 9 | 100092973 | A | AGGAG  GG | 0.915 | NM_020893 | CCDC180 | nonframeshift_in  sertion | exon18 | c.2330_2331ins  GGAGGG | p.E777delinsEE  G | . | . |
| CTC3 | 9 | 100092974 | G | GGAGG  AT | 0.913 | NM_020893 | CCDC180 | nonframeshift_in  sertion | exon18 | c.2331_2332ins  GAGGAT | p.E777delinsEE  D | . | . |
| CTC3 | 9 | 101608258 | GT | G | 0.556 | NM_024642 | GALNT12 | frameshift_deleti  on | exon9 | c.1459delT | p.F487fs | . | . |
| CTC3 | 9 | 101818545 | CA | C | 0.3 | NM_001855 | COL15A1 | frameshift_deleti  on | exon35 | c.3197delA | p.Q1066fs | . | . |
| CTC3 | 9 | 101980662 | CA | C | 0.272 | NM_033087 | ALG2 | frameshift_deleti  on | exon2 | c.804delT | p.G268fs | . | . |
| CTC3 | 9 | 107380326 | G | GC | 0.368 | NM_001001956 | OR13C9 | frameshift_inserti  on | exon1 | c.159_160insG | p.H54fs | . | . |
| CTC3 | 9 | 108136961 | CT | C | 1 | NM_001286730 | SLC44A1 | frameshift_deleti  on | exon13 | c.1578delT | p.A526fs | . | . |
| CTC3 | 9 | 112151604 | AG | A | 0.4 | NM_001145372 | PTPN3 | frameshift_deleti  on | exon11 | c.1165delC | p.L389fs | . | . |
| CTC3 | 9 | 115932119 | TGG | T | 0.261 | NM_015258 | FKBP15 | frameshift_deleti  on | exon26 | c.2868_2869del | p.S956fs | . | . |
| CTC3 | 9 | 116053207 | AC | A | 0.728 | NM_001244926 | PRPF4 | frameshift_deleti  on | exon13 | c.1284delC | p.N428fs | . | . |
| CTC3 | 9 | 116245283 | CTG | C | 0.15 | NM_001282923 | RGS3 | frameshift_deleti  on | exon3 | c.82_83del | p.C28fs | . | . |
| CTC3 | 9 | 117808802 | G | GC | 0.571 | NM_002160 | TNC | frameshift_inserti  on | exon17 | c.5011_5012ins  G | p.P1671fs | . | . |
| CTC3 | 9 | 119770526 | AC | A | 0.182 | NM_014010 | ASTN2 | frameshift_deleti  on | exon6 | c.1282delG | p.V428fs | . | . |
| CTC3 | 9 | 123216099 | TC | T | 0.849 | NM_001011649 | CDK5RAP2 | frameshift_deleti  on | exon21 | c.2427delG | p.L809fs | . | . |
| CTC3 | 9 | 123374792 | AT | A | 1 | NM_001080497 | MEGF9 | frameshift_deleti  on | exon4 | c.968delA | p.N323fs | . | . |
| CTC3 | 9 | 123782422 | AT | A | 0.6 | NM_001735 | C5 | frameshift_deleti  on | exon12 | c.1337delA | p.N446fs | . | . |
| CTC3 | 9 | 129979190 | CG | C | 0.545 | NM_001190728 | RALGPS1 | frameshift_deleti  on | exon17 | c.1501delG | p.G501fs | . | . |
| CTC3 | 9 | 130211594 | CT | C | 0.766 | NM_000976 | RPL12 | frameshift_deleti  on | exon4 | c.257delA | p.K86fs | . | . |
| CTC3 | 9 | 130939949 | GGC | G | 0.688 | NM_001257976 | CIZ1 | frameshift_deleti  on | exon9 | c.1409_1410del | p.R470fs | . | . |
| CTC3 | 9 | 131388112 | AAG | A | 0.2 | NM_001195532 | SPTAN1 | frameshift_deleti  on | exon46 | c.6060_6061del | p.K2020fs | . | . |
| CTC3 | 9 | 131388686 | TG | T | 0.571 | NM_001195532 | SPTAN1 | frameshift_deleti  on | exon47 | c.6222delG | p.L2074fs | . | . |

| CTC3 | 9 | 131469640 | AC | A | 1 | NM_013355 | PKN3 | frameshift_deleti  on | exon6 | c.792delC | p.Y264fs | . | . |
| --- | --- | --- | --- | --- | --- | --- | --- | --- | --- | --- | --- | --- | --- |
| CTC3 | 9 | 131708195 | TC | T | 0.636 | NM_014908 | DOLK | frameshift_deleti  on | exon1 | c.1387delG | p.E463fs | . | . |
| CTC3 | 9 | 133924508 | ACAG | A | 0.138 | NM_006059 | LAMC3 | nonframeshift_de  letion | exon9 | c.1622_1624del | p.541_542del | . | . |
| CTC3 | 9 | 134351134 | TC | T | 0.333 | NM_013318 | PRRC2B | frameshift_deleti  on | exon15 | c.3619delC | p.P1207fs | . | . |
| CTC3 | 9 | 135546074 | C | CA | 1 | NM_012204 | GTF3C4 | frameshift_inserti  on | exon1 | c.89_90insA | p.A30fs | . | . |
| CTC3 | 9 | 136287645 | TG | T | 0.765 | NM_139025 | ADAMTS13 | frameshift_deleti  on | exon1 | c.83delG | p.W28fs | . | . |
| CTC3 | 9 | 136523492 | AC | A | 0.375 | NM_000787 | DBH | frameshift_deleti  on | exon12 | c.1778delC | p.T593fs | . | . |
| CTC3 | 9 | 136570083 | CG | C | 0.714 | NM_001134707 | SARDH | frameshift_deleti  on | exon12 | c.1540delC | p.R514fs | . | . |
| CTC3 | 9 | 136677271 | CGTA | C | 0.123 | NM_001134398 | VAV2 | nonframeshift_de  letion | exon5 | c.514_516del | p.172_172del | . | . |
| CTC3 | 9 | 136901305 | TA | T | 1 | NM_007371 | BRD3 | frameshift_deleti  on | exon10 | c.1784delT | p.V595fs | . | . |
| CTC3 | 9 | 138706992 | CAAT | C | 0.141 | NM_015447 | CAMSAP1 | nonframeshift_de  letion | exon16 | c.4454_4456del | p.1485_1486de  l | . | . |
| CTC3 | 9 | 139390944 | TGTG | T | 0.161 | NM_017617 | NOTCH1 | nonframeshift_de  letion | exon34 | c.7244_7246del | p.2415_2416de  l | . | . |
| CTC3 | 9 | 140109584 | CT | C | 0.258 | NM_001144027 | NDOR1 | frameshift_deleti  on | exon8 | c.1002delT | p.P334fs | . | . |
| CTC3 | 9 | 140672410 | AC | A | 0.386 | NM_001145527 | EHMT1 | frameshift_deleti  on | exon13 | c.2096delC | p.T699fs | . | . |
| CTC3 | 10 | 372995 | GCC | G | 0.714 | NM_014974 | DIP2C | frameshift_deleti  on | exon31 | c.3873_3874del | p.R1291fs | . | . |
| CTC3 | 10 | 1284201 | GC | G | 0.173 | NM_018702 | ADARB2 | frameshift_deleti  on | exon5 | c.1353delG | p.L451fs | . | . |
| CTC3 | 10 | 4879709 | AC | A | 0.323 | NM_001040177 | AKR1E2 | frameshift_deleti  on | exon5 | c.519delC | p.N173fs | . | . |
| CTC3 | 10 | 5435964 | TCAG | T | 0.118 | NM_001171864 | TUBAL3 | nonframeshift_de  letion | exon4 | c.734_736del | p.245_246del | . | . |
| CTC3 | 10 | 5784098 | TG | T | 1 | NM_017782 | FAM208B | frameshift_deleti  on | exon14 | c.2367delG | p.V789fs | . | . |
| CTC3 | 10 | 5788458 | GC | G | 0.958 | NM_017782 | FAM208B | frameshift_deleti  on | exon15 | c.3075delC | p.S1025fs | . | . |
| CTC3 | 10 | 5789966 | CCTGCC  TCAG | C | 1 | NM_017782 | FAM208B | nonframeshift_de  letion | exon15 | c.4583_4591del | p.1528_1531de  l | . | . |
| CTC3 | 10 | 7745427 | CTTCT | C | 0.269 | NM_002216 | ITIH2 | frameshift_deleti  on | exon1 | c.31_34del | p.F11fs | . | . |
| CTC3 | 10 | 8007566 | A | AGGG | 0.25 | NM_031923 | TAF3 | nonframeshift_in  sertion | exon3 | c.2093_2094ins  GGG | p.E698delinsEG | . | . |
| CTC3 | 10 | 12009410 | GT | G | 0.377 | NM_015542 | UPF2 | frameshift_deleti  on | exon10 | c.1996delA | p.T666fs | . | . |
| CTC3 | 10 | 15648349 | AG | A | 0.929 | NM_003638 | ITGA8 | frameshift_deleti  on | exon18 | c.1836delC | p.T612fs | . | . |
| CTC3 | 10 | 19392911 | GT | G | 0.8 | NM_001142308 | MALRD1 | frameshift_deleti  on | exon5 | c.602delT | p.V201fs | . | . |
| CTC3 | 10 | 19546631 | G | GC | 0.157 | NM_001142308 | MALRD1 | frameshift_inserti  on | exon19 | c.3010_3011ins  C | p.V1004fs | . | . |
| CTC3 | 10 | 19572040 | TG | T | 0.514 | NM_001142308 | MALRD1 | frameshift_deleti  on | exon21 | c.3350delG | p.W1117fs | . | . |
| CTC3 | 10 | 21141548 | TG | T | 0.571 | NM_006393 | NEBL | frameshift_deleti  on | exon10 | c.933delC | p.N311fs | . | . |
| CTC3 | 10 | 24790366 | CAG | C | 0.5 | NM_001282769 | KIAA1217 | frameshift_deleti  on | exon4 | c.943_944del | p.S315fs | . | . |
| CTC3 | 10 | 24813546 | A | AT | 0.938 | NM_001282769 | KIAA1217 | frameshift_inserti  on | exon8 | c.1800_1801ins  T | p.P600fs | . | . |
| CTC3 | 10 | 25887431 | A | AG | 0.5 | NM_020752 | GPR158 | frameshift_inserti  on | exon11 | c.2876_2877ins  G | p.Q959fs | . | . |
| CTC3 | 10 | 26822390 | G | GA | 0.967 | NM_019043 | APBB1IP | frameshift_inserti  on | exon9 | c.837dupA | p.G279fs | . | . |
| CTC3 | 10 | 27324106 | AG | A | 0.643 | NM_001256053 | ANKRD26 | frameshift_deleti  on | exon24 | c.3269delC | p.T1090fs | . | . |
| CTC3 | 10 | 27389098 | GC | G | 0.2 | NM_001256053 | ANKRD26 | frameshift_deleti  on | exon1 | c.157delG | p.A53fs | . | . |
| CTC3 | 10 | 29770529 | TC | T | 0.889 | NM_003174 | SVIL | frameshift_deleti  on | exon26 | c.3805delG | p.E1269fs | . | . |
| CTC3 | 10 | 30317445 | A | AT | 0.667 | NM_020848 | KIAA1462 | frameshift_inserti  on | exon3 | c.1631_1632ins  A | p.P544fs | . | . |
| CTC3 | 10 | 44052534 | AGC | A | 0.235 | NM_001099283 | ZNF239 | frameshift_deleti  on | exon2 | c.992_993del | p.R331fs | . | . |
| CTC3 | 10 | 44112022 | TA | T | 1 | NM_145312 | ZNF485 | frameshift_deleti  on | exon5 | c.532delA | p.K178fs | . | . |
| CTC3 | 10 | 44788772 | GC | G | 0.3 | NM_001258000 | LOC1001305  39 | stopgain | exon2 | c.198delC | p.C66X | . | . |
| CTC3 | 10 | 45799658 | AG | A | 0.429 | NM_001004297 | OR13A1 | frameshift_deleti  on | exon4 | c.212delC | p.P71fs | . | . |
| CTC3 | 10 | 50272766 | AC | A | 0.278 | NM_001031746 | VSTM4 | stopgain | exon5 | c.649delG | p.V217X | . | . |
| CTC3 | 10 | 50533170 | CA | C | 1 | NM_001135196 | C10orf71 | frameshift_deleti on | exon3 | c.2581delA | p.T861fs | . | . |
| CTC3 | 10 | 52067020 | TG | T | 0.676 | NM_147156 | SGMS1 | frameshift_deleti  on | exon11 | c.1123delC | p.Q375fs | . | . |
| CTC3 | 10 | 53458635 | A | AG | 0.156 | NM_015235 | CSTF2T | frameshift_inserti  on | exon1 | c.674dupC | p.P225fs | . | . |
| CTC3 | 10 | 61834431 | GT | G | 0.923 | NM_020987 | ANK3 | frameshift_deleti  on | exon37 | c.6207delA | p.K2069fs | . | . |
| CTC3 | 10 | 69881697 | C | CA | 0.9 | NM_032578 | MYPN | frameshift_inserti  on | exon2 | c.503dupA | p.H168fs | . | . |
| CTC3 | 10 | 70098371 | AG | A | 0.4 | NM_012207 | HNRNPH3 | frameshift_deleti  on | exon4 | c.364delG | p.G122fs | . | . |
| CTC3 | 10 | 71912285 | TG | T | 0.957 | NM_020150 | SAR1A | frameshift_deleti  on | exon7 | c.543delC | p.L181fs | . | . |
| CTC3 | 10 | 72434645 | AT | A | 0.333 | NM_080722 | ADAMTS14 | frameshift_deleti  on | exon2 | c.417delT | p.D139fs | . | . |
| CTC3 | 10 | 74690383 | CA | C | 0.857 | NM_152635 | OIT3 | frameshift_deleti  on | exon8 | c.1456delA | p.K486fs | . | . |
| CTC3 | 10 | 75585051 | AG | A | 0.583 | NM_172170 | CAMK2G | frameshift_deleti  on | exon14 | c.1044delC | p.P348fs | . | . |
| CTC3 | 10 | 76790217 | AC | A | 0.879 | NM_001256468 | KAT6B | frameshift_deleti  on | exon18 | c.5087delC | p.T1696fs | . | . |
| CTC3 | 10 | 78844444 | TG | T | 0.667 | NM_001271518 | KCNMA1 | frameshift_deleti  on | exon11 | c.1311delC | p.A437fs | . | . |
| CTC3 | 10 | 85984552 | A | AT | 0.933 | NM_001017924 | LRIT2 | frameshift_inserti  on | exon2 | c.428_429insA | p.P143fs | . | . |
| CTC3 | 10 | 86273249 | TCA | T | 0.4 | NM_001284242 | CCSER2 | frameshift_deleti  on | exon7 | c.652_653del | p.H218fs | . | . |
| CTC3 | 10 | 88260066 | CA | C | 0.462 | NM_015045 | WAPAL | frameshift_deleti  on | exon3 | c.933delT | p.F311fs | . | . |

| CTC3 | 10 | 88260291 | AC | A | 0.722 | NM_015045 | WAPAL | frameshift_deleti  on | exon3 | c.708delG | p.L236fs | . | . |
| --- | --- | --- | --- | --- | --- | --- | --- | --- | --- | --- | --- | --- | --- |
| CTC3 | 10 | 89272897 | CT | C | 0.906 | NM_001178118 | MINPP1 | frameshift_deleti  on | exon3 | c.250delT | p.F84fs | . | . |
| CTC3 | 10 | 91479382 | TG | T | 0.438 | NM_001284259 | KIF20B | frameshift_deleti  on | exon13 | c.1642delG | p.V548fs | . | . |
| CTC3 | 10 | 94833823 | G | GC | 0.645 | NM_000783 | CYP26A1 | frameshift_inserti  on | exon1 | c.133dupC | p.L44fs | . | . |
| CTC3 | 10 | 95931179 | A | AT | 0.8 | NM_001165979 | PLCE1 | frameshift_inserti  on | exon3 | c.812dupT | p.I271fs | . | . |
| CTC3 | 10 | 96356709 | G | GA | 0.333 | NM_001289070 | HELLS | frameshift_inserti  on | exon18 | c.2048dupA | p.E683fs | . | . |
| CTC3 | 10 | 96447887 | CT | C | 0.292 | NM_000772 | CYP2C18 | frameshift_deleti  on | exon3 | c.338delT | p.L113fs | . | . |
| CTC3 | 10 | 98130068 | GCA | G | 1 | NM_012465 | TLL2 | frameshift_deleti  on | exon20 | c.2665_2666del | p.C889fs | . | . |
| CTC3 | 10 | 98145876 | AC | A | 0.372 | NM_012465 | TLL2 | frameshift_deleti  on | exon15 | c.1948delG | p.V650fs | . | . |
| CTC3 | 10 | 99025803 | C | CA | 0.407 | NM_001204300 | ARHGAP19 | frameshift_inserti  on | exon2 | c.135dupT | p.V46fs | . | . |
| CTC3 | 10 | 99125930 | TCGG | T | 0.159 | NM_001284337 | RRP12 | nonframeshift_de  letion | exon26 | c.3149_3151del | p.1050_1051de  l | . | . |
| CTC3 | 10 | 99219895 | GC | G | 0.571 | NM_001289403 | MMS19 | frameshift_deleti  on | exon25 | c.2434delG | p.A812fs | . | . |
| CTC3 | 10 | 101657870 | T | TA | 0.362 | NM_015221 | DNMBP | stopgain | exon9 | c.2892dupT | p.N965_E966de  linsX | . | . |
| CTC3 | 10 | 102258462 | CT | C | 0.594 | NM_015490 | SEC31B | frameshift_deleti  on | exon14 | c.1683delA | p.K561fs | . | . |
| CTC3 | 10 | 102768332 | CT | C | 0.694 | NM_001195263 | PDZD7 | frameshift_deleti  on | exon17 | c.2993delA | p.Q998fs | . | . |
| CTC3 | 10 | 103789408 | TC | T | 0.847 | NM_024541 | C10orf76 | frameshift_deleti  on | exon5 | c.400delG | p.E134fs | . | . |
| CTC3 | 10 | 104129005 | CT | C | 0.75 | NM_001199378 | GBF1 | frameshift_deleti  on | exon24 | c.3012delT | p.P1004fs | . | . |
| CTC3 | 10 | 104160778 | G | GC | 0.7 | NM_001261403 | NFKB2 | frameshift_inserti  on | exon17 | c.2043_2044ins  C | p.P681fs | . | . |
| CTC3 | 10 | 104831564 | GC | G | 1 | NM_017649 | CNNM2 | frameshift_deleti  on | exon6 | c.2202delC | p.S734fs | . | . |
| CTC3 | 10 | 112266825 | AC | A | 0.591 | NM_004419 | DUSP5 | frameshift_deleti  on | exon3 | c.662delC | p.T221fs | . | . |
| CTC3 | 10 | 112358030 | GA | G | 0.613 | NM_005445 | SMC3 | frameshift_deleti  on | exon20 | c.2251delA | p.K751fs | . | . |
| CTC3 | 10 | 112559547 | C | CT | 0.667 | NM_001134363 | RBM20 | frameshift_inserti  on | exon7 | c.1672dupT | p.A557fs | . | . |
| CTC3 | 10 | 112559664 | AT | A | 0.648 | NM_001134363 | RBM20 | frameshift_deleti  on | exon7 | c.1789delT | p.L597fs | . | . |
| CTC3 | 10 | 112724579 | GA | G | 1 | NM_001269039 | SHOC2 | frameshift_deleti  on | exon1 | c.464delA | p.E155fs | . | . |
| CTC3 | 10 | 115388723 | TC | T | 0.493 | NM_006175 | NRAP | frameshift_deleti  on | exon19 | c.1992delG | p.G664fs | . | . |
| CTC3 | 10 | 115489264 | TC | T | 1 | NM_001267058 | CASP7 | frameshift_deleti  on | exon6 | c.803delC | p.S268fs | . | . |
| CTC3 | 10 | 115971640 | AC | A | 0.368 | NM_198795 | TDRD1 | frameshift_deleti  on | exon14 | c.1677delC | p.Y559fs | . | . |
| CTC3 | 10 | 118609092 | CG | C | 0.681 | NM_001242699 | ENO4 | frameshift_deleti  on | exon1 | c.16delG | p.G6fs | . | . |
| CTC3 | 10 | 121580370 | TA | T | 1 | NM_001243194 | INPP5F | frameshift_deleti  on | exon2 | c.70delA | p.T24fs | . | . |
| CTC3 | 10 | 121586435 | GA | G | 0.75 | NM_001243194 | INPP5F | frameshift_deleti  on | exon6 | c.713delA | p.D238fs | . | . |
| CTC3 | 10 | 123843364 | TG | T | 0.857 | NM_206862 | TACC2 | frameshift_deleti  on | exon4 | c.1350delG | p.M450fs | . | . |
| CTC3 | 10 | 126683137 | GT | G | 0.51 | NM_022802 | CTBP2 | frameshift_deleti  on | exon5 | c.2300delA | p.Y767fs | . | . |
| CTC3 | 10 | 128836023 | CCTG | C | 1 | NM_001380 | DOCK1 | nonframeshift_de  letion | exon19 | c.1891_1893del | p.631_631del | . | . |
| CTC3 | 10 | 129201407 | TG | T | 0.207 | NM_001380 | DOCK1 | frameshift_deleti  on | exon39 | c.3954delG | p.L1318fs | . | . |
| CTC3 | 10 | 129905111 | CTG | C | 0.259 | NM_001145966 | MKI67 | frameshift_deleti  on | exon12 | c.3911_3912del | p.T1304fs | . | . |
| CTC3 | 10 | 129905292 | TG | T | 0.379 | NM_001145966 | MKI67 | stopgain | exon12 | c.3731delC | p.S1244X | . | . |
| CTC3 | 10 | 129905724 | CT | C | 0.526 | NM_001145966 | MKI67 | frameshift_deleti on | exon12 | c.3299delA | p.K1100fs | . | . |
| CTC3 | 10 | 129905791 | GC | G | 0.447 | NM_001145966 | MKI67 | frameshift_deleti  on | exon12 | c.3232delG | p.A1078fs | . | . |
| CTC3 | 10 | 129905843 | CTG | C | 0.375 | NM_001145966 | MKI67 | frameshift_deleti  on | exon12 | c.3179_3180del | p.T1060fs | . | . |
| CTC3 | 10 | 134738323 | CG | C | 1 | NM_001200049 | TTC40 | frameshift_deleti  on | exon11 | c.1132delC | p.R378fs | . | . |
| CTC3 | 10 | 134999796 | GC | G | 0.231 | NM_152643 | KNDC1 | frameshift_deleti  on | exon6 | c.945delC | p.S315fs | . | . |
| CTC3 | 11 | 3249911 | AG | A | 0.624 | NM_001039165 | MRGPRE | frameshift_deleti  on | exon2 | c.118delC | p.L40fs | . | . |
| CTC3 | 11 | 3733781 | CT | C | 0.909 | NM_005387 | NUP98 | frameshift_deleti  on | exon20 | c.2805delA | p.K935fs | . | . |
| CTC3 | 11 | 4936766 | GA | G | 0.3 | NM_001005238 | OR51G2 | frameshift_deleti  on | exon1 | c.127delT | p.S43fs | . | . |
| CTC3 | 11 | 5461984 | TA | T | 0.538 | NM_001005288 | OR51I1 | frameshift_deleti  on | exon1 | c.760delT | p.Y254fs | . | . |
| CTC3 | 11 | 5602410 | CT | C | 0.279 | NM_001005162 | OR52B6 | frameshift_deleti  on | exon1 | c.305delT | p.L102fs | . | . |
| CTC3 | 11 | 5989624 | AG | A | 0.389 | NM_001146033 | OR56A5 | frameshift_deleti  on | exon1 | c.100delC | p.L34fs | . | . |
| CTC3 | 11 | 6424414 | CT | C | 0.444 | NM_001257319 | APBB1 | frameshift_deleti  on | exon5 | c.404delA | p.E135fs | . | . |
| CTC3 | 11 | 6567895 | C | CTGCC  CTACT | 1 | NM_144666 | DNHD1 | nonframeshift_in  sertion | exon21 | c.5726_5727ins  TGCCCTACTGC | p.A1909delinsA  ALLH | . | . |
| CTC3 | 11 | 6592533 | TGG | T | 0.875 | NM_144666 | DNHD1 | frameshift_deleti  on | exon42 | c.13792_13793  del | p.G4598fs | . | . |
| CTC3 | 11 | 6662253 | CG | C | 0.294 | NM_003737 | DCHS1 | frameshift_deleti  on | exon2 | c.591delC | p.P197fs | . | . |
| CTC3 | 11 | 6662370 | CA | C | 0.727 | NM_003737 | DCHS1 | frameshift_deleti  on | exon2 | c.474delT | p.F158fs | . | . |
| CTC3 | 11 | 6942679 | TA | T | 1 | NM_001004684 | OR2D3 | frameshift_deleti  on | exon1 | c.448delA | p.T150fs | . | . |
| CTC3 | 11 | 6977412 | AC | A | 0.629 | NM_013250 | ZNF215 | frameshift_deleti  on | exon7 | c.1205delC | p.T402fs | . | . |
| CTC3 | 11 | 8720776 | TC | T | 1 | NM_139157 | ST5 | frameshift_deleti  on | exon14 | c.1621delG | p.E541fs | . | . |
| CTC3 | 11 | 10602097 | GT | G | 0.235 | NM_001206881 | MRVI1 | frameshift_deleti  on | exon18 | c.1454delA | p.D485fs | . | . |
| CTC3 | 11 | 10821725 | AC | A | 1 | NM_001042559 | EIF4G2 | frameshift_deleti  on | exon17 | c.1916delG | p.C639fs | . | . |
| CTC3 | 11 | 11314614 | TG | T | 0.579 | NM_198516 | GALNT18 | frameshift_deleti  on | exon10 | c.1638delC | p.A546fs | . | . |

| CTC3 | 11 | 12241933 | G | GA | 0.273 | NM_001282666 | MICAL2 | frameshift_inserti  on | exon7 | c.1135dupA | p.E378fs | . | . |
| --- | --- | --- | --- | --- | --- | --- | --- | --- | --- | --- | --- | --- | --- |
| CTC3 | 11 | 14902150 | CA | C | 0.566 | NM_024514 | CYP2R1 | frameshift_deleti  on | exon3 | c.531delT | p.F177fs | . | . |
| CTC3 | 11 | 17653391 | AG | A | 0.429 | NM_001277269 | OTOG | frameshift_deleti  on | exon40 | c.6892delG | p.D2298fs | . | . |
| CTC3 | 11 | 18010190 | CT | C | 0.807 | NM_012139 | SERGEF | frameshift_deleti  on | exon8 | c.797delA | p.K266fs | . | . |
| CTC3 | 11 | 18010281 | C | CG | 0.435 | NM_012139 | SERGEF | frameshift_inserti  on | exon8 | c.706_707insC | p.W236fs | . | . |
| CTC3 | 11 | 18127530 | A | ACCTC  CT | 1 | NM_138421 | SAAL1 | nonframeshift_in  sertion | exon1 | c.58_59insAGG  AGG | p.V20delinsEEV | . | . |
| CTC3 | 11 | 18127530 | A | ACCT | 1 | NM_138421 | SAAL1 | nonframeshift_in  sertion | exon1 | c.58_59insAGG | p.V20delinsEV | . | . |
| CTC3 | 11 | 18127548 | T | TCCTCC  A | 1 | NM_138421 | SAAL1 | nonframeshift_in  sertion | exon1 | c.40_41insTGG  AGG | p.K14delinsME  E | . | . |
| CTC3 | 11 | 18195762 | TG | T | 0.267 | NM_054032 | MRGPRX4 | frameshift_deleti  on | exon1 | c.960delG | p.L320fs | . | . |
| CTC3 | 11 | 19372566 | CA | C | 0.4 | NM_001111018 | NAV2 | frameshift_deleti  on | exon1 | c.68delA | p.Q23fs | . | . |
| CTC3 | 11 | 19955466 | C | CT | 0.467 | NM_001111018 | NAV2 | frameshift_inserti  on | exon7 | c.1485dupT | p.P495fs | . | . |
| CTC3 | 11 | 27679720 | TC | T | 0.286 | NM_170735 | BDNF | frameshift_deleti  on | exon1 | c.391delG | p.D131fs | . | . |
| CTC3 | 11 | 30033286 | CGG | C | 1 | NM_002233 | KCNA4 | frameshift_deleti  on | exon2 | c.938_939del | p.S313fs | . | . |
| CTC3 | 11 | 30944055 | AT | A | 0.314 | NM_020869 | DCDC5 | stopgain | exon5 | c.448delA | p.I150X | . | . |
| CTC3 | 11 | 33054507 | G | GA | 1 | NM_001077242 | DEPDC7 | frameshift_inserti on | exon8 | c.1337dupA | p.D446fs | . | . |
| CTC3 | 11 | 34130372 | GT | G | 0.25 | NM_024662 | NAT10 | frameshift_deleti  on | exon3 | c.193delT | p.F65fs | . | . |
| CTC3 | 11 | 35453912 | TA | T | 0.211 | NM_001282676 | PAMR1 | frameshift_deleti  on | exon9 | c.1821delT | p.N607fs | . | . |
| CTC3 | 11 | 45672297 | GC | G | 0.364 | NM_003654 | CHST1 | frameshift_deleti  on | exon4 | c.176delG | p.R59fs | . | . |
| CTC3 | 11 | 45927270 | T | TA | 1 | NM_005456 | MAPK8IP1 | frameshift_inserti  on | exon12 | c.2135dupA | p.X712delinsX | . | . |
| CTC3 | 11 | 45928464 | GA | G | 0.348 | NM_001080446 | C11orf94 | frameshift_deleti  on | exon2 | c.130delT | p.S44fs | . | . |
| CTC3 | 11 | 46724651 | GC | G | 1 | NM_001184751 | ZNF408 | frameshift_deleti  on | exon4 | c.487delC | p.P163fs | . | . |
| CTC3 | 11 | 46724716 | CAGTGG  TGACAG | C | 1 | NM_001184751 | ZNF408 | nonframeshift_de  letion | exon4 | c.552_563del | p.184_188del | . | . |
| CTC3 | 11 | 47744588 | TGGA | T | 0.2 | NM_015308 | FNBP4 | nonframeshift_de  letion | exon15 | c.2742_2744del | p.914_915del | . | . |
| CTC3 | 11 | 55419216 | GT | G | 0.25 | NM_001004059 | OR4S2 | stopgain | exon1 | c.838delT | p.L280X | . | . |
| CTC3 | 11 | 57472535 | GC | G | 1 | NM_153450 | MED19 | frameshift_deleti on | exon2 | c.383delG | p.R128fs | . | . |
| CTC3 | 11 | 57573416 | A | AG | 0.333 | NM_001085469 | CTNND1 | frameshift_inserti  on | exon7 | c.1482_1483ins  G | p.P494fs | . | . |
| CTC3 | 11 | 61109297 | AC | A | 0.204 | NM_015533 | DAK | frameshift_deleti  on | exon7 | c.569delC | p.T190fs | . | . |
| CTC3 | 11 | 61113829 | G | GA | 0.302 | NM_015533 | DAK | frameshift_inserti  on | exon18 | c.1583dupA | p.E528fs | . | . |
| CTC3 | 11 | 61183647 | GT | G | 0.409 | NM_001136040 | CPSF7 | frameshift_deleti  on | exon6 | c.894delA | p.P298fs | . | . |
| CTC3 | 11 | 62290651 | CG | C | 1 | NM_001620 | AHNAK | stopgain | exon5 | c.11237delC | p.S3746X | . | . |
| CTC3 | 11 | 62296572 | TG | T | 0.69 | NM_001620 | AHNAK | frameshift_deleti on | exon5 | c.5316delC | p.P1772fs | . | . |
| CTC3 | 11 | 62297153 | AT | A | 0.639 | NM_001620 | AHNAK | frameshift_deleti  on | exon5 | c.4735delA | p.I1579fs | . | . |
| CTC3 | 11 | 62455743 | CG | C | 0.792 | NM_203422 | LRRN4CL | frameshift_deleti  on | exon2 | c.237delC | p.P79fs | . | . |
| CTC3 | 11 | 62520827 | TG | T | 0.5 | NM_024784 | ZBTB3 | frameshift_deleti  on | exon2 | c.459delC | p.A153fs | . | . |
| CTC3 | 11 | 62564687 | GT | G | 1 | NM_006362 | NXF1 | frameshift_deleti  on | exon13 | c.1145delA | p.N382fs | . | . |
| CTC3 | 11 | 63987434 | AC | A | 0.912 | NM_031471 | FERMT3 | stopgain | exon10 | c.1152delC | p.Y384X | . | . |
| CTC3 | 11 | 63994162 | G | GT | 0.211 | NM_001128612 | NUDT22 | frameshift_inserti on | exon2 | c.38_39insT | p.G13fs | . | . |
| CTC3 | 11 | 64067125 | GC | G | 1 | NM_033310 | KCNK4 | frameshift_deleti  on | exon7 | c.1110delC | p.R370fs | . | . |
| CTC3 | 11 | 64622824 | AG | A | 0.6 | NM_006795 | EHD1 | frameshift_deleti  on | exon4 | c.1049delC | p.P350fs | . | . |
| CTC3 | 11 | 64855576 | GC | G | 0.308 | NM_006782 | ZFPL1 | frameshift_deleti  on | exon8 | c.924delC | p.G308fs | . | . |
| CTC3 | 11 | 64884651 | CA | C | 0.615 | NM_014205 | ZNHIT2 | frameshift_deleti  on | exon1 | c.474delT | p.P158fs | . | . |
| CTC3 | 11 | 65351161 | TG | T | 0.875 | NM_001099409 | EHBP1L1 | frameshift_deleti  on | exon9 | c.3019delG | p.G1007fs | . | . |
| CTC3 | 11 | 65351192 | TC | T | 1 | NM_001099409 | EHBP1L1 | frameshift_deleti  on | exon9 | c.3050delC | p.S1017fs | . | . |
| CTC3 | 11 | 65632255 | TC | T | 0.469 | NM_025128 | MUS81 | frameshift_deleti  on | exon12 | c.1242delC | p.L414fs | . | . |
| CTC3 | 11 | 65635391 | CG | C | 0.667 | NM_016938 | EFEMP2 | frameshift_deleti  on | exon10 | c.1110delC | p.P370fs | . | . |
| CTC3 | 11 | 65635420 | AAC | A | 0.75 | NM_016938 | EFEMP2 | frameshift_deleti  on | exon10 | c.1080_1081del | p.V360fs | . | . |
| CTC3 | 11 | 65995016 | TA | T | 0.6 | NM_018026 | PACS1 | frameshift_deleti  on | exon11 | c.1336delA | p.K446fs | . | . |
| CTC3 | 11 | 66033195 | GCA | G | 0.182 | NM_001134774 | KLC2 | frameshift_deleti  on | exon11 | c.1174_1175del | p.H392fs | . | . |
| CTC3 | 11 | 66243215 | GC | G | 0.3 | NM_001243136 | PELI3 | frameshift_deleti  on | exon6 | c.667delC | p.P223fs | . | . |
| CTC3 | 11 | 66411523 | GC | G | 0.57 | NM_001198845 | RBM14-  RBM4 | frameshift_deleti  on | exon2 | c.941delC | p.A314fs | . | . |
| CTC3 | 11 | 66472649 | TG | T | 1 | NM_006946 | SPTBN2 | frameshift_deleti  on | exon14 | c.2097delC | p.L699fs | . | . |
| CTC3 | 11 | 67200255 | A | AT | 0.931 | NM_003952 | RPS6KB2 | frameshift_inserti  on | exon7 | c.563_564insT | p.Q188fs | . | . |
| CTC3 | 11 | 68772928 | TG | T | 0.364 | NM_001098515 | MRGPRF | frameshift_deleti  on | exon3 | c.849delC | p.N283fs | . | . |
| CTC3 | 11 | 71189473 | CG | C | 0.667 | NM_018161 | NADSYN1 | frameshift_deleti  on | exon10 | c.832delG | p.V278fs | . | . |
| CTC3 | 11 | 71209418 | GT | G | 0.477 | NM_018161 | NADSYN1 | frameshift_deleti  on | exon20 | c.1915delT | p.F639fs | . | . |
| CTC3 | 11 | 71822213 | TG | T | 0.929 | NM_001278490 | ANAPC15 | frameshift_deleti  on | exon2 | c.109delC | p.H37fs | . | . |
| CTC3 | 11 | 73122567 | TC | T | 0.425 | NM_015159 | FAM168A | frameshift_deleti  on | exon5 | c.291delG | p.G97fs | . | . |

| CTC3 | 11 | 73689391 | AG | A | 0.319 | NM_003355 | UCP2 | frameshift_deleti  on | exon3 | c.32delC | p.P11fs | . | . |
| --- | --- | --- | --- | --- | --- | --- | --- | --- | --- | --- | --- | --- | --- |
| CTC3 | 11 | 73978213 | TC | T | 0.23 | NM_001288748 | P4HA3 | frameshift_deleti  on | exon13 | c.1707delG | p.E569fs | . | . |
| CTC3 | 11 | 75188718 | GC | G | 0.857 | NM_030792 | GDPD5 | frameshift_deleti  on | exon3 | c.62delG | p.G21fs | . | . |
| CTC3 | 11 | 77774950 | AC | A | 0.303 | NM_003251 | THRSP | frameshift_deleti  on | exon1 | c.24delC | p.Y8fs | . | . |
| CTC3 | 11 | 77820512 | GA | G | 0.204 | NM_001007027 | ALG8 | frameshift_deleti  on | exon9 | c.1013delT | p.L338fs | . | . |
| CTC3 | 11 | 77911204 | TC | T | 0.207 | NM_020798 | USP35 | frameshift_deleti  on | exon5 | c.963delC | p.I321fs | . | . |
| CTC3 | 11 | 78380982 | TG | T | 0.643 | NM_001098816 | TENM4 | frameshift_deleti  on | exon32 | c.6407delC | p.T2136fs | . | . |
| CTC3 | 11 | 78567054 | CA | C | 0.175 | NM_001098816 | TENM4 | frameshift_deleti  on | exon11 | c.1424delT | p.L475fs | . | . |
| CTC3 | 11 | 85374859 | AT | A | 1 | NM_001039618 | CREBZF | frameshift_deleti  on | exon1 | c.1060delA | p.M354fs | . | . |
| CTC3 | 11 | 92573806 | TC | T | 0.591 | NM_001008781 | FAT3 | frameshift_deleti  on | exon17 | c.10448delC | p.S3483fs | . | . |
| CTC3 | 11 | 92715219 | AC | A | 0.419 | NM_005959 | MTNR1B | frameshift_deleti  on | exon2 | c.831delC | p.N277fs | . | . |
| CTC3 | 11 | 93458982 | GA | G | 0.979 | NM_033395 | KIAA1731 | frameshift_deleti  on | exon23 | c.6485delA | p.E2162fs | . | . |
| CTC3 | 11 | 95562452 | CA | C | 0.3 | NM_001243777 | CEP57 | frameshift_deleti  on | exon9 | c.1152delA | p.A384fs | . | . |
| CTC3 | 11 | 95826635 | GC | G | 0.556 | NM_032427 | MAML2 | frameshift_deleti  on | exon2 | c.559delG | p.A187fs | . | . |
| CTC3 | 11 | 103041679 | GT | G | 0.632 | NM_001080463 | DYNC2H1 | frameshift_deleti  on | exon34 | c.5217delT | p.C1739fs | . | . |
| CTC3 | 11 | 104879572 | AG | A | 0.22 | NM_001136112 | CASP5 | stopgain | exon2 | c.181delC | p.L61X | . | . |
| CTC3 | 11 | 105923730 | TG | T | 0.98 | NM_152433 | KBTBD3 | frameshift_deleti on | exon3 | c.1685delC | p.P562fs | . | . |
| CTC3 | 11 | 108382219 | GC | G | 1 | NM_015065 | EXPH5 | frameshift_deleti  on | exon6 | c.4014delG | p.G1338fs | . | . |
| CTC3 | 11 | 108382250 | AT | A | 0.3 | NM_015065 | EXPH5 | frameshift_deleti  on | exon6 | c.3983delA | p.N1328fs | . | . |
| CTC3 | 11 | 108385002 | T | TC | 0.269 | NM_015065 | EXPH5 | frameshift_inserti  on | exon6 | c.1231dupG | p.E411fs | . | . |
| CTC3 | 11 | 111953338 | AG | A | 0.714 | NM_001082969 | C11orf57 | frameshift_deleti  on | exon6 | c.525delG | p.K175fs | . | . |
| CTC3 | 11 | 116627899 | TA | T | 0.5 | NM_001159736 | BUD13 | frameshift_deleti  on | exon9 | c.1326delT | p.F442fs | . | . |
| CTC3 | 11 | 116661432 | AG | A | 0.462 | NM_001166598 | APOA5 | frameshift_deleti  on | exon4 | c.512delC | p.A171fs | . | . |
| CTC3 | 11 | 116717146 | T | TC | 0.625 | NM_001281749 | SIK3 | frameshift_inserti  on | exon23 | c.3751dupG | p.E1251fs | . | . |
| CTC3 | 11 | 117052622 | CA | C | 0.712 | NM_001040455 | SIDT2 | frameshift_deleti  on | exon3 | c.406delA | p.T136fs | . | . |
| CTC3 | 11 | 117109405 | CA | C | 0.8 | NM_001077239 | RNF214 | frameshift_deleti  on | exon3 | c.197delA | p.H66fs | . | . |
| CTC3 | 11 | 119149355 | TATG | T | 0.208 | NM_005188 | CBL | nonframeshift_de  letion | exon9 | c.1364_1366del | p.455_456del | . | . |
| CTC3 | 11 | 119535677 | CCCT | C | 0.162 | NM_002855 | PVRL1 | nonframeshift_de  letion | exon6 | c.1331_1333del | p.444_445del | . | . |
| CTC3 | 11 | 120924332 | GT | G | 1 | NM_001130047 | TBCEL | frameshift_deleti  on | exon4 | c.347delT | p.V116fs | . | . |
| CTC3 | 11 | 122775087 | A | AC | 0.407 | NM_024806 | C11orf63 | frameshift_inserti  on | exon3 | c.800dupC | p.T267fs | . | . |
| CTC3 | 11 | 122955404 | AC | A | 0.545 | NM_024769 | CLMP | frameshift_deleti  on | exon3 | c.203delG | p.S68fs | . | . |
| CTC3 | 11 | 123516294 | CA | C | 1 | NM_018400 | SCN3B | frameshift_deleti  on | exon2 | c.219delT | p.L73fs | . | . |
| CTC3 | 11 | 124626457 | GT | G | 0.762 | NM_138961 | ESAM | frameshift_deleti  on | exon3 | c.430delA | p.T144fs | . | . |
| CTC3 | 11 | 124740940 | AC | A | 0.864 | NM_022370 | ROBO3 | frameshift_deleti  on | exon7 | c.1065delC | p.D355fs | . | . |
| CTC3 | 11 | 124933276 | CT | C | 0.359 | NM_001145290 | SLC37A2 | frameshift_deleti  on | exon1 | c.14delT | p.L5fs | . | . |
| CTC3 | 11 | 124971139 | GA | G | 0.282 | NM_001080546 | TMEM218 | frameshift_deleti  on | exon4 | c.170delT | p.F57fs | . | . |
| CTC3 | 11 | 125864872 | TA | T | 0.167 | NM_001243597 | CDON | frameshift_deleti  on | exon13 | c.2437delT | p.Y813fs | . | . |
| CTC3 | 11 | 125880410 | A | AT | 0.941 | NM_001243597 | CDON | frameshift_inserti  on | exon8 | c.1377dupA | p.S460fs | . | . |
| CTC3 | 11 | 126080895 | TG | T | 0.87 | NM_001144827 | RPUSD4 | frameshift_deleti  on | exon2 | c.244delC | p.Q82fs | . | . |
| CTC3 | 11 | 130289099 | AC | A | 0.216 | NM_007037 | ADAMTS8 | frameshift_deleti  on | exon2 | c.808delG | p.V270fs | . | . |
| CTC3 | 11 | 134048585 | T | TG | 0.935 | NM_015261 | NCAPD3 | frameshift_inserti  on | exon22 | c.2725dupC | p.Q909fs | . | . |
| CTC3 | 11 | 134240954 | G | GA | 0.75 | NM_138342 | GLB1L2 | frameshift_inserti  on | exon13 | c.1269dupA | p.G423fs | . | . |
| CTC3 | 12 | 2062323 | T | TTGC | 0.37 | NM_152640 | DCP1B | nonframeshift_in  sertion | exon7 | c.782_783insG  CA | p.Q261delinsQ  Q | . | . |
| CTC3 | 12 | 2927347 | TC | T | 0.909 | NM_018463 | ITFG2 | frameshift_deleti  on | exon4 | c.311delC | p.S104fs | . | . |
| CTC3 | 12 | 6031862 | C | CA | 0.314 | NM_001278596 | ANO2 | frameshift_inserti  on | exon2 | c.130_131insT | p.R44fs | . | . |
| CTC3 | 12 | 6647293 | AC | A | 0.286 | NM_001256799 | GAPDH | frameshift_deleti  on | exon8 | c.840delC | p.N280fs | . | . |
| CTC3 | 12 | 6959681 | CG | C | 0.647 | NM_031299 | CDCA3 | frameshift_deleti  on | exon3 | c.199delC | p.R67fs | . | . |
| CTC3 | 12 | 7043631 | GC | G | 0.429 | NM_001007026 | ATN1 | frameshift_deleti  on | exon4 | c.170delC | p.A57fs | . | . |
| CTC3 | 12 | 7045891 | ACAG | A | 0.318 | NM_001007026 | ATN1 | nonframeshift_de  letion | exon5 | c.1462_1464del | p.488_488del | . | . |
| CTC3 | 12 | 7048092 | TC | T | 1 | NM_001007026 | ATN1 | frameshift_deleti  on | exon7 | c.2967delC | p.F989fs | . | . |
| CTC3 | 12 | 8075424 | G | GT | 0.667 | NM_006931 | SLC2A3 | frameshift_inserti  on | exon9 | c.1264_1265ins  A | p.S422fs | . | . |
| CTC3 | 12 | 8374781 | C | CACG | 1 | NM_018088 | FAM90A1 | nonframeshift_in  sertion | exon7 | c.1031_1032ins  CGT | p.T344delinsTV | . | . |
| CTC3 | 12 | 9229487 | TC | T | 0.242 | NM_000014 | A2M | stopgain | exon28 | c.3396delG | p.W1132X | . | . |
| CTC3 | 12 | 9994445 | GTGT | G | 1 | NM_016523 | KLRF1 | nonframeshift_de letion | exon4 | c.373_375del | p.125_125del | . | . |
| CTC3 | 12 | 10224111 | AC | A | 0.333 | NM_016511 | CLEC1A | frameshift_deleti  on | exon6 | c.663delG | p.L221fs | . | . |
| CTC3 | 12 | 10978182 | CA | C | 0.531 | NM_023921 | TAS2R10 | stopgain | exon1 | c.686delT | p.L229X | . | . |
| CTC3 | 12 | 14927761 | G | GA | 0.5 | NM_177925 | H2AFJ | frameshift_inserti on | exon1 | c.358dupA | p.K119fs | . | . |

| CTC3 | 12 | 16397543 | GA | G | 1 | NM_001170798 | SLC15A5 | frameshift_deleti  on | exon4 | c.945delT | p.F315fs | . | . |
| --- | --- | --- | --- | --- | --- | --- | --- | --- | --- | --- | --- | --- | --- |
| CTC3 | 12 | 18891469 | AC | A | 0.25 | NM_033328 | CAPZA3 | frameshift_deleti  on | exon1 | c.268delC | p.L90fs | . | . |
| CTC3 | 12 | 21796930 | G | GA | 0.25 | NM_001174097 | LDHB | frameshift_inserti  on | exon4 | c.359dupT | p.F120fs | . | . |
| CTC3 | 12 | 27127067 | TG | T | 0.312 | NM_016551 | TM7SF3 | frameshift_deleti  on | exon12 | c.1543delC | p.H515fs | . | . |
| CTC3 | 12 | 27933908 | GC | G | 0.5 | NM_020782 | KLHL42 | frameshift_deleti  on | exon1 | c.646delC | p.P216fs | . | . |
| CTC3 | 12 | 29496161 | TC | T | 0.789 | NM_016570 | ERGIC2 | frameshift_deleti  on | exon12 | c.879delG | p.M293fs | . | . |
| CTC3 | 12 | 29604295 | CTG | C | 0.164 | NM_183378 | OVCH1 | frameshift_deleti  on | exon22 | c.2736_2737del | p.H912fs | . | . |
| CTC3 | 12 | 30869564 | A | AT | 0.19 | NM_001206856 | CAPRIN2 | frameshift_inserti  on | exon13 | c.2236dupA | p.I746fs | . | . |
| CTC3 | 12 | 31862322 | GA | G | 0.917 | NM_001113402 | AMN1 | frameshift_deleti  on | exon2 | c.75delT | p.Y25fs | . | . |
| CTC3 | 12 | 32490745 | GA | G | 0.298 | NM_001714 | BICD1 | frameshift_deleti  on | exon7 | c.2566delA | p.K856fs | . | . |
| CTC3 | 12 | 38714314 | TC | T | 1 | NM_001013620 | ALG10B | frameshift_deleti  on | exon3 | c.722delC | p.S241fs | . | . |
| CTC3 | 12 | 39745775 | AT | A | 0.825 | NM_001173463 | KIF21A | frameshift_deleti  on | exon11 | c.1476delA | p.K492fs | . | . |
| CTC3 | 12 | 45105084 | CT | C | 1 | NM_001145108 | NELL2 | frameshift_deleti  on | exon11 | c.1179delA | p.K393fs | . | . |
| CTC3 | 12 | 45782035 | AC | A | 0.364 | NM_001025356 | ANO6 | frameshift_deleti  on | exon11 | c.1258delC | p.P420fs | . | . |
| CTC3 | 12 | 49426905 | TTGC | T | 0.185 | NM_003482 | KMT2D | nonframeshift_de  letion | exon39 | c.11580_11582  del | p.3860_3861de  l | . | . |
| CTC3 | 12 | 49434894 | GC | G | 0.444 | NM_003482 | KMT2D | frameshift_deleti  on | exon31 | c.6658delG | p.A2220fs | . | . |
| CTC3 | 12 | 51084857 | TA | T | 0.333 | NM_173602 | DIP2B | frameshift_deleti  on | exon13 | c.1578delA | p.V526fs | . | . |
| CTC3 | 12 | 51447613 | C | CT | 1 | NM_001243689 | LETMD1 | frameshift_inserti  on | exon4 | c.483dupT | p.P161fs | . | . |
| CTC3 | 12 | 52379062 | C | CT | 0.164 | NM_004302 | ACVR1B | frameshift_inserti  on | exon6 | c.1067dupT | p.L356fs | . | . |
| CTC3 | 12 | 52636850 | CG | C | 0.757 | NM_005556 | KRT7 | frameshift_deleti  on | exon6 | c.914delG | p.R305fs | . | . |
| CTC3 | 12 | 53045615 | A | ACCTC  CAAAG CCGCT | 0.975 | NM_000423 | KRT2 | nonframeshift_in sertion | exon1 | c.311_312insC  GGCAGCGGCT TTGGAGG | p.G104delinsG GSGFGG | . | . |
| CTC3 | 12 | 53189413 | ACCAAA  GCCACC AGCCCC | A | 1 | NM_057088 | KRT3 | nonframeshift_de letion | exon1 | c.396_413del | p.132_138del | . | . |
| CTC3 | 12 | 53207583 | C | CCACC  AAAGC CACCA GTGCC GAAAC CAGCT | 1 | NM_002272 | KRT4 | nonframeshift_in sertion | exon1 | c.259_260insCC GGCGGCTTCG GAGCTGGTTTC GGCACTGGTG GCTTTGGTG | p.G87delinsAG GFGAGFGTGGF GG | . | . |
| CTC3 | 12 | 53670884 | TC | T | 0.455 | NM_012291 | ESPL1 | frameshift_deleti  on | exon9 | c.1959delC | p.I653fs | . | . |
| CTC3 | 12 | 53679944 | CA | C | 0.375 | NM_012291 | ESPL1 | frameshift_deleti  on | exon18 | c.3425delA | p.H1142fs | . | . |
| CTC3 | 12 | 53818979 | T | TG | 1 | NM_001164690 | AMHR2 | frameshift_inserti  on | exon4 | c.456dupG | p.L152fs | . | . |
| CTC3 | 12 | 53900858 | GA | G | 0.5 | NM_003717 | NPFF | frameshift_deleti  on | exon2 | c.170delT | p.L57fs | . | . |
| CTC3 | 12 | 53918417 | CT | C | 0.358 | NM_001130060 | ATF7 | frameshift_deleti  on | exon10 | c.992delA | p.K331fs | . | . |
| CTC3 | 12 | 54405048 | GA | G | 0.917 | NM_022658 | HOXC8 | frameshift_deleti  on | exon2 | c.613delA | p.K205fs | . | . |
| CTC3 | 12 | 55615276 | TA | T | 0.235 | NM_001005280 | OR10A7 | frameshift_deleti  on | exon1 | c.469delA | p.M157fs | . | . |
| CTC3 | 12 | 56115242 | GC | G | 0.283 | NM_001199771 | RDH5 | frameshift_deleti  on | exon2 | c.275delC | p.A92fs | . | . |
| CTC3 | 12 | 56221117 | TA | T | 0.8 | NM_032364 | DNAJC14 | frameshift_deleti  on | exon2 | c.1325delT | p.L442fs | . | . |
| CTC3 | 12 | 56351300 | CA | C | 1 | NM_001200053 | PMEL | frameshift_deleti  on | exon4 | c.528delT | p.F176fs | . | . |
| CTC3 | 12 | 57009123 | AC | A | 0.941 | NM_013449 | BAZ2A | frameshift_deleti  on | exon3 | c.410delG | p.S137fs | . | . |
| CTC3 | 12 | 57011273 | A | AG | 0.615 | NM_013449 | BAZ2A | frameshift_inserti  on | exon2 | c.47dupC | p.P16fs | . | . |
| CTC3 | 12 | 57593716 | TC | T | 0.356 | NM_002332 | LRP1 | frameshift_deleti  on | exon62 | c.9923delC | p.S3308fs | . | . |
| CTC3 | 12 | 57970607 | CG | C | 0.75 | NM_004984 | KIF5A | frameshift_deleti  on | exon20 | c.2263delG | p.E755fs | . | . |
| CTC3 | 12 | 58000801 | GC | G | 0.8 | NM_001286245 | DTX3 | frameshift_deleti  on | exon4 | c.165delC | p.G55fs | . | . |
| CTC3 | 12 | 58347400 | TA | T | 0.75 | NM_033276 | XRCC6BP1 | frameshift_deleti  on | exon5 | c.466delA | p.N156fs | . | . |
| CTC3 | 12 | 62954807 | TC | T | 1 | NM_001278469 | MON2 | frameshift_deleti  on | exon26 | c.3947delC | p.S1316fs | . | . |
| CTC3 | 12 | 69207352 | CT | C | 0.333 | NM_001145337 | MDM2 | frameshift_deleti  on | exon3 | c.101delT | p.L34fs | . | . |
| CTC3 | 12 | 70732298 | C | CA | 0.362 | NM_001199303 | CNOT2 | frameshift_inserti  on | exon10 | c.977dupA | p.Q326fs | . | . |
| CTC3 | 12 | 70964929 | GA | G | 0.4 | NM_001206971 | PTPRB | frameshift_deleti  on | exon10 | c.2322delT | p.F774fs | . | . |
| CTC3 | 12 | 76444309 | ACAT | A | 0.143 | NM_004537 | NAP1L1 | frameshift_deleti  on | exon12 | c.1058_1059del | p.D353fs | . | . |
| CTC3 | 12 | 76784339 | TC | T | 0.254 | NM_001003712 | OSBPL8 | frameshift_deleti  on | exon10 | c.901delG | p.D301fs | . | . |
| CTC3 | 12 | 78400260 | TG | T | 0.337 | NM_001024383 | NAV3 | frameshift_deleti  on | exon8 | c.943delG | p.A315fs | . | . |
| CTC3 | 12 | 85421761 | CA | C | 0.492 | NM_001100917 | TSPAN19 | stopgain | exon4 | c.179delT | p.L60X | . | . |
| CTC3 | 12 | 88477692 | GA | G | 0.333 | NM_025114 | CEP290 | frameshift_deleti on | exon36 | c.4743delT | p.L1581fs | . | . |
| CTC3 | 12 | 95868075 | AAAG | A | 0.333 | NM_006838 | METAP2 | nonframeshift_de  letion | exon1 | c.121_123del | p.41_41del | . | . |
| CTC3 | 12 | 95927551 | AT | A | 0.604 | NM_001042403 | USP44 | frameshift_deleti  on | exon2 | c.481delA | p.I161fs | . | . |
| CTC3 | 12 | 100452170 | G | GA | 0.857 | NM_015054 | UHRF1BP1L | frameshift_inserti  on | exon14 | c.2884dupT | p.S962fs | . | . |
| CTC3 | 12 | 102127373 | CCT | C | 0.129 | NM_001177948 | SYCP3 | frameshift_deleti  on | exon6 | c.431_432del | p.E144fs | . | . |
| CTC3 | 12 | 105509488 | AT | A | 0.583 | NM_015275 | KIAA1033 | frameshift_deleti  on | exon6 | c.418delT | p.F140fs | . | . |
| CTC3 | 12 | 106734692 | AC | A | 0.209 | NM_152772 | TCP11L2 | frameshift_deleti  on | exon9 | c.1232delC | p.T411fs | . | . |

| CTC3 | 12 | 107236449 | CA | C | 0.429 | NM_018157 | RIC8B | frameshift_deleti  on | exon5 | c.920delA | p.Q307fs | . | . |
| --- | --- | --- | --- | --- | --- | --- | --- | --- | --- | --- | --- | --- | --- |
| CTC3 | 12 | 109052643 | TA | T | 1 | NM_001105237 | CORO1C | frameshift_deleti  on | exon5 | c.659delT | p.I220fs | . | . |
| CTC3 | 12 | 109331097 | AT | A | 0.265 | NM_018711 | SVOP | stopgain | exon9 | c.754delA | p.M252X | . | . |
| CTC3 | 12 | 109495815 | TC | T | 0.893 | NM_032663 | USP30 | frameshift_deleti on | exon3 | c.279delC | p.I93fs | . | . |
| CTC3 | 12 | 109725743 | AC | A | 1 | NM_213596 | FOXN4 | frameshift_deleti  on | exon5 | c.373delG | p.V125fs | . | . |
| CTC3 | 12 | 109937586 | TG | T | 0.434 | NM_130466 | UBE3B | frameshift_deleti  on | exon12 | c.1090delG | p.G364fs | . | . |
| CTC3 | 12 | 111779662 | GC | G | 0.532 | NM_015267 | CUX2 | frameshift_deleti  on | exon21 | c.3465delC | p.S1155fs | . | . |
| CTC3 | 12 | 116446361 | AC | A | 1 | NM_015335 | MED13L | frameshift_deleti  on | exon10 | c.1856delG | p.C619fs | . | . |
| CTC3 | 12 | 118588854 | A | AT | 0.313 | NM_016281 | TAOK3 | frameshift_inserti  on | exon21 | c.2644dupA | p.M882fs | . | . |
| CTC3 | 12 | 120260769 | CA | C | 0.773 | NM_001206999 | CIT | stopgain | exon9 | c.965delT | p.L322X | . | . |
| CTC3 | 12 | 121134165 | GGAA | G | 0.294 | NM_014730 | MLEC | nonframeshift_de letion | exon5 | c.697_699del | p.233_233del | . | . |
| CTC3 | 12 | 121205248 | G | GC | 0.418 | NM_139015 | SPPL3 | frameshift_inserti  on | exon9 | c.921dupG | p.R308fs | . | . |
| CTC3 | 12 | 121465533 | CA | C | 0.917 | NM_003733 | OASL | frameshift_deleti  on | exon4 | c.744delT | p.T248fs | . | . |
| CTC3 | 12 | 121471544 | C | CA | 0.895 | NM_001261825 | OASL | frameshift_inserti  on | exon2 | c.200dupT | p.V67fs | . | . |
| CTC3 | 12 | 122359397 | G | GGAGG  AGGAG | 1 | NM_001178003 | WDR66 | nonframeshift_in  sertion | exon2 | c.186_187insG  AGGAGGAGGA | p.G62delinsGE  EEEK | . | . |
| CTC3 | 12 | 122359398 | G | GAGGA  GGAGG | 1 | NM_001178003 | WDR66 | stopgain | exon2 | c.187_188insA  GGAGGAGGAG | p.E63delinsEEE  EKX | . | . |
| CTC3 | 12 | 122729205 | CCT | C | 1 | NM_022916 | VPS33A | frameshift_deleti  on | exon7 | c.878_879del | p.E293fs | . | . |
| CTC3 | 12 | 124303583 | TC | T | 1 | NM_207437 | DNAH10 | frameshift_deleti  on | exon21 | c.3514delC | p.L1172fs | . | . |
| CTC3 | 12 | 124798843 | AC | A | 0.444 | NM_181709 | FAM101A | frameshift_deleti  on | exon3 | c.181delC | p.P61fs | . | . |
| CTC3 | 12 | 124887078 | C | CTGT | 1 | NM_001077261 | NCOR2 | nonframeshift_in  sertion | exon16 | c.1508_1509ins  ACA | p.Q503delinsQ  Q | . | . |
| CTC3 | 12 | 125434748 | GA | G | 0.5 | NM_032656 | DHX37 | frameshift_deleti  on | exon24 | c.3162delT | p.F1054fs | . | . |
| CTC3 | 12 | 125465269 | ACTC | A | 0.385 | NM_032656 | DHX37 | nonframeshift_de  letion | exon4 | c.502_504del | p.168_168del | . | . |
| CTC3 | 12 | 125465366 | TA | T | 1 | NM_032656 | DHX37 | frameshift_deleti  on | exon4 | c.407delT | p.V136fs | . | . |
| CTC3 | 12 | 132403104 | GC | G | 0.8 | NM_003565 | ULK1 | frameshift_deleti  on | exon23 | c.2390delC | p.A797fs | . | . |
| CTC3 | 12 | 132527853 | TC | T | 0.812 | NM_015409 | EP400 | frameshift_deleti  on | exon33 | c.6213delC | p.L2071fs | . | . |
| CTC3 | 12 | 132528253 | GA | G | 0.227 | NM_015409 | EP400 | frameshift_deleti  on | exon34 | c.6431delA | p.E2144fs | . | . |
| CTC3 | 12 | 132547102 | G | GCAA | 1 | NM_015409 | EP400 | nonframeshift_in  sertion | exon47 | c.8190_8191ins  CAA | p.Q2730delins  QQ | . | . |
| CTC3 | 12 | 132862951 | TGGCCG  CCAAGC CACCCT GGCCCA GGCCTC CTGGCC | T | 0.195 | NM_001122636 | GALNT9 | frameshift_deleti on | exon2 | c.252_303del | p.P84fs | . | . |
| CTC3 | 13 | 21265235 | CA | C | 1 | NM_006531 | IFT88 | frameshift_deleti  on | exon26 | c.2397delA | p.P799fs | . | . |
| CTC3 | 13 | 25479545 | TG | T | 0.667 | NM_018451 | CENPJ | frameshift_deleti  on | exon7 | c.2630delC | p.P877fs | . | . |
| CTC3 | 13 | 26789196 | AC | A | 0.889 | NM_005977 | RNF6 | frameshift_deleti  on | exon5 | c.822delG | p.R274fs | . | . |
| CTC3 | 13 | 28143187 | CA | C | 0.952 | NM_153371 | LNX2 | frameshift_deleti  on | exon3 | c.633delT | p.F211fs | . | . |
| CTC3 | 13 | 28611365 | AT | A | 0.722 | NM_004119 | FLT3 | frameshift_deleti  on | exon10 | c.1265delA | p.N422fs | . | . |
| CTC3 | 13 | 31287978 | G | GGTAT | 0.455 | NM_001204406 | ALOX5AP | frameshift_inserti  on | exon1 | c.116_117insGT  AT | p.W39fs | . | . |
| CTC3 | 13 | 32929216 | CT | C | 0.382 | NM_000059 | BRCA2 | frameshift_deleti  on | exon14 | c.7227delT | p.P2409fs | . | . |
| CTC3 | 13 | 36229078 | GC | G | 1 | NM_001204197 | NBEA | frameshift_deleti  on | exon13 | c.1439delC | p.A480fs | . | . |
| CTC3 | 13 | 39265975 | TG | T | 0.411 | NM_207361 | FREM2 | frameshift_deleti  on | exon1 | c.4495delG | p.D1499fs | . | . |
| CTC3 | 13 | 39541055 | AT | A | 0.341 | NM_145286 | STOML3 | frameshift_deleti  on | exon7 | c.782delA | p.N261fs | . | . |
| CTC3 | 13 | 41949650 | CCTT | C | 0.462 | NM_024561 | NAA16 | nonframeshift_de  letion | exon20 | c.2508_2510del | p.836_837del | . | . |
| CTC3 | 13 | 45580336 | GA | G | 1 | NM_018559 | GPALPP1 | frameshift_deleti  on | exon3 | c.222delA | p.R74fs | . | . |
| CTC3 | 13 | 46170719 | CCCAGA  TACTCTT CCTCCT | C | 0.316 | NM_182542 | FAM194B | nonframeshift_de letion | exon3 | c.404_421del | p.135_141del | . | . |
| CTC3 | 13 | 46801930 | AC | A | 0.187 | NM_001282460 | LRRC63 | frameshift_deleti  on | exon3 | c.370delC | p.Q124fs | . | . |
| CTC3 | 13 | 50321095 | TC | T | 0.857 | NM_002267 | KPNA3 | frameshift_deleti  on | exon2 | c.103delG | p.E35fs | . | . |
| CTC3 | 13 | 52516672 | AG | A | 0.356 | NM_001005918 | ATP7B | frameshift_deleti  on | exon11 | c.2640delC | p.T880fs | . | . |
| CTC3 | 13 | 52603924 | G | GA | 0.259 | NM_021645 | UTP14C | frameshift_inserti  on | exon2 | c.985dupA | p.K328fs | . | . |
| CTC3 | 13 | 52604555 | AC | A | 0.519 | NM_021645 | UTP14C | frameshift_deleti  on | exon2 | c.1616delC | p.T539fs | . | . |
| CTC3 | 13 | 52952510 | AG | A | 0.208 | NM_199263 | THSD1 | frameshift_deleti  on | exon4 | c.1435delC | p.L479fs | . | . |
| CTC3 | 13 | 53236794 | TA | T | 1 | NM_006704 | SUGT1 | frameshift_deleti  on | exon6 | c.340delA | p.N114fs | . | . |
| CTC3 | 13 | 76410588 | CA | C | 0.294 | NM_015842 | LMO7 | frameshift_deleti  on | exon16 | c.2691delA | p.S897fs | . | . |
| CTC3 | 13 | 76417034 | GT | G | 1 | NM_015842 | LMO7 | frameshift_deleti  on | exon22 | c.3381delT | p.G1127fs | . | . |
| CTC3 | 13 | 76419508 | TA | T | 0.329 | NM_015842 | LMO7 | frameshift_deleti  on | exon23 | c.3445delA | p.K1149fs | . | . |
| CTC3 | 13 | 77570192 | C | CT | 0.608 | NM_006493 | CLN5 | frameshift_inserti  on | exon3 | c.643dupT | p.C214fs | . | . |
| CTC3 | 13 | 77785330 | GAC | G | 0.4 | NM_015057 | MYCBP2 | frameshift_deleti  on | exon23 | c.3386_3387del | p.C1129fs | . | . |
| CTC3 | 13 | 103275310 | CT | C | 0.288 | NM_003291 | TPP2 | frameshift_deleti  on | exon6 | c.705delT | p.S235fs | . | . |

| CTC3 | 13 | 103346816 | AG | A | 0.833 | NM_001010977 | METTL21C | frameshift_deleti  on | exon1 | c.32delC | p.P11fs | . | . |
| --- | --- | --- | --- | --- | --- | --- | --- | --- | --- | --- | --- | --- | --- |
| CTC3 | 13 | 103384748 | AT | A | 1 | NM_001146197 | CCDC168 | frameshift_deleti  on | exon4 | c.18298delA | p.I6100fs | . | . |
| CTC3 | 13 | 103385924 | AT | A | 0.308 | NM_001146197 | CCDC168 | frameshift_deleti  on | exon4 | c.17122delA | p.M5708fs | . | . |
| CTC3 | 13 | 103387081 | T | TA | 0.698 | NM_001146197 | CCDC168 | frameshift_inserti  on | exon4 | c.15965dupT | p.L5322fs | . | . |
| CTC3 | 13 | 103506151 | GA | G | 0.312 | NM_000123 | ERCC5 | frameshift_deleti  on | exon3 | c.310delA | p.K104fs | . | . |
| CTC3 | 13 | 106119437 | GT | G | 1 | NM_172370 | DAOA | frameshift_deleti  on | exon2 | c.81delT | p.G27fs | . | . |
| CTC3 | 13 | 108881804 | AT | A | 0.444 | NM_032859 | ABHD13 | frameshift_deleti  on | exon2 | c.239delT | p.M80fs | . | . |
| CTC3 | 13 | 109779804 | G | GA | 0.274 | NM_001198950 | MYO16 | frameshift_inserti  on | exon31 | c.3957_3958ins  A | p.P1319fs | . | . |
| CTC3 | 13 | 110827000 | TA | T | 0.883 | NM_001845 | COL4A1 | frameshift_deleti  on | exon38 | c.3294delT | p.L1098fs | . | . |
| CTC3 | 14 | 20846216 | AC | A | 0.429 | NM_007110 | TEP1 | frameshift_deleti  on | exon39 | c.5687delG | p.G1896fs | . | . |
| CTC3 | 14 | 20871889 | C | CT | 0.378 | NM_007110 | TEP1 | frameshift_inserti  on | exon6 | c.1186_1187ins  A | p.R396fs | . | . |
| CTC3 | 14 | 21502418 | GA | G | 0.391 | NM_001012264 | RNASE13 | frameshift_deleti  on | exon2 | c.29delT | p.F10fs | . | . |
| CTC3 | 14 | 23371255 | G | GGGC | 0.921 | NM_001077352 | RBM23 | nonframeshift_in  sertion | exon10 | c.1077_1078ins  GCC | p.Q360delinsA  Q | . | . |
| CTC3 | 14 | 23848328 | CT | C | 1 | NM_001288744 | CMTM5 | frameshift_deleti  on | exon2 | c.216delT | p.A72fs | . | . |
| CTC3 | 14 | 24532570 | AT | A | 0.925 | NM_138360 | LRRC16B | frameshift_deleti  on | exon31 | c.2808delT | p.N936fs | . | . |
| CTC3 | 14 | 24532573 | TG | T | 1 | NM_138360 | LRRC16B | frameshift_deleti  on | exon31 | c.2811delG | p.L937fs | . | . |
| CTC3 | 14 | 24543942 | TG | T | 0.333 | NM_006032 | CPNE6 | frameshift_deleti  on | exon7 | c.611delG | p.W204fs | . | . |
| CTC3 | 14 | 24785215 | GC | G | 0.889 | NM_001143919 | LTB4R | frameshift_deleti  on | exon2 | c.359delC | p.A120fs | . | . |
| CTC3 | 14 | 24798317 | CA | C | 0.27 | NM_001198568 | ADCY4 | frameshift_deleti  on | exon10 | c.1473delT | p.F491fs | . | . |
| CTC3 | 14 | 26918086 | TG | T | 0.429 | NM_006489 | NOVA1 | stopgain | exon4 | c.530delC | p.S177X | . | . |
| CTC3 | 14 | 37203764 | TA | T | 0.369 | NM_001171170 | SLC25A21 | frameshift_deleti on | exon4 | c.217delT | p.Y73fs | . | . |
| CTC3 | 14 | 45403748 | AG | A | 1 | NM_017658 | KLHL28 | frameshift_deleti  on | exon3 | c.912delC | p.Y304fs | . | . |
| CTC3 | 14 | 50585189 | G | GA | 0.6 | NM_006939 | SOS2 | frameshift_inserti  on | exon23 | c.3871_3872ins  T | p.P1291fs | . | . |
| CTC3 | 14 | 53529914 | CT | C | 0.5 | NM_001160148 | DDHD1 | frameshift_deleti  on | exon7 | c.1512delA | p.K504fs | . | . |
| CTC3 | 14 | 55903654 | TC | T | 0.224 | NM_199047 | TBPL2 | frameshift_deleti  on | exon2 | c.232delG | p.D78fs | . | . |
| CTC3 | 14 | 58718931 | CT | C | 0.545 | NM_002788 | PSMA3 | frameshift_deleti  on | exon3 | c.200delT | p.L67fs | . | . |
| CTC3 | 14 | 59798115 | A | AG | 0.8 | NM_014992 | DAAM1 | frameshift_inserti  on | exon13 | c.1750dupG | p.L583fs | . | . |
| CTC3 | 14 | 62541946 | GA | G | 0.237 | NM_031914 | SYT16 | frameshift_deleti  on | exon3 | c.831delA | p.R277fs | . | . |
| CTC3 | 14 | 63174554 | TC | T | 0.75 | NM_139318 | KCNH5 | frameshift_deleti  on | exon11 | c.2638delG | p.E880fs | . | . |
| CTC3 | 14 | 64447879 | AG | A | 0.8 | NM_015180 | SYNE2 | frameshift_deleti  on | exon16 | c.1825delG | p.E609fs | . | . |
| CTC3 | 14 | 64494296 | GC | G | 1 | NM_015180 | SYNE2 | frameshift_deleti  on | exon43 | c.6500delC | p.A2167fs | . | . |
| CTC3 | 14 | 64588846 | TG | T | 0.84 | NM_015180 | SYNE2 | frameshift_deleti  on | exon69 | c.13276delG | p.A4426fs | . | . |
| CTC3 | 14 | 69697204 | T | TA | 0.343 | NM_018199 | EXD2 | frameshift_inserti  on | exon3 | c.232dupA | p.C77fs | . | . |
| CTC3 | 14 | 75555317 | TG | T | 0.75 | NM_033116 | NEK9 | frameshift_deleti  on | exon20 | c.2469delC | p.P823fs | . | . |
| CTC3 | 14 | 75593613 | CA | C | 0.438 | NM_033116 | NEK9 | frameshift_deleti  on | exon1 | c.11delT | p.L4fs | . | . |
| CTC3 | 14 | 78374249 | TCGA | T | 1 | NM_001142545 | ADCK1 | nonframeshift_de  letion | exon6 | c.642_644del | p.214_215del | . | . |
| CTC3 | 14 | 80327674 | CT | C | 0.196 | NM_001272020 | NRXN3 | frameshift_deleti  on | exon6 | c.1282delT | p.L428fs | . | . |
| CTC3 | 14 | 81223288 | GA | G | 0.643 | NM_152446 | CEP128 | frameshift_deleti  on | exon17 | c.2560delT | p.S854fs | . | . |
| CTC3 | 14 | 89338714 | TG | T | 0.422 | NM_001288782 | TTC8 | frameshift_deleti  on | exon12 | c.672delG | p.L224fs | . | . |
| CTC3 | 14 | 94394621 | GC | G | 0.448 | NM_138344 | FAM181A | frameshift_deleti  on | exon3 | c.177delC | p.G59fs | . | . |
| CTC3 | 14 | 94847262 | TC | T | 0.338 | NM_000295 | SERPINA1 | frameshift_deleti  on | exon3 | c.862delG | p.E288fs | . | . |
| CTC3 | 14 | 94912690 | AT | A | 0.778 | NM_001080451 | SERPINA11 | frameshift_deleti  on | exon3 | c.894delA | p.K298fs | . | . |
| CTC3 | 14 | 96157102 | GC | G | 0.758 | NM_004918 | TCL1B | frameshift_deleti  on | exon2 | c.193delC | p.Q65fs | . | . |
| CTC3 | 14 | 96770873 | CT | C | 0.667 | NM_018036 | ATG2B | frameshift_deleti  on | exon32 | c.4800delA | p.K1600fs | . | . |
| CTC3 | 14 | 99981595 | C | CT | 0.205 | NM_001144995 | CCDC85C | frameshift_inserti  on | exon6 | c.1247dupA | p.K416fs | . | . |
| CTC3 | 14 | 102502930 | G | GA | 1 | NM_001376 | DYNC1H1 | frameshift_inserti  on | exon57 | c.10860dupA | p.R3620fs | . | . |
| CTC3 | 14 | 102675375 | TC | T | 0.163 | NM_001242416 | WDR20 | frameshift_deleti  on | exon2 | c.686delC | p.S229fs | . | . |
| CTC3 | 14 | 102675673 | T | TC | 0.455 | NM_001242416 | WDR20 | frameshift_inserti  on | exon2 | c.984dupC | p.F328fs | . | . |
| CTC3 | 14 | 102717326 | TG | T | 0.259 | NM_001272011 | MOK | frameshift_deleti  on | exon6 | c.322delC | p.Q108fs | . | . |
| CTC3 | 14 | 103805975 | GT | G | 0.38 | NM_183004 | EIF5 | frameshift_deleti  on | exon9 | c.907delT | p.F303fs | . | . |
| CTC3 | 14 | 104139464 | G | GA | 0.151 | NM_001130107 | KLC1 | frameshift_inserti  on | exon8 | c.1102dupA | p.E367fs | . | . |
| CTC3 | 14 | 105361259 | CA | C | 0.2 | NM_015005 | CEP170B | frameshift_deleti  on | exon18 | c.4525delA | p.T1509fs | . | . |
| CTC3 | 14 | 105452981 | TG | T | 0.889 | NM_174891 | C14orf79 | frameshift_deleti  on | exon1 | c.214delG | p.G72fs | . | . |
| CTC3 | 15 | 23579214 | AGGATG  GGTAGG GAGGTG GGGATG GGTAGG | A | 0.684 | NM_001271664 | LOC440243 | nonframeshift_de letion | exon1 | c.39_74del | p.13_25del | . | . |

| CTC3 | 15 | 23579230 | TGGGGA  TGGGTA GGGAGG TGGGGA TGGGTA | T | 0.286 | NM_001271664 | LOC440243 | frameshift_deleti on | exon1 | c.22_58del | p.H8fs | . | . |
| --- | --- | --- | --- | --- | --- | --- | --- | --- | --- | --- | --- | --- | --- |
| CTC3 | 15 | 34021162 | T | TG | 0.68 | NM_001036 | RYR3 | frameshift_inserti  on | exon47 | c.7138_7139ins  G | p.F2380fs | . | . |
| CTC3 | 15 | 34048526 | GA | G | 1 | NM_001036 | RYR3 | frameshift_deleti  on | exon59 | c.8536delA | p.K2846fs | . | . |
| CTC3 | 15 | 34649469 | CA | C | 1 | NM_001284293 | NUTM1 | frameshift_deleti  on | exon7 | c.3231delA | p.A1077fs | . | . |
| CTC3 | 15 | 40068686 | GT | G | 0.255 | NM_152597 | FSIP1 | frameshift_deleti  on | exon2 | c.39delA | p.K13fs | . | . |
| CTC3 | 15 | 42057194 | GT | G | 1 | NM_001080541 | MGA | frameshift_deleti  on | exon22 | c.7229delT | p.V2410fs | . | . |
| CTC3 | 15 | 42133268 | GA | G | 0.545 | NM_001114633 | PLA2G4B | frameshift_deleti  on | exon5 | c.368delA | p.E123fs | . | . |
| CTC3 | 15 | 42492143 | C | CA | 0.323 | NM_015289 | VPS39 | frameshift_inserti  on | exon2 | c.89dupT | p.V30fs | . | . |
| CTC3 | 15 | 42983049 | GAAGC | G | 0.209 | NM_020759 | STARD9 | frameshift_deleti  on | exon23 | c.9274_9277del | p.K3092fs | . | . |
| CTC3 | 15 | 43507302 | AG | A | 1 | NM_000119 | EPB42 | frameshift_deleti  on | exon3 | c.510delC | p.P170fs | . | . |
| CTC3 | 15 | 45398364 | A | AT | 0.341 | NM_014080 | DUOX2 | frameshift_inserti  on | exon17 | c.2106dupA | p.C703fs | . | . |
| CTC3 | 15 | 48441386 | TG | T | 1 | NM_016132 | MYEF2 | frameshift_deleti  on | exon15 | c.1560delC | p.S520fs | . | . |
| CTC3 | 15 | 49304895 | CT | C | 1 | NM_014701 | SECISBP2L | frameshift_deleti  on | exon11 | c.1545delA | p.K515fs | . | . |
| CTC3 | 15 | 50785011 | G | GA | 1 | NM_001283049 | USP8 | frameshift_inserti  on | exon12 | c.2031dupA | p.G677fs | . | . |
| CTC3 | 15 | 51791606 | GC | G | 0.171 | NM_001174116 | DMXL2 | frameshift_deleti  on | exon18 | c.3814delG | p.A1272fs | . | . |
| CTC3 | 15 | 52613567 | TGAAG | T | 0.698 | NM_001142495 | MYO5A | frameshift_deleti  on | exon36 | c.4780_4783del | p.L1594fs | . | . |
| CTC3 | 15 | 54305443 | GA | G | 0.3 | NM_001080534 | UNC13C | frameshift_deleti  on | exon1 | c.344delA | p.D115fs | . | . |
| CTC3 | 15 | 55759181 | AT | A | 0.556 | NM_001033559 | DYX1C1 | stopgain | exon5 | c.583delA | p.I195X | . | . |
| CTC3 | 15 | 58287305 | GT | G | 0.333 | NM_170697 | ALDH1A2 | frameshift_deleti on | exon3 | c.237delA | p.E79fs | . | . |
| CTC3 | 15 | 59910561 | CT | C | 0.5 | NM_004751 | GCNT3 | frameshift_deleti  on | exon3 | c.125delT | p.L42fs | . | . |
| CTC3 | 15 | 59911270 | TA | T | 0.615 | NM_004751 | GCNT3 | frameshift_deleti  on | exon3 | c.834delA | p.L278fs | . | . |
| CTC3 | 15 | 60741675 | AT | A | 0.5 | NM_001018089 | NARG2 | frameshift_deleti  on | exon10 | c.1079delA | p.N360fs | . | . |
| CTC3 | 15 | 63031591 | G | GA | 1 | NM_015059 | TLN2 | frameshift_inserti  on | exon28 | c.3733dupA | p.L1244fs | . | . |
| CTC3 | 15 | 63632925 | AC | A | 0.38 | NM_001218 | CA12 | frameshift_deleti  on | exon6 | c.582delG | p.K194fs | . | . |
| CTC3 | 15 | 64972994 | A | ACAC | 0.24 | NM_015042 | ZNF609 | nonframeshift_in  sertion | exon7 | c.4095_4096ins  CAC | p.T1365delinsT  H | . | . |
| CTC3 | 15 | 65703597 | GC | G | 0.385 | NM_020962 | IGDCC4 | frameshift_deleti  on | exon2 | c.181delG | p.A61fs | . | . |
| CTC3 | 15 | 68378853 | CA | C | 0.571 | NM_016166 | PIAS1 | frameshift_deleti  on | exon2 | c.235delA | p.N79fs | . | . |
| CTC3 | 15 | 71124464 | AC | A | 0.459 | NM_001286679 | LARP6 | frameshift_deleti  on | exon3 | c.850delG | p.V284fs | . | . |
| CTC3 | 15 | 72104510 | G | GA | 0.798 | NM_014249 | NR2E3 | frameshift_inserti  on | exon4 | c.566dupA | p.E189fs | . | . |
| CTC3 | 15 | 72300287 | GA | G | 0.235 | NM_006901 | MYO9A | frameshift_deleti  on | exon8 | c.1259delT | p.F420fs | . | . |
| CTC3 | 15 | 73590764 | AC | A | 0.299 | NM_001172623 | NEO1 | frameshift_deleti  on | exon26 | c.3819delC | p.D1273fs | . | . |
| CTC3 | 15 | 74865241 | C | CG | 0.273 | NM_006465 | ARID3B | frameshift_inserti  on | exon3 | c.591_592insG | p.G197fs | . | . |
| CTC3 | 15 | 77407281 | AG | A | 0.25 | NM_024776 | PEAK1 | frameshift_deleti  on | exon8 | c.4457delC | p.P1486fs | . | . |
| CTC3 | 15 | 77407479 | CT | C | 0.809 | NM_024776 | PEAK1 | frameshift_deleti  on | exon8 | c.4259delA | p.E1420fs | . | . |
| CTC3 | 15 | 78500419 | A | AT | 0.283 | NM_001199377 | ACSBG1 | frameshift_inserti  on | exon2 | c.156_157insA | p.S53fs | . | . |
| CTC3 | 15 | 78923490 | CG | C | 0.577 | NM_000750 | CHRNB4 | frameshift_deleti  on | exon4 | c.286delC | p.R96fs | . | . |
| CTC3 | 15 | 79183352 | GA | G | 0.265 | NM_001265603 | MORF4L1 | frameshift_deleti  on | exon4 | c.82delA | p.K28fs | . | . |
| CTC3 | 15 | 79606227 | GT | G | 0.6 | NM_007364 | TMED3 | frameshift_deleti  on | exon2 | c.298delT | p.F100fs | . | . |
| CTC3 | 15 | 79749134 | CT | C | 0.643 | NM_015206 | KIAA1024 | frameshift_deleti  on | exon2 | c.646delT | p.F216fs | . | . |
| CTC3 | 15 | 81592161 | GCCT | G | 0.13 | NM_004513 | IL16 | nonframeshift_de  letion | exon2 | c.392_394del | p.131_132del | . | . |
| CTC3 | 15 | 82444012 | TG | T | 0.533 | NM_001040610 | EFTUD1 | frameshift_deleti  on | exon16 | c.2629delC | p.Q877fs | . | . |
| CTC3 | 15 | 85325929 | AT | A | 0.421 | NM_014630 | ZNF592 | frameshift_deleti  on | exon4 | c.24delT | p.D8fs | . | . |
| CTC3 | 15 | 85399869 | CG | C | 0.677 | NM_020778 | ALPK3 | frameshift_deleti  on | exon6 | c.2507delG | p.R836fs | . | . |
| CTC3 | 15 | 88678621 | TG | T | 0.4 | NM_001007156 | NTRK3 | frameshift_deleti  on | exon10 | c.914delC | p.P305fs | . | . |
| CTC3 | 15 | 89414719 | GC | G | 0.889 | NM_001135 | ACAN | frameshift_deleti  on | exon13 | c.6940delC | p.Q2314fs | . | . |
| CTC3 | 15 | 90335494 | ACTC | A | 1 | NM_001150 | ANPEP | nonframeshift_de  letion | exon18 | c.2422_2424del | p.808_808del | . | . |
| CTC3 | 15 | 90414743 | AT | A | 0.776 | NM_005829 | AP3S2 | frameshift_deleti  on | exon4 | c.308delA | p.N103fs | . | . |
| CTC3 | 15 | 90969423 | C | CA | 0.741 | NM_003870 | IQGAP1 | frameshift_inserti  on | exon3 | c.238dupA | p.A79fs | . | . |
| CTC3 | 15 | 91292880 | GT | G | 0.584 | NM_000057 | BLM | frameshift_deleti  on | exon3 | c.383delT | p.V128fs | . | . |
| CTC3 | 15 | 91292995 | C | CA | 0.513 | NM_000057 | BLM | frameshift_inserti  on | exon3 | c.498dupA | p.S166fs | . | . |
| CTC3 | 15 | 100252709 | CCAG | C | 1 | NM_001130928 | MEF2A | nonframeshift_de  letion | exon8 | c.1024_1026del | p.342_342del | . | . |
| CTC3 | 15 | 100252709 | CCAGCA  G | C | 1 | NM_001130928 | MEF2A | nonframeshift_de  letion | exon8 | c.1024_1029del | p.342_343del | . | . |
| CTC3 | 16 | 2139800 | AC | A | 1 | NM_000296 | PKD1 | frameshift_deleti  on | exon46 | c.12836delG | p.G4279fs | . | . |
| CTC3 | 16 | 3100064 | TG | T | 1 | NM_022468 | MMP25 | frameshift_deleti  on | exon3 | c.288delG | p.L96fs | . | . |
| CTC3 | 16 | 3118185 | AC | A | 0.191 | NM_001012636 | IL32 | frameshift_deleti  on | exon4 | c.120delC | p.D40fs | . | . |
| CTC3 | 16 | 4933899 | A | AT | 0.984 | NM_002705 | PPL | frameshift_inserti  on | exon22 | c.4756dupA | p.I1586fs | . | . |

| CTC3 | 16 | 4933902 | T | TC | 0.6 | NM_002705 | PPL | frameshift_inserti  on | exon22 | c.4753dupG | p.E1585fs | . | . |
| --- | --- | --- | --- | --- | --- | --- | --- | --- | --- | --- | --- | --- | --- |
| CTC3 | 16 | 5140179 | TA | T | 0.398 | NM_001289029 | FAM86A | stopgain | exon4 | c.464delT | p.L155X | . | . |
| CTC3 | 16 | 11362950 | CG | C | 1 | NM_005425 | TNP2 | frameshift_deleti on | exon1 | c.169delC | p.R57fs | . | . |
| CTC3 | 16 | 14041733 | GT | G | 1 | NM_005236 | ERCC4 | frameshift_deleti  on | exon11 | c.2281delT | p.F761fs | . | . |
| CTC3 | 16 | 15844084 | A | AG | 0.56 | NM_002474 | MYH11 | frameshift_inserti  on | exon16 | c.1968_1969ins  C | p.Y657fs | . | . |
| CTC3 | 16 | 18907504 | AG | A | 0.308 | NM_015092 | SMG1 | frameshift_deleti  on | exon3 | c.281delC | p.S94fs | . | . |
| CTC3 | 16 | 19058505 | CT | C | 1 | NM_001160364 | TMC7 | frameshift_deleti  on | exon12 | c.1345delT | p.F449fs | . | . |
| CTC3 | 16 | 19519079 | AT | A | 1 | NM_016641 | GDE1 | frameshift_deleti  on | exon4 | c.565delA | p.M189fs | . | . |
| CTC3 | 16 | 20376828 | CA | C | 0.8 | NM_174924 | PDILT | frameshift_deleti  on | exon9 | c.1150delT | p.W384fs | . | . |
| CTC3 | 16 | 20381003 | GA | G | 0.31 | NM_174924 | PDILT | frameshift_deleti  on | exon8 | c.926delT | p.F309fs | . | . |
| CTC3 | 16 | 20552049 | TC | T | 0.266 | NM_001105069 | ACSM2B | frameshift_deleti  on | exon13 | c.1555delG | p.D519fs | . | . |
| CTC3 | 16 | 20554579 | AT | A | 1 | NM_001105069 | ACSM2B | frameshift_deleti  on | exon11 | c.1286delA | p.N429fs | . | . |
| CTC3 | 16 | 20809950 | GA | G | 1 | NM_001142725 | ERI2 | frameshift_deleti  on | exon9 | c.1171delT | p.S391fs | . | . |
| CTC3 | 16 | 21065841 | CT | C | 0.5 | NM_017539 | DNAH3 | frameshift_deleti  on | exon28 | c.3938delA | p.K1313fs | . | . |
| CTC3 | 16 | 21123003 | AT | A | 0.409 | NM_017539 | DNAH3 | frameshift_deleti  on | exon14 | c.2042delA | p.N681fs | . | . |
| CTC3 | 16 | 23400286 | A | AG | 0.167 | NM_153603 | COG7 | frameshift_inserti  on | exon17 | c.2267_2268ins  C | p.R756fs | . | . |
| CTC3 | 16 | 27508983 | CG | C | 0.467 | NM_001286242 | GTF3C1 | frameshift_deleti  on | exon14 | c.2324delC | p.P775fs | . | . |
| CTC3 | 16 | 28855616 | AT | A | 1 | NM_003321 | TUFM | frameshift_deleti  on | exon7 | c.856delA | p.I286fs | . | . |
| CTC3 | 16 | 28884793 | CCAGCC  CCCTGA ACCCCC TTCATG GACAGA | C | 0.12 | NM_001145795 | SH2B1 | nonframeshift_de letion | exon9 | c.1924_1959del | p.642_653del | . | . |
| CTC3 | 16 | 28944601 | AC | A | 0.6 | NM_001178098 | CD19 | frameshift_deleti  on | exon4 | c.607delC | p.P203fs | . | . |
| CTC3 | 16 | 28994192 | TG | T | 0.438 | NM_001142449 | SPNS1 | frameshift_deleti  on | exon7 | c.949delG | p.G317fs | . | . |
| CTC3 | 16 | 28998160 | GC | G | 0.353 | NM_014387 | LAT | frameshift_deleti  on | exon7 | c.527delC | p.A176fs | . | . |
| CTC3 | 16 | 30545681 | GC | G | 0.446 | NM_023931 | ZNF747 | frameshift_deleti  on | exon1 | c.319delG | p.A107fs | . | . |
| CTC3 | 16 | 30731505 | AC | A | 0.196 | NM_006662 | SRCAP | frameshift_deleti  on | exon19 | c.2841delC | p.D947fs | . | . |
| CTC3 | 16 | 30732059 | GA | G | 0.358 | NM_006662 | SRCAP | frameshift_deleti  on | exon20 | c.3014delA | p.E1005fs | . | . |
| CTC3 | 16 | 30735726 | CA | C | 0.265 | NM_006662 | SRCAP | frameshift_deleti  on | exon25 | c.4982delA | p.Q1661fs | . | . |
| CTC3 | 16 | 30745885 | TGAA | T | 1 | NM_006662 | SRCAP | nonframeshift_de  letion | exon31 | c.6679_6681del | p.2227_2227de  l | . | . |
| CTC3 | 16 | 30794111 | AG | A | 0.625 | NM_001080417 | ZNF629 | frameshift_deleti  on | exon3 | c.1537delC | p.L513fs | . | . |
| CTC3 | 16 | 47005315 | TG | T | 1 | NM_005880 | DNAJA2 | frameshift_deleti  on | exon3 | c.307delC | p.Q103fs | . | . |
| CTC3 | 16 | 47143605 | TA | T | 0.202 | NM_001201477 | NETO2 | frameshift_deleti  on | exon7 | c.650delT | p.L217fs | . | . |
| CTC3 | 16 | 50368677 | CCT | C | 1 | NM_001173984 | BRD7 | frameshift_deleti  on | exon7 | c.830_831del | p.E277fs | . | . |
| CTC3 | 16 | 50368677 | CCTCT | C | 1 | NM_001173984 | BRD7 | frameshift_deleti  on | exon7 | c.828_831del | p.R276fs | . | . |
| CTC3 | 16 | 51172957 | TG | T | 1 | NM_001127892 | SALL1 | frameshift_deleti  on | exon2 | c.2884delC | p.Q962fs | . | . |
| CTC3 | 16 | 51175655 | CGCT | C | 0.25 | NM_001127892 | SALL1 | nonframeshift_de  letion | exon2 | c.184_186del | p.62_62del | . | . |
| CTC3 | 16 | 53265613 | TG | T | 0.197 | NM_025134 | CHD9 | frameshift_deleti  on | exon10 | c.2429delG | p.W810fs | . | . |
| CTC3 | 16 | 55907932 | GC | G | 0.5 | NM_001143685 | CES5A | frameshift_deleti  on | exon2 | c.90delG | p.G30fs | . | . |
| CTC3 | 16 | 55983774 | AC | A | 0.35 | NM_001190158 | CES5A | frameshift_deleti  on | exon2 | c.82delG | p.V28fs | . | . |
| CTC3 | 16 | 56692581 | TCGCAG  GTGTCTC CTGCAC CTGCGC | T | 0.361 | NM_005949 | MT1F | frameshift_deleti on | exon2 | c.29_54del | p.G10fs | . | . |
| CTC3 | 16 | 56692586 | GGTGTC  TCCTGC ACCTGC GCTGGT | G | 0.119 | NM_005949 | MT1F | frameshift_deleti on | exon2 | c.29_59del | p.G10fs | . | . |
| CTC3 | 16 | 56870529 | AG | A | 0.444 | NM_001242795 | NUP93 | frameshift_deleti  on | exon15 | c.1431delG | p.K477fs | . | . |
| CTC3 | 16 | 61851634 | A | AG | 0.5 | NM_001796 | CDH8 | frameshift_inserti  on | exon7 | c.1025dupC | p.P342fs | . | . |
| CTC3 | 16 | 66423375 | TG | T | 0.243 | NM_001795 | CDH5 | frameshift_deleti  on | exon5 | c.732delG | p.V244fs | . | . |
| CTC3 | 16 | 66824856 | TG | T | 1 | NM_001136505 | CCDC79 | frameshift_deleti  on | exon4 | c.97delC | p.Q33fs | . | . |
| CTC3 | 16 | 67271989 | A | AC | 0.449 | NM_013241 | FHOD1 | frameshift_inserti  on | exon6 | c.589dupG | p.V197fs | . | . |
| CTC3 | 16 | 67300016 | TGAG | T | 0.137 | NM_004594 | SLC9A5 | nonframeshift_de  letion | exon15 | c.2107_2109del | p.703_703del | . | . |
| CTC3 | 16 | 67861791 | CGA | C | 0.35 | NM_001288994 | TSNAXIP1 | frameshift_deleti  on | exon11 | c.997_998del | p.E333fs | . | . |
| CTC3 | 16 | 67964198 | GC | G | 1 | NM_001907 | CTRL | frameshift_deleti  on | exon6 | c.522delG | p.L174fs | . | . |
| CTC3 | 16 | 68156203 | AT | A | 0.375 | NM_004555 | NFATC3 | frameshift_deleti  on | exon2 | c.418delT | p.F140fs | . | . |
| CTC3 | 16 | 68264804 | AC | A | 1 | NM_024939 | ESRP2 | frameshift_deleti  on | exon13 | c.1837delG | p.V613fs | . | . |
| CTC3 | 16 | 70398985 | CA | C | 0.414 | NM_018332 | DDX19A | frameshift_deleti  on | exon7 | c.556delA | p.K186fs | . | . |
| CTC3 | 16 | 70501297 | CGG | C | 0.75 | NM_145059 | FUK | frameshift_deleti  on | exon7 | c.506_507del | p.R169fs | . | . |
| CTC3 | 16 | 70569230 | TC | T | 0.31 | NM_012426 | SF3B3 | frameshift_deleti  on | exon6 | c.733delC | p.P245fs | . | . |
| CTC3 | 16 | 72120661 | TG | T | 0.5 | NM_001142317 | TXNL4B | frameshift_deleti  on | exon4 | c.324delC | p.T108fs | . | . |

| CTC3 | 16 | 72830468 | TC | T | 0.75 | NM_001164766 | ZFHX3 | frameshift_deleti  on | exon8 | c.3370delG | p.E1124fs | . | . |
| --- | --- | --- | --- | --- | --- | --- | --- | --- | --- | --- | --- | --- | --- |
| CTC3 | 16 | 75670385 | TC | T | 0.5 | NM_005548 | KARS | frameshift_deleti  on | exon4 | c.448delG | p.E150fs | . | . |
| CTC3 | 16 | 76556141 | TC | T | 0.286 | NM_138994 | CNTNAP4 | frameshift_deleti  on | exon16 | c.2524delC | p.Q842fs | . | . |
| CTC3 | 16 | 77401594 | AT | A | 0.444 | NM_199355 | ADAMTS18 | frameshift_deleti  on | exon4 | c.521delA | p.N174fs | . | . |
| CTC3 | 16 | 80583477 | AT | A | 0.149 | NM_130897 | DYNLRB2 | frameshift_deleti  on | exon3 | c.177delT | p.D59fs | . | . |
| CTC3 | 16 | 81077073 | TCA | T | 0.533 | NM_015251 | ATMIN | frameshift_deleti  on | exon4 | c.971_972del | p.S324fs | . | . |
| CTC3 | 16 | 81078182 | C | CT | 0.368 | NM_015251 | ATMIN | frameshift_inserti  on | exon4 | c.2080dupT | p.N693fs | . | . |
| CTC3 | 16 | 81204375 | AG | A | 0.947 | NM_001278423 | PKD1L2 | frameshift_deleti  on | exon7 | c.890delC | p.P297fs | . | . |
| CTC3 | 16 | 82034403 | CA | C | 0.424 | NM_145168 | SDR42E1 | frameshift_deleti  on | exon2 | c.60delT | p.F20fs | . | . |
| CTC3 | 16 | 84127400 | CA | C | 0.448 | NM_003791 | MBTPS1 | frameshift_deleti  on | exon5 | c.651delT | p.F217fs | . | . |
| CTC3 | 16 | 84163626 | G | GT | 0.25 | NM_031463 | HSDL1 | frameshift_inserti  on | exon4 | c.630dupA | p.P211fs | . | . |
| CTC3 | 16 | 84270565 | G | GCCAG  CTCCT | 0.958 | NM_172347 | KCNG4 | nonframeshift_in  sertion | exon2 | c.526_527insA  GGAGCTGG | p.A176delinsEE  LA | . | . |
| CTC3 | 16 | 84271058 | G | GA | 0.9 | NM_172347 | KCNG4 | frameshift_inserti  on | exon2 | c.33dupT | p.P12fs | . | . |
| CTC3 | 16 | 85813388 | CG | C | 0.241 | NM_006067 | EMC8 | frameshift_deleti  on | exon5 | c.558delC | p.F186fs | . | . |
| CTC3 | 16 | 87344828 | GC | G | 0.361 | NM_001195124 | C16orf95 | frameshift_deleti  on | exon4 | c.404delG | p.G135fs | . | . |
| CTC3 | 16 | 87795566 | TG | T | 1 | NM_001184856 | KLHDC4 | frameshift_deleti  on | exon2 | c.179delC | p.P60fs | . | . |
| CTC3 | 16 | 88504778 | GC | G | 1 | NM_001127464 | ZNF469 | frameshift_deleti  on | exon2 | c.10817delC | p.A3606fs | . | . |
| CTC3 | 16 | 88800418 | TG | T | 0.8 | NM_001142864 | PIEZO1 | frameshift_deleti  on | exon17 | c.2224delC | p.Q742fs | . | . |
| CTC3 | 16 | 89348534 | CTCG | C | 0.3 | NM_001256183 | ANKRD11 | nonframeshift_de  letion | exon9 | c.4413_4415del | p.1471_1472de  l | . | . |
| CTC3 | 16 | 89349051 | AT | A | 0.314 | NM_001256183 | ANKRD11 | frameshift_deleti  on | exon9 | c.3898delA | p.I1300fs | . | . |
| CTC3 | 16 | 89351086 | G | GT | 0.727 | NM_001256183 | ANKRD11 | frameshift_inserti  on | exon9 | c.1863dupA | p.L622fs | . | . |
| CTC3 | 16 | 89351279 | CG | C | 0.556 | NM_001256183 | ANKRD11 | frameshift_deleti  on | exon9 | c.1670delC | p.P557fs | . | . |
| CTC3 | 17 | 260394 | GA | G | 0.441 | NM_001013672 | C17orf97 | frameshift_deleti  on | exon1 | c.262delA | p.K88fs | . | . |
| CTC3 | 17 | 1582127 | C | CT | 0.181 | NM_006445 | PRPF8 | frameshift_inserti  on | exon12 | c.1647dupA | p.V550fs | . | . |
| CTC3 | 17 | 1582667 | C | CT | 0.304 | NM_006445 | PRPF8 | frameshift_inserti  on | exon10 | c.1326dupA | p.V443fs | . | . |
| CTC3 | 17 | 2995642 | A | AT | 0.301 | NM_002548 | OR1D2 | frameshift_inserti  on | exon1 | c.648_649insA | p.S217fs | . | . |
| CTC3 | 17 | 3495390 | CT | C | 0.24 | NM_080706 | TRPV1 | frameshift_deleti  on | exon1 | c.254delA | p.Q85fs | . | . |
| CTC3 | 17 | 3561361 | A | AT | 0.923 | NM_001031681 | CTNS | frameshift_inserti  on | exon10 | c.745dupT | p.A248fs | . | . |
| CTC3 | 17 | 3967927 | AG | A | 0.455 | NM_015113 | ZZEF1 | frameshift_deleti  on | exon29 | c.4445delC | p.P1482fs | . | . |
| CTC3 | 17 | 4458202 | AC | A | 0.5 | NM_001105538 | MYBBP1A | frameshift_deleti  on | exon2 | c.247delG | p.V83fs | . | . |
| CTC3 | 17 | 5012719 | AT | A | 0.263 | NM_014519 | ZNF232 | frameshift_deleti  on | exon3 | c.467delA | p.D156fs | . | . |
| CTC3 | 17 | 5085129 | TA | T | 0.941 | NM_032530 | ZNF594 | stoploss | exon2 | c.2422delT | p.X808R | . | . |
| CTC3 | 17 | 6555546 | C | CG | 1 | NM_001105520 | C17orf100 | frameshift_inserti on | exon1 | c.314dupG | p.R105fs | . | . |
| CTC3 | 17 | 8053442 | TG | T | 1 | NM_002616 | PER1 | frameshift_deleti  on | exon4 | c.375delC | p.S125fs | . | . |
| CTC3 | 17 | 8168620 | C | CA | 0.571 | NM_012393 | PFAS | frameshift_inserti  on | exon19 | c.2295_2296ins  A | p.S765fs | . | . |
| CTC3 | 17 | 8192145 | CT | C | 0.983 | NM_001177801 | RANGRF | frameshift_deleti  on | exon1 | c.40delT | p.F14fs | . | . |
| CTC3 | 17 | 10317609 | A | AT | 0.17 | NM_002472 | MYH8 | frameshift_inserti  on | exon11 | c.907dupA | p.M303fs | . | . |
| CTC3 | 17 | 10400642 | TG | T | 0.8 | NM_005963 | MYH1 | frameshift_deleti  on | exon32 | c.4492delC | p.Q1498fs | . | . |
| CTC3 | 17 | 10448707 | TG | T | 0.282 | NM_001100112 | MYH2 | frameshift_deleti  on | exon5 | c.460delC | p.H154fs | . | . |
| CTC3 | 17 | 10451207 | CAA | C | 0.23 | NM_001100112 | MYH2 | frameshift_deleti  on | exon3 | c.29_30del | p.F10fs | . | . |
| CTC3 | 17 | 11666879 | AT | A | 0.283 | NM_001372 | DNAH9 | frameshift_deleti  on | exon36 | c.7119delT | p.Y2373fs | . | . |
| CTC3 | 17 | 17699837 | GC | G | 1 | NM_030665 | RAI1 | frameshift_deleti  on | exon3 | c.3576delC | p.S1192fs | . | . |
| CTC3 | 17 | 18880171 | TC | T | 0.833 | NM_001270648 | SLC5A10 | frameshift_deleti  on | exon8 | c.771delC | p.I257fs | . | . |
| CTC3 | 17 | 18880910 | TC | T | 0.429 | NM_001039999 | FAM83G | frameshift_deleti  on | exon5 | c.2068delG | p.D690fs | . | . |
| CTC3 | 17 | 19235215 | TG | T | 0.5 | NM_001102664 | EPN2 | frameshift_deleti  on | exon7 | c.607delG | p.D203fs | . | . |
| CTC3 | 17 | 19458975 | G | GA | 1 | NM_018242 | SLC47A1 | frameshift_inserti  on | exon8 | c.712dupA | p.G237fs | . | . |
| CTC3 | 17 | 25919586 | CA | C | 1 | NM_014238 | KSR1 | frameshift_deleti  on | exon9 | c.823delA | p.N275fs | . | . |
| CTC3 | 17 | 26109098 | TC | T | 1 | NM_000625 | NOS2 | frameshift_deleti  on | exon7 | c.664delG | p.E222fs | . | . |
| CTC3 | 17 | 26905742 | TG | T | 0.2 | NM_006461 | SPAG5 | frameshift_deleti  on | exon20 | c.3145delC | p.Q1049fs | . | . |
| CTC3 | 17 | 27065254 | TG | T | 0.895 | NM_178170 | NEK8 | frameshift_deleti  on | exon8 | c.1214delG | p.C405fs | . | . |
| CTC3 | 17 | 27438575 | T | TG | 0.595 | NM_078471 | MYO18A | frameshift_inserti  on | exon17 | c.2766dupC | p.K923fs | . | . |
| CTC3 | 17 | 27493318 | GGCA | G | 0.232 | NM_078471 | MYO18A | nonframeshift_de  letion | exon2 | c.638_640del | p.213_214del | . | . |
| CTC3 | 17 | 27493320 | C | CA | 0.176 | NM_078471 | MYO18A | frameshift_inserti  on | exon2 | c.638dupT | p.L213fs | . | . |
| CTC3 | 17 | 27614467 | TC | T | 0.667 | NM_020772 | NUFIP2 | frameshift_deleti  on | exon2 | c.544delG | p.D182fs | . | . |
| CTC3 | 17 | 28772788 | AC | A | 1 | NM_001199775 | CPD | frameshift_deleti  on | exon12 | c.1883delC | p.T628fs | . | . |
| CTC3 | 17 | 29196629 | G | GT | 0.909 | NM_024857 | ATAD5 | frameshift_inserti  on | exon14 | c.3573dupT | p.C1191fs | . | . |
| CTC3 | 17 | 32598265 | TC | T | 0.333 | NM_006273 | CCL7 | frameshift_deleti  on | exon2 | c.178delC | p.P60fs | . | . |
| CTC3 | 17 | 33772601 | CT | C | 1 | NM_144682 | SLFN13 | frameshift_deleti  on | exon3 | c.98delA | p.K33fs | . | . |

| CTC3 | 17 | 33806619 | TA | T | 0.868 | NM_001195790 | SLFN12L | frameshift_deleti  on | exon2 | c.609delT | p.F203fs | . | . |
| --- | --- | --- | --- | --- | --- | --- | --- | --- | --- | --- | --- | --- | --- |
| CTC3 | 17 | 34853517 | CT | C | 0.205 | NM_001281432 | ZNHIT3 | frameshift_deleti  on | exon5 | c.355delT | p.S119fs | . | . |
| CTC3 | 17 | 34854294 | TC | T | 0.8 | NM_025109 | MYO19 | frameshift_deleti  on | exon22 | c.1972delG | p.E658fs | . | . |
| CTC3 | 17 | 38324468 | AC | A | 0.6 | NM_007359 | CASC3 | frameshift_deleti  on | exon11 | c.1764delC | p.H588fs | . | . |
| CTC3 | 17 | 38421131 | TC | T | 0.636 | NM_133264 | WIPF2 | frameshift_deleti  on | exon5 | c.704delC | p.S235fs | . | . |
| CTC3 | 17 | 38816249 | CT | C | 0.8 | NM_152349 | KRT222 | frameshift_deleti  on | exon3 | c.435delA | p.K145fs | . | . |
| CTC3 | 17 | 38949427 | C | CT | 0.5 | NM_181535 | KRT28 | frameshift_inserti  on | exon7 | c.1231dupA | p.S411fs | . | . |
| CTC3 | 17 | 38978233 | GT | G | 0.927 | NM_000421 | KRT10 | frameshift_deleti  on | exon1 | c.604delA | p.T202fs | . | . |
| CTC3 | 17 | 39305774 | C | CCGGC  AGCAG | 0.385 | NM_033188 | KRTAP4-5 | nonframeshift_in  sertion | exon1 | c.245_246insCC  CCAGCTGCTG | p.Q82delinsHP  SCCR | . | . |
| CTC3 | 17 | 39305775 | T | TGGCA  GCAGC | 0.569 | NM_033188 | KRTAP4-5 | nonframeshift_in  sertion | exon1 | c.244_245insG  CCCCAGCTGC | p.Q82delinsRP  SCCQ | . | . |
| CTC3 | 17 | 39305784 | C | CTGGG  GCGGC | 0.483 | NM_033188 | KRTAP4-5 | nonframeshift_in  sertion | exon1 | c.235_236insCC  TGCTGCCGCC | p.C79delinsSC  CRPS | . | . |
| CTC3 | 17 | 39324229 | T | TGCAG  CAGGT | 0.667 | NM_033187 | KRTAP4-3 | nonframeshift_in  sertion | exon1 | c.195_196insCT  GACCACCTGC | p.R66delinsLTT  CCR | . | . |
| CTC3 | 17 | 39324232 | A | AGCAG  GTGGT | 0.632 | NM_033187 | KRTAP4-3 | nonframeshift_in  sertion | exon1 | c.192_193insA  GCCTGACCAC | p.C65delinsSLT  TCC | . | . |
| CTC3 | 17 | 39346595 | T | TGCTGT  GGGTC CAGCT GCTGC | 1 | NM_001190460 | KRTAP9-1 | nonframeshift_in sertion | exon1 | c.457_458insG  CTGTGGGTCC AGCTGCTGCC AGCCTA | p.C153delinsC CGSSCCQPS | . | . |
| CTC3 | 17 | 39464759 | AC | A | 0.481 | NM_001146182 | KRTAP16-1 | frameshift_deleti  on | exon1 | c.746delG | p.C249fs | . | . |
| CTC3 | 17 | 39550313 | AC | A | 1 | NM_002277 | KRT31 | frameshift_deleti  on | exon7 | c.1205delG | p.C402fs | . | . |
| CTC3 | 17 | 39623421 | GC | G | 0.194 | NM_002278 | KRT32 | frameshift_deleti  on | exon1 | c.156delG | p.L52fs | . | . |
| CTC3 | 17 | 39684319 | AG | A | 0.538 | NM_002276 | KRT19 | frameshift_deleti  on | exon1 | c.180delC | p.A60fs | . | . |
| CTC3 | 17 | 40063724 | GA | G | 0.571 | NM_001096 | ACLY | frameshift_deleti  on | exon7 | c.717delT | p.P239fs | . | . |
| CTC3 | 17 | 41243738 | GC | G | 0.517 | NM_007297 | BRCA1 | frameshift_deleti  on | exon9 | c.3668delG | p.C1223fs | . | . |
| CTC3 | 17 | 41576279 | AG | A | 0.357 | NM_004941 | DHX8 | frameshift_deleti  on | exon10 | c.1351delG | p.G451fs | . | . |
| CTC3 | 17 | 41931308 | AG | A | 0.294 | NM_001168323 | CD300LG | frameshift_deleti  on | exon3 | c.514delG | p.A172fs | . | . |
| CTC3 | 17 | 42284702 | CGT | C | 0.188 | NM_001076683 | UBTF | frameshift_deleti  on | exon20 | c.2090_2091del | p.D697fs | . | . |
| CTC3 | 17 | 42453281 | AG | A | 0.229 | NM_000419 | ITGA2B | frameshift_deleti  on | exon25 | c.2520delC | p.P840fs | . | . |
| CTC3 | 17 | 42818786 | GC | G | 0.551 | NM_025104 | DBF4B | frameshift_deleti  on | exon10 | c.797delC | p.A266fs | . | . |
| CTC3 | 17 | 42936478 | CA | C | 0.429 | NM_001142605 | EFTUD2 | frameshift_deleti  on | exon18 | c.1826delT | p.L609fs | . | . |
| CTC3 | 17 | 45669351 | TG | T | 0.442 | NM_006310 | NPEPPS | frameshift_deleti  on | exon11 | c.1291delG | p.E431fs | . | . |
| CTC3 | 17 | 47916010 | TG | T | 0.214 | NM_001077505 | TAC4 | stopgain | exon3 | c.224delC | p.S75X | . | . |
| CTC3 | 17 | 48435869 | TG | T | 0.386 | NM_022167 | XYLT2 | frameshift_deleti on | exon10 | c.2244delG | p.L748fs | . | . |
| CTC3 | 17 | 48557269 | AC | A | 1 | NM_018346 | RSAD1 | frameshift_deleti  on | exon3 | c.299delC | p.T100fs | . | . |
| CTC3 | 17 | 49123971 | CG | C | 0.6 | NM_001251971 | SPAG9 | frameshift_deleti  on | exon1 | c.50delC | p.A17fs | . | . |
| CTC3 | 17 | 55027903 | C | CT | 0.571 | NM_004645 | COIL | frameshift_inserti  on | exon2 | c.699dupA | p.G234fs | . | . |
| CTC3 | 17 | 55752360 | TC | T | 0.6 | NM_138962 | MSI2 | frameshift_deleti  on | exon12 | c.819delC | p.F273fs | . | . |
| CTC3 | 17 | 57058380 | TA | T | 0.817 | NM_014906 | PPM1E | frameshift_deleti  on | exon7 | c.2257delA | p.K753fs | . | . |
| CTC3 | 17 | 57134238 | TA | T | 0.431 | NM_001005207 | TRIM37 | stopgain | exon13 | c.1196delT | p.L399X | . | . |
| CTC3 | 17 | 57134313 | GC | G | 0.908 | NM_001005207 | TRIM37 | frameshift_deleti on | exon13 | c.1121delG | p.G374fs | . | . |
| CTC3 | 17 | 58740797 | AC | A | 0.5 | NM_003620 | PPM1D | frameshift_deleti  on | exon6 | c.1703delC | p.T568fs | . | . |
| CTC3 | 17 | 58756886 | GC | G | 0.692 | NM_001099432 | BCAS3 | frameshift_deleti  on | exon2 | c.69delC | p.R23fs | . | . |
| CTC3 | 17 | 59155786 | AG | A | 0.22 | NM_017679 | BCAS3 | frameshift_deleti  on | exon21 | c.2224delG | p.V742fs | . | . |
| CTC3 | 17 | 59480527 | GA | G | 1 | NM_005994 | TBX2 | frameshift_deleti  on | exon3 | c.770delA | p.E257fs | . | . |
| CTC3 | 17 | 62078473 | TGAG | T | 0.159 | NM_001164257 | C17orf72 | nonframeshift_de  letion | exon4 | c.274_276del | p.92_92del | . | . |
| CTC3 | 17 | 64128806 | AT | A | 0.625 | NM_001199165 | CEP112 | frameshift_deleti  on | exon5 | c.545delA | p.N182fs | . | . |
| CTC3 | 17 | 65052195 | GC | G | 0.9 | NM_000727 | CACNG1 | frameshift_deleti  on | exon4 | c.478delC | p.Q160fs | . | . |
| CTC3 | 17 | 65941650 | TCAGCT  CAGCCC CAGCCC CAAACC CAGCCC | T | 0.381 | NM_182641 | BPTF | nonframeshift_de letion | exon21 | c.6827_6862del | p.2276_2288de l | . | . |
| CTC3 | 17 | 66426189 | CA | C | 0.739 | NM_017983 | WIPI1 | frameshift_deleti  on | exon9 | c.912delT | p.F304fs | . | . |
| CTC3 | 17 | 71394240 | A | AG | 0.547 | NM_001144952 | SDK2 | frameshift_inserti  on | exon24 | c.3287dupC | p.P1096fs | . | . |
| CTC3 | 17 | 71752500 | C | CT | 1 | NM_001278587 | LOC1001343  91 | frameshift_inserti  on | exon6 | c.848_849insT | p.S283fs | . | . |
| CTC3 | 17 | 73317772 | TCTG | T | 0.95 | NM_203506 | GRB2 | nonframeshift_de  letion | exon4 | c.310_312del | p.104_104del | . | . |
| CTC3 | 17 | 73497191 | CAG | C | 0.667 | NM_001142643 | CASKIN2 | frameshift_deleti  on | exon19 | c.3331_3332del | p.L1111fs | . | . |
| CTC3 | 17 | 73497192 | AGG | A | 0.818 | NM_001142643 | CASKIN2 | frameshift_deleti  on | exon19 | c.3330_3331del | p.A1110fs | . | . |
| CTC3 | 17 | 74004092 | AG | A | 0.617 | NM_001988 | EVPL | frameshift_deleti  on | exon22 | c.5193delC | p.P1731fs | . | . |
| CTC3 | 17 | 74020199 | GC | G | 0.261 | NM_001988 | EVPL | frameshift_deleti  on | exon2 | c.100delG | p.A34fs | . | . |
| CTC3 | 17 | 74625213 | CA | C | 0.371 | NM_018414 | ST6GALNAC  1 | frameshift_deleti  on | exon2 | c.711delT | p.P237fs | . | . |
| CTC3 | 17 | 76079140 | CT | C | 0.429 | NM_001142640 | TNRC6C | frameshift_deleti  on | exon12 | c.3362delT | p.L1121fs | . | . |

| CTC3 | 17 | 76887040 | GT | G | 0.35 | NM_001243540 | LOC1006535  15 | frameshift_deleti  on | exon3 | c.1545delA | p.K515fs | . | . |
| --- | --- | --- | --- | --- | --- | --- | --- | --- | --- | --- | --- | --- | --- |
| CTC3 | 17 | 78064145 | A | ACAC | 0.991 | NM_001243342 | CCDC40 | nonframeshift_in  sertion | exon18 | c.3040_3041ins  CAC | p.R1014delinsT  R | . | . |
| CTC3 | 17 | 78338249 | AT | A | 0.5 | NM_001256071 | RNF213 | frameshift_deleti  on | exon42 | c.11768delT | p.I3923fs | . | . |
| CTC3 | 17 | 79172019 | GC | G | 1 | NM_001009811 | AZI1 | frameshift_deleti  on | exon12 | c.1365delG | p.G455fs | . | . |
| CTC3 | 17 | 79634824 | TC | T | 0.385 | NM_199287 | CCDC137 | frameshift_deleti  on | exon2 | c.201delC | p.L67fs | . | . |
| CTC3 | 17 | 80441603 | CA | C | 0.368 | NM_001083608 | NARF | frameshift_deleti  on | exon7 | c.638delA | p.Q213fs | . | . |
| CTC3 | 17 | 80789046 | CT | C | 0.625 | NM_024702 | ZNF750 | frameshift_deleti  on | exon2 | c.1284delA | p.E428fs | . | . |
| CTC3 | 18 | 10857103 | A | AT | 0.321 | NM_022068 | PIEZO2 | frameshift_inserti  on | exon6 | c.598dupA | p.M200fs | . | . |
| CTC3 | 18 | 11753675 | TA | T | 0.571 | NM_182978 | GNAL | frameshift_deleti  on | exon3 | c.499delA | p.I167fs | . | . |
| CTC3 | 18 | 13068158 | TC | T | 1 | NM_032142 | CEP192 | frameshift_deleti  on | exon23 | c.4681delC | p.P1561fs | . | . |
| CTC3 | 18 | 19057245 | A | AC | 0.2 | NM_001142966 | GREB1L | frameshift_inserti  on | exon17 | c.2484_2485ins  C | p.P828fs | . | . |
| CTC3 | 18 | 19080169 | GC | G | 1 | NM_001142966 | GREB1L | frameshift_deleti  on | exon22 | c.3872delC | p.A1291fs | . | . |
| CTC3 | 18 | 19378045 | AC | A | 0.411 | NM_020774 | MIB1 | frameshift_deleti  on | exon8 | c.1094delC | p.T365fs | . | . |
| CTC3 | 18 | 23637652 | GA | G | 0.333 | NM_001007559 | SS18 | frameshift_deleti  on | exon4 | c.285delT | p.P95fs | . | . |
| CTC3 | 18 | 23845147 | TA | T | 0.844 | NM_005640 | TAF4B | frameshift_deleti  on | exon2 | c.358delA | p.K120fs | . | . |
| CTC3 | 18 | 29115301 | CA | C | 1 | NM_001943 | DSG2 | frameshift_deleti  on | exon10 | c.1350delA | p.A450fs | . | . |
| CTC3 | 18 | 29867064 | GT | G | 1 | NM_001242409 | GAREM | frameshift_deleti  on | exon4 | c.1495delA | p.T499fs | . | . |
| CTC3 | 18 | 31324269 | C | CT | 0.667 | NM_030632 | ASXL3 | frameshift_inserti  on | exon12 | c.4458dupT | p.S1486fs | . | . |
| CTC3 | 18 | 34261458 | CAG | C | 0.308 | NM_001281739 | FHOD3 | frameshift_deleti  on | exon12 | c.1371_1372del | p.A457fs | . | . |
| CTC3 | 18 | 34297828 | T | TA | 0.342 | NM_001281739 | FHOD3 | frameshift_inserti  on | exon15 | c.1992dupA | p.V664fs | . | . |
| CTC3 | 18 | 34387862 | GT | G | 0.375 | NM_001271949 | TPGS2 | frameshift_deleti  on | exon3 | c.200delA | p.N67fs | . | . |
| CTC3 | 18 | 34647466 | TA | T | 0.283 | NM_020776 | KIAA1328 | frameshift_deleti  on | exon7 | c.1191delA | p.L397fs | . | . |
| CTC3 | 18 | 40853665 | CA | C | 0.583 | NM_020783 | SYT4 | stopgain | exon2 | c.728delT | p.L243X | . | . |
| CTC3 | 18 | 43833701 | T | TCTG | 1 | NM_001008239 | C18orf25 | nonframeshift_in sertion | exon3 | c.754_755insCT G | p.S252delinsSA | . | . |
| CTC3 | 18 | 43833704 | G | GCTA | 0.969 | NM_001008239 | C18orf25 | nonframeshift_in  sertion | exon3 | c.757_758insCT  A | p.G253delinsA  S | . | . |
| CTC3 | 18 | 44027527 | GA | G | 0.833 | NM_152470 | RNF165 | frameshift_deleti  on | exon4 | c.488delA | p.D163fs | . | . |
| CTC3 | 18 | 45422921 | GT | G | 1 | NM_001003652 | SMAD2 | frameshift_deleti  on | exon2 | c.206delA | p.N69fs | . | . |
| CTC3 | 18 | 47390650 | CCGTGG  CTGGAG TTATTCT | C | 0.13 | NM_001080467 | MYO5B | frameshift_deleti on | exon28 | c.3685_3703del | p.Q1229fs | . | . |
| CTC3 | 18 | 47390737 | TC | T | 0.362 | NM_001080467 | MYO5B | frameshift_deleti  on | exon28 | c.3616delG | p.E1206fs | . | . |
| CTC3 | 18 | 47404185 | GA | G | 0.171 | NM_001080467 | MYO5B | frameshift_deleti  on | exon25 | c.3343delT | p.S1115fs | . | . |
| CTC3 | 18 | 47405425 | T | TGAG | 1 | NM_001080467 | MYO5B | nonframeshift_in  sertion | exon24 | c.3165_3166ins  CTC | p.M1056delins  LM | . | . |
| CTC3 | 18 | 48450503 | AT | A | 0.182 | NM_001168335 | ME2 | frameshift_deleti  on | exon11 | c.1093delT | p.F365fs | . | . |
| CTC3 | 18 | 50923786 | A | AT | 0.286 | NM_005215 | DCC | frameshift_inserti  on | exon18 | c.2798dupT | p.M933fs | . | . |
| CTC3 | 18 | 55024210 | TG | T | 0.5 | NM_015879 | ST8SIA3 | frameshift_deleti  on | exon3 | c.370delG | p.G124fs | . | . |
| CTC3 | 18 | 60036551 | AC | A | 1 | NM_001278268 | TNFRSF11A | frameshift_deleti  on | exon9 | c.1360delC | p.P454fs | . | . |
| CTC3 | 18 | 61602288 | GT | G | 0.781 | NM_005024 | SERPINB10 | frameshift_deleti  on | exon7 | c.1007delT | p.V336fs | . | . |
| CTC3 | 18 | 71987766 | TC | T | 0.389 | NM_001174123 | C18orf63 | frameshift_deleti  on | exon3 | c.154delC | p.Q52fs | . | . |
| CTC3 | 19 | 2115555 | CG | C | 1 | NM_001261826 | AP3D1 | frameshift_deleti  on | exon19 | c.2130delC | p.S710fs | . | . |
| CTC3 | 19 | 4446114 | CA | C | 0.294 | NM_001171091 | UBXN6 | frameshift_deleti  on | exon10 | c.972delT | p.F324fs | . | . |
| CTC3 | 19 | 4852267 | G | GC | 1 | NM_001164189 | PLIN3 | frameshift_inserti  on | exon5 | c.394dupG | p.A132fs | . | . |
| CTC3 | 19 | 5896952 | CT | C | 0.889 | NM_001193375 | NDUFA11 | frameshift_deleti  on | exon2 | c.153delA | p.E51fs | . | . |
| CTC3 | 19 | 7809917 | CA | C | 1 | NM_001144893 | CD209 | frameshift_deleti  on | exon3 | c.401delT | p.M134fs | . | . |
| CTC3 | 19 | 8028495 | TG | T | 0.529 | NM_001419 | ELAVL1 | stopgain | exon6 | c.852delC | p.C284X | . | . |
| CTC3 | 19 | 9003307 | TC | T | 0.333 | NM_024690 | MUC16 | frameshift_deleti on | exon50 | c.40127delG | p.R13376fs | . | . |
| CTC3 | 19 | 9017383 | GC | G | 0.4 | NM_024690 | MUC16 | frameshift_deleti  on | exon26 | c.37940delG | p.G12647fs | . | . |
| CTC3 | 19 | 9067786 | CT | C | 0.505 | NM_024690 | MUC16 | frameshift_deleti  on | exon3 | c.19659delA | p.T6553fs | . | . |
| CTC3 | 19 | 9069942 | AT | A | 0.543 | NM_024690 | MUC16 | frameshift_deleti  on | exon3 | c.17503delA | p.M5835fs | . | . |
| CTC3 | 19 | 12036083 | G | GA | 0.913 | NM_001271848 | ZNF700 | frameshift_inserti  on | exon1 | c.59dupA | p.E20fs | . | . |
| CTC3 | 19 | 12298156 | C | CA | 0.417 | NM_003437 | ZNF136 | frameshift_inserti  on | exon4 | c.963_964insA | p.P321fs | . | . |
| CTC3 | 19 | 12383284 | C | CT | 0.619 | NM_016264 | ZNF44 | frameshift_inserti  on | exon4 | c.1785dupA | p.A596fs | . | . |
| CTC3 | 19 | 12461225 | AG | A | 0.3 | NM_030824 | ZNF442 | frameshift_deleti  on | exon6 | c.1173delC | p.S391fs | . | . |
| CTC3 | 19 | 13054426 | AC | A | 0.667 | NM_004343 | CALR | frameshift_deleti  on | exon8 | c.1037delC | p.T346fs | . | . |
| CTC3 | 19 | 13368319 | TG | T | 0.31 | NM_001127221 | CACNA1A | frameshift_deleti  on | exon28 | c.4437delC | p.P1479fs | . | . |
| CTC3 | 19 | 13445192 | CTG | C | 0.467 | NM_001127221 | CACNA1A | frameshift_deleti  on | exon8 | c.1196_1197del | p.A399fs | . | . |
| CTC3 | 19 | 14020740 | G | GC | 0.955 | NM_017721 | CC2D1A | frameshift_inserti  on | exon2 | c.166dupC | p.Q55fs | . | . |
| CTC3 | 19 | 14031423 | TC | T | 0.38 | NM_017721 | CC2D1A | frameshift_deleti  on | exon13 | c.1411delC | p.P471fs | . | . |
| CTC3 | 19 | 14165452 | AT | A | 0.875 | NM_001145028 | PALM3 | frameshift_deleti  on | exon6 | c.986delA | p.D329fs | . | . |

| CTC3 | 19 | 14513409 | GC | G | 0.452 | NM_001784 | CD97 | frameshift_deleti  on | exon10 | c.906delC | p.G302fs | . | . |
| --- | --- | --- | --- | --- | --- | --- | --- | --- | --- | --- | --- | --- | --- |
| CTC3 | 19 | 14829708 | GCA | G | 0.286 | NM_032433 | ZNF333 | frameshift_deleti  on | exon12 | c.1570_1571del | p.H524fs | . | . |
| CTC3 | 19 | 15228706 | TG | T | 0.521 | NM_006844 | ILVBL | frameshift_deleti  on | exon10 | c.1171delC | p.Q391fs | . | . |
| CTC3 | 19 | 17267720 | AT | A | 0.287 | NM_001130065 | MYO9B | frameshift_deleti  on | exon7 | c.1202delT | p.I401fs | . | . |
| CTC3 | 19 | 17660257 | GA | G | 1 | NM_001098524 | FAM129C | frameshift_deleti  on | exon15 | c.1765delA | p.K589fs | . | . |
| CTC3 | 19 | 17750274 | G | GA | 0.146 | NM_001080421 | UNC13A | frameshift_inserti  on | exon23 | c.2916dupT | p.L973fs | . | . |
| CTC3 | 19 | 17927770 | TC | T | 1 | NM_005543 | INSL3 | frameshift_deleti  on | exon2 | c.288delG | p.Q96fs | . | . |
| CTC3 | 19 | 17950432 | CG | C | 1 | NM_000215 | JAK3 | frameshift_deleti  on | exon10 | c.1294delC | p.R432fs | . | . |
| CTC3 | 19 | 18648411 | CT | C | 1 | NM_012181 | FKBP8 | frameshift_deleti  on | exon6 | c.944delA | p.K315fs | . | . |
| CTC3 | 19 | 19121027 | TCA | T | 0.545 | NM_001017392 | SUGP2 | frameshift_deleti  on | exon5 | c.1973_1974del | p.V658fs | . | . |
| CTC3 | 19 | 19655661 | CG | C | 0.667 | NM_153221 | CILP2 | frameshift_deleti  on | exon8 | c.2308delG | p.G770fs | . | . |
| CTC3 | 19 | 19656228 | A | AG | 0.429 | NM_153221 | CILP2 | frameshift_inserti  on | exon8 | c.2875dupG | p.A958fs | . | . |
| CTC3 | 19 | 20044279 | C | CT | 0.282 | NM_031218 | ZNF93 | frameshift_inserti  on | exon4 | c.516dupT | p.P172fs | . | . |
| CTC3 | 19 | 22364167 | AT | A | 0.333 | NM_001001411 | ZNF676 | frameshift_deleti  on | exon3 | c.351delA | p.K117fs | . | . |
| CTC3 | 19 | 23926622 | AG | A | 0.708 | NM_138286 | ZNF681 | frameshift_deleti  on | exon4 | c.1729delC | p.L577fs | . | . |
| CTC3 | 19 | 23927739 | TTCC | T | 1 | NM_138286 | ZNF681 | nonframeshift_de  letion | exon4 | c.610_612del | p.204_204del | . | . |
| CTC3 | 19 | 23927741 | CCA | C | 1 | NM_138286 | ZNF681 | frameshift_deleti  on | exon4 | c.609_610del | p.C203fs | . | . |
| CTC3 | 19 | 30164835 | CG | C | 0.394 | NM_024310 | PLEKHF1 | frameshift_deleti  on | exon2 | c.90delG | p.A30fs | . | . |
| CTC3 | 19 | 33699868 | CG | C | 1 | NM_019849 | SLC7A10 | frameshift_deleti  on | exon11 | c.1500delC | p.P500fs | . | . |
| CTC3 | 19 | 33703790 | TG | T | 1 | NM_019849 | SLC7A10 | frameshift_deleti  on | exon3 | c.474delC | p.T158fs | . | . |
| CTC3 | 19 | 35175076 | TAAAAA  AAAAAA AAAAAA AAAAAA | T | 1 | NM_001289188 | ZNF302 | frameshift_deleti on | exon5 | c.398_691del | p.*133fs | . | . |
| CTC3 | 19 | 35175076 | TAAAAA  AAAAAA AAAAAA AAAAAA | T | 1 | NM_001289188 | ZNF302 | frameshift_deleti on | exon5 | c.398_691del | p.*133fs | . | . |
| CTC3 | 19 | 35715888 | CG | C | 0.413 | NM_152481 | FAM187B | frameshift_deleti  on | exon2 | c.949delC | p.R317fs | . | . |
| CTC3 | 19 | 35940787 | CCTG | C | 0.2 | NM_005306 | FFAR2 | nonframeshift_de  letion | exon1 | c.172_174del | p.58_58del | . | . |
| CTC3 | 19 | 36303160 | TC | T | 0.209 | NM_021232 | PRODH2 | frameshift_deleti  on | exon4 | c.613delG | p.E205fs | . | . |
| CTC3 | 19 | 36832187 | A | AG | 0.314 | NM_020917 | ZFP14 | frameshift_inserti  on | exon5 | c.540dupC | p.F181fs | . | . |
| CTC3 | 19 | 37117185 | CA | C | 0.727 | NM_001256838 | ZNF382 | frameshift_deleti  on | exon5 | c.384delA | p.S128fs | . | . |
| CTC3 | 19 | 37904688 | AT | A | 0.495 | NM_152484 | ZNF569 | frameshift_deleti  on | exon6 | c.871delA | p.I291fs | . | . |
| CTC3 | 19 | 38378956 | CT | C | 0.605 | NM_031951 | WDR87 | frameshift_deleti  on | exon6 | c.5237delA | p.K1746fs | . | . |
| CTC3 | 19 | 38597212 | TA | T | 1 | NM_015073 | SIPA1L3 | frameshift_deleti  on | exon7 | c.2066delA | p.Y689fs | . | . |
| CTC3 | 19 | 38968394 | CGTGTG  GGCG | C | 0.429 | NM_000540 | RYR1 | nonframeshift_de  letion | exon30 | c.4339_4347del | p.1447_1449de  l | . | . |
| CTC3 | 19 | 39972632 | TG | T | 0.444 | NM_001001563 | TIMM50 | frameshift_deleti  on | exon2 | c.528delG | p.L176fs | . | . |
| CTC3 | 19 | 40541849 | AC | A | 0.421 | NM_001005851 | ZNF780B | frameshift_deleti  on | exon5 | c.916delG | p.V306fs | . | . |
| CTC3 | 19 | 41086682 | G | GT | 0.941 | NM_138392 | SHKBP1 | frameshift_inserti  on | exon9 | c.685dupT | p.V228fs | . | . |
| CTC3 | 19 | 41173874 | TTGC | T | 0.147 | NM_004756 | NUMBL | nonframeshift_de  letion | exon10 | c.1326_1328del | p.442_443del | . | . |
| CTC3 | 19 | 41173892 | CTGCTGT | C | 0.31 | NM_004756 | NUMBL | nonframeshift_de  letion | exon10 | c.1305_1310del | p.435_437del | . | . |
| CTC3 | 19 | 41173904 | TTGC | T | 0.216 | NM_004756 | NUMBL | nonframeshift_de  letion | exon10 | c.1296_1298del | p.432_433del | . | . |
| CTC3 | 19 | 41808592 | TG | T | 0.44 | NM_007040 | HNRNPUL1 | frameshift_deleti  on | exon12 | c.1711delG | p.G571fs | . | . |
| CTC3 | 19 | 42213843 | CA | C | 0.375 | NM_004363 | CEACAM5 | frameshift_deleti  on | exon2 | c.310delA | p.N104fs | . | . |
| CTC3 | 19 | 42224043 | AG | A | 0.6 | NM_004363 | CEACAM5 | frameshift_deleti  on | exon7 | c.1688delG | p.R563fs | . | . |
| CTC3 | 19 | 42265897 | AT | A | 0.278 | NM_002483 | CEACAM6 | frameshift_deleti  on | exon4 | c.725delT | p.I242fs | . | . |
| CTC3 | 19 | 42398613 | GC | G | 0.31 | NM_198977 | ARHGEF1 | frameshift_deleti  on | exon9 | c.736delC | p.P246fs | . | . |
| CTC3 | 19 | 42482352 | CG | C | 0.385 | NM_001256213 | ATP1A3 | frameshift_deleti  on | exon13 | c.1789delC | p.R597fs | . | . |
| CTC3 | 19 | 42583798 | GC | G | 0.204 | NM_022752 | ZNF574 | frameshift_deleti  on | exon2 | c.1041delC | p.S347fs | . | . |
| CTC3 | 19 | 42840383 | GA | G | 0.5 | NM_001271938 | MEGF8 | frameshift_deleti  on | exon6 | c.1130delA | p.E377fs | . | . |
| CTC3 | 19 | 42873061 | CA | C | 0.174 | NM_001410 | MEGF8 | frameshift_deleti  on | exon36 | c.6348delA | p.A2116fs | . | . |
| CTC3 | 19 | 43233990 | A | AT | 1 | NM_021016 | PSG3 | frameshift_inserti  on | exon4 | c.927dupA | p.C310fs | . | . |
| CTC3 | 19 | 43698584 | AT | A | 0.567 | NM_001276495 | PSG4 | stopgain | exon4 | c.871delA | p.I291X | . | . |
| CTC3 | 19 | 44271796 | GT | G | 0.257 | NM_002250 | KCNN4 | frameshift_deleti on | exon8 | c.1182delA | p.K394fs | . | . |
| CTC3 | 19 | 44471085 | CT | C | 0.275 | NM_013359 | ZNF221 | frameshift_deleti  on | exon6 | c.1432delT | p.Y478fs | . | . |
| CTC3 | 19 | 45912489 | CAAG | C | 0.165 | NM_012099 | CD3EAP | nonframeshift_de  letion | exon3 | c.1264_1266del | p.422_422del | . | . |
| CTC3 | 19 | 46215339 | TA | T | 1 | NM_001080469 | FBXO46 | frameshift_deleti  on | exon2 | c.1414delT | p.Y472fs | . | . |
| CTC3 | 19 | 47764063 | TG | T | 0.231 | NM_015603 | CCDC9 | frameshift_deleti  on | exon5 | c.430delG | p.G144fs | . | . |
| CTC3 | 19 | 47935680 | GTCC | G | 0.12 | NM_015063 | SLC8A2 | nonframeshift_de  letion | exon9 | c.2130_2132del | p.710_711del | . | . |

| CTC3 | 19 | 47979929 | GA | G | 0.613 | NM_007059 | KPTN | frameshift_deleti  on | exon11 | c.1041delT | p.L347fs | . | . |
| --- | --- | --- | --- | --- | --- | --- | --- | --- | --- | --- | --- | --- | --- |
| CTC3 | 19 | 47996235 | T | TA | 0.216 | NM_003827 | NAPA | frameshift_inserti  on | exon7 | c.543_544insT | p.I182fs | . | . |
| CTC3 | 19 | 48305612 | TTTGGG  CCTGAG | T | 1 | NM_198479 | TPRX1 | nonframeshift_de  letion | exon2 | c.644_655del | p.215_219del | . | . |
| CTC3 | 19 | 48800608 | CA | C | 0.481 | NM_144577 | CCDC114 | stopgain | exon14 | c.1637delT | p.L546X | . | . |
| CTC3 | 19 | 48846619 | GC | G | 1 | NM_018273 | TMEM143 | frameshift_deleti on | exon5 | c.653delG | p.G218fs | . | . |
| CTC3 | 19 | 49654555 | A | AG | 0.351 | NM_002152 | HRC | stoploss | exon6 | c.2097dupC | p.X700delinsL | . | . |
| CTC3 | 19 | 49657710 | ACAT | A | 0.929 | NM_002152 | HRC | nonframeshift_de letion | exon1 | c.782_784del | p.261_262del | . | . |
| CTC3 | 19 | 49657889 | T | TTCC | 0.947 | NM_002152 | HRC | nonframeshift_in  sertion | exon1 | c.605_606insG  GA | p.E202delinsEE | . | . |
| CTC3 | 19 | 49657892 | C | CTCT | 0.895 | NM_002152 | HRC | nonframeshift_in  sertion | exon1 | c.602_603insA  GA | p.E201delinsEE | . | . |
| CTC3 | 19 | 50193697 | C | CG | 0.909 | NM_001101340 | ADM5 | frameshift_inserti  on | exon2 | c.409_410insG | p.L137fs | . | . |
| CTC3 | 19 | 50314650 | GC | G | 1 | NM_001171937 | FUZ | frameshift_deleti  on | exon4 | c.353delG | p.C118fs | . | . |
| CTC3 | 19 | 50510971 | CCTGGT  CTTCTGA GGGCTA | C | 0.526 | NM_001025778 | VRK3 | nonframeshift_de letion | exon4 | c.231_251del | p.77_84del | . | . |
| CTC3 | 19 | 50733854 | TG | T | 0.37 | NM_024729 | MYH14 | frameshift_deleti  on | exon8 | c.924delG | p.L308fs | . | . |
| CTC3 | 19 | 50926117 | AG | A | 0.65 | NM_001243999 | SPIB | frameshift_deleti  on | exon4 | c.163delG | p.A55fs | . | . |
| CTC3 | 19 | 51128508 | CG | C | 0.769 | NM_032298 | SYT3 | frameshift_deleti  on | exon7 | c.1617delC | p.P539fs | . | . |
| CTC3 | 19 | 51835892 | T | TG | 1 | NM_001163922 | VSIG10L | frameshift_inserti  on | exon10 | c.2576dupC | p.A859fs | . | . |
| CTC3 | 19 | 51955656 | AG | A | 0.633 | NM_014442 | SIGLEC8 | frameshift_deleti  on | exon7 | c.1476delC | p.P492fs | . | . |
| CTC3 | 19 | 52496939 | CT | C | 0.356 | NM_198480 | ZNF615 | frameshift_deleti  on | exon6 | c.1389delA | p.K463fs | . | . |
| CTC3 | 19 | 52660287 | GT | G | 0.333 | NM_001102657 | ZNF836 | frameshift_deleti  on | exon5 | c.648delA | p.K216fs | . | . |
| CTC3 | 19 | 53644938 | G | GC | 0.364 | NM_001172674 | ZNF347 | frameshift_inserti  on | exon5 | c.1145_1146ins  G | p.A382fs | . | . |
| CTC3 | 19 | 53993882 | AAAC | A | 0.159 | NM_001004301 | ZNF813 | nonframeshift_de  letion | exon4 | c.397_399del | p.133_133del | . | . |
| CTC3 | 19 | 54377581 | GT | G | 0.286 | NM_001020818 | MYADM | frameshift_deleti  on | exon2 | c.799delT | p.Y267fs | . | . |
| CTC3 | 19 | 54649674 | C | CA | 1 | NM_014516 | CNOT3 | frameshift_inserti  on | exon9 | c.733dupA | p.P244fs | . | . |
| CTC3 | 19 | 54649675 | AG | A | 1 | NM_014516 | CNOT3 | frameshift_deleti  on | exon9 | c.734delG | p.S245fs | . | . |
| CTC3 | 19 | 54649675 | AGC | A | 1 | NM_014516 | CNOT3 | frameshift_deleti  on | exon9 | c.734_735del | p.S245fs | . | . |
| CTC3 | 19 | 54849785 | GT | G | 0.338 | NM_012276 | LILRA4 | frameshift_deleti  on | exon3 | c.236delA | p.N79fs | . | . |
| CTC3 | 19 | 55526103 | G | GCAGA | 1 | NM_001083899 | GP6 | frameshift_inserti  on | exon8 | c.1209_1210ins  TCTG | p.P404fs | . | . |
| CTC3 | 19 | 56935686 | CT | C | 1 | NM_001159860 | ZNF583 | frameshift_deleti  on | exon5 | c.1660delT | p.S554fs | . | . |
| CTC3 | 19 | 57646792 | T | TA | 0.312 | NM_052882 | ZIM3 | stopgain | exon5 | c.912_913insT | p.K305_P306de  linsX | . | . |
| CTC3 | 19 | 57988666 | A | AGCC | 1 | NM_001024596 | ZNF772 | nonframeshift_in  sertion | exon1 | c.11_12insGGC | p.A4delinsAA | . | . |
| CTC3 | 19 | 58058416 | TA | T | 0.25 | NM_001277090 | ZNF550 | frameshift_deleti  on | exon4 | c.1195delT | p.Y399fs | . | . |
| CTC3 | 19 | 58101845 | CA | C | 0.857 | NM_001010879 | ZIK1 | frameshift_deleti  on | exon4 | c.667delA | p.K223fs | . | . |
| CTC3 | 19 | 58131935 | CA | C | 1 | NM_003435 | ZNF134 | frameshift_deleti  on | exon3 | c.449delA | p.Q150fs | . | . |
| CTC3 | 19 | 58145417 | CAA | C | 0.161 | NM_001265597 | ZNF211 | frameshift_deleti  on | exon2 | c.118_119del | p.K40fs | . | . |
| CTC3 | 19 | 58982188 | T | TC | 0.384 | NM_014347 | ZNF324 | frameshift_inserti  on | exon4 | c.330dupC | p.F110fs | . | . |
| CTC3 | 20 | 126310 | ACC | A | 1 | NM_030931 | DEFB126 | frameshift_deleti  on | exon2 | c.314_315del | p.T105fs | . | . |
| CTC3 | 20 | 472924 | CT | C | 0.571 | NM_177560 | CSNK2A1 | frameshift_deleti  on | exon7 | c.186delA | p.K62fs | . | . |
| CTC3 | 20 | 1908542 | CA | C | 0.25 | NM_001040023 | SIRPA | frameshift_deleti  on | exon6 | c.1224delA | p.T408fs | . | . |
| CTC3 | 20 | 4679885 | TG | T | 1 | NM_000311 | PRNP | stopgain | exon2 | c.20delG | p.W7X | . | . |
| CTC3 | 20 | 5924861 | G | GA | 0.222 | NM_015939 | TRMT6 | frameshift_inserti on | exon4 | c.404dupT | p.F135fs | . | . |
| CTC3 | 20 | 11904095 | C | CT | 0.724 | NM_001282552 | BTBD3 | frameshift_inserti  on | exon4 | c.898dupT | p.T299fs | . | . |
| CTC3 | 20 | 18139728 | GA | G | 0.462 | NM_020536 | CSRP2BP | frameshift_deleti  on | exon4 | c.502delA | p.K168fs | . | . |
| CTC3 | 20 | 18296830 | AG | A | 0.222 | NM_001283005 | ZNF133 | frameshift_deleti  on | exon2 | c.1279delG | p.G427fs | . | . |
| CTC3 | 20 | 21309238 | CA | C | 0.25 | NM_012255 | XRN2 | frameshift_deleti  on | exon4 | c.358delA | p.R120fs | . | . |
| CTC3 | 20 | 21319706 | G | GA | 0.522 | NM_012255 | XRN2 | frameshift_inserti  on | exon14 | c.1259dupA | p.E420fs | . | . |
| CTC3 | 20 | 23965967 | ATGG | A | 0.308 | NM_178311 | GGTLC1 | nonframeshift_de  letion | exon6 | c.561_563del | p.187_188del | . | . |
| CTC3 | 20 | 24950267 | TC | T | 0.364 | NM_020531 | APMAP | frameshift_deleti  on | exon7 | c.742delG | p.E248fs | . | . |
| CTC3 | 20 | 25290099 | GC | G | 1 | NM_001042472 | ABHD12 | frameshift_deleti  on | exon7 | c.731delG | p.G244fs | . | . |
| CTC3 | 20 | 29965181 | C | CT | 0.897 | NM_153289 | DEFB119 | frameshift_inserti  on | exon2 | c.122dupA | p.K41fs | . | . |
| CTC3 | 20 | 31367942 | TC | T | 0.986 | NM_175850 | DNMT3B | frameshift_deleti  on | exon1 | c.22delC | p.P8fs | . | . |
| CTC3 | 20 | 31600615 | GA | G | 0.727 | NM_025227 | BPIFB2 | frameshift_deleti  on | exon4 | c.211delA | p.I71fs | . | . |
| CTC3 | 20 | 31601725 | TTC | T | 0.181 | NM_025227 | BPIFB2 | frameshift_deleti  on | exon5 | c.419_420del | p.F140fs | . | . |
| CTC3 | 20 | 31643272 | TG | T | 0.382 | NM_182658 | BPIFB3 | frameshift_deleti  on | exon1 | c.44delG | p.W15fs | . | . |
| CTC3 | 20 | 33345720 | TTGC | T | 0.152 | NM_001242539 | NCOA6 | nonframeshift_de  letion | exon7 | c.828_830del | p.276_277del | . | . |
| CTC3 | 20 | 33447357 | AG | A | 0.8 | NM_178026 | GGT7 | frameshift_deleti  on | exon7 | c.902delC | p.P301fs | . | . |
| CTC3 | 20 | 34116651 | G | GA | 0.273 | NM_001145350 | C20orf173 | frameshift_inserti  on | exon3 | c.366dupT | p.P123fs | . | . |

| CTC3 | 20 | 34218672 | AG | A | 0.732 | NM_001198863 | CPNE1 | frameshift_deleti  on | exon12 | c.1040delC | p.P347fs | . | . |
| --- | --- | --- | --- | --- | --- | --- | --- | --- | --- | --- | --- | --- | --- |
| CTC3 | 20 | 34241200 | A | AT | 0.5 | NM_001198840 | RBM12 | frameshift_inserti  on | exon2 | c.2044dupA | p.I682fs | . | . |
| CTC3 | 20 | 34328795 | AT | A | 0.889 | NM_001242599 | RBM39 | frameshift_deleti  on | exon2 | c.1delA | p.M1fs | . | . |
| CTC3 | 20 | 34446258 | TC | T | 0.364 | NM_016436 | PHF20 | frameshift_deleti  on | exon5 | c.376delC | p.Q126fs | . | . |
| CTC3 | 20 | 34763553 | GC | G | 1 | NM_001258331 | EPB41L1 | frameshift_deleti  on | exon3 | c.73delC | p.P25fs | . | . |
| CTC3 | 20 | 34843648 | AG | A | 0.857 | NM_001271874 | AAR2 | frameshift_deleti  on | exon4 | c.1137delG | p.E379fs | . | . |
| CTC3 | 20 | 37394104 | TG | T | 0.211 | NM_024855 | ACTR5 | frameshift_deleti  on | exon6 | c.1237delG | p.D413fs | . | . |
| CTC3 | 20 | 39831575 | GA | G | 0.304 | NM_015035 | ZHX3 | frameshift_deleti  on | exon3 | c.1981delT | p.S661fs | . | . |
| CTC3 | 20 | 40757420 | CA | C | 1 | NM_007050 | PTPRT | frameshift_deleti  on | exon19 | c.2820delT | p.S940fs | . | . |
| CTC3 | 20 | 42195736 | TG | T | 0.353 | NM_016276 | SGK2 | frameshift_deleti  on | exon2 | c.246delG | p.L82fs | . | . |
| CTC3 | 20 | 42935679 | TG | T | 0.4 | NM_001080472 | FITM2 | frameshift_deleti  on | exon2 | c.374delC | p.P125fs | . | . |
| CTC3 | 20 | 43355758 | T | TA | 0.278 | NM_003881 | WISP2 | frameshift_inserti  on | exon4 | c.563_564insA | p.L188fs | . | . |
| CTC3 | 20 | 43547577 | GC | G | 0.306 | NM_001124756 | PABPC1L | frameshift_deleti  on | exon4 | c.535delC | p.R179fs | . | . |
| CTC3 | 20 | 43929743 | CT | C | 1 | NM_030590 | MATN4 | frameshift_deleti  on | exon4 | c.764delA | p.Q255fs | . | . |
| CTC3 | 20 | 44485952 | C | CATCT | 1 | NM_005469 | ACOT8 | frameshift_inserti  on | exon1 | c.2_3insAGAT | p.M1fs | . | . |
| CTC3 | 20 | 44485953 | A | ATCTG | 0.909 | NM_005469 | ACOT8 | frameshift_inserti  on | exon1 | c.1_2insCAGA | p.M1fs | . | . |
| CTC3 | 20 | 45812031 | GC | G | 1 | NM_172110 | EYA2 | frameshift_deleti  on | exon13 | c.1292delC | p.A431fs | . | . |
| CTC3 | 20 | 48760073 | GC | G | 0.667 | NM_001162505 | TMEM189 | frameshift_deleti  on | exon2 | c.206delG | p.R69fs | . | . |
| CTC3 | 20 | 52192920 | TC | T | 0.5 | NM_006526 | ZNF217 | frameshift_deleti  on | exon3 | c.2382delG | p.Q794fs | . | . |
| CTC3 | 20 | 52192921 | CT | C | 1 | NM_006526 | ZNF217 | frameshift_deleti  on | exon3 | c.2381delA | p.Q794fs | . | . |
| CTC3 | 20 | 52192922 | TG | T | 1 | NM_006526 | ZNF217 | frameshift_deleti  on | exon3 | c.2380delC | p.Q794fs | . | . |
| CTC3 | 20 | 57569254 | C | CA | 0.277 | NM_198976 | NELFCD | frameshift_inserti  on | exon14 | c.1726dupA | p.I575fs | . | . |
| CTC3 | 20 | 62851225 | AG | A | 0.658 | NM_004535 | MYT1 | frameshift_deleti  on | exon13 | c.2132delG | p.S711fs | . | . |
| CTC3 | 21 | 10942758 | CT | C | 0.344 | NM_199260 | TPTE | frameshift_deleti  on | exon11 | c.568delA | p.R190fs | . | . |
| CTC3 | 21 | 10942924 | CCTT | C | 0.184 | NM_199260 | TPTE | nonframeshift_de  letion | exon10 | c.546_548del | p.182_183del | . | . |
| CTC3 | 21 | 31768591 | CCCA | C | 1 | NM_181599 | KRTAP13-1 | nonframeshift_de  letion | exon1 | c.188_190del | p.63_64del | . | . |
| CTC3 | 21 | 31802667 | AC | A | 0.447 | NM_181600 | KRTAP13-4 | frameshift_deleti  on | exon1 | c.75delC | p.Y25fs | . | . |
| CTC3 | 21 | 31913981 | AG | A | 1 | NM_181612 | KRTAP19-6 | frameshift_deleti  on | exon1 | c.171delC | p.F57fs | . | . |
| CTC3 | 21 | 34924062 | A | AT | 0.5 | NM_032195 | SON | frameshift_inserti  on | exon3 | c.2526dupT | p.D842fs | . | . |
| CTC3 | 21 | 34924672 | CGAGCG  CTCTATG ATGTCC CCTATG | C | 1 | NM_032195 | SON | nonframeshift_de letion | exon3 | c.3136_3162del | p.1046_1054de l | . | . |
| CTC3 | 21 | 34924694 | ATGGC | A | 1 | NM_032195 | SON | frameshift_deleti  on | exon3 | c.3158_3161del | p.M1053fs | . | . |
| CTC3 | 21 | 34924973 | GT | G | 1 | NM_032195 | SON | frameshift_deleti  on | exon3 | c.3437delT | p.V1146fs | . | . |
| CTC3 | 21 | 34948684 | G | GA | 1 | NM_138927 | SON | frameshift_inserti  on | exon12 | c.7236dupA | p.G2412fs | . | . |
| CTC3 | 21 | 34948696 | GA | G | 1 | NM_138927 | SON | frameshift_deleti  on | exon12 | c.7248delA | p.R2416fs | . | . |
| CTC3 | 21 | 38132067 | TA | T | 0.198 | NM_000411 | HLCS | frameshift_deleti  on | exon10 | c.1755delT | p.V585fs | . | . |
| CTC3 | 21 | 38537924 | GT | G | 0.5 | NM_001001894 | TTC3 | frameshift_deleti  on | exon33 | c.3409delT | p.F1137fs | . | . |
| CTC3 | 21 | 40551850 | CT | C | 0.846 | NM_001261824 | PSMG1 | frameshift_deleti  on | exon4 | c.440delA | p.K147fs | . | . |
| CTC3 | 21 | 40571323 | CT | C | 1 | NM_018963 | BRWD1 | frameshift_deleti  on | exon40 | c.5018delA | p.K1673fs | . | . |
| CTC3 | 21 | 40596370 | G | GT | 0.786 | NM_018963 | BRWD1 | frameshift_inserti  on | exon29 | c.3345dupA | p.L1116fs | . | . |
| CTC3 | 21 | 40717088 | TC | T | 1 | NM_004965 | HMGN1 | frameshift_deleti  on | exon5 | c.238delG | p.E80fs | . | . |
| CTC3 | 21 | 40883668 | A | AAGG | 0.912 | NM_001001713 | SH3BGR | nonframeshift_in  sertion | exon6 | c.353_354insA  GG | p.E118delinsEG | . | . |
| CTC3 | 21 | 40883671 | G | GAGA | 1 | NM_001001713 | SH3BGR | nonframeshift_in  sertion | exon6 | c.356_357insA  GA | p.G119delinsG  E | . | . |
| CTC3 | 21 | 40883676 | G | GAAT | 0.945 | NM_001001713 | SH3BGR | stopgain | exon6 | c.361_362insAA  T | p.E121delinsEX | . | . |
| CTC3 | 21 | 40883677 | A | AAGC | 0.946 | NM_001001713 | SH3BGR | nonframeshift_in  sertion | exon6 | c.362_363insA  GC | p.E121delinsEA | . | . |
| CTC3 | 21 | 42778819 | TG | T | 0.229 | NM_002463 | MX2 | frameshift_deleti  on | exon13 | c.1800delG | p.L600fs | . | . |
| CTC3 | 21 | 43771047 | TG | T | 0.379 | NM_005423 | TFF2 | frameshift_deleti  on | exon1 | c.19delC | p.Q7fs | . | . |
| CTC3 | 21 | 43836650 | TC | T | 0.333 | NM_001001895 | UBASH3A | frameshift_deleti  on | exon5 | c.654delC | p.I218fs | . | . |
| CTC3 | 21 | 45535691 | AGAG | A | 0.203 | NM_005049 | PWP2 | nonframeshift_de  letion | exon7 | c.727_729del | p.243_243del | . | . |
| CTC3 | 21 | 46074386 | AG | A | 0.247 | NM_198698 | KRTAP12-4 | frameshift_deleti  on | exon1 | c.145delC | p.L49fs | . | . |
| CTC3 | 21 | 46330658 | GC | G | 0.3 | NM_000211 | ITGB2 | frameshift_deleti  on | exon2 | c.39delG | p.G13fs | . | . |
| CTC3 | 22 | 17688099 | TG | T | 1 | NM_001282229 | CECR1 | frameshift_deleti  on | exon2 | c.43delC | p.H15fs | . | . |
| CTC3 | 22 | 18314824 | GCTC | G | 0.2 | NM_015241 | MICAL3 | nonframeshift_de  letion | exon21 | c.2848_2850del | p.950_950del | . | . |
| CTC3 | 22 | 18385504 | TG | T | 0.985 | NM_001122731 | MICAL3 | frameshift_deleti  on | exon3 | c.481delC | p.Q161fs | . | . |
| CTC3 | 22 | 19495356 | G | GA | 0.364 | NM_001178011 | CDC45 | frameshift_inserti  on | exon11 | c.887dupA | p.E296fs | . | . |
| CTC3 | 22 | 20229631 | TC | T | 1 | NM_023004 | RTN4R | frameshift_deleti  on | exon2 | c.1024delG | p.D342fs | . | . |
| CTC3 | 22 | 20819787 | C | CG | 1 | NM_032775 | KLHL22 | frameshift_inserti  on | exon4 | c.469dupC | p.R157fs | . | . |

| CTC3 | 22 | 22842594 | AT | A | 0.324 | NM_080764 | ZNF280B | frameshift_deleti  on | exon4 | c.1129delA | p.I377fs | . | . |
| --- | --- | --- | --- | --- | --- | --- | --- | --- | --- | --- | --- | --- | --- |
| CTC3 | 22 | 24836803 | TG | T | 0.5 | NM_000675 | ADORA2A | frameshift_deleti  on | exon3 | c.586delG | p.V196fs | . | . |
| CTC3 | 22 | 25294437 | GA | G | 0.229 | NM_001098498 | SGSM1 | frameshift_deleti  on | exon18 | c.2339delA | p.E780fs | . | . |
| CTC3 | 22 | 29107975 | GA | G | 0.217 | NM_007194 | CHEK2 | frameshift_deleti  on | exon6 | c.713delT | p.F238fs | . | . |
| CTC3 | 22 | 29885567 | A | AAAGT  CCCCT GAGAA | 0.974 | NM_021076 | NEFH | nonframeshift_in sertion | exon4 | c.1938_1939ins  AAGTCCCCTG AGAAGGCC | p.A646delinsA KSPEKA | . | . |
| CTC3 | 22 | 29885580 | AAGGCC  AAGTCC CCAGAG | A | 0.7 | NM_021076 | NEFH | nonframeshift_de letion | exon4 | c.1952_1975del | p.651_659del | . | . |
| CTC3 | 22 | 29886603 | GA | G | 1 | NM_021076 | NEFH | frameshift_deleti  on | exon4 | c.2975delA | p.E992fs | . | . |
| CTC3 | 22 | 32587030 | GC | G | 0.893 | NM_006605 | RFPL2 | frameshift_deleti  on | exon2 | c.682delG | p.A228fs | . | . |
| CTC3 | 22 | 32894394 | TG | T | 0.204 | NM_001033024 | FBXO7 | frameshift_deleti  on | exon9 | c.1210delG | p.D404fs | . | . |
| CTC3 | 22 | 36627395 | TCAG | T | 0.444 | NM_030882 | APOL2 | nonframeshift_de  letion | exon4 | c.125_127del | p.42_43del | . | . |
| CTC3 | 22 | 37333551 | CAG | C | 0.75 | NM_000395 | CSF2RB | frameshift_deleti  on | exon14 | c.1702_1703del | p.S568fs | . | . |
| CTC3 | 22 | 37471214 | CG | C | 0.8 | NM_001289000 | TMPRSS6 | frameshift_deleti  on | exon11 | c.1302delC | p.P434fs | . | . |
| CTC3 | 22 | 37603771 | TG | T | 1 | NM_001051 | SSTR3 | frameshift_deleti  on | exon2 | c.71delC | p.P24fs | . | . |
| CTC3 | 22 | 37964409 | CAG | C | 0.207 | NM_152243 | CDC42EP1 | frameshift_deleti  on | exon3 | c.759_760del | p.P253fs | . | . |
| CTC3 | 22 | 37964410 | AG | A | 0.526 | NM_152243 | CDC42EP1 | frameshift_deleti  on | exon3 | c.760delG | p.A254fs | . | . |
| CTC3 | 22 | 38211481 | TG | T | 0.353 | NM_001171690 | GCAT | frameshift_deleti  on | exon6 | c.867delG | p.L289fs | . | . |
| CTC3 | 22 | 39777822 | C | CCAA | 1 | NM_004711 | SYNGR1 | nonframeshift_in  sertion | exon4 | c.605_606insCA  A | p.P202delinsP  N | . | . |
| CTC3 | 22 | 40283697 | A | AT | 0.389 | NM_152512 | ENTHD1 | frameshift_inserti  on | exon2 | c.55dupA | p.I19fs | . | . |
| CTC3 | 22 | 40807614 | GA | G | 0.5 | NM_001282660 | MKL1 | frameshift_deleti  on | exon12 | c.2575delT | p.S859fs | . | . |
| CTC3 | 22 | 41253225 | GT | G | 1 | NM_001204827 | XPNPEP3 | frameshift_deleti  on | exon1 | c.41delT | p.V14fs | . | . |
| CTC3 | 22 | 42018017 | G | GT | 0.229 | NM_001288976 | XRCC6 | frameshift_inserti  on | exon2 | c.10dupT | p.G3fs | . | . |
| CTC3 | 22 | 42086789 | TC | T | 0.861 | NM_001142964 | C22orf46 | frameshift_deleti  on | exon1 | c.159delC | p.V53fs | . | . |
| CTC3 | 22 | 50852989 | GT | G | 0.462 | NM_001242898 | PPP6R2 | frameshift_deleti  on | exon5 | c.424delT | p.F142fs | . | . |
| CTC3 | X | 1712906 | TC | T | 1 | NM_005088 | AKAP17A | frameshift_deleti  on | exon2 | c.552delC | p.I184fs | . | . |
| CTC3 | X | 12828260 | CA | C | 0.68 | NM_001039091 | PRPS2 | frameshift_deleti  on | exon4 | c.535delA | p.K179fs | . | . |
| CTC3 | X | 17165563 | GA | G | 0.4 | NM_001080975 | REPS2 | frameshift_deleti  on | exon18 | c.1940delA | p.E647fs | . | . |
| CTC3 | X | 18924625 | GGCTCC  GTGCCA | G | 0.455 | NM_000292 | PHKA2 | frameshift_deleti  on | exon25 | c.2783_2793del | p.L928fs | . | . |
| CTC3 | X | 19971944 | TTG | T | 0.455 | NM_198279 | CXorf23 | frameshift_deleti  on | exon5 | c.1515_1516del | p.H505fs | . | . |
| CTC3 | X | 24228926 | CA | C | 0.566 | NM_001178086 | ZFX | frameshift_deleti  on | exon6 | c.1165delA | p.K389fs | . | . |
| CTC3 | X | 24382373 | TTGCTGC | T | 1 | NM_001136234 | SUPT20HL1 | nonframeshift_de  letion | exon1 | c.1497_1502del | p.499_501del | . | . |
| CTC3 | X | 39932964 | CG | C | 1 | NM_001123383 | BCOR | frameshift_deleti  on | exon4 | c.1634delC | p.P545fs | . | . |
| CTC3 | X | 40456890 | CT | C | 0.217 | NM_005765 | ATP6AP2 | frameshift_deleti  on | exon5 | c.509delT | p.L170fs | . | . |
| CTC3 | X | 48924868 | TG | T | 1 | NM_001163321 | CCDC120 | frameshift_deleti  on | exon10 | c.1219delG | p.G407fs | . | . |
| CTC3 | X | 57620779 | C | CT | 0.309 | NM_007157 | ZXDB | frameshift_inserti  on | exon1 | c.2299dupT | p.D766fs | . | . |
| CTC3 | X | 62917039 | TG | T | 0.671 | NM_001173479 | ARHGEF9 | frameshift_deleti  on | exon3 | c.367delC | p.H123fs | . | . |
| CTC3 | X | 65819890 | TC | T | 0.194 | NM_001242310 | EDA2R | frameshift_deleti  on | exon5 | c.564delG | p.L188fs | . | . |
| CTC3 | X | 68060393 | TA | T | 0.938 | NM_004429 | EFNB1 | frameshift_deleti  on | exon5 | c.938delA | p.Y313fs | . | . |
| CTC3 | X | 69176907 | GA | G | 0.789 | NM_001005609 | EDA | frameshift_deleti  on | exon2 | c.428delA | p.E143fs | . | . |
| CTC3 | X | 69626861 | AG | A | 0.392 | NM_012310 | KIF4A | frameshift_deleti  on | exon28 | c.3192delG | p.E1064fs | . | . |
| CTC3 | X | 70330818 | A | AT | 0.569 | NM_000206 | IL2RG | frameshift_inserti  on | exon2 | c.197dupA | p.N66fs | . | . |
| CTC3 | X | 70387535 | G | GT | 1 | NM_001166660 | NLGN3 | frameshift_inserti  on | exon5 | c.1469dupT | p.V490fs | . | . |
| CTC3 | X | 84363617 | TG | T | 0.241 | NM_001012980 | SATL1 | frameshift_deleti  on | exon1 | c.357delC | p.P119fs | . | . |
| CTC3 | X | 84526620 | TA | T | 0.989 | NM_021998 | ZNF711 | frameshift_deleti  on | exon9 | c.2073delA | p.L691fs | . | . |
| CTC3 | X | 84569461 | GT | G | 1 | NM_024921 | POF1B | frameshift_deleti  on | exon9 | c.933delA | p.K311fs | . | . |
| CTC3 | X | 89177709 | G | GA | 0.346 | NM_138960 | TGIF2LX | frameshift_inserti  on | exon2 | c.626dupA | p.E209fs | . | . |
| CTC3 | X | 99840248 | GA | G | 0.818 | NM_022144 | TNMD | frameshift_deleti  on | exon2 | c.70delA | p.K24fs | . | . |
| CTC3 | X | 102004834 | TA | T | 0.5 | NM_001142530 | BHLHB9 | frameshift_deleti  on | exon2 | c.912delA | p.V304fs | . | . |
| CTC3 | X | 106841056 | TGAG | T | 0.333 | NM_032428 | FRMPD3 | nonframeshift_de  letion | exon15 | c.2047_2049del | p.683_683del | . | . |
| CTC3 | X | 106846412 | CT | C | 0.471 | NM_032428 | FRMPD3 | frameshift_deleti  on | exon16 | c.5243delT | p.L1748fs | . | . |
| CTC3 | X | 118586956 | GC | G | 0.562 | NM_145305 | SLC25A43 | frameshift_deleti  on | exon5 | c.955delC | p.P319fs | . | . |
| CTC3 | X | 123227969 | AT | A | 0.562 | NM_006603 | STAG2 | frameshift_deleti  on | exon31 | c.3570delT | p.D1190fs | . | . |
| CTC3 | X | 129768980 | A | AT | 0.433 | NM_001281736 | ENOX2 | frameshift_inserti  on | exon11 | c.1396dupA | p.M466fs | . | . |
| CTC3 | X | 131212768 | TC | T | 0.292 | NM_194277 | FRMD7 | frameshift_deleti  on | exon12 | c.1276delG | p.E426fs | . | . |
| CTC3 | X | 134681121 | CA | C | 0.486 | NM_182540 | DDX26B | frameshift_deleti  on | exon6 | c.674delA | p.Q225fs | . | . |
| CTC3 | X | 134685974 | TG | T | 1 | NM_182540 | DDX26B | frameshift_deleti  on | exon8 | c.963delG | p.M321fs | . | . |
| CTC3 | X | 149807440 | GA | G | 0.692 | NM_000252 | MTM1 | frameshift_deleti  on | exon7 | c.470delA | p.E157fs | . | . |

| CTC3 | X | 149905104 | C | CA | 0.296 | NM_003828 | MTMR1 | frameshift_inserti  on | exon10 | c.1095dupA | p.P365fs | . | . |
| --- | --- | --- | --- | --- | --- | --- | --- | --- | --- | --- | --- | --- | --- |
| CTC3 | X | 151092492 | AT | A | 1 | NM_001011548 | MAGEA4 | frameshift_deleti  on | exon3 | c.357delT | p.H119fs | . | . |
| CTC3 | X | 153050278 | G | GC | 1 | NM_001170760 | SRPK3 | frameshift_inserti  on | exon12 | c.1320dupC | p.G440fs | . | . |
| CTC3 | X | 153062006 | AG | A | 0.333 | NM_006280 | SSR4 | frameshift_deleti  on | exon2 | c.186delG | p.Q62fs | . | . |
| CTC3 | X | 154293777 | CA | C | 0.521 | NM_001018025 | MTCP1 | frameshift_deleti  on | exon4 | c.311delT | p.L104fs | . | . |
| CTC4 | 1 | 3679799 | AG | A | 1 | NM_152492 | CCDC27 | frameshift_deleti  on | exon7 | c.1083delG | p.Q361fs | . | . |
| CTC4 | 1 | 3743302 | C | CT | 1 | NM_014704 | CEP104 | frameshift_inserti  on | exon16 | c.2157dupA | p.E720fs | . | . |
| CTC4 | 1 | 5967178 | CT | C | 1 | NM_015102 | NPHP4 | frameshift_deleti  on | exon13 | c.1607delA | p.Q536fs | . | . |
| CTC4 | 1 | 7796522 | CT | C | 0.261 | NM_015215 | CAMTA1 | frameshift_deleti  on | exon13 | c.3186delT | p.T1062fs | . | . |
| CTC4 | 1 | 8418636 | C | CT | 1 | NM_001042682 | RERE | frameshift_inserti  on | exon10 | c.2296_2297ins  A | p.R766fs | . | . |
| CTC4 | 1 | 9667831 | CT | C | 1 | NM_001130924 | TMEM201 | frameshift_deleti  on | exon7 | c.1378delT | p.S460fs | . | . |
| CTC4 | 1 | 10511649 | AT | A | 1 | NM_001302 | CORT | stoploss | exon2 | c.316delT | p.X106K | . | . |
| CTC4 | 1 | 11014130 | CA | C | 0.195 | NM_001170754 | C1orf127 | frameshift_deleti on | exon10 | c.1044delT | p.F348fs | . | . |
| CTC4 | 1 | 11141210 | A | AT | 0.833 | NM_001001998 | EXOSC10 | frameshift_inserti  on | exon11 | c.1365_1366ins  A | p.W456fs | . | . |
| CTC4 | 1 | 11142841 | A | AG | 0.214 | NM_001001998 | EXOSC10 | frameshift_inserti  on | exon10 | c.1183dupC | p.L395fs | . | . |
| CTC4 | 1 | 12907446 | C | CA | 0.52 | NM_001146181 | LOC649330 | stopgain | exon1 | c.696dupT | p.E233_T234del  insX | . | . |
| CTC4 | 1 | 12908024 | TA | T | 0.297 | NM_001146181 | LOC649330 | frameshift_deleti  on | exon1 | c.118delT | p.Y40fs | . | . |
| CTC4 | 1 | 12943022 | CG | C | 1 | NM_001009611 | PRAMEF4 | frameshift_deleti  on | exon2 | c.193delC | p.R65fs | . | . |
| CTC4 | 1 | 13183273 | GT | G | 1 | NM_001136561 | HNRNPCP5 | frameshift_deleti  on | exon2 | c.599delA | p.N200fs | . | . |
| CTC4 | 1 | 14105094 | AGAG | A | 0.357 | NM_001007257 | PRDM2 | nonframeshift_de  letion | exon3 | c.202_204del | p.68_68del | . | . |
| CTC4 | 1 | 14105559 | AC | A | 0.632 | NM_001007257 | PRDM2 | frameshift_deleti  on | exon3 | c.667delC | p.L223fs | . | . |
| CTC4 | 1 | 15850647 | CCAGCC  GCAGCC | C | 0.182 | NM_001229 | CASP9 | nonframeshift_de  letion | exon1 | c.34_48del | p.12_16del | . | . |
| CTC4 | 1 | 20141508 | AC | A | 0.554 | NM_019062 | RNF186 | frameshift_deleti  on | exon1 | c.86delG | p.G29fs | . | . |
| CTC4 | 1 | 21605789 | A | AT | 0.182 | NM_001113347 | ECE1 | frameshift_inserti  on | exon1 | c.138_139insA | p.W47fs | . | . |
| CTC4 | 1 | 21887131 | AC | A | 0.295 | NM_000478 | ALPL | frameshift_deleti  on | exon3 | c.75delC | p.D25fs | . | . |
| CTC4 | 1 | 22074760 | TC | T | 0.3 | NM_001032730 | USP48 | frameshift_deleti  on | exon7 | c.778delG | p.E260fs | . | . |
| CTC4 | 1 | 23759634 | CG | C | 0.983 | NM_001143778 | ASAP3 | frameshift_deleti  on | exon21 | c.2231delC | p.P744fs | . | . |
| CTC4 | 1 | 26608819 | AGGGAC  TGGGGC CGGGAC CGGGAC CGGGAC TGGGGC | A | 1 | NM_001077262 | UBXN11 | nonframeshift_de letion | exon11 | c.1126_1173del | p.376_391del | . | . |
| CTC4 | 1 | 26888007 | T | TTG | 1 | NM_001006665 | RPS6KA1 | frameshift_inserti  on | exon16 | c.1470_1471ins  TG | p.D490fs | . | . |
| CTC4 | 1 | 27720496 | TC | T | 0.657 | NM_005281 | GPR3 | frameshift_deleti  on | exon2 | c.195delC | p.I65fs | . | . |
| CTC4 | 1 | 28861822 | CT | C | 0.357 | NM_001269 | RCC1 | frameshift_deleti  on | exon6 | c.592delT | p.C198fs | . | . |
| CTC4 | 1 | 32050293 | AG | A | 0.516 | NM_001204414 | TINAGL1 | frameshift_deleti  on | exon5 | c.505delG | p.G169fs | . | . |
| CTC4 | 1 | 32846819 | TG | T | 1 | NM_001143890 | BSDC1 | frameshift_deleti  on | exon3 | c.122delC | p.P41fs | . | . |
| CTC4 | 1 | 33145266 | A | AC | 0.286 | NM_001135255 | RBBP4 | frameshift_inserti  on | exon12 | c.1236dupC | p.D412fs | . | . |
| CTC4 | 1 | 35223484 | AT | A | 0.242 | NM_005268 | GJB5 | frameshift_deleti  on | exon2 | c.554delT | p.I185fs | . | . |
| CTC4 | 1 | 35825030 | G | GT | 1 | NM_005095 | ZMYM4 | frameshift_inserti  on | exon3 | c.591dupT | p.S197fs | . | . |
| CTC4 | 1 | 35907902 | CA | C | 1 | NM_024874 | KIAA0319L | frameshift_deleti  on | exon19 | c.2814delT | p.F938fs | . | . |
| CTC4 | 1 | 36557551 | TG | T | 1 | NM_017825 | ADPRHL2 | frameshift_deleti  on | exon4 | c.558delG | p.L186fs | . | . |
| CTC4 | 1 | 37967413 | GT | G | 0.5 | NM_001270875 | MEAF6 | frameshift_deleti  on | exon5 | c.524delA | p.N175fs | . | . |
| CTC4 | 1 | 38034556 | AT | A | 1 | NM_013285 | GNL2 | frameshift_deleti  on | exon13 | c.1763delA | p.N588fs | . | . |
| CTC4 | 1 | 40981003 | TTC | T | 1 | NM_022774 | EXO5 | frameshift_deleti  on | exon3 | c.788_789del | p.F263fs | . | . |
| CTC4 | 1 | 40981007 | CTG | C | 1 | NM_022774 | EXO5 | frameshift_deleti  on | exon3 | c.792_793del | p.S264fs | . | . |
| CTC4 | 1 | 42630343 | GC | G | 0.5 | NM_033553 | GUCA2A | frameshift_deleti  on | exon1 | c.40delG | p.A14fs | . | . |
| CTC4 | 1 | 42654429 | GT | G | 0.613 | NM_001198852 | FOXJ3 | frameshift_deleti  on | exon10 | c.1521delA | p.K507fs | . | . |
| CTC4 | 1 | 43692680 | GA | G | 0.5 | NM_001195831 | WDR65 | frameshift_deleti  on | exon18 | c.2893delA | p.K965fs | . | . |
| CTC4 | 1 | 43903541 | TC | T | 1 | NM_015284 | SZT2 | frameshift_deleti  on | exon45 | c.6306delC | p.I2102fs | . | . |
| CTC4 | 1 | 43917632 | TG | T | 0.367 | NM_001190880 | HYI | frameshift_deleti  on | exon4 | c.478delC | p.Q160fs | . | . |
| CTC4 | 1 | 44477344 | TC | T | 1 | NM_201649 | SLC6A9 | frameshift_deleti  on | exon2 | c.137delG | p.G46fs | . | . |
| CTC4 | 1 | 45806856 | T | TA | 0.971 | NM_025077 | TOE1 | frameshift_inserti  on | exon2 | c.165dupA | p.I55fs | . | . |
| CTC4 | 1 | 45811558 | TG | T | 0.583 | NM_007170 | TESK2 | frameshift_deleti  on | exon10 | c.987delC | p.P329fs | . | . |
| CTC4 | 1 | 46812674 | TG | T | 0.735 | NM_001256127 | NSUN4 | frameshift_deleti  on | exon3 | c.373delG | p.G125fs | . | . |
| CTC4 | 1 | 46867868 | GTGGGA  AAGGTA AGGCCA GCCAAG GCCAGC | G | 0.889 | NM_001441 | FAAH | frameshift_deleti on | exon2 | c.302_309del | p.V101fs | . | . |
| CTC4 | 1 | 51776939 | A | AG | 0.212 | NM_001080494 | TTC39A | frameshift_inserti  on | exon5 | c.412dupC | p.L138fs | . | . |
| CTC4 | 1 | 52930822 | C | CA | 0.5 | NM_001009881 | ZCCHC11 | frameshift_inserti  on | exon16 | c.2988dupT | p.G997fs | . | . |

| CTC4 | 1 | 53676429 | CA | C | 0.178 | NM_000098 | CPT2 | frameshift_deleti  on | exon4 | c.1084delA | p.K362fs | . | . |
| --- | --- | --- | --- | --- | --- | --- | --- | --- | --- | --- | --- | --- | --- |
| CTC4 | 1 | 54562035 | GC | G | 0.981 | NM_153035 | TCEANC2 | frameshift_deleti  on | exon5 | c.517delC | p.R173fs | . | . |
| CTC4 | 1 | 62676732 | GA | G | 0.96 | NM_019079 | L1TD1 | frameshift_deleti  on | exon4 | c.2287delA | p.K763fs | . | . |
| CTC4 | 1 | 62676735 | AT | A | 0.958 | NM_019079 | L1TD1 | frameshift_deleti  on | exon4 | c.2290delT | p.F764fs | . | . |
| CTC4 | 1 | 62739246 | TC | T | 0.468 | NM_181712 | KANK4 | frameshift_deleti  on | exon3 | c.1529delG | p.G510fs | . | . |
| CTC4 | 1 | 65349058 | AC | A | 1 | NM_002227 | JAK1 | frameshift_deleti  on | exon3 | c.106delG | p.V36fs | . | . |
| CTC4 | 1 | 70890058 | AT | A | 0.562 | NM_001190463 | CTH | frameshift_deleti  on | exon4 | c.452delT | p.I151fs | . | . |
| CTC4 | 1 | 76257869 | TA | T | 1 | NM_004582 | RABGGTB | frameshift_deleti  on | exon7 | c.584delA | p.Y195fs | . | . |
| CTC4 | 1 | 78024345 | C | CTAT | 0.537 | NM_012093 | AK5 | nonframeshift_in  sertion | exon14 | c.1601_1602ins  TAT | p.S534delinsSI | . | . |
| CTC4 | 1 | 79392596 | AT | A | 0.473 | NM_022159 | ELTD1 | stopgain | exon8 | c.1057delA | p.I353X | . | . |
| CTC4 | 1 | 85122121 | TGA | T | 0.429 | NM_001166294 | SSX2IP | frameshift_deleti on | exon9 | c.1042_1043del | p.S348fs | . | . |
| CTC4 | 1 | 85135415 | CA | C | 1 | NM_001166294 | SSX2IP | stopgain | exon3 | c.293delT | p.L98X | . | . |
| CTC4 | 1 | 87012878 | CT | C | 0.259 | NM_012128 | CLCA4 | frameshift_deleti on | exon1 | c.77delT | p.L26fs | . | . |
| CTC4 | 1 | 89279291 | CT | C | 0.545 | NM_006256 | PKN2 | frameshift_deleti  on | exon16 | c.2155delT | p.F719fs | . | . |
| CTC4 | 1 | 97235344 | TG | T | 1 | NM_021190 | PTBP2 | frameshift_deleti  on | exon4 | c.202delG | p.G68fs | . | . |
| CTC4 | 1 | 100459135 | T | TC | 0.6 | NM_001271684 | SLC35A3 | frameshift_inserti  on | exon2 | c.26dupC | p.S9fs | . | . |
| CTC4 | 1 | 109955715 | AT | A | 0.357 | NM_001199772 | PSMA5 | frameshift_deleti  on | exon3 | c.91delA | p.I31fs | . | . |
| CTC4 | 1 | 110768748 | CT | C | 0.645 | NM_001039574 | KCNC4 | frameshift_deleti  on | exon3 | c.1768delT | p.F590fs | . | . |
| CTC4 | 1 | 117122269 | GGTCGT  C | G | 0.5 | NM_001007237 | IGSF3 | nonframeshift_de  letion | exon10 | c.3073_3078del | p.1025_1026de  l | . | . |
| CTC4 | 1 | 117122288 | GTCCTC  C | G | 0.353 | NM_001007237 | IGSF3 | nonframeshift_de  letion | exon10 | c.3054_3059del | p.1018_1020de  l | . | . |
| CTC4 | 1 | 117619350 | A | AC | 0.514 | NM_003594 | TTF2 | frameshift_inserti  on | exon7 | c.1478_1479ins  C | p.Q493fs | . | . |
| CTC4 | 1 | 118583369 | CT | C | 0.4 | NM_206996 | SPAG17 | frameshift_deleti  on | exon22 | c.3149delA | p.K1050fs | . | . |
| CTC4 | 1 | 120269480 | TC | T | 0.314 | NM_006623 | PHGDH | frameshift_deleti  on | exon4 | c.364delC | p.P122fs | . | . |
| CTC4 | 1 | 144923728 | GT | G | 1 | NM_001002811 | PDE4DIP | frameshift_deleti  on | exon2 | c.1218delA | p.E406fs | . | . |
| CTC4 | 1 | 149876736 | CA | C | 0.275 | NM_001278719 | SV2A | frameshift_deleti  on | exon3 | c.414delT | p.F138fs | . | . |
| CTC4 | 1 | 149895544 | GA | G | 0.538 | NM_005850 | SF3B4 | frameshift_deleti  on | exon6 | c.1164delT | p.P388fs | . | . |
| CTC4 | 1 | 150297513 | TG | T | 0.5 | NM_004698 | PRPF3 | frameshift_deleti  on | exon2 | c.114delG | p.V38fs | . | . |
| CTC4 | 1 | 150310714 | CT | C | 1 | NM_004698 | PRPF3 | frameshift_deleti  on | exon8 | c.1115delT | p.L372fs | . | . |
| CTC4 | 1 | 151215021 | AC | A | 0.7 | NM_001135636 | PIP5K1A | frameshift_deleti  on | exon12 | c.1499delC | p.T500fs | . | . |
| CTC4 | 1 | 151280139 | C | CT | 0.4 | NM_001198775 | PI4KB | frameshift_inserti  on | exon2 | c.96_97insA | p.A33fs | . | . |
| CTC4 | 1 | 152283064 | TC | T | 1 | NM_002016 | FLG | frameshift_deleti  on | exon3 | c.4297delG | p.E1433fs | . | . |
| CTC4 | 1 | 152733427 | TG | T | 1 | NM_001025231 | KPRP | frameshift_deleti  on | exon2 | c.1364delG | p.C455fs | . | . |
| CTC4 | 1 | 153270530 | T | TA | 0.532 | NM_052891 | PGLYRP3 | stopgain | exon7 | c.927_928insT | p.N310_Y311d  elinsX | . | . |
| CTC4 | 1 | 153751293 | TG | T | 0.263 | NM_024330 | SLC27A3 | frameshift_deleti  on | exon7 | c.1720delG | p.G574fs | . | . |
| CTC4 | 1 | 153907278 | CCTG | C | 0.19 | NM_014856 | DENND4B | nonframeshift_de  letion | exon18 | c.2728_2730del | p.910_910del | . | . |
| CTC4 | 1 | 154842199 | G | GGCTG  CTGCT | 1 | NM_001204087 | KCNN3 | nonframeshift_in  sertion | exon1 | c.241_242insA  GCAGCAGCAG | p.P81delinsQQ  QQQP | . | . |
| CTC4 | 1 | 154842199 | G | GGCTG  CTGCT | 1 | NM_001204087 | KCNN3 | nonframeshift_in  sertion | exon1 | c.241_242insA  GCAGCAGCAG | p.P81delinsQQ  QQP | . | . |
| CTC4 | 1 | 154842216 | C | CTGCT  GCTGC | 0.688 | NM_001204087 | KCNN3 | nonframeshift_in  sertion | exon1 | c.224_225insCC  AGCAGCAGCA | p.Q75delinsHQ  QQQQ | . | . |
| CTC4 | 1 | 154932087 | C | CA | 0.389 | NM_138300 | PYGO2 | frameshift_inserti  on | exon3 | c.388_389insT | p.R130fs | . | . |
| CTC4 | 1 | 154942610 | G | GC | 0.952 | NM_001130040 | SHC1 | frameshift_inserti  on | exon1 | c.392dupG | p.G131fs | . | . |
| CTC4 | 1 | 154999065 | AG | A | 1 | NM_144622 | DCST2 | frameshift_deleti  on | exon9 | c.1468delC | p.L490fs | . | . |
| CTC4 | 1 | 155110701 | CT | C | 0.333 | NM_001287589 | SLC50A1 | frameshift_deleti  on | exon4 | c.284delT | p.L95fs | . | . |
| CTC4 | 1 | 155247407 | CG | C | 1 | NM_020897 | HCN3 | frameshift_deleti  on | exon1 | c.27delG | p.A9fs | . | . |
| CTC4 | 1 | 155263265 | G | GC | 0.385 | NM_000298 | PKLR | frameshift_inserti  on | exon8 | c.1232dupG | p.G411fs | . | . |
| CTC4 | 1 | 155290654 | AG | A | 0.802 | NM_001039517 | RUSC1-AS1 | frameshift_deleti  on | exon2 | c.625delC | p.L209fs | . | . |
| CTC4 | 1 | 155490893 | GT | G | 0.582 | NM_018489 | ASH1L | frameshift_deleti  on | exon2 | c.417delA | p.K139fs | . | . |
| CTC4 | 1 | 155823231 | AG | A | 0.421 | NM_001282856 | GON4L | frameshift_deleti  on | exon2 | c.340delC | p.L114fs | . | . |
| CTC4 | 1 | 155903494 | ACAG | A | 0.129 | NM_014949 | KIAA0907 | nonframeshift_de  letion | exon2 | c.182_184del | p.61_62del | . | . |
| CTC4 | 1 | 156438070 | G | GA | 0.947 | NM_001271629 | MEF2D | frameshift_inserti  on | exon10 | c.1247_1248ins  T | p.H416fs | . | . |
| CTC4 | 1 | 156697301 | AG | A | 0.375 | NM_030980 | ISG20L2 | frameshift_deleti  on | exon1 | c.143delC | p.P48fs | . | . |
| CTC4 | 1 | 156815412 | TG | T | 1 | NM_014215 | INSRR | frameshift_deleti  on | exon10 | c.2172delC | p.P724fs | . | . |
| CTC4 | 1 | 156818778 | GC | G | 1 | NM_014215 | INSRR | frameshift_deleti  on | exon7 | c.1505delG | p.R502fs | . | . |
| CTC4 | 1 | 156828333 | TA | T | 0.231 | NM_014215 | INSRR | frameshift_deleti  on | exon1 | c.80delT | p.V27fs | . | . |
| CTC4 | 1 | 157062779 | GC | G | 0.29 | NM_001004341 | ETV3L | frameshift_deleti  on | exon5 | c.747delG | p.G249fs | . | . |
| CTC4 | 1 | 158576715 | AT | A | 0.782 | NM_001004478 | OR10Z1 | frameshift_deleti  on | exon1 | c.488delT | p.I163fs | . | . |
| CTC4 | 1 | 159175380 | TG | T | 0.485 | NM_001122951 | DARC | frameshift_deleti  on | exon1 | c.158delG | p.C53fs | . | . |
| CTC4 | 1 | 159505610 | AG | A | 0.368 | NM_001004469 | OR10J5 | stopgain | exon1 | c.187delC | p.L63X | . | . |

| CTC4 | 1 | 160090785 | GA | G | 1 | NM_000702 | ATP1A2 | frameshift_deleti  on | exon2 | c.103delA | p.K35fs | . | . |
| --- | --- | --- | --- | --- | --- | --- | --- | --- | --- | --- | --- | --- | --- |
| CTC4 | 1 | 160104404 | AC | A | 0.588 | NM_000702 | ATP1A2 | frameshift_deleti  on | exon14 | c.1959delC | p.N653fs | . | . |
| CTC4 | 1 | 160249896 | CT | C | 0.516 | NM_001193644 | PEX19 | frameshift_deleti  on | exon6 | c.734delA | p.K245fs | . | . |
| CTC4 | 1 | 160523915 | AT | A | 0.833 | NM_001184882 | CD84 | frameshift_deleti  on | exon2 | c.67delA | p.I23fs | . | . |
| CTC4 | 1 | 160969464 | GC | G | 0.222 | NM_016946 | F11R | frameshift_deleti  on | exon7 | c.767delG | p.G256fs | . | . |
| CTC4 | 1 | 161011132 | CT | C | 0.833 | NM_001276373 | USF1 | frameshift_deleti  on | exon7 | c.541delA | p.R181fs | . | . |
| CTC4 | 1 | 164815852 | CT | C | 0.672 | NM_002585 | PBX1 | frameshift_deleti  on | exon9 | c.1233delT | p.P411fs | . | . |
| CTC4 | 1 | 167095906 | GC | G | 0.8 | NM_001080426 | DUSP27 | frameshift_deleti  on | exon5 | c.1539delC | p.S513fs | . | . |
| CTC4 | 1 | 167096724 | CAG | C | 0.435 | NM_001080426 | DUSP27 | frameshift_deleti  on | exon5 | c.2357_2358del | p.Q786fs | . | . |
| CTC4 | 1 | 167381270 | TC | T | 0.762 | NM_001198786 | POU2F1 | frameshift_deleti  on | exon13 | c.1442delC | p.S481fs | . | . |
| CTC4 | 1 | 167792273 | CA | C | 0.239 | NM_001167749 | ADCY10 | frameshift_deleti  on | exon26 | c.3681delT | p.F1227fs | . | . |
| CTC4 | 1 | 173526644 | CA | C | 1 | NM_178527 | SLC9C2 | frameshift_deleti  on | exon10 | c.1049delT | p.L350fs | . | . |
| CTC4 | 1 | 179013151 | GA | G | 0.302 | NM_014864 | FAM20B | frameshift_deleti  on | exon2 | c.170delA | p.D57fs | . | . |
| CTC4 | 1 | 179013343 | GT | G | 0.355 | NM_014864 | FAM20B | frameshift_deleti  on | exon2 | c.362delT | p.V121fs | . | . |
| CTC4 | 1 | 184679532 | A | AT | 0.375 | NM_025191 | EDEM3 | frameshift_inserti  on | exon16 | c.1840dupA | p.I614fs | . | . |
| CTC4 | 1 | 186925308 | GC | G | 0.205 | NM_024420 | PLA2G4A | frameshift_deleti  on | exon14 | c.1412delC | p.A471fs | . | . |
| CTC4 | 1 | 193205436 | GT | G | 0.308 | NM_024529 | CDC73 | frameshift_deleti  on | exon15 | c.1368delT | p.G456fs | . | . |
| CTC4 | 1 | 196274431 | G | GT | 0.571 | NM_001287820 | KCNT2 | frameshift_inserti  on | exon20 | c.2305_2306ins  A | p.A769fs | . | . |
| CTC4 | 1 | 197168626 | CT | C | 1 | NM_194314 | ZBTB41 | frameshift_deleti  on | exon1 | c.977delA | p.K326fs | . | . |
| CTC4 | 1 | 200617644 | GT | G | 0.4 | NM_001031725 | DDX59 | frameshift_deleti  on | exon7 | c.1518delA | p.G506fs | . | . |
| CTC4 | 1 | 201869807 | CT | C | 1 | NM_012134 | LMOD1 | frameshift_deleti  on | exon2 | c.333delA | p.K111fs | . | . |
| CTC4 | 1 | 203186865 | ACT | A | 0.128 | NM_001256125 | CHIT1 | frameshift_deleti  on | exon9 | c.1099_1099del | p.S367fs | . | . |
| CTC4 | 1 | 203465351 | AC | A | 0.63 | NM_014359 | OPTC | frameshift_deleti  on | exon2 | c.219delC | p.D73fs | . | . |
| CTC4 | 1 | 204379197 | C | CG | 0.8 | NM_032833 | PPP1R15B | frameshift_inserti  on | exon1 | c.1342_1343ins  C | p.W448fs | . | . |
| CTC4 | 1 | 204379199 | AT | A | 0.5 | NM_032833 | PPP1R15B | frameshift_deleti  on | exon1 | c.1340delA | p.D447fs | . | . |
| CTC4 | 1 | 204401492 | GC | G | 0.184 | NM_002646 | PIK3C2B | frameshift_deleti  on | exon28 | c.3990delG | p.K1330fs | . | . |
| CTC4 | 1 | 204429777 | CA | C | 0.307 | NM_002646 | PIK3C2B | frameshift_deleti  on | exon7 | c.1322delT | p.L441fs | . | . |
| CTC4 | 1 | 204438642 | AG | A | 0.351 | NM_002646 | PIK3C2B | frameshift_deleti  on | exon3 | c.288delC | p.L96fs | . | . |
| CTC4 | 1 | 206781638 | GC | G | 0.303 | NM_001201478 | EIF2D | frameshift_deleti  on | exon4 | c.372delG | p.L124fs | . | . |
| CTC4 | 1 | 209788629 | GC | G | 1 | NM_001017402 | LAMB3 | frameshift_deleti  on | exon22 | c.3505delG | p.A1169fs | . | . |
| CTC4 | 1 | 210856712 | TG | T | 0.45 | NM_002238 | KCNH1 | frameshift_deleti  on | exon11 | c.2799delC | p.S933fs | . | . |
| CTC4 | 1 | 212220507 | TG | T | 0.385 | NM_001286230 | DTL | stopgain | exon3 | c.167delG | p.W56X | . | . |
| CTC4 | 1 | 212519159 | TG | T | 0.675 | NM_001199756 | PPP2R5A | frameshift_deleti on | exon5 | c.418delG | p.D140fs | . | . |
| CTC4 | 1 | 212969925 | TC | T | 0.382 | NM_001042552 | TATDN3 | stopgain | exon3 | c.167delC | p.S56X | . | . |
| CTC4 | 1 | 214557048 | GCCT | G | 0.139 | NM_005401 | PTPN14 | nonframeshift_de letion | exon13 | c.2147_2149del | p.716_717del | . | . |
| CTC4 | 1 | 220344458 | GA | G | 1 | NM_012414 | RAB3GAP2 | frameshift_deleti  on | exon24 | c.2581delT | p.S861fs | . | . |
| CTC4 | 1 | 224377593 | CG | C | 1 | NM_003676 | DEGS1 | frameshift_deleti  on | exon2 | c.398delG | p.R133fs | . | . |
| CTC4 | 1 | 225539561 | GA | G | 0.269 | NM_001373 | DNAH14 | frameshift_deleti  on | exon70 | c.11231delA | p.E3744fs | . | . |
| CTC4 | 1 | 226175860 | TC | T | 0.255 | NM_152608 | SDE2 | frameshift_deleti  on | exon6 | c.870delG | p.G290fs | . | . |
| CTC4 | 1 | 227182647 | CCT | C | 0.182 | NM_014826 | CDC42BPA | frameshift_deleti  on | exon34 | c.4660_4661del | p.R1554fs | . | . |
| CTC4 | 1 | 227182649 | TC | T | 0.296 | NM_014826 | CDC42BPA | frameshift_deleti  on | exon34 | c.4659delG | p.K1553fs | . | . |
| CTC4 | 1 | 230401100 | CT | C | 1 | NM_004481 | GALNT2 | frameshift_deleti  on | exon14 | c.1428delT | p.A476fs | . | . |
| CTC4 | 1 | 230401101 | TG | T | 1 | NM_004481 | GALNT2 | frameshift_deleti  on | exon14 | c.1429delG | p.G477fs | . | . |
| CTC4 | 1 | 231067533 | TC | T | 0.649 | NM_001122835 | TTC13 | frameshift_deleti  on | exon8 | c.914delG | p.G305fs | . | . |
| CTC4 | 1 | 231321895 | CA | C | 0.176 | NM_001256615 | RP5-  1097F14.3 | frameshift_deleti  on | exon2 | c.75delT | p.P25fs | . | . |
| CTC4 | 1 | 232162199 | GC | G | 1 | NM_001164540 | DISC1 | frameshift_deleti  on | exon10 | c.1961delC | p.A654fs | . | . |
| CTC4 | 1 | 232615418 | G | GA | 0.154 | NM_020808 | SIPA1L2 | frameshift_inserti  on | exon5 | c.2039dupT | p.F680fs | . | . |
| CTC4 | 1 | 232940815 | G | GA | 0.398 | NM_019090 | MAP10 | frameshift_inserti  on | exon1 | c.47dupA | p.E16fs | . | . |
| CTC4 | 1 | 235505951 | CT | C | 0.324 | NM_001037277 | GGPS1 | frameshift_deleti  on | exon4 | c.768delT | p.S256fs | . | . |
| CTC4 | 1 | 235891380 | CT | C | 0.275 | NM_000081 | LYST | frameshift_deleti  on | exon38 | c.9157delA | p.S3053fs | . | . |
| CTC4 | 1 | 236157115 | GC | G | 0.721 | NM_002508 | NID1 | frameshift_deleti  on | exon13 | c.2584delG | p.A862fs | . | . |
| CTC4 | 1 | 236702305 | A | AT | 0.714 | NM_201544 | LGALS8 | frameshift_inserti  on | exon4 | c.261_262insT | p.G87fs | . | . |
| CTC4 | 1 | 236719164 | GA | G | 0.625 | NM_018072 | HEATR1 | frameshift_deleti  on | exon39 | c.5589delT | p.H1863fs | . | . |
| CTC4 | 1 | 236998948 | AT | A | 1 | NM_000254 | MTR | frameshift_deleti  on | exon14 | c.1291delT | p.F431fs | . | . |
| CTC4 | 1 | 237801676 | GT | G | 0.94 | NM_001035 | RYR2 | frameshift_deleti  on | exon45 | c.6813delT | p.G2271fs | . | . |
| CTC4 | 1 | 237870511 | G | GA | 0.674 | NM_001035 | RYR2 | frameshift_inserti  on | exon68 | c.9844dupA | p.L3281fs | . | . |
| CTC4 | 1 | 242030259 | TG | T | 0.365 | NM_003686 | EXO1 | frameshift_deleti  on | exon9 | c.1170delG | p.L390fs | . | . |
| CTC4 | 1 | 242253265 | GT | G | 1 | NM_001195812 | PLD5 | frameshift_deleti  on | exon9 | c.877delA | p.T293fs | . | . |

| CTC4 | 1 | 242253359 | CA | C | 1 | NM_001195812 | PLD5 | frameshift_deleti  on | exon9 | c.783delT | p.L261fs | . | . |
| --- | --- | --- | --- | --- | --- | --- | --- | --- | --- | --- | --- | --- | --- |
| CTC4 | 1 | 245851085 | T | TA | 1 | NM_018012 | KIF26B | frameshift_inserti  on | exon12 | c.4800_4801ins  A | p.P1600fs | . | . |
| CTC4 | 1 | 247012969 | TG | T | 0.917 | NM_015446 | AHCTF1 | frameshift_deleti  on | exon33 | c.6365delC | p.P2122fs | . | . |
| CTC4 | 1 | 247013944 | GT | G | 0.74 | NM_015446 | AHCTF1 | frameshift_deleti  on | exon33 | c.5390delA | p.N1797fs | . | . |
| CTC4 | 1 | 248202484 | GA | G | 0.364 | NM_001004686 | OR2L2 | frameshift_deleti  on | exon1 | c.916delA | p.K306fs | . | . |
| CTC4 | 2 | 1890370 | GC | G | 1 | NM_015025 | MYT1L | frameshift_deleti  on | exon18 | c.2645delG | p.G882fs | . | . |
| CTC4 | 2 | 9645428 | GA | G | 0.423 | NM_003183 | ADAM17 | frameshift_deleti  on | exon12 | c.1410delT | p.F470fs | . | . |
| CTC4 | 2 | 18765868 | CGCT | C | 0.133 | NM_001199103 | NT5C1B-  RDH14 | nonframeshift_de  letion | exon4 | c.638_640del | p.213_214del | . | . |
| CTC4 | 2 | 20508287 | TG | T | 0.415 | NM_001282752 | PUM2 | frameshift_deleti  on | exon5 | c.408delC | p.P136fs | . | . |
| CTC4 | 2 | 20527106 | GA | G | 0.562 | NM_001282790 | PUM2 | frameshift_deleti  on | exon1 | c.15delT | p.F5fs | . | . |
| CTC4 | 2 | 21228331 | TG | T | 1 | NM_000384 | APOB | frameshift_deleti  on | exon26 | c.11408delC | p.P3803fs | . | . |
| CTC4 | 2 | 21231201 | CA | C | 0.484 | NM_000384 | APOB | frameshift_deleti  on | exon26 | c.8538delT | p.F2846fs | . | . |
| CTC4 | 2 | 21235108 | G | GT | 0.4 | NM_000384 | APOB | frameshift_inserti  on | exon26 | c.4631_4632ins  A | p.L1544fs | . | . |
| CTC4 | 2 | 24930593 | CT | C | 0.667 | NM_003743 | NCOA1 | frameshift_deleti  on | exon11 | c.2255delT | p.L752fs | . | . |
| CTC4 | 2 | 25180827 | TA | T | 0.892 | NM_001198559 | DNAJC27 | frameshift_deleti  on | exon4 | c.256delT | p.Y86fs | . | . |
| CTC4 | 2 | 26022273 | C | CT | 0.455 | NM_018263 | ASXL2 | frameshift_inserti  on | exon4 | c.383dupA | p.K128fs | . | . |
| CTC4 | 2 | 26029101 | CA | C | 0.281 | NM_018263 | ASXL2 | stopgain | exon3 | c.248delT | p.L83X | . | . |
| CTC4 | 2 | 26680923 | CT | C | 0.423 | NM_194323 | OTOF | frameshift_deleti on | exon29 | c.3677delA | p.K1226fs | . | . |
| CTC4 | 2 | 27262989 | AC | A | 1 | NM_001083590 | TMEM214 | frameshift_deleti  on | exon14 | c.1580delC | p.T527fs | . | . |
| CTC4 | 2 | 27282086 | AC | A | 1 | NM_001035507 | AGBL5 | frameshift_deleti  on | exon11 | c.1904delC | p.T635fs | . | . |
| CTC4 | 2 | 27352456 | C | CA | 0.307 | NM_032604 | ABHD1 | frameshift_inserti  on | exon5 | c.579_580insA | p.P193fs | . | . |
| CTC4 | 2 | 27851290 | TCA | T | 0.178 | NM_001142683 | CCDC121 | frameshift_deleti  on | exon1 | c.339_340del | p.R113fs | . | . |
| CTC4 | 2 | 27884158 | GT | G | 0.6 | NM_001282729 | SUPT7L | frameshift_deleti  on | exon3 | c.111delA | p.P37fs | . | . |
| CTC4 | 2 | 31600092 | G | GA | 0.5 | NM_000379 | XDH | frameshift_inserti  on | exon14 | c.1253dupT | p.F418fs | . | . |
| CTC4 | 2 | 32626313 | GCAA | G | 0.14 | NM_016252 | BIRC6 | nonframeshift_de  letion | exon7 | c.1118_1120del | p.373_374del | . | . |
| CTC4 | 2 | 37292050 | CCA | C | 0.429 | NM_019024 | HEATR5B | frameshift_deleti  on | exon10 | c.1415_1416del | p.V472fs | . | . |
| CTC4 | 2 | 37334619 | CT | C | 0.982 | NM_001135652 | EIF2AK2 | frameshift_deleti  on | exon13 | c.1407delA | p.K469fs | . | . |
| CTC4 | 2 | 39224476 | CT | C | 0.479 | NM_005633 | SOS1 | frameshift_deleti  on | exon18 | c.2881delA | p.R961fs | . | . |
| CTC4 | 2 | 39414801 | G | GAA | 0.993 | NM_001009565 | CDKL4 | frameshift_inserti  on | exon6 | c.701_702insTT | p.F234fs | . | . |
| CTC4 | 2 | 39414803 | A | AAC | 0.891 | NM_001009565 | CDKL4 | frameshift_inserti  on | exon6 | c.699_700insGT | p.F234fs | . | . |
| CTC4 | 2 | 39414805 | A | AAC | 0.943 | NM_001009565 | CDKL4 | frameshift_inserti  on | exon6 | c.697_698insGT | p.F233fs | . | . |
| CTC4 | 2 | 44209499 | G | GA | 0.609 | NM_133259 | LRPPRC | frameshift_inserti  on | exon2 | c.223dupT | p.S75fs | . | . |
| CTC4 | 2 | 46605201 | CCAG | C | 0.231 | NM_001430 | EPAS1 | nonframeshift_de  letion | exon10 | c.1419_1421del | p.473_474del | . | . |
| CTC4 | 2 | 46607634 | TC | T | 0.214 | NM_001430 | EPAS1 | frameshift_deleti  on | exon12 | c.1824delC | p.F608fs | . | . |
| CTC4 | 2 | 50758380 | G | GT | 0.962 | NM_004801 | NRXN1 | frameshift_inserti  on | exon11 | c.2331dupA | p.L778fs | . | . |
| CTC4 | 2 | 62449932 | GA | G | 0.545 | NM_006577 | B3GNT2 | frameshift_deleti  on | exon2 | c.578delA | p.D193fs | . | . |
| CTC4 | 2 | 63666996 | CA | C | 0.312 | NM_015910 | WDPCP | frameshift_deleti  on | exon7 | c.393delT | p.F131fs | . | . |
| CTC4 | 2 | 64113573 | CA | C | 0.588 | NM_001001521 | UGP2 | frameshift_deleti  on | exon7 | c.940delA | p.K314fs | . | . |
| CTC4 | 2 | 64335375 | CT | C | 1 | NM_020651 | PELI1 | frameshift_deleti  on | exon2 | c.69delA | p.L23fs | . | . |
| CTC4 | 2 | 64796761 | TC | T | 0.609 | NM_001002243 | AFTPH | frameshift_deleti  on | exon5 | c.2220delC | p.F740fs | . | . |
| CTC4 | 2 | 69472460 | CT | C | 0.31 | NM_032208 | ANTXR1 | frameshift_deleti  on | exon18 | c.1539delT | p.T513fs | . | . |
| CTC4 | 2 | 71058837 | AC | A | 0.281 | NM_015717 | CD207 | frameshift_deleti  on | exon5 | c.830delG | p.S277fs | . | . |
| CTC4 | 2 | 71607659 | TC | T | 0.455 | NM_001014972 | ZNF638 | stopgain | exon10 | c.2342delC | p.S781X | . | . |
| CTC4 | 2 | 71650707 | GA | G | 0.684 | NM_001014972 | ZNF638 | frameshift_deleti on | exon22 | c.4064delA | p.E1355fs | . | . |
| CTC4 | 2 | 71740970 | G | GC | 0.357 | NM_001130455 | DYSF | frameshift_inserti  on | exon6 | c.586dupC | p.P195fs | . | . |
| CTC4 | 2 | 71788881 | GC | G | 0.567 | NM_001130976 | DYSF | frameshift_deleti  on | exon22 | c.2121delC | p.S707fs | . | . |
| CTC4 | 2 | 74043357 | AG | A | 0.202 | NM_001080474 | C2orf78 | frameshift_deleti  on | exon3 | c.2008delG | p.A670fs | . | . |
| CTC4 | 2 | 74314999 | GTGCGG  CACCGG GCAGGC | G | 0.154 | NM_001287491 | TET3 | frameshift_deleti on | exon6 | c.2723_2745del | p.V908fs | . | . |
| CTC4 | 2 | 75100493 | AG | A | 0.838 | NM_000189 | HK2 | frameshift_deleti  on | exon5 | c.587delG | p.R196fs | . | . |
| CTC4 | 2 | 85571124 | TC | T | 0.357 | NM_017750 | RETSAT | frameshift_deleti  on | exon9 | c.1530delG | p.G510fs | . | . |
| CTC4 | 2 | 86259451 | CA | C | 0.528 | NM_015425 | POLR1A | frameshift_deleti  on | exon29 | c.4215delT | p.A1405fs | . | . |
| CTC4 | 2 | 88474330 | GA | G | 0.273 | NM_018271 | THNSL2 | frameshift_deleti  on | exon2 | c.397delA | p.K133fs | . | . |
| CTC4 | 2 | 89034131 | C | CT | 0.6 | NM_144563 | RPIA | frameshift_inserti  on | exon5 | c.525_526insT | p.G175fs | . | . |
| CTC4 | 2 | 97217569 | TC | T | 0.429 | NM_212481 | ARID5A | frameshift_deleti  on | exon7 | c.1305delC | p.F435fs | . | . |
| CTC4 | 2 | 99978270 | CA | C | 0.423 | NM_015904 | EIF5B | frameshift_deleti  on | exon4 | c.907delA | p.T303fs | . | . |
| CTC4 | 2 | 103063626 | TG | T | 0.246 | NM_003853 | IL18RAP | frameshift_deleti  on | exon10 | c.1170delG | p.L390fs | . | . |
| CTC4 | 2 | 108476295 | GA | G | 0.368 | NM_182588 | RGPD4 | frameshift_deleti  on | exon12 | c.1753delA | p.K585fs | . | . |

| CTC4 | 2 | 109369922 | CT | C | 0.688 | NM_006267 | RANBP2 | frameshift_deleti  on | exon14 | c.1959delT | p.T653fs | . | . |
| --- | --- | --- | --- | --- | --- | --- | --- | --- | --- | --- | --- | --- | --- |
| CTC4 | 2 | 112636425 | CT | C | 0.308 | NM_022662 | ANAPC1 | frameshift_deleti  on | exon3 | c.336delA | p.A112fs | . | . |
| CTC4 | 2 | 113057522 | G | GA | 0.848 | NM_198581 | ZC3H6 | frameshift_inserti  on | exon2 | c.130dupA | p.E43fs | . | . |
| CTC4 | 2 | 113057525 | A | AG | 0.375 | NM_198581 | ZC3H6 | frameshift_inserti  on | exon2 | c.133dupG | p.K44fs | . | . |
| CTC4 | 2 | 113676364 | GC | G | 1 | NM_173203 | IL37 | frameshift_deleti  on | exon3 | c.453delC | p.S151fs | . | . |
| CTC4 | 2 | 114500446 | C | CT | 0.875 | NM_025181 | SLC35F5 | frameshift_inserti  on | exon7 | c.572dupA | p.K191fs | . | . |
| CTC4 | 2 | 120404559 | C | CT | 0.271 | NM_001271049 | PCDP1 | frameshift_inserti  on | exon22 | c.2252dupT | p.L751fs | . | . |
| CTC4 | 2 | 128046387 | CA | C | 0.263 | NM_000122 | ERCC3 | frameshift_deleti  on | exon7 | c.875delT | p.L292fs | . | . |
| CTC4 | 2 | 128066288 | CA | C | 0.264 | NM_006609 | MAP3K2 | frameshift_deleti  on | exon15 | c.1506delT | p.F502fs | . | . |
| CTC4 | 2 | 128930218 | CA | C | 0.867 | NM_020120 | UGGT1 | frameshift_deleti  on | exon29 | c.3177delA | p.A1059fs | . | . |
| CTC4 | 2 | 128930329 | TA | T | 0.5 | NM_020120 | UGGT1 | frameshift_deleti  on | exon29 | c.3288delA | p.L1096fs | . | . |
| CTC4 | 2 | 131414249 | ATT | A | 0.25 | NM_001277083 | POTEJ | frameshift_deleti  on | exon15 | c.1917_1918del | p.Y639fs | . | . |
| CTC4 | 2 | 149501191 | CT | C | 1 | NM_015630 | EPC2 | frameshift_deleti  on | exon3 | c.315delT | p.P105fs | . | . |
| CTC4 | 2 | 152527708 | CT | C | 0.298 | NM_001164507 | NEB | frameshift_deleti  on | exon38 | c.4334delA | p.K1445fs | . | . |
| CTC4 | 2 | 152548463 | T | TA | 0.658 | NM_001164507 | NEB | frameshift_inserti  on | exon23 | c.2125dupT | p.Y709fs | . | . |
| CTC4 | 2 | 153476066 | G | GCCA | 0.688 | NM_052905 | FMNL2 | nonframeshift_in  sertion | exon15 | c.1671_1672ins  CCA | p.P557delinsPP | . | . |
| CTC4 | 2 | 160604599 | A | AT | 0.581 | NM_001282806 | 7-Mar | frameshift_inserti  on | exon4 | c.631dupT | p.P210fs | . | . |
| CTC4 | 2 | 171376042 | GA | G | 0.418 | NM_001083615 | MYO3B | frameshift_deleti  on | exon29 | c.3487delA | p.K1163fs | . | . |
| CTC4 | 2 | 171813026 | TA | T | 0.354 | NM_001201428 | GORASP2 | frameshift_deleti  on | exon7 | c.577delA | p.I193fs | . | . |
| CTC4 | 2 | 179428602 | CT | C | 0.229 | NM_003319 | TTN | frameshift_deleti  on | exon154 | c.55061delA | p.Q18354fs | . | . |
| CTC4 | 2 | 179468814 | GC | G | 0.466 | NM_003319 | TTN | frameshift_deleti  on | exon110 | c.27404delG | p.G9135fs | . | . |
| CTC4 | 2 | 179641466 | G | GT | 0.753 | NM_003319 | TTN | frameshift_inserti  on | exon27 | c.4986dupA | p.L1663fs | . | . |
| CTC4 | 2 | 185801826 | TA | T | 0.275 | NM_194250 | ZNF804A | frameshift_deleti  on | exon4 | c.1704delA | p.I568fs | . | . |
| CTC4 | 2 | 185802211 | C | CACA | 1 | NM_194250 | ZNF804A | nonframeshift_in  sertion | exon4 | c.2088_2089ins  ACA | p.N696delinsN  T | . | . |
| CTC4 | 2 | 185802212 | A | ACAT | 0.931 | NM_194250 | ZNF804A | nonframeshift_in  sertion | exon4 | c.2089_2090ins  CAT | p.T697delinsTS | . | . |
| CTC4 | 2 | 186660241 | C | CA | 0.192 | NM_173651 | FSIP2 | frameshift_inserti  on | exon16 | c.8645_8646ins  A | p.P2882fs | . | . |
| CTC4 | 2 | 186670303 | CA | C | 0.24 | NM_173651 | FSIP2 | frameshift_deleti  on | exon17 | c.16538delA | p.Q5513fs | . | . |
| CTC4 | 2 | 196722308 | C | CA | 1 | NM_018897 | DNAH7 | frameshift_inserti  on | exon44 | c.8206dupT | p.W2736fs | . | . |
| CTC4 | 2 | 202163985 | TA | T | 1 | NM_001127391 | ALS2CR12 | stopgain | exon12 | c.917delT | p.L306X | . | . |
| CTC4 | 2 | 202357616 | AT | A | 1 | NM_001168221 | ALS2CR11 | frameshift_deleti on | exon15 | c.3447delA | p.K1149fs | . | . |
| CTC4 | 2 | 202358646 | T | TA | 1 | NM_001168221 | ALS2CR11 | frameshift_inserti  on | exon15 | c.2417dupT | p.L806fs | . | . |
| CTC4 | 2 | 210594959 | TG | T | 0.423 | NM_031845 | MAP2 | frameshift_deleti  on | exon12 | c.1255delG | p.G419fs | . | . |
| CTC4 | 2 | 211053710 | GC | G | 1 | NM_001608 | ACADL | frameshift_deleti  on | exon11 | c.1267delG | p.A423fs | . | . |
| CTC4 | 2 | 216248122 | AC | A | 1 | NM_002026 | FN1 | frameshift_deleti  on | exon30 | c.4705delG | p.V1569fs | . | . |
| CTC4 | 2 | 218675007 | CA | C | 0.857 | NM_022648 | TNS1 | frameshift_deleti  on | exon29 | c.4755delT | p.P1585fs | . | . |
| CTC4 | 2 | 220023000 | TA | T | 0.571 | NM_024782 | NHEJ1 | frameshift_deleti  on | exon2 | c.84delT | p.F28fs | . | . |
| CTC4 | 2 | 225368412 | CT | C | 0.444 | NM_001257197 | CUL3 | frameshift_deleti  on | exon8 | c.1135delA | p.S379fs | . | . |
| CTC4 | 2 | 227659846 | TG | T | 0.5 | NM_005544 | IRS1 | frameshift_deleti  on | exon1 | c.3608delC | p.P1203fs | . | . |
| CTC4 | 2 | 228846534 | C | CT | 1 | NM_030623 | SPHKAP | frameshift_inserti  on | exon11 | c.4914dupA | p.V1639fs | . | . |
| CTC4 | 2 | 231867471 | TG | T | 0.319 | NM_139073 | SPATA3 | frameshift_deleti  on | exon3 | c.547delG | p.G183fs | . | . |
| CTC4 | 2 | 233001268 | G | GA | 1 | NM_001257281 | DIS3L2 | frameshift_inserti  on | exon8 | c.790dupA | p.R263fs | . | . |
| CTC4 | 2 | 233323834 | TG | T | 1 | NM_001631 | ALPI | frameshift_deleti  on | exon11 | c.1566delG | p.L522fs | . | . |
| CTC4 | 2 | 234183410 | GC | G | 0.493 | NM_198890 | ATG16L1 | frameshift_deleti  on | exon5 | c.452delC | p.A151fs | . | . |
| CTC4 | 2 | 238283148 | TC | T | 0.368 | NM_057166 | COL6A3 | frameshift_deleti  on | exon5 | c.1764delG | p.G588fs | . | . |
| CTC4 | 2 | 242163147 | AAG | A | 0.305 | NM_001001891 | ANO7 | frameshift_deleti  on | exon23 | c.2646_2647del | p.K882fs | . | . |
| CTC4 | 3 | 10085519 | GA | G | 0.982 | NM_001018115 | FANCD2 | frameshift_deleti  on | exon14 | c.1106delA | p.E369fs | . | . |
| CTC4 | 3 | 10085523 | AC | A | 0.3 | NM_001018115 | FANCD2 | frameshift_deleti  on | exon14 | c.1110delC | p.N370fs | . | . |
| CTC4 | 3 | 10452342 | CA | C | 0.235 | NM_001001331 | ATP2B2 | frameshift_deleti  on | exon3 | c.356delT | p.L119fs | . | . |
| CTC4 | 3 | 12983217 | GC | G | 0.419 | NM_001134382 | IQSEC1 | frameshift_deleti  on | exon2 | c.171delG | p.G57fs | . | . |
| CTC4 | 3 | 13522748 | TAC | T | 0.322 | NM_024827 | HDAC11 | frameshift_deleti  on | exon2 | c.6_7del | p.L2fs | . | . |
| CTC4 | 3 | 13611983 | AC | A | 0.85 | NM_001004019 | FBLN2 | frameshift_deleti  on | exon2 | c.129delC | p.N43fs | . | . |
| CTC4 | 3 | 13611985 | TG | T | 1 | NM_001004019 | FBLN2 | frameshift_deleti  on | exon2 | c.131delG | p.C44fs | . | . |
| CTC4 | 3 | 13612534 | GGC | G | 0.6 | NM_001004019 | FBLN2 | frameshift_deleti  on | exon2 | c.680_681del | p.G227fs | . | . |
| CTC4 | 3 | 13612535 | GC | G | 1 | NM_001004019 | FBLN2 | frameshift_deleti  on | exon2 | c.681delC | p.G227fs | . | . |
| CTC4 | 3 | 19575415 | TC | T | 0.327 | NM_144633 | KCNH8 | stopgain | exon16 | c.3149delC | p.S1050X | . | . |
| CTC4 | 3 | 25761604 | CT | C | 0.356 | NM_001145293 | NGLY1 | frameshift_deleti on | exon11 | c.1635delA | p.K545fs | . | . |
| CTC4 | 3 | 29977627 | C | CA | 0.603 | NM_001003792 | RBMS3 | frameshift_inserti  on | exon10 | c.937dupA | p.A312fs | . | . |
| CTC4 | 3 | 32737150 | TTG | T | 1 | NM_001256742 | CNOT10 | frameshift_deleti  on | exon1 | c.34_35del | p.C12fs | . | . |

| CTC4 | 3 | 32737151 | TG | T | 1 | NM_001256742 | CNOT10 | frameshift_deleti  on | exon1 | c.35delG | p.C12fs | . | . |
| --- | --- | --- | --- | --- | --- | --- | --- | --- | --- | --- | --- | --- | --- |
| CTC4 | 3 | 33614648 | CT | C | 1 | NM_001207044 | CLASP2 | frameshift_deleti  on | exon20 | c.2016delA | p.E672fs | . | . |
| CTC4 | 3 | 36937233 | CA | C | 0.452 | NM_014831 | TRANK1 | frameshift_deleti  on | exon4 | c.317delT | p.L106fs | . | . |
| CTC4 | 3 | 38175447 | G | GA | 0.2 | NM_001130410 | ACAA1 | frameshift_inserti  on | exon3 | c.318dupT | p.L107fs | . | . |
| CTC4 | 3 | 40503526 | G | GCTGC  TGCTG | 1 | NM_001034996 | RPL14 | nonframeshift_in  sertion | exon6 | c.451_452insCT  GCTGCTGCTG | p.A151delinsA  AAAAP | . | . |
| CTC4 | 3 | 40503529 | G | GCTGC  TGCTG | 1 | NM_001034996 | RPL14 | nonframeshift_in  sertion | exon6 | c.454_455insCT  GCTGCTGCTC | p.A152delinsA  AAAP | . | . |
| CTC4 | 3 | 40503529 | G | GCTGC  TGCTG | 1 | NM_001034996 | RPL14 | nonframeshift_in  sertion | exon6 | c.454_455insCT  GCTGCTGCTC | p.A152delinsA  AAAPP | . | . |
| CTC4 | 3 | 40503539 | C | CTCCT  GCTGC | 1 | NM_001034996 | RPL14 | nonframeshift_in  sertion | exon6 | c.464_465insTC  CTGCTGCTGCT | p.A155delinsA  PAAAV | . | . |
| CTC4 | 3 | 42251576 | A | AAGGA  GG | 0.477 | NM_001265609 | TRAK1 | nonframeshift_in  sertion | exon13 | c.1840_1841ins  AGGAGG | p.T614delinsKE  A | . | . |
| CTC4 | 3 | 42251577 | C | CGGAG  GA | 0.92 | NM_001265609 | TRAK1 | nonframeshift_in  sertion | exon13 | c.1841_1842ins  GGAGGA | p.T614delinsTE  E | . | . |
| CTC4 | 3 | 42251577 | C | CGGA | 0.92 | NM_001265609 | TRAK1 | nonframeshift_in  sertion | exon13 | c.1841_1842ins  GGA | p.T614delinsTE | . | . |
| CTC4 | 3 | 42251599 | G | GGAGG  AA | 0.652 | NM_001265609 | TRAK1 | nonframeshift_in  sertion | exon13 | c.1863_1864ins  GAGGAA | p.E621delinsEE  E | . | . |
| CTC4 | 3 | 42251602 | G | GGAGG  AC | 0.653 | NM_001265609 | TRAK1 | nonframeshift_in  sertion | exon13 | c.1866_1867ins  GAGGAC | p.E622delinsEE  D | . | . |
| CTC4 | 3 | 42251607 | A | AGGAG  GG | 0.6 | NM_001265609 | TRAK1 | nonframeshift_in  sertion | exon13 | c.1871_1872ins  GGAGGG | p.E624delinsEE  G | . | . |
| CTC4 | 3 | 42251608 | G | GGAGG  AA | 0.663 | NM_001265609 | TRAK1 | nonframeshift_in  sertion | exon13 | c.1872_1873ins  GAGGAA | p.E624delinsEE  E | . | . |
| CTC4 | 3 | 42251668 | GT | G | 0.261 | NM_001265609 | TRAK1 | frameshift_deleti  on | exon13 | c.1933delT | p.F645fs | . | . |
| CTC4 | 3 | 44684781 | GT | G | 0.345 | NM_006991 | ZNF197 | frameshift_deleti  on | exon6 | c.2160delT | p.S720fs | . | . |
| CTC4 | 3 | 48460434 | GT | G | 0.344 | NM_001130082 | PLXNB1 | frameshift_deleti  on | exon13 | c.2846delA | p.D949fs | . | . |
| CTC4 | 3 | 48628926 | TGG | T | 0.889 | NM_000094 | COL7A1 | frameshift_deleti  on | exon12 | c.1605_1606del | p.T535fs | . | . |
| CTC4 | 3 | 49456562 | GC | G | 0.317 | NM_001164710 | AMT | frameshift_deleti  on | exon6 | c.586delG | p.A196fs | . | . |
| CTC4 | 3 | 49939818 | TG | T | 0.92 | NM_001244937 | MST1R | frameshift_deleti  on | exon1 | c.1224delC | p.P408fs | . | . |
| CTC4 | 3 | 49940067 | AG | A | 0.273 | NM_001244937 | MST1R | frameshift_deleti  on | exon1 | c.975delC | p.H325fs | . | . |
| CTC4 | 3 | 49940224 | AG | A | 0.277 | NM_001244937 | MST1R | frameshift_deleti  on | exon1 | c.818delC | p.P273fs | . | . |
| CTC4 | 3 | 50155887 | TGAGA | T | 0.8 | NM_005778 | RBM5 | frameshift_deleti  on | exon25 | c.2447_2623del | p.*816fs | . | . |
| CTC4 | 3 | 50332198 | AG | A | 0.333 | NM_001200029 | HYAL3 | frameshift_deleti  on | exon2 | c.835delC | p.L279fs | . | . |
| CTC4 | 3 | 50645995 | GT | G | 0.429 | NM_145071 | CISH | frameshift_deleti  on | exon2 | c.49delA | p.T17fs | . | . |
| CTC4 | 3 | 51456205 | TTA | T | 1 | NM_001171904 | VPRBP | frameshift_deleti  on | exon14 | c.3198_3199del | p.Y1066fs | . | . |
| CTC4 | 3 | 51624505 | AGAG | A | 0.182 | NM_015106 | RAD54L2 | nonframeshift_de  letion | exon2 | c.70_72del | p.24_24del | . | . |
| CTC4 | 3 | 51696958 | TC | T | 0.392 | NM_015106 | RAD54L2 | frameshift_deleti  on | exon22 | c.3927delC | p.F1309fs | . | . |
| CTC4 | 3 | 52257395 | GC | G | 1 | NM_017442 | TLR9 | frameshift_deleti  on | exon2 | c.936delG | p.V312fs | . | . |
| CTC4 | 3 | 52777396 | CG | C | 0.333 | NM_001193533 | NEK4 | frameshift_deleti  on | exon11 | c.1708delC | p.R570fs | . | . |
| CTC4 | 3 | 53785920 | TG | T | 1 | NM_001128839 | CACNA1D | frameshift_deleti  on | exon28 | c.3662delG | p.C1221fs | . | . |
| CTC4 | 3 | 56650051 | A | ACTT | 1 | NM_001012506 | CCDC66 | nonframeshift_in  sertion | exon13 | c.1711_1712ins  CTT | p.T571delinsTS | . | . |
| CTC4 | 3 | 57303630 | AG | A | 0.365 | NM_012096 | APPL1 | frameshift_deleti  on | exon22 | c.2046delG | p.E682fs | . | . |
| CTC4 | 3 | 57386140 | GA | G | 0.244 | NM_178504 | DNAH12 | frameshift_deleti  on | exon45 | c.7042delT | p.S2348fs | . | . |
| CTC4 | 3 | 57616464 | CT | C | 0.26 | NM_152678 | DENND6A | frameshift_deleti  on | exon17 | c.1494delA | p.K498fs | . | . |
| CTC4 | 3 | 68788233 | GT | G | 0.963 | NM_001005527 | FAM19A4 | frameshift_deleti  on | exon5 | c.403delA | p.T135fs | . | . |
| CTC4 | 3 | 73111297 | AC | A | 1 | NM_018029 | EBLN2 | frameshift_deleti  on | exon1 | c.66delC | p.D22fs | . | . |
| CTC4 | 3 | 97614902 | TA | T | 0.762 | NM_153605 | CRYBG3 | frameshift_deleti  on | exon12 | c.7675delA | p.I2559fs | . | . |
| CTC4 | 3 | 100119725 | CA | C | 1 | NM_014820 | TOMM70A | frameshift_deleti  on | exon1 | c.68delT | p.V23fs | . | . |
| CTC4 | 3 | 101378782 | TG | T | 1 | NM_014415 | ZBTB11 | frameshift_deleti  on | exon6 | c.1890delC | p.S630fs | . | . |
| CTC4 | 3 | 101572147 | A | AT | 0.556 | NM_031419 | NFKBIZ | frameshift_inserti  on | exon5 | c.777_778insT | p.G259fs | . | . |
| CTC4 | 3 | 105397377 | G | GT | 1 | NM_170662 | CBLB | frameshift_inserti  on | exon17 | c.2466dupA | p.P823fs | . | . |
| CTC4 | 3 | 110791295 | TGC | T | 0.226 | NM_001243288 | PVRL3 | frameshift_deleti  on | exon1 | c.30_31del | p.V10fs | . | . |
| CTC4 | 3 | 110853012 | GT | G | 0.448 | NM_015480 | PVRL3 | frameshift_deleti  on | exon6 | c.1601delT | p.V534fs | . | . |
| CTC4 | 3 | 111793206 | CA | C | 1 | NM_001042575 | TMPRSS7 | frameshift_deleti  on | exon12 | c.1353delA | p.A451fs | . | . |
| CTC4 | 3 | 111793227 | A | AT | 0.237 | NM_001042575 | TMPRSS7 | frameshift_inserti  on | exon12 | c.1374dupT | p.D458fs | . | . |
| CTC4 | 3 | 112253058 | C | CA | 0.833 | NM_001278712 | ATG3 | frameshift_inserti  on | exon11 | c.920dupT | p.L307fs | . | . |
| CTC4 | 3 | 113077716 | CA | C | 0.638 | NM_001164496 | WDR52 | frameshift_deleti  on | exon22 | c.2940delT | p.F980fs | . | . |
| CTC4 | 3 | 113176064 | GA | G | 0.4 | NM_144718 | SPICE1 | frameshift_deleti  on | exon13 | c.1575delT | p.F525fs | . | . |
| CTC4 | 3 | 113379398 | AG | A | 0.7 | NM_001009899 | KIAA2018 | frameshift_deleti  on | exon7 | c.1130delC | p.P377fs | . | . |
| CTC4 | 3 | 115395068 | AG | A | 1 | NM_002045 | GAP43 | frameshift_deleti  on | exon2 | c.240delG | p.K80fs | . | . |
| CTC4 | 3 | 118935109 | CT | C | 0.5 | NM_003778 | B4GALT4 | frameshift_deleti  on | exon7 | c.879delA | p.K293fs | . | . |
| CTC4 | 3 | 119246631 | AC | A | 0.394 | NM_005191 | CD80 | frameshift_deleti  on | exon6 | c.853delG | p.V285fs | . | . |
| CTC4 | 3 | 121340931 | GA | G | 0.985 | NM_016298 | FBXO40 | frameshift_deleti  on | exon3 | c.656delA | p.E219fs | . | . |
| CTC4 | 3 | 121351293 | T | TCTCA  GGCTC | 0.471 | NM_005335 | HCLS1 | nonframeshift_in  sertion | exon12 | c.1125_1126ins  TCCGAGCCTG | p.N376delinsSE  PEN | . | . |

| CTC4 | 3 | 121351315 | G | GGGCT  CAGGC | 1 | NM_005335 | HCLS1 | nonframeshift_in  sertion | exon12 | c.1103_1104ins  TGAGCCTGAG | p.P368delinsPE  PEP | . | . |
| --- | --- | --- | --- | --- | --- | --- | --- | --- | --- | --- | --- | --- | --- |
| CTC4 | 3 | 124376600 | GA | G | 0.733 | NM_007064 | KALRN | frameshift_deleti  on | exon8 | c.893delA | p.E298fs | . | . |
| CTC4 | 3 | 124646705 | A | AAAG | 1 | NM_033049 | MUC13 | nonframeshift_in  sertion | exon2 | c.184_185insCT  T | p.F62delinsSF | . | . |
| CTC4 | 3 | 124689601 | GT | G | 1 | NM_020733 | HEG1 | frameshift_deleti  on | exon17 | c.4040delA | p.Y1347fs | . | . |
| CTC4 | 3 | 128292233 | GA | G | 1 | NM_007354 | C3orf27 | frameshift_deleti  on | exon3 | c.339delT | p.P113fs | . | . |
| CTC4 | 3 | 128292318 | ATC | A | 0.241 | NM_007354 | C3orf27 | frameshift_deleti  on | exon3 | c.253_254del | p.D85fs | . | . |
| CTC4 | 3 | 130380577 | TG | T | 0.571 | NM_001102608 | COL6A6 | frameshift_deleti  on | exon34 | c.5928delG | p.M1976fs | . | . |
| CTC4 | 3 | 133302920 | GT | G | 1 | NM_001134422 | CDV3 | frameshift_deleti  on | exon3 | c.393delT | p.G131fs | . | . |
| CTC4 | 3 | 133368243 | AT | A | 0.381 | NM_007027 | TOPBP1 | frameshift_deleti  on | exon10 | c.1487delA | p.N496fs | . | . |
| CTC4 | 3 | 134094090 | G | GC | 0.222 | NM_001278683 | AMOTL2 | frameshift_inserti  on | exon1 | c.52dupG | p.A18fs | . | . |
| CTC4 | 3 | 137796424 | GCC | G | 0.235 | NM_173543 | DZIP1L | frameshift_deleti  on | exon11 | c.1337_1338del | p.R446fs | . | . |
| CTC4 | 3 | 137811278 | AT | A | 0.857 | NM_173543 | DZIP1L | frameshift_deleti  on | exon5 | c.816delA | p.K272fs | . | . |
| CTC4 | 3 | 137811326 | CT | C | 1 | NM_173543 | DZIP1L | frameshift_deleti  on | exon5 | c.768delA | p.K256fs | . | . |
| CTC4 | 3 | 141162451 | CT | C | 1 | NM_001080412 | ZBTB38 | frameshift_deleti  on | exon8 | c.1222delT | p.F408fs | . | . |
| CTC4 | 3 | 141163193 | TG | T | 0.737 | NM_001080412 | ZBTB38 | frameshift_deleti  on | exon8 | c.1964delG | p.W655fs | . | . |
| CTC4 | 3 | 142521107 | GAT | G | 0.216 | NM_003304 | TRPC1 | frameshift_deleti  on | exon9 | c.1577_1578del | p.D526fs | . | . |
| CTC4 | 3 | 150845637 | AT | A | 0.36 | NM_053002 | MED12L | frameshift_deleti  on | exon4 | c.423delT | p.D141fs | . | . |
| CTC4 | 3 | 151012069 | GA | G | 0.341 | NM_023915 | GPR87 | frameshift_deleti  on | exon3 | c.964delT | p.S322fs | . | . |
| CTC4 | 3 | 154855982 | TG | T | 0.171 | NM_000902 | MME | frameshift_deleti  on | exon9 | c.813delG | p.L271fs | . | . |
| CTC4 | 3 | 159605670 | GA | G | 1 | NM_001197108 | SCHIP1 | frameshift_deleti  on | exon4 | c.475delA | p.K159fs | . | . |
| CTC4 | 3 | 159710816 | AT | A | 0.364 | NM_000882 | IL12A | frameshift_deleti  on | exon3 | c.283delT | p.F95fs | . | . |
| CTC4 | 3 | 160093633 | TC | T | 0.692 | NM_020800 | IFT80 | frameshift_deleti  on | exon5 | c.405delG | p.K135fs | . | . |
| CTC4 | 3 | 160137161 | CA | C | 1 | NM_005496 | SMC4 | frameshift_deleti  on | exon11 | c.1688delA | p.Q563fs | . | . |
| CTC4 | 3 | 167249012 | CA | C | 1 | NM_178824 | WDR49 | frameshift_deleti  on | exon9 | c.1052delT | p.M351fs | . | . |
| CTC4 | 3 | 168833382 | TG | T | 0.267 | NM_001105078 | MECOM | frameshift_deleti  on | exon7 | c.1713delC | p.S571fs | . | . |
| CTC4 | 3 | 172013153 | GT | G | 0.183 | NM_001135095 | FNDC3B | frameshift_deleti  on | exon8 | c.851delT | p.V284fs | . | . |
| CTC4 | 3 | 172835260 | CT | C | 0.263 | NM_031955 | SPATA16 | frameshift_deleti  on | exon2 | c.261delA | p.E87fs | . | . |
| CTC4 | 3 | 182683471 | G | GT | 0.52 | NM_020640 | DCUN1D1 | frameshift_inserti  on | exon2 | c.73dupA | p.T25fs | . | . |
| CTC4 | 3 | 182737989 | A | AT | 0.836 | NM_020166 | MCCC1 | frameshift_inserti  on | exon17 | c.1905dupA | p.Y636fs | . | . |
| CTC4 | 3 | 188477982 | GT | G | 0.933 | NM_001167672 | LPP | frameshift_deleti  on | exon7 | c.882delT | p.C294fs | . | . |
| CTC4 | 3 | 188584048 | AC | A | 1 | NM_001167672 | LPP | frameshift_deleti  on | exon8 | c.1031delC | p.T344fs | . | . |
| CTC4 | 3 | 190986221 | C | CA | 0.5 | NM_198152 | UTS2B | frameshift_inserti  on | exon9 | c.343dupT | p.W115fs | . | . |
| CTC4 | 3 | 194167785 | AG | A | 0.512 | NM_024524 | ATP13A3 | frameshift_deleti  on | exon13 | c.1367delC | p.P456fs | . | . |
| CTC4 | 3 | 196304670 | CA | C | 0.396 | NM_001105573 | FBXO45 | frameshift_deleti  on | exon2 | c.666delA | p.P222fs | . | . |
| CTC4 | 3 | 196674314 | AC | A | 0.344 | NM_025163 | PIGZ | frameshift_deleti  on | exon3 | c.1453delG | p.V485fs | . | . |
| CTC4 | 4 | 154774 | CA | C | 0.769 | NM_001039127 | ZNF718 | frameshift_deleti  on | exon5 | c.300delA | p.P100fs | . | . |
| CTC4 | 4 | 786376 | TC | T | 0.895 | NM_006651 | CPLX1 | frameshift_deleti  on | exon3 | c.51delG | p.G17fs | . | . |
| CTC4 | 4 | 1087327 | G | GCTGC  CCAGG CTGGA | 1 | NM_001193318 | RNF212 | nonframeshift_in sertion | exon4 | c.721_722insG  GCTGGCTCCA GCCTGGGCAG | p.S241delinsW LAPAWAA | . | . |
| CTC4 | 4 | 3117968 | C | CT | 0.185 | NM_002111 | HTT | frameshift_inserti  on | exon8 | c.1047dupT | p.P349fs | . | . |
| CTC4 | 4 | 7398032 | G | GA | 0.241 | NM_020777 | SORCS2 | frameshift_inserti  on | exon2 | c.499dupA | p.T166fs | . | . |
| CTC4 | 4 | 20706422 | TA | T | 0.583 | NM_001130727 | PACRGL | frameshift_deleti  on | exon3 | c.193delA | p.K65fs | . | . |
| CTC4 | 4 | 22444275 | TG | T | 1 | NM_145290 | GPR125 | frameshift_deleti  on | exon7 | c.917delC | p.A306fs | . | . |
| CTC4 | 4 | 39310557 | CT | C | 1 | NM_001204747 | RFC1 | frameshift_deleti  on | exon13 | c.1583delA | p.K528fs | . | . |
| CTC4 | 4 | 39436113 | TTG | T | 0.204 | NM_175737 | KLB | frameshift_deleti  on | exon2 | c.1110_1111del | p.F370fs | . | . |
| CTC4 | 4 | 42414969 | TG | T | 0.8 | NM_001105529 | ATP8A1 | frameshift_deleti  on | exon36 | c.3413delC | p.A1138fs | . | . |
| CTC4 | 4 | 46053640 | A | AG | 0.211 | NM_173536 | GABRG1 | frameshift_inserti  on | exon8 | c.931dupC | p.L311fs | . | . |
| CTC4 | 4 | 46736984 | T | TG | 0.377 | NM_130902 | COX7B2 | frameshift_inserti  on | exon3 | c.225_226insC | p.K76fs | . | . |
| CTC4 | 4 | 47645073 | TAA | T | 0.143 | NM_001278586 | CORIN | frameshift_deleti  on | exon14 | c.2045_2046del | p.F682fs | . | . |
| CTC4 | 4 | 48994106 | AG | A | 0.15 | NM_001286791 | CWH43 | frameshift_deleti  on | exon4 | c.430delG | p.D144fs | . | . |
| CTC4 | 4 | 49005767 | CG | C | 0.24 | NM_001286791 | CWH43 | frameshift_deleti  on | exon7 | c.738delG | p.A246fs | . | . |
| CTC4 | 4 | 55981093 | TG | T | 1 | NM_002253 | KDR | frameshift_deleti  on | exon5 | c.605delC | p.A202fs | . | . |
| CTC4 | 4 | 56435966 | TC | T | 0.4 | NM_152401 | PDCL2 | frameshift_deleti  on | exon4 | c.280delG | p.E94fs | . | . |
| CTC4 | 4 | 57215880 | AAC | A | 0.227 | NM_001286670 | AASDH | frameshift_deleti  on | exon8 | c.580_581del | p.V194fs | . | . |
| CTC4 | 4 | 57269493 | AG | A | 0.47 | NM_002703 | PPAT | frameshift_deleti  on | exon4 | c.476delC | p.P159fs | . | . |
| CTC4 | 4 | 57797605 | T | TCC | 1 | NM_001193508 | REST | frameshift_inserti  on | exon4 | c.2581_2582ins  CC | p.S861fs | . | . |
| CTC4 | 4 | 70146457 | CTT | C | 0.234 | NM_001207004 | UGT2B28 | frameshift_deleti  on | exon1 | c.240_241del | p.S80fs | . | . |
| CTC4 | 4 | 71500035 | TG | T | 1 | NM_031889 | ENAM | frameshift_deleti  on | exon6 | c.222delG | p.L74fs | . | . |

| CTC4 | 4 | 73957363 | GA | G | 0.847 | NM_198889 | ANKRD17 | frameshift_deleti  on | exon28 | c.5228delT | p.V1743fs | . | . |
| --- | --- | --- | --- | --- | --- | --- | --- | --- | --- | --- | --- | --- | --- |
| CTC4 | 4 | 76489718 | A | AT | 1 | NM_001206981 | C4orf26 | frameshift_inserti  on | exon3 | c.507dupT | p.D169fs | . | . |
| CTC4 | 4 | 77305791 | AC | A | 1 | NM_001042784 | CCDC158 | frameshift_deleti  on | exon4 | c.315delG | p.K105fs | . | . |
| CTC4 | 4 | 77305792 | CT | C | 1 | NM_001042784 | CCDC158 | frameshift_deleti  on | exon4 | c.314delA | p.K105fs | . | . |
| CTC4 | 4 | 77692020 | GC | G | 0.17 | NM_020859 | SHROOM3 | frameshift_deleti  on | exon10 | c.5592delC | p.G1864fs | . | . |
| CTC4 | 4 | 79394674 | G | GA | 1 | NM_025074 | FRAS1 | frameshift_inserti  on | exon53 | c.7606dupA | p.V2535fs | . | . |
| CTC4 | 4 | 79792160 | GCAGCA  C | G | 0.176 | NM_017593 | BMP2K | nonframeshift_de  letion | exon11 | c.1456_1461del | p.486_487del | . | . |
| CTC4 | 4 | 79792163 | GCAC | G | 0.277 | NM_017593 | BMP2K | nonframeshift_de  letion | exon11 | c.1459_1461del | p.487_487del | . | . |
| CTC4 | 4 | 79800081 | TA | T | 0.593 | NM_017593 | BMP2K | nonframeshift_de  letion | exon14 | c.1988delA | p.X663X | . | . |
| CTC4 | 4 | 81967246 | AG | A | 1 | NM_001201 | BMP3 | frameshift_deleti  on | exon2 | c.672delG | p.K224fs | . | . |
| CTC4 | 4 | 85704136 | GC | G | 0.364 | NM_014991 | WDFY3 | frameshift_deleti  on | exon25 | c.4153delG | p.A1385fs | . | . |
| CTC4 | 4 | 86898771 | GC | G | 0.179 | NM_031305 | ARHGAP24 | frameshift_deleti  on | exon5 | c.577delC | p.Q193fs | . | . |
| CTC4 | 4 | 87968324 | TC | T | 1 | NM_005935 | AFF1 | frameshift_deleti  on | exon3 | c.617delC | p.S206fs | . | . |
| CTC4 | 4 | 88035692 | T | TC | 1 | NM_005935 | AFF1 | frameshift_inserti  on | exon11 | c.1687dupC | p.P562fs | . | . |
| CTC4 | 4 | 88238331 | AG | A | 1 | NM_001136230 | HSD17B13 | frameshift_deleti  on | exon2 | c.254delC | p.A85fs | . | . |
| CTC4 | 4 | 89326750 | G | GT | 0.621 | NM_001165136 | HERC6 | frameshift_inserti  on | exon10 | c.1260dupT | p.S420fs | . | . |
| CTC4 | 4 | 89653221 | TG | T | 1 | NM_001265580 | FAM13A | frameshift_deleti  on | exon14 | c.1712delC | p.P571fs | . | . |
| CTC4 | 4 | 91701613 | CT | C | 1 | NM_207491 | CCSER1 | stoploss | exon8 | c.2032delT | p.X678E | . | . |
| CTC4 | 4 | 95147391 | A | AG | 0.294 | NM_001128429 | SMARCAD1 | frameshift_inserti on | exon3 | c.312_313insG | p.P104fs | . | . |
| CTC4 | 4 | 100504616 | GA | G | 0.231 | NM_000253 | MTTP | frameshift_deleti  on | exon4 | c.336delA | p.G112fs | . | . |
| CTC4 | 4 | 103592500 | A | AC | 0.5 | NM_005908 | MANBA | frameshift_inserti  on | exon9 | c.1167_1168ins  G | p.W390fs | . | . |
| CTC4 | 4 | 104031999 | C | CT | 0.952 | NM_001286734 | CENPE | frameshift_inserti  on | exon45 | c.7346dupA | p.K2449fs | . | . |
| CTC4 | 4 | 104031999 | C | CTT | 0.952 | NM_001286734 | CENPE | frameshift_inserti  on | exon45 | c.7346_7347ins  AA | p.K2449fs | . | . |
| CTC4 | 4 | 104032007 | T | TTTA | 0.385 | NM_001286734 | CENPE | stopgain | exon45 | c.7338_7339ins  TAA | p.K2447delinsX | . | . |
| CTC4 | 4 | 104115550 | GA | G | 0.286 | NM_001286734 | CENPE | frameshift_deleti  on | exon7 | c.607delT | p.S203fs | . | . |
| CTC4 | 4 | 126370704 | GT | G | 0.593 | NM_024582 | FAT4 | frameshift_deleti  on | exon9 | c.8534delT | p.V2845fs | . | . |
| CTC4 | 4 | 134072877 | C | CA | 1 | NM_020815 | PCDH10 | frameshift_inserti  on | exon1 | c.1582_1583ins  A | p.L528fs | . | . |
| CTC4 | 4 | 139980290 | CT | C | 0.431 | NM_001276457 | ELF2 | frameshift_deleti  on | exon6 | c.1361delA | p.K454fs | . | . |
| CTC4 | 4 | 144618597 | AG | A | 0.353 | NM_001168235 | FREM3 | frameshift_deleti  on | exon1 | c.3231delC | p.S1077fs | . | . |
| CTC4 | 4 | 144621493 | CG | C | 0.611 | NM_001168235 | FREM3 | frameshift_deleti  on | exon1 | c.335delC | p.P112fs | . | . |
| CTC4 | 4 | 146031276 | AT | A | 0.391 | NM_001040876 | ABCE1 | frameshift_deleti  on | exon6 | c.428delT | p.I143fs | . | . |
| CTC4 | 4 | 146806929 | GC | G | 1 | NM_178835 | ZNF827 | frameshift_deleti  on | exon4 | c.1647delG | p.K549fs | . | . |
| CTC4 | 4 | 151520246 | CT | C | 0.481 | NM_001199282 | LRBA | frameshift_deleti  on | exon38 | c.5958delA | p.E1986fs | . | . |
| CTC4 | 4 | 154525003 | A | AG | 0.584 | NM_001131007 | KIAA0922 | frameshift_inserti  on | exon25 | c.2840dupG | p.R947fs | . | . |
| CTC4 | 4 | 155157143 | GA | G | 1 | NM_017639 | DCHS2 | frameshift_deleti  on | exon25 | c.7295delT | p.F2432fs | . | . |
| CTC4 | 4 | 158074052 | GA | G | 0.514 | NM_000824 | GLRB | frameshift_deleti  on | exon9 | c.1088delA | p.E363fs | . | . |
| CTC4 | 4 | 169197250 | TA | T | 0.273 | NM_017631 | DDX60 | frameshift_deleti  on | exon15 | c.2060delT | p.L687fs | . | . |
| CTC4 | 4 | 169433276 | TA | T | 0.474 | NM_001166108 | PALLD | frameshift_deleti  on | exon2 | c.622delA | p.K208fs | . | . |
| CTC4 | 4 | 185339344 | C | CT | 0.444 | NM_002199 | IRF2 | frameshift_inserti  on | exon5 | c.387dupA | p.E130fs | . | . |
| CTC4 | 4 | 186998199 | C | CT | 0.625 | NM_003265 | TLR3 | frameshift_inserti  on | exon2 | c.427dupT | p.P142fs | . | . |
| CTC4 | 4 | 187084645 | GGA | G | 0.273 | NM_001006655 | FAM149A | frameshift_deleti  on | exon9 | c.902_903del | p.G301fs | . | . |
| CTC4 | 4 | 187084647 | AC | A | 0.312 | NM_001006655 | FAM149A | frameshift_deleti  on | exon9 | c.904delC | p.R302fs | . | . |
| CTC4 | 4 | 188924203 | T | TA | 0.887 | NM_174900 | ZFP42 | frameshift_inserti  on | exon4 | c.242_243insA | p.F81fs | . | . |
| CTC4 | 4 | 189012770 | AG | A | 0.889 | NM_173553 | TRIML2 | frameshift_deleti  on | exon7 | c.920delC | p.P307fs | . | . |
| CTC4 | 5 | 7892857 | CA | C | 0.248 | NM_002454 | MTRR | frameshift_deleti  on | exon11 | c.1389delA | p.P463fs | . | . |
| CTC4 | 5 | 9629656 | AT | A | 0.429 | NM_019599 | TAS2R1 | frameshift_deleti  on | exon1 | c.488delA | p.N163fs | . | . |
| CTC4 | 5 | 10286508 | TC | T | 0.303 | NM_138809 | CMBL | frameshift_deleti  on | exon4 | c.423delG | p.L141fs | . | . |
| CTC4 | 5 | 14474129 | TG | T | 0.471 | NM_007118 | TRIO | frameshift_deleti  on | exon40 | c.6007delG | p.G2003fs | . | . |
| CTC4 | 5 | 15936969 | A | AT | 1 | NM_001278317 | FBXL7 | frameshift_inserti  on | exon4 | c.1009_1010ins  T | p.R337fs | . | . |
| CTC4 | 5 | 21783507 | GC | G | 0.412 | NM_004061 | CDH12 | frameshift_deleti  on | exon11 | c.1352delG | p.S451fs | . | . |
| CTC4 | 5 | 32074136 | AG | A | 0.269 | NM_178140 | PDZD2 | frameshift_deleti  on | exon17 | c.2925delG | p.E975fs | . | . |
| CTC4 | 5 | 32074405 | GC | G | 0.762 | NM_178140 | PDZD2 | frameshift_deleti  on | exon17 | c.3194delC | p.A1065fs | . | . |
| CTC4 | 5 | 33938153 | GC | G | 0.284 | NM_016568 | RXFP3 | frameshift_deleti  on | exon1 | c.1309delC | p.P437fs | . | . |
| CTC4 | 5 | 35965546 | AG | A | 0.25 | NM_152404 | UGT3A1 | frameshift_deleti  on | exon4 | c.784delC | p.L262fs | . | . |
| CTC4 | 5 | 37148303 | GT | G | 1 | NM_023073 | C5orf42 | frameshift_deleti  on | exon42 | c.8278delA | p.T2760fs | . | . |
| CTC4 | 5 | 37183357 | TA | T | 0.471 | NM_023073 | C5orf42 | stopgain | exon26 | c.4925delT | p.L1642X | . | . |
| CTC4 | 5 | 38418229 | AC | A | 0.333 | NM_182798 | EGFLAM | frameshift_deleti on | exon7 | c.855delC | p.Y285fs | . | . |
| CTC4 | 5 | 38884000 | GT | G | 0.197 | NM_001168355 | OSMR | frameshift_deleti  on | exon5 | c.491delT | p.V164fs | . | . |

| CTC4 | 5 | 41061770 | GA | G | 0.26 | NM_173489 | MROH2B | frameshift_deleti  on | exon6 | c.516delT | p.F172fs | . | . |
| --- | --- | --- | --- | --- | --- | --- | --- | --- | --- | --- | --- | --- | --- |
| CTC4 | 5 | 43295951 | G | GT | 0.205 | NM_002130 | HMGCS1 | frameshift_inserti  on | exon5 | c.807dupA | p.L270fs | . | . |
| CTC4 | 5 | 52214623 | CA | C | 0.925 | NM_181501 | ITGA1 | frameshift_deleti  on | exon16 | c.2051delA | p.Q684fs | . | . |
| CTC4 | 5 | 54579191 | CT | C | 0.571 | NM_019030 | DHX29 | frameshift_deleti  on | exon11 | c.1804delA | p.S602fs | . | . |
| CTC4 | 5 | 56161270 | TA | T | 0.4 | NM_005921 | MAP3K1 | frameshift_deleti  on | exon5 | c.1140delA | p.L380fs | . | . |
| CTC4 | 5 | 57755760 | GT | G | 0.455 | NM_001252226 | PLK2 | frameshift_deleti  on | exon1 | c.26delA | p.Y9fs | . | . |
| CTC4 | 5 | 58120899 | GC | G | 0.93 | NM_138453 | RAB3C | frameshift_deleti  on | exon4 | c.407delC | p.A136fs | . | . |
| CTC4 | 5 | 66492426 | GA | G | 0.83 | NM_005582 | CD180 | frameshift_deleti  on | exon1 | c.43delT | p.S15fs | . | . |
| CTC4 | 5 | 67522771 | CG | C | 0.667 | NM_181523 | PIK3R1 | frameshift_deleti  on | exon2 | c.269delG | p.R90fs | . | . |
| CTC4 | 5 | 71495320 | GA | G | 1 | NM_005909 | MAP1B | frameshift_deleti  on | exon5 | c.6139delA | p.K2047fs | . | . |
| CTC4 | 5 | 74101014 | TG | T | 1 | NM_015566 | FAM169A | frameshift_deleti  on | exon7 | c.765delC | p.T255fs | . | . |
| CTC4 | 5 | 74325169 | GA | G | 0.815 | NM_016591 | GCNT4 | frameshift_deleti  on | exon1 | c.693delT | p.F231fs | . | . |
| CTC4 | 5 | 75927714 | GT | G | 0.571 | NM_001285461 | IQGAP2 | frameshift_deleti  on | exon4 | c.303delT | p.C101fs | . | . |
| CTC4 | 5 | 75927721 | A | AG | 0.667 | NM_001285461 | IQGAP2 | frameshift_inserti  on | exon4 | c.310dupG | p.E103fs | . | . |
| CTC4 | 5 | 76028275 | TA | T | 0.374 | NM_001992 | F2R | frameshift_deleti  on | exon2 | c.226delA | p.K76fs | . | . |
| CTC4 | 5 | 78329210 | AC | A | 0.667 | NM_013391 | DMGDH | frameshift_deleti  on | exon8 | c.1214delG | p.G405fs | . | . |
| CTC4 | 5 | 80408474 | TA | T | 0.854 | NM_006909 | RASGRF2 | frameshift_deleti  on | exon14 | c.1885delA | p.K629fs | . | . |
| CTC4 | 5 | 82835208 | CT | C | 0.655 | NM_001164097 | VCAN | frameshift_deleti  on | exon7 | c.3426delT | p.P1142fs | . | . |
| CTC4 | 5 | 90086734 | T | TC | 0.267 | NM_032119 | GPR98 | frameshift_inserti  on | exon70 | c.14089dupC | p.F4696fs | . | . |
| CTC4 | 5 | 94030629 | C | CA | 1 | NM_032290 | ANKRD32 | frameshift_inserti  on | exon21 | c.2790dupA | p.T930fs | . | . |
| CTC4 | 5 | 94030708 | CT | C | 0.667 | NM_032290 | ANKRD32 | frameshift_deleti  on | exon21 | c.2869delT | p.F957fs | . | . |
| CTC4 | 5 | 94922397 | AT | A | 0.297 | NM_198150 | ARSK | frameshift_deleti  on | exon5 | c.832delT | p.F278fs | . | . |
| CTC4 | 5 | 109963560 | G | GT | 0.911 | NM_001039763 | TMEM232 | frameshift_inserti  on | exon6 | c.522dupA | p.R175fs | . | . |
| CTC4 | 5 | 110440432 | CT | C | 0.519 | NM_139281 | WDR36 | frameshift_deleti  on | exon9 | c.1112delT | p.L371fs | . | . |
| CTC4 | 5 | 121495926 | T | TA | 0.2 | NM_001195535 | LOC1005058  41 | frameshift_inserti  on | exon1 | c.2_3insA | p.M1fs | . | . |
| CTC4 | 5 | 125805455 | GA | G | 1 | NM_001146319 | GRAMD3 | frameshift_deleti  on | exon3 | c.336delA | p.R112fs | . | . |
| CTC4 | 5 | 129100626 | GT | G | 0.992 | NM_001257308 | KIAA1024L | frameshift_deleti  on | exon3 | c.444delT | p.G148fs | . | . |
| CTC4 | 5 | 131298253 | AT | A | 0.4 | NM_001205251 | ACSL6 | frameshift_deleti  on | exon17 | c.1531delA | p.I511fs | . | . |
| CTC4 | 5 | 132262844 | GT | G | 0.429 | NM_014423 | AFF4 | frameshift_deleti  on | exon5 | c.1018delA | p.T340fs | . | . |
| CTC4 | 5 | 134782283 | GA | G | 0.519 | NM_130848 | C5orf20 | frameshift_deleti  on | exon1 | c.515delT | p.F172fs | . | . |
| CTC4 | 5 | 137753235 | GC | G | 0.909 | NM_016604 | KDM3B | frameshift_deleti  on | exon13 | c.3372delC | p.C1124fs | . | . |
| CTC4 | 5 | 137801683 | ACAG | A | 0.18 | NM_001964 | EGR1 | nonframeshift_de  letion | exon1 | c.234_236del | p.78_79del | . | . |
| CTC4 | 5 | 137802940 | AC | A | 1 | NM_001964 | EGR1 | frameshift_deleti  on | exon2 | c.803delC | p.T268fs | . | . |
| CTC4 | 5 | 138266229 | AAG | A | 0.862 | NM_001903 | CTNNA1 | frameshift_deleti  on | exon15 | c.2079_2080del | p.E693fs | . | . |
| CTC4 | 5 | 140346667 | GA | G | 1 | NM_018899 | PCDHAC2 | frameshift_deleti  on | exon1 | c.317delA | p.D106fs | . | . |
| CTC4 | 5 | 140698982 | GC | G | 1 | NM_005642 | TAF7 | frameshift_deleti  on | exon1 | c.629delG | p.G210fs | . | . |
| CTC4 | 5 | 140711020 | CT | C | 0.75 | NM_018912 | PCDHGA1 | frameshift_deleti  on | exon1 | c.770delT | p.L257fs | . | . |
| CTC4 | 5 | 140864838 | AC | A | 1 | NM_018928 | PCDHGC4 | frameshift_deleti  on | exon1 | c.99delC | p.Y33fs | . | . |
| CTC4 | 5 | 141052403 | GTC | G | 0.17 | NM_022481 | ARAP3 | frameshift_deleti  on | exon8 | c.1181_1182del | p.R394fs | . | . |
| CTC4 | 5 | 142693577 | TC | T | 0.273 | NM_000176 | NR3C1 | frameshift_deleti  on | exon3 | c.1340delG | p.R447fs | . | . |
| CTC4 | 5 | 145499934 | CT | C | 0.266 | NM_020117 | LARS | frameshift_deleti  on | exon31 | c.3324delA | p.K1108fs | . | . |
| CTC4 | 5 | 149279083 | TC | T | 0.182 | NM_000440 | PDE6A | frameshift_deleti  on | exon9 | c.1117delG | p.E373fs | . | . |
| CTC4 | 5 | 156899830 | CA | C | 0.704 | NM_001172292 | NIPAL4 | frameshift_deleti  on | exon5 | c.1207delA | p.K403fs | . | . |
| CTC4 | 5 | 158595923 | AT | A | 1 | NM_001199380 | RNF145 | frameshift_deleti  on | exon8 | c.1168delA | p.I390fs | . | . |
| CTC4 | 5 | 159519576 | G | GA | 0.944 | NM_001130864 | PWWP2A | frameshift_inserti  on | exon2 | c.2080dupT | p.S694fs | . | . |
| CTC4 | 5 | 159520408 | CT | C | 0.5 | NM_001130864 | PWWP2A | frameshift_deleti  on | exon2 | c.1248delA | p.K416fs | . | . |
| CTC4 | 5 | 168149289 | AG | A | 0.485 | NM_001271946 | SLIT3 | frameshift_deleti  on | exon23 | c.2454delC | p.A818fs | . | . |
| CTC4 | 5 | 169015520 | C | CA | 0.895 | NM_017785 | SPDL1 | frameshift_inserti  on | exon2 | c.101dupA | p.Q34fs | . | . |
| CTC4 | 5 | 172362230 | GC | G | 0.231 | NM_001031711 | ERGIC1 | frameshift_deleti  on | exon9 | c.683delC | p.A228fs | . | . |
| CTC4 | 5 | 179023626 | GC | G | 0.364 | NM_001040451 | RUFY1 | frameshift_deleti  on | exon12 | c.1252delC | p.L418fs | . | . |
| CTC4 | 5 | 180651249 | AC | A | 0.93 | NM_033549 | TRIM41 | frameshift_deleti  on | exon1 | c.251delC | p.T84fs | . | . |
| CTC4 | 6 | 2671571 | A | ATTC | 0.154 | NM_001012418 | MYLK4 | nonframeshift_in  sertion | exon12 | c.1130_1131ins  GAA | p.N377delinsK  N | . | . |
| CTC4 | 6 | 2693035 | CT | C | 1 | NM_001012418 | MYLK4 | frameshift_deleti  on | exon3 | c.217delA | p.R73fs | . | . |
| CTC4 | 6 | 3083378 | C | CA | 0.4 | NM_003804 | RIPK1 | frameshift_inserti  on | exon4 | c.520dupA | p.H173fs | . | . |
| CTC4 | 6 | 4037672 | CA | C | 0.358 | NM_003913 | PRPF4B | frameshift_deleti  on | exon3 | c.1281delA | p.S427fs | . | . |
| CTC4 | 6 | 7373940 | AT | A | 0.5 | NM_205864 | CAGE1 | stopgain | exon4 | c.703delA | p.I235X | . | . |
| CTC4 | 6 | 10529169 | CT | C | 0.943 | NM_145649 | GCNT2 | frameshift_deleti on | exon3 | c.26delT | p.L9fs | . | . |
| CTC4 | 6 | 12292614 | CG | C | 0.213 | NM_001168319 | EDN1 | frameshift_deleti  on | exon2 | c.103delG | p.G35fs | . | . |

| CTC4 | 6 | 17675197 | GT | G | 0.5 | NM_001278209 | NUP153 | frameshift_deleti  on | exon5 | c.790delA | p.T264fs | . | . |
| --- | --- | --- | --- | --- | --- | --- | --- | --- | --- | --- | --- | --- | --- |
| CTC4 | 6 | 24302015 | GT | G | 0.985 | NM_016356 | DCDC2 | frameshift_deleti  on | exon4 | c.484delA | p.T162fs | . | . |
| CTC4 | 6 | 28539847 | TG | T | 0.231 | NM_052923 | SCAND3 | stopgain | exon4 | c.3818delC | p.S1273X | . | . |
| CTC4 | 6 | 28543300 | CA | C | 1 | NM_052923 | SCAND3 | frameshift_deleti on | exon3 | c.1181delT | p.L394fs | . | . |
| CTC4 | 6 | 28964259 | CT | C | 0.246 | NM_001010877 | ZNF311 | frameshift_deleti  on | exon7 | c.519delA | p.K173fs | . | . |
| CTC4 | 6 | 29454906 | CT | C | 0.281 | NM_052967 | MAS1L | frameshift_deleti  on | exon1 | c.773delA | p.K258fs | . | . |
| CTC4 | 6 | 30917910 | GC | G | 0.75 | NM_080870 | DPCR1 | frameshift_deleti  on | exon2 | c.1670delC | p.A557fs | . | . |
| CTC4 | 6 | 30994246 | G | GACC | 1 | NM_001198815 | MUC22 | nonframeshift_in  sertion | exon3 | c.1038_1039ins  ACC | p.E346delinsET | . | . |
| CTC4 | 6 | 31595957 | TG | T | 0.371 | NM_004638 | PRRC2A | frameshift_deleti  on | exon12 | c.1707delG | p.L569fs | . | . |
| CTC4 | 6 | 31611915 | T | TG | 0.562 | NM_001098534 | BAG6 | frameshift_inserti  on | exon12 | c.1503dupC | p.T502fs | . | . |
| CTC4 | 6 | 31749887 | CA | C | 1 | NM_006295 | VARS | frameshift_deleti  on | exon18 | c.2217delT | p.S739fs | . | . |
| CTC4 | 6 | 31855914 | CG | C | 0.367 | NM_001289413 | EHMT2 | frameshift_deleti  on | exon11 | c.1717delC | p.R573fs | . | . |
| CTC4 | 6 | 32181612 | TG | T | 0.64 | NM_004557 | NOTCH4 | frameshift_deleti  on | exon14 | c.2172delC | p.P724fs | . | . |
| CTC4 | 6 | 32191658 | TAGC | T | 0.312 | NM_004557 | NOTCH4 | nonframeshift_de  letion | exon1 | c.45_47del | p.15_16del | . | . |
| CTC4 | 6 | 33283251 | G | GC | 1 | NM_001145338 | ZBTB22 | frameshift_inserti  on | exon2 | c.1442_1443ins  G | p.D481fs | . | . |
| CTC4 | 6 | 35436612 | GC | G | 1 | NM_007104 | RPL10A | frameshift_deleti  on | exon2 | c.43delC | p.R15fs | . | . |
| CTC4 | 6 | 35836882 | AT | A | 0.92 | NM_003137 | SRPK1 | frameshift_deleti  on | exon12 | c.1416delA | p.K472fs | . | . |
| CTC4 | 6 | 36447453 | AT | A | 0.556 | NM_001286580 | KCTD20 | frameshift_deleti  on | exon4 | c.189delT | p.N63fs | . | . |
| CTC4 | 6 | 36466165 | CA | C | 1 | NM_007271 | STK38 | frameshift_deleti  on | exon11 | c.1050delT | p.S350fs | . | . |
| CTC4 | 6 | 41029504 | AT | A | 0.917 | NM_006789 | APOBEC2 | frameshift_deleti  on | exon2 | c.570delT | p.N190fs | . | . |
| CTC4 | 6 | 43305183 | CT | C | 0.286 | NM_014345 | ZNF318 | frameshift_deleti  on | exon10 | c.6552delA | p.K2184fs | . | . |
| CTC4 | 6 | 43306790 | GT | G | 0.889 | NM_014345 | ZNF318 | frameshift_deleti  on | exon10 | c.4945delA | p.T1649fs | . | . |
| CTC4 | 6 | 43395832 | AC | A | 0.304 | NM_001198934 | ABCC10 | frameshift_deleti  on | exon2 | c.117delC | p.H39fs | . | . |
| CTC4 | 6 | 43472860 | A | AC | 0.368 | NM_001146020 | TJAP1 | frameshift_inserti  on | exon9 | c.912dupC | p.N304fs | . | . |
| CTC4 | 6 | 43572467 | CGG | C | 0.958 | NM_006502 | POLH | frameshift_deleti  on | exon8 | c.1001_1002del | p.R334fs | . | . |
| CTC4 | 6 | 43970503 | CGCG | C | 0.6 | NM_153246 | C6orf223 | nonframeshift_de  letion | exon4 | c.370_372del | p.124_124del | . | . |
| CTC4 | 6 | 46977412 | AT | A | 1 | NM_153840 | GPR110 | frameshift_deleti  on | exon11 | c.1758delA | p.K586fs | . | . |
| CTC4 | 6 | 51611556 | CT | C | 0.312 | NM_138694 | PKHD1 | frameshift_deleti  on | exon59 | c.9960delA | p.K3320fs | . | . |
| CTC4 | 6 | 54208119 | TG | T | 0.565 | NM_014464 | TINAG | frameshift_deleti  on | exon5 | c.721delG | p.A241fs | . | . |
| CTC4 | 6 | 62757823 | AT | A | 1 | NM_152688 | KHDRBS2 | frameshift_deleti  on | exon3 | c.295delA | p.M99fs | . | . |
| CTC4 | 6 | 69349148 | CA | C | 0.417 | NM_001704 | BAI3 | frameshift_deleti  on | exon3 | c.582delA | p.T194fs | . | . |
| CTC4 | 6 | 75875270 | TA | T | 0.531 | NM_004370 | COL12A1 | frameshift_deleti  on | exon14 | c.2935delT | p.Y979fs | . | . |
| CTC4 | 6 | 76423482 | CA | C | 0.6 | NM_001100409 | SENP6 | frameshift_deleti  on | exon22 | c.3050delA | p.Q1017fs | . | . |
| CTC4 | 6 | 83865752 | TA | T | 0.333 | NM_001199942 | DOPEY1 | frameshift_deleti  on | exon34 | c.6508delA | p.K2170fs | . | . |
| CTC4 | 6 | 84884542 | CT | C | 0.5 | NM_001286206 | KIAA1009 | frameshift_deleti  on | exon15 | c.1700delA | p.K567fs | . | . |
| CTC4 | 6 | 87967218 | GA | G | 1 | NM_015021 | ZNF292 | frameshift_deleti  on | exon8 | c.3872delA | p.E1291fs | . | . |
| CTC4 | 6 | 90468656 | CT | C | 0.514 | NM_014611 | MDN1 | frameshift_deleti  on | exon18 | c.2483delA | p.K828fs | . | . |
| CTC4 | 6 | 90578091 | CA | C | 0.316 | NM_001137667 | CASP8AP2 | frameshift_deleti  on | exon8 | c.5083delA | p.K1695fs | . | . |
| CTC4 | 6 | 105609360 | TG | T | 0.273 | NM_022361 | POPDC3 | frameshift_deleti  on | exon2 | c.424delC | p.H142fs | . | . |
| CTC4 | 6 | 106553231 | C | CG | 0.346 | NM_182907 | PRDM1 | frameshift_inserti  on | exon3 | c.795dupG | p.P265fs | . | . |
| CTC4 | 6 | 109906329 | GCTT | G | 1 | NM_001145128 | AK9 | nonframeshift_de  letion | exon19 | c.2108_2110del | p.703_704del | . | . |
| CTC4 | 6 | 109906339 | CTTCT | C | 1 | NM_001145128 | AK9 | frameshift_deleti  on | exon19 | c.2097_2100del | p.K699fs | . | . |
| CTC4 | 6 | 112029210 | AC | A | 1 | NM_153047 | FYN | frameshift_deleti  on | exon3 | c.357delG | p.W119fs | . | . |
| CTC4 | 6 | 116542389 | TG | T | 0.444 | NM_152729 | NT5DC1 | frameshift_deleti  on | exon7 | c.703delG | p.G235fs | . | . |
| CTC4 | 6 | 116972835 | CA | C | 0.469 | NM_145062 | ZUFSP | frameshift_deleti  on | exon7 | c.1215delT | p.F405fs | . | . |
| CTC4 | 6 | 138645201 | CG | C | 0.412 | NM_020340 | KIAA1244 | frameshift_deleti  on | exon31 | c.4912delG | p.G1638fs | . | . |
| CTC4 | 6 | 144033198 | CA | C | 0.583 | NM_001100164 | PHACTR2 | frameshift_deleti  on | exon2 | c.93delA | p.T31fs | . | . |
| CTC4 | 6 | 145115078 | CT | C | 0.682 | NM_007124 | UTRN | frameshift_deleti  on | exon62 | c.9030delT | p.A3010fs | . | . |
| CTC4 | 6 | 147612267 | CA | C | 0.417 | NM_001127715 | STXBP5 | frameshift_deleti  on | exon9 | c.877delA | p.K293fs | . | . |
| CTC4 | 6 | 150052827 | T | TA | 1 | NM_198887 | NUP43 | frameshift_inserti  on | exon7 | c.834dupT | p.T279fs | . | . |
| CTC4 | 6 | 151686974 | C | CA | 1 | NM_020861 | ZBTB2 | frameshift_inserti  on | exon3 | c.1226dupT | p.V409fs | . | . |
| CTC4 | 6 | 152523022 | C | CT | 0.347 | NM_033071 | SYNE1 | frameshift_inserti  on | exon126 | c.22868dupA | p.K7623fs | . | . |
| CTC4 | 6 | 152555036 | A | AT | 0.175 | NM_033071 | SYNE1 | frameshift_inserti  on | exon111 | c.20378dupA | p.N6793fs | . | . |
| CTC4 | 6 | 152748870 | AG | A | 0.425 | NM_033071 | SYNE1 | frameshift_deleti  on | exon38 | c.5078delC | p.S1693fs | . | . |
| CTC4 | 6 | 152757150 | AT | A | 0.5 | NM_033071 | SYNE1 | frameshift_deleti  on | exon33 | c.4256delA | p.N1419fs | . | . |
| CTC4 | 6 | 155575167 | CAG | C | 0.142 | NM_001010927 | TIAM2 | frameshift_deleti  on | exon11 | c.1062_1063del | p.P354fs | . | . |
| CTC4 | 6 | 158900789 | AT | A | 0.588 | NM_001007466 | TULP4 | frameshift_deleti  on | exon7 | c.1034delT | p.I345fs | . | . |
| CTC4 | 6 | 159331098 | AG | A | 0.237 | NM_001195032 | C6orf99 | frameshift_deleti  on | exon3 | c.232delG | p.G78fs | . | . |

| CTC4 | 6 | 159660745 | C | CT | 0.39 | NM_032532 | FNDC1 | frameshift_inserti  on | exon14 | c.4377_4378ins  T | p.P1459fs | . | . |
| --- | --- | --- | --- | --- | --- | --- | --- | --- | --- | --- | --- | --- | --- |
| CTC4 | 6 | 159660779 | GCCACC  ACCCGC CGCACG | G | 0.667 | NM_032532 | FNDC1 | nonframeshift_de letion | exon14 | c.4412_4429del | p.1471_1477de l | . | . |
| CTC4 | 6 | 167721388 | AC | A | 1 | NM_001143947 | UNC93A | frameshift_deleti  on | exon6 | c.973delC | p.Q325fs | . | . |
| CTC4 | 6 | 168299142 | AC | A | 0.235 | NM_001207008 | MLLT4 | frameshift_deleti  on | exon10 | c.1528delC | p.P510fs | . | . |
| CTC4 | 6 | 170871004 | G | GTAA | 0.315 | NM_001172085 | TBP | stopgain | exon2 | c.120_121insTA  A | p.Q40delinsQX | . | . |
| CTC4 | 6 | 170871013 | A | ACAG | 0.928 | NM_001172085 | TBP | nonframeshift_in  sertion | exon2 | c.129_130insCA  G | p.Q43delinsQQ | . | . |
| CTC4 | 6 | 170871027 | A | AGCT | 0.623 | NM_001172085 | TBP | nonframeshift_in  sertion | exon2 | c.143_144insG  CT | p.Q48delinsQL | . | . |
| CTC4 | 6 | 170871034 | G | GCAA | 0.86 | NM_001172085 | TBP | nonframeshift_in  sertion | exon2 | c.150_151insCA  A | p.Q50delinsQQ | . | . |
| CTC4 | 6 | 170871037 | GCAA | G | 0.882 | NM_001172085 | TBP | nonframeshift_de  letion | exon2 | c.154_156del | p.52_52del | . | . |
| CTC4 | 6 | 170871046 | ACAGCA  GCAG | A | 1 | NM_001172085 | TBP | nonframeshift_de  letion | exon2 | c.163_171del | p.55_57del | . | . |
| CTC4 | 7 | 5530900 | GA | G | 0.818 | NM_024963 | FBXL18 | frameshift_deleti  on | exon4 | c.1961delT | p.L654fs | . | . |
| CTC4 | 7 | 11632949 | C | CT | 0.396 | NM_015204 | THSD7A | frameshift_inserti  on | exon3 | c.1202dupA | p.K401fs | . | . |
| CTC4 | 7 | 18067193 | CA | C | 0.99 | NM_175886 | PRPS1L1 | stopgain | exon1 | c.212delT | p.L71X | . | . |
| CTC4 | 7 | 20421424 | TG | T | 0.969 | NM_002214 | ITGB8 | frameshift_deleti on | exon6 | c.877delG | p.D293fs | . | . |
| CTC4 | 7 | 21468303 | AAGG | A | 0.135 | NM_003112 | SP4 | nonframeshift_de  letion | exon2 | c.17_19del | p.6_7del | . | . |
| CTC4 | 7 | 22186587 | CA | C | 0.365 | NM_012294 | RAPGEF5 | frameshift_deleti  on | exon19 | c.1475delT | p.L492fs | . | . |
| CTC4 | 7 | 23183618 | AT | A | 0.244 | NM_001031710 | KLHL7 | frameshift_deleti  on | exon6 | c.768delT | p.N256fs | . | . |
| CTC4 | 7 | 30085906 | G | GA | 0.9 | NM_001197026 | PLEKHA8 | frameshift_inserti  on | exon3 | c.239dupA | p.E80fs | . | . |
| CTC4 | 7 | 31682733 | TG | T | 1 | NM_194300 | CCDC129 | frameshift_deleti  on | exon10 | c.1750delG | p.E584fs | . | . |
| CTC4 | 7 | 38797994 | C | CT | 0.464 | NM_080631 | VPS41 | frameshift_inserti  on | exon17 | c.1434dupA | p.D479fs | . | . |
| CTC4 | 7 | 42065976 | A | AC | 0.467 | NM_000168 | GLI3 | frameshift_inserti  on | exon8 | c.1063dupG | p.V355fs | . | . |
| CTC4 | 7 | 43843441 | GA | G | 0.344 | NM_000712 | BLVRA | frameshift_deleti  on | exon7 | c.628delA | p.K210fs | . | . |
| CTC4 | 7 | 51092949 | GT | G | 0.4 | NM_015198 | COBL | frameshift_deleti  on | exon12 | c.3624delA | p.P1208fs | . | . |
| CTC4 | 7 | 56151382 | TA | T | 1 | NM_001258460 | PHKG1 | frameshift_deleti  on | exon5 | c.316delT | p.Y106fs | . | . |
| CTC4 | 7 | 65419353 | T | TTTA | 0.714 | NM_001284342 | VKORC1L1 | nonframeshift_in  sertion | exon2 | c.487_488insTT  A | p.F163delinsFI | . | . |
| CTC4 | 7 | 65419364 | T | TATC | 0.485 | NM_001284342 | VKORC1L1 | nonframeshift_in  sertion | exon2 | c.498_499insAT  C | p.I166delinsII | . | . |
| CTC4 | 7 | 65426035 | AG | A | 0.4 | NM_001284290 | GUSB | frameshift_deleti  on | exon10 | c.1366delC | p.L456fs | . | . |
| CTC4 | 7 | 73482999 | TG | T | 0.833 | NM_001278918 | ELN | frameshift_deleti  on | exon28 | c.1824delG | p.L608fs | . | . |
| CTC4 | 7 | 77762256 | GA | G | 0.227 | NM_012301 | MAGI2 | frameshift_deleti  on | exon18 | c.3152delT | p.I1051fs | . | . |
| CTC4 | 7 | 82581488 | T | TTCA | 1 | NM_014510 | PCLO | nonframeshift_in  sertion | exon5 | c.8780_8781ins  TGA | p.E2927delinsD  E | . | . |
| CTC4 | 7 | 86542498 | AT | A | 1 | NM_152748 | KIAA1324L | frameshift_deleti  on | exon13 | c.1252delA | p.M418fs | . | . |
| CTC4 | 7 | 88965512 | A | AT | 0.429 | NM_181646 | ZNF804B | frameshift_inserti  on | exon4 | c.3217dupT | p.E1072fs | . | . |
| CTC4 | 7 | 91712638 | TA | T | 0.333 | NM_005751 | AKAP9 | frameshift_deleti  on | exon33 | c.8316delA | p.I2772fs | . | . |
| CTC4 | 7 | 92733755 | TG | T | 0.195 | NM_001193307 | SAMD9 | frameshift_deleti  on | exon2 | c.1655delC | p.P552fs | . | . |
| CTC4 | 7 | 92762349 | TA | T | 0.242 | NM_152703 | SAMD9L | frameshift_deleti  on | exon5 | c.2935delT | p.Y979fs | . | . |
| CTC4 | 7 | 93101744 | GT | G | 1 | NM_001164738 | CALCR | frameshift_deleti  on | exon5 | c.371delA | p.N124fs | . | . |
| CTC4 | 7 | 94049894 | CA | C | 1 | NM_000089 | COL1A2 | frameshift_deleti  on | exon37 | c.2230delA | p.K744fs | . | . |
| CTC4 | 7 | 94229998 | GA | G | 1 | NM_001099400 | SGCE | frameshift_deleti  on | exon7 | c.996delT | p.F332fs | . | . |
| CTC4 | 7 | 95025607 | TC | T | 0.974 | NM_000940 | PON3 | frameshift_deleti  on | exon1 | c.55delG | p.E19fs | . | . |
| CTC4 | 7 | 96810517 | CA | C | 0.258 | NM_020186 | ACN9 | frameshift_deleti  on | exon2 | c.369delA | p.P123fs | . | . |
| CTC4 | 7 | 97870413 | GA | G | 0.929 | NM_015395 | TECPR1 | frameshift_deleti  on | exon7 | c.800delT | p.V267fs | . | . |
| CTC4 | 7 | 98638068 | TC | T | 1 | NM_001199847 | SMURF1 | frameshift_deleti  on | exon13 | c.1482delG | p.G494fs | . | . |
| CTC4 | 7 | 98647232 | TA | T | 1 | NM_001199847 | SMURF1 | frameshift_deleti  on | exon9 | c.906delT | p.N302fs | . | . |
| CTC4 | 7 | 99017678 | TGC | T | 0.189 | NM_015545 | PTCD1 | frameshift_deleti  on | exon8 | c.2013_2014del | p.P671fs | . | . |
| CTC4 | 7 | 99103705 | A | AC | 0.795 | NM_014569 | ZKSCAN5 | frameshift_inserti  on | exon2 | c.39dupC | p.D13fs | . | . |
| CTC4 | 7 | 99662511 | GTAGT | G | 1 | NM_017715 | ZNF3 | frameshift_deleti  on | exon6 | c.292_295del | p.T98fs | . | . |
| CTC4 | 7 | 99686615 | TGGC | T | 0.143 | NM_006833 | COPS6 | nonframeshift_de  letion | exon1 | c.3_5del | p.1_2del | . | . |
| CTC4 | 7 | 99695837 | TG | T | 1 | NM_001278595 | MCM7 | frameshift_deleti  on | exon6 | c.268delC | p.Q90fs | . | . |
| CTC4 | 7 | 100074990 | GC | G | 1 | NM_030935 | TSC22D4 | frameshift_deleti  on | exon2 | c.671delG | p.G224fs | . | . |
| CTC4 | 7 | 100202795 | CG | C | 0.231 | NM_002593 | PCOLCE | frameshift_deleti  on | exon4 | c.546delG | p.P182fs | . | . |
| CTC4 | 7 | 104746345 | A | AT | 0.389 | NM_018682 | KMT2E | frameshift_inserti  on | exon18 | c.2492dupT | p.I831fs | . | . |
| CTC4 | 7 | 105254932 | TG | T | 1 | NM_138495 | ATXN7L1 | frameshift_deleti  on | exon8 | c.1476delC | p.H492fs | . | . |
| CTC4 | 7 | 107717482 | AC | A | 0.31 | NM_007356 | LAMB4 | frameshift_deleti  on | exon17 | c.2030delG | p.C677fs | . | . |
| CTC4 | 7 | 111368489 | GA | G | 1 | NM_014705 | DOCK4 | frameshift_deleti  on | exon52 | c.5741delT | p.V1914fs | . | . |
| CTC4 | 7 | 111430594 | CT | C | 1 | NM_014705 | DOCK4 | frameshift_deleti  on | exon31 | c.3233delA | p.Q1078fs | . | . |
| CTC4 | 7 | 111430595 | TG | T | 1 | NM_014705 | DOCK4 | frameshift_deleti  on | exon31 | c.3232delC | p.Q1078fs | . | . |

| CTC4 | 7 | 113519717 | AT | A | 1 | NM_002711 | PPP1R3A | frameshift_deleti  on | exon4 | c.1429delA | p.I477fs | . | . |
| --- | --- | --- | --- | --- | --- | --- | --- | --- | --- | --- | --- | --- | --- |
| CTC4 | 7 | 113519718 | TA | T | 1 | NM_002711 | PPP1R3A | frameshift_deleti  on | exon4 | c.1428delT | p.N476fs | . | . |
| CTC4 | 7 | 128587351 | CACTCT  GCAGCC GCCCAC TCTGCG | C | 1 | NM_001098627 | IRF5 | nonframeshift_de letion | exon6 | c.502_531del | p.168_177del | . | . |
| CTC4 | 7 | 134617842 | CA | C | 0.644 | NM_033139 | CALD1 | frameshift_deleti  on | exon3 | c.305delA | p.Q102fs | . | . |
| CTC4 | 7 | 135378982 | TA | T | 0.6 | NM_012450 | SLC13A4 | frameshift_deleti  on | exon10 | c.1020delT | p.F340fs | . | . |
| CTC4 | 7 | 138223409 | CA | C | 0.471 | NM_003852 | TRIM24 | frameshift_deleti  on | exon7 | c.1005delA | p.A335fs | . | . |
| CTC4 | 7 | 138603336 | TCA | T | 0.258 | NM_001164665 | KIAA1549 | frameshift_deleti  on | exon2 | c.1034_1035del | p.V345fs | . | . |
| CTC4 | 7 | 138719403 | AT | A | 0.5 | NM_080660 | ZC3HAV1L | frameshift_deleti  on | exon2 | c.386delA | p.H129fs | . | . |
| CTC4 | 7 | 138849881 | GT | G | 0.706 | NM_001287512 | TTC26 | frameshift_deleti  on | exon6 | c.404delT | p.V135fs | . | . |
| CTC4 | 7 | 138872160 | GT | G | 1 | NM_001287512 | TTC26 | frameshift_deleti  on | exon14 | c.1036delT | p.F346fs | . | . |
| CTC4 | 7 | 139094364 | AAG | A | 0.3 | NM_016019 | LUC7L2 | frameshift_deleti  on | exon7 | c.744_745del | p.E248fs | . | . |
| CTC4 | 7 | 139636031 | GT | G | 1 | NM_001061 | TBXAS1 | frameshift_deleti  on | exon5 | c.379delT | p.F127fs | . | . |
| CTC4 | 7 | 141478668 | C | CG | 0.6 | NM_016944 | TAS2R4 | frameshift_inserti  on | exon1 | c.380_381insG | p.P127fs | . | . |
| CTC4 | 7 | 142562051 | CCCT | C | 0.162 | NM_004445 | EPHB6 | nonframeshift_de  letion | exon4 | c.494_496del | p.165_166del | . | . |
| CTC4 | 7 | 142562446 | CG | C | 0.636 | NM_001280795 | EPHB6 | frameshift_deleti  on | exon3 | c.13delG | p.G5fs | . | . |
| CTC4 | 7 | 142983864 | GC | G | 0.411 | NM_001282876 | TMEM139 | frameshift_deleti  on | exon3 | c.594delC | p.C198fs | . | . |
| CTC4 | 7 | 143000960 | GA | G | 0.353 | NM_032982 | CASP2 | frameshift_deleti  on | exon9 | c.1052delA | p.E351fs | . | . |
| CTC4 | 7 | 148506206 | TG | T | 0.405 | NM_001203249 | EZH2 | frameshift_deleti  on | exon18 | c.1983delC | p.A661fs | . | . |
| CTC4 | 7 | 150554126 | CG | C | 0.263 | NM_001091 | AOC1 | frameshift_deleti  on | exon2 | c.569delG | p.R190fs | . | . |
| CTC4 | 7 | 150648038 | AC | A | 0.556 | NM_001204798 | KCNH2 | frameshift_deleti  on | exon4 | c.1095delG | p.W365fs | . | . |
| CTC4 | 7 | 150730769 | CT | C | 1 | NM_001282292 | ABCB8 | frameshift_deleti  on | exon2 | c.174delT | p.P58fs | . | . |
| CTC4 | 7 | 151093031 | T | TG | 0.294 | NM_001284260 | WDR86 | frameshift_inserti  on | exon3 | c.556dupC | p.H186fs | . | . |
| CTC4 | 7 | 151927047 | C | CAT | 0.344 | NM_170606 | KMT2C | frameshift_inserti  on | exon18 | c.2936_2937ins  AT | p.Q979fs | . | . |
| CTC4 | 8 | 2077153 | GA | G | 0.407 | NM_003970 | MYOM2 | frameshift_deleti  on | exon32 | c.3734delA | p.E1245fs | . | . |
| CTC4 | 8 | 3889436 | AG | A | 0.4 | NM_033225 | CSMD1 | frameshift_deleti  on | exon4 | c.600delC | p.P200fs | . | . |
| CTC4 | 8 | 21827694 | TG | T | 0.226 | NM_015024 | XPO7 | frameshift_deleti  on | exon4 | c.300delG | p.L100fs | . | . |
| CTC4 | 8 | 21986484 | TG | T | 0.5 | NM_005144 | HR | frameshift_deleti  on | exon2 | c.199delC | p.Q67fs | . | . |
| CTC4 | 8 | 22054911 | C | CT | 0.612 | NM_001199 | BMP1 | frameshift_inserti  on | exon15 | c.2086dupT | p.G695fs | . | . |
| CTC4 | 8 | 22269552 | TG | T | 0.301 | NM_001128431 | SLC39A14 | frameshift_deleti  on | exon4 | c.461delG | p.W154fs | . | . |
| CTC4 | 8 | 24254932 | GC | G | 0.594 | NM_001145272 | ADAMDEC1 | frameshift_deleti  on | exon5 | c.354delC | p.G118fs | . | . |
| CTC4 | 8 | 24813740 | AG | A | 1 | NM_006158 | NEFL | frameshift_deleti  on | exon1 | c.289delC | p.L97fs | . | . |
| CTC4 | 8 | 27277444 | GA | G | 0.156 | NM_173175 | PTK2B | frameshift_deleti  on | exon3 | c.238delA | p.I80fs | . | . |
| CTC4 | 8 | 27891119 | TA | T | 0.231 | NM_001010906 | NUGGC | frameshift_deleti  on | exon14 | c.1706delT | p.L569fs | . | . |
| CTC4 | 8 | 30703194 | AG | A | 0.75 | NM_031271 | TEX15 | frameshift_deleti  on | exon1 | c.3339delC | p.V1113fs | . | . |
| CTC4 | 8 | 33310884 | AT | A | 0.595 | NM_032664 | FUT10 | frameshift_deleti  on | exon3 | c.225delA | p.K75fs | . | . |
| CTC4 | 8 | 38066751 | AG | A | 0.632 | NM_001204878 | BAG4 | frameshift_deleti  on | exon3 | c.747delG | p.Q249fs | . | . |
| CTC4 | 8 | 38125891 | AG | A | 1 | NM_001102559 | PPAPDC1B | frameshift_deleti  on | exon3 | c.271delC | p.L91fs | . | . |
| CTC4 | 8 | 39581352 | AG | A | 0.514 | NM_014237 | ADAM18 | frameshift_deleti  on | exon19 | c.2104delG | p.V702fs | . | . |
| CTC4 | 8 | 39581353 | GT | G | 0.357 | NM_014237 | ADAM18 | frameshift_deleti  on | exon19 | c.2105delT | p.V702fs | . | . |
| CTC4 | 8 | 39624451 | GC | G | 0.364 | NM_001278113 | ADAM2 | frameshift_deleti  on | exon13 | c.1365delG | p.G455fs | . | . |
| CTC4 | 8 | 39679173 | GC | G | 0.253 | NM_001278113 | ADAM2 | frameshift_deleti  on | exon5 | c.275delG | p.C92fs | . | . |
| CTC4 | 8 | 41798419 | GCTC | G | 0.106 | NM_006766 | KAT6A | nonframeshift_de  letion | exon15 | c.2977_2979del | p.993_993del | . | . |
| CTC4 | 8 | 53536368 | C | CT | 0.398 | NM_001083617 | RB1CC1 | frameshift_inserti  on | exon24 | c.4749dupA | p.A1584fs | . | . |
| CTC4 | 8 | 54147361 | CA | C | 0.883 | NM_000912 | OPRK1 | frameshift_deleti  on | exon3 | c.567delT | p.V189fs | . | . |
| CTC4 | 8 | 56270301 | GT | G | 0.556 | NM_052898 | XKR4 | frameshift_deleti  on | exon2 | c.871delT | p.F291fs | . | . |
| CTC4 | 8 | 67039647 | G | GA | 0.209 | NM_033058 | TRIM55 | frameshift_inserti  on | exon1 | c.145dupA | p.R48fs | . | . |
| CTC4 | 8 | 67590185 | GC | G | 0.368 | NM_019607 | C8orf44 | frameshift_deleti  on | exon2 | c.243delC | p.S81fs | . | . |
| CTC4 | 8 | 69104654 | AAC | A | 1 | NM_024870 | PREX2 | frameshift_deleti  on | exon37 | c.4499_4500del | p.N1500fs | . | . |
| CTC4 | 8 | 73848703 | AT | A | 0.458 | NM_004770 | KCNB2 | frameshift_deleti  on | exon3 | c.1114delT | p.F372fs | . | . |
| CTC4 | 8 | 81430808 | GA | G | 0.857 | NM_001105539 | ZBTB10 | frameshift_deleti  on | exon4 | c.2132delA | p.E711fs | . | . |
| CTC4 | 8 | 81431472 | TA | T | 0.5 | NM_001105539 | ZBTB10 | frameshift_deleti  on | exon6 | c.2326delA | p.K776fs | . | . |
| CTC4 | 8 | 82606551 | AC | A | 0.3 | NM_001010893 | SLC10A5 | frameshift_deleti  on | exon1 | c.656delG | p.G219fs | . | . |
| CTC4 | 8 | 86042232 | CGA | C | 0.868 | NM_033402 | LRRCC1 | frameshift_deleti  on | exon11 | c.1706_1707del | p.R569fs | . | . |
| CTC4 | 8 | 86126827 | C | CAACA  TT | 1 | NM_001099673 | C8orf59 | nonframeshift_in  sertion | exon4 | c.264_265insAA  TGTT | p.D89delinsNV  D | . | . |
| CTC4 | 8 | 95893874 | GT | G | 0.222 | NM_057749 | CCNE2 | frameshift_deleti  on | exon12 | c.1200delA | p.P400fs | . | . |
| CTC4 | 8 | 96264418 | GA | G | 1 | NM_177965 | C8orf37 | frameshift_deleti  on | exon5 | c.467delT | p.F156fs | . | . |

| CTC4 | 8 | 97311941 | TC | T | 0.286 | NM_014754 | PTDSS1 | frameshift_deleti  on | exon6 | c.621delC | p.L207fs | . | . |
| --- | --- | --- | --- | --- | --- | --- | --- | --- | --- | --- | --- | --- | --- |
| CTC4 | 8 | 97312009 | A | AT | 0.261 | NM_014754 | PTDSS1 | frameshift_inserti  on | exon6 | c.689dupT | p.I230fs | . | . |
| CTC4 | 8 | 99042717 | GA | G | 0.271 | NM_002380 | MATN2 | frameshift_deleti  on | exon15 | c.2385delA | p.G795fs | . | . |
| CTC4 | 8 | 99539136 | GT | G | 0.5 | NM_001256313 | STK3 | frameshift_deleti  on | exon8 | c.817delA | p.T273fs | . | . |
| CTC4 | 8 | 100515140 | TA | T | 0.583 | NM_017890 | VPS13B | frameshift_deleti  on | exon27 | c.4120delA | p.K1374fs | . | . |
| CTC4 | 8 | 100789145 | GA | G | 0.344 | NM_017890 | VPS13B | frameshift_deleti  on | exon41 | c.7466delA | p.E2489fs | . | . |
| CTC4 | 8 | 100847540 | GA | G | 0.492 | NM_017890 | VPS13B | frameshift_deleti  on | exon53 | c.9806delA | p.E3269fs | . | . |
| CTC4 | 8 | 101153058 | AT | A | 1 | NM_001029860 | FBXO43 | stopgain | exon2 | c.1423delA | p.I475X | . | . |
| CTC4 | 8 | 116616622 | CT | C | 1 | NM_001282902 | TRPS1 | frameshift_deleti on | exon3 | c.1546delA | p.S516fs | . | . |
| CTC4 | 8 | 116616623 | TG | T | 1 | NM_001282902 | TRPS1 | frameshift_deleti  on | exon3 | c.1545delC | p.S515fs | . | . |
| CTC4 | 8 | 120435363 | GA | G | 1 | NM_002514 | NOV | frameshift_deleti  on | exon5 | c.1066delA | p.K356fs | . | . |
| CTC4 | 8 | 122626382 | CT | C | 0.6 | NM_005328 | HAS2 | frameshift_deleti  on | exon4 | c.1625delA | p.K542fs | . | . |
| CTC4 | 8 | 124701122 | TA | T | 0.333 | NM_004306 | ANXA13 | frameshift_deleti  on | exon9 | c.706delT | p.Y236fs | . | . |
| CTC4 | 8 | 125080022 | TC | T | 0.485 | NM_001039112 | FER1L6 | frameshift_deleti  on | exon28 | c.3694delC | p.P1232fs | . | . |
| CTC4 | 8 | 131414155 | GA | G | 0.659 | NM_018482 | ASAP1 | frameshift_deleti  on | exon2 | c.34delT | p.S12fs | . | . |
| CTC4 | 8 | 131797631 | GT | G | 0.623 | NM_001115 | ADCY8 | frameshift_deleti  on | exon16 | c.3150delA | p.K1050fs | . | . |
| CTC4 | 8 | 133141618 | TC | T | 0.483 | NM_001204824 | KCNQ3 | frameshift_deleti  on | exon15 | c.2149delG | p.E717fs | . | . |
| CTC4 | 8 | 142367648 | G | GT | 0.538 | NM_005293 | GPR20 | frameshift_inserti  on | exon2 | c.375_376insA | p.H126fs | . | . |
| CTC4 | 8 | 145223313 | C | CT | 0.481 | NM_001099281 | MROH1 | frameshift_inserti  on | exon3 | c.139dupT | p.A46fs | . | . |
| CTC4 | 8 | 145660217 | C | CA | 0.727 | NM_013432 | TONSL | frameshift_inserti  on | exon20 | c.3061_3062ins  T | p.R1021fs | . | . |
| CTC4 | 8 | 145999616 | CTTT | C | 0.599 | NM_001286770 | ZNF34 | nonframeshift_de  letion | exon5 | c.595_597del | p.199_199del | . | . |
| CTC4 | 9 | 463680 | CA | C | 1 | NM_001190458 | DOCK8 | frameshift_deleti  on | exon45 | c.5933delA | p.Q1978fs | . | . |
| CTC4 | 9 | 2652817 | GT | G | 1 | NM_001018056 | VLDLR | frameshift_deleti  on | exon17 | c.2371delT | p.W791fs | . | . |
| CTC4 | 9 | 4576096 | T | TCC | 0.312 | NM_004170 | SLC1A1 | frameshift_inserti  on | exon9 | c.971_972insCC | p.L324fs | . | . |
| CTC4 | 9 | 6250592 | GA | G | 0.226 | NM_001199640 | IL33 | frameshift_deleti  on | exon3 | c.211delA | p.K71fs | . | . |
| CTC4 | 9 | 6553485 | CA | C | 0.346 | NM_000170 | GLDC | frameshift_deleti  on | exon20 | c.2339delT | p.L780fs | . | . |
| CTC4 | 9 | 12775861 | T | TGGCG  GCGGC | 1 | NM_203403 | LURAP1L | nonframeshift_in  sertion | exon1 | c.147_148insG  GCGGCGGC | p.G49delinsGG  GG | . | . |
| CTC4 | 9 | 12775867 | C | CGGCG  GCGGG | 0.904 | NM_203403 | LURAP1L | nonframeshift_in  sertion | exon1 | c.153_154insG  GCGGCGGG | p.G51delinsGG  GG | . | . |
| CTC4 | 9 | 12775877 | G | GGCGG  CGGCT | 0.958 | NM_203403 | LURAP1L | nonframeshift_in  sertion | exon1 | c.163_164insG  CGGCGGCT | p.G55delinsGG  GC | . | . |
| CTC4 | 9 | 19345991 | TC | T | 0.25 | NM_017925 | DENND4C | frameshift_deleti  on | exon22 | c.3078delC | p.L1026fs | . | . |
| CTC4 | 9 | 19424727 | CT | C | 0.522 | NM_001010887 | ACER2 | frameshift_deleti  on | exon3 | c.254delT | p.L85fs | . | . |
| CTC4 | 9 | 20414310 | ACTG | A | 0.333 | NM_001286691 | MLLT3 | nonframeshift_de  letion | exon5 | c.522_524del | p.174_175del | . | . |
| CTC4 | 9 | 21333124 | CA | C | 1 | NM_018847 | KLHL9 | frameshift_deleti  on | exon1 | c.1734delT | p.F578fs | . | . |
| CTC4 | 9 | 32436344 | ATT | A | 0.291 | NM_002197 | ACO1 | frameshift_deleti  on | exon18 | c.2197_2198del | p.F733fs | . | . |
| CTC4 | 9 | 32467801 | TA | T | 0.448 | NM_014314 | DDX58 | frameshift_deleti  on | exon15 | c.2143delT | p.Y715fs | . | . |
| CTC4 | 9 | 33463082 | C | CT | 0.234 | NM_022917 | NOL6 | frameshift_inserti  on | exon25 | c.3239dupA | p.E1080fs | . | . |
| CTC4 | 9 | 34724972 | GA | G | 0.22 | NM_001141917 | FAM205A | frameshift_deleti  on | exon4 | c.2264delT | p.V755fs | . | . |
| CTC4 | 9 | 35105822 | CT | C | 0.966 | NM_025182 | FAM214B | frameshift_deleti  on | exon8 | c.1319delA | p.K440fs | . | . |
| CTC4 | 9 | 35310747 | CA | C | 0.378 | NM_006377 | UNC13B | frameshift_deleti  on | exon9 | c.1046delA | p.Q349fs | . | . |
| CTC4 | 9 | 74355055 | C | CT | 0.714 | NM_001135820 | TMEM2 | frameshift_inserti  on | exon5 | c.1127dupA | p.K376fs | . | . |
| CTC4 | 9 | 88201784 | GC | G | 1 | NM_001286715 | AGTPBP1 | frameshift_deleti  on | exon21 | c.3150delG | p.G1050fs | . | . |
| CTC4 | 9 | 95397540 | TG | T | 1 | NM_022755 | IPPK | stopgain | exon10 | c.966delC | p.Y322X | . | . |
| CTC4 | 9 | 96291687 | GA | G | 0.267 | NM_001286722 | FAM120A | frameshift_deleti on | exon9 | c.1557delA | p.G519fs | . | . |
| CTC4 | 9 | 97080944 | GAGA | G | 1 | NM_017561 | NUTM2F | nonframeshift_de  letion | exon7 | c.2071_2073del | p.691_691del | . | . |
| CTC4 | 9 | 100092968 | A | AGAGG  AG | 1 | NM_020893 | CCDC180 | nonframeshift_in  sertion | exon18 | c.2325_2326ins  GAGGAG | p.E775delinsEE  E | . | . |
| CTC4 | 9 | 100092968 | A | AGAG | 1 | NM_020893 | CCDC180 | nonframeshift_in  sertion | exon18 | c.2325_2326ins  GAG | p.E775delinsEE | . | . |
| CTC4 | 9 | 100092971 | G | GGAGG  AA | 1 | NM_020893 | CCDC180 | nonframeshift_in  sertion | exon18 | c.2328_2329ins  GAGGAA | p.E776delinsEE  E | . | . |
| CTC4 | 9 | 100092974 | G | GGAA | 0.8 | NM_020893 | CCDC180 | nonframeshift_in  sertion | exon18 | c.2331_2332ins  GAA | p.E777delinsEE | . | . |
| CTC4 | 9 | 100092981 | G | GAGGA  GA | 0.875 | NM_020893 | CCDC180 | nonframeshift_in  sertion | exon18 | c.2338_2339ins  AGGAGA | p.E780delinsEE  K | . | . |
| CTC4 | 9 | 101056137 | GC | G | 0.5 | NM_005458 | GABBR2 | frameshift_deleti  on | exon18 | c.2589delG | p.Q863fs | . | . |
| CTC4 | 9 | 109687514 | TG | T | 0.826 | NM_021224 | ZNF462 | frameshift_deleti  on | exon3 | c.1322delG | p.C441fs | . | . |
| CTC4 | 9 | 112141860 | TA | T | 0.88 | NM_001145372 | PTPN3 | stopgain | exon15 | c.1721delT | p.L574X | . | . |
| CTC4 | 9 | 112900341 | G | GGAAG CT | 1 | NM_001004065 | AKAP2 | nonframeshift_in sertion | exon2 | c.2091_2092ins GAAGCT | p.E697delinsEE A | . | . |
| CTC4 | 9 | 112900342 | G | GAAGC  TC | 0.917 | NM_001004065 | AKAP2 | nonframeshift_in  sertion | exon2 | c.2092_2093ins  AAGCTC | p.E698delinsEA  Q | . | . |
| CTC4 | 9 | 112900346 | C | CTGAA  GA | 0.897 | NM_001004065 | AKAP2 | nonframeshift_in  sertion | exon2 | c.2096_2097ins  TGAAGA | p.A699delinsAE  D | . | . |
| CTC4 | 9 | 112900348 | G | GAAGC  TA | 0.897 | NM_001004065 | AKAP2 | nonframeshift_in  sertion | exon2 | c.2098_2099ins  AAGCTA | p.E700delinsEA  K | . | . |

| CTC4 | 9 | 112900350 | A | AGCTG  AG | 0.928 | NM_001004065 | AKAP2 | nonframeshift_in  sertion | exon2 | c.2100_2101ins  GCTGAG | p.E700delinsEA  E | . | . |
| --- | --- | --- | --- | --- | --- | --- | --- | --- | --- | --- | --- | --- | --- |
| CTC4 | 9 | 112900354 | G | GAAGC  T | 0.867 | NM_001004065 | AKAP2 | frameshift_inserti  on | exon2 | c.2104_2105ins  AAGCT | p.A702fs | . | . |
| CTC4 | 9 | 113252027 | TG | T | 1 | NM_153366 | SVEP1 | frameshift_deleti  on | exon9 | c.1832delC | p.P611fs | . | . |
| CTC4 | 9 | 114337084 | C | CA | 0.25 | NM_001146108 | PTGR1 | frameshift_inserti  on | exon8 | c.689dupT | p.M230fs | . | . |
| CTC4 | 9 | 114348294 | TC | T | 0.315 | NM_001146108 | PTGR1 | frameshift_deleti  on | exon5 | c.360delG | p.G120fs | . | . |
| CTC4 | 9 | 115806517 | AT | A | 1 | NM_001282515 | ZFP37 | frameshift_deleti  on | exon4 | c.425delA | p.N142fs | . | . |
| CTC4 | 9 | 115983473 | GC | G | 1 | NM_015258 | FKBP15 | frameshift_deleti  on | exon1 | c.50delG | p.G17fs | . | . |
| CTC4 | 9 | 116930979 | AT | A | 0.2 | NM_032888 | COL27A1 | frameshift_deleti  on | exon3 | c.1145delT | p.I382fs | . | . |
| CTC4 | 9 | 116931002 | GA | G | 0.969 | NM_032888 | COL27A1 | frameshift_deleti  on | exon3 | c.1168delA | p.K390fs | . | . |
| CTC4 | 9 | 130265149 | CAG | C | 0.364 | NM_001005374 | LRSAM1 | frameshift_deleti  on | exon25 | c.2144_2145del | p.Q715fs | . | . |
| CTC4 | 9 | 130928515 | CT | C | 0.8 | NM_001257976 | CIZ1 | frameshift_deleti  on | exon16 | c.2354delA | p.Q785fs | . | . |
| CTC4 | 9 | 131186773 | AC | A | 0.524 | NM_001286760 | CERCAM | frameshift_deleti  on | exon5 | c.413delC | p.T138fs | . | . |
| CTC4 | 9 | 131296079 | GC | G | 0.179 | NM_001003722 | GLE1 | frameshift_deleti  on | exon11 | c.1496delC | p.A499fs | . | . |
| CTC4 | 9 | 132630664 | TGAC | T | 1 | NM_001008563 | USP20 | nonframeshift_de  letion | exon11 | c.1072_1074del | p.358_358del | . | . |
| CTC4 | 9 | 134305573 | G | GA | 0.22 | NM_013318 | PRRC2B | frameshift_inserti  on | exon1 | c.43dupA | p.G14fs | . | . |
| CTC4 | 9 | 138830143 | CG | C | 0.556 | NM_016172 | UBAC1 | frameshift_deleti  on | exon9 | c.1026delC | p.P342fs | . | . |
| CTC4 | 9 | 139273350 | TG | T | 1 | NM_003086 | SNAPC4 | frameshift_deleti  on | exon21 | c.2928delC | p.A976fs | . | . |
| CTC4 | 9 | 139277994 | CGCT | C | 0.126 | NM_003086 | SNAPC4 | nonframeshift_de  letion | exon15 | c.1624_1626del | p.542_542del | . | . |
| CTC4 | 9 | 139753175 | CT | C | 0.365 | NM_206920 | MAMDC4 | frameshift_deleti  on | exon23 | c.2827delT | p.F943fs | . | . |
| CTC4 | 9 | 140611220 | CG | C | 1 | NM_001145527 | EHMT1 | frameshift_deleti  on | exon3 | c.229delG | p.A77fs | . | . |
| CTC4 | 9 | 140611221 | GC | G | 1 | NM_001145527 | EHMT1 | frameshift_deleti  on | exon3 | c.230delC | p.A77fs | . | . |
| CTC4 | 10 | 436722 | CT | C | 1 | NM_014974 | DIP2C | frameshift_deleti  on | exon11 | c.1341delA | p.K447fs | . | . |
| CTC4 | 10 | 6470234 | TG | T | 0.333 | NM_001242413 | PRKCQ | frameshift_deleti  on | exon17 | c.1866delC | p.S622fs | . | . |
| CTC4 | 10 | 8006928 | GC | G | 1 | NM_031923 | TAF3 | frameshift_deleti  on | exon3 | c.1456delC | p.P486fs | . | . |
| CTC4 | 10 | 8007066 | TA | T | 0.429 | NM_031923 | TAF3 | frameshift_deleti  on | exon3 | c.1594delA | p.K532fs | . | . |
| CTC4 | 10 | 8007243 | TA | T | 0.833 | NM_031923 | TAF3 | frameshift_deleti  on | exon3 | c.1771delA | p.K591fs | . | . |
| CTC4 | 10 | 19417237 | GC | G | 0.417 | NM_001142308 | MALRD1 | frameshift_deleti  on | exon8 | c.1032delC | p.C344fs | . | . |
| CTC4 | 10 | 22498695 | TA | T | 0.952 | NM_001199938 | EBLN1 | frameshift_deleti  on | exon1 | c.217delT | p.Y73fs | . | . |
| CTC4 | 10 | 24910248 | A | AT | 0.273 | NM_020824 | ARHGAP21 | frameshift_inserti  on | exon9 | c.575dupA | p.N192fs | . | . |
| CTC4 | 10 | 25887380 | AC | A | 0.149 | NM_020752 | GPR158 | frameshift_deleti  on | exon11 | c.2826delC | p.N942fs | . | . |
| CTC4 | 10 | 27342285 | CTCT | C | 1 | NM_001256053 | ANKRD26 | nonframeshift_de  letion | exon16 | c.1596_1598del | p.532_533del | . | . |
| CTC4 | 10 | 33018276 | CA | C | 1 | NM_024688 | CCDC7 | frameshift_deleti  on | exon10 | c.718delA | p.K240fs | . | . |
| CTC4 | 10 | 38343669 | CTT | C | 0.132 | NM_001278170 | ZNF33A | frameshift_deleti  on | exon4 | c.636_637del | p.T212fs | . | . |
| CTC4 | 10 | 52569666 | TAGC | T | 0.167 | NM_014576 | A1CF | nonframeshift_de  letion | exon12 | c.1594_1596del | p.532_532del | . | . |
| CTC4 | 10 | 61412631 | TA | T | 0.562 | NM_194298 | SLC16A9 | frameshift_deleti  on | exon6 | c.1428delT | p.F476fs | . | . |
| CTC4 | 10 | 61413759 | AT | A | 0.361 | NM_194298 | SLC16A9 | frameshift_deleti  on | exon5 | c.1024delA | p.I342fs | . | . |
| CTC4 | 10 | 61946505 | CG | C | 0.2 | NM_001204404 | ANK3 | frameshift_deleti  on | exon17 | c.2001delC | p.L667fs | . | . |
| CTC4 | 10 | 73462378 | AC | A | 0.905 | NM_001171930 | CDH23 | frameshift_deleti  on | exon23 | c.2661delC | p.D887fs | . | . |
| CTC4 | 10 | 74167690 | TA | T | 0.54 | NM_001195519 | MICU1 | frameshift_deleti  on | exon5 | c.582delT | p.D194fs | . | . |
| CTC4 | 10 | 75296165 | TG | T | 0.206 | NM_152586 | USP54 | frameshift_deleti  on | exon9 | c.1005delC | p.T335fs | . | . |
| CTC4 | 10 | 76781905 | G | GGAA | 0.895 | NM_001256468 | KAT6B | nonframeshift_in  sertion | exon16 | c.2739_2740ins  GAA | p.E913delinsEE | . | . |
| CTC4 | 10 | 81923291 | TGA | T | 0.6 | NM_001157 | ANXA11 | frameshift_deleti  on | exon9 | c.1026_1027del | p.S342fs | . | . |
| CTC4 | 10 | 88441226 | GC | G | 0.95 | NM_001080115 | LDB3 | frameshift_deleti  on | exon4 | c.356delC | p.A119fs | . | . |
| CTC4 | 10 | 88477782 | TG | T | 0.592 | NM_001080114 | LDB3 | frameshift_deleti  on | exon10 | c.1409delG | p.C470fs | . | . |
| CTC4 | 10 | 88813145 | TC | T | 0.234 | NM_005271 | GLUD1 | frameshift_deleti  on | exon12 | c.1510delG | p.D504fs | . | . |
| CTC4 | 10 | 91099850 | AG | A | 1 | NM_001031683 | IFIT3 | frameshift_deleti  on | exon2 | c.1439delG | p.R480fs | . | . |
| CTC4 | 10 | 93272107 | GA | G | 0.365 | NM_182765 | HECTD2 | frameshift_deleti  on | exon21 | c.2298delA | p.G766fs | . | . |
| CTC4 | 10 | 94214206 | GT | G | 1 | NM_001165946 | IDE | frameshift_deleti  on | exon15 | c.1389delA | p.K463fs | . | . |
| CTC4 | 10 | 94834728 | GA | G | 1 | NM_000783 | CYP26A1 | frameshift_deleti  on | exon3 | c.608delA | p.D203fs | . | . |
| CTC4 | 10 | 95148853 | A | AT | 0.182 | NM_133337 | MYOF | frameshift_inserti  on | exon17 | c.1475dupA | p.N492fs | . | . |
| CTC4 | 10 | 95152688 | GC | G | 0.706 | NM_013451 | MYOF | frameshift_deleti  on | exon17 | c.1441delG | p.A481fs | . | . |
| CTC4 | 10 | 97096723 | GC | G | 1 | NM_001034954 | SORBS1 | frameshift_deleti  on | exon28 | c.3193delG | p.A1065fs | . | . |
| CTC4 | 10 | 98715468 | GA | G | 1 | NM_001170765 | LCOR | frameshift_deleti  on | exon8 | c.1092delA | p.G364fs | . | . |
| CTC4 | 10 | 98924595 | GC | G | 0.642 | NM_003061 | SLIT1 | frameshift_deleti  on | exon2 | c.249delG | p.G83fs | . | . |
| CTC4 | 10 | 99623715 | TG | T | 0.5 | NM_001010917 | GOLGA7B | frameshift_deleti  on | exon3 | c.168delG | p.V56fs | . | . |
| CTC4 | 10 | 100017883 | GC | G | 1 | NM_032211 | LOXL4 | frameshift_deleti  on | exon7 | c.959delG | p.G320fs | . | . |
| CTC4 | 10 | 101715334 | GT | G | 0.61 | NM_015221 | DNMBP | frameshift_deleti  on | exon4 | c.1896delA | p.K632fs | . | . |

| CTC4 | 10 | 101911906 | GT | G | 0.441 | NM_006459 | ERLIN1 | frameshift_deleti  on | exon11 | c.1028delA | p.N343fs | . | . |
| --- | --- | --- | --- | --- | --- | --- | --- | --- | --- | --- | --- | --- | --- |
| CTC4 | 10 | 104129685 | CG | C | 1 | NM_001199378 | GBF1 | frameshift_deleti  on | exon26 | c.3284delG | p.R1095fs | . | . |
| CTC4 | 10 | 105184742 | TG | T | 1 | NM_014976 | PDCD11 | frameshift_deleti  on | exon20 | c.2766delG | p.L922fs | . | . |
| CTC4 | 10 | 105932208 | AG | A | 0.267 | NM_025145 | WDR96 | stopgain | exon20 | c.2545delC | p.L849X | . | . |
| CTC4 | 10 | 115662300 | CA | C | 0.4 | NM_198514 | NHLRC2 | frameshift_deleti on | exon8 | c.1443delA | p.T481fs | . | . |
| CTC4 | 10 | 116731905 | TA | T | 0.704 | NM_139169 | TRUB1 | frameshift_deleti  on | exon6 | c.609delA | p.L203fs | . | . |
| CTC4 | 10 | 116931101 | C | CTT | 1 | NM_001276282 | ATRNL1 | frameshift_inserti  on | exon8 | c.1399_1400ins  TT | p.L467fs | . | . |
| CTC4 | 10 | 118231311 | TG | T | 0.976 | NM_001011709 | PNLIPRP3 | frameshift_deleti  on | exon10 | c.1093delG | p.G365fs | . | . |
| CTC4 | 10 | 118439228 | G | GT | 0.474 | NM_025015 | HSPA12A | frameshift_inserti  on | exon10 | c.1071dupA | p.L358fs | . | . |
| CTC4 | 10 | 121658050 | CT | C | 0.333 | NM_007190 | SEC23IP | frameshift_deleti  on | exon2 | c.276delT | p.P92fs | . | . |
| CTC4 | 10 | 124457832 | AT | A | 0.472 | NM_001010912 | C10orf120 | frameshift_deleti  on | exon3 | c.424delA | p.I142fs | . | . |
| CTC4 | 10 | 124922197 | AC | A | 0.241 | NM_001007793 | BUB3 | frameshift_deleti  on | exon7 | c.825delC | p.Y275fs | . | . |
| CTC4 | 10 | 125639805 | CT | C | 1 | NM_198148 | CPXM2 | frameshift_deleti  on | exon2 | c.324delA | p.K108fs | . | . |
| CTC4 | 10 | 126370348 | GA | G | 0.75 | NM_014661 | FAM53B | frameshift_deleti  on | exon4 | c.733delT | p.S245fs | . | . |
| CTC4 | 10 | 127413977 | CA | C | 0.55 | NM_001202438 | EDRF1 | frameshift_deleti  on | exon5 | c.602delA | p.Q201fs | . | . |
| CTC4 | 10 | 127512183 | TC | T | 0.833 | NM_016567 | BCCIP | frameshift_deleti  on | exon1 | c.58delC | p.P20fs | . | . |
| CTC4 | 10 | 128202466 | A | AT | 0.333 | NM_001004298 | C10orf90 | frameshift_inserti  on | exon2 | c.64_65insA | p.L22fs | . | . |
| CTC4 | 10 | 128936191 | AG | A | 0.523 | NM_001039762 | FAM196A | frameshift_deleti  on | exon6 | c.1349delC | p.P450fs | . | . |
| CTC4 | 10 | 134038003 | T | TA | 0.673 | NM_173575 | STK32C | stopgain | exon8 | c.900_901insT | p.N301_A302d  elinsX | . | . |
| CTC4 | 11 | 613806 | T | TC | 1 | NM_004031 | IRF7 | frameshift_inserti  on | exon6 | c.864_865insG | p.S289fs | . | . |
| CTC4 | 11 | 618690 | G | GC | 0.326 | NM_001171968 | CDHR5 | frameshift_inserti  on | exon13 | c.1850_1851ins  G | p.H617fs | . | . |
| CTC4 | 11 | 721938 | GA | G | 0.617 | NM_022772 | EPS8L2 | frameshift_deleti  on | exon11 | c.932delA | p.E311fs | . | . |
| CTC4 | 11 | 2444189 | GC | G | 0.49 | NM_014555 | TRPM5 | frameshift_deleti  on | exon1 | c.77delG | p.G26fs | . | . |
| CTC4 | 11 | 3849202 | C | CA | 0.36 | NM_001665 | RHOG | frameshift_inserti  on | exon2 | c.166dupT | p.W56fs | . | . |
| CTC4 | 11 | 4608821 | CA | C | 0.2 | NM_001005170 | OR52I2 | frameshift_deleti  on | exon1 | c.780delA | p.S260fs | . | . |
| CTC4 | 11 | 6592470 | A | AT | 0.211 | NM_144666 | DNHD1 | frameshift_inserti  on | exon42 | c.13728_13729i  nsT | p.V4576fs | . | . |
| CTC4 | 11 | 6807185 | GA | G | 0.643 | NM_001004489 | OR2AG1 | frameshift_deleti  on | exon1 | c.918delA | p.G306fs | . | . |
| CTC4 | 11 | 10647794 | TC | T | 0.802 | NM_001206881 | MRVI1 | frameshift_deleti  on | exon7 | c.141delG | p.G47fs | . | . |
| CTC4 | 11 | 11988601 | GA | G | 0.75 | NM_001018057 | DKK3 | frameshift_deleti  on | exon5 | c.572delT | p.V191fs | . | . |
| CTC4 | 11 | 12316344 | G | GCTCC  TC | 0.923 | NM_032867 | MICALCL | nonframeshift_in  sertion | exon3 | c.1366_1367ins  CTCCTC | p.A456delinsA  PP | . | . |
| CTC4 | 11 | 12316344 | G | GCTC | 0.923 | NM_032867 | MICALCL | nonframeshift_in  sertion | exon3 | c.1366_1367ins  CTC | p.A456delinsA  P | . | . |
| CTC4 | 11 | 17533518 | C | CT | 0.301 | NM_153676 | USH1C | frameshift_inserti  on | exon16 | c.1343dupA | p.K448fs | . | . |
| CTC4 | 11 | 18111033 | TCC | T | 0.4 | NM_138421 | SAAL1 | frameshift_deleti  on | exon7 | c.612_613del | p.G204fs | . | . |
| CTC4 | 11 | 18127558 | C | CCGG | 0.988 | NM_138421 | SAAL1 | nonframeshift_in  sertion | exon1 | c.30_31insCCG | p.G11delinsPG | . | . |
| CTC4 | 11 | 20948887 | GA | G | 1 | NM_001288714 | NELL1 | frameshift_deleti  on | exon7 | c.623delA | p.E208fs | . | . |
| CTC4 | 11 | 28311830 | C | CT | 1 | NM_001113528 | METTL15 | frameshift_inserti  on | exon5 | c.486dupT | p.T162fs | . | . |
| CTC4 | 11 | 33308610 | GA | G | 0.909 | NM_001048200 | HIPK3 | frameshift_deleti  on | exon2 | c.651delA | p.R217fs | . | . |
| CTC4 | 11 | 33374762 | AC | A | 0.692 | NM_001048200 | HIPK3 | frameshift_deleti  on | exon16 | c.3234delC | p.H1078fs | . | . |
| CTC4 | 11 | 34910321 | GA | G | 1 | NM_015957 | APIP | frameshift_deleti  on | exon4 | c.302delT | p.F101fs | . | . |
| CTC4 | 11 | 36594926 | AT | A | 0.75 | NM_000448 | RAG1 | frameshift_deleti  on | exon2 | c.73delT | p.F25fs | . | . |
| CTC4 | 11 | 46387872 | GC | G | 0.269 | NM_001105540 | DGKZ | frameshift_deleti  on | exon2 | c.67delC | p.P23fs | . | . |
| CTC4 | 11 | 46393271 | CAAG | C | 0.113 | NM_001199268 | DGKZ | nonframeshift_de  letion | exon8 | c.709_711del | p.237_237del | . | . |
| CTC4 | 11 | 46419275 | GC | G | 0.257 | NM_001267783 | AMBRA1 | frameshift_deleti  on | exon18 | c.3264delG | p.Q1088fs | . | . |
| CTC4 | 11 | 46783667 | TA | T | 1 | NM_001008938 | CKAP5 | stopgain | exon32 | c.4103delT | p.L1368X | . | . |
| CTC4 | 11 | 47510521 | TA | T | 1 | NM_001025596 | CELF1 | frameshift_deleti on | exon1 | c.45delT | p.A15fs | . | . |
| CTC4 | 11 | 48161074 | TC | T | 0.259 | NM_002843 | PTPRJ | frameshift_deleti  on | exon11 | c.2190delC | p.V730fs | . | . |
| CTC4 | 11 | 48511173 | A | AAC | 0.333 | NM_001005512 | OR4A47 | frameshift_inserti  on | exon1 | c.829_830insAC | p.T277fs | . | . |
| CTC4 | 11 | 55433079 | CT | C | 0.231 | NM_001004704 | OR4C6 | frameshift_deleti  on | exon1 | c.438delT | p.A146fs | . | . |
| CTC4 | 11 | 55607153 | CA | C | 1 | NM_001005496 | OR5D16 | frameshift_deleti  on | exon1 | c.927delA | p.T309fs | . | . |
| CTC4 | 11 | 55681419 | CT | C | 0.75 | NM_001001960 | OR5W2 | frameshift_deleti  on | exon1 | c.639delA | p.G213fs | . | . |
| CTC4 | 11 | 56344991 | T | TA | 0.61 | NM_001004741 | OR5M10 | frameshift_inserti  on | exon1 | c.206dupT | p.V69fs | . | . |
| CTC4 | 11 | 57193540 | CA | C | 0.242 | NM_001278201 | SLC43A3 | frameshift_deleti  on | exon3 | c.105delT | p.F35fs | . | . |
| CTC4 | 11 | 57947416 | CCTT | C | 0.123 | NM_001005212 | OR9Q1 | nonframeshift_de  letion | exon3 | c.501_503del | p.167_168del | . | . |
| CTC4 | 11 | 58035274 | GA | G | 0.286 | NM_207374 | OR10W1 | frameshift_deleti  on | exon1 | c.56delT | p.F19fs | . | . |
| CTC4 | 11 | 58978896 | GC | G | 1 | NM_001039396 | MPEG1 | frameshift_deleti  on | exon1 | c.1442delG | p.G481fs | . | . |
| CTC4 | 11 | 59245238 | AT | A | 0.8 | NM_001004705 | OR4D10 | frameshift_deleti  on | exon1 | c.337delT | p.F113fs | . | . |
| CTC4 | 11 | 60183251 | A | AT | 0.541 | NM_001079692 | MS4A14 | frameshift_inserti  on | exon4 | c.760dupT | p.T253fs | . | . |

| CTC4 | 11 | 60785399 | AC | A | 0.385 | NM_001254750 | CD6 | frameshift_deleti  on | exon10 | c.1656delC | p.N552fs | . | . |
| --- | --- | --- | --- | --- | --- | --- | --- | --- | --- | --- | --- | --- | --- |
| CTC4 | 11 | 61026606 | TA | T | 0.959 | NM_152718 | VWCE | stopgain | exon20 | c.2408delT | p.L803X | . | . |
| CTC4 | 11 | 61558000 | CA | C | 0.347 | NM_014206 | TMEM258 | frameshift_deleti on | exon2 | c.77delT | p.L26fs | . | . |
| CTC4 | 11 | 61558019 | TG | T | 0.349 | NM_014206 | TMEM258 | frameshift_deleti  on | exon2 | c.58delC | p.H20fs | . | . |
| CTC4 | 11 | 61644404 | CG | C | 1 | NM_021727 | FADS3 | frameshift_deleti  on | exon8 | c.916delC | p.R306fs | . | . |
| CTC4 | 11 | 61729769 | CGAG | C | 0.146 | NM_001139443 | BEST1 | nonframeshift_de  letion | exon9 | c.964_966del | p.322_322del | . | . |
| CTC4 | 11 | 62287807 | A | AT | 0.189 | NM_001620 | AHNAK | frameshift_inserti  on | exon5 | c.14081dupA | p.D4694fs | . | . |
| CTC4 | 11 | 62287934 | AT | A | 0.978 | NM_001620 | AHNAK | frameshift_deleti  on | exon5 | c.13954delA | p.M4652fs | . | . |
| CTC4 | 11 | 62288376 | GA | G | 0.214 | NM_001620 | AHNAK | frameshift_deleti  on | exon5 | c.13512delT | p.G4504fs | . | . |
| CTC4 | 11 | 62288661 | CT | C | 0.416 | NM_001620 | AHNAK | frameshift_deleti  on | exon5 | c.13227delA | p.K4409fs | . | . |
| CTC4 | 11 | 62367690 | GA | G | 0.366 | NM_004739 | MTA2 | frameshift_deleti  on | exon3 | c.137delT | p.F46fs | . | . |
| CTC4 | 11 | 62417274 | TC | T | 0.429 | NM_030628 | INTS5 | frameshift_deleti  on | exon2 | c.277delG | p.D93fs | . | . |
| CTC4 | 11 | 62848437 | GT | G | 1 | NM_001136506 | SLC22A24 | frameshift_deleti  on | exon9 | c.1552delA | p.T518fs | . | . |
| CTC4 | 11 | 64138764 | CG | C | 0.86 | NM_001006944 | RPS6KA4 | frameshift_deleti  on | exon17 | c.2114delG | p.R705fs | . | . |
| CTC4 | 11 | 64514205 | C | CA | 0.69 | NM_001164716 | PYGM | frameshift_inserti  on | exon18 | c.2190dupT | p.A731fs | . | . |
| CTC4 | 11 | 64863727 | CG | C | 1 | NM_013265 | VPS51 | frameshift_deleti  on | exon1 | c.6delG | p.A2fs | . | . |
| CTC4 | 11 | 65349054 | CT | C | 0.75 | NM_001099409 | EHBP1L1 | frameshift_deleti  on | exon9 | c.912delT | p.P304fs | . | . |
| CTC4 | 11 | 65685056 | GT | G | 0.164 | NM_001135635 | C11orf68 | frameshift_deleti  on | exon2 | c.755delA | p.Y252fs | . | . |
| CTC4 | 11 | 65820549 | GC | G | 0.25 | NM_006842 | SF3B2 | frameshift_deleti  on | exon3 | c.233delC | p.A78fs | . | . |
| CTC4 | 11 | 67012811 | GA | G | 0.514 | NM_001256405 | KDM2A | frameshift_deleti  on | exon3 | c.399delA | p.R133fs | . | . |
| CTC4 | 11 | 67814996 | TC | T | 0.4 | NM_006053 | TCIRG1 | frameshift_deleti  on | exon6 | c.615delC | p.V205fs | . | . |
| CTC4 | 11 | 67838280 | AG | A | 0.25 | NM_212469 | CHKA | frameshift_deleti  on | exon4 | c.620delC | p.S207fs | . | . |
| CTC4 | 11 | 68458418 | CCG | C | 0.667 | NM_015973 | GAL | frameshift_deleti  on | exon6 | c.336_337del | p.P112fs | . | . |
| CTC4 | 11 | 68458419 | CG | C | 0.875 | NM_015973 | GAL | frameshift_deleti  on | exon6 | c.337delG | p.A113fs | . | . |
| CTC4 | 11 | 68772960 | TC | T | 1 | NM_001098515 | MRGPRF | frameshift_deleti  on | exon3 | c.817delG | p.E273fs | . | . |
| CTC4 | 11 | 71152483 | AC | A | 0.964 | NM_001163817 | DHCR7 | frameshift_deleti  on | exon6 | c.415delG | p.V139fs | . | . |
| CTC4 | 11 | 72410564 | CCGT | C | 0.556 | NM_001135190 | ARAP1 | nonframeshift_de  letion | exon14 | c.1415_1417del | p.472_473del | . | . |
| CTC4 | 11 | 74053595 | CT | C | 0.569 | NM_173582 | PGM2L1 | frameshift_deleti  on | exon12 | c.1542delA | p.K514fs | . | . |
| CTC4 | 11 | 75439877 | AT | A | 0.231 | NM_025098 | MOGAT2 | frameshift_deleti  on | exon5 | c.694delT | p.F232fs | . | . |
| CTC4 | 11 | 76954788 | T | TA | 1 | NM_182833 | GDPD4 | frameshift_inserti  on | exon12 | c.1191dupT | p.I398fs | . | . |
| CTC4 | 11 | 78369485 | TTC | T | 0.132 | NM_001098816 | TENM4 | frameshift_deleti  on | exon34 | c.7926_7927del | p.E2642fs | . | . |
| CTC4 | 11 | 82879788 | TG | T | 0.159 | NM_015885 | PCF11 | frameshift_deleti  on | exon8 | c.2412delG | p.L804fs | . | . |
| CTC4 | 11 | 86125852 | AT | A | 0.545 | NM_021827 | CCDC81 | frameshift_deleti  on | exon11 | c.1144delT | p.F382fs | . | . |
| CTC4 | 11 | 88027568 | AT | A | 0.271 | NM_001814 | CTSC | stopgain | exon7 | c.997delA | p.M333X | . | . |
| CTC4 | 11 | 88300247 | TC | T | 1 | NM_001143831 | GRM5 | frameshift_deleti on | exon7 | c.2603delG | p.R868fs | . | . |
| CTC4 | 11 | 92532387 | GT | G | 0.192 | NM_001008781 | FAT3 | frameshift_deleti  on | exon9 | c.6209delT | p.V2070fs | . | . |
| CTC4 | 11 | 92568082 | GT | G | 0.492 | NM_001008781 | FAT3 | frameshift_deleti  on | exon14 | c.9919delT | p.F3307fs | . | . |
| CTC4 | 11 | 93471460 | TA | T | 0.298 | NM_024116 | TAF1D | stopgain | exon3 | c.273delT | p.Y91X | . | . |
| CTC4 | 11 | 94533006 | GT | G | 0.6 | NM_130847 | AMOTL1 | frameshift_deleti on | exon3 | c.651delT | p.G217fs | . | . |
| CTC4 | 11 | 94759865 | CCCA | C | 1 | NM_001161630 | KDM4E | nonframeshift_de  letion | exon1 | c.1145_1147del | p.382_383del | . | . |
| CTC4 | 11 | 102713486 | C | CT | 1 | NM_002422 | MMP3 | frameshift_inserti  on | exon2 | c.266dupA | p.K89fs | . | . |
| CTC4 | 11 | 105924673 | TC | T | 1 | NM_152433 | KBTBD3 | frameshift_deleti  on | exon3 | c.742delG | p.E248fs | . | . |
| CTC4 | 11 | 107427492 | CT | C | 1 | NM_138775 | ALKBH8 | frameshift_deleti  on | exon3 | c.366delA | p.K122fs | . | . |
| CTC4 | 11 | 107501445 | TA | T | 0.571 | NM_001130037 | ELMOD1 | frameshift_deleti  on | exon4 | c.184delA | p.K62fs | . | . |
| CTC4 | 11 | 108160450 | TA | T | 0.286 | NM_000051 | ATM | frameshift_deleti  on | exon29 | c.4359delA | p.I1453fs | . | . |
| CTC4 | 11 | 108546414 | C | CA | 0.622 | NM_004398 | DDX10 | frameshift_inserti  on | exon3 | c.340dupA | p.A113fs | . | . |
| CTC4 | 11 | 111941840 | CT | C | 0.452 | NM_001082619 | PIH1D2 | frameshift_deleti  on | exon4 | c.468delA | p.K156fs | . | . |
| CTC4 | 11 | 113607397 | GC | G | 0.525 | NM_004724 | ZW10 | frameshift_deleti  on | exon15 | c.2163delG | p.M721fs | . | . |
| CTC4 | 11 | 115080311 | ATGG | A | 0.297 | NM_014333 | CADM1 | nonframeshift_de  letion | exon8 | c.1058_1060del | p.353_354del | . | . |
| CTC4 | 11 | 116706673 | TG | T | 1 | NM_000039 | APOA1 | frameshift_deleti  on | exon4 | c.654delC | p.A218fs | . | . |
| CTC4 | 11 | 118253374 | AC | A | 0.329 | NM_001204077 | UBE4A | frameshift_deleti  on | exon13 | c.2081delC | p.T694fs | . | . |
| CTC4 | 11 | 121016419 | GC | G | 0.206 | NM_005422 | TECTA | frameshift_deleti  on | exon11 | c.3700delC | p.Q1234fs | . | . |
| CTC4 | 11 | 122775057 | GA | G | 1 | NM_024806 | C11orf63 | frameshift_deleti  on | exon3 | c.770delA | p.E257fs | . | . |
| CTC4 | 11 | 123597317 | CT | C | 1 | NM_003455 | ZNF202 | frameshift_deleti  on | exon9 | c.1334delA | p.K445fs | . | . |
| CTC4 | 11 | 123624955 | CA | C | 1 | NM_001005188 | OR6X1 | frameshift_deleti  on | exon1 | c.271delT | p.C91fs | . | . |
| CTC4 | 11 | 124135436 | TG | T | 0.609 | NM_001005198 | OR8G5 | frameshift_deleti  on | exon1 | c.715delG | p.G239fs | . | . |
| CTC4 | 11 | 124252906 | A | AT | 0.553 | NM_001005468 | OR8B2 | frameshift_inserti  on | exon1 | c.333dupA | p.C112fs | . | . |
| CTC4 | 11 | 124440206 | GC | G | 0.879 | NM_001005194 | OR8A1 | frameshift_deleti  on | exon1 | c.243delC | p.S81fs | . | . |

| CTC4 | 11 | 124508478 | T | TG | 0.364 | NM_170601 | SIAE | frameshift_inserti  on | exon9 | c.1279dupC | p.Q427fs | . | . |
| --- | --- | --- | --- | --- | --- | --- | --- | --- | --- | --- | --- | --- | --- |
| CTC4 | 11 | 124951784 | TG | T | 0.269 | NM_001145290 | SLC37A2 | frameshift_deleti  on | exon9 | c.868delG | p.G290fs | . | . |
| CTC4 | 11 | 125776098 | TG | T | 1 | NM_013264 | DDX25 | frameshift_deleti  on | exon4 | c.306delG | p.L102fs | . | . |
| CTC4 | 11 | 125853936 | AG | A | 0.411 | NM_001243597 | CDON | frameshift_deleti  on | exon16 | c.2825delC | p.P942fs | . | . |
| CTC4 | 11 | 125853937 | G | GT | 0.255 | NM_001243597 | CDON | frameshift_inserti  on | exon16 | c.2824_2825ins  A | p.P942fs | . | . |
| CTC4 | 11 | 125880349 | GC | G | 0.471 | NM_001243597 | CDON | frameshift_deleti  on | exon8 | c.1438delG | p.A480fs | . | . |
| CTC4 | 11 | 130275722 | T | TA | 0.6 | NM_007037 | ADAMTS8 | stopgain | exon9 | c.2400_2401ins  T | p.K801_V802de  linsX | . | . |
| CTC4 | 12 | 662753 | GC | G | 0.545 | NM_173593 | B4GALNT3 | frameshift_deleti  on | exon14 | c.1665delC | p.S555fs | . | . |
| CTC4 | 12 | 1036353 | GC | G | 0.967 | NM_134424 | RAD52 | frameshift_deleti  on | exon6 | c.424delG | p.A142fs | . | . |
| CTC4 | 12 | 1996194 | CA | C | 0.308 | NM_172364 | CACNA2D4 | frameshift_deleti  on | exon7 | c.822delT | p.F274fs | . | . |
| CTC4 | 12 | 3129916 | GAGC | G | 0.167 | NM_201443 | TEAD4 | nonframeshift_de  letion | exon7 | c.314_316del | p.105_106del | . | . |
| CTC4 | 12 | 4614476 | TA | T | 0.643 | NM_020374 | C12orf4 | frameshift_deleti  on | exon10 | c.1232delT | p.L411fs | . | . |
| CTC4 | 12 | 6092389 | TG | T | 0.489 | NM_000552 | VWF | frameshift_deleti  on | exon41 | c.7007delC | p.P2336fs | . | . |
| CTC4 | 12 | 6499982 | CT | C | 0.852 | NM_001270987 | LTBR | frameshift_deleti  on | exon10 | c.1131delT | p.P377fs | . | . |
| CTC4 | 12 | 6710686 | C | CA | 0.333 | NM_001273 | CHD4 | frameshift_inserti  on | exon6 | c.567dupT | p.A190fs | . | . |
| CTC4 | 12 | 6981501 | GC | G | 0.75 | NM_001146316 | SPSB2 | frameshift_deleti  on | exon2 | c.564delG | p.G188fs | . | . |
| CTC4 | 12 | 7023137 | G | GT | 0.533 | NM_006992 | LRRC23 | frameshift_inserti  on | exon7 | c.842dupT | p.V281fs | . | . |
| CTC4 | 12 | 7528399 | CT | C | 0.388 | NM_174941 | CD163L1 | frameshift_deleti  on | exon10 | c.2582delA | p.K861fs | . | . |
| CTC4 | 12 | 7655143 | CA | C | 0.534 | NM_004244 | CD163 | frameshift_deleti  on | exon2 | c.63delT | p.F21fs | . | . |
| CTC4 | 12 | 9247631 | AT | A | 0.579 | NM_000014 | A2M | frameshift_deleti  on | exon17 | c.2062delA | p.M688fs | . | . |
| CTC4 | 12 | 12630877 | CT | C | 0.269 | NM_030640 | DUSP16 | frameshift_deleti  on | exon7 | c.887delA | p.K296fs | . | . |
| CTC4 | 12 | 13061332 | CG | C | 0.684 | NM_003979 | GPRC5A | frameshift_deleti  on | exon2 | c.150delG | p.P50fs | . | . |
| CTC4 | 12 | 14650842 | TG | T | 0.469 | NM_001286514 | ATF7IP | frameshift_deleti  on | exon15 | c.3646delG | p.G1216fs | . | . |
| CTC4 | 12 | 15652463 | CT | C | 0.968 | NM_002848 | PTPRO | frameshift_deleti  on | exon4 | c.597delT | p.T199fs | . | . |
| CTC4 | 12 | 20852592 | G | GA | 0.234 | NM_017435 | SLCO1C1 | frameshift_inserti  on | exon2 | c.83dupA | p.E28fs | . | . |
| CTC4 | 12 | 21172257 | GT | G | 1 | NM_001009562 | SLCO1B7 | frameshift_deleti  on | exon2 | c.162delT | p.C54fs | . | . |
| CTC4 | 12 | 21958199 | GA | G | 0.562 | NM_005691 | ABCC9 | frameshift_deleti  on | exon38 | c.4558delT | p.S1520fs | . | . |
| CTC4 | 12 | 27472353 | TA | T | 0.226 | NM_015000 | STK38L | frameshift_deleti  on | exon12 | c.1173delA | p.I391fs | . | . |
| CTC4 | 12 | 32793372 | CT | C | 1 | NM_139241 | FGD4 | frameshift_deleti  on | exon17 | c.2207delT | p.L736fs | . | . |
| CTC4 | 12 | 43886324 | CA | C | 1 | NM_025003 | ADAMTS20 | frameshift_deleti  on | exon6 | c.1059delT | p.T353fs | . | . |
| CTC4 | 12 | 46321762 | A | AT | 0.781 | NM_004719 | SCAF11 | frameshift_inserti  on | exon11 | c.1721dupA | p.N574fs | . | . |
| CTC4 | 12 | 48528580 | TGG | T | 0.923 | NM_001166688 | PFKM | frameshift_deleti  on | exon7 | c.716_717del | p.W239fs | . | . |
| CTC4 | 12 | 48597027 | GA | G | 0.357 | NM_001004134 | OR10AD1 | frameshift_deleti  on | exon1 | c.48delT | p.F16fs | . | . |
| CTC4 | 12 | 49218461 | GA | G | 0.202 | NM_001206915 | CACNB3 | frameshift_deleti  on | exon4 | c.295delA | p.N99fs | . | . |
| CTC4 | 12 | 49445474 | TG | T | 0.434 | NM_003482 | KMT2D | frameshift_deleti  on | exon10 | c.1991delC | p.P664fs | . | . |
| CTC4 | 12 | 49445895 | T | TC | 0.633 | NM_003482 | KMT2D | frameshift_inserti  on | exon10 | c.1570dupG | p.E524fs | . | . |
| CTC4 | 12 | 51318928 | T | TC | 0.535 | NM_014033 | METTL7A | frameshift_inserti  on | exon1 | c.108dupC | p.F36fs | . | . |
| CTC4 | 12 | 51398581 | TG | T | 1 | NM_001174130 | SLC11A2 | frameshift_deleti  on | exon4 | c.406delC | p.Q136fs | . | . |
| CTC4 | 12 | 51639908 | G | GA | 0.438 | NM_001136269 | DAZAP2 | frameshift_inserti  on | exon4 | c.443dupA | p.E148fs | . | . |
| CTC4 | 12 | 52056653 | AC | A | 1 | NM_001177984 | SCN8A | frameshift_deleti  on | exon2 | c.53delC | p.T18fs | . | . |
| CTC4 | 12 | 52642498 | A | AT | 0.233 | NM_005556 | KRT7 | frameshift_inserti  on | exon9 | c.1365dupT | p.Y455fs | . | . |
| CTC4 | 12 | 52962134 | G | GA | 0.952 | NM_175053 | KRT74 | frameshift_inserti  on | exon7 | c.1173_1174ins  T | p.R392fs | . | . |
| CTC4 | 12 | 53679715 | TG | T | 1 | NM_012291 | ESPL1 | frameshift_deleti  on | exon18 | c.3196delG | p.G1066fs | . | . |
| CTC4 | 12 | 55356734 | C | CA | 0.292 | NM_014796 | TESPA1 | frameshift_inserti  on | exon7 | c.533dupT | p.V178fs | . | . |
| CTC4 | 12 | 55641675 | AT | A | 0.28 | NM_001005490 | OR6C74 | frameshift_deleti  on | exon1 | c.605delT | p.I202fs | . | . |
| CTC4 | 12 | 55968207 | TA | T | 0.589 | NM_001258285 | OR2AP1 | frameshift_deleti  on | exon1 | c.10delA | p.K4fs | . | . |
| CTC4 | 12 | 56221472 | AG | A | 0.16 | NM_032364 | DNAJC14 | frameshift_deleti  on | exon2 | c.970delC | p.L324fs | . | . |
| CTC4 | 12 | 56578660 | CT | C | 0.136 | NM_001130420 | SMARCC2 | frameshift_deleti  on | exon5 | c.459delA | p.L153fs | . | . |
| CTC4 | 12 | 57114975 | TG | T | 0.927 | NM_001113203 | NACA | frameshift_deleti  on | exon3 | c.338delC | p.P113fs | . | . |
| CTC4 | 12 | 57389671 | C | CT | 0.818 | NM_007264 | GPR182 | frameshift_inserti  on | exon2 | c.679dupT | p.G226fs | . | . |
| CTC4 | 12 | 57464516 | GT | G | 0.682 | NM_001130963 | TMEM194A | frameshift_deleti  on | exon3 | c.412delA | p.T138fs | . | . |
| CTC4 | 12 | 57498270 | C | CA | 0.381 | NM_001178081 | STAT6 | frameshift_inserti  on | exon9 | c.858_859insT | p.G287fs | . | . |
| CTC4 | 12 | 57501055 | T | TG | 0.547 | NM_001178078 | STAT6 | frameshift_inserti  on | exon4 | c.297dupC | p.R100fs | . | . |
| CTC4 | 12 | 57502026 | TG | T | 0.929 | NM_001178078 | STAT6 | frameshift_deleti  on | exon2 | c.35delC | p.P12fs | . | . |
| CTC4 | 12 | 57674204 | TTGC | T | 0.158 | NM_014925 | R3HDM2 | nonframeshift_de  letion | exon12 | c.1236_1238del | p.412_413del | . | . |
| CTC4 | 12 | 57927805 | TG | T | 0.889 | NM_001261412 | DCTN2 | frameshift_deleti  on | exon7 | c.605delC | p.P202fs | . | . |
| CTC4 | 12 | 58200234 | GC | G | 0.5 | NM_006576 | AVIL | frameshift_deleti  on | exon13 | c.1579delG | p.A527fs | . | . |
| CTC4 | 12 | 58350554 | GT | G | 0.863 | NM_033276 | XRCC6BP1 | frameshift_deleti  on | exon6 | c.623delT | p.V208fs | . | . |

| CTC4 | 12 | 60168614 | AT | A | 0.417 | NM_004731 | SLC16A7 | frameshift_deleti  on | exon4 | c.539delT | p.I180fs | . | . |
| --- | --- | --- | --- | --- | --- | --- | --- | --- | --- | --- | --- | --- | --- |
| CTC4 | 12 | 64712546 | A | AGTT | 0.682 | NM_001170633 | C12orf56 | nonframeshift_in  sertion | exon4 | c.702_703insAA  C | p.S235delinsNS | . | . |
| CTC4 | 12 | 64819171 | CT | C | 0.206 | NM_007235 | XPOT | frameshift_deleti  on | exon14 | c.1507delT | p.F503fs | . | . |
| CTC4 | 12 | 69964129 | GA | G | 0.724 | NM_001278357 | FRS2 | frameshift_deleti  on | exon4 | c.86delA | p.D29fs | . | . |
| CTC4 | 12 | 75897791 | ATC | A | 0.233 | NM_007043 | KRR1 | frameshift_deleti  on | exon7 | c.722_723del | p.R241fs | . | . |
| CTC4 | 12 | 78552999 | CT | C | 1 | NM_001024383 | NAV3 | frameshift_deleti  on | exon23 | c.4803delT | p.A1601fs | . | . |
| CTC4 | 12 | 80651647 | TA | T | 0.333 | NM_173591 | OTOGL | frameshift_deleti  on | exon17 | c.1728delA | p.L576fs | . | . |
| CTC4 | 12 | 81568682 | C | CT | 0.28 | NM_024560 | ACSS3 | frameshift_inserti  on | exon8 | c.1215dupT | p.P405fs | . | . |
| CTC4 | 12 | 85450034 | C | CA | 0.965 | NM_001079910 | LRRIQ1 | frameshift_inserti  on | exon8 | c.1464dupA | p.A488fs | . | . |
| CTC4 | 12 | 88439469 | CT | C | 0.352 | NM_001009894 | C12orf29 | frameshift_deleti  on | exon5 | c.533delT | p.L178fs | . | . |
| CTC4 | 12 | 89997017 | C | CA | 0.958 | NM_001001323 | ATP2B1 | stopgain | exon17 | c.2862dupT | p.D955_I956del  insX | . | . |
| CTC4 | 12 | 99007587 | CA | C | 0.347 | NM_201612 | IKBIP | frameshift_deleti  on | exon3 | c.828delT | p.F276fs | . | . |
| CTC4 | 12 | 99145184 | GT | G | 0.462 | NM_001204080 | ANKS1B | frameshift_deleti  on | exon9 | c.638delA | p.N213fs | . | . |
| CTC4 | 12 | 100995519 | TG | T | 0.619 | NM_174942 | GAS2L3 | frameshift_deleti  on | exon5 | c.292delG | p.E98fs | . | . |
| CTC4 | 12 | 105198971 | GA | G | 0.278 | NM_032148 | SLC41A2 | frameshift_deleti  on | exon10 | c.1680delT | p.F560fs | . | . |
| CTC4 | 12 | 109017650 | GGAGTG  GTCTGT GCCTCC GTGGGC | G | 1 | NM_001206609 | SELPLG | nonframeshift_de letion | exon2 | c.452_481del | p.151_161del | . | . |
| CTC4 | 12 | 109283287 | AC | A | 0.829 | NM_001917 | DAO | frameshift_deleti  on | exon4 | c.353delC | p.T118fs | . | . |
| CTC4 | 12 | 109536275 | GC | G | 0.964 | NM_003362 | UNG | frameshift_deleti  on | exon1 | c.145delC | p.P49fs | . | . |
| CTC4 | 12 | 109959337 | TG | T | 1 | NM_130466 | UBE3B | frameshift_deleti  on | exon21 | c.2346delG | p.L782fs | . | . |
| CTC4 | 12 | 110246257 | GGC | G | 0.72 | NM_001177428 | TRPV4 | frameshift_deleti  on | exon2 | c.401_402del | p.S134fs | . | . |
| CTC4 | 12 | 110764229 | C | CG | 1 | NM_001681 | ATP2A2 | frameshift_inserti  on | exon7 | c.580dupG | p.P193fs | . | . |
| CTC4 | 12 | 110834221 | CA | C | 1 | NM_001137664 | ANAPC7 | frameshift_deleti  on | exon2 | c.239delT | p.L80fs | . | . |
| CTC4 | 12 | 111800826 | TGCCAC  CCCC | T | 1 | NM_001177997 | FAM109A | nonframeshift_de  letion | exon3 | c.397_405del | p.133_135del | . | . |
| CTC4 | 12 | 113385796 | TG | T | 0.989 | NM_006187 | OAS3 | frameshift_deleti  on | exon5 | c.922delG | p.G308fs | . | . |
| CTC4 | 12 | 117657977 | GT | G | 1 | NM_001204213 | NOS1 | frameshift_deleti  on | exon26 | c.3064delA | p.T1022fs | . | . |
| CTC4 | 12 | 120595664 | GC | G | 0.571 | NM_006836 | GCN1L1 | frameshift_deleti  on | exon26 | c.3075delG | p.G1025fs | . | . |
| CTC4 | 12 | 120595667 | CG | C | 1 | NM_006836 | GCN1L1 | frameshift_deleti  on | exon26 | c.3072delC | p.P1024fs | . | . |
| CTC4 | 12 | 120800887 | TG | T | 1 | NM_002442 | MSI1 | frameshift_deleti  on | exon6 | c.360delC | p.T120fs | . | . |
| CTC4 | 12 | 121678327 | C | CTTT | 1 | NM_001270486 | CAMKK2 | nonframeshift_in  sertion | exon16 | c.1614_1615ins  AAA | p.G539delinsK  G | . | . |
| CTC4 | 12 | 121678327 | C | CTTTT | 1 | NM_001270486 | CAMKK2 | frameshift_inserti  on | exon16 | c.1614_1615ins  AAAA | p.G539fs | . | . |
| CTC4 | 12 | 123290739 | CA | C | 1 | NM_201435 | CCDC62 | frameshift_deleti  on | exon10 | c.1749delA | p.S583fs | . | . |
| CTC4 | 12 | 123482022 | TC | T | 1 | NM_020845 | PITPNM2 | frameshift_deleti  on | exon10 | c.1321delG | p.D441fs | . | . |
| CTC4 | 12 | 124421798 | AT | A | 0.205 | NM_025140 | CCDC92 | frameshift_deleti  on | exon5 | c.802delA | p.I268fs | . | . |
| CTC4 | 12 | 132466961 | CT | C | 1 | NM_015409 | EP400 | frameshift_deleti  on | exon5 | c.1868delT | p.L623fs | . | . |
| CTC4 | 13 | 24864969 | CA | C | 0.265 | NM_001286793 | SPATA13 | frameshift_deleti  on | exon5 | c.733delA | p.K245fs | . | . |
| CTC4 | 13 | 25274919 | TC | T | 1 | NM_001185085 | ATP12A | frameshift_deleti  on | exon13 | c.1759delC | p.P587fs | . | . |
| CTC4 | 13 | 25444866 | AT | A | 0.417 | NM_001184993 | RNF17 | frameshift_deleti  on | exon32 | c.4425delT | p.D1475fs | . | . |
| CTC4 | 13 | 28009164 | C | CA | 0.34 | NM_002097 | GTF3A | frameshift_inserti  on | exon7 | c.867dupA | p.A289fs | . | . |
| CTC4 | 13 | 28893622 | AT | A | 0.909 | NM_002019 | FLT1 | frameshift_deleti  on | exon24 | c.3223delA | p.I1075fs | . | . |
| CTC4 | 13 | 30062082 | GA | G | 1 | NM_015233 | MTUS2 | frameshift_deleti  on | exon4 | c.383delA | p.E128fs | . | . |
| CTC4 | 13 | 41936290 | GA | G | 1 | NM_024561 | NAA16 | frameshift_deleti  on | exon13 | c.1535delA | p.E512fs | . | . |
| CTC4 | 13 | 42266011 | CT | C | 1 | NM_015058 | VWA8 | frameshift_deleti  on | exon31 | c.3738delA | p.K1246fs | . | . |
| CTC4 | 13 | 45723920 | T | TG | 0.8 | NM_004128 | GTF2F2 | frameshift_inserti  on | exon3 | c.157dupG | p.T52fs | . | . |
| CTC4 | 13 | 50108287 | CT | C | 0.227 | NM_018191 | RCBTB1 | frameshift_deleti  on | exon13 | c.1566delA | p.K522fs | . | . |
| CTC4 | 13 | 66878858 | G | GA | 0.583 | NM_020403 | PCDH9 | frameshift_inserti  on | exon4 | c.3540dupT | p.P1181fs | . | . |
| CTC4 | 13 | 76111803 | GT | G | 0.714 | NM_203495 | COMMD6 | frameshift_deleti  on | exon2 | c.50delA | p.N17fs | . | . |
| CTC4 | 13 | 98658524 | TCA | T | 0.375 | NM_002271 | IPO5 | frameshift_deleti  on | exon17 | c.1693_1694del | p.Q565fs | . | . |
| CTC4 | 13 | 98658525 | CA | C | 1 | NM_002271 | IPO5 | frameshift_deleti  on | exon17 | c.1694delA | p.Q565fs | . | . |
| CTC4 | 13 | 103381933 | AG | A | 0.379 | NM_001146197 | CCDC168 | frameshift_deleti  on | exon4 | c.21113delC | p.P7038fs | . | . |
| CTC4 | 13 | 108881840 | A | AG | 0.682 | NM_032859 | ABHD13 | frameshift_inserti  on | exon2 | c.275dupG | p.R92fs | . | . |
| CTC4 | 13 | 109365042 | A | AG | 0.25 | NM_001198950 | MYO16 | frameshift_inserti  on | exon3 | c.326_327insG | p.H109fs | . | . |
| CTC4 | 13 | 111885622 | CA | C | 0.641 | NM_001113512 | ARHGEF7 | frameshift_deleti  on | exon5 | c.655delA | p.K219fs | . | . |
| CTC4 | 13 | 113760172 | T | TA | 0.444 | NM_000131 | F7 | frameshift_inserti  on | exon1 | c.17_18insA | p.L6fs | . | . |
| CTC4 | 13 | 113839835 | C | CA | 0.312 | NM_001127202 | PCID2 | frameshift_inserti  on | exon8 | c.506dupT | p.L169fs | . | . |
| CTC4 | 13 | 114622559 | TG | T | 0.8 | NM_001278674 | LINC00452 | frameshift_deleti  on | exon6 | c.466delG | p.G156fs | . | . |
| CTC4 | 14 | 20181604 | TG | T | 0.2 | NM_001197287 | OR11H2 | frameshift_deleti  on | exon2 | c.471delC | p.A157fs | . | . |

| CTC4 | 14 | 21560752 | GGAGGC  T | G | 1 | NM_001101672 | ZNF219 | nonframeshift_de  letion | exon3 | c.698_703del | p.233_235del | . | . |
| --- | --- | --- | --- | --- | --- | --- | --- | --- | --- | --- | --- | --- | --- |
| CTC4 | 14 | 21756215 | CA | C | 0.5 | NM_020366 | RPGRIP1 | frameshift_deleti  on | exon1 | c.81delA | p.S27fs | . | . |
| CTC4 | 14 | 21897243 | C | CG | 0.359 | NM_001170629 | CHD8 | frameshift_inserti  on | exon2 | c.1094_1095ins  C | p.Q365fs | . | . |
| CTC4 | 14 | 23243700 | AT | A | 0.167 | NM_001126105 | SLC7A7 | frameshift_deleti  on | exon9 | c.1107delA | p.A369fs | . | . |
| CTC4 | 14 | 23548783 | A | AGAAC  GT | 1 | NM_001164815 | ACIN1 | nonframeshift_in  sertion | exon5 | c.1814_1815ins  ACGTTC | p.S605delinsSR  S | . | . |
| CTC4 | 14 | 23829047 | GCC | G | 0.318 | NM_032459 | EFS | frameshift_deleti  on | exon3 | c.359_360del | p.G120fs | . | . |
| CTC4 | 14 | 23991931 | GT | G | 0.542 | NM_033400 | ZFHX2 | frameshift_deleti  on | exon10 | c.6958delA | p.T2320fs | . | . |
| CTC4 | 14 | 23994029 | C | CA | 0.368 | NM_033400 | ZFHX2 | frameshift_inserti  on | exon9 | c.5121_5122ins  T | p.G1708fs | . | . |
| CTC4 | 14 | 24470139 | GA | G | 1 | NM_001193636 | DHRS4L2 | frameshift_deleti  on | exon4 | c.174delA | p.R58fs | . | . |
| CTC4 | 14 | 24568908 | TG | T | 1 | NM_001018073 | PCK2 | stopgain | exon6 | c.995delG | p.W332X | . | . |
| CTC4 | 14 | 25043479 | TC | T | 0.296 | NM_001911 | CTSG | frameshift_deleti on | exon4 | c.565delG | p.D189fs | . | . |
| CTC4 | 14 | 31644190 | AT | A | 1 | NM_015382 | HECTD1 | frameshift_deleti  on | exon4 | c.529delA | p.M177fs | . | . |
| CTC4 | 14 | 35270325 | CA | C | 0.286 | NM_013448 | BAZ1A | stopgain | exon8 | c.935delT | p.L312X | . | . |
| CTC4 | 14 | 36197662 | TTA | T | 0.31 | NM_001283043 | RALGAPA1 | frameshift_deleti on | exon13 | c.1640_1641del | p.I547fs | . | . |
| CTC4 | 14 | 45605356 | C | CT | 0.958 | NM_020937 | FANCM | frameshift_inserti  on | exon1 | c.123dupT | p.P41fs | . | . |
| CTC4 | 14 | 50296075 | GC | G | 0.894 | NM_004713 | NEMF | frameshift_deleti  on | exon12 | c.1014delG | p.Q338fs | . | . |
| CTC4 | 14 | 56138509 | AT | A | 0.292 | NM_004986 | KTN1 | frameshift_deleti  on | exon36 | c.3359delT | p.I1120fs | . | . |
| CTC4 | 14 | 59014533 | GC | G | 0.571 | NM_014749 | KIAA0586 | frameshift_deleti  on | exon30 | c.4303delC | p.P1435fs | . | . |
| CTC4 | 14 | 64465006 | GA | G | 0.842 | NM_015180 | SYNE2 | frameshift_deleti  on | exon26 | c.3314delA | p.E1105fs | . | . |
| CTC4 | 14 | 64683074 | C | CA | 0.154 | NM_182910 | SYNE2 | frameshift_inserti  on | exon1 | c.35_36insA | p.P12fs | . | . |
| CTC4 | 14 | 65198186 | CT | C | 1 | NM_015549 | PLEKHG3 | frameshift_deleti  on | exon6 | c.790delT | p.F264fs | . | . |
| CTC4 | 14 | 67675040 | GA | G | 0.636 | NM_173526 | FAM71D | frameshift_deleti  on | exon6 | c.1035delA | p.R345fs | . | . |
| CTC4 | 14 | 68268859 | CT | C | 1 | NM_015346 | ZFYVE26 | frameshift_deleti  on | exon10 | c.1575delA | p.K525fs | . | . |
| CTC4 | 14 | 69866087 | A | AG | 1 | NM_001252148 | SLC39A9 | frameshift_inserti  on | exon1 | c.1_2insG | p.M1fs | . | . |
| CTC4 | 14 | 71227834 | CA | C | 1 | NM_001284230 | MAP3K9 | frameshift_deleti  on | exon3 | c.885delT | p.F295fs | . | . |
| CTC4 | 14 | 74205925 | CCTG | C | 0.288 | NM_001043318 | ELMSAN1 | nonframeshift_de  letion | exon2 | c.784_786del | p.262_262del | . | . |
| CTC4 | 14 | 75530195 | AC | A | 1 | NM_001107 | ACYP1 | frameshift_deleti  on | exon2 | c.61delG | p.V21fs | . | . |
| CTC4 | 14 | 75537770 | CA | C | 0.296 | NM_001042430 | ZC2HC1C | frameshift_deleti  on | exon2 | c.495delA | p.P165fs | . | . |
| CTC4 | 14 | 91951983 | AT | A | 0.333 | NM_001284280 | SMEK1 | frameshift_deleti  on | exon3 | c.289delA | p.I97fs | . | . |
| CTC4 | 14 | 92251650 | CT | C | 1 | NM_001289134 | TC2N | frameshift_deleti  on | exon10 | c.1025delA | p.K342fs | . | . |
| CTC4 | 14 | 93154537 | TGGC | T | 1 | NM_024832 | RIN3 | nonframeshift_de  letion | exon10 | c.2899_2901del | p.967_967del | . | . |
| CTC4 | 14 | 94245562 | CG | C | 0.875 | NM_178013 | PRIMA1 | frameshift_deleti  on | exon3 | c.188delC | p.P63fs | . | . |
| CTC4 | 14 | 96986402 | AC | A | 0.417 | NM_001252006 | PAPOLA | frameshift_deleti  on | exon2 | c.20delC | p.T7fs | . | . |
| CTC4 | 14 | 99976622 | C | CT | 0.7 | NM_001099402 | CCNK | frameshift_inserti  on | exon11 | c.1247dupT | p.L416fs | . | . |
| CTC4 | 14 | 100743862 | TA | T | 0.368 | NM_003403 | YY1 | frameshift_deleti  on | exon5 | c.1171delA | p.N391fs | . | . |
| CTC4 | 14 | 103958267 | TC | T | 0.315 | NM_001128921 | MARK3 | frameshift_deleti  on | exon14 | c.1504delC | p.P502fs | . | . |
| CTC4 | 15 | 22956366 | CT | C | 0.408 | NM_001033028 | CYFIP1 | frameshift_deleti  on | exon1 | c.311delT | p.L104fs | . | . |
| CTC4 | 15 | 23579214 | AGGATG  GGTAGG GAGGTG GGGATG GGTAGG | A | 0.953 | NM_001271664 | LOC440243 | nonframeshift_de letion | exon1 | c.39_74del | p.13_25del | . | . |
| CTC4 | 15 | 23811245 | C | CA | 0.462 | NM_005664 | MKRN3 | frameshift_inserti  on | exon1 | c.317dupA | p.H106fs | . | . |
| CTC4 | 15 | 27772665 | CT | C | 0.814 | NM_033223 | GABRG3 | frameshift_deleti  on | exon8 | c.953delT | p.L318fs | . | . |
| CTC4 | 15 | 28518094 | TGG | T | 0.129 | NM_004667 | HERC2 | frameshift_deleti  on | exon8 | c.855_856del | p.D285fs | . | . |
| CTC4 | 15 | 31294777 | AT | A | 0.864 | NM_001252020 | TRPM1 | frameshift_deleti  on | exon27 | c.4176delA | p.K1392fs | . | . |
| CTC4 | 15 | 33962642 | AGAG | A | 0.182 | NM_001036 | RYR3 | nonframeshift_de  letion | exon38 | c.5746_5748del | p.1916_1916de  l | . | . |
| CTC4 | 15 | 34030768 | AT | A | 0.619 | NM_001036 | RYR3 | frameshift_deleti  on | exon50 | c.7634delT | p.I2545fs | . | . |
| CTC4 | 15 | 42109897 | AG | A | 0.226 | NM_001265611 | MAPKBP1 | frameshift_deleti  on | exon16 | c.1869delG | p.K623fs | . | . |
| CTC4 | 15 | 42702148 | CG | C | 0.321 | NM_173089 | CAPN3 | frameshift_deleti  on | exon4 | c.76delG | p.G26fs | . | . |
| CTC4 | 15 | 42981975 | GA | G | 1 | NM_020759 | STARD9 | frameshift_deleti  on | exon23 | c.8200delA | p.R2734fs | . | . |
| CTC4 | 15 | 42985810 | C | CA | 0.233 | NM_020759 | STARD9 | frameshift_inserti  on | exon23 | c.12035dupA | p.Q4012fs | . | . |
| CTC4 | 15 | 43067610 | GT | G | 1 | NM_173500 | TTBK2 | frameshift_deleti  on | exon13 | c.1720delA | p.T574fs | . | . |
| CTC4 | 15 | 43814050 | CT | C | 0.214 | NM_002373 | MAP1A | frameshift_deleti  on | exon4 | c.380delT | p.L127fs | . | . |
| CTC4 | 15 | 44202151 | CT | C | 1 | NM_032892 | FRMD5 | frameshift_deleti  on | exon5 | c.355delA | p.R119fs | . | . |
| CTC4 | 15 | 48782048 | CT | C | 0.267 | NM_000138 | FBN1 | frameshift_deleti  on | exon25 | c.3081delA | p.K1027fs | . | . |
| CTC4 | 15 | 48826337 | AC | A | 0.5 | NM_000138 | FBN1 | frameshift_deleti  on | exon8 | c.801delG | p.G267fs | . | . |
| CTC4 | 15 | 50838681 | GA | G | 0.432 | NM_203494 | USP50 | frameshift_deleti  on | exon1 | c.41delT | p.I14fs | . | . |
| CTC4 | 15 | 51676072 | G | GA | 0.826 | NM_181789 | GLDN | frameshift_inserti  on | exon4 | c.525dupA | p.G175fs | . | . |
| CTC4 | 15 | 52258384 | CT | C | 0.35 | NM_001286430 | LEO1 | frameshift_deleti  on | exon2 | c.375delA | p.G125fs | . | . |

| CTC4 | 15 | 55912404 | TG | T | 0.291 | NM_173814 | PRTG | frameshift_deleti  on | exon20 | c.3258delC | p.I1086fs | . | . |
| --- | --- | --- | --- | --- | --- | --- | --- | --- | --- | --- | --- | --- | --- |
| CTC4 | 15 | 56032679 | CT | C | 0.927 | NM_173814 | PRTG | frameshift_deleti  on | exon2 | c.297delA | p.E99fs | . | . |
| CTC4 | 15 | 62146704 | CA | C | 0.306 | NM_017684 | VPS13C | stopgain | exon83 | c.11084delT | p.L3695X | . | . |
| CTC4 | 15 | 63578767 | G | GA | 0.765 | NM_001145646 | APH1B | frameshift_inserti on | exon3 | c.296dupA | p.E99fs | . | . |
| CTC4 | 15 | 64967457 | GC | G | 0.62 | NM_015042 | ZNF609 | frameshift_deleti  on | exon4 | c.2405delC | p.A802fs | . | . |
| CTC4 | 15 | 64967889 | GA | G | 0.727 | NM_015042 | ZNF609 | frameshift_deleti  on | exon4 | c.2837delA | p.E946fs | . | . |
| CTC4 | 15 | 65412230 | G | GT | 0.386 | NM_005707 | PDCD7 | frameshift_inserti  on | exon3 | c.1071dupA | p.L358fs | . | . |
| CTC4 | 15 | 65957024 | CT | C | 0.562 | NM_005848 | DENND4A | frameshift_deleti  on | exon30 | c.5263delA | p.S1755fs | . | . |
| CTC4 | 15 | 66737007 | TG | T | 0.523 | NM_002755 | MAP2K1 | frameshift_deleti  on | exon5 | c.531delG | p.L177fs | . | . |
| CTC4 | 15 | 66793726 | GA | G | 0.308 | NM_000968 | RPL4 | frameshift_deleti  on | exon6 | c.662delT | p.F221fs | . | . |
| CTC4 | 15 | 69237988 | GT | G | 0.392 | NM_145658 | SPESP1 | frameshift_deleti  on | exon2 | c.116delT | p.V39fs | . | . |
| CTC4 | 15 | 73889408 | AG | A | 1 | NM_001161363 | NPTN | frameshift_deleti  on | exon2 | c.393delC | p.P131fs | . | . |
| CTC4 | 15 | 74536399 | CTAA | C | 0.919 | NM_025055 | CCDC33 | nonframeshift_de  letion | exon2 | c.96_98del | p.32_33del | . | . |
| CTC4 | 15 | 74536400 | TAAG | T | 0.994 | NM_025055 | CCDC33 | nonframeshift_de  letion | exon2 | c.97_99del | p.33_33del | . | . |
| CTC4 | 15 | 74636137 | GA | G | 1 | NM_000781 | CYP11A1 | frameshift_deleti  on | exon4 | c.821delT | p.F274fs | . | . |
| CTC4 | 15 | 75500443 | CAT | C | 0.355 | NM_015492 | C15orf39 | frameshift_deleti  on | exon2 | c.2055_2056del | p.T685fs | . | . |
| CTC4 | 15 | 76225272 | G | GT | 1 | NM_147188 | FBXO22 | frameshift_inserti  on | exon7 | c.1042dupT | p.K347fs | . | . |
| CTC4 | 15 | 77407521 | C | CT | 0.568 | NM_024776 | PEAK1 | frameshift_inserti  on | exon8 | c.4217dupA | p.E1406fs | . | . |
| CTC4 | 15 | 77450875 | T | TG | 0.216 | NM_024776 | PEAK1 | frameshift_inserti  on | exon6 | c.3300_3301ins  C | p.N1101fs | . | . |
| CTC4 | 15 | 77906411 | CG | C | 0.358 | NM_032808 | LINGO1 | frameshift_deleti  on | exon2 | c.1837delC | p.R613fs | . | . |
| CTC4 | 15 | 78471092 | GC | G | 0.803 | NM_001199377 | ACSBG1 | frameshift_deleti  on | exon11 | c.1553delG | p.G518fs | . | . |
| CTC4 | 15 | 78566760 | GA | G | 1 | NM_001130182 | DNAJA4 | frameshift_deleti  on | exon4 | c.641delA | p.E214fs | . | . |
| CTC4 | 15 | 78755346 | TA | T | 0.235 | NM_004136 | IREB2 | stopgain | exon3 | c.190delA | p.M64X | . | . |
| CTC4 | 15 | 81625116 | G | GC | 0.594 | NM_001080532 | TMC3 | frameshift_inserti on | exon22 | c.2946_2947ins G | p.R983fs | . | . |
| CTC4 | 15 | 82519827 | CA | C | 1 | NM_001040610 | EFTUD1 | frameshift_deleti  on | exon9 | c.1002delT | p.F334fs | . | . |
| CTC4 | 15 | 84566752 | CA | C | 0.471 | NM_207517 | ADAMTSL3 | frameshift_deleti  on | exon14 | c.1611delA | p.P537fs | . | . |
| CTC4 | 15 | 85345171 | C | CA | 0.386 | NM_014630 | ZNF592 | frameshift_inserti  on | exon11 | c.3352dupA | p.T1117fs | . | . |
| CTC4 | 15 | 90270455 | GC | G | 0.657 | NM_001284395 | WDR93 | frameshift_deleti  on | exon9 | c.949delC | p.Q317fs | . | . |
| CTC4 | 15 | 91017886 | CA | C | 0.296 | NM_003870 | IQGAP1 | frameshift_deleti  on | exon23 | c.2746delA | p.K916fs | . | . |
| CTC4 | 15 | 91432819 | TG | T | 0.242 | NM_001143783 | FES | frameshift_deleti  on | exon5 | c.706delG | p.G236fs | . | . |
| CTC4 | 15 | 99903374 | T | TG | 0.4 | NM_144598 | LRRC28 | frameshift_inserti  on | exon9 | c.936dupG | p.L312fs | . | . |
| CTC4 | 15 | 100636626 | AC | A | 1 | NM_139057 | ADAMTS17 | frameshift_deleti  on | exon15 | c.2071delG | p.V691fs | . | . |
| CTC4 | 15 | 100943017 | C | CT | 0.961 | NM_178842 | CERS3 | frameshift_inserti  on | exon13 | c.1052dupA | p.E351fs | . | . |
| CTC4 | 16 | 2506844 | GT | G | 0.353 | NM_001761 | CCNF | frameshift_deleti  on | exon17 | c.2185delT | p.S729fs | . | . |
| CTC4 | 16 | 4903142 | TA | T | 0.55 | NM_001079514 | UBN1 | frameshift_deleti  on | exon2 | c.225delA | p.V75fs | . | . |
| CTC4 | 16 | 11940689 | GA | G | 1 | NM_015659 | RSL1D1 | frameshift_deleti  on | exon4 | c.395delT | p.L132fs | . | . |
| CTC4 | 16 | 13297320 | AC | A | 0.283 | NM_001145204 | SHISA9 | frameshift_deleti  on | exon3 | c.762delC | p.Y254fs | . | . |
| CTC4 | 16 | 15694497 | CT | C | 0.5 | NM_001184998 | KIAA0430 | frameshift_deleti  on | exon24 | c.4687delA | p.S1563fs | . | . |
| CTC4 | 16 | 16248602 | TG | T | 1 | NM_001171 | ABCC6 | frameshift_deleti  on | exon29 | c.4090delC | p.Q1364fs | . | . |
| CTC4 | 16 | 21051174 | CT | C | 0.418 | NM_017539 | DNAH3 | frameshift_deleti  on | exon33 | c.4729delA | p.S1577fs | . | . |
| CTC4 | 16 | 24802450 | GA | G | 0.33 | NM_014494 | TNRC6A | frameshift_deleti  on | exon6 | c.2488delA | p.N830fs | . | . |
| CTC4 | 16 | 27245541 | CA | C | 0.415 | NM_145080 | NSMCE1 | frameshift_deleti  on | exon4 | c.303delT | p.F101fs | . | . |
| CTC4 | 16 | 27374906 | GGCT | G | 1 | NM_001257406 | IL4R | nonframeshift_de  letion | exon10 | c.2234_2236del | p.745_746del | . | . |
| CTC4 | 16 | 30736173 | C | CA | 0.857 | NM_006662 | SRCAP | frameshift_inserti  on | exon25 | c.5428_5429ins  A | p.P1810fs | . | . |
| CTC4 | 16 | 31049879 | GC | G | 0.222 | NM_001272095 | STX4 | frameshift_deleti  on | exon7 | c.380delC | p.A127fs | . | . |
| CTC4 | 16 | 31143478 | TG | T | 0.25 | NM_002773 | PRSS8 | frameshift_deleti  on | exon6 | c.883delC | p.Q295fs | . | . |
| CTC4 | 16 | 47694669 | CG | C | 0.875 | NM_000293 | PHKB | frameshift_deleti  on | exon22 | c.2136delG | p.P712fs | . | . |
| CTC4 | 16 | 49430485 | TG | T | 0.988 | NM_144602 | C16orf78 | frameshift_deleti  on | exon4 | c.547delG | p.D183fs | . | . |
| CTC4 | 16 | 49672669 | CT | C | 0.977 | NM_001271620 | ZNF423 | frameshift_deleti  on | exon4 | c.213delA | p.E71fs | . | . |
| CTC4 | 16 | 50128647 | CAT | C | 0.286 | NM_182922 | HEATR3 | frameshift_deleti  on | exon12 | c.1543_1544del | p.I515fs | . | . |
| CTC4 | 16 | 50818294 | GA | G | 0.333 | NM_001042355 | CYLD | frameshift_deleti  on | exon11 | c.1873delA | p.N625fs | . | . |
| CTC4 | 16 | 55527118 | GC | G | 0.488 | NM_001127891 | MMP2 | frameshift_deleti  on | exon9 | c.1236delC | p.G412fs | . | . |
| CTC4 | 16 | 56970722 | A | AT | 0.867 | NM_001010989 | HERPUD1 | frameshift_inserti  on | exon4 | c.422dupT | p.I141fs | . | . |
| CTC4 | 16 | 57492162 | T | TA | 0.667 | NM_020312 | COQ9 | frameshift_inserti  on | exon6 | c.611_612insA | p.L204fs | . | . |
| CTC4 | 16 | 57685270 | CA | C | 0.6 | NM_001145773 | GPR56 | frameshift_deleti  on | exon3 | c.239delA | p.H80fs | . | . |
| CTC4 | 16 | 58562523 | CA | C | 0.492 | NM_001265612 | CNOT1 | frameshift_deleti  on | exon44 | c.6293delT | p.L2098fs | . | . |
| CTC4 | 16 | 66759794 | TA | T | 0.25 | NM_001286157 | DYNC1LI2 | frameshift_deleti  on | exon11 | c.1083delT | p.S361fs | . | . |
| CTC4 | 16 | 66766256 | CT | C | 1 | NM_001286157 | DYNC1LI2 | frameshift_deleti  on | exon6 | c.683delA | p.K228fs | . | . |

| CTC4 | 16 | 68100537 | GA | G | 0.667 | NM_001271763 | DUS2 | frameshift_deleti  on | exon8 | c.448delA | p.R150fs | . | . |
| --- | --- | --- | --- | --- | --- | --- | --- | --- | --- | --- | --- | --- | --- |
| CTC4 | 16 | 69177284 | GT | G | 0.283 | NM_032830 | CIRH1A | frameshift_deleti  on | exon6 | c.731delT | p.V244fs | . | . |
| CTC4 | 16 | 69964064 | TG | T | 0.258 | NM_199424 | WWP2 | frameshift_deleti  on | exon3 | c.32delG | p.W11fs | . | . |
| CTC4 | 16 | 71798322 | AT | A | 1 | NM_001128 | AP1G1 | frameshift_deleti  on | exon9 | c.848delA | p.N283fs | . | . |
| CTC4 | 16 | 81242148 | GTT | G | 1 | NM_001076780 | PKD1L2 | frameshift_deleti  on | exon4 | c.706_707del | p.N236fs | . | . |
| CTC4 | 16 | 84050296 | CA | C | 1 | NM_001080442 | SLC38A8 | frameshift_deleti  on | exon8 | c.989delT | p.L330fs | . | . |
| CTC4 | 16 | 84217059 | C | CG | 0.404 | NM_001243156 | TAF1C | frameshift_inserti  on | exon4 | c.274dupC | p.R92fs | . | . |
| CTC4 | 16 | 84270580 | T | TCCTCC  AGCC | 0.883 | NM_172347 | KCNG4 | nonframeshift_in  sertion | exon2 | c.511_512insG  GCTGGAGG | p.E171delinsGL  EE | . | . |
| CTC4 | 16 | 84270588 | CT | C | 0.209 | NM_172347 | KCNG4 | frameshift_deleti  on | exon2 | c.503delA | p.K168fs | . | . |
| CTC4 | 16 | 84879503 | TGG | T | 0.768 | NM_031476 | CRISPLD2 | frameshift_deleti  on | exon3 | c.353_354del | p.W118fs | . | . |
| CTC4 | 16 | 85743812 | TG | T | 0.944 | NM_206967 | C16orf74 | frameshift_deleti  on | exon3 | c.129delC | p.P43fs | . | . |
| CTC4 | 16 | 85942693 | AT | A | 1 | NM_002163 | IRF8 | frameshift_deleti  on | exon3 | c.273delT | p.D91fs | . | . |
| CTC4 | 17 | 3623672 | GC | G | 0.652 | NM_002208 | ITGAE | frameshift_deleti  on | exon29 | c.3261delG | p.L1087fs | . | . |
| CTC4 | 17 | 5392422 | CA | C | 1 | NM_001258218 | MIS12 | frameshift_deleti  on | exon2 | c.241delA | p.K81fs | . | . |
| CTC4 | 17 | 6367595 | ACC | A | 0.718 | NM_001165966 | PITPNM3 | frameshift_deleti  on | exon15 | c.1941_1942del | p.W647fs | . | . |
| CTC4 | 17 | 6511736 | CT | C | 0.692 | NM_014804 | KIAA0753 | frameshift_deleti  on | exon10 | c.1760delA | p.E587fs | . | . |
| CTC4 | 17 | 7158068 | T | TC | 0.5 | NM_203414 | ELP5 | frameshift_inserti  on | exon4 | c.403_404insC | p.C135fs | . | . |
| CTC4 | 17 | 7224754 | GA | G | 0.692 | NM_001005408 | NEURL4 | frameshift_deleti  on | exon19 | c.3117delT | p.R1039fs | . | . |
| CTC4 | 17 | 7287133 | CCA | C | 0.244 | NM_001251902 | TNK1 | frameshift_deleti  on | exon5 | c.533_534del | p.P178fs | . | . |
| CTC4 | 17 | 7460187 | AC | A | 0.478 | NM_003809 | TNFSF12 | frameshift_deleti  on | exon6 | c.467delC | p.T156fs | . | . |
| CTC4 | 17 | 7481785 | AT | A | 1 | NM_001416 | EIF4A1 | frameshift_deleti  on | exon11 | c.1203delT | p.N401fs | . | . |
| CTC4 | 17 | 7536630 | G | GC | 0.562 | NM_001146281 | SHBG | frameshift_inserti  on | exon6 | c.825dupC | p.C275fs | . | . |
| CTC4 | 17 | 7611338 | G | GC | 0.321 | NM_001406 | EFNB3 | frameshift_inserti  on | exon2 | c.186dupC | p.C62fs | . | . |
| CTC4 | 17 | 7612701 | T | TG | 0.926 | NM_001406 | EFNB3 | frameshift_inserti  on | exon5 | c.831dupG | p.L277fs | . | . |
| CTC4 | 17 | 8422050 | GA | G | 1 | NM_005964 | MYH10 | frameshift_deleti  on | exon19 | c.2309delT | p.F770fs | . | . |
| CTC4 | 17 | 9615350 | AG | A | 0.369 | NM_001267576 | USP43 | frameshift_deleti  on | exon14 | c.2222delG | p.R741fs | . | . |
| CTC4 | 17 | 16326132 | A | AC | 0.647 | NM_016113 | TRPV2 | frameshift_inserti  on | exon4 | c.554_555insC | p.N185fs | . | . |
| CTC4 | 17 | 17696913 | CA | C | 0.5 | NM_030665 | RAI1 | frameshift_deleti  on | exon3 | c.652delA | p.T218fs | . | . |
| CTC4 | 17 | 18874685 | C | CGGT | 1 | NM_001039999 | FAM83G | nonframeshift_in  sertion | exon6 | c.2458_2459ins  ACC | p.R820delinsH  R | . | . |
| CTC4 | 17 | 18874990 | AG | A | 0.311 | NM_001039999 | FAM83G | frameshift_deleti  on | exon6 | c.2153delC | p.P718fs | . | . |
| CTC4 | 17 | 19564501 | AT | A | 1 | NM_000382 | ALDH3A2 | frameshift_deleti  on | exon6 | c.861delT | p.H287fs | . | . |
| CTC4 | 17 | 19683901 | TG | T | 0.61 | NM_001142610 | ULK2 | frameshift_deleti  on | exon25 | c.2799delC | p.T933fs | . | . |
| CTC4 | 17 | 21318791 | TC | T | 0.306 | NM_001194958 | KCNJ18 | frameshift_deleti  on | exon3 | c.138delC | p.F46fs | . | . |
| CTC4 | 17 | 26817411 | CG | C | 0.486 | NM_001145975 | SLC13A2 | frameshift_deleti  on | exon3 | c.319delG | p.G107fs | . | . |
| CTC4 | 17 | 26919339 | A | AG | 0.475 | NM_006461 | SPAG5 | frameshift_inserti  on | exon3 | c.922dupC | p.L308fs | . | . |
| CTC4 | 17 | 26940705 | TG | T | 1 | NM_001174103 | SGK494 | frameshift_deleti  on | exon2 | c.76delC | p.Q26fs | . | . |
| CTC4 | 17 | 27066105 | A | AG | 0.312 | NM_178170 | NEK8 | frameshift_inserti  on | exon10 | c.1303_1304ins  G | p.T435fs | . | . |
| CTC4 | 17 | 27075566 | GC | G | 0.414 | NM_004295 | TRAF4 | frameshift_deleti  on | exon6 | c.663delC | p.C221fs | . | . |
| CTC4 | 17 | 28418924 | AT | A | 0.258 | NM_198529 | EFCAB5 | frameshift_deleti  on | exon21 | c.3974delT | p.I1325fs | . | . |
| CTC4 | 17 | 29162642 | CA | C | 0.6 | NM_024857 | ATAD5 | frameshift_deleti  on | exon2 | c.1544delA | p.Q515fs | . | . |
| CTC4 | 17 | 30696371 | GC | G | 0.554 | NM_001032293 | ZNF207 | frameshift_deleti  on | exon10 | c.1130delC | p.A377fs | . | . |
| CTC4 | 17 | 30986309 | TG | T | 0.264 | NM_015194 | MYO1D | frameshift_deleti  on | exon17 | c.2168delC | p.A723fs | . | . |
| CTC4 | 17 | 33323114 | CT | C | 1 | NM_002311 | LIG3 | frameshift_deleti  on | exon10 | c.1644delT | p.A548fs | . | . |
| CTC4 | 17 | 33510548 | CTG | C | 0.35 | NM_001033576 | UNC45B | frameshift_deleti  on | exon19 | c.2477_2478del | p.L826fs | . | . |
| CTC4 | 17 | 33689955 | CGT | C | 0.6 | NM_152270 | SLFN11 | frameshift_deleti  on | exon2 | c.870_871del | p.Q290fs | . | . |
| CTC4 | 17 | 33738387 | CT | C | 1 | NM_001289009 | SLFN12 | frameshift_deleti  on | exon4 | c.1706delA | p.K569fs | . | . |
| CTC4 | 17 | 33806840 | AAG | A | 1 | NM_001195790 | SLFN12L | frameshift_deleti  on | exon2 | c.387_388del | p.S129fs | . | . |
| CTC4 | 17 | 35902554 | GT | G | 1 | NM_001163544 | SYNRG | frameshift_deleti  on | exon14 | c.2487delA | p.K829fs | . | . |
| CTC4 | 17 | 36484364 | G | GT | 0.587 | NM_001004334 | GPR179 | frameshift_inserti  on | exon11 | c.5087dupA | p.N1696fs | . | . |
| CTC4 | 17 | 37657505 | GC | G | 0.559 | NM_015083 | CDK12 | frameshift_deleti  on | exon6 | c.2423delC | p.A808fs | . | . |
| CTC4 | 17 | 37902391 | AC | A | 0.667 | NM_001030002 | GRB7 | frameshift_deleti  on | exon14 | c.1389delC | p.N463fs | . | . |
| CTC4 | 17 | 38910161 | TA | T | 1 | NM_181534 | KRT25 | frameshift_deleti  on | exon3 | c.619delT | p.Y207fs | . | . |
| CTC4 | 17 | 39684320 | G | GC | 0.324 | NM_002276 | KRT19 | frameshift_inserti  on | exon1 | c.179_180insG | p.A60fs | . | . |
| CTC4 | 17 | 39768811 | T | TG | 0.391 | NM_005557 | KRT16 | frameshift_inserti  on | exon1 | c.129dupC | p.S44fs | . | . |
| CTC4 | 17 | 40715102 | CG | C | 1 | NM_025233 | COASY | frameshift_deleti  on | exon1 | c.463delG | p.D155fs | . | . |
| CTC4 | 17 | 40993555 | TC | T | 0.352 | NM_005789 | PSME3 | frameshift_deleti  on | exon11 | c.726delC | p.I242fs | . | . |
| CTC4 | 17 | 41170177 | A | AT | 0.267 | NM_006373 | VAT1 | frameshift_inserti  on | exon3 | c.641dupA | p.N214fs | . | . |
| CTC4 | 17 | 41847013 | AT | A | 0.349 | NM_004090 | DUSP3 | frameshift_deleti  on | exon3 | c.521delA | p.N174fs | . | . |

| CTC4 | 17 | 43246719 | GC | G | 0.413 | NM_144608 | HEXIM2 | frameshift_deleti  on | exon4 | c.405delC | p.G135fs | . | . |
| --- | --- | --- | --- | --- | --- | --- | --- | --- | --- | --- | --- | --- | --- |
| CTC4 | 17 | 43516987 | AC | A | 0.264 | NM_014798 | PLEKHM1 | frameshift_deleti  on | exon11 | c.2914delG | p.V972fs | . | . |
| CTC4 | 17 | 44115946 | AG | A | 0.294 | NM_001193466 | KANSL1 | frameshift_deleti  on | exon10 | c.2498delC | p.P833fs | . | . |
| CTC4 | 17 | 45012495 | AT | A | 0.203 | NM_001012511 | GOSR2 | frameshift_deleti  on | exon5 | c.438delT | p.N146fs | . | . |
| CTC4 | 17 | 45669404 | TG | T | 1 | NM_006310 | NPEPPS | frameshift_deleti  on | exon11 | c.1344delG | p.L448fs | . | . |
| CTC4 | 17 | 46135738 | GC | G | 0.518 | NM_003204 | NFE2L1 | frameshift_deleti  on | exon6 | c.1055delC | p.A352fs | . | . |
| CTC4 | 17 | 48460505 | TAGC | T | 0.185 | NM_018509 | LRRC59 | nonframeshift_de  letion | exon7 | c.765_767del | p.255_256del | . | . |
| CTC4 | 17 | 49824994 | TC | T | 1 | NM_020178 | CA10 | frameshift_deleti  on | exon4 | c.463delG | p.E155fs | . | . |
| CTC4 | 17 | 53798132 | TA | T | 1 | NM_018286 | TMEM100 | frameshift_deleti  on | exon2 | c.299delT | p.L100fs | . | . |
| CTC4 | 17 | 54543900 | AC | A | 0.26 | NM_153228 | ANKFN1 | frameshift_deleti  on | exon14 | c.1751delC | p.T584fs | . | . |
| CTC4 | 17 | 55918329 | C | CA | 0.217 | NM_016070 | MRPS23 | frameshift_inserti  on | exon4 | c.377dupT | p.L126fs | . | . |
| CTC4 | 17 | 56573313 | TC | T | 0.986 | NM_004687 | MTMR4 | frameshift_deleti  on | exon16 | c.2189delG | p.G730fs | . | . |
| CTC4 | 17 | 56573515 | AC | A | 0.642 | NM_004687 | MTMR4 | stopgain | exon16 | c.1987delG | p.V663X | . | . |
| CTC4 | 17 | 56770091 | TG | T | 0.857 | NM_002876 | RAD51C | frameshift_deleti on | exon1 | c.88delG | p.A30fs | . | . |
| CTC4 | 17 | 58144798 | AT | A | 0.75 | NM_022070 | HEATR6 | stopgain | exon8 | c.1234delA | p.M412X | . | . |
| CTC4 | 17 | 58260730 | CA | C | 0.37 | NM_032582 | USP32 | frameshift_deleti on | exon31 | c.3918delT | p.F1306fs | . | . |
| CTC4 | 17 | 60813462 | C | CA | 0.848 | NM_001100875 | 10-Mar | frameshift_inserti  on | exon6 | c.1766dupT | p.L589fs | . | . |
| CTC4 | 17 | 61497604 | GA | G | 0.309 | NM_025185 | TANC2 | frameshift_deleti  on | exon25 | c.4262delA | p.E1421fs | . | . |
| CTC4 | 17 | 61914850 | G | GA | 0.571 | NM_001098426 | SMARCD2 | frameshift_inserti  on | exon2 | c.351_352insT | p.R118fs | . | . |
| CTC4 | 17 | 62499568 | TG | T | 0.212 | NM_004396 | DDX5 | frameshift_deleti  on | exon6 | c.628delC | p.Q210fs | . | . |
| CTC4 | 17 | 62892684 | TC | T | 0.988 | NM_199340 | LRRC37A3 | frameshift_deleti  on | exon3 | c.691delG | p.E231fs | . | . |
| CTC4 | 17 | 63157007 | AT | A | 0.508 | NM_001081955 | RGS9 | frameshift_deleti  on | exon6 | c.404delT | p.I135fs | . | . |
| CTC4 | 17 | 65074543 | TG | T | 0.588 | NM_014877 | HELZ | frameshift_deleti  on | exon33 | c.5653delC | p.Q1885fs | . | . |
| CTC4 | 17 | 73836605 | CG | C | 1 | NM_199242 | UNC13D | frameshift_deleti  on | exon9 | c.735delC | p.N245fs | . | . |
| CTC4 | 17 | 74266482 | AC | A | 1 | NM_182565 | UBALD2 | frameshift_deleti  on | exon3 | c.392delC | p.T131fs | . | . |
| CTC4 | 17 | 74621948 | TG | T | 1 | NM_018414 | ST6GALNAC  1 | frameshift_deleti  on | exon8 | c.1557delC | p.P519fs | . | . |
| CTC4 | 17 | 76047420 | TACC | T | 0.217 | NM_001142640 | TNRC6C | nonframeshift_de  letion | exon4 | c.2278_2280del | p.760_760del | . | . |
| CTC4 | 17 | 76423153 | GA | G | 0.213 | NM_173628 | DNAH17 | frameshift_deleti  on | exon78 | c.12624delT | p.I4208fs | . | . |
| CTC4 | 17 | 76867005 | TC | T | 0.655 | NM_003255 | TIMP2 | frameshift_deleti  on | exon3 | c.314delG | p.G105fs | . | . |
| CTC4 | 17 | 77093419 | GTAT | G | 1 | NM_001082575 | RBFOX3 | nonframeshift_de  letion | exon11 | c.707_709del | p.236_237del | . | . |
| CTC4 | 17 | 78322073 | CCAT | C | 0.467 | NM_001256071 | RNF213 | nonframeshift_de  letion | exon29 | c.9939_9941del | p.3313_3314de  l | . | . |
| CTC4 | 17 | 78923270 | AT | A | 0.447 | NM_001163034 | RPTOR | frameshift_deleti  on | exon24 | c.2820delT | p.N940fs | . | . |
| CTC4 | 17 | 80400164 | GC | G | 1 | NM_173620 | HEXDC | stopgain | exon12 | c.1455delC | p.C485X | . | . |
| CTC4 | 18 | 6985352 | GA | G | 0.556 | NM_005559 | LAMA1 | frameshift_deleti on | exon39 | c.5543delT | p.I1848fs | . | . |
| CTC4 | 18 | 18549093 | AT | A | 0.5 | NM_005406 | ROCK1 | stopgain | exon24 | c.2896delA | p.M966X | . | . |
| CTC4 | 18 | 18550386 | CA | C | 0.414 | NM_005406 | ROCK1 | frameshift_deleti on | exon23 | c.2742delT | p.F914fs | . | . |
| CTC4 | 18 | 19024274 | GA | G | 0.491 | NM_001142966 | GREB1L | frameshift_deleti  on | exon11 | c.1298delA | p.E433fs | . | . |
| CTC4 | 18 | 29246283 | AT | A | 1 | NM_004775 | B4GALT6 | frameshift_deleti  on | exon2 | c.167delA | p.N56fs | . | . |
| CTC4 | 18 | 32834159 | AT | A | 0.311 | NM_001288711 | ZSCAN30 | frameshift_deleti  on | exon2 | c.178delA | p.I60fs | . | . |
| CTC4 | 18 | 33077731 | G | GCC | 0.564 | NM_001098817 | INO80C | frameshift_inserti  on | exon1 | c.107_108insG  G | p.G36fs | . | . |
| CTC4 | 18 | 43248358 | TG | T | 0.451 | NM_007163 | SLC14A2 | frameshift_deleti  on | exon15 | c.1953delG | p.V651fs | . | . |
| CTC4 | 18 | 44143148 | AC | A | 0.208 | NM_144612 | LOXHD1 | frameshift_deleti  on | exon18 | c.2477delG | p.G826fs | . | . |
| CTC4 | 18 | 46448224 | AG | A | 0.8 | NM_001190823 | SMAD7 | frameshift_deleti  on | exon2 | c.234delC | p.Y78fs | . | . |
| CTC4 | 18 | 51813700 | CG | C | 0.833 | NM_007195 | POLI | frameshift_deleti  on | exon8 | c.1118delG | p.R373fs | . | . |
| CTC4 | 18 | 52575077 | GT | G | 1 | NM_001143829 | CCDC68 | frameshift_deleti  on | exon11 | c.889delA | p.T297fs | . | . |
| CTC4 | 18 | 59878426 | TG | T | 0.451 | NM_020854 | KIAA1468 | frameshift_deleti  on | exon2 | c.568delG | p.A190fs | . | . |
| CTC4 | 18 | 61460475 | TG | T | 0.973 | NM_001261831 | SERPINB7 | frameshift_deleti  on | exon3 | c.250delG | p.G84fs | . | . |
| CTC4 | 18 | 65178862 | T | TA | 0.857 | NM_032160 | DSEL | frameshift_inserti  on | exon2 | c.3013dupT | p.Y1005fs | . | . |
| CTC4 | 18 | 67816264 | CG | C | 0.75 | NM_173630 | RTTN | frameshift_deleti  on | exon17 | c.2181delC | p.A727fs | . | . |
| CTC4 | 18 | 67863850 | T | TCTC | 1 | NM_173630 | RTTN | nonframeshift_in  sertion | exon7 | c.727_728insG  AG | p.D243delinsG  D | . | . |
| CTC4 | 18 | 67863853 | C | CCT | 0.952 | NM_173630 | RTTN | frameshift_inserti  on | exon7 | c.724_725insA  G | p.G242fs | . | . |
| CTC4 | 18 | 72347184 | TG | T | 0.981 | NM_001146189 | ZNF407 | frameshift_deleti  on | exon1 | c.4210delG | p.E1404fs | . | . |
| CTC4 | 18 | 72632618 | GA | G | 1 | NM_001146189 | ZNF407 | frameshift_deleti  on | exon7 | c.5399delA | p.E1800fs | . | . |
| CTC4 | 18 | 77893789 | AC | A | 0.246 | NM_014913 | ADNP2 | frameshift_deleti  on | exon4 | c.494delC | p.T165fs | . | . |
| CTC4 | 19 | 1073233 | G | GT | 0.8 | NM_001282335 | HMHA1 | frameshift_inserti  on | exon2 | c.156_157insT | p.P52fs | . | . |
| CTC4 | 19 | 3005515 | TC | T | 0.6 | NM_001144762 | TLE2 | stopgain | exon15 | c.1449delG | p.W483X | . | . |
| CTC4 | 19 | 3759970 | TC | T | 1 | NM_004886 | APBA3 | frameshift_deleti on | exon2 | c.292delG | p.D98fs | . | . |

| CTC4 | 19 | 4323110 | C | CT | 0.484 | NM_024333 | FSD1 | frameshift_inserti  on | exon11 | c.1168dupT | p.A389fs | . | . |
| --- | --- | --- | --- | --- | --- | --- | --- | --- | --- | --- | --- | --- | --- |
| CTC4 | 19 | 5711875 | CG | C | 0.429 | NM_001276480 | LONP1 | frameshift_deleti  on | exon4 | c.188delC | p.A63fs | . | . |
| CTC4 | 19 | 7593712 | TG | T | 0.328 | NM_020533 | MCOLN1 | frameshift_deleti  on | exon9 | c.991delG | p.V331fs | . | . |
| CTC4 | 19 | 9868240 | GT | G | 0.25 | NM_001077624 | ZNF846 | frameshift_deleti  on | exon6 | c.1512delA | p.K504fs | . | . |
| CTC4 | 19 | 10433881 | A | AG | 0.571 | NM_133452 | RAVER1 | frameshift_inserti  on | exon5 | c.1068_1069ins  C | p.S357fs | . | . |
| CTC4 | 19 | 10578121 | GT | G | 0.333 | NM_006202 | PDE4A | frameshift_deleti  on | exon10 | c.1769delT | p.V590fs | . | . |
| CTC4 | 19 | 11917605 | CT | C | 0.619 | NM_152356 | ZNF491 | frameshift_deleti  on | exon3 | c.838delT | p.F280fs | . | . |
| CTC4 | 19 | 12739517 | AC | A | 0.993 | NM_153358 | ZNF791 | frameshift_deleti  on | exon4 | c.1175delC | p.T392fs | . | . |
| CTC4 | 19 | 12807281 | TGA | T | 0.222 | NM_032301 | FBXW9 | frameshift_deleti  on | exon1 | c.113_114del | p.L38fs | . | . |
| CTC4 | 19 | 13136059 | GC | G | 1 | NM_001271043 | NFIX | frameshift_deleti  on | exon2 | c.277delC | p.P93fs | . | . |
| CTC4 | 19 | 15756556 | TAC | T | 0.173 | NM_000896 | CYP4F3 | frameshift_deleti  on | exon3 | c.227_228del | p.Y76fs | . | . |
| CTC4 | 19 | 15904975 | GC | G | 0.387 | NM_001004466 | OR10H5 | frameshift_deleti  on | exon1 | c.118delC | p.L40fs | . | . |
| CTC4 | 19 | 15904976 | C | CG | 0.487 | NM_001004466 | OR10H5 | frameshift_inserti  on | exon1 | c.118_119insG | p.L40fs | . | . |
| CTC4 | 19 | 16345235 | CG | C | 1 | NM_032493 | AP1M1 | frameshift_deleti  on | exon12 | c.1262delG | p.R421fs | . | . |
| CTC4 | 19 | 16910799 | TC | T | 1 | NM_001007525 | NWD1 | frameshift_deleti  on | exon17 | c.3563delC | p.S1188fs | . | . |
| CTC4 | 19 | 19115171 | GC | G | 0.417 | NM_001017392 | SUGP2 | frameshift_deleti  on | exon7 | c.2734delG | p.A912fs | . | . |
| CTC4 | 19 | 19260146 | GTT | G | 0.4 | NM_001145785 | MEF2B | frameshift_deleti  on | exon3 | c.145_146del | p.N49fs | . | . |
| CTC4 | 19 | 19260171 | CA | C | 0.4 | NM_001145785 | MEF2B | frameshift_deleti  on | exon3 | c.121delT | p.C41fs | . | . |
| CTC4 | 19 | 22846726 | TA | T | 0.71 | NM_020855 | ZNF492 | frameshift_deleti  on | exon4 | c.256delA | p.K86fs | . | . |
| CTC4 | 19 | 23159703 | GA | G | 0.333 | NM_001267716 | ZNF728 | frameshift_deleti  on | exon4 | c.435delT | p.F145fs | . | . |
| CTC4 | 19 | 23836778 | CT | C | 0.831 | NM_138330 | ZNF675 | frameshift_deleti  on | exon4 | c.956delA | p.K319fs | . | . |
| CTC4 | 19 | 29698830 | CG | C | 0.9 | NM_006003 | UQCRFS1 | frameshift_deleti  on | exon2 | c.449delC | p.A150fs | . | . |
| CTC4 | 19 | 35505281 | TG | T | 1 | NM_001136199 | GRAMD1A | frameshift_deleti  on | exon9 | c.1039delG | p.G347fs | . | . |
| CTC4 | 19 | 37004941 | CT | C | 0.292 | NM_001166037 | ZNF260 | frameshift_deleti  on | exon3 | c.1199delA | p.K400fs | . | . |
| CTC4 | 19 | 38376118 | CT | C | 0.282 | NM_031951 | WDR87 | frameshift_deleti  on | exon6 | c.8075delA | p.K2692fs | . | . |
| CTC4 | 19 | 38378548 | TC | T | 1 | NM_031951 | WDR87 | frameshift_deleti  on | exon6 | c.5645delG | p.G1882fs | . | . |
| CTC4 | 19 | 41173889 | CTGCTG  CTGT | C | 0.286 | NM_004756 | NUMBL | nonframeshift_de  letion | exon10 | c.1305_1313del | p.435_438del | . | . |
| CTC4 | 19 | 41173892 | CTGCTGT | C | 0.992 | NM_004756 | NUMBL | nonframeshift_de  letion | exon10 | c.1305_1310del | p.435_437del | . | . |
| CTC4 | 19 | 41173898 | TTGCTGT  TGCTGC | T | 0.933 | NM_004756 | NUMBL | nonframeshift_de  letion | exon10 | c.1293_1304del | p.431_435del | . | . |
| CTC4 | 19 | 41173898 | TTGCTGT  TGC | T | 0.933 | NM_004756 | NUMBL | nonframeshift_de  letion | exon10 | c.1296_1304del | p.432_435del | . | . |
| CTC4 | 19 | 41173904 | TTGCTGC | T | 0.923 | NM_004756 | NUMBL | nonframeshift_de  letion | exon10 | c.1293_1298del | p.431_433del | . | . |
| CTC4 | 19 | 41255592 | TG | T | 0.909 | NM_198476 | C19orf54 | stopgain | exon1 | c.116delC | p.S39X | . | . |
| CTC4 | 19 | 42132258 | C | CA | 0.267 | NM_001817 | CEACAM4 | frameshift_inserti on | exon2 | c.140_141insT | p.E47fs | . | . |
| CTC4 | 19 | 42219023 | G | GA | 0.917 | NM_004363 | CEACAM5 | frameshift_inserti  on | exon3 | c.558_559insA | p.P186fs | . | . |
| CTC4 | 19 | 42373165 | TG | T | 0.366 | NM_001022 | RPS19 | frameshift_deleti  on | exon4 | c.238delG | p.G80fs | . | . |
| CTC4 | 19 | 43268140 | AG | A | 1 | NM_001130167 | PSG8 | frameshift_deleti  on | exon2 | c.357delC | p.S119fs | . | . |
| CTC4 | 19 | 45381530 | G | GC | 0.4 | NM_002856 | PVRL2 | frameshift_inserti  on | exon6 | c.1093_1094ins  C | p.V365fs | . | . |
| CTC4 | 19 | 45916902 | C | CA | 1 | NM_202001 | ERCC1 | frameshift_inserti  on | exon8 | c.875_876insT | p.W292fs | . | . |
| CTC4 | 19 | 47844839 | GA | G | 1 | NM_001271749 | C5AR2 | frameshift_deleti  on | exon2 | c.784delA | p.N262fs | . | . |
| CTC4 | 19 | 48917808 | T | TC | 0.545 | NM_000836 | GRIN2D | frameshift_inserti  on | exon5 | c.1380dupC | p.V460fs | . | . |
| CTC4 | 19 | 49657886 | C | CTCT | 0.613 | NM_002152 | HRC | nonframeshift_in  sertion | exon1 | c.608_609insA  GA | p.E203delinsEE | . | . |
| CTC4 | 19 | 49657889 | T | TTCC | 0.873 | NM_002152 | HRC | nonframeshift_in  sertion | exon1 | c.605_606insG  GA | p.E202delinsEE | . | . |
| CTC4 | 19 | 49657890 | T | TCCC | 0.524 | NM_002152 | HRC | nonframeshift_in  sertion | exon1 | c.604_605insG  GG | p.E202delinsGE | . | . |
| CTC4 | 19 | 49657892 | C | CTCT | 0.561 | NM_002152 | HRC | nonframeshift_in  sertion | exon1 | c.602_603insA  GA | p.E201delinsEE | . | . |
| CTC4 | 19 | 49657899 | T | TCCC | 0.615 | NM_002152 | HRC | nonframeshift_in  sertion | exon1 | c.595_596insG  GG | p.E199delinsGE | . | . |
| CTC4 | 19 | 49657904 | C | CTCT | 0.656 | NM_002152 | HRC | nonframeshift_in  sertion | exon1 | c.590_591insA  GA | p.E197delinsEE | . | . |
| CTC4 | 19 | 49657905 | T | TCCA | 0.656 | NM_002152 | HRC | nonframeshift_in  sertion | exon1 | c.589_590insTG  G | p.E197delinsVE | . | . |
| CTC4 | 19 | 49793643 | CT | C | 0.889 | NM_014037 | SLC6A16 | frameshift_deleti  on | exon12 | c.1947delA | p.K649fs | . | . |
| CTC4 | 19 | 50156708 | CGGA | C | 0.211 | NM_021228 | SCAF1 | nonframeshift_de  letion | exon7 | c.3063_3065del | p.1021_1022de  l | . | . |
| CTC4 | 19 | 50412636 | GC | G | 0.201 | NM_001193357 | NUP62 | frameshift_deleti  on | exon2 | c.428delG | p.G143fs | . | . |
| CTC4 | 19 | 50868835 | GGCA | G | 0.194 | NM_004851 | NAPSA | nonframeshift_de  letion | exon1 | c.41_43del | p.14_15del | . | . |
| CTC4 | 19 | 50963399 | TG | T | 0.333 | NM_004533 | MYBPC2 | frameshift_deleti  on | exon24 | c.2895delG | p.M965fs | . | . |
| CTC4 | 19 | 51301530 | GC | G | 0.263 | NM_199250 | C19orf48 | frameshift_deleti  on | exon4 | c.175delG | p.A59fs | . | . |
| CTC4 | 19 | 51917028 | TG | T | 0.655 | NM_001171161 | SIGLEC10 | frameshift_deleti  on | exon8 | c.1029delC | p.P343fs | . | . |
| CTC4 | 19 | 53270558 | G | GT | 0.223 | NM_198457 | ZNF600 | frameshift_inserti  on | exon3 | c.450dupA | p.Q151fs | . | . |
| CTC4 | 19 | 53958929 | A | AC | 0.532 | NM_001289951 | ZNF761 | frameshift_inserti  on | exon6 | c.1169dupC | p.T390fs | . | . |

| CTC4 | 19 | 54967423 | AG | A | 0.505 | NM_052925 | LENG8 | frameshift_deleti  on | exon9 | c.1304delG | p.S435fs | . | . |
| --- | --- | --- | --- | --- | --- | --- | --- | --- | --- | --- | --- | --- | --- |
| CTC4 | 19 | 55699530 | TG | T | 0.342 | NM_001161440 | PTPRH | frameshift_deleti  on | exon11 | c.1856delC | p.P619fs | . | . |
| CTC4 | 19 | 55869879 | GGCTCC  TTCATCT CGCCCC | G | 1 | NM_001145402 | FAM71E2 | nonframeshift_de letion | exon9 | c.2333_2356del | p.778_786del | . | . |
| CTC4 | 19 | 57335003 | TC | T | 0.304 | NM_001146186 | PEG3 | frameshift_deleti  on | exon2 | c.438delG | p.R146fs | . | . |
| CTC4 | 19 | 57641288 | TG | T | 1 | NM_020903 | USP29 | frameshift_deleti  on | exon4 | c.1246delG | p.A416fs | . | . |
| CTC4 | 19 | 57647436 | AT | A | 0.316 | NM_052882 | ZIM3 | frameshift_deleti  on | exon5 | c.268delA | p.I90fs | . | . |
| CTC4 | 19 | 58579659 | GA | G | 0.481 | NM_007134 | ZNF135 | frameshift_deleti  on | exon4 | c.1880delA | p.E627fs | . | . |
| CTC4 | 19 | 58864383 | GT | G | 1 | NM_130786 | A1BG | frameshift_deleti  on | exon3 | c.250delA | p.T84fs | . | . |
| CTC4 | 19 | 59060447 | GC | G | 0.36 | NM_005762 | TRIM28 | frameshift_deleti  on | exon12 | c.1503delC | p.S501fs | . | . |
| CTC4 | 20 | 1187648 | CGA | C | 0.846 | NM_001009612 | C20orf202 | frameshift_deleti  on | exon2 | c.272_273del | p.R91fs | . | . |
| CTC4 | 20 | 2464015 | CA | C | 0.643 | NM_001282498 | ZNF343 | frameshift_deleti  on | exon4 | c.1321delT | p.C441fs | . | . |
| CTC4 | 20 | 3674190 | TG | T | 0.25 | NM_023068 | SIGLEC1 | frameshift_deleti  on | exon13 | c.3411delC | p.P1137fs | . | . |
| CTC4 | 20 | 3783830 | G | GA | 0.266 | NM_001287524 | CDC25B | frameshift_inserti  on | exon9 | c.758dupA | p.E253fs | . | . |
| CTC4 | 20 | 4158023 | C | CT | 0.844 | NM_001270691 | SMOX | frameshift_inserti  on | exon3 | c.234_235insT | p.A78fs | . | . |
| CTC4 | 20 | 25657230 | CTG | C | 0.217 | NM_015655 | ZNF337 | frameshift_deleti  on | exon5 | c.692_693del | p.T231fs | . | . |
| CTC4 | 20 | 31421626 | CA | C | 0.533 | NM_012325 | MAPRE1 | frameshift_deleti  on | exon3 | c.226delA | p.K76fs | . | . |
| CTC4 | 20 | 32211079 | TG | T | 0.198 | NM_001032999 | CBFA2T2 | frameshift_deleti  on | exon5 | c.670delG | p.G224fs | . | . |
| CTC4 | 20 | 32346541 | TC | T | 1 | NM_001282935 | ZNF341 | frameshift_deleti  on | exon6 | c.688delC | p.Q230fs | . | . |
| CTC4 | 20 | 33297078 | GC | G | 1 | NM_021202 | TP53INP2 | frameshift_deleti  on | exon4 | c.164delC | p.A55fs | . | . |
| CTC4 | 20 | 33297160 | AG | A | 0.676 | NM_021202 | TP53INP2 | frameshift_deleti  on | exon4 | c.246delG | p.E82fs | . | . |
| CTC4 | 20 | 33330016 | AT | A | 0.991 | NM_014071 | NCOA6 | frameshift_deleti  on | exon11 | c.4043delA | p.N1348fs | . | . |
| CTC4 | 20 | 33969805 | TG | T | 1 | NM_018244 | UQCC1 | frameshift_deleti  on | exon4 | c.248delC | p.P83fs | . | . |
| CTC4 | 20 | 34022064 | TG | T | 0.24 | NM_000557 | GDF5 | frameshift_deleti  on | exon2 | c.1148delC | p.P383fs | . | . |
| CTC4 | 20 | 34025498 | TG | T | 0.248 | NM_000557 | GDF5 | frameshift_deleti  on | exon1 | c.210delC | p.T70fs | . | . |
| CTC4 | 20 | 34528803 | CA | C | 0.919 | NM_016436 | PHF20 | frameshift_deleti  on | exon17 | c.2731delA | p.K911fs | . | . |
| CTC4 | 20 | 34763542 | GC | G | 1 | NM_001258331 | EPB41L1 | frameshift_deleti  on | exon3 | c.62delC | p.A21fs | . | . |
| CTC4 | 20 | 35242750 | AC | A | 0.294 | NM_175077 | SLA2 | frameshift_deleti  on | exon7 | c.572delG | p.C191fs | . | . |
| CTC4 | 20 | 35835736 | GA | G | 0.3 | NM_001135771 | RPN2 | frameshift_deleti  on | exon6 | c.656delA | p.E219fs | . | . |
| CTC4 | 20 | 37126146 | AC | A | 0.5 | NM_001282917 | RALGAPB | frameshift_deleti  on | exon4 | c.541delC | p.P181fs | . | . |
| CTC4 | 20 | 37128187 | GA | G | 0.253 | NM_001282917 | RALGAPB | frameshift_deleti  on | exon5 | c.652delA | p.K218fs | . | . |
| CTC4 | 20 | 44505517 | TG | T | 0.669 | NM_080752 | ZSWIM3 | frameshift_deleti  on | exon2 | c.321delG | p.V107fs | . | . |
| CTC4 | 20 | 44538163 | TC | T | 0.974 | NM_001242921 | PLTP | frameshift_deleti  on | exon3 | c.212delG | p.G71fs | . | . |
| CTC4 | 20 | 44581272 | TG | T | 0.514 | NM_022095 | ZNF335 | frameshift_deleti  on | exon19 | c.2778delC | p.I926fs | . | . |
| CTC4 | 20 | 44673696 | TG | T | 0.968 | NM_001134771 | SLC12A5 | frameshift_deleti  on | exon12 | c.1556delG | p.C519fs | . | . |
| CTC4 | 20 | 45865245 | TC | T | 0.889 | NM_001281771 | ZMYND8 | frameshift_deleti  on | exon15 | c.2224delG | p.E742fs | . | . |
| CTC4 | 20 | 48129757 | AG | A | 0.5 | NM_000961 | PTGIS | frameshift_deleti  on | exon8 | c.1065delC | p.P355fs | . | . |
| CTC4 | 20 | 50701657 | CG | C | 1 | NM_199427 | ZFP64 | frameshift_deleti  on | exon9 | c.1376delC | p.S459fs | . | . |
| CTC4 | 20 | 55108490 | AC | A | 1 | NM_001013646 | FAM209B | frameshift_deleti  on | exon1 | c.94delC | p.P32fs | . | . |
| CTC4 | 20 | 56227412 | TG | T | 0.667 | NM_001255976 | PMEPA1 | frameshift_deleti  on | exon4 | c.476delC | p.P159fs | . | . |
| CTC4 | 20 | 60498699 | CCG | C | 0.242 | NM_001252338 | CDH4 | frameshift_deleti  on | exon9 | c.1455_1456del | p.P485fs | . | . |
| CTC4 | 20 | 60498700 | CG | C | 0.911 | NM_001252338 | CDH4 | frameshift_deleti  on | exon9 | c.1456delG | p.G486fs | . | . |
| CTC4 | 20 | 60864293 | C | CT | 0.462 | NM_014835 | OSBPL2 | frameshift_inserti  on | exon12 | c.1121dupT | p.L374fs | . | . |
| CTC4 | 20 | 60885779 | TG | T | 1 | NM_005560 | LAMA5 | frameshift_deleti  on | exon75 | c.10387delC | p.Q3463fs | . | . |
| CTC4 | 20 | 60900522 | C | CA | 0.385 | NM_005560 | LAMA5 | frameshift_inserti  on | exon41 | c.5378dupT | p.L1793fs | . | . |
| CTC4 | 20 | 62854471 | AC | A | 0.896 | NM_004535 | MYT1 | frameshift_deleti  on | exon15 | c.2405delC | p.T802fs | . | . |
| CTC4 | 21 | 16340226 | TG | T | 0.583 | NM_003489 | NRIP1 | frameshift_deleti  on | exon4 | c.287delC | p.A96fs | . | . |
| CTC4 | 21 | 28296828 | CT | C | 0.979 | NM_007038 | ADAMTS5 | frameshift_deleti  on | exon8 | c.2336delA | p.K779fs | . | . |
| CTC4 | 21 | 30714911 | TA | T | 1 | NM_001186 | BACH1 | frameshift_deleti  on | exon5 | c.1969delA | p.K657fs | . | . |
| CTC4 | 21 | 31015162 | AT | A | 0.194 | NM_000830 | GRIK1 | stopgain | exon7 | c.1081delA | p.M361X | . | . |
| CTC4 | 21 | 31654985 | GA | G | 0.276 | NM_001085455 | KRTAP24-1 | frameshift_deleti on | exon1 | c.265delT | p.S89fs | . | . |
| CTC4 | 21 | 31744522 | T | TG | 0.35 | NM_181621 | KRTAP13-2 | frameshift_inserti  on | exon1 | c.9dupC | p.N4fs | . | . |
| CTC4 | 21 | 33063248 | TG | T | 1 | NM_001145444 | SCAF4 | frameshift_deleti  on | exon14 | c.1701delC | p.N567fs | . | . |
| CTC4 | 21 | 34037232 | A | AT | 0.538 | NM_001160306 | SYNJ1 | frameshift_inserti  on | exon17 | c.2279dupA | p.N760fs | . | . |
| CTC4 | 21 | 34923435 | CA | C | 0.228 | NM_032195 | SON | frameshift_deleti  on | exon3 | c.1899delA | p.P633fs | . | . |
| CTC4 | 21 | 38525559 | CA | C | 0.38 | NM_001001894 | TTC3 | frameshift_deleti  on | exon27 | c.2723delA | p.Q908fs | . | . |
| CTC4 | 21 | 40590534 | TA | T | 0.493 | NM_018963 | BRWD1 | frameshift_deleti  on | exon30 | c.3434delT | p.L1145fs | . | . |
| CTC4 | 21 | 46888193 | TG | T | 0.667 | NM_030582 | COL18A1 | frameshift_deleti  on | exon2 | c.685delG | p.G229fs | . | . |

| CTC4 | 21 | 46924425 | CGGCCC  CCCA | C | 1 | NM_030582 | COL18A1 | frameshift_deleti  on | exon33 | c.3364_3364del | p.G1122fs | . | . |
| --- | --- | --- | --- | --- | --- | --- | --- | --- | --- | --- | --- | --- | --- |
| CTC4 | 22 | 18121605 | TGCC | T | 0.31 | NM_001270726 | BCL2L13 | nonframeshift_de  letion | exon1 | c.147_149del | p.49_50del | . | . |
| CTC4 | 22 | 19379711 | CT | C | 0.41 | NM_003325 | HIRA | frameshift_deleti  on | exon9 | c.848delA | p.K283fs | . | . |
| CTC4 | 22 | 20918816 | ACAG | A | 0.152 | NM_001003891 | MED15 | nonframeshift_de  letion | exon6 | c.532_534del | p.178_178del | . | . |
| CTC4 | 22 | 20918915 | CCAG | C | 0.19 | NM_001003891 | MED15 | nonframeshift_de  letion | exon6 | c.631_633del | p.211_211del | . | . |
| CTC4 | 22 | 21105626 | GT | G | 0.552 | NM_058004 | PI4KA | frameshift_deleti  on | exon27 | c.3107delA | p.D1036fs | . | . |
| CTC4 | 22 | 24376903 | GTCC | G | 0.324 | NM_000853 | GSTT1 | nonframeshift_de  letion | exon4 | c.444_446del | p.148_149del | . | . |
| CTC4 | 22 | 26165029 | TGG | T | 0.24 | NM_032608 | MYO18B | frameshift_deleti  on | exon4 | c.1147_1148del | p.G383fs | . | . |
| CTC4 | 22 | 26892768 | CA | C | 0.94 | NM_012143 | TFIP11 | frameshift_deleti  on | exon11 | c.1523delT | p.L508fs | . | . |
| CTC4 | 22 | 26932377 | AG | A | 0.27 | NM_001008566 | TPST2 | frameshift_deleti  on | exon4 | c.917delC | p.P306fs | . | . |
| CTC4 | 22 | 29654822 | TG | T | 0.276 | NM_001267895 | EMID1 | frameshift_deleti  on | exon15 | c.1216delG | p.G406fs | . | . |
| CTC4 | 22 | 29660086 | CA | C | 0.357 | NM_012265 | RHBDD3 | frameshift_deleti  on | exon4 | c.269delT | p.L90fs | . | . |
| CTC4 | 22 | 30210661 | AC | A | 1 | NM_001242906 | ASCC2 | frameshift_deleti  on | exon6 | c.476delG | p.S159fs | . | . |
| CTC4 | 22 | 30412654 | CA | C | 0.615 | NM_021090 | MTMR3 | frameshift_deleti  on | exon15 | c.1642delA | p.K548fs | . | . |
| CTC4 | 22 | 31658714 | AC | A | 0.4 | NM_001031801 | LIMK2 | frameshift_deleti  on | exon6 | c.729delC | p.H243fs | . | . |
| CTC4 | 22 | 36886266 | CA | C | 0.974 | NM_001102371 | FOXRED2 | frameshift_deleti  on | exon9 | c.1843delT | p.C615fs | . | . |
| CTC4 | 22 | 38461095 | G | GA | 1 | NM_001039583 | PICK1 | frameshift_inserti  on | exon4 | c.241dupA | p.G80fs | . | . |
| CTC4 | 22 | 39085098 | GCC | G | 0.417 | NM_014876 | JOSD1 | frameshift_deleti  on | exon3 | c.349_350del | p.G117fs | . | . |
| CTC4 | 22 | 40045802 | TG | T | 1 | NM_001003406 | CACNA1I | frameshift_deleti  on | exon9 | c.1760delG | p.C587fs | . | . |
| CTC4 | 22 | 40807713 | AG | A | 0.344 | NM_001282660 | MKL1 | frameshift_deleti  on | exon12 | c.2476delC | p.L826fs | . | . |
| CTC4 | 22 | 41222589 | TA | T | 0.842 | NM_001278589 | ST13 | frameshift_deleti  on | exon12 | c.1032delT | p.V344fs | . | . |
| CTC4 | 22 | 42998783 | GT | G | 1 | NM_001278657 | POLDIP3 | frameshift_deleti  on | exon2 | c.442delA | p.T148fs | . | . |
| CTC4 | 22 | 43089461 | CAG | C | 0.224 | NM_017436 | A4GALT | frameshift_deleti  on | exon3 | c.495_496del | p.R165fs | . | . |
| CTC4 | 22 | 43950846 | TC | T | 1 | NM_198856 | EFCAB6 | frameshift_deleti  on | exon25 | c.3094delG | p.E1032fs | . | . |
| CTC4 | 22 | 43972227 | A | AG | 0.5 | NM_198856 | EFCAB6 | frameshift_inserti  on | exon24 | c.2913dupC | p.Y972fs | . | . |
| CTC4 | 22 | 46704360 | CG | C | 0.412 | NM_016426 | GTSE1 | frameshift_deleti  on | exon4 | c.283delG | p.G95fs | . | . |
| CTC4 | 22 | 50617508 | G | GC | 0.32 | NM_001160300 | PANX2 | frameshift_inserti  on | exon3 | c.1837dupC | p.E612fs | . | . |
| CTC4 | 22 | 50617638 | GC | G | 0.438 | NM_052839 | PANX2 | frameshift_deleti  on | exon3 | c.1967delC | p.A656fs | . | . |
| CTC4 | X | 322180 | TG | T | 0.824 | NM_013239 | PPP2R3B | frameshift_deleti  on | exon2 | c.469delC | p.H157fs | . | . |
| CTC4 | X | 2853110 | AG | A | 0.667 | NM_001282631 | ARSE | frameshift_deleti  on | exon10 | c.1397delC | p.P466fs | . | . |
| CTC4 | X | 19009131 | TA | T | 0.571 | NM_001184835 | GPR64 | frameshift_deleti  on | exon26 | c.2790delT | p.F930fs | . | . |
| CTC4 | X | 19418722 | TC | T | 0.262 | NM_001001671 | MAP3K15 | frameshift_deleti  on | exon14 | c.1903delG | p.E635fs | . | . |
| CTC4 | X | 20043806 | GT | G | 1 | NM_001168466 | MAP7D2 | frameshift_deleti  on | exon7 | c.1013delA | p.N338fs | . | . |
| CTC4 | X | 23410821 | A | AT | 1 | NM_173495 | PTCHD1 | frameshift_inserti  on | exon3 | c.1187dupT | p.I396fs | . | . |
| CTC4 | X | 37312718 | TC | T | 0.5 | NM_001142395 | PRRG1 | frameshift_deleti  on | exon4 | c.502delC | p.P168fs | . | . |
| CTC4 | X | 39922257 | CT | C | 1 | NM_001123384 | BCOR | frameshift_deleti  on | exon8 | c.3758delA | p.K1253fs | . | . |
| CTC4 | X | 39922985 | AG | A | 1 | NM_001123384 | BCOR | frameshift_deleti  on | exon7 | c.3566delC | p.T1189fs | . | . |
| CTC4 | X | 39922986 | GT | G | 1 | NM_001123384 | BCOR | frameshift_deleti  on | exon7 | c.3565delA | p.T1189fs | . | . |
| CTC4 | X | 41048601 | GT | G | 0.266 | NM_001039590 | USP9X | frameshift_deleti  on | exon26 | c.3851delT | p.V1284fs | . | . |
| CTC4 | X | 47271802 | TG | T | 0.489 | NM_003446 | ZNF157 | frameshift_deleti  on | exon4 | c.331delG | p.G111fs | . | . |
| CTC4 | X | 47307610 | TC | T | 1 | NM_007130 | ZNF41 | frameshift_deleti  on | exon5 | c.1558delG | p.E520fs | . | . |
| CTC4 | X | 47308193 | GT | G | 0.632 | NM_007130 | ZNF41 | frameshift_deleti  on | exon5 | c.975delA | p.K325fs | . | . |
| CTC4 | X | 48542805 | GC | G | 0.667 | NM_000377 | WAS | frameshift_deleti  on | exon2 | c.267delC | p.G89fs | . | . |
| CTC4 | X | 48978765 | CG | C | 0.748 | NM_015698 | GPKOW | frameshift_deleti  on | exon3 | c.438delC | p.S146fs | . | . |
| CTC4 | X | 49113932 | TG | T | 0.647 | NM_001114377 | FOXP3 | frameshift_deleti  on | exon3 | c.300delC | p.T100fs | . | . |
| CTC4 | X | 53578320 | TG | T | 0.3 | NM_031407 | HUWE1 | frameshift_deleti  on | exon64 | c.9002delC | p.T3001fs | . | . |
| CTC4 | X | 54012346 | C | CT | 1 | NM_001184897 | PHF8 | frameshift_inserti  on | exon16 | c.1728_1729ins  A | p.V577fs | . | . |
| CTC4 | X | 54160329 | TG | T | 1 | NM_017848 | FAM120C | frameshift_deleti  on | exon8 | c.1766delC | p.T589fs | . | . |
| CTC4 | X | 65819921 | GA | G | 0.469 | NM_001242310 | EDA2R | frameshift_deleti  on | exon5 | c.533delT | p.F178fs | . | . |
| CTC4 | X | 70145687 | GC | G | 1 | NM_001048164 | SLC7A3 | frameshift_deleti  on | exon12 | c.1835delG | p.G612fs | . | . |
| CTC4 | X | 70360647 | ACAG | A | 0.292 | NM_005120 | MED12 | nonframeshift_de  letion | exon42 | c.6208_6210del | p.2070_2070de  l | . | . |
| CTC4 | X | 70360679 | GGCA | G | 0.172 | NM_005120 | MED12 | nonframeshift_de  letion | exon42 | c.6240_6242del | p.2080_2081de  l | . | . |
| CTC4 | X | 84559367 | GA | G | 0.286 | NM_024921 | POF1B | frameshift_deleti  on | exon14 | c.1547delT | p.L516fs | . | . |
| CTC4 | X | 99551520 | GGGA | G | 0.265 | NM_001105243 | PCDH19 | nonframeshift_de  letion | exon5 | c.3058_3060del | p.1020_1020de  l | . | . |
| CTC4 | X | 100611809 | AC | A | 1 | NM_000061 | BTK | frameshift_deleti  on | exon14 | c.1311delG | p.M437fs | . | . |
| CTC4 | X | 101912485 | GT | G | 0.7 | NM_001099411 | GPRASP1 | frameshift_deleti  on | exon3 | c.3645delT | p.S1215fs | . | . |
| CTC4 | X | 105132326 | AG | A | 0.426 | NM_198465 | NRK | frameshift_deleti  on | exon5 | c.293delG | p.R98fs | . | . |

| CTC4 | X | 106084048 | C | CA | 0.222 | NM_017752 | TBC1D8B | frameshift_inserti  on | exon10 | c.1654dupA | p.G551fs | . | . |
| --- | --- | --- | --- | --- | --- | --- | --- | --- | --- | --- | --- | --- | --- |
| CTC4 | X | 109441773 | AG | A | 0.898 | NM_001025580 | AMMECR1 | frameshift_deleti  on | exon5 | c.865delC | p.L289fs | . | . |
| CTC4 | X | 112058776 | TGCTGT  G | T | 1 | NM_001113490 | AMOT | nonframeshift_de  letion | exon2 | c.1196_1201del | p.399_401del | . | . |
| CTC4 | X | 112058779 | TGTG | T | 1 | NM_001113490 | AMOT | nonframeshift_de  letion | exon2 | c.1196_1198del | p.399_400del | . | . |
| CTC4 | X | 123785959 | GT | G | 0.276 | NM_001163278 | TENM1 | frameshift_deleti  on | exon8 | c.1383delA | p.K461fs | . | . |
| CTC4 | X | 127185740 | GC | G | 0.294 | NM_138289 | ACTRT1 | frameshift_deleti  on | exon1 | c.445delG | p.A149fs | . | . |
| CTC4 | X | 129484622 | G | GA | 0.972 | NM_001282198 | SLC25A14 | frameshift_inserti  on | exon5 | c.311dupA | p.E104fs | . | . |
| CTC4 | X | 134989961 | GC | G | 1 | NM_018666 | SAGE1 | frameshift_deleti  on | exon10 | c.1121delC | p.A374fs | . | . |
| CTC4 | X | 135405114 | TG | T | 0.442 | NM_153834 | GPR112 | frameshift_deleti  on | exon5 | c.249delG | p.L83fs | . | . |
| CTC4 | X | 138643911 | G | GA | 0.375 | NM_000133 | F9 | stopgain | exon8 | c.1067_1068ins  A | p.W356_G357d  elinsX | . | . |
| CTC4 | X | 153133004 | AT | A | 0.259 | NM_001143963 | L1CAM | frameshift_deleti  on | exon15 | c.1928delA | p.Y643fs | . | . |
| CTC4 | X | 153215890 | GC | G | 0.316 | NM_005334 | HCFC1 | frameshift_deleti  on | exon24 | c.5807delG | p.G1936fs | . | . |
| CTC4 | X | 153296647 | CT | C | 0.358 | NM_001110792 | MECP2 | frameshift_deleti  on | exon3 | c.667delA | p.R223fs | . | . |
| CTC4 | X | 153296896 | TG | T | 1 | NM_001110792 | MECP2 | frameshift_deleti  on | exon3 | c.418delC | p.Q140fs | . | . |
| CTC5 | 1 | 985956 | GGGC | G | 0.989 | NM_198576 | AGRN | nonframeshift_de  letion | exon29 | c.5127_5129del | p.1709_1710de  l | . | . |
| CTC5 | 1 | 3551607 | A | AT | 0.545 | NM_017818 | WRAP73 | frameshift_inserti  on | exon8 | c.769dupA | p.M257fs | . | . |
| CTC5 | 1 | 6680014 | TG | T | 1 | NM_153812 | PHF13 | frameshift_deleti  on | exon3 | c.294delG | p.L98fs | . | . |
| CTC5 | 1 | 7792607 | GC | G | 0.581 | NM_015215 | CAMTA1 | frameshift_deleti  on | exon12 | c.3015delC | p.G1005fs | . | . |
| CTC5 | 1 | 8418396 | CG | C | 0.667 | NM_001042682 | RERE | frameshift_deleti  on | exon10 | c.2536delC | p.R846fs | . | . |
| CTC5 | 1 | 8421515 | C | CA | 0.857 | NM_001042682 | RERE | frameshift_inserti  on | exon8 | c.389_390insT | p.E130fs | . | . |
| CTC5 | 1 | 9165578 | CG | C | 0.32 | NM_024980 | GPR157 | frameshift_deleti  on | exon3 | c.758delC | p.P253fs | . | . |
| CTC5 | 1 | 11077030 | AT | A | 0.444 | NM_007375 | TARDBP | frameshift_deleti  on | exon3 | c.369delT | p.Y123fs | . | . |
| CTC5 | 1 | 11087063 | CA | C | 0.429 | NM_006610 | MASP2 | frameshift_deleti  on | exon11 | c.1939delT | p.W647fs | . | . |
| CTC5 | 1 | 11128711 | CG | C | 0.609 | NM_002685 | EXOSC10 | frameshift_deleti  on | exon22 | c.2465delC | p.P822fs | . | . |
| CTC5 | 1 | 11579531 | TC | T | 0.833 | NM_020780 | PTCHD2 | frameshift_deleti  on | exon8 | c.2010delC | p.I670fs | . | . |
| CTC5 | 1 | 11856289 | TG | T | 1 | NM_005957 | MTHFR | frameshift_deleti  on | exon5 | c.753delC | p.P251fs | . | . |
| CTC5 | 1 | 12919145 | GC | G | 0.326 | NM_023014 | PRAMEF2 | frameshift_deleti  on | exon2 | c.282delC | p.R94fs | . | . |
| CTC5 | 1 | 14106394 | A | ACTC | 0.889 | NM_001007257 | PRDM2 | nonframeshift_in  sertion | exon3 | c.1501_1502ins  CTC | p.T501delinsTP | . | . |
| CTC5 | 1 | 14106619 | AG | A | 0.667 | NM_001007257 | PRDM2 | frameshift_deleti  on | exon3 | c.1727delG | p.S576fs | . | . |
| CTC5 | 1 | 15655850 | GA | G | 0.875 | NM_052929 | FHAD1 | frameshift_deleti  on | exon14 | c.1720delA | p.K574fs | . | . |
| CTC5 | 1 | 16260450 | CG | C | 0.25 | NM_015001 | SPEN | frameshift_deleti  on | exon11 | c.7716delG | p.P2572fs | . | . |
| CTC5 | 1 | 16531593 | AG | A | 0.183 | NM_153213 | ARHGEF19 | frameshift_deleti  on | exon11 | c.1734delC | p.H578fs | . | . |
| CTC5 | 1 | 19473365 | TG | T | 0.684 | NM_020765 | UBR4 | frameshift_deleti  on | exon52 | c.7758delC | p.N2586fs | . | . |
| CTC5 | 1 | 22157735 | GC | G | 0.311 | NM_005529 | HSPG2 | frameshift_deleti  on | exon84 | c.11534delG | p.C3845fs | . | . |
| CTC5 | 1 | 22202437 | AC | A | 0.298 | NM_005529 | HSPG2 | frameshift_deleti  on | exon24 | c.3101delG | p.G1034fs | . | . |
| CTC5 | 1 | 22835199 | TG | T | 1 | NM_014870 | ZBTB40 | frameshift_deleti  on | exon8 | c.1675delG | p.G559fs | . | . |
| CTC5 | 1 | 22838560 | CAAG | C | 0.5 | NM_014870 | ZBTB40 | nonframeshift_de  letion | exon11 | c.2395_2397del | p.799_799del | . | . |
| CTC5 | 1 | 23233342 | GT | G | 0.275 | NM_004442 | EPHB2 | frameshift_deleti  on | exon11 | c.2032delT | p.F678fs | . | . |
| CTC5 | 1 | 23758301 | TGG | T | 0.722 | NM_001143778 | ASAP3 | frameshift_deleti  on | exon22 | c.2405_2406del | p.P802fs | . | . |
| CTC5 | 1 | 24397608 | GGC | G | 0.5 | NM_152372 | MYOM3 | frameshift_deleti  on | exon25 | c.3147_3148del | p.S1049fs | . | . |
| CTC5 | 1 | 24418722 | TC | T | 0.792 | NM_152372 | MYOM3 | frameshift_deleti  on | exon11 | c.1173delG | p.L391fs | . | . |
| CTC5 | 1 | 26366312 | GCC | G | 0.463 | NM_032513 | SLC30A2 | frameshift_deleti  on | exon6 | c.780_781del | p.W260fs | . | . |
| CTC5 | 1 | 26595119 | GC | G | 0.571 | NM_001281517 | CEP85 | frameshift_deleti  on | exon7 | c.1335delC | p.S445fs | . | . |
| CTC5 | 1 | 26608853 | GCCGGG  A | G | 0.902 | NM_001077262 | UBXN11 | nonframeshift_de  letion | exon11 | c.1134_1139del | p.378_380del | . | . |
| CTC5 | 1 | 26887256 | GT | G | 0.389 | NM_001006665 | RPS6KA1 | frameshift_deleti  on | exon14 | c.1283delT | p.V428fs | . | . |
| CTC5 | 1 | 27100114 | GC | G | 0.281 | NM_006015 | ARID1A | frameshift_deleti  on | exon16 | c.3911delC | p.A1304fs | . | . |
| CTC5 | 1 | 27268295 | TC | T | 1 | NM_006600 | NUDC | frameshift_deleti  on | exon4 | c.416delC | p.S139fs | . | . |
| CTC5 | 1 | 27428567 | CT | C | 0.5 | NM_003047 | SLC9A1 | frameshift_deleti  on | exon9 | c.1874delA | p.K625fs | . | . |
| CTC5 | 1 | 27720880 | A | AT | 0.774 | NM_005281 | GPR3 | frameshift_inserti  on | exon2 | c.578_579insT | p.N193fs | . | . |
| CTC5 | 1 | 29651796 | CAA | C | 0.265 | NM_001195001 | PTPRU | frameshift_deleti  on | exon29 | c.4198_4199del | p.K1400fs | . | . |
| CTC5 | 1 | 31819635 | AC | A | 0.417 | NM_001282567 | ZCCHC17 | frameshift_deleti  on | exon5 | c.395delC | p.T132fs | . | . |
| CTC5 | 1 | 31905889 | A | ACAG | 0.875 | NM_001199037 | SERINC2 | nonframeshift_in  sertion | exon9 | c.1101_1102ins  CAG | p.T367delinsTQ | . | . |
| CTC5 | 1 | 32680517 | AC | A | 0.228 | NM_001099434 | DCDC2B | frameshift_deleti  on | exon7 | c.835delC | p.L279fs | . | . |
| CTC5 | 1 | 33236452 | TG | T | 0.2 | NM_001198972 | KIAA1522 | frameshift_deleti  on | exon6 | c.1496delG | p.W499fs | . | . |
| CTC5 | 1 | 34035115 | TG | T | 0.722 | NM_001281956 | CSMD2 | frameshift_deleti  on | exon52 | c.7989delC | p.P2663fs | . | . |
| CTC5 | 1 | 35251063 | AG | A | 0.333 | NM_001005752 | GJB3 | frameshift_deleti  on | exon2 | c.701delG | p.S234fs | . | . |
| CTC5 | 1 | 36319131 | C | CT | 0.347 | NM_017629 | AGO4 | frameshift_inserti  on | exon18 | c.2511_2512ins  T | p.N837fs | . | . |

| CTC5 | 1 | 38005967 | G | GA | 0.583 | NM_024700 | SNIP1 | frameshift_inserti  on | exon3 | c.716dupT | p.F239fs | . | . |
| --- | --- | --- | --- | --- | --- | --- | --- | --- | --- | --- | --- | --- | --- |
| CTC5 | 1 | 39776501 | AGAG | A | 0.286 | NM_012090 | MACF1 | frameshift_deleti  on | exon25 | c.3116_3117del | p.E1039fs | . | . |
| CTC5 | 1 | 39824539 | AAC | A | 0.63 | NM_012090 | MACF1 | frameshift_deleti  on | exon40 | c.5929_5930del | p.T1977fs | . | . |
| CTC5 | 1 | 39853542 | CA | C | 1 | NM_012090 | MACF1 | frameshift_deleti  on | exon52 | c.8843delA | p.Q2948fs | . | . |
| CTC5 | 1 | 40214693 | TG | T | 0.437 | NM_001195007 | PPIE | frameshift_deleti  on | exon8 | c.628delG | p.G210fs | . | . |
| CTC5 | 1 | 40688299 | TG | T | 0.6 | NM_012421 | RLF | frameshift_deleti  on | exon6 | c.865delG | p.E289fs | . | . |
| CTC5 | 1 | 40928734 | GA | G | 0.385 | NM_023070 | ZFP69B | frameshift_deleti  on | exon5 | c.1079delA | p.E360fs | . | . |
| CTC5 | 1 | 41475151 | TG | T | 0.308 | NM_001905 | CTPS1 | frameshift_deleti  on | exon17 | c.1582delG | p.E528fs | . | . |
| CTC5 | 1 | 41976335 | AC | A | 1 | NM_001127714 | HIVEP3 | frameshift_deleti  on | exon8 | c.7004delG | p.R2335fs | . | . |
| CTC5 | 1 | 43685093 | TA | T | 0.588 | NM_001195831 | WDR65 | frameshift_deleti  on | exon13 | c.2133delA | p.L711fs | . | . |
| CTC5 | 1 | 43803571 | CA | C | 0.535 | NM_005373 | MPL | frameshift_deleti  on | exon1 | c.53delA | p.Q18fs | . | . |
| CTC5 | 1 | 43896361 | CT | C | 0.238 | NM_015284 | SZT2 | frameshift_deleti  on | exon31 | c.4505delT | p.L1502fs | . | . |
| CTC5 | 1 | 43903271 | TA | T | 0.3 | NM_015284 | SZT2 | frameshift_deleti  on | exon44 | c.6126delA | p.L2042fs | . | . |
| CTC5 | 1 | 44085208 | AG | A | 0.281 | NM_130440 | PTPRF | frameshift_deleti  on | exon27 | c.4870delG | p.G1624fs | . | . |
| CTC5 | 1 | 45813597 | CG | C | 0.588 | NM_007170 | TESK2 | frameshift_deleti  on | exon6 | c.615delC | p.P205fs | . | . |
| CTC5 | 1 | 46751512 | CT | C | 0.421 | NM_006369 | LRRC41 | frameshift_deleti  on | exon4 | c.1016delA | p.K339fs | . | . |
| CTC5 | 1 | 47124359 | CT | C | 1 | NM_001042546 | ATPAF1 | frameshift_deleti  on | exon3 | c.494delA | p.K165fs | . | . |
| CTC5 | 1 | 47610223 | TG | T | 0.904 | NM_001010969 | CYP4A22 | frameshift_deleti  on | exon8 | c.900delG | p.M300fs | . | . |
| CTC5 | 1 | 51756192 | CT | C | 0.848 | NM_001080494 | TTC39A | frameshift_deleti  on | exon15 | c.1374delA | p.K458fs | . | . |
| CTC5 | 1 | 52248266 | CAA | C | 0.682 | NM_148905 | OSBPL9 | frameshift_deleti  on | exon12 | c.931_932del | p.K311fs | . | . |
| CTC5 | 1 | 52824122 | TG | T | 0.789 | NM_032449 | CC2D1B | frameshift_deleti  on | exon13 | c.1341delC | p.P447fs | . | . |
| CTC5 | 1 | 53370472 | AC | A | 0.848 | NM_001198962 | ECHDC2 | frameshift_deleti  on | exon6 | c.454delG | p.V152fs | . | . |
| CTC5 | 1 | 53732230 | TG | T | 0.636 | NM_033300 | LRP8 | frameshift_deleti  on | exon7 | c.831delC | p.P277fs | . | . |
| CTC5 | 1 | 55637286 | TG | T | 0.923 | NM_015306 | USP24 | frameshift_deleti  on | exon5 | c.767delC | p.A256fs | . | . |
| CTC5 | 1 | 57372446 | AT | A | 0.216 | NM_000562 | C8A | frameshift_deleti  on | exon8 | c.1204delT | p.F402fs | . | . |
| CTC5 | 1 | 62160427 | CA | C | 0.45 | NM_032027 | TM2D1 | frameshift_deleti  on | exon5 | c.454delT | p.C152fs | . | . |
| CTC5 | 1 | 63944478 | TG | T | 1 | NM_014288 | ITGB3BP | frameshift_deleti  on | exon4 | c.210delC | p.P70fs | . | . |
| CTC5 | 1 | 65858397 | G | GT | 1 | NM_001256864 | DNAJC6 | frameshift_inserti  on | exon12 | c.1753dupT | p.L584fs | . | . |
| CTC5 | 1 | 67145379 | CA | C | 0.435 | NM_032291 | SGIP1 | frameshift_deleti  on | exon14 | c.759delA | p.P253fs | . | . |
| CTC5 | 1 | 67236143 | GA | G | 0.366 | NM_152665 | TCTEX1D1 | frameshift_deleti  on | exon3 | c.194delA | p.E65fs | . | . |
| CTC5 | 1 | 67337110 | TG | T | 0.24 | NM_024763 | WDR78 | frameshift_deleti  on | exon6 | c.882delC | p.F294fs | . | . |
| CTC5 | 1 | 67559225 | CA | C | 0.214 | NM_001276351 | C1orf141 | stopgain | exon8 | c.665delT | p.L222X | . | . |
| CTC5 | 1 | 78329613 | AT | A | 0.269 | NM_001270384 | FAM73A | frameshift_deleti on | exon13 | c.1410delT | p.D470fs | . | . |
| CTC5 | 1 | 78426137 | TG | T | 0.526 | NM_003902 | FUBP1 | frameshift_deleti  on | exon15 | c.1387delC | p.H463fs | . | . |
| CTC5 | 1 | 84376918 | A | AT | 0.393 | NM_024686 | TTLL7 | frameshift_inserti  on | exon15 | c.1715dupA | p.N572fs | . | . |
| CTC5 | 1 | 87045895 | ATCCTA  C | A | 0.995 | NM_012128 | CLCA4 | nonframeshift_de  letion | exon14 | c.2628_2633del | p.876_878del | . | . |
| CTC5 | 1 | 87045896 | TCCTAC  A | T | 0.994 | NM_012128 | CLCA4 | nonframeshift_de  letion | exon14 | c.2629_2634del | p.877_878del | . | . |
| CTC5 | 1 | 90048386 | AG | A | 0.882 | NM_015350 | LRRC8B | frameshift_deleti  on | exon5 | c.178delG | p.V60fs | . | . |
| CTC5 | 1 | 91404388 | CA | C | 1 | NM_201269 | ZNF644 | frameshift_deleti  on | exon3 | c.2522delT | p.L841fs | . | . |
| CTC5 | 1 | 93621965 | GA | G | 1 | NM_001167830 | TMED5 | frameshift_deleti  on | exon3 | c.362delT | p.F121fs | . | . |
| CTC5 | 1 | 94481330 | GT | G | 0.537 | NM_000350 | ABCA4 | frameshift_deleti  on | exon37 | c.5276delA | p.N1759fs | . | . |
| CTC5 | 1 | 94941223 | AC | A | 0.667 | NM_001122674 | ABCD3 | frameshift_deleti  on | exon7 | c.558delC | p.D186fs | . | . |
| CTC5 | 1 | 100964588 | ACAGC | A | 0.612 | NM_003672 | CDC14A | frameshift_deleti  on | exon15 | c.1526_1529del | p.T509fs | . | . |
| CTC5 | 1 | 104122096 | GC | G | 0.583 | NM_020978 | AMY2B | frameshift_deleti  on | exon12 | c.1511delC | p.A504fs | . | . |
| CTC5 | 1 | 110257856 | CT | C | 0.286 | NM_000851 | GSTM5 | frameshift_deleti  on | exon7 | c.562delT | p.F188fs | . | . |
| CTC5 | 1 | 110884184 | TA | T | 0.833 | NM_001201545 | RBM15 | frameshift_deleti  on | exon1 | c.2158delA | p.K720fs | . | . |
| CTC5 | 1 | 111957501 | CTCACA  GACTGA TGACTC | C | 1 | NM_002557 | OVGP1 | nonframeshift_de letion | exon11 | c.1598_1621del | p.533_541del | . | . |
| CTC5 | 1 | 112308925 | CA | C | 0.28 | NM_007204 | DDX20 | frameshift_deleti  on | exon11 | c.1880delA | p.Q627fs | . | . |
| CTC5 | 1 | 113239101 | CT | C | 0.458 | NM_001130079 | MOV10 | frameshift_deleti  on | exon13 | c.1927delT | p.F643fs | . | . |
| CTC5 | 1 | 117122490 | AG | A | 0.305 | NM_001007237 | IGSF3 | frameshift_deleti  on | exon10 | c.2857delC | p.L953fs | . | . |
| CTC5 | 1 | 117559754 | TG | T | 0.385 | NM_001256106 | CD101 | frameshift_deleti  on | exon5 | c.1272delG | p.V424fs | . | . |
| CTC5 | 1 | 119964595 | CA | C | 1 | NM_000198 | HSD3B2 | frameshift_deleti  on | exon4 | c.472delA | p.K158fs | . | . |
| CTC5 | 1 | 120502102 | CA | C | 0.565 | NM_001200001 | NOTCH2 | frameshift_deleti  on | exon12 | c.1938delT | p.F646fs | . | . |
| CTC5 | 1 | 120927190 | CT | C | 0.395 | NM_001004340 | FCGR1B | frameshift_deleti  on | exon3 | c.513delA | p.K171fs | . | . |
| CTC5 | 1 | 144877212 | AT | A | 0.542 | NM_001198834 | PDE4DIP | frameshift_deleti  on | exon28 | c.4474delA | p.I1492fs | . | . |
| CTC5 | 1 | 144886321 | CT | C | 0.197 | NM_001198834 | PDE4DIP | frameshift_deleti  on | exon23 | c.2912delA | p.K971fs | . | . |
| CTC5 | 1 | 145439821 | TA | T | 0.541 | NM_006472 | TXNIP | frameshift_deleti  on | exon3 | c.368delA | p.Y123fs | . | . |

| CTC5 | 1 | 145532173 | GT | G | 0.606 | NM_003637 | ITGA10 | frameshift_deleti  on | exon8 | c.818delT | p.V273fs | . | . |
| --- | --- | --- | --- | --- | --- | --- | --- | --- | --- | --- | --- | --- | --- |
| CTC5 | 1 | 145556601 | GT | G | 0.303 | NM_144698 | ANKRD35 | frameshift_deleti  on | exon3 | c.172delT | p.F58fs | . | . |
| CTC5 | 1 | 146465883 | AC | A | 1 | NM_001278141 | NBPF12 | frameshift_deleti  on | exon37 | c.4137delC | p.N1379fs | . | . |
| CTC5 | 1 | 146466055 | CT | C | 0.571 | NM_001278141 | NBPF12 | frameshift_deleti  on | exon37 | c.4309delT | p.F1437fs | . | . |
| CTC5 | 1 | 147092771 | CAA | C | 0.25 | NM_004326 | BCL9 | frameshift_deleti  on | exon8 | c.2811_2812del | p.P937fs | . | . |
| CTC5 | 1 | 150199039 | CTCCTCT | C | 1 | NM_001136478 | ANP32E | nonframeshift_de  letion | exon4 | c.453_458del | p.151_153del | . | . |
| CTC5 | 1 | 150316999 | AG | A | 0.478 | NM_004698 | PRPF3 | frameshift_deleti  on | exon12 | c.1617delG | p.Q539fs | . | . |
| CTC5 | 1 | 150337280 | AT | A | 1 | NM_015203 | RPRD2 | frameshift_deleti  on | exon1 | c.91delT | p.F31fs | . | . |
| CTC5 | 1 | 150936108 | AG | A | 0.339 | NM_001145415 | SETDB1 | frameshift_deleti  on | exon20 | c.3561delG | p.K1187fs | . | . |
| CTC5 | 1 | 151105971 | TG | T | 0.5 | NM_001178062 | SEMA6C | frameshift_deleti  on | exon19 | c.1757delC | p.P586fs | . | . |
| CTC5 | 1 | 151259097 | TG | T | 0.288 | NM_020832 | ZNF687 | frameshift_deleti  on | exon2 | c.331delG | p.G111fs | . | . |
| CTC5 | 1 | 151263407 | TC | T | 0.464 | NM_020832 | ZNF687 | frameshift_deleti  on | exon9 | c.3437delC | p.S1146fs | . | . |
| CTC5 | 1 | 151274738 | TG | T | 0.38 | NM_001198775 | PI4KB | frameshift_deleti  on | exon5 | c.560delC | p.T187fs | . | . |
| CTC5 | 1 | 151342235 | AC | A | 0.44 | NM_001258288 | SELENBP1 | frameshift_deleti  on | exon2 | c.14delG | p.C5fs | . | . |
| CTC5 | 1 | 151372112 | GC | G | 0.75 | NM_002796 | PSMB4 | frameshift_deleti  on | exon1 | c.50delC | p.A17fs | . | . |
| CTC5 | 1 | 151774724 | CA | C | 0.56 | NM_001004432 | LINGO4 | frameshift_deleti  on | exon2 | c.456delT | p.F152fs | . | . |
| CTC5 | 1 | 152195728 | AT | A | 0.906 | NM_001009931 | HRNR | frameshift_deleti  on | exon2 | c.1delA | p.M1fs | . | . |
| CTC5 | 1 | 152285430 | GT | G | 0.826 | NM_002016 | FLG | frameshift_deleti  on | exon3 | c.1931delA | p.N644fs | . | . |
| CTC5 | 1 | 152287861 | GT | G | 1 | NM_002016 | FLG | frameshift_deleti  on | exon2 | c.71delA | p.N24fs | . | . |
| CTC5 | 1 | 152659444 | CT | C | 0.4 | NM_014357 | LCE2B | frameshift_deleti  on | exon2 | c.126delT | p.P42fs | . | . |
| CTC5 | 1 | 152681680 | C | CAGCT  CTGGG GGCTG | 0.571 | NM_178356 | LCE4A | nonframeshift_in sertion | exon1 | c.129_130insA  GCTCTGGGGG CTGCTGT | p.S43delinsSSS GGCC | . | . |
| CTC5 | 1 | 152800040 | CA | C | 0.387 | NM_178348 | LCE1A | frameshift_deleti  on | exon1 | c.93delA | p.P31fs | . | . |
| CTC5 | 1 | 153904904 | GC | G | 0.333 | NM_014856 | DENND4B | frameshift_deleti  on | exon24 | c.3771delG | p.R1257fs | . | . |
| CTC5 | 1 | 154516545 | G | GT | 0.331 | NM_001098475 | TDRD10 | frameshift_inserti  on | exon9 | c.611dupT | p.V204fs | . | . |
| CTC5 | 1 | 154842199 | G | GGCTG  CTGCT | 1 | NM_001204087 | KCNN3 | nonframeshift_in  sertion | exon1 | c.241_242insA  GCAGCAGCAG | p.P81delinsQQ  QQP | . | . |
| CTC5 | 1 | 154842206 | G | GCTGC  TGCTG | 1 | NM_001204087 | KCNN3 | nonframeshift_in  sertion | exon1 | c.234_235insG  AGCAGCAGCA | p.Q79delinsEQ  QQQ | . | . |
| CTC5 | 1 | 154842206 | G | GCTGC  TGCTG | 1 | NM_001204087 | KCNN3 | nonframeshift_in  sertion | exon1 | c.234_235insG  AGCAGCAGCA | p.Q79delinsEQ  QQQQ | . | . |
| CTC5 | 1 | 154842330 | TTGC | T | 0.185 | NM_001204087 | KCNN3 | nonframeshift_de  letion | exon1 | c.108_110del | p.36_37del | . | . |
| CTC5 | 1 | 154920147 | CG | C | 0.899 | NM_020524 | PBXIP1 | frameshift_deleti  on | exon8 | c.708delC | p.P236fs | . | . |
| CTC5 | 1 | 155160035 | GA | G | 0.75 | NM_001044390 | MUC1 | frameshift_deleti  on | exon5 | c.282delT | p.F94fs | . | . |
| CTC5 | 1 | 155208353 | GA | G | 0.357 | NM_001171811 | GBA | frameshift_deleti  on | exon4 | c.281delT | p.F94fs | . | . |
| CTC5 | 1 | 155451412 | TC | T | 0.388 | NM_018489 | ASH1L | frameshift_deleti  on | exon3 | c.1248delG | p.L416fs | . | . |
| CTC5 | 1 | 155491255 | GA | G | 0.75 | NM_018489 | ASH1L | frameshift_deleti  on | exon2 | c.55delT | p.S19fs | . | . |
| CTC5 | 1 | 155887399 | TG | T | 0.405 | NM_014949 | KIAA0907 | frameshift_deleti  on | exon11 | c.1330delC | p.Q444fs | . | . |
| CTC5 | 1 | 156046686 | A | AGAG | 0.258 | NM_001093725 | MEX3A | nonframeshift_in  sertion | exon2 | c.1241_1242ins  CTC | p.S414delinsSS | . | . |
| CTC5 | 1 | 156144962 | TC | T | 0.408 | NM_001193302 | SEMA4A | frameshift_deleti  on | exon11 | c.1125delC | p.V375fs | . | . |
| CTC5 | 1 | 156565049 | A | AAC | 1 | NM_015590 | GPATCH4 | frameshift_inserti  on | exon8 | c.1083_1084ins  GT | p.F362fs | . | . |
| CTC5 | 1 | 156812958 | GC | G | 1 | NM_014215 | INSRR | frameshift_deleti  on | exon17 | c.2963delG | p.G988fs | . | . |
| CTC5 | 1 | 156814923 | CG | C | 0.529 | NM_014215 | INSRR | frameshift_deleti  on | exon12 | c.2381delC | p.A794fs | . | . |
| CTC5 | 1 | 157771274 | CA | C | 0.254 | NM_001159398 | FCRL1 | frameshift_deleti  on | exon6 | c.979delT | p.C327fs | . | . |
| CTC5 | 1 | 158687276 | GA | G | 0.583 | NM_001005327 | OR6K3 | frameshift_deleti  on | exon1 | c.629delT | p.F210fs | . | . |
| CTC5 | 1 | 158687379 | TG | T | 0.364 | NM_001005327 | OR6K3 | frameshift_deleti  on | exon1 | c.526delC | p.Q176fs | . | . |
| CTC5 | 1 | 158812100 | G | GA | 0.611 | NM_002432 | MNDA | frameshift_inserti  on | exon2 | c.158dupA | p.E53fs | . | . |
| CTC5 | 1 | 158815740 | C | CT | 0.25 | NM_002432 | MNDA | frameshift_inserti  on | exon5 | c.935dupT | p.L312fs | . | . |
| CTC5 | 1 | 160209553 | CA | C | 0.923 | NM_015726 | DCAF8 | frameshift_deleti  on | exon4 | c.656delT | p.V219fs | . | . |
| CTC5 | 1 | 160389164 | GT | G | 0.609 | NM_020335 | VANGL2 | frameshift_deleti  on | exon4 | c.566delT | p.V189fs | . | . |
| CTC5 | 1 | 160604610 | TGCA | T | 0.365 | NM_003037 | SLAMF1 | nonframeshift_de  letion | exon3 | c.490_492del | p.164_164del | . | . |
| CTC5 | 1 | 160851009 | GC | G | 0.5 | NM_017625 | ITLN1 | frameshift_deleti  on | exon5 | c.498delG | p.L166fs | . | . |
| CTC5 | 1 | 161089968 | G | GA | 0.341 | NM_001185093 | NIT1 | frameshift_inserti  on | exon5 | c.619dupA | p.E206fs | . | . |
| CTC5 | 1 | 161197679 | GC | G | 0.408 | NM_001286374 | TOMM40L | frameshift_deleti  on | exon4 | c.190delC | p.Q64fs | . | . |
| CTC5 | 1 | 162326887 | CCAG | C | 0.127 | NM_001164757 | NOS1AP | nonframeshift_de  letion | exon8 | c.886_888del | p.296_296del | . | . |
| CTC5 | 1 | 162724964 | AAC | A | 0.126 | NM_006182 | DDR2 | frameshift_deleti  on | exon6 | c.437_438del | p.N146fs | . | . |
| CTC5 | 1 | 162735826 | AC | A | 0.875 | NM_006182 | DDR2 | frameshift_deleti  on | exon10 | c.1136delC | p.T379fs | . | . |
| CTC5 | 1 | 162740277 | AT | A | 0.481 | NM_006182 | DDR2 | frameshift_deleti  on | exon12 | c.1480delT | p.F494fs | . | . |
| CTC5 | 1 | 165218675 | TGA | T | 0.6 | NM_001174069 | LMX1A | frameshift_deleti  on | exon4 | c.464_465del | p.L155fs | . | . |
| CTC5 | 1 | 165513749 | TC | T | 1 | NM_001005214 | LRRC52 | stopgain | exon1 | c.217delC | p.L73X | . | . |

| CTC5 | 1 | 165664553 | AT | A | 0.511 | NM_000696 | ALDH9A1 | frameshift_deleti  on | exon2 | c.262delA | p.I88fs | . | . |
| --- | --- | --- | --- | --- | --- | --- | --- | --- | --- | --- | --- | --- | --- |
| CTC5 | 1 | 167780124 | AT | A | 0.931 | NM_001167749 | ADCY10 | frameshift_deleti  on | exon29 | c.4049delA | p.N1350fs | . | . |
| CTC5 | 1 | 167874249 | TC | T | 0.892 | NM_018417 | ADCY10 | frameshift_deleti  on | exon2 | c.129delG | p.L43fs | . | . |
| CTC5 | 1 | 168274285 | CA | C | 0.23 | NM_005149 | TBX19 | frameshift_deleti  on | exon6 | c.768delA | p.T256fs | . | . |
| CTC5 | 1 | 169772385 | GA | G | 0.355 | NM_018186 | C1orf112 | frameshift_deleti  on | exon5 | c.248delA | p.E83fs | . | . |
| CTC5 | 1 | 169947321 | TG | T | 0.227 | NM_001204516 | KIFAP3 | frameshift_deleti  on | exon15 | c.1669delC | p.Q557fs | . | . |
| CTC5 | 1 | 170508434 | CA | C | 1 | NM_001146039 | GORAB | frameshift_deleti  on | exon2 | c.221delA | p.Q74fs | . | . |
| CTC5 | 1 | 171293392 | GC | G | 0.333 | NM_002022 | FMO4 | stopgain | exon5 | c.438delC | p.C146X | . | . |
| CTC5 | 1 | 171502067 | GA | G | 0.784 | NM_015172 | PRRC2C | frameshift_deleti on | exon12 | c.1835delA | p.E612fs | . | . |
| CTC5 | 1 | 171509511 | CA | C | 1 | NM_015172 | PRRC2C | frameshift_deleti  on | exon16 | c.2901delA | p.P967fs | . | . |
| CTC5 | 1 | 172555041 | TAA | T | 0.636 | NM_001282750 | SUCO | frameshift_deleti  on | exon16 | c.1501_1502del | p.K501fs | . | . |
| CTC5 | 1 | 172557984 | TC | T | 0.4 | NM_001282751 | SUCO | frameshift_deleti  on | exon16 | c.55delC | p.Q19fs | . | . |
| CTC5 | 1 | 173472410 | CT | C | 0.933 | NM_178527 | SLC9C2 | frameshift_deleti  on | exon27 | c.3365delA | p.K1122fs | . | . |
| CTC5 | 1 | 173825812 | GA | G | 1 | NM_018122 | DARS2 | frameshift_deleti  on | exon16 | c.1684delA | p.K562fs | . | . |
| CTC5 | 1 | 173883957 | G | GA | 0.857 | NM_000488 | SERPINC1 | frameshift_inserti  on | exon2 | c.141dupT | p.P48fs | . | . |
| CTC5 | 1 | 174959069 | CA | C | 0.292 | NM_001243764 | RABGAP1L | frameshift_deleti  on | exon7 | c.694delA | p.K232fs | . | . |
| CTC5 | 1 | 176526179 | CA | C | 0.253 | NM_020318 | PAPPA2 | frameshift_deleti  on | exon2 | c.722delA | p.Q241fs | . | . |
| CTC5 | 1 | 179078125 | CT | C | 0.486 | NM_001168236 | ABL2 | frameshift_deleti  on | exon11 | c.2213delA | p.K738fs | . | . |
| CTC5 | 1 | 179520521 | TGA | T | 0.429 | NM_014625 | NPHS2 | frameshift_deleti  on | exon8 | c.937_938del | p.S313fs | . | . |
| CTC5 | 1 | 179587810 | TA | T | 0.524 | NM_001199085 | TDRD5 | frameshift_deleti  on | exon5 | c.909delA | p.I303fs | . | . |
| CTC5 | 1 | 179983131 | GA | G | 0.556 | NM_014810 | CEP350 | frameshift_deleti  on | exon10 | c.1544delA | p.E515fs | . | . |
| CTC5 | 1 | 180147990 | GA | G | 0.792 | NM_001004128 | QSOX1 | frameshift_deleti  on | exon5 | c.578delA | p.E193fs | . | . |
| CTC5 | 1 | 180257541 | GT | G | 0.585 | NM_032360 | ACBD6 | frameshift_deleti  on | exon8 | c.805delA | p.T269fs | . | . |
| CTC5 | 1 | 180905112 | GA | G | 0.321 | NM_020950 | KIAA1614 | frameshift_deleti  on | exon5 | c.2068delA | p.K690fs | . | . |
| CTC5 | 1 | 184863330 | GC | G | 0.922 | NM_052966 | FAM129A | frameshift_deleti  on | exon3 | c.196delG | p.A66fs | . | . |
| CTC5 | 1 | 186158942 | G | GC | 0.7 | NM_031935 | HMCN1 | frameshift_inserti  on | exon107 | c.16841dupC | p.A5614fs | . | . |
| CTC5 | 1 | 186375324 | CA | C | 0.381 | NM_001164245 | C1orf27 | frameshift_deleti  on | exon11 | c.1042delA | p.R348fs | . | . |
| CTC5 | 1 | 196227361 | AT | A | 0.308 | NM_001287819 | KCNT2 | frameshift_deleti  on | exon25 | c.3101delA | p.N1034fs | . | . |
| CTC5 | 1 | 197030151 | GT | G | 0.375 | NM_001994 | F13B | frameshift_deleti  on | exon4 | c.505delA | p.T169fs | . | . |
| CTC5 | 1 | 197111783 | AT | A | 0.309 | NM_001206846 | ASPM | frameshift_deleti  on | exon3 | c.1598delA | p.N533fs | . | . |
| CTC5 | 1 | 197522118 | GA | G | 0.311 | NM_144977 | DENND1B | frameshift_deleti  on | exon16 | c.1273delT | p.S425fs | . | . |
| CTC5 | 1 | 197704889 | TC | T | 0.181 | NM_001195216 | DENND1B | frameshift_deleti  on | exon3 | c.119delG | p.G40fs | . | . |
| CTC5 | 1 | 201038686 | TAA | T | 0.21 | NM_000069 | CACNA1S | frameshift_deleti  on | exon18 | c.2402_2403del | p.F801fs | . | . |
| CTC5 | 1 | 201043679 | GC | G | 0.367 | NM_000069 | CACNA1S | frameshift_deleti  on | exon14 | c.2017delG | p.A673fs | . | . |
| CTC5 | 1 | 201181559 | A | AT | 0.476 | NM_001164586 | IGFN1 | frameshift_inserti  on | exon12 | c.7539dupT | p.D2513fs | . | . |
| CTC5 | 1 | 201868523 | GA | G | 1 | NM_012134 | LMOD1 | frameshift_deleti  on | exon2 | c.1617delT | p.P539fs | . | . |
| CTC5 | 1 | 202702883 | TG | T | 0.733 | NM_006618 | KDM5B | frameshift_deleti  on | exon23 | c.3554delC | p.P1185fs | . | . |
| CTC5 | 1 | 203186947 | G | GCCCA  GACCA TGGCC CCGAC | 0.955 | NM_001256125 | CHIT1 | nonframeshift_in sertion | exon9 | c.1018_1019ins  GACTGGTCGG GGCCATGGTC TGGG | p.A340delinsGL VGAMVWA | . | . |
| CTC5 | 1 | 203186950 | C | CAGAC  CATGG CCCCG ACCAG | 0.959 | NM_001256125 | CHIT1 | stopgain | exon9 | c.1015_1016ins  AGGGACTGGT CGGGGCCATG GTCT | p.W339delinsX | . | . |
| CTC5 | 1 | 203186960 | C | CCCCG  ACCAG TCCCTA | 0.792 | NM_001256125 | CHIT1 | frameshift_inserti on | exon9 | c.1005_1006ins  CCATGGTCTA GGGACTGGTC | p.A336fs | . | . |
| CTC5 | 1 | 204095109 | TG | T | 0.223 | NM_005686 | SOX13 | stopgain | exon14 | c.1717delG | p.V573X | . | . |
| CTC5 | 1 | 204438277 | AC | A | 1 | NM_002646 | PIK3C2B | frameshift_deleti on | exon3 | c.653delG | p.G218fs | . | . |
| CTC5 | 1 | 205064086 | CG | C | 0.324 | NM_001193273 | RBBP5 | frameshift_deleti  on | exon13 | c.1121delC | p.P374fs | . | . |
| CTC5 | 1 | 205238172 | AC | A | 0.553 | NM_001242925 | TMCC2 | frameshift_deleti  on | exon3 | c.609delC | p.D203fs | . | . |
| CTC5 | 1 | 205779451 | AG | A | 0.48 | NM_173854 | SLC41A1 | frameshift_deleti  on | exon2 | c.118delC | p.L40fs | . | . |
| CTC5 | 1 | 206769174 | AT | A | 0.577 | NM_001201478 | EIF2D | frameshift_deleti  on | exon11 | c.1029delA | p.K343fs | . | . |
| CTC5 | 1 | 206772957 | AG | A | 0.5 | NM_001201478 | EIF2D | frameshift_deleti  on | exon8 | c.689delC | p.S230fs | . | . |
| CTC5 | 1 | 207131882 | TG | T | 1 | NM_001170631 | FCAMR | frameshift_deleti  on | exon8 | c.1712delC | p.P571fs | . | . |
| CTC5 | 1 | 207222794 | A | AT | 0.533 | NM_018566 | YOD1 | frameshift_inserti  on | exon2 | c.617dupA | p.N206fs | . | . |
| CTC5 | 1 | 207304920 | C | CA | 0.684 | NM_000715 | C4BPA | frameshift_inserti  on | exon8 | c.920dupA | p.H307fs | . | . |
| CTC5 | 1 | 209605636 | TAGCAG  CAGCAG | T | 0.984 | NM_001104548 | MIR205HG | nonframeshift_de  letion | exon4 | c.252_263del | p.84_88del | . | . |
| CTC5 | 1 | 209605636 | TAGCAG  CAGCAG | T | 0.984 | NM_001104548 | MIR205HG | nonframeshift_de  letion | exon4 | c.252_266del | p.84_89del | . | . |
| CTC5 | 1 | 209849207 | G | GA | 0.787 | NM_015714 | G0S2 | frameshift_inserti  on | exon2 | c.179dupA | p.E60fs | . | . |
| CTC5 | 1 | 210329176 | TA | T | 0.227 | NM_001146262 | SYT14 | frameshift_deleti  on | exon7 | c.1276delA | p.N426fs | . | . |
| CTC5 | 1 | 210761427 | GC | G | 0.887 | NM_001170564 | HHAT | stopgain | exon8 | c.819delC | p.C273X | . | . |

| CTC5 | 1 | 213178491 | C | CT | 0.455 | NM_144567 | ANGEL2 | frameshift_inserti  on | exon5 | c.1017dupA | p.D340fs | . | . |
| --- | --- | --- | --- | --- | --- | --- | --- | --- | --- | --- | --- | --- | --- |
| CTC5 | 1 | 213415381 | TA | T | 0.571 | NM_001287220 | RPS6KC1 | frameshift_deleti  on | exon9 | c.1168delA | p.K390fs | . | . |
| CTC5 | 1 | 213434949 | T | TG | 0.556 | NM_001287220 | RPS6KC1 | frameshift_inserti  on | exon11 | c.1566dupG | p.L522fs | . | . |
| CTC5 | 1 | 214832318 | GA | G | 0.308 | NM_016343 | CENPF | frameshift_deleti  on | exon19 | c.9089delA | p.E3030fs | . | . |
| CTC5 | 1 | 216595249 | GC | G | 0.278 | NM_007123 | USH2A | frameshift_deleti  on | exon2 | c.429delG | p.K143fs | . | . |
| CTC5 | 1 | 216595422 | TG | T | 0.6 | NM_007123 | USH2A | frameshift_deleti  on | exon2 | c.256delC | p.Q86fs | . | . |
| CTC5 | 1 | 220162128 | AG | A | 0.6 | NM_004446 | EPRS | stopgain | exon19 | c.2578delC | p.L860X | . | . |
| CTC5 | 1 | 220279277 | G | GA | 0.909 | NM_018060 | IARS2 | frameshift_inserti on | exon9 | c.1112dupA | p.D371fs | . | . |
| CTC5 | 1 | 220986707 | GA | G | 0.376 | NM_022746 | 1-Mar | frameshift_deleti  on | exon7 | c.962delA | p.E321fs | . | . |
| CTC5 | 1 | 222802169 | CA | C | 0.758 | NM_198551 | MIA3 | frameshift_deleti  on | exon4 | c.1608delA | p.S536fs | . | . |
| CTC5 | 1 | 222802915 | AT | A | 0.455 | NM_198551 | MIA3 | frameshift_deleti  on | exon4 | c.2354delT | p.I785fs | . | . |
| CTC5 | 1 | 222825630 | C | CA | 0.333 | NM_198551 | MIA3 | frameshift_inserti  on | exon13 | c.4042_4043ins  A | p.R1348fs | . | . |
| CTC5 | 1 | 223286245 | CT | C | 0.545 | NM_003268 | TLR5 | frameshift_deleti  on | exon6 | c.128delA | p.Q43fs | . | . |
| CTC5 | 1 | 223983829 | CT | C | 1 | NM_001031685 | TP53BP2 | frameshift_deleti  on | exon13 | c.2411delA | p.K804fs | . | . |
| CTC5 | 1 | 224586257 | GA | G | 0.714 | NM_001115113 | WDR26 | frameshift_deleti  on | exon11 | c.1555delT | p.S519fs | . | . |
| CTC5 | 1 | 225394771 | TA | T | 0.636 | NM_001373 | DNAH14 | frameshift_deleti  on | exon40 | c.6223delA | p.K2075fs | . | . |
| CTC5 | 1 | 225394900 | T | TA | 0.491 | NM_001373 | DNAH14 | frameshift_inserti  on | exon40 | c.6352dupA | p.G2117fs | . | . |
| CTC5 | 1 | 227921184 | CA | C | 1 | NM_001161465 | JMJD4 | frameshift_deleti  on | exon4 | c.890delT | p.L297fs | . | . |
| CTC5 | 1 | 227946724 | TG | T | 0.251 | NM_053052 | SNAP47 | frameshift_deleti  on | exon3 | c.662delG | p.W221fs | . | . |
| CTC5 | 1 | 228033808 | AT | A | 0.7 | NM_183062 | PRSS38 | frameshift_deleti  on | exon5 | c.881delT | p.I294fs | . | . |
| CTC5 | 1 | 228109459 | AG | A | 0.235 | NM_003395 | WNT9A | frameshift_deleti  on | exon4 | c.857delC | p.T286fs | . | . |
| CTC5 | 1 | 228285557 | TC | T | 0.476 | NM_001024226 | ARF1 | frameshift_deleti  on | exon5 | c.390delC | p.L130fs | . | . |
| CTC5 | 1 | 228495170 | AC | A | 0.624 | NM_001098623 | OBSCN | frameshift_deleti  on | exon46 | c.12405delC | p.D4135fs | . | . |
| CTC5 | 1 | 228564528 | AC | A | 0.387 | NM_001098623 | OBSCN | frameshift_deleti  on | exon100 | c.22993delC | p.P7665fs | . | . |
| CTC5 | 1 | 231503357 | CA | C | 0.5 | NM_022051 | EGLN1 | frameshift_deleti  on | exon4 | c.1173delT | p.F391fs | . | . |
| CTC5 | 1 | 232144690 | CA | C | 0.306 | NM_001164540 | DISC1 | frameshift_deleti  on | exon9 | c.1837delA | p.R613fs | . | . |
| CTC5 | 1 | 235866183 | TG | T | 0.375 | NM_000081 | LYST | frameshift_deleti  on | exon45 | c.10237delC | p.Q3413fs | . | . |
| CTC5 | 1 | 236189420 | CG | C | 0.296 | NM_002508 | NID1 | frameshift_deleti  on | exon8 | c.1759delC | p.R587fs | . | . |
| CTC5 | 1 | 237711847 | CG | C | 0.444 | NM_001035 | RYR2 | frameshift_deleti  on | exon26 | c.3024delG | p.A1008fs | . | . |
| CTC5 | 1 | 237774130 | G | GT | 0.25 | NM_001035 | RYR2 | frameshift_inserti  on | exon36 | c.4752_4753ins  T | p.P1584fs | . | . |
| CTC5 | 1 | 241728314 | ATT | A | 0.194 | NM_003679 | KMO | frameshift_deleti  on | exon8 | c.644_645del | p.I215fs | . | . |
| CTC5 | 1 | 241842850 | G | GA | 0.315 | NM_144625 | WDR64 | frameshift_inserti  on | exon5 | c.548dupA | p.E183fs | . | . |
| CTC5 | 1 | 244587319 | TG | T | 0.289 | NM_001126 | ADSS | frameshift_deleti  on | exon6 | c.516delC | p.S172fs | . | . |
| CTC5 | 1 | 245018777 | GT | G | 0.241 | NM_004501 | HNRNPU | frameshift_deleti  on | exon12 | c.2243delA | p.N748fs | . | . |
| CTC5 | 1 | 247014348 | C | CT | 0.514 | NM_015446 | AHCTF1 | frameshift_inserti  on | exon33 | c.4986dupA | p.V1663fs | . | . |
| CTC5 | 1 | 247202732 | GA | G | 0.696 | NM_001204220 | ZNF670 | frameshift_deleti  on | exon2 | c.110delT | p.F37fs | . | . |
| CTC5 | 1 | 247978541 | AAGG | A | 1 | NM_001001966 | OR14A16 | nonframeshift_de  letion | exon1 | c.488_490del | p.163_164del | . | . |
| CTC5 | 1 | 248525638 | CA | C | 0.353 | NM_001004696 | OR2T4 | frameshift_deleti  on | exon1 | c.757delA | p.I253fs | . | . |
| CTC5 | 1 | 248616704 | CTGCTG  CG | C | 0.667 | NM_001004136 | OR2T2 | frameshift_deleti  on | exon1 | c.607_613del | p.C203fs | . | . |
| CTC5 | 1 | 248756962 | CA | C | 0.538 | NM_001004693 | OR2T10 | stopgain | exon1 | c.107delT | p.L36X | . | . |
| CTC5 | 2 | 1665908 | CCCA | C | 0.833 | NM_012293 | PXDN | nonframeshift_de letion | exon13 | c.1613_1615del | p.538_539del | . | . |
| CTC5 | 2 | 1667395 | GGT | G | 0.927 | NM_012293 | PXDN | frameshift_deleti  on | exon12 | c.1547_1548del | p.H516fs | . | . |
| CTC5 | 2 | 1946770 | TTCC | T | 0.16 | NM_015025 | MYT1L | nonframeshift_de  letion | exon9 | c.486_488del | p.162_163del | . | . |
| CTC5 | 2 | 3749151 | GGAA | G | 1 | NM_018436 | ALLC | nonframeshift_de  letion | exon11 | c.901_903del | p.301_301del | . | . |
| CTC5 | 2 | 10559900 | GC | G | 1 | NM_001258357 | HPCAL1 | frameshift_deleti  on | exon3 | c.18delC | p.S6fs | . | . |
| CTC5 | 2 | 15467886 | GA | G | 0.5 | NM_015909 | NBAS | frameshift_deleti  on | exon38 | c.4569delT | p.F1523fs | . | . |
| CTC5 | 2 | 15557735 | AT | A | 0.6 | NM_015909 | NBAS | frameshift_deleti  on | exon24 | c.2678delA | p.Y893fs | . | . |
| CTC5 | 2 | 15614282 | GC | G | 0.812 | NM_015909 | NBAS | frameshift_deleti  on | exon15 | c.1507delG | p.A503fs | . | . |
| CTC5 | 2 | 17699201 | C | CG | 0.311 | NM_001099218 | RAD51AP2 | frameshift_inserti  on | exon1 | c.481_482insC | p.S161fs | . | . |
| CTC5 | 2 | 21225111 | C | CT | 0.231 | NM_000384 | APOB | frameshift_inserti  on | exon29 | c.13182_13183i  nsA | p.G4395fs | . | . |
| CTC5 | 2 | 21235108 | G | GC | 0.909 | NM_000384 | APOB | frameshift_inserti  on | exon26 | c.4631_4632ins  G | p.L1544fs | . | . |
| CTC5 | 2 | 25050925 | GC | G | 0.224 | NM_004036 | ADCY3 | frameshift_deleti  on | exon13 | c.2277delG | p.V759fs | . | . |
| CTC5 | 2 | 25470992 | TG | T | 0.974 | NM_153759 | DNMT3A | frameshift_deleti  on | exon3 | c.201delC | p.P67fs | . | . |
| CTC5 | 2 | 26068420 | AT | A | 0.455 | NM_018263 | ASXL2 | frameshift_deleti  on | exon2 | c.69delA | p.K23fs | . | . |
| CTC5 | 2 | 26653669 | GA | G | 0.351 | NM_145038 | DRC1 | frameshift_deleti  on | exon6 | c.695delA | p.E232fs | . | . |
| CTC5 | 2 | 26696126 | TC | T | 0.559 | NM_194322 | OTOF | frameshift_deleti  on | exon11 | c.1536delG | p.L512fs | . | . |
| CTC5 | 2 | 27001339 | TG | T | 0.5 | NM_017877 | SLC35F6 | frameshift_deleti  on | exon6 | c.1077delG | p.L359fs | . | . |
| CTC5 | 2 | 27352419 | TAG | T | 0.519 | NM_032604 | ABHD1 | frameshift_deleti  on | exon5 | c.543_544del | p.L181fs | . | . |

| CTC5 | 2 | 27799580 | AT | A | 1 | NM_032266 | C2orf16 | frameshift_deleti  on | exon1 | c.142delT | p.F48fs | . | . |
| --- | --- | --- | --- | --- | --- | --- | --- | --- | --- | --- | --- | --- | --- |
| CTC5 | 2 | 27800204 | AC | A | 1 | NM_032266 | C2orf16 | frameshift_deleti  on | exon1 | c.766delC | p.L256fs | . | . |
| CTC5 | 2 | 27851406 | A | AT | 0.672 | NM_001142683 | CCDC121 | frameshift_inserti  on | exon1 | c.224dupA | p.N75fs | . | . |
| CTC5 | 2 | 29356629 | AT | A | 0.857 | NM_001287527 | CLIP4 | frameshift_deleti  on | exon5 | c.477delT | p.Y159fs | . | . |
| CTC5 | 2 | 36744520 | GC | G | 1 | NM_016441 | CRIM1 | frameshift_deleti  on | exon12 | c.2042delC | p.A681fs | . | . |
| CTC5 | 2 | 37487450 | CT | C | 0.385 | NM_005813 | PRKD3 | frameshift_deleti  on | exon14 | c.1961delA | p.K654fs | . | . |
| CTC5 | 2 | 38298228 | GT | G | 0.75 | NM_000104 | CYP1B1 | frameshift_deleti  on | exon3 | c.1268delA | p.N423fs | . | . |
| CTC5 | 2 | 38545778 | C | CA | 0.54 | NM_001135673 | ATL2 | frameshift_inserti  on | exon4 | c.501dupT | p.A168fs | . | . |
| CTC5 | 2 | 39414801 | G | GAA | 0.909 | NM_001009565 | CDKL4 | frameshift_inserti  on | exon6 | c.701_702insTT | p.F234fs | . | . |
| CTC5 | 2 | 43927371 | GA | G | 0.467 | NM_172069 | PLEKHH2 | frameshift_deleti  on | exon8 | c.1275delA | p.G425fs | . | . |
| CTC5 | 2 | 44032347 | GA | G | 0.407 | NM_001193464 | DYNC2LI1 | frameshift_deleti  on | exon12 | c.959delA | p.E320fs | . | . |
| CTC5 | 2 | 46985751 | GA | G | 0.556 | NM_014011 | SOCS5 | frameshift_deleti  on | exon2 | c.83delA | p.E28fs | . | . |
| CTC5 | 2 | 48018251 | AG | A | 0.375 | NM_000179 | MSH6 | frameshift_deleti  on | exon2 | c.447delG | p.K149fs | . | . |
| CTC5 | 2 | 54161761 | A | AT | 0.176 | NM_014614 | PSME4 | frameshift_inserti  on | exon8 | c.937dupA | p.I313fs | . | . |
| CTC5 | 2 | 55185072 | AG | A | 0.8 | NM_001039753 | EML6 | frameshift_deleti  on | exon32 | c.4633delG | p.G1545fs | . | . |
| CTC5 | 2 | 55489473 | TG | T | 1 | NM_002453 | MTIF2 | frameshift_deleti  on | exon5 | c.309delC | p.A103fs | . | . |
| CTC5 | 2 | 55771475 | GC | G | 0.485 | NM_080667 | CCDC104 | frameshift_deleti  on | exon9 | c.898delC | p.Q300fs | . | . |
| CTC5 | 2 | 58316767 | TG | T | 0.312 | NM_001288839 | VRK2 | frameshift_deleti  on | exon6 | c.99delG | p.L33fs | . | . |
| CTC5 | 2 | 61018972 | AC | A | 0.769 | NM_022894 | PAPOLG | frameshift_deleti  on | exon16 | c.1474delC | p.L492fs | . | . |
| CTC5 | 2 | 61473489 | A | AT | 0.194 | NM_014709 | USP34 | frameshift_inserti  on | exon50 | c.6517_6518ins  A | p.V2173fs | . | . |
| CTC5 | 2 | 61597657 | TA | T | 0.25 | NM_014709 | USP34 | frameshift_deleti  on | exon9 | c.1133delT | p.I378fs | . | . |
| CTC5 | 2 | 62729632 | T | TA | 0.528 | NM_198276 | TMEM17 | frameshift_inserti  on | exon3 | c.257dupT | p.L86fs | . | . |
| CTC5 | 2 | 65296910 | AC | A | 0.5 | NM_015147 | CEP68 | frameshift_deleti  on | exon2 | c.333delC | p.D111fs | . | . |
| CTC5 | 2 | 65543891 | T | TG | 0.727 | NM_001128210 | SPRED2 | frameshift_inserti  on | exon5 | c.555dupC | p.T186fs | . | . |
| CTC5 | 2 | 66665034 | GC | G | 0.939 | NM_002398 | MEIS1 | frameshift_deleti  on | exon2 | c.179delC | p.A60fs | . | . |
| CTC5 | 2 | 68384450 | GC | G | 0.515 | NM_001256476 | WDR92 | frameshift_deleti  on | exon1 | c.125delG | p.G42fs | . | . |
| CTC5 | 2 | 69741753 | CTGT | C | 1 | NM_014911 | AAK1 | nonframeshift_de  letion | exon13 | c.1623_1625del | p.541_542del | . | . |
| CTC5 | 2 | 69769770 | TG | T | 1 | NM_014911 | AAK1 | frameshift_deleti  on | exon5 | c.418delC | p.Q140fs | . | . |
| CTC5 | 2 | 71058200 | TA | T | 0.156 | NM_015717 | CD207 | frameshift_deleti  on | exon6 | c.967delT | p.Y323fs | . | . |
| CTC5 | 2 | 71148404 | TC | T | 1 | NM_012476 | VAX2 | frameshift_deleti  on | exon2 | c.425delC | p.S142fs | . | . |
| CTC5 | 2 | 72362514 | TC | T | 1 | NM_001277742 | CYP26B1 | frameshift_deleti  on | exon2 | c.238delG | p.E80fs | . | . |
| CTC5 | 2 | 73303274 | C | CT | 0.245 | NM_015470 | RAB11FIP5 | frameshift_inserti  on | exon4 | c.1604_1605ins  A | p.V535fs | . | . |
| CTC5 | 2 | 73493597 | GC | G | 1 | NM_001080410 | FBXO41 | frameshift_deleti  on | exon2 | c.1118delG | p.G373fs | . | . |
| CTC5 | 2 | 73675227 | T | TCTC | 1 | NM_015120 | ALMS1 | nonframeshift_in  sertion | exon8 | c.1570_1571ins  CTC | p.S524delinsSP | . | . |
| CTC5 | 2 | 73957839 | C | CA | 0.643 | NM_016058 | TPRKB | frameshift_inserti  on | exon4 | c.288dupT | p.G97fs | . | . |
| CTC5 | 2 | 74043669 | CA | C | 0.8 | NM_001080474 | C2orf78 | frameshift_deleti  on | exon3 | c.2320delA | p.N774fs | . | . |
| CTC5 | 2 | 74274315 | TCCCTCC  AGCAAC TCCTAG AACTGA GTTCCCT GAAGCC | T | 0.7 | NM_001287491 | TET3 | nonframeshift_de letion | exon3 | c.1272_1322del | p.424_441del | . | . |
| CTC5 | 2 | 74474205 | AG | A | 0.909 | NM_021196 | SLC4A5 | frameshift_deleti  on | exon14 | c.2016delC | p.N672fs | . | . |
| CTC5 | 2 | 74641780 | CT | C | 0.909 | NM_001145054 | C2orf81 | frameshift_deleti  on | exon4 | c.1442delA | p.E481fs | . | . |
| CTC5 | 2 | 74720090 | TG | T | 0.275 | NM_022492 | TTC31 | frameshift_deleti  on | exon13 | c.1306delG | p.G436fs | . | . |
| CTC5 | 2 | 75101456 | AG | A | 0.25 | NM_000189 | HK2 | frameshift_deleti  on | exon7 | c.756delG | p.E252fs | . | . |
| CTC5 | 2 | 85012877 | TC | T | 1 | NM_001370 | DNAH6 | frameshift_deleti  on | exon67 | c.11098delC | p.Q3700fs | . | . |
| CTC5 | 2 | 86255723 | C | CT | 0.571 | NM_015425 | POLR1A | frameshift_inserti  on | exon32 | c.4890_4891ins  A | p.V1631fs | . | . |
| CTC5 | 2 | 88828715 | CA | C | 0.208 | NM_152670 | TEX37 | frameshift_deleti  on | exon4 | c.267delA | p.S89fs | . | . |
| CTC5 | 2 | 95847040 | TGCG | T | 1 | NM_001017396 | ZNF2 | nonframeshift_de  letion | exon4 | c.342_344del | p.114_115del | . | . |
| CTC5 | 2 | 95847341 | AC | A | 0.235 | NM_001017396 | ZNF2 | frameshift_deleti  on | exon4 | c.643delC | p.P215fs | . | . |
| CTC5 | 2 | 96860740 | CA | C | 0.317 | NM_020151 | STARD7 | frameshift_deleti  on | exon3 | c.504delT | p.F168fs | . | . |
| CTC5 | 2 | 97329191 | CA | C | 0.343 | NM_001113382 | FER1L5 | frameshift_deleti  on | exon13 | c.1037delA | p.Q346fs | . | . |
| CTC5 | 2 | 97427261 | GC | G | 1 | NM_020184 | CNNM4 | frameshift_deleti  on | exon1 | c.526delC | p.P176fs | . | . |
| CTC5 | 2 | 99448843 | GC | G | 0.333 | NM_207362 | KIAA1211L | frameshift_deleti  on | exon5 | c.507delG | p.L169fs | . | . |
| CTC5 | 2 | 100218010 | CGCT | C | 0.2 | NM_001025108 | AFF3 | nonframeshift_de  letion | exon13 | c.1330_1332del | p.444_444del | . | . |
| CTC5 | 2 | 101624364 | AT | A | 0.833 | NM_001102426 | TBC1D8 | frameshift_deleti  on | exon20 | c.3341delA | p.N1114fs | . | . |
| CTC5 | 2 | 101656720 | GC | G | 0.333 | NM_001102426 | TBC1D8 | frameshift_deleti  on | exon6 | c.954delG | p.G318fs | . | . |
| CTC5 | 2 | 102475508 | TCAGCA  GCAGCT | T | 0.625 | NM_001242559 | MAP4K4 | nonframeshift_de  letion | exon14 | c.1447_1461del | p.483_487del | . | . |
| CTC5 | 2 | 103378747 | CT | C | 0.7 | NM_144632 | TMEM182 | frameshift_deleti  on | exon1 | c.72delT | p.A24fs | . | . |

| CTC5 | 2 | 110303805 | TG | T | 0.571 | NM_178584 | 10-Sep | frameshift_deleti  on | exon9 | c.1101delC | p.A367fs | . | . |
| --- | --- | --- | --- | --- | --- | --- | --- | --- | --- | --- | --- | --- | --- |
| CTC5 | 2 | 112582738 | TG | T | 0.615 | NM_022662 | ANAPC1 | frameshift_deleti  on | exon23 | c.2710delC | p.Q904fs | . | . |
| CTC5 | 2 | 113089245 | CA | C | 0.545 | NM_198581 | ZC3H6 | frameshift_deleti  on | exon12 | c.2751delA | p.T917fs | . | . |
| CTC5 | 2 | 113300001 | TTCCGG  CGTGTA CCGAGA | T | 1 | NM_001282772 | POLR1B | frameshift_deleti on | exon2 | c.45_69del | p.L15fs | . | . |
| CTC5 | 2 | 113416606 | CAG | C | 0.429 | NM_005415 | SLC20A1 | frameshift_deleti  on | exon7 | c.984_985del | p.P328fs | . | . |
| CTC5 | 2 | 118577352 | GC | G | 0.233 | NM_006773 | DDX18 | frameshift_deleti  on | exon3 | c.499delC | p.P167fs | . | . |
| CTC5 | 2 | 121740357 | CG | C | 0.337 | NM_005270 | GLI2 | frameshift_deleti  on | exon10 | c.1585delG | p.G529fs | . | . |
| CTC5 | 2 | 128390917 | CT | C | 0.238 | NM_001080527 | MYO7B | frameshift_deleti  on | exon39 | c.5413delT | p.F1805fs | . | . |
| CTC5 | 2 | 131520338 | C | CA | 0.667 | NM_001105193 | AMER3 | frameshift_inserti  on | exon2 | c.694dupA | p.C231fs | . | . |
| CTC5 | 2 | 133403641 | CT | C | 0.182 | NM_001077427 | LYPD1 | frameshift_deleti  on | exon3 | c.246delA | p.L82fs | . | . |
| CTC5 | 2 | 133542126 | CT | C | 1 | NM_207363 | NCKAP5 | frameshift_deleti  on | exon14 | c.2257delA | p.R753fs | . | . |
| CTC5 | 2 | 135711171 | GC | G | 0.677 | NM_001241 | CCNT2 | frameshift_deleti  on | exon9 | c.1147delC | p.Q383fs | . | . |
| CTC5 | 2 | 141751565 | GC | G | 1 | NM_018557 | LRP1B | frameshift_deleti  on | exon16 | c.2642delG | p.C881fs | . | . |
| CTC5 | 2 | 141819838 | GT | G | 0.357 | NM_018557 | LRP1B | frameshift_deleti  on | exon8 | c.1017delA | p.K339fs | . | . |
| CTC5 | 2 | 145147156 | AT | A | 0.75 | NM_001171653 | ZEB2 | frameshift_deleti  on | exon9 | c.3434delA | p.N1145fs | . | . |
| CTC5 | 2 | 149868100 | CG | C | 0.81 | NM_004522 | KIF5C | frameshift_deleti  on | exon25 | c.2785delG | p.G929fs | . | . |
| CTC5 | 2 | 152128230 | CA | C | 0.346 | NM_004688 | NMI | stopgain | exon7 | c.650delT | p.L217X | . | . |
| CTC5 | 2 | 155555845 | GCC | G | 0.921 | NM_001260508 | KCNJ3 | frameshift_deleti on | exon1 | c.559_560del | p.P187fs | . | . |
| CTC5 | 2 | 157439418 | TG | T | 0.444 | NM_000408 | GPD2 | frameshift_deleti  on | exon17 | c.2173delG | p.G725fs | . | . |
| CTC5 | 2 | 159499196 | AG | A | 0.389 | NM_001005476 | PKP4 | frameshift_deleti  on | exon11 | c.1895delG | p.R632fs | . | . |
| CTC5 | 2 | 159535149 | CA | C | 1 | NM_001005476 | PKP4 | frameshift_deleti  on | exon20 | c.3185delA | p.Q1062fs | . | . |
| CTC5 | 2 | 160086219 | CA | C | 0.471 | NM_033394 | TANC1 | frameshift_deleti  on | exon27 | c.4283delA | p.Q1428fs | . | . |
| CTC5 | 2 | 160994194 | TC | T | 0.308 | NM_001282354 | ITGB6 | frameshift_deleti  on | exon8 | c.1125delG | p.G375fs | . | . |
| CTC5 | 2 | 163393443 | GT | G | 0.474 | NM_033272 | KCNH7 | frameshift_deleti  on | exon3 | c.454delA | p.T152fs | . | . |
| CTC5 | 2 | 164467115 | AT | A | 0.773 | NM_018086 | FIGN | frameshift_deleti  on | exon3 | c.1226delA | p.N409fs | . | . |
| CTC5 | 2 | 165947415 | AG | A | 0.311 | NM_001081676 | SCN3A | frameshift_deleti  on | exon28 | c.5100delC | p.F1700fs | . | . |
| CTC5 | 2 | 165948937 | AC | A | 0.462 | NM_001081676 | SCN3A | frameshift_deleti  on | exon27 | c.4486delG | p.V1496fs | . | . |
| CTC5 | 2 | 166032749 | A | AT | 0.243 | NM_001081676 | SCN3A | frameshift_inserti  on | exon3 | c.155dupA | p.N52fs | . | . |
| CTC5 | 2 | 166243289 | AC | A | 0.75 | NM_001040143 | SCN2A | frameshift_deleti  on | exon25 | c.4586delC | p.T1529fs | . | . |
| CTC5 | 2 | 166908231 | G | GA | 0.4 | NM_001165963 | SCN1A | frameshift_inserti  on | exon6 | c.961dupT | p.S321fs | . | . |
| CTC5 | 2 | 167128960 | A | AT | 0.417 | NM_002977 | SCN9A | frameshift_inserti  on | exon17 | c.3266dupA | p.N1089fs | . | . |
| CTC5 | 2 | 168101608 | GT | G | 0.274 | NM_001199144 | XIRP2 | frameshift_deleti  on | exon7 | c.3041delT | p.V1014fs | . | . |
| CTC5 | 2 | 168103225 | GA | G | 0.787 | NM_001199144 | XIRP2 | frameshift_deleti  on | exon7 | c.4658delA | p.E1553fs | . | . |
| CTC5 | 2 | 169551513 | GT | G | 0.44 | NM_001256126 | CERS6 | frameshift_deleti  on | exon6 | c.562delT | p.F188fs | . | . |
| CTC5 | 2 | 170131698 | CT | C | 0.667 | NM_004525 | LRP2 | frameshift_deleti  on | exon14 | c.1822delA | p.S608fs | . | . |
| CTC5 | 2 | 170366731 | CG | C | 0.944 | NM_006063 | KLHL41 | frameshift_deleti  on | exon1 | c.444delG | p.P148fs | . | . |
| CTC5 | 2 | 171704238 | CT | C | 1 | NM_000817 | GAD1 | frameshift_deleti  on | exon11 | c.1056delT | p.A352fs | . | . |
| CTC5 | 2 | 173352730 | CA | C | 0.5 | NM_000210 | ITGA6 | frameshift_deleti  on | exon18 | c.2367delA | p.A789fs | . | . |
| CTC5 | 2 | 173955831 | AG | A | 0.429 | NM_016653 | ZAK | frameshift_deleti  on | exon2 | c.73delG | p.G25fs | . | . |
| CTC5 | 2 | 178482132 | ATT | A | 0.571 | NM_152275 | TTC30A | frameshift_deleti  on | exon1 | c.1296_1297del | p.K432fs | . | . |
| CTC5 | 2 | 178482475 | GA | G | 0.3 | NM_152275 | TTC30A | frameshift_deleti  on | exon1 | c.954delT | p.F318fs | . | . |
| CTC5 | 2 | 178977461 | AG | A | 0.292 | NM_152945 | RBM45 | frameshift_deleti  on | exon1 | c.189delG | p.K63fs | . | . |
| CTC5 | 2 | 179414756 | AT | A | 0.922 | NM_003319 | TTN | frameshift_deleti  on | exon165 | c.64613delA | p.N21538fs | . | . |
| CTC5 | 2 | 179434656 | ATC | A | 0.458 | NM_003319 | TTN | frameshift_deleti  on | exon154 | c.49006_49007  del | p.D16336fs | . | . |
| CTC5 | 2 | 179532213 | AT | A | 0.773 | NM_001267550 | TTN | frameshift_deleti  on | exon161 | c.35668delA | p.I11890fs | . | . |
| CTC5 | 2 | 179536710 | CT | C | 0.4 | NM_133378 | TTN | frameshift_deleti  on | exon150 | c.31311delA | p.K10437fs | . | . |
| CTC5 | 2 | 179588414 | GA | G | 0.857 | NM_133378 | TTN | frameshift_deleti  on | exon71 | c.17680delT | p.S5894fs | . | . |
| CTC5 | 2 | 180311348 | CA | C | 0.4 | NM_001113397 | ZNF385B | frameshift_deleti  on | exon5 | c.591delT | p.V197fs | . | . |
| CTC5 | 2 | 180311411 | GT | G | 0.615 | NM_001113397 | ZNF385B | frameshift_deleti  on | exon5 | c.528delA | p.T176fs | . | . |
| CTC5 | 2 | 182781060 | AC | A | 0.362 | NM_001287504 | SSFA2 | frameshift_deleti  on | exon10 | c.2235delC | p.Y745fs | . | . |
| CTC5 | 2 | 183619804 | TG | T | 0.452 | NM_001271581 | DNAJC10 | frameshift_deleti  on | exon16 | c.1489delG | p.A497fs | . | . |
| CTC5 | 2 | 186660243 | AT | A | 0.417 | NM_173651 | FSIP2 | frameshift_deleti  on | exon16 | c.8648delT | p.M2883fs | . | . |
| CTC5 | 2 | 187618665 | TG | T | 1 | NM_177454 | FAM171B | frameshift_deleti  on | exon6 | c.902delG | p.W301fs | . | . |
| CTC5 | 2 | 188348851 | CA | C | 0.684 | NM_001032281 | TFPI | frameshift_deleti  on | exon6 | c.627delT | p.F209fs | . | . |
| CTC5 | 2 | 189926290 | GA | G | 0.45 | NM_000393 | COL5A2 | frameshift_deleti  on | exon30 | c.2028delT | p.F676fs | . | . |
| CTC5 | 2 | 190322058 | CG | C | 0.812 | NM_032168 | WDR75 | frameshift_deleti  on | exon6 | c.521delG | p.R174fs | . | . |
| CTC5 | 2 | 191390028 | AT | A | 0.492 | NM_001142645 | TMEM194B | frameshift_deleti  on | exon2 | c.173delA | p.N58fs | . | . |

| CTC5 | 2 | 196729491 | TA | T | 1 | NM_018897 | DNAH7 | frameshift_deleti  on | exon41 | c.6887delT | p.V2296fs | . | . |
| --- | --- | --- | --- | --- | --- | --- | --- | --- | --- | --- | --- | --- | --- |
| CTC5 | 2 | 196852909 | A | AT | 0.4 | NM_018897 | DNAH7 | frameshift_inserti  on | exon13 | c.1397dupA | p.N466fs | . | . |
| CTC5 | 2 | 197559880 | TG | T | 1 | NM_001080539 | CCDC150 | frameshift_deleti  on | exon14 | c.1621delG | p.G541fs | . | . |
| CTC5 | 2 | 198508896 | CT | C | 1 | NM_144629 | RFTN2 | frameshift_deleti  on | exon3 | c.423delA | p.K141fs | . | . |
| CTC5 | 2 | 200173633 | TTC | T | 0.292 | NM_001172509 | SATB2 | frameshift_deleti  on | exon10 | c.1588_1589del | p.E530fs | . | . |
| CTC5 | 2 | 200173635 | C | CT | 0.364 | NM_001172509 | SATB2 | frameshift_inserti  on | exon10 | c.1587dupA | p.E530fs | . | . |
| CTC5 | 2 | 201473807 | TC | T | 0.167 | NM_001159 | AOX1 | frameshift_deleti  on | exon11 | c.1009delC | p.L337fs | . | . |
| CTC5 | 2 | 201757977 | ACC | A | 0.706 | NM_001142356 | NIF3L1 | frameshift_deleti  on | exon2 | c.446_447del | p.T149fs | . | . |
| CTC5 | 2 | 202900594 | CG | C | 0.278 | NM_003507 | FZD7 | frameshift_deleti  on | exon1 | c.1225delG | p.G409fs | . | . |
| CTC5 | 2 | 203922117 | AT | A | 0.333 | NM_001114132 | NBEAL1 | frameshift_deleti  on | exon6 | c.457delT | p.L153fs | . | . |
| CTC5 | 2 | 205912364 | A | AT | 0.389 | NM_057177 | PARD3B | frameshift_inserti  on | exon4 | c.455_456insT | p.Q152fs | . | . |
| CTC5 | 2 | 207620107 | CT | C | 0.245 | NM_001039845 | MDH1B | frameshift_deleti  on | exon5 | c.535delA | p.S179fs | . | . |
| CTC5 | 2 | 210882269 | C | CT | 0.188 | NM_001278288 | RPE | frameshift_inserti  on | exon4 | c.346_347insT | p.H116fs | . | . |
| CTC5 | 2 | 212488682 | CT | C | 0.379 | NM_001042599 | ERBB4 | frameshift_deleti  on | exon18 | c.2166delA | p.K722fs | . | . |
| CTC5 | 2 | 217279539 | TC | T | 0.228 | NM_001127207 | SMARCAL1 | frameshift_deleti  on | exon3 | c.113delC | p.S38fs | . | . |
| CTC5 | 2 | 218999694 | TG | T | 0.533 | NM_001557 | CXCR2 | frameshift_deleti  on | exon3 | c.171delG | p.L57fs | . | . |
| CTC5 | 2 | 219029449 | TC | T | 0.308 | NM_000634 | CXCR1 | frameshift_deleti  on | exon2 | c.485delG | p.G162fs | . | . |
| CTC5 | 2 | 220370793 | AG | A | 0.488 | NM_013335 | GMPPA | frameshift_deleti  on | exon11 | c.993delG | p.Q331fs | . | . |
| CTC5 | 2 | 220413941 | GA | G | 0.34 | NM_001005209 | TMEM198 | frameshift_deleti  on | exon4 | c.811delA | p.K271fs | . | . |
| CTC5 | 2 | 220414071 | TC | T | 0.956 | NM_001005209 | TMEM198 | frameshift_deleti  on | exon4 | c.941delC | p.S314fs | . | . |
| CTC5 | 2 | 220480011 | TGCTGCT  CTACCG CTCAGC CCCTGA | T | 0.4 | NM_052902 | STK11IP | nonframeshift_de letion | exon24 | c.3099_3131del | p.1033_1044de l | . | . |
| CTC5 | 2 | 220496782 | GC | G | 0.423 | NM_005070 | SLC4A3 | frameshift_deleti  on | exon7 | c.905delC | p.A302fs | . | . |
| CTC5 | 2 | 225634992 | TG | T | 0.5 | NM_014689 | DOCK10 | frameshift_deleti  on | exon55 | c.6379delC | p.Q2127fs | . | . |
| CTC5 | 2 | 230723706 | TG | T | 0.364 | NM_001284215 | TRIP12 | frameshift_deleti  on | exon2 | c.682delC | p.Q228fs | . | . |
| CTC5 | 2 | 231254654 | GA | G | 0.733 | NM_138402 | SP140L | frameshift_deleti  on | exon11 | c.881delA | p.E294fs | . | . |
| CTC5 | 2 | 231339123 | GAA | G | 0.174 | NM_001080391 | SP100 | frameshift_deleti  on | exon17 | c.1562_1563del | p.E521fs | . | . |
| CTC5 | 2 | 232325414 | TTCA | T | 0.919 | NM_005381 | NCL | nonframeshift_de  letion | exon4 | c.774_776del | p.258_259del | . | . |
| CTC5 | 2 | 234368483 | AG | A | 0.375 | NM_003648 | DGKD | frameshift_deleti  on | exon22 | c.2644delG | p.G882fs | . | . |
| CTC5 | 2 | 234375803 | GA | G | 0.5 | NM_003648 | DGKD | frameshift_deleti  on | exon27 | c.3247delA | p.K1083fs | . | . |
| CTC5 | 2 | 234638028 | CA | C | 0.37 | NM_019093 | UGT1A3 | frameshift_deleti  on | exon1 | c.257delA | p.Q86fs | . | . |
| CTC5 | 2 | 234726658 | AG | A | 0.341 | NM_001287395 | MROH2A | frameshift_deleti  on | exon28 | c.2982delG | p.K994fs | . | . |
| CTC5 | 2 | 234752872 | AG | A | 0.857 | NM_001282963 | HJURP | frameshift_deleti  on | exon4 | c.297delC | p.A99fs | . | . |
| CTC5 | 2 | 235961213 | TGAA | T | 0.4 | NM_014521 | SH3BP4 | nonframeshift_de  letion | exon5 | c.2487_2489del | p.829_830del | . | . |
| CTC5 | 2 | 236761414 | C | CCAGG | 0.5 | NM_001244888 | AGAP1 | frameshift_inserti  on | exon10 | c.1135_1136ins  CAGG | p.P379fs | . | . |
| CTC5 | 2 | 240036806 | TG | T | 0.4 | NM_006037 | HDAC4 | frameshift_deleti  on | exon13 | c.1718delC | p.P573fs | . | . |
| CTC5 | 2 | 240984547 | TC | T | 0.293 | NM_173351 | OR6B3 | frameshift_deleti  on | exon1 | c.942delG | p.G314fs | . | . |
| CTC5 | 2 | 242196133 | GT | G | 0.778 | NM_005336 | HDLBP | frameshift_deleti  on | exon6 | c.538delA | p.T180fs | . | . |
| CTC5 | 2 | 242432809 | CA | C | 0.353 | NM_014808 | FARP2 | frameshift_deleti  on | exon26 | c.2998delA | p.K1000fs | . | . |
| CTC5 | 2 | 242815106 | GT | G | 1 | NM_173821 | CXXC11 | frameshift_deleti  on | exon2 | c.1400delT | p.V467fs | . | . |
| CTC5 | 3 | 1269509 | CA | C | 0.692 | NM_001289080 | CNTN6 | frameshift_deleti  on | exon4 | c.191delA | p.Q64fs | . | . |
| CTC5 | 3 | 3195688 | TG | T | 0.647 | NM_001173482 | CRBN | frameshift_deleti  on | exon8 | c.903delC | p.G301fs | . | . |
| CTC5 | 3 | 4712461 | G | GA | 0.162 | NM_001168272 | ITPR1 | frameshift_inserti  on | exon19 | c.2011dupA | p.E670fs | . | . |
| CTC5 | 3 | 9512590 | CA | C | 0.435 | NM_001080517 | SETD5 | frameshift_deleti  on | exon19 | c.3173delA | p.Q1058fs | . | . |
| CTC5 | 3 | 12859308 | CA | C | 0.562 | NM_012298 | CAND2 | frameshift_deleti  on | exon8 | c.2599delA | p.I867fs | . | . |
| CTC5 | 3 | 18391132 | CCTG | C | 0.103 | NM_001131010 | SATB1 | nonframeshift_de  letion | exon11 | c.1819_1821del | p.607_607del | . | . |
| CTC5 | 3 | 30713129 | GA | G | 0.667 | NM_003242 | TGFBR2 | frameshift_deleti  on | exon4 | c.455delA | p.E152fs | . | . |
| CTC5 | 3 | 32932877 | TG | T | 0.233 | NM_001039111 | TRIM71 | frameshift_deleti  on | exon4 | c.2182delG | p.G728fs | . | . |
| CTC5 | 3 | 33038787 | CG | C | 0.469 | NM_001135602 | GLB1 | frameshift_deleti  on | exon13 | c.1390delC | p.R464fs | . | . |
| CTC5 | 3 | 33418820 | GC | G | 0.416 | NM_001171713 | FBXL2 | frameshift_deleti  on | exon10 | c.641delC | p.A214fs | . | . |
| CTC5 | 3 | 38051956 | C | CT | 0.235 | NM_001130964 | PLCD1 | frameshift_inserti  on | exon6 | c.950dupA | p.Q317fs | . | . |
| CTC5 | 3 | 38232289 | TA | T | 0.818 | NM_005109 | OXSR1 | frameshift_deleti  on | exon3 | c.252delA | p.V84fs | . | . |
| CTC5 | 3 | 38766748 | C | CT | 0.2 | NM_006514 | SCN10A | frameshift_inserti  on | exon17 | c.3144dupA | p.G1049fs | . | . |
| CTC5 | 3 | 38941546 | TA | T | 0.626 | NM_014139 | SCN11A | frameshift_deleti  on | exon13 | c.1860delT | p.F620fs | . | . |
| CTC5 | 3 | 39153963 | AGG | A | 0.143 | NM_145755 | TTC21A | frameshift_deleti  on | exon5 | c.451_452del | p.G151fs | . | . |
| CTC5 | 3 | 39185471 | AC | A | 0.449 | NM_033027 | CSRNP1 | frameshift_deleti  on | exon5 | c.844delG | p.V282fs | . | . |
| CTC5 | 3 | 39230294 | AG | A | 0.8 | NM_001198621 | XIRP1 | frameshift_deleti  on | exon2 | c.642delC | p.P214fs | . | . |

| CTC5 | 3 | 40503520 | A | ACTGC  TG | 1 | NM_001034996 | RPL14 | nonframeshift_in  sertion | exon6 | c.445_446insCT  GCTG | p.T149delinsTA  A | . | . |
| --- | --- | --- | --- | --- | --- | --- | --- | --- | --- | --- | --- | --- | --- |
| CTC5 | 3 | 41277878 | TG | T | 0.579 | NM_001098209 | CTNNB1 | frameshift_deleti  on | exon12 | c.1843delG | p.A615fs | . | . |
| CTC5 | 3 | 43618413 | C | CT | 0.172 | NM_001204833 | ANO10 | frameshift_inserti  on | exon4 | c.599dupA | p.Q200fs | . | . |
| CTC5 | 3 | 43647211 | T | TC | 0.75 | NM_001204831 | ANO10 | frameshift_inserti  on | exon2 | c.133dupG | p.D45fs | . | . |
| CTC5 | 3 | 44611727 | AG | A | 0.735 | NM_001288592 | ZKSCAN7 | frameshift_deleti  on | exon5 | c.673delG | p.V225fs | . | . |
| CTC5 | 3 | 44612495 | TG | T | 0.944 | NM_001288590 | ZKSCAN7 | frameshift_deleti  on | exon6 | c.1894delG | p.E632fs | . | . |
| CTC5 | 3 | 44700777 | TG | T | 0.472 | NM_003420 | ZNF35 | frameshift_deleti  on | exon4 | c.923delG | p.C308fs | . | . |
| CTC5 | 3 | 44986720 | TG | T | 0.393 | NM_001135179 | ZDHHC3 | frameshift_deleti  on | exon3 | c.370delC | p.Q124fs | . | . |
| CTC5 | 3 | 45077232 | GC | G | 0.503 | NM_003278 | CLEC3B | frameshift_deleti  on | exon3 | c.426delC | p.G142fs | . | . |
| CTC5 | 3 | 46306661 | ACT | A | 1 | NM_178329 | CCR3 | frameshift_deleti  on | exon2 | c.13_14del | p.L5fs | . | . |
| CTC5 | 3 | 46501284 | C | CCTT | 1 | NM_002343 | LTF | nonframeshift_in  sertion | exon2 | c.68_69insAAG | p.R23delinsRR | . | . |
| CTC5 | 3 | 46751073 | TAAG | T | 1 | NM_147196 | TMIE | nonframeshift_de  letion | exon4 | c.367_369del | p.123_123del | . | . |
| CTC5 | 3 | 47125609 | AT | A | 0.316 | NM_014159 | SETD2 | frameshift_deleti  on | exon12 | c.5660delA | p.N1887fs | . | . |
| CTC5 | 3 | 47163808 | GT | G | 0.906 | NM_014159 | SETD2 | frameshift_deleti  on | exon3 | c.2317delA | p.T773fs | . | . |
| CTC5 | 3 | 47165838 | CT | C | 0.5 | NM_014159 | SETD2 | frameshift_deleti  on | exon3 | c.287delA | p.K96fs | . | . |
| CTC5 | 3 | 47543281 | TG | T | 0.867 | NM_001031703 | ELP6 | frameshift_deleti  on | exon5 | c.383delC | p.P128fs | . | . |
| CTC5 | 3 | 47887879 | TC | T | 0.667 | NM_138615 | DHX30 | frameshift_deleti  on | exon11 | c.1318delC | p.R440fs | . | . |
| CTC5 | 3 | 48456281 | GT | G | 1 | NM_001130082 | PLXNB1 | frameshift_deleti  on | exon21 | c.4135delA | p.T1379fs | . | . |
| CTC5 | 3 | 48638193 | TG | T | 0.634 | NM_003365 | UQCRC1 | frameshift_deleti  on | exon9 | c.1046delC | p.A349fs | . | . |
| CTC5 | 3 | 49053697 | GC | G | 0.429 | NM_001009996 | DALRD3 | frameshift_deleti  on | exon9 | c.1222delG | p.A408fs | . | . |
| CTC5 | 3 | 49068012 | T | TA | 0.206 | NM_198880 | QRICH1 | frameshift_inserti  on | exon10 | c.2203_2204ins  T | p.N735fs | . | . |
| CTC5 | 3 | 49690944 | GC | G | 0.293 | NM_003458 | BSN | frameshift_deleti  on | exon5 | c.3956delC | p.A1319fs | . | . |
| CTC5 | 3 | 49695362 | T | TA | 0.444 | NM_003458 | BSN | frameshift_inserti  on | exon5 | c.8373_8374ins  A | p.P2791fs | . | . |
| CTC5 | 3 | 49713937 | TC | T | 0.983 | NM_001640 | APEH | frameshift_deleti  on | exon7 | c.734delC | p.S245fs | . | . |
| CTC5 | 3 | 50334564 | G | GT | 1 | NM_001200016 | NAT6 | frameshift_inserti  on | exon2 | c.330_331insA | p.H111fs | . | . |
| CTC5 | 3 | 50357542 | CA | C | 1 | NM_003773 | HYAL2 | frameshift_deleti  on | exon2 | c.378delT | p.S126fs | . | . |
| CTC5 | 3 | 51971723 | CA | C | 0.92 | NM_004704 | RRP9 | frameshift_deleti  on | exon4 | c.309delT | p.F103fs | . | . |
| CTC5 | 3 | 51993970 | A | AT | 0.256 | NM_033008 | PCBP4 | frameshift_inserti  on | exon7 | c.456dupA | p.S153fs | . | . |
| CTC5 | 3 | 52014447 | G | GA | 0.31 | NM_015407 | ABHD14A | frameshift_inserti  on | exon4 | c.437dupA | p.E146fs | . | . |
| CTC5 | 3 | 52233374 | T | TG | 0.281 | NM_199166 | ALAS1 | frameshift_inserti  on | exon2 | c.118dupG | p.V39fs | . | . |
| CTC5 | 3 | 52266061 | GC | G | 0.358 | NM_007284 | TWF2 | frameshift_deleti  on | exon3 | c.180delG | p.L60fs | . | . |
| CTC5 | 3 | 52436652 | GA | G | 0.286 | NM_004656 | BAP1 | frameshift_deleti  on | exon16 | c.2021delT | p.F674fs | . | . |
| CTC5 | 3 | 52620441 | C | CT | 1 | NM_018313 | PBRM1 | frameshift_inserti  on | exon21 | c.3311dupA | p.K1104fs | . | . |
| CTC5 | 3 | 52835025 | AT | A | 0.234 | NM_002217 | ITIH3 | frameshift_deleti  on | exon11 | c.1247delT | p.I416fs | . | . |
| CTC5 | 3 | 53778787 | TGAA | T | 0.306 | NM_001128839 | CACNA1D | nonframeshift_de  letion | exon23 | c.2940_2942del | p.980_981del | . | . |
| CTC5 | 3 | 53844280 | CA | C | 1 | NM_001128839 | CACNA1D | frameshift_deleti  on | exon45 | c.6076delA | p.K2026fs | . | . |
| CTC5 | 3 | 53889346 | T | TA | 1 | NM_018725 | IL17RB | frameshift_inserti  on | exon6 | c.508dupA | p.Y169fs | . | . |
| CTC5 | 3 | 54925454 | CG | C | 0.419 | NM_018398 | CACNA2D3 | frameshift_deleti  on | exon25 | c.2224delG | p.G742fs | . | . |
| CTC5 | 3 | 56593606 | CA | C | 0.667 | NM_001141947 | CCDC66 | frameshift_deleti  on | exon3 | c.87delA | p.S29fs | . | . |
| CTC5 | 3 | 56655611 | GA | G | 0.385 | NM_001012506 | CCDC66 | frameshift_deleti  on | exon18 | c.2711delA | p.E904fs | . | . |
| CTC5 | 3 | 56657097 | C | CA | 0.235 | NM_015224 | FAM208A | stopgain | exon17 | c.3669dupT | p.D1224_S1225  delinsX | . | . |
| CTC5 | 3 | 57314396 | TC | T | 0.17 | NM_001142733 | ASB14 | frameshift_deleti  on | exon7 | c.820delG | p.D274fs | . | . |
| CTC5 | 3 | 57432264 | CT | C | 1 | NM_178504 | DNAH12 | frameshift_deleti  on | exon26 | c.3869delA | p.K1290fs | . | . |
| CTC5 | 3 | 58097584 | GA | G | 0.242 | NM_001164317 | FLNB | frameshift_deleti  on | exon17 | c.2552delA | p.E851fs | . | . |
| CTC5 | 3 | 63981703 | GA | G | 0.796 | NM_001128149 | ATXN7 | frameshift_deleti  on | exon8 | c.1771delA | p.K591fs | . | . |
| CTC5 | 3 | 65342700 | GT | G | 0.5 | NM_001033057 | MAGI1 | frameshift_deleti  on | exon23 | c.3741delA | p.P1247fs | . | . |
| CTC5 | 3 | 65422875 | G | GC | 0.417 | NM_001033057 | MAGI1 | frameshift_inserti  on | exon10 | c.1317_1318ins  G | p.H440fs | . | . |
| CTC5 | 3 | 65425567 | C | CTGA | 1 | NM_001033057 | MAGI1 | nonframeshift_in  sertion | exon9 | c.1256_1257ins  TCA | p.Q419delinsH  Q | . | . |
| CTC5 | 3 | 66431212 | GT | G | 0.164 | NM_015541 | LRIG1 | frameshift_deleti  on | exon18 | c.2843delA | p.H948fs | . | . |
| CTC5 | 3 | 67054260 | GT | G | 0.455 | NM_032505 | KBTBD8 | frameshift_deleti  on | exon3 | c.870delT | p.C290fs | . | . |
| CTC5 | 3 | 69106448 | CT | C | 0.328 | NM_198195 | UBA3 | frameshift_deleti  on | exon11 | c.880delA | p.R294fs | . | . |
| CTC5 | 3 | 69988270 | CA | C | 0.808 | NM_000248 | MITF | frameshift_deleti  on | exon3 | c.284delA | p.Q95fs | . | . |
| CTC5 | 3 | 71021190 | CG | C | 0.651 | NM_001244810 | FOXP1 | frameshift_deleti  on | exon18 | c.1671delC | p.P557fs | . | . |
| CTC5 | 3 | 71021292 | TA | T | 0.662 | NM_001244810 | FOXP1 | frameshift_deleti  on | exon18 | c.1569delT | p.F523fs | . | . |
| CTC5 | 3 | 87027872 | CT | C | 0.281 | NM_016206 | VGLL3 | frameshift_deleti  on | exon2 | c.206delA | p.E69fs | . | . |
| CTC5 | 3 | 97594611 | GT | G | 0.375 | NM_153605 | CRYBG3 | frameshift_deleti  on | exon4 | c.4574delT | p.V1525fs | . | . |
| CTC5 | 3 | 100484715 | GA | G | 1 | NM_015429 | ABI3BP | frameshift_deleti  on | exon30 | c.2686delT | p.S896fs | . | . |

| CTC5 | 3 | 108103565 | AC | A | 0.5 | NM_014981 | MYH15 | frameshift_deleti  on | exon40 | c.5659delG | p.V1887fs | . | . |
| --- | --- | --- | --- | --- | --- | --- | --- | --- | --- | --- | --- | --- | --- |
| CTC5 | 3 | 108205292 | GC | G | 1 | NM_014981 | MYH15 | frameshift_deleti  on | exon11 | c.1012delG | p.A338fs | . | . |
| CTC5 | 3 | 111812285 | AT | A | 0.857 | NM_001171747 | C3orf52 | frameshift_deleti  on | exon2 | c.218delT | p.I73fs | . | . |
| CTC5 | 3 | 111831866 | G | GA | 0.91 | NM_024616 | C3orf52 | frameshift_inserti  on | exon5 | c.524dupA | p.E175fs | . | . |
| CTC5 | 3 | 112299876 | AT | A | 0.438 | NM_017945 | SLC35A5 | frameshift_deleti  on | exon6 | c.913delT | p.F305fs | . | . |
| CTC5 | 3 | 112548172 | CA | C | 0.452 | NM_001008784 | CD200R1L | frameshift_deleti  on | exon2 | c.105delT | p.F35fs | . | . |
| CTC5 | 3 | 112727194 | A | AT | 0.4 | NM_015412 | C3orf17 | frameshift_inserti  on | exon8 | c.1058dupA | p.H353fs | . | . |
| CTC5 | 3 | 112987264 | CA | C | 0.686 | NM_033254 | BOC | frameshift_deleti  on | exon5 | c.496delA | p.K166fs | . | . |
| CTC5 | 3 | 113376110 | TTGC | T | 1 | NM_001009899 | KIAA2018 | nonframeshift_de  letion | exon7 | c.4416_4418del | p.1472_1473de  l | . | . |
| CTC5 | 3 | 113376353 | TG | T | 0.373 | NM_001009899 | KIAA2018 | frameshift_deleti  on | exon7 | c.4175delC | p.P1392fs | . | . |
| CTC5 | 3 | 113378468 | AG | A | 1 | NM_001009899 | KIAA2018 | frameshift_deleti  on | exon7 | c.2060delC | p.P687fs | . | . |
| CTC5 | 3 | 113656971 | A | AT | 0.293 | NM_001172105 | GRAMD1C | frameshift_inserti  on | exon10 | c.1058dupT | p.I353fs | . | . |
| CTC5 | 3 | 113737652 | T | TC | 0.75 | NM_020817 | KIAA1407 | frameshift_inserti  on | exon8 | c.1035dupG | p.K346fs | . | . |
| CTC5 | 3 | 113737702 | TA | T | 0.333 | NM_020817 | KIAA1407 | frameshift_deleti  on | exon8 | c.985delT | p.Y329fs | . | . |
| CTC5 | 3 | 118865650 | G | GA | 0.198 | NM_152539 | C3orf30 | frameshift_inserti  on | exon1 | c.615dupA | p.R205fs | . | . |
| CTC5 | 3 | 119150895 | CG | C | 0.185 | NM_018266 | TMEM39A | frameshift_deleti  on | exon9 | c.1399delC | p.R467fs | . | . |
| CTC5 | 3 | 119150909 | TA | T | 0.303 | NM_018266 | TMEM39A | frameshift_deleti  on | exon9 | c.1385delT | p.L462fs | . | . |
| CTC5 | 3 | 121304902 | AAGC | A | 0.2 | NM_001012659 | ARGFX | nonframeshift_de  letion | exon5 | c.404_406del | p.135_136del | . | . |
| CTC5 | 3 | 121353230 | CA | C | 0.727 | NM_005335 | HCLS1 | frameshift_deleti  on | exon10 | c.726delT | p.F242fs | . | . |
| CTC5 | 3 | 122446706 | GA | G | 0.824 | NM_017554 | PARP14 | frameshift_deleti  on | exon16 | c.4990delA | p.K1664fs | . | . |
| CTC5 | 3 | 124351511 | TG | T | 0.341 | NM_007064 | KALRN | frameshift_deleti  on | exon2 | c.331delG | p.G111fs | . | . |
| CTC5 | 3 | 124436185 | GCT | G | 0.781 | NM_007064 | KALRN | frameshift_deleti  on | exon26 | c.3278_3279del | p.A1093fs | . | . |
| CTC5 | 3 | 127842558 | C | CA | 0.662 | NM_003707 | RUVBL1 | stopgain | exon1 | c.9dupT | p.E4_E5delinsX | . | . |
| CTC5 | 3 | 129238571 | TC | T | 0.333 | NM_052990 | IFT122 | frameshift_deleti on | exon26 | c.3303delC | p.F1101fs | . | . |
| CTC5 | 3 | 129546679 | TGCA | T | 0.221 | NM_001017395 | TMCC1 | nonframeshift_de  letion | exon3 | c.540_542del | p.180_181del | . | . |
| CTC5 | 3 | 133356724 | ACAT | A | 0.333 | NM_007027 | TOPBP1 | nonframeshift_de  letion | exon14 | c.2513_2515del | p.838_839del | . | . |
| CTC5 | 3 | 134277050 | GA | G | 0.455 | NM_025180 | CEP63 | frameshift_deleti  on | exon14 | c.1535delA | p.E512fs | . | . |
| CTC5 | 3 | 134362184 | TG | T | 0.327 | NM_178554 | KY | frameshift_deleti  on | exon3 | c.235delC | p.Q79fs | . | . |
| CTC5 | 3 | 135722083 | CA | C | 0.786 | NM_002718 | PPP2R3A | frameshift_deleti  on | exon2 | c.1744delA | p.K582fs | . | . |
| CTC5 | 3 | 137849806 | G | GA | 0.571 | NM_016161 | A4GNT | frameshift_inserti  on | exon2 | c.292dupT | p.S98fs | . | . |
| CTC5 | 3 | 139081250 | TCTG | T | 0.25 | NM_004766 | COPB2 | nonframeshift_de  letion | exon16 | c.1991_1993del | p.664_665del | . | . |
| CTC5 | 3 | 140407097 | CACA | C | 0.385 | NM_152616 | TRIM42 | nonframeshift_de  letion | exon3 | c.1574_1576del | p.525_526del | . | . |
| CTC5 | 3 | 140407098 | AC | A | 0.2 | NM_152616 | TRIM42 | frameshift_deleti  on | exon3 | c.1575delC | p.H525fs | . | . |
| CTC5 | 3 | 142242837 | CA | C | 0.4 | NM_001184 | ATR | frameshift_deleti  on | exon22 | c.4149delT | p.F1383fs | . | . |
| CTC5 | 3 | 142259834 | TG | T | 0.25 | NM_001184 | ATR | frameshift_deleti  on | exon18 | c.3492delC | p.P1164fs | . | . |
| CTC5 | 3 | 147123247 | TG | T | 1 | NM_001168379 | ZIC4 | frameshift_deleti  on | exon1 | c.11delC | p.P4fs | . | . |
| CTC5 | 3 | 148894124 | TTC | T | 0.154 | NM_000096 | CP | frameshift_deleti  on | exon18 | c.3092_3093del | p.R1031fs | . | . |
| CTC5 | 3 | 148925230 | GC | G | 0.787 | NM_000096 | CP | frameshift_deleti  on | exon5 | c.955delG | p.A319fs | . | . |
| CTC5 | 3 | 150881823 | TC | T | 0.355 | NM_053002 | MED12L | frameshift_deleti  on | exon8 | c.1252delC | p.Q418fs | . | . |
| CTC5 | 3 | 151090362 | CA | C | 0.364 | NM_053002 | MED12L | frameshift_deleti  on | exon25 | c.3568delA | p.K1190fs | . | . |
| CTC5 | 3 | 151105768 | C | CG | 0.182 | NM_053002 | MED12L | frameshift_inserti  on | exon35 | c.5154_5155ins  G | p.P1718fs | . | . |
| CTC5 | 3 | 151599057 | TG | T | 0.194 | NM_033050 | SUCNR1 | frameshift_deleti  on | exon3 | c.727delG | p.V243fs | . | . |
| CTC5 | 3 | 155838554 | G | GA | 0.208 | NM_172160 | KCNAB1 | frameshift_inserti  on | exon1 | c.155dupA | p.E52fs | . | . |
| CTC5 | 3 | 156867286 | CT | C | 1 | NM_020307 | CCNL1 | frameshift_deleti  on | exon9 | c.1120delA | p.S374fs | . | . |
| CTC5 | 3 | 160119733 | GT | G | 1 | NM_005496 | SMC4 | frameshift_deleti  on | exon2 | c.171delT | p.S57fs | . | . |
| CTC5 | 3 | 160156620 | CA | C | 0.176 | NM_173084 | TRIM59 | frameshift_deleti  on | exon3 | c.351delT | p.C117fs | . | . |
| CTC5 | 3 | 160156621 | AC | A | 0.222 | NM_173084 | TRIM59 | frameshift_deleti  on | exon3 | c.350delG | p.C117fs | . | . |
| CTC5 | 3 | 167189621 | AT | A | 0.824 | NM_006217 | SERPINI2 | frameshift_deleti  on | exon2 | c.1delA | p.M1fs | . | . |
| CTC5 | 3 | 168849292 | C | CA | 1 | NM_001105077 | MECOM | frameshift_inserti  on | exon4 | c.165dupT | p.A56fs | . | . |
| CTC5 | 3 | 169497261 | TA | T | 0.7 | NM_001185119 | MYNN | frameshift_deleti  on | exon2 | c.973delA | p.K325fs | . | . |
| CTC5 | 3 | 169497263 | AAG | A | 0.286 | NM_001185119 | MYNN | frameshift_deleti  on | exon2 | c.975_976del | p.K325fs | . | . |
| CTC5 | 3 | 170715736 | CT | C | 0.32 | NM_001278658 | SLC2A2 | frameshift_deleti  on | exon10 | c.1173delA | p.K391fs | . | . |
| CTC5 | 3 | 170723848 | ATG | A | 0.152 | NM_001278658 | SLC2A2 | frameshift_deleti  on | exon5 | c.300_301del | p.H100fs | . | . |
| CTC5 | 3 | 173997082 | GT | G | 0.247 | NM_014932 | NLGN1 | frameshift_deleti  on | exon6 | c.1292delT | p.V431fs | . | . |
| CTC5 | 3 | 176768330 | GC | G | 0.538 | NM_024665 | TBL1XR1 | frameshift_deleti  on | exon6 | c.495delG | p.L165fs | . | . |
| CTC5 | 3 | 179597879 | TC | T | 1 | NM_001256753 | PEX5L | frameshift_deleti  on | exon3 | c.165delG | p.L55fs | . | . |
| CTC5 | 3 | 180327613 | GA | G | 0.305 | NM_001288582 | TTC14 | frameshift_deleti  on | exon12 | c.1597delA | p.K533fs | . | . |

| CTC5 | 3 | 180334069 | C | CT | 0.167 | NM_181426 | CCDC39 | frameshift_inserti  on | exon19 | c.2668dupA | p.R890fs | . | . |
| --- | --- | --- | --- | --- | --- | --- | --- | --- | --- | --- | --- | --- | --- |
| CTC5 | 3 | 182788893 | TA | T | 0.305 | NM_020166 | MCCC1 | frameshift_deleti  on | exon7 | c.654delT | p.V218fs | . | . |
| CTC5 | 3 | 182925474 | TC | T | 0.455 | NM_015078 | MCF2L2 | frameshift_deleti  on | exon23 | c.2633delG | p.G878fs | . | . |
| CTC5 | 3 | 183490096 | GT | G | 0.556 | NM_018023 | YEATS2 | frameshift_deleti  on | exon16 | c.1952delT | p.V651fs | . | . |
| CTC5 | 3 | 183958687 | TG | T | 0.783 | NM_138345 | VWA5B2 | frameshift_deleti  on | exon16 | c.2850delG | p.L950fs | . | . |
| CTC5 | 3 | 185190961 | T | TG | 0.27 | NM_001242317 | MAP3K13 | frameshift_inserti  on | exon10 | c.1221_1222ins  G | p.N407fs | . | . |
| CTC5 | 3 | 195306262 | TG | T | 0.462 | NM_001647 | APOD | frameshift_deleti  on | exon2 | c.70delC | p.H24fs | . | . |
| CTC5 | 3 | 195518112 | T | TGTCTC  CTGCG | 1 | NM_018406 | MUC4 | nonframeshift_in  sertion | exon2 | c.338_339insTG  TTACGCAGGA | p.T113delinsTV  TQET | . | . |
| CTC5 | 3 | 196281096 | AG | A | 0.444 | NM_182627 | WDR53 | frameshift_deleti  on | exon4 | c.1062delC | p.P354fs | . | . |
| CTC5 | 3 | 196743997 | AGAT | A | 1 | NM_005929 | MFI2 | nonframeshift_de  letion | exon7 | c.874_876del | p.292_292del | . | . |
| CTC5 | 3 | 197678102 | TA | T | 0.519 | NM_000996 | RPL35A | frameshift_deleti  on | exon3 | c.85delA | p.K29fs | . | . |
| CTC5 | 4 | 1087327 | G | GCTGC  CCAGG CTGGA | 1 | NM_001193318 | RNF212 | nonframeshift_in sertion | exon4 | c.721_722insG  GCTGGCTCCA GCCTGGGCAG | p.S241delinsW LAPAWAA | . | . |
| CTC5 | 4 | 1902500 | TG | T | 0.364 | NM_133334 | WHSC1 | frameshift_deleti  on | exon1 | c.120delG | p.V40fs | . | . |
| CTC5 | 4 | 2461922 | GC | G | 0.262 | NM_001193282 | RP11-  503N18.1 | frameshift_deleti  on | exon14 | c.1615delC | p.Q539fs | . | . |
| CTC5 | 4 | 2641578 | AC | A | 0.296 | NM_001256666 | FAM193A | frameshift_deleti  on | exon4 | c.283delC | p.Q95fs | . | . |
| CTC5 | 4 | 2829034 | CG | C | 0.786 | NM_001122681 | SH3BP2 | frameshift_deleti  on | exon6 | c.507delG | p.T169fs | . | . |
| CTC5 | 4 | 2955283 | GT | G | 0.231 | NM_003703 | NOP14 | frameshift_deleti  on | exon5 | c.701delA | p.H234fs | . | . |
| CTC5 | 4 | 3021394 | CA | C | 0.833 | NM_001004056 | GRK4 | frameshift_deleti  on | exon8 | c.673delA | p.K225fs | . | . |
| CTC5 | 4 | 4411360 | TA | T | 0.375 | NM_001040101 | NSG1 | frameshift_deleti  on | exon4 | c.308delA | p.Y103fs | . | . |
| CTC5 | 4 | 8037917 | AG | A | 0.361 | NM_001286688 | ABLIM2 | frameshift_deleti  on | exon5 | c.276delC | p.P92fs | . | . |
| CTC5 | 4 | 8218717 | TGA | T | 0.5 | NM_018986 | SH3TC1 | frameshift_deleti  on | exon7 | c.663_664del | p.V221fs | . | . |
| CTC5 | 4 | 8398711 | CA | C | 0.2 | NM_001101667 | ACOX3 | frameshift_deleti  on | exon9 | c.1008delT | p.F336fs | . | . |
| CTC5 | 4 | 13606586 | AT | A | 0.96 | NM_148894 | BOD1L1 | frameshift_deleti  on | exon10 | c.1937delA | p.N646fs | . | . |
| CTC5 | 4 | 15504493 | A | AT | 0.364 | NM_001080522 | CC2D2A | frameshift_inserti  on | exon7 | c.385_386insT | p.R129fs | . | . |
| CTC5 | 4 | 15964507 | C | CT | 1 | NM_031950 | FGFBP2 | frameshift_inserti  on | exon1 | c.245dupA | p.Q82fs | . | . |
| CTC5 | 4 | 17885977 | GT | G | 0.857 | NM_001166139 | LCORL | frameshift_deleti  on | exon7 | c.1174delA | p.T392fs | . | . |
| CTC5 | 4 | 25676161 | ACT | A | 0.286 | NM_001177998 | SLC34A2 | frameshift_deleti  on | exon12 | c.1366_1367del | p.L456fs | . | . |
| CTC5 | 4 | 25783918 | A | AT | 0.333 | NM_015187 | SEL1L3 | frameshift_inserti  on | exon15 | c.2402dupA | p.N801fs | . | . |
| CTC5 | 4 | 37592529 | TG | T | 0.839 | NM_018302 | C4orf19 | frameshift_deleti  on | exon3 | c.853delG | p.G285fs | . | . |
| CTC5 | 4 | 38879932 | TG | T | 0.333 | NM_138389 | FAM114A1 | frameshift_deleti  on | exon3 | c.234delG | p.L78fs | . | . |
| CTC5 | 4 | 38879940 | C | CG | 0.324 | NM_138389 | FAM114A1 | frameshift_inserti  on | exon3 | c.241_242insG | p.P81fs | . | . |
| CTC5 | 4 | 39230259 | CG | C | 0.298 | NM_025132 | WDR19 | frameshift_deleti  on | exon17 | c.1932delG | p.T644fs | . | . |
| CTC5 | 4 | 39558079 | CT | C | 0.5 | NM_174921 | SMIM14 | frameshift_deleti  on | exon4 | c.235delA | p.S79fs | . | . |
| CTC5 | 4 | 40122489 | G | GA | 0.385 | NM_018177 | N4BP2 | frameshift_inserti  on | exon9 | c.2759dupA | p.E920fs | . | . |
| CTC5 | 4 | 42077774 | GA | G | 1 | NM_006345 | SLC30A9 | frameshift_deleti  on | exon16 | c.1520delA | p.E507fs | . | . |
| CTC5 | 4 | 42895586 | CA | C | 0.323 | NM_001080476 | GRXCR1 | frameshift_deleti  on | exon1 | c.304delA | p.K102fs | . | . |
| CTC5 | 4 | 46053606 | C | CT | 0.171 | NM_173536 | GABRG1 | frameshift_inserti  on | exon8 | c.965dupA | p.K322fs | . | . |
| CTC5 | 4 | 47645049 | AAC | A | 0.222 | NM_001278586 | CORIN | frameshift_deleti  on | exon14 | c.2069_2070del | p.C690fs | . | . |
| CTC5 | 4 | 47645050 | AC | A | 0.739 | NM_001278586 | CORIN | frameshift_deleti  on | exon14 | c.2069delG | p.C690fs | . | . |
| CTC5 | 4 | 47680007 | AC | A | 0.385 | NM_001278585 | CORIN | frameshift_deleti  on | exon7 | c.884delG | p.C295fs | . | . |
| CTC5 | 4 | 55139729 | A | AT | 0.462 | NM_006206 | PDGFRA | frameshift_inserti  on | exon10 | c.1391dupT | p.I464fs | . | . |
| CTC5 | 4 | 57182073 | GC | G | 0.604 | NM_020722 | KIAA1211 | frameshift_deleti  on | exon8 | c.2406delC | p.S802fs | . | . |
| CTC5 | 4 | 57865897 | AT | A | 0.25 | NM_000938 | POLR2B | frameshift_deleti  on | exon7 | c.851delT | p.I284fs | . | . |
| CTC5 | 4 | 57888322 | GT | G | 1 | NM_000938 | POLR2B | frameshift_deleti  on | exon18 | c.2426delT | p.V809fs | . | . |
| CTC5 | 4 | 69097053 | AT | A | 0.237 | NM_182502 | TMPRSS11B | frameshift_deleti  on | exon7 | c.553delA | p.I185fs | . | . |
| CTC5 | 4 | 69197826 | CT | C | 0.303 | NM_133370 | YTHDC1 | frameshift_deleti  on | exon6 | c.1062delA | p.K354fs | . | . |
| CTC5 | 4 | 69343324 | A | AT | 0.26 | NM_014058 | TMPRSS11E | frameshift_inserti  on | exon8 | c.946dupT | p.G315fs | . | . |
| CTC5 | 4 | 70080124 | CA | C | 0.342 | NM_001073 | UGT2B11 | frameshift_deleti  on | exon1 | c.316delT | p.W106fs | . | . |
| CTC5 | 4 | 70146347 | GA | G | 0.364 | NM_001207004 | UGT2B28 | frameshift_deleti  on | exon1 | c.130delA | p.K44fs | . | . |
| CTC5 | 4 | 70355157 | CT | C | 0.381 | NM_021139 | UGT2B4 | frameshift_deleti  on | exon3 | c.1001delA | p.K334fs | . | . |
| CTC5 | 4 | 73012960 | CT | C | 0.571 | NM_004885 | NPFFR2 | frameshift_deleti  on | exon4 | c.1001delT | p.L334fs | . | . |
| CTC5 | 4 | 73930533 | GC | G | 0.345 | NM_173827 | COX18 | frameshift_deleti  on | exon4 | c.681delG | p.L227fs | . | . |
| CTC5 | 4 | 73990935 | TC | T | 0.172 | NM_198889 | ANKRD17 | frameshift_deleti  on | exon16 | c.2575delG | p.E859fs | . | . |
| CTC5 | 4 | 74308116 | GA | G | 0.389 | NM_001134 | AFP | frameshift_deleti  on | exon5 | c.587delA | p.E196fs | . | . |
| CTC5 | 4 | 74442142 | TG | T | 0.593 | NM_001270391 | RASSF6 | frameshift_deleti  on | exon9 | c.820delC | p.Q274fs | . | . |
| CTC5 | 4 | 74853290 | GT | G | 0.4 | NM_002704 | PPBP | frameshift_deleti  on | exon2 | c.227delA | p.N76fs | . | . |
| CTC5 | 4 | 77025758 | A | AT | 0.276 | NM_001130016 | ART3 | frameshift_inserti  on | exon10 | c.980dupT | p.I327fs | . | . |

| CTC5 | 4 | 77303856 | CT | C | 0.714 | NM_001042784 | CCDC158 | frameshift_deleti  on | exon7 | c.820delA | p.S274fs | . | . |
| --- | --- | --- | --- | --- | --- | --- | --- | --- | --- | --- | --- | --- | --- |
| CTC5 | 4 | 77357321 | GT | G | 0.667 | NM_020859 | SHROOM3 | frameshift_deleti  on | exon1 | c.117delT | p.G39fs | . | . |
| CTC5 | 4 | 79362326 | GA | G | 0.556 | NM_001166133 | FRAS1 | frameshift_deleti  on | exon41 | c.5541delA | p.G1847fs | . | . |
| CTC5 | 4 | 79518499 | CA | C | 0.4 | NM_005139 | ANXA3 | frameshift_deleti  on | exon10 | c.662delA | p.Q221fs | . | . |
| CTC5 | 4 | 79792082 | CCAG | C | 0.188 | NM_017593 | BMP2K | nonframeshift_de  letion | exon11 | c.1378_1380del | p.460_460del | . | . |
| CTC5 | 4 | 79792136 | ACAGCA  GCAG | A | 1 | NM_017593 | BMP2K | nonframeshift_de  letion | exon11 | c.1432_1440del | p.478_480del | . | . |
| CTC5 | 4 | 79792136 | ACAGCA  GCAGCA | A | 1 | NM_017593 | BMP2K | nonframeshift_de  letion | exon11 | c.1432_1443del | p.478_481del | . | . |
| CTC5 | 4 | 79792154 | GCAGCA  GCAGCA | G | 0.882 | NM_017593 | BMP2K | nonframeshift_de  letion | exon11 | c.1450_1461del | p.484_487del | . | . |
| CTC5 | 4 | 79792154 | GCAGCA  GCAGCA | G | 0.882 | NM_017593 | BMP2K | nonframeshift_de  letion | exon11 | c.1450_1464del | p.484_488del | . | . |
| CTC5 | 4 | 85611757 | TG | T | 1 | NM_014991 | WDFY3 | frameshift_deleti  on | exon61 | c.9264delC | p.P3088fs | . | . |
| CTC5 | 4 | 86916615 | AC | A | 1 | NM_031305 | ARHGAP24 | frameshift_deleti  on | exon6 | c.1530delC | p.H510fs | . | . |
| CTC5 | 4 | 87656763 | A | AG | 0.727 | NM_006264 | PTPN13 | stopgain | exon15 | c.2168_2169ins  G | p.Y723_F724del  insX | . | . |
| CTC5 | 4 | 87656870 | GA | G | 0.769 | NM_006264 | PTPN13 | frameshift_deleti  on | exon15 | c.2276delA | p.E759fs | . | . |
| CTC5 | 4 | 87672254 | A | AC | 0.73 | NM_006264 | PTPN13 | frameshift_inserti  on | exon19 | c.3144dupC | p.D1048fs | . | . |
| CTC5 | 4 | 88766724 | AT | A | 0.975 | NM_001184694 | MEPE | frameshift_deleti  on | exon4 | c.705delT | p.D235fs | . | . |
| CTC5 | 4 | 89199490 | CT | C | 0.8 | NM_152542 | PPM1K | frameshift_deleti  on | exon2 | c.245delA | p.K82fs | . | . |
| CTC5 | 4 | 89414220 | TA | T | 0.308 | NM_016323 | HERC5 | frameshift_deleti  on | exon17 | c.2192delA | p.Y731fs | . | . |
| CTC5 | 4 | 89702398 | CA | C | 0.6 | NM_001265580 | FAM13A | frameshift_deleti  on | exon3 | c.313delT | p.C105fs | . | . |
| CTC5 | 4 | 94750143 | GC | G | 0.314 | NM_005172 | ATOH1 | frameshift_deleti  on | exon1 | c.67delC | p.P23fs | . | . |
| CTC5 | 4 | 95220696 | TC | T | 0.532 | NM_014485 | HPGDS | frameshift_deleti  on | exon6 | c.534delG | p.R178fs | . | . |
| CTC5 | 4 | 100131650 | AC | A | 0.167 | NM_000672 | ADH6 | frameshift_deleti  on | exon4 | c.271delG | p.V91fs | . | . |
| CTC5 | 4 | 100235073 | GA | G | 0.55 | NM_000668 | ADH1B | frameshift_deleti  on | exon6 | c.732delT | p.P244fs | . | . |
| CTC5 | 4 | 106616802 | TG | T | 0.55 | NM_001142471 | INTS12 | stopgain | exon3 | c.179delC | p.S60X | . | . |
| CTC5 | 4 | 106638821 | CT | C | 0.778 | NM_001031720 | GSTCD | frameshift_deleti on | exon2 | c.52delT | p.F18fs | . | . |
| CTC5 | 4 | 110902091 | GA | G | 0.382 | NM_001178131 | EGF | frameshift_deleti  on | exon14 | c.2206delA | p.K736fs | . | . |
| CTC5 | 4 | 111539596 | TG | T | 1 | NM_000325 | PITX2 | frameshift_deleti  on | exon3 | c.659delC | p.P220fs | . | . |
| CTC5 | 4 | 119947961 | AAAG | A | 0.5 | NM_001128933 | SYNPO2 | nonframeshift_de  letion | exon3 | c.438_440del | p.146_147del | . | . |
| CTC5 | 4 | 120221633 | A | AC | 0.625 | NM_001001701 | C4orf3 | frameshift_inserti  on | exon1 | c.57_58insG | p.F20fs | . | . |
| CTC5 | 4 | 123132148 | CT | C | 0.5 | NM_015312 | KIAA1109 | frameshift_deleti  on | exon18 | c.2146delT | p.S716fs | . | . |
| CTC5 | 4 | 123151198 | AT | A | 0.256 | NM_015312 | KIAA1109 | frameshift_deleti  on | exon24 | c.3156delT | p.N1052fs | . | . |
| CTC5 | 4 | 123161233 | G | GT | 0.286 | NM_015312 | KIAA1109 | frameshift_inserti  on | exon27 | c.4397dupT | p.V1466fs | . | . |
| CTC5 | 4 | 123280729 | AT | A | 1 | NM_015312 | KIAA1109 | frameshift_deleti  on | exon83 | c.14654delT | p.I4885fs | . | . |
| CTC5 | 4 | 125593448 | TC | T | 0.6 | NM_001167882 | ANKRD50 | frameshift_deleti  on | exon3 | c.446delG | p.G149fs | . | . |
| CTC5 | 4 | 126239245 | AC | A | 0.8 | NM_024582 | FAT4 | frameshift_deleti  on | exon1 | c.1680delC | p.H560fs | . | . |
| CTC5 | 4 | 126370479 | G | GC | 0.516 | NM_024582 | FAT4 | frameshift_inserti  on | exon9 | c.8309dupC | p.A2770fs | . | . |
| CTC5 | 4 | 126372305 | T | TG | 0.213 | NM_024582 | FAT4 | frameshift_inserti  on | exon9 | c.10135dupG | p.F3378fs | . | . |
| CTC5 | 4 | 128665924 | AT | A | 0.245 | NM_031291 | SLC25A31 | frameshift_deleti  on | exon2 | c.331delT | p.F111fs | . | . |
| CTC5 | 4 | 128807062 | AC | A | 0.903 | NM_001190799 | PLK4 | frameshift_deleti  on | exon4 | c.442delC | p.P148fs | . | . |
| CTC5 | 4 | 138442190 | TG | T | 1 | NM_019035 | PCDH18 | frameshift_deleti  on | exon4 | c.3400delC | p.Q1134fs | . | . |
| CTC5 | 4 | 139163201 | GAC | G | 0.25 | NM_014331 | SLC7A11 | frameshift_deleti  on | exon1 | c.21_22del | p.V7fs | . | . |
| CTC5 | 4 | 140811063 | TTGCTGC  TGCTGC | T | 1 | NM_018717 | MAML3 | frameshift_deleti  on | exon3 | c.1513_1514del | p.Q505fs | . | . |
| CTC5 | 4 | 140811063 | TTGCTGC  TGCTGCT | T | 1 | NM_018717 | MAML3 | nonframeshift_de  letion | exon2 | c.1512_1514del | p.504_505del | . | . |
| CTC5 | 4 | 141484544 | GT | G | 0.3 | NM_021833 | UCP1 | frameshift_deleti  on | exon3 | c.453delA | p.K151fs | . | . |
| CTC5 | 4 | 144141492 | TG | T | 0.511 | NM_032557 | USP38 | frameshift_deleti  on | exon10 | c.3013delG | p.A1005fs | . | . |
| CTC5 | 4 | 151850130 | AT | A | 0.926 | NM_001199282 | LRBA | frameshift_deleti  on | exon3 | c.303delA | p.K101fs | . | . |
| CTC5 | 4 | 153268089 | AT | A | 0.732 | NM_001013415 | FBXW7 | frameshift_deleti  on | exon3 | c.364delA | p.M122fs | . | . |
| CTC5 | 4 | 153896098 | TG | T | 0.357 | NM_033393 | FHDC1 | frameshift_deleti  on | exon11 | c.1656delG | p.L552fs | . | . |
| CTC5 | 4 | 155244401 | TTTTG | T | 1 | NM_001142552 | DCHS2 | frameshift_deleti  on | exon8 | c.4095_4098del | p.N1365fs | . | . |
| CTC5 | 4 | 159161505 | AC | A | 0.684 | NM_018342 | TMEM144 | frameshift_deleti  on | exon10 | c.738delC | p.Y246fs | . | . |
| CTC5 | 4 | 159894430 | AT | A | 1 | NM_152543 | C4orf45 | frameshift_deleti  on | exon2 | c.97delA | p.I33fs | . | . |
| CTC5 | 4 | 166924615 | TG | T | 1 | NM_001204760 | TLL1 | frameshift_deleti  on | exon6 | c.706delG | p.G236fs | . | . |
| CTC5 | 4 | 169305801 | CCAG | C | 0.333 | NM_001012967 | DDX60L | nonframeshift_de  letion | exon30 | c.4075_4077del | p.1359_1359de  l | . | . |
| CTC5 | 4 | 169931216 | CA | C | 0.571 | NM_032783 | CBR4 | frameshift_deleti  on | exon1 | c.24delT | p.F8fs | . | . |
| CTC5 | 4 | 170652868 | CA | C | 0.593 | NM_017867 | C4orf27 | frameshift_deleti  on | exon7 | c.895delT | p.C299fs | . | . |
| CTC5 | 4 | 175416732 | CT | C | 0.6 | NM_001256306 | HPGD | frameshift_deleti  on | exon3 | c.260delA | p.K87fs | . | . |
| CTC5 | 4 | 186444538 | AT | A | 0.312 | NM_001114107 | PDLIM3 | frameshift_deleti  on | exon3 | c.307delA | p.I103fs | . | . |
| CTC5 | 4 | 187455619 | AC | A | 0.586 | NM_005958 | MTNR1A | frameshift_deleti  on | exon2 | c.276delG | p.G92fs | . | . |

| CTC5 | 4 | 187557832 | GTCT | G | 0.176 | NM_005245 | FAT1 | nonframeshift_de  letion | exon5 | c.3876_3878del | p.1292_1293de  l | . | . |
| --- | --- | --- | --- | --- | --- | --- | --- | --- | --- | --- | --- | --- | --- |
| CTC5 | 4 | 189063579 | CA | C | 0.35 | NM_178556 | TRIML1 | frameshift_deleti  on | exon3 | c.679delA | p.K227fs | . | . |
| CTC5 | 5 | 475100 | CG | C | 0.236 | NM_001284351 | SLC9A3 | frameshift_deleti  on | exon16 | c.2371delC | p.R791fs | . | . |
| CTC5 | 5 | 5200352 | GC | G | 0.462 | NM_139056 | ADAMTS16 | stopgain | exon9 | c.1422delC | p.C474X | . | . |
| CTC5 | 5 | 6607022 | AG | A | 0.2 | NM_001193455 | NSUN2 | frameshift_deleti on | exon13 | c.1406delC | p.P469fs | . | . |
| CTC5 | 5 | 10391767 | AGAG | A | 0.312 | NM_001270661 | 6-Mar | nonframeshift_de  letion | exon4 | c.376_378del | p.126_126del | . | . |
| CTC5 | 5 | 13753444 | AT | A | 0.265 | NM_001369 | DNAH5 | frameshift_deleti  on | exon63 | c.10769delA | p.N3590fs | . | . |
| CTC5 | 5 | 13841159 | G | GA | 0.375 | NM_001369 | DNAH5 | frameshift_inserti  on | exon34 | c.5564dupT | p.I1855fs | . | . |
| CTC5 | 5 | 13841906 | CAA | C | 0.25 | NM_001369 | DNAH5 | frameshift_deleti  on | exon33 | c.5377_5378del | p.L1793fs | . | . |
| CTC5 | 5 | 16694592 | AC | A | 0.9 | NM_012334 | MYO10 | frameshift_deleti  on | exon27 | c.3687delG | p.L1229fs | . | . |
| CTC5 | 5 | 19483544 | GTGC | G | 0.3 | NM_001167667 | CDH18 | nonframeshift_de  letion | exon12 | c.1636_1638del | p.546_546del | . | . |
| CTC5 | 5 | 23522807 | AG | A | 1 | NM_020227 | PRDM9 | frameshift_deleti  on | exon8 | c.696delG | p.K232fs | . | . |
| CTC5 | 5 | 26881622 | C | CG | 0.667 | NM_016279 | CDH9 | frameshift_inserti  on | exon12 | c.1992dupC | p.G665fs | . | . |
| CTC5 | 5 | 33461063 | AT | A | 0.824 | NM_152295 | TARS | frameshift_deleti  on | exon12 | c.1308delT | p.D436fs | . | . |
| CTC5 | 5 | 33546231 | CT | C | 0.573 | NM_030955 | ADAMTS12 | frameshift_deleti  on | exon22 | c.4378delA | p.R1460fs | . | . |
| CTC5 | 5 | 35084606 | G | GA | 0.95 | NM_001204315 | PRLR | frameshift_inserti  on | exon4 | c.338dupT | p.F113fs | . | . |
| CTC5 | 5 | 35084607 | A | AT | 0.417 | NM_001204315 | PRLR | frameshift_inserti  on | exon4 | c.337_338insA | p.F113fs | . | . |
| CTC5 | 5 | 35776457 | GA | G | 0.438 | NM_024867 | SPEF2 | frameshift_deleti  on | exon29 | c.4178delA | p.E1393fs | . | . |
| CTC5 | 5 | 36117982 | TA | T | 0.825 | NM_001007527 | LMBRD2 | frameshift_deleti  on | exon10 | c.1156delT | p.Y386fs | . | . |
| CTC5 | 5 | 36163822 | AG | A | 0.429 | NM_005983 | SKP2 | frameshift_deleti  on | exon3 | c.357delG | p.K119fs | . | . |
| CTC5 | 5 | 37064104 | TA | T | 0.241 | NM_015384 | NIPBL | frameshift_deleti  on | exon46 | c.8074delA | p.I2692fs | . | . |
| CTC5 | 5 | 41004467 | G | GT | 0.323 | NM_173489 | MROH2B | frameshift_inserti  on | exon37 | c.4174dupA | p.T1392fs | . | . |
| CTC5 | 5 | 41203274 | TG | T | 0.278 | NM_000065 | C6 | frameshift_deleti  on | exon2 | c.58delC | p.Q20fs | . | . |
| CTC5 | 5 | 41382494 | CA | C | 0.675 | NM_001005473 | PLCXD3 | frameshift_deleti  on | exon2 | c.245delT | p.M82fs | . | . |
| CTC5 | 5 | 42719469 | G | GT | 0.273 | NM_001242460 | GHR | frameshift_inserti  on | exon8 | c.1795dupT | p.E598fs | . | . |
| CTC5 | 5 | 43161995 | AT | A | 1 | NM_003432 | ZNF131 | frameshift_deleti  on | exon6 | c.915delT | p.H305fs | . | . |
| CTC5 | 5 | 56177848 | TCAA | T | 0.16 | NM_005921 | MAP3K1 | nonframeshift_de  letion | exon14 | c.2822_2824del | p.941_942del | . | . |
| CTC5 | 5 | 57790543 | A | AT | 0.184 | NM_152687 | GAPT | frameshift_inserti  on | exon3 | c.181dupT | p.T60fs | . | . |
| CTC5 | 5 | 58272275 | GT | G | 0.472 | NM_001197222 | PDE4D | frameshift_deleti  on | exon8 | c.1059delA | p.K353fs | . | . |
| CTC5 | 5 | 64084780 | AT | A | 0.667 | NM_005869 | CWC27 | frameshift_deleti  on | exon7 | c.603delT | p.N201fs | . | . |
| CTC5 | 5 | 65455048 | TA | T | 0.455 | NM_001077199 | SREK1 | frameshift_deleti  on | exon3 | c.298delA | p.K100fs | . | . |
| CTC5 | 5 | 68805355 | CT | C | 0.281 | NM_001205254 | OCLN | frameshift_deleti  on | exon3 | c.439delT | p.F147fs | . | . |
| CTC5 | 5 | 68830634 | GT | G | 0.345 | NM_001205255 | OCLN | frameshift_deleti  on | exon3 | c.253delT | p.F85fs | . | . |
| CTC5 | 5 | 70806832 | G | GT | 0.433 | NM_018429 | BDP1 | frameshift_inserti  on | exon17 | c.3914dupT | p.V1305fs | . | . |
| CTC5 | 5 | 71490594 | CT | C | 0.32 | NM_005909 | MAP1B | frameshift_deleti  on | exon5 | c.1413delT | p.S471fs | . | . |
| CTC5 | 5 | 71491568 | GCT | G | 0.161 | NM_005909 | MAP1B | frameshift_deleti  on | exon5 | c.2387_2388del | p.A796fs | . | . |
| CTC5 | 5 | 72189445 | TA | T | 0.524 | NM_002270 | TNPO1 | frameshift_deleti  on | exon18 | c.2059delA | p.K687fs | . | . |
| CTC5 | 5 | 74021846 | TACTCA  A | T | 0.667 | NM_170691 | GFM2 | nonframeshift_de  letion | exon17 | c.1685_1690del | p.562_564del | . | . |
| CTC5 | 5 | 74807267 | AGCC | A | 0.26 | NM_001130105 | COL4A3BP | nonframeshift_de  letion | exon2 | c.147_149del | p.49_50del | . | . |
| CTC5 | 5 | 75964569 | AT | A | 0.286 | NM_001285461 | IQGAP2 | frameshift_deleti  on | exon11 | c.1232delT | p.M411fs | . | . |
| CTC5 | 5 | 78325853 | C | CA | 0.5 | NM_013391 | DMGDH | frameshift_inserti  on | exon11 | c.1687_1688ins  T | p.G563fs | . | . |
| CTC5 | 5 | 79361273 | TC | T | 0.411 | NM_003248 | THBS4 | frameshift_deleti  on | exon9 | c.1168delC | p.P390fs | . | . |
| CTC5 | 5 | 79443088 | CA | C | 0.316 | NM_001174071 | SERINC5 | frameshift_deleti  on | exon10 | c.1072delT | p.C358fs | . | . |
| CTC5 | 5 | 79616165 | C | CA | 0.875 | NM_032567 | SPZ1 | frameshift_inserti  on | exon1 | c.131_132insA | p.P44fs | . | . |
| CTC5 | 5 | 82940426 | CG | C | 0.821 | NM_001884 | HAPLN1 | frameshift_deleti  on | exon4 | c.530delC | p.A177fs | . | . |
| CTC5 | 5 | 83356280 | TC | T | 0.496 | NM_001278642 | EDIL3 | frameshift_deleti  on | exon8 | c.945delG | p.M315fs | . | . |
| CTC5 | 5 | 88100444 | CA | C | 0.187 | NM_001193348 | MEF2C | frameshift_deleti  on | exon2 | c.228delT | p.H76fs | . | . |
| CTC5 | 5 | 90083926 | CT | C | 0.6 | NM_032119 | GPR98 | frameshift_deleti  on | exon68 | c.13693delT | p.L4565fs | . | . |
| CTC5 | 5 | 96215637 | A | AC | 0.385 | NM_001130140 | ERAP2 | frameshift_inserti  on | exon2 | c.249dupC | p.H83fs | . | . |
| CTC5 | 5 | 96430711 | G | GA | 1 | NM_153234 | LIX1 | frameshift_inserti  on | exon6 | c.589dupT | p.S197fs | . | . |
| CTC5 | 5 | 109963560 | G | GT | 0.339 | NM_001039763 | TMEM232 | frameshift_inserti  on | exon6 | c.522dupA | p.R175fs | . | . |
| CTC5 | 5 | 110461453 | AT | A | 0.391 | NM_139281 | WDR36 | frameshift_deleti  on | exon22 | c.2667delT | p.D889fs | . | . |
| CTC5 | 5 | 110730425 | A | AT | 0.423 | NM_001744 | CAMK4 | frameshift_inserti  on | exon5 | c.405dupT | p.Y135fs | . | . |
| CTC5 | 5 | 111530273 | CT | C | 0.6 | NM_022140 | EPB41L4A | frameshift_deleti  on | exon17 | c.1493delA | p.K498fs | . | . |
| CTC5 | 5 | 114878975 | GC | G | 0.981 | NM_020177 | FEM1C | frameshift_deleti  on | exon2 | c.215delG | p.G72fs | . | . |
| CTC5 | 5 | 115387218 | GT | G | 0.317 | NM_001195581 | ARL14EPL | frameshift_deleti  on | exon1 | c.57delT | p.S19fs | . | . |
| CTC5 | 5 | 118469458 | CA | C | 0.5 | NM_005509 | DMXL1 | frameshift_deleti  on | exon12 | c.1840delA | p.S614fs | . | . |

| CTC5 | 5 | 118505985 | AT | A | 0.923 | NM_005509 | DMXL1 | frameshift_deleti  on | exon24 | c.5500delT | p.F1834fs | . | . |
| --- | --- | --- | --- | --- | --- | --- | --- | --- | --- | --- | --- | --- | --- |
| CTC5 | 5 | 118533486 | TA | T | 0.364 | NM_005509 | DMXL1 | frameshift_deleti  on | exon32 | c.7581delA | p.L2527fs | . | . |
| CTC5 | 5 | 129072819 | GA | G | 0.317 | NM_133638 | ADAMTS19 | frameshift_deleti  on | exon23 | c.3533delA | p.E1178fs | . | . |
| CTC5 | 5 | 131326657 | TG | T | 1 | NM_001009185 | ACSL6 | frameshift_deleti  on | exon3 | c.273delC | p.D91fs | . | . |
| CTC5 | 5 | 132010189 | TG | T | 0.267 | NM_000589 | IL4 | frameshift_deleti  on | exon2 | c.175delG | p.A59fs | . | . |
| CTC5 | 5 | 133695687 | TA | T | 0.439 | NM_001113575 | CDKL3 | stopgain | exon3 | c.260delT | p.L87X | . | . |
| CTC5 | 5 | 134366970 | ACG | A | 0.4 | NM_002653 | PITX1 | frameshift_deleti on | exon2 | c.396_397del | p.R132fs | . | . |
| CTC5 | 5 | 137089053 | CG | C | 0.993 | NM_006805 | HNRNPA0 | stopgain | exon1 | c.702delC | p.Y234X | . | . |
| CTC5 | 5 | 139745775 | AT | A | 0.308 | NM_001258427 | SLC4A9 | frameshift_deleti on | exon13 | c.1751delT | p.I584fs | . | . |
| CTC5 | 5 | 140039406 | GA | G | 0.333 | NM_006083 | IK | frameshift_deleti  on | exon14 | c.1262delA | p.E421fs | . | . |
| CTC5 | 5 | 140572679 | GTGA | G | 0.43 | NM_018930 | PCDHB10 | nonframeshift_de  letion | exon1 | c.555_557del | p.185_186del | . | . |
| CTC5 | 5 | 140588608 | CT | C | 0.303 | NM_018932 | PCDHB12 | frameshift_deleti  on | exon1 | c.130delT | p.F44fs | . | . |
| CTC5 | 5 | 140719125 | TG | T | 1 | NM_018915 | PCDHGA2 | frameshift_deleti  on | exon1 | c.588delG | p.L196fs | . | . |
| CTC5 | 5 | 140764779 | C | CG | 0.948 | NM_018920 | PCDHGA7 | frameshift_inserti  on | exon1 | c.2313_2314ins  G | p.P771fs | . | . |
| CTC5 | 5 | 140778451 | GA | G | 0.885 | NM_018925 | PCDHGB5 | frameshift_deleti  on | exon1 | c.758delA | p.E253fs | . | . |
| CTC5 | 5 | 141329078 | CT | C | 0.505 | NM_016580 | PCDH12 | frameshift_deleti  on | exon3 | c.3048delA | p.E1016fs | . | . |
| CTC5 | 5 | 141336090 | TG | T | 0.179 | NM_016580 | PCDH12 | frameshift_deleti  on | exon1 | c.1326delC | p.A442fs | . | . |
| CTC5 | 5 | 142680197 | ACAC | A | 0.4 | NM_000176 | NR3C1 | nonframeshift_de  letion | exon5 | c.1597_1599del | p.533_533del | . | . |
| CTC5 | 5 | 145894834 | CG | C | 0.864 | NM_194251 | GPR151 | frameshift_deleti  on | exon1 | c.842delC | p.P281fs | . | . |
| CTC5 | 5 | 147553878 | TG | T | 0.857 | NM_001001325 | SPINK14 | frameshift_deleti  on | exon3 | c.194delG | p.C65fs | . | . |
| CTC5 | 5 | 148743642 | CG | C | 0.455 | NM_024028 | PCYOX1L | frameshift_deleti  on | exon3 | c.340delG | p.G114fs | . | . |
| CTC5 | 5 | 149503909 | TG | T | 1 | NM_002609 | PDGFRB | frameshift_deleti  on | exon14 | c.1926delC | p.S642fs | . | . |
| CTC5 | 5 | 149619294 | TC | T | 0.213 | NM_015981 | CAMK2A | frameshift_deleti  on | exon14 | c.989delG | p.R330fs | . | . |
| CTC5 | 5 | 150632844 | GC | G | 0.875 | NM_000405 | GM2A | frameshift_deleti  on | exon1 | c.68delC | p.A23fs | . | . |
| CTC5 | 5 | 150947446 | GC | G | 0.667 | NM_001447 | FAT2 | frameshift_deleti  on | exon1 | c.1046delG | p.G349fs | . | . |
| CTC5 | 5 | 156589770 | G | GA | 0.274 | NM_130899 | FAM71B | frameshift_inserti  on | exon2 | c.1505_1506ins  T | p.H502fs | . | . |
| CTC5 | 5 | 157053489 | GT | G | 0.295 | NM_178424 | SOX30 | frameshift_deleti  on | exon5 | c.2120delA | p.N707fs | . | . |
| CTC5 | 5 | 167645297 | AC | A | 0.333 | NM_001122679 | TENM2 | stopgain | exon23 | c.4375delC | p.L1459X | . | . |
| CTC5 | 5 | 170235660 | AT | A | 0.94 | NM_014211 | GABRP | frameshift_deleti on | exon8 | c.737delT | p.I246fs | . | . |
| CTC5 | 5 | 173380109 | TA | T | 0.667 | NM_030627 | CPEB4 | frameshift_deleti  on | exon9 | c.1797delA | p.I599fs | . | . |
| CTC5 | 5 | 176662898 | AT | A | 0.6 | NM_022455 | NSD1 | frameshift_deleti  on | exon6 | c.3874delT | p.F1292fs | . | . |
| CTC5 | 5 | 176930171 | AGAG | A | 0.714 | NM_001144876 | DOK3 | nonframeshift_de  letion | exon5 | c.559_561del | p.187_187del | . | . |
| CTC5 | 5 | 180053119 | AC | A | 1 | NM_002020 | FLT4 | frameshift_deleti  on | exon9 | c.1249delG | p.V417fs | . | . |
| CTC5 | 5 | 180276001 | T | TC | 0.875 | NM_001172638 | ZFP62 | frameshift_inserti  on | exon2 | c.2493_2494ins  G | p.R832fs | . | . |
| CTC5 | 6 | 3226011 | GC | G | 0.379 | NM_178012 | TUBB2B | frameshift_deleti  on | exon4 | c.311delG | p.G104fs | . | . |
| CTC5 | 6 | 4995404 | CT | C | 0.688 | NM_001286132 | RPP40 | frameshift_deleti  on | exon7 | c.930delA | p.K310fs | . | . |
| CTC5 | 6 | 5771642 | GGT | G | 0.523 | NM_006567 | FARS2 | frameshift_deleti  on | exon7 | c.1337_1338del | p.G446fs | . | . |
| CTC5 | 6 | 11105511 | CG | C | 0.935 | NM_207582 | ERVFRD-1 | frameshift_deleti  on | exon2 | c.32delC | p.T11fs | . | . |
| CTC5 | 6 | 14135407 | AG | A | 0.333 | NM_001040280 | CD83 | frameshift_deleti  on | exon5 | c.556delG | p.V186fs | . | . |
| CTC5 | 6 | 15496695 | GA | G | 0.333 | NM_001267040 | JARID2 | frameshift_deleti  on | exon7 | c.724delA | p.N242fs | . | . |
| CTC5 | 6 | 15501263 | CA | C | 0.541 | NM_001267040 | JARID2 | frameshift_deleti  on | exon8 | c.1556delA | p.Q519fs | . | . |
| CTC5 | 6 | 16307054 | CA | C | 0.833 | NM_001128164 | ATXN1 | frameshift_deleti  on | exon8 | c.1953delT | p.F651fs | . | . |
| CTC5 | 6 | 18218075 | A | AT | 0.346 | NM_153042 | KDM1B | frameshift_inserti  on | exon17 | c.1649dupT | p.I550fs | . | . |
| CTC5 | 6 | 25529057 | GA | G | 0.333 | NM_001173977 | LRRC16A | frameshift_deleti  on | exon24 | c.2004delA | p.R668fs | . | . |
| CTC5 | 6 | 26027366 | CA | C | 0.29 | NM_003544 | HIST1H4B | frameshift_deleti  on | exon1 | c.114delT | p.L38fs | . | . |
| CTC5 | 6 | 26217397 | GCT | G | 0.19 | NM_021052 | HIST1H2AE | frameshift_deleti  on | exon1 | c.196_197del | p.L66fs | . | . |
| CTC5 | 6 | 26406268 | AG | A | 0.952 | NM_001145008 | BTN3A1 | frameshift_deleti  on | exon3 | c.218delG | p.R73fs | . | . |
| CTC5 | 6 | 27115170 | TC | T | 0.204 | NM_080596 | HIST1H2AH | frameshift_deleti  on | exon1 | c.264delC | p.I88fs | . | . |
| CTC5 | 6 | 27216883 | TC | T | 0.209 | NM_005865 | PRSS16 | frameshift_deleti  on | exon4 | c.343delC | p.P115fs | . | . |
| CTC5 | 6 | 27834858 | CT | C | 0.435 | NM_005322 | HIST1H1B | frameshift_deleti  on | exon1 | c.449delA | p.K150fs | . | . |
| CTC5 | 6 | 27858334 | G | GA | 0.636 | NM_003535 | HIST1H3J | frameshift_inserti  on | exon1 | c.236dupT | p.F79fs | . | . |
| CTC5 | 6 | 28269629 | GA | G | 1 | NM_001184743 | PGBD1 | frameshift_deleti  on | exon7 | c.1999delA | p.K667fs | . | . |
| CTC5 | 6 | 28540231 | GT | G | 0.889 | NM_052923 | SCAND3 | frameshift_deleti  on | exon4 | c.3434delA | p.N1145fs | . | . |
| CTC5 | 6 | 29408520 | AC | A | 0.333 | NM_013941 | OR10C1 | frameshift_deleti  on | exon1 | c.729delC | p.H243fs | . | . |
| CTC5 | 6 | 29627350 | CG | C | 0.182 | NM_001008228 | MOG | frameshift_deleti  on | exon2 | c.344delG | p.R115fs | . | . |
| CTC5 | 6 | 30558477 | G | GA | 1 | NM_001090 | ABCF1 | frameshift_inserti  on | exon24 | c.2424dupA | p.X808delinsX | . | . |
| CTC5 | 6 | 30627256 | GC | G | 0.938 | NM_001164239 | DHX16 | frameshift_deleti  on | exon12 | c.1819delG | p.A607fs | . | . |
| CTC5 | 6 | 30627543 | A | AG | 0.957 | NM_001164239 | DHX16 | frameshift_inserti  on | exon11 | c.1643dupC | p.P548fs | . | . |

| CTC5 | 6 | 30916939 | CA | C | 0.5 | NM_080870 | DPCR1 | frameshift_deleti  on | exon2 | c.699delA | p.S233fs | . | . |
| --- | --- | --- | --- | --- | --- | --- | --- | --- | --- | --- | --- | --- | --- |
| CTC5 | 6 | 31323944 | CAG | C | 0.519 | NM_005514 | HLA-B | frameshift_deleti  on | exon3 | c.617_618del | p.A206fs | . | . |
| CTC5 | 6 | 31692795 | C | CA | 0.133 | NM_138274 | C6orf25 | frameshift_inserti  on | exon4 | c.557_558insA | p.P186fs | . | . |
| CTC5 | 6 | 31696927 | CG | C | 0.917 | NM_013974 | DDAH2 | frameshift_deleti  on | exon2 | c.11delC | p.P4fs | . | . |
| CTC5 | 6 | 31832684 | AC | A | 0.417 | NM_001178044 | SLC44A4 | frameshift_deleti  on | exon18 | c.1708delG | p.V570fs | . | . |
| CTC5 | 6 | 32020462 | CG | C | 0.639 | NM_019105 | TNXB | frameshift_deleti  on | exon26 | c.9093delC | p.S3031fs | . | . |
| CTC5 | 6 | 32035626 | TG | T | 0.376 | NM_019105 | TNXB | frameshift_deleti  on | exon18 | c.6355delC | p.Q2119fs | . | . |
| CTC5 | 6 | 32083632 | GC | G | 0.6 | NM_001136153 | ATF6B | frameshift_deleti  on | exon18 | c.1986delG | p.S662fs | . | . |
| CTC5 | 6 | 32726658 | G | GC | 0.379 | NM_001198858 | HLA-DQB2 | frameshift_inserti  on | exon3 | c.614_615insG | p.P205fs | . | . |
| CTC5 | 6 | 33173662 | G | GA | 0.44 | NM_014234 | HSD17B8 | frameshift_inserti  on | exon6 | c.625dupA | p.Q208fs | . | . |
| CTC5 | 6 | 33631304 | TC | T | 0.273 | NM_002224 | ITPR3 | stopgain | exon10 | c.983delC | p.S328X | . | . |
| CTC5 | 6 | 34214512 | G | GA | 0.37 | NM_001287397 | C6orf1 | frameshift_inserti on | exon4 | c.198dupT | p.P67fs | . | . |
| CTC5 | 6 | 35209426 | AT | A | 0.407 | NM_152753 | SCUBE3 | frameshift_deleti  on | exon11 | c.1303delT | p.F435fs | . | . |
| CTC5 | 6 | 36181683 | AC | A | 0.385 | NM_015695 | BRPF3 | frameshift_deleti  on | exon8 | c.2510delC | p.T837fs | . | . |
| CTC5 | 6 | 36442625 | C | CT | 1 | NM_173562 | KCTD20 | frameshift_inserti  on | exon3 | c.221dupT | p.L74fs | . | . |
| CTC5 | 6 | 38773366 | TA | T | 0.636 | NM_001206927 | DNAH8 | frameshift_deleti  on | exon23 | c.3145delA | p.K1049fs | . | . |
| CTC5 | 6 | 38950203 | A | AT | 0.287 | NM_001206927 | DNAH8 | frameshift_inserti  on | exon86 | c.12917dupT | p.I4306fs | . | . |
| CTC5 | 6 | 41553185 | AG | A | 1 | NM_001012426 | FOXP4 | frameshift_deleti  on | exon5 | c.441delG | p.K147fs | . | . |
| CTC5 | 6 | 41566623 | TC | T | 0.429 | NM_001012426 | FOXP4 | frameshift_deleti  on | exon17 | c.1993delC | p.P665fs | . | . |
| CTC5 | 6 | 42890946 | A | AT | 0.6 | NM_001243168 | PTCRA | frameshift_inserti  on | exon2 | c.240_241insT | p.P80fs | . | . |
| CTC5 | 6 | 42975788 | AC | A | 0.902 | NM_180977 | PPP2R5D | frameshift_deleti  on | exon5 | c.525delC | p.N175fs | . | . |
| CTC5 | 6 | 43018020 | CA | C | 0.256 | NM_001168370 | CUL7 | frameshift_deleti  on | exon5 | c.1601delT | p.V534fs | . | . |
| CTC5 | 6 | 43181469 | AG | A | 0.423 | NM_015089 | CUL9 | frameshift_deleti  on | exon29 | c.5508delG | p.E1836fs | . | . |
| CTC5 | 6 | 43225637 | GC | G | 0.926 | NM_032538 | TTBK1 | frameshift_deleti  on | exon10 | c.950delC | p.A317fs | . | . |
| CTC5 | 6 | 43267365 | GT | G | 0.292 | NM_006672 | SLC22A7 | frameshift_deleti  on | exon3 | c.499delT | p.F167fs | . | . |
| CTC5 | 6 | 43276060 | TGCCC | T | 0.212 | NM_206922 | CRIP3 | frameshift_deleti  on | exon2 | c.127_130del | p.G43fs | . | . |
| CTC5 | 6 | 43308236 | T | TA | 0.272 | NM_014345 | ZNF318 | frameshift_inserti  on | exon10 | c.3499dupT | p.Y1167fs | . | . |
| CTC5 | 6 | 43418647 | TC | T | 0.6 | NM_001286655 | DLK2 | frameshift_deleti  on | exon6 | c.670delG | p.D224fs | . | . |
| CTC5 | 6 | 43491676 | GT | G | 0.765 | NM_020750 | XPO5 | frameshift_deleti  on | exon32 | c.3544delA | p.T1182fs | . | . |
| CTC5 | 6 | 43491704 | TA | T | 0.278 | NM_020750 | XPO5 | frameshift_deleti  on | exon32 | c.3516delT | p.I1172fs | . | . |
| CTC5 | 6 | 44200139 | CG | C | 0.713 | NM_001078174 | SLC29A1 | frameshift_deleti  on | exon11 | c.1034delG | p.R345fs | . | . |
| CTC5 | 6 | 46107699 | GA | G | 0.508 | NM_014936 | ENPP4 | frameshift_deleti  on | exon2 | c.380delA | p.E127fs | . | . |
| CTC5 | 6 | 46825937 | CA | C | 0.824 | NM_001098518 | GPR116 | frameshift_deleti  on | exon17 | c.3702delT | p.F1234fs | . | . |
| CTC5 | 6 | 47678560 | CA | C | 1 | NM_153838 | GPR115 | frameshift_deleti  on | exon4 | c.239delA | p.Q80fs | . | . |
| CTC5 | 6 | 47779420 | GT | G | 1 | NM_181744 | OPN5 | frameshift_deleti  on | exon6 | c.1010delT | p.V337fs | . | . |
| CTC5 | 6 | 47847579 | G | GA | 0.2 | NM_001013732 | PTCHD4 | frameshift_inserti  on | exon3 | c.1000dupT | p.S334fs | . | . |
| CTC5 | 6 | 49403268 | AGC | A | 1 | NM_000255 | MUT | frameshift_deleti  on | exon12 | c.2023_2024del | p.A675fs | . | . |
| CTC5 | 6 | 49754461 | AT | A | 0.556 | NM_138733 | PGK2 | stopgain | exon1 | c.439delA | p.I147X | . | . |
| CTC5 | 6 | 50696625 | TC | T | 0.756 | NM_172238 | TFAP2D | frameshift_deleti on | exon4 | c.656delC | p.S219fs | . | . |
| CTC5 | 6 | 52285223 | CG | C | 0.864 | NM_018100 | EFHC1 | frameshift_deleti  on | exon1 | c.16delG | p.V6fs | . | . |
| CTC5 | 6 | 54254612 | GT | G | 0.571 | NM_014464 | TINAG | frameshift_deleti  on | exon11 | c.1321delT | p.S441fs | . | . |
| CTC5 | 6 | 56347663 | CT | C | 0.786 | NM_015548 | DST | frameshift_deleti  on | exon70 | c.13350delA | p.K4450fs | . | . |
| CTC5 | 6 | 56357745 | AT | A | 0.343 | NM_015548 | DST | frameshift_deleti  on | exon65 | c.12667delA | p.I4223fs | . | . |
| CTC5 | 6 | 56362723 | AT | A | 0.798 | NM_015548 | DST | frameshift_deleti  on | exon62 | c.12155delA | p.N4052fs | . | . |
| CTC5 | 6 | 56484083 | A | AT | 0.167 | NM_001723 | DST | frameshift_inserti  on | exon23 | c.4748dupA | p.N1583fs | . | . |
| CTC5 | 6 | 56492031 | AT | A | 0.575 | NM_001723 | DST | frameshift_deleti  on | exon20 | c.3081delA | p.K1027fs | . | . |
| CTC5 | 6 | 64413548 | CA | C | 0.329 | NM_015153 | PHF3 | frameshift_deleti  on | exon10 | c.3355delA | p.K1119fs | . | . |
| CTC5 | 6 | 64431052 | AT | A | 0.407 | NM_001142800 | EYS | frameshift_deleti  on | exon43 | c.8874delA | p.K2958fs | . | . |
| CTC5 | 6 | 64431527 | AT | A | 0.667 | NM_001142800 | EYS | frameshift_deleti  on | exon43 | c.8399delA | p.D2800fs | . | . |
| CTC5 | 6 | 64487976 | GC | G | 0.86 | NM_001142800 | EYS | frameshift_deleti  on | exon40 | c.7820delG | p.G2607fs | . | . |
| CTC5 | 6 | 70646736 | TA | T | 0.979 | NM_001858 | COL19A1 | frameshift_deleti  on | exon8 | c.808delA | p.K270fs | . | . |
| CTC5 | 6 | 71011733 | CA | C | 0.414 | NM_001851 | COL9A1 | frameshift_deleti  on | exon2 | c.58delT | p.W20fs | . | . |
| CTC5 | 6 | 74498226 | CT | C | 0.5 | NM_001159588 | CD109 | frameshift_deleti  on | exon21 | c.2362delT | p.L788fs | . | . |
| CTC5 | 6 | 75833704 | TG | T | 0.533 | NM_080645 | COL12A1 | frameshift_deleti  on | exon27 | c.3338delC | p.P1113fs | . | . |
| CTC5 | 6 | 79700634 | CT | C | 0.857 | NM_017934 | PHIP | frameshift_deleti  on | exon20 | c.2269delA | p.R757fs | . | . |
| CTC5 | 6 | 84871600 | TA | T | 0.408 | NM_001286206 | KIAA1009 | stopgain | exon20 | c.2312delT | p.L771X | . | . |
| CTC5 | 6 | 85448293 | A | AT | 0.25 | NM_001080508 | TBX18 | frameshift_inserti on | exon7 | c.1020_1021ins A | p.L341fs | . | . |
| CTC5 | 6 | 86332262 | TC | T | 0.857 | NM_001159673 | SYNCRIP | frameshift_deleti  on | exon7 | c.651delG | p.G217fs | . | . |

| CTC5 | 6 | 87795513 | CG | C | 0.625 | NM_000735 | CGA | frameshift_deleti  on | exon4 | c.311delC | p.T104fs | . | . |
| --- | --- | --- | --- | --- | --- | --- | --- | --- | --- | --- | --- | --- | --- |
| CTC5 | 6 | 87968935 | TA | T | 0.32 | NM_015021 | ZNF292 | frameshift_deleti  on | exon8 | c.5589delA | p.I1863fs | . | . |
| CTC5 | 6 | 89650819 | TG | T | 0.253 | NM_001286426 | RNGTT | frameshift_deleti  on | exon2 | c.144delC | p.S48fs | . | . |
| CTC5 | 6 | 90450057 | CT | C | 0.882 | NM_014611 | MDN1 | frameshift_deleti  on | exon32 | c.4488delA | p.K1496fs | . | . |
| CTC5 | 6 | 90604622 | T | TA | 1 | NM_032602 | GJA10 | frameshift_inserti  on | exon1 | c.436dupA | p.H145fs | . | . |
| CTC5 | 6 | 99729131 | GA | G | 0.333 | NM_032511 | FAXC | frameshift_deleti  on | exon6 | c.1138delT | p.S380fs | . | . |
| CTC5 | 6 | 99849077 | CT | C | 0.273 | NM_015491 | PNISR | frameshift_deleti  on | exon11 | c.1756delA | p.R586fs | . | . |
| CTC5 | 6 | 100841614 | GA | G | 0.357 | NM_005068 | SIM1 | frameshift_deleti  on | exon10 | c.1318delT | p.S440fs | . | . |
| CTC5 | 6 | 106968998 | CG | C | 0.625 | NM_001624 | AIM1 | frameshift_deleti  on | exon2 | c.2692delG | p.D898fs | . | . |
| CTC5 | 6 | 108230172 | T | TA | 0.5 | NM_007214 | SEC63 | frameshift_inserti  on | exon8 | c.691dupT | p.Y231fs | . | . |
| CTC5 | 6 | 109714082 | CA | C | 0.629 | NM_001286360 | PPIL6 | frameshift_deleti  on | exon7 | c.786delT | p.N262fs | . | . |
| CTC5 | 6 | 110086340 | TA | T | 0.243 | NM_014845 | FIG4 | frameshift_deleti  on | exon14 | c.1560delA | p.L520fs | . | . |
| CTC5 | 6 | 112407770 | A | AT | 0.75 | NM_016262 | TUBE1 | frameshift_inserti  on | exon3 | c.140dupA | p.N47fs | . | . |
| CTC5 | 6 | 116441790 | GA | G | 0.286 | NM_000493 | COL10A1 | frameshift_deleti  on | exon3 | c.1488delT | p.G496fs | . | . |
| CTC5 | 6 | 116600465 | T | TCAC | 1 | NM_003309 | TSPYL1 | nonframeshift_in  sertion | exon1 | c.528_529insGT  G | p.K177delinsVK | . | . |
| CTC5 | 6 | 116600470 | A | ACCC | 1 | NM_003309 | TSPYL1 | nonframeshift_in  sertion | exon1 | c.523_524insG  GG | p.V175delinsG  V | . | . |
| CTC5 | 6 | 116966933 | GT | G | 0.528 | NM_145062 | ZUFSP | frameshift_deleti  on | exon9 | c.1632delA | p.K544fs | . | . |
| CTC5 | 6 | 117043292 | TC | T | 0.923 | NM_002269 | KPNA5 | frameshift_deleti  on | exon9 | c.761delC | p.S254fs | . | . |
| CTC5 | 6 | 117900103 | GT | G | 1 | NM_001017408 | GOPC | frameshift_deleti  on | exon2 | c.409delA | p.T137fs | . | . |
| CTC5 | 6 | 129802562 | GA | G | 0.286 | NM_001079823 | LAMA2 | frameshift_deleti  on | exon54 | c.7716delA | p.R2572fs | . | . |
| CTC5 | 6 | 129905244 | TGAG | T | 0.222 | NM_033515 | ARHGAP18 | nonframeshift_de  letion | exon13 | c.1724_1726del | p.575_576del | . | . |
| CTC5 | 6 | 132874279 | AT | A | 0.184 | NM_053278 | TAAR8 | frameshift_deleti  on | exon1 | c.449delT | p.I150fs | . | . |
| CTC5 | 6 | 133014320 | CA | C | 0.941 | NM_004666 | VNN1 | frameshift_deleti  on | exon4 | c.668delT | p.L223fs | . | . |
| CTC5 | 6 | 133092220 | AC | A | 1 | NM_052831 | SLC18B1 | frameshift_deleti  on | exon12 | c.1208delG | p.G403fs | . | . |
| CTC5 | 6 | 134303751 | TG | T | 0.233 | NM_001253676 | TBPL1 | frameshift_deleti  on | exon3 | c.182delG | p.W61fs | . | . |
| CTC5 | 6 | 134349812 | TG | T | 0.5 | NM_145176 | SLC2A12 | frameshift_deleti  on | exon2 | c.1150delC | p.Q384fs | . | . |
| CTC5 | 6 | 135539098 | GCC | G | 0.411 | NM_001161657 | MYB | frameshift_deleti  on | exon14 | c.1649_1650del | p.A550fs | . | . |
| CTC5 | 6 | 136565940 | AT | A | 0.289 | NM_001099286 | MTFR2 | frameshift_deleti  on | exon3 | c.130delA | p.M44fs | . | . |
| CTC5 | 6 | 136990424 | C | CT | 0.308 | NM_005923 | MAP3K5 | frameshift_inserti  on | exon8 | c.1362dupA | p.V455fs | . | . |
| CTC5 | 6 | 138199960 | GC | G | 0.37 | NM_001270507 | TNFAIP3 | frameshift_deleti  on | exon7 | c.1379delC | p.A460fs | . | . |
| CTC5 | 6 | 138655504 | CT | C | 0.883 | NM_020340 | KIAA1244 | frameshift_deleti  on | exon33 | c.5522delT | p.L1841fs | . | . |
| CTC5 | 6 | 138655790 | CG | C | 0.158 | NM_020340 | KIAA1244 | frameshift_deleti  on | exon33 | c.5808delG | p.P1936fs | . | . |
| CTC5 | 6 | 138752824 | TG | T | 0.222 | NM_020464 | NHSL1 | frameshift_deleti  on | exon5 | c.2669delC | p.P890fs | . | . |
| CTC5 | 6 | 155619624 | TC | T | 0.222 | NM_016020 | TFB1M | frameshift_deleti  on | exon3 | c.380delG | p.R127fs | . | . |
| CTC5 | 6 | 158349648 | CT | C | 0.25 | NM_016224 | SNX9 | frameshift_deleti  on | exon12 | c.1203delT | p.A401fs | . | . |
| CTC5 | 6 | 160328903 | G | GA | 0.958 | NM_002377 | MAS1 | frameshift_inserti  on | exon1 | c.917dupA | p.E306fs | . | . |
| CTC5 | 6 | 160560897 | CTGGTA  AGT | C | 0.763 | NM_003057 | SLC22A1 | frameshift_deleti  on | exon7 | c.1275_1276del | p.P425fs | . | . |
| CTC5 | 6 | 167753655 | TC | T | 0.5 | NM_031949 | TTLL2 | frameshift_deleti  on | exon3 | c.268delC | p.R90fs | . | . |
| CTC5 | 6 | 168303014 | CT | C | 0.375 | NM_001207008 | MLLT4 | frameshift_deleti  on | exon11 | c.1551delT | p.T517fs | . | . |
| CTC5 | 6 | 168315857 | TG | T | 0.75 | NM_001207008 | MLLT4 | frameshift_deleti  on | exon17 | c.2241delG | p.L747fs | . | . |
| CTC5 | 6 | 168708971 | AT | A | 0.75 | NM_001286350 | DACT2 | frameshift_deleti  on | exon3 | c.955delA | p.M319fs | . | . |
| CTC5 | 6 | 170871013 | A | ACAG | 0.887 | NM_001172085 | TBP | nonframeshift_in  sertion | exon2 | c.129_130insCA  G | p.Q43delinsQQ | . | . |
| CTC5 | 6 | 170871037 | GCAA | G | 0.83 | NM_001172085 | TBP | nonframeshift_de  letion | exon2 | c.154_156del | p.52_52del | . | . |
| CTC5 | 6 | 170871046 | ACAGCA  GCAG | A | 1 | NM_001172085 | TBP | nonframeshift_de  letion | exon2 | c.163_171del | p.55_57del | . | . |
| CTC5 | 6 | 170871046 | ACAGCA  GCAGCA | A | 1 | NM_001172085 | TBP | nonframeshift_de  letion | exon2 | c.163_174del | p.55_58del | . | . |
| CTC5 | 7 | 299936 | CG | C | 0.575 | NM_020223 | FAM20C | frameshift_deleti  on | exon10 | c.1746delG | p.S582fs | . | . |
| CTC5 | 7 | 1595020 | TG | T | 0.806 | NM_001097620 | TMEM184A | frameshift_deleti  on | exon2 | c.100delC | p.Q34fs | . | . |
| CTC5 | 7 | 2953082 | TC | T | 0.667 | NM_032415 | CARD11 | frameshift_deleti  on | exon22 | c.2857delG | p.E953fs | . | . |
| CTC5 | 7 | 2963940 | GGGA | G | 0.667 | NM_032415 | CARD11 | nonframeshift_de  letion | exon15 | c.1864_1866del | p.622_622del | . | . |
| CTC5 | 7 | 5103631 | CA | C | 0.394 | NM_021163 | RBAK | frameshift_deleti  on | exon5 | c.545delA | p.Q182fs | . | . |
| CTC5 | 7 | 5104274 | CG | C | 0.318 | NM_021163 | RBAK | frameshift_deleti  on | exon5 | c.1188delG | p.T396fs | . | . |
| CTC5 | 7 | 5781179 | T | TG | 0.389 | NM_207111 | RNF216 | frameshift_inserti  on | exon4 | c.468dupC | p.K157fs | . | . |
| CTC5 | 7 | 8261009 | GT | G | 1 | NM_001136020 | ICA1 | frameshift_deleti  on | exon5 | c.275delA | p.N92fs | . | . |
| CTC5 | 7 | 12391268 | T | TA | 0.875 | NM_001135924 | VWDE | stopgain | exon19 | c.3816dupT | p.K1273_E1274  delinsX | . | . |
| CTC5 | 7 | 15725797 | ATGG | A | 1 | NM_005924 | MEOX2 | nonframeshift_de  letion | exon1 | c.228_230del | p.76_77del | . | . |
| CTC5 | 7 | 16644347 | AT | A | 0.372 | NM_020319 | ANKMY2 | frameshift_deleti  on | exon8 | c.1009delA | p.M337fs | . | . |
| CTC5 | 7 | 21603991 | GT | G | 0.342 | NM_001277115 | DNAH11 | frameshift_deleti  on | exon6 | c.1171delT | p.F391fs | . | . |

| CTC5 | 7 | 21781578 | TC | T | 0.6 | NM_001277115 | DNAH11 | frameshift_deleti  on | exon49 | c.7949delC | p.S2650fs | . | . |
| --- | --- | --- | --- | --- | --- | --- | --- | --- | --- | --- | --- | --- | --- |
| CTC5 | 7 | 23306109 | TA | T | 0.778 | NM_001005340 | GPNMB | frameshift_deleti  on | exon7 | c.1029delA | p.L343fs | . | . |
| CTC5 | 7 | 23854810 | C | CA | 1 | NM_001260504 | STK31 | frameshift_inserti  on | exon23 | c.2740dupA | p.P913fs | . | . |
| CTC5 | 7 | 25207951 | TG | T | 0.667 | NM_138811 | C7orf31 | frameshift_deleti  on | exon3 | c.267delC | p.G89fs | . | . |
| CTC5 | 7 | 27148304 | GC | G | 0.55 | NM_030661 | HOXA3 | frameshift_deleti  on | exon3 | c.561delG | p.G187fs | . | . |
| CTC5 | 7 | 27203453 | TG | T | 0.537 | NM_152739 | HOXA9 | frameshift_deleti  on | exon2 | c.587delC | p.P196fs | . | . |
| CTC5 | 7 | 30084792 | AC | A | 0.5 | NM_001197026 | PLEKHA8 | frameshift_deleti  on | exon2 | c.133delC | p.Q45fs | . | . |
| CTC5 | 7 | 30915160 | CG | C | 0.255 | NM_032222 | FAM188B | frameshift_deleti  on | exon15 | c.1861delG | p.D621fs | . | . |
| CTC5 | 7 | 31877528 | GA | G | 0.5 | NM_001191056 | PDE1C | frameshift_deleti  on | exon10 | c.1037delT | p.F346fs | . | . |
| CTC5 | 7 | 32209472 | CG | C | 0.389 | NM_001191058 | PDE1C | frameshift_deleti  on | exon3 | c.232delC | p.R78fs | . | . |
| CTC5 | 7 | 36438918 | GA | G | 1 | NM_001284301 | ANLN | frameshift_deleti  on | exon3 | c.404delA | p.E135fs | . | . |
| CTC5 | 7 | 37934150 | GA | G | 0.5 | NM_016616 | NME8 | frameshift_deleti  on | exon16 | c.1483delA | p.K495fs | . | . |
| CTC5 | 7 | 38501002 | CT | C | 0.353 | NM_001635 | AMPH | frameshift_deleti  on | exon11 | c.897delA | p.K299fs | . | . |
| CTC5 | 7 | 38505815 | GT | G | 0.541 | NM_001635 | AMPH | frameshift_deleti  on | exon8 | c.623delA | p.N208fs | . | . |
| CTC5 | 7 | 40133827 | G | GT | 0.3 | NM_003718 | CDK13 | frameshift_inserti  on | exon14 | c.3787_3788ins  T | p.E1263fs | . | . |
| CTC5 | 7 | 43546810 | AG | A | 0.417 | NM_001287059 | HECW1 | frameshift_deleti  on | exon20 | c.3605delG | p.R1202fs | . | . |
| CTC5 | 7 | 43581586 | GT | G | 0.895 | NM_001287059 | HECW1 | frameshift_deleti  on | exon24 | c.4136delT | p.V1379fs | . | . |
| CTC5 | 7 | 43684854 | GC | G | 0.767 | NM_018224 | COA1 | frameshift_deleti  on | exon4 | c.259delG | p.A87fs | . | . |
| CTC5 | 7 | 44042193 | TG | T | 0.818 | NM_175064 | SPDYE1 | frameshift_deleti  on | exon2 | c.265delG | p.G89fs | . | . |
| CTC5 | 7 | 44573423 | TC | T | 1 | NM_001101648 | NPC1L1 | frameshift_deleti  on | exon7 | c.2195delG | p.R732fs | . | . |
| CTC5 | 7 | 48494724 | CG | C | 0.833 | NM_152701 | ABCA13 | frameshift_deleti  on | exon43 | c.12657delG | p.T4219fs | . | . |
| CTC5 | 7 | 50595918 | AG | A | 0.473 | NM_001242888 | DDC | frameshift_deleti  on | exon4 | c.396delC | p.P132fs | . | . |
| CTC5 | 7 | 51095730 | T | TG | 0.265 | NM_015198 | COBL | frameshift_inserti  on | exon10 | c.3062dupC | p.P1021fs | . | . |
| CTC5 | 7 | 56141891 | GA | G | 0.461 | NM_001146333 | SUMF2 | frameshift_deleti  on | exon3 | c.101delA | p.E34fs | . | . |
| CTC5 | 7 | 65419353 | TTTA | T | 0.333 | NM_001284342 | VKORC1L1 | nonframeshift_de  letion | exon2 | c.488_490del | p.163_164del | . | . |
| CTC5 | 7 | 70254787 | CG | C | 1 | NM_001127231 | AUTS2 | frameshift_deleti  on | exon18 | c.2514delG | p.P838fs | . | . |
| CTC5 | 7 | 70255576 | GCCA | G | 0.3 | NM_001127231 | AUTS2 | nonframeshift_de  letion | exon18 | c.3303_3305del | p.1101_1102de  l | . | . |
| CTC5 | 7 | 72745773 | TA | T | 0.281 | NM_001281304 | FKBP6 | frameshift_deleti  on | exon4 | c.493delA | p.K165fs | . | . |
| CTC5 | 7 | 72985117 | TG | T | 0.197 | NM_012453 | TBL2 | frameshift_deleti  on | exon7 | c.1063delC | p.H355fs | . | . |
| CTC5 | 7 | 76871159 | CA | C | 0.385 | NM_020879 | CCDC146 | frameshift_deleti  on | exon4 | c.392delA | p.Q131fs | . | . |
| CTC5 | 7 | 76941513 | CA | C | 0.308 | NM_017439 | GSAP | frameshift_deleti  on | exon29 | c.2277delT | p.I759fs | . | . |
| CTC5 | 7 | 78636470 | CT | C | 0.529 | NM_012301 | MAGI2 | frameshift_deleti  on | exon2 | c.353delA | p.K118fs | . | . |
| CTC5 | 7 | 81593615 | CT | C | 0.747 | NM_000722 | CACNA2D1 | frameshift_deleti  on | exon33 | c.2634delA | p.S878fs | . | . |
| CTC5 | 7 | 81593660 | CA | C | 0.889 | NM_000722 | CACNA2D1 | frameshift_deleti  on | exon33 | c.2589delT | p.F863fs | . | . |
| CTC5 | 7 | 82785374 | CT | C | 0.783 | NM_014510 | PCLO | frameshift_deleti  on | exon2 | c.582delA | p.K194fs | . | . |
| CTC5 | 7 | 83643640 | ATGAGG | A | 0.811 | NM_006080 | SEMA3A | frameshift_deleti  on | exon7 | c.690_694del | p.H230fs | . | . |
| CTC5 | 7 | 87179239 | AT | A | 0.5 | NM_000927 | ABCB1 | frameshift_deleti  on | exon14 | c.1481delA | p.N494fs | . | . |
| CTC5 | 7 | 88965297 | AC | A | 0.944 | NM_181646 | ZNF804B | frameshift_deleti  on | exon4 | c.3002delC | p.T1001fs | . | . |
| CTC5 | 7 | 90007454 | A | AT | 0.529 | NM_001042717 | GTPBP10 | frameshift_inserti  on | exon6 | c.470dupT | p.I157fs | . | . |
| CTC5 | 7 | 92017174 | TA | T | 0.97 | NM_019004 | ANKIB1 | frameshift_deleti  on | exon13 | c.1816delA | p.K606fs | . | . |
| CTC5 | 7 | 92763695 | TA | T | 0.875 | NM_152703 | SAMD9L | frameshift_deleti  on | exon5 | c.1589delT | p.L530fs | . | . |
| CTC5 | 7 | 92764915 | T | TCG | 0.163 | NM_152703 | SAMD9L | frameshift_inserti  on | exon5 | c.369_370insC  G | p.R124fs | . | . |
| CTC5 | 7 | 92848564 | AT | A | 0.896 | NM_001288810 | HEPACAM2 | frameshift_deleti  on | exon1 | c.243delA | p.E81fs | . | . |
| CTC5 | 7 | 92848612 | TG | T | 0.791 | NM_001288810 | HEPACAM2 | frameshift_deleti  on | exon1 | c.195delC | p.P65fs | . | . |
| CTC5 | 7 | 98576402 | G | GA | 0.195 | NM_003496 | TRRAP | frameshift_inserti  on | exon56 | c.8435dupA | p.E2812fs | . | . |
| CTC5 | 7 | 98581906 | C | CT | 0.317 | NM_003496 | TRRAP | frameshift_inserti  on | exon59 | c.9139dupT | p.C3046fs | . | . |
| CTC5 | 7 | 98609965 | AC | A | 1 | NM_003496 | TRRAP | frameshift_deleti  on | exon71 | c.11481delC | p.H3827fs | . | . |
| CTC5 | 7 | 99631578 | AC | A | 0.918 | NM_001287055 | ZKSCAN1 | frameshift_deleti  on | exon4 | c.812delC | p.T271fs | . | . |
| CTC5 | 7 | 99669116 | GAT | G | 0.263 | NM_001278291 | ZNF3 | frameshift_deleti  on | exon4 | c.881_882del | p.H294fs | . | . |
| CTC5 | 7 | 99710477 | TCCTGG | T | 0.4 | NM_001190415 | TAF6 | frameshift_deleti  on | exon6 | c.624_628del | p.G208fs | . | . |
| CTC5 | 7 | 99757606 | TG | T | 0.6 | NM_024637 | GAL3ST4 | frameshift_deleti  on | exon4 | c.1405delC | p.Q469fs | . | . |
| CTC5 | 7 | 100061014 | TG | T | 0.3 | NM_001004323 | C7orf61 | frameshift_deleti  on | exon2 | c.358delC | p.Q120fs | . | . |
| CTC5 | 7 | 100459137 | TG | T | 0.41 | NM_001267814 | SLC12A9 | frameshift_deleti  on | exon9 | c.1201delG | p.A401fs | . | . |
| CTC5 | 7 | 100482410 | AT | A | 0.3 | NM_001128852 | SRRT | frameshift_deleti  on | exon8 | c.993delT | p.D331fs | . | . |
| CTC5 | 7 | 100685045 | AC | A | 0.38 | NM_001040105 | MUC17 | frameshift_deleti  on | exon3 | c.10349delC | p.T3450fs | . | . |
| CTC5 | 7 | 101840288 | GC | G | 1 | NM_001202543 | CUX1 | frameshift_deleti  on | exon15 | c.1631delC | p.A544fs | . | . |
| CTC5 | 7 | 103244910 | CG | C | 0.24 | NM_005045 | RELN | frameshift_deleti  on | exon23 | c.3028delC | p.R1010fs | . | . |
| CTC5 | 7 | 104702682 | GC | G | 0.942 | NM_018682 | KMT2E | frameshift_deleti  on | exon3 | c.144delC | p.S48fs | . | . |

| CTC5 | 7 | 105283325 | AT | A | 0.471 | NM_138495 | ATXN7L1 | frameshift_deleti  on | exon3 | c.449delA | p.N150fs | . | . |
| --- | --- | --- | --- | --- | --- | --- | --- | --- | --- | --- | --- | --- | --- |
| CTC5 | 7 | 105739679 | GT | G | 0.6 | NM_182715 | SYPL1 | frameshift_deleti  on | exon2 | c.118delA | p.T40fs | . | . |
| CTC5 | 7 | 107336479 | TC | T | 0.318 | NM_000441 | SLC26A4 | frameshift_deleti  on | exon13 | c.1540delC | p.Q514fs | . | . |
| CTC5 | 7 | 107571902 | GC | G | 0.5 | NM_002291 | LAMB1 | frameshift_deleti  on | exon29 | c.4435delG | p.A1479fs | . | . |
| CTC5 | 7 | 107591746 | TGCACT  G | T | 0.182 | NM_002291 | LAMB1 | nonframeshift_de  letion | exon24 | c.3310_3315del | p.1104_1105de  l | . | . |
| CTC5 | 7 | 107600958 | GT | G | 0.963 | NM_002291 | LAMB1 | frameshift_deleti  on | exon18 | c.2245delA | p.T749fs | . | . |
| CTC5 | 7 | 107671285 | TC | T | 0.466 | NM_007356 | LAMB4 | frameshift_deleti  on | exon32 | c.4957delG | p.E1653fs | . | . |
| CTC5 | 7 | 107790525 | C | CT | 0.507 | NM_005010 | NRCAM | frameshift_inserti  on | exon28 | c.3381dupA | p.E1128fs | . | . |
| CTC5 | 7 | 108128352 | CTT | C | 0.4 | NM_001256008 | PNPLA8 | frameshift_deleti  on | exon8 | c.1727_1728del | p.K576fs | . | . |
| CTC5 | 7 | 111368690 | GT | G | 0.602 | NM_014705 | DOCK4 | frameshift_deleti  on | exon52 | c.5540delA | p.Y1847fs | . | . |
| CTC5 | 7 | 116418887 | AT | A | 1 | NM_000245 | MET | frameshift_deleti  on | exon17 | c.3399delT | p.D1133fs | . | . |
| CTC5 | 7 | 117022162 | TA | T | 1 | NM_130768 | ASZ1 | frameshift_deleti  on | exon8 | c.848delT | p.L283fs | . | . |
| CTC5 | 7 | 117062322 | CA | C | 0.24 | NM_130768 | ASZ1 | frameshift_deleti  on | exon3 | c.296delT | p.L99fs | . | . |
| CTC5 | 7 | 119915592 | G | GT | 1 | NM_012281 | KCND2 | frameshift_inserti  on | exon1 | c.907dupT | p.K302fs | . | . |
| CTC5 | 7 | 121636444 | TC | T | 0.783 | NM_001206838 | PTPRZ1 | stopgain | exon9 | c.938delC | p.S313X | . | . |
| CTC5 | 7 | 122342654 | CA | C | 0.5 | NM_198085 | RNF148 | frameshift_deleti on | exon1 | c.150delT | p.N50fs | . | . |
| CTC5 | 7 | 123303171 | AG | A | 0.179 | NM_207163 | LMOD2 | frameshift_deleti  on | exon2 | c.1532delG | p.S511fs | . | . |
| CTC5 | 7 | 123508594 | AT | A | 0.694 | NM_012269 | HYAL4 | frameshift_deleti  on | exon3 | c.268delT | p.F90fs | . | . |
| CTC5 | 7 | 123593962 | G | GA | 0.571 | NM_001174044 | SPAM1 | frameshift_inserti  on | exon3 | c.339dupA | p.G113fs | . | . |
| CTC5 | 7 | 127342576 | TC | T | 0.152 | NM_014390 | SND1 | frameshift_deleti  on | exon6 | c.678delC | p.I226fs | . | . |
| CTC5 | 7 | 127670051 | TGA | T | 1 | NM_022143 | LRRC4 | frameshift_deleti  on | exon2 | c.641_642del | p.L214fs | . | . |
| CTC5 | 7 | 127894622 | C | CT | 0.25 | NM_000230 | LEP | frameshift_inserti  on | exon3 | c.311dupT | p.L104fs | . | . |
| CTC5 | 7 | 127964742 | AC | A | 0.3 | NM_001166135 | RBM28 | frameshift_deleti  on | exon8 | c.785delG | p.G262fs | . | . |
| CTC5 | 7 | 127979368 | TG | T | 0.8 | NM_018077 | RBM28 | stopgain | exon3 | c.284delC | p.S95X | . | . |
| CTC5 | 7 | 128494512 | CG | C | 1 | NM_001127487 | FLNC | frameshift_deleti on | exon40 | c.6675delG | p.S2225fs | . | . |
| CTC5 | 7 | 128587351 | CACTCT  GCAGCC GCCCAC TCTGCG | C | 0.714 | NM_001098627 | IRF5 | nonframeshift_de letion | exon6 | c.502_531del | p.168_177del | . | . |
| CTC5 | 7 | 128626839 | GC | G | 1 | NM_012470 | TNPO3 | frameshift_deleti  on | exon12 | c.1633delG | p.A545fs | . | . |
| CTC5 | 7 | 134261755 | TC | T | 0.345 | NM_001080538 | AKR1B15 | frameshift_deleti  on | exon10 | c.867delC | p.I289fs | . | . |
| CTC5 | 7 | 134262522 | GC | G | 0.615 | NM_001080538 | AKR1B15 | frameshift_deleti  on | exon11 | c.977delC | p.A326fs | . | . |
| CTC5 | 7 | 135418855 | CA | C | 0.649 | NM_205855 | FAM180A | frameshift_deleti  on | exon3 | c.389delT | p.L130fs | . | . |
| CTC5 | 7 | 137597748 | GA | G | 1 | NM_001253775 | CREB3L2 | frameshift_deleti  on | exon4 | c.571delT | p.S191fs | . | . |
| CTC5 | 7 | 137612913 | CTGG | C | 1 | NM_001253775 | CREB3L2 | nonframeshift_de  letion | exon2 | c.299_301del | p.100_101del | . | . |
| CTC5 | 7 | 137613084 | GA | G | 0.283 | NM_001253775 | CREB3L2 | frameshift_deleti  on | exon2 | c.130delT | p.S44fs | . | . |
| CTC5 | 7 | 137792261 | AT | A | 0.261 | NM_001190906 | AKR1D1 | frameshift_deleti  on | exon6 | c.668delT | p.I223fs | . | . |
| CTC5 | 7 | 138269562 | GA | G | 0.5 | NM_003852 | TRIM24 | frameshift_deleti  on | exon19 | c.2918delA | p.E973fs | . | . |
| CTC5 | 7 | 138601825 | CG | C | 0.893 | NM_001164665 | KIAA1549 | frameshift_deleti  on | exon2 | c.2546delC | p.S849fs | . | . |
| CTC5 | 7 | 138602291 | G | GA | 0.781 | NM_001164665 | KIAA1549 | frameshift_inserti  on | exon2 | c.2080dupT | p.S694fs | . | . |
| CTC5 | 7 | 138732538 | GT | G | 0.748 | NM_020119 | ZC3HAV1 | frameshift_deleti  on | exon13 | c.2510delA | p.N837fs | . | . |
| CTC5 | 7 | 141301006 | A | AC | 0.889 | NM_018238 | AGK | frameshift_inserti  on | exon5 | c.223_224insC | p.K75fs | . | . |
| CTC5 | 7 | 141373932 | CT | C | 0.973 | NM_001080392 | KIAA1147 | frameshift_deleti  on | exon4 | c.615delA | p.R205fs | . | . |
| CTC5 | 7 | 142626646 | GC | G | 1 | NM_019841 | TRPV5 | frameshift_deleti  on | exon4 | c.363delG | p.L121fs | . | . |
| CTC5 | 7 | 142997513 | TG | T | 0.76 | NM_001224 | CASP2 | frameshift_deleti  on | exon9 | c.916delG | p.A306fs | . | . |
| CTC5 | 7 | 148523694 | TG | T | 1 | NM_152998 | EZH2 | frameshift_deleti  on | exon7 | c.641delC | p.P214fs | . | . |
| CTC5 | 7 | 149467597 | TC | T | 0.255 | NM_207336 | ZNF467 | frameshift_deleti  on | exon3 | c.82delG | p.E28fs | . | . |
| CTC5 | 7 | 149475093 | AG | A | 0.237 | NM_198455 | SSPO | frameshift_deleti  on | exon6 | c.795delG | p.K265fs | . | . |
| CTC5 | 7 | 149476670 | C | CA | 0.455 | NM_198455 | SSPO | frameshift_inserti  on | exon10 | c.1120_1121ins  A | p.L374fs | . | . |
| CTC5 | 7 | 150270119 | A | AT | 0.545 | NM_018326 | GIMAP4 | frameshift_inserti  on | exon3 | c.962dupT | p.I321fs | . | . |
| CTC5 | 7 | 150553943 | TC | T | 0.522 | NM_001091 | AOC1 | frameshift_deleti  on | exon2 | c.386delC | p.S129fs | . | . |
| CTC5 | 7 | 150656661 | TG | T | 0.5 | NM_000238 | KCNH2 | frameshift_deleti  on | exon3 | c.470delC | p.P157fs | . | . |
| CTC5 | 7 | 150912010 | CT | C | 0.206 | NM_005692 | ABCF2 | frameshift_deleti  on | exon14 | c.1688delA | p.E563fs | . | . |
| CTC5 | 7 | 151846130 | CT | C | 0.333 | NM_170606 | KMT2C | frameshift_deleti  on | exon52 | c.12881delA | p.Q4294fs | . | . |
| CTC5 | 7 | 151847997 | GT | G | 0.464 | NM_170606 | KMT2C | frameshift_deleti  on | exon51 | c.12761delA | p.N4254fs | . | . |
| CTC5 | 7 | 151945071 | G | GT | 0.833 | NM_170606 | KMT2C | stopgain | exon14 | c.2447dupA | p.Y816_I817deli  nsX | . | . |
| CTC5 | 7 | 154863132 | GC | G | 0.889 | NM_024012 | HTR5A | frameshift_deleti  on | exon1 | c.524delC | p.A175fs | . | . |
| CTC5 | 7 | 155532434 | CT | C | 0.25 | NM_053043 | RBM33 | frameshift_deleti  on | exon12 | c.1764delT | p.P588fs | . | . |
| CTC5 | 7 | 155534784 | CA | C | 0.982 | NM_053043 | RBM33 | frameshift_deleti  on | exon13 | c.2322delA | p.A774fs | . | . |
| CTC5 | 7 | 158695125 | CT | C | 0.4 | NM_018051 | WDR60 | frameshift_deleti  on | exon10 | c.1197delT | p.P399fs | . | . |

| CTC5 | 7 | 158695187 | CA | C | 0.391 | NM_018051 | WDR60 | frameshift_deleti  on | exon10 | c.1259delA | p.Q420fs | . | . |
| --- | --- | --- | --- | --- | --- | --- | --- | --- | --- | --- | --- | --- | --- |
| CTC5 | 8 | 6272290 | CA | C | 0.455 | NM_001172574 | MCPH1 | frameshift_deleti  on | exon3 | c.120delA | p.S40fs | . | . |
| CTC5 | 8 | 9577981 | AC | A | 0.545 | NM_003747 | TNKS | frameshift_deleti  on | exon12 | c.1848delC | p.D616fs | . | . |
| CTC5 | 8 | 10467272 | CCTCTG  CGGGGC | C | 0.863 | NM_178857 | RP1L1 | frameshift_deleti  on | exon4 | c.4319_4335del | p.E1440fs | . | . |
| CTC5 | 8 | 11615863 | TG | T | 0.883 | NM_002052 | GATA4 | frameshift_deleti  on | exon7 | c.1209delG | p.L403fs | . | . |
| CTC5 | 8 | 12943923 | AG | A | 1 | NM_001164271 | DLC1 | frameshift_deleti  on | exon13 | c.2808delC | p.T936fs | . | . |
| CTC5 | 8 | 13357134 | CT | C | 0.259 | NM_024767 | DLC1 | frameshift_deleti  on | exon2 | c.446delA | p.K149fs | . | . |
| CTC5 | 8 | 17872098 | CA | C | 0.409 | NM_006197 | PCM1 | frameshift_deleti  on | exon36 | c.5591delA | p.Q1864fs | . | . |
| CTC5 | 8 | 19315973 | C | CT | 0.923 | NM_001130518 | CSGALNACT  1 | frameshift_inserti  on | exon5 | c.814dupA | p.R272fs | . | . |
| CTC5 | 8 | 19315973 | C | CTT | 0.923 | NM_001130518 | CSGALNACT  1 | frameshift_inserti  on | exon5 | c.814_815insAA | p.R272fs | . | . |
| CTC5 | 8 | 22388825 | TC | T | 0.286 | NM_001243974 | PPP3CC | frameshift_deleti  on | exon11 | c.1153delC | p.P385fs | . | . |
| CTC5 | 8 | 23082279 | AC | A | 0.923 | NM_003844 | TNFRSF10A | frameshift_deleti  on | exon1 | c.295delG | p.V99fs | . | . |
| CTC5 | 8 | 23112899 | TG | T | 0.538 | NM_152272 | CHMP7 | frameshift_deleti  on | exon4 | c.612delG | p.L204fs | . | . |
| CTC5 | 8 | 23153590 | CG | C | 0.658 | NM_001136108 | R3HCC1 | frameshift_deleti  on | exon8 | c.743delG | p.R248fs | . | . |
| CTC5 | 8 | 23225714 | C | CG | 0.8 | NM_002318 | LOXL2 | frameshift_inserti  on | exon2 | c.150dupC | p.A51fs | . | . |
| CTC5 | 8 | 25280828 | GT | G | 0.65 | NM_000825 | GNRH1 | frameshift_deleti  on | exon1 | c.30delA | p.K10fs | . | . |
| CTC5 | 8 | 25346139 | GA | G | 0.418 | NM_152562 | CDCA2 | frameshift_deleti  on | exon13 | c.1606delA | p.K536fs | . | . |
| CTC5 | 8 | 28206660 | CG | C | 0.462 | NM_018660 | ZNF395 | frameshift_deleti  on | exon9 | c.1411delC | p.R471fs | . | . |
| CTC5 | 8 | 30695297 | TC | T | 0.857 | NM_031271 | TEX15 | frameshift_deleti  on | exon3 | c.7353delG | p.K2451fs | . | . |
| CTC5 | 8 | 30969274 | G | GA | 0.667 | NM_000553 | WRN | frameshift_inserti  on | exon19 | c.2233dupA | p.G744fs | . | . |
| CTC5 | 8 | 36766908 | TG | T | 0.25 | NM_001031836 | KCNU1 | frameshift_deleti  on | exon21 | c.2187delG | p.M729fs | . | . |
| CTC5 | 8 | 38091988 | TG | T | 1 | NM_001164232 | DDHD2 | frameshift_deleti  on | exon3 | c.298delG | p.A100fs | . | . |
| CTC5 | 8 | 38117607 | C | CA | 0.462 | NM_001164232 | DDHD2 | frameshift_inserti  on | exon17 | c.2105dupA | p.Q702fs | . | . |
| CTC5 | 8 | 38250235 | CA | C | 1 | NM_001199659 | LETM2 | frameshift_deleti  on | exon3 | c.83delA | p.Q28fs | . | . |
| CTC5 | 8 | 41397205 | GT | G | 0.414 | NM_032336 | GINS4 | frameshift_deleti  on | exon5 | c.307delT | p.F103fs | . | . |
| CTC5 | 8 | 42401637 | A | AC | 0.375 | NM_001135674 | SMIM19 | frameshift_inserti  on | exon2 | c.22_23insC | p.M8fs | . | . |
| CTC5 | 8 | 48648054 | GGGT | G | 0.152 | NM_001282919 | SPIDR | nonframeshift_de  letion | exon19 | c.2612_2614del | p.871_872del | . | . |
| CTC5 | 8 | 52359716 | TG | T | 0.889 | NM_144651 | PXDNL | frameshift_deleti  on | exon12 | c.1372delC | p.Q458fs | . | . |
| CTC5 | 8 | 57026334 | CT | C | 0.335 | NM_005372 | MOS | frameshift_deleti  on | exon1 | c.207delA | p.G69fs | . | . |
| CTC5 | 8 | 61764684 | TA | T | 0.407 | NM_017780 | CHD7 | frameshift_deleti  on | exon29 | c.5773delA | p.K1925fs | . | . |
| CTC5 | 8 | 61768722 | TG | T | 0.78 | NM_017780 | CHD7 | frameshift_deleti  on | exon33 | c.7126delG | p.G2376fs | . | . |
| CTC5 | 8 | 65509465 | TA | T | 0.314 | NM_004820 | CYP7B1 | frameshift_deleti  on | exon6 | c.1254delT | p.F418fs | . | . |
| CTC5 | 8 | 68031029 | TCA | T | 0.236 | NM_024790 | CSPP1 | frameshift_deleti  on | exon13 | c.1656_1657del | p.V552fs | . | . |
| CTC5 | 8 | 68188267 | G | GA | 0.273 | NM_006421 | ARFGEF1 | frameshift_inserti  on | exon9 | c.1280dupT | p.F427fs | . | . |
| CTC5 | 8 | 70744809 | TG | T | 0.273 | NM_001146008 | SLCO5A1 | frameshift_deleti  on | exon1 | c.99delC | p.L33fs | . | . |
| CTC5 | 8 | 73951428 | GA | G | 0.5 | NM_003218 | TERF1 | frameshift_deleti  on | exon8 | c.1058delA | p.E353fs | . | . |
| CTC5 | 8 | 74516072 | CA | C | 0.143 | NM_001164383 | STAU2 | frameshift_deleti  on | exon5 | c.401delT | p.M134fs | . | . |
| CTC5 | 8 | 81599640 | TG | T | 0.171 | NM_001033723 | ZNF704 | frameshift_deleti  on | exon4 | c.378delC | p.S126fs | . | . |
| CTC5 | 8 | 82627096 | GT | G | 0.835 | NM_001170796 | ZFAND1 | frameshift_deleti  on | exon5 | c.300delA | p.K100fs | . | . |
| CTC5 | 8 | 87081733 | GCCGCC  GCCTCG GGCCCA | G | 0.201 | NM_033126 | PSKH2 | frameshift_deleti on | exon1 | c.99_118del | p.G33fs | . | . |
| CTC5 | 8 | 87570562 | AG | A | 0.171 | NM_003909 | CPNE3 | frameshift_deleti  on | exon17 | c.1539delG | p.Q513fs | . | . |
| CTC5 | 8 | 95392456 | CT | C | 0.571 | NM_001205263 | RAD54B | frameshift_deleti  on | exon10 | c.1611delA | p.K537fs | . | . |
| CTC5 | 8 | 95952408 | C | CTCT | 0.719 | NM_001135733 | TP53INP1 | nonframeshift_in  sertion | exon3 | c.152_153insA  GA | p.E51delinsEE | . | . |
| CTC5 | 8 | 97318756 | A | AT | 0.417 | NM_014754 | PTDSS1 | frameshift_inserti  on | exon8 | c.980dupT | p.I327fs | . | . |
| CTC5 | 8 | 98290028 | GT | G | 1 | NM_033512 | TSPYL5 | frameshift_deleti  on | exon1 | c.44delA | p.N15fs | . | . |
| CTC5 | 8 | 99206989 | GC | G | 0.267 | NM_024759 | NIPAL2 | frameshift_deleti  on | exon10 | c.1005delG | p.L335fs | . | . |
| CTC5 | 8 | 99440800 | TG | T | 0.583 | NM_020697 | KCNS2 | frameshift_deleti  on | exon2 | c.594delG | p.M198fs | . | . |
| CTC5 | 8 | 99560295 | TG | T | 0.794 | NM_001256313 | STK3 | frameshift_deleti  on | exon7 | c.709delC | p.Q237fs | . | . |
| CTC5 | 8 | 101153763 | TC | T | 0.375 | NM_001029860 | FBXO43 | frameshift_deleti  on | exon2 | c.718delG | p.D240fs | . | . |
| CTC5 | 8 | 101232585 | G | GA | 0.854 | NM_003114 | SPAG1 | frameshift_inserti  on | exon13 | c.1615dupA | p.G538fs | . | . |
| CTC5 | 8 | 101232588 | A | AG | 0.879 | NM_003114 | SPAG1 | frameshift_inserti  on | exon13 | c.1618dupG | p.K539fs | . | . |
| CTC5 | 8 | 102570790 | TC | T | 0.368 | NM_024915 | GRHL2 | frameshift_deleti  on | exon4 | c.429delC | p.F143fs | . | . |
| CTC5 | 8 | 103848523 | TC | T | 0.571 | NM_148174 | AZIN1 | frameshift_deleti  on | exon6 | c.544delG | p.E182fs | . | . |
| CTC5 | 8 | 103850996 | AT | A | 0.667 | NM_148174 | AZIN1 | frameshift_deleti  on | exon5 | c.424delA | p.I142fs | . | . |
| CTC5 | 8 | 104053182 | A | AT | 0.245 | NM_001695 | ATP6V1C1 | frameshift_inserti  on | exon2 | c.119dupT | p.I40fs | . | . |
| CTC5 | 8 | 105405084 | TG | T | 0.75 | NM_001385 | DPYS | frameshift_deleti  on | exon8 | c.1370delC | p.A457fs | . | . |
| CTC5 | 8 | 105440265 | GA | G | 0.75 | NM_001385 | DPYS | frameshift_deleti  on | exon6 | c.1034delT | p.I345fs | . | . |

| CTC5 | 8 | 110422171 | G | GA | 0.74 | NM_177531 | PKHD1L1 | frameshift_inserti  on | exon19 | c.2050dupA | p.V683fs | . | . |
| --- | --- | --- | --- | --- | --- | --- | --- | --- | --- | --- | --- | --- | --- |
| CTC5 | 8 | 110520013 | GA | G | 0.4 | NM_177531 | PKHD1L1 | frameshift_deleti  on | exon69 | c.11117delA | p.E3706fs | . | . |
| CTC5 | 8 | 110980318 | CA | C | 0.83 | NM_014379 | KCNV1 | stoploss | exon3 | c.1501delT | p.X501E | . | . |
| CTC5 | 8 | 121209125 | CG | C | 0.238 | NM_021110 | COL14A1 | frameshift_deleti on | exon6 | c.533delG | p.R178fs | . | . |
| CTC5 | 8 | 121237396 | AT | A | 0.905 | NM_021110 | COL14A1 | frameshift_deleti  on | exon15 | c.1808delT | p.I603fs | . | . |
| CTC5 | 8 | 124219901 | CT | C | 0.667 | NM_032899 | FAM83A | frameshift_deleti  on | exon5 | c.1279delT | p.F427fs | . | . |
| CTC5 | 8 | 124238831 | GC | G | 0.4 | NM_032847 | C8orf76 | frameshift_deleti  on | exon5 | c.856delG | p.A286fs | . | . |
| CTC5 | 8 | 124267955 | GA | G | 0.455 | NM_001017926 | ZHX1 | frameshift_deleti  on | exon3 | c.231delT | p.F77fs | . | . |
| CTC5 | 8 | 125498263 | TC | T | 1 | NM_007218 | RNF139 | frameshift_deleti  on | exon2 | c.374delC | p.S125fs | . | . |
| CTC5 | 8 | 125555431 | G | GA | 0.21 | NM_001278645 | NDUFB9 | frameshift_inserti  on | exon2 | c.38dupA | p.E13fs | . | . |
| CTC5 | 8 | 125989259 | G | GA | 0.59 | NM_152412 | ZNF572 | frameshift_inserti  on | exon3 | c.750dupA | p.G250fs | . | . |
| CTC5 | 8 | 125989323 | TTA | T | 0.477 | NM_152412 | ZNF572 | frameshift_deleti  on | exon3 | c.814_815del | p.Y272fs | . | . |
| CTC5 | 8 | 136594218 | CG | C | 0.625 | NM_006558 | KHDRBS3 | frameshift_deleti  on | exon6 | c.710delG | p.R237fs | . | . |
| CTC5 | 8 | 139647271 | TG | T | 0.387 | NM_152888 | COL22A1 | frameshift_deleti  on | exon49 | c.3590delC | p.P1197fs | . | . |
| CTC5 | 8 | 143603383 | TG | T | 0.294 | NM_001702 | BAI1 | frameshift_deleti  on | exon20 | c.3083delG | p.W1028fs | . | . |
| CTC5 | 8 | 143694647 | AG | A | 0.42 | NM_015193 | ARC | frameshift_deleti  on | exon1 | c.985delC | p.L329fs | . | . |
| CTC5 | 8 | 143857051 | GC | G | 0.688 | NM_023946 | LYNX1 | frameshift_deleti  on | exon3 | c.113delG | p.R38fs | . | . |
| CTC5 | 8 | 143996304 | CA | C | 0.716 | NM_000498 | CYP11B2 | frameshift_deleti  on | exon4 | c.615delT | p.F205fs | . | . |
| CTC5 | 8 | 144296975 | C | CA | 0.836 | NM_178172 | GPIHBP1 | frameshift_inserti  on | exon3 | c.270dupA | p.T90fs | . | . |
| CTC5 | 8 | 144332337 | CCA | C | 0.129 | NM_001271156 | ZFP41 | frameshift_deleti  on | exon2 | c.325_326del | p.H109fs | . | . |
| CTC5 | 8 | 144672202 | TTG | T | 0.429 | NM_001130053 | EEF1D | frameshift_deleti  on | exon3 | c.48_49del | p.D16fs | . | . |
| CTC5 | 8 | 144906519 | TGCCGC  CGCC | T | 0.4 | NM_001271096 | PUF60 | nonframeshift_de  letion | exon2 | c.63_71del | p.21_24del | . | . |
| CTC5 | 8 | 145006864 | G | GT | 0.833 | NM_201378 | PLEC | frameshift_inserti  on | exon15 | c.1711_1712ins  A | p.A571fs | . | . |
| CTC5 | 8 | 145066204 | TG | T | 0.92 | NM_000837 | GRINA | frameshift_deleti  on | exon4 | c.652delG | p.G218fs | . | . |
| CTC5 | 8 | 145095130 | TC | T | 0.938 | NM_001134374 | SPATC1 | frameshift_deleti  on | exon2 | c.533delC | p.S178fs | . | . |
| CTC5 | 8 | 145138658 | GC | G | 0.741 | NM_003801 | GPAA1 | frameshift_deleti  on | exon4 | c.409delC | p.R137fs | . | . |
| CTC5 | 8 | 145140304 | TG | T | 0.211 | NM_003801 | GPAA1 | frameshift_deleti  on | exon10 | c.1374delG | p.V458fs | . | . |
| CTC5 | 8 | 145617466 | C | CA | 0.562 | NM_174922 | ADCK5 | frameshift_inserti  on | exon12 | c.1188_1189ins  A | p.A396fs | . | . |
| CTC5 | 8 | 145617534 | TGGGGG  TGCAAG | T | 1 | NM_174922 | ADCK5 | frameshift_deleti  on | exon12 | c.1257_1267del | p.L419fs | . | . |
| CTC5 | 8 | 146107634 | CA | C | 0.438 | NM_001109689 | ZNF250 | frameshift_deleti  on | exon6 | c.933delT | p.C311fs | . | . |
| CTC5 | 9 | 3257004 | AT | A | 0.659 | NM_002919 | RFX3 | frameshift_deleti  on | exon13 | c.1800delA | p.K600fs | . | . |
| CTC5 | 9 | 4663249 | TG | T | 0.429 | NM_203453 | PPAPDC2 | stopgain | exon1 | c.875delG | p.W292X | . | . |
| CTC5 | 9 | 4860125 | GAT | G | 0.9 | NM_001286701 | RCL1 | frameshift_deleti on | exon5 | c.415_416del | p.I139fs | . | . |
| CTC5 | 9 | 5054692 | A | AG | 0.179 | NM_004972 | JAK2 | frameshift_inserti  on | exon7 | c.744_745insG | p.R248fs | . | . |
| CTC5 | 9 | 5763426 | CT | C | 0.35 | NM_001206557 | KIAA1432 | frameshift_deleti  on | exon18 | c.2289delT | p.S763fs | . | . |
| CTC5 | 9 | 5787476 | G | GA | 0.29 | NM_024896 | ERMP1 | frameshift_inserti  on | exon14 | c.2503dupT | p.S835fs | . | . |
| CTC5 | 9 | 6013726 | GA | G | 1 | NM_012416 | RANBP6 | frameshift_deleti  on | exon1 | c.1881delT | p.F627fs | . | . |
| CTC5 | 9 | 7799549 | GC | G | 0.185 | NM_033428 | TMEM261 | frameshift_deleti  on | exon1 | c.185delG | p.G62fs | . | . |
| CTC5 | 9 | 12775861 | T | TGGCG  GCGGC | 0.912 | NM_203403 | LURAP1L | nonframeshift_in  sertion | exon1 | c.147_148insG  GCGGCGGC | p.G49delinsGG  GG | . | . |
| CTC5 | 9 | 12775861 | T | TGGCG  GC | 0.912 | NM_203403 | LURAP1L | nonframeshift_in  sertion | exon1 | c.147_148insG  GCGGC | p.G49delinsGG  G | . | . |
| CTC5 | 9 | 12775876 | C | CGGCG  GCGAA | 0.769 | NM_203403 | LURAP1L | nonframeshift_in  sertion | exon1 | c.162_163insG  GCGGCGAA | p.G54delinsGG  GE | . | . |
| CTC5 | 9 | 16727958 | G | GTC | 0.172 | NM_017637 | BNC2 | frameshift_inserti  on | exon3 | c.166_167insG  A | p.T56fs | . | . |
| CTC5 | 9 | 18928083 | GT | G | 0.821 | NM_001287049 | FAM154A | frameshift_deleti  on | exon4 | c.1196delA | p.N399fs | . | . |
| CTC5 | 9 | 18941635 | CT | C | 0.312 | NM_001287049 | FAM154A | frameshift_deleti  on | exon3 | c.225delA | p.K75fs | . | . |
| CTC5 | 9 | 21859335 | GA | G | 0.271 | NM_002451 | MTAP | frameshift_deleti  on | exon7 | c.725delA | p.E242fs | . | . |
| CTC5 | 9 | 26907857 | CA | C | 0.533 | NM_001031689 | PLAA | frameshift_deleti  on | exon13 | c.1796delT | p.L599fs | . | . |
| CTC5 | 9 | 27056454 | TC | T | 0.467 | NM_001099222 | IFT74 | frameshift_deleti  on | exon18 | c.1621delC | p.Q541fs | . | . |
| CTC5 | 9 | 27183471 | AC | A | 0.357 | NM_000459 | TEK | frameshift_deleti  on | exon8 | c.1046delC | p.T349fs | . | . |
| CTC5 | 9 | 27212793 | AT | A | 0.229 | NM_000459 | TEK | frameshift_deleti  on | exon17 | c.2776delT | p.F926fs | . | . |
| CTC5 | 9 | 32630414 | CT | C | 0.6 | NM_153809 | TAF1L | frameshift_deleti  on | exon1 | c.5163delA | p.E1721fs | . | . |
| CTC5 | 9 | 32630604 | T | TC | 0.667 | NM_153809 | TAF1L | frameshift_inserti  on | exon1 | c.4973_4974ins  G | p.P1658fs | . | . |
| CTC5 | 9 | 32632669 | GT | G | 0.357 | NM_153809 | TAF1L | frameshift_deleti  on | exon1 | c.2908delA | p.T970fs | . | . |
| CTC5 | 9 | 32987676 | TC | T | 0.207 | NM_001195250 | APTX | frameshift_deleti  on | exon3 | c.228delG | p.K76fs | . | . |
| CTC5 | 9 | 33030601 | TA | T | 0.833 | NM_001539 | DNAJA1 | frameshift_deleti  on | exon5 | c.580delA | p.K194fs | . | . |
| CTC5 | 9 | 33344123 | TG | T | 1 | NM_002504 | NFX1 | frameshift_deleti  on | exon14 | c.2282delG | p.C761fs | . | . |
| CTC5 | 9 | 33917200 | TATG | T | 0.455 | NM_017811 | UBE2R2 | nonframeshift_de  letion | exon5 | c.683_685del | p.228_229del | . | . |
| CTC5 | 9 | 33923212 | GT | G | 0.8 | NM_001282530 | UBAP2 | frameshift_deleti  on | exon10 | c.692delA | p.N231fs | . | . |

| CTC5 | 9 | 33943556 | GA | G | 0.563 | NM_001282529 | UBAP2 | frameshift_deleti  on | exon11 | c.775delT | p.S259fs | . | . |
| --- | --- | --- | --- | --- | --- | --- | --- | --- | --- | --- | --- | --- | --- |
| CTC5 | 9 | 33986819 | AT | A | 0.342 | NM_018449 | UBAP2 | frameshift_deleti  on | exon6 | c.458delA | p.N153fs | . | . |
| CTC5 | 9 | 34257242 | CT | C | 0.474 | NM_194313 | KIF24 | frameshift_deleti  on | exon11 | c.2362delA | p.R788fs | . | . |
| CTC5 | 9 | 34647216 | CA | C | 0.353 | NM_000155 | GALT | frameshift_deleti  on | exon2 | c.214delA | p.N72fs | . | . |
| CTC5 | 9 | 34648823 | AC | A | 0.984 | NM_001258332 | GALT | frameshift_deleti  on | exon6 | c.426delC | p.Y142fs | . | . |
| CTC5 | 9 | 34990740 | TG | T | 0.444 | NM_001135005 | DNAJB5 | frameshift_deleti  on | exon1 | c.114delG | p.L38fs | . | . |
| CTC5 | 9 | 35258995 | TA | T | 1 | NM_006377 | UNC13B | frameshift_deleti  on | exon7 | c.475delA | p.S159fs | . | . |
| CTC5 | 9 | 35548440 | GC | G | 1 | NM_001135999 | RUSC2 | frameshift_deleti  on | exon2 | c.1923delC | p.G641fs | . | . |
| CTC5 | 9 | 35562437 | GT | G | 0.231 | NM_001099951 | FAM166B | frameshift_deleti  on | exon5 | c.610delA | p.T204fs | . | . |
| CTC5 | 9 | 35650626 | A | AG | 0.574 | NM_014450 | SIT1 | frameshift_inserti  on | exon2 | c.108dupC | p.S37fs | . | . |
| CTC5 | 9 | 35673970 | G | GC | 0.833 | NM_001216 | CA9 | frameshift_inserti  on | exon1 | c.15dupC | p.C5fs | . | . |
| CTC5 | 9 | 35906347 | CCTG | C | 0.286 | NM_001039792 | HRCT1 | nonframeshift_de  letion | exon1 | c.64_66del | p.22_22del | . | . |
| CTC5 | 9 | 36677233 | CA | C | 1 | NM_001256692 | MELK | frameshift_deleti  on | exon15 | c.1463delA | p.Q488fs | . | . |
| CTC5 | 9 | 36677233 | CAA | C | 1 | NM_001256692 | MELK | frameshift_deleti  on | exon15 | c.1463_1464del | p.Q488fs | . | . |
| CTC5 | 9 | 37441119 | TG | T | 0.2 | NM_014872 | ZBTB5 | frameshift_deleti  on | exon2 | c.1429delC | p.H477fs | . | . |
| CTC5 | 9 | 73206067 | C | CA | 0.313 | NM_206944 | TRPM3 | frameshift_inserti  on | exon20 | c.2577dupT | p.G860fs | . | . |
| CTC5 | 9 | 73461480 | GT | G | 1 | NM_001007470 | TRPM3 | frameshift_deleti  on | exon4 | c.30delA | p.K10fs | . | . |
| CTC5 | 9 | 79318375 | GGTGAC  AGCCTG | G | 1 | NM_015225 | PRUNE2 | nonframeshift_de  letion | exon9 | c.8139_8153del | p.2713_2718de  l | . | . |
| CTC5 | 9 | 79320391 | AC | A | 1 | NM_015225 | PRUNE2 | frameshift_deleti  on | exon8 | c.6798delG | p.L2266fs | . | . |
| CTC5 | 9 | 84606857 | GC | G | 0.4 | NM_001001670 | SPATA31D1 | frameshift_deleti  on | exon4 | c.1473delC | p.C491fs | . | . |
| CTC5 | 9 | 86495353 | AT | A | 0.424 | NM_001271928 | KIF27 | frameshift_deleti  on | exon11 | c.2501delA | p.N834fs | . | . |
| CTC5 | 9 | 86587854 | A | AT | 0.235 | NM_002140 | HNRNPK | frameshift_inserti  on | exon10 | c.549dupA | p.C184fs | . | . |
| CTC5 | 9 | 87342711 | T | TA | 0.889 | NM_001007097 | NTRK2 | frameshift_inserti  on | exon9 | c.997dupA | p.T332fs | . | . |
| CTC5 | 9 | 90500889 | A | AT | 0.5 | NM_178828 | SPATA31E1 | frameshift_inserti  on | exon4 | c.1487_1488ins  T | p.Q496fs | . | . |
| CTC5 | 9 | 91262533 | CT | C | 0.667 | NM_001100111 | RP13-  60M5.2 | frameshift_deleti  on | exon2 | c.109delA | p.S37fs | . | . |
| CTC5 | 9 | 91616152 | CGG | C | 0.238 | NM_005226 | S1PR3 | frameshift_deleti  on | exon2 | c.38_39del | p.R13fs | . | . |
| CTC5 | 9 | 91940449 | T | TA | 0.2 | NM_001282688 | SECISBP2 | frameshift_inserti  on | exon3 | c.290_291insA | p.L97fs | . | . |
| CTC5 | 9 | 94984823 | GA | G | 0.5 | NM_002161 | IARS | frameshift_deleti  on | exon33 | c.3684delT | p.F1228fs | . | . |
| CTC5 | 9 | 95003185 | GC | G | 0.182 | NM_002161 | IARS | frameshift_deleti  on | exon30 | c.3235delG | p.A1079fs | . | . |
| CTC5 | 9 | 95237024 | CTCA | C | 1 | NM_001193335 | ASPN | nonframeshift_de  letion | exon2 | c.153_155del | p.51_52del | . | . |
| CTC5 | 9 | 95237024 | CTCATC  ATCA | C | 1 | NM_001193335 | ASPN | nonframeshift_de  letion | exon2 | c.147_155del | p.49_52del | . | . |
| CTC5 | 9 | 95609712 | AT | A | 0.857 | NM_001007101 | ZNF484 | frameshift_deleti  on | exon4 | c.1248delA | p.K416fs | . | . |
| CTC5 | 9 | 96291772 | GC | G | 0.921 | NM_001286722 | FAM120A | frameshift_deleti  on | exon9 | c.1642delC | p.P548fs | . | . |
| CTC5 | 9 | 97062595 | ACC | A | 0.118 | NM_194320 | ZNF169 | frameshift_deleti  on | exon5 | c.756_757del | p.H252fs | . | . |
| CTC5 | 9 | 98669508 | TA | T | 0.842 | NM_001010895 | ERCC6L2 | frameshift_deleti  on | exon4 | c.777delA | p.L259fs | . | . |
| CTC5 | 9 | 100194446 | ACACAC | A | 0.28 | NM_014290 | TDRD7 | frameshift_deleti  on | exon4 | c.490_494del | p.H164fs | . | . |
| CTC5 | 9 | 100893325 | GGC | G | 0.929 | NM_003389 | CORO2A | frameshift_deleti  on | exon7 | c.780_781del | p.V260fs | . | . |
| CTC5 | 9 | 100971280 | GC | G | 0.261 | NM_001267572 | TBC1D2 | frameshift_deleti  on | exon3 | c.439delG | p.A147fs | . | . |
| CTC5 | 9 | 101980290 | TG | T | 0.215 | NM_033087 | ALG2 | frameshift_deleti  on | exon2 | c.1176delC | p.A392fs | . | . |
| CTC5 | 9 | 104124731 | TG | T | 0.722 | NM_001127610 | BAAT | frameshift_deleti  on | exon4 | c.1235delC | p.P412fs | . | . |
| CTC5 | 9 | 107379545 | C | CT | 0.333 | NM_001001956 | OR13C9 | frameshift_inserti  on | exon1 | c.940dupA | p.R314fs | . | . |
| CTC5 | 9 | 108136961 | CT | C | 0.983 | NM_001286730 | SLC44A1 | frameshift_deleti  on | exon13 | c.1578delT | p.A526fs | . | . |
| CTC5 | 9 | 109694817 | GC | G | 0.294 | NM_021224 | ZNF462 | frameshift_deleti  on | exon5 | c.6104delC | p.A2035fs | . | . |
| CTC5 | 9 | 111618004 | CG | C | 1 | NM_006686 | ACTL7B | frameshift_deleti  on | exon1 | c.206delC | p.P69fs | . | . |
| CTC5 | 9 | 111701729 | GA | G | 0.349 | NM_017832 | FAM206A | frameshift_deleti  on | exon5 | c.404delA | p.E135fs | . | . |
| CTC5 | 9 | 112151604 | AG | A | 0.357 | NM_001145372 | PTPN3 | frameshift_deleti  on | exon11 | c.1165delC | p.L389fs | . | . |
| CTC5 | 9 | 113169067 | TC | T | 1 | NM_153366 | SVEP1 | frameshift_deleti  on | exon38 | c.8812delG | p.D2938fs | . | . |
| CTC5 | 9 | 116171120 | GTTC | G | 0.125 | NM_001278255 | POLE3 | nonframeshift_de  letion | exon5 | c.412_414del | p.138_138del | . | . |
| CTC5 | 9 | 116171217 | CT | C | 0.37 | NM_001278255 | POLE3 | frameshift_deleti  on | exon5 | c.317delA | p.K106fs | . | . |
| CTC5 | 9 | 116812284 | C | CA | 0.618 | NM_133374 | ZNF618 | frameshift_inserti  on | exon14 | c.2424dupA | p.T808fs | . | . |
| CTC5 | 9 | 116956692 | AG | A | 0.277 | NM_032888 | COL27A1 | frameshift_deleti  on | exon6 | c.2026delG | p.G676fs | . | . |
| CTC5 | 9 | 123222902 | TC | T | 0.5 | NM_001011649 | CDK5RAP2 | frameshift_deleti  on | exon19 | c.2149delG | p.E717fs | . | . |
| CTC5 | 9 | 123753498 | GT | G | 0.327 | NM_001735 | C5 | frameshift_deleti  on | exon23 | c.2911delA | p.T971fs | . | . |
| CTC5 | 9 | 125288863 | GA | G | 0.471 | NM_012363 | OR1N1 | frameshift_deleti  on | exon1 | c.709delT | p.S237fs | . | . |
| CTC5 | 9 | 125424289 | AC | A | 0.295 | NM_001005236 | OR1L1 | frameshift_deleti  on | exon1 | c.296delC | p.T99fs | . | . |
| CTC5 | 9 | 125797337 | CT | C | 0.395 | NM_005294 | GPR21 | frameshift_deleti  on | exon2 | c.493delT | p.F165fs | . | . |
| CTC5 | 9 | 128230325 | CA | C | 1 | NM_001006621 | MAPKAP1 | frameshift_deleti  on | exon8 | c.694delT | p.W232fs | . | . |

| CTC5 | 9 | 129979190 | CG | C | 0.395 | NM_001190728 | RALGPS1 | frameshift_deleti  on | exon17 | c.1501delG | p.G501fs | . | . |
| --- | --- | --- | --- | --- | --- | --- | --- | --- | --- | --- | --- | --- | --- |
| CTC5 | 9 | 130440805 | CA | C | 0.194 | NM_001032221 | STXBP1 | frameshift_deleti  on | exon16 | c.1456delA | p.M486fs | . | . |
| CTC5 | 9 | 130474532 | G | GT | 0.433 | NM_001012502 | C9orf117 | frameshift_inserti  on | exon6 | c.1041_1042ins  T | p.Q347fs | . | . |
| CTC5 | 9 | 130493139 | CGGAAC  TCCAGC AAGACC AAGGCC ACTATA | C | 0.882 | NM_144965 | TTC16 | nonframeshift_de letion | exon14 | c.2078_2113del | p.693_705del | . | . |
| CTC5 | 9 | 131247067 | GAC | G | 1 | NM_001242354 | ODF2 | frameshift_deleti  on | exon11 | c.950_951del | p.D317fs | . | . |
| CTC5 | 9 | 131388686 | TG | T | 0.604 | NM_001195532 | SPTAN1 | frameshift_deleti  on | exon47 | c.6222delG | p.L2074fs | . | . |
| CTC5 | 9 | 131469640 | AC | A | 1 | NM_013355 | PKN3 | frameshift_deleti  on | exon6 | c.792delC | p.Y264fs | . | . |
| CTC5 | 9 | 131583075 | ACC | A | 0.588 | NM_004435 | ENDOG | frameshift_deleti  on | exon2 | c.560_561del | p.T187fs | . | . |
| CTC5 | 9 | 131708195 | TC | T | 0.281 | NM_014908 | DOLK | frameshift_deleti  on | exon1 | c.1387delG | p.E463fs | . | . |
| CTC5 | 9 | 133577533 | GT | G | 0.279 | NM_001282708 | EXOSC2 | frameshift_deleti  on | exon6 | c.431delT | p.V144fs | . | . |
| CTC5 | 9 | 133759489 | CAAG | C | 0.194 | NM_005157 | ABL1 | nonframeshift_de  letion | exon11 | c.1813_1815del | p.605_605del | . | . |
| CTC5 | 9 | 134019968 | C | CG | 0.3 | NM_005085 | NUP214 | frameshift_inserti  on | exon12 | c.1596_1597ins  G | p.P532fs | . | . |
| CTC5 | 9 | 136404947 | TC | T | 0.338 | NM_001145320 | ADAMTSL2 | frameshift_deleti  on | exon5 | c.365delC | p.S122fs | . | . |
| CTC5 | 9 | 136570083 | CG | C | 0.875 | NM_001134707 | SARDH | frameshift_deleti  on | exon12 | c.1540delC | p.R514fs | . | . |
| CTC5 | 9 | 139277994 | CGCT | C | 0.194 | NM_003086 | SNAPC4 | nonframeshift_de  letion | exon15 | c.1624_1626del | p.542_542del | . | . |
| CTC5 | 9 | 139341772 | TC | T | 0.875 | NM_001276418 | SEC16A | frameshift_deleti  on | exon26 | c.6603delG | p.G2201fs | . | . |
| CTC5 | 9 | 139929414 | G | GA | 0.462 | NM_207511 | C9orf139 | frameshift_inserti  on | exon3 | c.482dupA | p.E161fs | . | . |
| CTC5 | 9 | 140040335 | TG | T | 1 | NM_000832 | GRIN1 | frameshift_deleti  on | exon3 | c.552delG | p.L184fs | . | . |
| CTC5 | 9 | 140707903 | GC | G | 1 | NM_024757 | EHMT1 | frameshift_deleti  on | exon21 | c.3102delC | p.C1034fs | . | . |
| CTC5 | 10 | 3177987 | AT | A | 0.531 | NM_002627 | PFKP | frameshift_deleti  on | exon21 | c.2183delT | p.I728fs | . | . |
| CTC5 | 10 | 5435964 | TCAG | T | 0.953 | NM_001171864 | TUBAL3 | nonframeshift_de  letion | exon4 | c.734_736del | p.245_246del | . | . |
| CTC5 | 10 | 5789879 | G | GT | 0.429 | NM_017782 | FAM208B | frameshift_inserti  on | exon15 | c.4496dupT | p.V1499fs | . | . |
| CTC5 | 10 | 5789966 | CCTGCC  TCAG | C | 1 | NM_017782 | FAM208B | nonframeshift_de  letion | exon15 | c.4583_4591del | p.1528_1531de  l | . | . |
| CTC5 | 10 | 13538833 | GC | G | 0.5 | NM_001100912 | BEND7 | frameshift_deleti  on | exon3 | c.386delG | p.C129fs | . | . |
| CTC5 | 10 | 17026144 | AT | A | 0.337 | NM_001081 | CUBN | frameshift_deleti  on | exon30 | c.4484delA | p.N1495fs | . | . |
| CTC5 | 10 | 21141548 | TG | T | 0.667 | NM_006393 | NEBL | frameshift_deleti  on | exon10 | c.933delC | p.N311fs | . | . |
| CTC5 | 10 | 22048195 | AT | A | 0.273 | NM_022365 | DNAJC1 | frameshift_deleti  on | exon11 | c.1499delA | p.N500fs | . | . |
| CTC5 | 10 | 24883937 | GT | G | 0.189 | NM_020824 | ARHGAP21 | frameshift_deleti  on | exon21 | c.3817delA | p.T1273fs | . | . |
| CTC5 | 10 | 27389098 | GC | G | 0.332 | NM_001256053 | ANKRD26 | frameshift_deleti  on | exon1 | c.157delG | p.A53fs | . | . |
| CTC5 | 10 | 27458921 | GA | G | 0.8 | NM_001172303 | MASTL | frameshift_deleti  on | exon8 | c.1034delA | p.E345fs | . | . |
| CTC5 | 10 | 27700779 | GC | G | 0.308 | NM_001034842 | PTCHD3 | frameshift_deleti  on | exon2 | c.1168delG | p.A390fs | . | . |
| CTC5 | 10 | 30316500 | ACTG | A | 0.167 | NM_020848 | KIAA1462 | nonframeshift_de  letion | exon3 | c.2574_2576del | p.858_859del | . | . |
| CTC5 | 10 | 30317445 | A | AT | 0.692 | NM_020848 | KIAA1462 | frameshift_inserti  on | exon3 | c.1631_1632ins  A | p.P544fs | . | . |
| CTC5 | 10 | 30336555 | T | TG | 0.842 | NM_020848 | KIAA1462 | frameshift_inserti  on | exon2 | c.186_187insC | p.K63fs | . | . |
| CTC5 | 10 | 30602602 | T | TA | 0.944 | NM_018109 | MTPAP | frameshift_inserti  on | exon9 | c.1684_1685ins  T | p.K562fs | . | . |
| CTC5 | 10 | 35351915 | CA | C | 0.778 | NM_001198777 | CUL2 | frameshift_deleti  on | exon3 | c.194delT | p.L65fs | . | . |
| CTC5 | 10 | 43289392 | GT | G | 0.265 | NM_014753 | BMS1 | frameshift_deleti  on | exon9 | c.1183delT | p.F395fs | . | . |
| CTC5 | 10 | 43292017 | GTGA | G | 0.438 | NM_014753 | BMS1 | nonframeshift_de  letion | exon10 | c.1326_1328del | p.442_443del | . | . |
| CTC5 | 10 | 43692494 | TA | T | 0.538 | NM_001282862 | RASGEF1A | frameshift_deleti  on | exon11 | c.1301delT | p.V434fs | . | . |
| CTC5 | 10 | 46999591 | C | CATGA  GGGAG | 0.889 | NM_014696 | GPRIN2 | nonframeshift_in  sertion | exon3 | c.711_712insAT  GAGGGAG | p.G237delinsG  MRE | . | . |
| CTC5 | 10 | 46999604 | A | AGGGA  GCTGG | 0.778 | NM_014696 | GPRIN2 | nonframeshift_in  sertion | exon3 | c.724_725insG  GGAGCTGG | p.R242delinsRE  LG | . | . |
| CTC5 | 10 | 50272766 | AC | A | 0.396 | NM_001031746 | VSTM4 | stopgain | exon5 | c.649delG | p.V217X | . | . |
| CTC5 | 10 | 50530597 | CA | C | 0.211 | NM_001135196 | C10orf71 | frameshift_deleti on | exon3 | c.8delA | p.Q3fs | . | . |
| CTC5 | 10 | 50533170 | CA | C | 0.359 | NM_001135196 | C10orf71 | frameshift_deleti  on | exon3 | c.2581delA | p.T861fs | . | . |
| CTC5 | 10 | 50668490 | A | AT | 0.237 | NM_000124 | ERCC6 | frameshift_inserti  on | exon20 | c.3990dupA | p.F1331fs | . | . |
| CTC5 | 10 | 53458115 | TG | T | 0.8 | NM_015235 | CSTF2T | frameshift_deleti  on | exon1 | c.1194delC | p.P398fs | . | . |
| CTC5 | 10 | 61834575 | CT | C | 0.8 | NM_020987 | ANK3 | frameshift_deleti  on | exon37 | c.6063delA | p.K2021fs | . | . |
| CTC5 | 10 | 63520712 | TA | T | 0.389 | NM_173554 | C10orf107 | frameshift_deleti  on | exon6 | c.504delA | p.V168fs | . | . |
| CTC5 | 10 | 63958042 | CT | C | 0.7 | NM_145307 | RTKN2 | frameshift_deleti  on | exon12 | c.1454delA | p.K485fs | . | . |
| CTC5 | 10 | 64022534 | TC | T | 1 | NM_001282941 | RTKN2 | frameshift_deleti  on | exon2 | c.106delG | p.E36fs | . | . |
| CTC5 | 10 | 64967950 | ACTAAA  C | A | 0.444 | NM_001282948 | JMJD1C | nonframeshift_de  letion | exon9 | c.2927_2932del | p.976_978del | . | . |
| CTC5 | 10 | 68686800 | CA | C | 0.353 | NM_178011 | LRRTM3 | frameshift_deleti  on | exon2 | c.127delA | p.K43fs | . | . |
| CTC5 | 10 | 70048390 | TG | T | 0.357 | NM_001033083 | PBLD | frameshift_deleti  on | exon8 | c.540delC | p.N180fs | . | . |
| CTC5 | 10 | 70450616 | AT | A | 0.364 | NM_030625 | TET1 | frameshift_deleti  on | exon12 | c.5457delT | p.H1819fs | . | . |
| CTC5 | 10 | 72100311 | CG | C | 0.283 | NM_001278214 | LRRC20 | frameshift_deleti  on | exon2 | c.22delC | p.R8fs | . | . |

| CTC5 | 10 | 72192791 | TG | T | 0.214 | NM_018055 | NODAL | frameshift_deleti  on | exon3 | c.944delC | p.P315fs | . | . |
| --- | --- | --- | --- | --- | --- | --- | --- | --- | --- | --- | --- | --- | --- |
| CTC5 | 10 | 72434645 | AT | A | 0.39 | NM_080722 | ADAMTS14 | frameshift_deleti  on | exon2 | c.417delT | p.D139fs | . | . |
| CTC5 | 10 | 73511567 | CT | C | 0.444 | NM_022153 | C10orf54 | frameshift_deleti  on | exon6 | c.755delA | p.Q252fs | . | . |
| CTC5 | 10 | 75058781 | GT | G | 0.806 | NM_145170 | TTC18 | frameshift_deleti  on | exon15 | c.1605delA | p.K535fs | . | . |
| CTC5 | 10 | 75107938 | TA | T | 0.408 | NM_145170 | TTC18 | stopgain | exon5 | c.404delT | p.L135X | . | . |
| CTC5 | 10 | 75676255 | AC | A | 0.273 | NM_001145031 | PLAU | frameshift_deleti on | exon10 | c.1178delC | p.T393fs | . | . |
| CTC5 | 10 | 76781905 | GGAA | G | 0.706 | NM_001256468 | KAT6B | nonframeshift_de  letion | exon16 | c.2740_2742del | p.914_914del | . | . |
| CTC5 | 10 | 76972038 | AT | A | 0.625 | NM_001184823 | VDAC2 | frameshift_deleti  on | exon3 | c.83delT | p.I28fs | . | . |
| CTC5 | 10 | 86273227 | GC | G | 0.234 | NM_001284242 | CCSER2 | frameshift_deleti  on | exon7 | c.630delC | p.S210fs | . | . |
| CTC5 | 10 | 88950317 | TTG | T | 0.273 | NM_019054 | FAM35A | frameshift_deleti  on | exon9 | c.2386_2387del | p.C796fs | . | . |
| CTC5 | 10 | 93024215 | A | AT | 0.25 | NM_001256549 | PCGF5 | frameshift_inserti  on | exon8 | c.602dupT | p.M201fs | . | . |
| CTC5 | 10 | 93617239 | G | GT | 0.326 | NM_025235 | TNKS2 | frameshift_inserti  on | exon24 | c.3047dupT | p.V1016fs | . | . |
| CTC5 | 10 | 95556984 | A | AT | 0.3 | NM_005097 | LGI1 | frameshift_inserti  on | exon8 | c.1099dupT | p.G366fs | . | . |
| CTC5 | 10 | 95931179 | A | AT | 0.694 | NM_001165979 | PLCE1 | frameshift_inserti  on | exon3 | c.812dupT | p.I271fs | . | . |
| CTC5 | 10 | 98130068 | GCA | G | 0.955 | NM_012465 | TLL2 | frameshift_deleti  on | exon20 | c.2665_2666del | p.C889fs | . | . |
| CTC5 | 10 | 99023182 | AT | A | 0.556 | NM_001204300 | ARHGAP19 | frameshift_deleti  on | exon4 | c.607delA | p.I203fs | . | . |
| CTC5 | 10 | 99140585 | C | CCT | 0.158 | NM_001284337 | RRP12 | frameshift_inserti  on | exon10 | c.1203_1204ins  AG | p.G402fs | . | . |
| CTC5 | 10 | 101496029 | G | GT | 0.245 | NM_015960 | CUTC | frameshift_inserti  on | exon2 | c.89dupT | p.V30fs | . | . |
| CTC5 | 10 | 102684031 | CA | C | 0.375 | NM_001136123 | FAM178A | frameshift_deleti  on | exon5 | c.1274delA | p.Q425fs | . | . |
| CTC5 | 10 | 102798448 | TG | T | 0.8 | NM_030971 | SFXN3 | frameshift_deleti  on | exon10 | c.833delG | p.C278fs | . | . |
| CTC5 | 10 | 102896663 | GC | G | 1 | NM_005521 | TLX1 | frameshift_deleti  on | exon3 | c.987delC | p.C329fs | . | . |
| CTC5 | 10 | 104359242 | AC | A | 0.4 | NM_001178133 | SUFU | frameshift_deleti  on | exon8 | c.964delC | p.P322fs | . | . |
| CTC5 | 10 | 104634380 | AAAC | A | 0.25 | NM_020682 | AS3MT | nonframeshift_de  letion | exon6 | c.491_493del | p.164_165del | . | . |
| CTC5 | 10 | 108489835 | GCA | G | 0.238 | NM_001013031 | SORCS1 | frameshift_deleti  on | exon6 | c.995_996del | p.V332fs | . | . |
| CTC5 | 10 | 112266825 | AC | A | 0.492 | NM_004419 | DUSP5 | frameshift_deleti  on | exon3 | c.662delC | p.T221fs | . | . |
| CTC5 | 10 | 112581645 | AT | A | 0.312 | NM_001134363 | RBM20 | frameshift_deleti  on | exon11 | c.3269delT | p.I1090fs | . | . |
| CTC5 | 10 | 112724579 | GA | G | 0.862 | NM_001269039 | SHOC2 | frameshift_deleti  on | exon1 | c.464delA | p.E155fs | . | . |
| CTC5 | 10 | 114205004 | CA | C | 0.227 | NM_022494 | ZDHHC6 | frameshift_deleti  on | exon2 | c.190delT | p.W64fs | . | . |
| CTC5 | 10 | 115489264 | TC | T | 0.845 | NM_001267058 | CASP7 | frameshift_deleti  on | exon6 | c.803delC | p.S268fs | . | . |
| CTC5 | 10 | 115610238 | A | AG | 0.5 | NM_014881 | DCLRE1A | frameshift_inserti  on | exon2 | c.625dupC | p.L209fs | . | . |
| CTC5 | 10 | 115636580 | T | TA | 0.8 | NM_198514 | NHLRC2 | frameshift_inserti  on | exon3 | c.633dupA | p.I211fs | . | . |
| CTC5 | 10 | 115971640 | AC | A | 0.282 | NM_198795 | TDRD1 | frameshift_deleti  on | exon14 | c.1677delC | p.Y559fs | . | . |
| CTC5 | 10 | 118231327 | CA | C | 0.233 | NM_001011709 | PNLIPRP3 | frameshift_deleti  on | exon10 | c.1109delA | p.Q370fs | . | . |
| CTC5 | 10 | 118363551 | AT | A | 0.6 | NM_006229 | PNLIPRP1 | stopgain | exon11 | c.1074delT | p.Y358X | . | . |
| CTC5 | 10 | 118630761 | G | GC | 1 | NM_001242699 | ENO4 | frameshift_inserti on | exon9 | c.1174dupC | p.L391fs | . | . |
| CTC5 | 10 | 120450825 | GC | G | 0.75 | NM_153810 | CACUL1 | frameshift_deleti  on | exon7 | c.976delG | p.A326fs | . | . |
| CTC5 | 10 | 121580370 | TA | T | 1 | NM_001243194 | INPP5F | frameshift_deleti  on | exon2 | c.70delA | p.T24fs | . | . |
| CTC5 | 10 | 123276893 | AC | A | 0.759 | NM_001144916 | FGFR2 | frameshift_deleti  on | exon5 | c.678delG | p.T226fs | . | . |
| CTC5 | 10 | 123843364 | TG | T | 0.405 | NM_206862 | TACC2 | frameshift_deleti  on | exon4 | c.1350delG | p.M450fs | . | . |
| CTC5 | 10 | 125780750 | TA | T | 0.6 | NM_001270765 | CHST15 | frameshift_deleti  on | exon6 | c.1368delT | p.R456fs | . | . |
| CTC5 | 10 | 129201407 | TG | T | 0.828 | NM_001380 | DOCK1 | frameshift_deleti  on | exon39 | c.3954delG | p.L1318fs | . | . |
| CTC5 | 10 | 129902930 | GT | G | 0.4 | NM_001145966 | MKI67 | frameshift_deleti  on | exon12 | c.6093delA | p.K2031fs | . | . |
| CTC5 | 10 | 129903542 | CT | C | 0.25 | NM_001145966 | MKI67 | frameshift_deleti  on | exon12 | c.5481delA | p.K1827fs | . | . |
| CTC5 | 10 | 129905111 | CTG | C | 0.375 | NM_001145966 | MKI67 | frameshift_deleti  on | exon12 | c.3911_3912del | p.T1304fs | . | . |
| CTC5 | 10 | 129905843 | CTG | C | 0.814 | NM_001145966 | MKI67 | frameshift_deleti  on | exon12 | c.3179_3180del | p.T1060fs | . | . |
| CTC5 | 10 | 131334563 | TG | T | 0.345 | NM_002412 | MGMT | frameshift_deleti  on | exon2 | c.141delG | p.L47fs | . | . |
| CTC5 | 10 | 131959096 | GC | G | 0.833 | NM_001199868 | GLRX3 | frameshift_deleti  on | exon4 | c.314delC | p.A105fs | . | . |
| CTC5 | 10 | 135009224 | C | CA | 0.353 | NM_152643 | KNDC1 | frameshift_inserti  on | exon10 | c.1633_1634ins  A | p.R545fs | . | . |
| CTC5 | 10 | 135106666 | TCAC | T | 0.267 | NM_001256618 | TUBGCP2 | nonframeshift_de  letion | exon6 | c.508_510del | p.170_170del | . | . |
| CTC5 | 10 | 135116377 | AT | A | 0.778 | NM_001256617 | TUBGCP2 | frameshift_deleti  on | exon2 | c.68delA | p.D23fs | . | . |
| CTC5 | 11 | 800857 | A | AC | 1 | NM_145886 | PIDD | frameshift_inserti  on | exon11 | c.1821_1822ins  G | p.W608fs | . | . |
| CTC5 | 11 | 1090951 | TACC | T | 0.241 | NM_002457 | MUC2 | nonframeshift_de  letion | exon28 | c.3847_3849del | p.1283_1283de  l | . | . |
| CTC5 | 11 | 1263535 | G | GA | 0.75 | NM_002458 | MUC5B | frameshift_inserti  on | exon31 | c.5426dupA | p.E1809fs | . | . |
| CTC5 | 11 | 1586939 | TG | T | 0.941 | NM_004420 | DUSP8 | frameshift_deleti  on | exon2 | c.117delC | p.N39fs | . | . |
| CTC5 | 11 | 1953710 | CT | C | 0.5 | NM_001042780 | TNNT3 | frameshift_deleti  on | exon8 | c.114delT | p.P38fs | . | . |
| CTC5 | 11 | 2424492 | AG | A | 0.385 | NM_005706 | TSSC4 | frameshift_deleti  on | exon2 | c.630delG | p.E210fs | . | . |
| CTC5 | 11 | 3733781 | CT | C | 0.835 | NM_005387 | NUP98 | frameshift_deleti  on | exon20 | c.2805delA | p.K935fs | . | . |
| CTC5 | 11 | 4592706 | T | TAC | 1 | NM_144663 | C11orf40 | frameshift_inserti  on | exon4 | c.600_601insGT | p.M201fs | . | . |

| CTC5 | 11 | 4790751 | TC | T | 0.857 | NM_001004752 | OR51F1 | frameshift_deleti  on | exon1 | c.396delG | p.L132fs | . | . |
| --- | --- | --- | --- | --- | --- | --- | --- | --- | --- | --- | --- | --- | --- |
| CTC5 | 11 | 4936766 | GA | G | 0.271 | NM_001005238 | OR51G2 | frameshift_deleti  on | exon1 | c.127delT | p.S43fs | . | . |
| CTC5 | 11 | 5322342 | GA | G | 1 | NM_033179 | OR51B4 | frameshift_deleti  on | exon1 | c.834delT | p.F278fs | . | . |
| CTC5 | 11 | 5364262 | GA | G | 0.473 | NM_001005567 | OR51B5 | frameshift_deleti  on | exon5 | c.492delT | p.F164fs | . | . |
| CTC5 | 11 | 5701329 | TC | T | 0.3 | NM_033034 | TRIM5 | frameshift_deleti  on | exon2 | c.78delG | p.L26fs | . | . |
| CTC5 | 11 | 5841604 | CT | C | 0.625 | NM_001005174 | OR52N2 | frameshift_deleti  on | exon1 | c.40delT | p.F14fs | . | . |
| CTC5 | 11 | 6520136 | GA | G | 1 | NM_173589 | DNHD1 | frameshift_deleti  on | exon2 | c.692delA | p.E231fs | . | . |
| CTC5 | 11 | 6567895 | C | CTGCC  CTACT | 1 | NM_144666 | DNHD1 | nonframeshift_in  sertion | exon21 | c.5726_5727ins  TGCCCTACTGC | p.A1909delinsA  ALLH | . | . |
| CTC5 | 11 | 6578613 | TGAG | T | 0.16 | NM_144666 | DNHD1 | nonframeshift_de  letion | exon25 | c.8089_8091del | p.2697_2697de  l | . | . |
| CTC5 | 11 | 6592533 | TGG | T | 0.969 | NM_144666 | DNHD1 | frameshift_deleti  on | exon42 | c.13792_13793  del | p.G4598fs | . | . |
| CTC5 | 11 | 7960360 | CT | C | 0.348 | NM_001003745 | OR10A3 | frameshift_deleti  on | exon1 | c.707delA | p.K236fs | . | . |
| CTC5 | 11 | 7960562 | CA | C | 0.31 | NM_001003745 | OR10A3 | frameshift_deleti  on | exon1 | c.505delT | p.C169fs | . | . |
| CTC5 | 11 | 8751924 | TGGGGA  GCTG | T | 0.222 | NM_213618 | ST5 | nonframeshift_de  letion | exon3 | c.904_912del | p.302_304del | . | . |
| CTC5 | 11 | 9191485 | CT | C | 0.6 | NM_001243254 | DENND5A | frameshift_deleti  on | exon10 | c.2068delA | p.R690fs | . | . |
| CTC5 | 11 | 9834104 | A | AT | 0.235 | NM_030962 | SBF2 | frameshift_inserti  on | exon30 | c.4129_4130ins  A | p.L1377fs | . | . |
| CTC5 | 11 | 10789533 | G | GA | 0.276 | NM_014633 | CTR9 | frameshift_inserti  on | exon14 | c.1868dupA | p.E623fs | . | . |
| CTC5 | 11 | 12316039 | AG | A | 0.75 | NM_032867 | MICALCL | frameshift_deleti  on | exon3 | c.1062delG | p.K354fs | . | . |
| CTC5 | 11 | 14501217 | TC | T | 0.75 | NM_001144061 | COPB1 | frameshift_deleti  on | exon11 | c.1255delG | p.D419fs | . | . |
| CTC5 | 11 | 17618533 | TG | T | 0.256 | NM_001277269 | OTOG | frameshift_deleti  on | exon29 | c.3698delG | p.W1233fs | . | . |
| CTC5 | 11 | 17650833 | AC | A | 0.32 | NM_001277269 | OTOG | frameshift_deleti  on | exon39 | c.6719delC | p.T2240fs | . | . |
| CTC5 | 11 | 18127530 | A | ACCTC  CT | 1 | NM_138421 | SAAL1 | nonframeshift_in  sertion | exon1 | c.58_59insAGG  AGG | p.V20delinsEEV | . | . |
| CTC5 | 11 | 18127548 | T | TCCTCC  A | 0.981 | NM_138421 | SAAL1 | nonframeshift_in  sertion | exon1 | c.40_41insTGG  AGG | p.K14delinsME  E | . | . |
| CTC5 | 11 | 18472559 | TG | T | 0.7 | NM_002301 | LDHC | frameshift_deleti  on | exon8 | c.885delG | p.L295fs | . | . |
| CTC5 | 11 | 19077286 | AC | A | 0.8 | NM_054030 | MRGPRX2 | frameshift_deleti  on | exon2 | c.663delG | p.L221fs | . | . |
| CTC5 | 11 | 19955425 | AG | A | 0.4 | NM_001111018 | NAV2 | frameshift_deleti  on | exon7 | c.1444delG | p.G482fs | . | . |
| CTC5 | 11 | 26538406 | TA | T | 0.394 | NM_031418 | ANO3 | frameshift_deleti  on | exon6 | c.625delA | p.K209fs | . | . |
| CTC5 | 11 | 26558963 | AC | A | 0.292 | NM_031418 | ANO3 | frameshift_deleti  on | exon10 | c.988delC | p.L330fs | . | . |
| CTC5 | 11 | 27390578 | A | AT | 0.375 | NM_018490 | LGR4 | frameshift_inserti  on | exon18 | c.1691_1692ins  A | p.F564fs | . | . |
| CTC5 | 11 | 27434348 | A | AC | 0.283 | NM_018490 | LGR4 | frameshift_inserti  on | exon2 | c.244_245insG | p.F82fs | . | . |
| CTC5 | 11 | 27679720 | TC | T | 0.218 | NM_170735 | BDNF | frameshift_deleti  on | exon1 | c.391delG | p.D131fs | . | . |
| CTC5 | 11 | 30033286 | CGG | C | 0.782 | NM_002233 | KCNA4 | frameshift_deleti  on | exon2 | c.938_939del | p.S313fs | . | . |
| CTC5 | 11 | 31784997 | TTA | T | 0.136 | NM_001288725 | ELP4 | frameshift_deleti  on | exon10 | c.1186_1187del | p.Y396fs | . | . |
| CTC5 | 11 | 33566554 | AT | A | 0.4 | NM_012194 | KIAA1549L | frameshift_deleti  on | exon2 | c.2125delT | p.F709fs | . | . |
| CTC5 | 11 | 36422725 | C | CT | 0.217 | NM_001160169 | PRR5L | frameshift_inserti  on | exon1 | c.55dupT | p.S18fs | . | . |
| CTC5 | 11 | 40137305 | C | CA | 0.5 | NM_020929 | LRRC4C | frameshift_inserti  on | exon5 | c.537_538insT | p.G180fs | . | . |
| CTC5 | 11 | 43515356 | TC | T | 0.667 | NM_018259 | TTC17 | frameshift_deleti  on | exon24 | c.3329delC | p.S1110fs | . | . |
| CTC5 | 11 | 45249326 | CA | C | 0.591 | NM_001256695 | PRDM11 | frameshift_deleti  on | exon8 | c.3151delA | p.K1051fs | . | . |
| CTC5 | 11 | 45927270 | T | TA | 0.75 | NM_005456 | MAPK8IP1 | frameshift_inserti  on | exon12 | c.2135dupA | p.X712delinsX | . | . |
| CTC5 | 11 | 46724651 | GC | G | 0.882 | NM_001184751 | ZNF408 | frameshift_deleti  on | exon4 | c.487delC | p.P163fs | . | . |
| CTC5 | 11 | 46724716 | CAGTGG  TGACAG | C | 1 | NM_001184751 | ZNF408 | nonframeshift_de  letion | exon4 | c.552_563del | p.184_188del | . | . |
| CTC5 | 11 | 47238503 | AC | A | 0.222 | NM_000107 | DDB2 | frameshift_deleti  on | exon3 | c.360delC | p.H120fs | . | . |
| CTC5 | 11 | 47441891 | TG | T | 0.714 | NM_002804 | PSMC3 | frameshift_deleti  on | exon10 | c.1050delC | p.R350fs | . | . |
| CTC5 | 11 | 47767836 | CTCT | C | 0.2 | NM_015308 | FNBP4 | nonframeshift_de  letion | exon7 | c.1014_1016del | p.338_339del | . | . |
| CTC5 | 11 | 48238560 | GT | G | 0.822 | NM_001005470 | OR4B1 | frameshift_deleti  on | exon1 | c.200delT | p.V67fs | . | . |
| CTC5 | 11 | 48285570 | TCA | T | 0.444 | NM_001004726 | OR4X1 | frameshift_deleti  on | exon1 | c.159_160del | p.L53fs | . | . |
| CTC5 | 11 | 48285981 | CCTT | C | 1 | NM_001004726 | OR4X1 | nonframeshift_de  letion | exon1 | c.570_572del | p.190_191del | . | . |
| CTC5 | 11 | 48286317 | GA | G | 0.571 | NM_001004726 | OR4X1 | frameshift_deleti  on | exon1 | c.906delA | p.G302fs | . | . |
| CTC5 | 11 | 48510904 | GC | G | 0.185 | NM_001005512 | OR4A47 | stopgain | exon1 | c.561delC | p.C187X | . | . |
| CTC5 | 11 | 48510999 | A | AT | 0.222 | NM_001005512 | OR4A47 | frameshift_inserti on | exon1 | c.656dupT | p.I219fs | . | . |
| CTC5 | 11 | 55419216 | GT | G | 0.227 | NM_001004059 | OR4S2 | stopgain | exon1 | c.838delT | p.L280X | . | . |
| CTC5 | 11 | 57087882 | TG | T | 0.875 | NM_033396 | TNKS1BP1 | frameshift_deleti on | exon3 | c.398delC | p.P133fs | . | . |
| CTC5 | 11 | 57886239 | CA | C | 0.3 | NM_001005211 | OR9I1 | stopgain | exon1 | c.677delT | p.L226X | . | . |
| CTC5 | 11 | 57947416 | CCTT | C | 0.66 | NM_001005212 | OR9Q1 | nonframeshift_de letion | exon3 | c.501_503del | p.167_168del | . | . |
| CTC5 | 11 | 57995878 | AG | A | 0.778 | NM_001004471 | OR10Q1 | frameshift_deleti  on | exon1 | c.469delC | p.L157fs | . | . |
| CTC5 | 11 | 58893009 | TC | T | 0.217 | NM_001142704 | FAM111B | frameshift_deleti  on | exon2 | c.1350delC | p.F450fs | . | . |
| CTC5 | 11 | 58967196 | C | CA | 0.36 | NM_015177 | DTX4 | frameshift_inserti  on | exon8 | c.1576_1577ins  A | p.R526fs | . | . |
| CTC5 | 11 | 58978300 | CG | C | 0.3 | NM_001039396 | MPEG1 | frameshift_deleti  on | exon1 | c.2038delC | p.R680fs | . | . |

| CTC5 | 11 | 61026295 | CT | C | 0.5 | NM_152718 | VWCE | frameshift_deleti  on | exon20 | c.2719delA | p.S907fs | . | . |
| --- | --- | --- | --- | --- | --- | --- | --- | --- | --- | --- | --- | --- | --- |
| CTC5 | 11 | 61071418 | C | CA | 0.163 | NM_001923 | DDB1 | frameshift_inserti  on | exon22 | c.2750_2751ins  T | p.K917fs | . | . |
| CTC5 | 11 | 61539012 | TC | T | 0.955 | NM_001127392 | MYRF | frameshift_deleti  on | exon6 | c.782delC | p.S261fs | . | . |
| CTC5 | 11 | 61572165 | TC | T | 0.233 | NM_013402 | FADS1 | frameshift_deleti  on | exon7 | c.1044delG | p.K348fs | . | . |
| CTC5 | 11 | 62290651 | CG | C | 0.747 | NM_001620 | AHNAK | stopgain | exon5 | c.11237delC | p.S3746X | . | . |
| CTC5 | 11 | 62296572 | TG | T | 0.637 | NM_001620 | AHNAK | frameshift_deleti on | exon5 | c.5316delC | p.P1772fs | . | . |
| CTC5 | 11 | 62349011 | TGTC | T | 0.19 | NM_022830 | TUT1 | nonframeshift_de  letion | exon3 | c.547_549del | p.183_183del | . | . |
| CTC5 | 11 | 62417239 | GA | G | 1 | NM_030628 | INTS5 | frameshift_deleti  on | exon2 | c.312delT | p.R104fs | . | . |
| CTC5 | 11 | 63594499 | AC | A | 0.359 | NM_138471 | C11orf84 | frameshift_deleti  on | exon6 | c.1035delC | p.H345fs | . | . |
| CTC5 | 11 | 63974947 | TG | T | 0.291 | NM_031471 | FERMT3 | frameshift_deleti  on | exon2 | c.112delG | p.G38fs | . | . |
| CTC5 | 11 | 64519098 | CA | C | 0.773 | NM_001164716 | PYGM | frameshift_deleti  on | exon13 | c.1533delT | p.F511fs | . | . |
| CTC5 | 11 | 64519435 | GT | G | 0.273 | NM_001164716 | PYGM | frameshift_deleti  on | exon12 | c.1464delA | p.R488fs | . | . |
| CTC5 | 11 | 64595197 | CA | C | 0.224 | NM_017525 | CDC42BPG | frameshift_deleti  on | exon31 | c.4025delT | p.V1342fs | . | . |
| CTC5 | 11 | 64622824 | AG | A | 0.68 | NM_006795 | EHD1 | frameshift_deleti  on | exon4 | c.1049delC | p.P350fs | . | . |
| CTC5 | 11 | 64674893 | GT | G | 0.571 | NM_015104 | ATG2A | frameshift_deleti  on | exon19 | c.2665delA | p.T889fs | . | . |
| CTC5 | 11 | 64700349 | AG | A | 0.209 | NM_006244 | PPP2R5B | frameshift_deleti  on | exon12 | c.1230delG | p.K410fs | . | . |
| CTC5 | 11 | 64847165 | GT | G | 0.312 | NM_080668 | CDCA5 | frameshift_deleti  on | exon5 | c.337delA | p.T113fs | . | . |
| CTC5 | 11 | 65351161 | TG | T | 0.385 | NM_001099409 | EHBP1L1 | frameshift_deleti  on | exon9 | c.3019delG | p.G1007fs | . | . |
| CTC5 | 11 | 65351192 | TC | T | 0.968 | NM_001099409 | EHBP1L1 | frameshift_deleti  on | exon9 | c.3050delC | p.S1017fs | . | . |
| CTC5 | 11 | 65416785 | GCC | G | 0.5 | NM_006747 | SIPA1 | frameshift_deleti  on | exon10 | c.2360_2361del | p.A787fs | . | . |
| CTC5 | 11 | 65634392 | GA | G | 0.692 | NM_016938 | EFEMP2 | frameshift_deleti  on | exon11 | c.1328delT | p.F443fs | . | . |
| CTC5 | 11 | 66243215 | GC | G | 0.345 | NM_001243136 | PELI3 | frameshift_deleti  on | exon6 | c.667delC | p.P223fs | . | . |
| CTC5 | 11 | 66366936 | TG | T | 0.46 | NM_005125 | CCS | frameshift_deleti  on | exon4 | c.258delG | p.L86fs | . | . |
| CTC5 | 11 | 66444216 | TC | T | 1 | NM_001286135 | RBM4B | frameshift_deleti  on | exon2 | c.334delG | p.D112fs | . | . |
| CTC5 | 11 | 67166423 | GC | G | 0.625 | NM_206873 | PPP1CA | frameshift_deleti  on | exon4 | c.602delG | p.C201fs | . | . |
| CTC5 | 11 | 68772928 | TG | T | 0.248 | NM_001098515 | MRGPRF | frameshift_deleti  on | exon3 | c.849delC | p.N283fs | . | . |
| CTC5 | 11 | 69063812 | CT | C | 0.295 | NM_138768 | MYEOV | frameshift_deleti  on | exon3 | c.896delT | p.L299fs | . | . |
| CTC5 | 11 | 71189473 | CG | C | 0.404 | NM_018161 | NADSYN1 | frameshift_deleti  on | exon10 | c.832delG | p.V278fs | . | . |
| CTC5 | 11 | 71192974 | GT | G | 1 | NM_018161 | NADSYN1 | frameshift_deleti  on | exon13 | c.1054delT | p.F352fs | . | . |
| CTC5 | 11 | 71821178 | TCTC | T | 0.231 | NM_001278490 | ANAPC15 | nonframeshift_de  letion | exon4 | c.271_273del | p.91_91del | . | . |
| CTC5 | 11 | 73670655 | GA | G | 1 | NM_153614 | DNAJB13 | frameshift_deleti  on | exon3 | c.290delA | p.E97fs | . | . |
| CTC5 | 11 | 74013461 | TG | T | 0.286 | NM_001288748 | P4HA3 | frameshift_deleti  on | exon3 | c.519delC | p.P173fs | . | . |
| CTC5 | 11 | 74324029 | TA | T | 0.6 | NM_006591 | POLD3 | frameshift_deleti  on | exon5 | c.367delA | p.K123fs | . | . |
| CTC5 | 11 | 75188718 | GC | G | 0.872 | NM_030792 | GDPD5 | frameshift_deleti  on | exon3 | c.62delG | p.G21fs | . | . |
| CTC5 | 11 | 75439945 | TG | T | 0.691 | NM_025098 | MOGAT2 | frameshift_deleti  on | exon5 | c.762delG | p.M254fs | . | . |
| CTC5 | 11 | 76063184 | GA | G | 0.667 | NM_004705 | PRKRIR | frameshift_deleti  on | exon5 | c.1009delT | p.S337fs | . | . |
| CTC5 | 11 | 76237631 | TA | T | 0.261 | NM_020193 | C11orf30 | frameshift_deleti  on | exon13 | c.1948delA | p.K650fs | . | . |
| CTC5 | 11 | 76506672 | CCTG | C | 0.171 | NM_001258210 | TSKU | nonframeshift_de  letion | exon2 | c.13_15del | p.5_5del | . | . |
| CTC5 | 11 | 77838466 | CA | C | 0.286 | NM_001007027 | ALG8 | frameshift_deleti  on | exon2 | c.111delT | p.F37fs | . | . |
| CTC5 | 11 | 82643975 | AT | A | 0.6 | NM_145018 | C11orf82 | frameshift_deleti  on | exon6 | c.1596delT | p.Y532fs | . | . |
| CTC5 | 11 | 85374859 | AT | A | 0.981 | NM_001039618 | CREBZF | frameshift_deleti  on | exon1 | c.1060delA | p.M354fs | . | . |
| CTC5 | 11 | 89951333 | TG | T | 0.25 | NM_001144073 | CHORDC1 | frameshift_deleti  on | exon2 | c.83delC | p.P28fs | . | . |
| CTC5 | 11 | 92086932 | TG | T | 0.8 | NM_001008781 | FAT3 | frameshift_deleti  on | exon1 | c.1655delG | p.W552fs | . | . |
| CTC5 | 11 | 92573806 | TC | T | 0.491 | NM_001008781 | FAT3 | frameshift_deleti  on | exon17 | c.10448delC | p.S3483fs | . | . |
| CTC5 | 11 | 94040457 | TG | T | 0.738 | NM_001199206 | FOLR4 | stopgain | exon3 | c.476delG | p.W159X | . | . |
| CTC5 | 11 | 95712406 | CA | C | 0.245 | NM_032427 | MAML2 | frameshift_deleti on | exon5 | c.3176delT | p.L1059fs | . | . |
| CTC5 | 11 | 96124107 | TG | T | 0.5 | NM_003772 | JRKL | frameshift_deleti  on | exon1 | c.295delG | p.G99fs | . | . |
| CTC5 | 11 | 101353895 | AT | A | 0.2 | NM_004621 | TRPC6 | frameshift_deleti  on | exon5 | c.1294delA | p.M432fs | . | . |
| CTC5 | 11 | 102248443 | TA | T | 0.833 | NM_001166 | BIRC2 | frameshift_deleti  on | exon7 | c.1584delA | p.L528fs | . | . |
| CTC5 | 11 | 102564789 | GT | G | 0.727 | NM_022122 | MMP27 | frameshift_deleti  on | exon8 | c.1040delA | p.N347fs | . | . |
| CTC5 | 11 | 102993584 | CT | C | 0.276 | NM_001080463 | DYNC2H1 | frameshift_deleti  on | exon11 | c.1517delT | p.L506fs | . | . |
| CTC5 | 11 | 103814180 | C | CT | 0.667 | NM_025208 | PDGFD | frameshift_inserti  on | exon5 | c.771dupA | p.V258fs | . | . |
| CTC5 | 11 | 106810872 | CA | C | 0.229 | NM_000855 | GUCY1A2 | frameshift_deleti  on | exon4 | c.519delT | p.F173fs | . | . |
| CTC5 | 11 | 108381286 | AT | A | 0.598 | NM_015065 | EXPH5 | frameshift_deleti  on | exon6 | c.4947delA | p.K1649fs | . | . |
| CTC5 | 11 | 108382006 | AT | A | 0.314 | NM_015065 | EXPH5 | frameshift_deleti  on | exon6 | c.4227delA | p.K1409fs | . | . |
| CTC5 | 11 | 108385511 | CTG | C | 0.32 | NM_015065 | EXPH5 | frameshift_deleti  on | exon6 | c.721_722del | p.Q241fs | . | . |
| CTC5 | 11 | 110035786 | AT | A | 0.419 | NM_033390 | ZC3H12C | frameshift_deleti  on | exon6 | c.1977delT | p.N659fs | . | . |
| CTC5 | 11 | 111706968 | GT | G | 0.767 | NM_001077691 | ALG9 | frameshift_deleti  on | exon13 | c.1008delA | p.K336fs | . | . |

| CTC5 | 11 | 113288843 | TC | T | 0.273 | NM_000795 | DRD2 | stopgain | exon3 | c.300delG | p.W100X | . | . |
| --- | --- | --- | --- | --- | --- | --- | --- | --- | --- | --- | --- | --- | --- |
| CTC5 | 11 | 115080311 | ATGG | A | 0.25 | NM_014333 | CADM1 | nonframeshift_de letion | exon8 | c.1058_1060del | p.353_354del | . | . |
| CTC5 | 11 | 116627899 | TA | T | 0.245 | NM_001159736 | BUD13 | frameshift_deleti  on | exon9 | c.1326delT | p.F442fs | . | . |
| CTC5 | 11 | 116661432 | AG | A | 0.344 | NM_001166598 | APOA5 | frameshift_deleti  on | exon4 | c.512delC | p.A171fs | . | . |
| CTC5 | 11 | 117109403 | TC | T | 0.683 | NM_001077239 | RNF214 | frameshift_deleti  on | exon3 | c.195delC | p.V65fs | . | . |
| CTC5 | 11 | 117109405 | CA | C | 0.672 | NM_001077239 | RNF214 | frameshift_deleti  on | exon3 | c.197delA | p.H66fs | . | . |
| CTC5 | 11 | 117310037 | GC | G | 1 | NM_020693 | DSCAML1 | frameshift_deleti  on | exon23 | c.4268delG | p.C1423fs | . | . |
| CTC5 | 11 | 117864115 | G | GA | 0.25 | NM_001558 | IL10RA | frameshift_inserti  on | exon4 | c.528dupA | p.G176fs | . | . |
| CTC5 | 11 | 117870124 | TA | T | 1 | NM_001558 | IL10RA | frameshift_deleti  on | exon7 | c.1506delA | p.L502fs | . | . |
| CTC5 | 11 | 118184492 | TG | T | 0.34 | NM_000733 | CD3E | frameshift_deleti  on | exon7 | c.424delG | p.G142fs | . | . |
| CTC5 | 11 | 118372396 | CA | C | 0.278 | NM_001197104 | KMT2A | frameshift_deleti  on | exon26 | c.6330delA | p.S2110fs | . | . |
| CTC5 | 11 | 118771620 | CA | C | 0.843 | NM_182557 | BCL9L | frameshift_deleti  on | exon6 | c.2831delT | p.L944fs | . | . |
| CTC5 | 11 | 118771793 | GC | G | 0.545 | NM_182557 | BCL9L | frameshift_deleti  on | exon6 | c.2658delG | p.R886fs | . | . |
| CTC5 | 11 | 119535677 | CCCT | C | 0.538 | NM_002855 | PVRL1 | nonframeshift_de  letion | exon6 | c.1331_1333del | p.444_445del | . | . |
| CTC5 | 11 | 120201007 | GT | G | 0.233 | NM_001198675 | TMEM136 | frameshift_deleti  on | exon2 | c.219delT | p.G73fs | . | . |
| CTC5 | 11 | 121028561 | AT | A | 0.32 | NM_005422 | TECTA | frameshift_deleti  on | exon13 | c.4318delT | p.F1440fs | . | . |
| CTC5 | 11 | 121476238 | C | CA | 0.625 | NM_003105 | SORL1 | frameshift_inserti  on | exon35 | c.4907dupA | p.H1636fs | . | . |
| CTC5 | 11 | 123516294 | CA | C | 0.923 | NM_018400 | SCN3B | frameshift_deleti  on | exon2 | c.219delT | p.L73fs | . | . |
| CTC5 | 11 | 124189857 | AG | A | 0.36 | NM_001002918 | OR8D2 | frameshift_deleti  on | exon1 | c.236delC | p.P79fs | . | . |
| CTC5 | 11 | 124440190 | AT | A | 0.261 | NM_001005194 | OR8A1 | frameshift_deleti  on | exon1 | c.227delT | p.M76fs | . | . |
| CTC5 | 11 | 124440401 | C | CT | 0.609 | NM_001005194 | OR8A1 | frameshift_inserti  on | exon1 | c.438dupT | p.P146fs | . | . |
| CTC5 | 11 | 124755096 | TG | T | 0.82 | NM_019055 | ROBO4 | frameshift_deleti  on | exon17 | c.2841delC | p.P947fs | . | . |
| CTC5 | 11 | 124793774 | AG | A | 0.326 | NM_152722 | HEPACAM | frameshift_deleti  on | exon3 | c.559delC | p.L187fs | . | . |
| CTC5 | 11 | 125452300 | A | AC | 1 | NM_001007277 | EI24 | frameshift_inserti  on | exon9 | c.733dupC | p.R244fs | . | . |
| CTC5 | 11 | 125547703 | C | CT | 0.389 | NM_001612 | ACRV1 | frameshift_inserti  on | exon2 | c.541dupA | p.S181fs | . | . |
| CTC5 | 11 | 125871629 | AG | A | 0.375 | NM_001243597 | CDON | frameshift_deleti  on | exon11 | c.2142delC | p.S714fs | . | . |
| CTC5 | 11 | 126073488 | AG | A | 0.941 | NM_001144827 | RPUSD4 | frameshift_deleti  on | exon7 | c.865delC | p.L289fs | . | . |
| CTC5 | 11 | 130059715 | CG | C | 0.692 | NM_021978 | ST14 | frameshift_deleti  on | exon5 | c.523delG | p.E175fs | . | . |
| CTC5 | 11 | 133807303 | TC | T | 0.9 | NM_001277285 | IGSF9B | frameshift_deleti  on | exon5 | c.646delG | p.E216fs | . | . |
| CTC5 | 11 | 134240954 | G | GA | 0.658 | NM_138342 | GLB1L2 | frameshift_inserti  on | exon13 | c.1269dupA | p.G423fs | . | . |
| CTC5 | 12 | 302486 | AG | A | 0.833 | NM_001206931 | SLC6A12 | frameshift_deleti  on | exon13 | c.1486delC | p.L496fs | . | . |
| CTC5 | 12 | 521060 | TC | T | 0.5 | NM_001130148 | CCDC77 | frameshift_deleti  on | exon2 | c.91delC | p.Q31fs | . | . |
| CTC5 | 12 | 659003 | TC | T | 0.8 | NM_173593 | B4GALNT3 | frameshift_deleti  on | exon10 | c.923delC | p.S308fs | . | . |
| CTC5 | 12 | 977748 | TG | T | 0.688 | NM_001184985 | WNK1 | frameshift_deleti  on | exon9 | c.2857delG | p.A953fs | . | . |
| CTC5 | 12 | 989147 | CT | C | 0.322 | NM_018979 | WNK1 | frameshift_deleti  on | exon11 | c.2783delT | p.L928fs | . | . |
| CTC5 | 12 | 2062323 | TTGC | T | 0.162 | NM_152640 | DCP1B | nonframeshift_de  letion | exon7 | c.780_782del | p.260_261del | . | . |
| CTC5 | 12 | 3806142 | TA | T | 1 | NM_001144958 | EFCAB4B | frameshift_deleti  on | exon4 | c.23delT | p.V8fs | . | . |
| CTC5 | 12 | 3806146 | CT | C | 1 | NM_001144958 | EFCAB4B | frameshift_deleti  on | exon4 | c.19delA | p.R7fs | . | . |
| CTC5 | 12 | 5721908 | G | GC | 0.409 | NM_001278596 | ANO2 | frameshift_inserti  on | exon20 | c.2015dupG | p.G672fs | . | . |
| CTC5 | 12 | 6125328 | CT | C | 0.571 | NM_000552 | VWF | frameshift_deleti  on | exon31 | c.5381delA | p.K1794fs | . | . |
| CTC5 | 12 | 6442941 | TG | T | 0.273 | NM_001065 | TNFRSF1A | frameshift_deleti  on | exon3 | c.283delC | p.H95fs | . | . |
| CTC5 | 12 | 6883869 | GC | G | 0.778 | NM_002286 | LAG3 | frameshift_deleti  on | exon4 | c.621delC | p.G207fs | . | . |
| CTC5 | 12 | 6924136 | CA | C | 0.484 | NM_000616 | CD4 | frameshift_deleti  on | exon5 | c.586delA | p.K196fs | . | . |
| CTC5 | 12 | 7045891 | ACAG | A | 0.286 | NM_001007026 | ATN1 | nonframeshift_de  letion | exon5 | c.1462_1464del | p.488_488del | . | . |
| CTC5 | 12 | 7261752 | TC | T | 1 | NM_016546 | C1RL | frameshift_deleti  on | exon1 | c.24delG | p.G8fs | . | . |
| CTC5 | 12 | 7362365 | CG | C | 0.264 | NM_000319 | PEX5 | frameshift_deleti  on | exon14 | c.1624delG | p.E542fs | . | . |
| CTC5 | 12 | 8083922 | TC | T | 1 | NM_006931 | SLC2A3 | frameshift_deleti  on | exon4 | c.428delG | p.G143fs | . | . |
| CTC5 | 12 | 8200557 | ACAG | A | 0.4 | NM_018416 | FOXJ2 | nonframeshift_de  letion | exon7 | c.898_900del | p.300_300del | . | . |
| CTC5 | 12 | 9090514 | TG | T | 0.24 | NM_004426 | PHC1 | frameshift_deleti  on | exon14 | c.2719delG | p.G907fs | . | . |
| CTC5 | 12 | 9098995 | G | GAAC | 0.364 | NM_001207024 | M6PR | nonframeshift_in  sertion | exon2 | c.5_6insGTT | p.F2delinsLF | . | . |
| CTC5 | 12 | 9147813 | TG | T | 0.328 | NM_005810 | KLRG1 | stopgain | exon3 | c.287delG | p.W96X | . | . |
| CTC5 | 12 | 9994445 | GTGT | G | 1 | NM_016523 | KLRF1 | nonframeshift_de letion | exon4 | c.373_375del | p.125_125del | . | . |
| CTC5 | 12 | 10228163 | AC | A | 0.185 | NM_016511 | CLEC1A | frameshift_deleti  on | exon4 | c.482delG | p.C161fs | . | . |
| CTC5 | 12 | 10868312 | TC | T | 0.8 | NM_001145426 | YBX3 | frameshift_deleti  on | exon4 | c.430delG | p.D144fs | . | . |
| CTC5 | 12 | 12022558 | AG | A | 1 | NM_001987 | ETV6 | frameshift_deleti  on | exon5 | c.665delG | p.R222fs | . | . |
| CTC5 | 12 | 12653541 | G | GA | 0.714 | NM_030640 | DUSP16 | frameshift_inserti  on | exon4 | c.442dupT | p.S148fs | . | . |
| CTC5 | 12 | 15070119 | TG | T | 0.344 | NM_152321 | ERP27 | frameshift_deleti  on | exon5 | c.568delC | p.Q190fs | . | . |

| CTC5 | 12 | 16397543 | GA | G | 1 | NM_001170798 | SLC15A5 | frameshift_deleti  on | exon4 | c.945delT | p.F315fs | . | . |
| --- | --- | --- | --- | --- | --- | --- | --- | --- | --- | --- | --- | --- | --- |
| CTC5 | 12 | 18435398 | GCCC | G | 0.837 | NM_001288772 | PIK3C2G | nonframeshift_de  letion | exon2 | c.384_386del | p.128_129del | . | . |
| CTC5 | 12 | 18466994 | GA | G | 0.714 | NM_001288772 | PIK3C2G | frameshift_deleti  on | exon6 | c.1134delA | p.R378fs | . | . |
| CTC5 | 12 | 21796930 | G | GA | 0.315 | NM_001174097 | LDHB | frameshift_inserti  on | exon4 | c.359dupT | p.F120fs | . | . |
| CTC5 | 12 | 26784827 | GT | G | 0.182 | NM_002223 | ITPR2 | frameshift_deleti  on | exon22 | c.2905delA | p.T969fs | . | . |
| CTC5 | 12 | 27623587 | TA | T | 0.667 | NM_001145010 | SMCO2 | frameshift_deleti  on | exon2 | c.24delA | p.L8fs | . | . |
| CTC5 | 12 | 27623623 | GC | G | 0.636 | NM_001145010 | SMCO2 | frameshift_deleti  on | exon2 | c.60delC | p.C20fs | . | . |
| CTC5 | 12 | 29469931 | TG | T | 0.25 | NM_001271784 | FAR2 | frameshift_deleti  on | exon8 | c.823delG | p.G275fs | . | . |
| CTC5 | 12 | 29617620 | GTT | G | 0.231 | NM_183378 | OVCH1 | frameshift_deleti  on | exon18 | c.1943_1944del | p.K648fs | . | . |
| CTC5 | 12 | 31862322 | GA | G | 0.6 | NM_001113402 | AMN1 | frameshift_deleti  on | exon2 | c.75delT | p.Y25fs | . | . |
| CTC5 | 12 | 41966888 | GA | G | 0.867 | NM_013377 | PDZRN4 | frameshift_deleti  on | exon8 | c.1534delA | p.K512fs | . | . |
| CTC5 | 12 | 45782035 | AC | A | 0.289 | NM_001025356 | ANO6 | frameshift_deleti  on | exon11 | c.1258delC | p.P420fs | . | . |
| CTC5 | 12 | 48370896 | G | GA | 0.233 | NM_033150 | COL2A1 | frameshift_inserti  on | exon46 | c.3108_3109ins  T | p.R1037fs | . | . |
| CTC5 | 12 | 48543193 | GA | G | 0.5 | NM_024095 | ASB8 | frameshift_deleti  on | exon4 | c.822delT | p.L274fs | . | . |
| CTC5 | 12 | 49428237 | GA | G | 0.523 | NM_003482 | KMT2D | frameshift_deleti  on | exon37 | c.10462delT | p.S3488fs | . | . |
| CTC5 | 12 | 49434894 | GC | G | 0.489 | NM_003482 | KMT2D | frameshift_deleti  on | exon31 | c.6658delG | p.A2220fs | . | . |
| CTC5 | 12 | 50039618 | GA | G | 0.256 | NM_198900 | FMNL3 | frameshift_deleti  on | exon25 | c.2910delT | p.P970fs | . | . |
| CTC5 | 12 | 50050608 | TG | T | 0.25 | NM_198900 | FMNL3 | frameshift_deleti  on | exon7 | c.635delC | p.P212fs | . | . |
| CTC5 | 12 | 50745054 | GCC | G | 0.25 | NM_001145475 | FAM186A | frameshift_deleti  on | exon4 | c.5559_5560del | p.W1853fs | . | . |
| CTC5 | 12 | 50749438 | CT | C | 1 | NM_001145475 | FAM186A | frameshift_deleti  on | exon4 | c.1176delA | p.K392fs | . | . |
| CTC5 | 12 | 51072477 | C | CA | 0.923 | NM_173602 | DIP2B | frameshift_inserti  on | exon8 | c.932_933insA | p.P311fs | . | . |
| CTC5 | 12 | 51446028 | AG | A | 0.545 | NM_001243689 | LETMD1 | frameshift_deleti  on | exon3 | c.429delG | p.E143fs | . | . |
| CTC5 | 12 | 51447613 | C | CT | 0.9 | NM_001243689 | LETMD1 | frameshift_inserti  on | exon4 | c.483dupT | p.P161fs | . | . |
| CTC5 | 12 | 51470309 | C | CT | 0.25 | NM_030809 | CSRNP2 | frameshift_inserti  on | exon2 | c.35dupA | p.K12fs | . | . |
| CTC5 | 12 | 52715043 | C | CA | 1 | NM_002282 | KRT83 | frameshift_inserti  on | exon1 | c.76_77insT | p.R26fs | . | . |
| CTC5 | 12 | 52963720 | G | GT | 0.304 | NM_175053 | KRT74 | frameshift_inserti  on | exon6 | c.1056dupA | p.H353fs | . | . |
| CTC5 | 12 | 53818979 | T | TG | 0.944 | NM_001164690 | AMHR2 | frameshift_inserti  on | exon4 | c.456dupG | p.L152fs | . | . |
| CTC5 | 12 | 53876739 | TC | T | 0.647 | NM_001193511 | MAP3K12 | frameshift_deleti  on | exon11 | c.1847delG | p.G616fs | . | . |
| CTC5 | 12 | 53900858 | GA | G | 0.369 | NM_003717 | NPFF | frameshift_deleti  on | exon2 | c.170delT | p.L57fs | . | . |
| CTC5 | 12 | 54405048 | GA | G | 0.863 | NM_022658 | HOXC8 | frameshift_deleti  on | exon2 | c.613delA | p.K205fs | . | . |
| CTC5 | 12 | 55615276 | TA | T | 0.291 | NM_001005280 | OR10A7 | frameshift_deleti  on | exon1 | c.469delA | p.M157fs | . | . |
| CTC5 | 12 | 56115242 | GC | G | 0.778 | NM_001199771 | RDH5 | frameshift_deleti  on | exon2 | c.275delC | p.A92fs | . | . |
| CTC5 | 12 | 56221117 | TA | T | 0.28 | NM_032364 | DNAJC14 | frameshift_deleti  on | exon2 | c.1325delT | p.L442fs | . | . |
| CTC5 | 12 | 56348096 | AC | A | 0.429 | NM_001200053 | PMEL | frameshift_deleti  on | exon9 | c.1629delG | p.Q543fs | . | . |
| CTC5 | 12 | 56351300 | CA | C | 0.966 | NM_001200053 | PMEL | frameshift_deleti  on | exon4 | c.528delT | p.F176fs | . | . |
| CTC5 | 12 | 56733251 | TC | T | 0.412 | NM_016584 | IL23A | frameshift_deleti  on | exon2 | c.205delC | p.P69fs | . | . |
| CTC5 | 12 | 56993571 | TCA | T | 0.35 | NM_013449 | BAZ2A | frameshift_deleti  on | exon26 | c.5091_5092del | p.C1697fs | . | . |
| CTC5 | 12 | 57011273 | A | AG | 0.46 | NM_013449 | BAZ2A | frameshift_inserti  on | exon2 | c.47dupC | p.P16fs | . | . |
| CTC5 | 12 | 57065540 | CT | C | 0.226 | NM_001282601 | PTGES3 | frameshift_deleti  on | exon4 | c.277delA | p.R93fs | . | . |
| CTC5 | 12 | 57625295 | TG | T | 0.571 | NM_001166356 | SHMT2 | frameshift_deleti  on | exon3 | c.264delG | p.L88fs | . | . |
| CTC5 | 12 | 57662237 | GA | G | 0.317 | NM_014925 | R3HDM2 | frameshift_deleti  on | exon16 | c.1836delT | p.V612fs | . | . |
| CTC5 | 12 | 57674204 | TTGC | T | 0.364 | NM_014925 | R3HDM2 | nonframeshift_de  letion | exon12 | c.1236_1238del | p.412_413del | . | . |
| CTC5 | 12 | 58004089 | GC | G | 0.697 | NM_001111270 | ARHGEF25 | frameshift_deleti  on | exon1 | c.91delC | p.Q31fs | . | . |
| CTC5 | 12 | 58008381 | AC | A | 0.259 | NM_182947 | ARHGEF25 | frameshift_deleti  on | exon8 | c.810delC | p.Y270fs | . | . |
| CTC5 | 12 | 58112020 | GGCA | G | 0.167 | NM_001261421 | OS9 | nonframeshift_de  letion | exon10 | c.1131_1133del | p.377_378del | . | . |
| CTC5 | 12 | 58201449 | TA | T | 0.455 | NM_006576 | AVIL | frameshift_deleti  on | exon11 | c.1255delT | p.Y419fs | . | . |
| CTC5 | 12 | 58347400 | TA | T | 0.721 | NM_033276 | XRCC6BP1 | frameshift_deleti  on | exon5 | c.466delA | p.N156fs | . | . |
| CTC5 | 12 | 58350585 | CT | C | 0.333 | NM_033276 | XRCC6BP1 | frameshift_deleti  on | exon6 | c.654delT | p.P218fs | . | . |
| CTC5 | 12 | 66563712 | CG | C | 0.342 | NM_001282606 | TMBIM4 | frameshift_deleti  on | exon1 | c.19delC | p.R7fs | . | . |
| CTC5 | 12 | 68051382 | GC | G | 0.364 | NM_003583 | DYRK2 | frameshift_deleti  on | exon2 | c.477delC | p.S159fs | . | . |
| CTC5 | 12 | 69207352 | CT | C | 0.516 | NM_001145337 | MDM2 | frameshift_deleti  on | exon3 | c.101delT | p.L34fs | . | . |
| CTC5 | 12 | 72092834 | TG | T | 1 | NM_018279 | TMEM19 | frameshift_deleti  on | exon5 | c.793delG | p.G265fs | . | . |
| CTC5 | 12 | 78512028 | AG | A | 0.412 | NM_001024383 | NAV3 | frameshift_deleti  on | exon14 | c.2992delG | p.G998fs | . | . |
| CTC5 | 12 | 79747361 | CA | C | 0.846 | NM_001135806 | SYT1 | frameshift_deleti  on | exon8 | c.891delA | p.A297fs | . | . |
| CTC5 | 12 | 81205372 | CT | C | 1 | NM_004664 | LIN7A | frameshift_deleti  on | exon5 | c.573delA | p.K191fs | . | . |
| CTC5 | 12 | 81839335 | CT | C | 0.316 | NM_001220477 | PPFIA2 | frameshift_deleti  on | exon3 | c.347delA | p.K116fs | . | . |
| CTC5 | 12 | 85277641 | TC | T | 0.207 | NM_001146335 | SLC6A15 | frameshift_deleti  on | exon4 | c.431delG | p.G144fs | . | . |
| CTC5 | 12 | 94697651 | AT | A | 0.8 | NM_005761 | PLXNC1 | frameshift_deleti  on | exon29 | c.4507delT | p.F1503fs | . | . |

| CTC5 | 12 | 96284666 | T | TC | 0.885 | NM_182496 | CCDC38 | frameshift_inserti  on | exon9 | c.814dupG | p.E272fs | . | . |
| --- | --- | --- | --- | --- | --- | --- | --- | --- | --- | --- | --- | --- | --- |
| CTC5 | 12 | 99052948 | AG | A | 0.981 | NM_001160 | APAF1 | frameshift_deleti  on | exon5 | c.505delG | p.G169fs | . | . |
| CTC5 | 12 | 99100355 | AT | A | 0.328 | NM_001160 | APAF1 | frameshift_deleti  on | exon18 | c.2527delT | p.F843fs | . | . |
| CTC5 | 12 | 101018175 | C | CG | 0.636 | NM_174942 | GAS2L3 | frameshift_inserti  on | exon10 | c.1593dupG | p.T531fs | . | . |
| CTC5 | 12 | 101739399 | TC | T | 0.333 | NM_014503 | UTP20 | frameshift_deleti  on | exon37 | c.4674delC | p.F1558fs | . | . |
| CTC5 | 12 | 104081985 | T | TA | 0.19 | NM_017564 | STAB2 | stopgain | exon28 | c.3037_3038ins  A | p.W1013_I1014  delinsX | . | . |
| CTC5 | 12 | 109017650 | GGAGTG  GTCTGT GCCTCC GTGGGC | G | 0.318 | NM_001206609 | SELPLG | nonframeshift_de letion | exon2 | c.452_481del | p.151_161del | . | . |
| CTC5 | 12 | 109052643 | TA | T | 0.986 | NM_001105237 | CORO1C | frameshift_deleti  on | exon5 | c.659delT | p.I220fs | . | . |
| CTC5 | 12 | 109937586 | TG | T | 0.688 | NM_130466 | UBE3B | frameshift_deleti  on | exon12 | c.1090delG | p.G364fs | . | . |
| CTC5 | 12 | 110252471 | T | TC | 0.325 | NM_001177428 | TRPV4 | frameshift_inserti  on | exon1 | c.130dupG | p.D44fs | . | . |
| CTC5 | 12 | 112130538 | TC | T | 1 | NM_001136538 | ACAD10 | frameshift_deleti  on | exon2 | c.26delC | p.S9fs | . | . |
| CTC5 | 12 | 112321506 | GC | G | 0.444 | NM_003668 | MAPKAPK5 | frameshift_deleti  on | exon9 | c.783delC | p.G261fs | . | . |
| CTC5 | 12 | 113355541 | T | TA | 0.179 | NM_002534 | OAS1 | frameshift_inserti  on | exon5 | c.1074_1075ins  A | p.P358fs | . | . |
| CTC5 | 12 | 113707592 | GC | G | 0.364 | NM_017901 | TPCN1 | frameshift_deleti  on | exon7 | c.691delC | p.P231fs | . | . |
| CTC5 | 12 | 116421316 | TA | T | 1 | NM_015335 | MED13L | frameshift_deleti  on | exon21 | c.4560delT | p.D1520fs | . | . |
| CTC5 | 12 | 117187890 | CG | C | 0.5 | NM_001109903 | RNFT2 | frameshift_deleti  on | exon4 | c.329delG | p.R110fs | . | . |
| CTC5 | 12 | 117204665 | CG | C | 0.912 | NM_001109903 | RNFT2 | frameshift_deleti  on | exon6 | c.675delG | p.A225fs | . | . |
| CTC5 | 12 | 117922236 | AC | A | 0.471 | NM_173598 | KSR2 | frameshift_deleti  on | exon16 | c.2347delG | p.V783fs | . | . |
| CTC5 | 12 | 118481092 | AC | A | 0.255 | NM_001278557 | WSB2 | frameshift_deleti  on | exon3 | c.323delG | p.G108fs | . | . |
| CTC5 | 12 | 118506327 | TTCCTCC | T | 1 | NM_019086 | VSIG10 | nonframeshift_de  letion | exon8 | c.1416_1421del | p.472_474del | . | . |
| CTC5 | 12 | 118506327 | TTCCTCC  TCC | T | 1 | NM_019086 | VSIG10 | nonframeshift_de  letion | exon8 | c.1413_1421del | p.471_474del | . | . |
| CTC5 | 12 | 121465533 | CA | C | 0.917 | NM_003733 | OASL | frameshift_deleti  on | exon4 | c.744delT | p.T248fs | . | . |
| CTC5 | 12 | 121678327 | C | CTTT | 0.95 | NM_001270486 | CAMKK2 | nonframeshift_in  sertion | exon16 | c.1614_1615ins  AAA | p.G539delinsK  G | . | . |
| CTC5 | 12 | 121678327 | C | CTTTT | 0.95 | NM_001270486 | CAMKK2 | frameshift_inserti  on | exon16 | c.1614_1615ins  AAAA | p.G539fs | . | . |
| CTC5 | 12 | 121678338 | T | TTTG | 0.818 | NM_001270486 | CAMKK2 | nonframeshift_in  sertion | exon16 | c.1603_1604ins  CAA | p.K535delinsTK | . | . |
| CTC5 | 12 | 121678338 | T | TTTTA | 0.818 | NM_001270486 | CAMKK2 | frameshift_inserti  on | exon16 | c.1603_1604ins  TAAA | p.K535fs | . | . |
| CTC5 | 12 | 121882070 | CT | C | 0.259 | NM_001005366 | KDM2B | frameshift_deleti  on | exon16 | c.2102delA | p.K701fs | . | . |
| CTC5 | 12 | 122284976 | CT | C | 1 | NM_002150 | HPD | frameshift_deleti  on | exon10 | c.740delA | p.K247fs | . | . |
| CTC5 | 12 | 122359397 | G | GGAGG  AGGAG | 1 | NM_001178003 | WDR66 | nonframeshift_in  sertion | exon2 | c.186_187insG  AGGAGGAGGA | p.G62delinsGE  EEEK | . | . |
| CTC5 | 12 | 122359397 | G | GGAGG  AGGAG | 1 | NM_001178003 | WDR66 | nonframeshift_in  sertion | exon2 | c.186_187insG  AGGAGGAGAA | p.G62delinsGE  EEK | . | . |
| CTC5 | 12 | 123252038 | GA | G | 0.571 | NM_003677 | DENR | frameshift_deleti  on | exon6 | c.300delA | p.G100fs | . | . |
| CTC5 | 12 | 124333289 | AT | A | 0.826 | NM_207437 | DNAH10 | frameshift_deleti  on | exon33 | c.5609delT | p.I1870fs | . | . |
| CTC5 | 12 | 124366303 | A | AG | 0.411 | NM_207437 | DNAH10 | frameshift_inserti  on | exon50 | c.8413dupG | p.V2804fs | . | . |
| CTC5 | 12 | 124798843 | AC | A | 1 | NM_181709 | FAM101A | frameshift_deleti  on | exon3 | c.181delC | p.P61fs | . | . |
| CTC5 | 12 | 124812123 | G | GC | 1 | NM_001077261 | NCOR2 | frameshift_inserti  on | exon46 | c.6984dupG | p.L2329fs | . | . |
| CTC5 | 12 | 124821585 | GGC | G | 0.122 | NM_001077261 | NCOR2 | frameshift_deleti  on | exon39 | c.5797_5798del | p.A1933fs | . | . |
| CTC5 | 12 | 124824721 | C | CGCCG  CTGCT | 1 | NM_001077261 | NCOR2 | nonframeshift_in  sertion | exon38 | c.5487_5488ins  AGCAGCGGC | p.G1830delinsS  SGG | . | . |
| CTC5 | 12 | 124840098 | GC | G | 0.818 | NM_001077261 | NCOR2 | frameshift_deleti  on | exon25 | c.3230delG | p.G1077fs | . | . |
| CTC5 | 12 | 124887058 | G | GGCTG  CT | 0.913 | NM_001077261 | NCOR2 | nonframeshift_in  sertion | exon16 | c.1528_1529ins  AGCAGC | p.P510delinsQ  QP | . | . |
| CTC5 | 12 | 124887058 | G | GGCT | 0.913 | NM_001077261 | NCOR2 | nonframeshift_in  sertion | exon16 | c.1528_1529ins  AGC | p.P510delinsQ  P | . | . |
| CTC5 | 12 | 125461967 | TG | T | 0.304 | NM_032656 | DHX37 | frameshift_deleti  on | exon5 | c.807delC | p.P269fs | . | . |
| CTC5 | 12 | 125465269 | ACTC | A | 0.214 | NM_032656 | DHX37 | nonframeshift_de  letion | exon4 | c.502_504del | p.168_168del | . | . |
| CTC5 | 12 | 125465366 | TA | T | 0.956 | NM_032656 | DHX37 | frameshift_deleti  on | exon4 | c.407delT | p.V136fs | . | . |
| CTC5 | 12 | 132546856 | C | CT | 0.475 | NM_015409 | EP400 | frameshift_inserti  on | exon46 | c.8086_8087ins  T | p.Q2696fs | . | . |
| CTC5 | 12 | 133182783 | CCAG | C | 0.175 | NM_001195520 | LRCOL1 | nonframeshift_de  letion | exon2 | c.52_54del | p.18_18del | . | . |
| CTC5 | 12 | 133197898 | CG | C | 1 | NM_012226 | P2RX2 | frameshift_deleti  on | exon7 | c.748delG | p.G250fs | . | . |
| CTC5 | 12 | 133313532 | TG | T | 0.917 | NM_015114 | ANKLE2 | frameshift_deleti  on | exon8 | c.1539delC | p.P513fs | . | . |
| CTC5 | 12 | 133331361 | AG | A | 0.5 | NM_015114 | ANKLE2 | frameshift_deleti  on | exon2 | c.539delC | p.P180fs | . | . |
| CTC5 | 13 | 21331654 | CT | C | 0.328 | NM_174928 | N6AMT2 | frameshift_deleti  on | exon2 | c.83delA | p.K28fs | . | . |
| CTC5 | 13 | 23914381 | CTT | C | 0.8 | NM_001278055 | SACS | frameshift_deleti  on | exon8 | c.3191_3192del | p.K1064fs | . | . |
| CTC5 | 13 | 25268636 | AT | A | 0.409 | NM_001185085 | ATP12A | frameshift_deleti  on | exon11 | c.1451delT | p.I484fs | . | . |
| CTC5 | 13 | 25479545 | TG | T | 0.598 | NM_018451 | CENPJ | frameshift_deleti  on | exon7 | c.2630delC | p.P877fs | . | . |
| CTC5 | 13 | 25895110 | CA | C | 1 | NM_001008564 | NUPL1 | frameshift_deleti  on | exon8 | c.795delA | p.S265fs | . | . |
| CTC5 | 13 | 29001433 | CT | C | 0.333 | NM_001159920 | FLT1 | frameshift_deleti  on | exon10 | c.1298delA | p.K433fs | . | . |
| CTC5 | 13 | 32351576 | GT | G | 0.365 | NM_001166058 | RXFP2 | frameshift_deleti  on | exon8 | c.706delT | p.F236fs | . | . |

| CTC5 | 13 | 32805393 | AT | A | 0.571 | NM_023037 | FRY | frameshift_deleti  on | exon41 | c.5484delT | p.N1828fs | . | . |
| --- | --- | --- | --- | --- | --- | --- | --- | --- | --- | --- | --- | --- | --- |
| CTC5 | 13 | 32914321 | T | TA | 1 | NM_000059 | BRCA2 | frameshift_inserti  on | exon11 | c.5830dupA | p.S1943fs | . | . |
| CTC5 | 13 | 32929216 | CT | C | 0.379 | NM_000059 | BRCA2 | frameshift_deleti  on | exon14 | c.7227delT | p.P2409fs | . | . |
| CTC5 | 13 | 33628110 | GA | G | 0.21 | NM_004795 | KL | frameshift_deleti  on | exon2 | c.1027delA | p.N343fs | . | . |
| CTC5 | 13 | 33704113 | TG | T | 0.237 | NM_001243466 | STARD13 | frameshift_deleti  on | exon5 | c.676delC | p.Q226fs | . | . |
| CTC5 | 13 | 34392385 | GC | G | 0.615 | NM_002915 | RFC3 | frameshift_deleti  on | exon1 | c.71delC | p.A24fs | . | . |
| CTC5 | 13 | 36006434 | TA | T | 0.25 | NM_015678 | NBEA | frameshift_deleti  on | exon39 | c.6209delA | p.Y2070fs | . | . |
| CTC5 | 13 | 36445426 | CGG | C | 0.857 | NM_004734 | DCLK1 | frameshift_deleti  on | exon5 | c.873_874del | p.S291fs | . | . |
| CTC5 | 13 | 36888477 | CG | C | 0.944 | NM_001142294 | SPG20 | frameshift_deleti  on | exon6 | c.1369delC | p.R457fs | . | . |
| CTC5 | 13 | 38211459 | CA | C | 0.227 | NM_001135958 | TRPC4 | frameshift_deleti  on | exon10 | c.1995delT | p.F665fs | . | . |
| CTC5 | 13 | 42873890 | TA | T | 0.444 | NM_016248 | AKAP11 | frameshift_deleti  on | exon8 | c.1009delA | p.K337fs | . | . |
| CTC5 | 13 | 45768200 | AT | A | 1 | NM_198404 | KCTD4 | stopgain | exon2 | c.502delA | p.I168X | . | . |
| CTC5 | 13 | 46170719 | CCCAGA TACTCTT CCTCCT | C | 1 | NM_182542 | FAM194B | nonframeshift_de letion | exon3 | c.404_421del | p.135_141del | . | . |
| CTC5 | 13 | 49772234 | TC | T | 0.524 | NM_014923 | FNDC3A | frameshift_deleti  on | exon20 | c.2440delC | p.P814fs | . | . |
| CTC5 | 13 | 49951104 | TC | T | 0.44 | NM_030925 | CAB39L | frameshift_deleti  on | exon3 | c.274delG | p.E92fs | . | . |
| CTC5 | 13 | 50280291 | TG | T | 0.4 | NM_002267 | KPNA3 | frameshift_deleti  on | exon14 | c.1151delC | p.T384fs | . | . |
| CTC5 | 13 | 50321095 | TC | T | 1 | NM_002267 | KPNA3 | frameshift_deleti  on | exon2 | c.103delG | p.E35fs | . | . |
| CTC5 | 13 | 51854655 | TC | T | 0.545 | NM_001242312 | FAM124A | frameshift_deleti  on | exon4 | c.905delC | p.S302fs | . | . |
| CTC5 | 13 | 52952510 | AG | A | 0.235 | NM_199263 | THSD1 | frameshift_deleti  on | exon4 | c.1435delC | p.L479fs | . | . |
| CTC5 | 13 | 53239843 | T | TA | 1 | NM_006704 | SUGT1 | frameshift_inserti  on | exon9 | c.495dupA | p.V165fs | . | . |
| CTC5 | 13 | 61986078 | A | AC | 0.85 | NM_022843 | PCDH20 | frameshift_inserti  on | exon2 | c.2153dupG | p.G718fs | . | . |
| CTC5 | 13 | 73309119 | CA | C | 0.299 | NM_001286747 | BORA | frameshift_deleti  on | exon3 | c.73delA | p.K25fs | . | . |
| CTC5 | 13 | 76395548 | AG | A | 0.368 | NM_015842 | LMO7 | frameshift_deleti  on | exon11 | c.1745delG | p.S582fs | . | . |
| CTC5 | 13 | 76410588 | CA | C | 0.929 | NM_015842 | LMO7 | frameshift_deleti  on | exon16 | c.2691delA | p.S897fs | . | . |
| CTC5 | 13 | 76417034 | GT | G | 1 | NM_015842 | LMO7 | frameshift_deleti  on | exon22 | c.3381delT | p.G1127fs | . | . |
| CTC5 | 13 | 77760018 | A | AT | 0.75 | NM_015057 | MYCBP2 | frameshift_inserti  on | exon31 | c.4431_4432ins  A | p.Y1478fs | . | . |
| CTC5 | 13 | 79929414 | TA | T | 0.571 | NM_001286631 | RBM26 | frameshift_deleti  on | exon12 | c.1809delT | p.F603fs | . | . |
| CTC5 | 13 | 96553139 | A | AT | 0.889 | NM_020121 | UGGT2 | frameshift_inserti  on | exon22 | c.2555_2556ins  A | p.I852fs | . | . |
| CTC5 | 13 | 96622484 | A | AT | 0.6 | NM_020121 | UGGT2 | frameshift_inserti  on | exon12 | c.1216dupA | p.M406fs | . | . |
| CTC5 | 13 | 98865548 | GA | G | 0.852 | NM_001001715 | FARP1 | frameshift_deleti  on | exon2 | c.53delA | p.E18fs | . | . |
| CTC5 | 13 | 99907816 | TA | T | 0.5 | NM_001098200 | GPR18 | frameshift_deleti  on | exon2 | c.310delT | p.Y104fs | . | . |
| CTC5 | 13 | 103421878 | AT | A | 0.889 | NM_001286775 | TEX30 | frameshift_deleti  on | exon3 | c.28delA | p.I10fs | . | . |
| CTC5 | 13 | 115090388 | AC | A | 0.194 | NM_001164144 | CHAMP1 | frameshift_deleti  on | exon3 | c.1072delC | p.P358fs | . | . |
| CTC5 | 14 | 19563524 | TTC | T | 0.857 | NM_001005356 | POTEG | frameshift_deleti  on | exon5 | c.1039_1040del | p.S347fs | . | . |
| CTC5 | 14 | 20665618 | AG | A | 0.4 | NM_001005503 | OR11G2 | frameshift_deleti  on | exon1 | c.125delG | p.S42fs | . | . |
| CTC5 | 14 | 21549690 | G | GA | 0.6 | NM_001278529 | ARHGEF40 | frameshift_inserti  on | exon14 | c.521_522insA | p.G174fs | . | . |
| CTC5 | 14 | 21829287 | CT | C | 0.179 | NM_007192 | SUPT16H | frameshift_deleti  on | exon16 | c.1878delA | p.K626fs | . | . |
| CTC5 | 14 | 21979298 | TG | T | 0.3 | NM_019852 | METTL3 | frameshift_deleti  on | exon1 | c.67delC | p.Q23fs | . | . |
| CTC5 | 14 | 23341526 | CCCT | C | 0.143 | NM_014045 | LRP10 | nonframeshift_de  letion | exon1 | c.15_17del | p.5_6del | . | . |
| CTC5 | 14 | 23345412 | GA | G | 1 | NM_014045 | LRP10 | frameshift_deleti  on | exon5 | c.1256delA | p.D419fs | . | . |
| CTC5 | 14 | 23371255 | G | GGGC | 0.857 | NM_001077352 | RBM23 | nonframeshift_in  sertion | exon10 | c.1077_1078ins  GCC | p.Q360delinsA  Q | . | . |
| CTC5 | 14 | 23371265 | G | GGCA | 0.786 | NM_001077352 | RBM23 | nonframeshift_in  sertion | exon10 | c.1067_1068ins  TGC | p.A356delinsA  A | . | . |
| CTC5 | 14 | 23548783 | A | AGAAC  GT | 1 | NM_001164815 | ACIN1 | nonframeshift_in  sertion | exon5 | c.1814_1815ins  ACGTTC | p.S605delinsSR  S | . | . |
| CTC5 | 14 | 23744826 | TTCC | T | 0.5 | NM_020834 | HOMEZ | nonframeshift_de  letion | exon2 | c.1608_1610del | p.536_537del | . | . |
| CTC5 | 14 | 23899846 | AG | A | 0.375 | NM_000257 | MYH7 | frameshift_deleti  on | exon11 | c.921delC | p.P307fs | . | . |
| CTC5 | 14 | 24546929 | CT | C | 0.6 | NM_006032 | CPNE6 | frameshift_deleti  on | exon16 | c.1665delT | p.P555fs | . | . |
| CTC5 | 14 | 24626832 | CT | C | 0.721 | NM_017999 | RNF31 | frameshift_deleti  on | exon16 | c.2713delT | p.F905fs | . | . |
| CTC5 | 14 | 24646406 | G | GGAA | 1 | NM_001048205 | REC8 | nonframeshift_in  sertion | exon8 | c.681_682insG  AA | p.E227delinsEE | . | . |
| CTC5 | 14 | 24785215 | GC | G | 0.912 | NM_001143919 | LTB4R | frameshift_deleti  on | exon2 | c.359delC | p.A120fs | . | . |
| CTC5 | 14 | 24902171 | CT | C | 0.818 | NM_015299 | KHNYN | frameshift_deleti  on | exon5 | c.1511delT | p.L504fs | . | . |
| CTC5 | 14 | 25044473 | TC | T | 0.5 | NM_001911 | CTSG | frameshift_deleti  on | exon2 | c.200delG | p.G67fs | . | . |
| CTC5 | 14 | 32902864 | AC | A | 0.643 | NM_004274 | AKAP6 | frameshift_deleti  on | exon2 | c.166delC | p.P56fs | . | . |
| CTC5 | 14 | 33292449 | GTC | G | 1 | NM_004274 | AKAP6 | frameshift_deleti  on | exon13 | c.5431_5432del | p.S1811fs | . | . |
| CTC5 | 14 | 33293104 | GA | G | 0.688 | NM_004274 | AKAP6 | frameshift_deleti  on | exon13 | c.6086delA | p.E2029fs | . | . |
| CTC5 | 14 | 50088156 | AC | A | 0.957 | NM_002408 | MGAT2 | frameshift_deleti  on | exon1 | c.171delC | p.H57fs | . | . |
| CTC5 | 14 | 50218339 | TG | T | 0.667 | NM_172193 | KLHDC1 | frameshift_deleti  on | exon13 | c.1071delG | p.M357fs | . | . |
| CTC5 | 14 | 52508972 | AC | A | 0.436 | NM_007361 | NID2 | frameshift_deleti  on | exon7 | c.1675delG | p.V559fs | . | . |

| CTC5 | 14 | 53331147 | CG | C | 1 | NM_001134999 | FERMT2 | frameshift_deleti  on | exon12 | c.1573delC | p.R525fs | . | . |
| --- | --- | --- | --- | --- | --- | --- | --- | --- | --- | --- | --- | --- | --- |
| CTC5 | 14 | 54897100 | TC | T | 1 | NM_005776 | CNIH1 | frameshift_deleti  on | exon4 | c.285delG | p.M95fs | . | . |
| CTC5 | 14 | 58718931 | CT | C | 0.833 | NM_002788 | PSMA3 | frameshift_deleti  on | exon3 | c.200delT | p.L67fs | . | . |
| CTC5 | 14 | 58910879 | C | CAAT | 0.4 | NM_001244193 | KIAA0586 | stopgain | exon3 | c.373_374insAA  T | p.Q125delinsQ  X | . | . |
| CTC5 | 14 | 59798115 | A | AG | 0.863 | NM_014992 | DAAM1 | frameshift_inserti  on | exon13 | c.1750dupG | p.L583fs | . | . |
| CTC5 | 14 | 64548201 | GC | G | 0.408 | NM_015180 | SYNE2 | frameshift_deleti  on | exon57 | c.11388delC | p.S3796fs | . | . |
| CTC5 | 14 | 64680980 | TG | T | 0.318 | NM_182913 | SYNE2 | frameshift_deleti  on | exon1 | c.28delG | p.D10fs | . | . |
| CTC5 | 14 | 65209058 | GC | G | 0.5 | NM_015549 | PLEKHG3 | frameshift_deleti  on | exon14 | c.2656delC | p.P886fs | . | . |
| CTC5 | 14 | 68026343 | G | GA | 1 | NM_020715 | PLEKHH1 | frameshift_inserti  on | exon5 | c.359dupA | p.E120fs | . | . |
| CTC5 | 14 | 68052719 | A | AT | 0.444 | NM_020715 | PLEKHH1 | frameshift_inserti  on | exon28 | c.3838_3839ins  T | p.R1280fs | . | . |
| CTC5 | 14 | 69358967 | GC | G | 0.558 | NM_001102 | ACTN1 | frameshift_deleti  on | exon10 | c.888delG | p.W296fs | . | . |
| CTC5 | 14 | 71201166 | TG | T | 0.455 | NM_001284232 | MAP3K9 | frameshift_deleti  on | exon8 | c.1119delC | p.P373fs | . | . |
| CTC5 | 14 | 73407000 | AC | A | 0.385 | NM_001163508 | DCAF4 | frameshift_deleti  on | exon4 | c.267delC | p.N89fs | . | . |
| CTC5 | 14 | 74205953 | TTGC | T | 0.161 | NM_001043318 | ELMSAN1 | nonframeshift_de  letion | exon2 | c.756_758del | p.252_253del | . | . |
| CTC5 | 14 | 74402667 | AT | A | 0.615 | NM_152445 | FAM161B | frameshift_deleti  on | exon8 | c.1978delA | p.I660fs | . | . |
| CTC5 | 14 | 75537745 | AC | A | 0.308 | NM_001042430 | ZC2HC1C | frameshift_deleti  on | exon2 | c.470delC | p.T157fs | . | . |
| CTC5 | 14 | 75989073 | TC | T | 1 | NM_006399 | BATF | frameshift_deleti  on | exon1 | c.49delC | p.P17fs | . | . |
| CTC5 | 14 | 76045320 | TG | T | 0.316 | NM_017791 | FLVCR2 | frameshift_deleti  on | exon1 | c.6delG | p.V2fs | . | . |
| CTC5 | 14 | 76173388 | AT | A | 0.75 | NM_015072 | TTLL5 | frameshift_deleti  on | exon8 | c.614delT | p.I205fs | . | . |
| CTC5 | 14 | 77810132 | TCC | T | 0.681 | NM_213601 | TMED8 | frameshift_deleti  on | exon4 | c.360_361del | p.G120fs | . | . |
| CTC5 | 14 | 80963885 | GT | G | 0.857 | NM_152446 | CEP128 | frameshift_deleti  on | exon24 | c.3221delA | p.N1074fs | . | . |
| CTC5 | 14 | 81670349 | GC | G | 0.5 | NM_015859 | GTF2A1 | frameshift_deleti  on | exon3 | c.231delG | p.Q77fs | . | . |
| CTC5 | 14 | 89044418 | ACC | A | 0.583 | NM_001160103 | ZC3H14 | frameshift_deleti  on | exon9 | c.1214_1215del | p.T405fs | . | . |
| CTC5 | 14 | 89305892 | GA | G | 1 | NM_001288781 | TTC8 | frameshift_deleti  on | exon3 | c.212delA | p.E71fs | . | . |
| CTC5 | 14 | 91121448 | CT | C | 0.345 | NM_001010854 | TTC7B | frameshift_deleti  on | exon12 | c.1347delA | p.E449fs | . | . |
| CTC5 | 14 | 91252645 | TC | T | 0.24 | NM_001010854 | TTC7B | frameshift_deleti  on | exon2 | c.148delG | p.E50fs | . | . |
| CTC5 | 14 | 91805746 | TG | T | 0.889 | NM_001080414 | CCDC88C | frameshift_deleti  on | exon8 | c.684delC | p.P228fs | . | . |
| CTC5 | 14 | 93396101 | GT | G | 0.75 | NM_001275 | CHGA | frameshift_deleti  on | exon5 | c.297delT | p.G99fs | . | . |
| CTC5 | 14 | 95034575 | TGG | T | 0.909 | NM_001289032 | SERPINA4 | frameshift_deleti  on | exon4 | c.1145_1146del | p.W382fs | . | . |
| CTC5 | 14 | 100847581 | TC | T | 0.273 | NM_001161476 | WDR25 | frameshift_deleti  on | exon2 | c.321delC | p.F107fs | . | . |
| CTC5 | 14 | 102252499 | CA | C | 1 | NM_001161726 | PPP2R5C | frameshift_deleti  on | exon3 | c.239delA | p.Q80fs | . | . |
| CTC5 | 14 | 102675673 | T | TC | 0.339 | NM_001242416 | WDR20 | frameshift_inserti  on | exon2 | c.984dupC | p.F328fs | . | . |
| CTC5 | 14 | 102843071 | TC | T | 0.5 | NM_001172631 | TECPR2 | stopgain | exon2 | c.14delC | p.S5X | . | . |
| CTC5 | 14 | 105409879 | AT | A | 1 | NM_138420 | AHNAK2 | frameshift_deleti on | exon7 | c.11908delA | p.M3970fs | . | . |
| CTC5 | 14 | 105415789 | AC | A | 1 | NM_138420 | AHNAK2 | frameshift_deleti  on | exon7 | c.5998delG | p.V2000fs | . | . |
| CTC5 | 14 | 105452981 | TG | T | 0.774 | NM_174891 | C14orf79 | frameshift_deleti  on | exon1 | c.214delG | p.G72fs | . | . |
| CTC5 | 15 | 23006301 | CATT | C | 0.259 | NM_001008894 | NIPA2 | nonframeshift_de  letion | exon5 | c.943_945del | p.315_315del | . | . |
| CTC5 | 15 | 23579214 | AGGATG  GGTAGG GAGGTG | A | 0.913 | NM_001271664 | LOC440243 | nonframeshift_de letion | exon1 | c.57_74del | p.19_25del | . | . |
| CTC5 | 15 | 23579214 | AGGATG  GGTAGG GAGGTG GGGATG GGTAGG | A | 0.913 | NM_001271664 | LOC440243 | nonframeshift_de letion | exon1 | c.39_74del | p.13_25del | . | . |
| CTC5 | 15 | 28422097 | A | AT | 0.368 | NM_004667 | HERC2 | frameshift_inserti  on | exon61 | c.9430dupA | p.M3144fs | . | . |
| CTC5 | 15 | 28508205 | TG | T | 1 | NM_004667 | HERC2 | frameshift_deleti  on | exon15 | c.1980delC | p.V660fs | . | . |
| CTC5 | 15 | 30065503 | GA | G | 0.547 | NM_003257 | TJP1 | frameshift_deleti  on | exon3 | c.141delT | p.F47fs | . | . |
| CTC5 | 15 | 31253221 | ACCT | A | 0.154 | NM_017762 | MTMR10 | nonframeshift_de  letion | exon7 | c.618_620del | p.206_207del | . | . |
| CTC5 | 15 | 33916175 | AG | A | 0.625 | NM_001036 | RYR3 | frameshift_deleti  on | exon20 | c.2526delG | p.Q842fs | . | . |
| CTC5 | 15 | 33928732 | TG | T | 0.912 | NM_001036 | RYR3 | frameshift_deleti  on | exon27 | c.3538delG | p.D1180fs | . | . |
| CTC5 | 15 | 34396080 | GT | G | 0.45 | NM_152595 | PGBD4 | frameshift_deleti  on | exon1 | c.1349delT | p.V450fs | . | . |
| CTC5 | 15 | 34628788 | TGG | T | 1 | NM_001042497 | SLC12A6 | frameshift_deleti  on | exon1 | c.92_93del | p.T31fs | . | . |
| CTC5 | 15 | 37184461 | TC | T | 0.429 | NM_170675 | MEIS2 | frameshift_deleti  on | exon12 | c.1346delG | p.G449fs | . | . |
| CTC5 | 15 | 38794544 | GA | G | 0.615 | NM_001128602 | RASGRP1 | frameshift_deleti  on | exon11 | c.1401delT | p.F467fs | . | . |
| CTC5 | 15 | 41067235 | CA | C | 0.364 | NM_018163 | DNAJC17 | frameshift_deleti  on | exon8 | c.593delT | p.L198fs | . | . |
| CTC5 | 15 | 41071772 | AG | A | 0.6 | NM_018163 | DNAJC17 | frameshift_deleti  on | exon3 | c.177delC | p.A59fs | . | . |
| CTC5 | 15 | 41191583 | C | CT | 0.389 | NM_020857 | VPS18 | frameshift_inserti  on | exon4 | c.568dupT | p.Y189fs | . | . |
| CTC5 | 15 | 42057194 | GT | G | 0.959 | NM_001080541 | MGA | frameshift_deleti  on | exon22 | c.7229delT | p.V2410fs | . | . |
| CTC5 | 15 | 42115873 | TC | T | 0.267 | NM_014994 | MAPKBP1 | frameshift_deleti  on | exon29 | c.3828delC | p.V1276fs | . | . |
| CTC5 | 15 | 42978446 | TG | T | 1 | NM_020759 | STARD9 | frameshift_deleti  on | exon23 | c.4671delG | p.L1557fs | . | . |

| CTC5 | 15 | 43242528 | AC | A | 0.538 | NM_174916 | UBR1 | frameshift_deleti  on | exon46 | c.5039delG | p.G1680fs | . | . |
| --- | --- | --- | --- | --- | --- | --- | --- | --- | --- | --- | --- | --- | --- |
| CTC5 | 15 | 43662007 | AG | A | 0.778 | NM_152455 | ZSCAN29 | frameshift_deleti  on | exon1 | c.104delC | p.A35fs | . | . |
| CTC5 | 15 | 44967799 | TCC | T | 0.6 | NM_001145112 | PATL2 | frameshift_deleti  on | exon3 | c.105_106del | p.E35fs | . | . |
| CTC5 | 15 | 48441386 | TG | T | 0.971 | NM_016132 | MYEF2 | frameshift_deleti  on | exon15 | c.1560delC | p.S520fs | . | . |
| CTC5 | 15 | 49061197 | G | GA | 0.75 | NM_001194998 | CEP152 | frameshift_inserti  on | exon14 | c.1863dupT | p.L622fs | . | . |
| CTC5 | 15 | 49304895 | CT | C | 0.729 | NM_014701 | SECISBP2L | frameshift_deleti  on | exon11 | c.1545delA | p.K515fs | . | . |
| CTC5 | 15 | 49319602 | GA | G | 0.489 | NM_001193489 | SECISBP2L | frameshift_deleti  on | exon7 | c.994delT | p.S332fs | . | . |
| CTC5 | 15 | 49575803 | TA | T | 0.722 | NM_001001556 | GALK2 | frameshift_deleti  on | exon7 | c.612delA | p.V204fs | . | . |
| CTC5 | 15 | 50223436 | AC | A | 0.203 | NM_024837 | ATP8B4 | frameshift_deleti  on | exon16 | c.1521delG | p.G507fs | . | . |
| CTC5 | 15 | 50830942 | G | GA | 0.255 | NM_203494 | USP50 | frameshift_inserti  on | exon5 | c.766dupT | p.S256fs | . | . |
| CTC5 | 15 | 51503059 | GT | G | 0.31 | NM_000103 | CYP19A1 | frameshift_deleti  on | exon10 | c.1457delA | p.N486fs | . | . |
| CTC5 | 15 | 51773580 | AT | A | 0.618 | NM_001174117 | DMXL2 | frameshift_deleti  on | exon22 | c.3814delA | p.I1272fs | . | . |
| CTC5 | 15 | 56756386 | GT | G | 0.353 | NM_018365 | MNS1 | frameshift_deleti  on | exon2 | c.62delA | p.N21fs | . | . |
| CTC5 | 15 | 57734627 | TC | T | 0.778 | NM_032866 | CGNL1 | frameshift_deleti  on | exon4 | c.1755delC | p.I585fs | . | . |
| CTC5 | 15 | 59465980 | CG | C | 0.636 | NM_004998 | MYO1E | frameshift_deleti  on | exon21 | c.2298delC | p.F766fs | . | . |
| CTC5 | 15 | 63031591 | G | GA | 0.622 | NM_015059 | TLN2 | frameshift_inserti  on | exon28 | c.3733dupA | p.L1244fs | . | . |
| CTC5 | 15 | 63862706 | GT | G | 0.321 | NM_001256702 | USP3 | frameshift_deleti  on | exon8 | c.705delT | p.G235fs | . | . |
| CTC5 | 15 | 63882978 | CT | C | 1 | NM_001256702 | USP3 | frameshift_deleti  on | exon14 | c.1385delT | p.L462fs | . | . |
| CTC5 | 15 | 64967273 | AC | A | 0.235 | NM_015042 | ZNF609 | frameshift_deleti  on | exon4 | c.2221delC | p.L741fs | . | . |
| CTC5 | 15 | 65684585 | GC | G | 0.72 | NM_020962 | IGDCC4 | frameshift_deleti  on | exon11 | c.2008delG | p.A670fs | . | . |
| CTC5 | 15 | 65703541 | GC | G | 0.542 | NM_020962 | IGDCC4 | frameshift_deleti  on | exon2 | c.237delG | p.L79fs | . | . |
| CTC5 | 15 | 65864661 | AT | A | 0.8 | NM_016395 | PTPLAD1 | frameshift_deleti  on | exon10 | c.970delT | p.F324fs | . | . |
| CTC5 | 15 | 65983727 | C | CT | 1 | NM_005848 | DENND4A | frameshift_inserti  on | exon22 | c.3072dupA | p.V1025fs | . | . |
| CTC5 | 15 | 71276480 | GCAA | G | 1 | NM_001199017 | LRRC49 | nonframeshift_de  letion | exon11 | c.1069_1071del | p.357_357del | . | . |
| CTC5 | 15 | 72647949 | AT | A | 1 | NM_000520 | HEXA | frameshift_deleti  on | exon3 | c.362delA | p.N121fs | . | . |
| CTC5 | 15 | 73996650 | GC | G | 1 | NM_025240 | CD276 | frameshift_deleti  on | exon4 | c.553delC | p.P185fs | . | . |
| CTC5 | 15 | 74176544 | GA | G | 0.583 | NM_001286434 | TBC1D21 | frameshift_deleti  on | exon3 | c.218delA | p.E73fs | . | . |
| CTC5 | 15 | 74920360 | CT | C | 0.286 | NM_001130028 | CLK3 | frameshift_deleti  on | exon10 | c.1537delT | p.F513fs | . | . |
| CTC5 | 15 | 75631438 | GC | G | 0.328 | NM_001284379 | COMMD4 | frameshift_deleti  on | exon4 | c.313delC | p.P105fs | . | . |
| CTC5 | 15 | 75648327 | GA | G | 0.875 | NM_001256496 | MAN2C1 | frameshift_deleti  on | exon24 | c.2745delT | p.L915fs | . | . |
| CTC5 | 15 | 76019703 | GC | G | 0.25 | NM_175881 | ODF3L1 | frameshift_deleti  on | exon4 | c.648delC | p.S216fs | . | . |
| CTC5 | 15 | 77059315 | C | CA | 0.222 | NM_020843 | SCAPER | stopgain | exon10 | c.1362dupT | p.E455_A456de  linsX | . | . |
| CTC5 | 15 | 77425681 | GA | G | 0.8 | NM_024776 | PEAK1 | frameshift_deleti  on | exon7 | c.3742delT | p.S1248fs | . | . |
| CTC5 | 15 | 78577617 | CA | C | 0.867 | NM_025234 | WDR61 | frameshift_deleti  on | exon10 | c.813delT | p.F271fs | . | . |
| CTC5 | 15 | 78893622 | AT | A | 0.562 | NM_000743 | CHRNA3 | frameshift_deleti  on | exon5 | c.1361delA | p.N454fs | . | . |
| CTC5 | 15 | 79090324 | AC | A | 0.561 | NM_014272 | ADAMTS7 | frameshift_deleti  on | exon3 | c.587delG | p.G196fs | . | . |
| CTC5 | 15 | 79606142 | AC | A | 0.373 | NM_007364 | TMED3 | frameshift_deleti  on | exon2 | c.213delC | p.D71fs | . | . |
| CTC5 | 15 | 81166235 | GA | G | 0.833 | NM_018689 | CEMIP | frameshift_deleti  on | exon2 | c.16delA | p.R6fs | . | . |
| CTC5 | 15 | 83523511 | AG | A | 0.333 | NM_004839 | HOMER2 | frameshift_deleti  on | exon6 | c.535delC | p.L179fs | . | . |
| CTC5 | 15 | 88678621 | TG | T | 0.438 | NM_001007156 | NTRK3 | frameshift_deleti  on | exon10 | c.914delC | p.P305fs | . | . |
| CTC5 | 15 | 89414719 | GC | G | 0.977 | NM_001135 | ACAN | frameshift_deleti  on | exon13 | c.6940delC | p.Q2314fs | . | . |
| CTC5 | 15 | 90335494 | ACTC | A | 0.923 | NM_001150 | ANPEP | nonframeshift_de  letion | exon18 | c.2422_2424del | p.808_808del | . | . |
| CTC5 | 15 | 90335498 | CTCCT | C | 0.5 | NM_001150 | ANPEP | frameshift_deleti  on | exon18 | c.2417_2420del | p.E806fs | . | . |
| CTC5 | 15 | 90764272 | GC | G | 0.178 | NM_198925 | SEMA4B | frameshift_deleti  on | exon5 | c.543delC | p.G181fs | . | . |
| CTC5 | 15 | 91293030 | GT | G | 0.412 | NM_000057 | BLM | frameshift_deleti  on | exon3 | c.533delT | p.V178fs | . | . |
| CTC5 | 15 | 91293055 | CA | C | 0.848 | NM_000057 | BLM | frameshift_deleti  on | exon3 | c.558delA | p.S186fs | . | . |
| CTC5 | 15 | 92671604 | AC | A | 0.312 | NM_001145044 | SLCO3A1 | frameshift_deleti  on | exon7 | c.1398delC | p.D466fs | . | . |
| CTC5 | 15 | 93487673 | G | GA | 0.257 | NM_001042572 | CHD2 | frameshift_inserti  on | exon10 | c.1082dupA | p.E361fs | . | . |
| CTC5 | 15 | 93498741 | AG | A | 0.375 | NM_001271 | CHD2 | frameshift_deleti  on | exon15 | c.1809delG | p.K603fs | . | . |
| CTC5 | 15 | 100252709 | CCAG | C | 0.984 | NM_001130928 | MEF2A | nonframeshift_de  letion | exon8 | c.1024_1026del | p.342_342del | . | . |
| CTC5 | 15 | 100252709 | CCAGCA  G | C | 0.984 | NM_001130928 | MEF2A | nonframeshift_de  letion | exon8 | c.1024_1029del | p.342_343del | . | . |
| CTC5 | 15 | 102358773 | A | AT | 0.667 | NM_001001674 | OR4F15 | frameshift_inserti  on | exon1 | c.384_385insT | p.K128fs | . | . |
| CTC5 | 16 | 341240 | CG | C | 0.938 | NM_003502 | AXIN1 | frameshift_deleti  on | exon9 | c.2243delC | p.P748fs | . | . |
| CTC5 | 16 | 450140 | C | CAG | 0.963 | NM_001286435 | NME4 | frameshift_inserti  on | exon5 | c.472_473insA  G | p.R158fs | . | . |
| CTC5 | 16 | 618280 | CT | C | 0.583 | NM_176677 | NHLRC4 | frameshift_deleti  on | exon2 | c.234delT | p.A78fs | . | . |
| CTC5 | 16 | 1719060 | TC | T | 0.232 | NM_020825 | CRAMP1L | frameshift_deleti  on | exon18 | c.3394delC | p.P1132fs | . | . |
| CTC5 | 16 | 3100064 | TG | T | 0.375 | NM_022468 | MMP25 | frameshift_deleti  on | exon3 | c.288delG | p.L96fs | . | . |
| CTC5 | 16 | 3633335 | GC | G | 0.25 | NM_032444 | SLX4 | frameshift_deleti  on | exon14 | c.4915delG | p.A1639fs | . | . |

| CTC5 | 16 | 11001396 | GC | G | 0.286 | NM_000246 | CIITA | frameshift_deleti  on | exon11 | c.2048delC | p.A683fs | . | . |
| --- | --- | --- | --- | --- | --- | --- | --- | --- | --- | --- | --- | --- | --- |
| CTC5 | 16 | 11063070 | CT | C | 1 | NM_001243403 | CLEC16A | frameshift_deleti  on | exon4 | c.397delT | p.F133fs | . | . |
| CTC5 | 16 | 14041733 | GT | G | 0.925 | NM_005236 | ERCC4 | frameshift_deleti  on | exon11 | c.2281delT | p.F761fs | . | . |
| CTC5 | 16 | 14334244 | C | CG | 0.278 | NM_014048 | MKL2 | frameshift_inserti  on | exon10 | c.983dupG | p.R328fs | . | . |
| CTC5 | 16 | 15831312 | AG | A | 0.651 | NM_002474 | MYH11 | frameshift_deleti  on | exon25 | c.3286delC | p.L1096fs | . | . |
| CTC5 | 16 | 16315487 | TC | T | 0.594 | NM_001079528 | ABCC6 | frameshift_deleti  on | exon2 | c.237delG | p.G79fs | . | . |
| CTC5 | 16 | 17294473 | CG | C | 0.357 | NM_022166 | XYLT1 | frameshift_deleti  on | exon4 | c.951delC | p.S317fs | . | . |
| CTC5 | 16 | 19519079 | AT | A | 0.947 | NM_016641 | GDE1 | frameshift_deleti  on | exon4 | c.565delA | p.M189fs | . | . |
| CTC5 | 16 | 19873222 | GCTGGG  TCTTTTC CCCAAG | G | 0.125 | NM_016235 | GPRC5B | nonframeshift_de letion | exon3 | c.1080_1103del | p.360_368del | . | . |
| CTC5 | 16 | 20331030 | TG | T | 0.714 | NM_001007242 | GP2 | frameshift_deleti  on | exon5 | c.477delC | p.I159fs | . | . |
| CTC5 | 16 | 20395999 | TC | T | 1 | NM_174924 | PDILT | frameshift_deleti  on | exon3 | c.376delG | p.E126fs | . | . |
| CTC5 | 16 | 20809950 | GA | G | 0.914 | NM_001142725 | ERI2 | frameshift_deleti  on | exon9 | c.1171delT | p.S391fs | . | . |
| CTC5 | 16 | 20809980 | G | GA | 0.2 | NM_001142725 | ERI2 | frameshift_inserti  on | exon9 | c.1141dupT | p.S381fs | . | . |
| CTC5 | 16 | 20833106 | AG | A | 0.173 | NM_001144924 | LOC81691 | frameshift_deleti  on | exon6 | c.499delG | p.A167fs | . | . |
| CTC5 | 16 | 20944793 | CG | C | 0.597 | NM_017539 | DNAH3 | frameshift_deleti  on | exon62 | c.12033delC | p.P4011fs | . | . |
| CTC5 | 16 | 21123003 | AT | A | 0.281 | NM_017539 | DNAH3 | frameshift_deleti  on | exon14 | c.2042delA | p.N681fs | . | . |
| CTC5 | 16 | 21209132 | CA | C | 0.194 | NM_003460 | ZP2 | frameshift_deleti  on | exon18 | c.2049delT | p.S683fs | . | . |
| CTC5 | 16 | 23570948 | A | AT | 1 | NM_019116 | UBFD1 | frameshift_inserti  on | exon3 | c.515_516insT | p.Q172fs | . | . |
| CTC5 | 16 | 24183614 | GT | G | 0.276 | NM_002738 | PRKCB | frameshift_deleti  on | exon11 | c.1264delT | p.Y422fs | . | . |
| CTC5 | 16 | 24581277 | GA | G | 0.95 | NM_018703 | RBBP6 | frameshift_deleti  on | exon16 | c.3165delA | p.R1055fs | . | . |
| CTC5 | 16 | 24788422 | AGCAGC  CACAGC | A | 1 | NM_014494 | TNRC6A | nonframeshift_de  letion | exon5 | c.333_344del | p.111_115del | . | . |
| CTC5 | 16 | 24816374 | GC | G | 0.818 | NM_014494 | TNRC6A | frameshift_deleti  on | exon14 | c.4025delC | p.A1342fs | . | . |
| CTC5 | 16 | 24834262 | TCC | T | 0.4 | NM_014494 | TNRC6A | frameshift_deleti  on | exon24 | c.5442_5443del | p.L1814fs | . | . |
| CTC5 | 16 | 24909331 | TC | T | 1 | NM_001258412 | SLC5A11 | frameshift_deleti  on | exon9 | c.698delC | p.S233fs | . | . |
| CTC5 | 16 | 27237058 | TG | T | 0.302 | NM_145080 | NSMCE1 | frameshift_deleti  on | exon7 | c.709delC | p.H237fs | . | . |
| CTC5 | 16 | 27481583 | TTG | T | 0.692 | NM_001286242 | GTF3C1 | frameshift_deleti  on | exon31 | c.4658_4659del | p.T1553fs | . | . |
| CTC5 | 16 | 27508983 | CG | C | 0.947 | NM_001286242 | GTF3C1 | frameshift_deleti  on | exon14 | c.2324delC | p.P775fs | . | . |
| CTC5 | 16 | 28855616 | AT | A | 0.628 | NM_003321 | TUFM | frameshift_deleti  on | exon7 | c.856delA | p.I286fs | . | . |
| CTC5 | 16 | 28943878 | GC | G | 0.409 | NM_001178098 | CD19 | frameshift_deleti  on | exon2 | c.301delC | p.P101fs | . | . |
| CTC5 | 16 | 28944601 | AC | A | 0.63 | NM_001178098 | CD19 | frameshift_deleti  on | exon4 | c.607delC | p.P203fs | . | . |
| CTC5 | 16 | 28994192 | TG | T | 0.306 | NM_001142449 | SPNS1 | frameshift_deleti  on | exon7 | c.949delG | p.G317fs | . | . |
| CTC5 | 16 | 30016640 | AC | A | 1 | NM_173618 | INO80E | frameshift_deleti  on | exon7 | c.613delC | p.P205fs | . | . |
| CTC5 | 16 | 30495526 | AT | A | 0.267 | NM_001114380 | ITGAL | frameshift_deleti  on | exon7 | c.700delT | p.F234fs | . | . |
| CTC5 | 16 | 30545760 | TG | T | 0.493 | NM_023931 | ZNF747 | frameshift_deleti  on | exon1 | c.240delC | p.P80fs | . | . |
| CTC5 | 16 | 30663226 | GA | G | 0.923 | NM_024031 | PRR14 | frameshift_deleti  on | exon3 | c.134delA | p.E45fs | . | . |
| CTC5 | 16 | 30745885 | TGAA | T | 1 | NM_006662 | SRCAP | nonframeshift_de  letion | exon31 | c.6679_6681del | p.2227_2227de  l | . | . |
| CTC5 | 16 | 30780190 | TG | T | 0.625 | NM_001207034 | RNF40 | frameshift_deleti  on | exon13 | c.1830delG | p.L610fs | . | . |
| CTC5 | 16 | 30785424 | TA | T | 0.212 | NM_001207034 | RNF40 | frameshift_deleti  on | exon18 | c.2696delA | p.Y899fs | . | . |
| CTC5 | 16 | 31143780 | GC | G | 0.545 | NM_002773 | PRSS8 | frameshift_deleti  on | exon5 | c.674delG | p.G225fs | . | . |
| CTC5 | 16 | 31196276 | TC | T | 0.75 | NM_001170634 | FUS | frameshift_deleti  on | exon6 | c.538delC | p.Q180fs | . | . |
| CTC5 | 16 | 31434435 | GC | G | 0.209 | NM_005353 | ITGAD | frameshift_deleti  on | exon24 | c.2782delC | p.Q928fs | . | . |
| CTC5 | 16 | 31504298 | CT | C | 1 | NM_022744 | C16orf58 | frameshift_deleti  on | exon10 | c.1087delA | p.N363fs | . | . |
| CTC5 | 16 | 46615958 | CT | C | 0.571 | NM_024745 | SHCBP1 | frameshift_deleti  on | exon13 | c.1701delA | p.K567fs | . | . |
| CTC5 | 16 | 46771795 | TC | T | 0.417 | NM_182493 | MYLK3 | frameshift_deleti  on | exon3 | c.828delG | p.P276fs | . | . |
| CTC5 | 16 | 47005315 | TG | T | 0.973 | NM_005880 | DNAJA2 | frameshift_deleti  on | exon3 | c.307delC | p.Q103fs | . | . |
| CTC5 | 16 | 48174716 | CA | C | 0.96 | NM_033226 | ABCC12 | frameshift_deleti  on | exon4 | c.538delT | p.W180fs | . | . |
| CTC5 | 16 | 51172761 | A | AT | 0.378 | NM_001127892 | SALL1 | frameshift_inserti  on | exon2 | c.3080_3081ins  A | p.P1027fs | . | . |
| CTC5 | 16 | 51173908 | G | GC | 0.338 | NM_001127892 | SALL1 | frameshift_inserti  on | exon2 | c.1933dupG | p.A645fs | . | . |
| CTC5 | 16 | 53320147 | A | AT | 0.333 | NM_025134 | CHD9 | frameshift_inserti  on | exon26 | c.5081_5082ins  T | p.K1694fs | . | . |
| CTC5 | 16 | 55846885 | TC | T | 0.25 | NM_001025194 | CES1 | frameshift_deleti  on | exon9 | c.1012delG | p.E338fs | . | . |
| CTC5 | 16 | 55907839 | AG | A | 0.451 | NM_001143685 | CES5A | frameshift_deleti  on | exon2 | c.183delC | p.P61fs | . | . |
| CTC5 | 16 | 57059430 | AG | A | 0.495 | NM_032206 | NLRC5 | frameshift_deleti  on | exon5 | c.576delG | p.E192fs | . | . |
| CTC5 | 16 | 57600578 | GC | G | 1 | NM_153837 | GPR114 | frameshift_deleti  on | exon7 | c.615delC | p.S205fs | . | . |
| CTC5 | 16 | 57717848 | TC | T | 1 | NM_170776 | GPR97 | frameshift_deleti  on | exon9 | c.887delC | p.S296fs | . | . |
| CTC5 | 16 | 58287968 | CA | C | 0.325 | NM_014157 | CCDC113 | frameshift_deleti  on | exon3 | c.296delA | p.Q99fs | . | . |
| CTC5 | 16 | 58314146 | GA | G | 1 | NM_001080492 | PRSS54 | frameshift_deleti  on | exon7 | c.1169delT | p.F390fs | . | . |
| CTC5 | 16 | 58314208 | CA | C | 0.917 | NM_001080492 | PRSS54 | frameshift_deleti  on | exon7 | c.1107delT | p.F369fs | . | . |

| CTC5 | 16 | 66547659 | A | AT | 0.4 | NM_001271935 | TK2 | frameshift_inserti  on | exon7 | c.411_412insA | p.F138fs | . | . |
| --- | --- | --- | --- | --- | --- | --- | --- | --- | --- | --- | --- | --- | --- |
| CTC5 | 16 | 66824856 | TG | T | 1 | NM_001136505 | CCDC79 | frameshift_deleti  on | exon4 | c.97delC | p.Q33fs | . | . |
| CTC5 | 16 | 67229793 | ACAG | A | 0.351 | NM_001950 | E2F4 | nonframeshift_de  letion | exon7 | c.918_920del | p.306_307del | . | . |
| CTC5 | 16 | 67683465 | TG | T | 1 | NM_001013838 | RLTPR | frameshift_deleti  on | exon20 | c.1863delG | p.M621fs | . | . |
| CTC5 | 16 | 67692831 | CG | C | 0.857 | NM_001082486 | ACD | frameshift_deleti  on | exon7 | c.902delC | p.T301fs | . | . |
| CTC5 | 16 | 67861791 | CGA | C | 0.298 | NM_001288994 | TSNAXIP1 | frameshift_deleti  on | exon11 | c.997_998del | p.E333fs | . | . |
| CTC5 | 16 | 69726121 | CA | C | 0.306 | NM_006599 | NFAT5 | frameshift_deleti  on | exon12 | c.2340delA | p.A780fs | . | . |
| CTC5 | 16 | 69972981 | AC | A | 1 | NM_199424 | WWP2 | frameshift_deleti  on | exon12 | c.1079delC | p.T360fs | . | . |
| CTC5 | 16 | 70501297 | CGG | C | 0.182 | NM_145059 | FUK | frameshift_deleti  on | exon7 | c.506_507del | p.R169fs | . | . |
| CTC5 | 16 | 70975639 | CA | C | 0.833 | NM_001270974 | HYDIN | frameshift_deleti  on | exon43 | c.6752delT | p.L2251fs | . | . |
| CTC5 | 16 | 71318611 | TA | T | 0.622 | NM_001099642 | CMTR2 | frameshift_deleti  on | exon3 | c.1212delT | p.F404fs | . | . |
| CTC5 | 16 | 71799475 | AT | A | 0.947 | NM_001128 | AP1G1 | frameshift_deleti  on | exon7 | c.654delA | p.Q218fs | . | . |
| CTC5 | 16 | 71799477 | TG | T | 0.944 | NM_001128 | AP1G1 | frameshift_deleti  on | exon7 | c.652delC | p.Q218fs | . | . |
| CTC5 | 16 | 71956511 | AATGCC  C | A | 0.667 | NM_001270979 | IST1 | nonframeshift_de  letion | exon3 | c.244_249del | p.82_83del | . | . |
| CTC5 | 16 | 72120661 | TG | T | 0.526 | NM_001142317 | TXNL4B | frameshift_deleti  on | exon4 | c.324delC | p.T108fs | . | . |
| CTC5 | 16 | 72830468 | TC | T | 0.59 | NM_001164766 | ZFHX3 | frameshift_deleti  on | exon8 | c.3370delG | p.E1124fs | . | . |
| CTC5 | 16 | 74695092 | AG | A | 0.429 | NM_018124 | RFWD3 | frameshift_deleti  on | exon2 | c.255delC | p.V85fs | . | . |
| CTC5 | 16 | 77401594 | AT | A | 0.712 | NM_199355 | ADAMTS18 | frameshift_deleti  on | exon4 | c.521delA | p.N174fs | . | . |
| CTC5 | 16 | 81213351 | CA | C | 0.625 | NM_001278423 | PKD1L2 | frameshift_deleti  on | exon2 | c.103delT | p.W35fs | . | . |
| CTC5 | 16 | 81733347 | GA | G | 1 | NM_030629 | CMIP | frameshift_deleti  on | exon15 | c.1415delA | p.E472fs | . | . |
| CTC5 | 16 | 84271058 | G | GA | 0.793 | NM_172347 | KCNG4 | frameshift_inserti  on | exon2 | c.33dupT | p.P12fs | . | . |
| CTC5 | 16 | 88800418 | TG | T | 0.973 | NM_001142864 | PIEZO1 | frameshift_deleti  on | exon17 | c.2224delC | p.Q742fs | . | . |
| CTC5 | 16 | 89346968 | G | GA | 0.5 | NM_001256183 | ANKRD11 | frameshift_inserti  on | exon9 | c.5981dupT | p.F1994fs | . | . |
| CTC5 | 16 | 89351086 | G | GT | 0.453 | NM_001256183 | ANKRD11 | frameshift_inserti  on | exon9 | c.1863dupA | p.L622fs | . | . |
| CTC5 | 16 | 89576919 | AC | A | 1 | NM_003119 | SPG7 | frameshift_deleti  on | exon2 | c.206delC | p.T69fs | . | . |
| CTC5 | 16 | 89702682 | TG | T | 0.204 | NM_001128141 | DPEP1 | frameshift_deleti  on | exon4 | c.249delG | p.V83fs | . | . |
| CTC5 | 16 | 89842212 | AT | A | 1 | NM_000135 | FANCA | frameshift_deleti  on | exon21 | c.1837delA | p.I613fs | . | . |
| CTC5 | 16 | 90095557 | CCTACG  GGGCAG | C | 1 | NM_001214 | C16orf3 | nonframeshift_de  letion | exon1 | c.182_193del | p.61_65del | . | . |
| CTC5 | 16 | 90095558 | C | CTACG  GGGCA | 1 | NM_001214 | C16orf3 | nonframeshift_in  sertion | exon1 | c.192_193insA  GCTGCCCCGT | p.G65delinsSC  PVG | . | . |
| CTC5 | 17 | 650303 | CA | C | 0.917 | NM_015721 | GEMIN4 | frameshift_deleti  on | exon2 | c.979delT | p.W327fs | . | . |
| CTC5 | 17 | 1989125 | TC | T | 0.195 | NM_001282326 | SMG6 | frameshift_deleti  on | exon2 | c.160delG | p.E54fs | . | . |
| CTC5 | 17 | 2218991 | GT | G | 0.769 | NM_021947 | SRR | frameshift_deleti  on | exon2 | c.138delT | p.C46fs | . | . |
| CTC5 | 17 | 3195255 | TA | T | 0.75 | NM_002550 | OR3A1 | frameshift_deleti  on | exon1 | c.621delT | p.F207fs | . | . |
| CTC5 | 17 | 3967927 | AG | A | 0.476 | NM_015113 | ZZEF1 | frameshift_deleti  on | exon29 | c.4445delC | p.P1482fs | . | . |
| CTC5 | 17 | 4458202 | AC | A | 0.476 | NM_001105538 | MYBBP1A | frameshift_deleti  on | exon2 | c.247delG | p.V83fs | . | . |
| CTC5 | 17 | 4621971 | CG | C | 0.824 | NM_001257331 | ARRB2 | frameshift_deleti  on | exon9 | c.727delG | p.E243fs | . | . |
| CTC5 | 17 | 5009128 | AG | A | 0.333 | NM_014519 | ZNF232 | frameshift_deleti  on | exon5 | c.1325delC | p.P442fs | . | . |
| CTC5 | 17 | 5085129 | TA | T | 1 | NM_032530 | ZNF594 | stoploss | exon2 | c.2422delT | p.X808R | . | . |
| CTC5 | 17 | 6483140 | GC | G | 1 | NM_014804 | KIAA0753 | frameshift_deleti on | exon19 | c.2830delG | p.A944fs | . | . |
| CTC5 | 17 | 6690719 | AT | A | 0.857 | NM_153230 | FBXO39 | frameshift_deleti  on | exon4 | c.1302delT | p.Y434fs | . | . |
| CTC5 | 17 | 7291774 | AG | A | 0.333 | NM_001251902 | TNK1 | frameshift_deleti  on | exon11 | c.1543delG | p.G515fs | . | . |
| CTC5 | 17 | 7329685 | TCGC | T | 0.148 | NM_175734 | C17orf74 | nonframeshift_de  letion | exon3 | c.376_378del | p.126_126del | . | . |
| CTC5 | 17 | 7369803 | GGAA | G | 0.11 | NM_001128833 | ZBTB4 | nonframeshift_de  letion | exon3 | c.315_317del | p.105_106del | . | . |
| CTC5 | 17 | 7495827 | TG | T | 1 | NM_004860 | FXR2 | frameshift_deleti  on | exon15 | c.1819delC | p.Q607fs | . | . |
| CTC5 | 17 | 7673898 | CT | C | 0.368 | NM_020877 | DNAH2 | frameshift_deleti  on | exon25 | c.4123delT | p.W1375fs | . | . |
| CTC5 | 17 | 7751858 | TCACCA  C | T | 1 | NM_001080424 | KDM6B | nonframeshift_de  letion | exon11 | c.2253_2258del | p.751_753del | . | . |
| CTC5 | 17 | 7751858 | TCACCA  CCAC | T | 1 | NM_001080424 | KDM6B | nonframeshift_de  letion | exon11 | c.2253_2261del | p.751_754del | . | . |
| CTC5 | 17 | 8168620 | C | CA | 0.312 | NM_012393 | PFAS | frameshift_inserti  on | exon19 | c.2295_2296ins  A | p.S765fs | . | . |
| CTC5 | 17 | 8170387 | TG | T | 0.8 | NM_012393 | PFAS | frameshift_deleti  on | exon24 | c.3010delG | p.V1004fs | . | . |
| CTC5 | 17 | 10312771 | GC | G | 0.448 | NM_002472 | MYH8 | frameshift_deleti  on | exon16 | c.1721delG | p.G574fs | . | . |
| CTC5 | 17 | 10408151 | AT | A | 1 | NM_005963 | MYH1 | frameshift_deleti  on | exon22 | c.2666delA | p.N889fs | . | . |
| CTC5 | 17 | 10415411 | TG | T | 0.222 | NM_005963 | MYH1 | frameshift_deleti  on | exon13 | c.1245delC | p.T415fs | . | . |
| CTC5 | 17 | 11631220 | TGTCA | T | 0.1 | NM_001372 | DNAH9 | frameshift_deleti  on | exon28 | c.5796_5799del | p.L1932fs | . | . |
| CTC5 | 17 | 17699275 | C | CG | 0.291 | NM_030665 | RAI1 | frameshift_inserti  on | exon3 | c.3014dupG | p.R1005fs | . | . |
| CTC5 | 17 | 17699837 | GC | G | 1 | NM_030665 | RAI1 | frameshift_deleti  on | exon3 | c.3576delC | p.S1192fs | . | . |
| CTC5 | 17 | 17699992 | CGCA | C | 0.122 | NM_030665 | RAI1 | nonframeshift_de  letion | exon3 | c.3731_3733del | p.1244_1245de  l | . | . |
| CTC5 | 17 | 18143894 | GC | G | 0.75 | NM_004140 | LLGL1 | frameshift_deleti  on | exon17 | c.2210delC | p.A737fs | . | . |

| CTC5 | 17 | 18154690 | CACG | C | 0.667 | NM_001256265 | FLII | nonframeshift_de  letion | exon12 | c.1317_1319del | p.439_440del | . | . |
| --- | --- | --- | --- | --- | --- | --- | --- | --- | --- | --- | --- | --- | --- |
| CTC5 | 17 | 18196149 | CG | C | 0.348 | NM_004618 | TOP3A | frameshift_deleti  on | exon11 | c.1090delC | p.R364fs | . | . |
| CTC5 | 17 | 18671964 | TG | T | 0.786 | NM_001267585 | FBXW10 | frameshift_deleti  on | exon10 | c.1823delG | p.C608fs | . | . |
| CTC5 | 17 | 18814544 | AC | A | 0.455 | NM_001243936 | PRPSAP2 | frameshift_deleti  on | exon7 | c.594delC | p.H198fs | . | . |
| CTC5 | 17 | 18880171 | TC | T | 0.892 | NM_001270648 | SLC5A10 | frameshift_deleti  on | exon8 | c.771delC | p.I257fs | . | . |
| CTC5 | 17 | 18880910 | TC | T | 0.941 | NM_001039999 | FAM83G | frameshift_deleti  on | exon5 | c.2068delG | p.D690fs | . | . |
| CTC5 | 17 | 19458975 | G | GA | 0.963 | NM_018242 | SLC47A1 | frameshift_inserti  on | exon8 | c.712dupA | p.G237fs | . | . |
| CTC5 | 17 | 19705201 | T | TG | 0.286 | NM_001142610 | ULK2 | frameshift_inserti  on | exon16 | c.1329dupC | p.M444fs | . | . |
| CTC5 | 17 | 25919586 | CA | C | 0.216 | NM_014238 | KSR1 | frameshift_deleti  on | exon9 | c.823delA | p.N275fs | . | . |
| CTC5 | 17 | 26109098 | TC | T | 0.711 | NM_000625 | NOS2 | frameshift_deleti  on | exon7 | c.664delG | p.E222fs | . | . |
| CTC5 | 17 | 26692224 | A | AG | 1 | NM_001080837 | SEBOX | frameshift_inserti  on | exon1 | c.27dupC | p.W10fs | . | . |
| CTC5 | 17 | 26696950 | TG | T | 0.692 | NM_000638 | VTN | frameshift_deleti  on | exon2 | c.181delC | p.Q61fs | . | . |
| CTC5 | 17 | 27049867 | TG | T | 1 | NM_000984 | RPL23A | stopgain | exon3 | c.337delG | p.V113X | . | . |
| CTC5 | 17 | 27614123 | CCA | C | 0.483 | NM_020772 | NUFIP2 | frameshift_deleti on | exon2 | c.887_888del | p.V296fs | . | . |
| CTC5 | 17 | 27614467 | TC | T | 0.989 | NM_020772 | NUFIP2 | frameshift_deleti  on | exon2 | c.544delG | p.D182fs | . | . |
| CTC5 | 17 | 28029997 | TGG | T | 0.962 | NM_001282131 | SSH2 | frameshift_deleti  on | exon2 | c.210_211del | p.S70fs | . | . |
| CTC5 | 17 | 29662043 | TG | T | 1 | NM_000267 | NF1 | frameshift_deleti  on | exon39 | c.5938delG | p.G1980fs | . | . |
| CTC5 | 17 | 30621404 | AC | A | 0.963 | NM_138328 | RHBDL3 | frameshift_deleti  on | exon5 | c.612delC | p.H204fs | . | . |
| CTC5 | 17 | 30692464 | AC | A | 0.375 | NM_003457 | ZNF207 | frameshift_deleti  on | exon7 | c.739delC | p.Q247fs | . | . |
| CTC5 | 17 | 33316501 | TG | T | 0.59 | NM_002311 | LIG3 | frameshift_deleti  on | exon4 | c.709delG | p.G237fs | . | . |
| CTC5 | 17 | 33772601 | CT | C | 0.941 | NM_144682 | SLFN13 | frameshift_deleti  on | exon3 | c.98delA | p.K33fs | . | . |
| CTC5 | 17 | 34252551 | CA | C | 0.917 | NM_001163122 | RDM1 | frameshift_deleti  on | exon2 | c.297delT | p.F99fs | . | . |
| CTC5 | 17 | 35830631 | TG | T | 0.35 | NM_001166105 | TADA2A | frameshift_deleti  on | exon13 | c.1024delG | p.D342fs | . | . |
| CTC5 | 17 | 36484468 | G | GA | 0.214 | NM_001004334 | GPR179 | frameshift_inserti  on | exon11 | c.4983_4984ins  T | p.Q1662fs | . | . |
| CTC5 | 17 | 36623278 | GC | G | 1 | NM_001199417 | ARHGAP23 | frameshift_deleti  on | exon7 | c.1355delC | p.A452fs | . | . |
| CTC5 | 17 | 36876791 | TG | T | 0.135 | NM_005937 | MLLT6 | frameshift_deleti  on | exon15 | c.2323delG | p.V775fs | . | . |
| CTC5 | 17 | 36927403 | AT | A | 0.176 | NM_003559 | PIP4K2B | frameshift_deleti  on | exon8 | c.929delA | p.D310fs | . | . |
| CTC5 | 17 | 37565039 | AT | A | 0.85 | NM_004774 | MED1 | frameshift_deleti  on | exon17 | c.3434delA | p.N1145fs | . | . |
| CTC5 | 17 | 37565500 | GT | G | 0.548 | NM_004774 | MED1 | frameshift_deleti  on | exon17 | c.2973delA | p.K991fs | . | . |
| CTC5 | 17 | 37819104 | GT | G | 0.357 | NM_001165938 | STARD3 | frameshift_deleti  on | exon14 | c.1228delT | p.F410fs | . | . |
| CTC5 | 17 | 38421131 | TC | T | 0.476 | NM_133264 | WIPF2 | frameshift_deleti  on | exon5 | c.704delC | p.S235fs | . | . |
| CTC5 | 17 | 39324229 | T | TGCAG  CAGGT | 0.6 | NM_033187 | KRTAP4-3 | nonframeshift_in  sertion | exon1 | c.195_196insCT  GACCACCTGC | p.R66delinsLTT  CCR | . | . |
| CTC5 | 17 | 39324232 | A | AGCAG  GTGGT | 0.538 | NM_033187 | KRTAP4-3 | nonframeshift_in  sertion | exon1 | c.192_193insA  GCCTGACCAC | p.C65delinsSLT  TCC | . | . |
| CTC5 | 17 | 39324234 | C | CAGGT  GGTCA | 0.449 | NM_033187 | KRTAP4-3 | nonframeshift_in  sertion | exon1 | c.190_191insAC  AGCCTGACCA | p.C64delinsYSL  TTC | . | . |
| CTC5 | 17 | 39324235 | A | AGGTG  GTCAG | 0.511 | NM_033187 | KRTAP4-3 | nonframeshift_in  sertion | exon1 | c.189_190insC  GCTGCCTGAC | p.C64delinsRCL  TTC | . | . |
| CTC5 | 17 | 39341064 | AG | A | 0.962 | NM_033060 | KRTAP4-1 | frameshift_deleti  on | exon1 | c.42delC | p.G14fs | . | . |
| CTC5 | 17 | 39341065 | GC | G | 0.961 | NM_033060 | KRTAP4-1 | frameshift_deleti  on | exon1 | c.41delG | p.G14fs | . | . |
| CTC5 | 17 | 39346595 | T | TGCTGT  GGGTC CAGCT GCTGC | 1 | NM_001190460 | KRTAP9-1 | nonframeshift_in sertion | exon1 | c.457_458insG  CTGTGGGTCC AGCTGCTGCC AGCCTA | p.C153delinsC CGSSCCQPS | . | . |
| CTC5 | 17 | 39346597 | C | CTGTG  GGTCC AGCTG CTGCC | 0.925 | NM_001190460 | KRTAP9-1 | nonframeshift_in sertion | exon1 | c.459_460insTG  TGGGTCCAGC TGCTGCCAGC CTAGA | p.C153delinsC CGSSCCQPR | . | . |
| CTC5 | 17 | 39595079 | TC | T | 0.326 | NM_006771 | KRT38 | frameshift_deleti  on | exon4 | c.763delG | p.E255fs | . | . |
| CTC5 | 17 | 39684319 | AG | A | 0.385 | NM_002276 | KRT19 | frameshift_deleti  on | exon1 | c.180delC | p.A60fs | . | . |
| CTC5 | 17 | 39767125 | AGC | A | 0.139 | NM_005557 | KRT16 | frameshift_deleti  on | exon5 | c.1038_1039del | p.E346fs | . | . |
| CTC5 | 17 | 39767126 | GC | G | 0.192 | NM_005557 | KRT16 | frameshift_deleti  on | exon5 | c.1038delG | p.E346fs | . | . |
| CTC5 | 17 | 40263866 | CA | C | 0.194 | NM_024119 | DHX58 | frameshift_deleti  on | exon3 | c.44delT | p.L15fs | . | . |
| CTC5 | 17 | 40738825 | GA | G | 0.389 | NM_178126 | FAM134C | frameshift_deleti  on | exon4 | c.461delT | p.F154fs | . | . |
| CTC5 | 17 | 40823434 | TA | T | 1 | NM_024927 | PLEKHH3 | stopgain | exon8 | c.1218delT | p.C406X | . | . |
| CTC5 | 17 | 41365264 | TC | T | 0.438 | NM_145041 | TMEM106A | frameshift_deleti on | exon3 | c.205delC | p.P69fs | . | . |
| CTC5 | 17 | 42083958 | GC | G | 0.405 | NM_153006 | NAGS | frameshift_deleti  on | exon4 | c.978delC | p.S326fs | . | . |
| CTC5 | 17 | 42153236 | TG | T | 0.192 | NM_138387 | G6PC3 | frameshift_deleti  on | exon6 | c.867delG | p.L289fs | . | . |
| CTC5 | 17 | 42287060 | AT | A | 0.177 | NM_001076683 | UBTF | frameshift_deleti  on | exon15 | c.1556delA | p.N519fs | . | . |
| CTC5 | 17 | 42452092 | A | AT | 0.156 | NM_000419 | ITGA2B | frameshift_inserti  on | exon28 | c.2877dupA | p.W960fs | . | . |
| CTC5 | 17 | 42476687 | TG | T | 0.204 | NM_001002909 | GPATCH8 | frameshift_deleti  on | exon8 | c.2757delC | p.S919fs | . | . |
| CTC5 | 17 | 42936478 | CA | C | 0.287 | NM_001142605 | EFTUD2 | frameshift_deleti  on | exon18 | c.1826delT | p.L609fs | . | . |
| CTC5 | 17 | 42987619 | CT | C | 1 | NM_001131019 | GFAP | frameshift_deleti  on | exon8 | c.1180delA | p.S394fs | . | . |
| CTC5 | 17 | 43227601 | GC | G | 0.267 | NM_006460 | HEXIM1 | frameshift_deleti  on | exon1 | c.1045delC | p.Q349fs | . | . |

| CTC5 | 17 | 43516844 | TG | T | 0.8 | NM_014798 | PLEKHM1 | frameshift_deleti  on | exon11 | c.3057delC | p.V1019fs | . | . |
| --- | --- | --- | --- | --- | --- | --- | --- | --- | --- | --- | --- | --- | --- |
| CTC5 | 17 | 44249435 | TG | T | 0.351 | NM_001193466 | KANSL1 | frameshift_deleti  on | exon2 | c.74delC | p.P25fs | . | . |
| CTC5 | 17 | 45695761 | AG | A | 1 | NM_006310 | NPEPPS | frameshift_deleti  on | exon20 | c.2342delG | p.R781fs | . | . |
| CTC5 | 17 | 46024146 | TA | T | 0.555 | NM_018129 | PNPO | stoploss | exon7 | c.785delA | p.X262Y | . | . |
| CTC5 | 17 | 46136093 | AG | A | 0.5 | NM_003204 | NFE2L1 | frameshift_deleti on | exon6 | c.1410delG | p.E470fs | . | . |
| CTC5 | 17 | 46700296 | AT | A | 0.71 | NM_024017 | HOXB9 | stopgain | exon2 | c.718delA | p.M240X | . | . |
| CTC5 | 17 | 46874559 | AT | A | 0.4 | NM_001130918 | TTLL6 | frameshift_deleti on | exon7 | c.775delA | p.I259fs | . | . |
| CTC5 | 17 | 48268847 | TC | T | 0.325 | NM_000088 | COL1A1 | frameshift_deleti  on | exon32 | c.2131delG | p.D711fs | . | . |
| CTC5 | 17 | 48557269 | AC | A | 0.78 | NM_018346 | RSAD1 | frameshift_deleti  on | exon3 | c.299delC | p.T100fs | . | . |
| CTC5 | 17 | 48560025 | TG | T | 0.193 | NM_018346 | RSAD1 | stopgain | exon5 | c.863delG | p.W288X | . | . |
| CTC5 | 17 | 48594977 | GC | G | 0.636 | NM_032133 | MYCBPAP | frameshift_deleti on | exon4 | c.516delC | p.G172fs | . | . |
| CTC5 | 17 | 48776958 | AG | A | 1 | NM_052855 | ANKRD40 | frameshift_deleti  on | exon3 | c.579delC | p.P193fs | . | . |
| CTC5 | 17 | 48917639 | GC | G | 0.963 | NM_175575 | WFIKKN2 | frameshift_deleti  on | exon2 | c.991delC | p.P331fs | . | . |
| CTC5 | 17 | 55072361 | AC | A | 0.333 | NM_021626 | SCPEP1 | frameshift_deleti  on | exon7 | c.640delC | p.P214fs | . | . |
| CTC5 | 17 | 55189254 | CA | C | 0.432 | NM_003488 | AKAP1 | frameshift_deleti  on | exon4 | c.1945delA | p.T649fs | . | . |
| CTC5 | 17 | 55945550 | AT | A | 0.228 | NM_001271875 | CUEDC1 | stopgain | exon8 | c.994delA | p.M332X | . | . |
| CTC5 | 17 | 56277007 | C | CA | 0.6 | NM_000502 | EPX | frameshift_inserti on | exon9 | c.1390dupA | p.A463fs | . | . |
| CTC5 | 17 | 56329857 | C | CT | 0.273 | NM_001160102 | LPO | frameshift_inserti  on | exon6 | c.847dupT | p.C282fs | . | . |
| CTC5 | 17 | 58022768 | GT | G | 0.333 | NM_001272042 | RPS6KB1 | frameshift_deleti  on | exon13 | c.1161delT | p.G387fs | . | . |
| CTC5 | 17 | 58756886 | GC | G | 0.614 | NM_001099432 | BCAS3 | frameshift_deleti  on | exon2 | c.69delC | p.R23fs | . | . |
| CTC5 | 17 | 59926539 | TC | T | 0.5 | NM_032043 | BRIP1 | frameshift_deleti  on | exon5 | c.457delG | p.D153fs | . | . |
| CTC5 | 17 | 61560898 | CA | C | 0.333 | NM_000789 | ACE | frameshift_deleti  on | exon10 | c.1566delA | p.P522fs | . | . |
| CTC5 | 17 | 61684760 | CA | C | 0.295 | NM_016360 | TACO1 | frameshift_deleti  on | exon4 | c.630delA | p.A210fs | . | . |
| CTC5 | 17 | 61890735 | AT | A | 0.385 | NM_203499 | DDX42 | frameshift_deleti  on | exon15 | c.1824delT | p.N608fs | . | . |
| CTC5 | 17 | 63533750 | GT | G | 0.527 | NM_004655 | AXIN2 | frameshift_deleti  on | exon6 | c.1403delA | p.D468fs | . | . |
| CTC5 | 17 | 65052195 | GC | G | 0.896 | NM_000727 | CACNG1 | frameshift_deleti  on | exon4 | c.478delC | p.Q160fs | . | . |
| CTC5 | 17 | 65338373 | TA | T | 0.5 | NM_174871 | PSMD12 | frameshift_deleti  on | exon9 | c.1029delT | p.I343fs | . | . |
| CTC5 | 17 | 66521956 | CT | C | 0.533 | NM_001276290 | PRKAR1A | frameshift_deleti  on | exon6 | c.612delT | p.A204fs | . | . |
| CTC5 | 17 | 68171646 | AT | A | 0.4 | NM_000891 | KCNJ2 | frameshift_deleti  on | exon2 | c.467delT | p.I156fs | . | . |
| CTC5 | 17 | 70943929 | GC | G | 0.182 | NM_001159770 | SLC39A11 | frameshift_deleti  on | exon5 | c.391delG | p.A131fs | . | . |
| CTC5 | 17 | 71248793 | TC | T | 0.6 | NM_001129885 | CPSF4L | frameshift_deleti  on | exon5 | c.472delG | p.E158fs | . | . |
| CTC5 | 17 | 71427673 | AC | A | 1 | NM_001144952 | SDK2 | frameshift_deleti  on | exon11 | c.1447delG | p.V483fs | . | . |
| CTC5 | 17 | 72945392 | GA | G | 0.26 | NM_001272005 | OTOP3 | frameshift_deleti  on | exon7 | c.1619delA | p.E540fs | . | . |
| CTC5 | 17 | 73499249 | CG | C | 0.276 | NM_001142643 | CASKIN2 | frameshift_deleti  on | exon17 | c.1659delC | p.G553fs | . | . |
| CTC5 | 17 | 74288564 | CTGA | C | 1 | NM_032134 | QRICH2 | nonframeshift_de  letion | exon4 | c.1743_1745del | p.581_582del | . | . |
| CTC5 | 17 | 74570619 | GC | G | 0.667 | NM_006456 | ST6GALNAC  2 | frameshift_deleti  on | exon3 | c.188delG | p.G63fs | . | . |
| CTC5 | 17 | 74944135 | TC | T | 0.652 | NM_198955 | MGAT5B | frameshift_deleti  on | exon15 | c.2175delC | p.F725fs | . | . |
| CTC5 | 17 | 76471483 | AT | A | 0.909 | NM_173628 | DNAH17 | frameshift_deleti  on | exon54 | c.8387delA | p.N2796fs | . | . |
| CTC5 | 17 | 76471485 | TC | T | 0.889 | NM_173628 | DNAH17 | frameshift_deleti  on | exon54 | c.8385delG | p.G2795fs | . | . |
| CTC5 | 17 | 76482131 | CA | C | 0.389 | NM_173628 | DNAH17 | frameshift_deleti  on | exon46 | c.7185delT | p.F2395fs | . | . |
| CTC5 | 17 | 76676339 | TC | T | 0.674 | NM_004762 | CYTH1 | frameshift_deleti  on | exon12 | c.1050delG | p.G350fs | . | . |
| CTC5 | 17 | 78086696 | TG | T | 0.947 | NM_000152 | GAA | frameshift_deleti  on | exon14 | c.1911delG | p.L637fs | . | . |
| CTC5 | 17 | 79172019 | GC | G | 0.978 | NM_001009811 | AZI1 | frameshift_deleti  on | exon12 | c.1365delG | p.G455fs | . | . |
| CTC5 | 17 | 79660698 | GGACGA | G | 0.8 | NM_004712 | HGS | frameshift_deleti  on | exon10 | c.757_761del | p.D253fs | . | . |
| CTC5 | 18 | 335178 | AG | A | 0.345 | NM_130386 | COLEC12 | frameshift_deleti  on | exon6 | c.1379delC | p.P460fs | . | . |
| CTC5 | 18 | 7955252 | TG | T | 0.33 | NM_001105244 | PTPRM | frameshift_deleti  on | exon7 | c.973delG | p.G325fs | . | . |
| CTC5 | 18 | 9256130 | CA | C | 0.692 | NM_001083625 | ANKRD12 | frameshift_deleti  on | exon8 | c.2797delA | p.R933fs | . | . |
| CTC5 | 18 | 11753675 | TA | T | 0.444 | NM_182978 | GNAL | frameshift_deleti  on | exon3 | c.499delA | p.I167fs | . | . |
| CTC5 | 18 | 13068158 | TC | T | 0.867 | NM_032142 | CEP192 | frameshift_deleti  on | exon23 | c.4681delC | p.P1561fs | . | . |
| CTC5 | 18 | 19070081 | AC | A | 0.867 | NM_001142966 | GREB1L | frameshift_deleti  on | exon19 | c.2800delC | p.P934fs | . | . |
| CTC5 | 18 | 19359545 | A | AT | 0.273 | NM_020774 | MIB1 | frameshift_inserti  on | exon6 | c.807_808insT | p.G269fs | . | . |
| CTC5 | 18 | 20577596 | CA | C | 0.8 | NM_002894 | RBBP8 | frameshift_deleti  on | exon14 | c.2043delA | p.S681fs | . | . |
| CTC5 | 18 | 28666594 | GA | G | 0.684 | NM_004949 | DSC2 | frameshift_deleti  on | exon7 | c.886delT | p.S296fs | . | . |
| CTC5 | 18 | 29178556 | GCC | G | 0.258 | NM_000371 | TTR | frameshift_deleti  on | exon4 | c.363_364del | p.G121fs | . | . |
| CTC5 | 18 | 29867064 | GT | G | 1 | NM_001242409 | GAREM | frameshift_deleti  on | exon4 | c.1495delA | p.T499fs | . | . |
| CTC5 | 18 | 31324269 | C | CT | 0.256 | NM_030632 | ASXL3 | frameshift_inserti  on | exon12 | c.4458dupT | p.S1486fs | . | . |
| CTC5 | 18 | 34261458 | CAG | C | 0.394 | NM_001281739 | FHOD3 | frameshift_deleti  on | exon12 | c.1371_1372del | p.A457fs | . | . |
| CTC5 | 18 | 34802137 | AC | A | 0.6 | NM_020776 | KIAA1328 | frameshift_deleti  on | exon10 | c.1682delC | p.T561fs | . | . |

| CTC5 | 18 | 43249270 | GC | G | 0.312 | NM_007163 | SLC14A2 | frameshift_deleti  on | exon16 | c.2037delC | p.C679fs | . | . |
| --- | --- | --- | --- | --- | --- | --- | --- | --- | --- | --- | --- | --- | --- |
| CTC5 | 18 | 43316442 | GA | G | 0.257 | NM_001146037 | SLC14A1 | frameshift_deleti  on | exon5 | c.661delA | p.N221fs | . | . |
| CTC5 | 18 | 43833701 | T | TCTG | 0.857 | NM_001008239 | C18orf25 | nonframeshift_in  sertion | exon3 | c.754_755insCT  G | p.S252delinsSA | . | . |
| CTC5 | 18 | 47799805 | AG | A | 0.2 | NM_001204141 | MBD1 | frameshift_deleti  on | exon12 | c.1332delC | p.A444fs | . | . |
| CTC5 | 18 | 47812274 | TCTG | T | 0.273 | NM_001101654 | CXXC1 | nonframeshift_de  letion | exon5 | c.481_483del | p.161_161del | . | . |
| CTC5 | 18 | 47812513 | AC | A | 1 | NM_001101654 | CXXC1 | frameshift_deleti  on | exon4 | c.337delG | p.V113fs | . | . |
| CTC5 | 18 | 52556639 | TG | T | 1 | NM_004163 | RAB27B | frameshift_deleti  on | exon6 | c.653delG | p.C218fs | . | . |
| CTC5 | 18 | 56985545 | CT | C | 0.42 | NM_181654 | CPLX4 | frameshift_deleti  on | exon1 | c.149delA | p.K50fs | . | . |
| CTC5 | 18 | 60036551 | AC | A | 1 | NM_001278268 | TNFRSF11A | frameshift_deleti  on | exon9 | c.1360delC | p.P454fs | . | . |
| CTC5 | 18 | 60242000 | AC | A | 0.247 | NM_017742 | ZCCHC2 | frameshift_deleti  on | exon13 | c.2687delC | p.T896fs | . | . |
| CTC5 | 18 | 61600349 | CA | C | 0.833 | NM_005024 | SERPINB10 | frameshift_deleti  on | exon6 | c.702delA | p.P234fs | . | . |
| CTC5 | 18 | 65180918 | TC | T | 0.207 | NM_032160 | DSEL | frameshift_deleti  on | exon2 | c.957delG | p.K319fs | . | . |
| CTC5 | 18 | 67727201 | CA | C | 0.448 | NM_173630 | RTTN | frameshift_deleti  on | exon36 | c.4824delT | p.F1608fs | . | . |
| CTC5 | 18 | 67863850 | T | TCTC | 1 | NM_173630 | RTTN | nonframeshift_in  sertion | exon7 | c.727_728insG  AG | p.D243delinsG  D | . | . |
| CTC5 | 18 | 72223591 | G | GTGC | 0.929 | NM_032649 | CNDP1 | nonframeshift_in  sertion | exon2 | c.43_44insTGC | p.V15delinsVL | . | . |
| CTC5 | 18 | 72223606 | C | CTGT | 0.846 | NM_032649 | CNDP1 | nonframeshift_in  sertion | exon2 | c.58_59insTGT | p.L20delinsLL | . | . |
| CTC5 | 18 | 72345011 | GA | G | 0.571 | NM_001146189 | ZNF407 | frameshift_deleti  on | exon1 | c.2037delA | p.G679fs | . | . |
| CTC5 | 18 | 74082488 | TG | T | 1 | NM_014643 | ZNF516 | frameshift_deleti  on | exon7 | c.3426delC | p.P1142fs | . | . |
| CTC5 | 18 | 74091958 | AC | A | 1 | NM_014643 | ZNF516 | frameshift_deleti  on | exon4 | c.2111delG | p.G704fs | . | . |
| CTC5 | 18 | 76936903 | TG | T | 0.5 | NM_198531 | ATP9B | frameshift_deleti  on | exon8 | c.870delG | p.L290fs | . | . |
| CTC5 | 18 | 77475187 | TGGA | T | 0.333 | NM_001202504 | CTDP1 | nonframeshift_de  letion | exon8 | c.1371_1373del | p.457_458del | . | . |
| CTC5 | 19 | 1827020 | TGGA | T | 0.231 | NM_020695 | REXO1 | nonframeshift_de  letion | exon2 | c.1765_1767del | p.589_589del | . | . |
| CTC5 | 19 | 4816490 | G | GC | 0.267 | NM_182919 | TICAM1 | frameshift_inserti  on | exon2 | c.1899dupG | p.H634fs | . | . |
| CTC5 | 19 | 4852267 | G | GC | 0.56 | NM_001164189 | PLIN3 | frameshift_inserti  on | exon5 | c.394dupG | p.A132fs | . | . |
| CTC5 | 19 | 5208307 | GC | G | 0.455 | NM_130853 | PTPRS | frameshift_deleti  on | exon27 | c.4241delG | p.G1414fs | . | . |
| CTC5 | 19 | 5751734 | CA | C | 0.3 | NM_152784 | CATSPERD | frameshift_deleti  on | exon12 | c.1065delA | p.P355fs | . | . |
| CTC5 | 19 | 6222400 | CGGGGT  G | C | 0.4 | NM_005934 | MLLT1 | nonframeshift_de  letion | exon6 | c.836_841del | p.279_281del | . | . |
| CTC5 | 19 | 6903891 | CA | C | 0.17 | NM_001256254 | EMR1 | frameshift_deleti  on | exon4 | c.310delA | p.T104fs | . | . |
| CTC5 | 19 | 7625596 | GA | G | 0.488 | NM_001166114 | PNPLA6 | frameshift_deleti  on | exon29 | c.3763delA | p.K1255fs | . | . |
| CTC5 | 19 | 7626411 | TG | T | 0.96 | NM_001166114 | PNPLA6 | frameshift_deleti  on | exon32 | c.4062delG | p.L1354fs | . | . |
| CTC5 | 19 | 7755391 | GC | G | 0.5 | NM_001207019 | FCER2 | frameshift_deleti  on | exon8 | c.518delG | p.C173fs | . | . |
| CTC5 | 19 | 8028495 | TG | T | 1 | NM_001419 | ELAVL1 | stopgain | exon6 | c.852delC | p.C284X | . | . |
| CTC5 | 19 | 8174578 | CG | C | 0.421 | NM_032447 | FBN3 | frameshift_deleti on | exon34 | c.4392delC | p.P1464fs | . | . |
| CTC5 | 19 | 9002656 | GC | G | 0.5 | NM_024690 | MUC16 | frameshift_deleti  on | exon51 | c.40159delG | p.A13387fs | . | . |
| CTC5 | 19 | 9072893 | GT | G | 0.748 | NM_024690 | MUC16 | frameshift_deleti  on | exon3 | c.14552delA | p.Y4851fs | . | . |
| CTC5 | 19 | 9361854 | CG | C | 0.429 | NM_001079935 | OR7E24 | frameshift_deleti  on | exon1 | c.136delG | p.A46fs | . | . |
| CTC5 | 19 | 10204141 | T | TG | 0.286 | NM_031917 | ANGPTL6 | frameshift_inserti  on | exon5 | c.1105_1106ins  C | p.E369fs | . | . |
| CTC5 | 19 | 10395682 | CA | C | 0.75 | NM_000201 | ICAM1 | frameshift_deleti  on | exon6 | c.1405delA | p.K469fs | . | . |
| CTC5 | 19 | 11145715 | TGAGGA  G | T | 1 | NM_001128845 | SMARCA4 | nonframeshift_de  letion | exon27 | c.3979_3984del | p.1327_1328de  l | . | . |
| CTC5 | 19 | 12383284 | C | CT | 0.624 | NM_016264 | ZNF44 | frameshift_inserti  on | exon4 | c.1785dupA | p.A596fs | . | . |
| CTC5 | 19 | 13255373 | TC | T | 0.526 | NM_001271610 | STX10 | frameshift_deleti  on | exon7 | c.690delG | p.G230fs | . | . |
| CTC5 | 19 | 13445192 | CTG | C | 0.337 | NM_001127221 | CACNA1A | frameshift_deleti  on | exon8 | c.1196_1197del | p.A399fs | . | . |
| CTC5 | 19 | 14020740 | G | GC | 0.965 | NM_017721 | CC2D1A | frameshift_inserti  on | exon2 | c.166dupC | p.Q55fs | . | . |
| CTC5 | 19 | 14194341 | TC | T | 0.366 | NM_001277378 | C19orf67 | frameshift_deleti  on | exon3 | c.556delG | p.D186fs | . | . |
| CTC5 | 19 | 14272354 | GT | G | 0.6 | NM_014921 | LPHN1 | frameshift_deleti  on | exon6 | c.1279delA | p.T427fs | . | . |
| CTC5 | 19 | 15073032 | AT | A | 0.29 | NM_005071 | SLC1A6 | frameshift_deleti  on | exon5 | c.716delA | p.N239fs | . | . |
| CTC5 | 19 | 15132253 | C | CA | 1 | NM_173482 | CCDC105 | frameshift_inserti  on | exon4 | c.963_964insA | p.V321fs | . | . |
| CTC5 | 19 | 15574935 | GT | G | 0.6 | NM_022904 | RASAL3 | frameshift_deleti  on | exon2 | c.234delA | p.S78fs | . | . |
| CTC5 | 19 | 15918058 | T | TA | 0.28 | NM_013940 | OR10H1 | stopgain | exon1 | c.789_790insT | p.K264_S265de  linsX | . | . |
| CTC5 | 19 | 16890227 | G | GT | 0.353 | NM_001007525 | NWD1 | frameshift_inserti  on | exon12 | c.2683dupT | p.V894fs | . | . |
| CTC5 | 19 | 17688021 | GA | G | 0.667 | NM_024656 | COLGALT1 | frameshift_deleti  on | exon7 | c.968delA | p.E323fs | . | . |
| CTC5 | 19 | 18648411 | CT | C | 0.608 | NM_012181 | FKBP8 | frameshift_deleti  on | exon6 | c.944delA | p.K315fs | . | . |
| CTC5 | 19 | 18648565 | TC | T | 0.233 | NM_012181 | FKBP8 | frameshift_deleti  on | exon6 | c.790delG | p.E264fs | . | . |
| CTC5 | 19 | 19121027 | TCA | T | 0.203 | NM_001017392 | SUGP2 | frameshift_deleti  on | exon5 | c.1973_1974del | p.V658fs | . | . |
| CTC5 | 19 | 19653201 | TG | T | 0.543 | NM_153221 | CILP2 | frameshift_deleti  on | exon5 | c.611delG | p.C204fs | . | . |
| CTC5 | 19 | 19655422 | TTG | T | 0.217 | NM_153221 | CILP2 | frameshift_deleti  on | exon8 | c.2069_2070del | p.L690fs | . | . |
| CTC5 | 19 | 19656228 | A | AG | 0.454 | NM_153221 | CILP2 | frameshift_inserti  on | exon8 | c.2875dupG | p.A958fs | . | . |

| CTC5 | 19 | 19656805 | CG | C | 0.562 | NM_153221 | CILP2 | frameshift_deleti  on | exon8 | c.3452delG | p.R1151fs | . | . |
| --- | --- | --- | --- | --- | --- | --- | --- | --- | --- | --- | --- | --- | --- |
| CTC5 | 19 | 20307870 | TG | T | 0.444 | NM_052852 | ZNF486 | frameshift_deleti  on | exon4 | c.352delG | p.G118fs | . | . |
| CTC5 | 19 | 20807177 | G | GA | 0.667 | NM_001076675 | ZNF626 | frameshift_inserti  on | exon4 | c.1505dupT | p.I502fs | . | . |
| CTC5 | 19 | 21299774 | T | TATA | 0.893 | NM_182515 | ZNF714 | nonframeshift_in  sertion | exon5 | c.304_305insAT  A | p.Y102delinsYN | . | . |
| CTC5 | 19 | 22364167 | AT | A | 0.369 | NM_001001411 | ZNF676 | frameshift_deleti  on | exon3 | c.351delA | p.K117fs | . | . |
| CTC5 | 19 | 23927741 | CCA | C | 1 | NM_138286 | ZNF681 | frameshift_deleti  on | exon4 | c.609_610del | p.C203fs | . | . |
| CTC5 | 19 | 35175076 | TAAAAA  AAAAAA AAAAAA AAAAAA | T | 1 | NM_001289188 | ZNF302 | frameshift_deleti on | exon5 | c.398_691del | p.*133fs | . | . |
| CTC5 | 19 | 35175076 | TAAAAA  AAAAAA AAAAAA AAAAAA | T | 1 | NM_001289188 | ZNF302 | frameshift_deleti on | exon5 | c.398_691del | p.*133fs | . | . |
| CTC5 | 19 | 35612167 | CTT | C | 0.538 | NM_001136010 | FXYD3 | frameshift_deleti  on | exon5 | c.116_117del | p.L39fs | . | . |
| CTC5 | 19 | 36002361 | ACTGCT  GCCACC ACTGCT GCCGCC ACTGCT GCCGCC | A | 0.929 | NM_001126057 | DMKN | nonframeshift_de letion | exon5 | c.819_869del | p.273_290del | . | . |
| CTC5 | 19 | 36210381 | GT | G | 0.294 | NM_014727 | KMT2B | frameshift_deleti  on | exon2 | c.375delT | p.G125fs | . | . |
| CTC5 | 19 | 36223214 | AGG | A | 0.2 | NM_014727 | KMT2B | frameshift_deleti  on | exon28 | c.5765_5766del | p.R1922fs | . | . |
| CTC5 | 19 | 36258936 | CA | C | 1 | NM_001039887 | C19orf55 | frameshift_deleti  on | exon9 | c.1190delA | p.Q397fs | . | . |
| CTC5 | 19 | 37117185 | CA | C | 0.643 | NM_001256838 | ZNF382 | frameshift_deleti  on | exon5 | c.384delA | p.S128fs | . | . |
| CTC5 | 19 | 37240884 | AT | A | 0.2 | NM_001267779 | ZNF850 | frameshift_deleti  on | exon4 | c.961delA | p.I321fs | . | . |
| CTC5 | 19 | 37310260 | TG | T | 1 | NM_001242800 | ZNF790 | frameshift_deleti  on | exon5 | c.985delC | p.Q329fs | . | . |
| CTC5 | 19 | 38103869 | G | GT | 0.174 | NM_001172226 | ZNF540 | frameshift_inserti  on | exon4 | c.1593dupT | p.R531fs | . | . |
| CTC5 | 19 | 38377977 | GT | G | 0.655 | NM_031951 | WDR87 | frameshift_deleti  on | exon6 | c.6216delA | p.K2072fs | . | . |
| CTC5 | 19 | 38378169 | T | TTC | 0.12 | NM_031951 | WDR87 | frameshift_inserti  on | exon6 | c.6024_6025ins  GA | p.K2009fs | . | . |
| CTC5 | 19 | 38597212 | TA | T | 1 | NM_015073 | SIPA1L3 | frameshift_deleti  on | exon7 | c.2066delA | p.Y689fs | . | . |
| CTC5 | 19 | 38795546 | TGAA | T | 1 | NM_033520 | C19orf33 | nonframeshift_de  letion | exon4 | c.264_266del | p.88_89del | . | . |
| CTC5 | 19 | 38968394 | CGTGTG  GGCG | C | 0.359 | NM_000540 | RYR1 | nonframeshift_de  letion | exon30 | c.4339_4347del | p.1447_1449de  l | . | . |
| CTC5 | 19 | 39208571 | TCAA | T | 1 | NM_004924 | ACTN4 | nonframeshift_de  letion | exon11 | c.1149_1151del | p.383_384del | . | . |
| CTC5 | 19 | 39401251 | CTGG | C | 0.333 | NM_001243212 | CCER2 | nonframeshift_de  letion | exon4 | c.660_662del | p.220_221del | . | . |
| CTC5 | 19 | 39964134 | AC | A | 0.399 | NM_001130825 | SUPT5H | frameshift_deleti  on | exon24 | c.2454delC | p.D818fs | . | . |
| CTC5 | 19 | 39972632 | TG | T | 0.277 | NM_001001563 | TIMM50 | frameshift_deleti  on | exon2 | c.528delG | p.L176fs | . | . |
| CTC5 | 19 | 40412003 | CG | C | 0.362 | NM_003890 | FCGBP | frameshift_deleti  on | exon7 | c.3624delC | p.P1208fs | . | . |
| CTC5 | 19 | 40828225 | CA | C | 0.786 | NM_001256440 | C19orf47 | frameshift_deleti  on | exon9 | c.832delT | p.W278fs | . | . |
| CTC5 | 19 | 41086682 | G | GT | 0.948 | NM_138392 | SHKBP1 | frameshift_inserti  on | exon9 | c.685dupT | p.V228fs | . | . |
| CTC5 | 19 | 42596352 | GC | G | 0.484 | NM_001207025 | POU2F2 | frameshift_deleti  on | exon13 | c.1268delG | p.G423fs | . | . |
| CTC5 | 19 | 42840383 | GA | G | 0.159 | NM_001271938 | MEGF8 | frameshift_deleti  on | exon6 | c.1130delA | p.E377fs | . | . |
| CTC5 | 19 | 42865132 | CT | C | 0.6 | NM_001410 | MEGF8 | frameshift_deleti  on | exon31 | c.5494delT | p.C1832fs | . | . |
| CTC5 | 19 | 43097980 | GC | G | 0.254 | NM_001816 | CEACAM8 | frameshift_deleti  on | exon2 | c.136delG | p.A46fs | . | . |
| CTC5 | 19 | 43585289 | CA | C | 0.245 | NM_031246 | PSG2 | frameshift_deleti  on | exon2 | c.173delT | p.L58fs | . | . |
| CTC5 | 19 | 44223309 | AG | A | 0.5 | NM_019612 | IRGC | frameshift_deleti  on | exon2 | c.600delG | p.E200fs | . | . |
| CTC5 | 19 | 44652989 | TG | T | 0.882 | NM_001144824 | ZNF234 | frameshift_deleti  on | exon4 | c.82delG | p.V28fs | . | . |
| CTC5 | 19 | 44832057 | G | GT | 0.444 | NM_013380 | ZNF112 | frameshift_inserti  on | exon4 | c.2252_2253ins  A | p.G751fs | . | . |
| CTC5 | 19 | 45028255 | GC | G | 0.273 | NM_001102597 | CEACAM20 | frameshift_deleti  on | exon3 | c.235delG | p.A79fs | . | . |
| CTC5 | 19 | 45381748 | TGAG | T | 0.212 | NM_002856 | PVRL2 | nonframeshift_de  letion | exon6 | c.1312_1314del | p.438_438del | . | . |
| CTC5 | 19 | 45735056 | GC | G | 1 | NM_138568 | EXOC3L2 | frameshift_deleti  on | exon2 | c.54delG | p.L18fs | . | . |
| CTC5 | 19 | 45895504 | AG | A | 0.333 | NM_001142502 | PPP1R13L | frameshift_deleti  on | exon8 | c.1448delC | p.P483fs | . | . |
| CTC5 | 19 | 46003736 | GCCC | G | 0.389 | NM_001080401 | PPM1N | nonframeshift_de  letion | exon3 | c.1081_1083del | p.361_361del | . | . |
| CTC5 | 19 | 46215339 | TA | T | 0.826 | NM_001080469 | FBXO46 | frameshift_deleti  on | exon2 | c.1414delT | p.Y472fs | . | . |
| CTC5 | 19 | 47764063 | TG | T | 0.206 | NM_015603 | CCDC9 | frameshift_deleti  on | exon5 | c.430delG | p.G144fs | . | . |
| CTC5 | 19 | 47979929 | GA | G | 0.537 | NM_007059 | KPTN | frameshift_deleti  on | exon11 | c.1041delT | p.L347fs | . | . |
| CTC5 | 19 | 48305612 | TTTGGG  CCTGAG | T | 1 | NM_198479 | TPRX1 | nonframeshift_de  letion | exon2 | c.644_655del | p.215_219del | . | . |
| CTC5 | 19 | 48588336 | CT | C | 1 | NM_001159322 | PLA2G4C | frameshift_deleti  on | exon10 | c.853delA | p.S285fs | . | . |
| CTC5 | 19 | 48675094 | CG | C | 0.6 | NM_199341 | C19orf68 | frameshift_deleti  on | exon2 | c.36delG | p.A12fs | . | . |
| CTC5 | 19 | 48800608 | CA | C | 0.399 | NM_144577 | CCDC114 | stopgain | exon14 | c.1637delT | p.L546X | . | . |
| CTC5 | 19 | 48886565 | CA | C | 0.164 | NM_006801 | KDELR1 | stopgain | exon5 | c.623delT | p.L208X | . | . |
| CTC5 | 19 | 49142619 | GC | G | 0.192 | NM_001217 | CA11 | frameshift_deleti on | exon7 | c.737delG | p.C246fs | . | . |
| CTC5 | 19 | 49362234 | GC | G | 0.412 | NM_001161354 | PLEKHA4 | frameshift_deleti  on | exon8 | c.854delG | p.G285fs | . | . |

| CTC5 | 19 | 49657710 | ACAT | A | 0.8 | NM_002152 | HRC | nonframeshift_de  letion | exon1 | c.782_784del | p.261_262del | . | . |
| --- | --- | --- | --- | --- | --- | --- | --- | --- | --- | --- | --- | --- | --- |
| CTC5 | 19 | 49657751 | ATCG | A | 0.538 | NM_002152 | HRC | nonframeshift_de  letion | exon1 | c.741_743del | p.247_248del | . | . |
| CTC5 | 19 | 49657889 | T | TTCC | 0.833 | NM_002152 | HRC | nonframeshift_in  sertion | exon1 | c.605_606insG  GA | p.E202delinsEE | . | . |
| CTC5 | 19 | 49657900 | C | CCTG | 0.778 | NM_002152 | HRC | nonframeshift_in  sertion | exon1 | c.594_595insCA  G | p.E199delinsQE | . | . |
| CTC5 | 19 | 49993174 | TG | T | 0.275 | NM_012423 | RPL13A | frameshift_deleti  on | exon2 | c.84delG | p.L28fs | . | . |
| CTC5 | 19 | 50314650 | GC | G | 0.961 | NM_001171937 | FUZ | frameshift_deleti  on | exon4 | c.353delG | p.C118fs | . | . |
| CTC5 | 19 | 50399284 | T | TC | 0.762 | NM_152899 | IL4I1 | frameshift_inserti  on | exon3 | c.39_40insG | p.I14fs | . | . |
| CTC5 | 19 | 50510971 | CCTGGT  CTTCTGA GGGCTA | C | 0.506 | NM_001025778 | VRK3 | nonframeshift_de letion | exon4 | c.231_251del | p.77_84del | . | . |
| CTC5 | 19 | 50760640 | CA | C | 0.517 | NM_024729 | MYH14 | frameshift_deleti  on | exon16 | c.2007delA | p.T669fs | . | . |
| CTC5 | 19 | 50862328 | TG | T | 0.341 | NM_004851 | NAPSA | frameshift_deleti  on | exon8 | c.974delC | p.A325fs | . | . |
| CTC5 | 19 | 50945502 | AG | A | 0.417 | NM_004533 | MYBPC2 | frameshift_deleti  on | exon9 | c.835delG | p.E279fs | . | . |
| CTC5 | 19 | 51845252 | AC | A | 1 | NM_001163922 | VSIG10L | stopgain | exon2 | c.49delG | p.V17X | . | . |
| CTC5 | 19 | 51961616 | GGCA | G | 0.167 | NM_014442 | SIGLEC8 | nonframeshift_de letion | exon1 | c.23_25del | p.8_9del | . | . |
| CTC5 | 19 | 52520325 | TA | T | 0.315 | NM_025040 | ZNF614 | frameshift_deleti  on | exon5 | c.525delT | p.T175fs | . | . |
| CTC5 | 19 | 52825130 | C | CA | 0.4 | NM_144684 | ZNF480 | frameshift_inserti  on | exon5 | c.628dupA | p.S209fs | . | . |
| CTC5 | 19 | 52857032 | TG | T | 0.455 | NM_001161425 | ZNF610 | frameshift_deleti  on | exon4 | c.162delG | p.L54fs | . | . |
| CTC5 | 19 | 53209694 | GA | G | 0.333 | NM_001161500 | ZNF611 | frameshift_deleti  on | exon5 | c.613delT | p.S205fs | . | . |
| CTC5 | 19 | 53304735 | AC | A | 0.895 | NM_006969 | ZNF28 | frameshift_deleti  on | exon4 | c.362delG | p.G121fs | . | . |
| CTC5 | 19 | 53644938 | G | GC | 0.169 | NM_001172674 | ZNF347 | frameshift_inserti  on | exon5 | c.1145_1146ins  G | p.A382fs | . | . |
| CTC5 | 19 | 53959887 | AT | A | 0.842 | NM_001289951 | ZNF761 | frameshift_deleti  on | exon6 | c.2127delT | p.H709fs | . | . |
| CTC5 | 19 | 54394932 | C | CA | 0.4 | NM_002739 | PRKCG | frameshift_inserti  on | exon6 | c.534_535insA | p.G178fs | . | . |
| CTC5 | 19 | 54649674 | C | CA | 0.667 | NM_014516 | CNOT3 | frameshift_inserti  on | exon9 | c.733dupA | p.P244fs | . | . |
| CTC5 | 19 | 54649675 | AGC | A | 0.886 | NM_014516 | CNOT3 | frameshift_deleti  on | exon9 | c.734_735del | p.S245fs | . | . |
| CTC5 | 19 | 54649675 | AG | A | 0.886 | NM_014516 | CNOT3 | frameshift_deleti  on | exon9 | c.734delG | p.S245fs | . | . |
| CTC5 | 19 | 55087313 | TC | T | 0.915 | NM_001130917 | LILRA2 | frameshift_deleti  on | exon6 | c.993delC | p.V331fs | . | . |
| CTC5 | 19 | 55333141 | G | GA | 0.533 | NM_013289 | KIR3DL1 | frameshift_inserti  on | exon5 | c.777_778insA | p.G259fs | . | . |
| CTC5 | 19 | 55449590 | G | GA | 0.667 | NM_001127255 | NLRP7 | frameshift_inserti  on | exon5 | c.1950dupT | p.P651fs | . | . |
| CTC5 | 19 | 55450691 | TC | T | 0.24 | NM_001127255 | NLRP7 | frameshift_deleti  on | exon4 | c.1495delG | p.D499fs | . | . |
| CTC5 | 19 | 55526100 | G | GAGGC | 0.759 | NM_001083899 | GP6 | frameshift_inserti  on | exon8 | c.1212_1213ins  GCCT | p.L405fs | . | . |
| CTC5 | 19 | 55526103 | G | GCAGA | 0.97 | NM_001083899 | GP6 | frameshift_inserti  on | exon8 | c.1209_1210ins  TCTG | p.P404fs | . | . |
| CTC5 | 19 | 55526106 | G | GACAT | 0.763 | NM_001083899 | GP6 | frameshift_inserti  on | exon8 | c.1206_1207ins  ATGT | p.L403fs | . | . |
| CTC5 | 19 | 55703106 | AC | A | 0.75 | NM_001161440 | PTPRH | frameshift_deleti  on | exon9 | c.1726delG | p.V576fs | . | . |
| CTC5 | 19 | 56701370 | CG | C | 0.75 | NM_001080456 | ZSCAN5B | frameshift_deleti  on | exon4 | c.1313delC | p.P438fs | . | . |
| CTC5 | 19 | 56895854 | TA | T | 0.262 | NM_144690 | ZNF582 | frameshift_deleti  on | exon5 | c.931delT | p.Y311fs | . | . |
| CTC5 | 19 | 57327509 | AT | A | 0.575 | NM_001146186 | PEG3 | frameshift_deleti  on | exon7 | c.2300delA | p.N767fs | . | . |
| CTC5 | 19 | 57803376 | AC | A | 0.917 | NM_006635 | ZNF460 | frameshift_deleti  on | exon3 | c.1468delC | p.P490fs | . | . |
| CTC5 | 19 | 57839696 | G | GA | 0.677 | NM_213598 | ZNF543 | frameshift_inserti  on | exon4 | c.867dupA | p.G289fs | . | . |
| CTC5 | 19 | 57839720 | CT | C | 0.7 | NM_213598 | ZNF543 | frameshift_deleti  on | exon4 | c.891delT | p.T297fs | . | . |
| CTC5 | 19 | 57868077 | GT | G | 0.25 | NM_020657 | ZNF304 | frameshift_deleti  on | exon3 | c.841delT | p.C281fs | . | . |
| CTC5 | 19 | 57988666 | A | AGCC | 1 | NM_001024596 | ZNF772 | nonframeshift_in  sertion | exon1 | c.11_12insGGC | p.A4delinsAA | . | . |
| CTC5 | 19 | 58058673 | CT | C | 0.644 | NM_001277090 | ZNF550 | frameshift_deleti  on | exon4 | c.938delA | p.K313fs | . | . |
| CTC5 | 19 | 58101845 | CA | C | 0.91 | NM_001010879 | ZIK1 | frameshift_deleti  on | exon4 | c.667delA | p.K223fs | . | . |
| CTC5 | 19 | 58131935 | CA | C | 1 | NM_003435 | ZNF134 | frameshift_deleti  on | exon3 | c.449delA | p.Q150fs | . | . |
| CTC5 | 19 | 58189848 | GA | G | 0.636 | NM_152677 | ZSCAN4 | frameshift_deleti  on | exon5 | c.878delA | p.E293fs | . | . |
| CTC5 | 19 | 58213644 | AT | A | 0.233 | NM_001085384 | ZNF154 | frameshift_deleti  on | exon3 | c.672delA | p.K224fs | . | . |
| CTC5 | 19 | 58490070 | AT | A | 1 | NM_025027 | ZNF606 | frameshift_deleti  on | exon7 | c.1977delA | p.K659fs | . | . |
| CTC5 | 19 | 58500009 | CA | C | 0.455 | NM_025027 | ZNF606 | frameshift_deleti  on | exon5 | c.257delT | p.L86fs | . | . |
| CTC5 | 19 | 58500035 | GC | G | 0.462 | NM_025027 | ZNF606 | frameshift_deleti  on | exon5 | c.231delG | p.G77fs | . | . |
| CTC5 | 19 | 58640663 | CA | C | 0.273 | NM_024620 | ZNF329 | frameshift_deleti  on | exon4 | c.207delT | p.F69fs | . | . |
| CTC5 | 19 | 58899601 | AT | A | 0.913 | NM_001009 | RPS5 | frameshift_deleti  on | exon2 | c.98delT | p.I33fs | . | . |
| CTC5 | 20 | 126310 | ACC | A | 0.8 | NM_030931 | DEFB126 | frameshift_deleti  on | exon2 | c.314_315del | p.T105fs | . | . |
| CTC5 | 20 | 472924 | CT | C | 0.567 | NM_177560 | CSNK2A1 | frameshift_deleti  on | exon7 | c.186delA | p.K62fs | . | . |
| CTC5 | 20 | 2739561 | CT | C | 0.333 | NM_001110514 | EBF4 | frameshift_deleti  on | exon17 | c.1734delT | p.S578fs | . | . |
| CTC5 | 20 | 3781415 | TG | T | 0.833 | NM_001287516 | CDC25B | frameshift_deleti  on | exon6 | c.294delG | p.V98fs | . | . |
| CTC5 | 20 | 3842040 | GC | G | 0.442 | NM_020746 | MAVS | frameshift_deleti  on | exon4 | c.355delC | p.P119fs | . | . |
| CTC5 | 20 | 3888833 | C | CA | 0.667 | NM_024960 | PANK2 | frameshift_inserti  on | exon2 | c.17dupA | p.Q6fs | . | . |
| CTC5 | 20 | 3897653 | AC | A | 0.429 | NM_024960 | PANK2 | frameshift_deleti  on | exon5 | c.620delC | p.T207fs | . | . |

| CTC5 | 20 | 4679885 | TG | T | 0.98 | NM_000311 | PRNP | stopgain | exon2 | c.20delG | p.W7X | . | . |
| --- | --- | --- | --- | --- | --- | --- | --- | --- | --- | --- | --- | --- | --- |
| CTC5 | 20 | 7980387 | AGCC | A | 0.4 | NM_021156 | TMX4 | nonframeshift_de letion | exon4 | c.456_458del | p.152_153del | . | . |
| CTC5 | 20 | 13509134 | AG | A | 0.267 | NM_017714 | TASP1 | frameshift_deleti  on | exon10 | c.819delC | p.C273fs | . | . |
| CTC5 | 20 | 16021851 | GGC | G | 0.429 | NM_001033087 | MACROD2 | frameshift_deleti  on | exon12 | c.455_456del | p.G152fs | . | . |
| CTC5 | 20 | 23729760 | C | CG | 0.618 | NM_001898 | CST1 | frameshift_inserti  on | exon2 | c.234_235insC | p.G79fs | . | . |
| CTC5 | 20 | 25290099 | GC | G | 0.824 | NM_001042472 | ABHD12 | frameshift_deleti  on | exon7 | c.731delG | p.G244fs | . | . |
| CTC5 | 20 | 30137908 | CG | C | 1 | NM_030789 | HM13 | frameshift_deleti  on | exon7 | c.709delG | p.E237fs | . | . |
| CTC5 | 20 | 31600615 | GA | G | 0.507 | NM_025227 | BPIFB2 | frameshift_deleti  on | exon4 | c.211delA | p.I71fs | . | . |
| CTC5 | 20 | 31626731 | C | CG | 1 | NM_174897 | BPIFB6 | frameshift_inserti  on | exon9 | c.863_864insG | p.P288fs | . | . |
| CTC5 | 20 | 32267695 | C | CT | 0.25 | NM_005225 | E2F1 | frameshift_inserti  on | exon3 | c.437_438insA | p.S146fs | . | . |
| CTC5 | 20 | 33328491 | GC | G | 0.944 | NM_014071 | NCOA6 | frameshift_deleti  on | exon11 | c.5568delG | p.G1856fs | . | . |
| CTC5 | 20 | 33447357 | AG | A | 1 | NM_178026 | GGT7 | frameshift_deleti  on | exon7 | c.902delC | p.P301fs | . | . |
| CTC5 | 20 | 34328795 | AT | A | 1 | NM_001242599 | RBM39 | frameshift_deleti  on | exon2 | c.1delA | p.M1fs | . | . |
| CTC5 | 20 | 39831575 | GA | G | 0.355 | NM_015035 | ZHX3 | frameshift_deleti  on | exon3 | c.1981delT | p.S661fs | . | . |
| CTC5 | 20 | 39987139 | CG | C | 0.409 | NM_022896 | LPIN3 | frameshift_deleti  on | exon19 | c.2369delG | p.R790fs | . | . |
| CTC5 | 20 | 40116423 | G | GT | 0.432 | NM_032221 | CHD6 | frameshift_inserti  on | exon14 | c.1882dupA | p.T628fs | . | . |
| CTC5 | 20 | 42195736 | TG | T | 0.281 | NM_016276 | SGK2 | frameshift_deleti  on | exon2 | c.246delG | p.L82fs | . | . |
| CTC5 | 20 | 43348606 | AC | A | 0.84 | NM_003881 | WISP2 | frameshift_deleti  on | exon2 | c.130delC | p.P44fs | . | . |
| CTC5 | 20 | 43850934 | CA | C | 0.812 | NM_003008 | SEMG2 | frameshift_deleti  on | exon2 | c.662delA | p.Q221fs | . | . |
| CTC5 | 20 | 44485952 | C | CATCT | 1 | NM_005469 | ACOT8 | frameshift_inserti  on | exon1 | c.2_3insAGAT | p.M1fs | . | . |
| CTC5 | 20 | 45725760 | AT | A | 0.319 | NM_005244 | EYA2 | frameshift_deleti  on | exon9 | c.842delT | p.I281fs | . | . |
| CTC5 | 20 | 46279836 | ACAG | A | 0.6 | NM_001174087 | NCOA3 | nonframeshift_de  letion | exon20 | c.3760_3762del | p.1254_1254de  l | . | . |
| CTC5 | 20 | 47602050 | C | CA | 0.143 | NM_006420 | ARFGEF2 | frameshift_inserti  on | exon16 | c.2176_2177ins  A | p.L726fs | . | . |
| CTC5 | 20 | 47741043 | CT | C | 0.194 | NM_017454 | STAU1 | frameshift_deleti  on | exon5 | c.447delA | p.E149fs | . | . |
| CTC5 | 20 | 47853022 | TG | T | 1 | NM_017895 | DDX27 | stopgain | exon14 | c.1756delG | p.V586X | . | . |
| CTC5 | 20 | 48760073 | GC | G | 0.387 | NM_001162505 | TMEM189 | frameshift_deleti on | exon2 | c.206delG | p.R69fs | . | . |
| CTC5 | 20 | 49493100 | CG | C | 0.8 | NM_001010974 | BCAS4 | frameshift_deleti  on | exon4 | c.433delG | p.G145fs | . | . |
| CTC5 | 20 | 55942086 | AT | A | 0.353 | NM_001015885 | RAE1 | frameshift_deleti  on | exon7 | c.486delT | p.N162fs | . | . |
| CTC5 | 20 | 57572701 | TC | T | 1 | NM_001336 | CTSZ | frameshift_deleti  on | exon4 | c.594delG | p.G198fs | . | . |
| CTC5 | 20 | 57611619 | CA | C | 0.243 | NM_001256403 | SLMO2 | stopgain | exon4 | c.281delT | p.L94X | . | . |
| CTC5 | 20 | 60708386 | AC | A | 0.286 | NM_144703 | LSM14B | frameshift_deleti on | exon8 | c.1028delC | p.T343fs | . | . |
| CTC5 | 20 | 61541322 | A | AG | 0.697 | NM_001193369 | DIDO1 | frameshift_inserti  on | exon4 | c.889_890insC | p.V297fs | . | . |
| CTC5 | 21 | 15974400 | GA | G | 0.371 | NM_001256579 | LOC388813 | frameshift_deleti  on | exon5 | c.258delT | p.F86fs | . | . |
| CTC5 | 21 | 30419625 | C | CA | 1 | NM_001001992 | USP16 | frameshift_inserti  on | exon14 | c.1992dupA | p.P664fs | . | . |
| CTC5 | 21 | 30959756 | AC | A | 0.231 | NM_175611 | GRIK1 | frameshift_deleti  on | exon11 | c.1677delG | p.L559fs | . | . |
| CTC5 | 21 | 31913981 | AG | A | 1 | NM_181612 | KRTAP19-6 | frameshift_deleti  on | exon1 | c.171delC | p.F57fs | . | . |
| CTC5 | 21 | 31973500 | AGCTAT  GGCTGT GGTCTT | A | 0.154 | NM_181620 | KRTAP22-1 | nonframeshift_de letion | exon1 | c.62_82del | p.21_28del | . | . |
| CTC5 | 21 | 33068945 | G | GA | 1 | NM_001145444 | SCAF4 | frameshift_inserti  on | exon7 | c.850_851insT | p.T284fs | . | . |
| CTC5 | 21 | 34003928 | A | AAGTA  TT | 1 | NM_001160306 | SYNJ1 | nonframeshift_in  sertion | exon28 | c.3957_3958ins  AATACT | p.L1320delinsN  TL | . | . |
| CTC5 | 21 | 34948684 | G | GA | 1 | NM_138927 | SON | frameshift_inserti  on | exon12 | c.7236dupA | p.G2412fs | . | . |
| CTC5 | 21 | 34948696 | GA | G | 1 | NM_138927 | SON | frameshift_deleti  on | exon12 | c.7248delA | p.R2416fs | . | . |
| CTC5 | 21 | 37443348 | AC | A | 0.75 | NM_001286789 | CBR1 | frameshift_deleti  on | exon2 | c.391delC | p.P131fs | . | . |
| CTC5 | 21 | 41385040 | CT | C | 0.312 | NM_001271534 | DSCAM | frameshift_deleti  on | exon33 | c.5905delA | p.S1969fs | . | . |
| CTC5 | 21 | 41719833 | CT | C | 0.237 | NM_001271534 | DSCAM | frameshift_deleti  on | exon6 | c.973delA | p.S325fs | . | . |
| CTC5 | 21 | 42804021 | CG | C | 0.75 | NM_001282920 | MX1 | frameshift_deleti  on | exon1 | c.25delG | p.A9fs | . | . |
| CTC5 | 21 | 43733676 | TG | T | 0.658 | NM_003226 | TFF3 | frameshift_deleti  on | exon2 | c.189delC | p.P63fs | . | . |
| CTC5 | 21 | 43783434 | GC | G | 0.413 | NM_003225 | TFF1 | frameshift_deleti  on | exon2 | c.167delG | p.C56fs | . | . |
| CTC5 | 21 | 44324012 | CA | C | 1 | NM_021075 | NDUFV3 | frameshift_deleti  on | exon3 | c.891delA | p.S297fs | . | . |
| CTC5 | 21 | 46945819 | AC | A | 0.414 | NM_001205207 | SLC19A1 | frameshift_deleti  on | exon4 | c.1084delG | p.V362fs | . | . |
| CTC5 | 21 | 47674320 | TGG | T | 0.246 | NM_003906 | MCM3AP | frameshift_deleti  on | exon19 | c.4120_4121del | p.P1374fs | . | . |
| CTC5 | 21 | 47845837 | GA | G | 0.79 | NM_006031 | PCNT | frameshift_deleti  on | exon33 | c.7273delA | p.K2425fs | . | . |
| CTC5 | 21 | 48064315 | CA | C | 0.177 | NM_001242864 | PRMT2 | frameshift_deleti  on | exon4 | c.243delA | p.A81fs | . | . |
| CTC5 | 22 | 17688099 | TG | T | 0.86 | NM_001282229 | CECR1 | frameshift_deleti  on | exon2 | c.43delC | p.H15fs | . | . |
| CTC5 | 22 | 18347522 | GA | G | 1 | NM_001136004 | MICAL3 | frameshift_deleti  on | exon22 | c.3119delT | p.F1040fs | . | . |
| CTC5 | 22 | 19495356 | G | GA | 0.194 | NM_001178011 | CDC45 | frameshift_inserti  on | exon11 | c.887dupA | p.E296fs | . | . |
| CTC5 | 22 | 20229631 | TC | T | 0.993 | NM_023004 | RTN4R | frameshift_deleti  on | exon2 | c.1024delG | p.D342fs | . | . |
| CTC5 | 22 | 21107255 | AG | A | 0.333 | NM_058004 | PI4KA | frameshift_deleti  on | exon25 | c.2922delC | p.N974fs | . | . |

| CTC5 | 22 | 24582005 | AC | A | 0.3 | NM_019601 | SUSD2 | frameshift_deleti  on | exon9 | c.1362delC | p.H454fs | . | . |
| --- | --- | --- | --- | --- | --- | --- | --- | --- | --- | --- | --- | --- | --- |
| CTC5 | 22 | 24836803 | TG | T | 0.429 | NM_000675 | ADORA2A | frameshift_deleti  on | exon3 | c.586delG | p.V196fs | . | . |
| CTC5 | 22 | 25270517 | C | CG | 0.688 | NM_001039948 | SGSM1 | frameshift_inserti  on | exon13 | c.1427_1428ins  G | p.P476fs | . | . |
| CTC5 | 22 | 25293972 | GA | G | 0.391 | NM_001098498 | SGSM1 | frameshift_deleti  on | exon18 | c.1874delA | p.E625fs | . | . |
| CTC5 | 22 | 26423508 | GC | G | 0.375 | NM_032608 | MYO18B | frameshift_deleti  on | exon43 | c.7569delC | p.S2523fs | . | . |
| CTC5 | 22 | 29446737 | T | TC | 0.308 | NM_001206998 | ZNRF3 | frameshift_inserti  on | exon8 | c.2568_2569ins  C | p.G856fs | . | . |
| CTC5 | 22 | 29521254 | GC | G | 0.5 | NM_001039570 | KREMEN1 | frameshift_deleti  on | exon5 | c.482delC | p.A161fs | . | . |
| CTC5 | 22 | 29886603 | GA | G | 0.805 | NM_021076 | NEFH | frameshift_deleti  on | exon4 | c.2975delA | p.E992fs | . | . |
| CTC5 | 22 | 30695520 | CT | C | 0.283 | NM_001204240 | TBC1D10A | frameshift_deleti  on | exon3 | c.350delA | p.K117fs | . | . |
| CTC5 | 22 | 32109640 | CA | C | 0.5 | NM_173566 | PRR14L | frameshift_deleti  on | exon4 | c.4184delT | p.L1395fs | . | . |
| CTC5 | 22 | 33255357 | A | AC | 0.273 | NM_000362 | TIMP3 | frameshift_inserti  on | exon5 | c.630dupC | p.D210fs | . | . |
| CTC5 | 22 | 37603771 | TG | T | 1 | NM_001051 | SSTR3 | frameshift_deleti  on | exon2 | c.71delC | p.P24fs | . | . |
| CTC5 | 22 | 38119754 | TCAA | T | 0.458 | NM_001039141 | TRIOBP | nonframeshift_de  letion | exon7 | c.1192_1194del | p.398_398del | . | . |
| CTC5 | 22 | 38154040 | GC | G | 0.321 | NM_007032 | TRIOBP | frameshift_deleti  on | exon6 | c.970delC | p.P324fs | . | . |
| CTC5 | 22 | 38318156 | CA | C | 0.81 | NM_033386 | MICALL1 | frameshift_deleti  on | exon6 | c.748delA | p.K250fs | . | . |
| CTC5 | 22 | 39175590 | A | AT | 0.385 | NM_005740 | DNAL4 | frameshift_inserti  on | exon4 | c.181dupA | p.M61fs | . | . |
| CTC5 | 22 | 39777822 | C | CCAA | 1 | NM_004711 | SYNGR1 | nonframeshift_in  sertion | exon4 | c.605_606insCA  A | p.P202delinsP  N | . | . |
| CTC5 | 22 | 39925608 | GC | G | 0.262 | NM_194326 | RPS19BP1 | frameshift_deleti  on | exon4 | c.299delG | p.G100fs | . | . |
| CTC5 | 22 | 40697176 | TGCA | T | 0.273 | NM_015088 | TNRC6B | nonframeshift_de  letion | exon13 | c.3630_3632del | p.1210_1211de  l | . | . |
| CTC5 | 22 | 40711406 | CG | C | 0.5 | NM_015088 | TNRC6B | frameshift_deleti  on | exon18 | c.4469delG | p.R1490fs | . | . |
| CTC5 | 22 | 40757339 | GC | G | 0.321 | NM_000026 | ADSL | frameshift_deleti  on | exon8 | c.856delC | p.Q286fs | . | . |
| CTC5 | 22 | 41253225 | GT | G | 0.912 | NM_001204827 | XPNPEP3 | frameshift_deleti  on | exon1 | c.41delT | p.V14fs | . | . |
| CTC5 | 22 | 41783416 | TG | T | 0.556 | NM_001145398 | TEF | frameshift_deleti  on | exon2 | c.130delG | p.V44fs | . | . |
| CTC5 | 22 | 43195151 | GAC | G | 0.286 | NM_001142293 | ARFGAP3 | frameshift_deleti  on | exon14 | c.1293_1294del | p.L431fs | . | . |
| CTC5 | 22 | 44360351 | AT | A | 0.373 | NM_015380 | SAMM50 | frameshift_deleti  on | exon3 | c.153delT | p.H51fs | . | . |
| CTC5 | 22 | 45595903 | CT | C | 0.75 | NM_001009880 | KIAA0930 | frameshift_deleti  on | exon8 | c.865delA | p.S289fs | . | . |
| CTC5 | 22 | 50217358 | TTC | T | 0.792 | NM_014577 | BRD1 | frameshift_deleti  on | exon1 | c.606_607del | p.Q202fs | . | . |
| CTC5 | 22 | 50852989 | GT | G | 0.606 | NM_001242898 | PPP6R2 | frameshift_deleti  on | exon5 | c.424delT | p.F142fs | . | . |
| CTC5 | 22 | 50886843 | C | CG | 0.725 | NM_002972 | SBF1 | frameshift_inserti  on | exon38 | c.5181dupC | p.A1728fs | . | . |
| CTC5 | X | 1712906 | TC | T | 1 | NM_005088 | AKAP17A | frameshift_deleti  on | exon2 | c.552delC | p.I184fs | . | . |
| CTC5 | X | 8536337 | TC | T | 0.625 | NM_000216 | KAL1 | frameshift_deleti  on | exon8 | c.1142delG | p.G381fs | . | . |
| CTC5 | X | 11215106 | CT | C | 1 | NM_001287242 | ARHGAP6 | frameshift_deleti  on | exon3 | c.218delA | p.K73fs | . | . |
| CTC5 | X | 15609951 | TA | T | 1 | NM_021804 | ACE2 | stopgain | exon5 | c.467delT | p.L156X | . | . |
| CTC5 | X | 18622354 | ACAGCA GATCT | A | 0.25 | NM_003159 | CDKL5 | frameshift_deleti on | exon12 | c.1311_1320del | p.N437fs | . | . |
| CTC5 | X | 18924625 | GGCTCC  GTGCCA | G | 0.205 | NM_000292 | PHKA2 | frameshift_deleti  on | exon25 | c.2783_2793del | p.L928fs | . | . |
| CTC5 | X | 19971944 | TTG | T | 0.676 | NM_198279 | CXorf23 | frameshift_deleti  on | exon5 | c.1515_1516del | p.H505fs | . | . |
| CTC5 | X | 21887643 | CT | C | 0.545 | NM_015884 | MBTPS2 | frameshift_deleti  on | exon7 | c.818delT | p.L273fs | . | . |
| CTC5 | X | 24745984 | TCCA | T | 0.571 | NM_016937 | POLA1 | nonframeshift_de  letion | exon15 | c.1600_1602del | p.534_534del | . | . |
| CTC5 | X | 30260385 | GT | G | 0.222 | NM_002367 | MAGEB4 | frameshift_deleti  on | exon1 | c.134delT | p.V45fs | . | . |
| CTC5 | X | 33038290 | GT | G | 0.32 | NM_000109 | DMD | frameshift_deleti  on | exon2 | c.34delA | p.T12fs | . | . |
| CTC5 | X | 34149747 | TG | T | 0.278 | NM_203408 | FAM47A | frameshift_deleti  on | exon1 | c.648delC | p.P216fs | . | . |
| CTC5 | X | 34648601 | TA | T | 0.361 | NM_031442 | TMEM47 | frameshift_deleti  on | exon3 | c.374delT | p.L125fs | . | . |
| CTC5 | X | 37312609 | C | CT | 0.292 | NM_001142395 | PRRG1 | frameshift_inserti  on | exon4 | c.392_393insT | p.P131fs | . | . |
| CTC5 | X | 37961620 | GC | G | 0.516 | NM_001163334 | SYTL5 | frameshift_deleti  on | exon9 | c.1089delC | p.S363fs | . | . |
| CTC5 | X | 39932964 | CG | C | 0.981 | NM_001123383 | BCOR | frameshift_deleti  on | exon4 | c.1634delC | p.P545fs | . | . |
| CTC5 | X | 41055604 | GT | G | 0.644 | NM_001039590 | USP9X | frameshift_deleti  on | exon27 | c.4079delT | p.V1360fs | . | . |
| CTC5 | X | 47030585 | TGAG | T | 0.15 | NM_001204467 | RBM10 | nonframeshift_de  letion | exon4 | c.361_363del | p.121_121del | . | . |
| CTC5 | X | 48924868 | TG | T | 0.976 | NM_001163321 | CCDC120 | frameshift_deleti  on | exon10 | c.1219delG | p.G407fs | . | . |
| CTC5 | X | 48974150 | CG | C | 0.453 | NM_015698 | GPKOW | frameshift_deleti  on | exon5 | c.580delC | p.R194fs | . | . |
| CTC5 | X | 53409195 | TC | T | 0.556 | NM_006306 | SMC1A | frameshift_deleti  on | exon22 | c.3394delG | p.E1132fs | . | . |
| CTC5 | X | 53432041 | TG | T | 1 | NM_006306 | SMC1A | frameshift_deleti  on | exon13 | c.2098delC | p.Q700fs | . | . |
| CTC5 | X | 54263832 | AT | A | 0.75 | NM_001002838 | WNK3 | frameshift_deleti  on | exon20 | c.4025delA | p.N1342fs | . | . |
| CTC5 | X | 54324819 | AT | A | 0.835 | NM_001002838 | WNK3 | frameshift_deleti  on | exon7 | c.1186delA | p.I396fs | . | . |
| CTC5 | X | 54491913 | TG | T | 0.338 | NM_004463 | FGD1 | frameshift_deleti  on | exon8 | c.1606delC | p.H536fs | . | . |
| CTC5 | X | 70347296 | AC | A | 0.714 | NM_005120 | MED12 | frameshift_deleti  on | exon21 | c.2961delC | p.N987fs | . | . |
| CTC5 | X | 70387535 | G | GT | 0.922 | NM_001166660 | NLGN3 | frameshift_inserti  on | exon5 | c.1469dupT | p.V490fs | . | . |
| CTC5 | X | 70389741 | CA | C | 0.36 | NM_001166660 | NLGN3 | frameshift_deleti  on | exon6 | c.2222delA | p.H741fs | . | . |

| CTC5 | X | 70783060 | AT | A | 0.312 | NM_181672 | OGT | frameshift_deleti  on | exon17 | c.2230delT | p.F744fs | . | . |
| --- | --- | --- | --- | --- | --- | --- | --- | --- | --- | --- | --- | --- | --- |
| CTC5 | X | 71425151 | TG | T | 0.579 | NM_017669 | ERCC6L | frameshift_deleti  on | exon2 | c.3465delC | p.N1155fs | . | . |
| CTC5 | X | 71427214 | AG | A | 1 | NM_017669 | ERCC6L | frameshift_deleti  on | exon2 | c.1402delC | p.L468fs | . | . |
| CTC5 | X | 76938366 | CT | C | 1 | NM_138270 | ATRX | frameshift_deleti  on | exon8 | c.2267delA | p.K756fs | . | . |
| CTC5 | X | 77913553 | TC | T | 0.458 | NM_152694 | ZCCHC5 | frameshift_deleti  on | exon2 | c.364delG | p.E122fs | . | . |
| CTC5 | X | 79951474 | CG | C | 0.938 | NM_153252 | BRWD3 | frameshift_deleti  on | exon27 | c.3083delC | p.P1028fs | . | . |
| CTC5 | X | 83372088 | CT | C | 0.5 | NM_014496 | RPS6KA6 | frameshift_deleti  on | exon11 | c.928delA | p.R310fs | . | . |
| CTC5 | X | 84569461 | GT | G | 0.8 | NM_024921 | POF1B | frameshift_deleti  on | exon9 | c.933delA | p.K311fs | . | . |
| CTC5 | X | 91132716 | GC | G | 0.191 | NM_001168360 | PCDH11X | frameshift_deleti  on | exon2 | c.1478delC | p.A493fs | . | . |
| CTC5 | X | 99840248 | GA | G | 0.885 | NM_022144 | TNMD | frameshift_deleti  on | exon2 | c.70delA | p.K24fs | . | . |
| CTC5 | X | 100615704 | GTGC | G | 0.173 | NM_000061 | BTK | nonframeshift_de  letion | exon8 | c.625_627del | p.209_209del | . | . |
| CTC5 | X | 100656678 | TC | T | 0.272 | NM_000169 | GLA | frameshift_deleti  on | exon3 | c.488delG | p.G163fs | . | . |
| CTC5 | X | 101858648 | CA | C | 0.538 | NM_001168482 | ARMCX5 | frameshift_deleti  on | exon3 | c.1580delA | p.Q527fs | . | . |
| CTC5 | X | 102004834 | TA | T | 0.559 | NM_001142530 | BHLHB9 | frameshift_deleti  on | exon2 | c.912delA | p.V304fs | . | . |
| CTC5 | X | 102842137 | GT | G | 0.579 | NM_001006935 | TCEAL4 | frameshift_deleti  on | exon3 | c.535delT | p.F179fs | . | . |
| CTC5 | X | 106093312 | TG | T | 0.444 | NM_017752 | TBC1D8B | frameshift_deleti  on | exon12 | c.1896delG | p.L632fs | . | . |
| CTC5 | X | 117743258 | TG | T | 0.267 | NM_144658 | DOCK11 | frameshift_deleti  on | exon27 | c.2896delG | p.E966fs | . | . |
| CTC5 | X | 118116865 | C | CT | 0.165 | NM_001031855 | LONRF3 | frameshift_inserti  on | exon3 | c.1019dupT | p.L340fs | . | . |
| CTC5 | X | 135430731 | CA | C | 0.857 | NM_153834 | GPR112 | frameshift_deleti  on | exon6 | c.4867delA | p.K1623fs | . | . |
| CTC5 | X | 135474444 | AGAT | A | 1 | NM_153834 | GPR112 | nonframeshift_de  letion | exon17 | c.7966_7968del | p.2656_2656de  l | . | . |
| CTC5 | X | 138684634 | TG | T | 0.185 | NM_001171877 | MCF2 | frameshift_deleti  on | exon15 | c.1649delC | p.A550fs | . | . |
| CTC5 | X | 140994658 | AC | A | 0.333 | NM_005462 | MAGEC1 | frameshift_deleti  on | exon4 | c.1469delC | p.T490fs | . | . |
| CTC5 | X | 149905104 | C | CA | 0.286 | NM_003828 | MTMR1 | frameshift_inserti  on | exon10 | c.1095dupA | p.P365fs | . | . |
| CTC5 | X | 151092492 | AT | A | 0.939 | NM_001011548 | MAGEA4 | frameshift_deleti  on | exon3 | c.357delT | p.H119fs | . | . |
| CTC5 | X | 153050278 | G | GC | 0.944 | NM_001170760 | SRPK3 | frameshift_inserti  on | exon12 | c.1320dupC | p.G440fs | . | . |
| CTC5 | X | 153537738 | AG | A | 0.6 | NM_001145934 | TKTL1 | frameshift_deleti  on | exon2 | c.127delG | p.G43fs | . | . |
| CTC5 | X | 153627859 | GA | G | 0.5 | NM_001256577 | RPL10 | frameshift_deleti  on | exon4 | c.115delA | p.K39fs | . | . |
| CTC5 | X | 154213000 | AT | A | 0.731 | NM_000132 | F8 | frameshift_deleti  on | exon6 | c.748delA | p.M250fs | . | . |
| CTC6 | 1 | 900386 | GT | G | 0.222 | NM_198317 | KLHL17 | frameshift_deleti  on | exon12 | c.1745delT | p.V582fs | . | . |
| CTC6 | 1 | 1267450 | TC | T | 1 | NM_152228 | TAS1R3 | frameshift_deleti  on | exon3 | c.540delC | p.F180fs | . | . |
| CTC6 | 1 | 1684347 | CCCT | C | 0.4 | NM_001198995 | NADK | nonframeshift_de  letion | exon10 | c.1238_1240del | p.413_414del | . | . |
| CTC6 | 1 | 2491386 | CT | C | 0.192 | NM_003820 | TNFRSF14 | frameshift_deleti  on | exon4 | c.430delT | p.S144fs | . | . |
| CTC6 | 1 | 2542736 | AG | A | 0.5 | NM_033467 | MMEL1 | frameshift_deleti  on | exon4 | c.275delC | p.P92fs | . | . |
| CTC6 | 1 | 2576885 | AG | A | 0.202 | NM_001242672 | TTC34 | frameshift_deleti  on | exon5 | c.804delC | p.H268fs | . | . |
| CTC6 | 1 | 5937183 | TC | T | 0.5 | NM_015102 | NPHP4 | frameshift_deleti  on | exon20 | c.2786delG | p.G929fs | . | . |
| CTC6 | 1 | 6214955 | TG | T | 1 | NM_015557 | CHD5 | frameshift_deleti  on | exon5 | c.509delC | p.P170fs | . | . |
| CTC6 | 1 | 6324746 | CG | C | 0.717 | NM_007274 | ACOT7 | frameshift_deleti  on | exon9 | c.1023delC | p.T341fs | . | . |
| CTC6 | 1 | 6680068 | TGAA | T | 0.14 | NM_153812 | PHF13 | nonframeshift_de  letion | exon3 | c.348_350del | p.116_117del | . | . |
| CTC6 | 1 | 6705952 | TTG | T | 0.152 | NM_018198 | DNAJC11 | frameshift_deleti  on | exon8 | c.789_790del | p.D263fs | . | . |
| CTC6 | 1 | 7724444 | TC | T | 0.463 | NM_015215 | CAMTA1 | frameshift_deleti  on | exon9 | c.1838delC | p.S613fs | . | . |
| CTC6 | 1 | 8390560 | GC | G | 0.316 | NM_001080397 | SLC45A1 | frameshift_deleti  on | exon4 | c.1008delC | p.S336fs | . | . |
| CTC6 | 1 | 8921448 | TG | T | 0.3 | NM_001201483 | ENO1 | frameshift_deleti  on | exon9 | c.996delC | p.G332fs | . | . |
| CTC6 | 1 | 9427562 | GA | G | 0.15 | NM_025106 | SPSB1 | frameshift_deleti  on | exon3 | c.751delA | p.R251fs | . | . |
| CTC6 | 1 | 10218500 | CA | C | 0.308 | NM_006048 | UBE4B | frameshift_deleti  on | exon21 | c.2627delA | p.Q876fs | . | . |
| CTC6 | 1 | 11589947 | CTT | C | 0.4 | NM_020780 | PTCHD2 | frameshift_deleti  on | exon15 | c.3034_3035del | p.F1012fs | . | . |
| CTC6 | 1 | 11907422 | CG | C | 0.674 | NM_006172 | NPPA | frameshift_deleti  on | exon2 | c.197delC | p.P66fs | . | . |
| CTC6 | 1 | 11983157 | CG | C | 0.5 | NM_138346 | KIAA2013 | frameshift_deleti  on | exon2 | c.1422delC | p.P474fs | . | . |
| CTC6 | 1 | 12336816 | A | AT | 0.36 | NM_015378 | VPS13D | frameshift_inserti  on | exon19 | c.3171_3172ins  T | p.T1057fs | . | . |
| CTC6 | 1 | 12337871 | AT | A | 0.275 | NM_015378 | VPS13D | frameshift_deleti  on | exon19 | c.4227delT | p.N1409fs | . | . |
| CTC6 | 1 | 12343426 | AG | A | 0.214 | NM_015378 | VPS13D | frameshift_deleti  on | exon21 | c.5268delG | p.K1756fs | . | . |
| CTC6 | 1 | 12704607 | CT | C | 0.514 | NM_001013630 | AADACL4 | frameshift_deleti  on | exon1 | c.43delT | p.F15fs | . | . |
| CTC6 | 1 | 14106112 | GA | G | 0.213 | NM_001007257 | PRDM2 | frameshift_deleti  on | exon3 | c.1220delA | p.E407fs | . | . |
| CTC6 | 1 | 14106394 | A | ACTC | 0.123 | NM_001007257 | PRDM2 | nonframeshift_in  sertion | exon3 | c.1501_1502ins  CTC | p.T501delinsTP | . | . |
| CTC6 | 1 | 15752492 | AC | A | 1 | NM_024329 | EFHD2 | frameshift_deleti  on | exon2 | c.435delC | p.D145fs | . | . |
| CTC6 | 1 | 17314727 | T | TC | 0.562 | NM_001141974 | ATP13A2 | frameshift_inserti  on | exon24 | c.2632dupG | p.E878fs | . | . |
| CTC6 | 1 | 17555526 | TGA | T | 0.117 | NM_013358 | PADI1 | frameshift_deleti  on | exon8 | c.910_911del | p.E304fs | . | . |
| CTC6 | 1 | 19166649 | A | AT | 0.846 | NM_152232 | TAS1R2 | frameshift_inserti  on | exon6 | c.1963dupA | p.I655fs | . | . |

| CTC6 | 1 | 19948600 | GT | G | 0.436 | NM_001032363 | MINOS1 | frameshift_deleti  on | exon2 | c.72delT | p.G24fs | . | . |
| --- | --- | --- | --- | --- | --- | --- | --- | --- | --- | --- | --- | --- | --- |
| CTC6 | 1 | 20680673 | GC | G | 0.353 | NM_001039500 | VWA5B1 | frameshift_deleti  on | exon22 | c.3566delC | p.A1189fs | . | . |
| CTC6 | 1 | 22190686 | CG | C | 0.486 | NM_005529 | HSPG2 | frameshift_deleti  on | exon37 | c.4646delC | p.T1549fs | . | . |
| CTC6 | 1 | 22199525 | TG | T | 0.176 | NM_005529 | HSPG2 | frameshift_deleti  on | exon31 | c.3852delC | p.S1284fs | . | . |
| CTC6 | 1 | 22838362 | C | CA | 0.667 | NM_014870 | ZBTB40 | frameshift_inserti  on | exon11 | c.2197dupA | p.A732fs | . | . |
| CTC6 | 1 | 22912999 | GC | G | 0.421 | NM_001006943 | EPHA8 | frameshift_deleti  on | exon4 | c.851delC | p.A284fs | . | . |
| CTC6 | 1 | 23836502 | GC | G | 0.161 | NM_004091 | E2F2 | frameshift_deleti  on | exon7 | c.1183delG | p.A395fs | . | . |
| CTC6 | 1 | 24394839 | CA | C | 0.364 | NM_152372 | MYOM3 | frameshift_deleti  on | exon26 | c.3168delT | p.F1056fs | . | . |
| CTC6 | 1 | 25718581 | CA | C | 0.581 | NM_020485 | RHCE | frameshift_deleti  on | exon4 | c.537delT | p.F179fs | . | . |
| CTC6 | 1 | 26586245 | AG | A | 0.333 | NM_001281517 | CEP85 | frameshift_deleti  on | exon6 | c.1141delG | p.V381fs | . | . |
| CTC6 | 1 | 27268295 | TC | T | 0.364 | NM_006600 | NUDC | frameshift_deleti  on | exon4 | c.416delC | p.S139fs | . | . |
| CTC6 | 1 | 27684716 | GCT | G | 0.217 | NM_004672 | MAP3K6 | frameshift_deleti  on | exon21 | c.2869_2870del | p.S957fs | . | . |
| CTC6 | 1 | 28282676 | AC | A | 0.537 | NM_001009568 | SMPDL3B | frameshift_deleti  on | exon7 | c.1089delC | p.H363fs | . | . |
| CTC6 | 1 | 29356989 | AT | A | 0.25 | NM_203343 | EPB41 | frameshift_deleti  on | exon7 | c.1098delT | p.H366fs | . | . |
| CTC6 | 1 | 29475218 | CCTT | C | 0.109 | NM_005626 | SRSF4 | nonframeshift_de  letion | exon6 | c.1186_1188del | p.396_396del | . | . |
| CTC6 | 1 | 31210482 | G | GA | 0.364 | NM_006762 | LAPTM5 | frameshift_inserti  on | exon6 | c.574dupT | p.S192fs | . | . |
| CTC6 | 1 | 32089210 | GC | G | 0.333 | NM_001525 | HCRTR1 | frameshift_deleti  on | exon7 | c.826delC | p.P276fs | . | . |
| CTC6 | 1 | 32713080 | GA | G | 0.444 | NM_032648 | FAM167B | frameshift_deleti  on | exon1 | c.59delA | p.E20fs | . | . |
| CTC6 | 1 | 33360919 | CT | C | 0.514 | NM_033504 | TMEM54 | frameshift_deleti  on | exon5 | c.580delA | p.S194fs | . | . |
| CTC6 | 1 | 35847323 | GT | G | 0.5 | NM_005095 | ZMYM4 | frameshift_deleti  on | exon9 | c.1534delT | p.F512fs | . | . |
| CTC6 | 1 | 36211053 | TG | T | 1 | NM_001190481 | CLSPN | frameshift_deleti  on | exon15 | c.2772delC | p.D924fs | . | . |
| CTC6 | 1 | 36474344 | TG | T | 0.227 | NM_177422 | AGO3 | frameshift_deleti  on | exon5 | c.146delG | p.C49fs | . | . |
| CTC6 | 1 | 36785645 | GC | G | 0.765 | NM_024676 | SH3D21 | frameshift_deleti  on | exon11 | c.1049delC | p.A350fs | . | . |
| CTC6 | 1 | 38201017 | TG | T | 0.667 | NM_001099439 | EPHA10 | frameshift_deleti  on | exon6 | c.1402delC | p.Q468fs | . | . |
| CTC6 | 1 | 38334174 | TG | T | 0.75 | NM_005540 | INPP5B | frameshift_deleti  on | exon20 | c.2299delC | p.Q767fs | . | . |
| CTC6 | 1 | 38488383 | GA | G | 1 | NM_016037 | UTP11L | frameshift_deleti  on | exon7 | c.581delA | p.E194fs | . | . |
| CTC6 | 1 | 39340811 | GC | G | 0.385 | NM_030772 | GJA9 | frameshift_deleti  on | exon2 | c.959delG | p.C320fs | . | . |
| CTC6 | 1 | 39835772 | T | TG | 0.214 | NM_012090 | MACF1 | frameshift_inserti  on | exon45 | c.6824dupG | p.W2275fs | . | . |
| CTC6 | 1 | 39879024 | AG | A | 0.189 | NM_015038 | KIAA0754 | frameshift_deleti  on | exon1 | c.3088delG | p.A1030fs | . | . |
| CTC6 | 1 | 39907710 | GT | G | 1 | NM_012090 | MACF1 | stopgain | exon70 | c.12583delT | p.L4195X | . | . |
| CTC6 | 1 | 40770194 | TG | T | 0.854 | NM_001852 | COL9A2 | frameshift_deleti on | exon23 | c.1168delC | p.Q390fs | . | . |
| CTC6 | 1 | 40981007 | CTG | C | 1 | NM_022774 | EXO5 | frameshift_deleti  on | exon3 | c.792_793del | p.S264fs | . | . |
| CTC6 | 1 | 41449019 | TG | T | 0.64 | NM_001905 | CTPS1 | frameshift_deleti  on | exon2 | c.58delG | p.A20fs | . | . |
| CTC6 | 1 | 42880446 | CG | C | 0.879 | NM_173642 | RIMKLA | frameshift_deleti  on | exon5 | c.978delG | p.P326fs | . | . |
| CTC6 | 1 | 43042736 | AC | A | 0.357 | NM_001080850 | CCDC30 | frameshift_deleti  on | exon7 | c.902delC | p.T301fs | . | . |
| CTC6 | 1 | 43905321 | CCTTGTC  ACTGGC GTTGTG GCCCCC CTCCTCT CCGGGG | C | 0.151 | NM_015284 | SZT2 | frameshift_deleti on | exon49 | c.6753_6804del | p.P2251fs | . | . |
| CTC6 | 1 | 44056886 | GGCC | G | 0.218 | NM_002840 | PTPRF | nonframeshift_de  letion | exon9 | c.1194_1196del | p.398_399del | . | . |
| CTC6 | 1 | 44149372 | GT | G | 0.412 | NM_014663 | KDM4A | frameshift_deleti  on | exon12 | c.1753delT | p.F585fs | . | . |
| CTC6 | 1 | 44435859 | G | GT | 0.348 | NM_001039589 | DPH2 | frameshift_inserti  on | exon1 | c.13dupT | p.M4fs | . | . |
| CTC6 | 1 | 44447006 | TAGC | T | 0.811 | NM_001005417 | B4GALT2 | nonframeshift_de  letion | exon2 | c.175_177del | p.59_59del | . | . |
| CTC6 | 1 | 44467164 | CA | C | 0.231 | NM_001261380 | SLC6A9 | frameshift_deleti  on | exon8 | c.1109delT | p.L370fs | . | . |
| CTC6 | 1 | 45226350 | TG | T | 0.301 | NM_006845 | KIF2C | frameshift_deleti  on | exon16 | c.1660delG | p.G554fs | . | . |
| CTC6 | 1 | 45270345 | GC | G | 0.653 | NM_004073 | PLK3 | frameshift_deleti  on | exon13 | c.1530delC | p.S510fs | . | . |
| CTC6 | 1 | 46033671 | TC | T | 0.305 | NM_153326 | AKR1A1 | frameshift_deleti  on | exon5 | c.375delC | p.F125fs | . | . |
| CTC6 | 1 | 46425131 | CCA | C | 0.4 | NM_015112 | MAST2 | frameshift_deleti  on | exon5 | c.575_576del | p.P192fs | . | . |
| CTC6 | 1 | 47131017 | CA | C | 0.278 | NM_001042546 | ATPAF1 | frameshift_deleti  on | exon2 | c.354delT | p.F118fs | . | . |
| CTC6 | 1 | 47155260 | TG | T | 0.8 | NM_014774 | EFCAB14 | frameshift_deleti  on | exon6 | c.793delC | p.Q265fs | . | . |
| CTC6 | 1 | 47284432 | CAAG | C | 0.167 | NM_000779 | CYP4B1 | nonframeshift_de  letion | exon12 | c.1483_1485del | p.495_495del | . | . |
| CTC6 | 1 | 47399639 | CA | C | 0.875 | NM_000778 | CYP4A11 | frameshift_deleti  on | exon9 | c.1200delT | p.P400fs | . | . |
| CTC6 | 1 | 52306063 | ATCT | A | 1 | NM_001101662 | NRD1 | nonframeshift_de  letion | exon2 | c.462_464del | p.154_155del | . | . |
| CTC6 | 1 | 52499075 | GC | G | 0.351 | NM_138417 | KTI12 | frameshift_deleti  on | exon1 | c.358delG | p.A120fs | . | . |
| CTC6 | 1 | 52827212 | TTCC | T | 0.184 | NM_032449 | CC2D1B | nonframeshift_de  letion | exon4 | c.288_290del | p.96_97del | . | . |
| CTC6 | 1 | 52896909 | A | AT | 0.3 | NM_001009881 | ZCCHC11 | frameshift_inserti  on | exon28 | c.4486dupA | p.M1496fs | . | . |
| CTC6 | 1 | 55625298 | CT | C | 0.875 | NM_015306 | USP24 | frameshift_deleti  on | exon9 | c.1053delA | p.K351fs | . | . |
| CTC6 | 1 | 60381691 | TG | T | 0.286 | NM_000775 | CYP2J2 | frameshift_deleti  on | exon2 | c.291delC | p.I97fs | . | . |

| CTC6 | 1 | 62321888 | CT | C | 0.296 | NM_176877 | INADL | frameshift_deleti  on | exon18 | c.2300delT | p.L767fs | . | . |
| --- | --- | --- | --- | --- | --- | --- | --- | --- | --- | --- | --- | --- | --- |
| CTC6 | 1 | 66070857 | AG | A | 1 | NM_001198687 | LEPR | frameshift_deleti  on | exon10 | c.1541delG | p.R514fs | . | . |
| CTC6 | 1 | 70819791 | CG | C | 0.377 | NM_030816 | ANKRD13C | frameshift_deleti  on | exon1 | c.300delC | p.P100fs | . | . |
| CTC6 | 1 | 74575230 | AC | A | 0.6 | NM_001105659 | LRRIQ3 | frameshift_deleti  on | exon5 | c.714delG | p.V238fs | . | . |
| CTC6 | 1 | 76282093 | A | AT | 0.833 | NM_002440 | MSH4 | frameshift_inserti  on | exon6 | c.851_852insT | p.K284fs | . | . |
| CTC6 | 1 | 78024345 | C | CTAT | 1 | NM_012093 | AK5 | nonframeshift_in  sertion | exon14 | c.1601_1602ins  TAT | p.S534delinsSI | . | . |
| CTC6 | 1 | 78097842 | TG | T | 0.25 | NM_015534 | ZZZ3 | stopgain | exon5 | c.1197delC | p.Y399X | . | . |
| CTC6 | 1 | 78959138 | AC | A | 0.625 | NM_000959 | PTGFR | frameshift_deleti on | exon2 | c.711delC | p.H237fs | . | . |
| CTC6 | 1 | 78959139 | CA | C | 0.571 | NM_000959 | PTGFR | frameshift_deleti  on | exon2 | c.712delA | p.R238fs | . | . |
| CTC6 | 1 | 82409328 | TC | T | 0.833 | NM_012302 | LPHN2 | frameshift_deleti  on | exon5 | c.1074delC | p.F358fs | . | . |
| CTC6 | 1 | 86591373 | CA | C | 0.352 | NM_152890 | COL24A1 | frameshift_deleti  on | exon3 | c.645delT | p.F215fs | . | . |
| CTC6 | 1 | 87043624 | AT | A | 0.462 | NM_012128 | CLCA4 | frameshift_deleti  on | exon12 | c.1992delT | p.Y664fs | . | . |
| CTC6 | 1 | 87045896 | TCCTAC  A | T | 1 | NM_012128 | CLCA4 | nonframeshift_de  letion | exon14 | c.2629_2634del | p.877_878del | . | . |
| CTC6 | 1 | 89655998 | AG | A | 0.75 | NM_052941 | GBP4 | frameshift_deleti  on | exon7 | c.919delC | p.L307fs | . | . |
| CTC6 | 1 | 93659180 | GA | G | 0.714 | NM_206886 | CCDC18 | frameshift_deleti  on | exon6 | c.578delA | p.E193fs | . | . |
| CTC6 | 1 | 93701809 | CA | C | 0.233 | NM_206886 | CCDC18 | frameshift_deleti  on | exon19 | c.2466delA | p.S822fs | . | . |
| CTC6 | 1 | 94476929 | AC | A | 0.417 | NM_000350 | ABCA4 | frameshift_deleti  on | exon39 | c.5472delG | p.R1824fs | . | . |
| CTC6 | 1 | 94639987 | TG | T | 0.217 | NM_004815 | ARHGAP29 | frameshift_deleti  on | exon23 | c.3223delC | p.Q1075fs | . | . |
| CTC6 | 1 | 101190437 | AT | A | 0.26 | NM_001078 | VCAM1 | frameshift_deleti  on | exon4 | c.920delT | p.I307fs | . | . |
| CTC6 | 1 | 103348763 | AT | A | 0.951 | NM_080630 | COL11A1 | frameshift_deleti  on | exon62 | c.4614delA | p.K1538fs | . | . |
| CTC6 | 1 | 104121998 | AT | A | 0.5 | NM_020978 | AMY2B | frameshift_deleti  on | exon12 | c.1413delT | p.D471fs | . | . |
| CTC6 | 1 | 108298068 | TG | T | 0.231 | NM_006113 | VAV3 | frameshift_deleti  on | exon12 | c.1153delC | p.Q385fs | . | . |
| CTC6 | 1 | 108679386 | TG | T | 1 | NM_013386 | SLC25A24 | frameshift_deleti  on | exon10 | c.1322delC | p.P441fs | . | . |
| CTC6 | 1 | 109242357 | CA | C | 0.447 | NM_018061 | PRPF38B | frameshift_deleti  on | exon6 | c.1357delA | p.K453fs | . | . |
| CTC6 | 1 | 109824398 | TC | T | 0.436 | NM_001005290 | PSRC1 | frameshift_deleti  on | exon4 | c.361delG | p.D121fs | . | . |
| CTC6 | 1 | 110466469 | GC | G | 0.31 | NM_000757 | CSF1 | frameshift_deleti  on | exon6 | c.1227delC | p.R409fs | . | . |
| CTC6 | 1 | 110466771 | CT | C | 0.263 | NM_000757 | CSF1 | frameshift_deleti  on | exon6 | c.1529delT | p.L510fs | . | . |
| CTC6 | 1 | 111957501 | CTCACA  GACTGA TGACTC | C | 1 | NM_002557 | OVGP1 | nonframeshift_de letion | exon11 | c.1598_1621del | p.533_541del | . | . |
| CTC6 | 1 | 112019427 | AG | A | 0.25 | NM_174896 | C1orf162 | frameshift_deleti  on | exon3 | c.46delG | p.G16fs | . | . |
| CTC6 | 1 | 114340335 | GT | G | 0.286 | NM_018364 | RSBN1 | frameshift_deleti  on | exon2 | c.1026delA | p.E342fs | . | . |
| CTC6 | 1 | 114499778 | GC | G | 0.889 | NM_181358 | HIPK1 | stopgain | exon5 | c.444delC | p.C148X | . | . |
| CTC6 | 1 | 120300027 | TA | T | 0.256 | NM_001166107 | HMGCS2 | frameshift_deleti on | exon4 | c.758delT | p.L253fs | . | . |
| CTC6 | 1 | 120437605 | GGAC | G | 0.152 | NM_021794 | ADAM30 | nonframeshift_de  letion | exon1 | c.1352_1354del | p.451_452del | . | . |
| CTC6 | 1 | 120458118 | CT | C | 0.588 | NM_024408 | NOTCH2 | frameshift_deleti  on | exon34 | c.7226delA | p.Q2409fs | . | . |
| CTC6 | 1 | 120458665 | TG | T | 0.194 | NM_024408 | NOTCH2 | frameshift_deleti  on | exon34 | c.6679delC | p.H2227fs | . | . |
| CTC6 | 1 | 120506343 | G | GA | 0.267 | NM_001200001 | NOTCH2 | frameshift_inserti  on | exon11 | c.1768dupT | p.S590fs | . | . |
| CTC6 | 1 | 145015865 | CA | C | 0.333 | NM_001198832 | PDE4DIP | frameshift_deleti  on | exon3 | c.222delT | p.F74fs | . | . |
| CTC6 | 1 | 145440123 | GA | G | 0.288 | NM_006472 | TXNIP | frameshift_deleti  on | exon4 | c.558delA | p.R186fs | . | . |
| CTC6 | 1 | 145487460 | G | GT | 0.246 | NM_153713 | LIX1L | frameshift_inserti  on | exon2 | c.450dupT | p.S150fs | . | . |
| CTC6 | 1 | 145561964 | CA | C | 0.714 | NM_001280799 | ANKRD35 | frameshift_deleti  on | exon8 | c.1383delA | p.A461fs | . | . |
| CTC6 | 1 | 145579322 | GCC | G | 0.435 | NM_006099 | PIAS3 | frameshift_deleti  on | exon5 | c.660_661del | p.C220fs | . | . |
| CTC6 | 1 | 145608604 | TG | T | 0.229 | NM_006468 | POLR3C | frameshift_deleti  on | exon3 | c.202delC | p.H68fs | . | . |
| CTC6 | 1 | 146751784 | TG | T | 0.282 | NM_001256338 | CHD1L | frameshift_deleti  on | exon9 | c.1014delG | p.L338fs | . | . |
| CTC6 | 1 | 147091857 | TCAG | T | 0.24 | NM_004326 | BCL9 | nonframeshift_de  letion | exon8 | c.1897_1899del | p.633_633del | . | . |
| CTC6 | 1 | 149858152 | CT | C | 0.46 | NM_003528 | HIST2H2BE | frameshift_deleti  on | exon1 | c.38delA | p.K13fs | . | . |
| CTC6 | 1 | 150065728 | AG | A | 0.5 | NM_001279355 | VPS45 | frameshift_deleti  on | exon10 | c.1109delG | p.S370fs | . | . |
| CTC6 | 1 | 150199039 | CTCCTCT | C | 1 | NM_001136478 | ANP32E | nonframeshift_de  letion | exon4 | c.453_458del | p.151_153del | . | . |
| CTC6 | 1 | 150445440 | AT | A | 0.269 | NM_015203 | RPRD2 | frameshift_deleti  on | exon11 | c.4017delT | p.H1339fs | . | . |
| CTC6 | 1 | 151015868 | GC | G | 0.207 | NM_001159642 | BNIPL | frameshift_deleti  on | exon6 | c.432delC | p.S144fs | . | . |
| CTC6 | 1 | 151384137 | AT | A | 0.636 | NM_145796 | POGZ | frameshift_deleti  on | exon10 | c.1604delA | p.N535fs | . | . |
| CTC6 | 1 | 151413512 | C | CA | 0.636 | NM_001194937 | POGZ | frameshift_inserti  on | exon3 | c.174dupT | p.A59fs | . | . |
| CTC6 | 1 | 151678722 | TTGC | T | 0.185 | NM_001172649 | CELF3 | nonframeshift_de  letion | exon9 | c.951_953del | p.317_318del | . | . |
| CTC6 | 1 | 152081681 | AT | A | 0.517 | NM_007113 | TCHH | frameshift_deleti  on | exon3 | c.4011delA | p.K1337fs | . | . |
| CTC6 | 1 | 152195728 | AT | A | 0.964 | NM_001009931 | HRNR | frameshift_deleti  on | exon2 | c.1delA | p.M1fs | . | . |
| CTC6 | 1 | 152484325 | TG | T | 0.317 | NM_178438 | LCE5A | frameshift_deleti  on | exon2 | c.316delG | p.G106fs | . | . |
| CTC6 | 1 | 152681584 | GC | G | 0.323 | NM_178356 | LCE4A | frameshift_deleti  on | exon1 | c.34delC | p.P12fs | . | . |

| CTC6 | 1 | 152681680 | C | CAGCT  CTGGG GGCTG | 0.792 | NM_178356 | LCE4A | nonframeshift_in sertion | exon1 | c.129_130insA  GCTCTGGGGG CTGCTGT | p.S43delinsSSS GGCC | . | . |
| --- | --- | --- | --- | --- | --- | --- | --- | --- | --- | --- | --- | --- | --- |
| CTC6 | 1 | 152681686 | T | TGGGG  GCTGC TGTAG | 0.707 | NM_178356 | LCE4A | nonframeshift_in sertion | exon1 | c.135_136insG  GGGGCTGCTG TAGCTGG | p.S45delinsSG GCCSW | . | . |
| CTC6 | 1 | 152681687 | G | GGGGG  CTGCT GTAGC | 0.727 | NM_178356 | LCE4A | frameshift_inserti on | exon1 | c.136_137insG  GGGCTGCTGT AGCTCT | p.G46fs | . | . |
| CTC6 | 1 | 153661961 | CT | C | 0.5 | NM_000906 | NPR1 | frameshift_deleti  on | exon18 | c.2722delT | p.F908fs | . | . |
| CTC6 | 1 | 153748613 | GGC | G | 0.157 | NM_024330 | SLC27A3 | frameshift_deleti  on | exon1 | c.782_783del | p.G261fs | . | . |
| CTC6 | 1 | 153785858 | AGG | A | 0.478 | NM_020699 | GATAD2B | frameshift_deleti  on | exon8 | c.1285_1286del | p.P429fs | . | . |
| CTC6 | 1 | 153930962 | CG | C | 0.667 | NM_181715 | CRTC2 | frameshift_deleti  on | exon1 | c.11delC | p.S4fs | . | . |
| CTC6 | 1 | 154033483 | GC | G | 1 | NM_001159484 | NUP210L | frameshift_deleti  on | exon19 | c.2682delG | p.L894fs | . | . |
| CTC6 | 1 | 154224019 | TGG | T | 0.119 | NM_001127320 | UBAP2L | frameshift_deleti  on | exon14 | c.1555_1556del | p.G519fs | . | . |
| CTC6 | 1 | 154842199 | G | GGCTG  CTGCT | 1 | NM_001204087 | KCNN3 | nonframeshift_in  sertion | exon1 | c.241_242insA  GCAGCAGCAG | p.P81delinsQQ  QQQP | . | . |
| CTC6 | 1 | 154842199 | G | GGCTG  CTGCT | 1 | NM_001204087 | KCNN3 | nonframeshift_in  sertion | exon1 | c.241_242insA  GCAGCAGCAG | p.P81delinsQQ  QQP | . | . |
| CTC6 | 1 | 154842202 | T | TGCTG  CTGCT | 0.344 | NM_001204087 | KCNN3 | nonframeshift_in  sertion | exon1 | c.238_239insTG  CAGCAGCAGC | p.Q80delinsLQ  QQQQ | . | . |
| CTC6 | 1 | 154842206 | G | GCTGC  TGCTG | 0.388 | NM_001204087 | KCNN3 | stopgain | exon1 | c.234_235insTA  GCAGCAGCAG | p.Q79delinsX | . | . |
| CTC6 | 1 | 154842222 | C | CTGCT  GCTGC | 0.517 | NM_001204087 | KCNN3 | nonframeshift_in  sertion | exon1 | c.218_219insTC  AGCAGCAGCA | p.Q73delinsHQ  QQQQ | . | . |
| CTC6 | 1 | 154842225 | C | CTGCT  GCTGA | 0.206 | NM_001204087 | KCNN3 | nonframeshift_in  sertion | exon1 | c.215_216insTC  ATCATCAGCA | p.Q72delinsHH  HQQQ | . | . |
| CTC6 | 1 | 154842228 | C | CTGCT  GCTGC | 0.769 | NM_001204087 | KCNN3 | nonframeshift_in  sertion | exon1 | c.212_213insAC  AGCAGCAGCA | p.Q71delinsQQ  QQQQ | . | . |
| CTC6 | 1 | 154842228 | C | CTGCT  GCTGC | 0.769 | NM_001204087 | KCNN3 | nonframeshift_in  sertion | exon1 | c.212_213insAC  AGCAGCAGCA | p.Q71delinsQQ  QQQ | . | . |
| CTC6 | 1 | 154842231 | C | CTGCT  GCTGC | 0.714 | NM_001204087 | KCNN3 | nonframeshift_in  sertion | exon1 | c.209_210insTC  AGCAGCAGCA | p.Q70delinsHQ  QQQQ | . | . |
| CTC6 | 1 | 154842231 | C | CTGCT  GCTGTT | 0.714 | NM_001204087 | KCNN3 | nonframeshift_in  sertion | exon1 | c.209_210insAC  AACAGCAGCA | p.Q70delinsQQ  QQQ | . | . |
| CTC6 | 1 | 154842239 | G | GCTGC  TGCTG | 0.533 | NM_001204087 | KCNN3 | frameshift_inserti  on | exon1 | c.201_202insA  GCAGCAGCAG | p.Q68fs | . | . |
| CTC6 | 1 | 154842330 | TTGC | T | 0.19 | NM_001204087 | KCNN3 | nonframeshift_de  letion | exon1 | c.108_110del | p.36_37del | . | . |
| CTC6 | 1 | 155168334 | TG | T | 0.26 | NM_001252608 | THBS3 | frameshift_deleti  on | exon15 | c.1579delC | p.Q527fs | . | . |
| CTC6 | 1 | 155179082 | TG | T | 0.667 | NM_002455 | MTX1 | frameshift_deleti  on | exon1 | c.488delG | p.W163fs | . | . |
| CTC6 | 1 | 155292373 | CT | C | 0.4 | NM_001105203 | RUSC1 | frameshift_deleti  on | exon2 | c.810delT | p.P270fs | . | . |
| CTC6 | 1 | 155447936 | TA | T | 0.205 | NM_018489 | ASH1L | frameshift_deleti  on | exon3 | c.4724delT | p.L1575fs | . | . |
| CTC6 | 1 | 155912591 | TG | T | 0.257 | NM_181885 | RXFP4 | frameshift_deleti  on | exon1 | c.1092delG | p.L364fs | . | . |
| CTC6 | 1 | 156565049 | A | AAC | 1 | NM_015590 | GPATCH4 | frameshift_inserti  on | exon8 | c.1083_1084ins  GT | p.F362fs | . | . |
| CTC6 | 1 | 156917982 | AT | A | 0.533 | NM_014784 | ARHGEF11 | frameshift_deleti  on | exon23 | c.2023delA | p.M675fs | . | . |
| CTC6 | 1 | 158151868 | G | GA | 0.857 | NM_001766 | CD1D | frameshift_inserti  on | exon4 | c.376dupA | p.G125fs | . | . |
| CTC6 | 1 | 158226671 | TG | T | 0.203 | NM_001763 | CD1A | frameshift_deleti  on | exon4 | c.701delG | p.W234fs | . | . |
| CTC6 | 1 | 158617431 | AT | A | 0.28 | NM_003126 | SPTA1 | frameshift_deleti  on | exon27 | c.3793delA | p.M1265fs | . | . |
| CTC6 | 1 | 158622406 | GTC | G | 0.126 | NM_003126 | SPTA1 | frameshift_deleti  on | exon23 | c.3224_3225del | p.R1075fs | . | . |
| CTC6 | 1 | 158637769 | GT | G | 0.52 | NM_003126 | SPTA1 | frameshift_deleti  on | exon15 | c.1916delA | p.N639fs | . | . |
| CTC6 | 1 | 158669502 | GA | G | 0.769 | NM_001005279 | OR6K2 | frameshift_deleti  on | exon1 | c.940delT | p.S314fs | . | . |
| CTC6 | 1 | 158990176 | AG | A | 0.286 | NM_001206567 | IFI16 | frameshift_deleti  on | exon5 | c.851delG | p.R284fs | . | . |
| CTC6 | 1 | 159898120 | TG | T | 0.184 | NM_001135050 | IGSF9 | frameshift_deleti  on | exon19 | c.3057delC | p.G1019fs | . | . |
| CTC6 | 1 | 160000960 | TA | T | 0.208 | NM_145167 | PIGM | frameshift_deleti  on | exon1 | c.569delT | p.I190fs | . | . |
| CTC6 | 1 | 160094143 | G | GC | 0.25 | NM_000702 | ATP1A2 | frameshift_inserti  on | exon6 | c.553_554insC | p.V185fs | . | . |
| CTC6 | 1 | 160160760 | ACC | A | 0.769 | NM_001231 | CASQ1 | frameshift_deleti  on | exon1 | c.220_221del | p.P74fs | . | . |
| CTC6 | 1 | 160194242 | A | AT | 0.286 | NM_015726 | DCAF8 | frameshift_inserti  on | exon10 | c.1298dupA | p.N433fs | . | . |
| CTC6 | 1 | 160650938 | CA | C | 0.909 | NM_001256030 | CD48 | frameshift_deleti  on | exon3 | c.705delT | p.F235fs | . | . |
| CTC6 | 1 | 160654898 | TG | T | 1 | NM_001256030 | CD48 | frameshift_deleti  on | exon2 | c.163delC | p.Q55fs | . | . |
| CTC6 | 1 | 160920889 | AG | A | 0.714 | NM_080878 | ITLN2 | frameshift_deleti  on | exon4 | c.384delC | p.N128fs | . | . |
| CTC6 | 1 | 161019096 | AG | A | 0.8 | NM_001287602 | ARHGAP30 | frameshift_deleti  on | exon8 | c.1183delC | p.L395fs | . | . |
| CTC6 | 1 | 161059014 | AT | A | 0.401 | NM_030916 | PVRL4 | frameshift_deleti  on | exon1 | c.72delA | p.S24fs | . | . |
| CTC6 | 1 | 161142041 | T | TC | 0.306 | NM_001199873 | B4GALT3 | frameshift_inserti  on | exon7 | c.883_884insG | p.K295fs | . | . |
| CTC6 | 1 | 161180173 | GT | G | 0.236 | NM_001166159 | NDUFS2 | frameshift_deleti  on | exon8 | c.861delT | p.G287fs | . | . |
| CTC6 | 1 | 162560281 | TC | T | 0.189 | NM_003115 | UAP1 | frameshift_deleti  on | exon8 | c.1339delC | p.R447fs | . | . |
| CTC6 | 1 | 163042222 | C | CA | 0.605 | NM_001113380 | RGS4 | frameshift_inserti  on | exon2 | c.29dupA | p.Q10fs | . | . |
| CTC6 | 1 | 163306544 | CA | C | 0.9 | NM_031423 | NUF2 | frameshift_deleti  on | exon6 | c.342delA | p.A114fs | . | . |
| CTC6 | 1 | 166818828 | A | AT | 0.197 | NM_017542 | POGK | frameshift_inserti  on | exon5 | c.1012_1013ins  T | p.K338fs | . | . |
| CTC6 | 1 | 167666421 | TC | T | 0.75 | NM_052862 | RCSD1 | frameshift_deleti  on | exon6 | c.561delC | p.F187fs | . | . |
| CTC6 | 1 | 169511149 | GA | G | 0.478 | NM_000130 | F5 | frameshift_deleti  on | exon13 | c.3178delT | p.S1060fs | . | . |
| CTC6 | 1 | 169762695 | AT | A | 0.412 | NM_033418 | METTL18 | frameshift_deleti  on | exon2 | c.141delA | p.K47fs | . | . |

| CTC6 | 1 | 172635044 | TG | T | 0.615 | NM_000639 | FASLG | frameshift_deleti  on | exon4 | c.735delG | p.L245fs | . | . |
| --- | --- | --- | --- | --- | --- | --- | --- | --- | --- | --- | --- | --- | --- |
| CTC6 | 1 | 173744821 | TG | T | 0.932 | NM_014458 | KLHL20 | frameshift_deleti  on | exon10 | c.1479delG | p.M493fs | . | . |
| CTC6 | 1 | 175129924 | CCTT | C | 0.175 | NM_001162895 | KIAA0040 | nonframeshift_de  letion | exon3 | c.223_225del | p.75_75del | . | . |
| CTC6 | 1 | 176132086 | TC | T | 0.6 | NM_001001740 | RFWD2 | frameshift_deleti  on | exon5 | c.668delG | p.G223fs | . | . |
| CTC6 | 1 | 176863720 | ACT | A | 0.487 | NM_001286164 | ASTN1 | frameshift_deleti  on | exon17 | c.2916_2917del | p.L972fs | . | . |
| CTC6 | 1 | 176863804 | AG | A | 0.463 | NM_001286164 | ASTN1 | frameshift_deleti  on | exon17 | c.2833delC | p.L945fs | . | . |
| CTC6 | 1 | 179562834 | GC | G | 0.333 | NM_001199085 | TDRD5 | frameshift_deleti  on | exon3 | c.473delC | p.A158fs | . | . |
| CTC6 | 1 | 180974460 | ACT | A | 0.284 | NM_005819 | STX6 | frameshift_deleti  on | exon2 | c.173_174del | p.E58fs | . | . |
| CTC6 | 1 | 182443131 | GA | G | 0.562 | NM_001137669 | RGSL1 | frameshift_deleti  on | exon6 | c.886delA | p.K296fs | . | . |
| CTC6 | 1 | 183885694 | CA | C | 0.462 | NM_015149 | RGL1 | frameshift_deleti  on | exon17 | c.1969delA | p.K657fs | . | . |
| CTC6 | 1 | 184686086 | AT | A | 0.5 | NM_025191 | EDEM3 | frameshift_deleti  on | exon13 | c.1305delA | p.K435fs | . | . |
| CTC6 | 1 | 185062395 | CA | C | 0.278 | NM_007212 | RNF2 | frameshift_deleti  on | exon4 | c.452delA | p.Q151fs | . | . |
| CTC6 | 1 | 186360871 | AT | A | 0.333 | NM_001164246 | C1orf27 | frameshift_deleti  on | exon7 | c.564delT | p.Y188fs | . | . |
| CTC6 | 1 | 186646029 | TA | T | 0.941 | NM_000963 | PTGS2 | frameshift_deleti  on | exon6 | c.658delT | p.Y220fs | . | . |
| CTC6 | 1 | 193053820 | GCT | G | 0.115 | NM_001042370 | TROVE2 | frameshift_deleti  on | exon9 | c.1577_1578del | p.A526fs | . | . |
| CTC6 | 1 | 196309649 | CA | C | 0.296 | NM_001287820 | KCNT2 | frameshift_deleti  on | exon15 | c.1454delT | p.L485fs | . | . |
| CTC6 | 1 | 196642131 | AG | A | 0.148 | NM_000186 | CFH | frameshift_deleti  on | exon2 | c.83delG | p.R28fs | . | . |
| CTC6 | 1 | 196706616 | TG | T | 0.4 | NM_000186 | CFH | frameshift_deleti  on | exon17 | c.2609delG | p.C870fs | . | . |
| CTC6 | 1 | 196715118 | CA | C | 0.655 | NM_000186 | CFH | frameshift_deleti  on | exon21 | c.3483delA | p.P1161fs | . | . |
| CTC6 | 1 | 197112726 | AT | A | 0.46 | NM_001206846 | ASPM | frameshift_deleti  on | exon3 | c.655delA | p.I219fs | . | . |
| CTC6 | 1 | 200012912 | TA | T | 0.452 | NM_003822 | NR5A2 | frameshift_deleti  on | exon2 | c.76delA | p.K26fs | . | . |
| CTC6 | 1 | 200376576 | TC | T | 0.238 | NM_001281293 | ZNF281 | frameshift_deleti  on | exon2 | c.2257delG | p.D753fs | . | . |
| CTC6 | 1 | 201104918 | GC | G | 0.264 | NM_001288565 | TMEM9 | frameshift_deleti  on | exon5 | c.424delG | p.A142fs | . | . |
| CTC6 | 1 | 201175960 | GA | G | 1 | NM_001164586 | IGFN1 | frameshift_deleti  on | exon12 | c.1940delA | p.E647fs | . | . |
| CTC6 | 1 | 201352251 | TC | T | 0.309 | NM_005558 | LAD1 | frameshift_deleti  on | exon7 | c.1336delG | p.E446fs | . | . |
| CTC6 | 1 | 201751383 | TG | T | 0.379 | NM_001167738 | NAV1 | frameshift_deleti  on | exon4 | c.571delG | p.G191fs | . | . |
| CTC6 | 1 | 201832694 | GC | G | 0.321 | NM_018085 | IPO9 | frameshift_deleti  on | exon14 | c.1588delC | p.P530fs | . | . |
| CTC6 | 1 | 201980341 | TG | T | 0.414 | NM_001114309 | ELF3 | frameshift_deleti  on | exon2 | c.78delG | p.L26fs | . | . |
| CTC6 | 1 | 201983100 | CA | C | 0.509 | NM_001114309 | ELF3 | frameshift_deleti  on | exon8 | c.950delA | p.Q317fs | . | . |
| CTC6 | 1 | 202096997 | TG | T | 0.749 | NM_004767 | GPR37L1 | frameshift_deleti  on | exon2 | c.760delG | p.V254fs | . | . |
| CTC6 | 1 | 203186950 | C | CAGAC  CATGG CCCCG CCCAG | 0.788 | NM_001256125 | CHIT1 | stopgain | exon9 | c.1015_1016ins  AGGGACTGGG CGGGGCCATG GTCT | p.W339delinsX | . | . |
| CTC6 | 1 | 203186960 | C | CCCCG  CCCAG TCCCTA GACCA | 0.736 | NM_001256125 | CHIT1 | stopgain | exon9 | c.1005_1006ins  TCCATGGTCTA GGGACTGGGC GGG | p.A336delinsS MVX | . | . |
| CTC6 | 1 | 204433601 | TC | T | 0.353 | NM_002646 | PIK3C2B | frameshift_deleti  on | exon5 | c.1165delG | p.E389fs | . | . |
| CTC6 | 1 | 205028370 | TC | T | 0.613 | NM_005076 | CNTN2 | frameshift_deleti  on | exon6 | c.647delC | p.S216fs | . | . |
| CTC6 | 1 | 206154049 | TA | T | 0.261 | NM_001123168 | FAM72A | nonframeshift_de  letion | exon4 | c.449delA | p.X150X | . | . |
| CTC6 | 1 | 206647777 | AC | A | 0.593 | NM_001193322 | IKBKE | frameshift_deleti  on | exon4 | c.192delC | p.H64fs | . | . |
| CTC6 | 1 | 207870988 | C | CA | 0.174 | NM_175710 | CR1L | frameshift_inserti  on | exon6 | c.1004dupA | p.Q335fs | . | . |
| CTC6 | 1 | 207941135 | TC | T | 0.26 | NM_153826 | CD46 | frameshift_deleti  on | exon7 | c.869delC | p.S290fs | . | . |
| CTC6 | 1 | 208234072 | CA | C | 0.344 | NM_025179 | PLXNA2 | frameshift_deleti  on | exon13 | c.2696delT | p.V899fs | . | . |
| CTC6 | 1 | 209605636 | TAGCAG  CAGCAG | T | 0.45 | NM_001104548 | MIR205HG | nonframeshift_de  letion | exon4 | c.252_263del | p.84_88del | . | . |
| CTC6 | 1 | 211544722 | CA | C | 0.818 | NM_001033910 | TRAF5 | frameshift_deleti  on | exon10 | c.1010delA | p.Q337fs | . | . |
| CTC6 | 1 | 212141955 | G | GA | 0.346 | NM_001199809 | INTS7 | frameshift_inserti  on | exon13 | c.1762_1763ins  T | p.T588fs | . | . |
| CTC6 | 1 | 214557048 | GCCT | G | 0.149 | NM_005401 | PTPN14 | nonframeshift_de  letion | exon13 | c.2147_2149del | p.716_717del | . | . |
| CTC6 | 1 | 216052383 | TG | T | 0.5 | NM_206933 | USH2A | frameshift_deleti  on | exon42 | c.8280delC | p.H2760fs | . | . |
| CTC6 | 1 | 218480809 | TC | T | 0.378 | NM_016052 | RRP15 | frameshift_deleti  on | exon4 | c.541delC | p.Q181fs | . | . |
| CTC6 | 1 | 220280456 | TC | T | 1 | NM_018060 | IARS2 | frameshift_deleti  on | exon10 | c.1291delC | p.Q431fs | . | . |
| CTC6 | 1 | 220316335 | GA | G | 0.88 | NM_018060 | IARS2 | frameshift_deleti  on | exon21 | c.2611delA | p.K871fs | . | . |
| CTC6 | 1 | 220325046 | GC | G | 0.344 | NM_012414 | RAB3GAP2 | frameshift_deleti  on | exon34 | c.3927delG | p.G1309fs | . | . |
| CTC6 | 1 | 220971271 | ATC | A | 0.6 | NM_022746 | 1-Mar | frameshift_deleti  on | exon4 | c.669_670del | p.D223fs | . | . |
| CTC6 | 1 | 221057642 | GC | G | 0.5 | NM_021958 | HLX | frameshift_deleti  on | exon4 | c.1064delC | p.A355fs | . | . |
| CTC6 | 1 | 222837380 | TC | T | 0.262 | NM_198551 | MIA3 | frameshift_deleti  on | exon27 | c.5304delC | p.V1768fs | . | . |
| CTC6 | 1 | 223177565 | GT | G | 0.267 | NM_032890 | DISP1 | frameshift_deleti  on | exon10 | c.2827delT | p.F943fs | . | . |
| CTC6 | 1 | 223536702 | TTGC | T | 1 | NM_001037175 | SUSD4 | nonframeshift_de  letion | exon2 | c.63_65del | p.21_22del | . | . |
| CTC6 | 1 | 223536702 | TTGCTGC | T | 1 | NM_001037175 | SUSD4 | nonframeshift_de  letion | exon2 | c.60_65del | p.20_22del | . | . |
| CTC6 | 1 | 223567834 | GC | G | 0.333 | NM_152610 | C1orf65 | frameshift_deleti  on | exon1 | c.1018delC | p.P340fs | . | . |

| CTC6 | 1 | 223987708 | C | CT | 0.167 | NM_001031685 | TP53BP2 | frameshift_inserti  on | exon11 | c.1377dupA | p.V460fs | . | . |
| --- | --- | --- | --- | --- | --- | --- | --- | --- | --- | --- | --- | --- | --- |
| CTC6 | 1 | 224463050 | TG | T | 0.244 | NM_001243146 | NVL | stopgain | exon15 | c.1505delC | p.S502X | . | . |
| CTC6 | 1 | 225268291 | GA | G | 0.655 | NM_001373 | DNAH14 | frameshift_deleti on | exon19 | c.2978delA | p.E993fs | . | . |
| CTC6 | 1 | 225541519 | GA | G | 0.377 | NM_001373 | DNAH14 | frameshift_deleti  on | exon71 | c.11270delA | p.E3757fs | . | . |
| CTC6 | 1 | 225586808 | CT | C | 0.417 | NM_001373 | DNAH14 | frameshift_deleti  on | exon84 | c.13386delT | p.A4462fs | . | . |
| CTC6 | 1 | 226027586 | TG | T | 0.186 | NM_000120 | EPHX1 | frameshift_deleti  on | exon6 | c.780delG | p.L260fs | . | . |
| CTC6 | 1 | 226034898 | AT | A | 0.177 | NM_014698 | TMEM63A | frameshift_deleti  on | exon24 | c.2266delA | p.I756fs | . | . |
| CTC6 | 1 | 226065266 | CG | C | 0.667 | NM_014698 | TMEM63A | frameshift_deleti  on | exon3 | c.14delC | p.P5fs | . | . |
| CTC6 | 1 | 226178961 | TC | T | 0.183 | NM_152608 | SDE2 | frameshift_deleti  on | exon5 | c.623delG | p.G208fs | . | . |
| CTC6 | 1 | 227842721 | GA | G | 0.211 | NM_178549 | ZNF678 | frameshift_deleti  on | exon4 | c.936delA | p.R312fs | . | . |
| CTC6 | 1 | 228464367 | TG | T | 0.403 | NM_001098623 | OBSCN | frameshift_deleti  on | exon22 | c.6438delG | p.M2146fs | . | . |
| CTC6 | 1 | 228468327 | ATG | A | 0.39 | NM_001098623 | OBSCN | frameshift_deleti  on | exon30 | c.8028_8029del | p.H2676fs | . | . |
| CTC6 | 1 | 228596197 | A | AC | 0.207 | NM_001024940 | TRIM17 | frameshift_inserti  on | exon7 | c.1138dupG | p.V380fs | . | . |
| CTC6 | 1 | 228598690 | TGCA | T | 0.161 | NM_001024940 | TRIM17 | nonframeshift_de  letion | exon4 | c.710_712del | p.237_238del | . | . |
| CTC6 | 1 | 228601563 | GA | G | 0.667 | NM_001024940 | TRIM17 | frameshift_deleti  on | exon3 | c.459delT | p.L153fs | . | . |
| CTC6 | 1 | 229567253 | C | CT | 0.727 | NM_001100 | ACTA1 | stopgain | exon7 | c.1126_1127ins  A | p.C376_F377de  linsX | . | . |
| CTC6 | 1 | 230800326 | GC | G | 0.162 | NM_001145036 | COG2 | frameshift_deleti  on | exon5 | c.479delC | p.A160fs | . | . |
| CTC6 | 1 | 234367238 | CG | C | 0.2 | NM_173508 | SLC35F3 | frameshift_deleti  on | exon3 | c.360delG | p.A120fs | . | . |
| CTC6 | 1 | 234534276 | GC | G | 0.188 | NM_005646 | TARBP1 | frameshift_deleti  on | exon26 | c.4094delG | p.R1365fs | . | . |
| CTC6 | 1 | 235345881 | C | CT | 0.25 | NM_031371 | ARID4B | frameshift_inserti  on | exon19 | c.2094dupA | p.D699fs | . | . |
| CTC6 | 1 | 235866251 | TG | T | 0.294 | NM_000081 | LYST | frameshift_deleti  on | exon45 | c.10169delC | p.A3390fs | . | . |
| CTC6 | 1 | 235922635 | GT | G | 0.379 | NM_000081 | LYST | frameshift_deleti  on | exon23 | c.6517delA | p.T2173fs | . | . |
| CTC6 | 1 | 236176783 | TA | T | 0.348 | NM_002508 | NID1 | stopgain | exon11 | c.2331delT | p.C777X | . | . |
| CTC6 | 1 | 236212197 | AG | A | 0.375 | NM_002508 | NID1 | frameshift_deleti on | exon2 | c.317delC | p.P106fs | . | . |
| CTC6 | 1 | 236703917 | GA | G | 0.306 | NM_201544 | LGALS8 | frameshift_deleti  on | exon5 | c.400delA | p.K134fs | . | . |
| CTC6 | 1 | 236899899 | TC | T | 1 | NM_001278344 | ACTN2 | frameshift_deleti  on | exon10 | c.91delC | p.P31fs | . | . |
| CTC6 | 1 | 237058705 | C | CT | 0.196 | NM_000254 | MTR | frameshift_inserti  on | exon31 | c.3454dupT | p.A1151fs | . | . |
| CTC6 | 1 | 237824209 | CT | C | 0.633 | NM_001035 | RYR2 | frameshift_deleti  on | exon56 | c.8399delT | p.L2800fs | . | . |
| CTC6 | 1 | 243456516 | G | GA | 0.235 | NM_006642 | SDCCAG8 | frameshift_inserti  on | exon6 | c.671dupA | p.E224fs | . | . |
| CTC6 | 1 | 245530362 | TG | T | 0.961 | NM_018012 | KIF26B | frameshift_deleti  on | exon3 | c.693delG | p.V231fs | . | . |
| CTC6 | 1 | 247719725 | CA | C | 0.28 | NM_145278 | GCSAML | frameshift_deleti  on | exon2 | c.47delA | p.Q16fs | . | . |
| CTC6 | 1 | 247978541 | AAGG | A | 1 | NM_001001966 | OR14A16 | nonframeshift_de  letion | exon1 | c.488_490del | p.163_164del | . | . |
| CTC6 | 1 | 248129589 | TG | T | 0.2 | NM_001004491 | OR2AK2 | frameshift_deleti  on | exon1 | c.957delG | p.L319fs | . | . |
| CTC6 | 1 | 248525328 | C | CTA | 0.326 | NM_001004696 | OR2T4 | frameshift_inserti  on | exon1 | c.446_447insTA | p.T149fs | . | . |
| CTC6 | 1 | 248525638 | CA | C | 0.433 | NM_001004696 | OR2T4 | frameshift_deleti  on | exon1 | c.757delA | p.I253fs | . | . |
| CTC6 | 1 | 248844816 | CT | C | 0.958 | NM_001004734 | OR14I1 | frameshift_deleti  on | exon1 | c.789delA | p.K263fs | . | . |
| CTC6 | 1 | 249149830 | CA | C | 0.333 | NM_001193328 | ZNF692 | frameshift_deleti  on | exon7 | c.750delT | p.T250fs | . | . |
| CTC6 | 2 | 3730533 | TA | T | 1 | NM_018436 | ALLC | frameshift_deleti  on | exon7 | c.381delA | p.L127fs | . | . |
| CTC6 | 2 | 3730536 | AAT | A | 1 | NM_018436 | ALLC | frameshift_deleti  on | exon7 | c.384_385del | p.K128fs | . | . |
| CTC6 | 2 | 10904521 | GCT | G | 0.485 | NM_001039362 | ATP6V1C2 | frameshift_deleti  on | exon5 | c.349_350del | p.L117fs | . | . |
| CTC6 | 2 | 11742611 | TC | T | 0.48 | NM_014668 | GREB1 | frameshift_deleti  on | exon17 | c.2610delC | p.L870fs | . | . |
| CTC6 | 2 | 11922619 | CT | C | 0.377 | NM_145693 | LPIN1 | frameshift_deleti  on | exon7 | c.1143delT | p.P381fs | . | . |
| CTC6 | 2 | 17696983 | A | AT | 0.279 | NM_001099218 | RAD51AP2 | frameshift_inserti  on | exon1 | c.2699dupA | p.N900fs | . | . |
| CTC6 | 2 | 23980394 | AT | A | 0.316 | NM_001242338 | ATAD2B | frameshift_deleti  on | exon25 | c.3956delA | p.N1319fs | . | . |
| CTC6 | 2 | 24033218 | TA | T | 0.727 | NM_001242338 | ATAD2B | frameshift_deleti  on | exon18 | c.2421delT | p.V807fs | . | . |
| CTC6 | 2 | 24261868 | TGG | T | 0.333 | NM_001142319 | C2orf44 | frameshift_deleti  on | exon2 | c.495_496del | p.T165fs | . | . |
| CTC6 | 2 | 24261981 | CA | C | 0.205 | NM_001142319 | C2orf44 | frameshift_deleti  on | exon2 | c.383delT | p.L128fs | . | . |
| CTC6 | 2 | 24443877 | G | GC | 0.577 | NM_019595 | ITSN2 | frameshift_inserti  on | exon29 | c.3554dupG | p.G1185fs | . | . |
| CTC6 | 2 | 25467142 | TC | T | 0.273 | NM_153759 | DNMT3A | frameshift_deleti  on | exon11 | c.1165delG | p.E389fs | . | . |
| CTC6 | 2 | 25467199 | CA | C | 0.6 | NM_153759 | DNMT3A | frameshift_deleti  on | exon11 | c.1108delT | p.C370fs | . | . |
| CTC6 | 2 | 26203790 | TC | T | 0.808 | NM_002254 | KIF3C | frameshift_deleti  on | exon1 | c.996delG | p.G332fs | . | . |
| CTC6 | 2 | 26687863 | GCTT | G | 0.135 | NM_194322 | OTOF | nonframeshift_de  letion | exon21 | c.2761_2763del | p.921_921del | . | . |
| CTC6 | 2 | 26781399 | CG | C | 0.5 | NM_001287489 | OTOF | frameshift_deleti  on | exon1 | c.40delC | p.R14fs | . | . |
| CTC6 | 2 | 27307395 | AC | A | 0.455 | NM_007046 | EMILIN1 | frameshift_deleti  on | exon5 | c.2554delC | p.P852fs | . | . |
| CTC6 | 2 | 27550975 | TG | T | 0.345 | NM_001035521 | GTF3C2 | frameshift_deleti  on | exon17 | c.2337delC | p.P779fs | . | . |
| CTC6 | 2 | 27850339 | TG | T | 0.6 | NM_001142683 | CCDC121 | frameshift_deleti  on | exon2 | c.813delC | p.D271fs | . | . |
| CTC6 | 2 | 28002360 | GA | G | 0.419 | NM_145330 | MRPL33 | frameshift_deleti  on | exon3 | c.103delA | p.K35fs | . | . |
| CTC6 | 2 | 29092786 | CG | C | 0.229 | NM_017910 | TRMT61B | frameshift_deleti  on | exon1 | c.357delC | p.S119fs | . | . |

| CTC6 | 2 | 29287926 | C | CGCT | 0.75 | NM_001029883 | C2orf71 | nonframeshift_in  sertion | exon2 | c.3675_3676ins  AGC | p.E1226delinsS  E | . | . |
| --- | --- | --- | --- | --- | --- | --- | --- | --- | --- | --- | --- | --- | --- |
| CTC6 | 2 | 29451789 | CT | C | 0.286 | NM_004304 | ALK | frameshift_deleti  on | exon16 | c.2775delA | p.G925fs | . | . |
| CTC6 | 2 | 29754877 | CTG | C | 0.12 | NM_004304 | ALK | frameshift_deleti  on | exon4 | c.1056_1057del | p.H352fs | . | . |
| CTC6 | 2 | 32640227 | TA | T | 0.5 | NM_016252 | BIRC6 | frameshift_deleti  on | exon10 | c.1869delA | p.V623fs | . | . |
| CTC6 | 2 | 33042556 | TC | T | 0.306 | NM_001193509 | TTC27 | frameshift_deleti  on | exon19 | c.2192delC | p.S731fs | . | . |
| CTC6 | 2 | 33572566 | G | GA | 0.235 | NM_001166264 | LTBP1 | frameshift_inserti  on | exon21 | c.2885_2886ins  A | p.R962fs | . | . |
| CTC6 | 2 | 33812366 | CT | C | 0.25 | NM_015475 | FAM98A | frameshift_deleti  on | exon5 | c.543delA | p.K181fs | . | . |
| CTC6 | 2 | 43520094 | TC | T | 0.6 | NM_001083953 | THADA | frameshift_deleti  on | exon32 | c.4696delG | p.E1566fs | . | . |
| CTC6 | 2 | 43986093 | TG | T | 0.378 | NM_172069 | PLEKHH2 | frameshift_deleti  on | exon27 | c.3997delG | p.A1333fs | . | . |
| CTC6 | 2 | 44128641 | TG | T | 0.218 | NM_133259 | LRPPRC | frameshift_deleti  on | exon32 | c.3386delC | p.T1129fs | . | . |
| CTC6 | 2 | 44428546 | TA | T | 1 | NM_001033556 | PPM1B | frameshift_deleti  on | exon2 | c.209delA | p.Y70fs | . | . |
| CTC6 | 2 | 45616733 | CA | C | 0.786 | NM_018079 | SRBD1 | frameshift_deleti  on | exon21 | c.2703delT | p.F901fs | . | . |
| CTC6 | 2 | 46237539 | GA | G | 0.214 | NM_005400 | PRKCE | frameshift_deleti  on | exon10 | c.1321delA | p.K441fs | . | . |
| CTC6 | 2 | 53943792 | CA | C | 0.25 | NM_001201965 | ASB3 | frameshift_deleti  on | exon5 | c.473delT | p.L158fs | . | . |
| CTC6 | 2 | 54482703 | GGGGCC  C | G | 0.39 | NM_001003937 | TSPYL6 | nonframeshift_de  letion | exon1 | c.580_585del | p.194_195del | . | . |
| CTC6 | 2 | 54872533 | TA | T | 0.331 | NM_178313 | SPTBN1 | frameshift_deleti  on | exon20 | c.4399delA | p.N1467fs | . | . |
| CTC6 | 2 | 55253982 | CT | C | 0.321 | NM_020532 | RTN4 | frameshift_deleti  on | exon3 | c.1252delA | p.S418fs | . | . |
| CTC6 | 2 | 61190005 | TG | T | 0.429 | NM_144709 | PUS10 | frameshift_deleti  on | exon9 | c.743delC | p.A248fs | . | . |
| CTC6 | 2 | 61709642 | T | TA | 0.204 | NM_003400 | XPO1 | frameshift_inserti  on | exon23 | c.2844dupT | p.M949fs | . | . |
| CTC6 | 2 | 61719785 | TC | T | 0.6 | NM_003400 | XPO1 | frameshift_deleti  on | exon14 | c.1482delG | p.L494fs | . | . |
| CTC6 | 2 | 64863345 | GT | G | 0.572 | NM_014755 | SERTAD2 | frameshift_deleti  on | exon2 | c.660delA | p.K220fs | . | . |
| CTC6 | 2 | 65299684 | TC | T | 0.923 | NM_015147 | CEP68 | frameshift_deleti  on | exon3 | c.1455delC | p.L485fs | . | . |
| CTC6 | 2 | 67630506 | AT | A | 0.4 | NM_019002 | ETAA1 | stopgain | exon5 | c.693delT | p.Y231X | . | . |
| CTC6 | 2 | 68358416 | TC | T | 0.211 | NM_138458 | WDR92 | frameshift_deleti on | exon8 | c.1027delG | p.D343fs | . | . |
| CTC6 | 2 | 68882113 | CT | C | 0.458 | NM_138964 | PROKR1 | frameshift_deleti  on | exon2 | c.588delT | p.P196fs | . | . |
| CTC6 | 2 | 68882175 | TG | T | 0.966 | NM_138964 | PROKR1 | frameshift_deleti  on | exon2 | c.650delG | p.C217fs | . | . |
| CTC6 | 2 | 68962338 | CT | C | 0.258 | NM_001007231 | ARHGAP25 | frameshift_deleti  on | exon1 | c.8delT | p.L3fs | . | . |
| CTC6 | 2 | 69240746 | TG | T | 0.789 | NM_018153 | ANTXR1 | frameshift_deleti  on | exon1 | c.116delG | p.C39fs | . | . |
| CTC6 | 2 | 69736489 | GT | G | 0.5 | NM_014911 | AAK1 | frameshift_deleti  on | exon14 | c.1879delA | p.T627fs | . | . |
| CTC6 | 2 | 69741753 | CTGT | C | 1 | NM_014911 | AAK1 | nonframeshift_de  letion | exon13 | c.1623_1625del | p.541_542del | . | . |
| CTC6 | 2 | 69741759 | TTGTTGC | T | 0.75 | NM_014911 | AAK1 | nonframeshift_de  letion | exon13 | c.1614_1619del | p.538_540del | . | . |
| CTC6 | 2 | 71209092 | TGCA | T | 0.205 | NM_001115116 | ANKRD53 | nonframeshift_de  letion | exon4 | c.645_647del | p.215_216del | . | . |
| CTC6 | 2 | 71209094 | CA | C | 0.434 | NM_001115116 | ANKRD53 | frameshift_deleti  on | exon4 | c.647delA | p.H216fs | . | . |
| CTC6 | 2 | 71827907 | CTG | C | 0.946 | NM_001130976 | DYSF | frameshift_deleti  on | exon33 | c.3737_3738del | p.L1246fs | . | . |
| CTC6 | 2 | 72359393 | A | AT | 0.17 | NM_001277742 | CYP26B1 | frameshift_inserti  on | exon5 | c.1276_1277ins  A | p.L426fs | . | . |
| CTC6 | 2 | 74042268 | AC | A | 0.983 | NM_001080474 | C2orf78 | frameshift_deleti  on | exon3 | c.919delC | p.Q307fs | . | . |
| CTC6 | 2 | 74329117 | GC | G | 0.52 | NM_001287491 | TET3 | frameshift_deleti  on | exon11 | c.5203delC | p.Q1735fs | . | . |
| CTC6 | 2 | 74684514 | TG | T | 1 | NM_031288 | INO80B | stopgain | exon5 | c.595delG | p.V199X | . | . |
| CTC6 | 2 | 85024714 | GA | G | 0.3 | NM_001370 | DNAH6 | frameshift_deleti on | exon70 | c.11414delA | p.E3805fs | . | . |
| CTC6 | 2 | 85280423 | AT | A | 0.5 | NM_020122 | KCMF1 | frameshift_deleti  on | exon7 | c.1038delT | p.D346fs | . | . |
| CTC6 | 2 | 85626393 | AC | A | 1 | NM_001256139 | CAPG | frameshift_deleti  on | exon6 | c.531delG | p.W177fs | . | . |
| CTC6 | 2 | 86305325 | AT | A | 0.289 | NM_015425 | POLR1A | frameshift_deleti  on | exon10 | c.1168delA | p.I390fs | . | . |
| CTC6 | 2 | 96780974 | T | TTCCTC  CTCA | 0.714 | NM_000682 | ADRA2B | nonframeshift_in  sertion | exon1 | c.914_915insTG  AGGAGGA | p.E305delinsDE  EE | . | . |
| CTC6 | 2 | 96780986 | C | CTCCTC  CTCT | 0.947 | NM_000682 | ADRA2B | nonframeshift_in  sertion | exon1 | c.902_903insA  GAGGAGGA | p.E301delinsEE  EE | . | . |
| CTC6 | 2 | 96780986 | C | CTCCTC  T | 0.947 | NM_000682 | ADRA2B | nonframeshift_in  sertion | exon1 | c.902_903insA  GAGGA | p.E301delinsEE  E | . | . |
| CTC6 | 2 | 96947611 | GTTCA | G | 0.25 | NM_014014 | SNRNP200 | frameshift_deleti  on | exon35 | c.4961_4964del | p.M1654fs | . | . |
| CTC6 | 2 | 97007505 | AC | A | 0.333 | NM_001281710 | NCAPH | frameshift_deleti  on | exon2 | c.113delC | p.T38fs | . | . |
| CTC6 | 2 | 97007619 | TC | T | 0.731 | NM_001281710 | NCAPH | frameshift_deleti  on | exon2 | c.227delC | p.S76fs | . | . |
| CTC6 | 2 | 97217086 | AGT | A | 0.777 | NM_212481 | ARID5A | frameshift_deleti  on | exon7 | c.822_823del | p.Q274fs | . | . |
| CTC6 | 2 | 97514128 | TG | T | 0.35 | NM_016466 | ANKRD39 | frameshift_deleti  on | exon4 | c.461delC | p.P154fs | . | . |
| CTC6 | 2 | 97594955 | CA | C | 0.275 | NM_001122646 | FAM178B | frameshift_deleti  on | exon9 | c.1180delT | p.W394fs | . | . |
| CTC6 | 2 | 97749749 | TC | T | 0.5 | NM_199336 | FAHD2B | frameshift_deleti  on | exon7 | c.817delG | p.D273fs | . | . |
| CTC6 | 2 | 98275062 | T | TC | 0.387 | NM_005735 | ACTR1B | frameshift_inserti  on | exon6 | c.484dupG | p.D162fs | . | . |
| CTC6 | 2 | 99149879 | CA | C | 0.278 | NM_001134224 | INPP4A | frameshift_deleti  on | exon5 | c.192delA | p.P64fs | . | . |
| CTC6 | 2 | 99811241 | GA | G | 0.692 | NM_145212 | MRPL30 | frameshift_deleti  on | exon4 | c.161delA | p.E54fs | . | . |
| CTC6 | 2 | 103040811 | TA | T | 0.176 | NM_003853 | IL18RAP | frameshift_deleti  on | exon5 | c.517delA | p.I173fs | . | . |
| CTC6 | 2 | 109086779 | GA | G | 1 | NM_181453 | GCC2 | frameshift_deleti  on | exon6 | c.995delA | p.E332fs | . | . |

| CTC6 | 2 | 109384156 | TC | T | 0.404 | NM_006267 | RANBP2 | frameshift_deleti  on | exon20 | c.7162delC | p.P2388fs | . | . |
| --- | --- | --- | --- | --- | --- | --- | --- | --- | --- | --- | --- | --- | --- |
| CTC6 | 2 | 111435555 | AT | A | 0.333 | NM_001278616 | BUB1 | frameshift_deleti  on | exon1 | c.17delA | p.N6fs | . | . |
| CTC6 | 2 | 112832491 | TA | T | 0.229 | NM_032824 | TMEM87B | frameshift_deleti  on | exon5 | c.454delA | p.K152fs | . | . |
| CTC6 | 2 | 120194475 | CT | C | 0.242 | NM_183240 | TMEM37 | frameshift_deleti  on | exon2 | c.33delT | p.P11fs | . | . |
| CTC6 | 2 | 120194651 | A | AGTGT  GC | 1 | NM_183240 | TMEM37 | nonframeshift_in  sertion | exon2 | c.208_209insGT  GTGC | p.T70delinsSVP | . | . |
| CTC6 | 2 | 120362515 | GT | G | 0.647 | NM_001271049 | PCDP1 | frameshift_deleti  on | exon10 | c.977delT | p.V326fs | . | . |
| CTC6 | 2 | 128408606 | TG | T | 0.491 | NM_001161417 | GPR17 | frameshift_deleti  on | exon2 | c.298delG | p.G100fs | . | . |
| CTC6 | 2 | 128408907 | GC | G | 0.207 | NM_001161417 | GPR17 | frameshift_deleti  on | exon2 | c.599delC | p.A200fs | . | . |
| CTC6 | 2 | 128945168 | CA | C | 0.815 | NM_020120 | UGGT1 | frameshift_deleti  on | exon40 | c.4623delA | p.T1541fs | . | . |
| CTC6 | 2 | 130951828 | TC | T | 0.176 | NM_207312 | TUBA3E | frameshift_deleti  on | exon4 | c.586delG | p.E196fs | . | . |
| CTC6 | 2 | 131415071 | TC | T | 0.194 | NM_001277083 | POTEJ | frameshift_deleti  on | exon15 | c.2739delC | p.I913fs | . | . |
| CTC6 | 2 | 131812889 | AC | A | 0.488 | NM_001009993 | FAM168B | frameshift_deleti  on | exon5 | c.430delG | p.V144fs | . | . |
| CTC6 | 2 | 133540324 | TC | T | 0.483 | NM_207363 | NCKAP5 | frameshift_deleti  on | exon14 | c.4059delG | p.G1353fs | . | . |
| CTC6 | 2 | 135745761 | AT | A | 0.294 | NM_001018044 | MAP3K19 | frameshift_deleti  on | exon5 | c.341delA | p.N114fs | . | . |
| CTC6 | 2 | 152977256 | TG | T | 0.8 | NM_005843 | STAM2 | stopgain | exon14 | c.1409delC | p.S470X | . | . |
| CTC6 | 2 | 153476066 | G | GCCA | 1 | NM_052905 | FMNL2 | nonframeshift_in sertion | exon15 | c.1671_1672ins CCA | p.P557delinsPP | . | . |
| CTC6 | 2 | 155711330 | C | CT | 0.3 | NM_002239 | KCNJ3 | frameshift_inserti  on | exon3 | c.1012dupT | p.F337fs | . | . |
| CTC6 | 2 | 158962627 | GT | G | 0.244 | NM_173355 | UPP2 | frameshift_deleti  on | exon2 | c.80delT | p.V27fs | . | . |
| CTC6 | 2 | 160605065 | CAT | C | 0.273 | NM_001282806 | 7-Mar | frameshift_deleti  on | exon4 | c.1097_1098del | p.H366fs | . | . |
| CTC6 | 2 | 160605067 | TA | T | 0.922 | NM_001282806 | 7-Mar | frameshift_deleti  on | exon4 | c.1099delA | p.I367fs | . | . |
| CTC6 | 2 | 160833910 | T | TA | 0.556 | NM_001007267 | PLA2R1 | frameshift_inserti  on | exon15 | c.2285dupT | p.L762fs | . | . |
| CTC6 | 2 | 163055338 | CT | C | 0.259 | NM_004460 | FAP | frameshift_deleti  on | exon16 | c.1330delA | p.R444fs | . | . |
| CTC6 | 2 | 163134773 | TG | T | 0.167 | NM_022168 | IFIH1 | frameshift_deleti  on | exon9 | c.1706delC | p.P569fs | . | . |
| CTC6 | 2 | 164466149 | AT | A | 0.444 | NM_018086 | FIGN | frameshift_deleti  on | exon3 | c.2192delA | p.N731fs | . | . |
| CTC6 | 2 | 166514319 | AT | A | 0.355 | NM_024969 | CSRNP3 | frameshift_deleti  on | exon3 | c.198delT | p.H66fs | . | . |
| CTC6 | 2 | 167266242 | G | GA | 0.688 | NM_002976 | SCN7A | frameshift_inserti  on | exon24 | c.3914dupT | p.F1305fs | . | . |
| CTC6 | 2 | 168100100 | TA | T | 0.714 | NM_001199144 | XIRP2 | frameshift_deleti  on | exon7 | c.1533delA | p.I511fs | . | . |
| CTC6 | 2 | 169985538 | AT | A | 0.269 | NM_004525 | LRP2 | frameshift_deleti  on | exon78 | c.13784delA | p.N4595fs | . | . |
| CTC6 | 2 | 170103308 | AG | A | 0.857 | NM_004525 | LRP2 | frameshift_deleti  on | exon21 | c.3096delC | p.P1032fs | . | . |
| CTC6 | 2 | 173916470 | AC | A | 0.167 | NM_001282901 | RAPGEF4 | frameshift_deleti  on | exon25 | c.2352delC | p.H784fs | . | . |
| CTC6 | 2 | 175436670 | TGAG | T | 0.292 | NM_001077269 | WIPF1 | nonframeshift_de  letion | exon5 | c.860_862del | p.287_288del | . | . |
| CTC6 | 2 | 175677131 | CTT | C | 0.833 | NM_001206602 | CHN1 | frameshift_deleti  on | exon3 | c.415_416del | p.K139fs | . | . |
| CTC6 | 2 | 175677131 | CT | C | 0.833 | NM_001206602 | CHN1 | frameshift_deleti  on | exon3 | c.416delA | p.K139fs | . | . |
| CTC6 | 2 | 178482494 | CA | C | 0.605 | NM_152275 | TTC30A | frameshift_deleti  on | exon1 | c.935delT | p.L312fs | . | . |
| CTC6 | 2 | 179396183 | GA | G | 0.375 | NM_003319 | TTN | frameshift_deleti  on | exon186 | c.77963delT | p.F25988fs | . | . |
| CTC6 | 2 | 179399052 | AG | A | 0.216 | NM_003319 | TTN | stopgain | exon186 | c.75094delC | p.L25032X | . | . |
| CTC6 | 2 | 179407843 | G | GA | 0.64 | NM_003319 | TTN | frameshift_inserti on | exon175 | c.69661dupT | p.S23221fs | . | . |
| CTC6 | 2 | 179407931 | GTT | G | 0.188 | NM_003319 | TTN | frameshift_deleti  on | exon175 | c.69572_69573  del | p.K23191fs | . | . |
| CTC6 | 2 | 179413145 | ATT | A | 0.379 | NM_003319 | TTN | frameshift_deleti  on | exon167 | c.66011_66012  del | p.K22004fs | . | . |
| CTC6 | 2 | 179425009 | TC | T | 0.201 | NM_003319 | TTN | frameshift_deleti  on | exon154 | c.58654delG | p.E19552fs | . | . |
| CTC6 | 2 | 179437185 | CT | C | 0.31 | NM_003319 | TTN | frameshift_deleti  on | exon154 | c.46478delA | p.K15493fs | . | . |
| CTC6 | 2 | 179463233 | CT | C | 0.313 | NM_003319 | TTN | frameshift_deleti  on | exon120 | c.29915delA | p.K9972fs | . | . |
| CTC6 | 2 | 179528546 | CT | C | 0.333 | NM_001267550 | TTN | frameshift_deleti  on | exon170 | c.36447delA | p.K12149fs | . | . |
| CTC6 | 2 | 179530484 | CT | C | 0.28 | NM_001267550 | TTN | frameshift_deleti  on | exon164 | c.35909delA | p.K11970fs | . | . |
| CTC6 | 2 | 179613969 | GA | G | 0.391 | NM_133379 | TTN | frameshift_deleti  on | exon46 | c.13157delT | p.F4386fs | . | . |
| CTC6 | 2 | 179615047 | G | GT | 0.471 | NM_133379 | TTN | frameshift_inserti  on | exon46 | c.12079dupA | p.T4027fs | . | . |
| CTC6 | 2 | 179632598 | CG | C | 0.473 | NM_003319 | TTN | frameshift_deleti  on | exon39 | c.9220delC | p.R3074fs | . | . |
| CTC6 | 2 | 179638044 | AT | A | 0.255 | NM_003319 | TTN | frameshift_deleti  on | exon32 | c.7508delA | p.N2503fs | . | . |
| CTC6 | 2 | 179641028 | CT | C | 0.722 | NM_003319 | TTN | frameshift_deleti  on | exon27 | c.5424delA | p.E1808fs | . | . |
| CTC6 | 2 | 179701942 | TG | T | 0.458 | NM_173648 | CCDC141 | frameshift_deleti  on | exon23 | c.4003delC | p.H1335fs | . | . |
| CTC6 | 2 | 182387003 | GC | G | 0.267 | NM_000885 | ITGA4 | frameshift_deleti  on | exon18 | c.2009delC | p.A670fs | . | . |
| CTC6 | 2 | 185802211 | C | CACA | 1 | NM_194250 | ZNF804A | nonframeshift_in  sertion | exon4 | c.2088_2089ins  ACA | p.N696delinsN  T | . | . |
| CTC6 | 2 | 186659444 | GA | G | 0.774 | NM_173651 | FSIP2 | frameshift_deleti  on | exon16 | c.7849delA | p.K2617fs | . | . |
| CTC6 | 2 | 189964848 | TG | T | 0.357 | NM_000393 | COL5A2 | frameshift_deleti  on | exon4 | c.353delC | p.P118fs | . | . |
| CTC6 | 2 | 190428559 | TC | T | 0.3 | NM_014585 | SLC40A1 | frameshift_deleti  on | exon7 | c.1152delG | p.L384fs | . | . |
| CTC6 | 2 | 190593430 | CA | C | 0.52 | NM_144708 | ANKAR | frameshift_deleti  on | exon15 | c.3077delA | p.Q1026fs | . | . |
| CTC6 | 2 | 190611220 | AT | A | 0.25 | NM_144708 | ANKAR | frameshift_deleti  on | exon23 | c.4173delT | p.D1391fs | . | . |
| CTC6 | 2 | 190922098 | AG | A | 0.8 | NM_005259 | MSTN | frameshift_deleti  on | exon3 | c.1013delC | p.P338fs | . | . |

| CTC6 | 2 | 191898280 | TA | T | 0.855 | NM_001243835 | STAT4 | frameshift_deleti  on | exon20 | c.1787delT | p.L596fs | . | . |
| --- | --- | --- | --- | --- | --- | --- | --- | --- | --- | --- | --- | --- | --- |
| CTC6 | 2 | 196852759 | AT | A | 0.5 | NM_018897 | DNAH7 | frameshift_deleti  on | exon13 | c.1547delA | p.N516fs | . | . |
| CTC6 | 2 | 198948784 | TG | T | 0.273 | NM_006226 | PLCL1 | frameshift_deleti  on | exon2 | c.544delG | p.D182fs | . | . |
| CTC6 | 2 | 201397774 | TC | T | 1 | NM_001160033 | SGOL2 | stopgain | exon2 | c.77delC | p.S26X | . | . |
| CTC6 | 2 | 202173963 | TA | T | 0.259 | NM_001127391 | ALS2CR12 | frameshift_deleti on | exon10 | c.685delT | p.Y229fs | . | . |
| CTC6 | 2 | 202211392 | T | TA | 0.198 | NM_001127391 | ALS2CR12 | frameshift_inserti  on | exon5 | c.240_241insT | p.I81fs | . | . |
| CTC6 | 2 | 202357392 | CA | C | 0.5 | NM_001168221 | ALS2CR11 | frameshift_deleti  on | exon15 | c.3671delT | p.L1224fs | . | . |
| CTC6 | 2 | 202582860 | AT | A | 0.5 | NM_020919 | ALS2 | frameshift_deleti  on | exon24 | c.3775delA | p.I1259fs | . | . |
| CTC6 | 2 | 204064131 | AC | A | 0.217 | NM_001114132 | NBEAL1 | frameshift_deleti  on | exon48 | c.7113delC | p.Y2371fs | . | . |
| CTC6 | 2 | 204320254 | AG | A | 0.364 | NM_213589 | RAPH1 | frameshift_deleti  on | exon9 | c.1207delC | p.L403fs | . | . |
| CTC6 | 2 | 204360051 | AT | A | 0.541 | NM_203365 | RAPH1 | frameshift_deleti  on | exon2 | c.25delA | p.I9fs | . | . |
| CTC6 | 2 | 207530694 | TG | T | 0.284 | NM_001093730 | DYTN | frameshift_deleti  on | exon10 | c.1039delC | p.Q347fs | . | . |
| CTC6 | 2 | 209025604 | TC | T | 0.35 | NM_014617 | CRYGA | frameshift_deleti  on | exon3 | c.448delG | p.D150fs | . | . |
| CTC6 | 2 | 209165687 | GA | G | 1 | NM_152671 | PIKFYVE | frameshift_deleti  on | exon7 | c.787delA | p.K263fs | . | . |
| CTC6 | 2 | 209358096 | G | GA | 0.229 | NM_005048 | PTH2R | frameshift_inserti  on | exon13 | c.1366dupA | p.V455fs | . | . |
| CTC6 | 2 | 210787002 | CG | C | 0.4 | NM_032504 | UNC80 | frameshift_deleti  on | exon34 | c.5360delG | p.R1787fs | . | . |
| CTC6 | 2 | 211305451 | TA | T | 0.667 | NM_001136574 | LANCL1 | stopgain | exon6 | c.560delT | p.L187X | . | . |
| CTC6 | 2 | 215896650 | A | AT | 0.667 | NM_015657 | ABCA12 | frameshift_inserti on | exon1 | c.1dupA | p.M1fs | . | . |
| CTC6 | 2 | 218937186 | CAT | C | 0.4 | NM_198483 | RUFY4 | frameshift_deleti  on | exon5 | c.244_245del | p.M82fs | . | . |
| CTC6 | 2 | 219458969 | TC | T | 0.367 | NM_005444 | RQCD1 | frameshift_deleti  on | exon8 | c.871delC | p.P291fs | . | . |
| CTC6 | 2 | 219920367 | TG | T | 0.654 | NM_002181 | IHH | frameshift_deleti  on | exon3 | c.797delC | p.P266fs | . | . |
| CTC6 | 2 | 220037356 | GA | G | 0.792 | NM_015680 | CNPPD1 | frameshift_deleti  on | exon8 | c.1184delT | p.F395fs | . | . |
| CTC6 | 2 | 220072416 | TGG | T | 0.142 | NM_138802 | ZFAND2B | frameshift_deleti  on | exon3 | c.198_199del | p.V66fs | . | . |
| CTC6 | 2 | 220082482 | CA | C | 0.45 | NM_005689 | ABCB6 | frameshift_deleti  on | exon2 | c.596delT | p.L199fs | . | . |
| CTC6 | 2 | 220097212 | GA | G | 0.324 | NM_001042410 | ANKZF1 | frameshift_deleti  on | exon5 | c.366delA | p.G122fs | . | . |
| CTC6 | 2 | 220104340 | AC | A | 0.291 | NM_001286427 | GLB1L | frameshift_deleti  on | exon6 | c.574delG | p.V192fs | . | . |
| CTC6 | 2 | 220110798 | TG | T | 0.714 | NM_001008910 | STK16 | frameshift_deleti  on | exon2 | c.78delG | p.L26fs | . | . |
| CTC6 | 2 | 220239672 | GC | G | 0.222 | NM_012100 | DNPEP | frameshift_deleti  on | exon14 | c.1311delG | p.L437fs | . | . |
| CTC6 | 2 | 220316491 | GC | G | 0.312 | NM_005876 | SPEG | frameshift_deleti  on | exon6 | c.2403delC | p.G801fs | . | . |
| CTC6 | 2 | 222347379 | TG | T | 0.3 | NM_004438 | EPHA4 | stopgain | exon5 | c.1010delC | p.S337X | . | . |
| CTC6 | 2 | 224463736 | GA | G | 0.603 | NM_003469 | SCG2 | frameshift_deleti on | exon2 | c.264delT | p.L88fs | . | . |
| CTC6 | 2 | 225367730 | G | GA | 0.429 | NM_001257197 | CUL3 | frameshift_inserti  on | exon9 | c.1238dupT | p.I413fs | . | . |
| CTC6 | 2 | 227729504 | TA | T | 1 | NM_032276 | RHBDD1 | frameshift_deleti  on | exon2 | c.96delA | p.L32fs | . | . |
| CTC6 | 2 | 228110687 | CG | C | 1 | NM_000091 | COL4A3 | frameshift_deleti  on | exon6 | c.343delG | p.G115fs | . | . |
| CTC6 | 2 | 228399683 | G | GT | 0.221 | NM_001135189 | AGFG1 | frameshift_inserti  on | exon7 | c.1028dupT | p.V343fs | . | . |
| CTC6 | 2 | 230924032 | CA | C | 0.423 | NM_152527 | SLC16A14 | frameshift_deleti  on | exon2 | c.36delT | p.F12fs | . | . |
| CTC6 | 2 | 231309015 | GC | G | 0.189 | NM_001206703 | SP100 | frameshift_deleti  on | exon3 | c.319delC | p.Q107fs | . | . |
| CTC6 | 2 | 231861032 | TCAGCA  GCCTAG CCCTGA ATCCAC | T | 0.9 | NM_139073 | SPATA3 | nonframeshift_de letion | exon1 | c.85_111del | p.29_37del | . | . |
| CTC6 | 2 | 232079647 | AG | A | 0.545 | NM_025139 | ARMC9 | frameshift_deleti  on | exon4 | c.282delG | p.Q94fs | . | . |
| CTC6 | 2 | 232325414 | TTCA | T | 0.818 | NM_005381 | NCL | nonframeshift_de  letion | exon4 | c.774_776del | p.258_259del | . | . |
| CTC6 | 2 | 233127997 | AAG | A | 0.643 | NM_001257281 | DIS3L2 | frameshift_deleti  on | exon13 | c.1507_1508del | p.S503fs | . | . |
| CTC6 | 2 | 233410307 | GC | G | 0.262 | NM_005199 | CHRNG | frameshift_deleti  on | exon12 | c.1436delC | p.A479fs | . | . |
| CTC6 | 2 | 233412922 | T | TG | 0.342 | NM_145702 | TIGD1 | frameshift_inserti  on | exon1 | c.1670dupC | p.P557fs | . | . |
| CTC6 | 2 | 233712226 | CACA | C | 0.962 | NM_001103148 | GIGYF2 | nonframeshift_de  letion | exon26 | c.3612_3614del | p.1204_1205de  l | . | . |
| CTC6 | 2 | 234372900 | TG | T | 1 | NM_003648 | DGKD | frameshift_deleti  on | exon26 | c.3146delG | p.C1049fs | . | . |
| CTC6 | 2 | 234621819 | TC | T | 0.396 | NM_019078 | UGT1A5 | frameshift_deleti  on | exon1 | c.183delC | p.L61fs | . | . |
| CTC6 | 2 | 235949993 | CT | C | 0.299 | NM_014521 | SH3BP4 | frameshift_deleti  on | exon4 | c.581delT | p.L194fs | . | . |
| CTC6 | 2 | 235950457 | TG | T | 0.381 | NM_014521 | SH3BP4 | frameshift_deleti  on | exon4 | c.1045delG | p.G349fs | . | . |
| CTC6 | 2 | 236649676 | GC | G | 0.533 | NM_001037131 | AGAP1 | frameshift_deleti  on | exon4 | c.381delC | p.G127fs | . | . |
| CTC6 | 2 | 236761414 | C | CCAGG | 0.429 | NM_001244888 | AGAP1 | frameshift_inserti  on | exon10 | c.1135_1136ins  CAGG | p.P379fs | . | . |
| CTC6 | 2 | 238242174 | GT | G | 0.222 | NM_057166 | COL6A3 | frameshift_deleti  on | exon39 | c.7425delA | p.P2475fs | . | . |
| CTC6 | 2 | 238287402 | TA | T | 0.583 | NM_057164 | COL6A3 | frameshift_deleti  on | exon4 | c.1152delT | p.F384fs | . | . |
| CTC6 | 2 | 238287844 | CA | C | 0.815 | NM_057164 | COL6A3 | frameshift_deleti  on | exon4 | c.710delT | p.L237fs | . | . |
| CTC6 | 2 | 239342260 | AC | A | 0.48 | NM_001040445 | ASB1 | frameshift_deleti  on | exon2 | c.116delC | p.T39fs | . | . |
| CTC6 | 2 | 241465698 | GC | G | 0.758 | NM_016552 | ANKMY1 | frameshift_deleti  on | exon5 | c.850delG | p.A284fs | . | . |
| CTC6 | 2 | 241696840 | ATCC | A | 0.184 | NM_001244008 | KIF1A | nonframeshift_de  letion | exon27 | c.2751_2753del | p.917_918del | . | . |

| CTC6 | 2 | 242007259 | CG | C | 0.266 | NM_001080437 | SNED1 | frameshift_deleti  on | exon22 | c.3092delG | p.R1031fs | . | . |
| --- | --- | --- | --- | --- | --- | --- | --- | --- | --- | --- | --- | --- | --- |
| CTC6 | 3 | 3084806 | GCC | G | 0.198 | NM_001206956 | CNTN4 | frameshift_deleti  on | exon13 | c.1671_1672del | p.G557fs | . | . |
| CTC6 | 3 | 4726878 | TCC | T | 0.933 | NM_001168272 | ITPR1 | frameshift_deleti  on | exon29 | c.3646_3647del | p.P1216fs | . | . |
| CTC6 | 3 | 10018684 | GA | G | 1 | NM_018447 | EMC3 | frameshift_deleti  on | exon3 | c.263delT | p.F88fs | . | . |
| CTC6 | 3 | 10018684 | GAA | G | 1 | NM_018447 | EMC3 | frameshift_deleti  on | exon3 | c.262_263del | p.F88fs | . | . |
| CTC6 | 3 | 10116232 | GA | G | 0.444 | NM_001018115 | FANCD2 | frameshift_deleti  on | exon29 | c.2735delA | p.E912fs | . | . |
| CTC6 | 3 | 10327571 | TG | T | 0.583 | NM_001134946 | GHRL | frameshift_deleti  on | exon3 | c.188delC | p.P63fs | . | . |
| CTC6 | 3 | 10387773 | GT | G | 0.206 | NM_001683 | ATP2B2 | frameshift_deleti  on | exon14 | c.2317delA | p.T773fs | . | . |
| CTC6 | 3 | 10491199 | TA | T | 0.5 | NM_001001331 | ATP2B2 | frameshift_deleti  on | exon2 | c.28delT | p.Y10fs | . | . |
| CTC6 | 3 | 12610495 | CG | C | 0.421 | NM_014160 | MKRN2 | frameshift_deleti  on | exon2 | c.149delG | p.R50fs | . | . |
| CTC6 | 3 | 14197943 | TTA | T | 0.211 | NM_001145769 | XPC | frameshift_deleti  on | exon10 | c.1812_1813del | p.Y604fs | . | . |
| CTC6 | 3 | 14219965 | CCCT | C | 0.127 | NM_001145769 | XPC | nonframeshift_de  letion | exon1 | c.101_103del | p.34_35del | . | . |
| CTC6 | 3 | 15507935 | CT | C | 0.364 | NM_080539 | COLQ | frameshift_deleti  on | exon11 | c.624delA | p.K208fs | . | . |
| CTC6 | 3 | 15606107 | CA | C | 0.75 | NM_001284416 | HACL1 | frameshift_deleti  on | exon12 | c.1221delT | p.F407fs | . | . |
| CTC6 | 3 | 15686236 | C | CT | 0.243 | NM_000060 | BTD | frameshift_inserti  on | exon4 | c.874dupT | p.A291fs | . | . |
| CTC6 | 3 | 15719831 | GTC | G | 0.353 | NM_001195099 | ANKRD28 | frameshift_deleti  on | exon22 | c.2038_2039del | p.D680fs | . | . |
| CTC6 | 3 | 17052905 | TC | T | 0.458 | NM_015184 | PLCL2 | frameshift_deleti  on | exon2 | c.1690delC | p.Q564fs | . | . |
| CTC6 | 3 | 20017220 | A | AT | 0.375 | NM_004162 | RAB5A | frameshift_inserti  on | exon3 | c.291_292insT | p.I97fs | . | . |
| CTC6 | 3 | 23942405 | GA | G | 0.311 | NM_020345 | NKIRAS1 | frameshift_deleti  on | exon4 | c.229delT | p.S77fs | . | . |
| CTC6 | 3 | 24188247 | AT | A | 0.8 | NM_000461 | THRB | frameshift_deleti  on | exon6 | c.450delA | p.K150fs | . | . |
| CTC6 | 3 | 32031367 | GT | G | 0.4 | NM_001137674 | ZNF860 | frameshift_deleti  on | exon2 | c.797delT | p.V266fs | . | . |
| CTC6 | 3 | 32169737 | GA | G | 0.714 | NM_015141 | GPD1L | frameshift_deleti  on | exon2 | c.218delA | p.E73fs | . | . |
| CTC6 | 3 | 33166059 | C | CT | 0.165 | NM_006371 | CRTAP | frameshift_inserti  on | exon3 | c.782dupT | p.L261fs | . | . |
| CTC6 | 3 | 33870331 | AT | A | 0.83 | NM_001162429 | PDCD6IP | frameshift_deleti  on | exon7 | c.720delT | p.Y240fs | . | . |
| CTC6 | 3 | 36779337 | GT | G | 0.263 | NM_033403 | DCLK3 | frameshift_deleti  on | exon2 | c.813delA | p.K271fs | . | . |
| CTC6 | 3 | 36887755 | CT | C | 0.263 | NM_014831 | TRANK1 | frameshift_deleti  on | exon16 | c.5042delA | p.K1681fs | . | . |
| CTC6 | 3 | 37081775 | AC | A | 0.263 | NM_001167619 | MLH1 | frameshift_deleti  on | exon13 | c.935delC | p.T312fs | . | . |
| CTC6 | 3 | 38017355 | TG | T | 0.837 | NM_005808 | CTDSPL | frameshift_deleti  on | exon6 | c.643delG | p.A215fs | . | . |
| CTC6 | 3 | 38049975 | AC | A | 0.339 | NM_001130964 | PLCD1 | frameshift_deleti  on | exon12 | c.1938delG | p.W646fs | . | . |
| CTC6 | 3 | 38263141 | GC | G | 0.262 | NM_005109 | OXSR1 | frameshift_deleti  on | exon6 | c.564delC | p.G188fs | . | . |
| CTC6 | 3 | 38317562 | TATC | T | 0.393 | NM_004256 | SLC22A13 | nonframeshift_de  letion | exon7 | c.1213_1215del | p.405_405del | . | . |
| CTC6 | 3 | 38812773 | AG | A | 0.727 | NM_006514 | SCN10A | frameshift_deleti  on | exon4 | c.595delC | p.L199fs | . | . |
| CTC6 | 3 | 40503520 | A | ACTGC  TG | 1 | NM_001034996 | RPL14 | nonframeshift_in  sertion | exon6 | c.445_446insCT  GCTG | p.T149delinsTA  A | . | . |
| CTC6 | 3 | 40503520 | A | ACTG | 1 | NM_001034996 | RPL14 | nonframeshift_in  sertion | exon6 | c.445_446insCT  G | p.T149delinsTA | . | . |
| CTC6 | 3 | 40503524 | C | CTGCT  GG | 0.789 | NM_001034996 | RPL14 | nonframeshift_in  sertion | exon6 | c.449_450insTG  CTGG | p.A150delinsA  AG | . | . |
| CTC6 | 3 | 42251577 | C | CGGAG  GA | 0.833 | NM_001265609 | TRAK1 | nonframeshift_in  sertion | exon13 | c.1841_1842ins  GGAGGA | p.T614delinsTE  E | . | . |
| CTC6 | 3 | 42251577 | C | CGGA | 0.833 | NM_001265609 | TRAK1 | nonframeshift_in  sertion | exon13 | c.1841_1842ins  GGA | p.T614delinsTE | . | . |
| CTC6 | 3 | 42251608 | G | GGAGG  AA | 0.462 | NM_001265609 | TRAK1 | nonframeshift_in  sertion | exon13 | c.1872_1873ins  GAGGAA | p.E624delinsEE  E | . | . |
| CTC6 | 3 | 44636646 | CAG | C | 0.429 | NM_173658 | ZNF660 | frameshift_deleti  on | exon3 | c.962_963del | p.Q321fs | . | . |
| CTC6 | 3 | 45038592 | AC | A | 0.939 | NM_015004 | EXOSC7 | frameshift_deleti  on | exon4 | c.269delC | p.T90fs | . | . |
| CTC6 | 3 | 45130546 | TTC | T | 0.5 | NM_022842 | CDCP1 | frameshift_deleti  on | exon8 | c.2079_2080del | p.K693fs | . | . |
| CTC6 | 3 | 45764379 | GT | G | 0.545 | NM_014016 | SACM1L | frameshift_deleti  on | exon10 | c.785delT | p.V262fs | . | . |
| CTC6 | 3 | 46306908 | CT | C | 0.455 | NM_178329 | CCR3 | frameshift_deleti  on | exon2 | c.260delT | p.L87fs | . | . |
| CTC6 | 3 | 46415215 | GT | G | 0.227 | NM_000579 | CCR5 | frameshift_deleti  on | exon3 | c.823delT | p.L275fs | . | . |
| CTC6 | 3 | 46491441 | CT | C | 0.6 | NM_001199149 | LTF | frameshift_deleti  on | exon8 | c.827delA | p.K276fs | . | . |
| CTC6 | 3 | 46751073 | TAAG | T | 1 | NM_147196 | TMIE | nonframeshift_de  letion | exon4 | c.367_369del | p.123_123del | . | . |
| CTC6 | 3 | 46751073 | TAAGAA  G | T | 1 | NM_147196 | TMIE | nonframeshift_de  letion | exon4 | c.367_372del | p.123_124del | . | . |
| CTC6 | 3 | 47037091 | CA | C | 0.273 | NM_015175 | NBEAL2 | frameshift_deleti  on | exon13 | c.1867delA | p.T623fs | . | . |
| CTC6 | 3 | 48602555 | AG | A | 0.429 | NM_000094 | COL7A1 | frameshift_deleti  on | exon116 | c.8606delC | p.P2869fs | . | . |
| CTC6 | 3 | 48828035 | TG | T | 0.429 | NM_004157 | PRKAR2A | frameshift_deleti  on | exon4 | c.376delC | p.Q126fs | . | . |
| CTC6 | 3 | 48965063 | GGAA | G | 0.204 | NM_006321 | ARIH2 | nonframeshift_de  letion | exon3 | c.73_75del | p.25_25del | . | . |
| CTC6 | 3 | 49701067 | AG | A | 0.636 | NM_003458 | BSN | frameshift_deleti  on | exon7 | c.11477delG | p.S3826fs | . | . |
| CTC6 | 3 | 49755522 | TG | T | 0.193 | NM_198722 | AMIGO3 | frameshift_deleti  on | exon1 | c.1376delC | p.P459fs | . | . |
| CTC6 | 3 | 49947903 | AT | A | 0.25 | NM_001142501 | MON1A | frameshift_deleti  on | exon3 | c.832delA | p.I278fs | . | . |
| CTC6 | 3 | 50005469 | AT | A | 0.862 | NM_005777 | RBM6 | frameshift_deleti  on | exon3 | c.612delT | p.D204fs | . | . |
| CTC6 | 3 | 50144247 | GA | G | 0.556 | NM_005778 | RBM5 | frameshift_deleti  on | exon11 | c.904delA | p.K302fs | . | . |
| CTC6 | 3 | 50386429 | GT | G | 0.207 | NM_006545 | NPRL2 | frameshift_deleti  on | exon5 | c.460delA | p.T154fs | . | . |

| CTC6 | 3 | 52240628 | G | GA | 0.259 | NM_199166 | ALAS1 | frameshift_inserti  on | exon7 | c.1027dupA | p.M342fs | . | . |
| --- | --- | --- | --- | --- | --- | --- | --- | --- | --- | --- | --- | --- | --- |
| CTC6 | 3 | 52544379 | TG | T | 0.174 | NM_015136 | STAB1 | frameshift_deleti  on | exon25 | c.2644delG | p.G882fs | . | . |
| CTC6 | 3 | 52554869 | C | CT | 0.4 | NM_015136 | STAB1 | frameshift_inserti  on | exon55 | c.5757dupT | p.P1919fs | . | . |
| CTC6 | 3 | 52817125 | CT | C | 0.378 | NM_001166435 | ITIH1 | frameshift_deleti  on | exon5 | c.220delT | p.F74fs | . | . |
| CTC6 | 3 | 53804012 | GA | G | 0.25 | NM_001128840 | CACNA1D | frameshift_deleti  on | exon31 | c.3884delA | p.E1295fs | . | . |
| CTC6 | 3 | 53853055 | GC | G | 0.352 | NM_018397 | CHDH | frameshift_deleti  on | exon8 | c.1275delG | p.G425fs | . | . |
| CTC6 | 3 | 53886138 | A | AC | 0.239 | NM_018725 | IL17RB | frameshift_inserti  on | exon4 | c.340dupC | p.R113fs | . | . |
| CTC6 | 3 | 56330062 | G | GT | 0.727 | NM_015576 | ERC2 | frameshift_inserti  on | exon3 | c.1058dupA | p.N353fs | . | . |
| CTC6 | 3 | 56591278 | T | TGGGG  TAAGC | 0.392 | NM_001141947 | CCDC66 | stopgain | exon1 | c.8_9insGGGGT  AAGCA | p.L3_G4delinsL  GX | . | . |
| CTC6 | 3 | 56591279 | G | GGGGT  AAGCA | 0.216 | NM_001141947 | CCDC66 | stopgain | exon1 | c.9_10insGGGT  AAGCA | p.L3delinsLGX | . | . |
| CTC6 | 3 | 56650054 | T | TCTC | 1 | NM_001012506 | CCDC66 | nonframeshift_in  sertion | exon13 | c.1714_1715ins  CTC | p.S572delinsSP | . | . |
| CTC6 | 3 | 56667527 | TCC | T | 0.4 | NM_015224 | FAM208A | frameshift_deleti  on | exon11 | c.1979_1980del | p.G660fs | . | . |
| CTC6 | 3 | 57394206 | GC | G | 0.435 | NM_178504 | DNAH12 | frameshift_deleti  on | exon40 | c.6019delG | p.A2007fs | . | . |
| CTC6 | 3 | 57614583 | TG | T | 0.75 | NM_152678 | DENND6A | frameshift_deleti  on | exon19 | c.1649delC | p.T550fs | . | . |
| CTC6 | 3 | 58106987 | TC | T | 0.519 | NM_001164317 | FLNB | frameshift_deleti  on | exon20 | c.2884delC | p.Q962fs | . | . |
| CTC6 | 3 | 58135704 | CA | C | 0.375 | NM_001164319 | FLNB | frameshift_deleti  on | exon36 | c.6148delA | p.K2050fs | . | . |
| CTC6 | 3 | 64608173 | CCA | C | 0.75 | NM_182920 | ADAMTS9 | frameshift_deleti  on | exon17 | c.2477_2478del | p.V826fs | . | . |
| CTC6 | 3 | 65425560 | T | TCTG | 1 | NM_001033057 | MAGI1 | nonframeshift_in  sertion | exon9 | c.1263_1264ins  CAG | p.T422delinsQT | . | . |
| CTC6 | 3 | 65425572 | G | GCTA | 1 | NM_001033057 | MAGI1 | stopgain | exon9 | c.1251_1252ins  TAG | p.Q418delinsX | . | . |
| CTC6 | 3 | 69928490 | AC | A | 0.571 | NM_001184967 | MITF | frameshift_deleti  on | exon2 | c.155delC | p.T52fs | . | . |
| CTC6 | 3 | 69987121 | TC | T | 0.429 | NM_000248 | MITF | frameshift_deleti  on | exon2 | c.183delC | p.V61fs | . | . |
| CTC6 | 3 | 71247356 | TTGC | T | 1 | NM_001244814 | FOXP1 | nonframeshift_de  letion | exon2 | c.174_176del | p.58_59del | . | . |
| CTC6 | 3 | 73112824 | GA | G | 0.778 | NM_174907 | PPP4R2 | frameshift_deleti  on | exon6 | c.421delA | p.K141fs | . | . |
| CTC6 | 3 | 97851660 | TG | T | 1 | NM_001005338 | OR5H1 | frameshift_deleti  on | exon1 | c.120delG | p.M40fs | . | . |
| CTC6 | 3 | 98250912 | A | AT | 0.387 | NM_005290 | GPR15 | frameshift_inserti  on | exon1 | c.36dupT | p.Y12fs | . | . |
| CTC6 | 3 | 100962415 | GT | G | 0.486 | NM_016247 | IMPG2 | frameshift_deleti  on | exon13 | c.2759delA | p.N920fs | . | . |
| CTC6 | 3 | 101576290 | CT | C | 1 | NM_031419 | NFKBIZ | frameshift_deleti  on | exon11 | c.2091delT | p.P697fs | . | . |
| CTC6 | 3 | 108406914 | TC | T | 0.267 | NM_014648 | DZIP3 | frameshift_deleti  on | exon29 | c.3242delC | p.S1081fs | . | . |
| CTC6 | 3 | 110831158 | TG | T | 0.213 | NM_001243286 | PVRL3 | frameshift_deleti  on | exon2 | c.443delG | p.C148fs | . | . |
| CTC6 | 3 | 111603841 | CT | C | 0.304 | NM_001134438 | PHLDB2 | frameshift_deleti  on | exon2 | c.918delT | p.S306fs | . | . |
| CTC6 | 3 | 111766783 | CT | C | 0.742 | NM_001042575 | TMPRSS7 | frameshift_deleti  on | exon5 | c.551delT | p.L184fs | . | . |
| CTC6 | 3 | 111795970 | TG | T | 0.739 | NM_001042575 | TMPRSS7 | frameshift_deleti  on | exon14 | c.1826delG | p.W609fs | . | . |
| CTC6 | 3 | 112709959 | GGCTGC  CGAAGC | G | 0.514 | NM_014170 | GTPBP8 | nonframeshift_de  letion | exon1 | c.114_125del | p.38_42del | . | . |
| CTC6 | 3 | 113172590 | AC | A | 0.304 | NM_144718 | SPICE1 | frameshift_deleti  on | exon14 | c.1864delG | p.V622fs | . | . |
| CTC6 | 3 | 113376110 | TTGC | T | 1 | NM_001009899 | KIAA2018 | nonframeshift_de  letion | exon7 | c.4416_4418del | p.1472_1473de  l | . | . |
| CTC6 | 3 | 113376399 | TG | T | 0.231 | NM_001009899 | KIAA2018 | frameshift_deleti  on | exon7 | c.4129delC | p.Q1377fs | . | . |
| CTC6 | 3 | 113379205 | TG | T | 0.805 | NM_001009899 | KIAA2018 | frameshift_deleti  on | exon7 | c.1323delC | p.N441fs | . | . |
| CTC6 | 3 | 113655271 | GC | G | 0.418 | NM_001172105 | GRAMD1C | frameshift_deleti  on | exon9 | c.1001delC | p.A334fs | . | . |
| CTC6 | 3 | 121207823 | CA | C | 0.7 | NM_199420 | POLQ | frameshift_deleti  on | exon16 | c.3954delT | p.F1318fs | . | . |
| CTC6 | 3 | 121415515 | TA | T | 0.625 | NM_001256488 | GOLGB1 | stopgain | exon12 | c.3614delT | p.L1205X | . | . |
| CTC6 | 3 | 122274337 | AC | A | 0.5 | NM_001146102 | PARP9 | frameshift_deleti on | exon4 | c.785delG | p.S262fs | . | . |
| CTC6 | 3 | 122474189 | AT | A | 0.286 | NM_024610 | HSPBAP1 | frameshift_deleti  on | exon5 | c.658delA | p.I220fs | . | . |
| CTC6 | 3 | 122564619 | AT | A | 0.818 | NM_032839 | DIRC2 | frameshift_deleti  on | exon5 | c.844delT | p.F282fs | . | . |
| CTC6 | 3 | 123337552 | AG | A | 0.583 | NM_053031 | MYLK | frameshift_deleti  on | exon2 | c.150delC | p.F50fs | . | . |
| CTC6 | 3 | 124393228 | CA | C | 0.8 | NM_007064 | KALRN | frameshift_deleti  on | exon16 | c.1870delA | p.N624fs | . | . |
| CTC6 | 3 | 124646705 | A | AAAG | 1 | NM_033049 | MUC13 | nonframeshift_in  sertion | exon2 | c.184_185insCT  T | p.F62delinsSF | . | . |
| CTC6 | 3 | 124738398 | AT | A | 0.6 | NM_020733 | HEG1 | frameshift_deleti  on | exon5 | c.1295delA | p.N432fs | . | . |
| CTC6 | 3 | 125266384 | AC | A | 1 | NM_022776 | OSBPL11 | frameshift_deleti  on | exon10 | c.1706delG | p.C569fs | . | . |
| CTC6 | 3 | 125854436 | AG | A | 1 | NM_001270365 | ALDH1L1 | frameshift_deleti  on | exon10 | c.1110delC | p.A370fs | . | . |
| CTC6 | 3 | 127379587 | TG | T | 0.333 | NM_015720 | PODXL2 | frameshift_deleti  on | exon3 | c.717delG | p.M239fs | . | . |
| CTC6 | 3 | 127965845 | TA | T | 0.545 | NM_021937 | EEFSEC | frameshift_deleti  on | exon2 | c.484delA | p.K162fs | . | . |
| CTC6 | 3 | 127965857 | GA | G | 0.562 | NM_021937 | EEFSEC | frameshift_deleti  on | exon2 | c.496delA | p.K166fs | . | . |
| CTC6 | 3 | 129278491 | C | CG | 0.22 | NM_015103 | PLXND1 | frameshift_inserti  on | exon32 | c.5268dupC | p.D1757fs | . | . |
| CTC6 | 3 | 131102018 | GT | G | 0.883 | NM_001171905 | NUDT16 | frameshift_deleti  on | exon3 | c.284delT | p.V95fs | . | . |
| CTC6 | 3 | 132400797 | GT | G | 0.444 | NM_153240 | NPHP3 | frameshift_deleti  on | exon27 | c.3949delA | p.T1317fs | . | . |
| CTC6 | 3 | 132423109 | TC | T | 0.314 | NM_153240 | NPHP3 | frameshift_deleti  on | exon9 | c.1456delG | p.D486fs | . | . |
| CTC6 | 3 | 133664074 | AG | A | 0.282 | NM_005630 | SLCO2A1 | frameshift_deleti  on | exon10 | c.1325delC | p.P442fs | . | . |

| CTC6 | 3 | 134078998 | G | GA | 0.8 | NM_001278683 | AMOTL2 | frameshift_inserti  on | exon7 | c.2006dupT | p.F669fs | . | . |
| --- | --- | --- | --- | --- | --- | --- | --- | --- | --- | --- | --- | --- | --- |
| CTC6 | 3 | 134968256 | CA | C | 0.239 | NM_004441 | EPHB1 | frameshift_deleti  on | exon15 | c.2770delA | p.K924fs | . | . |
| CTC6 | 3 | 136196145 | TC | T | 0.208 | NM_005862 | STAG1 | stopgain | exon10 | c.1011delG | p.W337X | . | . |
| CTC6 | 3 | 136196169 | TG | T | 0.422 | NM_005862 | STAG1 | frameshift_deleti on | exon10 | c.987delC | p.D329fs | . | . |
| CTC6 | 3 | 137889026 | CA | C | 0.9 | NM_016216 | DBR1 | frameshift_deleti  on | exon4 | c.411delT | p.F137fs | . | . |
| CTC6 | 3 | 140401722 | TG | T | 0.5 | NM_152616 | TRIM42 | frameshift_deleti  on | exon2 | c.761delG | p.C254fs | . | . |
| CTC6 | 3 | 141162127 | AC | A | 0.581 | NM_001080412 | ZBTB38 | frameshift_deleti  on | exon8 | c.898delC | p.P300fs | . | . |
| CTC6 | 3 | 142142408 | GT | G | 0.427 | NM_001282857 | XRN1 | frameshift_deleti  on | exon6 | c.703delA | p.T235fs | . | . |
| CTC6 | 3 | 142274961 | G | GA | 0.625 | NM_001184 | ATR | frameshift_inserti  on | exon10 | c.2098dupT | p.S700fs | . | . |
| CTC6 | 3 | 142389909 | TG | T | 0.288 | NM_001145319 | PLS1 | frameshift_deleti  on | exon4 | c.310delG | p.A104fs | . | . |
| CTC6 | 3 | 142772536 | GA | G | 0.295 | NM_001080415 | U2SURP | frameshift_deleti  on | exon26 | c.2675delA | p.E892fs | . | . |
| CTC6 | 3 | 148876541 | AC | A | 0.25 | NM_032383 | HPS3 | frameshift_deleti  on | exon10 | c.1781delC | p.T594fs | . | . |
| CTC6 | 3 | 149040106 | GC | G | 0.723 | NM_001184723 | TM4SF18 | frameshift_deleti  on | exon4 | c.527delG | p.C176fs | . | . |
| CTC6 | 3 | 150127235 | TG | T | 1 | NM_014779 | TSC22D2 | frameshift_deleti  on | exon1 | c.99delG | p.L33fs | . | . |
| CTC6 | 3 | 154834527 | GA | G | 0.968 | NM_000902 | MME | frameshift_deleti  on | exon6 | c.515delA | p.E172fs | . | . |
| CTC6 | 3 | 155199977 | CTGGTT | C | 0.25 | NM_014996 | PLCH1 | frameshift_deleti  on | exon22 | c.3743_3747del | p.K1248fs | . | . |
| CTC6 | 3 | 160073855 | TC | T | 0.282 | NM_001190242 | IFT80 | frameshift_deleti  on | exon7 | c.311delG | p.G104fs | . | . |
| CTC6 | 3 | 160155773 | AT | A | 0.385 | NM_173084 | TRIM59 | stopgain | exon3 | c.1198delA | p.I400X | . | . |
| CTC6 | 3 | 160155982 | TA | T | 0.467 | NM_173084 | TRIM59 | stopgain | exon3 | c.989delT | p.L330X | . | . |
| CTC6 | 3 | 169485334 | TG | T | 0.917 | NM_032487 | ACTRT3 | stopgain | exon2 | c.1004delC | p.S335X | . | . |
| CTC6 | 3 | 169866911 | G | GT | 0.8 | NM_024947 | PHC3 | frameshift_inserti on | exon5 | c.535dupA | p.T179fs | . | . |
| CTC6 | 3 | 170016838 | GTT | G | 0.15 | NM_002740 | PRKCI | frameshift_deleti  on | exon17 | c.1644_1645del | p.G548fs | . | . |
| CTC6 | 3 | 170102352 | GC | G | 0.56 | NM_001145097 | SKIL | frameshift_deleti  on | exon3 | c.1229delC | p.A410fs | . | . |
| CTC6 | 3 | 172502533 | CT | C | 0.318 | NM_018098 | ECT2 | frameshift_deleti  on | exon16 | c.1673delT | p.L558fs | . | . |
| CTC6 | 3 | 173999030 | AC | A | 0.75 | NM_014932 | NLGN1 | frameshift_deleti  on | exon7 | c.2410delC | p.Q804fs | . | . |
| CTC6 | 3 | 178962327 | C | CT | 0.429 | NM_171830 | KCNMB3 | frameshift_inserti  on | exon2 | c.402dupA | p.A135fs | . | . |
| CTC6 | 3 | 178968545 | C | CT | 0.304 | NM_171830 | KCNMB3 | frameshift_inserti  on | exon1 | c.233dupA | p.K78fs | . | . |
| CTC6 | 3 | 179137290 | TA | T | 0.586 | NM_021629 | GNB4 | frameshift_deleti  on | exon4 | c.99delT | p.I33fs | . | . |
| CTC6 | 3 | 182553917 | CA | C | 0.857 | NM_014616 | ATP11B | frameshift_deleti  on | exon5 | c.411delA | p.S137fs | . | . |
| CTC6 | 3 | 183493743 | CGGA | C | 0.968 | NM_018023 | YEATS2 | nonframeshift_de  letion | exon18 | c.2410_2412del | p.804_804del | . | . |
| CTC6 | 3 | 183677531 | CT | C | 0.26 | NM_005688 | ABCC5 | frameshift_deleti  on | exon17 | c.2471delA | p.K824fs | . | . |
| CTC6 | 3 | 184041694 | AT | A | 0.566 | NM_004953 | EIF4G1 | frameshift_deleti  on | exon9 | c.1817delT | p.I606fs | . | . |
| CTC6 | 3 | 184044688 | AG | A | 0.667 | NM_004953 | EIF4G1 | frameshift_deleti  on | exon16 | c.2761delG | p.A921fs | . | . |
| CTC6 | 3 | 184070540 | AG | A | 0.33 | NM_001171088 | CLCN2 | frameshift_deleti  on | exon18 | c.2069delC | p.P690fs | . | . |
| CTC6 | 3 | 184100865 | TG | T | 0.42 | NM_003741 | CHRD | frameshift_deleti  on | exon10 | c.1128delG | p.L376fs | . | . |
| CTC6 | 3 | 186370358 | GC | G | 0.393 | NM_014375 | FETUB | frameshift_deleti  on | exon7 | c.1088delC | p.A363fs | . | . |
| CTC6 | 3 | 186953706 | GC | G | 0.877 | NM_139125 | MASP1 | frameshift_deleti  on | exon11 | c.1952delG | p.G651fs | . | . |
| CTC6 | 3 | 190374160 | TG | T | 0.243 | NM_001167931 | IL1RAP | frameshift_deleti  on | exon12 | c.1829delG | p.C610fs | . | . |
| CTC6 | 3 | 193201754 | CT | C | 0.279 | NM_032279 | ATP13A4 | frameshift_deleti  on | exon8 | c.778delA | p.S260fs | . | . |
| CTC6 | 3 | 195518110 | G | GCTGT  CTCCT | 1 | NM_018406 | MUC4 | nonframeshift_in  sertion | exon2 | c.340_341insTT  ACGCAGGAGA | p.A114delinsVT  QETA | . | . |
| CTC6 | 3 | 195518112 | T | TGTCTC  CTGCG | 0.982 | NM_018406 | MUC4 | nonframeshift_in  sertion | exon2 | c.338_339insTG  TTACGCAGGA | p.T113delinsTV  TQET | . | . |
| CTC6 | 3 | 195594841 | C | CG | 0.6 | NM_005781 | TNK2 | frameshift_inserti  on | exon12 | c.2282dupC | p.P761fs | . | . |
| CTC6 | 3 | 196287914 | GA | G | 0.462 | NM_182627 | WDR53 | frameshift_deleti  on | exon3 | c.432delT | p.F144fs | . | . |
| CTC6 | 3 | 196529945 | CA | C | 1 | NM_002577 | PAK2 | frameshift_deleti  on | exon4 | c.347delA | p.Q116fs | . | . |
| CTC6 | 4 | 1725459 | CT | C | 1 | NM_006342 | TACC3 | frameshift_deleti  on | exon3 | c.168delT | p.T56fs | . | . |
| CTC6 | 4 | 2074702 | TG | T | 0.565 | NM_181808 | POLN | frameshift_deleti  on | exon23 | c.2509delC | p.Q837fs | . | . |
| CTC6 | 4 | 2831585 | TC | T | 0.218 | NM_001122681 | SH3BP2 | frameshift_deleti  on | exon8 | c.953delC | p.S318fs | . | . |
| CTC6 | 4 | 2955250 | CT | C | 0.8 | NM_003703 | NOP14 | frameshift_deleti  on | exon5 | c.734delA | p.K245fs | . | . |
| CTC6 | 4 | 3117874 | CT | C | 0.6 | NM_002111 | HTT | frameshift_deleti  on | exon8 | c.953delT | p.L318fs | . | . |
| CTC6 | 4 | 3517910 | CT | C | 0.909 | NM_002337 | LRPAP1 | frameshift_deleti  on | exon6 | c.766delA | p.R256fs | . | . |
| CTC6 | 4 | 4436503 | TA | T | 0.667 | NM_016930 | STX18 | frameshift_deleti  on | exon7 | c.695delT | p.I232fs | . | . |
| CTC6 | 4 | 6537000 | G | GA | 0.3 | NM_001206994 | PPP2R2C | frameshift_inserti  on | exon2 | c.46dupT | p.S16fs | . | . |
| CTC6 | 4 | 8472844 | TG | T | 0.902 | NM_152544 | TRMT44 | frameshift_deleti  on | exon10 | c.1962delG | p.L654fs | . | . |
| CTC6 | 4 | 15964661 | TC | T | 0.385 | NM_031950 | FGFBP2 | frameshift_deleti  on | exon1 | c.91delG | p.E31fs | . | . |
| CTC6 | 4 | 17528696 | CT | C | 0.7 | NM_001079827 | CLRN2 | frameshift_deleti  on | exon3 | c.691delT | p.L231fs | . | . |
| CTC6 | 4 | 17625285 | CA | C | 0.6 | NM_025205 | MED28 | frameshift_deleti  on | exon4 | c.402delA | p.T134fs | . | . |
| CTC6 | 4 | 17636707 | G | GA | 0.968 | NM_015688 | FAM184B | frameshift_inserti  on | exon16 | c.2813dupT | p.F938fs | . | . |
| CTC6 | 4 | 17885911 | AT | A | 0.75 | NM_001166139 | LCORL | frameshift_deleti  on | exon7 | c.1240delA | p.I414fs | . | . |

| CTC6 | 4 | 20482347 | AT | A | 0.207 | NM_001289135 | SLIT2 | frameshift_deleti  on | exon6 | c.477delT | p.D159fs | . | . |
| --- | --- | --- | --- | --- | --- | --- | --- | --- | --- | --- | --- | --- | --- |
| CTC6 | 4 | 25676149 | GA | G | 0.941 | NM_001177998 | SLC34A2 | frameshift_deleti  on | exon12 | c.1354delA | p.R452fs | . | . |
| CTC6 | 4 | 30725561 | C | CA | 0.15 | NM_001173523 | PCDH7 | frameshift_inserti  on | exon1 | c.2518dupA | p.V839fs | . | . |
| CTC6 | 4 | 36230655 | CG | C | 0.375 | NM_015230 | ARAP2 | frameshift_deleti  on | exon2 | c.453delC | p.R151fs | . | . |
| CTC6 | 4 | 37962553 | TG | T | 0.269 | NM_006607 | PTTG2 | stopgain | exon1 | c.499delG | p.V167X | . | . |
| CTC6 | 4 | 38798558 | TG | T | 0.357 | NM_003263 | TLR1 | frameshift_deleti on | exon4 | c.1894delC | p.Q632fs | . | . |
| CTC6 | 4 | 40809138 | AT | A | 0.692 | NM_024677 | NSUN7 | frameshift_deleti  on | exon11 | c.1462delT | p.F488fs | . | . |
| CTC6 | 4 | 47746476 | CA | C | 0.39 | NM_001278585 | CORIN | frameshift_deleti  on | exon4 | c.540delT | p.T180fs | . | . |
| CTC6 | 4 | 47880636 | CT | C | 0.22 | NM_001278623 | NFXL1 | frameshift_deleti  on | exon17 | c.1984delA | p.R662fs | . | . |
| CTC6 | 4 | 55561871 | AG | A | 0.455 | NM_000222 | KIT | frameshift_deleti  on | exon2 | c.262delG | p.E88fs | . | . |
| CTC6 | 4 | 56835235 | A | AT | 0.333 | NM_025009 | CEP135 | frameshift_inserti  on | exon9 | c.1052dupT | p.I351fs | . | . |
| CTC6 | 4 | 57272772 | TG | T | 0.603 | NM_002703 | PPAT | frameshift_deleti  on | exon3 | c.290delC | p.T97fs | . | . |
| CTC6 | 4 | 57829789 | GC | G | 0.25 | NM_032313 | NOA1 | frameshift_deleti  on | exon7 | c.1923delG | p.L641fs | . | . |
| CTC6 | 4 | 68708280 | GT | G | 0.836 | NM_004262 | TMPRSS11D | frameshift_deleti  on | exon4 | c.312delA | p.K104fs | . | . |
| CTC6 | 4 | 69184439 | GA | G | 0.348 | NM_133370 | YTHDC1 | frameshift_deleti  on | exon13 | c.1675delT | p.S559fs | . | . |
| CTC6 | 4 | 69334599 | GA | G | 0.607 | NM_014058 | TMPRSS11E | frameshift_deleti  on | exon4 | c.262delA | p.K88fs | . | . |
| CTC6 | 4 | 70361123 | CA | C | 0.957 | NM_021139 | UGT2B4 | frameshift_deleti  on | exon1 | c.456delT | p.A152fs | . | . |
| CTC6 | 4 | 70719973 | CA | C | 0.188 | NM_005420 | SULT1E1 | frameshift_deleti  on | exon4 | c.330delT | p.P110fs | . | . |
| CTC6 | 4 | 71099763 | AG | A | 0.233 | NM_152997 | FDCSP | frameshift_deleti  on | exon4 | c.118delG | p.G40fs | . | . |
| CTC6 | 4 | 74279288 | A | AT | 0.703 | NM_000477 | ALB | frameshift_inserti  on | exon8 | c.996dupT | p.D332fs | . | . |
| CTC6 | 4 | 76573905 | TG | T | 0.231 | NM_203504 | G3BP2 | frameshift_deleti  on | exon8 | c.746delC | p.P249fs | . | . |
| CTC6 | 4 | 79437098 | C | CT | 0.19 | NM_025074 | FRAS1 | frameshift_inserti  on | exon66 | c.10321dupT | p.T3440fs | . | . |
| CTC6 | 4 | 79440586 | CA | C | 0.484 | NM_025074 | FRAS1 | frameshift_deleti  on | exon67 | c.10492delA | p.T3498fs | . | . |
| CTC6 | 4 | 79792082 | CCAG | C | 0.312 | NM_017593 | BMP2K | nonframeshift_de  letion | exon11 | c.1378_1380del | p.460_460del | . | . |
| CTC6 | 4 | 82125786 | GGT | G | 0.368 | NM_001282485 | PRKG2 | frameshift_deleti  on | exon2 | c.414_415del | p.K138fs | . | . |
| CTC6 | 4 | 87968511 | AG | A | 0.6 | NM_005935 | AFF1 | frameshift_deleti  on | exon3 | c.804delG | p.Q268fs | . | . |
| CTC6 | 4 | 88536457 | T | TAGTA  GTGAC | 1 | NM_014208 | DSPP | nonframeshift_in  sertion | exon5 | c.2643_2644ins  AGTAGTGAC | p.D881delinsD  SSD | . | . |
| CTC6 | 4 | 88536868 | TAGTGA  CAGCAG CAATAG | T | 1 | NM_014208 | DSPP | nonframeshift_de letion | exon5 | c.3055_3072del | p.1019_1024de l | . | . |
| CTC6 | 4 | 88537072 | TAGCAG  TGAC | T | 1 | NM_014208 | DSPP | nonframeshift_de  letion | exon5 | c.3259_3267del | p.1087_1089de  l | . | . |
| CTC6 | 4 | 88973218 | CA | C | 0.643 | NM_000297 | PKD2 | frameshift_deleti  on | exon7 | c.1625delA | p.Q542fs | . | . |
| CTC6 | 4 | 89978098 | TAG | T | 0.257 | NM_014883 | FAM13A | frameshift_deleti  on | exon1 | c.16_17del | p.L6fs | . | . |
| CTC6 | 4 | 95204356 | TC | T | 0.402 | NM_001254949 | SMARCAD1 | stopgain | exon14 | c.1522delC | p.L508X | . | . |
| CTC6 | 4 | 108817090 | AT | A | 0.606 | NM_001136257 | SGMS2 | frameshift_deleti on | exon2 | c.382delT | p.F128fs | . | . |
| CTC6 | 4 | 108831566 | TA | T | 0.4 | NM_001136257 | SGMS2 | frameshift_deleti  on | exon6 | c.956delA | p.Y319fs | . | . |
| CTC6 | 4 | 109681440 | CA | C | 0.622 | NM_001146590 | ETNPPL | frameshift_deleti  on | exon2 | c.78delT | p.F26fs | . | . |
| CTC6 | 4 | 110670461 | TG | T | 0.393 | NM_000204 | CFI | frameshift_deleti  on | exon10 | c.1060delC | p.Q354fs | . | . |
| CTC6 | 4 | 110932610 | GA | G | 0.895 | NM_001178130 | EGF | nonframeshift_de  letion | exon23 | c.3501delA | p.X1167X | . | . |
| CTC6 | 4 | 115544593 | ACC | A | 0.154 | NM_003360 | UGT8 | frameshift_deleti  on | exon1 | c.558_559del | p.D186fs | . | . |
| CTC6 | 4 | 118005495 | ACT | A | 0.132 | NM_152402 | TRAM1L1 | frameshift_deleti  on | exon1 | c.1053_1054del | p.G351fs | . | . |
| CTC6 | 4 | 122853601 | TC | T | 0.434 | NM_003305 | TRPC3 | frameshift_deleti  on | exon1 | c.592delG | p.D198fs | . | . |
| CTC6 | 4 | 123168423 | CA | C | 0.467 | NM_015312 | KIAA1109 | frameshift_deleti  on | exon33 | c.5424delA | p.S1808fs | . | . |
| CTC6 | 4 | 123207788 | TC | T | 0.429 | NM_015312 | KIAA1109 | frameshift_deleti  on | exon51 | c.9131delC | p.S3044fs | . | . |
| CTC6 | 4 | 123664692 | TG | T | 0.309 | NM_152618 | BBS12 | frameshift_deleti  on | exon2 | c.1646delG | p.C549fs | . | . |
| CTC6 | 4 | 126371415 | GA | G | 0.257 | NM_024582 | FAT4 | frameshift_deleti  on | exon9 | c.9245delA | p.E3082fs | . | . |
| CTC6 | 4 | 128719767 | CT | C | 0.286 | NM_014278 | HSPA4L | frameshift_deleti  on | exon4 | c.333delT | p.P111fs | . | . |
| CTC6 | 4 | 128817974 | T | TTG | 0.2 | NM_001190799 | PLK4 | frameshift_inserti  on | exon14 | c.2626_2627ins  TG | p.W876fs | . | . |
| CTC6 | 4 | 128841882 | AG | A | 0.75 | NM_152778 | MFSD8 | frameshift_deleti  on | exon13 | c.1459delC | p.L487fs | . | . |
| CTC6 | 4 | 140640585 | AC | A | 0.531 | NM_018717 | MAML3 | frameshift_deleti  on | exon6 | c.3296delG | p.G1099fs | . | . |
| CTC6 | 4 | 146478988 | TG | T | 1 | NM_001003688 | SMAD1 | frameshift_deleti  on | exon7 | c.1301delG | p.C434fs | . | . |
| CTC6 | 4 | 148463645 | TG | T | 0.389 | NM_001166055 | EDNRA | frameshift_deleti  on | exon6 | c.833delG | p.C278fs | . | . |
| CTC6 | 4 | 152203384 | C | CA | 0.368 | NM_183375 | PRSS48 | frameshift_inserti  on | exon3 | c.301dupA | p.S100fs | . | . |
| CTC6 | 4 | 152204320 | G | GC | 0.4 | NM_183375 | PRSS48 | frameshift_inserti  on | exon4 | c.534dupC | p.R178fs | . | . |
| CTC6 | 4 | 153896841 | GA | G | 0.83 | NM_033393 | FHDC1 | frameshift_deleti  on | exon11 | c.2399delA | p.E800fs | . | . |
| CTC6 | 4 | 154557549 | AT | A | 0.185 | NM_001131007 | KIAA0922 | frameshift_deleti  on | exon35 | c.4655delT | p.I1552fs | . | . |
| CTC6 | 4 | 154625245 | CA | C | 0.667 | NM_003264 | TLR2 | frameshift_deleti  on | exon3 | c.1187delA | p.Q396fs | . | . |
| CTC6 | 4 | 155244401 | TTTTG | T | 1 | NM_001142552 | DCHS2 | frameshift_deleti  on | exon8 | c.4095_4098del | p.N1365fs | . | . |
| CTC6 | 4 | 155244409 | GTTTGT | G | 0.846 | NM_001142552 | DCHS2 | frameshift_deleti  on | exon8 | c.4086_4090del | p.K1362fs | . | . |

| CTC6 | 4 | 155491612 | GT | G | 0.278 | NM_001184741 | FGB | frameshift_deleti  on | exon8 | c.1110delT | p.G370fs | . | . |
| --- | --- | --- | --- | --- | --- | --- | --- | --- | --- | --- | --- | --- | --- |
| CTC6 | 4 | 159133893 | AT | A | 0.263 | NM_018342 | TMEM144 | frameshift_deleti  on | exon3 | c.75delT | p.N25fs | . | . |
| CTC6 | 4 | 159753090 | CA | C | 0.212 | NM_020840 | FNIP2 | frameshift_deleti  on | exon4 | c.460delA | p.T154fs | . | . |
| CTC6 | 4 | 159780776 | AG | A | 0.211 | NM_020840 | FNIP2 | frameshift_deleti  on | exon10 | c.1107delG | p.Q369fs | . | . |
| CTC6 | 4 | 165878539 | GA | G | 0.8 | NM_153027 | FAM218A | frameshift_deleti  on | exon1 | c.366delA | p.G122fs | . | . |
| CTC6 | 4 | 174219315 | GAA | G | 1 | NM_017423 | GALNT7 | frameshift_deleti  on | exon6 | c.1016_1017del | p.E339fs | . | . |
| CTC6 | 4 | 177056382 | TC | T | 0.571 | NM_181265 | WDR17 | frameshift_deleti  on | exon8 | c.1223delC | p.S408fs | . | . |
| CTC6 | 4 | 177605081 | CTCA | C | 1 | NM_005429 | VEGFC | nonframeshift_de  letion | exon7 | c.1256_1258del | p.419_420del | . | . |
| CTC6 | 4 | 184932475 | AG | A | 0.188 | NM_020225 | STOX2 | frameshift_deleti  on | exon3 | c.2485delG | p.E829fs | . | . |
| CTC6 | 4 | 187630286 | AT | A | 0.375 | NM_005245 | FAT1 | frameshift_deleti  on | exon2 | c.695delA | p.Y232fs | . | . |
| CTC6 | 5 | 271857 | CCCGGC  CCTGGG | C | 0.917 | NM_001267556 | PDCD6 | nonframeshift_de  letion | exon1 | c.23_34del | p.8_12del | . | . |
| CTC6 | 5 | 825346 | AG | A | 0.6 | NM_024786 | ZDHHC11 | frameshift_deleti  on | exon8 | c.955delC | p.L319fs | . | . |
| CTC6 | 5 | 1074697 | T | TG | 0.659 | NM_006598 | SLC12A7 | frameshift_inserti  on | exon16 | c.2056dupC | p.H686fs | . | . |
| CTC6 | 5 | 1432781 | AC | A | 0.253 | NM_001044 | SLC6A3 | frameshift_deleti  on | exon4 | c.450delG | p.L150fs | . | . |
| CTC6 | 5 | 5461198 | AT | A | 0.319 | NM_015325 | KIAA0947 | frameshift_deleti  on | exon13 | c.1752delT | p.H584fs | . | . |
| CTC6 | 5 | 10261785 | TA | T | 0.36 | NM_012073 | CCT5 | frameshift_deleti  on | exon8 | c.1108delA | p.K370fs | . | . |
| CTC6 | 5 | 13794069 | TG | T | 0.373 | NM_001369 | DNAH5 | frameshift_deleti  on | exon48 | c.7985delC | p.P2662fs | . | . |
| CTC6 | 5 | 13850841 | GC | G | 1 | NM_001369 | DNAH5 | frameshift_deleti  on | exon31 | c.5033delG | p.C1678fs | . | . |
| CTC6 | 5 | 14287035 | TG | T | 1 | NM_007118 | TRIO | frameshift_deleti  on | exon4 | c.404delG | p.W135fs | . | . |
| CTC6 | 5 | 14406084 | GAC | G | 0.361 | NM_007118 | TRIO | frameshift_deleti  on | exon32 | c.4845_4846del | p.R1615fs | . | . |
| CTC6 | 5 | 14693059 | A | AG | 0.286 | NM_138348 | FAM105B | frameshift_inserti  on | exon7 | c.961_962insG | p.T321fs | . | . |
| CTC6 | 5 | 16475274 | AT | A | 1 | NM_019000 | FAM134B | frameshift_deleti  on | exon7 | c.646delA | p.M216fs | . | . |
| CTC6 | 5 | 31538734 | GT | G | 0.273 | NM_018356 | C5orf22 | frameshift_deleti  on | exon4 | c.746delT | p.V249fs | . | . |
| CTC6 | 5 | 33937948 | T | TA | 0.217 | NM_016568 | RXFP3 | frameshift_inserti  on | exon1 | c.1104dupA | p.V368fs | . | . |
| CTC6 | 5 | 33963969 | CA | C | 0.9 | NM_001012509 | SLC45A2 | frameshift_deleti  on | exon3 | c.714delT | p.S238fs | . | . |
| CTC6 | 5 | 35039504 | AG | A | 0.5 | NM_031900 | AGXT2 | frameshift_deleti  on | exon3 | c.286delC | p.L96fs | . | . |
| CTC6 | 5 | 35670302 | AG | A | 0.171 | NM_024867 | SPEF2 | frameshift_deleti  on | exon10 | c.1498delG | p.D500fs | . | . |
| CTC6 | 5 | 36195284 | TA | T | 0.667 | NM_001085411 | NADK2 | frameshift_deleti  on | exon12 | c.1290delT | p.N430fs | . | . |
| CTC6 | 5 | 36677185 | CG | C | 0.471 | NM_001289939 | SLC1A3 | frameshift_deleti  on | exon5 | c.622delG | p.G208fs | . | . |
| CTC6 | 5 | 37169473 | GT | G | 0.75 | NM_023073 | C5orf42 | frameshift_deleti  on | exon34 | c.6652delA | p.T2218fs | . | . |
| CTC6 | 5 | 37226474 | G | GT | 1 | NM_023073 | C5orf42 | frameshift_inserti  on | exon12 | c.2222dupA | p.N741fs | . | . |
| CTC6 | 5 | 37333626 | G | GA | 0.95 | NM_001278312 | NUP155 | frameshift_inserti  on | exon13 | c.1456dupT | p.S486fs | . | . |
| CTC6 | 5 | 38485989 | AC | A | 0.256 | NM_001127671 | LIFR | frameshift_deleti  on | exon17 | c.2428delG | p.V810fs | . | . |
| CTC6 | 5 | 38496485 | A | AT | 0.208 | NM_001127671 | LIFR | frameshift_inserti  on | exon13 | c.1883dupA | p.N628fs | . | . |
| CTC6 | 5 | 41032901 | AG | A | 0.276 | NM_173489 | MROH2B | frameshift_deleti  on | exon24 | c.2383delC | p.L795fs | . | . |
| CTC6 | 5 | 43506243 | AT | A | 0.29 | NM_198566 | C5orf34 | frameshift_deleti  on | exon4 | c.538delA | p.I180fs | . | . |
| CTC6 | 5 | 50689426 | CA | C | 0.171 | NM_002202 | ISL1 | frameshift_deleti  on | exon6 | c.1033delA | p.S345fs | . | . |
| CTC6 | 5 | 52097259 | GAC | G | 0.705 | NM_015946 | PELO | frameshift_deleti  on | exon3 | c.744_745del | p.G248fs | . | . |
| CTC6 | 5 | 56168470 | GC | G | 0.375 | NM_005921 | MAP3K1 | frameshift_deleti  on | exon8 | c.1427delC | p.A476fs | . | . |
| CTC6 | 5 | 61651035 | AC | A | 1 | NM_001098511 | KIF2A | frameshift_deleti  on | exon7 | c.609delC | p.D203fs | . | . |
| CTC6 | 5 | 66440497 | GT | G | 0.312 | NM_015183 | MAST4 | frameshift_deleti  on | exon21 | c.2165delT | p.V722fs | . | . |
| CTC6 | 5 | 66449299 | GA | G | 0.741 | NM_015183 | MAST4 | frameshift_deleti  on | exon25 | c.2973delA | p.G991fs | . | . |
| CTC6 | 5 | 70308295 | TC | T | 0.6 | NM_004536 | NAIP | frameshift_deleti  on | exon4 | c.447delG | p.L149fs | . | . |
| CTC6 | 5 | 73153575 | AG | A | 0.538 | NM_001244364 | ARHGEF28 | frameshift_deleti  on | exon7 | c.947delG | p.R316fs | . | . |
| CTC6 | 5 | 73931276 | AG | A | 0.4 | NM_001256576 | ENC1 | frameshift_deleti  on | exon2 | c.815delC | p.T272fs | . | . |
| CTC6 | 5 | 74021846 | TACTCA  A | T | 0.815 | NM_170691 | GFM2 | nonframeshift_de  letion | exon17 | c.1685_1690del | p.562_564del | . | . |
| CTC6 | 5 | 74060882 | A | AT | 1 | NM_001281302 | GFM2 | frameshift_inserti  on | exon2 | c.49dupA | p.I17fs | . | . |
| CTC6 | 5 | 74443308 | CT | C | 0.515 | NM_001164443 | ANKRD31 | frameshift_deleti  on | exon14 | c.1927delA | p.S643fs | . | . |
| CTC6 | 5 | 74491715 | TTCA | T | 1 | NM_001164443 | ANKRD31 | nonframeshift_de  letion | exon7 | c.755_757del | p.252_253del | . | . |
| CTC6 | 5 | 75989191 | TG | T | 0.625 | NM_001285461 | IQGAP2 | frameshift_deleti  on | exon19 | c.2406delG | p.L802fs | . | . |
| CTC6 | 5 | 76703216 | GC | G | 0.474 | NM_001029851 | PDE8B | frameshift_deleti  on | exon10 | c.1009delC | p.Q337fs | . | . |
| CTC6 | 5 | 78076399 | AG | A | 1 | NM_000046 | ARSB | frameshift_deleti  on | exon8 | c.1422delC | p.L474fs | . | . |
| CTC6 | 5 | 79029978 | CA | C | 0.333 | NM_153610 | CMYA5 | frameshift_deleti  on | exon2 | c.5391delA | p.P1797fs | . | . |
| CTC6 | 5 | 79030319 | GC | G | 0.652 | NM_153610 | CMYA5 | frameshift_deleti  on | exon2 | c.5732delC | p.A1911fs | . | . |
| CTC6 | 5 | 82841369 | CA | C | 0.333 | NM_001126336 | VCAN | frameshift_deleti  on | exon7 | c.1057delA | p.K353fs | . | . |
| CTC6 | 5 | 94727156 | GT | G | 0.435 | NM_152548 | FAM81B | frameshift_deleti  on | exon1 | c.64delT | p.F22fs | . | . |
| CTC6 | 5 | 96139268 | TG | T | 0.806 | NM_001040458 | ERAP1 | frameshift_deleti  on | exon2 | c.361delC | p.Q121fs | . | . |
| CTC6 | 5 | 109963560 | G | GT | 0.889 | NM_001039763 | TMEM232 | frameshift_inserti  on | exon6 | c.522dupA | p.R175fs | . | . |

| CTC6 | 5 | 110428191 | CT | C | 0.64 | NM_139281 | WDR36 | frameshift_deleti  on | exon1 | c.206delT | p.L69fs | . | . |
| --- | --- | --- | --- | --- | --- | --- | --- | --- | --- | --- | --- | --- | --- |
| CTC6 | 5 | 111500815 | T | TATAA  A | 0.571 | NM_022140 | EPB41L4A | frameshift_inserti  on | exon23 | c.1932_1933ins  TTTAT | p.R645fs | . | . |
| CTC6 | 5 | 114607258 | CT | C | 1 | NM_001040440 | CCDC112 | frameshift_deleti  on | exon7 | c.983delA | p.K328fs | . | . |
| CTC6 | 5 | 114878678 | CT | C | 0.684 | NM_020177 | FEM1C | frameshift_deleti  on | exon2 | c.512delA | p.K171fs | . | . |
| CTC6 | 5 | 118469421 | CA | C | 0.417 | NM_005509 | DMXL1 | frameshift_deleti  on | exon12 | c.1803delA | p.S601fs | . | . |
| CTC6 | 5 | 118525454 | CA | C | 0.344 | NM_005509 | DMXL1 | frameshift_deleti  on | exon29 | c.7188delA | p.S2396fs | . | . |
| CTC6 | 5 | 118573025 | AG | A | 0.333 | NM_005509 | DMXL1 | frameshift_deleti  on | exon39 | c.8413delG | p.V2805fs | . | . |
| CTC6 | 5 | 127522321 | TA | T | 0.273 | NM_001256461 | SLC12A2 | nonframeshift_de  letion | exon26 | c.3590delA | p.X1197X | . | . |
| CTC6 | 5 | 127626509 | GT | G | 0.556 | NM_001999 | FBN2 | frameshift_deleti  on | exon50 | c.6359delA | p.N2120fs | . | . |
| CTC6 | 5 | 127648372 | TC | T | 0.262 | NM_001999 | FBN2 | frameshift_deleti  on | exon37 | c.4832delG | p.G1611fs | . | . |
| CTC6 | 5 | 128302136 | GA | G | 0.333 | NM_001017372 | SLC27A6 | frameshift_deleti  on | exon1 | c.307delA | p.K103fs | . | . |
| CTC6 | 5 | 128994407 | A | AT | 0.81 | NM_133638 | ADAMTS19 | frameshift_inserti  on | exon15 | c.2385dupT | p.D795fs | . | . |
| CTC6 | 5 | 131530648 | GC | G | 0.333 | NM_001017973 | P4HA2 | frameshift_deleti  on | exon14 | c.1501delG | p.A501fs | . | . |
| CTC6 | 5 | 132096919 | A | AG | 0.327 | NM_001098811 | 8-Sep | frameshift_inserti  on | exon8 | c.1000dupC | p.L334fs | . | . |
| CTC6 | 5 | 133895640 | C | CA | 0.87 | NM_015288 | JADE2 | frameshift_inserti  on | exon5 | c.432_433insA | p.A144fs | . | . |
| CTC6 | 5 | 134053789 | CCT | C | 0.778 | NM_021982 | SEC24A | frameshift_deleti  on | exon20 | c.2897_2898del | p.P966fs | . | . |
| CTC6 | 5 | 134076768 | CA | C | 0.394 | NM_001745 | CAMLG | frameshift_deleti  on | exon2 | c.189delA | p.T63fs | . | . |
| CTC6 | 5 | 137625229 | TG | T | 0.227 | NM_022809 | CDC25C | stopgain | exon7 | c.668delC | p.S223X | . | . |
| CTC6 | 5 | 138700267 | A | AT | 0.353 | NM_001033112 | PAIP2 | frameshift_inserti on | exon3 | c.154dupT | p.L51fs | . | . |
| CTC6 | 5 | 138728045 | TC | T | 1 | NM_001161546 | PROB1 | frameshift_deleti  on | exon1 | c.2725delG | p.E909fs | . | . |
| CTC6 | 5 | 140034299 | TA | T | 0.359 | NM_006083 | IK | frameshift_deleti  on | exon8 | c.625delA | p.K209fs | . | . |
| CTC6 | 5 | 140175136 | TG | T | 0.351 | NM_018905 | PCDHA2 | frameshift_deleti  on | exon1 | c.588delG | p.L196fs | . | . |
| CTC6 | 5 | 140175594 | GA | G | 0.235 | NM_018905 | PCDHA2 | frameshift_deleti  on | exon1 | c.1046delA | p.E349fs | . | . |
| CTC6 | 5 | 140590091 | TC | T | 0.5 | NM_018932 | PCDHB12 | frameshift_deleti  on | exon1 | c.1613delC | p.S538fs | . | . |
| CTC6 | 5 | 140720843 | AT | A | 1 | NM_018915 | PCDHGA2 | frameshift_deleti  on | exon1 | c.2306delT | p.I769fs | . | . |
| CTC6 | 5 | 140744032 | CG | C | 0.333 | NM_018918 | PCDHGA5 | frameshift_deleti  on | exon1 | c.136delG | p.G46fs | . | . |
| CTC6 | 5 | 140755048 | CA | C | 0.95 | NM_018919 | PCDHGA6 | frameshift_deleti  on | exon1 | c.1399delA | p.R467fs | . | . |
| CTC6 | 5 | 140769072 | AGC | A | 0.143 | NM_003736 | PCDHGB4 | frameshift_deleti  on | exon1 | c.1622_1623del | p.S541fs | . | . |
| CTC6 | 5 | 140783088 | TA | T | 0.417 | NM_018921 | PCDHGA9 | frameshift_deleti  on | exon1 | c.570delA | p.I190fs | . | . |
| CTC6 | 5 | 140858190 | GT | G | 1 | NM_032402 | PCDHGC3 | frameshift_deleti  on | exon1 | c.2508delT | p.C836fs | . | . |
| CTC6 | 5 | 140864910 | AC | A | 0.4 | NM_018928 | PCDHGC4 | frameshift_deleti  on | exon1 | c.171delC | p.D57fs | . | . |
| CTC6 | 5 | 140871138 | CT | C | 0.667 | NM_018929 | PCDHGC5 | frameshift_deleti  on | exon1 | c.2332delT | p.F778fs | . | . |
| CTC6 | 5 | 140953563 | TGGA | T | 0.667 | NM_001079812 | DIAPH1 | nonframeshift_de  letion | exon15 | c.1824_1826del | p.608_609del | . | . |
| CTC6 | 5 | 142779929 | GT | G | 0.682 | NM_000176 | NR3C1 | frameshift_deleti  on | exon2 | c.475delA | p.T159fs | . | . |
| CTC6 | 5 | 147492442 | CA | C | 0.278 | NM_001127698 | SPINK5 | frameshift_deleti  on | exon20 | c.1833delA | p.A611fs | . | . |
| CTC6 | 5 | 148407702 | GC | G | 1 | NM_024577 | SH3TC2 | frameshift_deleti  on | exon11 | c.1592delG | p.C531fs | . | . |
| CTC6 | 5 | 148747636 | AC | A | 1 | NM_024028 | PCYOX1L | frameshift_deleti  on | exon6 | c.905delC | p.T302fs | . | . |
| CTC6 | 5 | 149216456 | GGGA | G | 0.8 | NM_001172698 | PPARGC1B | nonframeshift_de  letion | exon7 | c.2322_2324del | p.774_775del | . | . |
| CTC6 | 5 | 149374879 | CT | C | 1 | NM_001243253 | TIGD6 | frameshift_deleti  on | exon2 | c.1032delA | p.Q344fs | . | . |
| CTC6 | 5 | 149386192 | GA | G | 0.9 | NM_014983 | HMGXB3 | frameshift_deleti  on | exon3 | c.295delA | p.K99fs | . | . |
| CTC6 | 5 | 149398190 | ATG | A | 0.395 | NM_014983 | HMGXB3 | frameshift_deleti  on | exon6 | c.972_973del | p.Y324fs | . | . |
| CTC6 | 5 | 149439372 | G | GA | 0.4 | NM_001288705 | CSF1R | frameshift_inserti  on | exon14 | c.2022dupT | p.L675fs | . | . |
| CTC6 | 5 | 150436409 | GTGA | G | 0.103 | NM_001252386 | TNIP1 | nonframeshift_de  letion | exon5 | c.383_385del | p.128_129del | . | . |
| CTC6 | 5 | 150682837 | TG | T | 0.978 | NM_001145017 | SLC36A3 | frameshift_deleti  on | exon1 | c.78delC | p.S26fs | . | . |
| CTC6 | 5 | 150901432 | CA | C | 0.235 | NM_001447 | FAT2 | frameshift_deleti  on | exon18 | c.10721delT | p.L3574fs | . | . |
| CTC6 | 5 | 150924985 | TG | T | 0.16 | NM_001447 | FAT2 | stopgain | exon9 | c.5702delC | p.S1901X | . | . |
| CTC6 | 5 | 151049224 | CA | C | 0.333 | NM_003118 | SPARC | frameshift_deleti on | exon6 | c.451delT | p.Y151fs | . | . |
| CTC6 | 5 | 156809620 | TG | T | 0.538 | NM_001037332 | CYFIP2 | frameshift_deleti  on | exon27 | c.3063delG | p.L1021fs | . | . |
| CTC6 | 5 | 156964988 | GC | G | 1 | NM_033274 | ADAM19 | frameshift_deleti  on | exon4 | c.262delG | p.A88fs | . | . |
| CTC6 | 5 | 160049429 | AC | A | 0.3 | NM_025153 | ATP10B | frameshift_deleti  on | exon14 | c.1783delG | p.V595fs | . | . |
| CTC6 | 5 | 167642084 | CG | C | 0.491 | NM_001122679 | TENM2 | frameshift_deleti  on | exon21 | c.3859delG | p.V1287fs | . | . |
| CTC6 | 5 | 167674497 | GT | G | 0.558 | NM_001122679 | TENM2 | frameshift_deleti  on | exon27 | c.6527delT | p.V2176fs | . | . |
| CTC6 | 5 | 167675356 | AT | A | 0.772 | NM_001122679 | TENM2 | frameshift_deleti  on | exon27 | c.7386delT | p.D2462fs | . | . |
| CTC6 | 5 | 167881026 | AGGT | A | 1 | NM_001161661 | WWC1 | nonframeshift_de  letion | exon18 | c.2580_2582del | p.860_861del | . | . |
| CTC6 | 5 | 167881029 | TGGA | T | 1 | NM_001161661 | WWC1 | nonframeshift_de  letion | exon18 | c.2583_2585del | p.861_862del | . | . |
| CTC6 | 5 | 167891942 | TG | T | 0.818 | NM_001161661 | WWC1 | frameshift_deleti  on | exon21 | c.3144delG | p.L1048fs | . | . |
| CTC6 | 5 | 170725840 | GC | G | 0.535 | NM_022897 | RANBP17 | stopgain | exon28 | c.3246delC | p.C1082X | . | . |

| CTC6 | 5 | 171766806 | TGG | T | 0.565 | NM_001017995 | SH3PXD2B | frameshift_deleti  on | exon13 | c.1301_1302del | p.P434fs | . | . |
| --- | --- | --- | --- | --- | --- | --- | --- | --- | --- | --- | --- | --- | --- |
| CTC6 | 5 | 172351013 | CG | C | 0.208 | NM_001031711 | ERGIC1 | frameshift_deleti  on | exon6 | c.382delG | p.G128fs | . | . |
| CTC6 | 5 | 176637107 | GT | G | 0.556 | NM_022455 | NSD1 | frameshift_deleti  on | exon5 | c.1708delT | p.F570fs | . | . |
| CTC6 | 5 | 176638795 | GA | G | 1 | NM_022455 | NSD1 | frameshift_deleti  on | exon5 | c.3396delA | p.G1132fs | . | . |
| CTC6 | 5 | 176798510 | AT | A | 1 | NM_006480 | RGS14 | frameshift_deleti  on | exon14 | c.1419delT | p.H473fs | . | . |
| CTC6 | 5 | 176930171 | AGAG | A | 0.966 | NM_001144876 | DOK3 | nonframeshift_de  letion | exon5 | c.559_561del | p.187_187del | . | . |
| CTC6 | 5 | 178040485 | TG | T | 0.625 | NM_020666 | CLK4 | frameshift_deleti  on | exon7 | c.814delC | p.Q272fs | . | . |
| CTC6 | 5 | 179105680 | CG | C | 0.576 | NM_001164444 | CBY3 | frameshift_deleti  on | exon2 | c.632delC | p.T211fs | . | . |
| CTC6 | 5 | 179739470 | GT | G | 0.765 | NM_005110 | GFPT2 | frameshift_deleti  on | exon15 | c.1505delA | p.N502fs | . | . |
| CTC6 | 5 | 180432756 | AT | A | 0.304 | NM_197975 | BTNL3 | frameshift_deleti  on | exon8 | c.1286delT | p.I429fs | . | . |
| CTC6 | 5 | 180687428 | CTCT | C | 0.196 | NM_032765 | TRIM52 | nonframeshift_de  letion | exon1 | c.384_386del | p.128_129del | . | . |
| CTC6 | 6 | 4059001 | CA | C | 0.25 | NM_003913 | PRPF4B | frameshift_deleti  on | exon14 | c.2774delA | p.Q925fs | . | . |
| CTC6 | 6 | 4069563 | A | AT | 0.28 | NM_173563 | FAM217A | frameshift_inserti  on | exon7 | c.893dupA | p.H298fs | . | . |
| CTC6 | 6 | 6174979 | AT | A | 1 | NM_000129 | F13A1 | frameshift_deleti  on | exon12 | c.1580delA | p.N527fs | . | . |
| CTC6 | 6 | 10400698 | GT | G | 0.366 | NM_001032280 | TFAP2A | frameshift_deleti  on | exon6 | c.989delA | p.N330fs | . | . |
| CTC6 | 6 | 10875096 | TC | T | 0.175 | NM_004752 | GCM2 | frameshift_deleti  on | exon5 | c.652delG | p.D218fs | . | . |
| CTC6 | 6 | 16145265 | TG | T | 0.35 | NM_013262 | MYLIP | frameshift_deleti  on | exon6 | c.966delG | p.V322fs | . | . |
| CTC6 | 6 | 16327864 | G | GTGCT  GC | 0.87 | NM_001128164 | ATXN1 | nonframeshift_in  sertion | exon7 | c.677_678insG  CAGCA | p.H226delinsQ  QH | . | . |
| CTC6 | 6 | 16327864 | G | GTGC | 0.87 | NM_001128164 | ATXN1 | nonframeshift_in  sertion | exon7 | c.677_678insG  CA | p.H226delinsQ  H | . | . |
| CTC6 | 6 | 16327885 | C | CTGCT  GT | 0.5 | NM_001128164 | ATXN1 | nonframeshift_in  sertion | exon7 | c.656_657insAC  AGCA | p.Q219delinsQ  QQ | . | . |
| CTC6 | 6 | 16327915 | ATGC | A | 0.125 | NM_001128164 | ATXN1 | nonframeshift_de  letion | exon7 | c.624_626del | p.208_209del | . | . |
| CTC6 | 6 | 22287701 | CT | C | 0.929 | NM_000948 | PRL | frameshift_deleti  on | exon5 | c.613delA | p.R205fs | . | . |
| CTC6 | 6 | 24357727 | GC | G | 0.909 | NM_016356 | DCDC2 | frameshift_deleti  on | exon1 | c.251delG | p.G84fs | . | . |
| CTC6 | 6 | 24570225 | TA | T | 0.652 | NM_001252328 | KIAA0319 | frameshift_deleti  on | exon8 | c.129delT | p.D43fs | . | . |
| CTC6 | 6 | 24576659 | GT | G | 0.19 | NM_001168376 | KIAA0319 | frameshift_deleti  on | exon9 | c.1535delA | p.H512fs | . | . |
| CTC6 | 6 | 24658884 | AT | A | 0.255 | NM_016614 | TDP2 | frameshift_deleti  on | exon3 | c.329delA | p.N110fs | . | . |
| CTC6 | 6 | 26465517 | CA | C | 0.808 | NM_001197233 | BTN2A1 | frameshift_deleti  on | exon4 | c.635delA | p.Q212fs | . | . |
| CTC6 | 6 | 26637643 | AC | A | 0.308 | NM_001242798 | ZNF322 | frameshift_deleti  on | exon3 | c.1138delG | p.V380fs | . | . |
| CTC6 | 6 | 28227152 | GCC | G | 0.292 | NM_001007531 | NKAPL | frameshift_deleti  on | exon1 | c.4_5del | p.P2fs | . | . |
| CTC6 | 6 | 29079724 | AT | A | 0.276 | NM_001005216 | OR2J3 | frameshift_deleti  on | exon1 | c.58delT | p.F20fs | . | . |
| CTC6 | 6 | 30899497 | TC | T | 0.462 | NM_205854 | SFTA2 | frameshift_deleti  on | exon2 | c.136delG | p.E46fs | . | . |
| CTC6 | 6 | 31239826 | GC | G | 1 | NM_001243042 | HLA-C | frameshift_deleti  on | exon1 | c.22delG | p.A8fs | . | . |
| CTC6 | 6 | 31239827 | CT | C | 1 | NM_001243042 | HLA-C | frameshift_deleti  on | exon1 | c.21delA | p.R7fs | . | . |
| CTC6 | 6 | 31599869 | CA | C | 0.705 | NM_004638 | PRRC2A | frameshift_deleti  on | exon16 | c.3420delA | p.P1140fs | . | . |
| CTC6 | 6 | 31670963 | CG | C | 0.333 | NM_021160 | ABHD16A | frameshift_deleti  on | exon1 | c.95delC | p.T32fs | . | . |
| CTC6 | 6 | 31696859 | TC | T | 0.383 | NM_013974 | DDAH2 | frameshift_deleti  on | exon2 | c.79delG | p.E27fs | . | . |
| CTC6 | 6 | 31747402 | AG | A | 0.5 | NM_006295 | VARS | frameshift_deleti  on | exon27 | c.3270delC | p.P1090fs | . | . |
| CTC6 | 6 | 31919805 | T | TA | 0.588 | NM_001710 | CFB | frameshift_inserti  on | exon18 | c.2294dupA | p.X765delinsX | . | . |
| CTC6 | 6 | 32151396 | GA | G | 0.538 | NM_001136 | AGER | frameshift_deleti  on | exon3 | c.290delT | p.F97fs | . | . |
| CTC6 | 6 | 32156049 | CA | C | 0.8 | NM_002586 | PBX2 | frameshift_deleti  on | exon3 | c.527delT | p.L176fs | . | . |
| CTC6 | 6 | 32191664 | CAGCA | C | 1 | NM_004557 | NOTCH4 | frameshift_deleti  on | exon1 | c.38_41del | p.L13fs | . | . |
| CTC6 | 6 | 32191664 | CA | C | 1 | NM_004557 | NOTCH4 | frameshift_deleti  on | exon1 | c.41delT | p.L14fs | . | . |
| CTC6 | 6 | 32798456 | A | AC | 0.5 | NM_018833 | TAP2 | frameshift_inserti  on | exon8 | c.1399dupG | p.V467fs | . | . |
| CTC6 | 6 | 33287888 | CTCT | C | 0.419 | NM_001254717 | DAXX | nonframeshift_de  letion | exon4 | c.1137_1139del | p.379_380del | . | . |
| CTC6 | 6 | 33287897 | TTCC | T | 0.534 | NM_001254717 | DAXX | nonframeshift_de  letion | exon4 | c.1128_1130del | p.376_377del | . | . |
| CTC6 | 6 | 33690536 | GT | G | 0.6 | NM_054111 | IP6K3 | frameshift_deleti  on | exon6 | c.1193delA | p.N398fs | . | . |
| CTC6 | 6 | 33690547 | CA | C | 0.636 | NM_054111 | IP6K3 | frameshift_deleti  on | exon6 | c.1182delT | p.F394fs | . | . |
| CTC6 | 6 | 34614534 | CG | C | 0.231 | NM_024294 | C6orf106 | frameshift_deleti  on | exon3 | c.354delC | p.V118fs | . | . |
| CTC6 | 6 | 35096918 | CA | C | 0.607 | NM_001261818 | TCP11 | frameshift_deleti  on | exon3 | c.178delT | p.W60fs | . | . |
| CTC6 | 6 | 35210496 | CA | C | 0.444 | NM_152753 | SCUBE3 | frameshift_deleti  on | exon14 | c.1633delA | p.K545fs | . | . |
| CTC6 | 6 | 36466174 | G | GA | 0.484 | NM_007271 | STK38 | frameshift_inserti  on | exon11 | c.1041dupT | p.P348fs | . | . |
| CTC6 | 6 | 36686279 | CGG | C | 0.29 | NM_001257357 | RAB44 | frameshift_deleti  on | exon7 | c.743_744del | p.R248fs | . | . |
| CTC6 | 6 | 36710247 | T | TC | 0.6 | NM_020939 | CPNE5 | frameshift_inserti  on | exon21 | c.1579dupG | p.D527fs | . | . |
| CTC6 | 6 | 39847206 | C | CG | 0.308 | NM_001201427 | DAAM2 | frameshift_inserti  on | exon14 | c.1798_1799ins  G | p.Q600fs | . | . |
| CTC6 | 6 | 41652535 | CG | C | 0.633 | NM_001271943 | TFEB | frameshift_deleti  on | exon8 | c.977delC | p.P326fs | . | . |
| CTC6 | 6 | 41754573 | C | CCTT | 0.146 | NM_013397 | PRICKLE4 | nonframeshift_in  sertion | exon8 | c.861_862insCT  T | p.L287delinsLL | . | . |
| CTC6 | 6 | 42156438 | GC | G | 0.535 | NM_002098 | GUCA1B | frameshift_deleti  on | exon2 | c.238delG | p.A80fs | . | . |

| CTC6 | 6 | 42231241 | CG | C | 0.357 | NM_033502 | TRERF1 | frameshift_deleti  on | exon8 | c.1700delC | p.P567fs | . | . |
| --- | --- | --- | --- | --- | --- | --- | --- | --- | --- | --- | --- | --- | --- |
| CTC6 | 6 | 42638435 | TC | T | 0.357 | NM_015255 | UBR2 | frameshift_deleti  on | exon36 | c.4069delC | p.P1357fs | . | . |
| CTC6 | 6 | 42930057 | AG | A | 0.842 | NM_018960 | GNMT | frameshift_deleti  on | exon2 | c.315delG | p.E105fs | . | . |
| CTC6 | 6 | 42985385 | CG | C | 0.702 | NM_057161 | KLHDC3 | frameshift_deleti  on | exon3 | c.284delG | p.R95fs | . | . |
| CTC6 | 6 | 43155111 | TA | T | 0.198 | NM_015089 | CUL9 | frameshift_deleti  on | exon6 | c.1516delA | p.I506fs | . | . |
| CTC6 | 6 | 43155705 | CA | C | 0.267 | NM_015089 | CUL9 | frameshift_deleti  on | exon7 | c.1837delA | p.K613fs | . | . |
| CTC6 | 6 | 43400109 | CG | C | 0.446 | NM_033450 | ABCC10 | frameshift_deleti  on | exon1 | c.263delG | p.R88fs | . | . |
| CTC6 | 6 | 43403613 | CA | C | 0.833 | NM_033450 | ABCC10 | frameshift_deleti  on | exon3 | c.1605delA | p.P535fs | . | . |
| CTC6 | 6 | 43488414 | TG | T | 0.4 | NM_203290 | POLR1C | frameshift_deleti  on | exon7 | c.708delG | p.L236fs | . | . |
| CTC6 | 6 | 44143886 | GA | G | 0.192 | NM_007058 | CAPN11 | frameshift_deleti  on | exon8 | c.914delA | p.D305fs | . | . |
| CTC6 | 6 | 44222723 | T | TA | 0.278 | NM_001286519 | SLC35B2 | frameshift_inserti  on | exon2 | c.619_620insT | p.H207fs | . | . |
| CTC6 | 6 | 46827142 | GA | G | 0.545 | NM_001098518 | GPR116 | frameshift_deleti  on | exon17 | c.2497delT | p.S833fs | . | . |
| CTC6 | 6 | 49753736 | CT | C | 0.218 | NM_138733 | PGK2 | frameshift_deleti  on | exon1 | c.1164delA | p.K388fs | . | . |
| CTC6 | 6 | 50683137 | GC | G | 0.903 | NM_172238 | TFAP2D | frameshift_deleti  on | exon2 | c.349delC | p.H117fs | . | . |
| CTC6 | 6 | 51918045 | GC | G | 0.5 | NM_138694 | PKHD1 | frameshift_deleti  on | exon21 | c.1968delG | p.W656fs | . | . |
| CTC6 | 6 | 53764599 | GA | G | 0.531 | NM_018214 | LRRC1 | frameshift_deleti  on | exon8 | c.698delA | p.E233fs | . | . |
| CTC6 | 6 | 54804780 | CA | C | 0.3 | NM_001010872 | FAM83B | frameshift_deleti  on | exon5 | c.1012delA | p.K338fs | . | . |
| CTC6 | 6 | 56420536 | C | CA | 0.75 | NM_015548 | DST | frameshift_inserti  on | exon41 | c.6873dupT | p.G2292fs | . | . |
| CTC6 | 6 | 56965538 | TCA | T | 0.15 | NM_001257273 | ZNF451 | frameshift_deleti  on | exon4 | c.325_326del | p.Q109fs | . | . |
| CTC6 | 6 | 71235690 | AT | A | 0.6 | NM_001162529 | FAM135A | frameshift_deleti  on | exon13 | c.2904delT | p.N968fs | . | . |
| CTC6 | 6 | 75887646 | CT | C | 0.326 | NM_004370 | COL12A1 | frameshift_deleti  on | exon12 | c.2169delA | p.K723fs | . | . |
| CTC6 | 6 | 76717203 | TTTTC | T | 0.222 | NM_001282368 | IMPG1 | frameshift_deleti  on | exon8 | c.643_646del | p.E215fs | . | . |
| CTC6 | 6 | 82461722 | CCGCCA  CCGCCG | C | 0.237 | NM_017633 | FAM46A | nonframeshift_de  letion | exon2 | c.122_136del | p.41_46del | . | . |
| CTC6 | 6 | 82461727 | A | ACCGC  CGAAG | 1 | NM_017633 | FAM46A | nonframeshift_in  sertion | exon2 | c.131_132insC  GGCGACTTCG | p.G44delinsGG  DFGG | . | . |
| CTC6 | 6 | 84233904 | CT | C | 0.571 | NM_153362 | PRSS35 | frameshift_deleti  on | exon2 | c.745delT | p.F249fs | . | . |
| CTC6 | 6 | 84618755 | ATGC | A | 0.308 | NM_016230 | CYB5R4 | nonframeshift_de  letion | exon4 | c.359_361del | p.120_121del | . | . |
| CTC6 | 6 | 90384088 | GC | G | 0.32 | NM_014611 | MDN1 | frameshift_deleti  on | exon79 | c.12981delG | p.G4327fs | . | . |
| CTC6 | 6 | 90604349 | C | CA | 0.266 | NM_032602 | GJA10 | frameshift_inserti  on | exon1 | c.163dupA | p.C54fs | . | . |
| CTC6 | 6 | 106554938 | GC | G | 0.485 | NM_182907 | PRDM1 | frameshift_deleti  on | exon5 | c.1654delC | p.P552fs | . | . |
| CTC6 | 6 | 106727692 | GA | G | 0.75 | NM_001286107 | ATG5 | frameshift_deleti  on | exon4 | c.87delT | p.F29fs | . | . |
| CTC6 | 6 | 106960420 | GGGC | G | 0.5 | NM_001624 | AIM1 | nonframeshift_de  letion | exon1 | c.205_207del | p.69_69del | . | . |
| CTC6 | 6 | 106960421 | GGC | G | 0.96 | NM_001624 | AIM1 | frameshift_deleti  on | exon1 | c.206_207del | p.G69fs | . | . |
| CTC6 | 6 | 109480625 | CT | C | 0.69 | NM_001271852 | CEP57L1 | frameshift_deleti  on | exon9 | c.977delT | p.L326fs | . | . |
| CTC6 | 6 | 109787616 | AT | A | 0.562 | NM_014797 | ZBTB24 | frameshift_deleti  on | exon7 | c.1531delA | p.I511fs | . | . |
| CTC6 | 6 | 109816590 | C | CA | 0.344 | NM_001145128 | AK9 | frameshift_inserti  on | exon39 | c.5368_5369ins  T | p.R1790fs | . | . |
| CTC6 | 6 | 111587972 | GT | G | 0.772 | NM_153369 | KIAA1919 | frameshift_deleti  on | exon4 | c.1208delT | p.V403fs | . | . |
| CTC6 | 6 | 116600465 | T | TCAC | 1 | NM_003309 | TSPYL1 | nonframeshift_in  sertion | exon1 | c.528_529insGT  G | p.K177delinsVK | . | . |
| CTC6 | 6 | 116914152 | AC | A | 0.5 | NM_015952 | RWDD1 | frameshift_deleti  on | exon7 | c.621delC | p.N207fs | . | . |
| CTC6 | 6 | 118635257 | TC | T | 0.66 | NM_001029858 | SLC35F1 | frameshift_deleti  on | exon8 | c.1070delC | p.S357fs | . | . |
| CTC6 | 6 | 119136179 | GT | G | 0.286 | NM_017696 | MCM9 | frameshift_deleti  on | exon12 | c.3239delA | p.N1080fs | . | . |
| CTC6 | 6 | 119238924 | CA | C | 0.444 | NM_017696 | MCM9 | frameshift_deleti  on | exon4 | c.705delT | p.G235fs | . | . |
| CTC6 | 6 | 125366400 | GT | G | 0.3 | NM_001286398 | RNF217 | frameshift_deleti  on | exon2 | c.927delT | p.C309fs | . | . |
| CTC6 | 6 | 127648201 | AT | A | 0.625 | NM_001105544 | ECHDC1 | frameshift_deleti  on | exon2 | c.83delA | p.N28fs | . | . |
| CTC6 | 6 | 129920489 | TA | T | 0.29 | NM_033515 | ARHGAP18 | frameshift_deleti  on | exon12 | c.1584delT | p.F528fs | . | . |
| CTC6 | 6 | 130370911 | TC | T | 0.628 | NM_032438 | L3MBTL3 | frameshift_deleti  on | exon5 | c.226delC | p.P76fs | . | . |
| CTC6 | 6 | 131277490 | CT | C | 0.729 | NM_001135554 | EPB41L2 | frameshift_deleti  on | exon2 | c.135delA | p.K45fs | . | . |
| CTC6 | 6 | 132171170 | G | GA | 0.474 | NM_006208 | ENPP1 | frameshift_inserti  on | exon3 | c.355dupA | p.G118fs | . | . |
| CTC6 | 6 | 133013675 | A | AT | 1 | NM_004666 | VNN1 | frameshift_inserti  on | exon5 | c.874dupA | p.M292fs | . | . |
| CTC6 | 6 | 133844297 | TA | T | 0.205 | NM_172103 | EYA4 | frameshift_deleti  on | exon17 | c.1652delA | p.Y551fs | . | . |
| CTC6 | 6 | 134492178 | C | CT | 0.5 | NM_001143677 | SGK1 | frameshift_inserti  on | exon10 | c.1104_1105ins  A | p.G369fs | . | . |
| CTC6 | 6 | 134492788 | A | AT | 0.212 | NM_001143677 | SGK1 | frameshift_inserti  on | exon9 | c.952dupA | p.M318fs | . | . |
| CTC6 | 6 | 135518455 | C | CCG | 0.154 | NM_001130173 | MYB | frameshift_inserti  on | exon10 | c.1560_1561ins  CG | p.P520fs | . | . |
| CTC6 | 6 | 135787078 | AT | A | 0.6 | NM_001134830 | AHI1 | stopgain | exon5 | c.622delA | p.I208X | . | . |
| CTC6 | 6 | 138539183 | A | AG | 1 | NM_021635 | PBOV1 | frameshift_inserti on | exon1 | c.349dupC | p.L117fs | . | . |
| CTC6 | 6 | 139226206 | GGT | G | 0.161 | NM_001286612 | REPS1 | frameshift_deleti  on | exon17 | c.2111_2112del | p.H704fs | . | . |
| CTC6 | 6 | 143091921 | CA | C | 0.382 | NM_006734 | HIVEP2 | frameshift_deleti  on | exon5 | c.3954delT | p.F1318fs | . | . |
| CTC6 | 6 | 143093646 | TG | T | 0.643 | NM_006734 | HIVEP2 | frameshift_deleti  on | exon5 | c.2229delC | p.A743fs | . | . |

| CTC6 | 6 | 145075902 | AT | A | 0.327 | NM_007124 | UTRN | frameshift_deleti  on | exon56 | c.8403delT | p.D2801fs | . | . |
| --- | --- | --- | --- | --- | --- | --- | --- | --- | --- | --- | --- | --- | --- |
| CTC6 | 6 | 146997509 | TC | T | 0.909 | NM_024694 | ADGB | frameshift_deleti  on | exon9 | c.1149delC | p.F383fs | . | . |
| CTC6 | 6 | 147635085 | AT | A | 0.833 | NM_001127715 | STXBP5 | frameshift_deleti  on | exon12 | c.1212delT | p.Y404fs | . | . |
| CTC6 | 6 | 147660480 | CA | C | 0.562 | NM_001127715 | STXBP5 | frameshift_deleti  on | exon21 | c.2215delA | p.K739fs | . | . |
| CTC6 | 6 | 150005514 | TG | T | 0.833 | NM_001270519 | LATS1 | frameshift_deleti  on | exon4 | c.710delC | p.P237fs | . | . |
| CTC6 | 6 | 150141741 | CT | C | 0.466 | NM_032832 | LRP11 | frameshift_deleti  on | exon7 | c.1446delA | p.K482fs | . | . |
| CTC6 | 6 | 150211241 | C | CA | 0.391 | NM_001243327 | RAET1E | frameshift_inserti  on | exon2 | c.125dupT | p.L42fs | . | . |
| CTC6 | 6 | 150289960 | TA | T | 0.273 | NM_025218 | ULBP1 | frameshift_deleti  on | exon2 | c.304delA | p.K102fs | . | . |
| CTC6 | 6 | 151687307 | AT | A | 0.474 | NM_020861 | ZBTB2 | frameshift_deleti  on | exon3 | c.893delA | p.N298fs | . | . |
| CTC6 | 6 | 151789830 | TA | T | 0.267 | NM_001286562 | C6orf211 | frameshift_deleti  on | exon4 | c.555delA | p.V185fs | . | . |
| CTC6 | 6 | 152542053 | GT | G | 0.7 | NM_033071 | SYNE1 | frameshift_deleti  on | exon118 | c.21571delA | p.T7191fs | . | . |
| CTC6 | 6 | 152738121 | CT | C | 0.349 | NM_033071 | SYNE1 | frameshift_deleti  on | exon41 | c.5471delA | p.K1824fs | . | . |
| CTC6 | 6 | 153311185 | GA | G | 0.75 | NM_019041 | MTRF1L | frameshift_deleti  on | exon7 | c.987delT | p.F329fs | . | . |
| CTC6 | 6 | 154481054 | TG | T | 0.6 | NM_001130700 | IPCEF1 | frameshift_deleti  on | exon12 | c.1225delC | p.Q409fs | . | . |
| CTC6 | 6 | 155113993 | AAT | A | 0.182 | NM_014892 | SCAF8 | frameshift_deleti  on | exon5 | c.359_360del | p.N120fs | . | . |
| CTC6 | 6 | 155153329 | AC | A | 0.368 | NM_014892 | SCAF8 | frameshift_deleti  on | exon20 | c.2617delC | p.P873fs | . | . |
| CTC6 | 6 | 155596890 | C | CT | 0.167 | NM_001001346 | CLDN20 | frameshift_inserti  on | exon2 | c.38dupT | p.L13fs | . | . |
| CTC6 | 6 | 157522323 | TC | T | 0.263 | NM_017519 | ARID1B | frameshift_deleti  on | exon17 | c.4557delC | p.I1519fs | . | . |
| CTC6 | 6 | 159010813 | CA | C | 0.259 | NM_020823 | TMEM181 | frameshift_deleti  on | exon6 | c.903delA | p.T301fs | . | . |
| CTC6 | 6 | 159660779 | GCCACC  ACCCGC CGCACG | G | 1 | NM_032532 | FNDC1 | nonframeshift_de letion | exon14 | c.4412_4429del | p.1471_1477de l | . | . |
| CTC6 | 6 | 160164718 | GA | G | 0.278 | NM_001270531 | WTAP | frameshift_deleti  on | exon5 | c.168delA | p.R56fs | . | . |
| CTC6 | 6 | 160450624 | CT | C | 0.241 | NM_000876 | IGF2R | frameshift_deleti  on | exon7 | c.820delT | p.F274fs | . | . |
| CTC6 | 6 | 160560897 | CTGGTA  AGT | C | 0.674 | NM_003057 | SLC22A1 | frameshift_deleti  on | exon7 | c.1275_1276del | p.P425fs | . | . |
| CTC6 | 6 | 168323537 | AT | A | 0.571 | NM_001207008 | MLLT4 | frameshift_deleti  on | exon20 | c.2842delT | p.F948fs | . | . |
| CTC6 | 6 | 168709246 | AC | A | 1 | NM_001286350 | DACT2 | frameshift_deleti  on | exon3 | c.680delG | p.G227fs | . | . |
| CTC6 | 6 | 169053671 | CG | C | 0.216 | NM_001166412 | SMOC2 | frameshift_deleti  on | exon11 | c.1049delG | p.R350fs | . | . |
| CTC6 | 6 | 169649038 | TC | T | 0.444 | NM_003247 | THBS2 | frameshift_deleti  on | exon4 | c.82delG | p.D28fs | . | . |
| CTC6 | 6 | 170870994 | GGCAGC  AGCAGC AACAAC AACAGC | G | 0.412 | NM_001172085 | TBP | nonframeshift_de letion | exon2 | c.111_140del | p.37_47del | . | . |
| CTC6 | 6 | 170871013 | A | ACAG | 0.857 | NM_001172085 | TBP | nonframeshift_in  sertion | exon2 | c.129_130insCA  G | p.Q43delinsQQ | . | . |
| CTC6 | 6 | 170871034 | G | GCAA | 0.6 | NM_001172085 | TBP | nonframeshift_in  sertion | exon2 | c.150_151insCA  A | p.Q50delinsQQ | . | . |
| CTC6 | 6 | 170871037 | GCAA | G | 0.562 | NM_001172085 | TBP | nonframeshift_de  letion | exon2 | c.154_156del | p.52_52del | . | . |
| CTC6 | 6 | 170871046 | ACAGCA  GCAG | A | 1 | NM_001172085 | TBP | nonframeshift_de  letion | exon2 | c.163_171del | p.55_57del | . | . |
| CTC6 | 7 | 2274952 | TG | T | 0.889 | NM_013393 | FTSJ2 | frameshift_deleti  on | exon3 | c.545delC | p.P182fs | . | . |
| CTC6 | 7 | 4780545 | TC | T | 0.5 | NM_001037165 | FOXK1 | frameshift_deleti  on | exon2 | c.638delC | p.S213fs | . | . |
| CTC6 | 7 | 5103445 | CA | C | 0.405 | NM_021163 | RBAK | frameshift_deleti  on | exon5 | c.359delA | p.Q120fs | . | . |
| CTC6 | 7 | 5353101 | CT | C | 0.636 | NM_001080495 | TNRC18 | frameshift_deleti  on | exon27 | c.7420delA | p.S2474fs | . | . |
| CTC6 | 7 | 5355603 | A | AG | 0.688 | NM_001080495 | TNRC18 | frameshift_inserti  on | exon25 | c.6845dupC | p.P2282fs | . | . |
| CTC6 | 7 | 5396885 | ATCT | A | 0.136 | NM_001080495 | TNRC18 | nonframeshift_de  letion | exon16 | c.4853_4855del | p.1618_1619de  l | . | . |
| CTC6 | 7 | 6187382 | GA | G | 0.421 | NM_032172 | USP42 | frameshift_deleti  on | exon12 | c.1246delA | p.K416fs | . | . |
| CTC6 | 7 | 6370150 | GA | G | 0.404 | NM_001037163 | FAM220A | frameshift_deleti  on | exon2 | c.635delT | p.F212fs | . | . |
| CTC6 | 7 | 6554038 | AT | A | 0.579 | NM_001145118 | GRID2IP | frameshift_deleti  on | exon8 | c.1390delA | p.I464fs | . | . |
| CTC6 | 7 | 6731081 | A | AT | 0.211 | NM_016265 | ZNF12 | frameshift_inserti  on | exon5 | c.1491dupA | p.C498fs | . | . |
| CTC6 | 7 | 7612435 | C | CA | 0.583 | NM_019005 | MIOS | frameshift_inserti  on | exon4 | c.330dupA | p.P110fs | . | . |
| CTC6 | 7 | 11485875 | GTC | G | 0.75 | NM_015204 | THSD7A | frameshift_deleti  on | exon13 | c.2875_2876del | p.D959fs | . | . |
| CTC6 | 7 | 12391268 | T | TA | 1 | NM_001135924 | VWDE | stopgain | exon19 | c.3816dupT | p.K1273_E1274  delinsX | . | . |
| CTC6 | 7 | 12409198 | TA | T | 0.242 | NM_001135924 | VWDE | frameshift_deleti  on | exon12 | c.2733delT | p.F911fs | . | . |
| CTC6 | 7 | 18067193 | CA | C | 0.265 | NM_175886 | PRPS1L1 | stopgain | exon1 | c.212delT | p.L71X | . | . |
| CTC6 | 7 | 18067354 | TC | T | 0.333 | NM_175886 | PRPS1L1 | frameshift_deleti on | exon1 | c.51delG | p.Q17fs | . | . |
| CTC6 | 7 | 22478153 | AT | A | 0.6 | NM_001164460 | STEAP1B | frameshift_deleti  on | exon5 | c.983delA | p.N328fs | . | . |
| CTC6 | 7 | 22768313 | C | CA | 0.444 | NM_000600 | IL6 | frameshift_inserti  on | exon3 | c.213dupA | p.T71fs | . | . |
| CTC6 | 7 | 23023615 | TTG | T | 0.4 | NM_032581 | FAM126A | frameshift_deleti  on | exon3 | c.99_100del | p.D33fs | . | . |
| CTC6 | 7 | 23221714 | TG | T | 0.206 | NM_007342 | NUPL2 | frameshift_deleti  on | exon1 | c.11delG | p.C4fs | . | . |
| CTC6 | 7 | 25218830 | AG | A | 0.952 | NM_138811 | C7orf31 | frameshift_deleti  on | exon2 | c.97delC | p.L33fs | . | . |
| CTC6 | 7 | 30363279 | CA | C | 0.298 | NM_147128 | ZNRF2 | frameshift_deleti  on | exon2 | c.492delA | p.S164fs | . | . |
| CTC6 | 7 | 30825397 | AT | A | 0.625 | NM_032222 | FAM188B | frameshift_deleti  on | exon4 | c.453delT | p.N151fs | . | . |

| CTC6 | 7 | 31378770 | G | GA | 0.5 | NM_022728 | NEUROD6 | frameshift_inserti  on | exon2 | c.112dupT | p.S38fs | . | . |
| --- | --- | --- | --- | --- | --- | --- | --- | --- | --- | --- | --- | --- | --- |
| CTC6 | 7 | 34989411 | CT | C | 0.487 | NM_015283 | DPY19L1 | frameshift_deleti  on | exon14 | c.1200delA | p.K400fs | . | . |
| CTC6 | 7 | 35912339 | AC | A | 0.652 | NM_001242956 | 7-Sep | frameshift_deleti  on | exon3 | c.233delC | p.T78fs | . | . |
| CTC6 | 7 | 36324398 | CG | C | 0.526 | NM_030636 | EEPD1 | frameshift_deleti  on | exon5 | c.1146delG | p.A382fs | . | . |
| CTC6 | 7 | 38768374 | GC | G | 0.25 | NM_080631 | VPS41 | frameshift_deleti  on | exon25 | c.2201delG | p.G734fs | . | . |
| CTC6 | 7 | 38812210 | TA | T | 1 | NM_080631 | VPS41 | frameshift_deleti  on | exon12 | c.964delT | p.Y322fs | . | . |
| CTC6 | 7 | 43916972 | GT | G | 0.433 | NM_017920 | URGCP | frameshift_deleti  on | exon5 | c.2062delA | p.T688fs | . | . |
| CTC6 | 7 | 44152501 | GA | G | 0.348 | NM_001129 | AEBP1 | frameshift_deleti  on | exon18 | c.2563delA | p.T855fs | . | . |
| CTC6 | 7 | 44573423 | TC | T | 0.5 | NM_001101648 | NPC1L1 | frameshift_deleti  on | exon7 | c.2195delG | p.R732fs | . | . |
| CTC6 | 7 | 44687062 | CA | C | 0.611 | NM_001165036 | OGDH | frameshift_deleti  on | exon4 | c.435delA | p.A145fs | . | . |
| CTC6 | 7 | 44839430 | AC | A | 0.5 | NM_021130 | PPIA | frameshift_deleti  on | exon4 | c.320delC | p.T107fs | . | . |
| CTC6 | 7 | 45956222 | C | CT | 0.244 | NM_000598 | IGFBP3 | frameshift_inserti  on | exon3 | c.674dupA | p.K225fs | . | . |
| CTC6 | 7 | 48081012 | AT | A | 0.875 | NM_001100159 | C7orf57 | frameshift_deleti  on | exon3 | c.138delT | p.N46fs | . | . |
| CTC6 | 7 | 48528852 | GT | G | 0.722 | NM_152701 | ABCA13 | frameshift_deleti  on | exon48 | c.13203delT | p.G4401fs | . | . |
| CTC6 | 7 | 48545964 | GC | G | 0.367 | NM_152701 | ABCA13 | frameshift_deleti  on | exon49 | c.13325delC | p.A4442fs | . | . |
| CTC6 | 7 | 50605660 | GC | G | 0.205 | NM_001242886 | DDC | frameshift_deleti  on | exon3 | c.218delG | p.C73fs | . | . |
| CTC6 | 7 | 50742157 | TG | T | 0.269 | NM_001001549 | GRB10 | frameshift_deleti  on | exon3 | c.337delC | p.Q113fs | . | . |
| CTC6 | 7 | 51096416 | AG | A | 0.532 | NM_015198 | COBL | frameshift_deleti  on | exon10 | c.2376delC | p.P792fs | . | . |
| CTC6 | 7 | 55238092 | GC | G | 0.207 | NM_201284 | EGFR | frameshift_deleti  on | exon16 | c.1974delC | p.S658fs | . | . |
| CTC6 | 7 | 55560101 | TC | T | 0.25 | NM_001284282 | VOPP1 | frameshift_deleti  on | exon4 | c.150delG | p.L50fs | . | . |
| CTC6 | 7 | 64292107 | GA | G | 0.8 | NM_001271649 | ZNF138 | frameshift_deleti  on | exon2 | c.47delA | p.E16fs | . | . |
| CTC6 | 7 | 64389314 | TA | T | 1 | NM_021148 | ZNF273 | frameshift_deleti  on | exon4 | c.1609delA | p.T537fs | . | . |
| CTC6 | 7 | 64864011 | TA | T | 0.533 | NM_001287534 | ZNF92 | frameshift_deleti  on | exon2 | c.757delA | p.K253fs | . | . |
| CTC6 | 7 | 64864339 | TC | T | 0.421 | NM_001287534 | ZNF92 | stopgain | exon2 | c.1085delC | p.S362X | . | . |
| CTC6 | 7 | 65419353 | T | TTTA | 0.61 | NM_001284342 | VKORC1L1 | nonframeshift_in sertion | exon2 | c.487_488insTT A | p.F163delinsFI | . | . |
| CTC6 | 7 | 65557053 | TA | T | 0.364 | NM_001024944 | ASL | frameshift_deleti  on | exon13 | c.1064delA | p.Y355fs | . | . |
| CTC6 | 7 | 75053395 | TG | T | 0.9 | NM_001099415 | POM121C | frameshift_deleti  on | exon12 | c.984delC | p.S328fs | . | . |
| CTC6 | 7 | 75190645 | G | GT | 0.371 | NM_001243198 | HIP1 | frameshift_inserti  on | exon13 | c.1178dupA | p.N393fs | . | . |
| CTC6 | 7 | 75615329 | CA | C | 0.172 | NM_000941 | POR | frameshift_deleti  on | exon14 | c.1759delA | p.R587fs | . | . |
| CTC6 | 7 | 75659778 | GT | G | 0.686 | NM_016086 | STYXL1 | frameshift_deleti  on | exon2 | c.63delA | p.K21fs | . | . |
| CTC6 | 7 | 76240785 | CA | C | 0.615 | NM_012230 | POMZP3 | frameshift_deleti  on | exon6 | c.560delT | p.L187fs | . | . |
| CTC6 | 7 | 76952966 | GT | G | 0.374 | NM_017439 | GSAP | frameshift_deleti  on | exon24 | c.1920delA | p.K640fs | . | . |
| CTC6 | 7 | 77423345 | AG | A | 0.5 | NM_032936 | TMEM60 | frameshift_deleti  on | exon2 | c.345delC | p.A115fs | . | . |
| CTC6 | 7 | 82581488 | T | TTCA | 1 | NM_014510 | PCLO | nonframeshift_in  sertion | exon5 | c.8780_8781ins  TGA | p.E2927delinsD  E | . | . |
| CTC6 | 7 | 82595521 | GT | G | 0.562 | NM_014510 | PCLO | frameshift_deleti  on | exon4 | c.3582delA | p.K1194fs | . | . |
| CTC6 | 7 | 87908718 | GT | G | 0.353 | NM_001205316 | STEAP4 | frameshift_deleti  on | exon4 | c.846delA | p.K282fs | . | . |
| CTC6 | 7 | 90355944 | GAC | G | 0.6 | NM_001287136 | CDK14 | frameshift_deleti  on | exon2 | c.50_51del | p.D17fs | . | . |
| CTC6 | 7 | 92735377 | CT | C | 0.76 | NM_001193307 | SAMD9 | frameshift_deleti  on | exon2 | c.33delA | p.T11fs | . | . |
| CTC6 | 7 | 92763222 | TA | T | 1 | NM_152703 | SAMD9L | frameshift_deleti  on | exon5 | c.2062delT | p.Y688fs | . | . |
| CTC6 | 7 | 92764992 | GT | G | 0.333 | NM_152703 | SAMD9L | frameshift_deleti  on | exon5 | c.292delA | p.T98fs | . | . |
| CTC6 | 7 | 93536089 | GA | G | 0.579 | NM_021955 | GNGT1 | frameshift_deleti  on | exon2 | c.32delA | p.E11fs | . | . |
| CTC6 | 7 | 94218014 | T | TG | 0.214 | NM_001099400 | SGCE | frameshift_inserti  on | exon9 | c.1256_1257ins  C | p.Q419fs | . | . |
| CTC6 | 7 | 94937354 | CA | C | 1 | NM_000446 | PON1 | frameshift_deleti  on | exon6 | c.666delT | p.F222fs | . | . |
| CTC6 | 7 | 98491424 | GA | G | 1 | NM_001244580 | TRRAP | frameshift_deleti  on | exon6 | c.371delA | p.E124fs | . | . |
| CTC6 | 7 | 99091508 | A | AT | 0.193 | NM_032164 | ZNF394 | frameshift_inserti  on | exon3 | c.1329dupA | p.C444fs | . | . |
| CTC6 | 7 | 99123892 | C | CT | 0.226 | NM_014569 | ZKSCAN5 | frameshift_inserti  on | exon6 | c.1230dupT | p.A410fs | . | . |
| CTC6 | 7 | 99247740 | TG | T | 0.625 | NM_000777 | CYP3A5 | frameshift_deleti  on | exon12 | c.1368delC | p.I456fs | . | . |
| CTC6 | 7 | 99355806 | GT | G | 0.6 | NM_001202855 | CYP3A4 | frameshift_deleti  on | exon13 | c.1458delA | p.K486fs | . | . |
| CTC6 | 7 | 99662511 | GTAGT | G | 1 | NM_017715 | ZNF3 | frameshift_deleti  on | exon6 | c.292_295del | p.T98fs | . | . |
| CTC6 | 7 | 99709857 | CG | C | 0.175 | NM_001190415 | TAF6 | frameshift_deleti  on | exon7 | c.704delC | p.P235fs | . | . |
| CTC6 | 7 | 99758055 | GC | G | 0.652 | NM_024637 | GAL3ST4 | frameshift_deleti  on | exon4 | c.956delG | p.C319fs | . | . |
| CTC6 | 7 | 100635720 | AC | A | 0.431 | NM_001164462 | MUC12 | frameshift_deleti  on | exon2 | c.1877delC | p.T626fs | . | . |
| CTC6 | 7 | 100636556 | TC | T | 0.321 | NM_001164462 | MUC12 | frameshift_deleti  on | exon2 | c.2713delC | p.Q905fs | . | . |
| CTC6 | 7 | 100646724 | TC | T | 0.244 | NM_001164462 | MUC12 | stopgain | exon2 | c.12881delC | p.S4294X | . | . |
| CTC6 | 7 | 100685954 | AC | A | 0.6 | NM_001040105 | MUC17 | frameshift_deleti on | exon3 | c.11258delC | p.T3753fs | . | . |
| CTC6 | 7 | 102107872 | TG | T | 0.323 | NM_152892 | LRWD1 | frameshift_deleti  on | exon4 | c.520delG | p.V174fs | . | . |
| CTC6 | 7 | 103136210 | CG | C | 0.189 | NM_005045 | RELN | frameshift_deleti  on | exon57 | c.9328delC | p.R3110fs | . | . |
| CTC6 | 7 | 103205966 | G | GA | 0.773 | NM_005045 | RELN | frameshift_inserti  on | exon34 | c.4968dupT | p.H1657fs | . | . |

| CTC6 | 7 | 103276898 | GA | G | 0.39 | NM_005045 | RELN | frameshift_deleti  on | exon18 | c.2086delT | p.S696fs | . | . |
| --- | --- | --- | --- | --- | --- | --- | --- | --- | --- | --- | --- | --- | --- |
| CTC6 | 7 | 104751229 | GA | G | 0.333 | NM_018682 | KMT2E | frameshift_deleti  on | exon25 | c.3983delA | p.E1328fs | . | . |
| CTC6 | 7 | 104844169 | CGGT | C | 0.109 | NM_182691 | SRPK2 | nonframeshift_de  letion | exon2 | c.99_101del | p.33_34del | . | . |
| CTC6 | 7 | 106820390 | CT | C | 0.212 | NM_001244262 | HBP1 | frameshift_deleti  on | exon2 | c.83delT | p.L28fs | . | . |
| CTC6 | 7 | 107211676 | TA | T | 0.254 | NM_001270419 | DUS4L | frameshift_deleti  on | exon4 | c.204delA | p.I68fs | . | . |
| CTC6 | 7 | 107752257 | AT | A | 0.385 | NM_007356 | LAMB4 | frameshift_deleti  on | exon4 | c.326delA | p.N109fs | . | . |
| CTC6 | 7 | 107834609 | GT | G | 0.419 | NM_001037132 | NRCAM | frameshift_deleti  on | exon14 | c.1636delA | p.T546fs | . | . |
| CTC6 | 7 | 113517920 | AT | A | 0.282 | NM_002711 | PPP1R3A | frameshift_deleti  on | exon4 | c.3226delA | p.I1076fs | . | . |
| CTC6 | 7 | 113519154 | AT | A | 0.533 | NM_002711 | PPP1R3A | frameshift_deleti  on | exon4 | c.1992delA | p.K664fs | . | . |
| CTC6 | 7 | 113519717 | A | AT | 0.377 | NM_002711 | PPP1R3A | frameshift_inserti  on | exon4 | c.1429dupA | p.I477fs | . | . |
| CTC6 | 7 | 120884264 | AT | A | 0.875 | NM_001105533 | CPED1 | frameshift_deleti  on | exon17 | c.2183delT | p.I728fs | . | . |
| CTC6 | 7 | 121650570 | TG | T | 0.449 | NM_001206838 | PTPRZ1 | frameshift_deleti  on | exon12 | c.1471delG | p.G491fs | . | . |
| CTC6 | 7 | 122338109 | TA | T | 0.818 | NM_139175 | RNF133 | frameshift_deleti  on | exon1 | c.863delT | p.L288fs | . | . |
| CTC6 | 7 | 122809285 | TG | T | 1 | NM_022444 | SLC13A1 | frameshift_deleti  on | exon4 | c.469delC | p.Q157fs | . | . |
| CTC6 | 7 | 123508637 | CA | C | 0.821 | NM_012269 | HYAL4 | frameshift_deleti  on | exon3 | c.311delA | p.Q104fs | . | . |
| CTC6 | 7 | 127979697 | CT | C | 0.381 | NM_018077 | RBM28 | frameshift_deleti  on | exon2 | c.266delA | p.K89fs | . | . |
| CTC6 | 7 | 128034563 | TG | T | 0.333 | NM_001142575 | IMPDH1 | frameshift_deleti  on | exon11 | c.1310delC | p.A437fs | . | . |
| CTC6 | 7 | 128119459 | TA | T | 0.4 | NM_018396 | METTL2B | frameshift_deleti  on | exon3 | c.451delA | p.K151fs | . | . |
| CTC6 | 7 | 128587351 | CACTCT  GCAGCC GCCCAC TCTGCG | C | 1 | NM_001098627 | IRF5 | nonframeshift_de letion | exon6 | c.502_531del | p.168_177del | . | . |
| CTC6 | 7 | 129045680 | TA | T | 0.8 | NM_001130720 | AHCYL2 | frameshift_deleti  on | exon9 | c.1144delA | p.K382fs | . | . |
| CTC6 | 7 | 129098196 | GC | G | 0.231 | NM_001134336 | STRIP2 | frameshift_deleti  on | exon10 | c.1180delC | p.Q394fs | . | . |
| CTC6 | 7 | 131195722 | GT | G | 0.667 | NM_001018111 | PODXL | frameshift_deleti  on | exon2 | c.570delA | p.R190fs | . | . |
| CTC6 | 7 | 131195880 | GT | G | 0.204 | NM_001018111 | PODXL | frameshift_deleti  on | exon2 | c.412delA | p.T138fs | . | . |
| CTC6 | 7 | 135277803 | TA | T | 0.375 | NM_015135 | NUP205 | frameshift_deleti  on | exon12 | c.1694delA | p.Y565fs | . | . |
| CTC6 | 7 | 137154355 | GT | G | 0.347 | NM_004717 | DGKI | frameshift_deleti  on | exon25 | c.2437delA | p.T813fs | . | . |
| CTC6 | 7 | 137567175 | CA | C | 0.434 | NM_194071 | CREB3L2 | frameshift_deleti  on | exon11 | c.1469delT | p.L490fs | . | . |
| CTC6 | 7 | 137569822 | CT | C | 0.375 | NM_194071 | CREB3L2 | frameshift_deleti  on | exon10 | c.1188delA | p.Q396fs | . | . |
| CTC6 | 7 | 137612913 | CTGG | C | 1 | NM_001253775 | CREB3L2 | nonframeshift_de  letion | exon2 | c.299_301del | p.100_101del | . | . |
| CTC6 | 7 | 137792195 | G | GC | 0.205 | NM_001190906 | AKR1D1 | frameshift_inserti  on | exon6 | c.602dupC | p.A201fs | . | . |
| CTC6 | 7 | 138555946 | GA | G | 1 | NM_001164665 | KIAA1549 | frameshift_deleti  on | exon13 | c.4507delT | p.S1503fs | . | . |
| CTC6 | 7 | 138764615 | TG | T | 0.325 | NM_020119 | ZC3HAV1 | frameshift_deleti  on | exon4 | c.1071delC | p.P357fs | . | . |
| CTC6 | 7 | 139652356 | CT | C | 0.603 | NM_001166253 | TBXAS1 | frameshift_deleti  on | exon6 | c.461delT | p.L154fs | . | . |
| CTC6 | 7 | 139719898 | GAC | G | 0.5 | NM_001061 | TBXAS1 | frameshift_deleti  on | exon13 | c.1605_1840del | p.*535fs | . | . |
| CTC6 | 7 | 141464360 | GT | G | 0.273 | NM_016943 | TAS2R3 | frameshift_deleti  on | exon1 | c.403delT | p.L135fs | . | . |
| CTC6 | 7 | 141730528 | TC | T | 0.3 | NM_004668 | MGAM | stopgain | exon12 | c.1442delC | p.S481X | . | . |
| CTC6 | 7 | 143087005 | CG | C | 0.412 | NM_001010972 | ZYX | frameshift_deleti on | exon9 | c.1550delG | p.R517fs | . | . |
| CTC6 | 7 | 143095014 | TG | T | 0.242 | NM_005232 | EPHA1 | frameshift_deleti  on | exon8 | c.1613delC | p.P538fs | . | . |
| CTC6 | 7 | 143826729 | ACTT | A | 1 | NM_001001659 | OR2A14 | nonframeshift_de  letion | exon1 | c.525_527del | p.175_176del | . | . |
| CTC6 | 7 | 144345957 | GA | G | 0.176 | NM_001042482 | TPK1 | frameshift_deleti  on | exon5 | c.200delT | p.F67fs | . | . |
| CTC6 | 7 | 148508744 | AT | A | 0.625 | NM_001203249 | EZH2 | frameshift_deleti  on | exon15 | c.1751delA | p.N584fs | . | . |
| CTC6 | 7 | 149506211 | G | GC | 1 | NM_198455 | SSPO | frameshift_inserti  on | exon63 | c.9204dupC | p.S3068fs | . | . |
| CTC6 | 7 | 150554798 | CG | C | 0.25 | NM_001091 | AOC1 | frameshift_deleti  on | exon2 | c.1241delG | p.R414fs | . | . |
| CTC6 | 7 | 150710383 | CCG | C | 0.233 | NM_000603 | NOS3 | frameshift_deleti  on | exon25 | c.3172_3173del | p.R1058fs | . | . |
| CTC6 | 7 | 150813886 | TTG | T | 0.154 | NM_001042535 | AGAP3 | frameshift_deleti  on | exon2 | c.339_340del | p.F113fs | . | . |
| CTC6 | 7 | 154172044 | CA | C | 0.306 | NM_001039350 | DPP6 | frameshift_deleti  on | exon3 | c.188delA | p.Q63fs | . | . |
| CTC6 | 7 | 154760692 | CCTG | C | 0.278 | NM_007349 | PAXIP1 | nonframeshift_de  letion | exon7 | c.1216_1218del | p.406_406del | . | . |
| CTC6 | 7 | 156742964 | CG | C | 0.611 | NM_138400 | NOM1 | frameshift_deleti  on | exon1 | c.534delG | p.A178fs | . | . |
| CTC6 | 7 | 156743025 | CAAA | C | 1 | NM_138400 | NOM1 | nonframeshift_de  letion | exon1 | c.595_597del | p.199_199del | . | . |
| CTC6 | 7 | 158457364 | TCAC | T | 0.163 | NM_001281933 | NCAPG2 | nonframeshift_de  letion | exon14 | c.1555_1557del | p.519_519del | . | . |
| CTC6 | 8 | 1626388 | GT | G | 0.61 | NM_001277161 | DLGAP2 | frameshift_deleti  on | exon8 | c.2016delT | p.R672fs | . | . |
| CTC6 | 8 | 2954455 | TA | T | 0.237 | NM_033225 | CSMD1 | frameshift_deleti  on | exon47 | c.7053delT | p.I2351fs | . | . |
| CTC6 | 8 | 8234868 | C | CGCCG  CT | 1 | NM_001080826 | SGK223 | nonframeshift_in  sertion | exon2 | c.1050_1051ins  AGCGGC | p.A351delinsS  GA | . | . |
| CTC6 | 8 | 10467670 | TCTC | T | 1 | NM_178857 | RP1L1 | nonframeshift_de  letion | exon4 | c.3935_3937del | p.1312_1313de  l | . | . |
| CTC6 | 8 | 10474023 | TG | T | 0.5 | NM_178857 | RP1L1 | frameshift_deleti  on | exon3 | c.683delC | p.P228fs | . | . |
| CTC6 | 8 | 11666218 | GTCCCA  C | G | 1 | NM_001287750 | FDFT1 | nonframeshift_de  letion | exon1 | c.193_198del | p.65_66del | . | . |
| CTC6 | 8 | 11831472 | GC | G | 0.804 | NM_001033018 | DEFB136 | frameshift_deleti  on | exon2 | c.210delG | p.P70fs | . | . |

| CTC6 | 8 | 16963061 | GA | G | 0.442 | NM_181723 | MICU3 | frameshift_deleti  on | exon11 | c.1226delA | p.E409fs | . | . |
| --- | --- | --- | --- | --- | --- | --- | --- | --- | --- | --- | --- | --- | --- |
| CTC6 | 8 | 19192239 | AT | A | 0.308 | NM_001174160 | SH2D4A | frameshift_deleti  on | exon3 | c.250delT | p.S84fs | . | . |
| CTC6 | 8 | 20069232 | GA | G | 0.24 | NM_001693 | ATP6V1B2 | frameshift_deleti  on | exon7 | c.674delA | p.E225fs | . | . |
| CTC6 | 8 | 21964980 | A | AT | 0.133 | NM_024815 | NUDT18 | frameshift_inserti  on | exon5 | c.802dupA | p.I268fs | . | . |
| CTC6 | 8 | 21965228 | AC | A | 0.296 | NM_024815 | NUDT18 | frameshift_deleti  on | exon5 | c.554delG | p.C185fs | . | . |
| CTC6 | 8 | 22459362 | AG | A | 0.667 | NM_001013842 | C8orf58 | frameshift_deleti  on | exon3 | c.655delG | p.G219fs | . | . |
| CTC6 | 8 | 23049371 | A | AT | 0.4 | NM_003844 | TNFRSF10A | frameshift_inserti  on | exon10 | c.1242dupA | p.W415fs | . | . |
| CTC6 | 8 | 23305349 | TG | T | 0.75 | NM_001128930 | ENTPD4 | frameshift_deleti  on | exon4 | c.255delC | p.P85fs | . | . |
| CTC6 | 8 | 24344703 | CT | C | 0.833 | NM_003817 | ADAM7 | frameshift_deleti  on | exon11 | c.965delT | p.L322fs | . | . |
| CTC6 | 8 | 25132954 | TG | T | 0.8 | NM_024940 | DOCK5 | frameshift_deleti  on | exon4 | c.222delG | p.L74fs | . | . |
| CTC6 | 8 | 25266358 | T | TG | 0.28 | NM_024940 | DOCK5 | frameshift_inserti  on | exon50 | c.5307dupG | p.L1769fs | . | . |
| CTC6 | 8 | 25298125 | T | TA | 0.6 | NM_017634 | KCTD9 | frameshift_inserti  on | exon4 | c.278dupT | p.L93fs | . | . |
| CTC6 | 8 | 27528748 | AG | A | 0.194 | NM_016240 | SCARA3 | frameshift_deleti  on | exon6 | c.1702delG | p.G568fs | . | . |
| CTC6 | 8 | 27884543 | GC | G | 0.853 | NM_001010906 | NUGGC | frameshift_deleti  on | exon18 | c.2180delG | p.G727fs | . | . |
| CTC6 | 8 | 28209225 | GGCA | G | 0.181 | NM_018660 | ZNF395 | nonframeshift_de  letion | exon7 | c.1017_1019del | p.339_340del | . | . |
| CTC6 | 8 | 28588787 | G | GA | 0.778 | NM_001440 | EXTL3 | frameshift_inserti  on | exon4 | c.2197dupA | p.W732fs | . | . |
| CTC6 | 8 | 30706067 | GA | G | 0.667 | NM_031271 | TEX15 | frameshift_deleti  on | exon1 | c.466delT | p.S156fs | . | . |
| CTC6 | 8 | 33416263 | TA | T | 0.184 | NM_024787 | RNF122 | frameshift_deleti  on | exon2 | c.51delT | p.V17fs | . | . |
| CTC6 | 8 | 37707186 | AC | A | 0.37 | NM_018310 | BRF2 | frameshift_deleti  on | exon1 | c.115delG | p.V39fs | . | . |
| CTC6 | 8 | 37792669 | GT | G | 0.211 | NM_152413 | GOT1L1 | frameshift_deleti  on | exon8 | c.993delA | p.K331fs | . | . |
| CTC6 | 8 | 38123710 | C | CA | 0.941 | NM_001102559 | PPAPDC1B | frameshift_inserti  on | exon6 | c.582dupT | p.A195fs | . | . |
| CTC6 | 8 | 38275489 | AG | A | 0.441 | NM_001174066 | FGFR1 | frameshift_deleti  on | exon10 | c.1183delC | p.L395fs | . | . |
| CTC6 | 8 | 39836682 | GA | G | 0.214 | NM_194294 | IDO2 | frameshift_deleti  on | exon4 | c.332delA | p.E111fs | . | . |
| CTC6 | 8 | 40683095 | TC | T | 0.595 | NM_001135731 | ZMAT4 | frameshift_deleti  on | exon2 | c.100delG | p.E34fs | . | . |
| CTC6 | 8 | 42046568 | GT | G | 0.636 | NM_000930 | PLAT | frameshift_deleti  on | exon4 | c.136delA | p.T46fs | . | . |
| CTC6 | 8 | 42256307 | T | TA | 0.375 | NM_001135694 | VDAC3 | frameshift_inserti  on | exon5 | c.199dupA | p.C66fs | . | . |
| CTC6 | 8 | 54163481 | GT | G | 0.375 | NM_000912 | OPRK1 | frameshift_deleti  on | exon2 | c.116delA | p.N39fs | . | . |
| CTC6 | 8 | 55540213 | TC | T | 1 | NM_006269 | RP1 | frameshift_deleti  on | exon4 | c.3772delC | p.P1258fs | . | . |
| CTC6 | 8 | 56698314 | CCT | C | 0.727 | NM_024831 | TGS1 | frameshift_deleti  on | exon3 | c.204_205del | p.S68fs | . | . |
| CTC6 | 8 | 57221530 | GC | G | 0.556 | NM_138969 | SDR16C5 | frameshift_deleti  on | exon4 | c.521delG | p.C174fs | . | . |
| CTC6 | 8 | 59483454 | TG | T | 0.375 | NM_001007067 | SDCBP | frameshift_deleti  on | exon3 | c.61delG | p.A21fs | . | . |
| CTC6 | 8 | 61768671 | GC | G | 0.872 | NM_017780 | CHD7 | frameshift_deleti  on | exon33 | c.7075delC | p.Q2359fs | . | . |
| CTC6 | 8 | 62212818 | TG | T | 0.74 | NM_173519 | CLVS1 | frameshift_deleti  on | exon2 | c.433delG | p.A145fs | . | . |
| CTC6 | 8 | 62489376 | TA | T | 0.2 | NM_001164750 | ASPH | frameshift_deleti  on | exon16 | c.1016delT | p.V339fs | . | . |
| CTC6 | 8 | 68992794 | GA | G | 0.25 | NM_024870 | PREX2 | frameshift_deleti  on | exon16 | c.1760delA | p.E587fs | . | . |
| CTC6 | 8 | 73921283 | CGAG | C | 0.4 | NM_003218 | TERF1 | nonframeshift_de  letion | exon1 | c.163_165del | p.55_55del | . | . |
| CTC6 | 8 | 77767130 | CG | C | 0.409 | NM_024721 | ZFHX4 | frameshift_deleti  on | exon10 | c.7974delG | p.A2658fs | . | . |
| CTC6 | 8 | 82355669 | C | CT | 0.4 | NM_002677 | PMP2 | frameshift_inserti  on | exon4 | c.362dupA | p.K121fs | . | . |
| CTC6 | 8 | 82370844 | AT | A | 0.429 | NM_001080526 | FABP9 | frameshift_deleti  on | exon3 | c.340delA | p.M114fs | . | . |
| CTC6 | 8 | 87081849 | CA | C | 0.333 | NM_033126 | PSKH2 | frameshift_deleti  on | exon1 | c.2delT | p.M1fs | . | . |
| CTC6 | 8 | 91090614 | AT | A | 0.727 | NM_004929 | CALB1 | stopgain | exon3 | c.217delA | p.I73X | . | . |
| CTC6 | 8 | 95404000 | CA | C | 0.493 | NM_001205263 | RAD54B | frameshift_deleti on | exon8 | c.1093delT | p.C365fs | . | . |
| CTC6 | 8 | 99560295 | TG | T | 1 | NM_001256313 | STK3 | frameshift_deleti  on | exon7 | c.709delC | p.Q237fs | . | . |
| CTC6 | 8 | 99560297 | GGC | G | 1 | NM_001256313 | STK3 | frameshift_deleti  on | exon7 | c.706_707del | p.A236fs | . | . |
| CTC6 | 8 | 99591945 | CT | C | 0.169 | NM_001256313 | STK3 | frameshift_deleti  on | exon6 | c.561delA | p.K187fs | . | . |
| CTC6 | 8 | 100830013 | GT | G | 0.207 | NM_017890 | VPS13B | frameshift_deleti  on | exon45 | c.8419delT | p.Y2807fs | . | . |
| CTC6 | 8 | 103357675 | CA | C | 0.361 | NM_001282873 | UBR5 | frameshift_deleti  on | exon8 | c.834delT | p.F278fs | . | . |
| CTC6 | 8 | 108264092 | TA | T | 0.333 | NM_001146 | ANGPT1 | stopgain | exon9 | c.1487delT | p.L496X | . | . |
| CTC6 | 8 | 109247248 | AT | A | 0.267 | NM_001568 | EIF3E | frameshift_deleti on | exon5 | c.449delA | p.Y150fs | . | . |
| CTC6 | 8 | 110302036 | TG | T | 0.596 | NM_001128211 | NUDCD1 | frameshift_deleti  on | exon5 | c.679delC | p.Q227fs | . | . |
| CTC6 | 8 | 110530634 | CA | C | 0.273 | NM_177531 | PKHD1L1 | frameshift_deleti  on | exon73 | c.11929delA | p.K3977fs | . | . |
| CTC6 | 8 | 117782589 | AT | A | 0.306 | NM_032334 | UTP23 | frameshift_deleti  on | exon2 | c.348delT | p.Y116fs | . | . |
| CTC6 | 8 | 120744314 | ATGG | A | 0.151 | NM_003184 | TAF2 | nonframeshift_de  letion | exon26 | c.3447_3449del | p.1149_1150de  l | . | . |
| CTC6 | 8 | 124154695 | AG | A | 0.421 | NM_145647 | TBC1D31 | frameshift_deleti  on | exon19 | c.2835delG | p.K945fs | . | . |
| CTC6 | 8 | 125061916 | GC | G | 0.923 | NM_001039112 | FER1L6 | frameshift_deleti  on | exon22 | c.2794delC | p.P932fs | . | . |
| CTC6 | 8 | 125464345 | C | CA | 0.208 | NM_017956 | TRMT12 | frameshift_inserti  on | exon1 | c.1178dupA | p.Q393fs | . | . |
| CTC6 | 8 | 125464493 | GC | G | 0.474 | NM_017956 | TRMT12 | frameshift_deleti  on | exon1 | c.1326delC | p.C442fs | . | . |
| CTC6 | 8 | 125989637 | G | GA | 0.522 | NM_152412 | ZNF572 | frameshift_inserti  on | exon3 | c.1128dupA | p.R376fs | . | . |

| CTC6 | 8 | 126033111 | TG | T | 0.283 | NM_003129 | SQLE | frameshift_deleti  on | exon10 | c.1531delG | p.V511fs | . | . |
| --- | --- | --- | --- | --- | --- | --- | --- | --- | --- | --- | --- | --- | --- |
| CTC6 | 8 | 135622735 | TA | T | 0.367 | NM_001167583 | ZFAT | stopgain | exon4 | c.575delT | p.L192X | . | . |
| CTC6 | 8 | 136659319 | AG | A | 0.3 | NM_006558 | KHDRBS3 | frameshift_deleti on | exon9 | c.1034delG | p.R345fs | . | . |
| CTC6 | 8 | 139164006 | TG | T | 0.917 | NM_015912 | FAM135B | frameshift_deleti  on | exon13 | c.2711delC | p.P904fs | . | . |
| CTC6 | 8 | 139164814 | AG | A | 0.467 | NM_015912 | FAM135B | frameshift_deleti  on | exon13 | c.1903delC | p.L635fs | . | . |
| CTC6 | 8 | 143784581 | CAG | C | 0.417 | NM_001160355 | LY6K | frameshift_deleti  on | exon3 | c.259_260del | p.R87fs | . | . |
| CTC6 | 8 | 144358625 | A | AT | 0.19 | NM_138465 | GLI4 | frameshift_inserti  on | exon4 | c.782_783insT | p.H261fs | . | . |
| CTC6 | 8 | 144522386 | TGAG | T | 0.19 | NM_015117 | ZC3H3 | nonframeshift_de  letion | exon11 | c.2637_2639del | p.879_880del | . | . |
| CTC6 | 8 | 144523240 | CG | C | 0.161 | NM_015117 | ZC3H3 | frameshift_deleti  on | exon10 | c.2357delC | p.A786fs | . | . |
| CTC6 | 8 | 144658978 | ACC | A | 0.769 | NM_001286829 | NAPRT1 | frameshift_deleti  on | exon6 | c.717_718del | p.G239fs | . | . |
| CTC6 | 8 | 144890798 | AC | A | 0.239 | NM_015356 | SCRIB | frameshift_deleti  on | exon15 | c.2095delG | p.V699fs | . | . |
| CTC6 | 8 | 144947133 | TG | T | 0.402 | NM_031308 | EPPK1 | frameshift_deleti  on | exon2 | c.288delC | p.S96fs | . | . |
| CTC6 | 8 | 144996097 | TCC | T | 0.14 | NM_201378 | PLEC | frameshift_deleti  on | exon32 | c.7848_7849del | p.R2616fs | . | . |
| CTC6 | 8 | 145111357 | GC | G | 0.725 | NM_017570 | OPLAH | frameshift_deleti  on | exon14 | c.1913delG | p.G638fs | . | . |
| CTC6 | 8 | 145113751 | GC | G | 0.447 | NM_017570 | OPLAH | frameshift_deleti  on | exon5 | c.511delG | p.A171fs | . | . |
| CTC6 | 8 | 145161805 | GA | G | 0.6 | NM_032272 | MAF1 | frameshift_deleti  on | exon7 | c.689delA | p.E230fs | . | . |
| CTC6 | 8 | 145617534 | TGGGGG  TGCAAG | T | 0.385 | NM_174922 | ADCK5 | frameshift_deleti  on | exon12 | c.1257_1267del | p.L419fs | . | . |
| CTC6 | 8 | 145618058 | CG | C | 1 | NM_174922 | ADCK5 | frameshift_deleti  on | exon14 | c.1589delG | p.R530fs | . | . |
| CTC6 | 8 | 145736930 | AG | A | 0.324 | NM_004260 | RECQL4 | frameshift_deleti  on | exon22 | c.3510delC | p.P1170fs | . | . |
| CTC6 | 8 | 145745178 | G | GC | 0.583 | NM_014665 | LRRC14 | frameshift_inserti  on | exon2 | c.70dupC | p.L23fs | . | . |
| CTC6 | 8 | 146068316 | GT | G | 0.303 | NM_001282797 | ZNF7 | frameshift_deleti  on | exon4 | c.1537delT | p.F513fs | . | . |
| CTC6 | 9 | 386420 | CA | C | 0.605 | NM_001193536 | DOCK8 | frameshift_deleti  on | exon22 | c.2665delA | p.K889fs | . | . |
| CTC6 | 9 | 745207 | GC | G | 0.271 | NM_153186 | KANK1 | frameshift_deleti  on | exon11 | c.3558delC | p.G1186fs | . | . |
| CTC6 | 9 | 6498154 | TA | T | 0.723 | NM_152896 | UHRF2 | frameshift_deleti  on | exon12 | c.1905delA | p.L635fs | . | . |
| CTC6 | 9 | 6506063 | GT | G | 0.455 | NM_152896 | UHRF2 | frameshift_deleti  on | exon16 | c.2294delT | p.V765fs | . | . |
| CTC6 | 9 | 7799594 | GC | G | 0.429 | NM_033428 | TMEM261 | frameshift_deleti  on | exon1 | c.140delG | p.C47fs | . | . |
| CTC6 | 9 | 12775861 | T | TGGCG  GCGGC | 1 | NM_203403 | LURAP1L | nonframeshift_in  sertion | exon1 | c.147_148insG  GCGGCGGC | p.G49delinsGG  GG | . | . |
| CTC6 | 9 | 12775861 | T | TGGCG  GC | 1 | NM_203403 | LURAP1L | nonframeshift_in  sertion | exon1 | c.147_148insG  GCGGC | p.G49delinsGG  G | . | . |
| CTC6 | 9 | 12775864 | C | CGGCG  GT | 0.765 | NM_203403 | LURAP1L | nonframeshift_in  sertion | exon1 | c.150_151insG  GCGGT | p.G50delinsGG  G | . | . |
| CTC6 | 9 | 12775870 | C | CGGCG  GCGGT | 0.879 | NM_203403 | LURAP1L | nonframeshift_in  sertion | exon1 | c.156_157insG  GCGGCGGT | p.G52delinsGG  GG | . | . |
| CTC6 | 9 | 12775871 | G | GGCGG  CGGCA | 0.927 | NM_203403 | LURAP1L | nonframeshift_in  sertion | exon1 | c.157_158insG  CGGCGGCA | p.G53delinsGG  GS | . | . |
| CTC6 | 9 | 12775875 | G | GCGGC  GGCGT | 0.903 | NM_203403 | LURAP1L | nonframeshift_in  sertion | exon1 | c.161_162insC  GGCGGCGT | p.G54delinsGG  GV | . | . |
| CTC6 | 9 | 12775877 | G | GGCGG  CGGCA | 0.873 | NM_203403 | LURAP1L | nonframeshift_in  sertion | exon1 | c.163_164insG  CGGCGGCA | p.G55delinsGG  GS | . | . |
| CTC6 | 9 | 14720347 | TG | T | 0.235 | NM_005454 | CER1 | frameshift_deleti  on | exon2 | c.544delC | p.Q182fs | . | . |
| CTC6 | 9 | 19063091 | TTC | T | 0.2 | NM_001270890 | HAUS6 | frameshift_deleti  on | exon14 | c.1437_1438del | p.K479fs | . | . |
| CTC6 | 9 | 19063092 | TC | T | 0.744 | NM_001270890 | HAUS6 | frameshift_deleti  on | exon14 | c.1437delG | p.K479fs | . | . |
| CTC6 | 9 | 19296163 | CT | C | 0.9 | NM_017925 | DENND4C | frameshift_deleti  on | exon6 | c.960delT | p.P320fs | . | . |
| CTC6 | 9 | 21217262 | TG | T | 0.372 | NM_002173 | IFNA16 | frameshift_deleti  on | exon1 | c.42delC | p.L14fs | . | . |
| CTC6 | 9 | 21333913 | CT | C | 0.97 | NM_018847 | KLHL9 | frameshift_deleti  on | exon1 | c.945delA | p.K315fs | . | . |
| CTC6 | 9 | 21974529 | TG | T | 0.221 | NM_058197 | CDKN2A | frameshift_deleti  on | exon1 | c.297delC | p.H99fs | . | . |
| CTC6 | 9 | 21974549 | GA | G | 0.305 | NM_058197 | CDKN2A | frameshift_deleti  on | exon1 | c.277delT | p.S93fs | . | . |
| CTC6 | 9 | 22451789 | CT | C | 0.769 | NM_022160 | DMRTA1 | frameshift_deleti  on | exon2 | c.1395delT | p.P465fs | . | . |
| CTC6 | 9 | 32488117 | TA | T | 0.474 | NM_014314 | DDX58 | stopgain | exon8 | c.1037delT | p.L346X | . | . |
| CTC6 | 9 | 32488815 | CT | C | 0.441 | NM_014314 | DDX58 | frameshift_deleti on | exon7 | c.869delA | p.K290fs | . | . |
| CTC6 | 9 | 32784558 | AG | A | 0.571 | NM_212558 | TMEM215 | frameshift_deleti  on | exon2 | c.378delG | p.Q126fs | . | . |
| CTC6 | 9 | 33294985 | CAA | C | 0.529 | NM_002504 | NFX1 | frameshift_deleti  on | exon2 | c.594_595del | p.P198fs | . | . |
| CTC6 | 9 | 33467732 | GC | G | 0.5 | NM_022917 | NOL6 | frameshift_deleti  on | exon12 | c.1558delG | p.A520fs | . | . |
| CTC6 | 9 | 34385712 | AC | A | 0.205 | NM_032596 | C9orf24 | frameshift_deleti  on | exon2 | c.202delG | p.V68fs | . | . |
| CTC6 | 9 | 35090071 | C | CA | 0.257 | NM_032634 | PIGO | frameshift_inserti  on | exon9 | c.3060dupT | p.G1021fs | . | . |
| CTC6 | 9 | 35740592 | CA | C | 0.903 | NM_020944 | GBA2 | frameshift_deleti  on | exon6 | c.1059delT | p.F353fs | . | . |
| CTC6 | 9 | 35853625 | C | CA | 0.222 | NM_001042590 | TMEM8B | frameshift_inserti  on | exon13 | c.1207_1208ins  A | p.R403fs | . | . |
| CTC6 | 9 | 35906347 | CCTG | C | 0.165 | NM_001039792 | HRCT1 | nonframeshift_de  letion | exon1 | c.64_66del | p.22_22del | . | . |
| CTC6 | 9 | 36236903 | A | AT | 0.594 | NM_001190388 | GNE | frameshift_inserti  on | exon3 | c.679dupA | p.M227fs | . | . |
| CTC6 | 9 | 37354804 | TA | T | 0.778 | NM_001289119 | ZCCHC7 | frameshift_deleti  on | exon8 | c.1182delA | p.L394fs | . | . |
| CTC6 | 9 | 71152336 | CCT | C | 0.321 | NM_153237 | TMEM252 | frameshift_deleti  on | exon2 | c.350_351del | p.E117fs | . | . |
| CTC6 | 9 | 71395514 | C | CA | 0.22 | NM_138333 | FAM122A | frameshift_inserti  on | exon1 | c.435dupA | p.A145fs | . | . |
| CTC6 | 9 | 72833404 | GA | G | 0.647 | NM_153267 | MAMDC2 | frameshift_deleti  on | exon12 | c.1804delA | p.K602fs | . | . |

| CTC6 | 9 | 79117342 | CA | C | 0.5 | NM_001097636 | GCNT1 | frameshift_deleti  on | exon2 | c.46delA | p.K16fs | . | . |
| --- | --- | --- | --- | --- | --- | --- | --- | --- | --- | --- | --- | --- | --- |
| CTC6 | 9 | 79267550 | CTTGA | C | 0.16 | NM_015225 | PRUNE2 | frameshift_deleti  on | exon11 | c.8402_8405del | p.I2801fs | . | . |
| CTC6 | 9 | 79318375 | GGTGAC  AGCCTG | G | 1 | NM_015225 | PRUNE2 | nonframeshift_de  letion | exon9 | c.8139_8153del | p.2713_2718de  l | . | . |
| CTC6 | 9 | 79318378 | GACAGC  CTGCAA | G | 1 | NM_015225 | PRUNE2 | nonframeshift_de  letion | exon9 | c.8136_8150del | p.2712_2717de  l | . | . |
| CTC6 | 9 | 79322474 | GT | G | 0.348 | NM_015225 | PRUNE2 | frameshift_deleti  on | exon8 | c.4715delA | p.N1572fs | . | . |
| CTC6 | 9 | 79323774 | T | TC | 1 | NM_015225 | PRUNE2 | frameshift_inserti  on | exon8 | c.3415dupG | p.D1139fs | . | . |
| CTC6 | 9 | 79324135 | A | ATGAC  TGTTGC | 1 | NM_015225 | PRUNE2 | nonframeshift_in  sertion | exon8 | c.3054_3055ins  CTGCAACAGT | p.S1019delinsL  QQSS | . | . |
| CTC6 | 9 | 84610094 | GA | G | 0.545 | NM_001001670 | SPATA31D1 | frameshift_deleti  on | exon4 | c.4710delA | p.G1570fs | . | . |
| CTC6 | 9 | 86452154 | GT | G | 0.75 | NM_001271928 | KIF27 | frameshift_deleti  on | exon16 | c.3676delA | p.T1226fs | . | . |
| CTC6 | 9 | 86457280 | G | GT | 0.5 | NM_001271928 | KIF27 | frameshift_inserti  on | exon15 | c.3301dupA | p.T1101fs | . | . |
| CTC6 | 9 | 86893134 | CA | C | 0.829 | NM_001199633 | SLC28A3 | stoploss | exon18 | c.2074delT | p.X692E | . | . |
| CTC6 | 9 | 90538330 | GT | G | 0.444 | NM_001145124 | SPATA31C1 | frameshift_deleti on | exon4 | c.3509delT | p.V1170fs | . | . |
| CTC6 | 9 | 92002413 | TG | T | 0.311 | NM_001142287 | SEMA4D | frameshift_deleti  on | exon14 | c.1217delC | p.P406fs | . | . |
| CTC6 | 9 | 93640001 | TG | T | 0.286 | NM_001135052 | SYK | stopgain | exon9 | c.1262delG | p.W421X | . | . |
| CTC6 | 9 | 95030495 | GC | G | 0.5 | NM_002161 | IARS | frameshift_deleti on | exon14 | c.1391delG | p.G464fs | . | . |
| CTC6 | 9 | 95237024 | CTCA | C | 1 | NM_001193335 | ASPN | nonframeshift_de  letion | exon2 | c.153_155del | p.51_52del | . | . |
| CTC6 | 9 | 96051444 | GC | G | 0.281 | NM_006648 | WNK2 | frameshift_deleti  on | exon19 | c.4409delC | p.A1470fs | . | . |
| CTC6 | 9 | 96291736 | C | CA | 0.2 | NM_001286722 | FAM120A | frameshift_inserti  on | exon9 | c.1606dupA | p.P535fs | . | . |
| CTC6 | 9 | 96294525 | A | AT | 0.643 | NM_001286722 | FAM120A | frameshift_inserti  on | exon10 | c.1821dupT | p.Y607fs | . | . |
| CTC6 | 9 | 97080944 | GAGA | G | 1 | NM_017561 | NUTM2F | nonframeshift_de  letion | exon7 | c.2071_2073del | p.691_691del | . | . |
| CTC6 | 9 | 99008854 | T | TC | 0.25 | NM_000197 | HSD17B3 | frameshift_inserti  on | exon7 | c.507dupG | p.K170fs | . | . |
| CTC6 | 9 | 100076708 | C | CTTT | 0.75 | NM_020893 | CCDC180 | nonframeshift_in  sertion | exon6 | c.625_626insTT  T | p.L209delinsLF | . | . |
| CTC6 | 9 | 100092858 | G | GA | 0.714 | NM_020893 | CCDC180 | frameshift_inserti  on | exon18 | c.2216dupA | p.E739fs | . | . |
| CTC6 | 9 | 100092968 | A | AGAGG  AG | 1 | NM_020893 | CCDC180 | nonframeshift_in  sertion | exon18 | c.2325_2326ins  GAGGAG | p.E775delinsEE  E | . | . |
| CTC6 | 9 | 100092968 | A | AGAG | 1 | NM_020893 | CCDC180 | nonframeshift_in  sertion | exon18 | c.2325_2326ins  GAG | p.E775delinsEE | . | . |
| CTC6 | 9 | 100222837 | CA | C | 0.3 | NM_014290 | TDRD7 | frameshift_deleti  on | exon7 | c.1234delA | p.K412fs | . | . |
| CTC6 | 9 | 100872248 | AAC | A | 0.25 | NM_014788 | TRIM14 | frameshift_deleti  on | exon2 | c.224_225del | p.C75fs | . | . |
| CTC6 | 9 | 101829268 | A | AT | 0.304 | NM_001855 | COL15A1 | frameshift_inserti  on | exon40 | c.3757dupT | p.A1252fs | . | . |
| CTC6 | 9 | 102590615 | TCAC | T | 0.333 | NM_006981 | NR4A3 | nonframeshift_de  letion | exon3 | c.292_294del | p.98_98del | . | . |
| CTC6 | 9 | 103015383 | GA | G | 0.593 | NM_014425 | INVS | frameshift_deleti  on | exon10 | c.1430delA | p.E477fs | . | . |
| CTC6 | 9 | 107550842 | TA | T | 0.267 | NM_005502 | ABCA1 | frameshift_deleti  on | exon45 | c.5933delT | p.L1978fs | . | . |
| CTC6 | 9 | 109689385 | GA | G | 0.5 | NM_021224 | ZNF462 | frameshift_deleti  on | exon3 | c.3193delA | p.K1065fs | . | . |
| CTC6 | 9 | 112145781 | GC | G | 0.333 | NM_001145372 | PTPN3 | frameshift_deleti  on | exon12 | c.1307delG | p.G436fs | . | . |
| CTC6 | 9 | 112900341 | G | GGAAG  CT | 0.374 | NM_001004065 | AKAP2 | nonframeshift_in  sertion | exon2 | c.2091_2092ins  GAAGCT | p.E697delinsEE  A | . | . |
| CTC6 | 9 | 112900345 | G | GCTGA  AT | 0.315 | NM_001004065 | AKAP2 | nonframeshift_in  sertion | exon2 | c.2095_2096ins  CTGAAT | p.A699delinsAE  S | . | . |
| CTC6 | 9 | 114134080 | GC | G | 0.267 | NM_001080398 | KIAA0368 | frameshift_deleti  on | exon44 | c.5091delG | p.L1697fs | . | . |
| CTC6 | 9 | 114155137 | CT | C | 0.261 | NM_001080398 | KIAA0368 | frameshift_deleti  on | exon29 | c.3536delA | p.K1179fs | . | . |
| CTC6 | 9 | 114503776 | A | AT | 0.308 | NM_001080551 | C9orf84 | frameshift_inserti  on | exon7 | c.844_845insA | p.L282fs | . | . |
| CTC6 | 9 | 115969555 | GT | G | 0.733 | NM_015258 | FKBP15 | frameshift_deleti  on | exon3 | c.190delA | p.T64fs | . | . |
| CTC6 | 9 | 116857509 | GC | G | 0.833 | NM_138424 | KIF12 | frameshift_deleti  on | exon7 | c.584delG | p.R195fs | . | . |
| CTC6 | 9 | 117844201 | CT | C | 0.6 | NM_002160 | TNC | frameshift_deleti  on | exon6 | c.2253delA | p.K751fs | . | . |
| CTC6 | 9 | 123722612 | TC | T | 0.8 | NM_001735 | C5 | frameshift_deleti  on | exon38 | c.4591delG | p.D1531fs | . | . |
| CTC6 | 9 | 124535135 | CG | C | 1 | NM_138709 | DAB2IP | frameshift_deleti  on | exon10 | c.1957delG | p.A653fs | . | . |
| CTC6 | 9 | 125033269 | AG | A | 0.6 | NM_199177 | MRRF | frameshift_deleti  on | exon1 | c.100delG | p.V34fs | . | . |
| CTC6 | 9 | 125391770 | C | CA | 0.593 | NM_001004450 | OR1B1 | frameshift_inserti  on | exon1 | c.44dupT | p.L15fs | . | . |
| CTC6 | 9 | 125864004 | AC | A | 0.417 | NM_012197 | RABGAP1 | frameshift_deleti  on | exon25 | c.3050delC | p.T1017fs | . | . |
| CTC6 | 9 | 127734086 | C | CAA | 0.615 | NM_001144877 | SCAI | frameshift_inserti  on | exon16 | c.1436_1437ins  TT | p.L479fs | . | . |
| CTC6 | 9 | 127920521 | AT | A | 0.175 | NM_001123369 | PPP6C | frameshift_deleti  on | exon3 | c.311delA | p.Y104fs | . | . |
| CTC6 | 9 | 129642903 | TG | T | 0.18 | NM_001099270 | ZBTB34 | frameshift_deleti  on | exon2 | c.1214delG | p.C405fs | . | . |
| CTC6 | 9 | 130263417 | CG | C | 1 | NM_001005374 | LRSAM1 | frameshift_deleti  on | exon24 | c.2042delG | p.R681fs | . | . |
| CTC6 | 9 | 130507007 | TG | T | 0.393 | NM_001142533 | SH2D3C | frameshift_deleti  on | exon5 | c.1161delC | p.P387fs | . | . |
| CTC6 | 9 | 130687528 | TC | T | 0.857 | NM_173492 | PIP5KL1 | frameshift_deleti  on | exon4 | c.165delG | p.R55fs | . | . |
| CTC6 | 9 | 130828937 | TG | T | 0.6 | NM_197956 | NAIF1 | frameshift_deleti  on | exon1 | c.443delC | p.T148fs | . | . |
| CTC6 | 9 | 131046854 | G | GA | 0.444 | NM_001040011 | SWI5 | frameshift_inserti  on | exon3 | c.493dupA | p.Q164fs | . | . |
| CTC6 | 9 | 131256797 | G | GA | 0.233 | NM_001242354 | ODF2 | frameshift_inserti  on | exon16 | c.1518_1519ins  A | p.R506fs | . | . |
| CTC6 | 9 | 131388727 | GA | G | 0.621 | NM_001195532 | SPTAN1 | frameshift_deleti  on | exon47 | c.6263delA | p.E2088fs | . | . |
| CTC6 | 9 | 131476523 | TG | T | 0.667 | NM_013355 | PKN3 | frameshift_deleti  on | exon11 | c.1361delG | p.W454fs | . | . |

| CTC6 | 9 | 131708397 | GA | G | 0.9 | NM_014908 | DOLK | frameshift_deleti  on | exon1 | c.1185delT | p.F395fs | . | . |
| --- | --- | --- | --- | --- | --- | --- | --- | --- | --- | --- | --- | --- | --- |
| CTC6 | 9 | 132571742 | TG | T | 0.474 | NM_014506 | TOR1B | frameshift_deleti  on | exon5 | c.892delG | p.A298fs | . | . |
| CTC6 | 9 | 134393915 | TG | T | 0.947 | NM_001136114 | POMT1 | frameshift_deleti  on | exon12 | c.1006delG | p.V336fs | . | . |
| CTC6 | 9 | 135203910 | TTCA | T | 0.286 | NM_015046 | SETX | nonframeshift_de  letion | exon10 | c.3072_3074del | p.1024_1025de  l | . | . |
| CTC6 | 9 | 136202963 | AG | A | 0.385 | NM_001278942 | SURF6 | frameshift_deleti  on | exon1 | c.6delC | p.A2fs | . | . |
| CTC6 | 9 | 136307648 | TG | T | 0.409 | NM_139025 | ADAMTS13 | frameshift_deleti  on | exon17 | c.2098delG | p.G700fs | . | . |
| CTC6 | 9 | 136404952 | CG | C | 0.2 | NM_001145320 | ADAMTSL2 | frameshift_deleti  on | exon5 | c.370delG | p.V124fs | . | . |
| CTC6 | 9 | 136550393 | C | CT | 0.3 | NM_001134707 | SARDH | frameshift_inserti  on | exon17 | c.2084dupA | p.Q695fs | . | . |
| CTC6 | 9 | 139277994 | CGCTGC  TGCT | C | 0.9 | NM_003086 | SNAPC4 | nonframeshift_de  letion | exon15 | c.1618_1626del | p.540_542del | . | . |
| CTC6 | 9 | 139369456 | CT | C | 0.37 | NM_001276418 | SEC16A | frameshift_deleti  on | exon2 | c.2611delA | p.S871fs | . | . |
| CTC6 | 9 | 139744522 | GTC | G | 1 | NM_001135861 | PHPT1 | frameshift_deleti  on | exon2 | c.219_220del | p.C73fs | . | . |
| CTC6 | 9 | 139744531 | G | GC | 0.138 | NM_001135861 | PHPT1 | frameshift_inserti  on | exon2 | c.228dupC | p.G76fs | . | . |
| CTC6 | 9 | 139748343 | TC | T | 0.267 | NM_206920 | MAMDC4 | frameshift_deleti  on | exon5 | c.570delC | p.I190fs | . | . |
| CTC6 | 9 | 139878942 | GC | G | 0.833 | NM_207510 | LCNL1 | frameshift_deleti  on | exon2 | c.124delC | p.P42fs | . | . |
| CTC6 | 9 | 139914917 | GC | G | 1 | NM_001606 | ABCA2 | frameshift_deleti  on | exon10 | c.1295delG | p.G432fs | . | . |
| CTC6 | 9 | 139929237 | C | CG | 0.2 | NM_207511 | C9orf139 | frameshift_inserti  on | exon3 | c.305dupG | p.R102fs | . | . |
| CTC6 | 9 | 140437129 | GC | G | 0.286 | NM_152286 | PNPLA7 | frameshift_deleti  on | exon6 | c.555delG | p.V185fs | . | . |
| CTC6 | 9 | 140508813 | TG | T | 0.889 | NM_152285 | ARRDC1 | frameshift_deleti  on | exon6 | c.682delG | p.A228fs | . | . |
| CTC6 | 10 | 395354 | TTG | T | 0.582 | NM_014974 | DIP2C | frameshift_deleti  on | exon25 | c.3024_3025del | p.H1008fs | . | . |
| CTC6 | 10 | 395357 | TGC | T | 0.27 | NM_014974 | DIP2C | frameshift_deleti  on | exon25 | c.3021_3022del | p.L1007fs | . | . |
| CTC6 | 10 | 1118148 | GC | G | 0.664 | NM_014023 | WDR37 | frameshift_deleti  on | exon2 | c.54delC | p.R18fs | . | . |
| CTC6 | 10 | 5415732 | GGC | G | 0.5 | NM_053049 | UCN3 | frameshift_deleti  on | exon2 | c.50_51del | p.G17fs | . | . |
| CTC6 | 10 | 5415733 | GCC | G | 0.714 | NM_053049 | UCN3 | frameshift_deleti  on | exon2 | c.51_52del | p.G17fs | . | . |
| CTC6 | 10 | 5924997 | TG | T | 0.292 | NM_001009941 | ANKRD16 | frameshift_deleti  on | exon5 | c.820delC | p.H274fs | . | . |
| CTC6 | 10 | 7755230 | AG | A | 0.24 | NM_002216 | ITIH2 | frameshift_deleti  on | exon5 | c.450delG | p.K150fs | . | . |
| CTC6 | 10 | 8115770 | CA | C | 0.284 | NM_001002295 | GATA3 | frameshift_deleti  on | exon6 | c.1120delA | p.K374fs | . | . |
| CTC6 | 10 | 11504502 | GC | G | 0.238 | NM_001080491 | USP6NL | frameshift_deleti  on | exon14 | c.2475delG | p.P825fs | . | . |
| CTC6 | 10 | 11990458 | AT | A | 0.297 | NM_015542 | UPF2 | frameshift_deleti  on | exon16 | c.3083delA | p.N1028fs | . | . |
| CTC6 | 10 | 13534803 | TC | T | 0.375 | NM_001100912 | BEND7 | frameshift_deleti  on | exon4 | c.527delG | p.R176fs | . | . |
| CTC6 | 10 | 15145336 | CG | C | 0.846 | NM_001265601 | RPP38 | frameshift_deleti  on | exon2 | c.24delG | p.P8fs | . | . |
| CTC6 | 10 | 15255336 | AT | A | 0.3 | NM_001010924 | FAM171A1 | frameshift_deleti  on | exon8 | c.2250delA | p.K750fs | . | . |
| CTC6 | 10 | 26409666 | C | CT | 0.237 | NM_017433 | MYO3A | frameshift_inserti  on | exon18 | c.1839dupT | p.S613fs | . | . |
| CTC6 | 10 | 26463098 | AAAAAG | A | 0.933 | NM_017433 | MYO3A | frameshift_deleti  on | exon30 | c.3906_3910del | p.E1302fs | . | . |
| CTC6 | 10 | 27687501 | GT | G | 0.828 | NM_001034842 | PTCHD3 | frameshift_deleti  on | exon4 | c.2025delA | p.K675fs | . | . |
| CTC6 | 10 | 30317264 | GC | G | 0.184 | NM_020848 | KIAA1462 | frameshift_deleti  on | exon3 | c.1812delG | p.K604fs | . | . |
| CTC6 | 10 | 31138145 | G | GA | 0.833 | NM_001143767 | ZNF438 | frameshift_inserti  on | exon5 | c.1188dupT | p.Q397fs | . | . |
| CTC6 | 10 | 31816009 | AGAG | A | 0.222 | NM_001174093 | ZEB1 | nonframeshift_de  letion | exon8 | c.3133_3135del | p.1045_1045de  l | . | . |
| CTC6 | 10 | 32106806 | AG | A | 0.286 | NM_001270699 | ARHGAP12 | frameshift_deleti  on | exon9 | c.1484delC | p.T495fs | . | . |
| CTC6 | 10 | 35818929 | AT | A | 0.324 | NM_001282854 | CCNY | frameshift_deleti  on | exon4 | c.32delT | p.I11fs | . | . |
| CTC6 | 10 | 43089555 | C | CT | 0.183 | NM_006955 | ZNF33B | frameshift_inserti  on | exon5 | c.842dupA | p.E281fs | . | . |
| CTC6 | 10 | 43287148 | GTT | G | 0.129 | NM_014753 | BMS1 | frameshift_deleti  on | exon6 | c.711_712del | p.R237fs | . | . |
| CTC6 | 10 | 43597921 | TG | T | 0.722 | NM_020630 | RET | frameshift_deleti  on | exon3 | c.470delG | p.C157fs | . | . |
| CTC6 | 10 | 43651045 | AG | A | 0.275 | NM_018590 | CSGALNACT  2 | frameshift_deleti  on | exon2 | c.449delG | p.S150fs | . | . |
| CTC6 | 10 | 45936010 | AC | A | 0.4 | NM_000698 | ALOX5 | frameshift_deleti  on | exon8 | c.1115delC | p.T372fs | . | . |
| CTC6 | 10 | 46999591 | C | CATGA  GGGAG | 0.944 | NM_014696 | GPRIN2 | nonframeshift_in  sertion | exon3 | c.711_712insAT  GAGGGAG | p.G237delinsG  MRE | . | . |
| CTC6 | 10 | 47658540 | CCTG | C | 0.1 | NM_001278688 | ANTXRL | nonframeshift_de  letion | exon1 | c.43_45del | p.15_15del | . | . |
| CTC6 | 10 | 49381042 | GA | G | 0.28 | NM_001042512 | FRMPD2 | frameshift_deleti  on | exon2 | c.202delT | p.S68fs | . | . |
| CTC6 | 10 | 50018590 | GT | G | 1 | NM_020945 | WDFY4 | frameshift_deleti  on | exon29 | c.4855delT | p.F1619fs | . | . |
| CTC6 | 10 | 50151374 | GA | G | 0.478 | NM_020945 | WDFY4 | frameshift_deleti  on | exon49 | c.7630delA | p.K2544fs | . | . |
| CTC6 | 10 | 50178274 | GGT | G | 0.474 | NM_020945 | WDFY4 | frameshift_deleti  on | exon57 | c.8869_8870del | p.V2957fs | . | . |
| CTC6 | 10 | 51562393 | TA | T | 0.2 | NM_002443 | MSMB | frameshift_deleti  on | exon4 | c.339delA | p.I113fs | . | . |
| CTC6 | 10 | 51581326 | AC | A | 0.3 | NM_001145262 | NCOA4 | frameshift_deleti  on | exon5 | c.429delC | p.D143fs | . | . |
| CTC6 | 10 | 63188854 | TG | T | 0.417 | NM_178505 | TMEM26 | frameshift_deleti  on | exon4 | c.434delC | p.T145fs | . | . |
| CTC6 | 10 | 64573035 | TG | T | 0.467 | NM_000399 | EGR2 | stopgain | exon2 | c.1362delC | p.C454X | . | . |
| CTC6 | 10 | 64573486 | G | GGCT | 0.667 | NM_000399 | EGR2 | nonframeshift_in sertion | exon2 | c.911_912insA GC | p.A304delinsA A | . | . |
| CTC6 | 10 | 64957317 | AT | A | 0.476 | NM_001282948 | JMJD1C | frameshift_deleti  on | exon12 | c.4951delA | p.I1651fs | . | . |
| CTC6 | 10 | 64966752 | TG | T | 0.821 | NM_001282948 | JMJD1C | stopgain | exon9 | c.4130delC | p.S1377X | . | . |

| CTC6 | 10 | 64967950 | ACTAAA  C | A | 0.429 | NM_001282948 | JMJD1C | nonframeshift_de  letion | exon9 | c.2927_2932del | p.976_978del | . | . |
| --- | --- | --- | --- | --- | --- | --- | --- | --- | --- | --- | --- | --- | --- |
| CTC6 | 10 | 68687257 | AG | A | 0.706 | NM_178011 | LRRTM3 | frameshift_deleti  on | exon2 | c.584delG | p.S195fs | . | . |
| CTC6 | 10 | 69565465 | AT | A | 1 | NM_021800 | DNAJC12 | frameshift_deleti  on | exon4 | c.377delA | p.N126fs | . | . |
| CTC6 | 10 | 70097713 | GA | G | 0.262 | NM_012207 | HNRNPH3 | frameshift_deleti  on | exon3 | c.212delA | p.E71fs | . | . |
| CTC6 | 10 | 72498740 | AC | A | 0.636 | NM_080722 | ADAMTS14 | frameshift_deleti  on | exon11 | c.1743delC | p.N581fs | . | . |
| CTC6 | 10 | 73450238 | G | GT | 0.412 | NM_001171930 | CDH23 | frameshift_inserti  on | exon19 | c.2074dupT | p.L691fs | . | . |
| CTC6 | 10 | 73476016 | CGG | C | 0.161 | NM_001164375 | C10orf105 | frameshift_deleti  on | exon2 | c.77_78del | p.P26fs | . | . |
| CTC6 | 10 | 73574785 | C | CT | 0.462 | NM_001171936 | CDH23 | frameshift_inserti  on | exon4 | c.401_402insT | p.P134fs | . | . |
| CTC6 | 10 | 75258658 | GA | G | 0.3 | NM_152586 | USP54 | frameshift_deleti  on | exon22 | c.4783delT | p.S1595fs | . | . |
| CTC6 | 10 | 75276468 | TG | T | 0.3 | NM_152586 | USP54 | frameshift_deleti  on | exon18 | c.3715delC | p.Q1239fs | . | . |
| CTC6 | 10 | 76781905 | GGAA | G | 0.808 | NM_001256468 | KAT6B | nonframeshift_de  letion | exon16 | c.2740_2742del | p.914_914del | . | . |
| CTC6 | 10 | 77542773 | AAT | A | 0.188 | NM_032024 | C10orf11 | frameshift_deleti  on | exon1 | c.41_42del | p.N14fs | . | . |
| CTC6 | 10 | 85968044 | C | CA | 0.36 | NM_001171971 | CDHR1 | frameshift_inserti  on | exon11 | c.1079dupA | p.Q360fs | . | . |
| CTC6 | 10 | 86131868 | GT | G | 0.264 | NM_001284240 | CCSER2 | frameshift_deleti  on | exon2 | c.1061delT | p.V354fs | . | . |
| CTC6 | 10 | 86131963 | T | TA | 0.407 | NM_001284240 | CCSER2 | frameshift_inserti  on | exon2 | c.1156dupA | p.A385fs | . | . |
| CTC6 | 10 | 88466433 | GC | G | 0.691 | NM_007078 | LDB3 | frameshift_deleti  on | exon7 | c.1043delC | p.A348fs | . | . |
| CTC6 | 10 | 88911877 | CA | C | 0.5 | NM_019054 | FAM35A | frameshift_deleti  on | exon3 | c.767delA | p.Q256fs | . | . |
| CTC6 | 10 | 89621769 | TC | T | 0.667 | NM_001126049 | KLLN | frameshift_deleti  on | exon1 | c.475delG | p.E159fs | . | . |
| CTC6 | 10 | 90492288 | C | CT | 0.37 | NM_001080518 | LIPK | frameshift_inserti  on | exon5 | c.650dupT | p.L217fs | . | . |
| CTC6 | 10 | 90984980 | TA | T | 0.278 | NM_001288979 | LIPA | frameshift_deleti  on | exon4 | c.195delT | p.F65fs | . | . |
| CTC6 | 10 | 91469716 | CT | C | 1 | NM_001284259 | KIF20B | frameshift_deleti  on | exon5 | c.385delT | p.F129fs | . | . |
| CTC6 | 10 | 93773744 | TA | T | 0.471 | NM_003972 | BTAF1 | frameshift_deleti  on | exon32 | c.4543delA | p.K1515fs | . | . |
| CTC6 | 10 | 94110911 | T | TC | 0.667 | NM_017824 | 5-Mar | frameshift_inserti  on | exon6 | c.784_785insC | p.L262fs | . | . |
| CTC6 | 10 | 98376411 | A | AT | 0.318 | NM_152309 | PIK3AP1 | frameshift_inserti  on | exon13 | c.1998dupA | p.S667fs | . | . |
| CTC6 | 10 | 99211901 | CTG | C | 0.3 | NM_001287804 | ZDHHC16 | frameshift_deleti  on | exon3 | c.299_300del | p.L100fs | . | . |
| CTC6 | 10 | 100010859 | A | AT | 0.737 | NM_032211 | LOXL4 | stopgain | exon14 | c.2162dupA | p.Y721_D722de  linsX | . | . |
| CTC6 | 10 | 101657940 | TG | T | 0.579 | NM_015221 | DNMBP | frameshift_deleti  on | exon9 | c.2822delC | p.P941fs | . | . |
| CTC6 | 10 | 102676810 | CCAGG | C | 0.182 | NM_001136123 | FAM178A | frameshift_deleti  on | exon3 | c.669_672del | p.S223fs | . | . |
| CTC6 | 10 | 103827294 | TC | T | 0.4 | NM_024747 | HPS6 | frameshift_deleti  on | exon1 | c.2064delC | p.L688fs | . | . |
| CTC6 | 10 | 104103844 | CT | C | 0.646 | NM_001199378 | GBF1 | frameshift_deleti  on | exon4 | c.201delT | p.P67fs | . | . |
| CTC6 | 10 | 105048270 | AGAG | A | 0.164 | NM_032727 | INA | nonframeshift_de  letion | exon3 | c.1345_1347del | p.449_449del | . | . |
| CTC6 | 10 | 105178327 | AT | A | 0.216 | NM_014976 | PDCD11 | frameshift_deleti  on | exon15 | c.2043delT | p.H681fs | . | . |
| CTC6 | 10 | 105238594 | AG | A | 0.32 | NM_001129742 | CALHM3 | frameshift_deleti  on | exon1 | c.195delC | p.L65fs | . | . |
| CTC6 | 10 | 105778031 | GC | G | 1 | NM_014720 | SLK | frameshift_deleti  on | exon14 | c.2992delC | p.Q998fs | . | . |
| CTC6 | 10 | 105778034 | AC | A | 1 | NM_014720 | SLK | frameshift_deleti  on | exon14 | c.2995delC | p.Q999fs | . | . |
| CTC6 | 10 | 105812855 | GC | G | 0.562 | NM_000494 | COL17A1 | frameshift_deleti  on | exon23 | c.1872delG | p.G624fs | . | . |
| CTC6 | 10 | 105837254 | GT | G | 0.286 | NM_000494 | COL17A1 | frameshift_deleti  on | exon4 | c.127delA | p.T43fs | . | . |
| CTC6 | 10 | 105938853 | CT | C | 0.667 | NM_025145 | WDR96 | frameshift_deleti  on | exon19 | c.2393delA | p.K798fs | . | . |
| CTC6 | 10 | 106927052 | TG | T | 0.455 | NM_014978 | SORCS3 | frameshift_deleti  on | exon13 | c.1847delG | p.W616fs | . | . |
| CTC6 | 10 | 108489838 | C | CA | 0.278 | NM_001013031 | SORCS1 | frameshift_inserti  on | exon6 | c.993dupT | p.V332fs | . | . |
| CTC6 | 10 | 115489122 | CT | C | 0.36 | NM_001267058 | CASP7 | frameshift_deleti  on | exon6 | c.661delT | p.C221fs | . | . |
| CTC6 | 10 | 115644124 | GT | G | 0.6 | NM_198514 | NHLRC2 | frameshift_deleti  on | exon5 | c.1025delT | p.V342fs | . | . |
| CTC6 | 10 | 115947677 | GA | G | 0.835 | NM_198795 | TDRD1 | frameshift_deleti  on | exon2 | c.88delA | p.K30fs | . | . |
| CTC6 | 10 | 116085659 | TC | T | 0.32 | NM_001287824 | AFAP1L2 | frameshift_deleti  on | exon5 | c.455delG | p.G152fs | . | . |
| CTC6 | 10 | 116085784 | C | CCCG | 1 | NM_001287824 | AFAP1L2 | nonframeshift_in  sertion | exon5 | c.330_331insC  GG | p.G111delinsR  G | . | . |
| CTC6 | 10 | 121691736 | TG | T | 0.236 | NM_007190 | SEC23IP | frameshift_deleti  on | exon16 | c.2665delG | p.A889fs | . | . |
| CTC6 | 10 | 123846803 | TG | T | 0.429 | NM_206862 | TACC2 | frameshift_deleti  on | exon4 | c.4789delG | p.G1597fs | . | . |
| CTC6 | 10 | 127413977 | C | CA | 0.957 | NM_001202438 | EDRF1 | frameshift_inserti  on | exon5 | c.602dupA | p.Q201fs | . | . |
| CTC6 | 10 | 127548493 | CA | C | 1 | NM_018180 | DHX32 | frameshift_deleti  on | exon3 | c.527delT | p.L176fs | . | . |
| CTC6 | 10 | 128974027 | GT | G | 0.5 | NM_001039762 | FAM196A | frameshift_deleti  on | exon4 | c.632delA | p.N211fs | . | . |
| CTC6 | 10 | 135367810 | ACTGGT  CTAC | A | 0.9 | NM_001143763 | SYCE1 | nonframeshift_de  letion | exon13 | c.932_940del | p.311_314del | . | . |
| CTC6 | 11 | 373460 | GTGGAC  AGCGCC | G | 0.473 | NM_178537 | B4GALNT4 | frameshift_deleti  on | exon7 | c.649_659del | p.W217fs | . | . |
| CTC6 | 11 | 379621 | GC | G | 0.278 | NM_178537 | B4GALNT4 | frameshift_deleti  on | exon15 | c.2409delC | p.R803fs | . | . |
| CTC6 | 11 | 803491 | TC | T | 0.534 | NM_145886 | PIDD | frameshift_deleti  on | exon3 | c.391delG | p.D131fs | . | . |
| CTC6 | 11 | 900953 | GC | G | 0.226 | NM_001142675 | CHID1 | frameshift_deleti  on | exon5 | c.421delG | p.A141fs | . | . |
| CTC6 | 11 | 1782557 | CA | C | 0.5 | NM_001909 | CTSD | frameshift_deleti  on | exon2 | c.209delT | p.V70fs | . | . |
| CTC6 | 11 | 2439529 | GCC | G | 1 | NM_014555 | TRPM5 | frameshift_deleti  on | exon6 | c.772_773del | p.G258fs | . | . |

| CTC6 | 11 | 2981047 | CT | C | 0.292 | NM_005969 | NAP1L4 | frameshift_deleti  on | exon9 | c.698delA | p.K233fs | . | . |
| --- | --- | --- | --- | --- | --- | --- | --- | --- | --- | --- | --- | --- | --- |
| CTC6 | 11 | 2997269 | GA | G | 0.6 | NM_005969 | NAP1L4 | frameshift_deleti  on | exon4 | c.157delT | p.S53fs | . | . |
| CTC6 | 11 | 3381418 | AT | A | 0.75 | NM_007152 | ZNF195 | frameshift_deleti  on | exon4 | c.603delA | p.K201fs | . | . |
| CTC6 | 11 | 4661534 | CT | C | 0.667 | NM_001004751 | OR51D1 | frameshift_deleti  on | exon1 | c.515delT | p.L172fs | . | . |
| CTC6 | 11 | 4936171 | CT | C | 0.786 | NM_001005238 | OR51G2 | frameshift_deleti  on | exon1 | c.722delA | p.K241fs | . | . |
| CTC6 | 11 | 5809803 | TG | T | 0.438 | NM_001001913 | OR52N1 | frameshift_deleti  on | exon1 | c.243delC | p.P81fs | . | . |
| CTC6 | 11 | 6416896 | C | CT | 0.353 | NM_001257319 | APBB1 | frameshift_inserti  on | exon13 | c.1340dupA | p.Q447fs | . | . |
| CTC6 | 11 | 6424924 | CG | C | 0.385 | NM_001257319 | APBB1 | frameshift_deleti  on | exon2 | c.189delC | p.P63fs | . | . |
| CTC6 | 11 | 6432089 | ATCC | A | 0.4 | NM_001164 | APBB1 | nonframeshift_de  letion | exon2 | c.486_488del | p.162_163del | . | . |
| CTC6 | 11 | 6567895 | C | CTGCC  CTACT | 1 | NM_144666 | DNHD1 | nonframeshift_in  sertion | exon21 | c.5726_5727ins  TGCCCTACTGC | p.A1909delinsA  ALLH | . | . |
| CTC6 | 11 | 6592144 | GC | G | 0.237 | NM_144666 | DNHD1 | frameshift_deleti  on | exon42 | c.13403delC | p.A4468fs | . | . |
| CTC6 | 11 | 6655486 | AG | A | 0.219 | NM_003737 | DCHS1 | frameshift_deleti  on | exon3 | c.1848delC | p.S616fs | . | . |
| CTC6 | 11 | 6662745 | CCAG | C | 0.308 | NM_003737 | DCHS1 | nonframeshift_de  letion | exon2 | c.97_99del | p.33_33del | . | . |
| CTC6 | 11 | 6942866 | G | GA | 0.621 | NM_001004684 | OR2D3 | frameshift_inserti  on | exon1 | c.635dupA | p.E212fs | . | . |
| CTC6 | 11 | 7065044 | GC | G | 0.162 | NM_176822 | NLRP14 | frameshift_deleti  on | exon4 | c.1788delC | p.S596fs | . | . |
| CTC6 | 11 | 7531104 | GC | G | 0.223 | NM_198474 | OLFML1 | frameshift_deleti  on | exon3 | c.895delC | p.P299fs | . | . |
| CTC6 | 11 | 7725304 | AC | A | 0.455 | NM_198185 | OVCH2 | frameshift_deleti  on | exon3 | c.230delG | p.C77fs | . | . |
| CTC6 | 11 | 11314698 | G | GA | 0.303 | NM_198516 | GALNT18 | frameshift_inserti  on | exon10 | c.1554dupT | p.L519fs | . | . |
| CTC6 | 11 | 14808104 | GT | G | 1 | NM_000922 | PDE3B | frameshift_deleti  on | exon3 | c.1152delT | p.S384fs | . | . |
| CTC6 | 11 | 17416725 | C | CT | 0.9 | NM_000352 | ABCC8 | frameshift_inserti  on | exon36 | c.4404dupA | p.G1469fs | . | . |
| CTC6 | 11 | 18127527 | G | GCCTC  CT | 0.491 | NM_138421 | SAAL1 | nonframeshift_in  sertion | exon1 | c.61_62insAGG  AGG | p.A21delinsEEA | . | . |
| CTC6 | 11 | 18127530 | A | ACCTC  CT | 0.988 | NM_138421 | SAAL1 | nonframeshift_in  sertion | exon1 | c.58_59insAGG  AGG | p.V20delinsEEV | . | . |
| CTC6 | 11 | 18127530 | A | ACCT | 0.988 | NM_138421 | SAAL1 | nonframeshift_in  sertion | exon1 | c.58_59insAGG | p.V20delinsEV | . | . |
| CTC6 | 11 | 18362941 | CA | C | 0.733 | NM_005316 | GTF2H1 | frameshift_deleti  on | exon6 | c.742delA | p.K248fs | . | . |
| CTC6 | 11 | 19246804 | TG | T | 0.333 | NM_001256371 | E2F8 | frameshift_deleti  on | exon12 | c.2384delC | p.P795fs | . | . |
| CTC6 | 11 | 20385905 | AG | A | 0.314 | NM_001098522 | HTATIP2 | frameshift_deleti  on | exon1 | c.123delG | p.Q41fs | . | . |
| CTC6 | 11 | 22248958 | TC | T | 0.5 | NM_001142649 | ANO5 | frameshift_deleti  on | exon7 | c.472delC | p.P158fs | . | . |
| CTC6 | 11 | 22647028 | TG | T | 0.857 | NM_022725 | FANCF | frameshift_deleti  on | exon1 | c.328delC | p.H110fs | . | . |
| CTC6 | 11 | 28135072 | AT | A | 0.488 | NM_001113528 | METTL15 | frameshift_deleti  on | exon3 | c.192delT | p.D64fs | . | . |
| CTC6 | 11 | 31312355 | AC | A | 0.941 | NM_181807 | DCDC1 | frameshift_deleti  on | exon7 | c.798delG | p.G266fs | . | . |
| CTC6 | 11 | 32875006 | CA | C | 0.5 | NM_024081 | PRRG4 | frameshift_deleti  on | exon6 | c.615delA | p.P205fs | . | . |
| CTC6 | 11 | 43511788 | GA | G | 0.172 | NM_018259 | TTC17 | frameshift_deleti  on | exon22 | c.3031delA | p.N1011fs | . | . |
| CTC6 | 11 | 43515347 | TG | T | 0.267 | NM_018259 | TTC17 | frameshift_deleti  on | exon24 | c.3320delG | p.W1107fs | . | . |
| CTC6 | 11 | 45671825 | GC | G | 0.5 | NM_003654 | CHST1 | frameshift_deleti  on | exon4 | c.648delG | p.L216fs | . | . |
| CTC6 | 11 | 46407326 | TC | T | 1 | NM_000741 | CHRM4 | frameshift_deleti  on | exon1 | c.781delG | p.E261fs | . | . |
| CTC6 | 11 | 46724716 | CAGTGG  TGACAG | C | 1 | NM_001184751 | ZNF408 | nonframeshift_de  letion | exon4 | c.552_563del | p.184_188del | . | . |
| CTC6 | 11 | 46726452 | TG | T | 0.472 | NM_001184751 | ZNF408 | frameshift_deleti  on | exon5 | c.1179delG | p.L393fs | . | . |
| CTC6 | 11 | 46772683 | CT | C | 0.432 | NM_014756 | CKAP5 | frameshift_deleti  on | exon39 | c.5264delA | p.K1755fs | . | . |
| CTC6 | 11 | 47189555 | GC | G | 0.451 | NM_001242832 | ARFGAP2 | frameshift_deleti  on | exon11 | c.1025delG | p.G342fs | . | . |
| CTC6 | 11 | 47259447 | CA | C | 0.282 | NM_000107 | DDB2 | frameshift_deleti  on | exon8 | c.1084delA | p.K362fs | . | . |
| CTC6 | 11 | 47307108 | AC | A | 0.393 | NM_001135943 | MADD | frameshift_deleti  on | exon14 | c.2390delC | p.T797fs | . | . |
| CTC6 | 11 | 47688568 | GA | G | 0.284 | NM_024783 | AGBL2 | frameshift_deleti  on | exon17 | c.2387delT | p.F796fs | . | . |
| CTC6 | 11 | 47744588 | TGGA | T | 0.222 | NM_015308 | FNBP4 | nonframeshift_de  letion | exon15 | c.2742_2744del | p.914_915del | . | . |
| CTC6 | 11 | 48327805 | GT | G | 0.615 | NM_001004725 | OR4S1 | frameshift_deleti  on | exon1 | c.32delT | p.V11fs | . | . |
| CTC6 | 11 | 49080561 | CA | C | 1 | NM_001206631 | TRIM64C | frameshift_deleti  on | exon1 | c.103delT | p.C35fs | . | . |
| CTC6 | 11 | 55135744 | C | CT | 0.375 | NM_001005275 | OR4A15 | frameshift_inserti  on | exon1 | c.386dupT | p.L129fs | . | . |
| CTC6 | 11 | 55418773 | AC | A | 0.186 | NM_001004059 | OR4S2 | frameshift_deleti  on | exon1 | c.395delC | p.T132fs | . | . |
| CTC6 | 11 | 55872764 | CT | C | 0.231 | NM_001005200 | OR8H2 | stopgain | exon1 | c.247delT | p.L83X | . | . |
| CTC6 | 11 | 57118364 | C | CA | 0.679 | NM_002559 | P2RX3 | frameshift_inserti on | exon8 | c.835dupA | p.Y278fs | . | . |
| CTC6 | 11 | 57193631 | GCC | G | 0.171 | NM_001278201 | SLC43A3 | frameshift_deleti  on | exon3 | c.13_14del | p.G5fs | . | . |
| CTC6 | 11 | 57310798 | CA | C | 0.296 | NM_001105565 | SMTNL1 | frameshift_deleti  on | exon1 | c.684delA | p.A228fs | . | . |
| CTC6 | 11 | 57947416 | CCTT | C | 0.979 | NM_001005212 | OR9Q1 | nonframeshift_de  letion | exon3 | c.501_503del | p.167_168del | . | . |
| CTC6 | 11 | 57995878 | AG | A | 0.208 | NM_001004471 | OR10Q1 | frameshift_deleti  on | exon1 | c.469delC | p.L157fs | . | . |
| CTC6 | 11 | 58391682 | AT | A | 0.531 | NM_000614 | CNTF | frameshift_deleti  on | exon2 | c.291delT | p.H97fs | . | . |
| CTC6 | 11 | 59574192 | G | GA | 0.195 | NM_017840 | MRPL16 | frameshift_inserti  on | exon4 | c.383dupT | p.F128fs | . | . |
| CTC6 | 11 | 60476211 | AC | A | 0.25 | NM_031457 | MS4A8 | frameshift_deleti  on | exon5 | c.492delC | p.H164fs | . | . |
| CTC6 | 11 | 60696294 | AC | A | 0.397 | NM_017870 | TMEM132A | frameshift_deleti  on | exon4 | c.729delC | p.D243fs | . | . |

| CTC6 | 11 | 61026496 | GC | G | 0.492 | NM_152718 | VWCE | frameshift_deleti  on | exon20 | c.2518delG | p.A840fs | . | . |
| --- | --- | --- | --- | --- | --- | --- | --- | --- | --- | --- | --- | --- | --- |
| CTC6 | 11 | 61032018 | C | CA | 0.195 | NM_152718 | VWCE | frameshift_inserti  on | exon19 | c.2170dupT | p.C724fs | . | . |
| CTC6 | 11 | 61536747 | TC | T | 0.345 | NM_001127392 | MYRF | frameshift_deleti  on | exon4 | c.415delC | p.P139fs | . | . |
| CTC6 | 11 | 61917657 | G | GC | 0.571 | NM_020238 | INCENP | frameshift_inserti  on | exon17 | c.2509dupC | p.K836fs | . | . |
| CTC6 | 11 | 62123857 | GA | G | 0.692 | NM_001083926 | ASRGL1 | frameshift_deleti  on | exon3 | c.252delA | p.G84fs | . | . |
| CTC6 | 11 | 62285217 | CA | C | 0.406 | NM_001620 | AHNAK | frameshift_deleti  on | exon5 | c.16671delT | p.F5557fs | . | . |
| CTC6 | 11 | 62291683 | C | CT | 0.5 | NM_001620 | AHNAK | frameshift_inserti  on | exon5 | c.10205dupA | p.K3402fs | . | . |
| CTC6 | 11 | 62363226 | TG | T | 0.714 | NM_004739 | MTA2 | frameshift_deleti  on | exon13 | c.1251delC | p.T417fs | . | . |
| CTC6 | 11 | 62372604 | CG | C | 0.194 | NM_153265 | EML3 | frameshift_deleti  on | exon16 | c.1962delC | p.A654fs | . | . |
| CTC6 | 11 | 62381846 | AC | A | 0.417 | NM_000327 | ROM1 | frameshift_deleti  on | exon2 | c.708delC | p.H236fs | . | . |
| CTC6 | 11 | 62433943 | TC | T | 0.244 | NM_001043229 | METTL12 | frameshift_deleti  on | exon3 | c.144delC | p.V48fs | . | . |
| CTC6 | 11 | 62444387 | C | CA | 0.444 | NM_001286078 | UBXN1 | frameshift_inserti  on | exon6 | c.564dupT | p.G189fs | . | . |
| CTC6 | 11 | 63414010 | GC | G | 0.571 | NM_015459 | ATL3 | frameshift_deleti  on | exon6 | c.586delG | p.A196fs | . | . |
| CTC6 | 11 | 64034919 | T | TG | 0.649 | NM_001184883 | PLCB3 | frameshift_inserti  on | exon29 | c.3396dupG | p.L1132fs | . | . |
| CTC6 | 11 | 64662496 | GT | G | 0.221 | NM_015104 | ATG2A | frameshift_deleti  on | exon41 | c.5765delA | p.D1922fs | . | . |
| CTC6 | 11 | 65144411 | CCCT | C | 0.674 | NM_001278250 | SLC25A45 | nonframeshift_de  letion | exon4 | c.401_403del | p.134_135del | . | . |
| CTC6 | 11 | 65303432 | CA | C | 0.576 | NM_001048218 | SCYL1 | frameshift_deleti  on | exon11 | c.1396delA | p.R466fs | . | . |
| CTC6 | 11 | 65350702 | GC | G | 0.188 | NM_001099409 | EHBP1L1 | frameshift_deleti  on | exon9 | c.2560delC | p.P854fs | . | . |
| CTC6 | 11 | 65651082 | CT | C | 0.273 | NM_001335 | CTSW | frameshift_deleti  on | exon10 | c.1125delT | p.P375fs | . | . |
| CTC6 | 11 | 65823042 | GAGCAG  C | G | 0.25 | NM_006842 | SF3B2 | nonframeshift_de  letion | exon5 | c.536_541del | p.179_181del | . | . |
| CTC6 | 11 | 66039287 | CT | C | 0.368 | NM_030981 | RAB1B | frameshift_deleti  on | exon2 | c.35delT | p.L12fs | . | . |
| CTC6 | 11 | 66192144 | CG | C | 0.351 | NM_178864 | NPAS4 | frameshift_deleti  on | exon7 | c.1784delG | p.R595fs | . | . |
| CTC6 | 11 | 66260218 | TG | T | 0.25 | NM_001256670 | DPP3 | frameshift_deleti  on | exon9 | c.931delG | p.A311fs | . | . |
| CTC6 | 11 | 66455047 | TG | T | 0.539 | NM_006946 | SPTBN2 | frameshift_deleti  on | exon34 | c.6572delC | p.P2191fs | . | . |
| CTC6 | 11 | 66595804 | TTC | T | 0.667 | NM_024650 | C11orf80 | frameshift_deleti  on | exon14 | c.1638_1639del | p.F546fs | . | . |
| CTC6 | 11 | 66595805 | TC | T | 0.974 | NM_024650 | C11orf80 | frameshift_deleti  on | exon14 | c.1639delC | p.L547fs | . | . |
| CTC6 | 11 | 66611450 | G | GA | 0.556 | NM_005133 | RCE1 | frameshift_inserti  on | exon3 | c.313dupA | p.L104fs | . | . |
| CTC6 | 11 | 67018078 | AGAG | A | 0.221 | NM_001256405 | KDM2A | nonframeshift_de  letion | exon6 | c.1261_1263del | p.421_421del | . | . |
| CTC6 | 11 | 67198919 | TA | T | 0.364 | NM_003952 | RPS6KB2 | frameshift_deleti  on | exon5 | c.391delA | p.I131fs | . | . |
| CTC6 | 11 | 67206223 | CG | C | 0.635 | NM_020441 | CORO1B | frameshift_deleti  on | exon10 | c.1262delC | p.P421fs | . | . |
| CTC6 | 11 | 68115501 | AC | A | 0.341 | NM_002335 | LRP5 | frameshift_deleti  on | exon2 | c.279delC | p.N93fs | . | . |
| CTC6 | 11 | 68682481 | AC | A | 0.369 | NM_002180 | IGHMBP2 | frameshift_deleti  on | exon6 | c.903delC | p.D301fs | . | . |
| CTC6 | 11 | 70208444 | GA | G | 0.4 | NM_003626 | PPFIA1 | frameshift_deleti  on | exon21 | c.2716delA | p.K906fs | . | . |
| CTC6 | 11 | 71276834 | T | TG | 0.467 | NM_001012710 | KRTAP5-10 | frameshift_inserti  on | exon1 | c.202dupG | p.C67fs | . | . |
| CTC6 | 11 | 71293806 | AGAGCC  ACAGCC CCCACT | A | 0.366 | NM_001005405 | KRTAP5-11 | nonframeshift_de letion | exon1 | c.57_77del | p.19_26del | . | . |
| CTC6 | 11 | 72288620 | G | GA | 0.3 | NM_001143839 | PDE2A | frameshift_inserti  on | exon30 | c.2612dupT | p.F871fs | . | . |
| CTC6 | 11 | 72301562 | TC | T | 0.294 | NM_001143839 | PDE2A | frameshift_deleti  on | exon7 | c.590delG | p.R197fs | . | . |
| CTC6 | 11 | 73078753 | TG | T | 0.75 | NM_014786 | ARHGEF17 | frameshift_deleti  on | exon21 | c.6121delG | p.G2041fs | . | . |
| CTC6 | 11 | 73635715 | TG | T | 1 | NM_001267806 | PAAF1 | frameshift_deleti  on | exon11 | c.730delG | p.G244fs | . | . |
| CTC6 | 11 | 73677295 | AG | A | 0.214 | NM_153614 | DNAJB13 | frameshift_deleti  on | exon5 | c.606delG | p.Q202fs | . | . |
| CTC6 | 11 | 73789440 | G | GA | 0.324 | NM_001286577 | C2CD3 | frameshift_inserti  on | exon23 | c.4322dupT | p.F1441fs | . | . |
| CTC6 | 11 | 75508281 | TC | T | 0.941 | NM_001253891 | DGAT2 | frameshift_deleti  on | exon5 | c.585delC | p.V195fs | . | . |
| CTC6 | 11 | 75508282 | CG | C | 0.933 | NM_001253891 | DGAT2 | frameshift_deleti  on | exon5 | c.586delG | p.G196fs | . | . |
| CTC6 | 11 | 76831890 | C | CT | 0.222 | NM_004055 | CAPN5 | frameshift_inserti  on | exon10 | c.1423dupT | p.T474fs | . | . |
| CTC6 | 11 | 76867064 | C | CA | 0.5 | NM_000260 | MYO7A | frameshift_inserti  on | exon5 | c.398dupA | p.H133fs | . | . |
| CTC6 | 11 | 76892613 | GC | G | 0.5 | NM_000260 | MYO7A | frameshift_deleti  on | exon23 | c.2883delC | p.G961fs | . | . |
| CTC6 | 11 | 76954788 | T | TA | 0.667 | NM_182833 | GDPD4 | frameshift_inserti  on | exon12 | c.1191dupT | p.I398fs | . | . |
| CTC6 | 11 | 78775901 | AG | A | 0.316 | NM_001098816 | TENM4 | frameshift_deleti  on | exon6 | c.374delC | p.P125fs | . | . |
| CTC6 | 11 | 82639894 | TG | T | 0.875 | NM_145018 | C11orf82 | frameshift_deleti  on | exon4 | c.190delG | p.A64fs | . | . |
| CTC6 | 11 | 82643466 | TA | T | 0.667 | NM_145018 | C11orf82 | frameshift_deleti  on | exon6 | c.1087delA | p.K363fs | . | . |
| CTC6 | 11 | 82643611 | CA | C | 0.189 | NM_145018 | C11orf82 | frameshift_deleti  on | exon6 | c.1232delA | p.Q411fs | . | . |
| CTC6 | 11 | 82876994 | TA | T | 0.216 | NM_015885 | PCF11 | frameshift_deleti  on | exon5 | c.1056delA | p.I352fs | . | . |
| CTC6 | 11 | 85435696 | CA | C | 0.287 | NM_206927 | SYTL2 | frameshift_deleti  on | exon1 | c.1803delT | p.F601fs | . | . |
| CTC6 | 11 | 85456790 | AT | A | 0.34 | NM_001162951 | SYTL2 | frameshift_deleti  on | exon3 | c.278delA | p.N93fs | . | . |
| CTC6 | 11 | 89133426 | TC | T | 0.278 | NM_001143836 | NOX4 | frameshift_deleti  on | exon10 | c.967delG | p.E323fs | . | . |
| CTC6 | 11 | 89819892 | CA | C | 0.25 | NM_001143975 | UBTFL1 | frameshift_deleti  on | exon1 | c.776delA | p.Q259fs | . | . |
| CTC6 | 11 | 89939378 | GA | G | 0.365 | NM_001144073 | CHORDC1 | frameshift_deleti  on | exon6 | c.497delT | p.F166fs | . | . |

| CTC6 | 11 | 92533860 | GA | G | 0.458 | NM_001008781 | FAT3 | frameshift_deleti  on | exon9 | c.7682delA | p.E2561fs | . | . |
| --- | --- | --- | --- | --- | --- | --- | --- | --- | --- | --- | --- | --- | --- |
| CTC6 | 11 | 93431256 | CT | C | 0.308 | NM_033395 | KIAA1731 | frameshift_deleti  on | exon15 | c.3179delT | p.L1060fs | . | . |
| CTC6 | 11 | 93432728 | AT | A | 0.31 | NM_033395 | KIAA1731 | frameshift_deleti  on | exon15 | c.4651delT | p.F1551fs | . | . |
| CTC6 | 11 | 94335140 | CA | C | 0.265 | NM_152431 | PIWIL4 | frameshift_deleti  on | exon12 | c.1561delA | p.K521fs | . | . |
| CTC6 | 11 | 94862057 | GAGC | G | 0.343 | NM_015036 | ENDOD1 | nonframeshift_de  letion | exon2 | c.818_820del | p.273_274del | . | . |
| CTC6 | 11 | 95555056 | AAT | A | 0.4 | NM_001243777 | CEP57 | frameshift_deleti  on | exon7 | c.722_723del | p.N241fs | . | . |
| CTC6 | 11 | 95825374 | TTGCTGC  TGC | T | 1 | NM_032427 | MAML2 | nonframeshift_de  letion | exon2 | c.1812_1820del | p.604_607del | . | . |
| CTC6 | 11 | 95825374 | TTGCTGC  TGCTGC | T | 1 | NM_032427 | MAML2 | nonframeshift_de  letion | exon2 | c.1809_1820del | p.603_607del | . | . |
| CTC6 | 11 | 101340226 | CA | C | 0.222 | NM_004621 | TRPC6 | frameshift_deleti  on | exon10 | c.2415delT | p.N805fs | . | . |
| CTC6 | 11 | 102033297 | C | CT | 0.431 | NM_001130145 | YAP1 | frameshift_inserti  on | exon3 | c.684dupT | p.A228fs | . | . |
| CTC6 | 11 | 102056823 | AC | A | 0.643 | NM_001130145 | YAP1 | frameshift_deleti  on | exon4 | c.764delC | p.T255fs | . | . |
| CTC6 | 11 | 103229025 | GAT | G | 0.16 | NM_001377 | DYNC2H1 | frameshift_deleti  on | exon83 | c.12095_12096  del | p.D4032fs | . | . |
| CTC6 | 11 | 105924420 | CA | C | 0.286 | NM_152433 | KBTBD3 | frameshift_deleti  on | exon3 | c.995delT | p.L332fs | . | . |
| CTC6 | 11 | 108032119 | GT | G | 0.75 | NM_002519 | NPAT | frameshift_deleti  on | exon17 | c.3693delA | p.K1231fs | . | . |
| CTC6 | 11 | 108154967 | G | GT | 1 | NM_000051 | ATM | frameshift_inserti  on | exon26 | c.3761dupT | p.V1254fs | . | . |
| CTC6 | 11 | 108204624 | A | AT | 0.429 | NM_000051 | ATM | frameshift_inserti  on | exon54 | c.7940dupT | p.I2647fs | . | . |
| CTC6 | 11 | 111594551 | CCA | C | 0.318 | NM_015191 | SIK2 | frameshift_deleti  on | exon15 | c.2480_2481del | p.P827fs | . | . |
| CTC6 | 11 | 111943755 | TG | T | 0.36 | NM_001082619 | PIH1D2 | frameshift_deleti  on | exon2 | c.143delC | p.P48fs | . | . |
| CTC6 | 11 | 113802564 | GC | G | 0.627 | NM_006028 | HTR3B | frameshift_deleti  on | exon4 | c.344delC | p.A115fs | . | . |
| CTC6 | 11 | 116729010 | CTGT | C | 0.375 | NM_001281749 | SIK3 | nonframeshift_de  letion | exon20 | c.2844_2846del | p.948_949del | . | . |
| CTC6 | 11 | 117789312 | CGGGCT  GGAGAT | C | 0.857 | NM_001077263 | TMPRSS13 | nonframeshift_de  letion | exon2 | c.248_262del | p.83_88del | . | . |
| CTC6 | 11 | 117870276 | GC | G | 0.294 | NM_001558 | IL10RA | frameshift_deleti  on | exon7 | c.1658delC | p.A553fs | . | . |
| CTC6 | 11 | 118243355 | TG | T | 1 | NM_001204077 | UBE4A | frameshift_deleti  on | exon6 | c.696delG | p.L232fs | . | . |
| CTC6 | 11 | 118352642 | TC | T | 0.288 | NM_001197104 | KMT2A | frameshift_deleti  on | exon7 | c.3848delC | p.S1283fs | . | . |
| CTC6 | 11 | 118367060 | CT | C | 0.194 | NM_001197104 | KMT2A | frameshift_deleti  on | exon20 | c.5643delT | p.T1881fs | . | . |
| CTC6 | 11 | 118380754 | TGA | T | 0.667 | NM_001197104 | KMT2A | frameshift_deleti  on | exon30 | c.10993_10994  del | p.E3665fs | . | . |
| CTC6 | 11 | 118427682 | AATC | A | 0.129 | NM_001168618 | IFT46 | nonframeshift_de  letion | exon4 | c.121_123del | p.41_41del | . | . |
| CTC6 | 11 | 119148527 | TCC | T | 0.222 | NM_005188 | CBL | frameshift_deleti  on | exon7 | c.1069_1070del | p.P357fs | . | . |
| CTC6 | 11 | 119182015 | GT | G | 0.3 | NM_006500 | MCAM | frameshift_deleti  on | exon12 | c.1523delA | p.N508fs | . | . |
| CTC6 | 11 | 119535677 | CCCT | C | 0.143 | NM_002855 | PVRL1 | nonframeshift_de  letion | exon6 | c.1331_1333del | p.444_445del | . | . |
| CTC6 | 11 | 120310885 | TA | T | 0.5 | NM_001198665 | ARHGEF12 | frameshift_deleti  on | exon12 | c.991delA | p.I331fs | . | . |
| CTC6 | 11 | 121008705 | CA | C | 0.183 | NM_005422 | TECTA | frameshift_deleti  on | exon10 | c.3518delA | p.H1173fs | . | . |
| CTC6 | 11 | 121059862 | CTA | C | 0.167 | NM_005422 | TECTA | frameshift_deleti  on | exon21 | c.6237_6238del | p.P2079fs | . | . |
| CTC6 | 11 | 121424799 | TG | T | 0.766 | NM_003105 | SORL1 | frameshift_deleti  on | exon17 | c.2421delG | p.L807fs | . | . |
| CTC6 | 11 | 121444957 | C | CA | 0.317 | NM_003105 | SORL1 | frameshift_inserti  on | exon24 | c.3346dupA | p.T1115fs | . | . |
| CTC6 | 11 | 122650335 | AG | A | 0.19 | NM_032873 | UBASH3B | frameshift_deleti  on | exon4 | c.534delG | p.K178fs | . | . |
| CTC6 | 11 | 122828102 | CA | C | 0.264 | NM_024806 | C11orf63 | frameshift_deleti  on | exon8 | c.2043delA | p.A681fs | . | . |
| CTC6 | 11 | 123676202 | TA | T | 0.611 | NM_001005325 | OR6M1 | frameshift_deleti  on | exon1 | c.855delT | p.F285fs | . | . |
| CTC6 | 11 | 124095966 | AT | A | 0.667 | NM_001007249 | OR8G2 | frameshift_deleti  on | exon1 | c.570delT | p.Y190fs | . | . |
| CTC6 | 11 | 124793734 | CT | C | 0.292 | NM_152722 | HEPACAM | frameshift_deleti  on | exon3 | c.599delA | p.K200fs | . | . |
| CTC6 | 11 | 125452300 | A | AC | 1 | NM_001007277 | EI24 | frameshift_inserti  on | exon9 | c.733dupC | p.R244fs | . | . |
| CTC6 | 11 | 125499185 | AG | A | 0.25 | NM_001114121 | CHEK1 | frameshift_deleti  on | exon4 | c.349delG | p.G117fs | . | . |
| CTC6 | 11 | 130070014 | TC | T | 0.182 | NM_021978 | ST14 | frameshift_deleti  on | exon16 | c.1977delC | p.I659fs | . | . |
| CTC6 | 11 | 130343487 | T | TC | 1 | NM_139055 | ADAMTS15 | frameshift_inserti  on | exon8 | c.2625dupC | p.V875fs | . | . |
| CTC6 | 11 | 130780029 | G | GT | 0.7 | NM_014758 | SNX19 | frameshift_inserti  on | exon4 | c.1917dupA | p.L640fs | . | . |
| CTC6 | 11 | 133790690 | TC | T | 0.955 | NM_001277285 | IGSF9B | frameshift_deleti  on | exon18 | c.2929delG | p.E977fs | . | . |
| CTC6 | 11 | 134073618 | TG | T | 0.381 | NM_015261 | NCAPD3 | frameshift_deleti  on | exon11 | c.1398delC | p.S466fs | . | . |
| CTC6 | 12 | 234892 | G | GA | 0.913 | NM_001170738 | IQSEC3 | frameshift_inserti  on | exon3 | c.718dupA | p.P239fs | . | . |
| CTC6 | 12 | 336732 | TG | T | 0.333 | NM_001190997 | SLC6A13 | stopgain | exon6 | c.657delC | p.Y219X | . | . |
| CTC6 | 12 | 978053 | CA | C | 0.254 | NM_001184985 | WNK1 | frameshift_deleti on | exon9 | c.3162delA | p.S1054fs | . | . |
| CTC6 | 12 | 1005592 | CT | C | 0.526 | NM_014823 | WNK1 | frameshift_deleti  on | exon22 | c.5196delT | p.P1732fs | . | . |
| CTC6 | 12 | 2062322 | C | CTTG | 0.623 | NM_152640 | DCP1B | nonframeshift_in  sertion | exon7 | c.783_784insCA  A | p.E262delinsQE | . | . |
| CTC6 | 12 | 2062323 | T | TTGC | 0.8 | NM_152640 | DCP1B | nonframeshift_in  sertion | exon7 | c.782_783insG  CA | p.Q261delinsQ  Q | . | . |
| CTC6 | 12 | 2062337 | G | GCTT | 0.625 | NM_152640 | DCP1B | nonframeshift_in  sertion | exon7 | c.768_769insAA  G | p.Q257delinsK  Q | . | . |
| CTC6 | 12 | 2062338 | C | CTGT | 0.644 | NM_152640 | DCP1B | nonframeshift_in  sertion | exon7 | c.767_768insAC  A | p.Q256delinsQ  Q | . | . |
| CTC6 | 12 | 2967995 | TG | T | 0.27 | NM_001243088 | FOXM1 | frameshift_deleti  on | exon8 | c.2055delC | p.P685fs | . | . |
| CTC6 | 12 | 4719429 | AC | A | 0.294 | NM_001282285 | DYRK4 | frameshift_deleti  on | exon3 | c.267delC | p.D89fs | . | . |

| CTC6 | 12 | 4920709 | TC | T | 0.506 | NM_002235 | KCNA6 | frameshift_deleti  on | exon1 | c.1503delC | p.F501fs | . | . |
| --- | --- | --- | --- | --- | --- | --- | --- | --- | --- | --- | --- | --- | --- |
| CTC6 | 12 | 6562696 | TA | T | 0.652 | NM_018009 | TAPBPL | frameshift_deleti  on | exon3 | c.380delA | p.Y127fs | . | . |
| CTC6 | 12 | 6707413 | TG | T | 1 | NM_001273 | CHD4 | frameshift_deleti  on | exon11 | c.1660delC | p.H554fs | . | . |
| CTC6 | 12 | 6933727 | TG | T | 0.259 | NM_019858 | GPR162 | frameshift_deleti  on | exon2 | c.664delG | p.G222fs | . | . |
| CTC6 | 12 | 7027152 | A | AT | 0.363 | NM_001975 | ENO2 | frameshift_inserti  on | exon7 | c.494dupT | p.M165fs | . | . |
| CTC6 | 12 | 7069148 | TC | T | 0.25 | NM_002831 | PTPN6 | frameshift_deleti  on | exon12 | c.1421delC | p.S474fs | . | . |
| CTC6 | 12 | 7559445 | CTA | C | 0.6 | NM_174941 | CD163L1 | frameshift_deleti  on | exon5 | c.768_769del | p.D256fs | . | . |
| CTC6 | 12 | 7651599 | C | CA | 0.236 | NM_004244 | CD163 | frameshift_inserti  on | exon4 | c.642dupT | p.G215fs | . | . |
| CTC6 | 12 | 8200557 | ACAG | A | 0.182 | NM_018416 | FOXJ2 | nonframeshift_de  letion | exon7 | c.898_900del | p.300_300del | . | . |
| CTC6 | 12 | 8200779 | AGGC | A | 0.333 | NM_018416 | FOXJ2 | nonframeshift_de  letion | exon7 | c.1120_1122del | p.374_374del | . | . |
| CTC6 | 12 | 9098995 | G | GAAC | 0.217 | NM_001207024 | M6PR | nonframeshift_in  sertion | exon2 | c.5_6insGTT | p.F2delinsLF | . | . |
| CTC6 | 12 | 9229485 | C | CT | 0.556 | NM_000014 | A2M | frameshift_inserti  on | exon28 | c.3398dupA | p.K1133fs | . | . |
| CTC6 | 12 | 9994445 | GTGT | G | 1 | NM_016523 | KLRF1 | nonframeshift_de  letion | exon4 | c.373_375del | p.125_125del | . | . |
| CTC6 | 12 | 10150883 | CA | C | 0.844 | NM_016509 | CLEC1B | frameshift_deleti  on | exon2 | c.160delT | p.W54fs | . | . |
| CTC6 | 12 | 10603669 | GT | G | 0.826 | NM_002259 | KLRC1 | frameshift_deleti  on | exon2 | c.86delA | p.N29fs | . | . |
| CTC6 | 12 | 10763242 | AT | A | 0.455 | NM_018048 | MAGOHB | frameshift_deleti  on | exon2 | c.131delA | p.N44fs | . | . |
| CTC6 | 12 | 10782179 | GC | G | 0.636 | NM_018423 | STYK1 | frameshift_deleti  on | exon6 | c.545delG | p.G182fs | . | . |
| CTC6 | 12 | 14778754 | CT | C | 0.75 | NM_004963 | GUCY2C | frameshift_deleti  on | exon21 | c.2344delA | p.R782fs | . | . |
| CTC6 | 12 | 14781648 | AC | A | 0.531 | NM_004963 | GUCY2C | frameshift_deleti  on | exon20 | c.2180delG | p.C727fs | . | . |
| CTC6 | 12 | 15654850 | G | GA | 0.455 | NM_002848 | PTPRO | frameshift_inserti  on | exon5 | c.959dupA | p.E320fs | . | . |
| CTC6 | 12 | 16185503 | GA | G | 0.571 | NM_015954 | DERA | frameshift_deleti  on | exon7 | c.666delA | p.G222fs | . | . |
| CTC6 | 12 | 20782921 | CA | C | 0.588 | NM_001244683 | PDE3A | frameshift_deleti  on | exon5 | c.655delA | p.K219fs | . | . |
| CTC6 | 12 | 20905318 | GA | G | 0.5 | NM_001145944 | SLCO1C1 | frameshift_deleti  on | exon13 | c.1745delA | p.E582fs | . | . |
| CTC6 | 12 | 22354922 | AT | A | 0.6 | NM_003034 | ST8SIA1 | frameshift_deleti  on | exon5 | c.634delA | p.I212fs | . | . |
| CTC6 | 12 | 22401987 | TC | T | 0.889 | NM_003034 | ST8SIA1 | frameshift_deleti  on | exon4 | c.536delG | p.G179fs | . | . |
| CTC6 | 12 | 25149162 | GA | G | 0.6 | NM_001101339 | C12orf77 | frameshift_deleti  on | exon2 | c.114delT | p.R38fs | . | . |
| CTC6 | 12 | 26564312 | GT | G | 0.25 | NM_002223 | ITPR2 | frameshift_deleti  on | exon52 | c.7339delA | p.T2447fs | . | . |
| CTC6 | 12 | 29450109 | CA | C | 1 | NM_001271784 | FAR2 | frameshift_deleti  on | exon3 | c.231delA | p.P77fs | . | . |
| CTC6 | 12 | 32136956 | C | CA | 0.9 | NM_018169 | KIAA1551 | frameshift_inserti  on | exon4 | c.3068dupA | p.Q1023fs | . | . |
| CTC6 | 12 | 40223981 | TC | T | 0.27 | NM_052885 | SLC2A13 | frameshift_deleti  on | exon7 | c.1368delG | p.K456fs | . | . |
| CTC6 | 12 | 40748152 | GA | G | 0.625 | NM_198578 | LRRK2 | frameshift_deleti  on | exon45 | c.6629delA | p.E2210fs | . | . |
| CTC6 | 12 | 44605104 | AT | A | 0.613 | NM_001286212 | TMEM117 | frameshift_deleti  on | exon4 | c.231delT | p.Y77fs | . | . |
| CTC6 | 12 | 45410270 | A | AC | 0.3 | NM_001004329 | DBX2 | frameshift_inserti  on | exon4 | c.818_819insG | p.P273fs | . | . |
| CTC6 | 12 | 45742398 | C | CT | 0.5 | NM_001025356 | ANO6 | frameshift_inserti  on | exon6 | c.732dupT | p.A244fs | . | . |
| CTC6 | 12 | 45761571 | TC | T | 0.333 | NM_001025356 | ANO6 | stopgain | exon9 | c.1100delC | p.S367X | . | . |
| CTC6 | 12 | 47172515 | CA | C | 0.746 | NM_001143824 | SLC38A4 | frameshift_deleti on | exon10 | c.761delT | p.L254fs | . | . |
| CTC6 | 12 | 47471379 | CA | C | 1 | NM_181847 | AMIGO2 | frameshift_deleti  on | exon2 | c.1406delT | p.L469fs | . | . |
| CTC6 | 12 | 48185089 | GC | G | 0.301 | NM_001098416 | HDAC7 | frameshift_deleti  on | exon15 | c.1824delG | p.G608fs | . | . |
| CTC6 | 12 | 48737355 | GC | G | 0.4 | NM_001172682 | ZNF641 | frameshift_deleti  on | exon5 | c.648delG | p.G216fs | . | . |
| CTC6 | 12 | 49391099 | TA | T | 0.312 | NM_015086 | DDN | frameshift_deleti  on | exon2 | c.1559delT | p.L520fs | . | . |
| CTC6 | 12 | 49420986 | CA | C | 0.359 | NM_003482 | KMT2D | frameshift_deleti  on | exon48 | c.14762delT | p.L4921fs | . | . |
| CTC6 | 12 | 49426729 | AGCT | A | 0.122 | NM_003482 | KMT2D | nonframeshift_de  letion | exon39 | c.11756_11758  del | p.3919_3920de  l | . | . |
| CTC6 | 12 | 49427265 | TTGC | T | 0.187 | NM_003482 | KMT2D | nonframeshift_de  letion | exon39 | c.11220_11222  del | p.3740_3741de  l | . | . |
| CTC6 | 12 | 49724290 | GC | G | 0.224 | NM_005480 | TROAP | frameshift_deleti  on | exon13 | c.1663delC | p.P555fs | . | . |
| CTC6 | 12 | 49934828 | AC | A | 0.464 | NM_012284 | KCNH3 | frameshift_deleti  on | exon2 | c.224delC | p.T75fs | . | . |
| CTC6 | 12 | 50190298 | G | GA | 0.324 | NM_001037806 | NCKAP5L | frameshift_inserti  on | exon8 | c.1344dupT | p.L449fs | . | . |
| CTC6 | 12 | 50481211 | TG | T | 0.407 | NM_003076 | SMARCD1 | frameshift_deleti  on | exon5 | c.598delG | p.G200fs | . | . |
| CTC6 | 12 | 51121499 | GA | G | 0.6 | NM_173602 | DIP2B | frameshift_deleti  on | exon29 | c.3415delA | p.K1139fs | . | . |
| CTC6 | 12 | 51449979 | G | GT | 0.617 | NM_001243689 | LETMD1 | frameshift_inserti  on | exon6 | c.753dupT | p.C251fs | . | . |
| CTC6 | 12 | 51888812 | AG | A | 0.262 | NM_001039960 | SLC4A8 | frameshift_deleti  on | exon21 | c.2854delG | p.V952fs | . | . |
| CTC6 | 12 | 52376582 | A | AT | 0.295 | NM_020328 | ACVR1B | frameshift_inserti  on | exon5 | c.925dupT | p.L308fs | . | . |
| CTC6 | 12 | 52387805 | T | TG | 0.474 | NM_004302 | ACVR1B | frameshift_inserti  on | exon9 | c.1430dupG | p.W477fs | . | . |
| CTC6 | 12 | 52884731 | CCA | C | 0.179 | NM_005554 | KRT6A | frameshift_deleti  on | exon4 | c.821_822del | p.V274fs | . | . |
| CTC6 | 12 | 53045615 | A | ACCTC  CAAAG CCGCT | 1 | NM_000423 | KRT2 | nonframeshift_in sertion | exon1 | c.311_312insC  GGCAGCGGCT TTGGAGG | p.G104delinsG GSGFGG | . | . |
| CTC6 | 12 | 53680651 | AC | A | 0.226 | NM_012291 | ESPL1 | frameshift_deleti  on | exon18 | c.4132delC | p.P1378fs | . | . |
| CTC6 | 12 | 53681908 | T | TA | 0.667 | NM_012291 | ESPL1 | frameshift_inserti  on | exon19 | c.4330dupA | p.G1443fs | . | . |
| CTC6 | 12 | 53691877 | AC | A | 0.244 | NM_145897 | PFDN5 | frameshift_deleti  on | exon3 | c.197delC | p.T66fs | . | . |

| CTC6 | 12 | 53722473 | AC | A | 0.4 | NM_152860 | SP7 | frameshift_deleti  on | exon2 | c.752delG | p.G251fs | . | . |
| --- | --- | --- | --- | --- | --- | --- | --- | --- | --- | --- | --- | --- | --- |
| CTC6 | 12 | 53837554 | CA | C | 0.222 | NM_001005354 | PRR13 | frameshift_deleti  on | exon3 | c.250delA | p.K84fs | . | . |
| CTC6 | 12 | 53899495 | AC | A | 0.972 | NM_004178 | TARBP2 | frameshift_deleti  on | exon8 | c.742delC | p.R248fs | . | . |
| CTC6 | 12 | 53899496 | CG | C | 0.971 | NM_004178 | TARBP2 | frameshift_deleti  on | exon8 | c.743delG | p.R248fs | . | . |
| CTC6 | 12 | 55420750 | TG | T | 0.407 | NM_021191 | NEUROD4 | frameshift_deleti  on | exon2 | c.528delG | p.L176fs | . | . |
| CTC6 | 12 | 56031604 | GC | G | 0.333 | NM_206899 | OR10P1 | frameshift_deleti  on | exon1 | c.930delC | p.R310fs | . | . |
| CTC6 | 12 | 56351345 | TG | T | 0.571 | NM_001200053 | PMEL | frameshift_deleti  on | exon4 | c.483delC | p.P161fs | . | . |
| CTC6 | 12 | 56397887 | TGG | T | 0.184 | NM_001032387 | SUOX | frameshift_deleti  on | exon4 | c.715_716del | p.G239fs | . | . |
| CTC6 | 12 | 56420680 | G | GC | 0.357 | NM_022465 | IKZF4 | frameshift_inserti  on | exon4 | c.403dupC | p.E134fs | . | . |
| CTC6 | 12 | 56481891 | AC | A | 0.192 | NM_001982 | ERBB3 | frameshift_deleti  on | exon7 | c.820delC | p.P274fs | . | . |
| CTC6 | 12 | 56514400 | TG | T | 0.276 | NM_032786 | ZC3H10 | frameshift_deleti  on | exon3 | c.55delG | p.G19fs | . | . |
| CTC6 | 12 | 56578653 | A | AT | 0.171 | NM_001130420 | SMARCC2 | frameshift_inserti  on | exon5 | c.466_467insA | p.L156fs | . | . |
| CTC6 | 12 | 57005636 | AT | A | 0.256 | NM_013449 | BAZ2A | frameshift_deleti  on | exon6 | c.1535delA | p.N512fs | . | . |
| CTC6 | 12 | 57011313 | A | AT | 0.5 | NM_013449 | BAZ2A | frameshift_inserti  on | exon2 | c.7dupA | p.M3fs | . | . |
| CTC6 | 12 | 57396611 | G | GA | 0.207 | NM_014830 | ZBTB39 | frameshift_inserti  on | exon2 | c.2090dupT | p.F697fs | . | . |
| CTC6 | 12 | 57424935 | C | CA | 0.273 | NM_005379 | MYO1A | frameshift_inserti  on | exon23 | c.2372dupT | p.L791fs | . | . |
| CTC6 | 12 | 57492323 | TG | T | 0.688 | NM_001178081 | STAT6 | frameshift_deleti  on | exon17 | c.1793delC | p.P598fs | . | . |
| CTC6 | 12 | 57628058 | G | GA | 0.222 | NM_001166356 | SHMT2 | frameshift_inserti  on | exon12 | c.1400dupA | p.E467fs | . | . |
| CTC6 | 12 | 57860148 | TG | T | 0.582 | NM_001160045 | GLI1 | frameshift_deleti  on | exon6 | c.505delG | p.G169fs | . | . |
| CTC6 | 12 | 57884031 | A | AC | 0.385 | NM_004990 | MARS | frameshift_inserti  on | exon6 | c.533dupC | p.T178fs | . | . |
| CTC6 | 12 | 57910931 | A | AT | 0.163 | NM_001195056 | DDIT3 | frameshift_inserti  on | exon3 | c.239_240insA | p.P80fs | . | . |
| CTC6 | 12 | 58157881 | A | AT | 0.312 | NM_000785 | CYP27B1 | frameshift_inserti  on | exon7 | c.1214dupA | p.N405fs | . | . |
| CTC6 | 12 | 63974560 | TA | T | 0.333 | NM_173812 | DPY19L2 | stopgain | exon19 | c.1781delT | p.L594X | . | . |
| CTC6 | 12 | 64011144 | CA | C | 0.727 | NM_173812 | DPY19L2 | stopgain | exon11 | c.1157delT | p.L386X | . | . |
| CTC6 | 12 | 64712546 | A | AGTT | 0.645 | NM_001170633 | C12orf56 | nonframeshift_in sertion | exon4 | c.702_703insAA C | p.S235delinsNS | . | . |
| CTC6 | 12 | 64818879 | GC | G | 0.973 | NM_007235 | XPOT | frameshift_deleti  on | exon13 | c.1370delC | p.A457fs | . | . |
| CTC6 | 12 | 70960284 | AT | A | 0.184 | NM_001206971 | PTPRB | frameshift_deleti  on | exon12 | c.2910delA | p.K970fs | . | . |
| CTC6 | 12 | 70989920 | G | GA | 0.391 | NM_001206971 | PTPRB | frameshift_inserti  on | exon3 | c.512dupT | p.F171fs | . | . |
| CTC6 | 12 | 72092782 | AT | A | 0.889 | NM_018279 | TMEM19 | frameshift_deleti  on | exon5 | c.741delT | p.D247fs | . | . |
| CTC6 | 12 | 76739837 | AC | A | 0.4 | NM_024685 | BBS10 | frameshift_deleti  on | exon2 | c.1927delG | p.V643fs | . | . |
| CTC6 | 12 | 76765335 | CT | C | 0.543 | NM_001003712 | OSBPL8 | frameshift_deleti  on | exon18 | c.1820delA | p.K607fs | . | . |
| CTC6 | 12 | 80672871 | CG | C | 0.455 | NM_173591 | OTOGL | frameshift_deleti  on | exon25 | c.2827delG | p.G943fs | . | . |
| CTC6 | 12 | 83324312 | TG | T | 0.267 | NM_152588 | TMTC2 | frameshift_deleti  on | exon4 | c.1587delG | p.M529fs | . | . |
| CTC6 | 12 | 88478556 | T | TC | 0.364 | NM_025114 | CEP290 | frameshift_inserti  on | exon35 | c.4510dupG | p.E1504fs | . | . |
| CTC6 | 12 | 89918230 | G | GA | 0.35 | NM_003774 | GALNT4 | frameshift_inserti  on | exon1 | c.96dupT | p.H33fs | . | . |
| CTC6 | 12 | 91348253 | TC | T | 1 | NM_152638 | CCER1 | frameshift_deleti  on | exon1 | c.266delG | p.G89fs | . | . |
| CTC6 | 12 | 91502554 | A | AT | 0.5 | NM_002345 | LUM | frameshift_inserti  on | exon2 | c.202dupA | p.I68fs | . | . |
| CTC6 | 12 | 93100884 | CA | C | 0.211 | NM_001037671 | C12orf74 | frameshift_deleti  on | exon2 | c.478delA | p.S160fs | . | . |
| CTC6 | 12 | 93968774 | GT | G | 0.75 | NM_001270471 | SOCS2 | frameshift_deleti  on | exon2 | c.417delT | p.G139fs | . | . |
| CTC6 | 12 | 94706692 | A | AT | 0.824 | NM_001042399 | CCDC41 | frameshift_inserti  on | exon14 | c.1808dupA | p.N603fs | . | . |
| CTC6 | 12 | 94796980 | G | GT | 0.224 | NM_001042399 | CCDC41 | frameshift_inserti  on | exon4 | c.382dupA | p.T128fs | . | . |
| CTC6 | 12 | 95668561 | TG | T | 0.161 | NM_017599 | VEZT | frameshift_deleti  on | exon7 | c.893delG | p.C298fs | . | . |
| CTC6 | 12 | 96412583 | G | GC | 0.379 | NM_000895 | LTA4H | frameshift_inserti  on | exon8 | c.809dupG | p.G270fs | . | . |
| CTC6 | 12 | 99126215 | TG | T | 0.301 | NM_181869 | APAF1 | frameshift_deleti  on | exon8 | c.974delG | p.W325fs | . | . |
| CTC6 | 12 | 100453104 | TA | T | 0.406 | NM_015054 | UHRF1BP1L | frameshift_deleti  on | exon14 | c.1950delT | p.F650fs | . | . |
| CTC6 | 12 | 100657577 | T | TA | 0.375 | NM_152317 | DEPDC4 | frameshift_inserti  on | exon2 | c.251dupT | p.L84fs | . | . |
| CTC6 | 12 | 103762768 | AG | A | 0.304 | NM_001099336 | C12orf42 | frameshift_deleti  on | exon4 | c.155delC | p.P52fs | . | . |
| CTC6 | 12 | 104326711 | CA | C | 0.481 | NM_003299 | HSP90B1 | frameshift_deleti  on | exon4 | c.403delA | p.K135fs | . | . |
| CTC6 | 12 | 104327913 | CG | C | 0.944 | NM_003299 | HSP90B1 | frameshift_deleti  on | exon5 | c.592delG | p.G198fs | . | . |
| CTC6 | 12 | 104327928 | CT | C | 0.885 | NM_003299 | HSP90B1 | frameshift_deleti  on | exon5 | c.607delT | p.F203fs | . | . |
| CTC6 | 12 | 104336538 | CA | C | 0.25 | NM_003299 | HSP90B1 | frameshift_deleti  on | exon12 | c.1609delA | p.K537fs | . | . |
| CTC6 | 12 | 104341187 | TGAA | T | 1 | NM_003299 | HSP90B1 | nonframeshift_de  letion | exon17 | c.2362_2364del | p.788_788del | . | . |
| CTC6 | 12 | 106461423 | AC | A | 0.349 | NM_014840 | NUAK1 | frameshift_deleti  on | exon7 | c.1142delG | p.G381fs | . | . |
| CTC6 | 12 | 108012035 | GC | G | 0.478 | NM_001017523 | BTBD11 | frameshift_deleti  on | exon8 | c.944delC | p.A315fs | . | . |
| CTC6 | 12 | 109505378 | CT | C | 0.217 | NM_032663 | USP30 | frameshift_deleti  on | exon4 | c.427delT | p.L143fs | . | . |
| CTC6 | 12 | 110778723 | GT | G | 0.273 | NM_001681 | ATP2A2 | frameshift_deleti  on | exon14 | c.2022delT | p.C674fs | . | . |
| CTC6 | 12 | 110927899 | CA | C | 0.833 | NM_013300 | FAM216A | frameshift_deleti  on | exon7 | c.780delA | p.S260fs | . | . |
| CTC6 | 12 | 111800826 | TGCCAC  CCCC | T | 1 | NM_001177997 | FAM109A | nonframeshift_de  letion | exon3 | c.397_405del | p.133_135del | . | . |

| CTC6 | 12 | 112589674 | CT | C | 0.667 | NM_001143906 | TRAFD1 | frameshift_deleti  on | exon10 | c.1350delT | p.P450fs | . | . |
| --- | --- | --- | --- | --- | --- | --- | --- | --- | --- | --- | --- | --- | --- |
| CTC6 | 12 | 113629184 | CG | C | 0.214 | NM_001286215 | RITA1 | frameshift_deleti  on | exon3 | c.445delG | p.G149fs | . | . |
| CTC6 | 12 | 117476993 | TC | T | 0.786 | NM_001168325 | TESC | frameshift_deleti  on | exon7 | c.508delG | p.E170fs | . | . |
| CTC6 | 12 | 117494635 | AT | A | 0.6 | NM_017899 | TESC | frameshift_deleti  on | exon3 | c.184delA | p.I62fs | . | . |
| CTC6 | 12 | 117665244 | CG | C | 0.6 | NM_001204213 | NOS1 | frameshift_deleti  on | exon22 | c.2599delC | p.R867fs | . | . |
| CTC6 | 12 | 120158321 | GA | G | 0.6 | NM_007174 | CIT | frameshift_deleti  on | exon29 | c.3675delT | p.F1225fs | . | . |
| CTC6 | 12 | 120221778 | CA | C | 0.7 | NM_001206999 | CIT | frameshift_deleti  on | exon12 | c.1478delT | p.L493fs | . | . |
| CTC6 | 12 | 120998620 | GC | G | 0.889 | NM_014868 | RNF10 | frameshift_deleti  on | exon7 | c.1060delC | p.Q354fs | . | . |
| CTC6 | 12 | 121678327 | C | CTTT | 0.909 | NM_001270486 | CAMKK2 | nonframeshift_in  sertion | exon16 | c.1614_1615ins  AAA | p.G539delinsK  G | . | . |
| CTC6 | 12 | 122255537 | CGGA | C | 0.141 | NM_015048 | SETD1B | nonframeshift_de  letion | exon9 | c.3162_3164del | p.1054_1055de  l | . | . |
| CTC6 | 12 | 122716917 | GC | G | 0.702 | NM_022916 | VPS33A | frameshift_deleti  on | exon13 | c.1666delG | p.A556fs | . | . |
| CTC6 | 12 | 122839723 | GC | G | 0.5 | NM_001247997 | CLIP1 | frameshift_deleti  on | exon6 | c.1141delG | p.A381fs | . | . |
| CTC6 | 12 | 123340834 | C | CA | 1 | NM_003959 | HIP1R | frameshift_inserti  on | exon15 | c.1345dupA | p.N448fs | . | . |
| CTC6 | 12 | 123497209 | GT | G | 0.825 | NM_020845 | PITPNM2 | frameshift_deleti  on | exon4 | c.365delA | p.N122fs | . | . |
| CTC6 | 12 | 123712139 | AT | A | 0.526 | NM_022782 | MPHOSPH9 | frameshift_deleti  on | exon3 | c.128delA | p.N43fs | . | . |
| CTC6 | 12 | 124097724 | AG | A | 0.393 | NM_020936 | DDX55 | frameshift_deleti  on | exon8 | c.750delG | p.K250fs | . | . |
| CTC6 | 12 | 124358214 | GA | G | 0.355 | NM_207437 | DNAH10 | frameshift_deleti  on | exon45 | c.7542delA | p.G2514fs | . | . |
| CTC6 | 12 | 124857012 | TC | T | 0.5 | NM_001077261 | NCOR2 | frameshift_deleti  on | exon21 | c.2308delG | p.E770fs | . | . |
| CTC6 | 12 | 124887058 | G | GGCT | 1 | NM_001077261 | NCOR2 | nonframeshift_in  sertion | exon16 | c.1528_1529ins  AGC | p.P510delinsQ  P | . | . |
| CTC6 | 12 | 124887058 | G | GGCTG  CT | 1 | NM_001077261 | NCOR2 | nonframeshift_in  sertion | exon16 | c.1528_1529ins  AGCAGC | p.P510delinsQ  QP | . | . |
| CTC6 | 12 | 125465269 | ACTC | A | 0.25 | NM_032656 | DHX37 | nonframeshift_de  letion | exon4 | c.502_504del | p.168_168del | . | . |
| CTC6 | 12 | 130840147 | TC | T | 0.314 | NM_001190971 | PIWIL1 | frameshift_deleti  on | exon12 | c.1340delC | p.S447fs | . | . |
| CTC6 | 12 | 130847651 | GT | G | 0.366 | NM_001190971 | PIWIL1 | frameshift_deleti  on | exon18 | c.2158delT | p.F720fs | . | . |
| CTC6 | 12 | 133182783 | CCAG | C | 0.154 | NM_001195520 | LRCOL1 | nonframeshift_de  letion | exon2 | c.52_54del | p.18_18del | . | . |
| CTC6 | 12 | 133249785 | CA | C | 0.24 | NM_006231 | POLE | frameshift_deleti  on | exon14 | c.1437delT | p.F479fs | . | . |
| CTC6 | 13 | 20233410 | AAT | A | 0.429 | NM_017520 | MPHOSPH8 | frameshift_deleti  on | exon7 | c.1773_1774del | p.E591fs | . | . |
| CTC6 | 13 | 23914799 | AT | A | 0.164 | NM_001278055 | SACS | frameshift_deleti  on | exon8 | c.2774delA | p.Y925fs | . | . |
| CTC6 | 13 | 25479835 | A | AG | 0.179 | NM_018451 | CENPJ | frameshift_inserti  on | exon7 | c.2340dupC | p.S781fs | . | . |
| CTC6 | 13 | 28014376 | CT | C | 0.667 | NM_001166261 | MTIF3 | frameshift_deleti  on | exon3 | c.209delA | p.K70fs | . | . |
| CTC6 | 13 | 28127542 | CA | C | 0.378 | NM_153371 | LNX2 | stopgain | exon8 | c.1580delT | p.L527X | . | . |
| CTC6 | 13 | 29608209 | AC | A | 0.328 | NM_001033602 | MTUS2 | frameshift_deleti on | exon2 | c.2424delC | p.H808fs | . | . |
| CTC6 | 13 | 31221080 | GTC | G | 0.364 | NM_005800 | USPL1 | frameshift_deleti  on | exon7 | c.1125_1126del | p.S375fs | . | . |
| CTC6 | 13 | 31531127 | A | AT | 0.714 | NM_152325 | TEX26 | frameshift_inserti  on | exon4 | c.431dupT | p.I144fs | . | . |
| CTC6 | 13 | 39261567 | GGCT | G | 0.167 | NM_207361 | FREM2 | nonframeshift_de  letion | exon1 | c.87_89del | p.29_30del | . | . |
| CTC6 | 13 | 41814474 | CG | C | 0.267 | NM_004294 | MTRF1 | frameshift_deleti  on | exon6 | c.792delC | p.P264fs | . | . |
| CTC6 | 13 | 41947850 | GA | G | 0.538 | NM_024561 | NAA16 | frameshift_deleti  on | exon18 | c.2234delA | p.D745fs | . | . |
| CTC6 | 13 | 46170718 | CCCCAG  ATACTCT TCCTCCT | C | 0.786 | NM_182542 | FAM194B | frameshift_deleti on | exon3 | c.404_422del | p.E135fs | . | . |
| CTC6 | 13 | 46170719 | CCCAGA  TACTCTT CCTCCT | C | 1 | NM_182542 | FAM194B | nonframeshift_de letion | exon3 | c.404_421del | p.135_141del | . | . |
| CTC6 | 13 | 46357945 | CGCAGG  TGGGGC | C | 0.121 | NM_198849 | SIAH3 | nonframeshift_de  letion | exon2 | c.368_382del | p.123_128del | . | . |
| CTC6 | 13 | 47324775 | TG | T | 0.186 | NM_001164213 | LRCH1 | frameshift_deleti  on | exon19 | c.2082delG | p.L694fs | . | . |
| CTC6 | 13 | 48881429 | GA | G | 0.529 | NM_000321 | RB1 | frameshift_deleti  on | exon2 | c.152delA | p.E51fs | . | . |
| CTC6 | 13 | 49762731 | T | TA | 0.262 | NM_014923 | FNDC3A | frameshift_inserti  on | exon15 | c.1738_1739ins  A | p.L580fs | . | . |
| CTC6 | 13 | 50059856 | CA | C | 0.389 | NM_001160308 | SETDB2 | frameshift_deleti  on | exon10 | c.1574delA | p.Q525fs | . | . |
| CTC6 | 13 | 51918509 | CT | C | 0.311 | NM_001101320 | SERPINE3 | frameshift_deleti  on | exon2 | c.379delT | p.F127fs | . | . |
| CTC6 | 13 | 52511656 | CG | C | 0.457 | NM_001005918 | ATP7B | frameshift_deleti  on | exon14 | c.3237delC | p.T1079fs | . | . |
| CTC6 | 13 | 52725286 | A | AT | 0.156 | NM_001146099 | NEK3 | frameshift_inserti  on | exon7 | c.542dupA | p.N181fs | . | . |
| CTC6 | 13 | 52725354 | CA | C | 0.211 | NM_001146099 | NEK3 | frameshift_deleti  on | exon7 | c.474delT | p.F158fs | . | . |
| CTC6 | 13 | 67801537 | TG | T | 0.255 | NM_020403 | PCDH9 | frameshift_deleti  on | exon2 | c.1035delC | p.T345fs | . | . |
| CTC6 | 13 | 79911267 | GCT | G | 0.206 | NM_001286632 | RBM26 | frameshift_deleti  on | exon18 | c.2629_2630del | p.S877fs | . | . |
| CTC6 | 13 | 84455004 | AG | A | 0.203 | NM_052910 | SLITRK1 | frameshift_deleti  on | exon1 | c.638delC | p.P213fs | . | . |
| CTC6 | 13 | 92101040 | CAA | C | 0.278 | NM_004466 | GPC5 | frameshift_deleti  on | exon2 | c.190_191del | p.K64fs | . | . |
| CTC6 | 13 | 95715056 | TA | T | 0.333 | NM_005845 | ABCC4 | frameshift_deleti  on | exon26 | c.3267delT | p.F1089fs | . | . |
| CTC6 | 13 | 99378507 | G | GA | 0.17 | NM_005073 | SLC15A1 | frameshift_inserti  on | exon4 | c.114dupT | p.L39fs | . | . |
| CTC6 | 13 | 99907901 | GT | G | 0.362 | NM_001098200 | GPR18 | frameshift_deleti  on | exon2 | c.225delA | p.L75fs | . | . |
| CTC6 | 13 | 99948343 | A | AT | 0.754 | NM_004951 | GPR183 | frameshift_inserti  on | exon2 | c.56dupA | p.N19fs | . | . |
| CTC6 | 13 | 100518622 | CT | C | 0.302 | NM_206808 | CLYBL | frameshift_deleti  on | exon6 | c.764delT | p.L255fs | . | . |

| CTC6 | 13 | 100617724 | GC | G | 0.7 | NM_033132 | ZIC5 | frameshift_deleti  on | exon2 | c.1898delG | p.S633fs | . | . |
| --- | --- | --- | --- | --- | --- | --- | --- | --- | --- | --- | --- | --- | --- |
| CTC6 | 13 | 103387145 | AC | A | 0.802 | NM_001146197 | CCDC168 | frameshift_deleti  on | exon4 | c.15901delG | p.V5301fs | . | . |
| CTC6 | 13 | 103394150 | TG | T | 0.333 | NM_001146197 | CCDC168 | frameshift_deleti  on | exon4 | c.8896delC | p.Q2966fs | . | . |
| CTC6 | 13 | 107822863 | GT | G | 0.734 | NM_001080396 | FAM155A | frameshift_deleti  on | exon3 | c.1358delA | p.N453fs | . | . |
| CTC6 | 13 | 108861681 | CT | C | 0.333 | NM_001098268 | LIG4 | frameshift_deleti  on | exon2 | c.1935delA | p.K645fs | . | . |
| CTC6 | 13 | 110862327 | TG | T | 0.444 | NM_001845 | COL4A1 | frameshift_deleti  on | exon10 | c.614delC | p.P205fs | . | . |
| CTC6 | 13 | 111298378 | CT | C | 0.189 | NM_024537 | CARS2 | frameshift_deleti  on | exon12 | c.1252delA | p.R418fs | . | . |
| CTC6 | 13 | 113201846 | ACGTGC  ATGGGA | A | 0.636 | NM_001286279 | TUBGCP3 | nonframeshift_de  letion | exon10 | c.1241_1255del | p.414_419del | . | . |
| CTC6 | 13 | 113201852 | ATGGGA  AAGTCG | A | 1 | NM_001286279 | TUBGCP3 | nonframeshift_de  letion | exon10 | c.1235_1249del | p.412_417del | . | . |
| CTC6 | 13 | 113730384 | GT | G | 0.727 | NM_001112732 | MCF2L | frameshift_deleti  on | exon12 | c.1414delT | p.F472fs | . | . |
| CTC6 | 13 | 114839280 | GC | G | 0.294 | NM_007368 | RASA3 | frameshift_deleti  on | exon2 | c.87delG | p.G29fs | . | . |
| CTC6 | 14 | 20482972 | T | TA | 0.267 | NM_001004712 | OR4K14 | frameshift_inserti  on | exon1 | c.380dupT | p.I127fs | . | . |
| CTC6 | 14 | 20764589 | T | TA | 1 | NM_138376 | TTC5 | frameshift_inserti  on | exon6 | c.655_656insT | p.K219fs | . | . |
| CTC6 | 14 | 20813104 | CA | C | 1 | NM_001042618 | PARP2 | frameshift_deleti  on | exon2 | c.61delA | p.K21fs | . | . |
| CTC6 | 14 | 20813185 | GA | G | 0.909 | NM_001042618 | PARP2 | frameshift_deleti  on | exon2 | c.142delA | p.K48fs | . | . |
| CTC6 | 14 | 21464953 | TC | T | 0.551 | NM_001029991 | METTL17 | frameshift_deleti  on | exon13 | c.1349delC | p.S450fs | . | . |
| CTC6 | 14 | 21955646 | G | GA | 0.9 | NM_014828 | TOX4 | frameshift_inserti  on | exon3 | c.113dupA | p.E38fs | . | . |
| CTC6 | 14 | 23236602 | CT | C | 0.588 | NM_005015 | OXA1L | frameshift_deleti  on | exon2 | c.390delT | p.S130fs | . | . |
| CTC6 | 14 | 23354177 | CT | C | 0.438 | NM_173527 | REM2 | frameshift_deleti  on | exon2 | c.399delT | p.T133fs | . | . |
| CTC6 | 14 | 23745439 | TG | T | 0.478 | NM_020834 | HOMEZ | frameshift_deleti  on | exon2 | c.997delC | p.Q333fs | . | . |
| CTC6 | 14 | 23854137 | C | CT | 0.5 | NM_002471 | MYH6 | frameshift_inserti  on | exon35 | c.5276dupA | p.K1759fs | . | . |
| CTC6 | 14 | 23876395 | G | GC | 0.667 | NM_002471 | MYH6 | frameshift_inserti  on | exon3 | c.37dupG | p.A13fs | . | . |
| CTC6 | 14 | 23900986 | TC | T | 0.364 | NM_000257 | MYH7 | frameshift_deleti  on | exon7 | c.622delG | p.D208fs | . | . |
| CTC6 | 14 | 24527256 | GC | G | 0.262 | NM_138360 | LRRC16B | frameshift_deleti  on | exon16 | c.1306delC | p.P436fs | . | . |
| CTC6 | 14 | 24601439 | TG | T | 0.484 | NM_203402 | FITM1 | frameshift_deleti  on | exon2 | c.287delG | p.W96fs | . | . |
| CTC6 | 14 | 24646406 | G | GGAA | 1 | NM_001048205 | REC8 | nonframeshift_in  sertion | exon8 | c.681_682insG  AA | p.E227delinsEE | . | . |
| CTC6 | 14 | 24656651 | CA | C | 0.382 | NM_024658 | IPO4 | frameshift_deleti  on | exon6 | c.542delT | p.L181fs | . | . |
| CTC6 | 14 | 24799154 | CA | C | 0.263 | NM_001198568 | ADCY4 | frameshift_deleti  on | exon8 | c.1128delT | p.C376fs | . | . |
| CTC6 | 14 | 24807151 | CG | C | 0.315 | NM_006871 | RIPK3 | frameshift_deleti  on | exon6 | c.759delC | p.P253fs | . | . |
| CTC6 | 14 | 24839041 | AC | A | 0.744 | NM_001198965 | NFATC4 | frameshift_deleti  on | exon2 | c.438delC | p.Y146fs | . | . |
| CTC6 | 14 | 31570213 | GC | G | 0.194 | NM_015382 | HECTD1 | frameshift_deleti  on | exon43 | c.7755delG | p.L2585fs | . | . |
| CTC6 | 14 | 45501560 | CT | C | 0.286 | NM_015091 | FAM179B | frameshift_deleti  on | exon11 | c.3794delT | p.L1265fs | . | . |
| CTC6 | 14 | 50655244 | GA | G | 0.5 | NM_006939 | SOS2 | frameshift_deleti  on | exon5 | c.684delT | p.F228fs | . | . |
| CTC6 | 14 | 51239057 | GC | G | 0.585 | NM_016350 | NIN | frameshift_deleti  on | exon9 | c.942delG | p.W314fs | . | . |
| CTC6 | 14 | 51475798 | CT | C | 0.404 | NM_015163 | TRIM9 | frameshift_deleti  on | exon5 | c.1305delA | p.K435fs | . | . |
| CTC6 | 14 | 53385841 | TA | T | 1 | NM_001134999 | FERMT2 | frameshift_deleti  on | exon3 | c.390delT | p.F130fs | . | . |
| CTC6 | 14 | 55236871 | TG | T | 0.647 | NM_001161577 | SAMD4A | frameshift_deleti  on | exon4 | c.420delG | p.V140fs | . | . |
| CTC6 | 14 | 56078899 | CA | C | 0.636 | NM_001079521 | KTN1 | frameshift_deleti  on | exon2 | c.134delA | p.Q45fs | . | . |
| CTC6 | 14 | 56138509 | A | AT | 0.297 | NM_004986 | KTN1 | frameshift_inserti  on | exon36 | c.3359dupT | p.I1120fs | . | . |
| CTC6 | 14 | 57858198 | CAGG | C | 0.167 | NM_001011713 | NAA30 | nonframeshift_de  letion | exon2 | c.524_526del | p.175_176del | . | . |
| CTC6 | 14 | 58917336 | AG | A | 1 | NM_001244193 | KIAA0586 | frameshift_deleti  on | exon5 | c.582delG | p.Q194fs | . | . |
| CTC6 | 14 | 60749672 | AT | A | 0.716 | NM_021003 | PPM1A | frameshift_deleti  on | exon2 | c.252delT | p.D84fs | . | . |
| CTC6 | 14 | 61518782 | AT | A | 1 | NM_001172702 | SLC38A6 | frameshift_deleti  on | exon15 | c.1220delT | p.I407fs | . | . |
| CTC6 | 14 | 67664993 | GC | G | 0.625 | NM_173526 | FAM71D | frameshift_deleti  on | exon3 | c.116delC | p.A39fs | . | . |
| CTC6 | 14 | 68251925 | TGG | T | 0.5 | NM_015346 | ZFYVE26 | frameshift_deleti  on | exon19 | c.3372_3373del | p.A1124fs | . | . |
| CTC6 | 14 | 70175758 | GC | G | 0.429 | NM_014734 | KIAA0247 | frameshift_deleti  on | exon5 | c.824delC | p.A275fs | . | . |
| CTC6 | 14 | 71197375 | TG | T | 0.452 | NM_001284232 | MAP3K9 | frameshift_deleti  on | exon11 | c.2235delC | p.P745fs | . | . |
| CTC6 | 14 | 73577837 | CA | C | 0.75 | NM_021239 | RBM25 | frameshift_deleti  on | exon15 | c.1992delA | p.P664fs | . | . |
| CTC6 | 14 | 73750805 | GA | G | 0.278 | NM_001005745 | NUMB | frameshift_deleti  on | exon9 | c.899delT | p.F300fs | . | . |
| CTC6 | 14 | 74086159 | CA | C | 0.833 | NM_001037162 | ACOT6 | frameshift_deleti  on | exon2 | c.241delA | p.T81fs | . | . |
| CTC6 | 14 | 74189486 | AT | A | 0.262 | NM_001043318 | ELMSAN1 | frameshift_deleti  on | exon10 | c.2638delA | p.I880fs | . | . |
| CTC6 | 14 | 74495923 | GA | G | 0.5 | NM_025057 | CCDC176 | frameshift_deleti  on | exon3 | c.323delA | p.E108fs | . | . |
| CTC6 | 14 | 75283767 | AT | A | 0.267 | NM_019589 | YLPM1 | frameshift_deleti  on | exon14 | c.5820delT | p.H1940fs | . | . |
| CTC6 | 14 | 75514267 | GA | G | 0.544 | NM_001040108 | MLH3 | frameshift_deleti  on | exon2 | c.2091delT | p.F697fs | . | . |
| CTC6 | 14 | 75558150 | GC | G | 0.5 | NM_033116 | NEK9 | frameshift_deleti  on | exon19 | c.2264delG | p.G755fs | . | . |
| CTC6 | 14 | 75593494 | GCGC | G | 0.25 | NM_033116 | NEK9 | nonframeshift_de  letion | exon1 | c.128_130del | p.43_44del | . | . |
| CTC6 | 14 | 76245996 | TA | T | 0.699 | NM_015072 | TTLL5 | frameshift_deleti  on | exon24 | c.2467delA | p.K823fs | . | . |

| CTC6 | 14 | 77572173 | ACT | A | 0.156 | NM_033426 | CIPC | frameshift_deleti  on | exon2 | c.123_124del | p.D41fs | . | . |
| --- | --- | --- | --- | --- | --- | --- | --- | --- | --- | --- | --- | --- | --- |
| CTC6 | 14 | 77685269 | TC | T | 0.38 | NM_020431 | TMEM63C | frameshift_deleti  on | exon3 | c.114delC | p.V38fs | . | . |
| CTC6 | 14 | 78399670 | AG | A | 0.58 | NM_001142545 | ADCK1 | frameshift_deleti  on | exon10 | c.1305delG | p.K435fs | . | . |
| CTC6 | 14 | 89160696 | GT | G | 1 | NM_183387 | EML5 | frameshift_deleti  on | exon17 | c.2493delA | p.K831fs | . | . |
| CTC6 | 14 | 91929176 | TC | T | 1 | NM_001284281 | SMEK1 | stopgain | exon10 | c.1158delG | p.W386X | . | . |
| CTC6 | 14 | 92537353 | C | CGCTG CTGCT GCTGC TGCTG CTGCT | 0.556 | NM_001164782 | ATXN3 | nonframeshift_in sertion | exon2 | c.68_69insAGC AGCAGCAGCA GCAGCAGCAG CAGCAGCAGC AGC | p.G23delinsGA AAAAAAAAAA A | . | . |
| CTC6 | 14 | 92908514 | A | AT | 0.273 | NM_153646 | SLC24A4 | frameshift_inserti  on | exon5 | c.476dupT | p.I159fs | . | . |
| CTC6 | 14 | 93154537 | TGGC | T | 1 | NM_024832 | RIN3 | nonframeshift_de  letion | exon10 | c.2899_2901del | p.967_967del | . | . |
| CTC6 | 14 | 96779493 | GT | G | 0.286 | NM_018036 | ATG2B | frameshift_deleti  on | exon25 | c.3750delA | p.R1250fs | . | . |
| CTC6 | 14 | 96782994 | GT | G | 0.652 | NM_018036 | ATG2B | frameshift_deleti  on | exon21 | c.3236delA | p.N1079fs | . | . |
| CTC6 | 14 | 100158159 | CA | C | 0.407 | NM_006668 | CYP46A1 | frameshift_deleti  on | exon3 | c.244delA | p.K82fs | . | . |
| CTC6 | 14 | 100743936 | GA | G | 0.4 | NM_003403 | YY1 | nonframeshift_de  letion | exon5 | c.1245delA | p.X415X | . | . |
| CTC6 | 14 | 101351026 | CG | C | 0.5 | NM_001134888 | RTL1 | frameshift_deleti  on | exon1 | c.99delC | p.T33fs | . | . |
| CTC6 | 14 | 102483255 | GC | G | 0.333 | NM_001376 | DYNC1H1 | frameshift_deleti  on | exon38 | c.7768delC | p.P2590fs | . | . |
| CTC6 | 14 | 102881097 | CT | C | 0.5 | NM_001172631 | TECPR2 | frameshift_deleti  on | exon5 | c.606delT | p.S202fs | . | . |
| CTC6 | 14 | 104056559 | GA | G | 0.292 | NM_032374 | APOPT1 | frameshift_deleti  on | exon5 | c.558delA | p.G186fs | . | . |
| CTC6 | 14 | 104508416 | TC | T | 0.818 | NM_153046 | TDRD9 | frameshift_deleti  on | exon34 | c.3867delC | p.L1289fs | . | . |
| CTC6 | 14 | 105055118 | TGACGG  GCAG | T | 1 | NM_001008404 | C14orf180 | frameshift_deleti  on | exon5 | c.482_819del | p.*161fs | . | . |
| CTC6 | 14 | 105411303 | CG | C | 0.415 | NM_138420 | AHNAK2 | frameshift_deleti  on | exon7 | c.10484delC | p.S3495fs | . | . |
| CTC6 | 14 | 105414829 | AT | A | 0.513 | NM_138420 | AHNAK2 | frameshift_deleti  on | exon7 | c.6958delA | p.M2320fs | . | . |
| CTC6 | 14 | 105415004 | TG | T | 0.373 | NM_138420 | AHNAK2 | frameshift_deleti  on | exon7 | c.6783delC | p.P2261fs | . | . |
| CTC6 | 14 | 105416066 | T | TG | 0.271 | NM_138420 | AHNAK2 | frameshift_inserti  on | exon7 | c.5721dupC | p.K1908fs | . | . |
| CTC6 | 14 | 105916484 | TC | T | 0.725 | NM_001203258 | MTA1 | frameshift_deleti  on | exon5 | c.332delC | p.S111fs | . | . |
| CTC6 | 15 | 23579235 | ATGGGT  AGGGAG GTGGGG ATGGGT AGGGAG | A | 0.5 | NM_001271664 | LOC440243 | nonframeshift_de letion | exon1 | c.18_53del | p.6_18del | . | . |
| CTC6 | 15 | 26866635 | CT | C | 0.5 | NM_001191320 | GABRB3 | frameshift_deleti  on | exon2 | c.31delA | p.R11fs | . | . |
| CTC6 | 15 | 27765189 | A | AT | 0.333 | NM_033223 | GABRG3 | frameshift_inserti  on | exon7 | c.785dupT | p.I262fs | . | . |
| CTC6 | 15 | 30700168 | C | CTTG | 0.746 | NM_001282484 | GOLGA8R | nonframeshift_in  sertion | exon11 | c.813_814insCA  A | p.D272delinsQ  D | . | . |
| CTC6 | 15 | 33180429 | TG | T | 0.908 | NM_001103184 | FMN1 | frameshift_deleti  on | exon12 | c.3109delC | p.Q1037fs | . | . |
| CTC6 | 15 | 33872325 | CA | C | 0.286 | NM_001036 | RYR3 | frameshift_deleti  on | exon13 | c.1418delA | p.Q473fs | . | . |
| CTC6 | 15 | 34393990 | TAGC | T | 0.833 | NM_020154 | EMC7 | nonframeshift_de  letion | exon1 | c.48_50del | p.16_17del | . | . |
| CTC6 | 15 | 40599795 | C | CT | 0.232 | NM_001284297 | PLCB2 | frameshift_inserti  on | exon1 | c.78_79insA | p.D27fs | . | . |
| CTC6 | 15 | 40708526 | CCA | C | 0.18 | NM_001159508 | IVD | frameshift_deleti  on | exon10 | c.1023_1024del | p.A341fs | . | . |
| CTC6 | 15 | 41487997 | CA | C | 0.778 | NM_152596 | EXD1 | frameshift_deleti  on | exon7 | c.521delT | p.L174fs | . | . |
| CTC6 | 15 | 42046654 | TTGAGG  ATGA | T | 0.25 | NM_001080541 | MGA | nonframeshift_de  letion | exon17 | c.6402_6410del | p.2134_2137de  l | . | . |
| CTC6 | 15 | 42169471 | AC | A | 0.25 | NM_016642 | SPTBN5 | frameshift_deleti  on | exon18 | c.3553delG | p.V1185fs | . | . |
| CTC6 | 15 | 42749175 | GT | G | 0.406 | NM_022473 | ZNF106 | frameshift_deleti  on | exon1 | c.228delA | p.K76fs | . | . |
| CTC6 | 15 | 42977757 | AAGAG | A | 0.25 | NM_020759 | STARD9 | frameshift_deleti  on | exon23 | c.3982_3985del | p.R1328fs | . | . |
| CTC6 | 15 | 43500480 | CT | C | 1 | NM_000119 | EPB42 | frameshift_deleti  on | exon8 | c.1112delA | p.Q371fs | . | . |
| CTC6 | 15 | 43500481 | TG | T | 1 | NM_000119 | EPB42 | frameshift_deleti  on | exon8 | c.1111delC | p.Q371fs | . | . |
| CTC6 | 15 | 43668404 | AC | A | 0.3 | NM_001286414 | TUBGCP4 | frameshift_deleti  on | exon2 | c.188delC | p.T63fs | . | . |
| CTC6 | 15 | 43814604 | CA | C | 0.333 | NM_002373 | MAP1A | frameshift_deleti  on | exon4 | c.934delA | p.K312fs | . | . |
| CTC6 | 15 | 43818721 | AG | A | 0.877 | NM_002373 | MAP1A | frameshift_deleti  on | exon4 | c.5051delG | p.R1684fs | . | . |
| CTC6 | 15 | 43831733 | GA | G | 0.348 | NM_001130859 | PPIP5K1 | frameshift_deleti  on | exon28 | c.3358delT | p.S1120fs | . | . |
| CTC6 | 15 | 44890950 | G | GC | 0.522 | NM_001160227 | SPG11 | frameshift_inserti  on | exon22 | c.3770_3771ins  G | p.F1257fs | . | . |
| CTC6 | 15 | 44914463 | TA | T | 0.4 | NM_001160227 | SPG11 | frameshift_deleti  on | exon13 | c.2398delT | p.Y800fs | . | . |
| CTC6 | 15 | 45427819 | A | AT | 0.538 | NM_175940 | DUOX1 | frameshift_inserti  on | exon6 | c.644dupT | p.M215fs | . | . |
| CTC6 | 15 | 45777407 | TG | T | 1 | NM_013309 | SLC30A4 | frameshift_deleti  on | exon8 | c.1243delC | p.Q415fs | . | . |
| CTC6 | 15 | 48518722 | TG | T | 0.667 | NM_000338 | SLC12A1 | frameshift_deleti  on | exon5 | c.679delG | p.G227fs | . | . |
| CTC6 | 15 | 48595055 | CA | C | 0.889 | NM_000338 | SLC12A1 | frameshift_deleti  on | exon27 | c.3274delA | p.K1092fs | . | . |
| CTC6 | 15 | 48829888 | TG | T | 0.692 | NM_000138 | FBN1 | frameshift_deleti  on | exon7 | c.655delC | p.H219fs | . | . |
| CTC6 | 15 | 48829890 | GC | G | 0.429 | NM_000138 | FBN1 | frameshift_deleti  on | exon7 | c.653delG | p.G218fs | . | . |
| CTC6 | 15 | 49293085 | AT | A | 0.643 | NM_014701 | SECISBP2L | frameshift_deleti  on | exon14 | c.2101delA | p.I701fs | . | . |
| CTC6 | 15 | 50158627 | TC | T | 0.54 | NM_024837 | ATP8B4 | frameshift_deleti  on | exon26 | c.3081delG | p.G1027fs | . | . |
| CTC6 | 15 | 52531986 | GC | G | 0.923 | NM_018728 | MYO5C | frameshift_deleti  on | exon21 | c.2646delG | p.V882fs | . | . |

| CTC6 | 15 | 52697559 | GA | G | 0.75 | NM_000259 | MYO5A | frameshift_deleti  on | exon9 | c.977delT | p.F326fs | . | . |
| --- | --- | --- | --- | --- | --- | --- | --- | --- | --- | --- | --- | --- | --- |
| CTC6 | 15 | 57543582 | AC | A | 0.294 | NM_207040 | TCF12 | frameshift_deleti  on | exon7 | c.640delC | p.P214fs | . | . |
| CTC6 | 15 | 57565397 | A | AC | 0.382 | NM_207040 | TCF12 | frameshift_inserti  on | exon11 | c.1334dupC | p.T445fs | . | . |
| CTC6 | 15 | 63363290 | G | GA | 0.378 | NM_001018004 | TPM1 | frameshift_inserti  on | exon9 | c.775dupA | p.E258fs | . | . |
| CTC6 | 15 | 64404832 | GA | G | 0.571 | NM_001242933 | SNX1 | frameshift_deleti  on | exon2 | c.221delA | p.E74fs | . | . |
| CTC6 | 15 | 64429120 | TCA | T | 0.315 | NM_148955 | SNX1 | frameshift_deleti  on | exon12 | c.1313_1314del | p.S438fs | . | . |
| CTC6 | 15 | 65345475 | TC | T | 0.364 | NM_178859 | SLC51B | frameshift_deleti  on | exon4 | c.361delC | p.P121fs | . | . |
| CTC6 | 15 | 66022002 | G | GA | 0.389 | NM_001144823 | DENND4A | frameshift_inserti  on | exon10 | c.1180dupT | p.S394fs | . | . |
| CTC6 | 15 | 69331225 | CT | C | 0.921 | NM_001184779 | NOX5 | frameshift_deleti  on | exon9 | c.1317delT | p.P439fs | . | . |
| CTC6 | 15 | 69561544 | G | GA | 0.346 | NM_015554 | GLCE | frameshift_inserti  on | exon5 | c.1816dupA | p.W605fs | . | . |
| CTC6 | 15 | 72118975 | GT | G | 0.5 | NM_006901 | MYO9A | frameshift_deleti  on | exon42 | c.7592delA | p.D2531fs | . | . |
| CTC6 | 15 | 73045096 | G | GAA | 0.218 | NM_031284 | ADPGK | frameshift_inserti  on | exon7 | c.1076_1077ins  TT | p.F359fs | . | . |
| CTC6 | 15 | 73581489 | CA | C | 0.452 | NM_001172624 | NEO1 | frameshift_deleti  on | exon24 | c.3620delA | p.H1207fs | . | . |
| CTC6 | 15 | 74536400 | TAAG | T | 0.93 | NM_025055 | CCDC33 | nonframeshift_de  letion | exon2 | c.97_99del | p.33_33del | . | . |
| CTC6 | 15 | 75045530 | CA | C | 1 | NM_000761 | CYP1A2 | frameshift_deleti  on | exon6 | c.1173delA | p.T391fs | . | . |
| CTC6 | 15 | 75115965 | A | AT | 0.439 | NM_021819 | LMAN1L | frameshift_inserti  on | exon12 | c.1266dupT | p.H422fs | . | . |
| CTC6 | 15 | 75130666 | TC | T | 0.287 | NM_001099436 | ULK3 | frameshift_deleti  on | exon12 | c.1315delG | p.E439fs | . | . |
| CTC6 | 15 | 78894133 | GT | G | 0.2 | NM_000743 | CHRNA3 | frameshift_deleti  on | exon5 | c.850delA | p.T284fs | . | . |
| CTC6 | 15 | 81517997 | TC | T | 0.4 | NM_172217 | IL16 | frameshift_deleti  on | exon1 | c.258delC | p.L86fs | . | . |
| CTC6 | 15 | 81558011 | TA | T | 0.34 | NM_172217 | IL16 | frameshift_deleti  on | exon3 | c.434delA | p.Y145fs | . | . |
| CTC6 | 15 | 81630197 | TG | T | 0.333 | NM_001080532 | TMC3 | frameshift_deleti  on | exon19 | c.2149delC | p.Q717fs | . | . |
| CTC6 | 15 | 81631011 | GC | G | 0.667 | NM_001080532 | TMC3 | frameshift_deleti  on | exon18 | c.2065delG | p.A689fs | . | . |
| CTC6 | 15 | 85186876 | ACTTGG  CTCCGT GTTCCAT | A | 0.109 | NM_032856 | WDR73 | nonframeshift_de letion | exon8 | c.944_961del | p.315_321del | . | . |
| CTC6 | 15 | 85327008 | TC | T | 0.632 | NM_014630 | ZNF592 | frameshift_deleti  on | exon4 | c.1103delC | p.S368fs | . | . |
| CTC6 | 15 | 85664142 | AT | A | 0.5 | NM_173454 | PDE8A | frameshift_deleti  on | exon17 | c.1712delT | p.I571fs | . | . |
| CTC6 | 15 | 86265342 | GT | G | 0.25 | NM_001270546 | AKAP13 | frameshift_deleti  on | exon16 | c.2237delT | p.V746fs | . | . |
| CTC6 | 15 | 90334260 | TG | T | 0.434 | NM_001150 | ANPEP | frameshift_deleti  on | exon19 | c.2592delC | p.I864fs | . | . |
| CTC6 | 15 | 90771538 | TG | T | 0.474 | NM_198925 | SEMA4B | frameshift_deleti  on | exon14 | c.2178delG | p.V726fs | . | . |
| CTC6 | 15 | 99511804 | A | AC | 0.393 | NM_001102612 | PGPEP1L | frameshift_inserti  on | exon5 | c.493dupG | p.V165fs | . | . |
| CTC6 | 15 | 99759208 | A | AT | 0.227 | NM_001288616 | TTC23 | frameshift_inserti  on | exon5 | c.349dupA | p.I117fs | . | . |
| CTC6 | 15 | 100252709 | CCAGCA  G | C | 1 | NM_001130928 | MEF2A | nonframeshift_de  letion | exon8 | c.1024_1029del | p.342_343del | . | . |
| CTC6 | 16 | 450140 | C | CAG | 0.965 | NM_001286435 | NME4 | frameshift_inserti  on | exon5 | c.472_473insA  G | p.R158fs | . | . |
| CTC6 | 16 | 1306559 | AG | A | 1 | NM_012217 | TPSD1 | frameshift_deleti  on | exon2 | c.126delG | p.Q42fs | . | . |
| CTC6 | 16 | 1536182 | CT | C | 0.583 | NM_001013658 | PTX4 | frameshift_deleti  on | exon3 | c.1179delA | p.G393fs | . | . |
| CTC6 | 16 | 1712578 | CG | C | 0.333 | NM_020825 | CRAMP1L | frameshift_deleti  on | exon11 | c.2502delG | p.P834fs | . | . |
| CTC6 | 16 | 1837790 | TC | T | 0.263 | NM_001284502 | NUBP2 | frameshift_deleti  on | exon3 | c.25delC | p.P9fs | . | . |
| CTC6 | 16 | 2810344 | TG | T | 0.269 | NM_016333 | SRRM2 | frameshift_deleti  on | exon10 | c.877delG | p.G293fs | . | . |
| CTC6 | 16 | 2819118 | TTCC | T | 0.2 | NM_016333 | SRRM2 | nonframeshift_de  letion | exon12 | c.7855_7857del | p.2619_2619de  l | . | . |
| CTC6 | 16 | 3025501 | GCAC | G | 0.189 | NM_001258450 | PKMYT1 | nonframeshift_de  letion | exon4 | c.481_483del | p.161_161del | . | . |
| CTC6 | 16 | 3117588 | CA | C | 0.931 | NM_001012636 | IL32 | frameshift_deleti  on | exon3 | c.89delA | p.Q30fs | . | . |
| CTC6 | 16 | 3786070 | CT | C | 0.571 | NM_001079846 | CREBBP | frameshift_deleti  on | exon27 | c.4580delA | p.K1527fs | . | . |
| CTC6 | 16 | 5058601 | CAG | C | 0.2 | NM_014692 | SEC14L5 | frameshift_deleti  on | exon14 | c.1753_1754del | p.S585fs | . | . |
| CTC6 | 16 | 10274031 | TG | T | 1 | NM_001134407 | GRIN2A | frameshift_deleti  on | exon2 | c.237delC | p.P79fs | . | . |
| CTC6 | 16 | 11650585 | AT | A | 0.578 | NM_001136472 | LITAF | frameshift_deleti  on | exon2 | c.1delA | p.M1fs | . | . |
| CTC6 | 16 | 14334252 | CCAG | C | 0.931 | NM_014048 | MKL2 | nonframeshift_de  letion | exon10 | c.991_993del | p.331_331del | . | . |
| CTC6 | 16 | 15127254 | AGG | A | 0.791 | NM_001285444 | PDXDC1 | frameshift_deleti  on | exon18 | c.1730_1731del | p.R577fs | . | . |
| CTC6 | 16 | 17221576 | CT | C | 0.9 | NM_022166 | XYLT1 | frameshift_deleti  on | exon10 | c.2169delA | p.K723fs | . | . |
| CTC6 | 16 | 18846284 | G | GA | 0.241 | NM_015092 | SMG1 | frameshift_inserti  on | exon49 | c.8259dupT | p.H2754fs | . | . |
| CTC6 | 16 | 19547579 | A | AG | 0.176 | NM_001199022 | CCP110 | frameshift_inserti  on | exon4 | c.589dupG | p.Q196fs | . | . |
| CTC6 | 16 | 19603133 | AC | A | 0.656 | NM_020314 | C16orf62 | frameshift_deleti  on | exon8 | c.929delC | p.T310fs | . | . |
| CTC6 | 16 | 19693633 | GA | G | 0.233 | NM_020314 | C16orf62 | frameshift_deleti  on | exon28 | c.2716delA | p.K906fs | . | . |
| CTC6 | 16 | 21038386 | C | CG | 0.375 | NM_017539 | DNAH3 | frameshift_inserti  on | exon38 | c.5502dupC | p.A1835fs | . | . |
| CTC6 | 16 | 21666551 | GT | G | 0.3 | NM_001077180 | METTL9 | frameshift_deleti  on | exon5 | c.753delT | p.G251fs | . | . |
| CTC6 | 16 | 22269976 | A | AG | 0.263 | NM_013302 | EEF2K | frameshift_inserti  on | exon10 | c.1191_1192ins  G | p.P397fs | . | . |
| CTC6 | 16 | 23700858 | T | TG | 0.247 | NM_005030 | PLK1 | frameshift_inserti  on | exon9 | c.1470dupG | p.L490fs | . | . |
| CTC6 | 16 | 24358089 | C | CT | 0.235 | NM_006539 | CACNG3 | frameshift_inserti  on | exon2 | c.247dupT | p.H82fs | . | . |
| CTC6 | 16 | 24570935 | TG | T | 0.214 | NM_006910 | RBBP6 | frameshift_deleti  on | exon8 | c.785delG | p.C262fs | . | . |

| CTC6 | 16 | 24583232 | AC | A | 0.727 | NM_018703 | RBBP6 | frameshift_deleti  on | exon17 | c.4744delC | p.P1582fs | . | . |
| --- | --- | --- | --- | --- | --- | --- | --- | --- | --- | --- | --- | --- | --- |
| CTC6 | 16 | 24788422 | AGCAGC  CACAGC | A | 0.357 | NM_014494 | TNRC6A | nonframeshift_de  letion | exon5 | c.333_344del | p.111_115del | . | . |
| CTC6 | 16 | 27487846 | G | GA | 0.929 | NM_001286242 | GTF3C1 | frameshift_inserti  on | exon29 | c.4278dupT | p.L1427fs | . | . |
| CTC6 | 16 | 27487849 | A | AG | 0.292 | NM_001286242 | GTF3C1 | frameshift_inserti  on | exon29 | c.4275dupC | p.F1426fs | . | . |
| CTC6 | 16 | 28157474 | CA | C | 0.8 | NM_015171 | XPO6 | frameshift_deleti  on | exon9 | c.1274delT | p.L425fs | . | . |
| CTC6 | 16 | 28947851 | TC | T | 0.625 | NM_001178098 | CD19 | frameshift_deleti  on | exon7 | c.1015delC | p.P339fs | . | . |
| CTC6 | 16 | 29889686 | TG | T | 0.462 | NM_001114100 | SEZ6L2 | frameshift_deleti  on | exon8 | c.1291delC | p.Q431fs | . | . |
| CTC6 | 16 | 29994614 | CA | C | 0.264 | NM_001252043 | TAOK2 | frameshift_deleti  on | exon12 | c.1222delA | p.T408fs | . | . |
| CTC6 | 16 | 29994897 | CA | C | 0.567 | NM_001252043 | TAOK2 | frameshift_deleti  on | exon13 | c.1335delA | p.P445fs | . | . |
| CTC6 | 16 | 30365326 | TA | T | 0.224 | NM_001243646 | CD2BP2 | frameshift_deleti  on | exon3 | c.270delT | p.F90fs | . | . |
| CTC6 | 16 | 30522219 | GTT | G | 0.122 | NM_001114380 | ITGAL | frameshift_deleti  on | exon21 | c.2386_2387del | p.F796fs | . | . |
| CTC6 | 16 | 30970419 | AG | A | 0.136 | NM_014712 | SETD1A | frameshift_deleti  on | exon3 | c.159delG | p.K53fs | . | . |
| CTC6 | 16 | 30982808 | ATCC | A | 0.375 | NM_014712 | SETD1A | nonframeshift_de  letion | exon13 | c.3127_3129del | p.1043_1043de  l | . | . |
| CTC6 | 16 | 31366581 | CA | C | 0.667 | NM_000887 | ITGAX | frameshift_deleti  on | exon1 | c.7delA | p.R3fs | . | . |
| CTC6 | 16 | 31485768 | CA | C | 0.416 | NM_001042454 | TGFB1I1 | frameshift_deleti  on | exon6 | c.512delA | p.Q171fs | . | . |
| CTC6 | 16 | 31504925 | TG | T | 0.16 | NM_022744 | C16orf58 | frameshift_deleti  on | exon8 | c.956delC | p.T319fs | . | . |
| CTC6 | 16 | 31926195 | GT | G | 0.5 | NM_003414 | ZNF267 | frameshift_deleti  on | exon4 | c.626delT | p.V209fs | . | . |
| CTC6 | 16 | 31926326 | CT | C | 0.643 | NM_003414 | ZNF267 | frameshift_deleti  on | exon4 | c.757delT | p.F253fs | . | . |
| CTC6 | 16 | 47117425 | A | AG | 0.364 | NM_001201477 | NETO2 | frameshift_inserti  on | exon9 | c.1263dupC | p.S422fs | . | . |
| CTC6 | 16 | 51175655 | CGCT | C | 0.228 | NM_001127892 | SALL1 | nonframeshift_de  letion | exon2 | c.184_186del | p.62_62del | . | . |
| CTC6 | 16 | 56871587 | CG | C | 0.429 | NM_001242795 | NUP93 | frameshift_deleti  on | exon16 | c.1599delG | p.P533fs | . | . |
| CTC6 | 16 | 56913041 | TG | T | 0.286 | NM_000339 | SLC12A3 | frameshift_deleti  on | exon10 | c.1238delG | p.W413fs | . | . |
| CTC6 | 16 | 57057731 | AC | A | 0.706 | NM_032206 | NLRC5 | frameshift_deleti  on | exon4 | c.391delC | p.P131fs | . | . |
| CTC6 | 16 | 57265106 | C | CA | 0.784 | NM_133368 | RSPRY1 | frameshift_inserti  on | exon13 | c.1405dupA | p.F468fs | . | . |
| CTC6 | 16 | 57465154 | GC | G | 1 | NM_020313 | CIAPIN1 | frameshift_deleti  on | exon7 | c.654delG | p.L218fs | . | . |
| CTC6 | 16 | 57509017 | TG | T | 0.219 | NM_018110 | DOK4 | frameshift_deleti  on | exon5 | c.403delC | p.Q135fs | . | . |
| CTC6 | 16 | 57731948 | GA | G | 0.938 | NM_001289163 | CCDC135 | frameshift_deleti  on | exon2 | c.88delA | p.K30fs | . | . |
| CTC6 | 16 | 57847634 | A | ATGCA  GGTG | 1 | NM_001278081 | LOC388282 | frameshift_inserti  on | exon2 | c.137_138insTG  CAGGTG | p.Q46fs | . | . |
| CTC6 | 16 | 57953114 | CGG | C | 0.176 | NM_001286130 | CNGB1 | frameshift_deleti  on | exon20 | c.1826_1827del | p.P609fs | . | . |
| CTC6 | 16 | 58079095 | CG | C | 0.935 | NM_002428 | MMP15 | frameshift_deleti  on | exon10 | c.1756delG | p.G586fs | . | . |
| CTC6 | 16 | 58538175 | AG | A | 0.279 | NM_001242834 | NDRG4 | frameshift_deleti  on | exon3 | c.300delG | p.Q100fs | . | . |
| CTC6 | 16 | 66423350 | CG | C | 0.5 | NM_001795 | CDH5 | frameshift_deleti  on | exon5 | c.707delG | p.R236fs | . | . |
| CTC6 | 16 | 67183960 | A | ACC | 0.304 | NM_033309 | B3GNT9 | frameshift_inserti  on | exon2 | c.428_429insG  G | p.G143fs | . | . |
| CTC6 | 16 | 67202989 | C | CA | 0.345 | NM_001040667 | HSF4 | frameshift_inserti  on | exon13 | c.1240dupA | p.D413fs | . | . |
| CTC6 | 16 | 67237473 | C | CT | 0.828 | NM_024712 | ELMO3 | frameshift_inserti  on | exon19 | c.2096_2097ins  T | p.P699fs | . | . |
| CTC6 | 16 | 67693667 | CTG | C | 0.218 | NM_001082486 | ACD | frameshift_deleti  on | exon3 | c.530_531del | p.T177fs | . | . |
| CTC6 | 16 | 67763278 | CGAG | C | 0.146 | NM_020850 | RANBP10 | nonframeshift_de  letion | exon10 | c.1254_1256del | p.418_419del | . | . |
| CTC6 | 16 | 67969995 | GC | G | 0.523 | NM_002801 | PSMB10 | frameshift_deleti  on | exon4 | c.253delG | p.A85fs | . | . |
| CTC6 | 16 | 67977953 | GC | G | 0.203 | NM_000229 | LCAT | frameshift_deleti  on | exon1 | c.51delG | p.L17fs | . | . |
| CTC6 | 16 | 68389681 | AC | A | 0.25 | NM_001184824 | PRMT7 | frameshift_deleti  on | exon15 | c.1557delC | p.Y519fs | . | . |
| CTC6 | 16 | 68398672 | AG | A | 0.167 | NM_018667 | SMPD3 | frameshift_deleti  on | exon5 | c.1536delC | p.N512fs | . | . |
| CTC6 | 16 | 68856027 | TA | T | 0.275 | NM_004360 | CDH1 | frameshift_deleti  on | exon12 | c.1836delA | p.I612fs | . | . |
| CTC6 | 16 | 70398963 | GA | G | 0.44 | NM_018332 | DDX19A | frameshift_deleti  on | exon7 | c.534delA | p.G178fs | . | . |
| CTC6 | 16 | 70902478 | GT | G | 0.556 | NM_001270974 | HYDIN | frameshift_deleti  on | exon66 | c.11304delA | p.K3768fs | . | . |
| CTC6 | 16 | 71482961 | AT | A | 0.242 | NM_145911 | ZNF23 | frameshift_deleti  on | exon6 | c.966delA | p.K322fs | . | . |
| CTC6 | 16 | 71956511 | AATGCC  C | A | 0.188 | NM_001270979 | IST1 | nonframeshift_de  letion | exon3 | c.244_249del | p.82_83del | . | . |
| CTC6 | 16 | 75240031 | TC | T | 0.667 | NM_001025200 | CTRB2 | frameshift_deleti  on | exon2 | c.112delG | p.E38fs | . | . |
| CTC6 | 16 | 82069190 | C | CT | 0.682 | NM_002153 | HSD17B2 | frameshift_inserti  on | exon1 | c.162dupT | p.P54fs | . | . |
| CTC6 | 16 | 87637893 | CCTG | C | 0.345 | NM_001271604 | JPH3 | nonframeshift_de  letion | exon2 | c.431_433del | p.144_145del | . | . |
| CTC6 | 16 | 88066729 | AC | A | 0.556 | NM_001173540 | BANP | frameshift_deleti  on | exon8 | c.980delC | p.T327fs | . | . |
| CTC6 | 16 | 88494467 | AC | A | 0.727 | NM_001127464 | ZNF469 | frameshift_deleti  on | exon1 | c.590delC | p.T197fs | . | . |
| CTC6 | 16 | 88780634 | AGGTGT  G | A | 1 | NM_001012759 | CTU2 | frameshift_deleti  on | exon10 | c.1097_1097del | p.R366fs | . | . |
| CTC6 | 16 | 88799131 | TG | T | 0.889 | NM_001142864 | PIEZO1 | frameshift_deleti  on | exon20 | c.2673delC | p.P891fs | . | . |
| CTC6 | 16 | 89254656 | AC | A | 0.297 | NM_004933 | CDH15 | frameshift_deleti  on | exon7 | c.942delC | p.D314fs | . | . |
| CTC6 | 16 | 89291210 | G | GGTGA | 1 | NM_001201407 | ZNF778 | frameshift_inserti  on | exon5 | c.328_329insGT  GA | p.E110fs | . | . |
| CTC6 | 16 | 89350086 | A | AG | 0.307 | NM_001256183 | ANKRD11 | frameshift_inserti  on | exon9 | c.2863dupC | p.L955fs | . | . |

| CTC6 | 16 | 90095597 | T | TGGGG  CAGCC TACGG GGCAG | 0.889 | NM_001214 | C16orf3 | nonframeshift_in sertion | exon1 | c.153_154insG  CAGCCTGCCC CGTAGGCTGC CCC | p.I52delinsAAC PVGCPI | . | . |
| --- | --- | --- | --- | --- | --- | --- | --- | --- | --- | --- | --- | --- | --- |
| CTC6 | 17 | 914015 | CT | C | 1 | NM_001256847 | ABR | frameshift_deleti  on | exon5 | c.542delA | p.E181fs | . | . |
| CTC6 | 17 | 961235 | TTC | T | 0.406 | NM_001282149 | ABR | frameshift_deleti  on | exon7 | c.700_701del | p.E234fs | . | . |
| CTC6 | 17 | 1582972 | GC | G | 0.545 | NM_006445 | PRPF8 | frameshift_deleti  on | exon9 | c.1219delG | p.A407fs | . | . |
| CTC6 | 17 | 1629564 | TC | T | 0.352 | NM_001163809 | WDR81 | frameshift_deleti  on | exon1 | c.1312delC | p.P438fs | . | . |
| CTC6 | 17 | 1657757 | CT | C | 0.786 | NM_001165921 | SERPINF2 | frameshift_deleti  on | exon9 | c.1214delT | p.L405fs | . | . |
| CTC6 | 17 | 2381169 | TA | T | 0.521 | NM_024086 | METTL16 | frameshift_deleti  on | exon3 | c.138delT | p.F46fs | . | . |
| CTC6 | 17 | 3916895 | CA | C | 0.286 | NM_015113 | ZZEF1 | stopgain | exon52 | c.8426delT | p.L2809X | . | . |
| CTC6 | 17 | 4027330 | CA | C | 0.25 | NM_015113 | ZZEF1 | frameshift_deleti on | exon2 | c.369delT | p.F123fs | . | . |
| CTC6 | 17 | 4462340 | CA | C | 0.333 | NM_153338 | GGT6 | frameshift_deleti  on | exon3 | c.355delT | p.W119fs | . | . |
| CTC6 | 17 | 4789806 | ACC | A | 0.279 | NM_001024937 | MINK1 | frameshift_deleti  on | exon10 | c.835_836del | p.P279fs | . | . |
| CTC6 | 17 | 4803062 | AG | A | 1 | NM_001145536 | C17orf107 | frameshift_deleti  on | exon1 | c.6delG | p.K2fs | . | . |
| CTC6 | 17 | 5290959 | GT | G | 0.724 | NM_002532 | NUP88 | frameshift_deleti  on | exon13 | c.1800delA | p.K600fs | . | . |
| CTC6 | 17 | 5486146 | AG | A | 0.643 | NM_001033053 | NLRP1 | frameshift_deleti  on | exon2 | c.291delC | p.P97fs | . | . |
| CTC6 | 17 | 6373654 | TG | T | 0.5 | NM_001165966 | PITPNM3 | frameshift_deleti  on | exon12 | c.1590delC | p.P530fs | . | . |
| CTC6 | 17 | 6555546 | C | CG | 1 | NM_001105520 | C17orf100 | frameshift_inserti  on | exon1 | c.314dupG | p.R105fs | . | . |
| CTC6 | 17 | 6616590 | CG | C | 0.808 | NM_001143838 | SLC13A5 | frameshift_deleti  on | exon1 | c.62delC | p.P21fs | . | . |
| CTC6 | 17 | 6943131 | TG | T | 0.386 | NM_201566 | SLC16A13 | frameshift_deleti  on | exon4 | c.1132delG | p.G378fs | . | . |
| CTC6 | 17 | 7139910 | TG | T | 0.455 | NM_001284518 | PHF23 | frameshift_deleti  on | exon4 | c.323delC | p.P108fs | . | . |
| CTC6 | 17 | 7386141 | GC | G | 0.267 | NM_001102614 | SLC35G6 | frameshift_deleti  on | exon2 | c.839delC | p.A280fs | . | . |
| CTC6 | 17 | 7804199 | AT | A | 0.583 | NM_001005271 | CHD3 | frameshift_deleti  on | exon19 | c.3186delT | p.N1062fs | . | . |
| CTC6 | 17 | 7807868 | CG | C | 0.275 | NM_001005271 | CHD3 | frameshift_deleti  on | exon25 | c.4181delG | p.R1394fs | . | . |
| CTC6 | 17 | 7918801 | CCG | C | 0.889 | NM_000180 | GUCY2D | frameshift_deleti  on | exon15 | c.2926_2927del | p.R976fs | . | . |
| CTC6 | 17 | 8064949 | TG | T | 0.184 | NM_014232 | VAMP2 | frameshift_deleti  on | exon3 | c.258delC | p.R86fs | . | . |
| CTC6 | 17 | 8351953 | GA | G | 0.316 | NM_001025579 | NDEL1 | frameshift_deleti  on | exon5 | c.476delA | p.E159fs | . | . |
| CTC6 | 17 | 9460801 | CT | C | 0.306 | NM_004853 | STX8 | frameshift_deleti  on | exon3 | c.161delA | p.K54fs | . | . |
| CTC6 | 17 | 9684804 | TG | T | 0.364 | NM_001105571 | DHRS7C | frameshift_deleti  on | exon2 | c.261delC | p.P87fs | . | . |
| CTC6 | 17 | 10224996 | TG | T | 0.45 | NM_003802 | MYH13 | frameshift_deleti  on | exon24 | c.2963delC | p.A988fs | . | . |
| CTC6 | 17 | 10541683 | G | GT | 0.8 | NM_002470 | MYH3 | frameshift_inserti  on | exon27 | c.3405dupA | p.Q1136fs | . | . |
| CTC6 | 17 | 11687717 | CGG | C | 0.195 | NM_001372 | DNAH9 | frameshift_deleti  on | exon41 | c.7923_7924del | p.P2641fs | . | . |
| CTC6 | 17 | 15878045 | TG | T | 0.286 | NM_000676 | ADORA2B | frameshift_deleti  on | exon2 | c.389delG | p.W130fs | . | . |
| CTC6 | 17 | 16068454 | GT | G | 0.273 | NM_001190438 | NCOR1 | frameshift_deleti  on | exon2 | c.129delA | p.K43fs | . | . |
| CTC6 | 17 | 16527395 | AT | A | 0.583 | NM_020787 | ZNF624 | frameshift_deleti  on | exon6 | c.804delA | p.K268fs | . | . |
| CTC6 | 17 | 17699992 | CGCA | C | 0.4 | NM_030665 | RAI1 | nonframeshift_de  letion | exon3 | c.3731_3733del | p.1244_1245de  l | . | . |
| CTC6 | 17 | 18001650 | ACG | A | 0.139 | NM_001388 | DRG2 | frameshift_deleti  on | exon3 | c.293_294del | p.T98fs | . | . |
| CTC6 | 17 | 18059603 | CA | C | 0.319 | NM_016239 | MYO15A | frameshift_deleti  on | exon47 | c.8555delA | p.H2852fs | . | . |
| CTC6 | 17 | 18149743 | TG | T | 0.25 | NM_001256265 | FLII | frameshift_deleti  on | exon23 | c.2919delC | p.P973fs | . | . |
| CTC6 | 17 | 18661648 | CG | C | 0.474 | NM_001267585 | FBXW10 | frameshift_deleti  on | exon7 | c.1264delG | p.G422fs | . | . |
| CTC6 | 17 | 19583319 | TCTG | T | 0.18 | NM_001099646 | SLC47A2 | nonframeshift_de  letion | exon16 | c.1423_1425del | p.475_475del | . | . |
| CTC6 | 17 | 20483647 | GA | G | 0.333 | NM_001190790 | CDRT15L2 | frameshift_deleti  on | exon2 | c.452delA | p.E151fs | . | . |
| CTC6 | 17 | 26692224 | A | AG | 1 | NM_001080837 | SEBOX | frameshift_inserti  on | exon1 | c.27dupC | p.W10fs | . | . |
| CTC6 | 17 | 26862048 | AC | A | 1 | NM_003593 | FOXN1 | frameshift_deleti  on | exon7 | c.1460delC | p.T487fs | . | . |
| CTC6 | 17 | 27001315 | GA | G | 0.571 | NM_003170 | SUPT6H | frameshift_deleti  on | exon3 | c.125delA | p.E42fs | . | . |
| CTC6 | 17 | 27068171 | CA | C | 0.203 | NM_178170 | NEK8 | frameshift_deleti  on | exon13 | c.1809delA | p.A603fs | . | . |
| CTC6 | 17 | 27233979 | CA | C | 0.25 | NM_001033561 | PHF12 | frameshift_deleti  on | exon14 | c.2574delT | p.S858fs | . | . |
| CTC6 | 17 | 27421998 | CCTTCTC  CCGCTG CAGCTT CTCCCG | C | 0.15 | NM_078471 | MYO18A | nonframeshift_de letion | exon29 | c.4435_4464del | p.1479_1488de l | . | . |
| CTC6 | 17 | 27829669 | AC | A | 0.9 | NM_020791 | TAOK1 | frameshift_deleti  on | exon13 | c.1267delC | p.P423fs | . | . |
| CTC6 | 17 | 27889788 | AG | A | 1 | NM_198147 | ABHD15 | frameshift_deleti  on | exon2 | c.1197delC | p.P399fs | . | . |
| CTC6 | 17 | 29276354 | TA | T | 0.375 | NM_018404 | ADAP2 | frameshift_deleti  on | exon7 | c.708delA | p.L236fs | . | . |
| CTC6 | 17 | 31039006 | C | CT | 0.293 | NM_015194 | MYO1D | frameshift_inserti  on | exon16 | c.2120dupA | p.K707fs | . | . |
| CTC6 | 17 | 31091908 | GTC | G | 0.189 | NM_015194 | MYO1D | frameshift_deleti  on | exon8 | c.1021_1022del | p.D341fs | . | . |
| CTC6 | 17 | 33679684 | G | GC | 0.714 | NM_152270 | SLFN11 | frameshift_inserti  on | exon5 | c.2396_2397ins  G | p.F799fs | . | . |
| CTC6 | 17 | 34251648 | GC | G | 0.4 | NM_001163122 | RDM1 | frameshift_deleti  on | exon3 | c.458delG | p.G153fs | . | . |
| CTC6 | 17 | 35298129 | G | GA | 0.421 | NM_005568 | LHX1 | frameshift_inserti  on | exon3 | c.620_621insA | p.R207fs | . | . |
| CTC6 | 17 | 36484621 | GC | G | 0.188 | NM_001004334 | GPR179 | frameshift_deleti  on | exon11 | c.4830delG | p.V1610fs | . | . |

| CTC6 | 17 | 36622694 | TC | T | 0.733 | NM_001199417 | ARHGAP23 | frameshift_deleti  on | exon7 | c.771delC | p.F257fs | . | . |
| --- | --- | --- | --- | --- | --- | --- | --- | --- | --- | --- | --- | --- | --- |
| CTC6 | 17 | 36878298 | CG | C | 0.438 | NM_005937 | MLLT6 | frameshift_deleti  on | exon17 | c.2611delG | p.G871fs | . | . |
| CTC6 | 17 | 38062476 | TC | T | 0.205 | NM_018530 | GSDMB | frameshift_deleti  on | exon5 | c.709delG | p.E237fs | . | . |
| CTC6 | 17 | 38447818 | G | GA | 0.229 | NM_001254 | CDC6 | frameshift_inserti  on | exon4 | c.559dupA | p.L186fs | . | . |
| CTC6 | 17 | 38933878 | ACC | A | 0.172 | NM_181537 | KRT27 | frameshift_deleti  on | exon6 | c.1077_1078del | p.Q359fs | . | . |
| CTC6 | 17 | 38955945 | TC | T | 0.636 | NM_181535 | KRT28 | frameshift_deleti  on | exon1 | c.200delG | p.G67fs | . | . |
| CTC6 | 17 | 39023055 | AG | A | 0.344 | NM_000223 | KRT12 | frameshift_deleti  on | exon1 | c.383delC | p.T128fs | . | . |
| CTC6 | 17 | 39041177 | CT | C | 0.989 | NM_019010 | KRT20 | frameshift_deleti  on | exon1 | c.260delA | p.K87fs | . | . |
| CTC6 | 17 | 39538235 | G | GA | 0.463 | NM_021013 | KRT34 | frameshift_inserti  on | exon1 | c.389dupT | p.I130fs | . | . |
| CTC6 | 17 | 39550299 | CG | C | 0.5 | NM_002277 | KRT31 | frameshift_deleti  on | exon7 | c.1219delC | p.R407fs | . | . |
| CTC6 | 17 | 39684307 | AGCC | A | 0.55 | NM_002276 | KRT19 | nonframeshift_de  letion | exon1 | c.190_192del | p.64_64del | . | . |
| CTC6 | 17 | 39740089 | G | GA | 0.151 | NM_000526 | KRT14 | frameshift_inserti  on | exon4 | c.849dupT | p.L284fs | . | . |
| CTC6 | 17 | 40125727 | A | AT | 0.32 | NM_033133 | CNP | frameshift_inserti  on | exon4 | c.1052dupT | p.I351fs | . | . |
| CTC6 | 17 | 40152506 | CT | C | 0.667 | NM_003315 | DNAJC7 | frameshift_deleti  on | exon2 | c.159delA | p.K53fs | . | . |
| CTC6 | 17 | 40452229 | TG | T | 0.349 | NM_001288719 | STAT5A | stopgain | exon6 | c.674delG | p.W225X | . | . |
| CTC6 | 17 | 40490761 | TG | T | 0.667 | NM_003150 | STAT3 | frameshift_deleti on | exon6 | c.537delC | p.L179fs | . | . |
| CTC6 | 17 | 40695254 | TG | T | 0.378 | NM_000263 | NAGLU | frameshift_deleti  on | exon6 | c.1231delG | p.G411fs | . | . |
| CTC6 | 17 | 40705481 | TG | T | 0.893 | NM_000413 | HSD17B1 | frameshift_deleti  on | exon3 | c.291delG | p.L97fs | . | . |
| CTC6 | 17 | 40716873 | AG | A | 0.277 | NM_025233 | COASY | frameshift_deleti  on | exon4 | c.1195delG | p.G399fs | . | . |
| CTC6 | 17 | 40946873 | TCTC | T | 0.5 | NM_032387 | WNK4 | nonframeshift_de  letion | exon14 | c.2435_2437del | p.812_813del | . | . |
| CTC6 | 17 | 40961480 | CG | C | 0.524 | NM_173478 | CNTD1 | frameshift_deleti  on | exon7 | c.921delG | p.P307fs | . | . |
| CTC6 | 17 | 40970627 | TG | T | 0.533 | NM_003766 | BECN1 | frameshift_deleti  on | exon6 | c.422delC | p.T141fs | . | . |
| CTC6 | 17 | 40986814 | TA | T | 0.325 | NM_005789 | PSME3 | frameshift_deleti  on | exon4 | c.165delA | p.L55fs | . | . |
| CTC6 | 17 | 41056014 | GC | G | 0.381 | NM_000151 | G6PC | frameshift_deleti  on | exon2 | c.298delC | p.P100fs | . | . |
| CTC6 | 17 | 41367876 | TC | T | 0.933 | NM_145041 | TMEM106A | frameshift_deleti  on | exon5 | c.346delC | p.P116fs | . | . |
| CTC6 | 17 | 41901342 | AT | A | 0.328 | NM_001932 | MPP3 | frameshift_deleti  on | exon10 | c.640delA | p.I214fs | . | . |
| CTC6 | 17 | 42987531 | TA | T | 0.92 | NM_001131019 | GFAP | frameshift_deleti  on | exon8 | c.1268delT | p.V423fs | . | . |
| CTC6 | 17 | 43215337 | GT | G | 0.364 | NM_024722 | ACBD4 | frameshift_deleti  on | exon7 | c.632delT | p.V211fs | . | . |
| CTC6 | 17 | 43473457 | GC | G | 0.25 | NM_001282290 | ARHGAP27 | frameshift_deleti  on | exon15 | c.2277delG | p.G759fs | . | . |
| CTC6 | 17 | 44408925 | GCT | G | 0.5 | NM_014834 | LRRC37A | frameshift_deleti  on | exon9 | c.4283_4284del | p.A1428fs | . | . |
| CTC6 | 17 | 46606996 | TG | T | 0.953 | NM_002144 | HOXB1 | frameshift_deleti  on | exon2 | c.818delC | p.P273fs | . | . |
| CTC6 | 17 | 46607967 | A | AG | 0.329 | NM_002144 | HOXB1 | frameshift_inserti  on | exon1 | c.299dupC | p.P100fs | . | . |
| CTC6 | 17 | 46863538 | T | TG | 0.364 | NM_173623 | TTLL6 | frameshift_inserti  on | exon5 | c.827_828insC | p.Q276fs | . | . |
| CTC6 | 17 | 47301944 | TC | T | 0.318 | NM_001143804 | PHOSPHO1 | frameshift_deleti  on | exon3 | c.542delG | p.G181fs | . | . |
| CTC6 | 17 | 48247543 | AC | A | 1 | NM_000023 | SGCA | frameshift_deleti  on | exon7 | c.788delC | p.T263fs | . | . |
| CTC6 | 17 | 48266769 | TCG | T | 0.176 | NM_000088 | COL1A1 | frameshift_deleti  on | exon39 | c.2796_2797del | p.G932fs | . | . |
| CTC6 | 17 | 48269359 | AG | A | 0.255 | NM_000088 | COL1A1 | frameshift_deleti  on | exon30 | c.2009delC | p.P670fs | . | . |
| CTC6 | 17 | 48734113 | CT | C | 0.312 | NM_001144070 | ABCC3 | frameshift_deleti  on | exon3 | c.274delT | p.F92fs | . | . |
| CTC6 | 17 | 48753014 | GA | G | 0.231 | NM_003786 | ABCC3 | frameshift_deleti  on | exon21 | c.2738delA | p.D913fs | . | . |
| CTC6 | 17 | 54969178 | AGC | A | 0.167 | NM_005082 | TRIM25 | frameshift_deleti  on | exon9 | c.1774_1775del | p.A592fs | . | . |
| CTC6 | 17 | 56649379 | GT | G | 0.308 | NM_031272 | TEX14 | frameshift_deleti  on | exon24 | c.3619delA | p.T1207fs | . | . |
| CTC6 | 17 | 56679813 | TC | T | 0.186 | NM_001201457 | TEX14 | frameshift_deleti  on | exon12 | c.1492delG | p.D498fs | . | . |
| CTC6 | 17 | 57271001 | TTCC | T | 0.128 | NM_018304 | PRR11 | nonframeshift_de  letion | exon5 | c.552_554del | p.184_185del | . | . |
| CTC6 | 17 | 57760011 | GA | G | 0.5 | NM_001288653 | CLTC | frameshift_deleti  on | exon23 | c.3635delA | p.E1212fs | . | . |
| CTC6 | 17 | 59955427 | A | AT | 0.908 | NM_020748 | INTS2 | frameshift_inserti  on | exon18 | c.2300dupA | p.N767fs | . | . |
| CTC6 | 17 | 60033143 | CT | C | 0.857 | NM_005121 | MED13 | frameshift_deleti  on | exon25 | c.5679delA | p.K1893fs | . | . |
| CTC6 | 17 | 60814271 | C | CA | 0.322 | NM_001100875 | 10-Mar | frameshift_inserti  on | exon6 | c.957dupT | p.G320fs | . | . |
| CTC6 | 17 | 61611651 | C | CT | 0.214 | NM_001278919 | KCNH6 | frameshift_inserti  on | exon5 | c.1081dupT | p.I360fs | . | . |
| CTC6 | 17 | 61619706 | CG | C | 0.177 | NM_001278919 | KCNH6 | frameshift_deleti  on | exon9 | c.2060delG | p.R687fs | . | . |
| CTC6 | 17 | 61869869 | GT | G | 0.75 | NM_203499 | DDX42 | frameshift_deleti  on | exon3 | c.320delT | p.V107fs | . | . |
| CTC6 | 17 | 62492646 | TA | T | 0.887 | NM_007215 | POLG2 | stopgain | exon1 | c.440delT | p.L147X | . | . |
| CTC6 | 17 | 62552073 | A | AT | 0.342 | NM_022739 | SMURF2 | frameshift_inserti on | exon14 | c.1474dupA | p.M492fs | . | . |
| CTC6 | 17 | 63533607 | TG | T | 0.333 | NM_004655 | AXIN2 | frameshift_deleti  on | exon6 | c.1546delC | p.H516fs | . | . |
| CTC6 | 17 | 65941580 | CA | C | 0.418 | NM_182641 | BPTF | frameshift_deleti  on | exon21 | c.6757delA | p.K2253fs | . | . |
| CTC6 | 17 | 66978734 | AG | A | 0.2 | NM_080283 | ABCA9 | frameshift_deleti  on | exon37 | c.4688delC | p.P1563fs | . | . |
| CTC6 | 17 | 72699202 | T | TG | 0.6 | NM_001289083 | CD300LF | frameshift_inserti  on | exon3 | c.417_418insC | p.T140fs | . | . |
| CTC6 | 17 | 75190928 | AG | A | 0.333 | NM_001144001 | SEC14L1 | frameshift_deleti  on | exon5 | c.543delG | p.E181fs | . | . |
| CTC6 | 17 | 76421528 | AG | A | 0.268 | NM_173628 | DNAH17 | frameshift_deleti  on | exon80 | c.13039delC | p.L4347fs | . | . |

| CTC6 | 17 | 76851905 | CG | C | 0.462 | NM_003255 | TIMP2 | frameshift_deleti  on | exon5 | c.506delC | p.P169fs | . | . |
| --- | --- | --- | --- | --- | --- | --- | --- | --- | --- | --- | --- | --- | --- |
| CTC6 | 17 | 76888018 | TG | T | 0.491 | NM_001243540 | LOC1006535  15 | frameshift_deleti  on | exon3 | c.567delC | p.P189fs | . | . |
| CTC6 | 17 | 77093419 | GTAT | G | 0.818 | NM_001082575 | RBFOX3 | nonframeshift_de  letion | exon11 | c.707_709del | p.236_237del | . | . |
| CTC6 | 17 | 77768491 | CA | C | 0.511 | NM_020649 | CBX8 | stopgain | exon5 | c.1112delT | p.L371X | . | . |
| CTC6 | 17 | 77769090 | GCT | G | 0.16 | NM_020649 | CBX8 | frameshift_deleti on | exon5 | c.512_513del | p.E171fs | . | . |
| CTC6 | 17 | 77769113 | CT | C | 0.462 | NM_020649 | CBX8 | frameshift_deleti  on | exon5 | c.490delA | p.R164fs | . | . |
| CTC6 | 17 | 78064140 | G | GAAC | 0.99 | NM_001243342 | CCDC40 | nonframeshift_in  sertion | exon18 | c.3035_3036ins  AAC | p.R1012delinsR  T | . | . |
| CTC6 | 17 | 78064143 | C | CAAA | 0.909 | NM_001243342 | CCDC40 | nonframeshift_in  sertion | exon18 | c.3038_3039ins  AAA | p.T1013delinsT  K | . | . |
| CTC6 | 17 | 78064144 | A | AAC | 1 | NM_001243342 | CCDC40 | frameshift_inserti  on | exon18 | c.3039_3040ins  AC | p.T1013fs | . | . |
| CTC6 | 17 | 78064145 | A | ACAC | 0.993 | NM_001243342 | CCDC40 | nonframeshift_in  sertion | exon18 | c.3040_3041ins  CAC | p.R1014delinsT  R | . | . |
| CTC6 | 17 | 78086784 | C | CT | 0.246 | NM_000152 | GAA | frameshift_inserti  on | exon14 | c.1999dupT | p.A666fs | . | . |
| CTC6 | 17 | 78857277 | CG | C | 0.311 | NM_020761 | RPTOR | frameshift_deleti  on | exon15 | c.1644delG | p.T548fs | . | . |
| CTC6 | 17 | 79180993 | TG | T | 0.438 | NM_001009811 | AZI1 | frameshift_deleti  on | exon4 | c.318delC | p.P106fs | . | . |
| CTC6 | 17 | 79219500 | AATG | A | 1 | NM_001037984 | SLC38A10 | nonframeshift_de  letion | exon16 | c.3213_3215del | p.1071_1072de  l | . | . |
| CTC6 | 17 | 79792728 | G | GA | 1 | NM_001007533 | PPP1R27 | frameshift_inserti  on | exon1 | c.101dupT | p.F34fs | . | . |
| CTC6 | 17 | 80042219 | AG | A | 0.895 | NM_004104 | FASN | frameshift_deleti  on | exon28 | c.4809delC | p.F1603fs | . | . |
| CTC6 | 17 | 80045044 | CG | C | 0.803 | NM_004104 | FASN | frameshift_deleti  on | exon21 | c.3308delC | p.P1103fs | . | . |
| CTC6 | 17 | 80085596 | AC | A | 0.462 | NM_198082 | CCDC57 | frameshift_deleti  on | exon16 | c.2534delG | p.G845fs | . | . |
| CTC6 | 17 | 80195756 | GC | G | 0.31 | NM_001042422 | SLC16A3 | frameshift_deleti  on | exon4 | c.1111delC | p.P371fs | . | . |
| CTC6 | 17 | 80209297 | G | GC | 0.5 | NM_001893 | CSNK1D | frameshift_inserti  on | exon6 | c.842dupG | p.G281fs | . | . |
| CTC6 | 17 | 80211031 | GC | G | 0.387 | NM_001893 | CSNK1D | frameshift_deleti  on | exon4 | c.425delG | p.G142fs | . | . |
| CTC6 | 18 | 627479 | C | CA | 0.5 | NM_014410 | CLUL1 | frameshift_inserti  on | exon5 | c.807dupA | p.T269fs | . | . |
| CTC6 | 18 | 2891468 | CA | C | 0.786 | NM_032048 | EMILIN2 | frameshift_deleti  on | exon4 | c.1344delA | p.A448fs | . | . |
| CTC6 | 18 | 6971934 | TC | T | 0.593 | NM_005559 | LAMA1 | frameshift_deleti  on | exon48 | c.6820delG | p.E2274fs | . | . |
| CTC6 | 18 | 7023281 | AC | A | 0.882 | NM_005559 | LAMA1 | frameshift_deleti  on | exon19 | c.2582delG | p.G861fs | . | . |
| CTC6 | 18 | 9211743 | AT | A | 0.598 | NM_001083625 | ANKRD12 | frameshift_deleti  on | exon5 | c.545delT | p.I182fs | . | . |
| CTC6 | 18 | 9256619 | TA | T | 0.5 | NM_001083625 | ANKRD12 | frameshift_deleti  on | exon8 | c.3286delA | p.K1096fs | . | . |
| CTC6 | 18 | 12506592 | GT | G | 0.9 | NM_001128626 | SPIRE1 | frameshift_deleti  on | exon6 | c.855delA | p.K285fs | . | . |
| CTC6 | 18 | 12720644 | TC | T | 0.75 | NM_020232 | PSMG2 | frameshift_deleti  on | exon5 | c.544delC | p.P182fs | . | . |
| CTC6 | 18 | 14105458 | AC | A | 0.615 | NM_145287 | ZNF519 | frameshift_deleti  on | exon3 | c.1080delG | p.G360fs | . | . |
| CTC6 | 18 | 20606142 | C | CAA | 1 | NM_203292 | RBBP8 | frameshift_inserti  on | exon18 | c.2536_2537ins  AA | p.Q846fs | . | . |
| CTC6 | 18 | 21819237 | C | CG | 0.667 | NM_001242508 | OSBPL1A | frameshift_inserti  on | exon4 | c.244_245insC | p.G82fs | . | . |
| CTC6 | 18 | 28667642 | AT | A | 0.45 | NM_004949 | DSC2 | frameshift_deleti  on | exon6 | c.764delA | p.N255fs | . | . |
| CTC6 | 18 | 28710546 | C | CA | 0.6 | NM_024421 | DSC1 | frameshift_inserti  on | exon16 | c.2615_2616ins  T | p.E872fs | . | . |
| CTC6 | 18 | 28968450 | TC | T | 0.727 | NM_001134453 | DSG4 | stopgain | exon4 | c.338delC | p.S113X | . | . |
| CTC6 | 18 | 30795567 | T | TA | 0.333 | NM_001105528 | CCDC178 | frameshift_inserti on | exon18 | c.2024dupT | p.L675fs | . | . |
| CTC6 | 18 | 32822623 | GC | G | 0.385 | NM_001135178 | ZNF397 | frameshift_deleti  on | exon2 | c.190delC | p.P64fs | . | . |
| CTC6 | 18 | 32834215 | AG | A | 0.333 | NM_001288711 | ZSCAN30 | frameshift_deleti  on | exon2 | c.122delC | p.A41fs | . | . |
| CTC6 | 18 | 33831193 | TG | T | 0.235 | NM_017947 | MOCOS | frameshift_deleti  on | exon11 | c.2112delG | p.L704fs | . | . |
| CTC6 | 18 | 39629501 | A | AT | 0.483 | NM_002647 | PIK3C3 | frameshift_inserti  on | exon21 | c.2196dupT | p.Y732fs | . | . |
| CTC6 | 18 | 40854049 | AG | A | 0.6 | NM_020783 | SYT4 | frameshift_deleti  on | exon2 | c.344delC | p.P115fs | . | . |
| CTC6 | 18 | 42456670 | C | CTCTT | 0.63 | NM_001130110 | SETBP1 | frameshift_inserti  on | exon4 | c.681_682insTC  TT | p.L227fs | . | . |
| CTC6 | 18 | 43619941 | TG | T | 0.273 | NM_024430 | PSTPIP2 | stopgain | exon2 | c.105delC | p.C35X | . | . |
| CTC6 | 18 | 43669809 | CA | C | 0.933 | NM_001001935 | ATP5A1 | frameshift_deleti on | exon4 | c.312delT | p.L104fs | . | . |
| CTC6 | 18 | 43833781 | TG | T | 0.281 | NM_001008239 | C18orf25 | frameshift_deleti  on | exon3 | c.835delG | p.A279fs | . | . |
| CTC6 | 18 | 44140074 | C | CA | 0.432 | NM_144612 | LOXHD1 | frameshift_inserti  on | exon19 | c.3032dupT | p.L1011fs | . | . |
| CTC6 | 18 | 50976997 | GA | G | 0.5 | NM_005215 | DCC | frameshift_deleti  on | exon23 | c.3358delA | p.I1120fs | . | . |
| CTC6 | 18 | 56247512 | AG | A | 0.4 | NM_052947 | ALPK2 | frameshift_deleti  on | exon4 | c.495delC | p.P165fs | . | . |
| CTC6 | 18 | 60630690 | CG | C | 0.5 | NM_194449 | PHLPP1 | frameshift_deleti  on | exon14 | c.3546delG | p.S1182fs | . | . |
| CTC6 | 18 | 61233861 | GA | G | 0.615 | NM_080474 | SERPINB12 | frameshift_deleti  on | exon7 | c.836delA | p.E279fs | . | . |
| CTC6 | 18 | 67715293 | A | AT | 0.667 | NM_173630 | RTTN | frameshift_inserti  on | exon40 | c.5454dupA | p.C1819fs | . | . |
| CTC6 | 18 | 67863850 | T | TCTC | 1 | NM_173630 | RTTN | nonframeshift_in  sertion | exon7 | c.727_728insG  AG | p.D243delinsG  D | . | . |
| CTC6 | 18 | 72223591 | G | GTGC | 0.792 | NM_032649 | CNDP1 | nonframeshift_in  sertion | exon2 | c.43_44insTGC | p.V15delinsVL | . | . |
| CTC6 | 18 | 72775412 | TCG | T | 0.923 | NM_017757 | ZNF407 | frameshift_deleti  on | exon8 | c.5736_5737del | p.I1912fs | . | . |
| CTC6 | 18 | 72997836 | C | CA | 0.308 | NM_005786 | TSHZ1 | frameshift_inserti  on | exon2 | c.340dupA | p.P113fs | . | . |
| CTC6 | 18 | 77737640 | AT | A | 0.375 | NM_006701 | TXNL4A | frameshift_deleti  on | exon2 | c.214delA | p.M72fs | . | . |
| CTC6 | 19 | 375772 | CG | C | 0.609 | NM_016585 | THEG | frameshift_deleti  on | exon1 | c.198delC | p.P66fs | . | . |

| CTC6 | 19 | 685832 | CG | C | 0.302 | NM_214710 | PRSS57 | frameshift_deleti  on | exon5 | c.735delC | p.P245fs | . | . |
| --- | --- | --- | --- | --- | --- | --- | --- | --- | --- | --- | --- | --- | --- |
| CTC6 | 19 | 1082890 | GC | G | 0.579 | NM_001282334 | HMHA1 | frameshift_deleti  on | exon10 | c.1475delC | p.A492fs | . | . |
| CTC6 | 19 | 1578371 | GCTC | G | 0.194 | NM_001281453 | MBD3 | nonframeshift_de  letion | exon6 | c.841_843del | p.281_281del | . | . |
| CTC6 | 19 | 3600523 | AC | A | 1 | NM_001060 | TBXA2R | frameshift_deleti  on | exon2 | c.109delG | p.V37fs | . | . |
| CTC6 | 19 | 3976697 | TG | T | 0.667 | NM_001961 | EEF2 | frameshift_deleti  on | exon15 | c.2431delC | p.Q811fs | . | . |
| CTC6 | 19 | 4231273 | GC | G | 0.714 | NM_005755 | EBI3 | frameshift_deleti  on | exon2 | c.154delC | p.P52fs | . | . |
| CTC6 | 19 | 4359178 | TAC | T | 0.855 | NM_001159846 | MPND | frameshift_deleti  on | exon10 | c.1196_1197del | p.Y399fs | . | . |
| CTC6 | 19 | 4453475 | CG | C | 0.333 | NM_001171091 | UBXN6 | frameshift_deleti  on | exon3 | c.132delC | p.P44fs | . | . |
| CTC6 | 19 | 4499632 | AAGCTG  GCCGGG | A | 1 | NM_001001520 | HDGFRP2 | frameshift_deleti  on | exon14 | c.1721_1722del | p.K574fs | . | . |
| CTC6 | 19 | 4682878 | CAG | C | 0.25 | NM_001242901 | AC005594.3 | frameshift_deleti  on | exon2 | c.9_10del | p.A3fs | . | . |
| CTC6 | 19 | 5218507 | G | GA | 0.333 | NM_130853 | PTPRS | frameshift_inserti  on | exon17 | c.2678dupT | p.L893fs | . | . |
| CTC6 | 19 | 5616244 | CA | C | 0.333 | NM_014649 | SAFB2 | frameshift_deleti  on | exon4 | c.441delT | p.F147fs | . | . |
| CTC6 | 19 | 7126651 | C | CA | 1 | NM_001079817 | INSR | frameshift_inserti  on | exon15 | c.2920_2921ins  T | p.G974fs | . | . |
| CTC6 | 19 | 7601114 | GAA | G | 0.286 | NM_001166114 | PNPLA6 | frameshift_deleti  on | exon2 | c.271_272del | p.K91fs | . | . |
| CTC6 | 19 | 7607647 | AC | A | 0.649 | NM_001166114 | PNPLA6 | frameshift_deleti  on | exon12 | c.1364delC | p.T455fs | . | . |
| CTC6 | 19 | 7607672 | AG | A | 0.227 | NM_001166114 | PNPLA6 | frameshift_deleti  on | exon12 | c.1389delG | p.Q463fs | . | . |
| CTC6 | 19 | 7965456 | GT | G | 0.526 | NM_001268285 | LRRC8E | frameshift_deleti  on | exon2 | c.1663delT | p.S555fs | . | . |
| CTC6 | 19 | 8431138 | G | GT | 0.194 | NM_001039667 | ANGPTL4 | frameshift_inserti  on | exon3 | c.482_483insT | p.R161fs | . | . |
| CTC6 | 19 | 9011377 | A | AT | 0.429 | NM_024690 | MUC16 | frameshift_inserti  on | exon36 | c.38855dupA | p.H12952fs | . | . |
| CTC6 | 19 | 9017471 | TG | T | 0.222 | NM_024690 | MUC16 | frameshift_deleti  on | exon26 | c.37852delC | p.H12618fs | . | . |
| CTC6 | 19 | 9046411 | TG | T | 0.99 | NM_024690 | MUC16 | frameshift_deleti  on | exon5 | c.35219delC | p.P11740fs | . | . |
| CTC6 | 19 | 9046413 | GT | G | 0.99 | NM_024690 | MUC16 | frameshift_deleti  on | exon5 | c.35217delA | p.A11739fs | . | . |
| CTC6 | 19 | 9071841 | CT | C | 0.273 | NM_024690 | MUC16 | frameshift_deleti  on | exon3 | c.15604delA | p.S5202fs | . | . |
| CTC6 | 19 | 9072695 | G | GTT | 0.167 | NM_024690 | MUC16 | stopgain | exon3 | c.14750_14751i  nsAA | p.Y4917_K4918  delinsX | . | . |
| CTC6 | 19 | 9085905 | TG | T | 0.308 | NM_024690 | MUC16 | frameshift_deleti  on | exon1 | c.5909delC | p.P1970fs | . | . |
| CTC6 | 19 | 9089159 | TC | T | 0.267 | NM_024690 | MUC16 | frameshift_deleti  on | exon1 | c.2655delG | p.R885fs | . | . |
| CTC6 | 19 | 9801055 | T | TA | 0.42 | NM_001199814 | ZNF812 | frameshift_inserti  on | exon6 | c.1123dupT | p.Y375fs | . | . |
| CTC6 | 19 | 10081304 | G | GC | 0.213 | NM_015719 | COL5A3 | frameshift_inserti  on | exon54 | c.3929dupG | p.G1310fs | . | . |
| CTC6 | 19 | 10089862 | AG | A | 1 | NM_015719 | COL5A3 | frameshift_deleti  on | exon39 | c.2819delC | p.P940fs | . | . |
| CTC6 | 19 | 10114749 | CG | C | 0.375 | NM_015719 | COL5A3 | frameshift_deleti  on | exon5 | c.666delC | p.P222fs | . | . |
| CTC6 | 19 | 10799323 | TG | T | 0.774 | NM_012218 | ILF3 | frameshift_deleti  on | exon19 | c.2521delG | p.G841fs | . | . |
| CTC6 | 19 | 11350495 | CA | C | 0.6 | NM_018687 | C19orf80 | frameshift_deleti  on | exon1 | c.183delA | p.T61fs | . | . |
| CTC6 | 19 | 11354520 | GCACT | G | 0.2 | NM_020812 | DOCK6 | frameshift_deleti  on | exon10 | c.1058_1061del | p.E353fs | . | . |
| CTC6 | 19 | 11546949 | CGCT | C | 0.182 | NM_001289102 | PRKCSH | nonframeshift_de  letion | exon1 | c.12_14del | p.4_5del | . | . |
| CTC6 | 19 | 11617233 | GC | G | 0.375 | NM_001142465 | ECSIT | frameshift_deleti  on | exon6 | c.419delG | p.G140fs | . | . |
| CTC6 | 19 | 11687958 | GC | G | 0.192 | NM_001611 | ACP5 | frameshift_deleti  on | exon2 | c.174delG | p.V58fs | . | . |
| CTC6 | 19 | 11941466 | GC | G | 0.35 | NM_152357 | ZNF440 | frameshift_deleti  on | exon3 | c.166delC | p.H56fs | . | . |
| CTC6 | 19 | 12061046 | A | AT | 0.667 | NM_001271848 | ZNF700 | frameshift_inserti  on | exon4 | c.2217dupT | p.D739fs | . | . |
| CTC6 | 19 | 12638215 | G | GA | 0.261 | NM_144976 | ZNF564 | frameshift_inserti  on | exon4 | c.706dupT | p.S236fs | . | . |
| CTC6 | 19 | 12739513 | GA | G | 0.562 | NM_153358 | ZNF791 | frameshift_deleti  on | exon4 | c.1171delA | p.K391fs | . | . |
| CTC6 | 19 | 12848327 | C | CA | 0.333 | NM_004317 | ASNA1 | frameshift_inserti  on | exon1 | c.9dupA | p.A3fs | . | . |
| CTC6 | 19 | 13319691 | GGAT | G | 0.412 | NM_001127221 | CACNA1A | nonframeshift_de  letion | exon46 | c.6659_6661del | p.2220_2221de  l | . | . |
| CTC6 | 19 | 14150576 | GC | G | 0.521 | NM_004843 | IL27RA | frameshift_deleti  on | exon4 | c.389delC | p.A130fs | . | . |
| CTC6 | 19 | 14163075 | GC | G | 0.396 | NM_004843 | IL27RA | frameshift_deleti  on | exon14 | c.1885delC | p.P629fs | . | . |
| CTC6 | 19 | 14262096 | CT | C | 0.385 | NM_014921 | LPHN1 | frameshift_deleti  on | exon23 | c.3998delA | p.K1333fs | . | . |
| CTC6 | 19 | 14590245 | GC | G | 0.556 | NM_202494 | GIPC1 | frameshift_deleti  on | exon4 | c.455delG | p.G152fs | . | . |
| CTC6 | 19 | 14910354 | TC | T | 0.235 | NM_198944 | OR7C1 | frameshift_deleti  on | exon1 | c.594delG | p.V198fs | . | . |
| CTC6 | 19 | 15221005 | A | AG | 0.92 | NM_033025 | SYDE1 | frameshift_inserti  on | exon3 | c.922dupG | p.R307fs | . | . |
| CTC6 | 19 | 15484611 | T | TA | 0.212 | NM_005858 | AKAP8 | frameshift_inserti  on | exon4 | c.356dupT | p.I119fs | . | . |
| CTC6 | 19 | 15511792 | G | GA | 0.232 | NM_014371 | AKAP8L | frameshift_inserti  on | exon6 | c.878_879insT | p.R293fs | . | . |
| CTC6 | 19 | 15636258 | CCTG | C | 0.429 | NM_173483 | CYP4F22 | nonframeshift_de  letion | exon3 | c.112_114del | p.38_38del | . | . |
| CTC6 | 19 | 17015326 | TCC | T | 0.185 | NM_015692 | CPAMD8 | frameshift_deleti  on | exon31 | c.4203_4204del | p.W1401fs | . | . |
| CTC6 | 19 | 17393505 | TG | T | 0.278 | NM_001278443 | ANKLE1 | frameshift_deleti  on | exon3 | c.386delG | p.W129fs | . | . |
| CTC6 | 19 | 17612209 | GCC | G | 0.25 | NM_198580 | SLC27A1 | frameshift_deleti  on | exon11 | c.1765_1766del | p.P589fs | . | . |
| CTC6 | 19 | 17837593 | CG | C | 0.441 | NM_018174 | MAP1S | frameshift_deleti  on | exon5 | c.1401delG | p.P467fs | . | . |
| CTC6 | 19 | 17838523 | TG | T | 0.333 | NM_018174 | MAP1S | frameshift_deleti  on | exon5 | c.2331delG | p.L777fs | . | . |
| CTC6 | 19 | 19414610 | CA | C | 0.179 | NM_172231 | SUGP1 | frameshift_deleti  on | exon5 | c.584delT | p.L195fs | . | . |

| CTC6 | 19 | 20117899 | AC | A | 0.553 | NM_001077349 | ZNF682 | frameshift_deleti  on | exon4 | c.315delG | p.L105fs | . | . |
| --- | --- | --- | --- | --- | --- | --- | --- | --- | --- | --- | --- | --- | --- |
| CTC6 | 19 | 21299774 | T | TATA | 0.472 | NM_182515 | ZNF714 | nonframeshift_in  sertion | exon5 | c.304_305insAT  A | p.Y102delinsYN | . | . |
| CTC6 | 19 | 22155527 | AT | A | 0.981 | NM_007153 | ZNF208 | frameshift_deleti  on | exon4 | c.2308delA | p.I770fs | . | . |
| CTC6 | 19 | 22364162 | TG | T | 0.917 | NM_001001411 | ZNF676 | frameshift_deleti  on | exon3 | c.356delC | p.A119fs | . | . |
| CTC6 | 19 | 22940288 | TC | T | 1 | NM_001080409 | ZNF99 | frameshift_deleti  on | exon4 | c.2422delG | p.E808fs | . | . |
| CTC6 | 19 | 23544249 | AT | A | 0.318 | NM_003430 | ZNF91 | frameshift_deleti  on | exon4 | c.1531delA | p.I511fs | . | . |
| CTC6 | 19 | 23926647 | TA | T | 0.506 | NM_138286 | ZNF681 | frameshift_deleti  on | exon4 | c.1704delT | p.G568fs | . | . |
| CTC6 | 19 | 31768586 | TG | T | 0.806 | NM_020856 | TSHZ3 | frameshift_deleti  on | exon2 | c.2112delC | p.G704fs | . | . |
| CTC6 | 19 | 33493208 | GT | G | 0.231 | NM_033103 | RHPN2 | frameshift_deleti  on | exon9 | c.1049delA | p.H350fs | . | . |
| CTC6 | 19 | 34791833 | AT | A | 1 | NM_014686 | KIAA0355 | frameshift_deleti  on | exon2 | c.456delT | p.N152fs | . | . |
| CTC6 | 19 | 34810904 | CT | C | 0.875 | NM_014686 | KIAA0355 | frameshift_deleti  on | exon3 | c.589delT | p.F197fs | . | . |
| CTC6 | 19 | 34818429 | TA | T | 0.667 | NM_014686 | KIAA0355 | frameshift_deleti  on | exon4 | c.810delA | p.L270fs | . | . |
| CTC6 | 19 | 35175076 | TAAAAA  AAAAAA AAAAAA | T | 1 | NM_001289188 | ZNF302 | frameshift_deleti on | exon5 | c.398_691del | p.*133fs | . | . |
| CTC6 | 19 | 35175076 | TAAAAA  AAAAAA AAAAAA | T | 1 | NM_001289188 | ZNF302 | frameshift_deleti on | exon5 | c.398_691del | p.*133fs | . | . |
| CTC6 | 19 | 35550791 | TC | T | 0.248 | NM_182983 | HPN | frameshift_deleti  on | exon6 | c.305delC | p.S102fs | . | . |
| CTC6 | 19 | 35551630 | CG | C | 0.309 | NM_182983 | HPN | frameshift_deleti  on | exon9 | c.721delG | p.G241fs | . | . |
| CTC6 | 19 | 35556789 | CT | C | 0.298 | NM_182983 | HPN | frameshift_deleti  on | exon12 | c.1069delT | p.F357fs | . | . |
| CTC6 | 19 | 35719550 | C | CA | 0.211 | NM_152481 | FAM187B | frameshift_inserti  on | exon1 | c.33dupT | p.A12fs | . | . |
| CTC6 | 19 | 35758082 | CCCGAT  CCGGGA TGAGGA | C | 1 | NM_001260490 | LSR | frameshift_deleti on | exon6 | c.1036_1061del | p.P346fs | . | . |
| CTC6 | 19 | 35758084 | C | CACTC  CTCATC | 1 | NM_001260490 | LSR | frameshift_inserti  on | exon6 | c.1037_1038ins  ACTCCTCATCC | p.P346fs | . | . |
| CTC6 | 19 | 35842974 | TG | T | 1 | NM_005303 | FFAR1 | frameshift_deleti  on | exon1 | c.521delG | p.W174fs | . | . |
| CTC6 | 19 | 36002361 | ACTGCT  GCCACC ACTGCT GCCGCC ACTGCT GCCGCC | A | 1 | NM_001126057 | DMKN | nonframeshift_de letion | exon5 | c.819_869del | p.273_290del | . | . |
| CTC6 | 19 | 36216152 | T | TG | 0.655 | NM_014727 | KMT2B | frameshift_inserti  on | exon11 | c.3561dupG | p.M1187fs | . | . |
| CTC6 | 19 | 36220977 | A | AT | 0.17 | NM_014727 | KMT2B | frameshift_inserti  on | exon23 | c.5028dupT | p.D1676fs | . | . |
| CTC6 | 19 | 36221262 | GT | G | 0.375 | NM_014727 | KMT2B | frameshift_deleti  on | exon24 | c.5097delT | p.G1699fs | . | . |
| CTC6 | 19 | 36221612 | TC | T | 0.5 | NM_014727 | KMT2B | frameshift_deleti  on | exon26 | c.5282delC | p.S1761fs | . | . |
| CTC6 | 19 | 36231388 | CAG | C | 0.185 | NM_024660 | IGFLR1 | frameshift_deleti  on | exon3 | c.233_234del | p.S78fs | . | . |
| CTC6 | 19 | 36243829 | T | TC | 0.388 | NM_019104 | LIN37 | frameshift_inserti  on | exon5 | c.207dupC | p.F69fs | . | . |
| CTC6 | 19 | 36278283 | TG | T | 0.235 | NM_001172630 | ARHGAP33 | frameshift_deleti  on | exon20 | c.2409delG | p.V803fs | . | . |
| CTC6 | 19 | 37117077 | ACC | A | 0.214 | NM_001256838 | ZNF382 | frameshift_deleti  on | exon5 | c.276_277del | p.Y92fs | . | . |
| CTC6 | 19 | 37117408 | GA | G | 0.286 | NM_001256838 | ZNF382 | frameshift_deleti  on | exon5 | c.607delA | p.K203fs | . | . |
| CTC6 | 19 | 38023309 | CA | C | 0.217 | NM_001013659 | ZNF793 | frameshift_deleti  on | exon6 | c.68delA | p.H23fs | . | . |
| CTC6 | 19 | 38189230 | GC | G | 0.603 | NM_001172677 | ZNF607 | frameshift_deleti  on | exon5 | c.1798delG | p.A600fs | . | . |
| CTC6 | 19 | 38230322 | GT | G | 0.246 | NM_001172692 | ZNF573 | frameshift_deleti  on | exon4 | c.804delA | p.K268fs | . | . |
| CTC6 | 19 | 38380004 | TG | T | 0.5 | NM_031951 | WDR87 | frameshift_deleti  on | exon6 | c.4189delC | p.Q1397fs | . | . |
| CTC6 | 19 | 38895614 | CT | C | 0.333 | NM_174905 | FAM98C | frameshift_deleti  on | exon4 | c.417delT | p.P139fs | . | . |
| CTC6 | 19 | 38979872 | CT | C | 0.706 | NM_000540 | RYR1 | frameshift_deleti  on | exon35 | c.5604delT | p.P1868fs | . | . |
| CTC6 | 19 | 38985174 | AT | A | 0.214 | NM_000540 | RYR1 | frameshift_deleti  on | exon39 | c.6458delT | p.M2153fs | . | . |
| CTC6 | 19 | 39013931 | CA | C | 0.4 | NM_000540 | RYR1 | frameshift_deleti  on | exon69 | c.10423delA | p.K3475fs | . | . |
| CTC6 | 19 | 40581145 | AG | A | 0.625 | NM_001010880 | ZNF780A | frameshift_deleti  on | exon6 | c.1203delC | p.S401fs | . | . |
| CTC6 | 19 | 40875915 | TG | T | 0.167 | NM_001031696 | PLD3 | frameshift_deleti  on | exon7 | c.531delG | p.L177fs | . | . |
| CTC6 | 19 | 40900179 | TTCC | T | 0.189 | NM_181882 | PRX | nonframeshift_de  letion | exon7 | c.4077_4079del | p.1359_1360de  l | . | . |
| CTC6 | 19 | 40900619 | GC | G | 1 | NM_181882 | PRX | frameshift_deleti  on | exon7 | c.3639delG | p.M1213fs | . | . |
| CTC6 | 19 | 40993617 | C | CA | 1 | NM_020971 | SPTBN4 | frameshift_inserti  on | exon3 | c.183_184insA | p.A61fs | . | . |
| CTC6 | 19 | 42563536 | CG | C | 0.19 | NM_002088 | GRIK5 | frameshift_deleti  on | exon5 | c.651delC | p.D217fs | . | . |
| CTC6 | 19 | 42566670 | AG | A | 0.45 | NM_002088 | GRIK5 | frameshift_deleti  on | exon4 | c.477delC | p.P159fs | . | . |
| CTC6 | 19 | 42860576 | GC | G | 0.314 | NM_001410 | MEGF8 | frameshift_deleti  on | exon25 | c.4393delC | p.P1465fs | . | . |
| CTC6 | 19 | 43234004 | GTT | G | 0.281 | NM_021016 | PSG3 | frameshift_deleti  on | exon4 | c.912_913del | p.E304fs | . | . |
| CTC6 | 19 | 43268140 | AG | A | 1 | NM_001130167 | PSG8 | frameshift_deleti  on | exon2 | c.357delC | p.S119fs | . | . |
| CTC6 | 19 | 43523156 | TG | T | 0.235 | NM_001113410 | PSG11 | frameshift_deleti  on | exon2 | c.108delC | p.P36fs | . | . |
| CTC6 | 19 | 44050056 | TC | T | 0.333 | NM_006297 | XRCC1 | frameshift_deleti  on | exon14 | c.1534delG | p.E512fs | . | . |
| CTC6 | 19 | 44515225 | TC | T | 0.429 | NM_006300 | ZNF230 | frameshift_deleti  on | exon5 | c.1035delC | p.F345fs | . | . |
| CTC6 | 19 | 45769508 | AC | A | 0.234 | NM_001199867 | MARK4 | frameshift_deleti  on | exon7 | c.519delC | p.H173fs | . | . |

| CTC6 | 19 | 45864828 | GA | G | 0.6 | NM_001130867 | ERCC2 | frameshift_deleti  on | exon11 | c.1118delT | p.L373fs | . | . |
| --- | --- | --- | --- | --- | --- | --- | --- | --- | --- | --- | --- | --- | --- |
| CTC6 | 19 | 46417871 | AC | A | 0.746 | NM_001029861 | NANOS2 | frameshift_deleti  on | exon1 | c.80delG | p.G27fs | . | . |
| CTC6 | 19 | 47228171 | GC | G | 0.303 | NM_001039877 | STRN4 | frameshift_deleti  on | exon12 | c.1507delG | p.A503fs | . | . |
| CTC6 | 19 | 47280594 | CG | C | 0.411 | NM_001145145 | SLC1A5 | frameshift_deleti  on | exon5 | c.520delC | p.R174fs | . | . |
| CTC6 | 19 | 47879700 | AT | A | 0.931 | NM_014681 | DHX34 | frameshift_deleti  on | exon12 | c.2483delT | p.I828fs | . | . |
| CTC6 | 19 | 47969458 | TTG | T | 1 | NM_015063 | SLC8A2 | frameshift_deleti  on | exon2 | c.201_202del | p.D67fs | . | . |
| CTC6 | 19 | 48715049 | TC | T | 0.298 | NM_001184900 | CARD8 | frameshift_deleti  on | exon11 | c.1531delG | p.D511fs | . | . |
| CTC6 | 19 | 49134111 | G | GC | 0.553 | NM_001352 | DBP | frameshift_inserti  on | exon4 | c.960_961insG | p.Q321fs | . | . |
| CTC6 | 19 | 49134200 | TC | T | 0.28 | NM_001352 | DBP | frameshift_deleti  on | exon4 | c.871delG | p.E291fs | . | . |
| CTC6 | 19 | 49143499 | GGA | G | 0.172 | NM_001217 | CA11 | frameshift_deleti  on | exon4 | c.322_323del | p.S108fs | . | . |
| CTC6 | 19 | 49238533 | CG | C | 1 | NM_017805 | RASIP1 | frameshift_deleti  on | exon4 | c.1098delC | p.F366fs | . | . |
| CTC6 | 19 | 49464225 | TG | T | 0.231 | NM_004324 | BAX | frameshift_deleti  on | exon5 | c.529delG | p.A177fs | . | . |
| CTC6 | 19 | 50154209 | CT | C | 1 | NM_021228 | SCAF1 | frameshift_deleti  on | exon7 | c.564delT | p.P188fs | . | . |
| CTC6 | 19 | 50881822 | A | ACAG | 1 | NM_001256647 | NR1H2 | nonframeshift_in  sertion | exon5 | c.225_226insCA  G | p.K75delinsKQ | . | . |
| CTC6 | 19 | 51217084 | CG | C | 0.208 | NM_016148 | SHANK1 | frameshift_deleti  on | exon5 | c.762delC | p.A254fs | . | . |
| CTC6 | 19 | 51451975 | CT | C | 0.242 | NM_001077492 | KLK5 | frameshift_deleti  on | exon5 | c.646delA | p.R216fs | . | . |
| CTC6 | 19 | 51563313 | GC | G | 0.241 | NM_015596 | KLK13 | frameshift_deleti  on | exon3 | c.276delG | p.G92fs | . | . |
| CTC6 | 19 | 51835892 | T | TG | 1 | NM_001163922 | VSIG10L | frameshift_inserti  on | exon10 | c.2576dupC | p.A859fs | . | . |
| CTC6 | 19 | 51983721 | TG | T | 0.307 | NM_001278392 | CEACAM18 | frameshift_deleti  on | exon2 | c.188delG | p.W63fs | . | . |
| CTC6 | 19 | 52133125 | AT | A | 0.279 | NM_003830 | SIGLEC5 | frameshift_deleti  on | exon2 | c.381delA | p.K127fs | . | . |
| CTC6 | 19 | 52222588 | AG | A | 0.5 | NM_001523 | HAS1 | frameshift_deleti  on | exon2 | c.572delC | p.P191fs | . | . |
| CTC6 | 19 | 52794033 | AT | A | 1 | NM_001010851 | ZNF766 | frameshift_deleti  on | exon4 | c.990delT | p.N330fs | . | . |
| CTC6 | 19 | 52803668 | GCT | G | 0.571 | NM_144684 | ZNF480 | frameshift_deleti  on | exon2 | c.4_5del | p.L2fs | . | . |
| CTC6 | 19 | 52803669 | CTG | C | 1 | NM_144684 | ZNF480 | frameshift_deleti  on | exon2 | c.5_6del | p.L2fs | . | . |
| CTC6 | 19 | 53014193 | GC | G | 0.5 | NM_001099694 | ZNF578 | frameshift_deleti  on | exon6 | c.560delC | p.A187fs | . | . |
| CTC6 | 19 | 53116616 | TA | T | 0.231 | NM_001277951 | ZNF83 | frameshift_deleti  on | exon3 | c.1201delT | p.Y401fs | . | . |
| CTC6 | 19 | 53454475 | C | CACT | 1 | NM_001202456 | ZNF816 | nonframeshift_in  sertion | exon4 | c.552_553insA  GT | p.G185delinsS  G | . | . |
| CTC6 | 19 | 54667535 | CA | C | 0.75 | NM_001145303 | TMC4 | frameshift_deleti  on | exon8 | c.1215delT | p.F405fs | . | . |
| CTC6 | 19 | 54780706 | AGAG | A | 0.147 | NM_001278404 | LILRB2 | nonframeshift_de  letion | exon9 | c.1087_1089del | p.363_363del | . | . |
| CTC6 | 19 | 54967403 | C | CA | 0.25 | NM_052925 | LENG8 | frameshift_inserti  on | exon9 | c.1283_1284ins  A | p.P428fs | . | . |
| CTC6 | 19 | 54968073 | CG | C | 0.462 | NM_052925 | LENG8 | frameshift_deleti  on | exon11 | c.1705delG | p.D569fs | . | . |
| CTC6 | 19 | 55525851 | T | TGG | 0.17 | NM_001083899 | GP6 | frameshift_inserti  on | exon8 | c.1461_1462ins  CC | p.R488fs | . | . |
| CTC6 | 19 | 55832354 | G | GA | 0.25 | NM_001085488 | TMEM150B | frameshift_inserti  on | exon3 | c.50dupT | p.I17fs | . | . |
| CTC6 | 19 | 55869879 | GGCTCC  TTCATCT CGCCCC | G | 1 | NM_001145402 | FAM71E2 | nonframeshift_de letion | exon9 | c.2333_2356del | p.778_786del | . | . |
| CTC6 | 19 | 56052337 | A | AC | 1 | NM_001199824 | SBK3 | frameshift_inserti  on | exon4 | c.954dupG | p.F319fs | . | . |
| CTC6 | 19 | 56244376 | GC | G | 0.241 | NM_176820 | NLRP9 | frameshift_deleti  on | exon2 | c.820delG | p.A274fs | . | . |
| CTC6 | 19 | 56416330 | TG | T | 0.917 | NM_176810 | NLRP13 | frameshift_deleti  on | exon8 | c.2595delC | p.P865fs | . | . |
| CTC6 | 19 | 56935051 | CA | C | 0.481 | NM_001159860 | ZNF583 | frameshift_deleti  on | exon5 | c.1025delA | p.H342fs | . | . |
| CTC6 | 19 | 56953525 | GT | G | 0.241 | NM_022103 | ZNF667 | frameshift_deleti  on | exon5 | c.838delA | p.T280fs | . | . |
| CTC6 | 19 | 57327658 | AC | A | 0.619 | NM_001146186 | PEG3 | frameshift_deleti  on | exon7 | c.2151delG | p.G717fs | . | . |
| CTC6 | 19 | 57640495 | CA | C | 0.238 | NM_020903 | USP29 | frameshift_deleti  on | exon4 | c.453delA | p.S151fs | . | . |
| CTC6 | 19 | 57802788 | CA | C | 0.278 | NM_006635 | ZNF460 | frameshift_deleti  on | exon3 | c.880delA | p.N294fs | . | . |
| CTC6 | 19 | 57931454 | CT | C | 0.5 | NM_006959 | ZNF17 | frameshift_deleti  on | exon3 | c.595delT | p.F199fs | . | . |
| CTC6 | 19 | 57988666 | A | AGCC | 0.993 | NM_001024596 | ZNF772 | nonframeshift_in  sertion | exon1 | c.11_12insGGC | p.A4delinsAA | . | . |
| CTC6 | 19 | 58189361 | AAC | A | 0.286 | NM_152677 | ZSCAN4 | frameshift_deleti  on | exon4 | c.477_478del | p.K159fs | . | . |
| CTC6 | 19 | 58352656 | CT | C | 1 | NM_001204818 | ZNF587B | frameshift_deleti  on | exon3 | c.615delT | p.S205fs | . | . |
| CTC6 | 19 | 58385869 | C | CATA | 1 | NM_001144989 | ZNF814 | nonframeshift_in  sertion | exon3 | c.888_889insTA  T | p.E297delinsYE | . | . |
| CTC6 | 19 | 58640555 | CG | C | 0.514 | NM_024620 | ZNF329 | frameshift_deleti  on | exon4 | c.315delC | p.P105fs | . | . |
| CTC6 | 20 | 126310 | ACC | A | 0.223 | NM_030931 | DEFB126 | frameshift_deleti  on | exon2 | c.314_315del | p.T105fs | . | . |
| CTC6 | 20 | 746198 | AT | A | 0.225 | NM_033409 | SLC52A3 | frameshift_deleti  on | exon2 | c.220delA | p.I74fs | . | . |
| CTC6 | 20 | 1896051 | CCGA | C | 1 | NM_001040023 | SIRPA | nonframeshift_de  letion | exon2 | c.387_389del | p.129_130del | . | . |
| CTC6 | 20 | 2847166 | TG | T | 0.333 | NM_080413 | VPS16 | frameshift_deleti  on | exon20 | c.2035delG | p.G679fs | . | . |
| CTC6 | 20 | 3005237 | CA | C | 0.196 | NM_080840 | PTPRA | frameshift_deleti  on | exon16 | c.1558delA | p.S520fs | . | . |
| CTC6 | 20 | 3171332 | C | CA | 0.69 | NM_023935 | DDRGK1 | frameshift_inserti  on | exon9 | c.911_912insT | p.W304fs | . | . |
| CTC6 | 20 | 3670627 | TG | T | 0.667 | NM_023068 | SIGLEC1 | frameshift_deleti  on | exon18 | c.4875delC | p.S1625fs | . | . |

| CTC6 | 20 | 3673672 | GAGGGC  CAGCTG GGCGGG CGGGCG GCTGTC CACAGT | G | 0.143 | NM_023068 | SIGLEC1 | nonframeshift_de letion | exon14 | c.3570_3614del | p.1190_1205de l | . | . |
| --- | --- | --- | --- | --- | --- | --- | --- | --- | --- | --- | --- | --- | --- |
| CTC6 | 20 | 3675062 | TC | T | 0.438 | NM_023068 | SIGLEC1 | frameshift_deleti  on | exon12 | c.3061delG | p.D1021fs | . | . |
| CTC6 | 20 | 3781443 | GC | G | 0.444 | NM_001287516 | CDC25B | frameshift_deleti  on | exon6 | c.322delC | p.P108fs | . | . |
| CTC6 | 20 | 3781980 | AG | A | 0.321 | NM_001287524 | CDC25B | frameshift_deleti  on | exon4 | c.210delG | p.E70fs | . | . |
| CTC6 | 20 | 9346119 | TG | T | 0.966 | NM_000933 | PLCB4 | frameshift_deleti  on | exon6 | c.462delG | p.L154fs | . | . |
| CTC6 | 20 | 9561416 | TTC | T | 0.126 | NM_177990 | PAK7 | frameshift_deleti  on | exon4 | c.364_365del | p.E122fs | . | . |
| CTC6 | 20 | 19566170 | TG | T | 0.875 | NM_020689 | SLC24A3 | frameshift_deleti  on | exon6 | c.595delG | p.G199fs | . | . |
| CTC6 | 20 | 23066049 | T | TG | 0.96 | NM_012072 | CD93 | frameshift_inserti  on | exon1 | c.780dupC | p.K261fs | . | . |
| CTC6 | 20 | 25597014 | GA | G | 0.583 | NM_152667 | NANP | frameshift_deleti  on | exon2 | c.293delT | p.F98fs | . | . |
| CTC6 | 20 | 30510835 | GA | G | 0.519 | NM_001008409 | TTLL9 | frameshift_deleti  on | exon8 | c.644delA | p.E215fs | . | . |
| CTC6 | 20 | 31024187 | GA | G | 0.708 | NM_015338 | ASXL1 | frameshift_deleti  on | exon12 | c.3673delA | p.K1225fs | . | . |
| CTC6 | 20 | 32379221 | TG | T | 0.149 | NM_001282935 | ZNF341 | frameshift_deleti  on | exon14 | c.2194delG | p.E732fs | . | . |
| CTC6 | 20 | 32436432 | AG | A | 0.385 | NM_176812 | CHMP4B | frameshift_deleti  on | exon2 | c.351delG | p.K117fs | . | . |
| CTC6 | 20 | 33328412 | GC | G | 0.235 | NM_014071 | NCOA6 | frameshift_deleti  on | exon11 | c.5647delG | p.A1883fs | . | . |
| CTC6 | 20 | 33330967 | TTGC | T | 0.25 | NM_014071 | NCOA6 | nonframeshift_de  letion | exon11 | c.3090_3092del | p.1030_1031de  l | . | . |
| CTC6 | 20 | 33509633 | CG | C | 0.318 | NM_001242393 | ACSS2 | frameshift_deleti  on | exon13 | c.1228delG | p.G410fs | . | . |
| CTC6 | 20 | 34022414 | TG | T | 0.922 | NM_000557 | GDF5 | frameshift_deleti  on | exon2 | c.798delC | p.S266fs | . | . |
| CTC6 | 20 | 34085748 | GC | G | 0.224 | NM_007186 | CEP250 | frameshift_deleti  on | exon26 | c.3508delC | p.Q1170fs | . | . |
| CTC6 | 20 | 34241818 | AC | A | 0.5 | NM_001198840 | RBM12 | stopgain | exon2 | c.1426delG | p.V476X | . | . |
| CTC6 | 20 | 34262968 | TC | T | 0.5 | NM_001198989 | NFS1 | frameshift_deleti on | exon7 | c.793delG | p.E265fs | . | . |
| CTC6 | 20 | 35521415 | TG | T | 0.526 | NM_015474 | SAMHD1 | frameshift_deleti  on | exon16 | c.1800delC | p.D600fs | . | . |
| CTC6 | 20 | 42204969 | A | AC | 0.229 | NM_016276 | SGK2 | frameshift_inserti  on | exon10 | c.979_980insC | p.S327fs | . | . |
| CTC6 | 20 | 42340238 | TG | T | 0.406 | NM_001278610 | MYBL2 | frameshift_deleti  on | exon10 | c.1645delG | p.G549fs | . | . |
| CTC6 | 20 | 42635275 | TG | T | 0.61 | NM_001098796 | TOX2 | frameshift_deleti  on | exon3 | c.129delG | p.L43fs | . | . |
| CTC6 | 20 | 43933326 | CG | C | 0.153 | NM_030590 | MATN4 | frameshift_deleti  on | exon3 | c.184delC | p.R62fs | . | . |
| CTC6 | 20 | 44049320 | AC | A | 0.667 | NM_001184730 | PIGT | frameshift_deleti  on | exon6 | c.715delC | p.P239fs | . | . |
| CTC6 | 20 | 44520237 | CCTGCT  G | C | 0.333 | NM_000308 | CTSA | nonframeshift_de  letion | exon2 | c.85_90del | p.29_30del | . | . |
| CTC6 | 20 | 44527031 | AC | A | 0.714 | NM_001167594 | CTSA | frameshift_deleti  on | exon14 | c.1389delC | p.D463fs | . | . |
| CTC6 | 20 | 44638604 | C | CG | 0.357 | NM_004994 | MMP9 | frameshift_inserti  on | exon2 | c.238_239insG | p.L80fs | . | . |
| CTC6 | 20 | 45212284 | GC | G | 0.345 | NM_001193339 | SLC13A3 | frameshift_deleti  on | exon8 | c.995delG | p.G332fs | . | . |
| CTC6 | 20 | 45315744 | TG | T | 0.692 | NM_033550 | TP53RK | frameshift_deleti  on | exon2 | c.409delC | p.Q137fs | . | . |
| CTC6 | 20 | 45354882 | CG | C | 0.786 | NM_030777 | SLC2A10 | frameshift_deleti  on | exon2 | c.1208delG | p.R403fs | . | . |
| CTC6 | 20 | 46264859 | CA | C | 1 | NM_001174087 | NCOA3 | frameshift_deleti  on | exon12 | c.1730delA | p.Q577fs | . | . |
| CTC6 | 20 | 46267923 | AG | A | 0.19 | NM_001174087 | NCOA3 | frameshift_deleti  on | exon14 | c.2685delG | p.Q895fs | . | . |
| CTC6 | 20 | 46279814 | TGCA | T | 0.312 | NM_001174087 | NCOA3 | nonframeshift_de  letion | exon20 | c.3738_3740del | p.1246_1247de  l | . | . |
| CTC6 | 20 | 46279830 | GCAGCA  A | G | 0.214 | NM_001174087 | NCOA3 | nonframeshift_de  letion | exon20 | c.3754_3759del | p.1252_1253de  l | . | . |
| CTC6 | 20 | 46279833 | GCAA | G | 0.324 | NM_001174087 | NCOA3 | nonframeshift_de  letion | exon20 | c.3757_3759del | p.1253_1253de  l | . | . |
| CTC6 | 20 | 46279836 | ACAG | A | 0.364 | NM_001174087 | NCOA3 | nonframeshift_de  letion | exon20 | c.3760_3762del | p.1254_1254de  l | . | . |
| CTC6 | 20 | 47692038 | CA | C | 0.458 | NM_001256135 | CSE1L | frameshift_deleti  on | exon11 | c.1149delA | p.S383fs | . | . |
| CTC6 | 20 | 47855512 | AT | A | 0.348 | NM_017895 | DDX27 | frameshift_deleti  on | exon15 | c.1808delT | p.I603fs | . | . |
| CTC6 | 20 | 47887458 | CT | C | 0.75 | NM_021035 | ZNFX1 | frameshift_deleti  on | exon3 | c.890delA | p.K297fs | . | . |
| CTC6 | 20 | 48712800 | CG | C | 0.245 | NM_001282580 | UBE2V1 | frameshift_deleti  on | exon3 | c.178delC | p.R60fs | . | . |
| CTC6 | 20 | 49211153 | TCAG | T | 0.875 | NM_080829 | FAM65C | nonframeshift_de  letion | exon17 | c.2134_2136del | p.712_712del | . | . |
| CTC6 | 20 | 50769259 | TG | T | 0.394 | NM_022088 | ZFP64 | frameshift_deleti  on | exon5 | c.1309delC | p.Q437fs | . | . |
| CTC6 | 20 | 50769456 | GA | G | 0.368 | NM_022088 | ZFP64 | frameshift_deleti  on | exon5 | c.1112delT | p.F371fs | . | . |
| CTC6 | 20 | 51870084 | AGAG | A | 0.231 | NM_001193421 | TSHZ2 | nonframeshift_de  letion | exon2 | c.79_81del | p.27_27del | . | . |
| CTC6 | 20 | 56136614 | TGAG | T | 0.273 | NM_002591 | PCK1 | nonframeshift_de  letion | exon2 | c.148_150del | p.50_50del | . | . |
| CTC6 | 20 | 58519076 | AC | A | 0.81 | NM_022106 | FAM217B | frameshift_deleti  on | exon4 | c.79delC | p.P27fs | . | . |
| CTC6 | 20 | 58533819 | TGCTTCT  AGTGCT | T | 0.167 | NM_177980 | CDH26 | frameshift_deleti  on | exon1 | c.39_54del | p.L13fs | . | . |
| CTC6 | 20 | 60791161 | A | AT | 0.484 | NM_007232 | HRH3 | frameshift_inserti  on | exon3 | c.1238_1239ins  A | p.P413fs | . | . |
| CTC6 | 20 | 60882442 | TG | T | 0.632 | NM_001281437 | ADRM1 | frameshift_deleti  on | exon5 | c.441delG | p.L147fs | . | . |
| CTC6 | 20 | 61594100 | GA | G | 0.556 | NM_022082 | SLC17A9 | frameshift_deleti  on | exon5 | c.623delA | p.E208fs | . | . |
| CTC6 | 20 | 61959723 | GC | G | 0.491 | NM_020882 | COL20A1 | frameshift_deleti  on | exon34 | c.3655delC | p.P1219fs | . | . |
| CTC6 | 21 | 15753670 | TA | T | 0.244 | NM_006948 | HSPA13 | frameshift_deleti  on | exon2 | c.219delT | p.F73fs | . | . |

| CTC6 | 21 | 27066181 | CA | C | 0.857 | NM_001270407 | JAM2 | frameshift_deleti  on | exon3 | c.248delA | p.Q83fs | . | . |
| --- | --- | --- | --- | --- | --- | --- | --- | --- | --- | --- | --- | --- | --- |
| CTC6 | 21 | 28210323 | T | TA | 0.6 | NM_006988 | ADAMTS1 | frameshift_inserti  on | exon9 | c.2478dupT | p.T827fs | . | . |
| CTC6 | 21 | 28210475 | G | GT | 0.375 | NM_006988 | ADAMTS1 | frameshift_inserti  on | exon9 | c.2326dupA | p.T776fs | . | . |
| CTC6 | 21 | 30378768 | GA | G | 0.648 | NM_016940 | RWDD2B | frameshift_deleti  on | exon5 | c.929delT | p.F310fs | . | . |
| CTC6 | 21 | 30464820 | C | CA | 0.6 | NM_001286634 | MAP3K7CL | frameshift_inserti  on | exon5 | c.289dupA | p.S96fs | . | . |
| CTC6 | 21 | 31015193 | GTC | G | 0.154 | NM_000830 | GRIK1 | frameshift_deleti  on | exon7 | c.1049_1050del | p.R350fs | . | . |
| CTC6 | 21 | 31744306 | AG | A | 0.288 | NM_181621 | KRTAP13-2 | frameshift_deleti  on | exon1 | c.225delC | p.P75fs | . | . |
| CTC6 | 21 | 31913981 | AG | A | 1 | NM_181612 | KRTAP19-6 | frameshift_deleti  on | exon1 | c.171delC | p.F57fs | . | . |
| CTC6 | 21 | 33696808 | CA | C | 0.444 | NM_014825 | URB1 | stopgain | exon32 | c.5225delT | p.L1742X | . | . |
| CTC6 | 21 | 34003928 | A | AAGTA TT | 1 | NM_001160306 | SYNJ1 | nonframeshift_in sertion | exon28 | c.3957_3958ins AATACT | p.L1320delinsN TL | . | . |
| CTC6 | 21 | 34045762 | TC | T | 0.6 | NM_001160306 | SYNJ1 | frameshift_deleti  on | exon13 | c.1598delG | p.G533fs | . | . |
| CTC6 | 21 | 34166169 | GT | G | 0.646 | NM_001162496 | C21orf62 | frameshift_deleti  on | exon2 | c.563delA | p.N188fs | . | . |
| CTC6 | 21 | 34640742 | TG | T | 1 | NM_000628 | IL10RB | frameshift_deleti  on | exon2 | c.94delG | p.V32fs | . | . |
| CTC6 | 21 | 38309300 | CG | C | 0.647 | NM_000411 | HLCS | frameshift_deleti  on | exon5 | c.444delC | p.P148fs | . | . |
| CTC6 | 21 | 38507810 | TA | T | 0.375 | NM_001001894 | TTC3 | frameshift_deleti  on | exon18 | c.1575delA | p.I525fs | . | . |
| CTC6 | 21 | 39087308 | CT | C | 1 | NM_002240 | KCNJ6 | frameshift_deleti  on | exon3 | c.151delA | p.R51fs | . | . |
| CTC6 | 21 | 39087374 | TG | T | 1 | NM_002240 | KCNJ6 | frameshift_deleti  on | exon3 | c.85delC | p.Q29fs | . | . |
| CTC6 | 21 | 40194610 | TG | T | 1 | NM_005239 | ETS2 | frameshift_deleti  on | exon10 | c.1208delG | p.W403fs | . | . |
| CTC6 | 21 | 40794949 | AT | A | 0.208 | NM_152505 | LCA5L | frameshift_deleti  on | exon5 | c.789delA | p.K263fs | . | . |
| CTC6 | 21 | 43236102 | GC | G | 0.607 | NM_001040424 | PRDM15 | frameshift_deleti  on | exon19 | c.2461delG | p.A821fs | . | . |
| CTC6 | 21 | 43309334 | TC | T | 0.667 | NM_199050 | C2CD2 | frameshift_deleti  on | exon13 | c.1524delG | p.R508fs | . | . |
| CTC6 | 21 | 43411247 | AGGT | A | 0.943 | NM_020727 | ZBTB21 | nonframeshift_de  letion | exon2 | c.2955_2957del | p.985_986del | . | . |
| CTC6 | 21 | 43836714 | CT | C | 1 | NM_001001895 | UBASH3A | frameshift_deleti  on | exon5 | c.718delT | p.Y240fs | . | . |
| CTC6 | 21 | 43846881 | AT | A | 0.25 | NM_001001895 | UBASH3A | frameshift_deleti  on | exon7 | c.1009delT | p.F337fs | . | . |
| CTC6 | 21 | 46596010 | CT | C | 0.719 | NM_001112 | ADARB1 | frameshift_deleti  on | exon4 | c.395delT | p.L132fs | . | . |
| CTC6 | 21 | 46596247 | TC | T | 0.721 | NM_001112 | ADARB1 | frameshift_deleti  on | exon4 | c.632delC | p.S211fs | . | . |
| CTC6 | 21 | 46924425 | CGGCCC  CCCA | C | 1 | NM_030582 | COL18A1 | frameshift_deleti  on | exon33 | c.3364_3364del | p.G1122fs | . | . |
| CTC6 | 21 | 46935712 | A | ACG | 0.227 | NM_001205207 | SLC19A1 | frameshift_inserti  on | exon5 | c.1515_1516ins  CG | p.C506fs | . | . |
| CTC6 | 21 | 47546037 | GA | G | 0.857 | NM_001849 | COL6A2 | frameshift_deleti  on | exon26 | c.2309delA | p.E770fs | . | . |
| CTC6 | 22 | 18209881 | CCA | C | 0.618 | NM_001270733 | BCL2L13 | frameshift_deleti  on | exon2 | c.200_201del | p.P67fs | . | . |
| CTC6 | 22 | 18606976 | AC | A | 0.286 | NM_001193414 | TUBA8 | frameshift_deleti  on | exon3 | c.83delC | p.T28fs | . | . |
| CTC6 | 22 | 19189003 | A | AC | 1 | NM_001835 | CLTCL1 | frameshift_inserti  on | exon23 | c.3601dupG | p.V1201fs | . | . |
| CTC6 | 22 | 19210275 | CA | C | 0.172 | NM_001835 | CLTCL1 | frameshift_deleti  on | exon15 | c.2349delT | p.F783fs | . | . |
| CTC6 | 22 | 19371219 | TC | T | 1 | NM_003325 | HIRA | frameshift_deleti  on | exon13 | c.1338delG | p.L446fs | . | . |
| CTC6 | 22 | 20130543 | AT | A | 0.559 | NM_001185024 | ZDHHC8 | frameshift_deleti  on | exon10 | c.1391delT | p.I464fs | . | . |
| CTC6 | 22 | 20918816 | ACAG | A | 0.167 | NM_001003891 | MED15 | nonframeshift_de  letion | exon6 | c.532_534del | p.178_178del | . | . |
| CTC6 | 22 | 20920813 | ACAG | A | 0.333 | NM_001003891 | MED15 | nonframeshift_de  letion | exon7 | c.751_753del | p.251_251del | . | . |
| CTC6 | 22 | 23498201 | TA | T | 1 | NM_004914 | RAB36 | frameshift_deleti  on | exon6 | c.575delA | p.Y192fs | . | . |
| CTC6 | 22 | 23964282 | GCAT | G | 0.826 | NM_016449 | C22orf43 | nonframeshift_de  letion | exon4 | c.377_379del | p.126_127del | . | . |
| CTC6 | 22 | 24039380 | AT | A | 0.349 | NM_153615 | RGL4 | frameshift_deleti  on | exon8 | c.1185delT | p.D395fs | . | . |
| CTC6 | 22 | 24095262 | TG | T | 0.286 | NM_013378 | VPREB3 | frameshift_deleti  on | exon2 | c.172delC | p.Q58fs | . | . |
| CTC6 | 22 | 24479290 | TC | T | 0.833 | NM_001201429 | CABIN1 | frameshift_deleti  on | exon19 | c.2709delC | p.F903fs | . | . |
| CTC6 | 22 | 25755922 | TC | T | 0.357 | NM_182492 | LRP5L | frameshift_deleti  on | exon1 | c.137delG | p.R46fs | . | . |
| CTC6 | 22 | 29533471 | TC | T | 0.349 | NM_001039570 | KREMEN1 | frameshift_deleti  on | exon6 | c.774delC | p.F258fs | . | . |
| CTC6 | 22 | 29885984 | CA | C | 0.312 | NM_021076 | NEFH | frameshift_deleti  on | exon4 | c.2356delA | p.K786fs | . | . |
| CTC6 | 22 | 29916080 | TC | T | 0.196 | NM_001002879 | THOC5 | frameshift_deleti  on | exon14 | c.1290delG | p.L430fs | . | . |
| CTC6 | 22 | 30186468 | CGA | C | 0.385 | NM_001242906 | ASCC2 | frameshift_deleti  on | exon18 | c.1862_1863del | p.L621fs | . | . |
| CTC6 | 22 | 30384548 | TC | T | 0.915 | NM_021090 | MTMR3 | frameshift_deleti  on | exon6 | c.291delC | p.I97fs | . | . |
| CTC6 | 22 | 30824478 | TG | T | 0.267 | NM_001003704 | MTFP1 | frameshift_deleti  on | exon3 | c.226delG | p.A76fs | . | . |
| CTC6 | 22 | 31671291 | GC | G | 1 | NM_001031801 | LIMK2 | frameshift_deleti  on | exon14 | c.1707delC | p.S569fs | . | . |
| CTC6 | 22 | 31837905 | TG | T | 0.243 | NM_001164502 | EIF4ENIF1 | frameshift_deleti  on | exon15 | c.1883delC | p.T628fs | . | . |
| CTC6 | 22 | 32215089 | TG | T | 0.667 | NM_001136029 | DEPDC5 | frameshift_deleti  on | exon21 | c.1749delG | p.M583fs | . | . |
| CTC6 | 22 | 32643407 | CA | C | 0.789 | NM_014227 | SLC5A4 | frameshift_deleti  on | exon5 | c.467delT | p.L156fs | . | . |
| CTC6 | 22 | 37697037 | AC | A | 0.333 | NM_013385 | CYTH4 | frameshift_deleti  on | exon7 | c.525delC | p.N175fs | . | . |
| CTC6 | 22 | 37964569 | AG | A | 0.706 | NM_152243 | CDC42EP1 | frameshift_deleti  on | exon3 | c.919delG | p.G307fs | . | . |
| CTC6 | 22 | 38119754 | TCAA | T | 0.647 | NM_001039141 | TRIOBP | nonframeshift_de  letion | exon7 | c.1192_1194del | p.398_398del | . | . |
| CTC6 | 22 | 38120175 | GCCT | G | 0.333 | NM_001039141 | TRIOBP | nonframeshift_de  letion | exon7 | c.1613_1615del | p.538_539del | . | . |

| CTC6 | 22 | 38470897 | CG | C | 0.214 | NM_001039583 | PICK1 | frameshift_deleti  on | exon13 | c.1007delG | p.R336fs | . | . |
| --- | --- | --- | --- | --- | --- | --- | --- | --- | --- | --- | --- | --- | --- |
| CTC6 | 22 | 39421146 | TA | T | 0.99 | NM_152426 | APOBEC3D | frameshift_deleti  on | exon3 | c.283delA | p.N95fs | . | . |
| CTC6 | 22 | 39777822 | C | CCAA | 1 | NM_004711 | SYNGR1 | nonframeshift_in  sertion | exon4 | c.605_606insCA  A | p.P202delinsP  N | . | . |
| CTC6 | 22 | 39777824 | A | AACT | 0.962 | NM_004711 | SYNGR1 | nonframeshift_in  sertion | exon4 | c.607_608insAC  T | p.T203delinsNS | . | . |
| CTC6 | 22 | 39908496 | GC | G | 0.252 | NM_019008 | MIEF1 | frameshift_deleti  on | exon5 | c.583delC | p.Q195fs | . | . |
| CTC6 | 22 | 40055501 | GC | G | 0.778 | NM_001003406 | CACNA1I | frameshift_deleti  on | exon12 | c.2290delC | p.P764fs | . | . |
| CTC6 | 22 | 40361999 | A | AT | 0.2 | NM_004810 | GRAP2 | frameshift_inserti  on | exon5 | c.296_297insT | p.E99fs | . | . |
| CTC6 | 22 | 40660772 | TC | T | 0.304 | NM_001162501 | TNRC6B | frameshift_deleti  on | exon5 | c.539delC | p.S180fs | . | . |
| CTC6 | 22 | 40660823 | AT | A | 0.716 | NM_001162501 | TNRC6B | frameshift_deleti  on | exon5 | c.590delT | p.M197fs | . | . |
| CTC6 | 22 | 40666203 | TG | T | 0.552 | NM_001162501 | TNRC6B | frameshift_deleti  on | exon6 | c.2885delG | p.W962fs | . | . |
| CTC6 | 22 | 40697176 | TGCA | T | 0.157 | NM_015088 | TNRC6B | nonframeshift_de  letion | exon13 | c.3630_3632del | p.1210_1211de  l | . | . |
| CTC6 | 22 | 41625728 | TC | T | 0.651 | NM_138481 | CHADL | frameshift_deleti  on | exon6 | c.2268delG | p.G756fs | . | . |
| CTC6 | 22 | 41650468 | TTCC | T | 0.206 | NM_002883 | RANGAP1 | nonframeshift_de  letion | exon11 | c.1101_1103del | p.367_368del | . | . |
| CTC6 | 22 | 42114230 | TC | T | 0.429 | NM_152513 | MEI1 | frameshift_deleti  on | exon6 | c.686delC | p.S229fs | . | . |
| CTC6 | 22 | 42321554 | TG | T | 0.286 | NM_052945 | TNFRSF13C | frameshift_deleti  on | exon3 | c.371delC | p.P124fs | . | . |
| CTC6 | 22 | 42463786 | G | GA | 0.562 | NM_000262 | NAGA | frameshift_inserti  on | exon3 | c.306dupT | p.P103fs | . | . |
| CTC6 | 22 | 42611250 | A | AC | 0.371 | NM_005650 | TCF20 | frameshift_inserti  on | exon1 | c.61dupG | p.V21fs | . | . |
| CTC6 | 22 | 44559739 | TCC | T | 0.4 | NM_001243386 | PARVB | frameshift_deleti  on | exon11 | c.723_724del | p.V241fs | . | . |
| CTC6 | 22 | 45595854 | TG | T | 0.309 | NM_001009880 | KIAA0930 | frameshift_deleti  on | exon8 | c.914delC | p.P305fs | . | . |
| CTC6 | 22 | 46319192 | CA | C | 0.304 | NM_058238 | WNT7B | frameshift_deleti  on | exon4 | c.593delT | p.L198fs | . | . |
| CTC6 | 22 | 46709909 | AG | A | 0.909 | NM_016426 | GTSE1 | frameshift_deleti  on | exon6 | c.1051delG | p.A351fs | . | . |
| CTC6 | 22 | 46725345 | TG | T | 0.774 | NM_016426 | GTSE1 | frameshift_deleti  on | exon11 | c.2018delG | p.C673fs | . | . |
| CTC6 | 22 | 46742425 | GT | G | 0.255 | NM_001282782 | TRMU | frameshift_deleti  on | exon3 | c.121delT | p.F41fs | . | . |
| CTC6 | 22 | 47085979 | C | CA | 0.4 | NM_022766 | CERK | frameshift_inserti  on | exon12 | c.1450dupT | p.C484fs | . | . |
| CTC6 | 22 | 50728425 | AG | A | 0.3 | NM_012401 | PLXNB2 | frameshift_deleti  on | exon3 | c.588delC | p.A196fs | . | . |
| CTC6 | 22 | 51142341 | TACG | T | 0.259 | NM_033517 | SHANK3 | nonframeshift_de  letion | exon13 | c.1625_1627del | p.542_543del | . | . |
| CTC6 | 22 | 51153432 | AAC | A | 0.133 | NM_033517 | SHANK3 | frameshift_deleti  on | exon19 | c.2181_2182del | p.K727fs | . | . |
| CTC6 | X | 1719853 | AC | A | 1 | NM_005088 | AKAP17A | frameshift_deleti  on | exon5 | c.1455delC | p.H485fs | . | . |
| CTC6 | X | 2729389 | CA | C | 0.952 | NM_001141920 | XG | frameshift_deleti  on | exon9 | c.426delA | p.A142fs | . | . |
| CTC6 | X | 3228005 | GC | G | 0.236 | NM_015419 | MXRA5 | frameshift_deleti  on | exon7 | c.8238delG | p.R2746fs | . | . |
| CTC6 | X | 8555880 | GA | G | 0.722 | NM_000216 | KAL1 | frameshift_deleti  on | exon5 | c.680delT | p.I227fs | . | . |
| CTC6 | X | 10176395 | AC | A | 0.809 | NM_001256944 | CLCN4 | frameshift_deleti  on | exon7 | c.873delC | p.Y291fs | . | . |
| CTC6 | X | 11139099 | TC | T | 0.75 | NM_001122608 | HCCS | frameshift_deleti  on | exon6 | c.595delC | p.R199fs | . | . |
| CTC6 | X | 15339689 | GT | G | 0.49 | NM_020473 | PIGA | frameshift_deleti  on | exon5 | c.691delA | p.T231fs | . | . |
| CTC6 | X | 15540621 | GA | G | 0.382 | NM_001721 | BMX | frameshift_deleti  on | exon7 | c.664delA | p.K222fs | . | . |
| CTC6 | X | 18797237 | AC | A | 0.444 | NM_006240 | PPEF1 | frameshift_deleti  on | exon10 | c.669delC | p.Y223fs | . | . |
| CTC6 | X | 18797276 | ATT | A | 0.611 | NM_006240 | PPEF1 | frameshift_deleti  on | exon10 | c.708_709del | p.D236fs | . | . |
| CTC6 | X | 18936809 | CA | C | 0.216 | NM_000292 | PHKA2 | frameshift_deleti  on | exon19 | c.2126delT | p.L709fs | . | . |
| CTC6 | X | 19425332 | GT | G | 0.221 | NM_001001671 | MAP3K15 | frameshift_deleti  on | exon13 | c.1817delA | p.Y606fs | . | . |
| CTC6 | X | 20060696 | TCA | T | 0.167 | NM_001168466 | MAP7D2 | frameshift_deleti  on | exon6 | c.671_672del | p.V224fs | . | . |
| CTC6 | X | 21613469 | CA | C | 0.636 | NM_001168647 | CNKSR2 | frameshift_deleti  on | exon16 | c.1843delA | p.K615fs | . | . |
| CTC6 | X | 21627677 | AGAG | A | 0.167 | NM_001168647 | CNKSR2 | nonframeshift_de  letion | exon19 | c.2545_2547del | p.849_849del | . | . |
| CTC6 | X | 23397895 | TA | T | 0.467 | NM_173495 | PTCHD1 | frameshift_deleti  on | exon2 | c.540delA | p.L180fs | . | . |
| CTC6 | X | 34150200 | CG | C | 0.408 | NM_203408 | FAM47A | frameshift_deleti  on | exon1 | c.195delC | p.P65fs | . | . |
| CTC6 | X | 38525408 | TG | T | 0.736 | NM_004615 | TSPAN7 | frameshift_deleti  on | exon2 | c.116delG | p.W39fs | . | . |
| CTC6 | X | 41075500 | AG | A | 0.952 | NM_001039590 | USP9X | frameshift_deleti  on | exon35 | c.5681delG | p.R1894fs | . | . |
| CTC6 | X | 47030560 | GGGA | G | 0.182 | NM_001204467 | RBM10 | nonframeshift_de  letion | exon4 | c.336_338del | p.112_113del | . | . |
| CTC6 | X | 47100271 | TG | T | 0.34 | NM_004651 | USP11 | frameshift_deleti  on | exon7 | c.972delG | p.L324fs | . | . |
| CTC6 | X | 47107268 | GC | G | 0.675 | NM_004651 | USP11 | frameshift_deleti  on | exon21 | c.2832delC | p.G944fs | . | . |
| CTC6 | X | 47919308 | AT | A | 0.308 | NM_001037735 | ZNF630 | frameshift_deleti  on | exon5 | c.522delA | p.K174fs | . | . |
| CTC6 | X | 49076224 | TTCC | T | 0.128 | NM_001256789 | CACNA1F | nonframeshift_de  letion | exon20 | c.2409_2411del | p.803_804del | . | . |
| CTC6 | X | 49854845 | TG | T | 0.522 | NM_000084 | CLCN5 | frameshift_deleti  on | exon10 | c.1608delG | p.V536fs | . | . |
| CTC6 | X | 53277321 | CTT | C | 0.286 | NM_001111125 | IQSEC2 | frameshift_deleti  on | exon7 | c.2555_2556del | p.K852fs | . | . |
| CTC6 | X | 54106676 | CT | C | 0.9 | NM_017848 | FAM120C | frameshift_deleti  on | exon15 | c.3024delA | p.K1008fs | . | . |
| CTC6 | X | 54328244 | CT | C | 0.6 | NM_001002838 | WNK3 | frameshift_deleti  on | exon6 | c.1137delA | p.K379fs | . | . |
| CTC6 | X | 54817343 | TG | T | 0.378 | NM_198510 | ITIH6 | stopgain | exon4 | c.542delC | p.S181X | . | . |
| CTC6 | X | 69424348 | C | CA | 0.2 | NM_198512 | DGAT2L6 | frameshift_inserti on | exon6 | c.841_842insA | p.R281fs | . | . |

| CTC6 | X | 70357648 | AG | A | 0.417 | NM_005120 | MED12 | frameshift_deleti  on | exon41 | c.5900delG | p.S1967fs | . | . |
| --- | --- | --- | --- | --- | --- | --- | --- | --- | --- | --- | --- | --- | --- |
| CTC6 | X | 70598851 | AG | A | 0.304 | NM_001286074 | TAF1 | frameshift_deleti  on | exon8 | c.1391delG | p.R464fs | . | . |
| CTC6 | X | 70823676 | TG | T | 0.406 | NM_052957 | ACRC | frameshift_deleti  on | exon8 | c.550delG | p.D184fs | . | . |
| CTC6 | X | 71358456 | AC | A | 0.294 | NM_001013627 | NHSL2 | frameshift_deleti  on | exon6 | c.1059delC | p.D353fs | . | . |
| CTC6 | X | 71425846 | G | GA | 0.727 | NM_017669 | ERCC6L | frameshift_inserti  on | exon2 | c.2770dupT | p.S924fs | . | . |
| CTC6 | X | 72433663 | GTCC | G | 0.281 | NM_021963 | NAP1L2 | nonframeshift_de  letion | exon1 | c.663_665del | p.221_222del | . | . |
| CTC6 | X | 75648594 | TC | T | 0.263 | NM_020932 | MAGEE1 | frameshift_deleti  on | exon1 | c.272delC | p.S91fs | . | . |
| CTC6 | X | 76711870 | TA | T | 0.333 | NM_003868 | FGF16 | frameshift_deleti  on | exon2 | c.209delA | p.Y70fs | . | . |
| CTC6 | X | 82763783 | AC | A | 0.494 | NM_000307 | POU3F4 | frameshift_deleti  on | exon1 | c.452delC | p.T151fs | . | . |
| CTC6 | X | 84526620 | TA | T | 1 | NM_021998 | ZNF711 | frameshift_deleti  on | exon9 | c.2073delA | p.L691fs | . | . |
| CTC6 | X | 100494085 | GC | G | 0.185 | NM_001171184 | DRP2 | frameshift_deleti  on | exon4 | c.321delC | p.S107fs | . | . |
| CTC6 | X | 102612625 | CA | C | 0.929 | NM_001006613 | WBP5 | frameshift_deleti  on | exon2 | c.14delA | p.Q5fs | . | . |
| CTC6 | X | 107426982 | C | CCTT | 1 | NM_001287758 | COL4A6 | nonframeshift_in  sertion | exon24 | c.1971_1972ins  AAG | p.V658delinsKV | . | . |
| CTC6 | X | 107977498 | CA | C | 0.692 | NM_003604 | IRS4 | frameshift_deleti  on | exon1 | c.2076delT | p.F692fs | . | . |
| CTC6 | X | 110968258 | GA | G | 1 | NM_001099922 | ALG13 | frameshift_deleti  on | exon15 | c.1699delA | p.K567fs | . | . |
| CTC6 | X | 112035128 | GT | G | 0.469 | NM_001113490 | AMOT | frameshift_deleti  on | exon6 | c.1857delA | p.K619fs | . | . |
| CTC6 | X | 114874721 | A | AT | 0.232 | NM_001136025 | PLS3 | frameshift_inserti  on | exon9 | c.894dupT | p.D298fs | . | . |
| CTC6 | X | 117892157 | GC | G | 0.18 | NM_001560 | IL13RA1 | frameshift_deleti  on | exon5 | c.629delC | p.A210fs | . | . |
| CTC6 | X | 118222379 | CTG | C | 0.571 | NM_020721 | KIAA1210 | frameshift_deleti  on | exon11 | c.2812_2813del | p.Q938fs | . | . |
| CTC6 | X | 118222785 | TG | T | 0.259 | NM_020721 | KIAA1210 | frameshift_deleti  on | exon11 | c.2407delC | p.Q803fs | . | . |
| CTC6 | X | 118222841 | CT | C | 0.258 | NM_020721 | KIAA1210 | frameshift_deleti  on | exon11 | c.2351delA | p.E784fs | . | . |
| CTC6 | X | 118971759 | C | CT | 0.448 | NM_023010 | UPF3B | frameshift_inserti  on | exon9 | c.1223dupA | p.K408fs | . | . |
| CTC6 | X | 119387833 | C | CTGA | 0.7 | NM_006777 | ZBTB33 | nonframeshift_in  sertion | exon2 | c.563_564insTG  A | p.S188delinsSD | . | . |
| CTC6 | X | 122319767 | AC | A | 0.516 | NM_000828 | GRIA3 | frameshift_deleti  on | exon2 | c.194delC | p.T65fs | . | . |
| CTC6 | X | 123554309 | CG | C | 0.375 | NM_014253 | TENM1 | frameshift_deleti  on | exon24 | c.4812delC | p.G1604fs | . | . |
| CTC6 | X | 129060229 | GC | G | 0.5 | NM_001166221 | UTP14A | frameshift_deleti  on | exon13 | c.1802delC | p.A601fs | . | . |
| CTC6 | X | 129200880 | CT | C | 0.375 | NM_001127197 | ELF4 | frameshift_deleti  on | exon9 | c.1807delA | p.S603fs | . | . |
| CTC6 | X | 129360770 | AT | A | 1 | NM_017666 | ZNF280C | frameshift_deleti  on | exon12 | c.1357delA | p.M453fs | . | . |
| CTC6 | X | 130416957 | TC | T | 0.206 | NM_001170962 | IGSF1 | frameshift_deleti  on | exon5 | c.921delG | p.V307fs | . | . |
| CTC6 | X | 135047255 | AT | A | 0.459 | NM_173470 | MMGT1 | frameshift_deleti  on | exon4 | c.323delA | p.N108fs | . | . |
| CTC6 | X | 135290616 | CA | C | 0.375 | NM_001159699 | FHL1 | frameshift_deleti  on | exon5 | c.553delA | p.I185fs | . | . |
| CTC6 | X | 135313720 | CT | C | 0.826 | NM_001173517 | MAP7D3 | frameshift_deleti  on | exon7 | c.1290delA | p.K430fs | . | . |
| CTC6 | X | 135313765 | TG | T | 0.846 | NM_001173517 | MAP7D3 | frameshift_deleti  on | exon7 | c.1245delC | p.P415fs | . | . |
| CTC6 | X | 135474444 | AGAT | A | 1 | NM_153834 | GPR112 | nonframeshift_de  letion | exon17 | c.7966_7968del | p.2656_2656de  l | . | . |
| CTC6 | X | 135757228 | TG | T | 0.174 | NM_004840 | ARHGEF6 | frameshift_deleti  on | exon19 | c.1972delC | p.Q658fs | . | . |
| CTC6 | X | 138695375 | CT | C | 0.308 | NM_001171879 | MCF2 | frameshift_deleti  on | exon11 | c.1410delA | p.K470fs | . | . |
| CTC6 | X | 148627374 | TC | T | 0.339 | NM_001171909 | CXorf40A | frameshift_deleti  on | exon3 | c.199delC | p.Q67fs | . | . |
| CTC6 | X | 149101893 | TG | T | 0.259 | NM_001013845 | CXorf40B | frameshift_deleti  on | exon4 | c.199delC | p.Q67fs | . | . |
| CTC6 | X | 149639324 | ACAG | A | 0.6 | NM_001177466 | MAMLD1 | nonframeshift_de  letion | exon2 | c.1405_1407del | p.469_469del | . | . |
| CTC6 | X | 152083715 | CT | C | 0.301 | NM_001178106 | ZNF185 | frameshift_deleti  on | exon3 | c.193delT | p.F65fs | . | . |
| CTC6 | X | 152721069 | TGG | T | 0.241 | NM_017518 | HAUS7 | frameshift_deleti  on | exon8 | c.889_890del | p.P297fs | . | . |
| CTC6 | X | 152823631 | TC | T | 0.227 | NM_001001344 | ATP2B3 | frameshift_deleti  on | exon15 | c.2496delC | p.F832fs | . | . |
| CTC6 | X | 153556254 | CA | C | 0.556 | NM_001145934 | TKTL1 | frameshift_deleti  on | exon11 | c.1401delA | p.A467fs | . | . |
| CTC6 | X | 153585825 | TC | T | 1 | NM_001110556 | FLNA | frameshift_deleti  on | exon29 | c.4921delG | p.D1641fs | . | . |
| CTC6 | X | 153592420 | TC | T | 0.333 | NM_001110556 | FLNA | frameshift_deleti  on | exon15 | c.2249delG | p.G750fs | . | . |
| CTC6 | X | 153631065 | GC | G | 0.273 | NM_001009934 | DNASE1L1 | frameshift_deleti  on | exon8 | c.891delG | p.Q297fs | . | . |
| CTC6 | X | 153717232 | CA | C | 0.833 | NM_019848 | SLC10A3 | frameshift_deleti  on | exon2 | c.47delT | p.L16fs | . | . |
| CTC6 | X | 153762670 | TC | T | 0.788 | NM_000402 | G6PD | frameshift_deleti  on | exon6 | c.616delG | p.D206fs | . | . |
| CTC6 | X | 154467071 | AT | A | 0.19 | NM_003372 | VBP1 | frameshift_deleti  on | exon6 | c.543delT | p.N181fs | . | . |
| XS1 | 1 | 1163910 | AC | A | 0.333 | NM_016176 | SDF4 | frameshift_deleti  on | exon2 | c.263delG | p.G88fs | . | . |
| XS1 | 1 | 2235539 | AATGTA  CGTGTG AGTCGC TTTCTGT GCCTCC TCCCTGT | A | 0.894 | NM_003036 | SKI | frameshift_deleti on | exon4 | c.1473_1474del | p.E491fs | . | . |
| XS1 | 1 | 3551575 | T | TA | 0.667 | NM_017818 | WRAP73 | stopgain | exon8 | c.801dupT | p.N268_D269d  elinsX | . | . |
| XS1 | 1 | 6522928 | AC | A | 1 | NM_148967 | TNFRSF25 | frameshift_deleti  on | exon6 | c.565delG | p.V189fs | . | . |
| XS1 | 1 | 7723623 | GC | G | 0.792 | NM_015215 | CAMTA1 | frameshift_deleti  on | exon9 | c.1017delC | p.S339fs | . | . |
| XS1 | 1 | 7804918 | AC | A | 0.294 | NM_015215 | CAMTA1 | frameshift_deleti  on | exon17 | c.4207delC | p.H1403fs | . | . |

| XS1 | 1 | 9627395 | AT | A | 0.444 | NM_032315 | SLC25A33 | frameshift_deleti  on | exon3 | c.291delT | p.N97fs | . | . |
| --- | --- | --- | --- | --- | --- | --- | --- | --- | --- | --- | --- | --- | --- |
| XS1 | 1 | 10431259 | CT | C | 0.857 | NM_015074 | KIF1B | frameshift_deleti  on | exon43 | c.4748delT | p.L1583fs | . | . |
| XS1 | 1 | 11206844 | CT | C | 0.714 | NM_004958 | MTOR | frameshift_deleti  on | exon32 | c.4574delA | p.Q1525fs | . | . |
| XS1 | 1 | 11307769 | AT | A | 0.379 | NM_004958 | MTOR | frameshift_deleti  on | exon8 | c.1137delA | p.K379fs | . | . |
| XS1 | 1 | 12267044 | TG | T | 0.179 | NM_001066 | TNFRSF1B | frameshift_deleti  on | exon10 | c.1354delG | p.G452fs | . | . |
| XS1 | 1 | 12820856 | AT | A | 0.754 | NM_152290 | C1orf158 | frameshift_deleti  on | exon4 | c.558delT | p.H186fs | . | . |
| XS1 | 1 | 12943139 | GC | G | 0.217 | NM_001009611 | PRAMEF4 | frameshift_deleti  on | exon2 | c.76delG | p.A26fs | . | . |
| XS1 | 1 | 16056404 | GC | G | 0.533 | NM_015164 | PLEKHM2 | frameshift_deleti  on | exon14 | c.2189delC | p.A730fs | . | . |
| XS1 | 1 | 16382968 | C | CG | 0.5 | NM_001165945 | CLCNKB | frameshift_inserti  on | exon12 | c.1472dupG | p.R491fs | . | . |
| XS1 | 1 | 17084514 | TG | T | 0.222 | NM_001271733 | MST1L | frameshift_deleti  on | exon12 | c.1583delC | p.P528fs | . | . |
| XS1 | 1 | 17087541 | GGTGCT | G | 0.5 | NM_001271733 | MST1L | frameshift_deleti  on | exon2 | c.119_123del | p.Q40fs | . | . |
| XS1 | 1 | 18618465 | TA | T | 0.227 | NM_032880 | IGSF21 | frameshift_deleti  on | exon3 | c.290delA | p.Y97fs | . | . |
| XS1 | 1 | 19062531 | TA | T | 0.75 | NM_002584 | PAX7 | nonframeshift_de  letion | exon8 | c.1562delA | p.X521X | . | . |
| XS1 | 1 | 19524273 | TC | T | 0.843 | NM_020765 | UBR4 | frameshift_deleti  on | exon7 | c.783delG | p.L261fs | . | . |
| XS1 | 1 | 20979144 | TAG | T | 0.5 | NM_005216 | DDOST | frameshift_deleti  on | exon10 | c.1189_1190del | p.L397fs | . | . |
| XS1 | 1 | 21889634 | G | GC | 0.368 | NM_001177520 | ALPL | frameshift_inserti  on | exon3 | c.98_99insC | p.S33fs | . | . |
| XS1 | 1 | 22973919 | GA | G | 0.184 | NM_001114101 | C1QC | frameshift_deleti  on | exon3 | c.382delA | p.T128fs | . | . |
| XS1 | 1 | 23850973 | CT | C | 1 | NM_004091 | E2F2 | frameshift_deleti  on | exon2 | c.259delA | p.R87fs | . | . |
| XS1 | 1 | 24484360 | GGT | G | 0.155 | NM_173065 | IFNLR1 | frameshift_deleti  on | exon6 | c.690_691del | p.T230fs | . | . |
| XS1 | 1 | 26104656 | CG | C | 0.422 | NM_001289010 | MAN1C1 | frameshift_deleti  on | exon9 | c.1319delG | p.R440fs | . | . |
| XS1 | 1 | 26670650 | TTCCTCC | T | 0.333 | NM_001039775 | AIM1L | nonframeshift_de  letion | exon2 | c.2493_2498del | p.831_833del | . | . |
| XS1 | 1 | 27736473 | G | GA | 0.561 | NM_006990 | WASF2 | frameshift_inserti  on | exon8 | c.1051dupT | p.S351fs | . | . |
| XS1 | 1 | 28209210 | GC | G | 0.248 | NM_001105556 | THEMIS2 | frameshift_deleti  on | exon4 | c.1376delC | p.A459fs | . | . |
| XS1 | 1 | 28271807 | CCA | C | 0.375 | NM_001009568 | SMPDL3B | frameshift_deleti  on | exon2 | c.127_128del | p.Q43fs | . | . |
| XS1 | 1 | 28562908 | GA | G | 0.791 | NM_016311 | ATPIF1 | frameshift_deleti  on | exon2 | c.125delA | p.E42fs | . | . |
| XS1 | 1 | 31188967 | GA | G | 0.433 | NM_002379 | MATN1 | frameshift_deleti  on | exon5 | c.995delT | p.F332fs | . | . |
| XS1 | 1 | 31409648 | GT | G | 0.882 | NM_001020658 | PUM1 | frameshift_deleti  on | exon21 | c.3276delA | p.S1092fs | . | . |
| XS1 | 1 | 32160797 | CG | C | 0.429 | NM_001856 | COL16A1 | frameshift_deleti  on | exon11 | c.970delC | p.R324fs | . | . |
| XS1 | 1 | 32566076 | GC | G | 0.261 | NM_018056 | TMEM39B | frameshift_deleti  on | exon8 | c.1150delC | p.L384fs | . | . |
| XS1 | 1 | 32623872 | TA | T | 1 | NM_012316 | KPNA6 | frameshift_deleti  on | exon5 | c.337delA | p.S113fs | . | . |
| XS1 | 1 | 32742056 | GC | G | 0.25 | NM_001042771 | LCK | frameshift_deleti  on | exon8 | c.751delC | p.L251fs | . | . |
| XS1 | 1 | 34037250 | C | CT | 0.375 | NM_001281956 | CSMD2 | frameshift_inserti  on | exon51 | c.7838dupA | p.Q2613fs | . | . |
| XS1 | 1 | 35333206 | A | AG | 0.281 | NM_001080418 | DLGAP3 | frameshift_inserti  on | exon8 | c.2507dupC | p.A836fs | . | . |
| XS1 | 1 | 35654794 | CA | C | 1 | NM_005066 | SFPQ | frameshift_deleti  on | exon5 | c.1604delT | p.L535fs | . | . |
| XS1 | 1 | 36359730 | C | CA | 0.171 | NM_012199 | AGO1 | frameshift_inserti  on | exon6 | c.742_743insA | p.L248fs | . | . |
| XS1 | 1 | 36557526 | GC | G | 0.5 | NM_017825 | ADPRHL2 | frameshift_deleti  on | exon4 | c.533delC | p.A178fs | . | . |
| XS1 | 1 | 36754805 | CA | C | 0.65 | NM_005119 | THRAP3 | frameshift_deleti  on | exon5 | c.1186delA | p.K396fs | . | . |
| XS1 | 1 | 36859473 | AT | A | 0.417 | NM_032881 | LSM10 | frameshift_deleti  on | exon2 | c.257delA | p.D86fs | . | . |
| XS1 | 1 | 38034709 | GT | G | 0.211 | NM_013285 | GNL2 | frameshift_deleti  on | exon13 | c.1610delA | p.N537fs | . | . |
| XS1 | 1 | 40735756 | A | AT | 0.447 | NM_005857 | ZMPSTE24 | frameshift_inserti  on | exon5 | c.585dupT | p.Y195fs | . | . |
| XS1 | 1 | 40773144 | CGG | C | 0.145 | NM_001852 | COL9A2 | frameshift_deleti  on | exon19 | c.980_981del | p.P327fs | . | . |
| XS1 | 1 | 40776815 | GC | G | 0.5 | NM_001852 | COL9A2 | frameshift_deleti  on | exon12 | c.579delG | p.G193fs | . | . |
| XS1 | 1 | 40981007 | CTG | C | 0.529 | NM_022774 | EXO5 | frameshift_deleti  on | exon3 | c.792_793del | p.S264fs | . | . |
| XS1 | 1 | 41300660 | CA | C | 0.381 | NM_172163 | KCNQ4 | frameshift_deleti  on | exon11 | c.1474delA | p.K492fs | . | . |
| XS1 | 1 | 41483805 | GTCC | G | 0.17 | NM_144990 | SLFNL1 | nonframeshift_de  letion | exon3 | c.456_458del | p.152_153del | . | . |
| XS1 | 1 | 44057612 | AC | A | 0.971 | NM_002840 | PTPRF | frameshift_deleti  on | exon10 | c.1662delC | p.D554fs | . | . |
| XS1 | 1 | 46073529 | GA | G | 0.229 | NM_001195193 | NASP | frameshift_deleti  on | exon4 | c.755delA | p.E252fs | . | . |
| XS1 | 1 | 46487649 | CT | C | 0.272 | NM_015112 | MAST2 | frameshift_deleti  on | exon12 | c.1319delT | p.L440fs | . | . |
| XS1 | 1 | 47280746 | GAT | G | 0.99 | NM_000779 | CYP4B1 | frameshift_deleti  on | exon8 | c.881_882del | p.D294fs | . | . |
| XS1 | 1 | 52703746 | GA | G | 1 | NM_004799 | ZFYVE9 | frameshift_deleti  on | exon4 | c.658delA | p.K220fs | . | . |
| XS1 | 1 | 52854974 | CT | C | 0.333 | NM_001190818 | ORC1 | frameshift_deleti  on | exon7 | c.1101delA | p.K367fs | . | . |
| XS1 | 1 | 53724026 | A | AT | 0.295 | NM_033300 | LRP8 | frameshift_inserti  on | exon12 | c.1663dupA | p.M555fs | . | . |
| XS1 | 1 | 54405683 | GT | G | 1 | NM_016126 | HSPB11 | frameshift_deleti  on | exon2 | c.72delA | p.K24fs | . | . |
| XS1 | 1 | 54605318 | T | TGG | 0.947 | NM_201546 | CDCP2 | frameshift_inserti  on | exon4 | c.1224_1225ins  CC | p.M409fs | . | . |
| XS1 | 1 | 54605318 | T | TGGG | 0.947 | NM_201546 | CDCP2 | nonframeshift_in  sertion | exon4 | c.1224_1225ins  CCC | p.M409delinsP  M | . | . |
| XS1 | 1 | 55074673 | TGA | T | 0.152 | NM_147161 | ACOT11 | frameshift_deleti  on | exon16 | c.1674_1675del | p.V558fs | . | . |
| XS1 | 1 | 55473899 | TC | T | 0.522 | NM_057176 | BSND | frameshift_deleti  on | exon4 | c.562delC | p.P188fs | . | . |
| XS1 | 1 | 60299114 | AT | A | 0.6 | NM_015888 | HOOK1 | frameshift_deleti  on | exon5 | c.312delT | p.D104fs | . | . |

| XS1 | 1 | 62979238 | TA | T | 0.5 | NM_001272000 | DOCK7 | frameshift_deleti  on | exon32 | c.4065delT | p.F1355fs | . | . |
| --- | --- | --- | --- | --- | --- | --- | --- | --- | --- | --- | --- | --- | --- |
| XS1 | 1 | 65348978 | TG | T | 0.174 | NM_002227 | JAK1 | frameshift_deleti  on | exon3 | c.186delC | p.I62fs | . | . |
| XS1 | 1 | 67263835 | TC | T | 0.273 | NM_005478 | INSL5 | frameshift_deleti  on | exon2 | c.268delG | p.E90fs | . | . |
| XS1 | 1 | 78024345 | C | CTAT | 0.963 | NM_012093 | AK5 | nonframeshift_in  sertion | exon14 | c.1601_1602ins  TAT | p.S534delinsSI | . | . |
| XS1 | 1 | 78024348 | T | TTA | 0.923 | NM_012093 | AK5 | frameshift_inserti  on | exon14 | c.1604_1605ins  TA | p.I535fs | . | . |
| XS1 | 1 | 78181506 | AC | A | 0.3 | NM_201624 | USP33 | frameshift_deleti  on | exon18 | c.1965delG | p.W655fs | . | . |
| XS1 | 1 | 84417530 | AT | A | 0.651 | NM_024686 | TTLL7 | frameshift_deleti  on | exon3 | c.154delA | p.I52fs | . | . |
| XS1 | 1 | 86591460 | TA | T | 0.336 | NM_152890 | COL24A1 | frameshift_deleti  on | exon3 | c.558delT | p.F186fs | . | . |
| XS1 | 1 | 87045895 | ATCCTA  C | A | 1 | NM_012128 | CLCA4 | nonframeshift_de  letion | exon14 | c.2628_2633del | p.876_878del | . | . |
| XS1 | 1 | 87045896 | TCCTAC  A | T | 1 | NM_012128 | CLCA4 | nonframeshift_de  letion | exon14 | c.2629_2634del | p.877_878del | . | . |
| XS1 | 1 | 89270197 | CA | C | 0.286 | NM_006256 | PKN2 | frameshift_deleti  on | exon9 | c.1406delA | p.Q469fs | . | . |
| XS1 | 1 | 89287645 | TC | T | 0.667 | NM_006256 | PKN2 | frameshift_deleti  on | exon17 | c.2302delC | p.L768fs | . | . |
| XS1 | 1 | 89575863 | CT | C | 0.351 | NM_004120 | GBP2 | frameshift_deleti  on | exon9 | c.1448delA | p.K483fs | . | . |
| XS1 | 1 | 89658602 | AG | A | 0.349 | NM_052941 | GBP4 | frameshift_deleti  on | exon5 | c.654delC | p.A218fs | . | . |
| XS1 | 1 | 90058389 | GA | G | 0.303 | NM_015350 | LRRC8B | frameshift_deleti  on | exon6 | c.2200delA | p.K734fs | . | . |
| XS1 | 1 | 90484300 | TC | T | 0.6 | NM_182976 | ZNF326 | frameshift_deleti  on | exon9 | c.1132delC | p.Q378fs | . | . |
| XS1 | 1 | 97236266 | A | AT | 0.5 | NM_021190 | PTBP2 | frameshift_inserti  on | exon5 | c.292dupT | p.A97fs | . | . |
| XS1 | 1 | 99771918 | CAA | C | 0.161 | NM_001166252 | LPPR4 | frameshift_deleti  on | exon6 | c.1471_1472del | p.K491fs | . | . |
| XS1 | 1 | 104089631 | CT | C | 0.6 | NM_017619 | RNPC3 | frameshift_deleti  on | exon12 | c.1336delT | p.F446fs | . | . |
| XS1 | 1 | 108697672 | C | CT | 0.667 | NM_013386 | SLC25A24 | frameshift_inserti  on | exon6 | c.754dupA | p.R252fs | . | . |
| XS1 | 1 | 109242455 | CA | C | 0.745 | NM_018061 | PRPF38B | frameshift_deleti  on | exon6 | c.1455delA | p.S485fs | . | . |
| XS1 | 1 | 110033679 | GC | G | 0.59 | NM_153340 | ATXN7L2 | frameshift_deleti  on | exon10 | c.1495delC | p.P499fs | . | . |
| XS1 | 1 | 110148701 | TG | T | 0.56 | NM_005272 | GNAT2 | frameshift_deleti  on | exon6 | c.610delC | p.Q204fs | . | . |
| XS1 | 1 | 110282518 | TG | T | 0.774 | NM_000849 | GSTM3 | frameshift_deleti  on | exon3 | c.60delC | p.A20fs | . | . |
| XS1 | 1 | 110925572 | AT | A | 0.286 | NM_001201546 | SLC16A4 | frameshift_deleti  on | exon3 | c.103delA | p.M35fs | . | . |
| XS1 | 1 | 111957501 | CTCACA  GACTGA TGACTC | C | 1 | NM_002557 | OVGP1 | nonframeshift_de letion | exon11 | c.1598_1621del | p.533_541del | . | . |
| XS1 | 1 | 113235436 | TG | T | 0.952 | NM_001130079 | MOV10 | frameshift_deleti  on | exon7 | c.1026delG | p.L342fs | . | . |
| XS1 | 1 | 113657251 | TG | T | 0.8 | NM_014813 | LRIG2 | frameshift_deleti  on | exon15 | c.2284delG | p.G762fs | . | . |
| XS1 | 1 | 114267494 | AT | A | 0.484 | NM_006608 | PHTF1 | frameshift_deleti  on | exon6 | c.509delA | p.N170fs | . | . |
| XS1 | 1 | 114437689 | A | AT | 0.333 | NM_001253852 | AP4B1 | stoploss | exon10 | c.2217dupA | p.X740delinsI | . | . |
| XS1 | 1 | 114680416 | CA | C | 0.786 | NM_001270805 | SYT6 | frameshift_deleti on | exon3 | c.516delT | p.P172fs | . | . |
| XS1 | 1 | 117122269 | GGTC | G | 0.3 | NM_001007237 | IGSF3 | nonframeshift_de  letion | exon10 | c.3076_3078del | p.1026_1026de  l | . | . |
| XS1 | 1 | 117122282 | GTCGTC  GTCC | G | 0.117 | NM_001007237 | IGSF3 | nonframeshift_de  letion | exon10 | c.3057_3065del | p.1019_1022de  l | . | . |
| XS1 | 1 | 117122288 | GTCC | G | 0.472 | NM_001007237 | IGSF3 | nonframeshift_de  letion | exon10 | c.3057_3059del | p.1019_1020de  l | . | . |
| XS1 | 1 | 117661074 | C | CT | 0.216 | NM_001145635 | TRIM45 | frameshift_inserti  on | exon2 | c.803dupA | p.K268fs | . | . |
| XS1 | 1 | 118430335 | CT | C | 0.44 | NM_001135589 | GDAP2 | frameshift_deleti  on | exon9 | c.1014delA | p.K338fs | . | . |
| XS1 | 1 | 118565927 | CT | C | 0.247 | NM_206996 | SPAG17 | frameshift_deleti  on | exon28 | c.4068delA | p.K1356fs | . | . |
| XS1 | 1 | 145439786 | GA | G | 0.333 | NM_006472 | TXNIP | frameshift_deleti  on | exon3 | c.333delA | p.G111fs | . | . |
| XS1 | 1 | 145609243 | CG | C | 0.429 | NM_006468 | POLR3C | frameshift_deleti  on | exon2 | c.93delC | p.T31fs | . | . |
| XS1 | 1 | 149915866 | TG | T | 0.722 | NM_020205 | OTUD7B | stopgain | exon12 | c.2421delC | p.C807X | . | . |
| XS1 | 1 | 150129660 | AG | A | 0.652 | NM_016274 | PLEKHO1 | frameshift_deleti on | exon5 | c.506delG | p.R169fs | . | . |
| XS1 | 1 | 150199039 | CTCCTCT | C | 1 | NM_001136478 | ANP32E | nonframeshift_de  letion | exon4 | c.453_458del | p.151_153del | . | . |
| XS1 | 1 | 150551491 | GTCC | G | 0.25 | NM_021960 | MCL1 | nonframeshift_de  letion | exon1 | c.513_515del | p.171_172del | . | . |
| XS1 | 1 | 151259722 | T | TC | 0.679 | NM_020832 | ZNF687 | frameshift_inserti  on | exon2 | c.956dupC | p.S319fs | . | . |
| XS1 | 1 | 151259793 | CACA | C | 0.2 | NM_020832 | ZNF687 | nonframeshift_de  letion | exon2 | c.1027_1029del | p.343_343del | . | . |
| XS1 | 1 | 151315080 | TC | T | 0.5 | NM_000449 | RFX5 | frameshift_deleti  on | exon11 | c.1432delG | p.E478fs | . | . |
| XS1 | 1 | 151783886 | CT | C | 0.319 | NM_001001523 | RORC | frameshift_deleti  on | exon9 | c.1246delA | p.R416fs | . | . |
| XS1 | 1 | 152127466 | GT | G | 0.299 | NM_001122965 | RPTN | frameshift_deleti  on | exon3 | c.2108delA | p.H703fs | . | . |
| XS1 | 1 | 152185611 | GA | G | 0.8 | NM_001009931 | HRNR | frameshift_deleti  on | exon3 | c.8493delT | p.F2831fs | . | . |
| XS1 | 1 | 152681680 | C | CAGCT  CTGGG GGCTG | 0.393 | NM_178356 | LCE4A | nonframeshift_in sertion | exon1 | c.129_130insA  GCTCTGGGGG CTGCTGT | p.S43delinsSSS GGCC | . | . |
| XS1 | 1 | 153748613 | GGC | G | 0.179 | NM_024330 | SLC27A3 | frameshift_deleti  on | exon1 | c.782_783del | p.G261fs | . | . |
| XS1 | 1 | 153907278 | CCTG | C | 0.455 | NM_014856 | DENND4B | nonframeshift_de  letion | exon18 | c.2728_2730del | p.910_910del | . | . |
| XS1 | 1 | 153907306 | TTGC | T | 0.348 | NM_014856 | DENND4B | nonframeshift_de  letion | exon18 | c.2700_2702del | p.900_901del | . | . |
| XS1 | 1 | 153921011 | TG | T | 0.852 | NM_181715 | CRTC2 | frameshift_deleti  on | exon13 | c.1783delC | p.Q595fs | . | . |
| XS1 | 1 | 153924636 | TG | T | 0.433 | NM_181715 | CRTC2 | frameshift_deleti  on | exon10 | c.854delC | p.P285fs | . | . |

| XS1 | 1 | 153935171 | TG | T | 0.367 | NM_001271957 | SLC39A1 | frameshift_deleti  on | exon2 | c.20delC | p.P7fs | . | . |
| --- | --- | --- | --- | --- | --- | --- | --- | --- | --- | --- | --- | --- | --- |
| XS1 | 1 | 154067437 | TC | T | 0.938 | NM_001159484 | NUP210L | frameshift_deleti  on | exon15 | c.2160delG | p.R720fs | . | . |
| XS1 | 1 | 154474013 | TG | T | 0.962 | NM_001010846 | SHE | frameshift_deleti  on | exon1 | c.489delC | p.S163fs | . | . |
| XS1 | 1 | 154842199 | G | GGCGG  CT | 1 | NM_001204087 | KCNN3 | nonframeshift_in  sertion | exon1 | c.241_242insA  GCCGC | p.P81delinsQP  P | . | . |
| XS1 | 1 | 154842199 | G | GGCT | 1 | NM_001204087 | KCNN3 | nonframeshift_in  sertion | exon1 | c.241_242insA  GC | p.P81delinsQP | . | . |
| XS1 | 1 | 154842330 | TTGC | T | 0.273 | NM_001204087 | KCNN3 | nonframeshift_de  letion | exon1 | c.108_110del | p.36_37del | . | . |
| XS1 | 1 | 155112532 | GC | G | 0.6 | NM_018973 | DPM3 | frameshift_deleti  on | exon1 | c.274delG | p.A92fs | . | . |
| XS1 | 1 | 155230335 | CT | C | 0.875 | NM_052837 | SCAMP3 | frameshift_deleti  on | exon2 | c.181delA | p.S61fs | . | . |
| XS1 | 1 | 155886501 | CT | C | 0.6 | NM_014949 | KIAA0907 | frameshift_deleti  on | exon12 | c.1467delA | p.L489fs | . | . |
| XS1 | 1 | 155935423 | CA | C | 0.421 | NM_001162383 | ARHGEF2 | frameshift_deleti  on | exon5 | c.468delT | p.A156fs | . | . |
| XS1 | 1 | 156047412 | GC | G | 0.244 | NM_001093725 | MEX3A | frameshift_deleti  on | exon2 | c.515delG | p.G172fs | . | . |
| XS1 | 1 | 156215004 | GC | G | 0.3 | NM_001272106 | PAQR6 | frameshift_deleti  on | exon4 | c.137delG | p.G46fs | . | . |
| XS1 | 1 | 156233251 | G | GA | 1 | NM_015327 | SMG5 | frameshift_inserti  on | exon13 | c.1965dupT | p.P656fs | . | . |
| XS1 | 1 | 156256163 | CTT | C | 0.455 | NM_032323 | TMEM79 | frameshift_deleti  on | exon3 | c.871_872del | p.F291fs | . | . |
| XS1 | 1 | 156565049 | A | AAC | 1 | NM_015590 | GPATCH4 | frameshift_inserti  on | exon8 | c.1083_1084ins  GT | p.F362fs | . | . |
| XS1 | 1 | 156696975 | TG | T | 0.5 | NM_030980 | ISG20L2 | frameshift_deleti  on | exon1 | c.469delC | p.Q157fs | . | . |
| XS1 | 1 | 156897416 | TC | T | 1 | NM_144702 | LRRC71 | frameshift_deleti  on | exon7 | c.792delC | p.I264fs | . | . |
| XS1 | 1 | 156926294 | GCC | G | 0.961 | NM_014784 | ARHGEF11 | frameshift_deleti  on | exon18 | c.1467_1468del | p.E489fs | . | . |
| XS1 | 1 | 156926295 | CCT | C | 0.959 | NM_014784 | ARHGEF11 | frameshift_deleti  on | exon18 | c.1466_1467del | p.E489fs | . | . |
| XS1 | 1 | 158532870 | GT | G | 0.176 | NM_001160325 | OR6P1 | frameshift_deleti  on | exon1 | c.524delA | p.N175fs | . | . |
| XS1 | 1 | 158687607 | TG | T | 0.34 | NM_001005327 | OR6K3 | frameshift_deleti  on | exon1 | c.298delC | p.Q100fs | . | . |
| XS1 | 1 | 159019242 | CA | C | 0.49 | NM_001206567 | IFI16 | frameshift_deleti  on | exon8 | c.1351delA | p.K451fs | . | . |
| XS1 | 1 | 159858276 | CT | C | 0.175 | NM_012337 | CCDC19 | frameshift_deleti  on | exon4 | c.282delA | p.T94fs | . | . |
| XS1 | 1 | 161011462 | GC | G | 0.268 | NM_001276373 | USF1 | frameshift_deleti  on | exon6 | c.450delG | p.G150fs | . | . |
| XS1 | 1 | 161196308 | TG | T | 0.4 | NM_001286373 | TOMM40L | frameshift_deleti  on | exon2 | c.30delG | p.M10fs | . | . |
| XS1 | 1 | 161202999 | CG | C | 0.333 | NM_001077470 | NR1I3 | frameshift_deleti  on | exon3 | c.280delC | p.R94fs | . | . |
| XS1 | 1 | 161642879 | GA | G | 0.833 | NM_001190828 | FCGR2B | frameshift_deleti  on | exon3 | c.486delA | p.G162fs | . | . |
| XS1 | 1 | 161823081 | AC | A | 0.625 | NM_007348 | ATF6 | frameshift_deleti  on | exon12 | c.1502delC | p.T501fs | . | . |
| XS1 | 1 | 165380301 | TC | T | 0.418 | NM_001256571 | RXRG | frameshift_deleti  on | exon4 | c.298delG | p.E100fs | . | . |
| XS1 | 1 | 167973980 | GA | G | 0.244 | NM_001198957 | DCAF6 | frameshift_deleti  on | exon9 | c.1235delA | p.E412fs | . | . |
| XS1 | 1 | 169454827 | GC | G | 0.326 | NM_006996 | SLC19A2 | frameshift_deleti  on | exon1 | c.177delG | p.G59fs | . | . |
| XS1 | 1 | 169494076 | CA | C | 0.857 | NM_000130 | F5 | frameshift_deleti  on | exon19 | c.5786delT | p.L1929fs | . | . |
| XS1 | 1 | 169566304 | AG | A | 0.235 | NM_003005 | SELP | frameshift_deleti  on | exon11 | c.1815delC | p.G605fs | . | . |
| XS1 | 1 | 171502027 | G | GA | 0.5 | NM_015172 | PRRC2C | frameshift_inserti  on | exon12 | c.1795dupA | p.E598fs | . | . |
| XS1 | 1 | 172525114 | A | AT | 0.241 | NM_001282750 | SUCO | frameshift_inserti  on | exon3 | c.283_284insT | p.N95fs | . | . |
| XS1 | 1 | 173454533 | GA | G | 0.342 | NM_004905 | PRDX6 | frameshift_deleti  on | exon3 | c.287delA | p.E96fs | . | . |
| XS1 | 1 | 174190258 | CA | C | 0.544 | NM_014857 | RABGAP1L | frameshift_deleti  on | exon3 | c.288delA | p.T96fs | . | . |
| XS1 | 1 | 175372606 | C | CCA | 0.109 | NM_003285 | TNR | frameshift_inserti  on | exon4 | c.645_646insTG | p.D216fs | . | . |
| XS1 | 1 | 176640116 | AGT | A | 0.25 | NM_020318 | PAPPA2 | frameshift_deleti  on | exon4 | c.2003_2004del | p.S668fs | . | . |
| XS1 | 1 | 176934328 | CA | C | 0.244 | NM_001286164 | ASTN1 | frameshift_deleti  on | exon9 | c.1568delT | p.L523fs | . | . |
| XS1 | 1 | 179077629 | T | TA | 0.321 | NM_001168236 | ABL2 | frameshift_inserti  on | exon11 | c.2709_2710ins  T | p.T904fs | . | . |
| XS1 | 1 | 179526285 | GC | G | 0.275 | NM_014625 | NPHS2 | frameshift_deleti  on | exon5 | c.614delG | p.S205fs | . | . |
| XS1 | 1 | 180063416 | GA | G | 0.667 | NM_014810 | CEP350 | frameshift_deleti  on | exon34 | c.8177delA | p.E2726fs | . | . |
| XS1 | 1 | 182856279 | AG | A | 1 | NM_001357 | DHX9 | frameshift_deleti  on | exon28 | c.3524delG | p.R1175fs | . | . |
| XS1 | 1 | 184675823 | CT | C | 0.647 | NM_025191 | EDEM3 | frameshift_deleti  on | exon18 | c.2156delA | p.K719fs | . | . |
| XS1 | 1 | 184764223 | TG | T | 0.594 | NM_052966 | FAM129A | frameshift_deleti  on | exon14 | c.2674delC | p.Q892fs | . | . |
| XS1 | 1 | 197611930 | C | CA | 0.45 | NM_001195215 | DENND1B | frameshift_inserti  on | exon10 | c.582dupT | p.V195fs | . | . |
| XS1 | 1 | 200817470 | CA | C | 0.776 | NM_203459 | CAMSAP2 | frameshift_deleti  on | exon11 | c.1574delA | p.Q525fs | . | . |
| XS1 | 1 | 200818955 | GTA | G | 0.238 | NM_203459 | CAMSAP2 | frameshift_deleti  on | exon11 | c.3059_3060del | p.V1020fs | . | . |
| XS1 | 1 | 202700994 | GA | G | 0.5 | NM_006618 | KDM5B | frameshift_deleti  on | exon24 | c.3982delT | p.S1328fs | . | . |
| XS1 | 1 | 203022914 | GC | G | 0.375 | NM_015053 | PPFIA4 | frameshift_deleti  on | exon2 | c.137delC | p.A46fs | . | . |
| XS1 | 1 | 203186950 | C | CAGAC  CATGG GCCCG CCCAG | 0.429 | NM_001256125 | CHIT1 | stopgain | exon9 | c.1015_1016ins  AGGGACTGGG CGGGCCCATG GTCT | p.W339delinsX | . | . |
| XS1 | 1 | 204380141 | GTC | G | 0.188 | NM_032833 | PPP1R15B | frameshift_deleti  on | exon1 | c.397_398del | p.D133fs | . | . |
| XS1 | 1 | 204438041 | TTTCGG | T | 0.109 | NM_002646 | PIK3C2B | frameshift_deleti  on | exon3 | c.885_889del | p.N295fs | . | . |
| XS1 | 1 | 204957891 | TG | T | 0.25 | NM_001005388 | NFASC | frameshift_deleti  on | exon23 | c.2725delG | p.G909fs | . | . |
| XS1 | 1 | 205741687 | CA | C | 0.8 | NM_001135662 | RAB7L1 | frameshift_deleti  on | exon3 | c.132delT | p.F44fs | . | . |

| XS1 | 1 | 205770128 | GC | G | 0.351 | NM_173854 | SLC41A1 | frameshift_deleti  on | exon3 | c.432delG | p.G144fs | . | . |
| --- | --- | --- | --- | --- | --- | --- | --- | --- | --- | --- | --- | --- | --- |
| XS1 | 1 | 205813295 | GC | G | 0.421 | NM_152491 | PM20D1 | frameshift_deleti  on | exon5 | c.626delG | p.G209fs | . | . |
| XS1 | 1 | 206666642 | AG | A | 0.543 | NM_001193321 | IKBKE | frameshift_deleti  on | exon19 | c.1722delG | p.K574fs | . | . |
| XS1 | 1 | 206765134 | CT | C | 0.31 | NM_001201478 | EIF2D | frameshift_deleti  on | exon13 | c.1355delA | p.K452fs | . | . |
| XS1 | 1 | 206772925 | TG | T | 0.476 | NM_001201478 | EIF2D | frameshift_deleti  on | exon8 | c.721delC | p.Q241fs | . | . |
| XS1 | 1 | 207286381 | CA | C | 0.611 | NM_000715 | C4BPA | frameshift_deleti  on | exon2 | c.12delA | p.P4fs | . | . |
| XS1 | 1 | 207627788 | GT | G | 1 | NM_001006658 | CR2 | frameshift_deleti  on | exon1 | c.26delT | p.V9fs | . | . |
| XS1 | 1 | 207871200 | AT | A | 0.617 | NM_175710 | CR1L | frameshift_deleti  on | exon7 | c.1125delT | p.D375fs | . | . |
| XS1 | 1 | 207874908 | CA | C | 0.857 | NM_175710 | CR1L | frameshift_deleti  on | exon9 | c.1274delA | p.H425fs | . | . |
| XS1 | 1 | 214537862 | TG | T | 0.386 | NM_005401 | PTPN14 | frameshift_deleti  on | exon18 | c.3427delC | p.H1143fs | . | . |
| XS1 | 1 | 216062030 | AC | A | 0.789 | NM_206933 | USH2A | frameshift_deleti  on | exon41 | c.7960delG | p.V2654fs | . | . |
| XS1 | 1 | 220363418 | AT | A | 0.296 | NM_012414 | RAB3GAP2 | frameshift_deleti  on | exon16 | c.1701delA | p.K567fs | . | . |
| XS1 | 1 | 222833330 | CA | C | 0.786 | NM_198551 | MIA3 | frameshift_deleti  on | exon23 | c.4958delA | p.Q1653fs | . | . |
| XS1 | 1 | 223285692 | G | GC | 0.708 | NM_003268 | TLR5 | frameshift_inserti  on | exon6 | c.681dupG | p.L228fs | . | . |
| XS1 | 1 | 223401014 | G | GT | 0.324 | NM_017982 | SUSD4 | frameshift_inserti  on | exon7 | c.982dupA | p.T328fs | . | . |
| XS1 | 1 | 223934835 | CA | C | 0.767 | NM_001146068 | CAPN2 | frameshift_deleti  on | exon5 | c.464delA | p.Q155fs | . | . |
| XS1 | 1 | 225156507 | CT | C | 0.23 | NM_001145154 | DNAH14 | frameshift_deleti  on | exon7 | c.699delT | p.T233fs | . | . |
| XS1 | 1 | 225231636 | GT | G | 0.229 | NM_001373 | DNAH14 | frameshift_deleti  on | exon15 | c.1864delT | p.F622fs | . | . |
| XS1 | 1 | 225539568 | CA | C | 0.571 | NM_001373 | DNAH14 | frameshift_deleti  on | exon70 | c.11238delA | p.T3746fs | . | . |
| XS1 | 1 | 226034839 | TCTG | T | 0.222 | NM_014698 | TMEM63A | nonframeshift_de  letion | exon24 | c.2323_2325del | p.775_775del | . | . |
| XS1 | 1 | 226552718 | CG | C | 0.743 | NM_001618 | PARP1 | frameshift_deleti  on | exon19 | c.2642delC | p.P881fs | . | . |
| XS1 | 1 | 227441792 | CT | C | 0.857 | NM_003607 | CDC42BPA | frameshift_deleti  on | exon2 | c.242delA | p.K81fs | . | . |
| XS1 | 1 | 228288915 | AG | A | 1 | NM_024319 | C1orf35 | frameshift_deleti  on | exon8 | c.708delC | p.P236fs | . | . |
| XS1 | 1 | 228479620 | A | AG | 0.579 | NM_001098623 | OBSCN | frameshift_inserti  on | exon39 | c.10362dupG | p.E3454fs | . | . |
| XS1 | 1 | 228509453 | TG | T | 0.75 | NM_001098623 | OBSCN | frameshift_deleti  on | exon55 | c.14912delG | p.C4971fs | . | . |
| XS1 | 1 | 228547864 | TGGA | T | 0.235 | NM_052843 | OBSCN | nonframeshift_de  letion | exon81 | c.19272_19274  del | p.6424_6425de  l | . | . |
| XS1 | 1 | 228556041 | TC | T | 0.348 | NM_001098623 | OBSCN | frameshift_deleti  on | exon88 | c.19692delC | p.V6564fs | . | . |
| XS1 | 1 | 228558880 | GC | G | 0.236 | NM_001098623 | OBSCN | frameshift_deleti  on | exon94 | c.20402delC | p.A6801fs | . | . |
| XS1 | 1 | 229773596 | AG | A | 0.875 | NM_014777 | URB2 | frameshift_deleti  on | exon4 | c.3237delG | p.E1079fs | . | . |
| XS1 | 1 | 229773598 | GC | G | 0.868 | NM_014777 | URB2 | frameshift_deleti  on | exon4 | c.3239delC | p.A1080fs | . | . |
| XS1 | 1 | 231067170 | C | CT | 0.375 | NM_001122835 | TTC13 | frameshift_inserti  on | exon9 | c.1014dupA | p.G339fs | . | . |
| XS1 | 1 | 231830164 | CG | C | 0.529 | NM_001012957 | DISC1 | frameshift_deleti  on | exon2 | c.661delG | p.G221fs | . | . |
| XS1 | 1 | 233363045 | TG | T | 0.212 | NM_014801 | PCNXL2 | stopgain | exon10 | c.2430delC | p.Y810X | . | . |
| XS1 | 1 | 235605135 | GT | G | 0.227 | NM_001287802 | TBCE | frameshift_deleti on | exon13 | c.939delT | p.G313fs | . | . |
| XS1 | 1 | 235918905 | CT | C | 0.474 | NM_000081 | LYST | frameshift_deleti  on | exon25 | c.7101delA | p.K2367fs | . | . |
| XS1 | 1 | 236702306 | CG | C | 0.857 | NM_201544 | LGALS8 | frameshift_deleti  on | exon4 | c.263delG | p.R88fs | . | . |
| XS1 | 1 | 236899899 | TC | T | 1 | NM_001278344 | ACTN2 | frameshift_deleti  on | exon10 | c.91delC | p.P31fs | . | . |
| XS1 | 1 | 243388559 | CA | C | 1 | NM_001042404 | CEP170 | frameshift_deleti  on | exon2 | c.23delT | p.L8fs | . | . |
| XS1 | 1 | 247201386 | CA | C | 0.591 | NM_001204220 | ZNF670 | frameshift_deleti  on | exon4 | c.531delT | p.F177fs | . | . |
| XS1 | 1 | 247597424 | TC | T | 1 | NM_001127462 | NLRP3 | frameshift_deleti  on | exon4 | c.2177delC | p.S726fs | . | . |
| XS1 | 1 | 247978541 | AAGG | A | 0.989 | NM_001001966 | OR14A16 | nonframeshift_de  letion | exon1 | c.488_490del | p.163_164del | . | . |
| XS1 | 1 | 248202102 | G | GA | 0.429 | NM_001004686 | OR2L2 | stopgain | exon1 | c.533_534insA | p.C178_D179d  elinsX | . | . |
| XS1 | 1 | 248512406 | TC | T | 0.8 | NM_001001918 | OR14C36 | frameshift_deleti  on | exon1 | c.331delC | p.L111fs | . | . |
| XS1 | 1 | 248525638 | CA | C | 0.602 | NM_001004696 | OR2T4 | frameshift_deleti  on | exon1 | c.757delA | p.I253fs | . | . |
| XS1 | 1 | 248637494 | CT | C | 0.262 | NM_001005495 | OR2T3 | frameshift_deleti  on | exon1 | c.844delT | p.F282fs | . | . |
| XS1 | 1 | 248801944 | TCAGCA  CG | T | 0.2 | NM_001001827 | OR2T35 | frameshift_deleti  on | exon1 | c.609_615del | p.C203fs | . | . |
| XS1 | 1 | 249106430 | AG | A | 0.323 | NM_030645 | SH3BP5L | frameshift_deleti  on | exon7 | c.850delC | p.L284fs | . | . |
| XS1 | 2 | 8871962 | CG | C | 0.247 | NM_020738 | KIDINS220 | frameshift_deleti  on | exon30 | c.4203delC | p.P1401fs | . | . |
| XS1 | 2 | 10053346 | GA | G | 0.662 | NM_005680 | TAF1B | frameshift_deleti  on | exon12 | c.1240delA | p.K414fs | . | . |
| XS1 | 2 | 17696694 | GC | G | 0.806 | NM_001099218 | RAD51AP2 | frameshift_deleti  on | exon1 | c.2988delG | p.G996fs | . | . |
| XS1 | 2 | 20237355 | CA | C | 0.895 | NM_014713 | LAPTM4A | frameshift_deleti  on | exon3 | c.252delT | p.F84fs | . | . |
| XS1 | 2 | 21229804 | A | AT | 0.5 | NM_000384 | APOB | frameshift_inserti  on | exon26 | c.9935dupA | p.N3312fs | . | . |
| XS1 | 2 | 24413343 | TA | T | 0.2 | NM_001040710 | FAM228A | frameshift_deleti  on | exon6 | c.465delA | p.L155fs | . | . |
| XS1 | 2 | 26415211 | TA | T | 0.588 | NM_000182 | HADHA | stopgain | exon18 | c.1967delT | p.L656X | . | . |
| XS1 | 2 | 27247058 | GA | G | 1 | NM_012326 | MAPRE3 | frameshift_deleti on | exon4 | c.363delA | p.G121fs | . | . |
| XS1 | 2 | 27263276 | CT | C | 0.889 | NM_001083590 | TMEM214 | frameshift_deleti  on | exon15 | c.1721delT | p.L574fs | . | . |
| XS1 | 2 | 27361153 | CA | C | 0.244 | NM_178553 | C2orf53 | frameshift_deleti  on | exon3 | c.44delT | p.V15fs | . | . |
| XS1 | 2 | 27456981 | AC | A | 1 | NM_004341 | CAD | frameshift_deleti  on | exon22 | c.3506delC | p.T1169fs | . | . |

| XS1 | 2 | 27465778 | TG | T | 0.362 | NM_004341 | CAD | frameshift_deleti  on | exon42 | c.6418delG | p.D2140fs | . | . |
| --- | --- | --- | --- | --- | --- | --- | --- | --- | --- | --- | --- | --- | --- |
| XS1 | 2 | 27565043 | A | AG | 0.524 | NM_001035521 | GTF3C2 | frameshift_inserti  on | exon4 | c.626dupC | p.P209fs | . | . |
| XS1 | 2 | 27601809 | TG | T | 1 | NM_001201459 | ZNF513 | frameshift_deleti  on | exon2 | c.137delC | p.P46fs | . | . |
| XS1 | 2 | 27883889 | G | GA | 0.9 | NM_001282729 | SUPT7L | frameshift_inserti  on | exon3 | c.380dupT | p.F127fs | . | . |
| XS1 | 2 | 29294741 | AT | A | 0.756 | NM_001029883 | C2orf71 | stopgain | exon1 | c.2386delA | p.I796X | . | . |
| XS1 | 2 | 29358441 | AT | A | 0.571 | NM_001287527 | CLIP4 | frameshift_deleti on | exon6 | c.558delT | p.N186fs | . | . |
| XS1 | 2 | 29386766 | AT | A | 0.829 | NM_001287527 | CLIP4 | frameshift_deleti  on | exon13 | c.1605delT | p.Y535fs | . | . |
| XS1 | 2 | 30966411 | GA | G | 1 | NM_144575 | CAPN13 | frameshift_deleti  on | exon13 | c.1282delT | p.S428fs | . | . |
| XS1 | 2 | 30987131 | TC | T | 0.167 | NM_144575 | CAPN13 | frameshift_deleti  on | exon6 | c.565delG | p.E189fs | . | . |
| XS1 | 2 | 32475325 | AC | A | 0.312 | NM_001199138 | NLRC4 | frameshift_deleti  on | exon4 | c.1607delG | p.S536fs | . | . |
| XS1 | 2 | 32640122 | AC | A | 0.833 | NM_016252 | BIRC6 | frameshift_deleti  on | exon10 | c.1764delC | p.H588fs | . | . |
| XS1 | 2 | 32889449 | GT | G | 1 | NM_001193509 | TTC27 | frameshift_deleti  on | exon6 | c.571delT | p.F191fs | . | . |
| XS1 | 2 | 36669772 | TG | T | 0.553 | NM_016441 | CRIM1 | frameshift_deleti  on | exon4 | c.764delG | p.C255fs | . | . |
| XS1 | 2 | 36749241 | CT | C | 0.5 | NM_016441 | CRIM1 | frameshift_deleti  on | exon13 | c.2214delT | p.P738fs | . | . |
| XS1 | 2 | 37316943 | GA | G | 0.556 | NM_174931 | GPATCH11 | frameshift_deleti  on | exon3 | c.287delA | p.E96fs | . | . |
| XS1 | 2 | 38178884 | AC | A | 0.453 | NM_144713 | RMDN2 | frameshift_deleti  on | exon2 | c.527delC | p.T176fs | . | . |
| XS1 | 2 | 39479010 | GA | G | 1 | NM_001270425 | MAP4K3 | frameshift_deleti  on | exon32 | c.2494delT | p.S832fs | . | . |
| XS1 | 2 | 44566399 | A | AG | 0.6 | NM_001042385 | PREPL | frameshift_inserti  on | exon6 | c.855_856insC | p.W286fs | . | . |
| XS1 | 2 | 46411896 | AT | A | 0.559 | NM_005400 | PRKCE | frameshift_deleti  on | exon15 | c.2091delT | p.N697fs | . | . |
| XS1 | 2 | 54080879 | TC | T | 1 | NM_006794 | GPR75 | frameshift_deleti  on | exon2 | c.1014delG | p.G338fs | . | . |
| XS1 | 2 | 54081368 | CA | C | 0.889 | NM_006794 | GPR75 | frameshift_deleti  on | exon2 | c.525delT | p.L175fs | . | . |
| XS1 | 2 | 55047560 | GC | G | 0.412 | NM_001039753 | EML6 | frameshift_deleti  on | exon4 | c.487delC | p.P163fs | . | . |
| XS1 | 2 | 55071356 | TG | T | 0.667 | NM_001039753 | EML6 | stopgain | exon7 | c.1021delG | p.V341X | . | . |
| XS1 | 2 | 55254269 | T | TA | 0.75 | NM_020532 | RTN4 | frameshift_inserti on | exon3 | c.965_966insT | p.E322fs | . | . |
| XS1 | 2 | 55522942 | AT | A | 0.385 | NM_018084 | CCDC88A | stopgain | exon30 | c.5257delA | p.I1753X | . | . |
| XS1 | 2 | 55536064 | G | GT | 0.222 | NM_001135597 | CCDC88A | frameshift_inserti on | exon25 | c.4264dupA | p.T1422fs | . | . |
| XS1 | 2 | 56420535 | CG | C | 0.308 | NM_001080433 | CCDC85A | frameshift_deleti  on | exon2 | c.1201delG | p.G401fs | . | . |
| XS1 | 2 | 56599535 | CT | C | 0.5 | NM_001080433 | CCDC85A | frameshift_deleti  on | exon4 | c.1375delT | p.W459fs | . | . |
| XS1 | 2 | 61180162 | C | CT | 0.176 | NM_144709 | PUS10 | frameshift_inserti  on | exon15 | c.1277dupA | p.K426fs | . | . |
| XS1 | 2 | 63631604 | TG | T | 0.8 | NM_015910 | WDPCP | stopgain | exon10 | c.1013delC | p.S338X | . | . |
| XS1 | 2 | 65299068 | GAT | G | 0.611 | NM_015147 | CEP68 | frameshift_deleti on | exon3 | c.839_840del | p.D280fs | . | . |
| XS1 | 2 | 68364551 | CT | C | 0.6 | NM_001256476 | WDR92 | frameshift_deleti  on | exon6 | c.647delA | p.E216fs | . | . |
| XS1 | 2 | 69049553 | GA | G | 0.466 | NM_001166276 | ARHGAP25 | frameshift_deleti  on | exon9 | c.1262delA | p.E421fs | . | . |
| XS1 | 2 | 69420468 | G | GA | 0.357 | NM_032208 | ANTXR1 | frameshift_inserti  on | exon17 | c.1356dupA | p.G452fs | . | . |
| XS1 | 2 | 69741753 | CTGT | C | 1 | NM_014911 | AAK1 | nonframeshift_de  letion | exon13 | c.1623_1625del | p.541_542del | . | . |
| XS1 | 2 | 70035049 | TG | T | 0.704 | NM_001153 | ANXA4 | frameshift_deleti  on | exon6 | c.319delG | p.D107fs | . | . |
| XS1 | 2 | 70045824 | TA | T | 0.474 | NM_001153 | ANXA4 | frameshift_deleti  on | exon10 | c.723delA | p.I241fs | . | . |
| XS1 | 2 | 70188725 | CG | C | 0.333 | NM_152792 | ASPRV1 | frameshift_deleti  on | exon1 | c.95delC | p.P32fs | . | . |
| XS1 | 2 | 70933496 | C | CG | 1 | NM_001185055 | ADD2 | frameshift_inserti  on | exon2 | c.92dupC | p.P31fs | . | . |
| XS1 | 2 | 71577285 | GC | G | 0.684 | NM_001014972 | ZNF638 | frameshift_deleti  on | exon2 | c.1202delC | p.A401fs | . | . |
| XS1 | 2 | 73675227 | T | TCTC | 1 | NM_015120 | ALMS1 | nonframeshift_in  sertion | exon8 | c.1570_1571ins  CTC | p.S524delinsSP | . | . |
| XS1 | 2 | 73676430 | CT | C | 0.44 | NM_015120 | ALMS1 | frameshift_deleti  on | exon8 | c.2774delT | p.L925fs | . | . |
| XS1 | 2 | 73762042 | CA | C | 1 | NM_015120 | ALMS1 | frameshift_deleti  on | exon12 | c.9871delA | p.K3291fs | . | . |
| XS1 | 2 | 74146562 | AT | A | 0.75 | NM_001199893 | ACTG2 | frameshift_deleti  on | exon8 | c.863delT | p.I288fs | . | . |
| XS1 | 2 | 74482995 | TCG | T | 0.159 | NM_021196 | SLC4A5 | frameshift_deleti  on | exon8 | c.930_931del | p.G310fs | . | . |
| XS1 | 2 | 85011901 | A | AT | 0.197 | NM_001370 | DNAH6 | frameshift_inserti  on | exon66 | c.10921dupT | p.T3640fs | . | . |
| XS1 | 2 | 85554588 | CT | C | 0.286 | NM_001206841 | TGOLN2 | frameshift_deleti  on | exon2 | c.266delA | p.K89fs | . | . |
| XS1 | 2 | 86832370 | AT | A | 0.242 | NM_005667 | RNF103 | frameshift_deleti  on | exon4 | c.653delA | p.Y218fs | . | . |
| XS1 | 2 | 95943167 | GC | G | 0.333 | NM_001165977 | PROM2 | frameshift_deleti  on | exon7 | c.829delC | p.Q277fs | . | . |
| XS1 | 2 | 96691322 | TG | T | 0.46 | NM_207328 | GPAT2 | frameshift_deleti  on | exon13 | c.1267delC | p.Q423fs | . | . |
| XS1 | 2 | 96780974 | T | TTCCTC  CTCC | 0.909 | NM_000682 | ADRA2B | nonframeshift_in  sertion | exon1 | c.914_915insG  GAGGAGGA | p.E305delinsEE  EE | . | . |
| XS1 | 2 | 96780986 | C | CTCCTC  CTCT | 1 | NM_000682 | ADRA2B | nonframeshift_in  sertion | exon1 | c.902_903insA  GAGGAGGA | p.E301delinsEE  EE | . | . |
| XS1 | 2 | 96781870 | AG | A | 1 | NM_000682 | ADRA2B | frameshift_deleti  on | exon1 | c.18delC | p.P6fs | . | . |
| XS1 | 2 | 96852580 | TC | T | 0.29 | NM_020151 | STARD7 | frameshift_deleti  on | exon8 | c.1000delG | p.D334fs | . | . |
| XS1 | 2 | 96920647 | G | GA | 0.238 | NM_001193304 | TMEM127 | frameshift_inserti  on | exon3 | c.332dupT | p.F111fs | . | . |
| XS1 | 2 | 96992727 | CT | C | 0.708 | NM_178495 | ITPRIPL1 | frameshift_deleti  on | exon1 | c.383delT | p.L128fs | . | . |
| XS1 | 2 | 96992792 | TGAG | T | 0.144 | NM_178495 | ITPRIPL1 | nonframeshift_de  letion | exon1 | c.448_450del | p.150_150del | . | . |

| XS1 | 2 | 97359661 | TG | T | 0.27 | NM_001113382 | FER1L5 | frameshift_deleti  on | exon32 | c.3570delG | p.L1190fs | . | . |
| --- | --- | --- | --- | --- | --- | --- | --- | --- | --- | --- | --- | --- | --- |
| XS1 | 2 | 97823898 | GA | G | 0.41 | NM_001164315 | ANKRD36 | frameshift_deleti  on | exon16 | c.1316delA | p.E439fs | . | . |
| XS1 | 2 | 98264555 | AG | A | 0.4 | NM_001862 | COX5B | frameshift_deleti  on | exon4 | c.375delG | p.Q125fs | . | . |
| XS1 | 2 | 98914463 | TG | T | 0.889 | NM_144992 | VWA3B | frameshift_deleti  on | exon24 | c.3252delG | p.V1084fs | . | . |
| XS1 | 2 | 99778897 | G | GT | 0.222 | NM_145199 | LIPT1 | frameshift_inserti  on | exon2 | c.478dupT | p.Q159fs | . | . |
| XS1 | 2 | 99978150 | TA | T | 1 | NM_015904 | EIF5B | frameshift_deleti  on | exon4 | c.787delA | p.K263fs | . | . |
| XS1 | 2 | 99980158 | GA | G | 0.462 | NM_015904 | EIF5B | frameshift_deleti  on | exon5 | c.971delA | p.E324fs | . | . |
| XS1 | 2 | 99980285 | TA | T | 0.575 | NM_015904 | EIF5B | frameshift_deleti  on | exon5 | c.1098delA | p.I366fs | . | . |
| XS1 | 2 | 103124683 | GA | G | 0.667 | NM_001011552 | SLC9A4 | frameshift_deleti  on | exon5 | c.1345delA | p.K449fs | . | . |
| XS1 | 2 | 106002964 | GC | G | 0.544 | NM_001450 | FHL2 | frameshift_deleti  on | exon2 | c.9delG | p.E3fs | . | . |
| XS1 | 2 | 106498301 | CA | C | 0.929 | NM_001004720 | NCK2 | frameshift_deleti  on | exon3 | c.745delA | p.K249fs | . | . |
| XS1 | 2 | 107057789 | AT | A | 0.486 | NM_001144013 | RGPD3 | frameshift_deleti  on | exon8 | c.1025delA | p.N342fs | . | . |
| XS1 | 2 | 108488322 | TCAA | T | 0.16 | NM_182588 | RGPD4 | nonframeshift_de  letion | exon20 | c.3863_3865del | p.1288_1289de  l | . | . |
| XS1 | 2 | 109092298 | A | AG | 0.538 | NM_181453 | GCC2 | frameshift_inserti  on | exon9 | c.3053dupG | p.S1018fs | . | . |
| XS1 | 2 | 109380096 | AC | A | 0.333 | NM_006267 | RANBP2 | frameshift_deleti  on | exon20 | c.3102delC | p.N1034fs | . | . |
| XS1 | 2 | 109384298 | AT | A | 0.857 | NM_006267 | RANBP2 | frameshift_deleti  on | exon20 | c.7304delT | p.I2435fs | . | . |
| XS1 | 2 | 109389025 | GA | G | 0.435 | NM_006267 | RANBP2 | frameshift_deleti  on | exon22 | c.8102delA | p.E2701fs | . | . |
| XS1 | 2 | 109513417 | T | TA | 0.278 | NM_022336 | EDAR | frameshift_inserti  on | exon12 | c.1292dupT | p.I431fs | . | . |
| XS1 | 2 | 111419275 | AT | A | 0.35 | NM_001278616 | BUB1 | frameshift_deleti  on | exon9 | c.1040delA | p.N347fs | . | . |
| XS1 | 2 | 112614252 | AT | A | 0.75 | NM_022662 | ANAPC1 | stopgain | exon13 | c.1453delA | p.I485X | . | . |
| XS1 | 2 | 114391739 | GC | G | 0.287 | NM_007082 | RABL2A | frameshift_deleti on | exon4 | c.148delC | p.Q50fs | . | . |
| XS1 | 2 | 116447485 | A | AG | 0.278 | NM_001004360 | DPP10 | frameshift_inserti  on | exon7 | c.544dupG | p.Q181fs | . | . |
| XS1 | 2 | 118578740 | CT | C | 0.276 | NM_006773 | DDX18 | frameshift_deleti  on | exon4 | c.519delT | p.A173fs | . | . |
| XS1 | 2 | 118582233 | TG | T | 0.212 | NM_006773 | DDX18 | frameshift_deleti  on | exon8 | c.1156delG | p.V386fs | . | . |
| XS1 | 2 | 118732840 | GT | G | 0.558 | NM_019044 | CCDC93 | frameshift_deleti  on | exon9 | c.673delA | p.T225fs | . | . |
| XS1 | 2 | 120194651 | A | AGTGT  GC | 1 | NM_183240 | TMEM37 | nonframeshift_in  sertion | exon2 | c.208_209insGT  GTGC | p.T70delinsSVP | . | . |
| XS1 | 2 | 120303785 | G | GA | 0.167 | NM_001271049 | PCDP1 | frameshift_inserti  on | exon2 | c.79dupA | p.L26fs | . | . |
| XS1 | 2 | 125281952 | TG | T | 0.708 | NM_130773 | CNTNAP5 | frameshift_deleti  on | exon9 | c.1398delG | p.L466fs | . | . |
| XS1 | 2 | 131688786 | GA | G | 0.43 | NM_032995 | ARHGEF4 | frameshift_deleti  on | exon2 | c.257delA | p.E86fs | . | . |
| XS1 | 2 | 135180401 | GT | G | 0.276 | NM_002410 | MGAT5 | frameshift_deleti  on | exon13 | c.1706delT | p.V569fs | . | . |
| XS1 | 2 | 135710873 | TC | T | 0.375 | NM_001241 | CCNT2 | frameshift_deleti  on | exon9 | c.849delC | p.V283fs | . | . |
| XS1 | 2 | 141242980 | CA | C | 0.509 | NM_018557 | LRP1B | frameshift_deleti  on | exon59 | c.9356delT | p.L3119fs | . | . |
| XS1 | 2 | 141283472 | G | GT | 0.667 | NM_018557 | LRP1B | frameshift_inserti  on | exon49 | c.7966dupA | p.T2656fs | . | . |
| XS1 | 2 | 145156869 | CA | C | 0.5 | NM_001171653 | ZEB2 | frameshift_deleti  on | exon7 | c.1812delT | p.F604fs | . | . |
| XS1 | 2 | 149246986 | AC | A | 0.194 | NM_018328 | MBD5 | frameshift_deleti  on | exon12 | c.3087delC | p.N1029fs | . | . |
| XS1 | 2 | 152320575 | AG | A | 0.412 | NM_001177665 | RIF1 | frameshift_deleti  on | exon29 | c.4542delG | p.E1514fs | . | . |
| XS1 | 2 | 152322127 | TG | T | 0.364 | NM_001177665 | RIF1 | frameshift_deleti  on | exon29 | c.6094delG | p.E2032fs | . | . |
| XS1 | 2 | 152522787 | CT | C | 1 | NM_001164507 | NEB | frameshift_deleti  on | exon41 | c.4847delA | p.K1616fs | . | . |
| XS1 | 2 | 153476066 | G | GCCA | 1 | NM_052905 | FMNL2 | nonframeshift_in  sertion | exon15 | c.1671_1672ins  CCA | p.P557delinsPP | . | . |
| XS1 | 2 | 158157201 | GT | G | 0.75 | NM_014568 | GALNT5 | frameshift_deleti  on | exon7 | c.2130delT | p.G710fs | . | . |
| XS1 | 2 | 162730483 | GA | G | 0.308 | NM_001178015 | SLC4A10 | frameshift_deleti  on | exon8 | c.917delA | p.E306fs | . | . |
| XS1 | 2 | 163291942 | CA | C | 0.556 | NM_173162 | KCNH7 | frameshift_deleti  on | exon7 | c.1698delT | p.I566fs | . | . |
| XS1 | 2 | 165551342 | TA | T | 0.714 | NM_001278461 | COBLL1 | frameshift_deleti  on | exon11 | c.2559delT | p.F853fs | . | . |
| XS1 | 2 | 165557121 | AT | A | 0.867 | NM_001278461 | COBLL1 | frameshift_deleti  on | exon9 | c.1370delA | p.N457fs | . | . |
| XS1 | 2 | 165947104 | TA | T | 0.35 | NM_001081676 | SCN3A | frameshift_deleti  on | exon28 | c.5411delT | p.L1804fs | . | . |
| XS1 | 2 | 165969510 | GC | G | 0.216 | NM_001081676 | SCN3A | frameshift_deleti  on | exon21 | c.3580delG | p.A1194fs | . | . |
| XS1 | 2 | 166900384 | CG | C | 0.182 | NM_001165963 | SCN1A | frameshift_deleti  on | exon11 | c.1837delC | p.R613fs | . | . |
| XS1 | 2 | 167099088 | GT | G | 0.846 | NM_002977 | SCN9A | frameshift_deleti  on | exon19 | c.3517delA | p.T1173fs | . | . |
| XS1 | 2 | 167133580 | TA | T | 0.704 | NM_002977 | SCN9A | frameshift_deleti  on | exon16 | c.2753delT | p.I918fs | . | . |
| XS1 | 2 | 170028559 | AT | A | 0.75 | NM_004525 | LRP2 | frameshift_deleti  on | exon58 | c.11228delA | p.N3743fs | . | . |
| XS1 | 2 | 170028601 | AT | A | 0.5 | NM_004525 | LRP2 | frameshift_deleti  on | exon58 | c.11186delA | p.N3729fs | . | . |
| XS1 | 2 | 171258027 | AG | A | 0.209 | NM_001083615 | MYO3B | frameshift_deleti  on | exon18 | c.1956delG | p.Q652fs | . | . |
| XS1 | 2 | 173429721 | GA | G | 0.579 | NM_002610 | PDK1 | frameshift_deleti  on | exon5 | c.612delA | p.G204fs | . | . |
| XS1 | 2 | 173825878 | GA | G | 0.353 | NM_001282900 | RAPGEF4 | frameshift_deleti  on | exon4 | c.108delA | p.G36fs | . | . |
| XS1 | 2 | 175436644 | TG | T | 0.263 | NM_001077269 | WIPF1 | frameshift_deleti  on | exon5 | c.888delC | p.S296fs | . | . |
| XS1 | 2 | 175711858 | AT | A | 0.444 | NM_001206602 | CHN1 | frameshift_deleti  on | exon1 | c.1delA | p.M1fs | . | . |
| XS1 | 2 | 176964703 | CGCCCC  CGCGCA | C | 1 | NM_021193 | HOXD12 | nonframeshift_de  letion | exon1 | c.175_189del | p.59_63del | . | . |
| XS1 | 2 | 179397343 | TG | T | 0.259 | NM_003319 | TTN | frameshift_deleti  on | exon186 | c.76803delC | p.P25601fs | . | . |

| XS1 | 2 | 179402594 | AT | A | 0.8 | NM_003319 | TTN | frameshift_deleti  on | exon183 | c.72144delA | p.K24048fs | . | . |
| --- | --- | --- | --- | --- | --- | --- | --- | --- | --- | --- | --- | --- | --- |
| XS1 | 2 | 179432061 | TA | T | 0.454 | NM_003319 | TTN | stopgain | exon154 | c.51602delT | p.L17201X | . | . |
| XS1 | 2 | 179438551 | GT | G | 0.653 | NM_003319 | TTN | frameshift_deleti on | exon154 | c.45112delA | p.T15038fs | . | . |
| XS1 | 2 | 179458148 | GC | G | 1 | NM_003319 | TTN | frameshift_deleti  on | exon127 | c.31591delG | p.A10531fs | . | . |
| XS1 | 2 | 179477065 | AT | A | 0.8 | NM_003319 | TTN | frameshift_deleti  on | exon94 | c.22991delA | p.N7664fs | . | . |
| XS1 | 2 | 179483442 | GT | G | 0.75 | NM_003319 | TTN | frameshift_deleti  on | exon79 | c.19639delA | p.T6547fs | . | . |
| XS1 | 2 | 179598013 | GA | G | 0.339 | NM_133378 | TTN | frameshift_deleti  on | exon51 | c.12274delT | p.S4092fs | . | . |
| XS1 | 2 | 179616709 | GA | G | 0.947 | NM_133379 | TTN | frameshift_deleti  on | exon46 | c.10417delT | p.S3473fs | . | . |
| XS1 | 2 | 179702144 | GT | G | 0.182 | NM_173648 | CCDC141 | frameshift_deleti  on | exon23 | c.3801delA | p.P1267fs | . | . |
| XS1 | 2 | 182339912 | AT | A | 0.444 | NM_000885 | ITGA4 | frameshift_deleti  on | exon4 | c.454delT | p.F152fs | . | . |
| XS1 | 2 | 182438491 | GT | G | 1 | NM_001030311 | CERKL | frameshift_deleti  on | exon3 | c.601delA | p.T201fs | . | . |
| XS1 | 2 | 182760804 | AG | A | 1 | NM_001130445 | SSFA2 | frameshift_deleti  on | exon3 | c.306delG | p.K102fs | . | . |
| XS1 | 2 | 185802211 | C | CACA | 1 | NM_194250 | ZNF804A | nonframeshift_in  sertion | exon4 | c.2088_2089ins  ACA | p.N696delinsN  T | . | . |
| XS1 | 2 | 185802213 | C | CAAG | 1 | NM_194250 | ZNF804A | nonframeshift_in  sertion | exon4 | c.2090_2091ins  AAG | p.T697delinsTR | . | . |
| XS1 | 2 | 185802215 | A | ACAC | 1 | NM_194250 | ZNF804A | nonframeshift_in  sertion | exon4 | c.2092_2093ins  CAC | p.I698delinsTL | . | . |
| XS1 | 2 | 186660111 | AT | A | 0.667 | NM_173651 | FSIP2 | frameshift_deleti  on | exon16 | c.8516delT | p.I2839fs | . | . |
| XS1 | 2 | 186662094 | CA | C | 1 | NM_173651 | FSIP2 | frameshift_deleti  on | exon16 | c.10499delA | p.Q3500fs | . | . |
| XS1 | 2 | 186666325 | G | GA | 0.295 | NM_173651 | FSIP2 | frameshift_inserti  on | exon17 | c.12560dupA | p.E4187fs | . | . |
| XS1 | 2 | 186689172 | A | AT | 0.209 | NM_173651 | FSIP2 | frameshift_inserti  on | exon20 | c.20706dupT | p.D6902fs | . | . |
| XS1 | 2 | 187559047 | G | GCAA | 0.976 | NM_177454 | FAM171B | nonframeshift_in  sertion | exon1 | c.147_148insCA  A | p.Q49delinsQQ | . | . |
| XS1 | 2 | 190334947 | GT | G | 0.472 | NM_032168 | WDR75 | frameshift_deleti  on | exon17 | c.1966delT | p.F656fs | . | . |
| XS1 | 2 | 190338944 | AT | A | 0.324 | NM_032168 | WDR75 | frameshift_deleti  on | exon19 | c.2083delT | p.F695fs | . | . |
| XS1 | 2 | 190338961 | GA | G | 0.308 | NM_032168 | WDR75 | frameshift_deleti  on | exon19 | c.2100delA | p.G700fs | . | . |
| XS1 | 2 | 190338991 | T | TA | 0.265 | NM_032168 | WDR75 | frameshift_inserti  on | exon19 | c.2130dupA | p.L710fs | . | . |
| XS1 | 2 | 190593057 | TA | T | 0.455 | NM_144708 | ANKAR | frameshift_deleti  on | exon14 | c.2943delA | p.L981fs | . | . |
| XS1 | 2 | 196674534 | AG | A | 0.8 | NM_018897 | DNAH7 | frameshift_deleti  on | exon52 | c.9822delC | p.L3274fs | . | . |
| XS1 | 2 | 198498679 | TG | T | 0.192 | NM_144629 | RFTN2 | frameshift_deleti  on | exon4 | c.480delC | p.F160fs | . | . |
| XS1 | 2 | 201342545 | CA | C | 0.333 | NM_001100424 | SPATS2L | frameshift_deleti  on | exon12 | c.1262delA | p.Q421fs | . | . |
| XS1 | 2 | 201399881 | AG | A | 0.526 | NM_001160033 | SGOL2 | frameshift_deleti  on | exon3 | c.297delG | p.K99fs | . | . |
| XS1 | 2 | 201719358 | GT | G | 0.778 | NM_001162407 | CLK1 | frameshift_deleti  on | exon11 | c.1326delA | p.K442fs | . | . |
| XS1 | 2 | 201800553 | C | CT | 0.429 | NM_006190 | ORC2 | frameshift_inserti  on | exon9 | c.576dupA | p.G193fs | . | . |
| XS1 | 2 | 202068465 | CA | C | 1 | NM_001206524 | CASP10 | frameshift_deleti  on | exon6 | c.698delA | p.Q233fs | . | . |
| XS1 | 2 | 207041451 | TA | T | 0.857 | NM_001098199 | GPR1 | frameshift_deleti  on | exon3 | c.520delT | p.Y174fs | . | . |
| XS1 | 2 | 209190105 | TA | T | 0.857 | NM_015040 | PIKFYVE | frameshift_deleti  on | exon20 | c.2571delA | p.I857fs | . | . |
| XS1 | 2 | 210804351 | GT | G | 1 | NM_032504 | UNC80 | frameshift_deleti  on | exon42 | c.6424delT | p.F2142fs | . | . |
| XS1 | 2 | 214142603 | C | CA | 0.149 | NM_001242575 | AC079610.2 | frameshift_inserti  on | exon3 | c.272dupT | p.L91fs | . | . |
| XS1 | 2 | 216960860 | C | CA | 0.267 | NM_001142311 | TMEM169 | frameshift_inserti  on | exon2 | c.175dupA | p.D58fs | . | . |
| XS1 | 2 | 217559427 | GA | G | 0.273 | NM_000599 | IGFBP5 | frameshift_deleti  on | exon1 | c.71delT | p.F24fs | . | . |
| XS1 | 2 | 219301230 | AC | A | 0.6 | NM_007127 | VIL1 | frameshift_deleti  on | exon16 | c.1853delC | p.T618fs | . | . |
| XS1 | 2 | 219895544 | TG | T | 0.674 | NM_001278296 | CCDC108 | frameshift_deleti  on | exon8 | c.832delC | p.Q278fs | . | . |
| XS1 | 2 | 220029938 | CCA | C | 0.28 | NM_001144889 | SLC23A3 | frameshift_deleti  on | exon8 | c.1118_1119del | p.M373fs | . | . |
| XS1 | 2 | 220402416 | TG | T | 0.308 | NM_182847 | ASIC4 | frameshift_deleti  on | exon8 | c.1789delG | p.G597fs | . | . |
| XS1 | 2 | 220404814 | GC | G | 0.176 | NM_001195731 | CHPF | frameshift_deleti  on | exon4 | c.1132delG | p.A378fs | . | . |
| XS1 | 2 | 224863047 | AT | A | 0.333 | NM_001136528 | SERPINE2 | frameshift_deleti  on | exon3 | c.271delA | p.I91fs | . | . |
| XS1 | 2 | 225698921 | A | AT | 0.909 | NM_014689 | DOCK10 | frameshift_inserti  on | exon26 | c.2909dupA | p.N970fs | . | . |
| XS1 | 2 | 227663015 | TC | T | 0.491 | NM_005544 | IRS1 | frameshift_deleti  on | exon1 | c.439delG | p.E147fs | . | . |
| XS1 | 2 | 227729505 | AG | A | 0.533 | NM_032276 | RHBDD1 | frameshift_deleti  on | exon2 | c.97delG | p.A33fs | . | . |
| XS1 | 2 | 229890414 | GT | G | 0.667 | NM_001100818 | PID1 | frameshift_deleti  on | exon3 | c.587delA | p.D196fs | . | . |
| XS1 | 2 | 231155252 | AT | A | 0.364 | NM_001278453 | SP140 | frameshift_deleti  on | exon16 | c.1457delT | p.I486fs | . | . |
| XS1 | 2 | 231331051 | G | GA | 0.333 | NM_001206703 | SP100 | frameshift_inserti  on | exon11 | c.1103dupA | p.E368fs | . | . |
| XS1 | 2 | 231371112 | G | GA | 0.441 | NM_001080391 | SP100 | frameshift_inserti  on | exon22 | c.1966dupA | p.W655fs | . | . |
| XS1 | 2 | 231682604 | GT | G | 1 | NM_001130849 | CAB39 | frameshift_deleti  on | exon8 | c.830delT | p.V277fs | . | . |
| XS1 | 2 | 231861032 | TCAGCA  GCCTAG CCCTGA ATCCAC | T | 0.333 | NM_139073 | SPATA3 | nonframeshift_de letion | exon1 | c.85_111del | p.29_37del | . | . |
| XS1 | 2 | 232325414 | TTCA | T | 0.571 | NM_005381 | NCL | nonframeshift_de  letion | exon4 | c.774_776del | p.258_259del | . | . |
| XS1 | 2 | 233243528 | ATGC | A | 0.25 | NM_001632 | ALPP | nonframeshift_de  letion | exon1 | c.17_19del | p.6_7del | . | . |
| XS1 | 2 | 233785143 | GCTC | G | 1 | NM_001114090 | NGEF | nonframeshift_de  letion | exon3 | c.400_402del | p.134_134del | . | . |

| XS1 | 2 | 234418766 | CA | C | 0.677 | NM_018218 | USP40 | frameshift_deleti  on | exon20 | c.2574delT | p.F858fs | . | . |
| --- | --- | --- | --- | --- | --- | --- | --- | --- | --- | --- | --- | --- | --- |
| XS1 | 2 | 234429739 | T | TTTC | 0.9 | NM_018218 | USP40 | nonframeshift_in  sertion | exon16 | c.2255_2256ins  GAA | p.K752delinsKK | . | . |
| XS1 | 2 | 234429740 | T | TTCA | 0.857 | NM_018218 | USP40 | nonframeshift_in  sertion | exon16 | c.2254_2255ins  TGA | p.K752delinsM  K | . | . |
| XS1 | 2 | 234622047 | TG | T | 0.179 | NM_019078 | UGT1A5 | frameshift_deleti  on | exon1 | c.411delG | p.L137fs | . | . |
| XS1 | 2 | 234728594 | AC | A | 0.444 | NM_001287395 | MROH2A | frameshift_deleti  on | exon31 | c.3414delC | p.D1138fs | . | . |
| XS1 | 2 | 234738352 | AG | A | 1 | NM_001287395 | MROH2A | frameshift_deleti  on | exon38 | c.4576delG | p.G1526fs | . | . |
| XS1 | 2 | 235949993 | CT | C | 0.384 | NM_014521 | SH3BP4 | frameshift_deleti  on | exon4 | c.581delT | p.L194fs | . | . |
| XS1 | 2 | 235950715 | TG | T | 0.488 | NM_014521 | SH3BP4 | frameshift_deleti  on | exon4 | c.1303delG | p.G435fs | . | . |
| XS1 | 2 | 235951246 | AC | A | 0.75 | NM_014521 | SH3BP4 | frameshift_deleti  on | exon4 | c.1834delC | p.P612fs | . | . |
| XS1 | 2 | 236761414 | C | CCAGG | 1 | NM_001244888 | AGAP1 | frameshift_inserti  on | exon10 | c.1135_1136ins  CAGG | p.P379fs | . | . |
| XS1 | 2 | 237272512 | CT | C | 0.267 | NM_001270584 | IQCA1 | frameshift_deleti  on | exon14 | c.1656delA | p.G552fs | . | . |
| XS1 | 2 | 238483728 | TA | T | 0.269 | NM_022449 | RAB17 | frameshift_deleti  on | exon6 | c.572delT | p.L191fs | . | . |
| XS1 | 2 | 241528844 | CTG | C | 0.549 | NM_023083 | CAPN10 | frameshift_deleti  on | exon2 | c.227_228del | p.L76fs | . | . |
| XS1 | 2 | 242008354 | AT | A | 0.168 | NM_001080437 | SNED1 | frameshift_deleti  on | exon23 | c.3224delT | p.I1075fs | . | . |
| XS1 | 2 | 242051716 | AT | A | 0.857 | NM_001252119 | PASK | frameshift_deleti  on | exon15 | c.3492delA | p.K1164fs | . | . |
| XS1 | 2 | 242077378 | TG | T | 0.259 | NM_001252119 | PASK | frameshift_deleti  on | exon6 | c.865delC | p.H289fs | . | . |
| XS1 | 3 | 4354847 | TC | T | 0.312 | NM_001243723 | SETMAR | frameshift_deleti  on | exon2 | c.423delC | p.F141fs | . | . |
| XS1 | 3 | 4725370 | TG | T | 0.263 | NM_001168272 | ITPR1 | frameshift_deleti  on | exon27 | c.3342delG | p.L1114fs | . | . |
| XS1 | 3 | 8677029 | TC | T | 0.991 | NM_001256748 | SSUH2 | frameshift_deleti  on | exon3 | c.165delG | p.G55fs | . | . |
| XS1 | 3 | 9512591 | AG | A | 0.364 | NM_001080517 | SETD5 | frameshift_deleti  on | exon19 | c.3174delG | p.Q1058fs | . | . |
| XS1 | 3 | 9788853 | GT | G | 0.621 | NM_001003694 | BRPF1 | frameshift_deleti  on | exon14 | c.3484delT | p.W1162fs | . | . |
| XS1 | 3 | 9798190 | GC | G | 0.964 | NM_002542 | OGG1 | frameshift_deleti  on | exon5 | c.784delC | p.P262fs | . | . |
| XS1 | 3 | 9798896 | TC | T | 0.4 | NM_016820 | OGG1 | frameshift_deleti  on | exon7 | c.1118delC | p.S373fs | . | . |
| XS1 | 3 | 9854692 | C | CG | 0.909 | NM_001025930 | TTLL3 | frameshift_inserti  on | exon3 | c.543_544insG | p.G181fs | . | . |
| XS1 | 3 | 9854693 | T | TA | 0.889 | NM_001025930 | TTLL3 | stopgain | exon3 | c.544_545insA | p.W182_V183d  elinsX | . | . |
| XS1 | 3 | 10400474 | G | GA | 0.46 | NM_001683 | ATP2B2 | frameshift_inserti  on | exon11 | c.1901dupT | p.F634fs | . | . |
| XS1 | 3 | 12983167 | G | GT | 0.333 | NM_001134382 | IQSEC1 | frameshift_inserti  on | exon2 | c.221_222insA | p.T74fs | . | . |
| XS1 | 3 | 14219965 | CCCT | C | 0.474 | NM_001145769 | XPC | nonframeshift_de  letion | exon1 | c.101_103del | p.34_35del | . | . |
| XS1 | 3 | 14706610 | TA | T | 0.333 | NM_016474 | CCDC174 | frameshift_deleti  on | exon6 | c.562delA | p.K188fs | . | . |
| XS1 | 3 | 14861978 | AG | A | 0.412 | NM_152536 | FGD5 | frameshift_deleti  on | exon1 | c.1401delG | p.E467fs | . | . |
| XS1 | 3 | 15269455 | AGT | A | 0.2 | NM_014296 | CAPN7 | frameshift_deleti  on | exon7 | c.836_837del | p.S279fs | . | . |
| XS1 | 3 | 15731555 | A | AT | 0.361 | NM_001195099 | ANKRD28 | frameshift_inserti  on | exon16 | c.1381dupA | p.I461fs | . | . |
| XS1 | 3 | 15737722 | TG | T | 0.8 | NM_001195099 | ANKRD28 | frameshift_deleti  on | exon13 | c.1025delC | p.A342fs | . | . |
| XS1 | 3 | 16268915 | GA | G | 0.295 | NM_054110 | GALNT15 | frameshift_deleti  on | exon10 | c.1829delA | p.E610fs | . | . |
| XS1 | 3 | 16305717 | TC | T | 0.444 | NM_206831 | DPH3 | frameshift_deleti  on | exon2 | c.127delG | p.E43fs | . | . |
| XS1 | 3 | 16411764 | GA | G | 0.585 | NM_015150 | RFTN1 | frameshift_deleti  on | exon6 | c.848delT | p.F283fs | . | . |
| XS1 | 3 | 17279829 | CT | C | 0.238 | NM_014744 | TBC1D5 | frameshift_deleti  on | exon17 | c.1413delA | p.P471fs | . | . |
| XS1 | 3 | 32570035 | CT | C | 1 | NM_016141 | DYNC1LI1 | frameshift_deleti  on | exon12 | c.1364delA | p.K455fs | . | . |
| XS1 | 3 | 33894180 | AG | A | 0.432 | NM_001162429 | PDCD6IP | frameshift_deleti  on | exon13 | c.1858delG | p.V620fs | . | . |
| XS1 | 3 | 38629047 | GA | G | 0.444 | NM_000335 | SCN5A | frameshift_deleti  on | exon15 | c.2279delT | p.F760fs | . | . |
| XS1 | 3 | 38648240 | TG | T | 0.308 | NM_000335 | SCN5A | frameshift_deleti  on | exon9 | c.1059delC | p.T353fs | . | . |
| XS1 | 3 | 38770216 | AT | A | 0.591 | NM_006514 | SCN10A | frameshift_deleti  on | exon15 | c.2456delA | p.N819fs | . | . |
| XS1 | 3 | 40275409 | C | CA | 0.172 | NM_001284426 | MYRIP | frameshift_inserti  on | exon10 | c.1405dupA | p.A468fs | . | . |
| XS1 | 3 | 40503520 | A | ACTGC  TG | 0.943 | NM_001034996 | RPL14 | nonframeshift_in  sertion | exon6 | c.445_446insCT  GCTG | p.T149delinsTA  A | . | . |
| XS1 | 3 | 40503520 | ACTGCT  G | A | 0.943 | NM_001034996 | RPL14 | nonframeshift_de  letion | exon6 | c.446_451del | p.149_151del | . | . |
| XS1 | 3 | 40503540 | T | TGCTG  CA | 0.774 | NM_001034996 | RPL14 | nonframeshift_in  sertion | exon6 | c.465_466insG  CTGCA | p.A155delinsA  AA | . | . |
| XS1 | 3 | 40524164 | TG | T | 0.588 | NM_001145083 | ZNF619 | frameshift_deleti  on | exon3 | c.133delG | p.G45fs | . | . |
| XS1 | 3 | 42251577 | C | CGGA | 0.75 | NM_001265609 | TRAK1 | nonframeshift_in  sertion | exon13 | c.1841_1842ins  GGA | p.T614delinsTE | . | . |
| XS1 | 3 | 42568961 | TC | T | 0.857 | NM_001251884 | VIPR1 | frameshift_deleti  on | exon4 | c.333delC | p.V111fs | . | . |
| XS1 | 3 | 42680098 | G | GA | 0.862 | NM_005385 | NKTR | frameshift_inserti  on | exon13 | c.2903dupA | p.E968fs | . | . |
| XS1 | 3 | 42754691 | TG | T | 0.285 | NM_144719 | CCDC13 | frameshift_deleti  on | exon14 | c.1835delC | p.A612fs | . | . |
| XS1 | 3 | 45518017 | AT | A | 0.267 | NM_015340 | LARS2 | frameshift_deleti  on | exon10 | c.917delT | p.I306fs | . | . |
| XS1 | 3 | 45518059 | CT | C | 0.633 | NM_015340 | LARS2 | frameshift_deleti  on | exon10 | c.959delT | p.L320fs | . | . |
| XS1 | 3 | 46399464 | TA | T | 0.338 | NM_001123041 | CCR2 | frameshift_deleti  on | exon2 | c.447delA | p.L149fs | . | . |
| XS1 | 3 | 46963757 | CT | C | 0.428 | NM_001277074 | CCDC12 | frameshift_deleti  on | exon6 | c.386delA | p.K129fs | . | . |
| XS1 | 3 | 47098613 | G | GT | 0.173 | NM_014159 | SETD2 | frameshift_inserti  on | exon15 | c.6660_6661ins  A | p.P2221fs | . | . |
| XS1 | 3 | 47461014 | G | GC | 0.5 | NM_012235 | SCAP | frameshift_inserti  on | exon13 | c.1743_1744ins  G | p.P582fs | . | . |

| XS1 | 3 | 48207210 | CA | C | 0.337 | NM_201567 | CDC25A | stopgain | exon11 | c.986delT | p.L329X | . | . |
| --- | --- | --- | --- | --- | --- | --- | --- | --- | --- | --- | --- | --- | --- |
| XS1 | 3 | 48309528 | TC | T | 0.429 | NM_016089 | ZNF589 | frameshift_deleti on | exon4 | c.348delC | p.L116fs | . | . |
| XS1 | 3 | 49042496 | TGG | T | 0.205 | NM_177938 | P4HTM | frameshift_deleti  on | exon6 | c.1091_1092del | p.W364fs | . | . |
| XS1 | 3 | 50155887 | TGA | T | 0.424 | NM_005778 | RBM5 | frameshift_deleti  on | exon25 | c.2447_2448del | p.*816fs | . | . |
| XS1 | 3 | 50327661 | CG | C | 0.556 | NM_006764 | IFRD2 | frameshift_deleti  on | exon4 | c.519delC | p.P173fs | . | . |
| XS1 | 3 | 50332219 | AG | A | 0.424 | NM_001200029 | HYAL3 | frameshift_deleti  on | exon2 | c.814delC | p.L272fs | . | . |
| XS1 | 3 | 51812768 | TG | T | 0.241 | NM_001143833 | IQCF6 | frameshift_deleti  on | exon2 | c.194delC | p.A65fs | . | . |
| XS1 | 3 | 52264013 | CG | C | 0.171 | NM_007284 | TWF2 | frameshift_deleti  on | exon7 | c.682delC | p.R228fs | . | . |
| XS1 | 3 | 52356625 | AC | A | 0.371 | NM_015512 | DNAH1 | frameshift_deleti  on | exon2 | c.168delC | p.D56fs | . | . |
| XS1 | 3 | 52429446 | C | CA | 0.45 | NM_015512 | DNAH1 | frameshift_inserti  on | exon69 | c.11092dupA | p.S3697fs | . | . |
| XS1 | 3 | 52551934 | GC | G | 0.939 | NM_015136 | STAB1 | frameshift_deleti  on | exon45 | c.4677delC | p.S1559fs | . | . |
| XS1 | 3 | 52836402 | AC | A | 0.265 | NM_002217 | ITIH3 | frameshift_deleti  on | exon12 | c.1437delC | p.Y479fs | . | . |
| XS1 | 3 | 53906525 | TG | T | 0.278 | NM_022899 | ACTR8 | frameshift_deleti  on | exon10 | c.1187delC | p.A396fs | . | . |
| XS1 | 3 | 56591278 | T | TGGGG  TAAGC | 0.973 | NM_001141947 | CCDC66 | stopgain | exon1 | c.8_9insGGGGT  AAGCA | p.L3_G4delinsL  GX | . | . |
| XS1 | 3 | 56591278 | T | TGGGT  AAGCA | 0.973 | NM_001141947 | CCDC66 | nonframeshift_in  sertion | exon1 | c.8_9insGGGTA  AGCA | p.L3delinsLGK  Q | . | . |
| XS1 | 3 | 56650054 | T | TCTC | 1 | NM_001012506 | CCDC66 | nonframeshift_in  sertion | exon13 | c.1714_1715ins  CTC | p.S572delinsSP | . | . |
| XS1 | 3 | 57528531 | T | TG | 0.532 | NM_178504 | DNAH12 | frameshift_inserti  on | exon2 | c.66dupC | p.I23fs | . | . |
| XS1 | 3 | 59999803 | TG | T | 0.417 | NM_001166243 | FHIT | frameshift_deleti  on | exon6 | c.178delC | p.Q60fs | . | . |
| XS1 | 3 | 63981827 | AG | A | 0.417 | NM_001128149 | ATXN7 | frameshift_deleti  on | exon8 | c.1895delG | p.R632fs | . | . |
| XS1 | 3 | 65416400 | GC | G | 0.244 | NM_001033057 | MAGI1 | frameshift_deleti  on | exon11 | c.1519delG | p.A507fs | . | . |
| XS1 | 3 | 67054582 | TC | T | 0.571 | NM_032505 | KBTBD8 | frameshift_deleti  on | exon3 | c.1192delC | p.R398fs | . | . |
| XS1 | 3 | 68788233 | GT | G | 0.4 | NM_001005527 | FAM19A4 | frameshift_deleti  on | exon5 | c.403delA | p.T135fs | . | . |
| XS1 | 3 | 69168287 | GT | G | 0.562 | NM_198271 | LMOD3 | frameshift_deleti  on | exon2 | c.1218delA | p.K406fs | . | . |
| XS1 | 3 | 69242991 | GA | G | 1 | NM_015123 | FRMD4B | frameshift_deleti  on | exon17 | c.1521delT | p.F507fs | . | . |
| XS1 | 3 | 73096368 | AT | A | 0.5 | NM_174907 | PPP4R2 | frameshift_deleti  on | exon3 | c.149delT | p.I50fs | . | . |
| XS1 | 3 | 74315671 | C | CA | 0.8 | NM_020872 | CNTN3 | frameshift_inserti  on | exon21 | c.2946dupT | p.G983fs | . | . |
| XS1 | 3 | 77645884 | GA | G | 0.699 | NM_002942 | ROBO2 | frameshift_deleti  on | exon19 | c.2838delA | p.G946fs | . | . |
| XS1 | 3 | 97631054 | AT | A | 0.633 | NM_153605 | CRYBG3 | frameshift_deleti  on | exon16 | c.8049delT | p.D2683fs | . | . |
| XS1 | 3 | 97669728 | AT | A | 0.273 | NM_001042533 | MINA | frameshift_deleti  on | exon6 | c.789delA | p.S263fs | . | . |
| XS1 | 3 | 98530315 | TG | T | 0.444 | NM_080927 | DCBLD2 | frameshift_deleti  on | exon11 | c.1446delC | p.A482fs | . | . |
| XS1 | 3 | 100275609 | GC | G | 0.222 | NM_018004 | TMEM45A | frameshift_deleti  on | exon3 | c.197delC | p.A66fs | . | . |
| XS1 | 3 | 100489609 | CT | C | 0.889 | NM_015429 | ABI3BP | frameshift_deleti  on | exon29 | c.2585delA | p.K862fs | . | . |
| XS1 | 3 | 107097011 | GC | G | 0.392 | NM_032600 | CCDC54 | frameshift_deleti  on | exon1 | c.578delC | p.A193fs | . | . |
| XS1 | 3 | 108754291 | CA | C | 0.6 | NM_014429 | MORC1 | frameshift_deleti  on | exon15 | c.1354delT | p.C452fs | . | . |
| XS1 | 3 | 111312540 | C | CG | 0.25 | NM_024508 | ZBED2 | frameshift_inserti  on | exon2 | c.508dupC | p.R170fs | . | . |
| XS1 | 3 | 111312541 | G | GA | 0.333 | NM_024508 | ZBED2 | frameshift_inserti  on | exon2 | c.507_508insT | p.R170fs | . | . |
| XS1 | 3 | 111394214 | CT | C | 0.353 | NM_001185106 | PLCXD2 | frameshift_deleti  on | exon1 | c.123delT | p.P41fs | . | . |
| XS1 | 3 | 111988823 | CA | C | 1 | NM_183061 | SLC9C1 | frameshift_deleti  on | exon7 | c.714delT | p.F238fs | . | . |
| XS1 | 3 | 113376110 | TTGC | T | 1 | NM_001009899 | KIAA2018 | nonframeshift_de  letion | exon7 | c.4416_4418del | p.1472_1473de  l | . | . |
| XS1 | 3 | 113627868 | GA | G | 0.429 | NM_001172105 | GRAMD1C | frameshift_deleti  on | exon4 | c.239delA | p.E80fs | . | . |
| XS1 | 3 | 113697039 | CTG | C | 0.146 | NM_020817 | KIAA1407 | frameshift_deleti  on | exon16 | c.2598_2599del | p.H866fs | . | . |
| XS1 | 3 | 120352150 | TC | T | 0.578 | NM_000187 | HGD | frameshift_deleti  on | exon13 | c.1031delG | p.G344fs | . | . |
| XS1 | 3 | 120408687 | TC | T | 0.333 | NM_173825 | RABL3 | frameshift_deleti  on | exon8 | c.693delG | p.K231fs | . | . |
| XS1 | 3 | 121255988 | CA | C | 0.682 | NM_199420 | POLQ | frameshift_deleti  on | exon5 | c.698delT | p.L233fs | . | . |
| XS1 | 3 | 121351315 | G | GGGCT  CAGGC | 0.994 | NM_005335 | HCLS1 | nonframeshift_in  sertion | exon12 | c.1103_1104ins  TGAGCCTGAG | p.P368delinsPE  PEP | . | . |
| XS1 | 3 | 121351318 | C | CTCAG  GCTCA | 0.897 | NM_005335 | HCLS1 | nonframeshift_in  sertion | exon12 | c.1100_1101ins  ACCTGAGCCT | p.E367delinsEP  EPE | . | . |
| XS1 | 3 | 121414289 | AT | A | 0.811 | NM_001256488 | GOLGB1 | frameshift_deleti  on | exon12 | c.4840delA | p.I1614fs | . | . |
| XS1 | 3 | 121980542 | GC | G | 0.273 | NM_000388 | CASR | frameshift_deleti  on | exon4 | c.661delC | p.P221fs | . | . |
| XS1 | 3 | 122287749 | TC | T | 0.846 | NM_138287 | DTX3L | frameshift_deleti  on | exon3 | c.814delC | p.P272fs | . | . |
| XS1 | 3 | 122418900 | G | GT | 0.257 | NM_017554 | PARP14 | frameshift_inserti  on | exon6 | c.1500dupT | p.C500fs | . | . |
| XS1 | 3 | 123003517 | TG | T | 0.424 | NM_001199642 | ADCY5 | frameshift_deleti  on | exon21 | c.2673delC | p.V891fs | . | . |
| XS1 | 3 | 123634283 | AT | A | 0.987 | NM_022757 | CCDC14 | frameshift_deleti  on | exon12 | c.2081delA | p.N694fs | . | . |
| XS1 | 3 | 124646705 | A | AAAG | 1 | NM_033049 | MUC13 | nonframeshift_in  sertion | exon2 | c.184_185insCT  T | p.F62delinsSF | . | . |
| XS1 | 3 | 125690942 | GA | G | 0.66 | NM_001012337 | ROPN1B | frameshift_deleti  on | exon2 | c.46delA | p.K16fs | . | . |
| XS1 | 3 | 127774524 | GT | G | 0.386 | NM_013336 | SEC61A1 | frameshift_deleti  on | exon4 | c.151delT | p.F51fs | . | . |
| XS1 | 3 | 128780780 | GC | G | 0.287 | NM_000174 | GP9 | frameshift_deleti  on | exon3 | c.199delC | p.P67fs | . | . |
| XS1 | 3 | 130282324 | GA | G | 0.8 | NM_001102608 | COL6A6 | frameshift_deleti  on | exon2 | c.478delA | p.K160fs | . | . |

| XS1 | 3 | 134670694 | AT | A | 0.368 | NM_004441 | EPHB1 | frameshift_deleti  on | exon3 | c.606delT | p.N202fs | . | . |
| --- | --- | --- | --- | --- | --- | --- | --- | --- | --- | --- | --- | --- | --- |
| XS1 | 3 | 135720711 | TA | T | 0.25 | NM_002718 | PPP2R3A | frameshift_deleti  on | exon2 | c.372delA | p.I124fs | . | . |
| XS1 | 3 | 137892417 | TC | T | 1 | NM_016216 | DBR1 | frameshift_deleti  on | exon2 | c.248delG | p.G83fs | . | . |
| XS1 | 3 | 138153747 | GT | G | 0.174 | NM_031913 | ESYT3 | stopgain | exon1 | c.108delT | p.C36X | . | . |
| XS1 | 3 | 141164115 | TG | T | 0.286 | NM_001080412 | ZBTB38 | frameshift_deleti on | exon8 | c.2886delG | p.V962fs | . | . |
| XS1 | 3 | 142383142 | TA | T | 0.417 | NM_001145319 | PLS1 | frameshift_deleti  on | exon2 | c.64delA | p.K22fs | . | . |
| XS1 | 3 | 142987748 | GA | G | 0.235 | NM_173653 | SLC9A9 | frameshift_deleti  on | exon15 | c.1678delT | p.S560fs | . | . |
| XS1 | 3 | 147114192 | GC | G | 0.333 | NM_001168378 | ZIC4 | frameshift_deleti  on | exon3 | c.284delG | p.G95fs | . | . |
| XS1 | 3 | 148459050 | CT | C | 0.575 | NM_009585 | AGTR1 | frameshift_deleti  on | exon2 | c.229delT | p.F77fs | . | . |
| XS1 | 3 | 149678561 | TA | T | 1 | NM_183381 | RNF13 | frameshift_deleti  on | exon10 | c.817delA | p.K273fs | . | . |
| XS1 | 3 | 150883650 | GT | G | 0.667 | NM_053002 | MED12L | frameshift_deleti  on | exon10 | c.1376delT | p.V459fs | . | . |
| XS1 | 3 | 151012090 | AAG | A | 0.893 | NM_023915 | GPR87 | frameshift_deleti  on | exon3 | c.942_943del | p.Y314fs | . | . |
| XS1 | 3 | 151012168 | TG | T | 0.885 | NM_023915 | GPR87 | frameshift_deleti  on | exon3 | c.865delC | p.Q289fs | . | . |
| XS1 | 3 | 151055778 | ACC | A | 0.393 | NM_176876 | P2RY12 | frameshift_deleti  on | exon2 | c.854_855del | p.W285fs | . | . |
| XS1 | 3 | 151155304 | C | CA | 0.261 | NM_001178145 | IGSF10 | frameshift_inserti  on | exon2 | c.1125dupT | p.A376fs | . | . |
| XS1 | 3 | 154147269 | CA | C | 0.386 | NM_001038705 | GPR149 | frameshift_deleti  on | exon1 | c.135delT | p.F45fs | . | . |
| XS1 | 3 | 154864942 | AT | A | 0.5 | NM_000902 | MME | frameshift_deleti  on | exon15 | c.1427delT | p.I476fs | . | . |
| XS1 | 3 | 155199675 | T | TA | 0.962 | NM_014996 | PLCH1 | frameshift_inserti  on | exon22 | c.4049dupT | p.L1350fs | . | . |
| XS1 | 3 | 155861059 | A | AC | 0.286 | NM_003471 | KCNAB1 | frameshift_inserti  on | exon1 | c.93dupC | p.H31fs | . | . |
| XS1 | 3 | 161221067 | TG | T | 0.27 | NM_001080440 | OTOL1 | frameshift_deleti  on | exon4 | c.772delG | p.E258fs | . | . |
| XS1 | 3 | 164750433 | A | AG | 0.75 | NM_001041 | SI | frameshift_inserti  on | exon24 | c.2612dupC | p.T871fs | . | . |
| XS1 | 3 | 168813026 | AT | A | 0.75 | NM_001163999 | MECOM | frameshift_deleti  on | exon11 | c.2268delA | p.K756fs | . | . |
| XS1 | 3 | 169993062 | GT | G | 0.6 | NM_002740 | PRKCI | frameshift_deleti  on | exon8 | c.693delT | p.G231fs | . | . |
| XS1 | 3 | 179298985 | AT | A | 1 | NM_004301 | ACTL6A | frameshift_deleti  on | exon11 | c.1004delT | p.M335fs | . | . |
| XS1 | 3 | 182897909 | CA | C | 0.773 | NM_015078 | MCF2L2 | frameshift_deleti  on | exon28 | c.3054delT | p.F1018fs | . | . |
| XS1 | 3 | 183368478 | AT | A | 0.409 | NM_017644 | KLHL24 | frameshift_deleti  on | exon3 | c.335delT | p.I112fs | . | . |
| XS1 | 3 | 183495335 | AT | A | 1 | NM_018023 | YEATS2 | frameshift_deleti  on | exon19 | c.2584delT | p.F862fs | . | . |
| XS1 | 3 | 183882954 | AGCG | A | 0.274 | NM_004423 | DVL3 | nonframeshift_de  letion | exon6 | c.654_656del | p.218_219del | . | . |
| XS1 | 3 | 183952480 | C | CT | 0.333 | NM_138345 | VWA5B2 | frameshift_inserti  on | exon6 | c.882_883insT | p.A294fs | . | . |
| XS1 | 3 | 184003285 | ATC | A | 0.163 | NM_001037324 | ECE2 | frameshift_deleti  on | exon9 | c.1082_1083del | p.I361fs | . | . |
| XS1 | 3 | 184045727 | TG | T | 1 | NM_004953 | EIF4G1 | frameshift_deleti  on | exon19 | c.3306delG | p.M1102fs | . | . |
| XS1 | 3 | 185214753 | AG | A | 0.625 | NM_080652 | TMEM41A | frameshift_deleti  on | exon2 | c.135delC | p.P45fs | . | . |
| XS1 | 3 | 185906164 | TC | T | 0.326 | NM_001080744 | DGKG | frameshift_deleti  on | exon21 | c.1846delG | p.D616fs | . | . |
| XS1 | 3 | 186502424 | TG | T | 0.231 | NM_001967 | EIF4A2 | frameshift_deleti  on | exon3 | c.148delG | p.A50fs | . | . |
| XS1 | 3 | 188426132 | CA | C | 0.714 | NM_001167672 | LPP | frameshift_deleti  on | exon6 | c.751delA | p.K251fs | . | . |
| XS1 | 3 | 189612009 | GA | G | 1 | NM_001114980 | TP63 | frameshift_deleti  on | exon12 | c.1480delA | p.K494fs | . | . |
| XS1 | 3 | 192517003 | AT | A | 0.706 | NM_178496 | MB21D2 | frameshift_deleti  on | exon2 | c.647delA | p.N216fs | . | . |
| XS1 | 3 | 194126830 | A | AG | 0.2 | NM_024524 | ATP13A3 | frameshift_inserti  on | exon32 | c.3498_3499ins  C | p.W1167fs | . | . |
| XS1 | 3 | 194181470 | AGAG | A | 0.138 | NM_024524 | ATP13A3 | nonframeshift_de  letion | exon3 | c.139_141del | p.47_47del | . | . |
| XS1 | 3 | 195047651 | CT | C | 1 | NM_012287 | ACAP2 | frameshift_deleti  on | exon9 | c.706delA | p.R236fs | . | . |
| XS1 | 3 | 195518110 | G | GATGT  CTCCT | 0.899 | NM_018406 | MUC4 | nonframeshift_in  sertion | exon2 | c.340_341insTT  ACGCAGGAGA | p.A114delinsVT  QETS | . | . |
| XS1 | 3 | 195518112 | T | TGTCTC  CTGCG | 1 | NM_018406 | MUC4 | nonframeshift_in  sertion | exon2 | c.338_339insTG  TTACGCAGGA | p.T113delinsTV  TQET | . | . |
| XS1 | 3 | 195594791 | AC | A | 0.429 | NM_005781 | TNK2 | frameshift_deleti  on | exon12 | c.2332delG | p.V778fs | . | . |
| XS1 | 3 | 196744004 | GC | G | 0.424 | NM_005929 | MFI2 | frameshift_deleti  on | exon7 | c.869delG | p.G290fs | . | . |
| XS1 | 4 | 59955 | TG | T | 0.238 | NM_001039127 | ZNF718 | frameshift_deleti  on | exon3 | c.137delG | p.C46fs | . | . |
| XS1 | 4 | 338176 | GC | G | 0.8 | NM_003441 | ZNF141 | frameshift_deleti  on | exon3 | c.184delC | p.P62fs | . | . |
| XS1 | 4 | 367133 | CG | C | 0.185 | NM_003441 | ZNF141 | frameshift_deleti  on | exon4 | c.908delG | p.R303fs | . | . |
| XS1 | 4 | 436382 | TA | T | 0.818 | NM_133474 | ZNF721 | frameshift_deleti  on | exon3 | c.1873delT | p.Y625fs | . | . |
| XS1 | 4 | 1087327 | G | GCTGC  CCAGG CTGGA | 1 | NM_001193318 | RNF212 | nonframeshift_in sertion | exon4 | c.721_722insG  GCTGGCTCCA GCCTGGGCAG | p.S241delinsW LAPAWAA | . | . |
| XS1 | 4 | 1843381 | A | AC | 0.222 | NM_012318 | LETM1 | frameshift_inserti  on | exon3 | c.286dupG | p.V96fs | . | . |
| XS1 | 4 | 2438718 | C | CT | 0.208 | NM_001193282 | RP11-  503N18.1 | frameshift_inserti  on | exon3 | c.230dupT | p.L77fs | . | . |
| XS1 | 4 | 2648432 | AC | A | 0.824 | NM_001256666 | FAM193A | frameshift_deleti  on | exon5 | c.312delC | p.D104fs | . | . |
| XS1 | 4 | 4304135 | CA | C | 0.852 | NM_145291 | ZBTB49 | frameshift_deleti  on | exon3 | c.573delA | p.S191fs | . | . |
| XS1 | 4 | 5841302 | AG | A | 0.8 | NM_001014809 | CRMP1 | frameshift_deleti  on | exon9 | c.1256delC | p.P419fs | . | . |
| XS1 | 4 | 8079366 | GC | G | 0.82 | NM_001130083 | ABLIM2 | frameshift_deleti  on | exon6 | c.663delG | p.G221fs | . | . |
| XS1 | 4 | 24578130 | AT | A | 0.238 | NM_001358 | DHX15 | frameshift_deleti  on | exon2 | c.242delA | p.H81fs | . | . |
| XS1 | 4 | 26744189 | C | CT | 0.935 | NM_018317 | TBC1D19 | frameshift_inserti  on | exon18 | c.1288dupT | p.L429fs | . | . |

| XS1 | 4 | 27000858 | GC | G | 0.667 | NM_001169117 | STIM2 | frameshift_deleti  on | exon5 | c.515delC | p.A172fs | . | . |
| --- | --- | --- | --- | --- | --- | --- | --- | --- | --- | --- | --- | --- | --- |
| XS1 | 4 | 36231034 | TA | T | 0.9 | NM_015230 | ARAP2 | frameshift_deleti  on | exon2 | c.74delT | p.L25fs | . | . |
| XS1 | 4 | 38119733 | GCC | G | 0.338 | NM_001253913 | TBC1D1 | frameshift_deleti  on | exon3 | c.174_175del | p.G58fs | . | . |
| XS1 | 4 | 39271660 | CA | C | 0.764 | NM_025132 | WDR19 | frameshift_deleti  on | exon31 | c.3424delA | p.K1142fs | . | . |
| XS1 | 4 | 39322069 | AT | A | 1 | NM_001204747 | RFC1 | frameshift_deleti  on | exon9 | c.1028delA | p.N343fs | . | . |
| XS1 | 4 | 39839534 | TG | T | 0.235 | NM_001100399 | PDS5A | frameshift_deleti  on | exon32 | c.3951delC | p.P1317fs | . | . |
| XS1 | 4 | 40103911 | C | CT | 0.467 | NM_018177 | N4BP2 | frameshift_inserti  on | exon4 | c.447dupT | p.A149fs | . | . |
| XS1 | 4 | 48529605 | TG | T | 0.333 | NM_015030 | FRYL | frameshift_deleti  on | exon53 | c.7205delC | p.T2402fs | . | . |
| XS1 | 4 | 48581079 | AT | A | 0.788 | NM_015030 | FRYL | frameshift_deleti  on | exon23 | c.2438delA | p.N813fs | . | . |
| XS1 | 4 | 48608508 | GA | G | 1 | NM_015030 | FRYL | frameshift_deleti  on | exon10 | c.687delT | p.F229fs | . | . |
| XS1 | 4 | 52861791 | TG | T | 0.228 | NM_001024611 | LRRC66 | frameshift_deleti  on | exon4 | c.1396delC | p.H466fs | . | . |
| XS1 | 4 | 55976624 | TA | T | 0.772 | NM_002253 | KDR | frameshift_deleti  on | exon9 | c.1200delT | p.L400fs | . | . |
| XS1 | 4 | 56304529 | TCTG | T | 0.152 | NM_004898 | CLOCK | nonframeshift_de  letion | exon22 | c.2278_2280del | p.760_760del | . | . |
| XS1 | 4 | 57366799 | TA | T | 0.442 | NM_001267722 | SRP72 | frameshift_deleti  on | exon16 | c.1594delA | p.K532fs | . | . |
| XS1 | 4 | 68619966 | TG | T | 1 | NM_000406 | GNRHR | frameshift_deleti  on | exon1 | c.87delC | p.P29fs | . | . |
| XS1 | 4 | 68777212 | CA | C | 0.5 | NM_001114387 | TMPRSS11A | frameshift_deleti  on | exon10 | c.1104delT | p.S368fs | . | . |
| XS1 | 4 | 69513031 | CT | C | 0.286 | NM_001076 | UGT2B15 | frameshift_deleti  on | exon6 | c.1383delA | p.A461fs | . | . |
| XS1 | 4 | 71508588 | TC | T | 0.231 | NM_031889 | ENAM | frameshift_deleti  on | exon9 | c.1446delC | p.V482fs | . | . |
| XS1 | 4 | 71554871 | AGAG | A | 0.205 | NM_020368 | UTP3 | nonframeshift_de  letion | exon1 | c.478_480del | p.160_160del | . | . |
| XS1 | 4 | 72306403 | AC | A | 0.75 | NM_003759 | SLC4A4 | frameshift_deleti  on | exon5 | c.747delC | p.D249fs | . | . |
| XS1 | 4 | 72338545 | CT | C | 0.333 | NM_003759 | SLC4A4 | frameshift_deleti  on | exon11 | c.1630delT | p.F544fs | . | . |
| XS1 | 4 | 74005897 | C | CA | 0.786 | NM_001286771 | ANKRD17 | frameshift_inserti  on | exon15 | c.2096_2097ins  T | p.Q699fs | . | . |
| XS1 | 4 | 74012960 | GC | G | 0.278 | NM_001286771 | ANKRD17 | frameshift_deleti  on | exon9 | c.1417delG | p.A473fs | . | . |
| XS1 | 4 | 74124078 | CCA | C | 1 | NM_032217 | ANKRD17 | frameshift_deleti  on | exon1 | c.306_307del | p.G102fs | . | . |
| XS1 | 4 | 76452353 | AC | A | 0.667 | NM_144721 | THAP6 | frameshift_deleti  on | exon5 | c.599delC | p.T200fs | . | . |
| XS1 | 4 | 76522178 | TG | T | 0.421 | NM_003948 | CDKL2 | frameshift_deleti  on | exon9 | c.1262delC | p.A421fs | . | . |
| XS1 | 4 | 77065318 | C | CTGT | 0.395 | NM_017426 | NUP54 | nonframeshift_in  sertion | exon3 | c.278_279insAC  A | p.Q93delinsQQ | . | . |
| XS1 | 4 | 77305520 | AT | A | 0.395 | NM_001042784 | CCDC158 | frameshift_deleti  on | exon5 | c.446delA | p.N149fs | . | . |
| XS1 | 4 | 79792082 | CCAG | C | 0.132 | NM_017593 | BMP2K | nonframeshift_de  letion | exon11 | c.1378_1380del | p.460_460del | . | . |
| XS1 | 4 | 79792136 | ACAGCA  GCAG | A | 0.966 | NM_017593 | BMP2K | nonframeshift_de  letion | exon11 | c.1432_1440del | p.478_480del | . | . |
| XS1 | 4 | 79792136 | ACAGCA  GCAGCA | A | 0.966 | NM_017593 | BMP2K | nonframeshift_de  letion | exon11 | c.1432_1443del | p.478_481del | . | . |
| XS1 | 4 | 79792151 | GCAGCA  GCAGCA | G | 0.75 | NM_017593 | BMP2K | nonframeshift_de  letion | exon11 | c.1447_1461del | p.483_487del | . | . |
| XS1 | 4 | 79792151 | GCAGCA  GCAGCA GCACCA | G | 0.75 | NM_017593 | BMP2K | nonframeshift_de letion | exon11 | c.1447_1467del | p.483_489del | . | . |
| XS1 | 4 | 79792154 | GCAGCA  GCAGCA | G | 0.886 | NM_017593 | BMP2K | nonframeshift_de  letion | exon11 | c.1450_1461del | p.484_487del | . | . |
| XS1 | 4 | 81207764 | C | CT | 0.189 | NM_004464 | FGF5 | frameshift_inserti  on | exon3 | c.746dupT | p.L249fs | . | . |
| XS1 | 4 | 81967469 | G | GA | 0.4 | NM_001201 | BMP3 | frameshift_inserti  on | exon2 | c.895dupA | p.Q298fs | . | . |
| XS1 | 4 | 85687080 | G | GA | 0.273 | NM_014991 | WDFY3 | frameshift_inserti  on | exon32 | c.5070dupT | p.L1691fs | . | . |
| XS1 | 4 | 88940681 | G | GA | 0.271 | NM_000297 | PKD2 | frameshift_inserti  on | exon2 | c.668dupA | p.E223fs | . | . |
| XS1 | 4 | 89352369 | TC | T | 0.385 | NM_001165136 | HERC6 | frameshift_deleti  on | exon16 | c.2055delC | p.F685fs | . | . |
| XS1 | 4 | 89391780 | AT | A | 0.265 | NM_016323 | HERC5 | frameshift_deleti  on | exon10 | c.1255delT | p.F419fs | . | . |
| XS1 | 4 | 89407331 | C | CA | 0.611 | NM_016323 | HERC5 | frameshift_inserti  on | exon14 | c.1804dupA | p.L601fs | . | . |
| XS1 | 4 | 91230254 | AT | A | 0.954 | NM_001145065 | CCSER1 | frameshift_deleti  on | exon2 | c.820delT | p.F274fs | . | . |
| XS1 | 4 | 92519750 | CA | C | 1 | NM_001145065 | CCSER1 | frameshift_deleti  on | exon11 | c.2246delA | p.Q749fs | . | . |
| XS1 | 4 | 95539245 | AC | A | 0.208 | NM_001256425 | PDLIM5 | frameshift_deleti  on | exon7 | c.37delC | p.P13fs | . | . |
| XS1 | 4 | 99399940 | CA | C | 0.455 | NM_005723 | TSPAN5 | frameshift_deleti  on | exon5 | c.471delT | p.F157fs | . | . |
| XS1 | 4 | 100460405 | AC | A | 0.933 | NM_032149 | C4orf17 | frameshift_deleti  on | exon7 | c.715delC | p.P239fs | . | . |
| XS1 | 4 | 101109228 | TTC | T | 0.625 | NM_145244 | DDIT4L | frameshift_deleti  on | exon3 | c.186_187del | p.E62fs | . | . |
| XS1 | 4 | 104098154 | AT | A | 0.391 | NM_001286734 | CENPE | stopgain | exon14 | c.1291delA | p.M431X | . | . |
| XS1 | 4 | 109543320 | CA | C | 0.833 | NM_000995 | RPL34 | frameshift_deleti on | exon3 | c.126delA | p.P42fs | . | . |
| XS1 | 4 | 111430854 | G | GC | 0.545 | NM_001977 | ENPEP | frameshift_inserti  on | exon5 | c.1085_1086ins  C | p.W362fs | . | . |
| XS1 | 4 | 113539443 | CT | C | 0.24 | NM_018392 | C4orf21 | frameshift_deleti  on | exon6 | c.1754delA | p.E585fs | . | . |
| XS1 | 4 | 114214678 | TCAC | T | 0.194 | NM_001148 | ANK2 | nonframeshift_de  letion | exon22 | c.2460_2462del | p.820_821del | . | . |
| XS1 | 4 | 119736192 | GA | G | 0.6 | NM_014822 | SEC24D | frameshift_deleti  on | exon6 | c.791delT | p.I264fs | . | . |
| XS1 | 4 | 119947961 | AAAG | A | 1 | NM_001128933 | SYNPO2 | nonframeshift_de  letion | exon3 | c.438_440del | p.146_147del | . | . |
| XS1 | 4 | 122250739 | AT | A | 0.647 | NM_198179 | QRFPR | frameshift_deleti  on | exon6 | c.1025delA | p.N342fs | . | . |
| XS1 | 4 | 123097024 | AT | A | 0.583 | NM_015312 | KIAA1109 | frameshift_deleti  on | exon4 | c.314delT | p.I105fs | . | . |

| XS1 | 4 | 123161437 | CT | C | 1 | NM_015312 | KIAA1109 | frameshift_deleti  on | exon27 | c.4601delT | p.L1534fs | . | . |
| --- | --- | --- | --- | --- | --- | --- | --- | --- | --- | --- | --- | --- | --- |
| XS1 | 4 | 123164204 | C | CT | 0.24 | NM_015312 | KIAA1109 | frameshift_inserti  on | exon28 | c.4724dupT | p.L1575fs | . | . |
| XS1 | 4 | 123271046 | AT | A | 0.427 | NM_015312 | KIAA1109 | frameshift_deleti  on | exon78 | c.13667delT | p.I4556fs | . | . |
| XS1 | 4 | 125591029 | AT | A | 0.657 | NM_001167882 | ANKRD50 | frameshift_deleti  on | exon3 | c.2865delA | p.K955fs | . | . |
| XS1 | 4 | 125591237 | T | TA | 0.18 | NM_001167882 | ANKRD50 | frameshift_inserti  on | exon3 | c.2657dupT | p.L886fs | . | . |
| XS1 | 4 | 126370072 | A | AT | 0.767 | NM_024582 | FAT4 | frameshift_inserti  on | exon9 | c.7901_7902ins  T | p.K2634fs | . | . |
| XS1 | 4 | 128817971 | GT | G | 0.222 | NM_001190799 | PLK4 | frameshift_deleti  on | exon14 | c.2624delT | p.V875fs | . | . |
| XS1 | 4 | 129792829 | GA | G | 0.281 | NM_001287437 | JADE1 | frameshift_deleti  on | exon11 | c.1906delA | p.K636fs | . | . |
| XS1 | 4 | 135121545 | GA | G | 0.4 | NM_001114734 | PABPC4L | frameshift_deleti  on | exon2 | c.803delT | p.F268fs | . | . |
| XS1 | 4 | 141481159 | AC | A | 0.545 | NM_021833 | UCP1 | frameshift_deleti  on | exon6 | c.814delG | p.V272fs | . | . |
| XS1 | 4 | 141832389 | CA | C | 0.96 | NM_020724 | RNF150 | frameshift_deleti  on | exon6 | c.1106delT | p.L369fs | . | . |
| XS1 | 4 | 146686171 | TC | T | 0.532 | NM_178835 | ZNF827 | frameshift_deleti  on | exon13 | c.3198delG | p.K1066fs | . | . |
| XS1 | 4 | 146806917 | AG | A | 0.306 | NM_178835 | ZNF827 | frameshift_deleti  on | exon4 | c.1659delC | p.P553fs | . | . |
| XS1 | 4 | 148578978 | C | CT | 0.263 | NM_138364 | PRMT9 | stopgain | exon8 | c.1294_1295ins  A | p.W432_E433d  elinsX | . | . |
| XS1 | 4 | 149356815 | GT | G | 0.793 | NM_000901 | NR3C2 | frameshift_deleti  on | exon2 | c.1197delA | p.K399fs | . | . |
| XS1 | 4 | 149357958 | A | AG | 0.818 | NM_000901 | NR3C2 | frameshift_inserti  on | exon2 | c.54_55insC | p.W19fs | . | . |
| XS1 | 4 | 151177305 | C | CA | 0.217 | NM_001040260 | DCLK2 | frameshift_inserti  on | exon16 | c.2207_2208ins  A | p.P736fs | . | . |
| XS1 | 4 | 151271225 | GA | G | 0.875 | NM_001199282 | LRBA | frameshift_deleti  on | exon48 | c.7280delT | p.V2427fs | . | . |
| XS1 | 4 | 151836845 | CG | C | 0.375 | NM_001199282 | LRBA | frameshift_deleti  on | exon8 | c.928delC | p.R310fs | . | . |
| XS1 | 4 | 153244292 | TTG | T | 0.852 | NM_001013415 | FBXW7 | frameshift_deleti  on | exon11 | c.1509_1510del | p.N503fs | . | . |
| XS1 | 4 | 153691256 | AT | A | 0.667 | NM_145720 | TIGD4 | frameshift_deleti  on | exon2 | c.900delA | p.K300fs | . | . |
| XS1 | 4 | 154624690 | AT | A | 0.275 | NM_003264 | TLR2 | frameshift_deleti  on | exon3 | c.632delT | p.I211fs | . | . |
| XS1 | 4 | 155180870 | CA | C | 0.667 | NM_017639 | DCHS2 | frameshift_deleti  on | exon20 | c.5250delT | p.F1750fs | . | . |
| XS1 | 4 | 155468935 | CT | C | 0.444 | NM_001201564 | PLRG1 | frameshift_deleti  on | exon3 | c.186delA | p.K62fs | . | . |
| XS1 | 4 | 155665486 | AC | A | 0.571 | NM_004744 | LRAT | frameshift_deleti  on | exon2 | c.9delC | p.N3fs | . | . |
| XS1 | 4 | 159629550 | TG | T | 1 | NM_001281738 | ETFDH | frameshift_deleti  on | exon11 | c.1543delG | p.G515fs | . | . |
| XS1 | 4 | 169146682 | AT | A | 0.9 | NM_017631 | DDX60 | frameshift_deleti  on | exon34 | c.4678delA | p.I1560fs | . | . |
| XS1 | 4 | 169209414 | CA | C | 0.821 | NM_017631 | DDX60 | frameshift_deleti  on | exon9 | c.1093delT | p.W365fs | . | . |
| XS1 | 4 | 170037497 | C | CA | 0.212 | NM_020870 | SH3RF1 | frameshift_inserti  on | exon10 | c.2061dupT | p.G688fs | . | . |
| XS1 | 4 | 170618732 | TC | T | 0.4 | NM_001243372 | CLCN3 | frameshift_deleti  on | exon8 | c.1330delC | p.L444fs | . | . |
| XS1 | 4 | 174219315 | GAA | G | 0.8 | NM_017423 | GALNT7 | frameshift_deleti  on | exon6 | c.1016_1017del | p.E339fs | . | . |
| XS1 | 4 | 186339601 | TG | T | 0.938 | NM_018359 | UFSP2 | stopgain | exon4 | c.326delC | p.S109X | . | . |
| XS1 | 4 | 187557183 | G | GA | 0.667 | NM_005245 | FAT1 | frameshift_inserti on | exon6 | c.4178dupT | p.I1393fs | . | . |
| XS1 | 5 | 11082881 | A | AT | 0.237 | NM_001288716 | CTNND2 | frameshift_inserti  on | exon13 | c.1703dupA | p.N568fs | . | . |
| XS1 | 5 | 13901481 | GA | G | 0.643 | NM_001369 | DNAH5 | frameshift_deleti  on | exon14 | c.1931delT | p.F644fs | . | . |
| XS1 | 5 | 14359497 | CA | C | 0.667 | NM_007118 | TRIO | frameshift_deleti  on | exon13 | c.2249delA | p.H750fs | . | . |
| XS1 | 5 | 16675065 | CG | C | 0.271 | NM_012334 | MYO10 | frameshift_deleti  on | exon35 | c.4860delC | p.P1620fs | . | . |
| XS1 | 5 | 31323034 | TG | T | 0.975 | NM_004932 | CDH6 | frameshift_deleti  on | exon12 | c.1993delG | p.G665fs | . | . |
| XS1 | 5 | 33576961 | GA | G | 0.57 | NM_030955 | ADAMTS12 | frameshift_deleti  on | exon19 | c.3169delT | p.S1057fs | . | . |
| XS1 | 5 | 33658407 | AG | A | 0.33 | NM_030955 | ADAMTS12 | frameshift_deleti  on | exon7 | c.1071delC | p.P357fs | . | . |
| XS1 | 5 | 33937419 | CG | C | 0.727 | NM_016568 | RXFP3 | frameshift_deleti  on | exon1 | c.575delG | p.R192fs | . | . |
| XS1 | 5 | 36037917 | TG | T | 0.4 | NM_001168316 | UGT3A2 | frameshift_deleti  on | exon5 | c.1174delC | p.Q392fs | . | . |
| XS1 | 5 | 36064435 | G | GA | 0.196 | NM_174914 | UGT3A2 | frameshift_inserti  on | exon2 | c.111_112insT | p.L38fs | . | . |
| XS1 | 5 | 36066818 | AT | A | 0.278 | NM_001168316 | UGT3A2 | frameshift_deleti  on | exon1 | c.73delA | p.I25fs | . | . |
| XS1 | 5 | 36995898 | CT | C | 0.375 | NM_015384 | NIPBL | frameshift_deleti  on | exon11 | c.3297delT | p.A1099fs | . | . |
| XS1 | 5 | 37326066 | G | GT | 0.25 | NM_001278312 | NUP155 | frameshift_inserti  on | exon19 | c.2027dupA | p.N676fs | . | . |
| XS1 | 5 | 39134365 | TA | T | 1 | NM_001243093 | FYB | frameshift_deleti  on | exon9 | c.1791delT | p.P597fs | . | . |
| XS1 | 5 | 41018792 | CAT | C | 0.156 | NM_173489 | MROH2B | frameshift_deleti  on | exon26 | c.2672_2673del | p.N891fs | . | . |
| XS1 | 5 | 41853578 | AT | A | 0.241 | NM_000436 | OXCT1 | frameshift_deleti  on | exon4 | c.356delA | p.N119fs | . | . |
| XS1 | 5 | 45396654 | GAC | G | 1 | NM_021072 | HCN1 | frameshift_deleti  on | exon4 | c.1168_1169del | p.V390fs | . | . |
| XS1 | 5 | 54579551 | AT | A | 0.667 | NM_019030 | DHX29 | frameshift_deleti  on | exon11 | c.1444delA | p.M482fs | . | . |
| XS1 | 5 | 56778466 | AC | A | 0.9 | NM_001017992 | ACTBL2 | frameshift_deleti  on | exon1 | c.68delG | p.G23fs | . | . |
| XS1 | 5 | 61876798 | GA | G | 0.455 | NM_181506 | LRRC70 | frameshift_deleti  on | exon2 | c.1534delA | p.K512fs | . | . |
| XS1 | 5 | 66416852 | AT | A | 0.706 | NM_015183 | MAST4 | frameshift_deleti  on | exon13 | c.1101delT | p.N367fs | . | . |
| XS1 | 5 | 67591303 | GA | G | 0.714 | NM_001242466 | PIK3R1 | frameshift_deleti  on | exon7 | c.713delA | p.E238fs | . | . |
| XS1 | 5 | 68390120 | CCGG | C | 0.2 | NM_001251969 | SLC30A5 | nonframeshift_de  letion | exon1 | c.39_41del | p.13_14del | . | . |
| XS1 | 5 | 68669733 | TA | T | 0.588 | NM_133339 | RAD17 | frameshift_deleti  on | exon2 | c.120delA | p.L40fs | . | . |
| XS1 | 5 | 71494081 | AC | A | 0.423 | NM_005909 | MAP1B | frameshift_deleti  on | exon5 | c.4900delC | p.P1634fs | . | . |

| XS1 | 5 | 71528284 | TC | T | 0.333 | NM_001286751 | MRPS27 | frameshift_deleti  on | exon7 | c.408delG | p.K136fs | . | . |
| --- | --- | --- | --- | --- | --- | --- | --- | --- | --- | --- | --- | --- | --- |
| XS1 | 5 | 74017513 | AT | A | 0.636 | NM_170691 | GFM2 | frameshift_deleti  on | exon20 | c.2165delA | p.N722fs | . | . |
| XS1 | 5 | 74026208 | TA | T | 0.375 | NM_170691 | GFM2 | frameshift_deleti  on | exon16 | c.1461delT | p.G487fs | . | . |
| XS1 | 5 | 74488492 | CTT | C | 1 | NM_001164443 | ANKRD31 | frameshift_deleti  on | exon9 | c.1406_1407del | p.K469fs | . | . |
| XS1 | 5 | 74488492 | CT | C | 1 | NM_001164443 | ANKRD31 | frameshift_deleti  on | exon9 | c.1407delA | p.K469fs | . | . |
| XS1 | 5 | 74491715 | TTCA | T | 1 | NM_001164443 | ANKRD31 | nonframeshift_de  letion | exon7 | c.755_757del | p.252_253del | . | . |
| XS1 | 5 | 74807103 | CG | C | 0.273 | NM_001130105 | COL4A3BP | frameshift_deleti  on | exon2 | c.313delC | p.R105fs | . | . |
| XS1 | 5 | 75581051 | TGC | T | 0.333 | NM_014979 | SV2C | frameshift_deleti  on | exon5 | c.979_980del | p.A327fs | . | . |
| XS1 | 5 | 75960985 | GT | G | 0.667 | NM_001285461 | IQGAP2 | frameshift_deleti  on | exon10 | c.1153delT | p.F385fs | . | . |
| XS1 | 5 | 76734164 | C | CT | 0.286 | NM_018268 | WDR41 | frameshift_inserti  on | exon10 | c.904dupA | p.R302fs | . | . |
| XS1 | 5 | 78610432 | CT | C | 0.333 | NM_152405 | JMY | frameshift_deleti  on | exon9 | c.2418delT | p.P806fs | . | . |
| XS1 | 5 | 79025853 | TAA | T | 0.438 | NM_153610 | CMYA5 | frameshift_deleti  on | exon2 | c.1266_1267del | p.V422fs | . | . |
| XS1 | 5 | 79026097 | AC | A | 0.267 | NM_153610 | CMYA5 | frameshift_deleti  on | exon2 | c.1510delC | p.P504fs | . | . |
| XS1 | 5 | 80555018 | AC | A | 0.296 | NM_001099735 | CKMT2 | frameshift_deleti  on | exon8 | c.960delC | p.N320fs | . | . |
| XS1 | 5 | 82836960 | CA | C | 0.891 | NM_001164097 | VCAN | frameshift_deleti  on | exon7 | c.5178delA | p.A1726fs | . | . |
| XS1 | 5 | 82837055 | GA | G | 0.184 | NM_001164097 | VCAN | frameshift_deleti  on | exon7 | c.5273delA | p.E1758fs | . | . |
| XS1 | 5 | 90052801 | TG | T | 1 | NM_032119 | GPR98 | frameshift_deleti  on | exon57 | c.11764delG | p.G3922fs | . | . |
| XS1 | 5 | 93077147 | AG | A | 0.299 | NM_153216 | POU5F2 | frameshift_deleti  on | exon1 | c.122delC | p.P41fs | . | . |
| XS1 | 5 | 96513456 | AT | A | 0.8 | NM_001159749 | RIOK2 | frameshift_deleti  on | exon3 | c.301delA | p.M101fs | . | . |
| XS1 | 5 | 112177188 | A | AT | 1 | NM_001127511 | APC | frameshift_inserti  on | exon14 | c.5844dupT | p.N1948fs | . | . |
| XS1 | 5 | 114860101 | C | CA | 1 | NM_020177 | FEM1C | frameshift_inserti  on | exon3 | c.1757dupT | p.L586fs | . | . |
| XS1 | 5 | 115394573 | TGG | T | 0.19 | NM_001195581 | ARL14EPL | frameshift_deleti  on | exon3 | c.389_390del | p.W130fs | . | . |
| XS1 | 5 | 118810141 | CT | C | 0.228 | NM_001199292 | HSD17B4 | frameshift_deleti  on | exon3 | c.213delT | p.A71fs | . | . |
| XS1 | 5 | 121776364 | AG | A | 0.429 | NM_001242935 | SNCAIP | frameshift_deleti  on | exon5 | c.240delG | p.Q80fs | . | . |
| XS1 | 5 | 126860590 | AT | A | 0.68 | NM_001286808 | PRRC1 | frameshift_deleti  on | exon3 | c.472delT | p.F158fs | . | . |
| XS1 | 5 | 133326716 | TG | T | 0.174 | NM_003374 | VDAC1 | frameshift_deleti  on | exon4 | c.246delC | p.G82fs | . | . |
| XS1 | 5 | 133914786 | CG | C | 0.262 | NM_015288 | JADE2 | frameshift_deleti  on | exon11 | c.2153delG | p.R718fs | . | . |
| XS1 | 5 | 135382676 | AC | A | 1 | NM_000358 | TGFBI | frameshift_deleti  on | exon5 | c.597delC | p.N199fs | . | . |
| XS1 | 5 | 138264975 | AT | A | 0.8 | NM_001903 | CTNNA1 | frameshift_deleti  on | exon14 | c.1941delT | p.D647fs | . | . |
| XS1 | 5 | 138386644 | A | AT | 0.293 | NM_022464 | SIL1 | frameshift_inserti  on | exon4 | c.335dupA | p.N112fs | . | . |
| XS1 | 5 | 138661185 | TA | T | 0.517 | NM_001194956 | MATR3 | frameshift_deleti  on | exon12 | c.1342delA | p.K448fs | . | . |
| XS1 | 5 | 139221857 | A | AT | 0.318 | NM_032289 | PSD2 | frameshift_inserti  on | exon15 | c.2114_2115ins  T | p.K705fs | . | . |
| XS1 | 5 | 139876860 | GC | G | 0.184 | NM_017747 | ANKHD1 | frameshift_deleti  on | exon15 | c.3002delC | p.A1001fs | . | . |
| XS1 | 5 | 140168117 | TG | T | 0.491 | NM_018900 | PCDHA1 | frameshift_deleti  on | exon1 | c.2243delG | p.W748fs | . | . |
| XS1 | 5 | 140255970 | TA | T | 0.27 | NM_018903 | PCDHA12 | frameshift_deleti  on | exon1 | c.914delA | p.Y305fs | . | . |
| XS1 | 5 | 140262243 | GC | G | 0.8 | NM_018904 | PCDHA13 | frameshift_deleti  on | exon1 | c.391delC | p.P131fs | . | . |
| XS1 | 5 | 140553031 | AC | A | 0.352 | NM_018940 | PCDHB7 | frameshift_deleti  on | exon1 | c.616delC | p.P206fs | . | . |
| XS1 | 5 | 140579998 | TG | T | 0.625 | NM_018931 | PCDHB11 | frameshift_deleti  on | exon1 | c.652delG | p.G218fs | . | . |
| XS1 | 5 | 140683253 | GA | G | 0.5 | NM_031947 | SLC25A2 | frameshift_deleti  on | exon1 | c.179delT | p.L60fs | . | . |
| XS1 | 5 | 140755047 | CCA | C | 0.857 | NM_018919 | PCDHGA6 | frameshift_deleti  on | exon1 | c.1398_1399del | p.P466fs | . | . |
| XS1 | 5 | 140755048 | CA | C | 1 | NM_018919 | PCDHGA6 | frameshift_deleti  on | exon1 | c.1399delA | p.R467fs | . | . |
| XS1 | 5 | 140788082 | CA | C | 0.578 | NM_018926 | PCDHGB6 | frameshift_deleti  on | exon1 | c.314delA | p.Q105fs | . | . |
| XS1 | 5 | 140953563 | TGGA | T | 0.2 | NM_001079812 | DIAPH1 | nonframeshift_de  letion | exon15 | c.1824_1826del | p.608_609del | . | . |
| XS1 | 5 | 141033629 | TG | T | 0.22 | NM_022481 | ARAP3 | frameshift_deleti  on | exon33 | c.4522delC | p.Q1508fs | . | . |
| XS1 | 5 | 141041653 | A | AT | 0.207 | NM_022481 | ARAP3 | frameshift_inserti  on | exon20 | c.2969_2970ins  A | p.P990fs | . | . |
| XS1 | 5 | 141335906 | AC | A | 0.571 | NM_016580 | PCDH12 | frameshift_deleti  on | exon1 | c.1510delG | p.V504fs | . | . |
| XS1 | 5 | 141517421 | A | AG | 0.648 | NM_030571 | NDFIP1 | frameshift_inserti  on | exon5 | c.494dupG | p.R165fs | . | . |
| XS1 | 5 | 145826961 | GGGGA | G | 0.4 | NM_001040006 | TCERG1 | frameshift_deleti  on | exon1 | c.50_53del | p.G17fs | . | . |
| XS1 | 5 | 146754698 | AT | A | 0.295 | NM_001112724 | STK32A | frameshift_deleti  on | exon11 | c.950delT | p.I317fs | . | . |
| XS1 | 5 | 149360836 | TG | T | 0.667 | NM_000112 | SLC26A2 | frameshift_deleti  on | exon3 | c.1681delG | p.E561fs | . | . |
| XS1 | 5 | 149433956 | TG | T | 0.375 | NM_001288705 | CSF1R | frameshift_deleti  on | exon20 | c.2691delC | p.P897fs | . | . |
| XS1 | 5 | 149624726 | TCA | T | 0.239 | NM_015981 | CAMK2A | frameshift_deleti  on | exon13 | c.980_981del | p.V327fs | . | . |
| XS1 | 5 | 149754226 | C | CA | 0.375 | NM_000356 | TCOF1 | frameshift_inserti  on | exon8 | c.899_900insA | p.P300fs | . | . |
| XS1 | 5 | 150076231 | GC | G | 0.444 | NM_018047 | RBM22 | frameshift_deleti  on | exon6 | c.408delG | p.M136fs | . | . |
| XS1 | 5 | 150413226 | AG | A | 0.8 | NM_001252386 | TNIP1 | frameshift_deleti  on | exon15 | c.1562delC | p.P521fs | . | . |
| XS1 | 5 | 150885451 | CG | C | 0.336 | NM_001447 | FAT2 | frameshift_deleti  on | exon23 | c.12724delC | p.R4242fs | . | . |
| XS1 | 5 | 150942947 | C | CT | 0.875 | NM_001447 | FAT2 | frameshift_inserti  on | exon2 | c.3512dupA | p.K1171fs | . | . |
| XS1 | 5 | 150945817 | CT | C | 0.2 | NM_001447 | FAT2 | frameshift_deleti  on | exon1 | c.2675delA | p.K892fs | . | . |

| XS1 | 5 | 153765931 | GGC | G | 0.174 | NM_198321 | GALNT10 | frameshift_deleti  on | exon7 | c.998_999del | p.G333fs | . | . |
| --- | --- | --- | --- | --- | --- | --- | --- | --- | --- | --- | --- | --- | --- |
| XS1 | 5 | 156378617 | GT | G | 0.308 | NM_001146726 | TIMD4 | frameshift_deleti  on | exon3 | c.584delA | p.N195fs | . | . |
| XS1 | 5 | 156479568 | C | CGTT | 1 | NM_012206 | HAVCR1 | nonframeshift_in  sertion | exon3 | c.476_477insAA  C | p.T159delinsTT | . | . |
| XS1 | 5 | 156479570 | T | TTGC | 0.979 | NM_012206 | HAVCR1 | nonframeshift_in  sertion | exon3 | c.474_475insG  CA | p.T159delinsAT | . | . |
| XS1 | 5 | 156592871 | GC | G | 0.37 | NM_130899 | FAM71B | frameshift_deleti  on | exon1 | c.308delG | p.G103fs | . | . |
| XS1 | 5 | 157158501 | TC | T | 0.208 | NM_017872 | THG1L | frameshift_deleti  on | exon1 | c.54delC | p.I18fs | . | . |
| XS1 | 5 | 157158525 | TG | T | 0.676 | NM_017872 | THG1L | frameshift_deleti  on | exon1 | c.78delG | p.L26fs | . | . |
| XS1 | 5 | 162905751 | GA | G | 0.333 | NM_001142557 | HMMR | frameshift_deleti  on | exon9 | c.1070delA | p.E357fs | . | . |
| XS1 | 5 | 167850890 | C | CT | 0.469 | NM_001161661 | WWC1 | frameshift_inserti  on | exon11 | c.1627_1628ins  T | p.P543fs | . | . |
| XS1 | 5 | 168100313 | TG | T | 0.231 | NM_001271946 | SLIT3 | frameshift_deleti  on | exon33 | c.3730delC | p.Q1244fs | . | . |
| XS1 | 5 | 169535348 | CG | C | 0.407 | NM_012188 | FOXI1 | frameshift_deleti  on | exon2 | c.871delG | p.G291fs | . | . |
| XS1 | 5 | 172447282 | GA | G | 0.407 | NM_003945 | ATP6V0E1 | frameshift_deleti  on | exon3 | c.195delA | p.G65fs | . | . |
| XS1 | 5 | 175811094 | C | CGT | 0.983 | NM_001256539 | NOP16 | frameshift_inserti  on | exon5 | c.586_587insAC | p.R196fs | . | . |
| XS1 | 5 | 175811099 | G | GTA | 0.929 | NM_001256539 | NOP16 | frameshift_inserti  on | exon5 | c.581_582insTA | p.H194fs | . | . |
| XS1 | 5 | 176008440 | AC | A | 0.459 | NM_001171976 | CDHR2 | frameshift_deleti  on | exon17 | c.1916delC | p.T639fs | . | . |
| XS1 | 5 | 176395638 | GT | G | 0.833 | NM_001199298 | UIMC1 | frameshift_deleti  on | exon6 | c.1117delA | p.T373fs | . | . |
| XS1 | 5 | 176930171 | AGAG | A | 1 | NM_001144876 | DOK3 | nonframeshift_de  letion | exon5 | c.559_561del | p.187_187del | . | . |
| XS1 | 5 | 177546761 | GC | G | 0.963 | NM_015111 | N4BP3 | frameshift_deleti  on | exon2 | c.178delC | p.H60fs | . | . |
| XS1 | 5 | 177573225 | AC | A | 0.344 | NM_001288795 | RMND5B | frameshift_deleti  on | exon8 | c.767delC | p.T256fs | . | . |
| XS1 | 5 | 178358534 | CA | C | 0.667 | NM_030613 | ZFP2 | frameshift_deleti  on | exon5 | c.221delA | p.H74fs | . | . |
| XS1 | 5 | 179034626 | AG | A | 0.182 | NM_001040451 | RUFY1 | frameshift_deleti  on | exon16 | c.1626delG | p.Q542fs | . | . |
| XS1 | 5 | 179196031 | CA | C | 0.312 | NM_014757 | MAML1 | frameshift_deleti  on | exon3 | c.1913delA | p.Q638fs | . | . |
| XS1 | 5 | 179290517 | CT | C | 0.735 | NM_015043 | TBC1D9B | frameshift_deleti  on | exon21 | c.3632delA | p.K1211fs | . | . |
| XS1 | 5 | 179977089 | GC | G | 1 | NM_015455 | CNOT6 | frameshift_deleti  on | exon3 | c.272delC | p.A91fs | . | . |
| XS1 | 6 | 629954 | CA | C | 1 | NM_018303 | EXOC2 | frameshift_deleti  on | exon4 | c.302delT | p.L101fs | . | . |
| XS1 | 6 | 7240725 | AC | A | 0.412 | NM_001003699 | RREB1 | frameshift_deleti  on | exon11 | c.3864delC | p.N1288fs | . | . |
| XS1 | 6 | 10557468 | T | TG | 0.169 | NM_001491 | GCNT2 | frameshift_inserti  on | exon1 | c.812_813insG | p.F271fs | . | . |
| XS1 | 6 | 10874617 | C | CT | 0.625 | NM_004752 | GCM2 | frameshift_inserti  on | exon5 | c.1131_1132ins  A | p.A378fs | . | . |
| XS1 | 6 | 12164048 | C | CA | 0.883 | NM_002114 | HIVEP1 | frameshift_inserti  on | exon9 | c.7512dupA | p.T2504fs | . | . |
| XS1 | 6 | 16327903 | CTGCTG  ATGCTG | C | 0.778 | NM_001128164 | ATXN1 | nonframeshift_de  letion | exon7 | c.627_638del | p.209_213del | . | . |
| XS1 | 6 | 17629486 | TA | T | 0.246 | NM_001278210 | NUP153 | frameshift_deleti  on | exon17 | c.2817delT | p.F939fs | . | . |
| XS1 | 6 | 21000588 | AT | A | 0.538 | NM_017774 | CDKAL1 | frameshift_deleti  on | exon11 | c.1041delT | p.D347fs | . | . |
| XS1 | 6 | 24810010 | AC | A | 0.96 | NM_001286445 | FAM65B | stopgain | exon21 | c.2977delG | p.V993X | . | . |
| XS1 | 6 | 25923975 | AC | A | 0.189 | NM_001286123 | SLC17A2 | frameshift_deleti on | exon2 | c.187delG | p.V63fs | . | . |
| XS1 | 6 | 26123924 | A | AT | 0.214 | NM_003526 | HIST1H2BC | frameshift_inserti  on | exon1 | c.208dupA | p.I70fs | . | . |
| XS1 | 6 | 26235055 | GC | G | 0.289 | NM_005320 | HIST1H1D | frameshift_deleti  on | exon1 | c.106delG | p.A36fs | . | . |
| XS1 | 6 | 27222906 | GC | G | 0.286 | NM_005865 | PRSS16 | frameshift_deleti  on | exon11 | c.1473delC | p.R491fs | . | . |
| XS1 | 6 | 27424605 | C | CT | 0.227 | NM_007149 | ZNF184 | frameshift_inserti  on | exon5 | c.297dupA | p.D100fs | . | . |
| XS1 | 6 | 27834633 | CT | C | 0.909 | NM_005322 | HIST1H1B | frameshift_deleti  on | exon1 | c.674delA | p.K225fs | . | . |
| XS1 | 6 | 27861552 | CG | C | 0.74 | NM_003527 | HIST1H2BO | frameshift_deleti  on | exon1 | c.313delG | p.G105fs | . | . |
| XS1 | 6 | 28402449 | GA | G | 0.924 | NM_001012455 | ZSCAN23 | frameshift_deleti  on | exon4 | c.962delT | p.F321fs | . | . |
| XS1 | 6 | 28891003 | ACCG | A | 0.232 | NM_006510 | TRIM27 | nonframeshift_de  letion | exon1 | c.404_406del | p.135_136del | . | . |
| XS1 | 6 | 30558477 | G | GA | 0.846 | NM_001090 | ABCF1 | frameshift_inserti  on | exon24 | c.2424dupA | p.X808delinsX | . | . |
| XS1 | 6 | 30856999 | AT | A | 0.571 | NM_001202521 | DDR1 | frameshift_deleti  on | exon3 | c.210delT | p.D70fs | . | . |
| XS1 | 6 | 30996487 | GACC | G | 0.25 | NM_001198815 | MUC22 | nonframeshift_de  letion | exon3 | c.3280_3282del | p.1094_1094de  l | . | . |
| XS1 | 6 | 31474878 | T | TC | 0.455 | NM_001289160 | MICB | frameshift_inserti  on | exon4 | c.598dupC | p.Y199fs | . | . |
| XS1 | 6 | 31615587 | TG | T | 0.318 | NM_001098534 | BAG6 | frameshift_deleti  on | exon7 | c.568delC | p.Q190fs | . | . |
| XS1 | 6 | 31708956 | TG | T | 0.257 | NM_002441 | MSH5 | frameshift_deleti  on | exon3 | c.165delG | p.L55fs | . | . |
| XS1 | 6 | 31844836 | AG | A | 0.276 | NM_001178044 | SLC44A4 | frameshift_deleti  on | exon2 | c.63delC | p.P21fs | . | . |
| XS1 | 6 | 31903706 | AG | A | 1 | NM_001282457 | C2 | frameshift_deleti  on | exon3 | c.119delG | p.S40fs | . | . |
| XS1 | 6 | 31903707 | GC | G | 1 | NM_001282457 | C2 | frameshift_deleti  on | exon3 | c.120delC | p.S40fs | . | . |
| XS1 | 6 | 31903708 | CT | C | 1 | NM_001282457 | C2 | frameshift_deleti  on | exon3 | c.121delT | p.F41fs | . | . |
| XS1 | 6 | 31915215 | GC | G | 0.189 | NM_001710 | CFB | frameshift_deleti  on | exon4 | c.576delC | p.S192fs | . | . |
| XS1 | 6 | 32713770 | CT | C | 0.247 | NM_020056 | HLA-DQA2 | frameshift_deleti  on | exon3 | c.535delT | p.F179fs | . | . |
| XS1 | 6 | 32917458 | TG | T | 1 | NM_006120 | HLA-DMA | frameshift_deleti  on | exon3 | c.581delC | p.P194fs | . | . |
| XS1 | 6 | 33273072 | TG | T | 0.545 | NM_172209 | TAPBP | frameshift_deleti  on | exon3 | c.300delC | p.P100fs | . | . |
| XS1 | 6 | 33282854 | C | CA | 0.5 | NM_001145338 | ZBTB22 | frameshift_inserti  on | exon2 | c.1839dupT | p.V614fs | . | . |

| XS1 | 6 | 34839648 | GC | G | 0.636 | NM_017754 | UHRF1BP1 | frameshift_deleti  on | exon20 | c.4144delC | p.P1382fs | . | . |
| --- | --- | --- | --- | --- | --- | --- | --- | --- | --- | --- | --- | --- | --- |
| XS1 | 6 | 35434094 | AG | A | 0.257 | NM_021922 | FANCE | frameshift_deleti  on | exon10 | c.1584delG | p.K528fs | . | . |
| XS1 | 6 | 36689226 | AC | A | 0.32 | NM_001257357 | RAB44 | frameshift_deleti  on | exon9 | c.1316delC | p.T439fs | . | . |
| XS1 | 6 | 36976620 | GC | G | 0.355 | NM_173558 | FGD2 | frameshift_deleti  on | exon2 | c.80delC | p.A27fs | . | . |
| XS1 | 6 | 38649832 | CT | C | 0.729 | NM_006708 | GLO1 | frameshift_deleti  on | exon5 | c.421delA | p.R141fs | . | . |
| XS1 | 6 | 38670755 | TG | T | 1 | NM_006708 | GLO1 | frameshift_deleti  on | exon1 | c.75delC | p.P25fs | . | . |
| XS1 | 6 | 41658829 | TTGC | T | 0.22 | NM_001167827 | TFEB | nonframeshift_de  letion | exon2 | c.162_164del | p.54_55del | . | . |
| XS1 | 6 | 42541534 | CA | C | 0.379 | NM_001184801 | UBR2 | frameshift_deleti  on | exon2 | c.142delA | p.K48fs | . | . |
| XS1 | 6 | 42977148 | T | TC | 0.471 | NM_180977 | PPP2R5D | frameshift_inserti  on | exon10 | c.1023dupC | p.F341fs | . | . |
| XS1 | 6 | 42993834 | CA | C | 0.636 | NM_033112 | RRP36 | frameshift_deleti  on | exon4 | c.406delA | p.K136fs | . | . |
| XS1 | 6 | 43008081 | CT | C | 0.833 | NM_001168370 | CUL7 | frameshift_deleti  on | exon22 | c.4358delA | p.E1453fs | . | . |
| XS1 | 6 | 43250849 | GA | G | 0.3 | NM_032538 | TTBK1 | frameshift_deleti  on | exon14 | c.2372delA | p.E791fs | . | . |
| XS1 | 6 | 43306738 | GCT | G | 0.5 | NM_014345 | ZNF318 | frameshift_deleti  on | exon10 | c.4996_4997del | p.S1666fs | . | . |
| XS1 | 6 | 43416705 | AC | A | 0.667 | NM_033450 | ABCC10 | frameshift_deleti  on | exon17 | c.3963delC | p.D1321fs | . | . |
| XS1 | 6 | 43593199 | GC | G | 0.885 | NM_001286216 | GTPBP2 | frameshift_deleti  on | exon5 | c.341delG | p.G114fs | . | . |
| XS1 | 6 | 44114585 | G | GA | 0.339 | NM_018426 | TMEM63B | frameshift_inserti  on | exon11 | c.785dupA | p.E262fs | . | . |
| XS1 | 6 | 44197165 | TG | T | 0.667 | NM_001078174 | SLC29A1 | frameshift_deleti  on | exon3 | c.69delG | p.L23fs | . | . |
| XS1 | 6 | 44200657 | GC | G | 0.364 | NM_001078174 | SLC29A1 | frameshift_deleti  on | exon12 | c.1174delC | p.H392fs | . | . |
| XS1 | 6 | 46714683 | A | AT | 0.234 | NM_001162435 | ANKRD66 | frameshift_inserti  on | exon1 | c.25_26insT | p.R9fs | . | . |
| XS1 | 6 | 49518597 | CT | C | 0.269 | NM_001145652 | C6orf141 | frameshift_deleti  on | exon1 | c.93delT | p.P31fs | . | . |
| XS1 | 6 | 49585866 | GT | G | 0.663 | NM_000324 | RHAG | frameshift_deleti  on | exon3 | c.406delA | p.T136fs | . | . |
| XS1 | 6 | 51915017 | CG | C | 1 | NM_138694 | PKHD1 | frameshift_deleti  on | exon22 | c.2216delC | p.P739fs | . | . |
| XS1 | 6 | 52142373 | AT | A | 1 | NM_001270472 | MCM3 | frameshift_deleti  on | exon6 | c.1022delA | p.N341fs | . | . |
| XS1 | 6 | 53519987 | GT | G | 1 | NM_001003760 | KLHL31 | frameshift_deleti  on | exon2 | c.83delA | p.N28fs | . | . |
| XS1 | 6 | 54791223 | G | GT | 0.8 | NM_001010872 | FAM83B | frameshift_inserti  on | exon3 | c.500dupT | p.V167fs | . | . |
| XS1 | 6 | 56327882 | CT | C | 0.25 | NM_015548 | DST | frameshift_deleti  on | exon82 | c.15090delA | p.K5030fs | . | . |
| XS1 | 6 | 56335075 | G | GA | 0.5 | NM_015548 | DST | frameshift_inserti  on | exon77 | c.14457dupT | p.P4820fs | . | . |
| XS1 | 6 | 56382083 | CA | C | 1 | NM_015548 | DST | stopgain | exon52 | c.10400delT | p.L3467X | . | . |
| XS1 | 6 | 56463418 | CT | C | 1 | NM_015548 | DST | frameshift_deleti on | exon27 | c.3914delA | p.K1305fs | . | . |
| XS1 | 6 | 56965825 | GT | G | 0.8 | NM_001257273 | ZNF451 | frameshift_deleti  on | exon4 | c.612delT | p.C204fs | . | . |
| XS1 | 6 | 66012921 | GT | G | 0.471 | NM_001271675 | LOC441155 | frameshift_deleti  on | exon2 | c.508delT | p.F170fs | . | . |
| XS1 | 6 | 69348775 | TA | T | 0.231 | NM_001704 | BAI3 | frameshift_deleti  on | exon3 | c.209delA | p.Y70fs | . | . |
| XS1 | 6 | 71212486 | AC | A | 0.381 | NM_001162529 | FAM135A | frameshift_deleti  on | exon10 | c.1022delC | p.T341fs | . | . |
| XS1 | 6 | 74117731 | TA | T | 1 | NM_018665 | DDX43 | frameshift_deleti  on | exon9 | c.1087delA | p.K363fs | . | . |
| XS1 | 6 | 76022394 | T | TC | 0.412 | NM_015687 | FILIP1 | frameshift_inserti  on | exon5 | c.3153dupG | p.K1052fs | . | . |
| XS1 | 6 | 76124483 | GT | G | 0.433 | NM_015687 | FILIP1 | frameshift_deleti  on | exon2 | c.205delA | p.T69fs | . | . |
| XS1 | 6 | 76604966 | CA | C | 0.375 | NM_004999 | MYO6 | frameshift_deleti  on | exon29 | c.3127delA | p.K1043fs | . | . |
| XS1 | 6 | 79577303 | CA | C | 0.353 | NM_001010844 | IRAK1BP1 | frameshift_deleti  on | exon1 | c.11delA | p.Q4fs | . | . |
| XS1 | 6 | 81053440 | AT | A | 0.562 | NM_000056 | BCKDHB | frameshift_deleti  on | exon10 | c.1099delT | p.F367fs | . | . |
| XS1 | 6 | 84366528 | GA | G | 0.36 | NM_001256718 | SNAP91 | frameshift_deleti  on | exon6 | c.245delT | p.F82fs | . | . |
| XS1 | 6 | 89907818 | GT | G | 0.483 | NM_001256703 | GABRR1 | frameshift_deleti  on | exon4 | c.441delA | p.K147fs | . | . |
| XS1 | 6 | 90097168 | GT | G | 0.192 | NM_021244 | RRAGD | frameshift_deleti  on | exon2 | c.289delA | p.T97fs | . | . |
| XS1 | 6 | 100957376 | GC | G | 0.5 | NM_006828 | ASCC3 | frameshift_deleti  on | exon42 | c.6494delG | p.C2165fs | . | . |
| XS1 | 6 | 102069829 | AT | A | 1 | NM_001166247 | GRIK2 | frameshift_deleti  on | exon2 | c.122delT | p.I41fs | . | . |
| XS1 | 6 | 109309800 | CT | C | 0.963 | NM_001199933 | SESN1 | frameshift_deleti  on | exon9 | c.1337delA | p.K446fs | . | . |
| XS1 | 6 | 109714127 | CT | C | 0.263 | NM_001286360 | PPIL6 | frameshift_deleti  on | exon7 | c.741delA | p.E247fs | . | . |
| XS1 | 6 | 109885301 | CT | C | 0.8 | NM_001145128 | AK9 | frameshift_deleti  on | exon23 | c.2616delA | p.K872fs | . | . |
| XS1 | 6 | 110644040 | TG | T | 0.207 | NM_001123364 | METTL24 | frameshift_deleti  on | exon2 | c.353delC | p.A118fs | . | . |
| XS1 | 6 | 111696533 | C | CT | 0.3 | NM_002912 | REV3L | frameshift_inserti  on | exon14 | c.3024dupA | p.E1009fs | . | . |
| XS1 | 6 | 111697437 | GA | G | 0.308 | NM_002912 | REV3L | frameshift_deleti  on | exon14 | c.2120delT | p.F707fs | . | . |
| XS1 | 6 | 111697938 | ATT | A | 0.17 | NM_002912 | REV3L | frameshift_deleti  on | exon14 | c.1618_1619del | p.N540fs | . | . |
| XS1 | 6 | 114379112 | ACT | A | 0.683 | NM_153612 | HS3ST5 | frameshift_deleti  on | exon2 | c.348_349del | p.V116fs | . | . |
| XS1 | 6 | 116600216 | TC | T | 0.316 | NM_003309 | TSPYL1 | frameshift_deleti  on | exon1 | c.777delG | p.R259fs | . | . |
| XS1 | 6 | 116600465 | T | TCAC | 1 | NM_003309 | TSPYL1 | nonframeshift_in  sertion | exon1 | c.528_529insGT  G | p.K177delinsVK | . | . |
| XS1 | 6 | 117662376 | TG | T | 0.75 | NM_002944 | ROS1 | frameshift_deleti  on | exon30 | c.5000delC | p.P1667fs | . | . |
| XS1 | 6 | 117709105 | CT | C | 0.75 | NM_002944 | ROS1 | frameshift_deleti  on | exon13 | c.1851delA | p.Q617fs | . | . |
| XS1 | 6 | 117864342 | TG | T | 0.373 | NM_173674 | DCBLD1 | frameshift_deleti  on | exon12 | c.1414delG | p.A472fs | . | . |
| XS1 | 6 | 126203598 | GA | G | 0.432 | NM_001199621 | NCOA7 | frameshift_deleti  on | exon6 | c.289delA | p.K97fs | . | . |

| XS1 | 6 | 126203626 | AC | A | 0.269 | NM_001199621 | NCOA7 | frameshift_deleti  on | exon6 | c.317delC | p.T106fs | . | . |
| --- | --- | --- | --- | --- | --- | --- | --- | --- | --- | --- | --- | --- | --- |
| XS1 | 6 | 128410923 | AG | A | 0.25 | NM_001135648 | PTPRK | frameshift_deleti  on | exon8 | c.1376delC | p.P459fs | . | . |
| XS1 | 6 | 130762692 | TG | T | 0.29 | NM_001258276 | TMEM200A | frameshift_deleti  on | exon2 | c.1126delG | p.G376fs | . | . |
| XS1 | 6 | 135507123 | CG | C | 0.463 | NM_001130172 | MYB | frameshift_deleti  on | exon2 | c.107delG | p.R36fs | . | . |
| XS1 | 6 | 135763835 | GTT | G | 0.286 | NM_001134830 | AHI1 | frameshift_deleti  on | exon12 | c.1795_1796del | p.N599fs | . | . |
| XS1 | 6 | 138199856 | CGG | C | 0.139 | NM_001270507 | TNFAIP3 | frameshift_deleti  on | exon7 | c.1275_1276del | p.P425fs | . | . |
| XS1 | 6 | 138608215 | TGCTGCT  AACTGC GCCTCA GCCCTT GCCCAG | T | 0.714 | NM_020340 | KIAA1244 | nonframeshift_de letion | exon17 | c.2791_2826del | p.931_942del | . | . |
| XS1 | 6 | 146125508 | CT | C | 0.296 | NM_032145 | FBXO30 | frameshift_deleti  on | exon2 | c.2033delA | p.K678fs | . | . |
| XS1 | 6 | 146215287 | TG | T | 0.833 | NM_001042683 | SHPRH | frameshift_deleti  on | exon27 | c.4693delC | p.Q1565fs | . | . |
| XS1 | 6 | 146625788 | AGGT | A | 0.141 | NM_001278064 | GRM1 | nonframeshift_de  letion | exon3 | c.993_995del | p.331_332del | . | . |
| XS1 | 6 | 146625801 | CG | C | 0.452 | NM_001278064 | GRM1 | frameshift_deleti  on | exon3 | c.1006delG | p.G336fs | . | . |
| XS1 | 6 | 146625952 | GA | G | 0.288 | NM_001278064 | GRM1 | frameshift_deleti  on | exon3 | c.1157delA | p.E386fs | . | . |
| XS1 | 6 | 147062372 | CA | C | 0.5 | NM_024694 | ADGB | frameshift_deleti  on | exon25 | c.3143delA | p.Q1048fs | . | . |
| XS1 | 6 | 150174230 | AC | A | 0.929 | NM_032832 | LRP11 | frameshift_deleti  on | exon2 | c.679delG | p.V227fs | . | . |
| XS1 | 6 | 151673415 | GA | G | 0.5 | NM_144497 | AKAP12 | frameshift_deleti  on | exon2 | c.3596delA | p.E1199fs | . | . |
| XS1 | 6 | 152265375 | CA | C | 0.8 | NM_000125 | ESR1 | frameshift_deleti  on | exon4 | c.829delA | p.R277fs | . | . |
| XS1 | 6 | 152652222 | A | AT | 0.588 | NM_033071 | SYNE1 | frameshift_inserti  on | exon77 | c.13384_13385i  nsA | p.L4462fs | . | . |
| XS1 | 6 | 158507940 | CG | C | 0.883 | NM_001178088 | SYNJ2 | frameshift_deleti  on | exon22 | c.2552delG | p.R851fs | . | . |
| XS1 | 6 | 158516743 | G | GC | 1 | NM_001178088 | SYNJ2 | frameshift_inserti  on | exon26 | c.3128dupC | p.A1043fs | . | . |
| XS1 | 6 | 160211644 | GGGTT | G | 0.583 | NM_014161 | MRPL18 | frameshift_deleti  on | exon1 | c.26_29del | p.G9fs | . | . |
| XS1 | 6 | 160211645 | GGTT | G | 0.667 | NM_014161 | MRPL18 | nonframeshift_de  letion | exon1 | c.27_29del | p.9_10del | . | . |
| XS1 | 6 | 160469420 | GA | G | 0.778 | NM_000876 | IGF2R | frameshift_deleti  on | exon18 | c.2360delA | p.E787fs | . | . |
| XS1 | 6 | 160510279 | AT | A | 0.394 | NM_000876 | IGF2R | frameshift_deleti  on | exon43 | c.6462delT | p.Y2154fs | . | . |
| XS1 | 6 | 160858206 | AG | A | 0.429 | NM_021977 | SLC22A3 | frameshift_deleti  on | exon7 | c.1252delG | p.G418fs | . | . |
| XS1 | 6 | 160969539 | A | AC | 0.333 | NM_005577 | LPA | frameshift_inserti  on | exon32 | c.5125dupG | p.V1709fs | . | . |
| XS1 | 6 | 161152881 | AT | A | 0.282 | NM_000301 | PLG | frameshift_deleti  on | exon12 | c.1544delT | p.I515fs | . | . |
| XS1 | 6 | 161557583 | GT | G | 1 | NM_020133 | AGPAT4 | frameshift_deleti  on | exon9 | c.1125delA | p.K375fs | . | . |
| XS1 | 6 | 162864356 | CA | C | 0.429 | NM_004562 | PARK2 | frameshift_deleti  on | exon2 | c.156delT | p.N52fs | . | . |
| XS1 | 6 | 167754899 | TG | T | 0.286 | NM_031949 | TTLL2 | frameshift_deleti  on | exon3 | c.1512delG | p.M504fs | . | . |
| XS1 | 6 | 170089035 | AT | A | 0.529 | NM_001202550 | WDR27 | frameshift_deleti  on | exon2 | c.66delA | p.K22fs | . | . |
| XS1 | 6 | 170871013 | A | ACAG | 0.964 | NM_001172085 | TBP | nonframeshift_in  sertion | exon2 | c.129_130insCA  G | p.Q43delinsQQ | . | . |
| XS1 | 6 | 170871037 | GCAA | G | 0.864 | NM_001172085 | TBP | nonframeshift_de  letion | exon2 | c.154_156del | p.52_52del | . | . |
| XS1 | 6 | 170871046 | ACAGCA  GCAG | A | 1 | NM_001172085 | TBP | nonframeshift_de  letion | exon2 | c.163_171del | p.55_57del | . | . |
| XS1 | 6 | 170871046 | ACAG | A | 1 | NM_001172085 | TBP | nonframeshift_de  letion | exon2 | c.163_165del | p.55_55del | . | . |
| XS1 | 7 | 1097771 | TG | T | 0.545 | NM_138445 | GPR146 | frameshift_deleti  on | exon1 | c.621delG | p.L207fs | . | . |
| XS1 | 7 | 2581825 | TG | T | 0.26 | NM_152743 | BRAT1 | frameshift_deleti  on | exon7 | c.943delC | p.Q315fs | . | . |
| XS1 | 7 | 2617947 | C | CA | 0.174 | NM_001287501 | IQCE | frameshift_inserti  on | exon5 | c.343dupA | p.S114fs | . | . |
| XS1 | 7 | 4900104 | CT | C | 0.719 | NM_020144 | PAPOLB | frameshift_deleti  on | exon1 | c.1337delA | p.K446fs | . | . |
| XS1 | 7 | 6075690 | C | CA | 0.458 | NM_001271700 | ANKRD61 | frameshift_inserti  on | exon3 | c.930_931insA | p.N310fs | . | . |
| XS1 | 7 | 6193494 | GC | G | 0.444 | NM_032172 | USP42 | frameshift_deleti  on | exon15 | c.2310delC | p.S770fs | . | . |
| XS1 | 7 | 7412878 | TG | T | 0.963 | NM_001037763 | COL28A1 | frameshift_deleti  on | exon32 | c.2658delC | p.D886fs | . | . |
| XS1 | 7 | 8791038 | A | AT | 0.267 | NM_152745 | NXPH1 | frameshift_inserti  on | exon3 | c.456dupT | p.Y152fs | . | . |
| XS1 | 7 | 15725797 | ATGG | A | 1 | NM_005924 | MEOX2 | nonframeshift_de  letion | exon1 | c.228_230del | p.76_77del | . | . |
| XS1 | 7 | 17861184 | GT | G | 0.931 | NM_015132 | SNX13 | frameshift_deleti  on | exon18 | c.1792delA | p.T598fs | . | . |
| XS1 | 7 | 17915406 | CT | C | 0.625 | NM_015132 | SNX13 | frameshift_deleti  on | exon6 | c.447delA | p.K149fs | . | . |
| XS1 | 7 | 21947779 | CT | C | 0.276 | NM_001127371 | CDCA7L | frameshift_deleti  on | exon3 | c.511delA | p.R171fs | . | . |
| XS1 | 7 | 22175721 | GC | G | 0.667 | NM_012294 | RAPGEF5 | frameshift_deleti  on | exon23 | c.1891delG | p.A631fs | . | . |
| XS1 | 7 | 22478258 | G | GA | 0.448 | NM_001164460 | STEAP1B | frameshift_inserti  on | exon5 | c.878dupT | p.F293fs | . | . |
| XS1 | 7 | 22532299 | A | AT | 0.364 | NM_001164460 | STEAP1B | frameshift_inserti  on | exon4 | c.646dupA | p.M216fs | . | . |
| XS1 | 7 | 23358838 | T | TC | 0.692 | NM_006547 | IGF2BP3 | frameshift_inserti  on | exon11 | c.1238_1239ins  G | p.P413fs | . | . |
| XS1 | 7 | 27135316 | A | ATGG | 0.75 | NM_005522 | HOXA1 | nonframeshift_in  sertion | exon1 | c.215_216insCC  A | p.H72delinsHH | . | . |
| XS1 | 7 | 27135338 | T | TGGA | 0.575 | NM_005522 | HOXA1 | nonframeshift_in  sertion | exon1 | c.193_194insTC  C | p.H65delinsLH | . | . |
| XS1 | 7 | 27135340 | G | GTGT | 0.553 | NM_005522 | HOXA1 | nonframeshift_in  sertion | exon1 | c.191_192insAC  A | p.P64delinsPH | . | . |
| XS1 | 7 | 27672031 | C | CT | 0.722 | NM_152740 | HIBADH | frameshift_inserti  on | exon3 | c.285dupA | p.A96fs | . | . |
| XS1 | 7 | 30085865 | CT | C | 0.162 | NM_001197026 | PLEKHA8 | frameshift_deleti  on | exon3 | c.198delT | p.P66fs | . | . |

| XS1 | 7 | 30363279 | CA | C | 0.833 | NM_147128 | ZNRF2 | frameshift_deleti  on | exon2 | c.492delA | p.S164fs | . | . |
| --- | --- | --- | --- | --- | --- | --- | --- | --- | --- | --- | --- | --- | --- |
| XS1 | 7 | 30638425 | GA | G | 0.44 | NM_002047 | GARS | frameshift_deleti  on | exon2 | c.237delA | p.R79fs | . | . |
| XS1 | 7 | 30721590 | CCGG | C | 0.185 | NM_001202482 | CRHR2 | nonframeshift_de  letion | exon2 | c.167_169del | p.56_57del | . | . |
| XS1 | 7 | 38256805 | CT | C | 0.227 | NM_032016 | STARD3NL | frameshift_deleti  on | exon6 | c.453delT | p.A151fs | . | . |
| XS1 | 7 | 40027367 | GC | G | 0.256 | NM_003718 | CDK13 | frameshift_deleti  on | exon2 | c.1382delC | p.A461fs | . | . |
| XS1 | 7 | 40127818 | AG | A | 1 | NM_003718 | CDK13 | frameshift_deleti  on | exon12 | c.3124delG | p.A1042fs | . | . |
| XS1 | 7 | 42966260 | AT | A | 1 | NM_002787 | PSMA2 | frameshift_deleti  on | exon3 | c.125delA | p.N42fs | . | . |
| XS1 | 7 | 43594364 | TG | T | 0.727 | NM_001287059 | HECW1 | frameshift_deleti  on | exon27 | c.4583delG | p.W1528fs | . | . |
| XS1 | 7 | 43917268 | GC | G | 0.438 | NM_017920 | URGCP | frameshift_deleti  on | exon5 | c.1766delG | p.G589fs | . | . |
| XS1 | 7 | 44147093 | A | AC | 0.292 | NM_001129 | AEBP1 | frameshift_inserti  on | exon3 | c.652dupC | p.A217fs | . | . |
| XS1 | 7 | 44147506 | GC | G | 0.337 | NM_001129 | AEBP1 | frameshift_deleti  on | exon5 | c.839delC | p.A280fs | . | . |
| XS1 | 7 | 44150637 | GC | G | 0.368 | NM_001129 | AEBP1 | frameshift_deleti  on | exon13 | c.1612delC | p.L538fs | . | . |
| XS1 | 7 | 44798962 | CT | C | 0.667 | NM_174929 | ZMIZ2 | frameshift_deleti  on | exon6 | c.897delT | p.P299fs | . | . |
| XS1 | 7 | 45142988 | G | GA | 0.471 | NM_030900 | TBRG4 | frameshift_inserti  on | exon4 | c.789dupT | p.L264fs | . | . |
| XS1 | 7 | 45148730 | T | TAG | 0.116 | NM_001261834 | TBRG4 | frameshift_inserti  on | exon2 | c.139_140insCT | p.H47fs | . | . |
| XS1 | 7 | 45753450 | GA | G | 0.414 | NM_021116 | ADCY1 | frameshift_deleti  on | exon20 | c.3217delA | p.K1073fs | . | . |
| XS1 | 7 | 48081096 | CG | C | 0.667 | NM_001100159 | C7orf57 | frameshift_deleti  on | exon3 | c.222delG | p.A74fs | . | . |
| XS1 | 7 | 51098567 | GTCT | G | 0.429 | NM_015198 | COBL | nonframeshift_de  letion | exon9 | c.1443_1445del | p.481_482del | . | . |
| XS1 | 7 | 55540608 | GGGC | G | 0.273 | NM_001284282 | VOPP1 | nonframeshift_de  letion | exon5 | c.405_407del | p.135_136del | . | . |
| XS1 | 7 | 56045919 | CT | C | 0.235 | NM_001202469 | GBAS | frameshift_deleti  on | exon2 | c.194delT | p.L65fs | . | . |
| XS1 | 7 | 64168983 | GA | G | 0.348 | NM_001282360 | ZNF107 | frameshift_deleti  on | exon3 | c.2413delA | p.K805fs | . | . |
| XS1 | 7 | 65419353 | TTTA | T | 0.342 | NM_001284342 | VKORC1L1 | nonframeshift_de  letion | exon2 | c.488_490del | p.163_164del | . | . |
| XS1 | 7 | 66104041 | C | CT | 0.5 | NM_001167961 | KCTD7 | frameshift_inserti  on | exon4 | c.693dupT | p.S231fs | . | . |
| XS1 | 7 | 72873909 | GT | G | 0.409 | NM_032408 | BAZ1B | frameshift_deleti  on | exon13 | c.3388delA | p.T1130fs | . | . |
| XS1 | 7 | 73651704 | GT | G | 0.4 | NM_001278792 | RFC2 | frameshift_deleti  on | exon8 | c.518delA | p.D173fs | . | . |
| XS1 | 7 | 73950604 | CG | C | 0.75 | NM_001199207 | GTF2IRD1 | frameshift_deleti  on | exon11 | c.1505delG | p.R502fs | . | . |
| XS1 | 7 | 76240785 | CA | C | 0.8 | NM_012230 | POMZP3 | frameshift_deleti  on | exon6 | c.560delT | p.L187fs | . | . |
| XS1 | 7 | 77394940 | AT | A | 0.303 | NM_198467 | RSBN1L | frameshift_deleti  on | exon4 | c.1474delT | p.F492fs | . | . |
| XS1 | 7 | 77538115 | GC | G | 0.872 | NM_020432 | PHTF2 | frameshift_deleti  on | exon5 | c.338delC | p.A113fs | . | . |
| XS1 | 7 | 77762255 | CG | C | 0.289 | NM_012301 | MAGI2 | frameshift_deleti  on | exon18 | c.3153delC | p.I1051fs | . | . |
| XS1 | 7 | 77762256 | GA | G | 0.24 | NM_012301 | MAGI2 | frameshift_deleti  on | exon18 | c.3152delT | p.I1051fs | . | . |
| XS1 | 7 | 77824196 | C | CT | 0.298 | NM_012301 | MAGI2 | frameshift_inserti  on | exon12 | c.2263dupA | p.S755fs | . | . |
| XS1 | 7 | 82581488 | T | TTCA | 1 | NM_014510 | PCLO | nonframeshift_in  sertion | exon5 | c.8780_8781ins  TGA | p.E2927delinsD  E | . | . |
| XS1 | 7 | 82785621 | T | TA | 0.297 | NM_014510 | PCLO | frameshift_inserti  on | exon2 | c.335_336insT | p.K112fs | . | . |
| XS1 | 7 | 83029419 | AT | A | 0.733 | NM_001178129 | SEMA3E | frameshift_deleti  on | exon11 | c.1110delA | p.K370fs | . | . |
| XS1 | 7 | 95442569 | TA | T | 0.625 | NM_001135556 | DYNC1I1 | frameshift_deleti  on | exon4 | c.235delA | p.M79fs | . | . |
| XS1 | 7 | 96747051 | GTTT | G | 0.632 | NM_020186 | ACN9 | nonframeshift_de  letion | exon1 | c.17_19del | p.6_7del | . | . |
| XS1 | 7 | 96747104 | GC | G | 0.562 | NM_020186 | ACN9 | frameshift_deleti  on | exon1 | c.70delC | p.P24fs | . | . |
| XS1 | 7 | 98601839 | AT | A | 0.571 | NM_003496 | TRRAP | frameshift_deleti  on | exon66 | c.10208delT | p.M3403fs | . | . |
| XS1 | 7 | 98649873 | TC | T | 0.5 | NM_001199847 | SMURF1 | frameshift_deleti  on | exon7 | c.675delG | p.Q225fs | . | . |
| XS1 | 7 | 99129833 | TC | T | 0.186 | NM_014569 | ZKSCAN5 | frameshift_deleti  on | exon7 | c.2482delC | p.P828fs | . | . |
| XS1 | 7 | 99377639 | CA | C | 0.4 | NM_001202855 | CYP3A4 | frameshift_deleti  on | exon2 | c.140delT | p.L47fs | . | . |
| XS1 | 7 | 99669746 | A | AG | 0.222 | NM_001278291 | ZNF3 | frameshift_inserti  on | exon4 | c.252_253insC | p.S85fs | . | . |
| XS1 | 7 | 99711255 | C | CT | 0.154 | NM_001190415 | TAF6 | frameshift_inserti  on | exon4 | c.491_492insA | p.L164fs | . | . |
| XS1 | 7 | 99817847 | TGG | T | 0.683 | NM_024070 | PVRIG | frameshift_deleti  on | exon3 | c.230_231del | p.W77fs | . | . |
| XS1 | 7 | 99987721 | GA | G | 0.261 | NM_013439 | PILRA | frameshift_deleti  on | exon3 | c.666delA | p.R222fs | . | . |
| XS1 | 7 | 100682388 | TG | T | 1 | NM_001040105 | MUC17 | frameshift_deleti  on | exon3 | c.7692delG | p.M2564fs | . | . |
| XS1 | 7 | 101870901 | TA | T | 0.538 | NM_001202543 | CUX1 | frameshift_deleti  on | exon21 | c.3419delA | p.Y1140fs | . | . |
| XS1 | 7 | 101923405 | GC | G | 1 | NM_001202544 | CUX1 | frameshift_deleti  on | exon18 | c.1710delC | p.S570fs | . | . |
| XS1 | 7 | 102389980 | A | AT | 0.292 | NM_001145268 | FAM185A | frameshift_inserti  on | exon1 | c.326_327insT | p.E109fs | . | . |
| XS1 | 7 | 103338478 | TC | T | 0.857 | NM_005045 | RELN | frameshift_deleti  on | exon10 | c.964delG | p.E322fs | . | . |
| XS1 | 7 | 103841274 | T | TA | 0.194 | NM_002553 | ORC5 | stopgain | exon3 | c.297dupT | p.N100_D101d  elinsX | . | . |
| XS1 | 7 | 104703901 | TTGA | T | 0.25 | NM_018682 | KMT2E | nonframeshift_de  letion | exon4 | c.291_293del | p.97_98del | . | . |
| XS1 | 7 | 105254801 | AGAG | A | 0.154 | NM_138495 | ATXN7L1 | nonframeshift_de  letion | exon8 | c.1605_1607del | p.535_536del | . | . |
| XS1 | 7 | 107350510 | GA | G | 0.327 | NM_000441 | SLC26A4 | frameshift_deleti  on | exon19 | c.2102delA | p.E701fs | . | . |
| XS1 | 7 | 107408042 | T | TA | 1 | NM_000111 | SLC26A3 | frameshift_inserti  on | exon20 | c.2252dupT | p.L751fs | . | . |
| XS1 | 7 | 107542831 | CT | C | 0.4 | NM_000108 | DLD | frameshift_deleti  on | exon4 | c.261delT | p.P87fs | . | . |

| XS1 | 7 | 107618579 | TG | T | 0.538 | NM_002291 | LAMB1 | frameshift_deleti  on | exon9 | c.912delC | p.T304fs | . | . |
| --- | --- | --- | --- | --- | --- | --- | --- | --- | --- | --- | --- | --- | --- |
| XS1 | 7 | 112098999 | AT | A | 0.494 | NM_001197079 | IFRD1 | frameshift_deleti  on | exon5 | c.344delT | p.I115fs | . | . |
| XS1 | 7 | 120607607 | AC | A | 0.864 | NM_019071 | ING3 | frameshift_deleti  on | exon7 | c.462delC | p.H154fs | . | . |
| XS1 | 7 | 123302915 | CCCT | C | 0.263 | NM_207163 | LMOD2 | nonframeshift_de  letion | exon2 | c.1276_1278del | p.426_426del | . | . |
| XS1 | 7 | 127222177 | T | TG | 0.3 | NM_024523 | GCC1 | frameshift_inserti  on | exon2 | c.2218dupC | p.Q740fs | . | . |
| XS1 | 7 | 128489593 | CGG | C | 0.891 | NM_001127487 | FLNC | frameshift_deleti  on | exon30 | c.5161_5162del | p.G1721fs | . | . |
| XS1 | 7 | 128587351 | CACTCT  GCAGCC GCCCAC TCTGCG | C | 1 | NM_001098627 | IRF5 | nonframeshift_de letion | exon6 | c.502_531del | p.168_177del | . | . |
| XS1 | 7 | 128587448 | CT | C | 0.714 | NM_001098627 | IRF5 | frameshift_deleti  on | exon6 | c.599delT | p.L200fs | . | . |
| XS1 | 7 | 129062749 | TG | T | 0.371 | NM_001130720 | AHCYL2 | frameshift_deleti  on | exon13 | c.1528delG | p.D510fs | . | . |
| XS1 | 7 | 129824032 | AT | A | 1 | NM_032842 | TMEM209 | frameshift_deleti  on | exon8 | c.1014delA | p.K338fs | . | . |
| XS1 | 7 | 129999472 | C | CA | 0.188 | NM_001127442 | CPA5 | frameshift_inserti  on | exon5 | c.376_377insA | p.R126fs | . | . |
| XS1 | 7 | 135330233 | C | CA | 0.3 | NM_015135 | NUP205 | frameshift_inserti  on | exon41 | c.5701_5702ins  A | p.L1901fs | . | . |
| XS1 | 7 | 137339507 | G | GA | 0.636 | NM_004717 | DGKI | frameshift_inserti  on | exon5 | c.708dupT | p.R237fs | . | . |
| XS1 | 7 | 137612913 | CTGG | C | 1 | NM_001253775 | CREB3L2 | nonframeshift_de  letion | exon2 | c.299_301del | p.100_101del | . | . |
| XS1 | 7 | 138794021 | GC | G | 0.576 | NM_020119 | ZC3HAV1 | frameshift_deleti  on | exon1 | c.56delG | p.G19fs | . | . |
| XS1 | 7 | 138969183 | CT | C | 0.5 | NM_173569 | UBN2 | frameshift_deleti  on | exon15 | c.3533delT | p.L1178fs | . | . |
| XS1 | 7 | 139813339 | CT | C | 0.438 | NM_030647 | KDM7A | frameshift_deleti  on | exon10 | c.1289delA | p.Q430fs | . | . |
| XS1 | 7 | 140107462 | TCAGC | T | 0.526 | NM_001008749 | RAB19 | frameshift_deleti  on | exon2 | c.17_20del | p.S6fs | . | . |
| XS1 | 7 | 142458418 | TTGA | T | 0.214 | NM_002769 | PRSS1 | nonframeshift_de  letion | exon2 | c.54_56del | p.18_19del | . | . |
| XS1 | 7 | 142612695 | TC | T | 0.269 | NM_019841 | TRPV5 | frameshift_deleti  on | exon9 | c.1165delG | p.E389fs | . | . |
| XS1 | 7 | 144095647 | TC | T | 0.417 | NM_001080413 | NOBOX | frameshift_deleti  on | exon9 | c.1501delG | p.E501fs | . | . |
| XS1 | 7 | 148506446 | AT | A | 0.308 | NM_001203249 | EZH2 | frameshift_deleti  on | exon17 | c.1897delA | p.I633fs | . | . |
| XS1 | 7 | 148718187 | TTCC | T | 0.118 | NM_004911 | PDIA4 | nonframeshift_de  letion | exon2 | c.138_140del | p.46_47del | . | . |
| XS1 | 7 | 149481076 | T | TA | 0.583 | NM_198455 | SSPO | frameshift_inserti  on | exon18 | c.2558_2559ins  A | p.L853fs | . | . |
| XS1 | 7 | 149506211 | G | GC | 1 | NM_198455 | SSPO | frameshift_inserti  on | exon63 | c.9204dupC | p.S3068fs | . | . |
| XS1 | 7 | 151135157 | CT | C | 0.989 | NM_144727 | CRYGN | frameshift_deleti  on | exon2 | c.194delA | p.E65fs | . | . |
| XS1 | 7 | 151668075 | TA | T | 0.7 | NM_145292 | GALNTL5 | frameshift_deleti  on | exon3 | c.294delA | p.L98fs | . | . |
| XS1 | 7 | 151835887 | TC | T | 0.576 | NM_170606 | KMT2C | frameshift_deleti  on | exon58 | c.14636delG | p.G4879fs | . | . |
| XS1 | 7 | 154752604 | TA | T | 0.762 | NM_007349 | PAXIP1 | stopgain | exon12 | c.2432delT | p.L811X | . | . |
| XS1 | 7 | 154760540 | TTGC | T | 0.13 | NM_007349 | PAXIP1 | nonframeshift_de letion | exon7 | c.1368_1370del | p.456_457del | . | . |
| XS1 | 7 | 156976596 | TG | T | 0.237 | NM_014671 | UBE3C | frameshift_deleti  on | exon9 | c.1017delG | p.L339fs | . | . |
| XS1 | 8 | 1808092 | AC | A | 0.707 | NM_014629 | ARHGEF10 | frameshift_deleti  on | exon4 | c.224delC | p.T75fs | . | . |
| XS1 | 8 | 6728287 | TC | T | 0.18 | NM_005218 | DEFB1 | frameshift_deleti  on | exon2 | c.122delG | p.G41fs | . | . |
| XS1 | 8 | 8234868 | C | CGCCG  CT | 1 | NM_001080826 | SGK223 | nonframeshift_in  sertion | exon2 | c.1050_1051ins  AGCGGC | p.A351delinsS  GA | . | . |
| XS1 | 8 | 8234884 | G | GCTGC  CA | 0.857 | NM_001080826 | SGK223 | nonframeshift_in  sertion | exon2 | c.1034_1035ins  TGGCAG | p.S345delinsSG  S | . | . |
| XS1 | 8 | 8235288 | G | GT | 0.357 | NM_001080826 | SGK223 | frameshift_inserti  on | exon2 | c.630dupA | p.L211fs | . | . |
| XS1 | 8 | 10389054 | TG | T | 1 | NM_001197020 | PRSS55 | frameshift_deleti  on | exon3 | c.598delG | p.A200fs | . | . |
| XS1 | 8 | 11666218 | GTCCCA  C | G | 1 | NM_001287750 | FDFT1 | nonframeshift_de  letion | exon1 | c.193_198del | p.65_66del | . | . |
| XS1 | 8 | 11705329 | AC | A | 0.213 | NM_001908 | CTSB | frameshift_deleti  on | exon7 | c.534delG | p.G178fs | . | . |
| XS1 | 8 | 11994859 | A | AT | 0.6 | NM_201402 | USP17L2 | frameshift_inserti  on | exon1 | c.1410dupA | p.Y471fs | . | . |
| XS1 | 8 | 11995733 | GC | G | 0.3 | NM_201402 | USP17L2 | frameshift_deleti  on | exon1 | c.536delG | p.G179fs | . | . |
| XS1 | 8 | 17092231 | A | AT | 0.752 | NM_013354 | CNOT7 | frameshift_inserti  on | exon5 | c.611dupA | p.N204fs | . | . |
| XS1 | 8 | 17170867 | A | AC | 0.333 | NM_004686 | MTMR7 | frameshift_inserti  on | exon8 | c.908dupG | p.G303fs | . | . |
| XS1 | 8 | 18793594 | GC | G | 0.214 | NM_015310 | PSD3 | frameshift_deleti  on | exon2 | c.79delG | p.A27fs | . | . |
| XS1 | 8 | 21840257 | CT | C | 0.34 | NM_015024 | XPO7 | frameshift_deleti  on | exon11 | c.1212delT | p.T404fs | . | . |
| XS1 | 8 | 21953856 | AC | A | 0.857 | NM_022749 | FAM160B2 | frameshift_deleti  on | exon3 | c.134delC | p.T45fs | . | . |
| XS1 | 8 | 22167511 | T | TA | 0.758 | NM_001135721 | PIWIL2 | frameshift_inserti  on | exon15 | c.1725dupA | p.L575fs | . | . |
| XS1 | 8 | 23004526 | CT | C | 0.316 | NM_003840 | TNFRSF10D | frameshift_deleti  on | exon4 | c.429delA | p.K143fs | . | . |
| XS1 | 8 | 24251590 | TG | T | 0.5 | NM_001145272 | ADAMDEC1 | frameshift_deleti  on | exon3 | c.57delG | p.L19fs | . | . |
| XS1 | 8 | 24811071 | GC | G | 1 | NM_006158 | NEFL | frameshift_deleti  on | exon3 | c.1407delG | p.E469fs | . | . |
| XS1 | 8 | 25365098 | T | TG | 0.3 | NM_152562 | CDCA2 | frameshift_inserti  on | exon15 | c.2916_2917ins  G | p.G972fs | . | . |
| XS1 | 8 | 26212130 | GT | G | 0.579 | NM_001177591 | PPP2R2A | frameshift_deleti  on | exon4 | c.358delT | p.F120fs | . | . |
| XS1 | 8 | 27887915 | GCC | G | 0.175 | NM_001010906 | NUGGC | frameshift_deleti  on | exon16 | c.1927_1928del | p.G643fs | . | . |
| XS1 | 8 | 30539451 | CGT | C | 0.125 | NM_001195104 | GSR | frameshift_deleti  on | exon9 | c.1033_1034del | p.T345fs | . | . |
| XS1 | 8 | 30546776 | CG | C | 0.9 | NM_001195102 | GSR | frameshift_deleti  on | exon8 | c.855delC | p.P285fs | . | . |
| XS1 | 8 | 30703447 | TA | T | 0.451 | NM_031271 | TEX15 | frameshift_deleti  on | exon1 | c.3086delT | p.L1029fs | . | . |

| XS1 | 8 | 35579735 | G | GA | 0.5 | NM_080872 | UNC5D | frameshift_inserti  on | exon9 | c.1126dupA | p.E375fs | . | . |
| --- | --- | --- | --- | --- | --- | --- | --- | --- | --- | --- | --- | --- | --- |
| XS1 | 8 | 37888095 | TC | T | 0.5 | NM_004095 | EIF4EBP1 | frameshift_deleti  on | exon1 | c.5delC | p.S2fs | . | . |
| XS1 | 8 | 41521257 | TC | T | 0.483 | NM_001142445 | ANK1 | frameshift_deleti  on | exon2 | c.222delG | p.G74fs | . | . |
| XS1 | 8 | 41790673 | GC | G | 0.227 | NM_006766 | KAT6A | frameshift_deleti  on | exon17 | c.5064delG | p.Q1688fs | . | . |
| XS1 | 8 | 42233262 | AC | A | 0.333 | NM_014420 | DKK4 | frameshift_deleti  on | exon2 | c.197delG | p.C66fs | . | . |
| XS1 | 8 | 43037308 | A | AT | 0.196 | NM_152419 | HGSNAT | frameshift_inserti  on | exon11 | c.1034dupT | p.I345fs | . | . |
| XS1 | 8 | 48647991 | AG | A | 0.349 | NM_001282916 | SPIDR | frameshift_deleti  on | exon19 | c.2518delG | p.G840fs | . | . |
| XS1 | 8 | 52384844 | CA | C | 0.824 | NM_144651 | PXDNL | frameshift_deleti  on | exon8 | c.714delT | p.F238fs | . | . |
| XS1 | 8 | 54142003 | GA | G | 0.51 | NM_000912 | OPRK1 | frameshift_deleti  on | exon4 | c.996delT | p.F332fs | . | . |
| XS1 | 8 | 54906281 | CT | C | 0.263 | NM_201437 | TCEA1 | frameshift_deleti  on | exon3 | c.203delA | p.K68fs | . | . |
| XS1 | 8 | 55537701 | GC | G | 0.395 | NM_006269 | RP1 | stopgain | exon4 | c.1260delC | p.C420X | . | . |
| XS1 | 8 | 55538759 | C | CT | 0.516 | NM_006269 | RP1 | frameshift_inserti on | exon4 | c.2318dupT | p.L773fs | . | . |
| XS1 | 8 | 55538924 | CA | C | 0.558 | NM_006269 | RP1 | frameshift_deleti  on | exon4 | c.2483delA | p.Q828fs | . | . |
| XS1 | 8 | 55540115 | GA | G | 0.852 | NM_006269 | RP1 | frameshift_deleti  on | exon4 | c.3674delA | p.E1225fs | . | . |
| XS1 | 8 | 55540698 | AT | A | 0.654 | NM_006269 | RP1 | frameshift_deleti  on | exon4 | c.4257delT | p.D1419fs | . | . |
| XS1 | 8 | 59536318 | CA | C | 0.314 | NM_001144772 | NSMAF | frameshift_deleti  on | exon7 | c.498delT | p.F166fs | . | . |
| XS1 | 8 | 61766980 | TG | T | 0.2 | NM_017780 | CHD7 | frameshift_deleti  on | exon32 | c.6835delG | p.A2279fs | . | . |
| XS1 | 8 | 65494020 | GGCA | G | 1 | NM_152414 | BHLHE22 | nonframeshift_de  letion | exon1 | c.674_676del | p.225_226del | . | . |
| XS1 | 8 | 67716671 | AT | A | 0.303 | NM_001033578 | SGK3 | frameshift_deleti  on | exon4 | c.243delT | p.N81fs | . | . |
| XS1 | 8 | 68062041 | CA | C | 0.957 | NM_024790 | CSPP1 | frameshift_deleti  on | exon16 | c.1985delA | p.Q662fs | . | . |
| XS1 | 8 | 72946008 | TA | T | 0.556 | NM_007332 | TRPA1 | frameshift_deleti  on | exon23 | c.2738delT | p.L913fs | . | . |
| XS1 | 8 | 73921283 | CGAG | C | 0.286 | NM_003218 | TERF1 | nonframeshift_de  letion | exon1 | c.163_165del | p.55_55del | . | . |
| XS1 | 8 | 74742672 | AT | A | 0.348 | NM_001271015 | UBE2W | frameshift_deleti  on | exon2 | c.137delA | p.N46fs | . | . |
| XS1 | 8 | 75928819 | TC | T | 1 | NM_001286777 | CRISPLD1 | frameshift_deleti  on | exon5 | c.190delC | p.P64fs | . | . |
| XS1 | 8 | 77767159 | GT | G | 0.191 | NM_024721 | ZFHX4 | frameshift_deleti  on | exon10 | c.8003delT | p.V2668fs | . | . |
| XS1 | 8 | 81411778 | CA | C | 0.4 | NM_001105539 | ZBTB10 | frameshift_deleti  on | exon2 | c.1023delA | p.P341fs | . | . |
| XS1 | 8 | 86025289 | AT | A | 0.357 | NM_033402 | LRRCC1 | frameshift_deleti  on | exon4 | c.500delT | p.I167fs | . | . |
| XS1 | 8 | 86126827 | C | CAACA  TT | 1 | NM_001099673 | C8orf59 | nonframeshift_in  sertion | exon4 | c.264_265insAA  TGTT | p.D89delinsNV  D | . | . |
| XS1 | 8 | 86385928 | AG | A | 0.28 | NM_000067 | CA2 | frameshift_deleti  on | exon3 | c.240delG | p.K80fs | . | . |
| XS1 | 8 | 87486575 | C | CT | 0.217 | NM_001286707 | RMDN1 | frameshift_inserti  on | exon9 | c.777dupA | p.A260fs | . | . |
| XS1 | 8 | 92261979 | AT | A | 0.933 | NM_052832 | SLC26A7 | frameshift_deleti  on | exon2 | c.101delT | p.I34fs | . | . |
| XS1 | 8 | 95412477 | TA | T | 0.194 | NM_001205263 | RAD54B | frameshift_deleti  on | exon5 | c.606delT | p.F202fs | . | . |
| XS1 | 8 | 95444423 | AT | A | 0.34 | NM_001256141 | FSBP | frameshift_deleti  on | exon2 | c.835delA | p.I279fs | . | . |
| XS1 | 8 | 95751731 | CT | C | 0.313 | NM_181787 | DPY19L4 | frameshift_deleti  on | exon5 | c.435delT | p.A145fs | . | . |
| XS1 | 8 | 96044284 | GT | G | 0.438 | NM_152416 | NDUFAF6 | frameshift_deleti  on | exon2 | c.260delT | p.V87fs | . | . |
| XS1 | 8 | 96057773 | GA | G | 0.84 | NM_152416 | NDUFAF6 | frameshift_deleti  on | exon5 | c.479delA | p.E160fs | . | . |
| XS1 | 8 | 96275986 | TA | T | 0.238 | NM_177965 | C8orf37 | frameshift_deleti  on | exon2 | c.171delT | p.F57fs | . | . |
| XS1 | 8 | 97274288 | GC | G | 0.194 | NM_014754 | PTDSS1 | frameshift_deleti  on | exon1 | c.21delC | p.S7fs | . | . |
| XS1 | 8 | 99161083 | CA | C | 0.171 | NM_001145860 | POP1 | frameshift_deleti  on | exon13 | c.1752delA | p.S584fs | . | . |
| XS1 | 8 | 99170111 | AG | A | 0.375 | NM_001145860 | POP1 | frameshift_deleti  on | exon16 | c.2688delG | p.Q896fs | . | . |
| XS1 | 8 | 99560295 | TG | T | 0.887 | NM_001256313 | STK3 | frameshift_deleti  on | exon7 | c.709delC | p.Q237fs | . | . |
| XS1 | 8 | 99560337 | AC | A | 1 | NM_001256313 | STK3 | frameshift_deleti  on | exon7 | c.667delG | p.V223fs | . | . |
| XS1 | 8 | 100733138 | AC | A | 0.178 | NM_017890 | VPS13B | frameshift_deleti  on | exon39 | c.6989delC | p.T2330fs | . | . |
| XS1 | 8 | 101245664 | CA | C | 1 | NM_003114 | SPAG1 | frameshift_deleti  on | exon16 | c.2015delA | p.Q672fs | . | . |
| XS1 | 8 | 101534968 | TGG | T | 0.712 | NM_001270377 | ANKRD46 | frameshift_deleti  on | exon5 | c.500_501del | p.P167fs | . | . |
| XS1 | 8 | 101724618 | GA | G | 0.474 | NM_002568 | PABPC1 | frameshift_deleti  on | exon7 | c.943delT | p.S315fs | . | . |
| XS1 | 8 | 104075254 | GAC | G | 0.2 | NM_001695 | ATP6V1C1 | frameshift_deleti  on | exon9 | c.714_715del | p.R238fs | . | . |
| XS1 | 8 | 104384834 | A | AG | 0.676 | NM_001256099 | CTHRC1 | frameshift_inserti  on | exon1 | c.56dupG | p.R19fs | . | . |
| XS1 | 8 | 104415464 | GT | G | 0.348 | NM_030780 | SLC25A32 | frameshift_deleti  on | exon4 | c.479delA | p.H160fs | . | . |
| XS1 | 8 | 105361106 | GAC | G | 0.14 | NM_001257317 | DCSTAMP | frameshift_deleti  on | exon2 | c.327_328del | p.G109fs | . | . |
| XS1 | 8 | 106431488 | GA | G | 0.333 | NM_012082 | ZFPM2 | frameshift_deleti  on | exon2 | c.158delA | p.E53fs | . | . |
| XS1 | 8 | 110305708 | TG | T | 0.667 | NM_001128211 | NUDCD1 | frameshift_deleti  on | exon4 | c.417delC | p.H139fs | . | . |
| XS1 | 8 | 110530520 | AT | A | 0.5 | NM_177531 | PKHD1L1 | frameshift_deleti  on | exon73 | c.11815delT | p.F3939fs | . | . |
| XS1 | 8 | 110984893 | C | CT | 0.281 | NM_014379 | KCNV1 | frameshift_inserti  on | exon2 | c.584dupA | p.K195fs | . | . |
| XS1 | 8 | 118819522 | C | CG | 0.2 | NM_000127 | EXT1 | frameshift_inserti  on | exon9 | c.1816_1817ins  C | p.W606fs | . | . |
| XS1 | 8 | 121432112 | AC | A | 0.429 | NM_014078 | MRPL13 | frameshift_deleti  on | exon5 | c.372delG | p.R124fs | . | . |
| XS1 | 8 | 122627024 | TG | T | 0.706 | NM_005328 | HAS2 | frameshift_deleti  on | exon4 | c.983delC | p.T328fs | . | . |
| XS1 | 8 | 124267665 | CG | C | 0.211 | NM_001017926 | ZHX1 | frameshift_deleti  on | exon3 | c.521delC | p.S174fs | . | . |

| XS1 | 8 | 124346520 | GC | G | 0.2 | NM_014109 | ATAD2 | frameshift_deleti  on | exon23 | c.3253delG | p.A1085fs | . | . |
| --- | --- | --- | --- | --- | --- | --- | --- | --- | --- | --- | --- | --- | --- |
| XS1 | 8 | 125052131 | GT | G | 0.583 | NM_001039112 | FER1L6 | frameshift_deleti  on | exon20 | c.2474delT | p.V825fs | . | . |
| XS1 | 8 | 125072482 | AC | A | 0.424 | NM_001039112 | FER1L6 | frameshift_deleti  on | exon23 | c.2937delC | p.Y979fs | . | . |
| XS1 | 8 | 125568529 | C | CA | 0.511 | NM_001282974 | MTSS1 | frameshift_inserti  on | exon12 | c.1272dupT | p.A425fs | . | . |
| XS1 | 8 | 130762720 | GC | G | 0.471 | NM_031415 | GSDMC | frameshift_deleti  on | exon11 | c.1039delG | p.A347fs | . | . |
| XS1 | 8 | 133909936 | CG | C | 0.337 | NM_003235 | TG | frameshift_deleti  on | exon12 | c.3045delG | p.S1015fs | . | . |
| XS1 | 8 | 133920565 | C | CG | 0.5 | NM_003235 | TG | frameshift_inserti  on | exon18 | c.3983dupG | p.R1328fs | . | . |
| XS1 | 8 | 139820025 | GC | G | 0.313 | NM_152888 | COL22A1 | frameshift_deleti  on | exon10 | c.1479delG | p.G493fs | . | . |
| XS1 | 8 | 139890464 | TG | T | 0.368 | NM_152888 | COL22A1 | frameshift_deleti  on | exon3 | c.186delC | p.A62fs | . | . |
| XS1 | 8 | 141685545 | CT | C | 0.6 | NM_001199649 | PTK2 | frameshift_deleti  on | exon28 | c.2615delA | p.K872fs | . | . |
| XS1 | 8 | 143856776 | T | TA | 0.232 | NM_177457 | LYNX1 | frameshift_inserti  on | exon4 | c.159_160insT | p.T54fs | . | . |
| XS1 | 8 | 144461606 | CCT | C | 0.625 | NM_052924 | RHPN1 | frameshift_deleti  on | exon8 | c.874_875del | p.L292fs | . | . |
| XS1 | 8 | 144644675 | TG | T | 0.5 | NM_024736 | GSDMD | frameshift_deleti  on | exon10 | c.1197delG | p.L399fs | . | . |
| XS1 | 8 | 144663310 | GC | G | 0.5 | NM_001130056 | EEF1D | frameshift_deleti  on | exon4 | c.231delG | p.L77fs | . | . |
| XS1 | 8 | 144943187 | AC | A | 0.8 | NM_031308 | EPPK1 | stopgain | exon2 | c.4234delG | p.V1412X | . | . |
| XS1 | 8 | 145052080 | CG | C | 1 | NM_032789 | PARP10 | frameshift_deleti on | exon10 | c.2725delC | p.R909fs | . | . |
| XS1 | 8 | 145617534 | TGGGGG  TGCAAG | T | 0.5 | NM_174922 | ADCK5 | frameshift_deleti  on | exon12 | c.1257_1267del | p.L419fs | . | . |
| XS1 | 9 | 990824 | GT | G | 0.9 | NM_021240 | DMRT3 | frameshift_deleti  on | exon2 | c.1239delT | p.S413fs | . | . |
| XS1 | 9 | 2039776 | ACAG | A | 0.247 | NM_001289396 | SMARCA2 | nonframeshift_de  letion | exon4 | c.667_669del | p.223_223del | . | . |
| XS1 | 9 | 4572301 | GA | G | 0.787 | NM_004170 | SLC1A1 | frameshift_deleti  on | exon7 | c.681delA | p.G227fs | . | . |
| XS1 | 9 | 5456122 | AT | A | 0.562 | NM_001267706 | CD274 | frameshift_deleti  on | exon2 | c.10delT | p.F4fs | . | . |
| XS1 | 9 | 5457402 | AT | A | 0.449 | NM_014143 | CD274 | frameshift_deleti  on | exon3 | c.377delT | p.I126fs | . | . |
| XS1 | 9 | 5805749 | CA | C | 0.591 | NM_024896 | ERMP1 | frameshift_deleti  on | exon9 | c.1584delT | p.F528fs | . | . |
| XS1 | 9 | 12708017 | GA | G | 0.532 | NM_000550 | TYRP1 | frameshift_deleti  on | exon7 | c.1283delA | p.E428fs | . | . |
| XS1 | 9 | 12775861 | T | TGGCG  GCGGC | 1 | NM_203403 | LURAP1L | nonframeshift_in  sertion | exon1 | c.147_148insG  GCGGCGGC | p.G49delinsGG  GG | . | . |
| XS1 | 9 | 12775865 | G | GGCGG  CGGCA | 0.947 | NM_203403 | LURAP1L | nonframeshift_in  sertion | exon1 | c.151_152insG  CGGCGGCA | p.G51delinsGG  GS | . | . |
| XS1 | 9 | 12775868 | G | GGCGG  CGGCT | 0.938 | NM_203403 | LURAP1L | nonframeshift_in  sertion | exon1 | c.154_155insG  CGGCGGCT | p.G52delinsGG  GC | . | . |
| XS1 | 9 | 13125234 | TG | T | 0.429 | NM_001261406 | MPDZ | frameshift_deleti  on | exon34 | c.4688delC | p.P1563fs | . | . |
| XS1 | 9 | 14792801 | G | GT | 0.188 | NM_144966 | FREM1 | frameshift_inserti  on | exon23 | c.3920_3921ins  A | p.P1307fs | . | . |
| XS1 | 9 | 15724903 | GA | G | 1 | NM_173550 | CCDC171 | frameshift_deleti  on | exon14 | c.1622delA | p.E541fs | . | . |
| XS1 | 9 | 16436090 | GA | G | 0.8 | NM_017637 | BNC2 | frameshift_deleti  on | exon6 | c.2101delT | p.S701fs | . | . |
| XS1 | 9 | 18777751 | AGC | A | 0.407 | NM_001040272 | ADAMTSL1 | frameshift_deleti  on | exon19 | c.3525_3526del | p.Q1175fs | . | . |
| XS1 | 9 | 19346530 | CT | C | 0.579 | NM_017925 | DENND4C | frameshift_deleti  on | exon22 | c.3617delT | p.L1206fs | . | . |
| XS1 | 9 | 32631420 | CA | C | 0.764 | NM_153809 | TAF1L | stopgain | exon1 | c.4157delT | p.L1386X | . | . |
| XS1 | 9 | 32635022 | AGAT | A | 0.5 | NM_153809 | TAF1L | nonframeshift_de letion | exon1 | c.553_555del | p.185_185del | . | . |
| XS1 | 9 | 33056211 | GT | G | 0.274 | NM_018225 | SMU1 | frameshift_deleti  on | exon9 | c.1021delA | p.T341fs | . | . |
| XS1 | 9 | 33541169 | AT | A | 0.818 | NM_001244752 | ANKRD18B | frameshift_deleti  on | exon7 | c.834delT | p.N278fs | . | . |
| XS1 | 9 | 33923988 | TG | T | 0.75 | NM_001282530 | UBAP2 | frameshift_deleti  on | exon8 | c.317delC | p.T106fs | . | . |
| XS1 | 9 | 34568948 | GC | G | 0.722 | NM_001842 | CNTFR | frameshift_deleti  on | exon2 | c.31delG | p.A11fs | . | . |
| XS1 | 9 | 34611139 | GC | G | 0.192 | NM_148178 | RPP25L | frameshift_deleti  on | exon2 | c.154delG | p.A52fs | . | . |
| XS1 | 9 | 35043789 | CT | C | 0.646 | NM_001040410 | C9orf131 | frameshift_deleti  on | exon2 | c.1059delT | p.P353fs | . | . |
| XS1 | 9 | 35093991 | GC | G | 0.212 | NM_032634 | PIGO | frameshift_deleti  on | exon4 | c.685delG | p.A229fs | . | . |
| XS1 | 9 | 35107582 | AG | A | 0.5 | NM_025182 | FAM214B | frameshift_deleti  on | exon3 | c.689delC | p.P230fs | . | . |
| XS1 | 9 | 35108047 | AC | A | 0.781 | NM_025182 | FAM214B | frameshift_deleti  on | exon3 | c.224delG | p.G75fs | . | . |
| XS1 | 9 | 35752674 | GA | G | 0.409 | NM_001080496 | RGP1 | frameshift_deleti  on | exon9 | c.980delA | p.E327fs | . | . |
| XS1 | 9 | 35811485 | ACCAGG  ACCAGA | A | 1 | NM_001039592 | SPAG8 | nonframeshift_de  letion | exon2 | c.546_557del | p.182_186del | . | . |
| XS1 | 9 | 35906347 | CCTG | C | 0.232 | NM_001039792 | HRCT1 | nonframeshift_de  letion | exon1 | c.64_66del | p.22_22del | . | . |
| XS1 | 9 | 37487914 | CT | C | 0.206 | NM_022490 | POLR1E | frameshift_deleti  on | exon3 | c.236delT | p.L79fs | . | . |
| XS1 | 9 | 71661386 | TG | T | 0.45 | NM_000144 | FXN | frameshift_deleti  on | exon2 | c.252delG | p.L84fs | . | . |
| XS1 | 9 | 71851002 | TG | T | 0.282 | NM_001170415 | TJP2 | frameshift_deleti  on | exon13 | c.1852delG | p.E618fs | . | . |
| XS1 | 9 | 71869156 | TC | T | 0.867 | NM_201629 | TJP2 | frameshift_deleti  on | exon21 | c.2999delC | p.S1000fs | . | . |
| XS1 | 9 | 72472888 | CA | C | 1 | NM_001010940 | C9orf135 | frameshift_deleti  on | exon4 | c.445delA | p.K149fs | . | . |
| XS1 | 9 | 74347370 | AT | A | 1 | NM_001135820 | TMEM2 | frameshift_deleti  on | exon6 | c.1270delA | p.I424fs | . | . |
| XS1 | 9 | 77377195 | AC | A | 0.222 | NM_001177310 | TRPM6 | frameshift_deleti  on | exon26 | c.4376delG | p.G1459fs | . | . |
| XS1 | 9 | 78854044 | CA | C | 0.706 | NM_001190482 | PCSK5 | frameshift_deleti  on | exon23 | c.3037delA | p.T1013fs | . | . |
| XS1 | 9 | 78854045 | AC | A | 0.625 | NM_001190482 | PCSK5 | frameshift_deleti  on | exon23 | c.3038delC | p.T1013fs | . | . |
| XS1 | 9 | 79324135 | A | ATGAC  TGTTGC | 0.857 | NM_015225 | PRUNE2 | nonframeshift_in  sertion | exon8 | c.3054_3055ins  CTGCAACAGT | p.S1019delinsL  QQSS | . | . |

| XS1 | 9 | 85677651 | CG | C | 0.294 | NM_152573 | RASEF | frameshift_deleti  on | exon1 | c.131delC | p.P44fs | . | . |
| --- | --- | --- | --- | --- | --- | --- | --- | --- | --- | --- | --- | --- | --- |
| XS1 | 9 | 86258451 | AG | A | 0.3 | NM_001001551 | IDNK | frameshift_deleti  on | exon5 | c.321delG | p.E107fs | . | . |
| XS1 | 9 | 86485508 | GT | G | 0.538 | NM_001271927 | KIF27 | frameshift_deleti  on | exon11 | c.2484delA | p.K828fs | . | . |
| XS1 | 9 | 88842934 | TA | T | 0.429 | NM_001276366 | C9orf153 | frameshift_deleti  on | exon3 | c.77delT | p.L26fs | . | . |
| XS1 | 9 | 88937851 | ATTT | A | 0.5 | NM_001185074 | ZCCHC6 | nonframeshift_de  letion | exon9 | c.2442_2444del | p.814_815del | . | . |
| XS1 | 9 | 90534203 | C | CAGCG  TCATCT TGTCTC | 0.69 | NM_001145124 | SPATA31C1 | stopgain | exon2 | c.223_224insA  GCGTCATCTTG TCTCCT | p.Q75delinsQR HLVSX | . | . |
| XS1 | 9 | 92006294 | CT | C | 0.297 | NM_001142287 | SEMA4D | frameshift_deleti  on | exon11 | c.658delA | p.S220fs | . | . |
| XS1 | 9 | 95019002 | G | GA | 0.333 | NM_002161 | IARS | frameshift_inserti  on | exon20 | c.2096dupT | p.F699fs | . | . |
| XS1 | 9 | 95027326 | CA | C | 0.83 | NM_002161 | IARS | frameshift_deleti  on | exon16 | c.1584delT | p.F528fs | . | . |
| XS1 | 9 | 95068083 | TG | T | 1 | NM_017948 | NOL8 | frameshift_deleti  on | exon12 | c.2854delC | p.Q952fs | . | . |
| XS1 | 9 | 96278464 | GC | G | 0.994 | NM_001286722 | FAM120A | frameshift_deleti  on | exon7 | c.1329delC | p.S443fs | . | . |
| XS1 | 9 | 96438998 | T | TATCAC  CCCTG | 0.567 | NM_005392 | PHF2 | nonframeshift_in  sertion | exon21 | c.2955_2956ins  ATCACCCCTG | p.S985delinsSIT  PAS | . | . |
| XS1 | 9 | 96439004 | C | CCCTG  CCTCC | 0.989 | NM_005392 | PHF2 | nonframeshift_in  sertion | exon21 | c.2961_2962ins  CCTGCCTCCA | p.T987delinsTP  ASTT | . | . |
| XS1 | 9 | 96439005 | C | CA | 0.35 | NM_005392 | PHF2 | frameshift_inserti  on | exon21 | c.2962_2963ins  A | p.P988fs | . | . |
| XS1 | 9 | 97203354 | GC | G | 1 | NM_032558 | HIATL1 | frameshift_deleti  on | exon5 | c.484delC | p.H162fs | . | . |
| XS1 | 9 | 97369146 | TC | T | 0.291 | NM_000507 | FBP1 | frameshift_deleti  on | exon5 | c.655delG | p.D219fs | . | . |
| XS1 | 9 | 100092968 | A | AGAGG  AG | 1 | NM_020893 | CCDC180 | nonframeshift_in  sertion | exon18 | c.2325_2326ins  GAGGAG | p.E775delinsEE  E | . | . |
| XS1 | 9 | 100092968 | A | AGAG | 1 | NM_020893 | CCDC180 | nonframeshift_in  sertion | exon18 | c.2325_2326ins  GAG | p.E775delinsEE | . | . |
| XS1 | 9 | 100245626 | GA | G | 0.8 | NM_014290 | TDRD7 | frameshift_deleti  on | exon15 | c.2909delA | p.E970fs | . | . |
| XS1 | 9 | 100692581 | AT | A | 0.667 | NM_197978 | HEMGN | frameshift_deleti  on | exon3 | c.1095delA | p.K365fs | . | . |
| XS1 | 9 | 100843183 | TG | T | 0.972 | NM_018946 | NANS | frameshift_deleti  on | exon5 | c.690delG | p.L230fs | . | . |
| XS1 | 9 | 101014113 | AG | A | 0.538 | NM_001267571 | TBC1D2 | frameshift_deleti  on | exon2 | c.464delC | p.P155fs | . | . |
| XS1 | 9 | 101052997 | GGAT | G | 0.3 | NM_005458 | GABBR2 | nonframeshift_de  letion | exon19 | c.2692_2694del | p.898_898del | . | . |
| XS1 | 9 | 101536278 | CA | C | 0.273 | NM_173551 | ANKS6 | frameshift_deleti  on | exon9 | c.1701delT | p.F567fs | . | . |
| XS1 | 9 | 101980271 | GA | G | 0.947 | NM_033087 | ALG2 | frameshift_deleti  on | exon2 | c.1195delT | p.S399fs | . | . |
| XS1 | 9 | 104187179 | AC | A | 0.618 | NM_000035 | ALDOB | frameshift_deleti  on | exon8 | c.944delG | p.G315fs | . | . |
| XS1 | 9 | 109690968 | TA | T | 0.784 | NM_021224 | ZNF462 | frameshift_deleti  on | exon3 | c.4776delA | p.L1592fs | . | . |
| XS1 | 9 | 111800387 | CA | C | 0.444 | NM_032012 | TMEM245 | frameshift_deleti  on | exon15 | c.2133delT | p.F711fs | . | . |
| XS1 | 9 | 113637793 | TG | T | 0.3 | NM_001401 | LPAR1 | frameshift_deleti  on | exon4 | c.1002delC | p.P334fs | . | . |
| XS1 | 9 | 114090661 | GA | G | 0.5 | NM_205859 | OR2K2 | frameshift_deleti  on | exon1 | c.52delT | p.S18fs | . | . |
| XS1 | 9 | 114475385 | AT | A | 1 | NM_001080551 | C9orf84 | stopgain | exon14 | c.2173delA | p.I725X | . | . |
| XS1 | 9 | 117044824 | CG | C | 0.529 | NM_032888 | COL27A1 | frameshift_deleti on | exon39 | c.3860delG | p.R1287fs | . | . |
| XS1 | 9 | 117186770 | GT | G | 0.714 | NM_001083885 | DFNB31 | frameshift_deleti  on | exon6 | c.110delA | p.N37fs | . | . |
| XS1 | 9 | 123177351 | G | GT | 0.171 | NM_001272039 | CDK5RAP2 | frameshift_inserti  on | exon25 | c.3573dupA | p.Q1192fs | . | . |
| XS1 | 9 | 124526024 | CA | C | 0.76 | NM_138709 | DAB2IP | frameshift_deleti  on | exon6 | c.955delA | p.K319fs | . | . |
| XS1 | 9 | 125777834 | CT | C | 0.6 | NM_012197 | RABGAP1 | frameshift_deleti  on | exon12 | c.1563delT | p.P521fs | . | . |
| XS1 | 9 | 128070096 | T | TC | 0.375 | NM_001282681 | GAPVD1 | frameshift_inserti  on | exon5 | c.1378_1379ins  C | p.Y460fs | . | . |
| XS1 | 9 | 130289507 | TG | T | 0.333 | NM_001035534 | FAM129B | frameshift_deleti  on | exon3 | c.241delC | p.H81fs | . | . |
| XS1 | 9 | 130571151 | CA | C | 0.571 | NM_001288803 | FPGS | frameshift_deleti  on | exon10 | c.966delA | p.T322fs | . | . |
| XS1 | 9 | 131670678 | CG | C | 0.975 | NM_001127245 | LRRC8A | frameshift_deleti  on | exon2 | c.1236delG | p.T412fs | . | . |
| XS1 | 9 | 132630664 | TGAC | T | 1 | NM_001008563 | USP20 | nonframeshift_de  letion | exon11 | c.1072_1074del | p.358_358del | . | . |
| XS1 | 9 | 133730271 | AG | A | 0.224 | NM_005157 | ABL1 | frameshift_deleti  on | exon3 | c.338delG | p.S113fs | . | . |
| XS1 | 9 | 134351509 | GC | G | 0.769 | NM_013318 | PRRC2B | frameshift_deleti  on | exon15 | c.3994delC | p.P1332fs | . | . |
| XS1 | 9 | 135203910 | TTCA | T | 0.3 | NM_015046 | SETX | nonframeshift_de  letion | exon10 | c.3072_3074del | p.1024_1025de  l | . | . |
| XS1 | 9 | 135786870 | TG | T | 0.5 | NM_001162427 | TSC1 | frameshift_deleti  on | exon9 | c.845delC | p.P282fs | . | . |
| XS1 | 9 | 138377389 | AG | A | 0.75 | NM_014811 | PPP1R26 | frameshift_deleti  on | exon4 | c.1034delG | p.R345fs | . | . |
| XS1 | 9 | 138377965 | G | GA | 0.857 | NM_014811 | PPP1R26 | frameshift_inserti  on | exon4 | c.1610dupA | p.E537fs | . | . |
| XS1 | 9 | 138393754 | GT | G | 0.88 | NM_016034 | MRPS2 | frameshift_deleti  on | exon3 | c.235delT | p.F79fs | . | . |
| XS1 | 9 | 138710959 | TC | T | 0.933 | NM_015447 | CAMSAP1 | frameshift_deleti  on | exon12 | c.3855delG | p.K1285fs | . | . |
| XS1 | 9 | 139277994 | CGCT | C | 0.121 | NM_003086 | SNAPC4 | nonframeshift_de  letion | exon15 | c.1624_1626del | p.542_542del | . | . |
| XS1 | 9 | 139391293 | C | CG | 0.3 | NM_017617 | NOTCH1 | frameshift_inserti  on | exon34 | c.6897dupC | p.G2300fs | . | . |
| XS1 | 9 | 139734632 | GAGA | G | 0.147 | NM_001173988 | RABL6 | nonframeshift_de  letion | exon14 | c.1961_1963del | p.654_655del | . | . |
| XS1 | 9 | 139840617 | G | GT | 0.429 | NM_000606 | C8G | frameshift_inserti  on | exon4 | c.427_428insT | p.A143fs | . | . |
| XS1 | 9 | 139887668 | GT | G | 0.741 | NM_183241 | C9orf142 | frameshift_deleti  on | exon4 | c.395delT | p.V132fs | . | . |
| XS1 | 9 | 140358863 | TG | T | 0.5 | NM_152286 | PNPLA7 | frameshift_deleti  on | exon26 | c.3009delC | p.P1003fs | . | . |
| XS1 | 9 | 140372569 | CG | C | 0.81 | NM_152286 | PNPLA7 | frameshift_deleti  on | exon24 | c.2723delC | p.P908fs | . | . |

| XS1 | 10 | 436709 | CT | C | 0.804 | NM_014974 | DIP2C | frameshift_deleti  on | exon11 | c.1354delA | p.S452fs | . | . |
| --- | --- | --- | --- | --- | --- | --- | --- | --- | --- | --- | --- | --- | --- |
| XS1 | 10 | 866756 | CT | C | 0.278 | NM_015155 | LARP4B | frameshift_deleti  on | exon14 | c.1513delA | p.R505fs | . | . |
| XS1 | 10 | 6525514 | AT | A | 0.735 | NM_001282645 | PRKCQ | frameshift_deleti  on | exon10 | c.691delA | p.M231fs | . | . |
| XS1 | 10 | 7621882 | C | CT | 0.419 | NM_032817 | ITIH5 | frameshift_inserti  on | exon5 | c.611dupA | p.K204fs | . | . |
| XS1 | 10 | 7844296 | TG | T | 0.938 | NM_001001973 | ATP5C1 | frameshift_deleti  on | exon7 | c.702delG | p.L234fs | . | . |
| XS1 | 10 | 12191617 | TC | T | 0.98 | NM_001142628 | SEC61A2 | frameshift_deleti  on | exon4 | c.179delC | p.S60fs | . | . |
| XS1 | 10 | 12191664 | CA | C | 0.941 | NM_001142628 | SEC61A2 | frameshift_deleti  on | exon4 | c.226delA | p.K76fs | . | . |
| XS1 | 10 | 13167535 | CA | C | 0.391 | NM_021980 | OPTN | frameshift_deleti  on | exon9 | c.1117delA | p.K373fs | . | . |
| XS1 | 10 | 13234300 | TG | T | 0.211 | NM_018518 | MCM10 | frameshift_deleti  on | exon12 | c.1563delG | p.L521fs | . | . |
| XS1 | 10 | 24884102 | TA | T | 0.408 | NM_020824 | ARHGAP21 | frameshift_deleti  on | exon20 | c.3729delT | p.F1243fs | . | . |
| XS1 | 10 | 25313939 | GT | G | 0.25 | NM_024838 | THNSL1 | frameshift_deleti  on | exon3 | c.1788delT | p.S596fs | . | . |
| XS1 | 10 | 26575391 | GT | G | 0.412 | NM_000818 | GAD2 | frameshift_deleti  on | exon13 | c.1355delT | p.V452fs | . | . |
| XS1 | 10 | 33199340 | CT | C | 0.5 | NM_033668 | ITGB1 | frameshift_deleti  on | exon13 | c.1974delA | p.K658fs | . | . |
| XS1 | 10 | 43623689 | CA | C | 1 | NM_020975 | RET | frameshift_deleti  on | exon20 | c.3318delA | p.A1106fs | . | . |
| XS1 | 10 | 46143790 | AT | A | 0.236 | NM_001128324 | ZFAND4 | stopgain | exon5 | c.520delA | p.I174X | . | . |
| XS1 | 10 | 48387898 | CG | C | 0.375 | NM_002900 | RBP3 | frameshift_deleti on | exon1 | c.2979delC | p.S993fs | . | . |
| XS1 | 10 | 50025375 | A | AC | 0.854 | NM_020945 | WDFY4 | frameshift_inserti  on | exon32 | c.5427dupC | p.Y1809fs | . | . |
| XS1 | 10 | 50172062 | TG | T | 1 | NM_020945 | WDFY4 | frameshift_deleti  on | exon54 | c.8400delG | p.L2800fs | . | . |
| XS1 | 10 | 50598256 | CA | C | 1 | NM_001276451 | DRGX | frameshift_deleti  on | exon4 | c.156delT | p.F52fs | . | . |
| XS1 | 10 | 51584732 | TA | T | 0.843 | NM_001145262 | NCOA4 | frameshift_deleti  on | exon8 | c.832delA | p.T278fs | . | . |
| XS1 | 10 | 61023863 | C | CT | 0.198 | NM_198215 | FAM13C | frameshift_inserti  on | exon9 | c.1005dupA | p.G336fs | . | . |
| XS1 | 10 | 61830868 | AG | A | 0.278 | NM_020987 | ANK3 | frameshift_deleti  on | exon37 | c.9770delC | p.P3257fs | . | . |
| XS1 | 10 | 61833450 | G | GTTC | 1 | NM_020987 | ANK3 | nonframeshift_in  sertion | exon37 | c.7188_7189ins  GAA | p.L2397delinsE  L | . | . |
| XS1 | 10 | 62631304 | GC | G | 0.95 | NM_014836 | RHOBTB1 | frameshift_deleti  on | exon11 | c.2026delG | p.A676fs | . | . |
| XS1 | 10 | 63195985 | T | TA | 0.875 | NM_178505 | TMEM26 | frameshift_inserti  on | exon2 | c.212dupT | p.L71fs | . | . |
| XS1 | 10 | 64573470 | A | AGGCG  GC | 0.923 | NM_000399 | EGR2 | nonframeshift_in  sertion | exon2 | c.927_928insG  CCGCC | p.Y310delinsAA  Y | . | . |
| XS1 | 10 | 64967950 | ACTAAA  C | A | 0.944 | NM_001282948 | JMJD1C | nonframeshift_de  letion | exon9 | c.2927_2932del | p.976_978del | . | . |
| XS1 | 10 | 69908195 | GC | G | 0.909 | NM_032578 | MYPN | frameshift_deleti  on | exon5 | c.1217delC | p.A406fs | . | . |
| XS1 | 10 | 70332869 | C | CA | 0.186 | NM_030625 | TET1 | frameshift_inserti  on | exon2 | c.775dupA | p.P258fs | . | . |
| XS1 | 10 | 70332889 | AC | A | 0.277 | NM_030625 | TET1 | frameshift_deleti  on | exon2 | c.795delC | p.N265fs | . | . |
| XS1 | 10 | 70404660 | AT | A | 0.613 | NM_030625 | TET1 | frameshift_deleti  on | exon4 | c.2175delT | p.D725fs | . | . |
| XS1 | 10 | 70723100 | TA | T | 0.702 | NM_001256910 | DDX21 | frameshift_deleti  on | exon4 | c.458delA | p.Y153fs | . | . |
| XS1 | 10 | 71164603 | GGT | G | 0.2 | NM_001057 | TACR2 | frameshift_deleti  on | exon5 | c.1174_1175del | p.T392fs | . | . |
| XS1 | 10 | 72192835 | TC | T | 0.824 | NM_018055 | NODAL | frameshift_deleti  on | exon3 | c.900delG | p.L300fs | . | . |
| XS1 | 10 | 72537076 | CCTCCTT  CTG | C | 0.121 | NM_152710 | TBATA | nonframeshift_de  letion | exon7 | c.514_522del | p.172_174del | . | . |
| XS1 | 10 | 72636308 | TG | T | 0.471 | NM_003901 | SGPL1 | frameshift_deleti  on | exon14 | c.1457delG | p.C486fs | . | . |
| XS1 | 10 | 73551052 | T | TC | 0.4 | NM_022124 | CDH23 | frameshift_inserti  on | exon45 | c.6213_6214ins  C | p.P2071fs | . | . |
| XS1 | 10 | 73558298 | GC | G | 0.471 | NM_001171933 | CDH23 | frameshift_deleti  on | exon3 | c.298delC | p.P100fs | . | . |
| XS1 | 10 | 74594138 | TG | T | 0.513 | NM_001270679 | MCU | frameshift_deleti  on | exon2 | c.173delG | p.W58fs | . | . |
| XS1 | 10 | 76781905 | GGAA | G | 0.941 | NM_001256468 | KAT6B | nonframeshift_de  letion | exon16 | c.2740_2742del | p.914_914del | . | . |
| XS1 | 10 | 76788689 | GGAA | G | 0.463 | NM_001256468 | KAT6B | nonframeshift_de  letion | exon18 | c.3559_3561del | p.1187_1187de  l | . | . |
| XS1 | 10 | 78846289 | TA | T | 0.518 | NM_001271518 | KCNMA1 | frameshift_deleti  on | exon10 | c.1234delT | p.Y412fs | . | . |
| XS1 | 10 | 82248992 | GC | G | 0.178 | NM_001128309 | TSPAN14 | frameshift_deleti  on | exon2 | c.4delC | p.H2fs | . | . |
| XS1 | 10 | 84745279 | TG | T | 0.75 | NM_001010848 | NRG3 | frameshift_deleti  on | exon9 | c.2010delG | p.L670fs | . | . |
| XS1 | 10 | 85909841 | C | CA | 0.2 | NM_014394 | GHITM | frameshift_inserti  on | exon7 | c.624dupA | p.T208fs | . | . |
| XS1 | 10 | 85944480 | GC | G | 0.299 | NM_207373 | C10orf99 | frameshift_deleti  on | exon3 | c.205delC | p.P69fs | . | . |
| XS1 | 10 | 90583051 | TG | T | 0.607 | NM_144590 | ANKRD22 | frameshift_deleti  on | exon5 | c.483delC | p.P161fs | . | . |
| XS1 | 10 | 95791037 | GA | G | 1 | NM_001288989 | PLCE1 | frameshift_deleti  on | exon2 | c.235delA | p.K79fs | . | . |
| XS1 | 10 | 96068410 | G | GA | 0.283 | NM_001165979 | PLCE1 | frameshift_inserti  on | exon26 | c.5034dupA | p.R1678fs | . | . |
| XS1 | 10 | 96602682 | TG | T | 0.259 | NM_000769 | CYP2C19 | frameshift_deleti  on | exon7 | c.1051delG | p.V351fs | . | . |
| XS1 | 10 | 96745819 | TG | T | 0.675 | NM_000771 | CYP2C9 | frameshift_deleti  on | exon8 | c.1180delG | p.V394fs | . | . |
| XS1 | 10 | 98097964 | TG | T | 0.457 | NM_001017520 | DNTT | frameshift_deleti  on | exon11 | c.1511delG | p.W504fs | . | . |
| XS1 | 10 | 98760925 | AG | A | 0.429 | NM_003061 | SLIT1 | frameshift_deleti  on | exon37 | c.4548delC | p.T1516fs | . | . |
| XS1 | 10 | 101090192 | CA | C | 0.565 | NM_020348 | CNNM1 | frameshift_deleti  on | exon1 | c.1049delA | p.H350fs | . | . |
| XS1 | 10 | 101439491 | CA | C | 0.387 | NM_020354 | ENTPD7 | frameshift_deleti  on | exon5 | c.408delA | p.A136fs | . | . |
| XS1 | 10 | 102269098 | CTG | C | 0.833 | NM_015490 | SEC31B | frameshift_deleti  on | exon4 | c.372_373del | p.V124fs | . | . |
| XS1 | 10 | 102762667 | AC | A | 0.25 | NM_032429 | LZTS2 | frameshift_deleti  on | exon2 | c.373delC | p.P125fs | . | . |

| XS1 | 10 | 103547209 | CAG | C | 0.955 | NM_001142434 | MGEA5 | frameshift_deleti  on | exon14 | c.2365_2366del | p.L789fs | . | . |
| --- | --- | --- | --- | --- | --- | --- | --- | --- | --- | --- | --- | --- | --- |
| XS1 | 10 | 103572868 | AT | A | 0.375 | NM_001142434 | MGEA5 | frameshift_deleti  on | exon3 | c.267delA | p.E89fs | . | . |
| XS1 | 10 | 103921961 | CG | C | 0.56 | NM_001284388 | NOLC1 | frameshift_deleti  on | exon13 | c.2066delG | p.R689fs | . | . |
| XS1 | 10 | 104181137 | TG | T | 0.6 | NM_024326 | FBXL15 | frameshift_deleti  on | exon2 | c.81delG | p.V27fs | . | . |
| XS1 | 10 | 104228795 | GGGTCA  GCCCCA GGCCTG GTTGCT | G | 0.375 | NM_024789 | TMEM180 | frameshift_deleti on | exon3 | c.10_38del | p.G4fs | . | . |
| XS1 | 10 | 104228825 | G | GA | 0.375 | NM_024789 | TMEM180 | frameshift_inserti  on | exon3 | c.39_40insA | p.L13fs | . | . |
| XS1 | 10 | 104629914 | TC | T | 0.25 | NM_020682 | AS3MT | frameshift_deleti  on | exon3 | c.117delC | p.V39fs | . | . |
| XS1 | 10 | 104849435 | TTCC | T | 0.5 | NM_001134373 | NT5C2 | nonframeshift_de  letion | exon18 | c.1677_1679del | p.559_560del | . | . |
| XS1 | 10 | 105331358 | GCC | G | 0.167 | NM_004210 | NEURL1 | frameshift_deleti  on | exon3 | c.429_430del | p.C143fs | . | . |
| XS1 | 10 | 107015466 | G | GA | 0.519 | NM_014978 | SORCS3 | frameshift_inserti  on | exon24 | c.3245dupA | p.E1082fs | . | . |
| XS1 | 10 | 108536400 | TG | T | 0.708 | NM_001013031 | SORCS1 | stopgain | exon4 | c.776delC | p.S259X | . | . |
| XS1 | 10 | 112338386 | GA | G | 1 | NM_005445 | SMC3 | frameshift_deleti on | exon7 | c.352delA | p.K118fs | . | . |
| XS1 | 10 | 115609158 | CT | C | 0.625 | NM_014881 | DCLRE1A | frameshift_deleti  on | exon2 | c.1705delA | p.R569fs | . | . |
| XS1 | 10 | 115922972 | G | GT | 0.493 | NM_018017 | C10orf118 | frameshift_inserti  on | exon2 | c.55dupA | p.T19fs | . | . |
| XS1 | 10 | 115947787 | AT | A | 0.407 | NM_198795 | TDRD1 | frameshift_deleti  on | exon2 | c.198delT | p.N66fs | . | . |
| XS1 | 10 | 118764565 | TG | T | 0.429 | NM_001127211 | KIAA1598 | frameshift_deleti  on | exon1 | c.25delC | p.Q9fs | . | . |
| XS1 | 10 | 120802207 | TC | T | 0.581 | NM_003750 | EIF3A | frameshift_deleti  on | exon19 | c.2824delG | p.D942fs | . | . |
| XS1 | 10 | 120817655 | C | CT | 0.5 | NM_003750 | EIF3A | frameshift_inserti  on | exon12 | c.1789dupA | p.R597fs | . | . |
| XS1 | 10 | 123842624 | AC | A | 0.748 | NM_206862 | TACC2 | frameshift_deleti  on | exon4 | c.610delC | p.P204fs | . | . |
| XS1 | 10 | 124593247 | GT | G | 0.714 | NM_022034 | CUZD1 | frameshift_deleti  on | exon8 | c.1591delA | p.T531fs | . | . |
| XS1 | 10 | 129904431 | TA | T | 0.957 | NM_001145966 | MKI67 | stopgain | exon12 | c.4592delT | p.L1531X | . | . |
| XS1 | 10 | 129908737 | TG | T | 1 | NM_001145966 | MKI67 | frameshift_deleti on | exon11 | c.1240delC | p.H414fs | . | . |
| XS1 | 10 | 131506175 | GC | G | 0.571 | NM_002412 | MGMT | frameshift_deleti  on | exon3 | c.236delC | p.A79fs | . | . |
| XS1 | 10 | 133930872 | GC | G | 0.5 | NM_001105521 | JAKMIP3 | frameshift_deleti  on | exon2 | c.428delC | p.A143fs | . | . |
| XS1 | 10 | 134161640 | GA | G | 0.366 | NM_001143757 | LRRC27 | frameshift_deleti  on | exon6 | c.707delA | p.E236fs | . | . |
| XS1 | 10 | 134691587 | G | GT | 0.75 | NM_001200049 | TTC40 | frameshift_inserti  on | exon30 | c.4009_4010ins  A | p.S1337fs | . | . |
| XS1 | 10 | 134722614 | CCTCCC  GGGACC CCTGGG GACACC | C | 0.182 | NM_001200049 | TTC40 | frameshift_deleti on | exon21 | c.2753_2757del | p.A918fs | . | . |
| XS1 | 10 | 135032372 | CA | C | 0.239 | NM_152643 | KNDC1 | frameshift_deleti  on | exon27 | c.4716delA | p.A1572fs | . | . |
| XS1 | 10 | 135113040 | CT | C | 0.75 | NM_001256617 | TUBGCP2 | frameshift_deleti  on | exon4 | c.346delA | p.S116fs | . | . |
| XS1 | 11 | 1090922 | CT | C | 0.727 | NM_002457 | MUC2 | frameshift_deleti  on | exon28 | c.3818delT | p.L1273fs | . | . |
| XS1 | 11 | 1298279 | TC | T | 0.815 | NM_019009 | TOLLIP | frameshift_deleti  on | exon6 | c.814delG | p.E272fs | . | . |
| XS1 | 11 | 2592603 | A | AG | 1 | NM_000218 | KCNQ1 | frameshift_inserti  on | exon4 | c.654dupG | p.K218fs | . | . |
| XS1 | 11 | 3661585 | A | ATGG | 1 | NM_053017 | ART5 | nonframeshift_in  sertion | exon2 | c.73_74insCCA | p.I25delinsTI | . | . |
| XS1 | 11 | 4790806 | AG | A | 0.286 | NM_001004752 | OR51F1 | frameshift_deleti  on | exon1 | c.341delC | p.S114fs | . | . |
| XS1 | 11 | 6432089 | ATCC | A | 0.158 | NM_001164 | APBB1 | nonframeshift_de  letion | exon2 | c.486_488del | p.162_163del | . | . |
| XS1 | 11 | 6623122 | AG | A | 0.59 | NM_015324 | RRP8 | frameshift_deleti  on | exon2 | c.422delC | p.P141fs | . | . |
| XS1 | 11 | 6623404 | TA | T | 0.267 | NM_015324 | RRP8 | frameshift_deleti  on | exon2 | c.140delT | p.L47fs | . | . |
| XS1 | 11 | 6645467 | C | CG | 0.944 | NM_003737 | DCHS1 | frameshift_inserti  on | exon21 | c.7439dupC | p.P2480fs | . | . |
| XS1 | 11 | 6646035 | TC | T | 0.532 | NM_003737 | DCHS1 | frameshift_deleti  on | exon20 | c.7210delG | p.D2404fs | . | . |
| XS1 | 11 | 7531003 | TC | T | 0.429 | NM_198474 | OLFML1 | frameshift_deleti  on | exon3 | c.794delC | p.S265fs | . | . |
| XS1 | 11 | 7661047 | TCTC | T | 1 | NM_001256569 | PPFIBP2 | nonframeshift_de  letion | exon11 | c.893_895del | p.298_299del | . | . |
| XS1 | 11 | 8646612 | AT | A | 0.545 | NM_014818 | TRIM66 | frameshift_deleti  on | exon11 | c.2038delA | p.M680fs | . | . |
| XS1 | 11 | 9042695 | TTC | T | 0.13 | NM_001170690 | SCUBE2 | frameshift_deleti  on | exon18 | c.2319_2320del | p.Q773fs | . | . |
| XS1 | 11 | 13410536 | AT | A | 0.5 | NM_032320 | BTBD10 | frameshift_deleti  on | exon9 | c.1269delA | p.K423fs | . | . |
| XS1 | 11 | 16812718 | AG | A | 0.833 | NM_175058 | PLEKHA7 | frameshift_deleti  on | exon20 | c.2773delC | p.L925fs | . | . |
| XS1 | 11 | 16838747 | CGG | C | 0.209 | NM_175058 | PLEKHA7 | frameshift_deleti  on | exon11 | c.1464_1465del | p.P488fs | . | . |
| XS1 | 11 | 17351782 | CA | C | 0.769 | NM_005013 | NUCB2 | frameshift_deleti  on | exon12 | c.1112delA | p.Q371fs | . | . |
| XS1 | 11 | 17600005 | GC | G | 1 | NM_001277269 | OTOG | frameshift_deleti  on | exon22 | c.2728delC | p.Q910fs | . | . |
| XS1 | 11 | 18127527 | G | GCCGC  CT | 0.722 | NM_138421 | SAAL1 | nonframeshift_in  sertion | exon1 | c.61_62insAGG  CGG | p.A21delinsEA  A | . | . |
| XS1 | 11 | 18127530 | A | ACCTC  CT | 1 | NM_138421 | SAAL1 | nonframeshift_in  sertion | exon1 | c.58_59insAGG  AGG | p.V20delinsEEV | . | . |
| XS1 | 11 | 18127530 | A | ACCT | 1 | NM_138421 | SAAL1 | nonframeshift_in  sertion | exon1 | c.58_59insAGG | p.V20delinsEV | . | . |
| XS1 | 11 | 18127538 | C | CTCCTC  A | 0.793 | NM_138421 | SAAL1 | nonframeshift_in  sertion | exon1 | c.50_51insTGA  GGA | p.E17delinsDEE | . | . |
| XS1 | 11 | 18127544 | C | CTCCTC  G | 0.954 | NM_138421 | SAAL1 | nonframeshift_in  sertion | exon1 | c.44_45insCGA  GGA | p.E15delinsDEE | . | . |
| XS1 | 11 | 18428808 | CA | C | 0.429 | NM_001135239 | LDHA | frameshift_deleti  on | exon7 | c.806delA | p.Q269fs | . | . |

| XS1 | 11 | 18497949 | T | TGG | 0.25 | NM_144972 | LDHAL6A | frameshift_inserti  on | exon5 | c.611_612insG  G | p.V204fs | . | . |
| --- | --- | --- | --- | --- | --- | --- | --- | --- | --- | --- | --- | --- | --- |
| XS1 | 11 | 20113755 | G | GA | 0.351 | NM_001111019 | NAV2 | frameshift_inserti  on | exon18 | c.2849dupA | p.E950fs | . | . |
| XS1 | 11 | 27679518 | GC | G | 0.528 | NM_170735 | BDNF | frameshift_deleti  on | exon1 | c.593delG | p.G198fs | . | . |
| XS1 | 11 | 30942340 | AT | A | 0.579 | NM_020869 | DCDC5 | frameshift_deleti  on | exon6 | c.596delA | p.N199fs | . | . |
| XS1 | 11 | 32956931 | C | CA | 0.147 | NM_001076786 | QSER1 | frameshift_inserti  on | exon4 | c.3740_3741ins  A | p.S1247fs | . | . |
| XS1 | 11 | 34378976 | GC | G | 0.4 | NM_145804 | ABTB2 | frameshift_deleti  on | exon1 | c.154delG | p.A52fs | . | . |
| XS1 | 11 | 36119999 | G | GA | 0.5 | NM_174902 | LDLRAD3 | frameshift_inserti  on | exon4 | c.443dupA | p.E148fs | . | . |
| XS1 | 11 | 44089197 | A | AT | 0.667 | NM_001127219 | ACCS | frameshift_inserti  on | exon2 | c.20_21insT | p.K7fs | . | . |
| XS1 | 11 | 44129613 | CT | C | 0.197 | NM_000401 | EXT2 | frameshift_deleti  on | exon2 | c.451delT | p.F151fs | . | . |
| XS1 | 11 | 46681030 | TC | T | 1 | NM_001205122 | ATG13 | frameshift_deleti  on | exon9 | c.548delC | p.S183fs | . | . |
| XS1 | 11 | 46724716 | CAGTGG  TGACAG | C | 1 | NM_001184751 | ZNF408 | nonframeshift_de  letion | exon4 | c.552_563del | p.184_188del | . | . |
| XS1 | 11 | 47189691 | GA | G | 0.225 | NM_001242832 | ARFGAP2 | frameshift_deleti  on | exon10 | c.968delT | p.F323fs | . | . |
| XS1 | 11 | 47367777 | G | GC | 0.316 | NM_000256 | MYBPC3 | frameshift_inserti  on | exon12 | c.1070dupG | p.R357fs | . | . |
| XS1 | 11 | 47442259 | C | CT | 0.357 | NM_002804 | PSMC3 | frameshift_inserti  on | exon9 | c.903_904insA | p.G302fs | . | . |
| XS1 | 11 | 47754215 | AT | A | 0.196 | NM_015308 | FNBP4 | frameshift_deleti  on | exon11 | c.1693delA | p.I565fs | . | . |
| XS1 | 11 | 47819571 | GTA | G | 0.337 | NM_015231 | NUP160 | frameshift_deleti  on | exon26 | c.3140_3141del | p.L1047fs | . | . |
| XS1 | 11 | 48239237 | GA | G | 0.462 | NM_001005470 | OR4B1 | frameshift_deleti  on | exon1 | c.877delA | p.K293fs | . | . |
| XS1 | 11 | 48267417 | TA | T | 0.286 | NM_001004727 | OR4X2 | frameshift_deleti  on | exon1 | c.763delA | p.T255fs | . | . |
| XS1 | 11 | 49055730 | AG | A | 0.231 | NM_001206626 | TRIM49B | frameshift_deleti  on | exon3 | c.541delG | p.A181fs | . | . |
| XS1 | 11 | 55890156 | GT | G | 0.511 | NM_001005201 | OR8H3 | frameshift_deleti  on | exon1 | c.309delT | p.C103fs | . | . |
| XS1 | 11 | 55890494 | GC | G | 1 | NM_001005201 | OR8H3 | frameshift_deleti  on | exon1 | c.647delC | p.A216fs | . | . |
| XS1 | 11 | 56310399 | TA | T | 1 | NM_001005245 | OR5M11 | frameshift_deleti  on | exon1 | c.334delT | p.Y112fs | . | . |
| XS1 | 11 | 57075880 | GC | G | 0.235 | NM_033396 | TNKS1BP1 | frameshift_deleti  on | exon6 | c.4304delG | p.S1435fs | . | . |
| XS1 | 11 | 57077640 | TC | T | 0.917 | NM_033396 | TNKS1BP1 | frameshift_deleti  on | exon6 | c.2544delG | p.K848fs | . | . |
| XS1 | 11 | 57244241 | TA | T | 0.944 | NM_178570 | RTN4RL2 | frameshift_deleti  on | exon3 | c.1121delA | p.Y374fs | . | . |
| XS1 | 11 | 57259071 | TG | T | 0.5 | NM_001198810 | SLC43A1 | frameshift_deleti  on | exon10 | c.1046delC | p.A349fs | . | . |
| XS1 | 11 | 57381915 | AGGC | A | 0.417 | NM_001032295 | SERPING1 | nonframeshift_de  letion | exon7 | c.1365_1367del | p.455_456del | . | . |
| XS1 | 11 | 57511933 | A | AT | 0.599 | NM_001145101 | BTBD18 | frameshift_inserti  on | exon3 | c.1811dupA | p.D604fs | . | . |
| XS1 | 11 | 57958767 | GA | G | 0.865 | NM_001005283 | OR9Q2 | frameshift_deleti  on | exon1 | c.806delA | p.E269fs | . | . |
| XS1 | 11 | 57995878 | AG | A | 1 | NM_001004471 | OR10Q1 | frameshift_deleti  on | exon1 | c.469delC | p.L157fs | . | . |
| XS1 | 11 | 58189810 | GA | G | 0.595 | NM_001005566 | OR5B2 | frameshift_deleti  on | exon1 | c.924delT | p.F308fs | . | . |
| XS1 | 11 | 59376033 | GC | G | 0.345 | NM_002556 | OSBP | frameshift_deleti  on | exon3 | c.745delG | p.A249fs | . | . |
| XS1 | 11 | 59426846 | T | TA | 0.156 | NM_152716 | PATL1 | frameshift_inserti  on | exon3 | c.223_224insT | p.E75fs | . | . |
| XS1 | 11 | 61539125 | TC | T | 0.385 | NM_001127392 | MYRF | frameshift_deleti  on | exon6 | c.895delC | p.P299fs | . | . |
| XS1 | 11 | 61550995 | CT | C | 0.944 | NM_013279 | MYRF | frameshift_deleti  on | exon22 | c.2923delT | p.S975fs | . | . |
| XS1 | 11 | 61908248 | G | GC | 0.604 | NM_001040694 | INCENP | frameshift_inserti  on | exon9 | c.1417dupC | p.Q472fs | . | . |
| XS1 | 11 | 62300079 | C | CA | 0.186 | NM_001620 | AHNAK | stopgain | exon5 | c.1809dupT | p.D604_V605d  elinsX | . | . |
| XS1 | 11 | 62415506 | TGG | T | 0.919 | NM_030628 | INTS5 | frameshift_deleti  on | exon2 | c.2044_2045del | p.P682fs | . | . |
| XS1 | 11 | 62439232 | GA | G | 0.833 | NM_001085372 | C11orf83 | frameshift_deleti  on | exon1 | c.16delA | p.K6fs | . | . |
| XS1 | 11 | 62933712 | AG | A | 0.417 | NM_199352 | SLC22A25 | frameshift_deleti  on | exon7 | c.1088delC | p.P363fs | . | . |
| XS1 | 11 | 63057699 | TG | T | 0.75 | NM_001039752 | SLC22A10 | frameshift_deleti  on | exon1 | c.63delG | p.L21fs | . | . |
| XS1 | 11 | 63312227 | CA | C | 1 | NM_004585 | RARRES3 | frameshift_deleti  on | exon3 | c.254delA | p.Q85fs | . | . |
| XS1 | 11 | 64056641 | CA | C | 0.5 | NM_001170880 | GPR137 | frameshift_deleti  on | exon7 | c.1165delA | p.S389fs | . | . |
| XS1 | 11 | 64108616 | AG | A | 0.264 | NM_032251 | CCDC88B | frameshift_deleti  on | exon4 | c.355delG | p.D119fs | . | . |
| XS1 | 11 | 64369004 | TA | T | 0.343 | NM_001276327 | SLC22A12 | frameshift_deleti  on | exon8 | c.1320delA | p.L440fs | . | . |
| XS1 | 11 | 64375113 | AT | A | 0.455 | NM_138734 | NRXN2 | stopgain | exon7 | c.1555delA | p.M519X | . | . |
| XS1 | 11 | 64519436 | TC | T | 0.218 | NM_001164716 | PYGM | frameshift_deleti on | exon12 | c.1463delG | p.R488fs | . | . |
| XS1 | 11 | 64694325 | TG | T | 0.892 | NM_006244 | PPP2R5B | frameshift_deleti  on | exon3 | c.342delG | p.V114fs | . | . |
| XS1 | 11 | 64864533 | C | CG | 0.875 | NM_013265 | VPS51 | frameshift_inserti  on | exon2 | c.293dupG | p.R98fs | . | . |
| XS1 | 11 | 64974043 | GC | G | 0.211 | NM_001198868 | CAPN1 | frameshift_deleti  on | exon13 | c.1464delC | p.R488fs | . | . |
| XS1 | 11 | 65144036 | TG | T | 0.265 | NM_001278250 | SLC25A45 | frameshift_deleti  on | exon5 | c.636delC | p.R212fs | . | . |
| XS1 | 11 | 65304130 | CG | C | 0.262 | NM_001048218 | SCYL1 | frameshift_deleti  on | exon13 | c.1726delG | p.G576fs | . | . |
| XS1 | 11 | 65617622 | TC | T | 0.802 | NM_152760 | SNX32 | frameshift_deleti  on | exon4 | c.255delC | p.I85fs | . | . |
| XS1 | 11 | 67018078 | AGAG | A | 0.164 | NM_001256405 | KDM2A | nonframeshift_de  letion | exon6 | c.1261_1263del | p.421_421del | . | . |
| XS1 | 11 | 67220095 | AC | A | 0.205 | NM_206997 | GPR152 | frameshift_deleti  on | exon1 | c.100delG | p.V34fs | . | . |
| XS1 | 11 | 68338512 | TC | T | 0.692 | NM_001164160 | PPP6R3 | frameshift_deleti  on | exon12 | c.1284delC | p.F428fs | . | . |
| XS1 | 11 | 68675800 | GA | G | 1 | NM_002180 | IGHMBP2 | frameshift_deleti  on | exon3 | c.445delA | p.K149fs | . | . |
| XS1 | 11 | 68703811 | A | AT | 0.562 | NM_002180 | IGHMBP2 | frameshift_inserti  on | exon13 | c.1864dupT | p.A621fs | . | . |

| XS1 | 11 | 68855361 | CG | C | 1 | NM_139075 | TPCN2 | frameshift_deleti  on | exon25 | c.2200delG | p.G734fs | . | . |
| --- | --- | --- | --- | --- | --- | --- | --- | --- | --- | --- | --- | --- | --- |
| XS1 | 11 | 71249125 | A | AGGCT  GTGGC TCCGG | 0.44 | NM_021046 | KRTAP5-8 | nonframeshift_in sertion | exon1 | c.24_25insGGC  TGTGGCTCCG GCTGTGGG | p.G8delinsGGC GSGCG | . | . |
| XS1 | 11 | 71276714 | CT | C | 0.429 | NM_001012710 | KRTAP5-10 | frameshift_deleti  on | exon1 | c.82delT | p.C28fs | . | . |
| XS1 | 11 | 71276921 | CTGTGG  GGGCTC CAAGGG GGGCTG | C | 0.34 | NM_001012710 | KRTAP5-10 | nonframeshift_de letion | exon1 | c.289_318del | p.97_106del | . | . |
| XS1 | 11 | 72141393 | CCAA | C | 0.198 | NM_001258392 | CLPB | nonframeshift_de  letion | exon2 | c.415_417del | p.139_139del | . | . |
| XS1 | 11 | 73429709 | G | GA | 0.3 | NM_198896 | RAB6A | frameshift_inserti  on | exon4 | c.237dupT | p.P80fs | . | . |
| XS1 | 11 | 74547711 | GCC | G | 0.286 | NM_001098638 | RNF169 | frameshift_deleti  on | exon6 | c.2064_2065del | p.S688fs | . | . |
| XS1 | 11 | 74688075 | GA | G | 1 | NM_014752 | SPCS2 | frameshift_deleti  on | exon5 | c.669delA | p.R223fs | . | . |
| XS1 | 11 | 76954788 | T | TA | 0.481 | NM_182833 | GDPD4 | frameshift_inserti  on | exon12 | c.1191dupT | p.I398fs | . | . |
| XS1 | 11 | 82571141 | T | TA | 1 | NM_005040 | PRCP | stopgain | exon2 | c.186dupT | p.N63_T64delin  sX | . | . |
| XS1 | 11 | 82879928 | TC | T | 0.4 | NM_015885 | PCF11 | frameshift_deleti  on | exon8 | c.2552delC | p.S851fs | . | . |
| XS1 | 11 | 83195226 | AT | A | 0.545 | NM_001206769 | DLG2 | frameshift_deleti  on | exon16 | c.2040delA | p.K680fs | . | . |
| XS1 | 11 | 85420429 | CA | C | 0.321 | NM_032379 | SYTL2 | frameshift_deleti  on | exon3 | c.270delT | p.F90fs | . | . |
| XS1 | 11 | 85437265 | TA | T | 0.364 | NM_206927 | SYTL2 | frameshift_deleti  on | exon1 | c.234delT | p.V78fs | . | . |
| XS1 | 11 | 87030397 | CT | C | 0.583 | NM_001168724 | TMEM135 | frameshift_deleti  on | exon13 | c.1157delT | p.L386fs | . | . |
| XS1 | 11 | 89896763 | TG | T | 0.75 | NM_005467 | NAALAD2 | frameshift_deleti  on | exon11 | c.1257delG | p.L419fs | . | . |
| XS1 | 11 | 93460459 | G | GA | 0.952 | NM_033395 | KIAA1731 | frameshift_inserti  on | exon24 | c.6818dupA | p.E2273fs | . | . |
| XS1 | 11 | 93492966 | AT | A | 0.9 | NM_001286070 | C11orf54 | frameshift_deleti  on | exon7 | c.660delT | p.H220fs | . | . |
| XS1 | 11 | 93779012 | AT | A | 0.431 | NM_001098672 | HEPHL1 | frameshift_deleti  on | exon2 | c.345delT | p.H115fs | . | . |
| XS1 | 11 | 96117365 | TTC | T | 0.5 | NM_024725 | CCDC82 | frameshift_deleti  on | exon4 | c.545_546del | p.R182fs | . | . |
| XS1 | 11 | 101353895 | AT | A | 0.39 | NM_004621 | TRPC6 | frameshift_deleti  on | exon5 | c.1294delA | p.M432fs | . | . |
| XS1 | 11 | 101832972 | CA | C | 0.324 | NM_020802 | KIAA1377 | frameshift_deleti  on | exon6 | c.1207delA | p.K403fs | . | . |
| XS1 | 11 | 102221078 | GC | G | 0.318 | NM_001166 | BIRC2 | frameshift_deleti  on | exon2 | c.494delC | p.A165fs | . | . |
| XS1 | 11 | 102593278 | TC | T | 0.6 | NM_002424 | MMP8 | frameshift_deleti  on | exon2 | c.228delG | p.G76fs | . | . |
| XS1 | 11 | 103006470 | GC | G | 0.8 | NM_001080463 | DYNC2H1 | frameshift_deleti  on | exon17 | c.2368delC | p.P790fs | . | . |
| XS1 | 11 | 104768119 | ATC | A | 0.714 | NM_001191016 | CASP12 | frameshift_deleti  on | exon2 | c.82_83del | p.D28fs | . | . |
| XS1 | 11 | 104899948 | TC | T | 0.318 | NM_001223 | CASP1 | frameshift_deleti  on | exon6 | c.845delG | p.G282fs | . | . |
| XS1 | 11 | 105880626 | C | CT | 0.4 | NM_032424 | MSANTD4 | frameshift_inserti  on | exon3 | c.673dupA | p.R225fs | . | . |
| XS1 | 11 | 108014740 | AT | A | 0.64 | NM_000019 | ACAT1 | frameshift_deleti  on | exon10 | c.972delT | p.D324fs | . | . |
| XS1 | 11 | 108383332 | TA | T | 0.333 | NM_015065 | EXPH5 | frameshift_deleti  on | exon6 | c.2901delT | p.F967fs | . | . |
| XS1 | 11 | 108547954 | TCA | T | 0.25 | NM_004398 | DDX10 | frameshift_deleti  on | exon4 | c.522_523del | p.L174fs | . | . |
| XS1 | 11 | 110007949 | AT | A | 0.487 | NM_033390 | ZC3H12C | frameshift_deleti  on | exon2 | c.584delT | p.I195fs | . | . |
| XS1 | 11 | 110450593 | TG | T | 0.375 | NM_001258415 | ARHGAP20 | frameshift_deleti  on | exon15 | c.3007delC | p.Q1003fs | . | . |
| XS1 | 11 | 111916685 | AC | A | 0.219 | NM_001931 | DLAT | frameshift_deleti  on | exon10 | c.1390delC | p.L464fs | . | . |
| XS1 | 11 | 113803727 | AT | A | 0.905 | NM_006028 | HTR3B | frameshift_deleti  on | exon6 | c.609delT | p.N203fs | . | . |
| XS1 | 11 | 114393609 | AC | A | 0.6 | NM_152315 | NXPE1 | frameshift_deleti  on | exon5 | c.673delG | p.V225fs | . | . |
| XS1 | 11 | 114442102 | TA | T | 0.402 | NM_001077639 | NXPE4 | frameshift_deleti  on | exon6 | c.1192delT | p.Y398fs | . | . |
| XS1 | 11 | 114453668 | GT | G | 0.212 | NM_001077639 | NXPE4 | frameshift_deleti  on | exon3 | c.171delA | p.T57fs | . | . |
| XS1 | 11 | 114568971 | ACAGC | A | 0.167 | NM_182495 | NXPE2 | frameshift_deleti  on | exon3 | c.338_341del | p.T113fs | . | . |
| XS1 | 11 | 117395554 | AC | A | 0.714 | NM_020693 | DSCAML1 | frameshift_deleti  on | exon5 | c.1082delG | p.G361fs | . | . |
| XS1 | 11 | 118769794 | TG | T | 1 | NM_182557 | BCL9L | frameshift_deleti  on | exon8 | c.3829delC | p.Q1277fs | . | . |
| XS1 | 11 | 118965860 | CG | C | 0.421 | NM_002105 | H2AFX | frameshift_deleti  on | exon1 | c.244delC | p.R82fs | . | . |
| XS1 | 11 | 119050547 | GC | G | 0.333 | NM_001282143 | NLRX1 | frameshift_deleti  on | exon7 | c.1818delC | p.G606fs | . | . |
| XS1 | 11 | 119053858 | CG | C | 0.873 | NM_001282143 | NLRX1 | frameshift_deleti  on | exon10 | c.2639delG | p.R880fs | . | . |
| XS1 | 11 | 119535677 | CCCT | C | 0.153 | NM_002855 | PVRL1 | nonframeshift_de  letion | exon6 | c.1331_1333del | p.444_445del | . | . |
| XS1 | 11 | 123601474 | CA | C | 0.7 | NM_003455 | ZNF202 | frameshift_deleti  on | exon4 | c.122delT | p.V41fs | . | . |
| XS1 | 11 | 124189975 | AC | A | 0.873 | NM_001002918 | OR8D2 | frameshift_deleti  on | exon1 | c.118delG | p.V40fs | . | . |
| XS1 | 11 | 124628354 | T | TC | 0.923 | NM_138961 | ESAM | frameshift_inserti  on | exon2 | c.139dupG | p.E47fs | . | . |
| XS1 | 11 | 124908333 | GA | G | 0.467 | NM_025004 | CCDC15 | frameshift_deleti  on | exon14 | c.2419delA | p.K807fs | . | . |
| XS1 | 11 | 125452300 | A | AC | 1 | NM_001007277 | EI24 | frameshift_inserti  on | exon9 | c.733dupC | p.R244fs | . | . |
| XS1 | 11 | 129321279 | A | AC | 0.483 | NM_003658 | BARX2 | frameshift_inserti  on | exon4 | c.823dupC | p.E274fs | . | . |
| XS1 | 11 | 129785563 | TG | T | 0.267 | NM_199439 | PRDM10 | stopgain | exon12 | c.2259delC | p.Y753X | . | . |
| XS1 | 11 | 129785597 | AG | A | 0.316 | NM_199439 | PRDM10 | frameshift_deleti on | exon12 | c.2225delC | p.P742fs | . | . |
| XS1 | 11 | 134038867 | GA | G | 0.283 | NM_015261 | NCAPD3 | frameshift_deleti  on | exon25 | c.3183delT | p.F1061fs | . | . |
| XS1 | 11 | 134238540 | GT | G | 0.571 | NM_138342 | GLB1L2 | frameshift_deleti  on | exon10 | c.893delT | p.V298fs | . | . |
| XS1 | 12 | 974355 | T | TC | 0.429 | NM_213655 | WNK1 | frameshift_inserti  on | exon9 | c.2220dupC | p.L740fs | . | . |

| XS1 | 12 | 2062323 | T | TTGC | 0.555 | NM_152640 | DCP1B | nonframeshift_in  sertion | exon7 | c.782_783insG  CA | p.Q261delinsQ  Q | . | . |
| --- | --- | --- | --- | --- | --- | --- | --- | --- | --- | --- | --- | --- | --- |
| XS1 | 12 | 2062345 | T | TGCC | 0.399 | NM_152640 | DCP1B | nonframeshift_in  sertion | exon7 | c.760_761insG  GC | p.Q254delinsR  Q | . | . |
| XS1 | 12 | 2062348 | T | TGCC | 0.411 | NM_152640 | DCP1B | nonframeshift_in  sertion | exon7 | c.757_758insG  GC | p.Q253delinsR  Q | . | . |
| XS1 | 12 | 2062350 | C | CTGG | 0.425 | NM_152640 | DCP1B | nonframeshift_in  sertion | exon7 | c.755_756insCC  A | p.Q252delinsH  Q | . | . |
| XS1 | 12 | 4446207 | CA | C | 0.345 | NM_020375 | C12orf5 | frameshift_deleti  on | exon3 | c.74delA | p.Q25fs | . | . |
| XS1 | 12 | 4835974 | TC | T | 0.345 | NM_017417 | GALNT8 | frameshift_deleti  on | exon2 | c.489delC | p.I163fs | . | . |
| XS1 | 12 | 6122680 | AG | A | 0.4 | NM_000552 | VWF | frameshift_deleti  on | exon32 | c.5586delC | p.T1862fs | . | . |
| XS1 | 12 | 6443313 | TG | T | 0.387 | NM_001065 | TNFRSF1A | frameshift_deleti  on | exon2 | c.136delC | p.Q46fs | . | . |
| XS1 | 12 | 6777069 | TTGC | T | 0.364 | NM_001039920 | ZNF384 | nonframeshift_de  letion | exon9 | c.1194_1196del | p.398_399del | . | . |
| XS1 | 12 | 6782485 | AG | A | 0.198 | NM_001039920 | ZNF384 | frameshift_deleti  on | exon6 | c.642delC | p.S214fs | . | . |
| XS1 | 12 | 6806713 | GC | G | 1 | NM_001244014 | PIANP | frameshift_deleti  on | exon3 | c.262delG | p.A88fs | . | . |
| XS1 | 12 | 7475087 | A | AG | 0.258 | NM_001080454 | ACSM4 | frameshift_inserti  on | exon7 | c.1076dupG | p.R359fs | . | . |
| XS1 | 12 | 7519865 | TGA | T | 0.512 | NM_174941 | CD163L1 | frameshift_deleti  on | exon18 | c.4244_4245del | p.L1415fs | . | . |
| XS1 | 12 | 7647893 | AG | A | 0.206 | NM_004244 | CD163 | frameshift_deleti  on | exon6 | c.1203delC | p.G401fs | . | . |
| XS1 | 12 | 7848212 | GC | G | 0.888 | NM_020634 | GDF3 | frameshift_deleti  on | exon1 | c.112delG | p.A38fs | . | . |
| XS1 | 12 | 8200557 | ACAG | A | 0.158 | NM_018416 | FOXJ2 | nonframeshift_de  letion | exon7 | c.898_900del | p.300_300del | . | . |
| XS1 | 12 | 8687303 | AC | A | 0.199 | NM_014358 | CLEC4E | frameshift_deleti  on | exon6 | c.590delG | p.C197fs | . | . |
| XS1 | 12 | 8904572 | TA | T | 0.389 | NM_020734 | RIMKLB | frameshift_deleti  on | exon5 | c.427delA | p.K143fs | . | . |
| XS1 | 12 | 9002777 | CT | C | 0.3 | NM_001282424 | A2ML1 | frameshift_deleti  on | exon7 | c.669delT | p.A223fs | . | . |
| XS1 | 12 | 9083137 | T | TCCC | 0.137 | NM_004426 | PHC1 | nonframeshift_in  sertion | exon7 | c.719_720insCC  C | p.L240delinsLP | . | . |
| XS1 | 12 | 9098995 | G | GAAC | 1 | NM_001207024 | M6PR | nonframeshift_in  sertion | exon2 | c.5_6insGTT | p.F2delinsLF | . | . |
| XS1 | 12 | 9252104 | GA | G | 1 | NM_000014 | A2M | frameshift_deleti  on | exon14 | c.1573delT | p.S525fs | . | . |
| XS1 | 12 | 10568363 | G | GT | 0.5 | NM_002261 | KLRC3 | frameshift_inserti  on | exon6 | c.617dupA | p.N206fs | . | . |
| XS1 | 12 | 10862657 | AG | A | 0.375 | NM_003651 | YBX3 | frameshift_deleti  on | exon6 | c.629delC | p.P210fs | . | . |
| XS1 | 12 | 11150408 | CA | C | 0.583 | NM_176889 | TAS2R20 | frameshift_deleti  on | exon1 | c.66delT | p.F22fs | . | . |
| XS1 | 12 | 11174672 | TG | T | 0.417 | NM_176888 | TAS2R19 | frameshift_deleti  on | exon1 | c.498delC | p.I166fs | . | . |
| XS1 | 12 | 11286777 | CA | C | 0.5 | NM_001097643 | TAS2R30 | frameshift_deleti  on | exon1 | c.66delT | p.F22fs | . | . |
| XS1 | 12 | 12318164 | TC | T | 0.259 | NM_002336 | LRP6 | frameshift_deleti  on | exon8 | c.1610delG | p.G537fs | . | . |
| XS1 | 12 | 12397397 | G | GT | 0.95 | NM_002336 | LRP6 | frameshift_inserti  on | exon2 | c.247dupA | p.T83fs | . | . |
| XS1 | 12 | 13228987 | A | AC | 0.364 | NM_020853 | KIAA1467 | frameshift_inserti  on | exon11 | c.1552_1553ins  C | p.S518fs | . | . |
| XS1 | 12 | 13716624 | G | GT | 0.282 | NM_000834 | GRIN2B | frameshift_inserti  on | exon13 | c.3547dupA | p.T1183fs | . | . |
| XS1 | 12 | 18435221 | CA | C | 0.306 | NM_001288772 | PIK3C2G | frameshift_deleti  on | exon2 | c.207delA | p.P69fs | . | . |
| XS1 | 12 | 18435398 | GCCC | G | 0.944 | NM_001288772 | PIK3C2G | nonframeshift_de  letion | exon2 | c.384_386del | p.128_129del | . | . |
| XS1 | 12 | 18847931 | A | AT | 0.622 | NM_033123 | PLCZ1 | frameshift_inserti  on | exon12 | c.1373_1374ins  A | p.I458fs | . | . |
| XS1 | 12 | 21011471 | AT | A | 0.857 | NM_019844 | SLCO1B3 | frameshift_deleti  on | exon5 | c.326delT | p.I109fs | . | . |
| XS1 | 12 | 21448636 | TGG | T | 0.5 | NM_021094 | SLCO1A2 | frameshift_deleti  on | exon9 | c.1164_1165del | p.A388fs | . | . |
| XS1 | 12 | 21997466 | A | AG | 0.861 | NM_005691 | ABCC9 | frameshift_inserti  on | exon26 | c.3265dupC | p.L1089fs | . | . |
| XS1 | 12 | 26647155 | AT | A | 0.9 | NM_002223 | ITPR2 | frameshift_deleti  on | exon39 | c.5300delA | p.N1767fs | . | . |
| XS1 | 12 | 27156222 | G | GA | 0.65 | NM_016551 | TM7SF3 | frameshift_inserti  on | exon2 | c.192dupT | p.L65fs | . | . |
| XS1 | 12 | 27533332 | TA | T | 0.319 | NM_001248003 | ARNTL2 | frameshift_deleti  on | exon3 | c.369delA | p.L123fs | . | . |
| XS1 | 12 | 27809598 | TA | T | 0.5 | NM_001198915 | PPFIBP1 | frameshift_deleti  on | exon8 | c.381delA | p.V127fs | . | . |
| XS1 | 12 | 28702014 | C | CA | 0.198 | NM_018318 | CCDC91 | frameshift_inserti  on | exon12 | c.1235dupA | p.Q412fs | . | . |
| XS1 | 12 | 31256271 | AC | A | 0.993 | NM_004399 | DDX11 | frameshift_deleti  on | exon24 | c.2329delC | p.P777fs | . | . |
| XS1 | 12 | 31545565 | G | GA | 1 | NM_144973 | DENND5B | frameshift_inserti  on | exon18 | c.3321dupT | p.H1108fs | . | . |
| XS1 | 12 | 32858763 | TG | T | 0.375 | NM_001278464 | DNM1L | frameshift_deleti  on | exon3 | c.256delG | p.A86fs | . | . |
| XS1 | 12 | 41330678 | AC | A | 0.467 | NM_175038 | CNTN1 | frameshift_deleti  on | exon8 | c.1049delC | p.T350fs | . | . |
| XS1 | 12 | 44782164 | GA | G | 0.989 | NM_001286212 | TMEM117 | frameshift_deleti  on | exon7 | c.943delA | p.K315fs | . | . |
| XS1 | 12 | 47471231 | CA | C | 1 | NM_181847 | AMIGO2 | frameshift_deleti  on | exon2 | c.1554delT | p.F518fs | . | . |
| XS1 | 12 | 48110723 | GT | G | 0.855 | NM_001172440 | ENDOU | frameshift_deleti  on | exon3 | c.311delA | p.N104fs | . | . |
| XS1 | 12 | 48501965 | GT | G | 0.374 | NM_001166686 | PFKM | frameshift_deleti  on | exon3 | c.194delT | p.V65fs | . | . |
| XS1 | 12 | 49420815 | GC | G | 0.333 | NM_003482 | KMT2D | frameshift_deleti  on | exon48 | c.14933delG | p.R4978fs | . | . |
| XS1 | 12 | 49444565 | GAA | G | 0.692 | NM_003482 | KMT2D | frameshift_deleti  on | exon11 | c.2804_2805del | p.L935fs | . | . |
| XS1 | 12 | 50358872 | CCAT | C | 0.667 | NM_001651 | AQP5 | nonframeshift_de  letion | exon4 | c.711_713del | p.237_238del | . | . |
| XS1 | 12 | 50481159 | TG | T | 0.136 | NM_003076 | SMARCD1 | frameshift_deleti  on | exon5 | c.546delG | p.L182fs | . | . |
| XS1 | 12 | 50500597 | AC | A | 0.216 | NM_001257199 | GPD1 | frameshift_deleti  on | exon5 | c.441delC | p.D147fs | . | . |
| XS1 | 12 | 50528349 | TC | T | 1 | NM_147190 | CERS5 | frameshift_deleti  on | exon9 | c.1008delG | p.L336fs | . | . |
| XS1 | 12 | 50747664 | GT | G | 0.356 | NM_001145475 | FAM186A | frameshift_deleti  on | exon4 | c.2950delA | p.T984fs | . | . |

| XS1 | 12 | 51899650 | G | GA | 0.19 | NM_001039960 | SLC4A8 | frameshift_inserti  on | exon24 | c.3206dupA | p.E1069fs | . | . |
| --- | --- | --- | --- | --- | --- | --- | --- | --- | --- | --- | --- | --- | --- |
| XS1 | 12 | 52369166 | TC | T | 0.692 | NM_004302 | ACVR1B | frameshift_deleti  on | exon2 | c.210delC | p.I70fs | . | . |
| XS1 | 12 | 52629129 | TG | T | 0.616 | NM_005556 | KRT7 | frameshift_deleti  on | exon2 | c.516delG | p.V172fs | . | . |
| XS1 | 12 | 53045376 | GT | G | 0.388 | NM_000423 | KRT2 | frameshift_deleti  on | exon1 | c.550delA | p.T184fs | . | . |
| XS1 | 12 | 53162568 | CA | C | 0.227 | NM_015848 | KRT76 | frameshift_deleti  on | exon9 | c.1845delT | p.S615fs | . | . |
| XS1 | 12 | 53189413 | ACCAAA  GCCACC AGCCCC | A | 0.929 | NM_057088 | KRT3 | nonframeshift_de letion | exon1 | c.396_413del | p.132_138del | . | . |
| XS1 | 12 | 53462039 | GC | G | 0.462 | NM_032840 | SPRYD3 | frameshift_deleti  on | exon7 | c.742delG | p.A248fs | . | . |
| XS1 | 12 | 53512971 | A | AT | 0.291 | NM_003578 | SOAT2 | frameshift_inserti  on | exon10 | c.973_974insT | p.N325fs | . | . |
| XS1 | 12 | 53687184 | CG | C | 0.515 | NM_012291 | ESPL1 | frameshift_deleti  on | exon31 | c.6290delG | p.R2097fs | . | . |
| XS1 | 12 | 54117392 | CT | C | 0.45 | NM_001143682 | CALCOCO1 | frameshift_deleti  on | exon4 | c.335delA | p.K112fs | . | . |
| XS1 | 12 | 55420898 | GC | G | 0.796 | NM_021191 | NEUROD4 | frameshift_deleti  on | exon2 | c.676delC | p.P226fs | . | . |
| XS1 | 12 | 55615200 | A | AT | 0.429 | NM_001005280 | OR10A7 | frameshift_inserti  on | exon1 | c.393dupT | p.H131fs | . | . |
| XS1 | 12 | 55714919 | AT | A | 0.529 | NM_001005182 | OR6C1 | frameshift_deleti  on | exon1 | c.537delT | p.Y179fs | . | . |
| XS1 | 12 | 55794587 | AT | A | 0.375 | NM_001005518 | OR6C65 | stopgain | exon1 | c.276delT | p.Y92X | . | . |
| XS1 | 12 | 55863676 | TG | T | 0.25 | NM_001005499 | OR6C70 | frameshift_deleti on | exon1 | c.246delC | p.T82fs | . | . |
| XS1 | 12 | 56082019 | TA | T | 0.292 | NM_001144996 | ITGA7 | frameshift_deleti  on | exon23 | c.3035delT | p.I1012fs | . | . |
| XS1 | 12 | 56666904 | CT | C | 0.353 | NM_004077 | CS | frameshift_deleti  on | exon11 | c.1364delA | p.E455fs | . | . |
| XS1 | 12 | 57397482 | GT | G | 0.345 | NM_014830 | ZBTB39 | frameshift_deleti  on | exon2 | c.1219delA | p.T407fs | . | . |
[truncated: 443,449 more chars]
